# Supplementary material for: Facing the infinity: tackling large samples of challenging Chironomidae (Diptera) with an integrative approach
Source: PeerJ. 2023 May 22;11:e15336. doi: 10.7717/peerj.15336 (PMC10211366; doi:10.7717/peerj.15336)
Supplement: Supplemental Information 1 — All sequences have been renamed to include the ASAP- and RESL-OTU IDs. [file peerj-11-15336-s001.pdf]

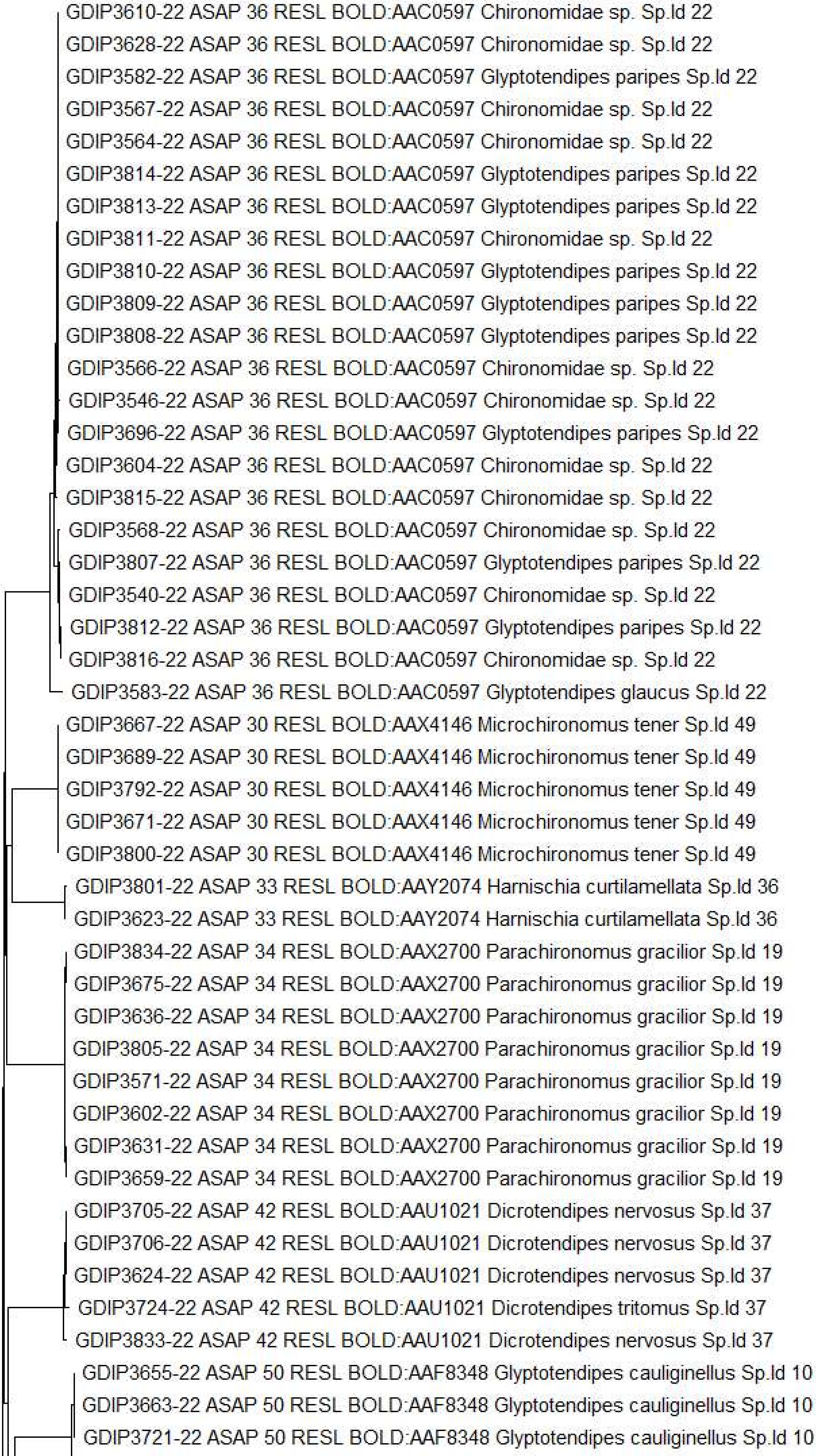
GDIP3610-22 ASAP 36 RESL BOLD:AAC0597 Chironomidae sp. Sp.Id 22
GDIP3628-22 ASAP 36 RESL BOLD:AAC0597 Chironomidae sp. Sp.Id 22
GDIP3582-22 ASAP 36 RESL BOLD:AAC0597 Glyptotendipes paripes Sp.Id 22
GDIP3567-22 ASAP 36 RESL BOLD:AAC0597 Chironomidae sp. Sp.Id 22
GDIP3564-22 ASAP 36 RESL BOLD:AAC0597 Chironomidae sp. Sp.Id 22
GDIP3814-22 ASAP 36 RESL BOLD:AAC0597 Glyptotendipes paripes Sp.Id 22
GDIP3813-22 ASAP 36 RESL BOLD:AAC0597 Glyptotendipes paripes Sp.Id 22
GDIP3811-22 ASAP 36 RESL BOLD:AAC0597 Chironomidae sp. Sp.Id 22
GDIP3810-22 ASAP 36 RESL BOLD:AAC0597 Glyptotendipes paripes Sp.Id 22
GDIP3809-22 ASAP 36 RESL BOLD:AAC0597 Glyptotendipes paripes Sp.Id 22
GDIP3808-22 ASAP 36 RESL BOLD:AAC0597 Glyptotendipes paripes Sp.Id 22
GDIP3566-22 ASAP 36 RESL BOLD:AAC0597 Chironomidae sp. Sp.Id 22
GDIP3546-22 ASAP 36 RESL BOLD:AAC0597 Chironomidae sp. Sp.Id 22
GDIP3696-22 ASAP 36 RESL BOLD:AAC0597 Glyptotendipes paripes Sp.Id 22
GDIP3604-22 ASAP 36 RESL BOLD:AAC0597 Chironomidae sp. Sp.Id 22
GDIP3815-22 ASAP 36 RESL BOLD:AAC0597 Chironomidae sp. Sp.Id 22
GDIP3568-22 ASAP 36 RESL BOLD:AAC0597 Chironomidae sp. Sp.Id 22
GDIP3807-22 ASAP 36 RESL BOLD:AAC0597 Glyptotendipes paripes Sp.Id 22
GDIP3540-22 ASAP 36 RESL BOLD:AAC0597 Chironomidae sp. Sp.Id 22
GDIP3812-22 ASAP 36 RESL BOLD:AAC0597 Glyptotendipes paripes Sp.Id 22
GDIP3816-22 ASAP 36 RESL BOLD:AAC0597 Chironomidae sp. Sp.Id 22
GDIP3583-22 ASAP 36 RESL BOLD:AAC0597 Glyptotendipes glaucus Sp.Id 22
GDIP3667-22 ASAP 30 RESL BOLD:AAX4146 Microchironomus tener Sp.Id 49
GDIP3689-22 ASAP 30 RESL BOLD:AAX4146 Microchironomus tener Sp.Id 49
GDIP3792-22 ASAP 30 RESL BOLD:AAX4146 Microchironomus tener Sp.Id 49
GDIP3671-22 ASAP 30 RESL BOLD:AAX4146 Microchironomus tener Sp.Id 49
GDIP3800-22 ASAP 30 RESL BOLD:AAX4146 Microchironomus tener Sp.Id 49
GDIP3801-22 ASAP 33 RESL BOLD:AAY2074 Harnischia curtilamellata Sp.Id 36
GDIP3623-22 ASAP 33 RESL BOLD:AAY2074 Harnischia curtilamellata Sp.Id 36
GDIP3834-22 ASAP 34 RESL BOLD:AAX2700 Parachironomus gracilior Sp.Id 19
GDIP3675-22 ASAP 34 RESL BOLD:AAX2700 Parachironomus gracilior Sp.Id 19
GDIP3636-22 ASAP 34 RESL BOLD:AAX2700 Parachironomus gracilior Sp.Id 19
GDIP3805-22 ASAP 34 RESL BOLD:AAX2700 Parachironomus gracilior Sp.Id 19
GDIP3571-22 ASAP 34 RESL BOLD:AAX2700 Parachironomus gracilior Sp.Id 19
GDIP3602-22 ASAP 34 RESL BOLD:AAX2700 Parachironomus gracilior Sp.Id 19
GDIP3631-22 ASAP 34 RESL BOLD:AAX2700 Parachironomus gracilior Sp.Id 19
GDIP3659-22 ASAP 34 RESL BOLD:AAX2700 Parachironomus gracilior Sp.Id 19
GDIP3705-22 ASAP 42 RESL BOLD:AAU1021 Dicrotendipes nervosus Sp.Id 37
GDIP3706-22 ASAP 42 RESL BOLD:AAU1021 Dicrotendipes nervosus Sp.Id 37
GDIP3624-22 ASAP 42 RESL BOLD:AAU1021 Dicrotendipes nervosus Sp.Id 37
GDIP3724-22 ASAP 42 RESL BOLD:AAU1021 Dicrotendipes tritomus Sp.Id 37
GDIP3833-22 ASAP 42 RESL BOLD:AAU1021 Dicrotendipes nervosus Sp.Id 37
GDIP3655-22 ASAP 50 RESL BOLD:AAF8348 Glyptotendipes cauliginellus Sp.Id 10
GDIP3663-22 ASAP 50 RESL BOLD:AAF8348 Glyptotendipes cauliginellus Sp.Id 10
GDIP3721-22 ASAP 50 RESL BOLD:AAF8348 Glyptotendipes cauliginellus Sp.Id 10

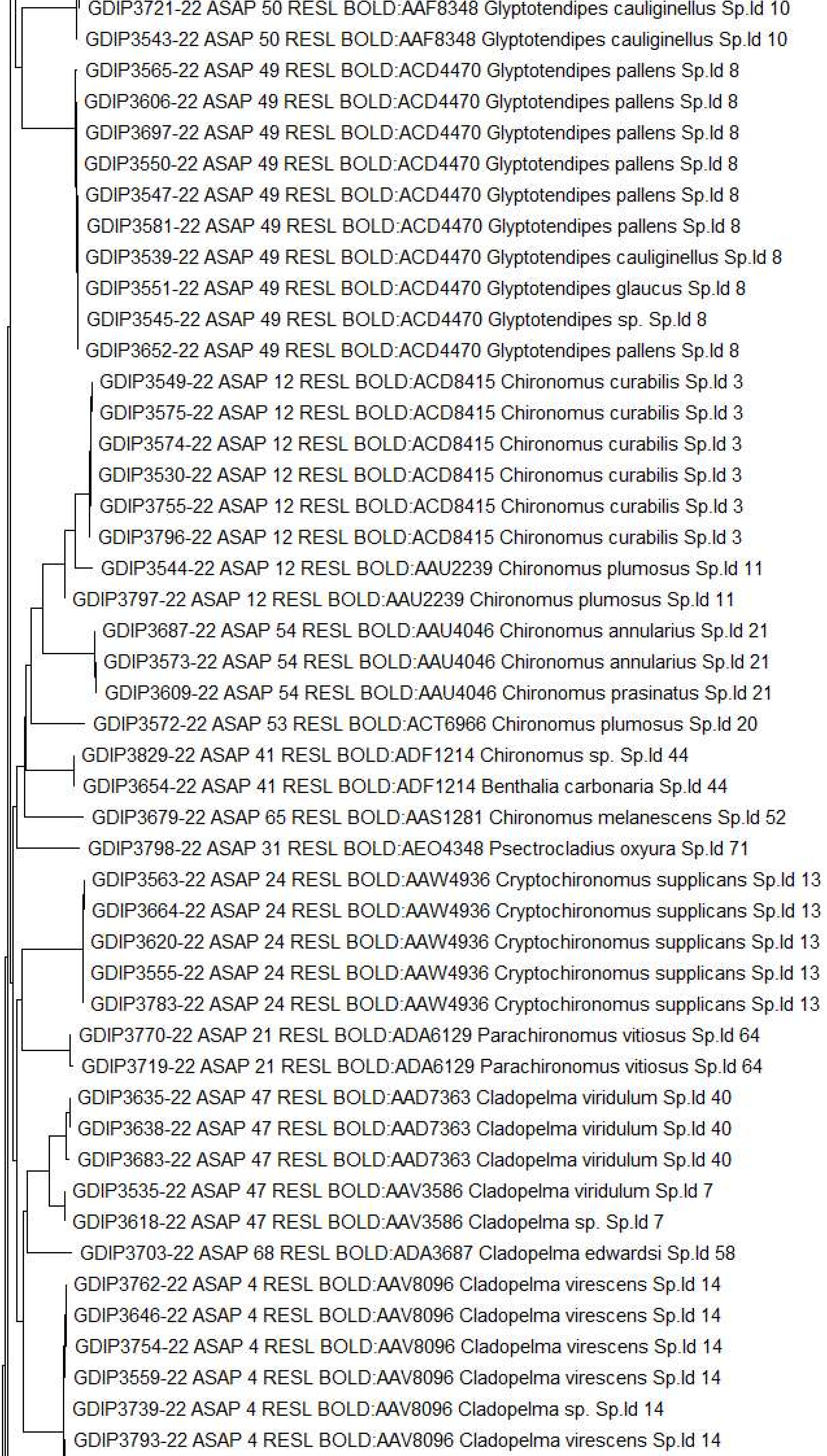
GDIP3721-22 ASAP 50 RESL BOLD:AAF8348 Glyptotendipes cauliginellus Sp.Id 10
GDIP3543-22 ASAP 50 RESL BOLD:AAF8348 Glyptotendipes cauliginellus Sp.Id 10
GDIP3565-22 ASAP 49 RESL BOLD:ACD4470 Glyptotendipes pallens Sp.Id 8
GDIP3606-22 ASAP 49 RESL BOLD:ACD4470 Glyptotendipes pallens Sp.Id 8
GDIP3697-22 ASAP 49 RESL BOLD:ACD4470 Glyptotendipes pallens Sp.Id 8
GDIP3550-22 ASAP 49 RESL BOLD:ACD4470 Glyptotendipes pallens Sp.Id 8
GDIP3547-22 ASAP 49 RESL BOLD:ACD4470 Glyptotendipes pallens Sp.Id 8
GDIP3581-22 ASAP 49 RESL BOLD:ACD4470 Glyptotendipes pallens Sp.Id 8
GDIP3539-22 ASAP 49 RESL BOLD:ACD4470 Glyptotendipes cauliginellus Sp.Id 8
GDIP3551-22 ASAP 49 RESL BOLD:ACD4470 Glyptotendipes glaucus Sp.Id 8
GDIP3545-22 ASAP 49 RESL BOLD:ACD4470 Glyptotendipes sp. Sp.Id 8
GDIP3652-22 ASAP 49 RESL BOLD:ACD4470 Glyptotendipes pallens Sp.Id 8
GDIP3549-22 ASAP 12 RESL BOLD:ACD8415 Chironomus curabilis Sp.Id 3
GDIP3575-22 ASAP 12 RESL BOLD:ACD8415 Chironomus curabilis Sp.Id 3
GDIP3574-22 ASAP 12 RESL BOLD:ACD8415 Chironomus curabilis Sp.Id 3
GDIP3530-22 ASAP 12 RESL BOLD:ACD8415 Chironomus curabilis Sp.Id 3
GDIP3755-22 ASAP 12 RESL BOLD:ACD8415 Chironomus curabilis Sp.Id 3
GDIP3796-22 ASAP 12 RESL BOLD:ACD8415 Chironomus curabilis Sp.Id 3
GDIP3544-22 ASAP 12 RESL BOLD:AAU2239 Chironomus plumosus Sp.Id 11
GDIP3797-22 ASAP 12 RESL BOLD:AAU2239 Chironomus plumosus Sp.Id 11
GDIP3687-22 ASAP 54 RESL BOLD:AAU4046 Chironomus annularius Sp.Id 21
GDIP3573-22 ASAP 54 RESL BOLD:AAU4046 Chironomus annularius Sp.Id 21
GDIP3609-22 ASAP 54 RESL BOLD:AAU4046 Chironomus prasinatus Sp.Id 21
GDIP3572-22 ASAP 53 RESL BOLD:ACT6966 Chironomus plumosus Sp.Id 20
GDIP3829-22 ASAP 41 RESL BOLD:ADF1214 Chironomus sp. Sp.Id 44
GDIP3654-22 ASAP 41 RESL BOLD:ADF1214 Benthalia carbonaria Sp.Id 44
GDIP3679-22 ASAP 65 RESL BOLD:AAS1281 Chironomus melanescens Sp.Id 52
GDIP3798-22 ASAP 31 RESL BOLD:AEO4348 Psectrocladius oxyura Sp.Id 71
GDIP3563-22 ASAP 24 RESL BOLD:AAW4936 Cryptochironomus supplicans Sp.Id 13
GDIP3664-22 ASAP 24 RESL BOLD:AAW4936 Cryptochironomus supplicans Sp.Id 13
GDIP3620-22 ASAP 24 RESL BOLD:AAW4936 Cryptochironomus supplicans Sp.Id 13
GDIP3555-22 ASAP 24 RESL BOLD:AAW4936 Cryptochironomus supplicans Sp.Id 13
GDIP3783-22 ASAP 24 RESL BOLD:AAW4936 Cryptochironomus supplicans Sp.Id 13
GDIP3770-22 ASAP 21 RESL BOLD:ADA6129 Parachironomus vitiosus Sp.Id 64
GDIP3719-22 ASAP 21 RESL BOLD:ADA6129 Parachironomus vitiosus Sp.Id 64
GDIP3635-22 ASAP 47 RESL BOLD:AAD7363 Cladopelma viridulum Sp.Id 40
GDIP3638-22 ASAP 47 RESL BOLD:AAD7363 Cladopelma viridulum Sp.Id 40
GDIP3683-22 ASAP 47 RESL BOLD:AAD7363 Cladopelma viridulum Sp.Id 40
GDIP3535-22 ASAP 47 RESL BOLD:AAV3586 Cladopelma viridulum Sp.Id 7
GDIP3618-22 ASAP 47 RESL BOLD:AAV3586 Cladopelma sp. Sp.Id 7
GDIP3703-22 ASAP 68 RESL BOLD:ADA3687 Cladopelma edwardsi Sp.Id 58
GDIP3762-22 ASAP 4 RESL BOLD:AAV8096 Cladopelma virescens Sp.Id 14
GDIP3646-22 ASAP 4 RESL BOLD:AAV8096 Cladopelma virescens Sp.Id 14
GDIP3754-22 ASAP 4 RESL BOLD:AAV8096 Cladopelma virescens Sp.Id 14
GDIP3559-22 ASAP 4 RESL BOLD:AAV8096 Cladopelma virescens Sp.Id 14
GDIP3739-22 ASAP 4 RESL BOLD:AAV8096 Cladopelma sp. Sp.Id 14
GDIP3793-22 ASAP 4 RESL BOLD:AAV8096 Cladopelma virescens Sp.Id 14

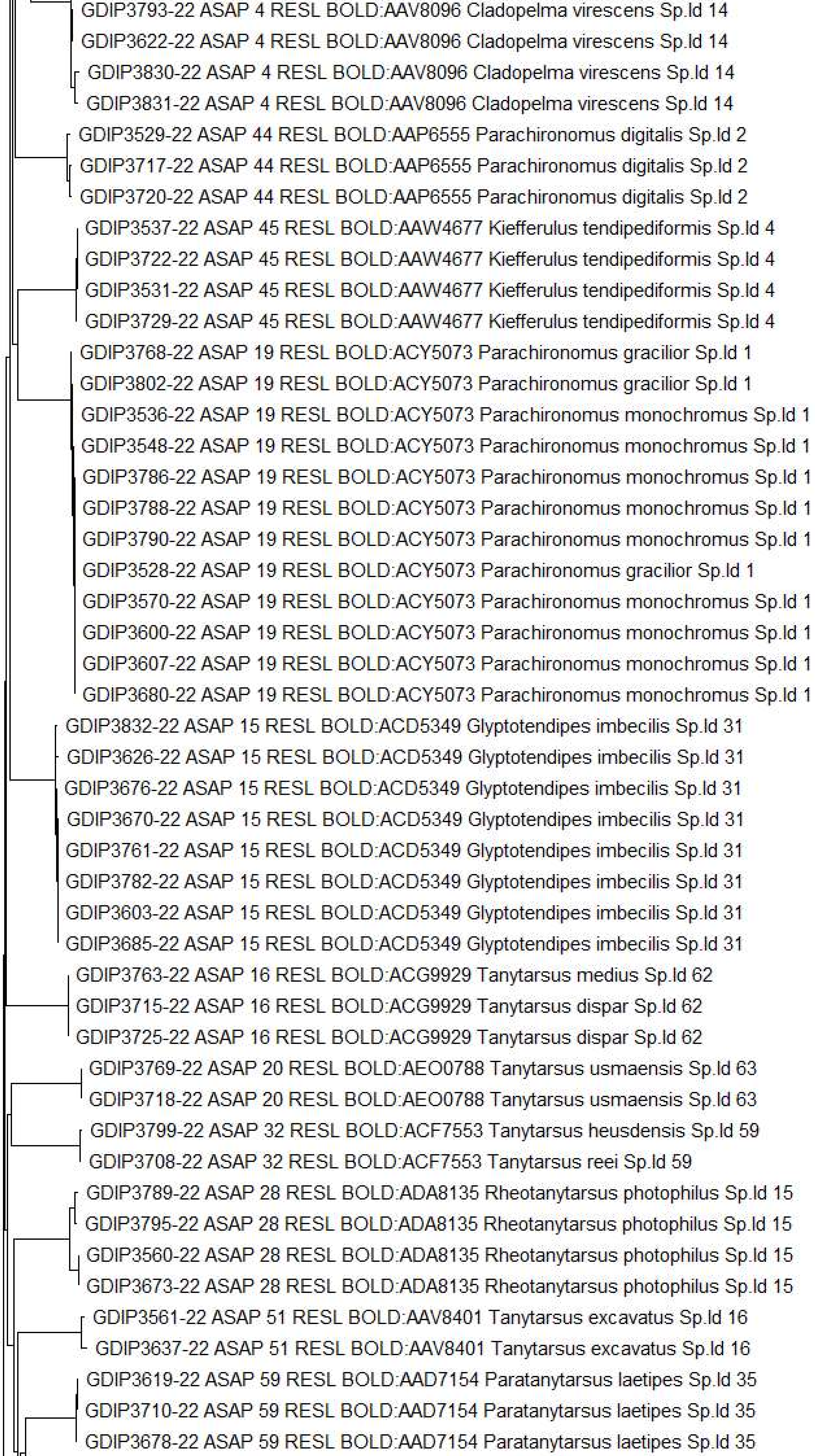
GDIP3793-22 ASAP 4 RESL BOLD:AAV8096 Cladopelma virescens Sp.Id 14
GDIP3622-22 ASAP 4 RESL BOLD:AAV8096 Cladopelma virescens Sp.Id 14
GDIP3830-22 ASAP 4 RESL BOLD:AAV8096 Cladopelma virescens Sp.Id 14
GDIP3831-22 ASAP 4 RESL BOLD:AAV8096 Cladopelma virescens Sp.Id 14
GDIP3529-22 ASAP 44 RESL BOLD:AAP6555 Parachironomus digitalis Sp.Id 2
GDIP3717-22 ASAP 44 RESL BOLD:AAP6555 Parachironomus digitalis Sp.Id 2
GDIP3720-22 ASAP 44 RESL BOLD:AAP6555 Parachironomus digitalis Sp.Id 2
GDIP3537-22 ASAP 45 RESL BOLD:AAW4677 Kiefferulus tendipediformis Sp.Id 4
GDIP3722-22 ASAP 45 RESL BOLD:AAW4677 Kiefferulus tendipediformis Sp.Id 4
GDIP3531-22 ASAP 45 RESL BOLD:AAW4677 Kiefferulus tendipediformis Sp.Id 4
GDIP3729-22 ASAP 45 RESL BOLD:AAW4677 Kiefferulus tendipediformis Sp.Id 4
GDIP3768-22 ASAP 19 RESL BOLD:ACY5073 Parachironomus gracilior Sp.Id 1
GDIP3802-22 ASAP 19 RESL BOLD:ACY5073 Parachironomus gracilior Sp.Id 1
GDIP3536-22 ASAP 19 RESL BOLD:ACY5073 Parachironomus monochromus Sp.Id 1
GDIP3548-22 ASAP 19 RESL BOLD:ACY5073 Parachironomus monochromus Sp.Id 1
GDIP3786-22 ASAP 19 RESL BOLD:ACY5073 Parachironomus monochromus Sp.Id 1
GDIP3788-22 ASAP 19 RESL BOLD:ACY5073 Parachironomus monochromus Sp.Id 1
GDIP3790-22 ASAP 19 RESL BOLD:ACY5073 Parachironomus monochromus Sp.Id 1
GDIP3528-22 ASAP 19 RESL BOLD:ACY5073 Parachironomus gracilior Sp.Id 1
GDIP3570-22 ASAP 19 RESL BOLD:ACY5073 Parachironomus monochromus Sp.Id 1
GDIP3600-22 ASAP 19 RESL BOLD:ACY5073 Parachironomus monochromus Sp.Id 1
GDIP3607-22 ASAP 19 RESL BOLD:ACY5073 Parachironomus monochromus Sp.Id 1
GDIP3680-22 ASAP 19 RESL BOLD:ACY5073 Parachironomus monochromus Sp.Id 1
GDIP3832-22 ASAP 15 RESL BOLD:ACD5349 Glyptotendipes imbecilis Sp.Id 31
GDIP3626-22 ASAP 15 RESL BOLD:ACD5349 Glyptotendipes imbecilis Sp.Id 31
GDIP3676-22 ASAP 15 RESL BOLD:ACD5349 Glyptotendipes imbecilis Sp.Id 31
GDIP3670-22 ASAP 15 RESL BOLD:ACD5349 Glyptotendipes imbecilis Sp.Id 31
GDIP3761-22 ASAP 15 RESL BOLD:ACD5349 Glyptotendipes imbecilis Sp.Id 31
GDIP3782-22 ASAP 15 RESL BOLD:ACD5349 Glyptotendipes imbecilis Sp.Id 31
GDIP3603-22 ASAP 15 RESL BOLD:ACD5349 Glyptotendipes imbecilis Sp.Id 31
GDIP3685-22 ASAP 15 RESL BOLD:ACD5349 Glyptotendipes imbecilis Sp.Id 31
GDIP3763-22 ASAP 16 RESL BOLD:ACG9929 Tanytarsus medius Sp.Id 62
GDIP3715-22 ASAP 16 RESL BOLD:ACG9929 Tanytarsus dispar Sp.Id 62
GDIP3725-22 ASAP 16 RESL BOLD:ACG9929 Tanytarsus dispar Sp.Id 62
GDIP3769-22 ASAP 20 RESL BOLD:AEO0788 Tanytarsus usmaensis Sp.Id 63
GDIP3718-22 ASAP 20 RESL BOLD:AEO0788 Tanytarsus usmaensis Sp.Id 63
GDIP3799-22 ASAP 32 RESL BOLD:ACF7553 Tanytarsus heusdensis Sp.Id 59
GDIP3708-22 ASAP 32 RESL BOLD:ACF7553 Tanytarsus reei Sp.Id 59
GDIP3789-22 ASAP 28 RESL BOLD:ADA8135 Rheotanytarsus photophilus Sp.Id 15
GDIP3795-22 ASAP 28 RESL BOLD:ADA8135 Rheotanytarsus photophilus Sp.Id 15
GDIP3560-22 ASAP 28 RESL BOLD:ADA8135 Rheotanytarsus photophilus Sp.Id 15
GDIP3673-22 ASAP 28 RESL BOLD:ADA8135 Rheotanytarsus photophilus Sp.Id 15
GDIP3561-22 ASAP 51 RESL BOLD:AAV8401 Tanytarsus excavatus Sp.Id 16
GDIP3637-22 ASAP 51 RESL BOLD:AAV8401 Tanytarsus excavatus Sp.Id 16
GDIP3619-22 ASAP 59 RESL BOLD:AAD7154 Paratanytarsus laetipes Sp.Id 35
GDIP3710-22 ASAP 59 RESL BOLD:AAD7154 Paratanytarsus laetipes Sp.Id 35
GDIP3678-22 ASAP 59 RESL BOLD:AAD7154 Paratanytarsus laetipes Sp.Id 35

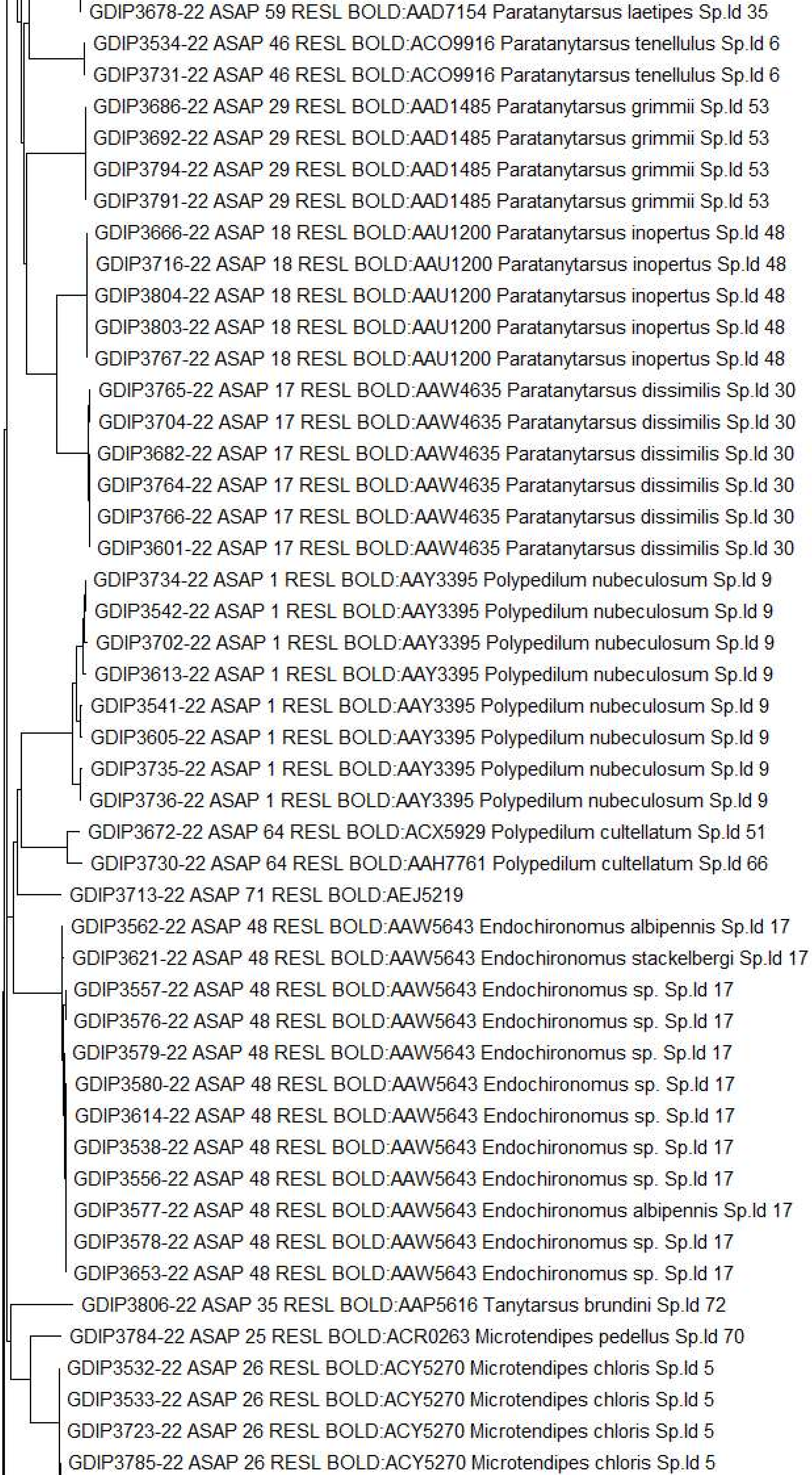
GDIP3678-22 ASAP 59 RESL BOLD:AAD7154 Paratanytarsus laetipes Sp.Id 35
GDIP3534-22 ASAP 46 RESL BOLD:ACO9916 Paratanytarsus tenellulus Sp.Id 6
GDIP3731-22 ASAP 46 RESL BOLD:ACO9916 Paratanytarsus tenellulus Sp.Id 6
GDIP3686-22 ASAP 29 RESL BOLD:AAD1485 Paratanytarsus grimmii Sp.Id 53
GDIP3692-22 ASAP 29 RESL BOLD:AAD1485 Paratanytarsus grimmii Sp.Id 53
GDIP3794-22 ASAP 29 RESL BOLD:AAD1485 Paratanytarsus grimmii Sp.Id 53
GDIP3791-22 ASAP 29 RESL BOLD:AAD1485 Paratanytarsus grimmii Sp.Id 53
GDIP3666-22 ASAP 18 RESL BOLD:AAU1200 Paratanytarsus inopertus Sp.Id 48
GDIP3716-22 ASAP 18 RESL BOLD:AAU1200 Paratanytarsus inopertus Sp.Id 48
GDIP3804-22 ASAP 18 RESL BOLD:AAU1200 Paratanytarsus inopertus Sp.Id 48
GDIP3803-22 ASAP 18 RESL BOLD:AAU1200 Paratanytarsus inopertus Sp.Id 48
GDIP3767-22 ASAP 18 RESL BOLD:AAU1200 Paratanytarsus inopertus Sp.Id 48
GDIP3765-22 ASAP 17 RESL BOLD:AAW4635 Paratanytarsus dissimilis Sp.Id 30
GDIP3704-22 ASAP 17 RESL BOLD:AAW4635 Paratanytarsus dissimilis Sp.Id 30
GDIP3682-22 ASAP 17 RESL BOLD:AAW4635 Paratanytarsus dissimilis Sp.Id 30
GDIP3764-22 ASAP 17 RESL BOLD:AAW4635 Paratanytarsus dissimilis Sp.Id 30
GDIP3766-22 ASAP 17 RESL BOLD:AAW4635 Paratanytarsus dissimilis Sp.Id 30
GDIP3601-22 ASAP 17 RESL BOLD:AAW4635 Paratanytarsus dissimilis Sp.Id 30
GDIP3734-22 ASAP 1 RESL BOLD:AAY3395 Polypedilum nubeculosum Sp.Id 9
GDIP3542-22 ASAP 1 RESL BOLD:AAY3395 Polypedilum nubeculosum Sp.Id 9
GDIP3702-22 ASAP 1 RESL BOLD:AAY3395 Polypedilum nubeculosum Sp.Id 9
GDIP3613-22 ASAP 1 RESL BOLD:AAY3395 Polypedilum nubeculosum Sp.Id 9
GDIP3541-22 ASAP 1 RESL BOLD:AAY3395 Polypedilum nubeculosum Sp.Id 9
GDIP3605-22 ASAP 1 RESL BOLD:AAY3395 Polypedilum nubeculosum Sp.Id 9
GDIP3735-22 ASAP 1 RESL BOLD:AAY3395 Polypedilum nubeculosum Sp.Id 9
GDIP3736-22 ASAP 1 RESL BOLD:AAY3395 Polypedilum nubeculosum Sp.Id 9
GDIP3672-22 ASAP 64 RESL BOLD:ACX5929 Polypedilum cultellatum Sp.Id 51
GDIP3730-22 ASAP 64 RESL BOLD:AAH7761 Polypedilum cultellatum Sp.Id 66
GDIP3713-22 ASAP 71 RESL BOLD:AEJ5219
GDIP3562-22 ASAP 48 RESL BOLD:AAW5643 Endochironomus albipennis Sp.Id 17
GDIP3621-22 ASAP 48 RESL BOLD:AAW5643 Endochironomus stackelbergi Sp.Id 17
GDIP3557-22 ASAP 48 RESL BOLD:AAW5643 Endochironomus sp. Sp.Id 17
GDIP3576-22 ASAP 48 RESL BOLD:AAW5643 Endochironomus sp. Sp.Id 17
GDIP3579-22 ASAP 48 RESL BOLD:AAW5643 Endochironomus sp. Sp.Id 17
GDIP3580-22 ASAP 48 RESL BOLD:AAW5643 Endochironomus sp. Sp.Id 17
GDIP3614-22 ASAP 48 RESL BOLD:AAW5643 Endochironomus sp. Sp.Id 17
GDIP3538-22 ASAP 48 RESL BOLD:AAW5643 Endochironomus sp. Sp.Id 17
GDIP3556-22 ASAP 48 RESL BOLD:AAW5643 Endochironomus sp. Sp.Id 17
GDIP3577-22 ASAP 48 RESL BOLD:AAW5643 Endochironomus albipennis Sp.Id 17
GDIP3578-22 ASAP 48 RESL BOLD:AAW5643 Endochironomus sp. Sp.Id 17
GDIP3653-22 ASAP 48 RESL BOLD:AAW5643 Endochironomus sp. Sp.Id 17
GDIP3806-22 ASAP 35 RESL BOLD:AAP5616 Tanytarsus brundini Sp.Id 72
GDIP3784-22 ASAP 25 RESL BOLD:ACR0263 Microtendipes pedellus Sp.Id 70
GDIP3532-22 ASAP 26 RESL BOLD:ACY5270 Microtendipes chloris Sp.Id 5
GDIP3533-22 ASAP 26 RESL BOLD:ACY5270 Microtendipes chloris Sp.Id 5
GDIP3723-22 ASAP 26 RESL BOLD:ACY5270 Microtendipes chloris Sp.Id 5
GDIP3785-22 ASAP 26 RESL BOLD:ACY5270 Microtendipes chloris Sp.Id 5

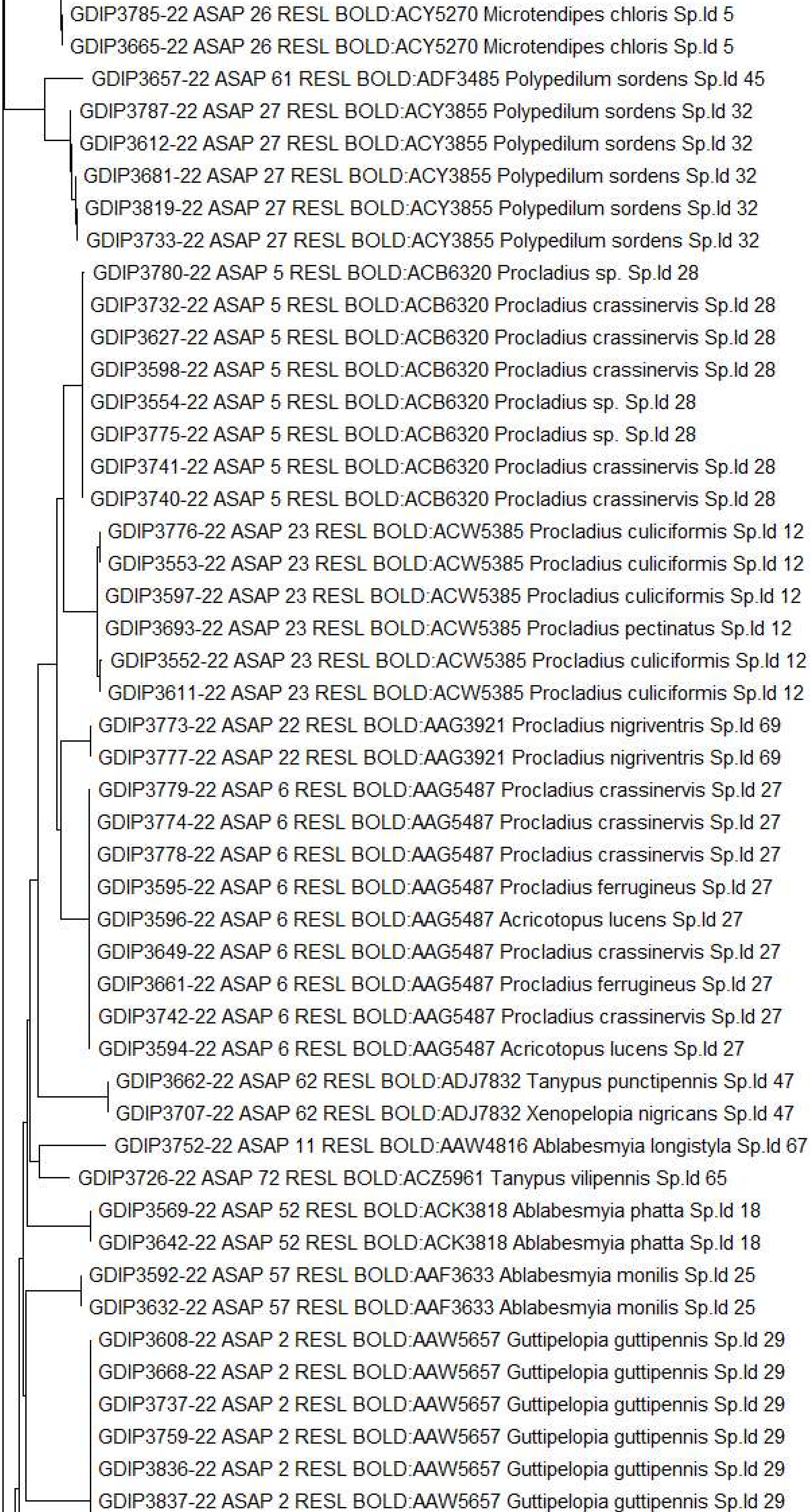

GDIP3785-22 ASAP 26 RESL BOLD:ACY5270 Microtendipes chloris Sp.Id 5
GDIP3665-22 ASAP 26 RESL BOLD:ACY5270 Microtendipes chloris Sp.Id 5
GDIP3657-22 ASAP 61 RESL BOLD:ADF3485 Polypedilum sordens Sp.Id 45
GDIP3787-22 ASAP 27 RESL BOLD:ACY3855 Polypedilum sordens Sp.Id 32
GDIP3612-22 ASAP 27 RESL BOLD:ACY3855 Polypedilum sordens Sp.Id 32
GDIP3681-22 ASAP 27 RESL BOLD:ACY3855 Polypedilum sordens Sp.Id 32
GDIP3819-22 ASAP 27 RESL BOLD:ACY3855 Polypedilum sordens Sp.Id 32
GDIP3733-22 ASAP 27 RESL BOLD:ACY3855 Polypedilum sordens Sp.Id 32
GDIP3780-22 ASAP 5 RESL BOLD:ACB6320 Procladius sp. Sp.Id 28
GDIP3732-22 ASAP 5 RESL BOLD:ACB6320 Procladius crassinervis Sp.Id 28
GDIP3627-22 ASAP 5 RESL BOLD:ACB6320 Procladius crassinervis Sp.Id 28
GDIP3598-22 ASAP 5 RESL BOLD:ACB6320 Procladius crassinervis Sp.Id 28
GDIP3554-22 ASAP 5 RESL BOLD:ACB6320 Procladius sp. Sp.Id 28
GDIP3775-22 ASAP 5 RESL BOLD:ACB6320 Procladius sp. Sp.Id 28
GDIP3741-22 ASAP 5 RESL BOLD:ACB6320 Procladius crassinervis Sp.Id 28
GDIP3740-22 ASAP 5 RESL BOLD:ACB6320 Procladius crassinervis Sp.Id 28
GDIP3776-22 ASAP 23 RESL BOLD:ACW5385 Procladius culiciformis Sp.Id 12
GDIP3553-22 ASAP 23 RESL BOLD:ACW5385 Procladius culiciformis Sp.Id 12
GDIP3597-22 ASAP 23 RESL BOLD:ACW5385 Procladius culiciformis Sp.Id 12
GDIP3693-22 ASAP 23 RESL BOLD:ACW5385 Procladius pectinatus Sp.Id 12
GDIP3552-22 ASAP 23 RESL BOLD:ACW5385 Procladius culiciformis Sp.Id 12
GDIP3611-22 ASAP 23 RESL BOLD:ACW5385 Procladius culiciformis Sp.Id 12
GDIP3773-22 ASAP 22 RESL BOLD:AAG3921 Procladius nigriventris Sp.Id 69
GDIP3777-22 ASAP 22 RESL BOLD:AAG3921 Procladius nigriventris Sp.Id 69
GDIP3779-22 ASAP 6 RESL BOLD:AAG5487 Procladius crassinervis Sp.Id 27
GDIP3774-22 ASAP 6 RESL BOLD:AAG5487 Procladius crassinervis Sp.Id 27
GDIP3778-22 ASAP 6 RESL BOLD:AAG5487 Procladius crassinervis Sp.Id 27
GDIP3595-22 ASAP 6 RESL BOLD:AAG5487 Procladius ferrugineus Sp.Id 27
GDIP3596-22 ASAP 6 RESL BOLD:AAG5487 Acricotopus lucens Sp.Id 27
GDIP3649-22 ASAP 6 RESL BOLD:AAG5487 Procladius crassinervis Sp.Id 27
GDIP3661-22 ASAP 6 RESL BOLD:AAG5487 Procladius ferrugineus Sp.Id 27
GDIP3742-22 ASAP 6 RESL BOLD:AAG5487 Procladius crassinervis Sp.Id 27
GDIP3594-22 ASAP 6 RESL BOLD:AAG5487 Acricotopus lucens Sp.Id 27
GDIP3662-22 ASAP 62 RESL BOLD:ADJ7832 Tanypus punctipennis Sp.Id 47
GDIP3707-22 ASAP 62 RESL BOLD:ADJ7832 Xenopelopia nigricans Sp.Id 47
GDIP3752-22 ASAP 11 RESL BOLD:AAW4816 Ablabesmyia longistyla Sp.Id 67
GDIP3726-22 ASAP 72 RESL BOLD:ACZ5961 Tanypus vilipennis Sp.Id 65
GDIP3569-22 ASAP 52 RESL BOLD:ACK3818 Ablabesmyia phatta Sp.Id 18
GDIP3642-22 ASAP 52 RESL BOLD:ACK3818 Ablabesmyia phatta Sp.Id 18
GDIP3592-22 ASAP 57 RESL BOLD:AAF3633 Ablabesmyia monilis Sp.Id 25
GDIP3632-22 ASAP 57 RESL BOLD:AAF3633 Ablabesmyia monilis Sp.Id 25
GDIP3608-22 ASAP 2 RESL BOLD:AAW5657 Guttipelopia guttipennis Sp.Id 29
GDIP3668-22 ASAP 2 RESL BOLD:AAW5657 Guttipelopia guttipennis Sp.Id 29
GDIP3737-22 ASAP 2 RESL BOLD:AAW5657 Guttipelopia guttipennis Sp.Id 29
GDIP3759-22 ASAP 2 RESL BOLD:AAW5657 Guttipelopia guttipennis Sp.Id 29
GDIP3836-22 ASAP 2 RESL BOLD:AAW5657 Guttipelopia guttipennis Sp.Id 29
GDIP3837-22 ASAP 2 RESL BOLD:AAW5657 Guttipelopia guttipennis Sp.Id 29

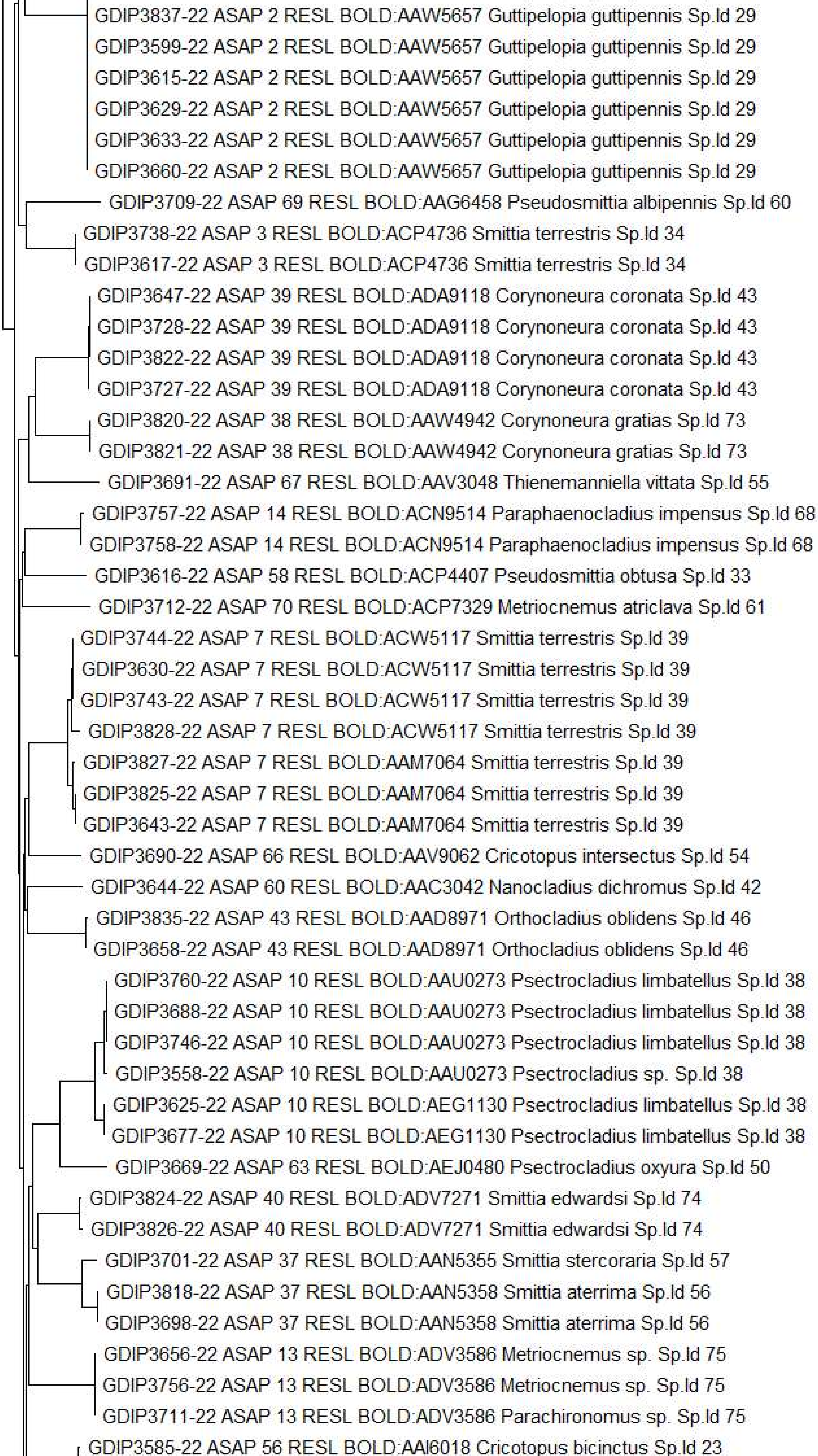
GDIP3837-22 ASAP 2 RESL BOLD:AAW5657 Guttipelopia guttipennis Sp.Id 29
GDIP3599-22 ASAP 2 RESL BOLD:AAW5657 Guttipelopia guttipennis Sp.Id 29
GDIP3615-22 ASAP 2 RESL BOLD:AAW5657 Guttipelopia guttipennis Sp.Id 29
GDIP3629-22 ASAP 2 RESL BOLD:AAW5657 Guttipelopia guttipennis Sp.Id 29
GDIP3633-22 ASAP 2 RESL BOLD:AAW5657 Guttipelopia guttipennis Sp.Id 29
GDIP3660-22 ASAP 2 RESL BOLD:AAW5657 Guttipelopia guttipennis Sp.Id 29
GDIP3709-22 ASAP 69 RESL BOLD:AAG6458 Pseudosmittia albipennis Sp.Id 60
GDIP3738-22 ASAP 3 RESL BOLD:ACP4736 Smittia terrestris Sp.Id 34
GDIP3617-22 ASAP 3 RESL BOLD:ACP4736 Smittia terrestris Sp.Id 34
GDIP3647-22 ASAP 39 RESL BOLD:ADA9118 Corynoneura coronata Sp.Id 43
GDIP3728-22 ASAP 39 RESL BOLD:ADA9118 Corynoneura coronata Sp.Id 43
GDIP3822-22 ASAP 39 RESL BOLD:ADA9118 Corynoneura coronata Sp.Id 43
GDIP3727-22 ASAP 39 RESL BOLD:ADA9118 Corynoneura coronata Sp.Id 43
GDIP3820-22 ASAP 38 RESL BOLD:AAW4942 Corynoneura gratias Sp.Id 73
GDIP3821-22 ASAP 38 RESL BOLD:AAW4942 Corynoneura gratias Sp.Id 73
GDIP3691-22 ASAP 67 RESL BOLD:AAV3048 Thienemanniella vittata Sp.Id 55
GDIP3757-22 ASAP 14 RESL BOLD:ACN9514 Paraphaenocladius impensus Sp.Id 68
GDIP3758-22 ASAP 14 RESL BOLD:ACN9514 Paraphaenocladius impensus Sp.Id 68
GDIP3616-22 ASAP 58 RESL BOLD:ACP4407 Pseudosmittia obtusa Sp.Id 33
GDIP3712-22 ASAP 70 RESL BOLD:ACP7329 Metriocnemus atriclava Sp.Id 61
GDIP3744-22 ASAP 7 RESL BOLD:ACW5117 Smittia terrestris Sp.Id 39
GDIP3630-22 ASAP 7 RESL BOLD:ACW5117 Smittia terrestris Sp.Id 39
GDIP3743-22 ASAP 7 RESL BOLD:ACW5117 Smittia terrestris Sp.Id 39
GDIP3828-22 ASAP 7 RESL BOLD:ACW5117 Smittia terrestris Sp.Id 39
GDIP3827-22 ASAP 7 RESL BOLD:AAM7064 Smittia terrestris Sp.Id 39
GDIP3825-22 ASAP 7 RESL BOLD:AAM7064 Smittia terrestris Sp.Id 39
GDIP3643-22 ASAP 7 RESL BOLD:AAM7064 Smittia terrestris Sp.Id 39
GDIP3690-22 ASAP 66 RESL BOLD:AAV9062 Cricotopus intersectus Sp.Id 54
GDIP3644-22 ASAP 60 RESL BOLD:AAC3042 Nanocladius dichromus Sp.Id 42
GDIP3835-22 ASAP 43 RESL BOLD:AAD8971 Orthocladius oblidens Sp.Id 46
GDIP3658-22 ASAP 43 RESL BOLD:AAD8971 Orthocladius oblidens Sp.Id 46
GDIP3760-22 ASAP 10 RESL BOLD:AAU0273 Psectrocladius limbatellus Sp.Id 38
GDIP3688-22 ASAP 10 RESL BOLD:AAU0273 Psectrocladius limbatellus Sp.Id 38
GDIP3746-22 ASAP 10 RESL BOLD:AAU0273 Psectrocladius limbatellus Sp.Id 38
GDIP3558-22 ASAP 10 RESL BOLD:AAU0273 Psectrocladius sp. Sp.Id 38
GDIP3625-22 ASAP 10 RESL BOLD:AEG1130 Psectrocladius limbatellus Sp.Id 38
GDIP3677-22 ASAP 10 RESL BOLD:AEG1130 Psectrocladius limbatellus Sp.Id 38
GDIP3669-22 ASAP 63 RESL BOLD:AEJ0480 Psectrocladius oxyura Sp.Id 50
GDIP3824-22 ASAP 40 RESL BOLD:ADV7271 Smittia edwardsi Sp.Id 74
GDIP3826-22 ASAP 40 RESL BOLD:ADV7271 Smittia edwardsi Sp.Id 74
GDIP3701-22 ASAP 37 RESL BOLD:AAN5355 Smittia stercoraria Sp.Id 57
GDIP3818-22 ASAP 37 RESL BOLD:AAN5358 Smittia aterrima Sp.Id 56
GDIP3698-22 ASAP 37 RESL BOLD:AAN5358 Smittia aterrima Sp.Id 56
GDIP3656-22 ASAP 13 RESL BOLD:ADV3586 Metriocnemus sp. Sp.Id 75
GDIP3756-22 ASAP 13 RESL BOLD:ADV3586 Metriocnemus sp. Sp.Id 75
GDIP3711-22 ASAP 13 RESL BOLD:ADV3586 Parachironomus sp. Sp.Id 75
GDIP3585-22 ASAP 56 RESL BOLD:AAI6018 Cricotopus bicinctus Sp.Id 23

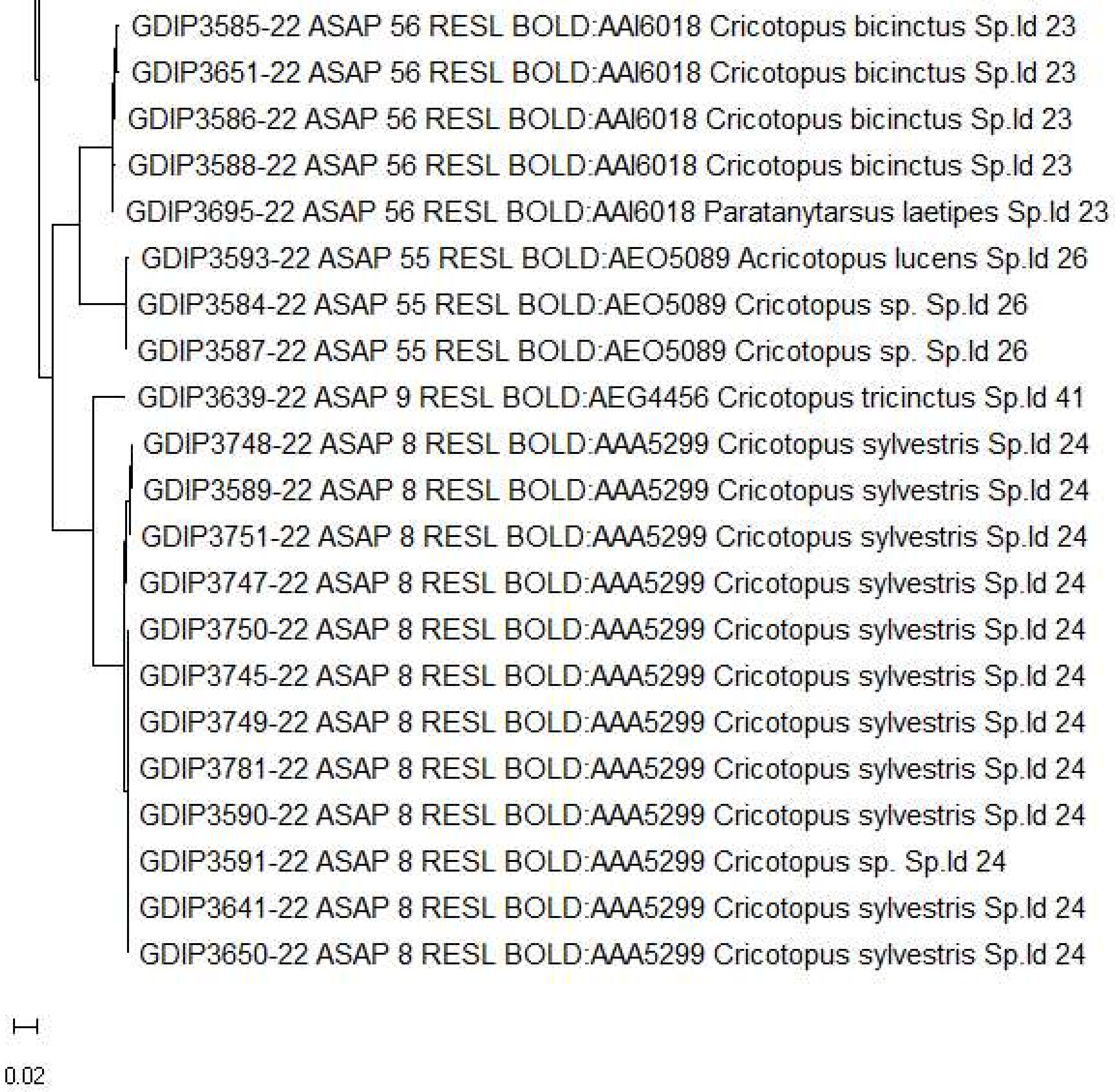
GDIP3585-22 ASAP 56 RESL BOLD:AAI6018 Cricotopus bicinctus Sp.Id 23
GDIP3651-22 ASAP 56 RESL BOLD:AAI6018 Cricotopus bicinctus Sp.Id 23
GDIP3586-22 ASAP 56 RESL BOLD:AAI6018 Cricotopus bicinctus Sp.Id 23
GDIP3588-22 ASAP 56 RESL BOLD:AAI6018 Cricotopus bicinctus Sp.Id 23
GDIP3695-22 ASAP 56 RESL BOLD:AAI6018 Paratanytarsus laetipes Sp.Id 23
GDIP3593-22 ASAP 55 RESL BOLD:AEO5089 Acricotopus lucens Sp.Id 26
GDIP3584-22 ASAP 55 RESL BOLD:AEO5089 Cricotopus sp. Sp.Id 26
GDIP3587-22 ASAP 55 RESL BOLD:AEO5089 Cricotopus sp. Sp.Id 26
GDIP3639-22 ASAP 9 RESL BOLD:AEG4456 Cricotopus tricinctus Sp.Id 41
GDIP3748-22 ASAP 8 RESL BOLD:AAA5299 Cricotopus sylvestris Sp.Id 24
GDIP3589-22 ASAP 8 RESL BOLD:AAA5299 Cricotopus sylvestris Sp.Id 24
GDIP3751-22 ASAP 8 RESL BOLD:AAA5299 Cricotopus sylvestris Sp.Id 24
GDIP3747-22 ASAP 8 RESL BOLD:AAA5299 Cricotopus sylvestris Sp.Id 24
GDIP3750-22 ASAP 8 RESL BOLD:AAA5299 Cricotopus sylvestris Sp.Id 24
GDIP3745-22 ASAP 8 RESL BOLD:AAA5299 Cricotopus sylvestris Sp.Id 24
GDIP3749-22 ASAP 8 RESL BOLD:AAA5299 Cricotopus sylvestris Sp.Id 24
GDIP3781-22 ASAP 8 RESL BOLD:AAA5299 Cricotopus sylvestris Sp.Id 24
GDIP3590-22 ASAP 8 RESL BOLD:AAA5299 Cricotopus sylvestris Sp.Id 24
GDIP3591-22 ASAP 8 RESL BOLD:AAA5299 Cricotopus sp. Sp.Id 24
GDIP3641-22 ASAP 8 RESL BOLD:AAA5299 Cricotopus sylvestris Sp.Id 24
GDIP3650-22 ASAP 8 RESL BOLD:AAA5299 Cricotopus sylvestris Sp.Id 24
0.02
