# Supplementary material for: Facing the infinity: tackling large samples of challenging Chironomidae (Diptera) with an integrative approach
Source: PeerJ. 2023 May 22;11:e15336. doi: 10.7717/peerj.15336 (PMC10211366; doi:10.7717/peerj.15336)
Supplement: Supplemental Information 3 — Neighbor-Joining tree including 19,045 public COI sequences of chironomids collected in Europe. Downloaded from BOLD. [file peerj-11-15336-s003.pdf]

BOLD TaxonID Tree

Title	:	Tree Result - DS-CHRTR
Date	:	05-Aug-2022
Data Type	:	Nucleotide
Distance Model	:	Kimura 2 Parameter
Marker	:	COI-5P
Colourization	:	Barcode Cluster (BIN)
Label	:	Sample ID
Label	:	Identifier
Label	:	Identification Method
Label	:	Taxon
Label	:	Barcode Cluster (BIN)
Filter	:	length > 200bp only
Filter	:	exclude records flagged as misidentifications
Filter	:	exclude records with stop codons
Filter	:	exclude contaminants
Sequence Count	:	19045
Species count	:	1026
Genus count	:	152
Family count	:	1
Unidentified	:	3952
BIN Count	:	1613

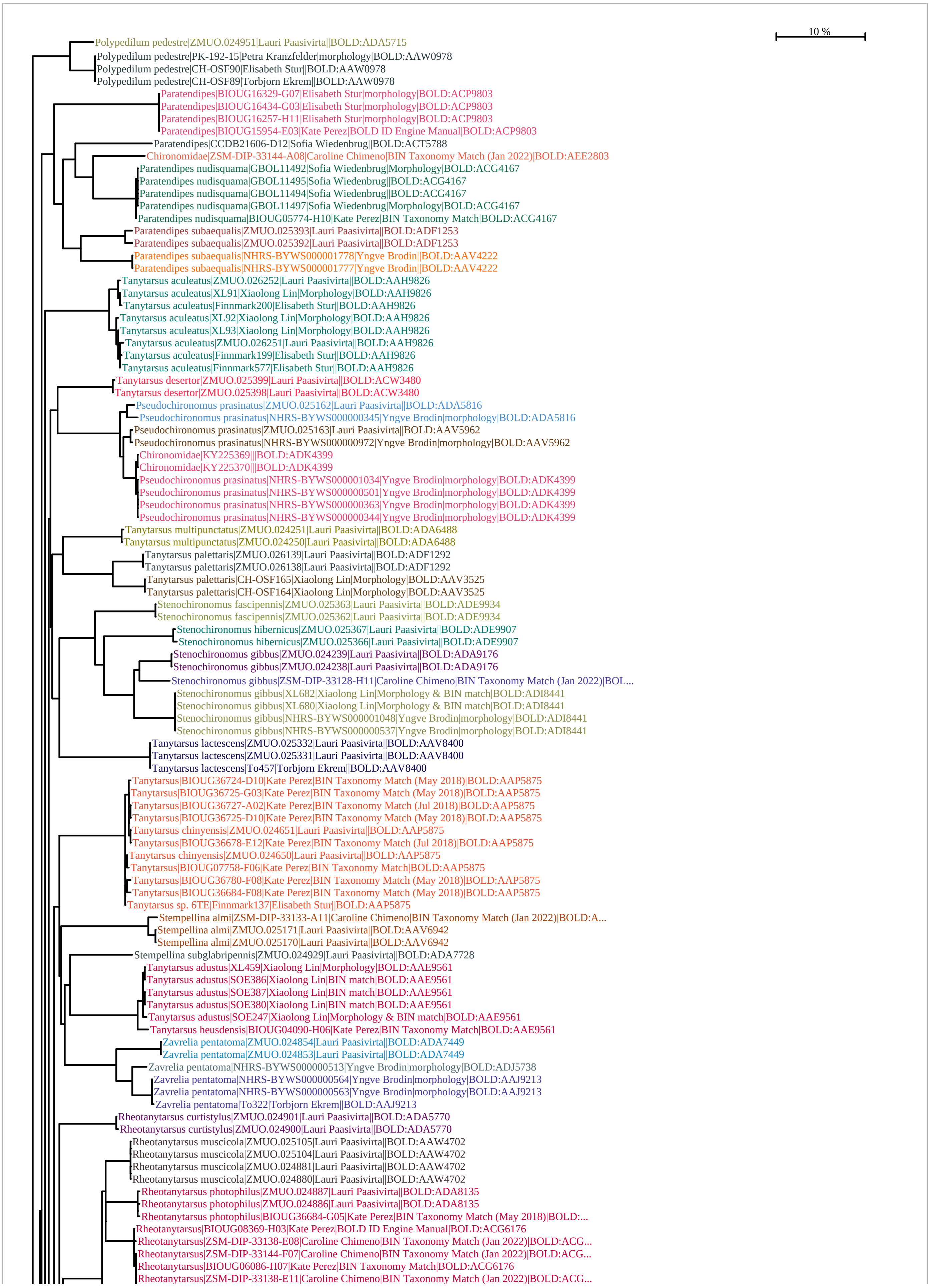
10 %
Polypedilum pedestre|ZMUO.024951|Lauri Paasivirta||BOLD:ADA5715
Polypedilum pedestre|PK-192-15|Petra Kranzfelder|morphology|BOLD:AAW0978
Polypedilum pedestre|CH-OSF90|Elisabeth Stur||BOLD:AAW0978
Polypedilum pedestre|CH-OSF89|Torbjorn Ekrem||BOLD:AAW0978
Paratendipes|BIOUG16329-G07|Elisabeth Stur|morphology|BOLD:ACP9803
Paratendipes|BIOUG16434-G03|Elisabeth Stur|morphology|BOLD:ACP9803
Paratendipes|BIOUG16257-H11|Elisabeth Stur|morphology|BOLD:ACP9803
Paratendipes|BIOUG15954-E03|Kate Perez|BOLD ID Engine Manual|BOLD:ACP9803
Paratendipes|CCDB21606-D12|Sofia Wiedenbrug||BOLD:ACT5788
Chironomidae|ZSM-DIP-33144-A08|Caroline Chimeno|BIN Taxonomy Match (Jan 2022)|BOLD:AEE2803
Paratendipes nudisquama|GBOL11492|Sofia Wiedenbrug|Morphology|BOLD:ACG4167
Paratendipes nudisquama|GBOL11495|Sofia Wiedenbrug||BOLD:ACG4167
Paratendipes nudisquama|GBOL11494|Sofia Wiedenbrug||BOLD:ACG4167
Paratendipes nudisquama|GBOL11497|Sofia Wiedenbrug|Morphology|BOLD:ACG4167
Paratendipes nudisquama|BIOUG05774-H10|Kate Perez|BIN Taxonomy Match|BOLD:ACG4167
Paratendipes subaequalis|ZMUO.025393|Lauri Paasivirta||BOLD:ADF1253
Paratendipes subaequalis|ZMUO.025392|Lauri Paasivirta||BOLD:ADF1253
Paratendipes subaequalis|NHRS-BYWS000001778|Yngve Brodin||BOLD:AAV4222
Paratendipes subaequalis|NHRS-BYWS000001777|Yngve Brodin||BOLD:AAV4222
Tanytarsus aculeatus|ZMUO.026252|Lauri Paasivirta||BOLD:AAH9826
Tanytarsus aculeatus|XL91|Xiaolong Lin|Morphology|BOLD:AAH9826
Tanytarsus aculeatus|Finnmark200|Elisabeth Stur||BOLD:AAH9826
Tanytarsus aculeatus|XL92|Xiaolong Lin|Morphology|BOLD:AAH9826
Tanytarsus aculeatus|XL93|Xiaolong Lin|Morphology|BOLD:AAH9826
Tanytarsus aculeatus|ZMUO.026251|Lauri Paasivirta||BOLD:AAH9826
Tanytarsus aculeatus|Finnmark199|Elisabeth Stur||BOLD:AAH9826
Tanytarsus aculeatus|Finnmark577|Elisabeth Stur||BOLD:AAH9826
Tanytarsus desertor|ZMUO.025399|Lauri Paasivirta||BOLD:ACW3480
Tanytarsus desertor|ZMUO.025398|Lauri Paasivirta||BOLD:ACW3480
Pseudochironomus prasinatus|ZMUO.025162|Lauri Paasivirta||BOLD:ADA5816
Pseudochironomus prasinatus|NHRS-BYWS000000345|Yngve Brodin|morphology|BOLD:ADA5816
Pseudochironomus prasinatus|ZMUO.025163|Lauri Paasivirta||BOLD:AAV5962
Pseudochironomus prasinatus|NHRS-BYWS000000972|Yngve Brodin|morphology|BOLD:AAV5962
Chironomidae|KY225369|||BOLD:ADK4399
Chironomidae|KY225370|||BOLD:ADK4399
Pseudochironomus prasinatus|NHRS-BYWS000001034|Yngve Brodin|morphology|BOLD:ADK4399
Pseudochironomus prasinatus|NHRS-BYWS000000501|Yngve Brodin|morphology|BOLD:ADK4399
Pseudochironomus prasinatus|NHRS-BYWS000000363|Yngve Brodin|morphology|BOLD:ADK4399
Pseudochironomus prasinatus|NHRS-BYWS000000344|Yngve Brodin|morphology|BOLD:ADK4399
Tanytarsus multipunctatus|ZMUO.024251|Lauri Paasivirta||BOLD:ADA6488
Tanytarsus multipunctatus|ZMUO.024250|Lauri Paasivirta||BOLD:ADA6488
Tanytarsus palettaris|ZMUO.026139|Lauri Paasivirta||BOLD:ADF1292
Tanytarsus palettaris|ZMUO.026138|Lauri Paasivirta||BOLD:ADF1292
Tanytarsus palettaris|CH-OSF165|Xiaolong Lin|Morphology|BOLD:AAV3525
Tanytarsus palettaris|CH-OSF164|Xiaolong Lin|Morphology|BOLD:AAV3525
Stenochironomus fascipennis|ZMUO.025363|Lauri Paasivirta||BOLD:ADE9934
Stenochironomus fascipennis|ZMUO.025362|Lauri Paasivirta||BOLD:ADE9934
Stenochironomus hibernicus|ZMUO.025367|Lauri Paasivirta||BOLD:ADE9907
Stenochironomus hibernicus|ZMUO.025366|Lauri Paasivirta||BOLD:ADE9907
Stenochironomus gibbus|ZMUO.024239|Lauri Paasivirta||BOLD:ADA9176
Stenochironomus gibbus|ZMUO.024238|Lauri Paasivirta||BOLD:ADA9176
Stenochironomus gibbus|ZSM-DIP-33128-H11|Caroline Chimeno|BIN Taxonomy Match (Jan 2022)|BOL...
Stenochironomus gibbus|XL682|Xiaolong Lin|Morphology & BIN match|BOLD:ADI8441
Stenochironomus gibbus|XL680|Xiaolong Lin|Morphology & BIN match|BOLD:ADI8441
Stenochironomus gibbus|NHRS-BYWS000001048|Yngve Brodin|morphology|BOLD:ADI8441
Stenochironomus gibbus|NHRS-BYWS000000537|Yngve Brodin|morphology|BOLD:ADI8441
Tanytarsus lactescens|ZMUO.025332|Lauri Paasivirta||BOLD:AAV8400
Tanytarsus lactescens|ZMUO.025331|Lauri Paasivirta||BOLD:AAV8400
Tanytarsus lactescens|To457|Torbjorn Ekrem||BOLD:AAV8400
Tanytarsus|BIOUG36724-D10|Kate Perez|BIN Taxonomy Match (May 2018)|BOLD:AAP5875
Tanytarsus|BIOUG36725-G03|Kate Perez|BIN Taxonomy Match (May 2018)|BOLD:AAP5875
Tanytarsus|BIOUG36727-A02|Kate Perez|BIN Taxonomy Match (Jul 2018)|BOLD:AAP5875
Tanytarsus|BIOUG36725-D10|Kate Perez|BIN Taxonomy Match (May 2018)|BOLD:AAP5875
Tanytarsus chinyensis|ZMUO.024651|Lauri Paasivirta||BOLD:AAP5875
Tanytarsus|BIOUG36678-E12|Kate Perez|BIN Taxonomy Match (Jul 2018)|BOLD:AAP5875
Tanytarsus chinyensis|ZMUO.024650|Lauri Paasivirta||BOLD:AAP5875
Tanytarsus|BIOUG07758-F06|Kate Perez|BIN Taxonomy Match|BOLD:AAP5875
Tanytarsus|BIOUG36780-F08|Kate Perez|BIN Taxonomy Match (May 2018)|BOLD:AAP5875
Tanytarsus|BIOUG36684-F08|Kate Perez|BIN Taxonomy Match (May 2018)|BOLD:AAP5875
Tanytarsus sp. 6TE|Finnmark137|Elisabeth Stur||BOLD:AAP5875
Stempellina almi|ZSM-DIP-33133-A11|Caroline Chimeno|BIN Taxonomy Match (Jan 2022)|BOLD:A...
Stempellina almi|ZMUO.025171|Lauri Paasivirta||BOLD:AAV6942
Stempellina almi|ZMUO.025170|Lauri Paasivirta||BOLD:AAV6942
Stempellina subglabripennis|ZMUO.024929|Lauri Paasivirta||BOLD:ADA7728
Tanytarsus adustus|XL459|Xiaolong Lin|Morphology|BOLD:AAE9561
Tanytarsus adustus|SOE386|Xiaolong Lin|BIN match|BOLD:AAE9561
Tanytarsus adustus|SOE387|Xiaolong Lin|BIN match|BOLD:AAE9561
Tanytarsus adustus|SOE380|Xiaolong Lin|BIN match|BOLD:AAE9561
Tanytarsus adustus|SOE247|Xiaolong Lin|Morphology & BIN match|BOLD:AAE9561
Tanytarsus heusdensis|BIOUG04090-H06|Kate Perez|BIN Taxonomy Match|BOLD:AAE9561
Zavrelia pentatoma|ZMUO.024854|Lauri Paasivirta||BOLD:ADA7449
Zavrelia pentatoma|ZMUO.024853|Lauri Paasivirta||BOLD:ADA7449
Zavrelia pentatoma|NHRS-BYWS000000513|Yngve Brodin|morphology|BOLD:ADJ5738
Zavrelia pentatoma|NHRS-BYWS000000564|Yngve Brodin|morphology|BOLD:AAJ9213
Zavrelia pentatoma|NHRS-BYWS000000563|Yngve Brodin|morphology|BOLD:AAJ9213
Zavrelia pentatoma|To322|Torbjorn Ekrem||BOLD:AAJ9213
Rheotanytarsus curtistylus|ZMUO.024901|Lauri Paasivirta||BOLD:ADA5770
Rheotanytarsus curtistylus|ZMUO.024900|Lauri Paasivirta||BOLD:ADA5770
Rheotanytarsus muscicola|ZMUO.025105|Lauri Paasivirta||BOLD:AAW4702
Rheotanytarsus muscicola|ZMUO.025104|Lauri Paasivirta||BOLD:AAW4702
Rheotanytarsus muscicola|ZMUO.024881|Lauri Paasivirta||BOLD:AAW4702
Rheotanytarsus muscicola|ZMUO.024880|Lauri Paasivirta||BOLD:AAW4702
Rheotanytarsus photophilus|ZMUO.024887|Lauri Paasivirta||BOLD:ADA8135
Rheotanytarsus photophilus|ZMUO.024886|Lauri Paasivirta||BOLD:ADA8135
Rheotanytarsus photophilus|BIOUG36684-G05|Kate Perez|BIN Taxonomy Match (May 2018)|BOLD:...
Rheotanytarsus|BIOUG08369-H03|Kate Perez|BOLD ID Engine Manual|BOLD:ACG6176
Rheotanytarsus|ZSM-DIP-33138-E08|Caroline Chimeno|BIN Taxonomy Match (Jan 2022)|BOLD:ACG...
Rheotanytarsus|ZSM-DIP-33144-F07|Caroline Chimeno|BIN Taxonomy Match (Jan 2022)|BOLD:ACG...
Rheotanytarsus|BIOUG06086-H07|Kate Perez|BIN Taxonomy Match|BOLD:ACG6176
Rheotanytarsus|ZSM-DIP-33138-E11|Caroline Chimeno|BIN Taxonomy Match (Jan 2022)|BOLD:ACG...

Rheotanytarsus|ZSM-DIP-33144-F07|Caroline Chimeno|BIN Taxonomy Match (Jan 2022)|BOLD:ACG...
Rheotanytarsus|BIOUG06086-H07|Kate Perez|BIN Taxonomy Match|BOLD:ACG6176
Rheotanytarsus|ZSM-DIP-33138-E11|Caroline Chimeno|BIN Taxonomy Match (Jan 2022)|BOLD:ACG...
Rheotanytarsus|BIOUG08174-D11|Kate Perez|BIN Taxonomy Match|BOLD:ACG6176
Rheotanytarsus pentapoda|ZMUO.024702|Lauri Paasivirta||BOLD:ACL5271
Rheotanytarsus|BIOUG15952-B08|Kate Perez|BOLD ID Engine Manual|BOLD:ACQ0249
Rheotanytarsus|BIOUG16258-A01|Kate Perez|BOLD ID Engine Manual|BOLD:ACQ0249
Rheotanytarsus|BIOUG15967-C12|Kate Perez|BOLD ID Engine Manual|BOLD:ACQ0249
Rheotanytarsus|BIOUG15951-C02|Kate Perez|BOLD ID Engine Manual|BOLD:ACQ0251
Rheotanytarsus|BIOUG15966-F11|Kate Perez|BOLD ID Engine Manual|BOLD:ACQ0251
Rheotanytarsus|BIOUG16329-H11|Kate Perez|BOLD ID Engine Manual|BOLD:ACQ0251
Rheotanytarsus|BIOUG15967-G10|Kate Perez|BOLD ID Engine Manual|BOLD:ACQ0250
Rheotanytarsus illiesi|Finnmark119|Torbjorn Ekrem||BOLD:AAM9415
Rheotanytarsus pentapoda|BIOUG16477-B04|Kate Perez|Tree based identification
Rheotanytarsus pentapoda|TRD-CH409|Elisabeth Stur|morphology|BOLD:ABZ1633
Rheotanytarsus pentapoda|EJV-20110147|Lauri Paasivirta||BOLD:ABZ1633
Rheotanytarsus pentapoda|Finnmark720|Elisabeth Stur||BOLD:ACE3020
Rheotanytarsus pentapoda|Finnmark716|Elisabeth Stur||BOLD:ACE3020
Rheotanytarsus pentapoda|Finnmark243|Elisabeth Stur||BOLD:ACE3020
Rheotanytarsus pentapoda|BIOUG15951-G02|Kate Perez|BIN Taxonomy Match|BOLD:ACE3020
Rheotanytarsus pentapoda|BIOUG15950-D06|Kate Perez|BIN Taxonomy Match|BOLD:ACE3020
Rheotanytarsus pentapoda|BIOUG16433-E01|Kate Perez|BIN Taxonomy Match|BOLD:ACE3020
Rheotanytarsus pentapoda|BIOUG16297-A01|Kate Perez|BIN Taxonomy Match|BOLD:ACE3020
Rheotanytarsus pentapoda|BIOUG16396-B10|Kate Perez|BIN Taxonomy Match|BOLD:ACE3020
Rheotanytarsus pentapoda|BIOUG16198-C06|Kate Perez|BIN Taxonomy Match|BOLD:ACE3020
Rheotanytarsus pentapoda|BIOUG16198-D01|Kate Perez|BIN Taxonomy Match|BOLD:ACE3020
Rheotanytarsus pentapoda|BIOUG15951-F02|Kate Perez|BIN Taxonomy Match|BOLD:ACE3020
Rheotanytarsus pentapoda|BIOUG16330-A02|Kate Perez|BIN Taxonomy Match|BOLD:ACE3020
Rheotanytarsus pentapoda|BIOUG16597-H08|Kate Perez|BIN Taxonomy Match|BOLD:ACE3020
Rheotanytarsus pentapoda|BIOUG15951-A12|Kate Perez|BIN Taxonomy Match|BOLD:ACE3020
Rheotanytarsus pentapoda|BIOUG15948-F02|Kate Perez|BIN Taxonomy Match|BOLD:ACE3020
Rheotanytarsus pentapoda|BIOUG15965-A10|Kate Perez|BIN Taxonomy Match|BOLD:ACE3020
Rheotanytarsus pentapoda|BIOUG16433-C02|Kate Perez|BIN Taxonomy Match|BOLD:ACE3020
Rheotanytarsus pentapoda|BIOUG15965-H07|Kate Perez|BIN Taxonomy Match|BOLD:ACE3020
Rheotanytarsus pentapoda|ZMUO.024703|Lauri Paasivirta||BOLD:AAD5143
Rheotanytarsus pentapoda|EJV-20110148|Lauri Paasivirta||BOLD:AAD5143
Rheotanytarsus pentapoda|EJV-20110146|Lauri Paasivirta||BOLD:AAD5143
Tanytarsus signatus|To83|Xiaolong Lin|Morphology|BOLD:ACG9673
Tanytarsus signatus|CH-eik75|Elisabeth Stur||BOLD:ACG9673
Tanytarsus signatus|CCDB22605-E06|Sofia Wiedenbrug||BOLD:AAW4817
Tanytarsus signatus|ZSM-DIP-33129-C04|Caroline Chimeno|BIN Taxonomy Match (Jan 2022)|BOLD ...
Tanytarsus signatus|TRD-CH411|Elisabeth Stur|morphology|BOLD:AAW4817
Tanytarsus signatus|TRD-CH389|Elisabeth Stur|morphology|BOLD:AAW4817
Tanytarsus signatus|ZMUO.024956|Lauri Paasivirta||BOLD:AAW4817
Tanytarsus signatus|ZSM-DIP-33129-A06|Caroline Chimeno|BIN Taxonomy Match (Jan 2022)|BOLD ...
Tanytarsus signatus|BIOUG42666-G03|Kate Perez|BIN Taxonomy Match below Phylum (Jun 2019)|BO...
Tanytarsus signatus|TRD-CH313|Elisabeth Stur||BOLD:AAW4817
Tanytarsus signatus|TRD-CH388|Elisabeth Stur|morphology|BOLD:AAW4817
Tanytarsus signatus|EJV-20110150|Lauri Paasivirta||BOLD:AAW4817
Tanytarsus signatus|ZSM-DIP-33129-B04|Caroline Chimeno|BIN Taxonomy Match (Jan 2022)|BOLD ...
Tanytarsus signatus|ZSM-DIP-33129-A12|Caroline Chimeno|BIN Taxonomy Match (Jan 2022)|BOLD ...
Tanytarsus signatus|ZSM-DIP-33129-B06|Caroline Chimeno|BIN Taxonomy Match (Jan 2022)|BOLD ...
Tanytarsus signatus|EJV-20110151|Lauri Paasivirta||BOLD:AAW4817
Tanytarsus signatus|EJV-20110152|Lauri Paasivirta||BOLD:AAW4817
Tanytarsus signatus|EJV-20110149|Lauri Paasivirta||BOLD:AAW4817
Rheotanytarsus ringei|Finnmark183|Torbjorn Ekrem||BOLD:AAF7635
Rheotanytarsus ringei|BIOUG16256-C08|Kate Perez|BIN Taxonomy Match|BOLD:AAF7635
Rheotanytarsus ringei|BIOUG16331-D02|Kate Perez|BIN Taxonomy Match|BOLD:AAF7635
Rheotanytarsus ringei|BIOUG16298-D08|Kate Perez|BIN Taxonomy Match|BOLD:AAF7635
Rheotanytarsus ringei|BIOUG16333-G07|Kate Perez|BIN Taxonomy Match|BOLD:AAF7635
Rheotanytarsus ringei|BIOUG16298-H07|Kate Perez|BIN Taxonomy Match|BOLD:AAF7635
Rheotanytarsus ringei|BIOUG16297-H02|Kate Perez|BIN Taxonomy Match|BOLD:AAF7635
Rheotanytarsus ringei|BIOUG16257-A12|Kate Perez|BIN Taxonomy Match|BOLD:AAF7635
Rheotanytarsus ringei|BIOUG16333-E11|Kate Perez|BIN Taxonomy Match|BOLD:AAF7635
Rheotanytarsus ringei|BIOUG16331-E08|Kate Perez|BIN Taxonomy Match|BOLD:AAF7635
Rheotanytarsus ringei|BIOUG15950-D10|Kate Perez|BIN Taxonomy Match|BOLD:AAF7635
Rheotanytarsus ringei|BIOUG15905-A04|Kate Perez|BIN Taxonomy Match|BOLD:AAF7635
Rheotanytarsus ringei|EJV-20110132|Lauri Paasivirta||BOLD:AAF7635
Rheotanytarsus ringei|EJV-20110131|Lauri Paasivirta||BOLD:AAF7635
Rheotanytarsus ringei|BIOUG16261-B06|Kate Perez|BIN Taxonomy Match|BOLD:AAF7635
Rheotanytarsus ringei|BIOUG16328-G07|Kate Perez|BIN Taxonomy Match|BOLD:AAF7635
Rheotanytarsus ringei|BIOUG16297-B11|Kate Perez|BIN Taxonomy Match|BOLD:AAF7635
Rheotanytarsus ringei|BIOUG16432-D11|Kate Perez|BIN Taxonomy Match|BOLD:AAF7635
Rheotanytarsus ringei|BIOUG16328-C03|Kate Perez|BIN Taxonomy Match|BOLD:AAF7635
Rheotanytarsus ringei|BIOUG16299-B03|Kate Perez|BIN Taxonomy Match|BOLD:AAF7635
Rheotanytarsus ringei|BIOUG16396-B12|Kate Perez|BIN Taxonomy Match|BOLD:AAF7635
Rheotanytarsus ringei|BIOUG16296-H04|Kate Perez|BIN Taxonomy Match|BOLD:AAF7635
Rheotanytarsus ringei|BIOUG16296-C08|Kate Perez|BIN Taxonomy Match|BOLD:AAF7635
Rheotanytarsus ringei|BIOUG16259-A02|Kate Perez|BIN Taxonomy Match|BOLD:AAF7635
Rheotanytarsus ringei|BIOUG15948-A07|Kate Perez|BIN Taxonomy Match|BOLD:AAF7635
Rheotanytarsus ringei|TRD-CH175|Elisabeth Stur||BOLD:AAF7635
Rheotanytarsus ringei|BIOUG16329-F05|Kate Perez|BIN Taxonomy Match|BOLD:AAF7635
Rheotanytarsus ringei|BIOUG16329-G06|Kate Perez|BIN Taxonomy Match|BOLD:AAF7635
Rheotanytarsus ringei|BIOUG16329-C11|Kate Perez|BIN Taxonomy Match|BOLD:AAF7635
Rheotanytarsus ringei|BIOUG15963-E07|Kate Perez|BIN Taxonomy Match|BOLD:AAF7635
Rheotanytarsus ringei|BIOUG16261-A10|Kate Perez|BIN Taxonomy Match|BOLD:AAF7635
Rheotanytarsus ringei|BIOUG16298-B09|Kate Perez|BIN Taxonomy Match|BOLD:AAF7635
Rheotanytarsus ringei|BIOUG16296-D10|Kate Perez|BIN Taxonomy Match|BOLD:AAF7635
Rheotanytarsus ringei|BIOUG16257-F11|Kate Perez|BIN Taxonomy Match|BOLD:AAF7635
Rheotanytarsus ringei|BIOUG16330-B11|Kate Perez|BIN Taxonomy Match|BOLD:AAF7635
Rheotanytarsus ringei|BIOUG16332-D06|Kate Perez|BIN Taxonomy Match|BOLD:AAF7635
Rheotanytarsus ringei|BIOUG16333-C09|Kate Perez|BIN Taxonomy Match|BOLD:AAF7635
Rheotanytarsus ringei|BIOUG16258-D01|Kate Perez|BIN Taxonomy Match|BOLD:AAF7635
Rheotanytarsus ringei|BIOUG16198-E09|Kate Perez|BIN Taxonomy Match|BOLD:AAF7635
Rheotanytarsus ringei|BIOUG16331-F06|Kate Perez|BIN Taxonomy Match|BOLD:AAF7635
Rheotanytarsus ringei|BIOUG16261-E12|Kate Perez|BIN Taxonomy Match|BOLD:AAF7635
Rheotanytarsus ringei|BIOUG15905-H03|Kate Perez|BIN Taxonomy Match|BOLD:AAF7635
Rheotanytarsus ringei|BIOUG16328-A12|Kate Perez|BIN Taxonomy Match|BOLD:AAF7635
Rheotanytarsus ringei|BIOUG15964-G07|Kate Perez|BIN Taxonomy Match|BOLD:AAF7635
Rheotanytarsus ringei|BIOUG16298-E04|Kate Perez|BIN Taxonomy Match|BOLD:AAF7635
Rheotanytarsus ringei|BIOUG16328-E08|Kate Perez|BIN Taxonomy Match|BOLD:AAF7635
Rheotanytarsus ringei|BIOUG16299-C02|Kate Perez|BIN Taxonomy Match|BOLD:AAF7635
Rheotanytarsus ringei|BIOUG16260-F06|Kate Perez|BIN Taxonomy Match|BOLD:AAF7635

[illegible]
Rheotanytarsus ringei|BIOUG16328-E08|Kate Perez|BIN Taxonomy Match|BOLD:AAF7635
Rheotanytarsus ringei|BIOUG16299-C02|Kate Perez|BIN Taxonomy Match|BOLD:AAF7635
Rheotanytarsus ringei|BIOUG16260-E06|Kate Perez|BIN Taxonomy Match|BOLD:AAF7635
Rheotanytarsus ringei|BIOUG16328-H03|Kate Perez|BIN Taxonomy Match|BOLD:AAF7635
Rheotanytarsus ringei|BIOUG16328-F04|Kate Perez|BIN Taxonomy Match|BOLD:AAF7635
Rheotanytarsus ringei|BIOUG16332-D02|Kate Perez|BIN Taxonomy Match|BOLD:AAF7635
Rheotanytarsus ringei|BIOUG16256-F08|Kate Perez|BIN Taxonomy Match|BOLD:AAF7635
Rheotanytarsus ringei|BIOUG16261-H06|Kate Perez|BIN Taxonomy Match|BOLD:AAF7635
Rheotanytarsus ringei|BIOUG16299-A08|Kate Perez|BIN Taxonomy Match|BOLD:AAF7635
Rheotanytarsus ringei|BIOUG16198-G11|Kate Perez|BIN Taxonomy Match|BOLD:AAF7635
Rheotanytarsus ringei|BIOUG16257-D04|Kate Perez|BIN Taxonomy Match|BOLD:AAF7635
Rheotanytarsus ringei|BIOUG16261-F03|Kate Perez|BIN Taxonomy Match|BOLD:AAF7635
Rheotanytarsus ringei|BIOUG16260-B12|Kate Perez|BIN Taxonomy Match|BOLD:AAF7635
Rheotanytarsus ringei|BIOUG16260-F10|Kate Perez|BIN Taxonomy Match|BOLD:AAF7635
Rheotanytarsus ringei|BIOUG16330-F12|Kate Perez|BIN Taxonomy Match|BOLD:AAF7635
Rheotanytarsus ringei|BIOUG16479-H08|Kate Perez|BIN Taxonomy Match|BOLD:AAF7635
Rheotanytarsus ringei|BIOUG16296-D08|Kate Perez|BIN Taxonomy Match|BOLD:AAF7635
Rheotanytarsus ringei|BIOUG16259-H05|Kate Perez|BIN Taxonomy Match|BOLD:AAF7635
Rheotanytarsus ringei|BIOUG16298-G02|Kate Perez|BIN Taxonomy Match|BOLD:AAF7635
Rheotanytarsus ringei|BIOUG16261-E07|Kate Perez|BIN Taxonomy Match|BOLD:AAF7635
Rheotanytarsus ringei|BIOUG15953-E07|Kate Perez|BIN Taxonomy Match|BOLD:AAF7635
Rheotanytarsus ringei|BIOUG15952-D09|Kate Perez|BIN Taxonomy Match|BOLD:AAF7635
Rheotanytarsus ringei|BIOUG15948-D11|Kate Perez|BIN Taxonomy Match|BOLD:AAF7635
Rheotanytarsus ringei|ZSM-DIP-33138-G09|Caroline Chimeno|BIN Taxonomy Match (Jan 2022)|BOLD...
Rheotanytarsus ringei|ZSM-DIP-33138-G08|Caroline Chimeno|BIN Taxonomy Match (Jan 2022)|BOLD...
Rheotanytarsus ringei|To41|Torbjorn Ekrem||BOLD:AAF7635
Rheotanytarsus ringei|Finnmark179|Torbjorn Ekrem||BOLD:AAF7635
Rheotanytarsus ringei|BIOUG16328-H02|Kate Perez|BIN Taxonomy Match|BOLD:AAF7635
Rheotanytarsus ringei|BIOUG16329-B02|Kate Perez|BIN Taxonomy Match|BOLD:AAF7635
Rheotanytarsus ringei|BIOUG16329-D03|Kate Perez|BIN Taxonomy Match|BOLD:AAF7635
Rheotanytarsus ringei|BIOUG16329-F07|Kate Perez|BIN Taxonomy Match|BOLD:AAF7635
Rheotanytarsus ringei|BIOUG16198-F10|Kate Perez|BIN Taxonomy Match|BOLD:AAF7635
Rheotanytarsus ringei|BIOUG15965-H02|Kate Perez|BIN Taxonomy Match|BOLD:AAF7635
Rheotanytarsus ringei|BIOUG16258-C04|Kate Perez|BIN Taxonomy Match|BOLD:AAF7635
Rheotanytarsus ringei|BIOUG16298-H08|Kate Perez|BIN Taxonomy Match|BOLD:AAF7635
Rheotanytarsus ringei|BIOUG16297-F12|Kate Perez|BIN Taxonomy Match|BOLD:AAF7635
Rheotanytarsus ringei|BIOUG15954-A01|Kate Perez|BIN Taxonomy Match|BOLD:AAF7635
Rheotanytarsus ringei|BIOUG15905-B04|Kate Perez|BIN Taxonomy Match|BOLD:AAF7635
Rheotanytarsus ringei|BIOUG15895-C04|Kate Perez|BIN Taxonomy Match|BOLD:AAF7635
Rheotanytarsus ringei|BIOUG16258-C11|Kate Perez|BIN Taxonomy Match|BOLD:AAF7635
Rheotanytarsus ringei|BIOUG16261-D03|Kate Perez|BIN Taxonomy Match|BOLD:AAF7635
Rheotanytarsus ringei|BIOUG16259-B05|Kate Perez|BIN Taxonomy Match|BOLD:AAF7635
Rheotanytarsus ringei|BIOUG16333-B05|Kate Perez|BIN Taxonomy Match|BOLD:AAF7635
Rheotanytarsus ringei|BIOUG16330-E08|Kate Perez|BIN Taxonomy Match|BOLD:AAF7635
Rheotanytarsus ringei|BIOUG16333-C02|Kate Perez|BIN Taxonomy Match|BOLD:AAF7635
Rheotanytarsus ringei|BIOUG15947-A03|Kate Perez|BIN Taxonomy Match|BOLD:AAF7635
Rheotanytarsus ringei|BIOUG16332-A08|Kate Perez|BIN Taxonomy Match|BOLD:AAF7635
Rheotanytarsus ringei|BIOUG15948-B11|Kate Perez|BIN Taxonomy Match|BOLD:AAF7635
Rheotanytarsus ringei|BIOUG15949-G10|Kate Perez|BIN Taxonomy Match|BOLD:AAF7635
Rheotanytarsus ringei|BIOUG16331-B09|Kate Perez|BIN Taxonomy Match|BOLD:AAF7635
Rheotanytarsus ringei|BIOUG16256-F07|Kate Perez|BIN Taxonomy Match|BOLD:AAF7635
Rheotanytarsus ringei|BIOUG16298-E12|Kate Perez|BIN Taxonomy Match|BOLD:AAF7635
Rheotanytarsus ringei|BIOUG15947-D06|Kate Perez|BIN Taxonomy Match|BOLD:AAF7635
Rheotanytarsus ringei|BIOUG16396-C09|Kate Perez|BIN Taxonomy Match|BOLD:AAF7635
Rheotanytarsus ringei|BIOUG16260-A10|Kate Perez|BIN Taxonomy Match|BOLD:AAF7635
Rheotanytarsus ringei|BIOUG16298-G10|Kate Perez|BIN Taxonomy Match|BOLD:AAF7635
Rheotanytarsus ringei|BIOUG16260-C03|Kate Perez|BIN Taxonomy Match|BOLD:AAF7635
Rheotanytarsus ringei|BIOUG15965-B09|Kate Perez|BIN Taxonomy Match|BOLD:AAF7635
Rheotanytarsus ringei|BIOUG16333-A05|Kate Perez|BIN Taxonomy Match|BOLD:AAF7635
Rheotanytarsus ringei|BIOUG15905-F01|Kate Perez|BIN Taxonomy Match|BOLD:AAF7635
Rheotanytarsus ringei|BIOUG15965-E11|Kate Perez|BIN Taxonomy Match|BOLD:AAF7635
Rheotanytarsus ringei|BIOUG16258-A04|Kate Perez|BIN Taxonomy Match|BOLD:AAF7635
Rheotanytarsus ringei|BIOUG16259-E07|Kate Perez|BIN Taxonomy Match|BOLD:AAF7635
Rheotanytarsus ringei|BIOUG16296-B07|Kate Perez|BIN Taxonomy Match|BOLD:AAF7635
Rheotanytarsus ringei|BIOUG16298-H05|Kate Perez|BIN Taxonomy Match|BOLD:AAF7635
Rheotanytarsus ringei|BIOUG16396-A11|Kate Perez|BIN Taxonomy Match|BOLD:AAF7635
Rheotanytarsus ringei|BIOUG16261-B10|Kate Perez|BIN Taxonomy Match|BOLD:AAF7635
Rheotanytarsus ringei|BIOUG16296-D09|Kate Perez|BIN Taxonomy Match|BOLD:AAF7635
Rheotanytarsus ringei|BIOUG16296-A12|Kate Perez|BIN Taxonomy Match|BOLD:AAF7635
Rheotanytarsus ringei|BIOUG16256-B09|Kate Perez|BIN Taxonomy Match|BOLD:AAF7635
Rheotanytarsus ringei|BIOUG16329-C07|Kate Perez|BIN Taxonomy Match|BOLD:AAF7635
Rheotanytarsus ringei|BIOUG16258-G12|Kate Perez|BIN Taxonomy Match|BOLD:AAF7635
Rheotanytarsus ringei|BIOUG15951-B01|Kate Perez|BIN Taxonomy Match|BOLD:AAF7635
Rheotanytarsus ringei|BIOUG16256-G08|Kate Perez|BIN Taxonomy Match|BOLD:AAF7635
Rheotanytarsus ringei|BIOUG16198-E08|Kate Perez|BIN Taxonomy Match|BOLD:AAF7635
Rheotanytarsus ringei|BIOUG16198-C02|Kate Perez|BIN Taxonomy Match|BOLD:AAF7635
Rheotanytarsus ringei|BIOUG16552-F03|Kate Perez|BIN Taxonomy Match|BOLD:AAF7635
Rheotanytarsus ringei|BIOUG15964-G12|Kate Perez|BIN Taxonomy Match|BOLD:AAF7635
Rheotanytarsus ringei|BIOUG16256-C02|Kate Perez|BIN Taxonomy Match|BOLD:AAF7635
Rheotanytarsus ringei|BIOUG16257-B03|Kate Perez|BIN Taxonomy Match|BOLD:AAF7635
Rheotanytarsus ringei|BIOUG16259-A07|Kate Perez|BIN Taxonomy Match|BOLD:AAF7635
Rheotanytarsus ringei|BIOUG16330-H02|Kate Perez|BIN Taxonomy Match|BOLD:AAF7635
Rheotanytarsus ringei|BIOUG16257-F03|Kate Perez|BIN Taxonomy Match|BOLD:AAF7635
Rheotanytarsus ringei|BIOUG16332-A01|Kate Perez|BIN Taxonomy Match|BOLD:AAF7635
Rheotanytarsus ringei|BIOUG16256-A11|Kate Perez|BIN Taxonomy Match|BOLD:AAF7635
Rheotanytarsus ringei|BIOUG16258-G11|Kate Perez|BIN Taxonomy Match|BOLD:AAF7635
Rheotanytarsus ringei|BIOUG15963-G02|Kate Perez|BIN Taxonomy Match|BOLD:AAF7635
Rheotanytarsus ringei|BIOUG16330-H07|Kate Perez|BIN Taxonomy Match|BOLD:AAF7635
Rheotanytarsus ringei|BIOUG16330-B04|Kate Perez|BIN Taxonomy Match|BOLD:AAF7635
Rheotanytarsus ringei|BIOUG16332-C09|Kate Perez|BIN Taxonomy Match|BOLD:AAF7635
Rheotanytarsus ringei|BIOUG16207-E11|Kate Perez|BIN Taxonomy Match|BOLD:AAF7635
Rheotanytarsus ringei|BIOUG16256-E11|Kate Perez|BIN Taxonomy Match|BOLD:AAF7635
Rheotanytarsus ringei|BIOUG16258-C09|Kate Perez|BIN Taxonomy Match|BOLD:AAF7635
Rheotanytarsus ringei|BIOUG16258-H02|Kate Perez|BIN Taxonomy Match|BOLD:AAF7635
Rheotanytarsus ringei|BIOUG16259-B12|Kate Perez|BIN Taxonomy Match|BOLD:AAF7635
Rheotanytarsus ringei|BIOUG16261-H05|Kate Perez|BIN Taxonomy Match|BOLD:AAF7635
Rheotanytarsus ringei|BIOUG16296-D04|Kate Perez|BIN Taxonomy Match|BOLD:AAF7635
Rheotanytarsus ringei|BIOUG16296-D06|Kate Perez|BIN Taxonomy Match|BOLD:AAF7635
Rheotanytarsus ringei|BIOUG16297-E10|Kate Perez|BIN Taxonomy Match|BOLD:AAF7635
Rheotanytarsus ringei|BIOUG16330-G03|Kate Perez|BIN Taxonomy Match|BOLD:AAF7635
Rheotanytarsus ringei|BIOUG16331-G03|Kate Perez|BIN Taxonomy Match|BOLD:AAF7635
Rheotanytarsus ringei|BIOUG15949-G08|Kate Perez|BIN Taxonomy Match|BOLD:AAF7635
[illegible]

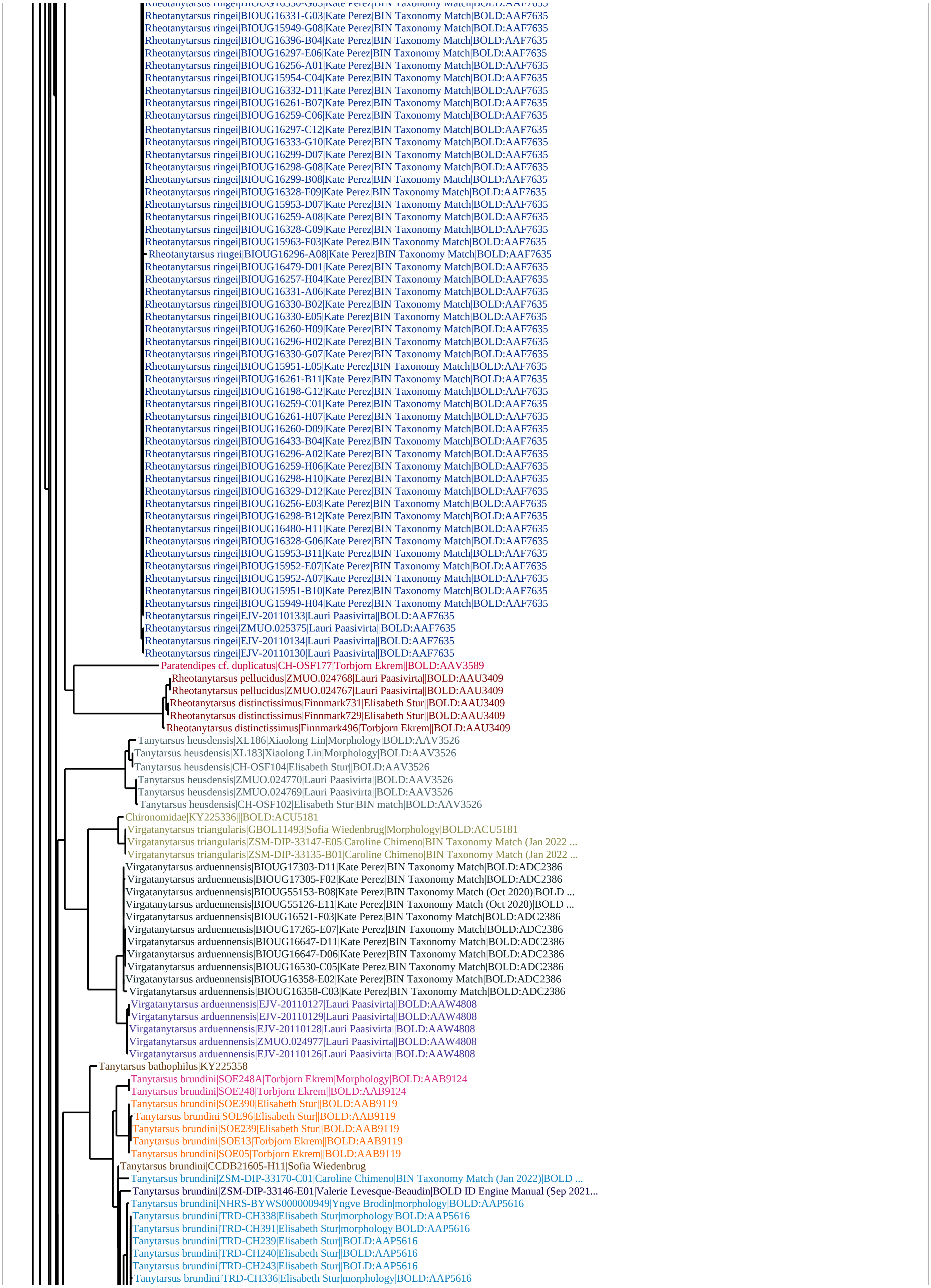
Rheotanytarsus ringei|BIOUG16330-G03|Kate Perez|BIN Taxonomy Match|BOLD:AAF7635
Rheotanytarsus ringei|BIOUG16331-G03|Kate Perez|BIN Taxonomy Match|BOLD:AAF7635
Rheotanytarsus ringei|BIOUG15949-G08|Kate Perez|BIN Taxonomy Match|BOLD:AAF7635
Rheotanytarsus ringei|BIOUG16396-B04|Kate Perez|BIN Taxonomy Match|BOLD:AAF7635
Rheotanytarsus ringei|BIOUG16297-E06|Kate Perez|BIN Taxonomy Match|BOLD:AAF7635
Rheotanytarsus ringei|BIOUG16256-A01|Kate Perez|BIN Taxonomy Match|BOLD:AAF7635
Rheotanytarsus ringei|BIOUG15954-C04|Kate Perez|BIN Taxonomy Match|BOLD:AAF7635
Rheotanytarsus ringei|BIOUG16332-D11|Kate Perez|BIN Taxonomy Match|BOLD:AAF7635
Rheotanytarsus ringei|BIOUG16261-B07|Kate Perez|BIN Taxonomy Match|BOLD:AAF7635
Rheotanytarsus ringei|BIOUG16259-C06|Kate Perez|BIN Taxonomy Match|BOLD:AAF7635
Rheotanytarsus ringei|BIOUG16297-C12|Kate Perez|BIN Taxonomy Match|BOLD:AAF7635
Rheotanytarsus ringei|BIOUG16333-G10|Kate Perez|BIN Taxonomy Match|BOLD:AAF7635
Rheotanytarsus ringei|BIOUG16299-D07|Kate Perez|BIN Taxonomy Match|BOLD:AAF7635
Rheotanytarsus ringei|BIOUG16298-G08|Kate Perez|BIN Taxonomy Match|BOLD:AAF7635
Rheotanytarsus ringei|BIOUG16299-B08|Kate Perez|BIN Taxonomy Match|BOLD:AAF7635
Rheotanytarsus ringei|BIOUG16328-F09|Kate Perez|BIN Taxonomy Match|BOLD:AAF7635
Rheotanytarsus ringei|BIOUG15953-D07|Kate Perez|BIN Taxonomy Match|BOLD:AAF7635
Rheotanytarsus ringei|BIOUG16259-A08|Kate Perez|BIN Taxonomy Match|BOLD:AAF7635
Rheotanytarsus ringei|BIOUG16328-G09|Kate Perez|BIN Taxonomy Match|BOLD:AAF7635
Rheotanytarsus ringei|BIOUG15963-F03|Kate Perez|BIN Taxonomy Match|BOLD:AAF7635
Rheotanytarsus ringei|BIOUG16296-A08|Kate Perez|BIN Taxonomy Match|BOLD:AAF7635
Rheotanytarsus ringei|BIOUG16479-D01|Kate Perez|BIN Taxonomy Match|BOLD:AAF7635
Rheotanytarsus ringei|BIOUG16257-H04|Kate Perez|BIN Taxonomy Match|BOLD:AAF7635
Rheotanytarsus ringei|BIOUG16331-A06|Kate Perez|BIN Taxonomy Match|BOLD:AAF7635
Rheotanytarsus ringei|BIOUG16330-B02|Kate Perez|BIN Taxonomy Match|BOLD:AAF7635
Rheotanytarsus ringei|BIOUG16330-E05|Kate Perez|BIN Taxonomy Match|BOLD:AAF7635
Rheotanytarsus ringei|BIOUG16260-H09|Kate Perez|BIN Taxonomy Match|BOLD:AAF7635
Rheotanytarsus ringei|BIOUG16296-H02|Kate Perez|BIN Taxonomy Match|BOLD:AAF7635
Rheotanytarsus ringei|BIOUG16330-G07|Kate Perez|BIN Taxonomy Match|BOLD:AAF7635
Rheotanytarsus ringei|BIOUG15951-E05|Kate Perez|BIN Taxonomy Match|BOLD:AAF7635
Rheotanytarsus ringei|BIOUG16261-B11|Kate Perez|BIN Taxonomy Match|BOLD:AAF7635
Rheotanytarsus ringei|BIOUG16198-G12|Kate Perez|BIN Taxonomy Match|BOLD:AAF7635
Rheotanytarsus ringei|BIOUG16259-C01|Kate Perez|BIN Taxonomy Match|BOLD:AAF7635
Rheotanytarsus ringei|BIOUG16261-H07|Kate Perez|BIN Taxonomy Match|BOLD:AAF7635
Rheotanytarsus ringei|BIOUG16260-D09|Kate Perez|BIN Taxonomy Match|BOLD:AAF7635
Rheotanytarsus ringei|BIOUG16433-B04|Kate Perez|BIN Taxonomy Match|BOLD:AAF7635
Rheotanytarsus ringei|BIOUG16296-A02|Kate Perez|BIN Taxonomy Match|BOLD:AAF7635
Rheotanytarsus ringei|BIOUG16259-H06|Kate Perez|BIN Taxonomy Match|BOLD:AAF7635
Rheotanytarsus ringei|BIOUG16298-H10|Kate Perez|BIN Taxonomy Match|BOLD:AAF7635
Rheotanytarsus ringei|BIOUG16329-D12|Kate Perez|BIN Taxonomy Match|BOLD:AAF7635
Rheotanytarsus ringei|BIOUG16256-E03|Kate Perez|BIN Taxonomy Match|BOLD:AAF7635
Rheotanytarsus ringei|BIOUG16298-B12|Kate Perez|BIN Taxonomy Match|BOLD:AAF7635
Rheotanytarsus ringei|BIOUG16480-H11|Kate Perez|BIN Taxonomy Match|BOLD:AAF7635
Rheotanytarsus ringei|BIOUG16328-G06|Kate Perez|BIN Taxonomy Match|BOLD:AAF7635
Rheotanytarsus ringei|BIOUG15953-B11|Kate Perez|BIN Taxonomy Match|BOLD:AAF7635
Rheotanytarsus ringei|BIOUG15952-E07|Kate Perez|BIN Taxonomy Match|BOLD:AAF7635
Rheotanytarsus ringei|BIOUG15952-A07|Kate Perez|BIN Taxonomy Match|BOLD:AAF7635
Rheotanytarsus ringei|BIOUG15951-B10|Kate Perez|BIN Taxonomy Match|BOLD:AAF7635
Rheotanytarsus ringei|BIOUG15949-H04|Kate Perez|BIN Taxonomy Match|BOLD:AAF7635
Rheotanytarsus ringei|EJV-20110133|Lauri Paasivirta||BOLD:AAF7635
Rheotanytarsus ringei|ZMUO.025375|Lauri Paasivirta||BOLD:AAF7635
Rheotanytarsus ringei|EJV-20110134|Lauri Paasivirta||BOLD:AAF7635
Rheotanytarsus ringei|EJV-20110130|Lauri Paasivirta||BOLD:AAF7635
Paratendipes cf. duplicatus|CH-OSF177|Torbjorn Ekrem||BOLD:AAV3589
Rheotanytarsus pellucidus|ZMUO.024768|Lauri Paasivirta||BOLD:AAU3409
Rheotanytarsus pellucidus|ZMUO.024767|Lauri Paasivirta||BOLD:AAU3409
Rheotanytarsus distinctissimus|Finnmark731|Elisabeth Stur||BOLD:AAU3409
Rheotanytarsus distinctissimus|Finnmark729|Elisabeth Stur||BOLD:AAU3409
Rheotanytarsus distinctissimus|Finnmark496|Torbjorn Ekrem||BOLD:AAU3409
Tanytarsus heusdensis|XL186|Xiaolong Lin|Morphology|BOLD:AAV3526
Tanytarsus heusdensis|XL183|Xiaolong Lin|Morphology|BOLD:AAV3526
Tanytarsus heusdensis|CH-OSF104|Elisabeth Stur||BOLD:AAV3526
Tanytarsus heusdensis|ZMUO.024770|Lauri Paasivirta||BOLD:AAV3526
Tanytarsus heusdensis|ZMUO.024769|Lauri Paasivirta||BOLD:AAV3526
Tanytarsus heusdensis|CH-OSF102|Elisabeth Stur|BIN match|BOLD:AAV3526
Chironomidae|KY225336|||BOLD:ACU5181
Virgatanytarsus triangularis|GBOL11493|Sofia Wiedenbrug|Morphology|BOLD:ACU5181
Virgatanytarsus triangularis|ZSM-DIP-33147-E05|Caroline Chimeno|BIN Taxonomy Match (Jan 2022 ...
Virgatanytarsus triangularis|ZSM-DIP-33135-B01|Caroline Chimeno|BIN Taxonomy Match (Jan 2022 ...
Virgatanytarsus arduennensis|BIOUG17303-D11|Kate Perez|BIN Taxonomy Match|BOLD:ADC2386
Virgatanytarsus arduennensis|BIOUG17305-F02|Kate Perez|BIN Taxonomy Match|BOLD:ADC2386
Virgatanytarsus arduennensis|BIOUG55153-B08|Kate Perez|BIN Taxonomy Match (Oct 2020)|BOLD ...
Virgatanytarsus arduennensis|BIOUG55126-E11|Kate Perez|BIN Taxonomy Match (Oct 2020)|BOLD ...
Virgatanytarsus arduennensis|BIOUG16521-F03|Kate Perez|BIN Taxonomy Match|BOLD:ADC2386
Virgatanytarsus arduennensis|BIOUG17265-E07|Kate Perez|BIN Taxonomy Match|BOLD:ADC2386
Virgatanytarsus arduennensis|BIOUG16647-D11|Kate Perez|BIN Taxonomy Match|BOLD:ADC2386
Virgatanytarsus arduennensis|BIOUG16647-D06|Kate Perez|BIN Taxonomy Match|BOLD:ADC2386
Virgatanytarsus arduennensis|BIOUG16530-C05|Kate Perez|BIN Taxonomy Match|BOLD:ADC2386
Virgatanytarsus arduennensis|BIOUG16358-E02|Kate Perez|BIN Taxonomy Match|BOLD:ADC2386
Virgatanytarsus arduennensis|BIOUG16358-C03|Kate Perez|BIN Taxonomy Match|BOLD:ADC2386
Virgatanytarsus arduennensis|EJV-20110127|Lauri Paasivirta||BOLD:AAW4808
Virgatanytarsus arduennensis|EJV-20110129|Lauri Paasivirta||BOLD:AAW4808
Virgatanytarsus arduennensis|EJV-20110128|Lauri Paasivirta||BOLD:AAW4808
Virgatanytarsus arduennensis|ZMUO.024977|Lauri Paasivirta||BOLD:AAW4808
Virgatanytarsus arduennensis|EJV-20110126|Lauri Paasivirta||BOLD:AAW4808
Tanytarsus bathophilus|KY225358
Tanytarsus brundini|SOE248A|Torbjorn Ekrem|Morphology|BOLD:AAB9124
Tanytarsus brundini|SOE248|Torbjorn Ekrem||BOLD:AAB9124
Tanytarsus brundini|SOE390|Elisabeth Stur||BOLD:AAB9119
Tanytarsus brundini|SOE96|Elisabeth Stur||BOLD:AAB9119
Tanytarsus brundini|SOE239|Elisabeth Stur||BOLD:AAB9119
Tanytarsus brundini|SOE13|Torbjorn Ekrem||BOLD:AAB9119
Tanytarsus brundini|SOE05|Torbjorn Ekrem||BOLD:AAB9119
Tanytarsus brundini|CCDB21605-H11|Sofia Wiedenbrug
Tanytarsus brundini|ZSM-DIP-33170-C01|Caroline Chimeno|BIN Taxonomy Match (Jan 2022)|BOLD ...
Tanytarsus brundini|ZSM-DIP-33146-E01|Valerie Levesque-Beaudin|BOLD ID Engine Manual (Sep 2021...
Tanytarsus brundini|NHRS-BYWS000000949|Yngve Brodin|morphology|BOLD:AAP5616
Tanytarsus brundini|TRD-CH338|Elisabeth Stur|morphology|BOLD:AAP5616
Tanytarsus brundini|TRD-CH391|Elisabeth Stur|morphology|BOLD:AAP5616
Tanytarsus brundini|TRD-CH239|Elisabeth Stur||BOLD:AAP5616
Tanytarsus brundini|TRD-CH240|Elisabeth Stur||BOLD:AAP5616
Tanytarsus brundini|TRD-CH243|Elisabeth Stur||BOLD:AAP5616
Tanytarsus brundini|TRD-CH336|Elisabeth Stur|morphology|BOLD:AAP5616

Tanytarsus brundini|TRD-CH240|Elisabeth Stur||BOLD:AAP5616
Tanytarsus brundini|TRD-CH243|Elisabeth Stur||BOLD:AAP5616
Tanytarsus brundini|TRD-CH336|Elisabeth Stur|morphology|BOLD:AAP5616
Tanytarsus brundini|TRD-CH225|Elisabeth Stur||BOLD:AAP5616
Tanytarsus brundini|TRD-CH238|Elisabeth Stur||BOLD:AAP5616
Tanytarsus brundini|TRD-CH335|Elisabeth Stur|morphology|BOLD:AAP5616
Tanytarsus brundini|NHRS-BYWS000000407|Yngve Brodin|morphology|BOLD:AAP5616
Tanytarsus brundini|NHRS-BYWS000001093|Yngve Brodin|morphology|BOLD:AAP5616
Tanytarsus brundini|TRD-CH189|Elisabeth Stur||BOLD:AAP5616
Tanytarsus brundini|BIOUG16477-B12|Kate Perez|BIN Taxonomy Match|BOLD:AAP5616
Tanytarsus brundini|BIOUG16476-H09|Kate Perez|BIN Taxonomy Match|BOLD:AAP5616
Tanytarsus brundini|TRD-CH137|Elisabeth Stur|morphology|BOLD:AAP5616
Tanytarsus brundini|Finnmark240|Torbjorn Ekrem||BOLD:ACQ8983
Tanytarsus brundini|BIOUG16434-E06|Elisabeth Stur|BOLD ID Engine Manual|BOLD:ACQ8985
Tanytarsus brundini|BIOUG16430-E08|Elisabeth Stur|BOLD ID Engine Manual|BOLD:ACQ8985
Tanytarsus brundini|BIOUG16480-D07|Elisabeth Stur|BOLD ID Engine Manual|BOLD:ACQ8985
Tanytarsus brundini|BIOUG16332-E11|Elisabeth Stur|BOLD ID Engine Manual|BOLD:ACQ8985
Tanytarsus brundini|ZSM-DIP-33135-G09|Caroline Chimeno|BIN Taxonomy Match (Jan 2022)|BOLD ...
Tanytarsus brundini|ZSM-DIP-33142-G09|Caroline Chimeno|BIN Taxonomy Match (Jan 2022)|BOLD ...
Tanytarsus brundini|ZSM-DIP-33134-G09|Caroline Chimeno|BIN Taxonomy Match (Jan 2022)|BOLD ...
Tanytarsus brundini|XL148|Xiaolong Lin|Morphology|BOLD:AAP5616
Tanytarsus brundini|NHRS-BYWS000001134|Yngve Brodin|morphology|BOLD:AAP5616
Tanytarsus brundini|NHRS-BYWS000000920|Yngve Brodin|morphology|BOLD:AAP5616
Tanytarsus brundini|BIOUG16435-H08|Kate Perez|BIN Taxonomy Match|BOLD:AAP5616
Tanytarsus brundini|BIOUG16432-G03|Kate Perez|BIN Taxonomy Match|BOLD:AAP5616
Tanytarsus brundini|TRD-CH399|Elisabeth Stur|morphology|BOLD:AAP5616
Tanytarsus brundini|BIOUG16479-A06|Kate Perez|BIN Taxonomy Match|BOLD:AAP5616
Tanytarsus brundini|BIOUG16426-F11|Kate Perez|BIN Taxonomy Match|BOLD:AAP5616
Tanytarsus brundini|CH-OSF103|Elisabeth Stur||BOLD:AAP5616
Tanytarsus brundini|ZSM-DIP-33142-A04|Caroline Chimeno|BIN Taxonomy Match (Jan 2022)|BOLD ...
Tanytarsus brundini|BIOUG16436-C01|Kate Perez|BIN Taxonomy Match|BOLD:AAP5616
Tanytarsus brundini|TRD-CH405|Elisabeth Stur|morphology|BOLD:AAP5616
Tanytarsus brundini|BIOUG16431-F01|Kate Perez|BIN Taxonomy Match|BOLD:AAP5616
Tanytarsus brundini|TRD-CH381|Elisabeth Stur|morphology|BOLD:ACQ8984
Tanytarsus brundini|BIOUG16432-F03|Elisabeth Stur|BOLD ID Engine Manual|BOLD:ACQ8984
Tanytarsus brundini|BIOUG16433-B01|Elisabeth Stur|BOLD ID Engine Manual|BOLD:ACQ8984
Tanytarsus brundini|BIOUG16435-D06|Elisabeth Stur|BOLD ID Engine Manual|BOLD:ACQ8984
Tanytarsus brundini|BIOUG16205-D03|Elisabeth Stur|BOLD ID Engine Manual|BOLD:ACQ8984
Tanytarsus brundini|BIOUG16476-E04|Kate Perez|BIN Taxonomy Match|BOLD:AAP5616
Tanytarsus brundini|ZSM-DIP-33135-G10|Caroline Chimeno|BIN Taxonomy Match (Jan 2022)|BOLD ...
Tanytarsus brundini|Finnmark239|Torbjorn Ekrem||BOLD:AAP5616
Tanytarsus brundini|NHRS-BYWS000000432|Yngve Brodin|morphology|BOLD:AAP5616
Tanytarsus brundini|To137|Torbjorn Ekrem|Morphology|BOLD:AAP5616
Tanytarsus brundini|BIOUG16436-C08|Elisabeth Stur|Tree based identification
Tanytarsus brundini|BIOUG16396-E01|Elisabeth Stur|BOLD ID Engine Manual|BOLD:ACR0797
Tanytarsus brundini|EJV-20110143|Lauri Paasivirta||BOLD:ACQ9072
Tanytarsus brundini|ZMUO.024782|Lauri Paasivirta||BOLD:AAP5616
Tanytarsus brundini|NHRS-BYWS000000158|Yngve Brodin|morphology|BOLD:AAP5616
Tanytarsus brundini|NHRS-BYWS000000159|Yngve Brodin|morphology|BOLD:AAP5616
Tanytarsus brundini|ZMUO.024783|Lauri Paasivirta||BOLD:AAP5616
Tanytarsus brundini|EJV-20110140|Lauri Paasivirta||BOLD:AAP5616
Tanytarsus brundini|EJV-20110142|Lauri Paasivirta||BOLD:AAP5616
Tanytarsus brundini|EJV-20110141|Lauri Paasivirta||BOLD:AAP5616
Chironomidae|BIOUG36753-B02|Meredith Miller|Tree-based Identification (Jan 2019)|BOLD:AD...
Stempellina bausei|Finnmark760|G. A. Halvorsen||BOLD:ACD1924
Stempellina bausei|SOE92|Torbjorn Ekrem
Stempellina bausei|SOE173|Elisabeth Stur||BOLD:AAD0308
Stempellina bausei|SOE25|Elisabeth Stur||BOLD:AAD0308
Stempellina bausei|SOE249|Elisabeth Stur||BOLD:AAD0309
Stempellina bausei|NO17|Torbjorn Ekrem||BOLD:AAD0309
Stempellina bausei|SOE391|Torbjorn Ekrem||BOLD:AAD0309
Stempellina bausei|To71|Torbjorn Ekrem||BOLD:AAD0309
Stempellina bausei|ZMUO.024914|Lauri Paasivirta||BOLD:AAD0309
Stempellina bausei|NHRS-BYWS000001128|Yngve Brodin|morphology|BOLD:AAD0309
Stempellina bausei|SOE389|Torbjorn Ekrem||BOLD:AAD0309
Stempellina bausei|ZMUO.024915|Lauri Paasivirta||BOLD:AAD0309
Stempellina bausei|NO16|Torbjorn Ekrem||BOLD:AAD0309
Stempellina bausei|TRD-CH334|Elisabeth Stur|morphology|BOLD:AAD0309
Stempellina bausei|NO 84|Elisabeth Stur||BOLD:AAD0309
Stempellinella flavidula|ZMUO.026235|Lauri Paasivirta||BOLD:ADF1889
Stempellinella brevis|BIOUG06274-H10|Kate Perez|BIN Taxonomy Match|BOLD:AAF3516
Stempellinella brevis|BIOUG05483-E11|Kate Perez|BIN Taxonomy Match|BOLD:AAF3516
Stempellinella brevis|BIOUG06056-H04|Kate Perez|BIN Taxonomy Match|BOLD:AAF3516
Stempellinella brevis|BIOUG05396-G05|Kate Perez|BIN Taxonomy Match|BOLD:AAF3516
Stempellinella brevis|BIOUG06055-G07|Kate Perez|BIN Taxonomy Match|BOLD:AAF3516
Stempellinella brevis|BIOUG05484-E05|Kate Perez|BIN Taxonomy Match|BOLD:AAF3516
Stempellinella brevis|BIOUG05777-B08|Kate Perez|BIN Taxonomy Match|BOLD:AAF3516
Stempellinella brevis|BIOUG06054-E02|Kate Perez|BIN Taxonomy Match|BOLD:AAF3516
Stempellinella brevis|To67|Torbjorn Ekrem||BOLD:AAF3516
Stempellinella brevis|To52|Torbjorn Ekrem||BOLD:AAF3516
Stempellinella brevis|CH-eik93|Elisabeth Stur||BOLD:AAF3516
Stempellinella brevis|ATNA571|Elisabeth Stur||BOLD:AAF3516
Stempellinella brevis|BIOUG05778-E08|Kate Perez|BIN Taxonomy Match|BOLD:AAF3516
Stempellinella brevis|ZMUO.024772|Lauri Paasivirta||BOLD:AAF3516
Stempellinella brevis|ZMUO.024771|Lauri Paasivirta||BOLD:AAF3516
Stempellinella brevis|Finnmark718|Elisabeth Stur||BOLD:AAF3516
Stempellinella brevis|Finnmark118|Elisabeth Stur||BOLD:AAF3516
Stempellinella brevis|To59|Torbjorn Ekrem||BOLD:AAF3516
Stempellinella brevis|Finnmark595|Elisabeth Stur||BOLD:AAF3516
Chironomidae|CCDB24229-H01|Jerome Moriniere|BIN Taxonomy Match|BOLD:ACU4730
Chironomidae|CCDB24229-B06|Jerome Moriniere|BIN Taxonomy Match|BOLD:ACU4730
Chironomidae|CCDB24229-B05|Jerome Moriniere|BIN Taxonomy Match|BOLD:ACU4730
Chironomidae|CCDB24229-B04|Jerome Moriniere|BIN Taxonomy Match|BOLD:ACU4730
Chironomidae|CCDB24229-B03|Jerome Moriniere|BIN Taxonomy Match|BOLD:ACU4730
Chironomidae|BIOUG16900-C04|Jerome Moriniere|BIN Taxonomy Match|BOLD:ACR1089
Chironomidae|BIOUG17262-C12|Jerome Moriniere|BIN Taxonomy Match|BOLD:ACR1089
Chironomidae|BIOUG16996-A02|Jerome Moriniere|BIN Taxonomy Match|BOLD:ACR1089
Cladotanytarsus cyrylae|PP0258|Lauri Paasivirta||BOLD:AAV9225
Cladotanytarsus cyrylae|ZSM-DIP-33133-G10|Caroline Chimeno|BIN Taxonomy Match (Jan 2022)|BO...
Cladotanytarsus cyrylae|To462|Torbjorn Ekrem||BOLD:AAV9225
Tanytarsus volgensis|ZMUO.025187|Lauri Paasivirta||BOLD:AAD2169
Tanytarsus volgensis|ZMUO.025186|Lauri Paasivirta||BOLD:AAD2169
Tanytarsus volgensis|BIOUG36987-D03|Kate Perez|BIN Taxonomy Match (Jul 2018)|BOLD:AAD2169

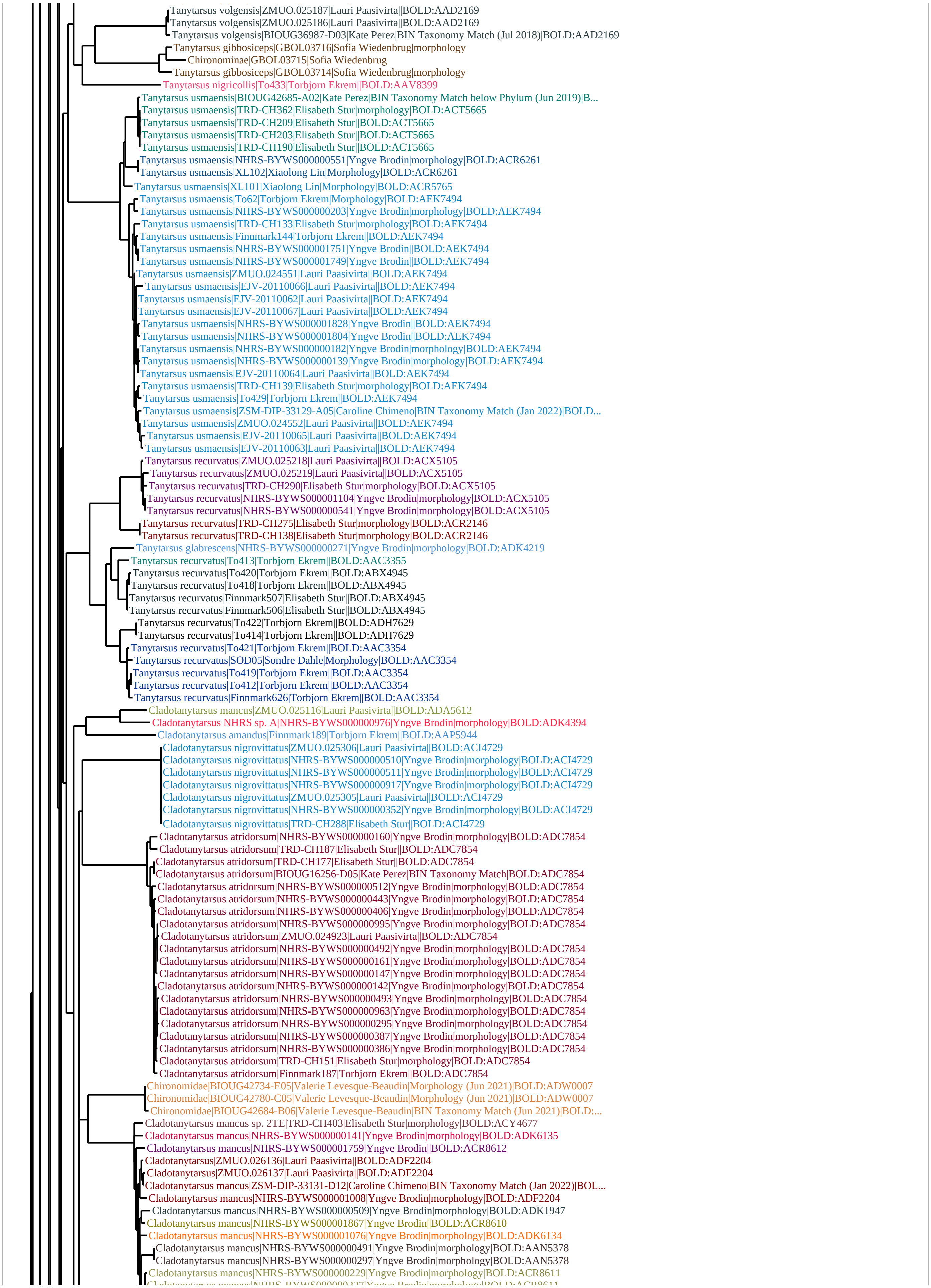
Tanytarsus volgensis|ZMUO.025187|Lauri Paasivirta||BOLD:AAD2169
Tanytarsus volgensis|ZMUO.025186|Lauri Paasivirta||BOLD:AAD2169
Tanytarsus volgensis|BIOUG36987-D03|Kate Perez|BIN Taxonomy Match (Jul 2018)|BOLD:AAD2169
Tanytarsus gibbosiceps|GBOL03716|Sofia Wiedenbrug|morphology
Chironominae|GBOL03715|Sofia Wiedenbrug
Tanytarsus gibbosiceps|GBOL03714|Sofia Wiedenbrug|morphology
Tanytarsus nigricollis|To433|Torbjorn Ekrem||BOLD:AAV8399
Tanytarsus usmaensis|BIOUG42685-A02|Kate Perez|BIN Taxonomy Match below Phylum (Jun 2019)|B...
Tanytarsus usmaensis|TRD-CH362|Elisabeth Stur|morphology|BOLD:ACT5665
Tanytarsus usmaensis|TRD-CH209|Elisabeth Stur||BOLD:ACT5665
Tanytarsus usmaensis|TRD-CH203|Elisabeth Stur||BOLD:ACT5665
Tanytarsus usmaensis|TRD-CH190|Elisabeth Stur||BOLD:ACT5665
Tanytarsus usmaensis|NHRS-BYWS000000551|Yngve Brodin|morphology|BOLD:ACR6261
Tanytarsus usmaensis|XL102|Xiaolong Lin|Morphology|BOLD:ACR6261
Tanytarsus usmaensis|XL101|Xiaolong Lin|Morphology|BOLD:ACR5765
Tanytarsus usmaensis|To62|Torbjorn Ekrem|Morphology|BOLD:AEK7494
Tanytarsus usmaensis|NHRS-BYWS000000203|Yngve Brodin|morphology|BOLD:AEK7494
Tanytarsus usmaensis|TRD-CH133|Elisabeth Stur|morphology|BOLD:AEK7494
Tanytarsus usmaensis|Finnmark144|Torbjorn Ekrem||BOLD:AEK7494
Tanytarsus usmaensis|NHRS-BYWS000001751|Yngve Brodin||BOLD:AEK7494
Tanytarsus usmaensis|NHRS-BYWS000001749|Yngve Brodin||BOLD:AEK7494
Tanytarsus usmaensis|ZMUO.024551|Lauri Paasivirta||BOLD:AEK7494
Tanytarsus usmaensis|EJV-20110066|Lauri Paasivirta||BOLD:AEK7494
Tanytarsus usmaensis|EJV-20110062|Lauri Paasivirta||BOLD:AEK7494
Tanytarsus usmaensis|EJV-20110067|Lauri Paasivirta||BOLD:AEK7494
Tanytarsus usmaensis|NHRS-BYWS000001828|Yngve Brodin||BOLD:AEK7494
Tanytarsus usmaensis|NHRS-BYWS000001804|Yngve Brodin||BOLD:AEK7494
Tanytarsus usmaensis|NHRS-BYWS000000182|Yngve Brodin|morphology|BOLD:AEK7494
Tanytarsus usmaensis|NHRS-BYWS000000139|Yngve Brodin|morphology|BOLD:AEK7494
Tanytarsus usmaensis|EJV-20110064|Lauri Paasivirta||BOLD:AEK7494
Tanytarsus usmaensis|TRD-CH139|Elisabeth Stur|morphology|BOLD:AEK7494
Tanytarsus usmaensis|To429|Torbjorn Ekrem||BOLD:AEK7494
Tanytarsus usmaensis|ZSM-DIP-33129-A05|Caroline Chimeno|BIN Taxonomy Match (Jan 2022)|BOLD...
Tanytarsus usmaensis|ZMUO.024552|Lauri Paasivirta||BOLD:AEK7494
Tanytarsus usmaensis|EJV-20110065|Lauri Paasivirta||BOLD:AEK7494
Tanytarsus usmaensis|EJV-20110063|Lauri Paasivirta||BOLD:AEK7494
Tanytarsus recurvatus|ZMUO.025218|Lauri Paasivirta||BOLD:ACX5105
Tanytarsus recurvatus|ZMUO.025219|Lauri Paasivirta||BOLD:ACX5105
Tanytarsus recurvatus|TRD-CH290|Elisabeth Stur|morphology|BOLD:ACX5105
Tanytarsus recurvatus|NHRS-BYWS000001104|Yngve Brodin|morphology|BOLD:ACX5105
Tanytarsus recurvatus|NHRS-BYWS000000541|Yngve Brodin|morphology|BOLD:ACX5105
Tanytarsus recurvatus|TRD-CH275|Elisabeth Stur|morphology|BOLD:ACR2146
Tanytarsus recurvatus|TRD-CH138|Elisabeth Stur|morphology|BOLD:ACR2146
Tanytarsus glabrescens|NHRS-BYWS000000271|Yngve Brodin|morphology|BOLD:ADK4219
Tanytarsus recurvatus|To413|Torbjorn Ekrem||BOLD:AAC3355
Tanytarsus recurvatus|To420|Torbjorn Ekrem||BOLD:ABX4945
Tanytarsus recurvatus|To418|Torbjorn Ekrem||BOLD:ABX4945
Tanytarsus recurvatus|Finnmark507|Elisabeth Stur||BOLD:ABX4945
Tanytarsus recurvatus|Finnmark506|Elisabeth Stur||BOLD:ABX4945
Tanytarsus recurvatus|To422|Torbjorn Ekrem||BOLD:ADH7629
Tanytarsus recurvatus|To414|Torbjorn Ekrem||BOLD:ADH7629
Tanytarsus recurvatus|To421|Torbjorn Ekrem||BOLD:AAC3354
Tanytarsus recurvatus|SOD05|Sondre Dahle|Morphology|BOLD:AAC3354
Tanytarsus recurvatus|To419|Torbjorn Ekrem||BOLD:AAC3354
Tanytarsus recurvatus|To412|Torbjorn Ekrem||BOLD:AAC3354
Tanytarsus recurvatus|Finnmark626|Torbjorn Ekrem||BOLD:AAC3354
Cladotanytarsus mancus|ZMUO.025116|Lauri Paasivirta||BOLD:ADA5612
Cladotanytarsus NHRS sp. A|NHRS-BYWS000000976|Yngve Brodin|morphology|BOLD:ADK4394
Cladotanytarsus amandus|Finnmark189|Torbjorn Ekrem||BOLD:AAP5944
Cladotanytarsus nigrovittatus|ZMUO.025306|Lauri Paasivirta||BOLD:ACI4729
Cladotanytarsus nigrovittatus|NHRS-BYWS000000510|Yngve Brodin|morphology|BOLD:ACI4729
Cladotanytarsus nigrovittatus|NHRS-BYWS000000511|Yngve Brodin|morphology|BOLD:ACI4729
Cladotanytarsus nigrovittatus|NHRS-BYWS000000917|Yngve Brodin|morphology|BOLD:ACI4729
Cladotanytarsus nigrovittatus|ZMUO.025305|Lauri Paasivirta||BOLD:ACI4729
Cladotanytarsus nigrovittatus|NHRS-BYWS000000352|Yngve Brodin|morphology|BOLD:ACI4729
Cladotanytarsus nigrovittatus|TRD-CH288|Elisabeth Stur||BOLD:ACI4729
Cladotanytarsus atridorsum|NHRS-BYWS000000160|Yngve Brodin|morphology|BOLD:ADC7854
Cladotanytarsus atridorsum|TRD-CH187|Elisabeth Stur||BOLD:ADC7854
Cladotanytarsus atridorsum|TRD-CH177|Elisabeth Stur||BOLD:ADC7854
Cladotanytarsus atridorsum|BIOUG16256-D05|Kate Perez|BIN Taxonomy Match|BOLD:ADC7854
Cladotanytarsus atridorsum|NHRS-BYWS000000512|Yngve Brodin|morphology|BOLD:ADC7854
Cladotanytarsus atridorsum|NHRS-BYWS000000443|Yngve Brodin|morphology|BOLD:ADC7854
Cladotanytarsus atridorsum|NHRS-BYWS000000406|Yngve Brodin|morphology|BOLD:ADC7854
Cladotanytarsus atridorsum|NHRS-BYWS000000995|Yngve Brodin|morphology|BOLD:ADC7854
Cladotanytarsus atridorsum|ZMUO.024923|Lauri Paasivirta||BOLD:ADC7854
Cladotanytarsus atridorsum|NHRS-BYWS000000492|Yngve Brodin|morphology|BOLD:ADC7854
Cladotanytarsus atridorsum|NHRS-BYWS000000161|Yngve Brodin|morphology|BOLD:ADC7854
Cladotanytarsus atridorsum|NHRS-BYWS000000147|Yngve Brodin|morphology|BOLD:ADC7854
Cladotanytarsus atridorsum|NHRS-BYWS000000142|Yngve Brodin|morphology|BOLD:ADC7854
Cladotanytarsus atridorsum|NHRS-BYWS000000493|Yngve Brodin|morphology|BOLD:ADC7854
Cladotanytarsus atridorsum|NHRS-BYWS000000963|Yngve Brodin|morphology|BOLD:ADC7854
Cladotanytarsus atridorsum|NHRS-BYWS000000295|Yngve Brodin|morphology|BOLD:ADC7854
Cladotanytarsus atridorsum|NHRS-BYWS000000387|Yngve Brodin|morphology|BOLD:ADC7854
Cladotanytarsus atridorsum|NHRS-BYWS000000386|Yngve Brodin|morphology|BOLD:ADC7854
Cladotanytarsus atridorsum|TRD-CH151|Elisabeth Stur|morphology|BOLD:ADC7854
Cladotanytarsus atridorsum|Finnmark187|Torbjorn Ekrem||BOLD:ADC7854
Chironomidae|BIOUG42734-E05|Valerie Levesque-Beaudin|Morphology (Jun 2021)|BOLD:ADW0007
Chironomidae|BIOUG42780-C05|Valerie Levesque-Beaudin|Morphology (Jun 2021)|BOLD:ADW0007
Chironomidae|BIOUG42684-B06|Valerie Levesque-Beaudin|BIN Taxonomy Match (Jun 2021)|BOLD:...
Cladotanytarsus mancus sp. 2TE|TRD-CH403|Elisabeth Stur|morphology|BOLD:ACY4677
Cladotanytarsus mancus|NHRS-BYWS000000141|Yngve Brodin|morphology|BOLD:ADK6135
Cladotanytarsus mancus|NHRS-BYWS000001759|Yngve Brodin||BOLD:ACR8612
Cladotanytarsus|ZMUO.026136|Lauri Paasivirta||BOLD:ADF2204
Cladotanytarsus|ZMUO.026137|Lauri Paasivirta||BOLD:ADF2204
Cladotanytarsus mancus|ZSM-DIP-33131-D12|Caroline Chimeno|BIN Taxonomy Match (Jan 2022)|BOL...
Cladotanytarsus mancus|NHRS-BYWS000001008|Yngve Brodin|morphology|BOLD:ADF2204
Cladotanytarsus mancus|NHRS-BYWS000000509|Yngve Brodin|morphology|BOLD:ADK1947
Cladotanytarsus mancus|NHRS-BYWS000001867|Yngve Brodin||BOLD:ACR8610
Cladotanytarsus mancus|NHRS-BYWS000001076|Yngve Brodin|morphology|BOLD:ADK6134
Cladotanytarsus mancus|NHRS-BYWS000000491|Yngve Brodin|morphology|BOLD:AAN5378
Cladotanytarsus mancus|NHRS-BYWS000000297|Yngve Brodin|morphology|BOLD:AAN5378
Cladotanytarsus mancus|NHRS-BYWS000000229|Yngve Brodin|morphology|BOLD:ACR8611
Cladotanytarsus mancus|NHRS-BYWS000000227|Yngve Brodin|morphology|BOLD:ACR8611

Cladotanytarsus mancus|NHRS-BYWS[illegible]|Yngve Brodin|morphology|BOLD:AAN5378
Cladotanytarsus mancus|NHRS-BYWS000000297|Yngve Brodin|morphology|BOLD:AAN5378
Cladotanytarsus mancus|NHRS-BYWS000000229|Yngve Brodin|morphology|BOLD:ACR8611
Cladotanytarsus mancus|NHRS-BYWS000000227|Yngve Brodin|morphology|BOLD:ACR8611
Cladotanytarsus mancus|NHRS-BYWS000000467|Yngve Brodin|morphology|BOLD:ACR8611
Cladotanytarsus mancus sp. 2TE|Finnmark736|Torbjorn Ekrem||BOLD:ACR8611
Cladotanytarsus mancus|ZMUO.025117|Lauri Paasivirta||BOLD:ACR8611
Cladotanytarsus mancus|NHRS-BYWS000001182|Yngve Brodin|morphology|BOLD:ACR8611
Cladotanytarsus mancus|NHRS-BYWS000001866|Yngve Brodin||BOLD:ACR8611
Cladotanytarsus mancus|ZSM-DIP-33134-E10|Caroline Chimeno|BIN Taxonomy Match (Jan 2022)|BOL...
Cladotanytarsus mancus|NHRS-BYWS000000230|Yngve Brodin|morphology|BOLD:ACR8609
Cladotanytarsus mancus|NHRS-BYWS000001801|Yngve Brodin||BOLD:ACR8609
Cladotanytarsus mancus|NHRS-BYWS000001758|Yngve Brodin||BOLD:ACR8609
Cladotanytarsus mancus|NHRS-BYWS000001757|Yngve Brodin||BOLD:ACR8609
Cladotanytarsus mancus|NHRS-BYWS000000202|Yngve Brodin|morphology|BOLD:ACR8609
Cladotanytarsus mancus|ZMUO.024925|Lauri Paasivirta||BOLD:ACR8609
Cladotanytarsus mancus-agg.|EJV-20110112|Lauri Paasivirta||BOLD:ACR8609
Cladotanytarsus gedanicus|ZMUO.025355|Lauri Paasivirta||BOLD:AAV9224
Cladotanytarsus gedanicus|NHRS-BYWS000000306|Yngve Brodin|morphology|BOLD:AAV9224
Cladotanytarsus gedanicus|NHRS-BYWS000000463|Yngve Brodin|morphology|BOLD:AAV9224
Cladotanytarsus gedanicus|NHRS-BYWS000000571|Yngve Brodin|morphology|BOLD:AAV9224
Cladotanytarsus gedanicus|NHRS-BYWS000000994|Yngve Brodin|morphology|BOLD:AAV9224
Cladotanytarsus gedanicus|NHRS-BYWS000000957|Yngve Brodin|morphology|BOLD:AAV9224
Cladotanytarsus gedanicus|NHRS-BYWS000000394|Yngve Brodin|morphology|BOLD:AAV9224
Cladotanytarsus gedanicus|NHRS-BYWS000000466|Yngve Brodin|morphology|BOLD:AAV9224
Cladotanytarsus gedanicus|NHRS-BYWS000000390|Yngve Brodin|morphology|BOLD:AAV9224
Cladotanytarsus gedanicus|NHRS-BYWS000000358|Yngve Brodin|morphology|BOLD:AAV9224
Cladotanytarsus gedanicus|NHRS-BYWS000000357|Yngve Brodin|morphology|BOLD:AAV9224
Cladotanytarsus gedanicus|NHRS-BYWS000000389|Yngve Brodin|morphology|BOLD:AAV9224
Cladotanytarsus gedanicus|To450|Torbjorn Ekrem||BOLD:AAV9224
Cladotanytarsus wexionensis|NHRS-BYWS000000533|Yngve Brodin|morphology|BOLD:ACZ8808
Cladotanytarsus bicornutus|ZMUO.025217|Lauri Paasivirta||BOLD:ACZ8808
Cladotanytarsus bicornutus|ZMUO.025216|Lauri Paasivirta||BOLD:ACZ8808
Cladotanytarsus wexionensis|NHRS-BYWS000000257|Yngve Brodin|morphology|BOLD:ACZ8808
Cladotanytarsus teres|ZMUO.024667|Lauri Paasivirta||BOLD:ADA7765
Cladotanytarsus molestus|PP0263|Lauri Paasivirta||BOLD:ADA7765
Cladotanytarsus pallidus|TRD-CH294|Elisabeth Stur||BOLD:AAR9373
Cladotanytarsus iucundus|NHRS-BYWS000001107|Yngve Brodin|morphology|BOLD:AAR9373
Cladotanytarsus iucundus|NHRS-BYWS000000307|Yngve Brodin|morphology|BOLD:AAR9373
Cladotanytarsus|BIOUG36737-C04|Kate Perez|BIN Taxonomy Match (May 2018)|BOLD:AAR9373
Cladotanytarsus|BIOUG55147-E01|Kate Perez|BIN Taxonomy Match (Oct 2020)|BOLD:AAR9373
Cladotanytarsus|BIOUG55118-B11|Kate Perez|BIN Taxonomy Match (Oct 2020)|BOLD:AAR9373
Cladotanytarsus iucundus|NHRS-BYWS000001021|Yngve Brodin|morphology|BOLD:AAR9373
Cladotanytarsus|ZSM-DIP-33129-A07|Caroline Chimeno|BIN Taxonomy Match (Jan 2022)|BOLD:AA...
Cladotanytarsus iucundus|NHRS-BYWS000001086|Yngve Brodin|morphology|BOLD:AAR9373
Cladotanytarsus iucundus|NHRS-BYWS000000181|Yngve Brodin|morphology|BOLD:AAR9373
Cladotanytarsus iucundus|NHRS-BYWS000000180|Yngve Brodin|morphology|BOLD:AAR9373
Cladotanytarsus iucundus|NHRS-BYWS000000140|Yngve Brodin|morphology|BOLD:AAR9373
Cladotanytarsus pallidus|To02|Torbjorn Ekrem||BOLD:AAR9373
Cladotanytarsus mancus sp. 3TE|Finnmark708|Torbjorn Ekrem||BOLD:AAJ1096
Cladotanytarsus mancus sp. 3TE|Finnmark706|Torbjorn Ekrem||BOLD:AAJ1096
Cladotanytarsus mancus sp. 3TE|Finnmark686|Torbjorn Ekrem||BOLD:AAJ1096
Cladotanytarsus mancus sp. 3TE|Finnmark623|Torbjorn Ekrem||BOLD:AAJ1096
Cladotanytarsus nr. lepidocalcar|ZMUO.025343|Lauri Paasivirta||BOLD:AAJ1096
Cladotanytarsus mancus|ZMUO.024926|Lauri Paasivirta||BOLD:AAJ1096
Cladotanytarsus mancus sp. 3TE|To81|Torbjorn Ekrem||BOLD:AAJ1096
Cladotanytarsus mancus sp. 3TE|Finnmark621|Torbjorn Ekrem|morphology|BOLD:AAJ1096
Cladotanytarsus difficilis|NHRS-BYWS000001106|Yngve Brodin|morphology|BOLD:AAQ2211
Cladotanytarsus difficilis|NHRS-BYWS000000366|Yngve Brodin|morphology|BOLD:AAQ2211
Cladotanytarsus difficilis|ZMUO.025130|Lauri Paasivirta||BOLD:AAQ2211
Cladotanytarsus difficilis|ZMUO.025131|Lauri Paasivirta||BOLD:AAQ2211
Cladotanytarsus difficilis|NHRS-BYWS000001852|Yngve Brodin||BOLD:AAQ2211
Cladotanytarsus difficilis|Finnmark594|Torbjorn Ekrem||BOLD:AAQ2211
Cladotanytarsus nr. vanderwulpi|ZMUO.025113|Lauri Paasivirta||BOLD:ADA7665
Cladotanytarsus nr. vanderwulpi|ZMUO.025112|Lauri Paasivirta||BOLD:ADA7665
Cladotanytarsus vanderwulpi|ZMUO.025223|Lauri Paasivirta||BOLD:ADA3644
Cladotanytarsus vanderwulpi|ZMUO.025222|Lauri Paasivirta||BOLD:ADA3644
Tanytarsus sp. 5TE|NO 80|Xiaolong Lin|morphology|BOLD:ACE7742
Cladotanytarsus|ZSM-DIP-33146-F05|Caroline Chimeno|BIN Taxonomy Match (Jan 2022)|BOLD:AC...
Cladotanytarsus vanderwulpi|Finnmark184|Torbjorn Ekrem||BOLD:AAP5943
Tanytarsus verralli|ZMUO.024758|Lauri Paasivirta||BOLD:ADA6495
Tanytarsus innarensis|ZMUO.025273|Lauri Paasivirta||BOLD:ACE7740
Tanytarsus innarensis|ZMUO.025274|Lauri Paasivirta||BOLD:ACE7740
Tanytarsus innarensis|NO 79|Elisabeth Stur||BOLD:ACE7740
Tanytarsus debilis|TRD-CH222|Elisabeth Stur||BOLD:AAV7044
Tanytarsus debilis|ZMUO.024935|Lauri Paasivirta||BOLD:AAV7044
Tanytarsus debilis|ZSM34343-D09|Sofia Wiedenbrug|Morphology|BOLD:AAV7044
Tanytarsus debilis|Finnmark450|Elisabeth Stur||BOLD:AAV7044
Tanytarsus debilis|Finnmark441|Elisabeth Stur||BOLD:AAV7043
Tanytarsus nemorosus|ZMUO.024269|Lauri Paasivirta||BOLD:ADA6297
Tanytarsus nemorosus|ZMUO.024861|Lauri Paasivirta||BOLD:ADA6297
Tanytarsus nemorosus|ZMUO.024860|Lauri Paasivirta||BOLD:ADA6297
Tanytarsus nemorosus|ZMUO.024270|Lauri Paasivirta||BOLD:ADA6297
Tanytarsus nemorosus|Finnmark864|Elisabeth Stur|morphology|BOLD:AAV3524
Tanytarsus nemorosus|Finnmark867|Elisabeth Stur|morphology|BOLD:AAV3524
Tanytarsus nemorosus|Finnmark863|Elisabeth Stur|morphology|BOLD:AAV3524
Tanytarsus nemorosus|Finnmark684|Elisabeth Stur||BOLD:AAV3524
Tanytarsus nemorosus|Finnmark427|Torbjorn Ekrem||BOLD:AAV3524
Chironomidae|BIOUG42667-G08|Valerie Levesque-Beaudin|Morphology (Jun 2021)|BOLD:ADW3052
Neozavrelia cuneipennis|To331|Torbjorn Ekrem||BOLD:AAG5550
Tanytarsus verralli|NHRS-BYWS000000413|Yngve Brodin|morphology|BOLD:ADK1446
Tanytarsus verralli|NHRS-BYWS000000552|Yngve Brodin|morphology|BOLD:ADK1445
Tanytarsus verralli|ZMUO.025232|Lauri Paasivirta||BOLD:ADA4951
Tanytarsus verralli|NHRS-BYWS000000381|Yngve Brodin|morphology|BOLD:ACB9674
Tanytarsus verralli|NHRS-BYWS000000382|Yngve Brodin|morphology|BOLD:ACB9674
Tanytarsus verralli|ZMUO.025233|Lauri Paasivirta||BOLD:ADA4952
Tanytarsus verralli|NHRS-BYWS000000355|Yngve Brodin|morphology|BOLD:AAQ0622
Tanytarsus verralli|To453|W. Gilka||BOLD:AAV8406
Tanytarsus verralli|To452|Torbjorn Ekrem||BOLD:AAV8406
Tanytarsus glabrescens|PP0274|Lauri Paasivirta||BOLD:ADZ0237
Micropsectra radialis|EBAI-Ch178|Elisabeth Stur|morphology
Micropsectra radialis|ATNA44|Elisabeth Stur||BOLD:AAA9787
Micropsectra radialis|To37|Torbjorn Ekrem||BOLD:AAA9787
[illegible]

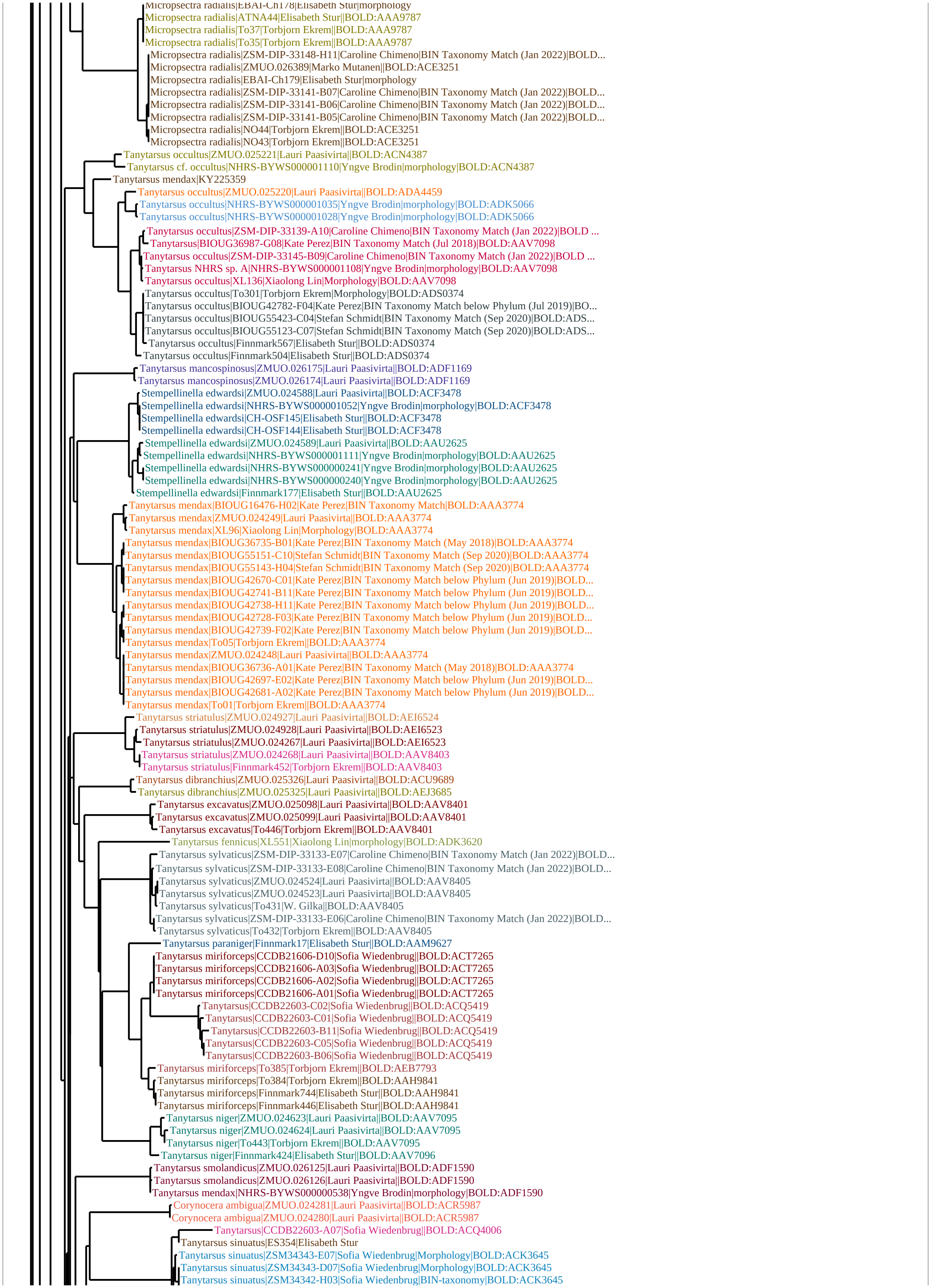
Micropsectra radialis|EBAI-Ch178|Elisabeth Stur|morphology
Micropsectra radialis|ATNA44|Elisabeth Stur||BOLD:AAA9787
Micropsectra radialis|To37|Torbjorn Ekrem||BOLD:AAA9787
Micropsectra radialis|To35|Torbjorn Ekrem||BOLD:AAA9787
Micropsectra radialis|ZSM-DIP-33148-H11|Caroline Chimeno|BIN Taxonomy Match (Jan 2022)|BOLD...
Micropsectra radialis|ZMUO.026389|Marko Mutanen||BOLD:ACE3251
Micropsectra radialis|EBAI-Ch179|Elisabeth Stur|morphology
Micropsectra radialis|ZSM-DIP-33141-B07|Caroline Chimeno|BIN Taxonomy Match (Jan 2022)|BOLD...
Micropsectra radialis|ZSM-DIP-33141-B06|Caroline Chimeno|BIN Taxonomy Match (Jan 2022)|BOLD...
Micropsectra radialis|ZSM-DIP-33141-B05|Caroline Chimeno|BIN Taxonomy Match (Jan 2022)|BOLD...
Micropsectra radialis|NO44|Torbjorn Ekrem||BOLD:ACE3251
Micropsectra radialis|NO43|Torbjorn Ekrem||BOLD:ACE3251
Tanytarsus occultus|ZMUO.025221|Lauri Paasivirta||BOLD:ACN4387
Tanytarsus cf. occultus|NHRS-BYWS000001110|Yngve Brodin|morphology|BOLD:ACN4387
Tanytarsus mendax|KY225359
Tanytarsus occultus|ZMUO.025220|Lauri Paasivirta||BOLD:ADA4459
Tanytarsus occultus|NHRS-BYWS000001035|Yngve Brodin|morphology|BOLD:ADK5066
Tanytarsus occultus|NHRS-BYWS000001028|Yngve Brodin|morphology|BOLD:ADK5066
Tanytarsus occultus|ZSM-DIP-33139-A10|Caroline Chimeno|BIN Taxonomy Match (Jan 2022)|BOLD ...
Tanytarsus|BIOUG36987-G08|Kate Perez|BIN Taxonomy Match (Jul 2018)|BOLD:AAV7098
Tanytarsus occultus|ZSM-DIP-33145-B09|Caroline Chimeno|BIN Taxonomy Match (Jan 2022)|BOLD ...
Tanytarsus NHRS sp. A|NHRS-BYWS000001108|Yngve Brodin|morphology|BOLD:AAV7098
Tanytarsus occultus|XL136|Xiaolong Lin|Morphology|BOLD:AAV7098
Tanytarsus occultus|To301|Torbjorn Ekrem|Morphology|BOLD:ADS0374
Tanytarsus occultus|BIOUG42782-F04|Kate Perez|BIN Taxonomy Match below Phylum (Jul 2019)|BO...
Tanytarsus occultus|BIOUG55423-C04|Stefan Schmidt|BIN Taxonomy Match (Sep 2020)|BOLD:ADS...
Tanytarsus occultus|BIOUG55123-C07|Stefan Schmidt|BIN Taxonomy Match (Sep 2020)|BOLD:ADS...
Tanytarsus occultus|Finnmark567|Elisabeth Stur||BOLD:ADS0374
Tanytarsus occultus|Finnmark504|Elisabeth Stur||BOLD:ADS0374
Tanytarsus mancospinosus|ZMUO.026175|Lauri Paasivirta||BOLD:ADF1169
Tanytarsus mancospinosus|ZMUO.026174|Lauri Paasivirta||BOLD:ADF1169
Stempellinella edwardsi|ZMUO.024588|Lauri Paasivirta||BOLD:ACF3478
Stempellinella edwardsi|NHRS-BYWS000001052|Yngve Brodin|morphology|BOLD:ACF3478
Stempellinella edwardsi|CH-OSF145|Elisabeth Stur||BOLD:ACF3478
Stempellinella edwardsi|CH-OSF144|Elisabeth Stur||BOLD:ACF3478
Stempellinella edwardsi|ZMUO.024589|Lauri Paasivirta||BOLD:AAU2625
Stempellinella edwardsi|NHRS-BYWS000001111|Yngve Brodin|morphology|BOLD:AAU2625
Stempellinella edwardsi|NHRS-BYWS000000241|Yngve Brodin|morphology|BOLD:AAU2625
Stempellinella edwardsi|NHRS-BYWS000000240|Yngve Brodin|morphology|BOLD:AAU2625
Stempellinella edwardsi|Finnmark177|Elisabeth Stur||BOLD:AAU2625
Tanytarsus mendax|BIOUG16476-H02|Kate Perez|BIN Taxonomy Match|BOLD:AAA3774
Tanytarsus mendax|ZMUO.024249|Lauri Paasivirta||BOLD:AAA3774
Tanytarsus mendax|XL96|Xiaolong Lin|Morphology|BOLD:AAA3774
Tanytarsus mendax|BIOUG36735-B01|Kate Perez|BIN Taxonomy Match (May 2018)|BOLD:AAA3774
Tanytarsus mendax|BIOUG55151-C10|Stefan Schmidt|BIN Taxonomy Match (Sep 2020)|BOLD:AAA3774
Tanytarsus mendax|BIOUG55143-H04|Stefan Schmidt|BIN Taxonomy Match (Sep 2020)|BOLD:AAA3774
Tanytarsus mendax|BIOUG42670-C01|Kate Perez|BIN Taxonomy Match below Phylum (Jun 2019)|BOLD...
Tanytarsus mendax|BIOUG42741-B11|Kate Perez|BIN Taxonomy Match below Phylum (Jun 2019)|BOLD...
Tanytarsus mendax|BIOUG42738-H11|Kate Perez|BIN Taxonomy Match below Phylum (Jun 2019)|BOLD...
Tanytarsus mendax|BIOUG42728-F03|Kate Perez|BIN Taxonomy Match below Phylum (Jun 2019)|BOLD...
Tanytarsus mendax|BIOUG42739-F02|Kate Perez|BIN Taxonomy Match below Phylum (Jun 2019)|BOLD...
Tanytarsus mendax|To05|Torbjorn Ekrem||BOLD:AAA3774
Tanytarsus mendax|ZMUO.024248|Lauri Paasivirta||BOLD:AAA3774
Tanytarsus mendax|BIOUG36736-A01|Kate Perez|BIN Taxonomy Match (May 2018)|BOLD:AAA3774
Tanytarsus mendax|BIOUG42697-E02|Kate Perez|BIN Taxonomy Match below Phylum (Jun 2019)|BOLD...
Tanytarsus mendax|BIOUG42681-A02|Kate Perez|BIN Taxonomy Match below Phylum (Jun 2019)|BOLD...
Tanytarsus mendax|To01|Torbjorn Ekrem||BOLD:AAA3774
Tanytarsus striatulus|ZMUO.024927|Lauri Paasivirta||BOLD:AEI6524
Tanytarsus striatulus|ZMUO.024928|Lauri Paasivirta||BOLD:AEI6523
Tanytarsus striatulus|ZMUO.024267|Lauri Paasivirta||BOLD:AEI6523
Tanytarsus striatulus|ZMUO.024268|Lauri Paasivirta||BOLD:AAV8403
Tanytarsus striatulus|Finnmark452|Torbjorn Ekrem||BOLD:AAV8403
Tanytarsus dibranchius|ZMUO.025326|Lauri Paasivirta||BOLD:ACU9689
Tanytarsus dibranchius|ZMUO.025325|Lauri Paasivirta||BOLD:AEJ3685
Tanytarsus excavatus|ZMUO.025098|Lauri Paasivirta||BOLD:AAV8401
Tanytarsus excavatus|ZMUO.025099|Lauri Paasivirta||BOLD:AAV8401
Tanytarsus excavatus|To446|Torbjorn Ekrem||BOLD:AAV8401
Tanytarsus fennicus|XL551|Xiaolong Lin|morphology|BOLD:ADK3620
Tanytarsus sylvaticus|ZSM-DIP-33133-E07|Caroline Chimeno|BIN Taxonomy Match (Jan 2022)|BOLD...
Tanytarsus sylvaticus|ZSM-DIP-33133-E08|Caroline Chimeno|BIN Taxonomy Match (Jan 2022)|BOLD...
Tanytarsus sylvaticus|ZMUO.024524|Lauri Paasivirta||BOLD:AAV8405
Tanytarsus sylvaticus|ZMUO.024523|Lauri Paasivirta||BOLD:AAV8405
Tanytarsus sylvaticus|To431|W. Gilka||BOLD:AAV8405
Tanytarsus sylvaticus|ZSM-DIP-33133-E06|Caroline Chimeno|BIN Taxonomy Match (Jan 2022)|BOLD...
Tanytarsus sylvaticus|To432|Torbjorn Ekrem||BOLD:AAV8405
Tanytarsus paraniger|Finnmark17|Elisabeth Stur||BOLD:AAM9627
Tanytarsus miriforceps|CCDB21606-D10|Sofia Wiedenbrug||BOLD:ACT7265
Tanytarsus miriforceps|CCDB21606-A03|Sofia Wiedenbrug||BOLD:ACT7265
Tanytarsus miriforceps|CCDB21606-A02|Sofia Wiedenbrug||BOLD:ACT7265
Tanytarsus miriforceps|CCDB21606-A01|Sofia Wiedenbrug||BOLD:ACT7265
Tanytarsus|CCDB22603-C02|Sofia Wiedenbrug||BOLD:ACQ5419
Tanytarsus|CCDB22603-C01|Sofia Wiedenbrug||BOLD:ACQ5419
Tanytarsus|CCDB22603-B11|Sofia Wiedenbrug||BOLD:ACQ5419
Tanytarsus|CCDB22603-C05|Sofia Wiedenbrug||BOLD:ACQ5419
Tanytarsus|CCDB22603-B06|Sofia Wiedenbrug||BOLD:ACQ5419
Tanytarsus miriforceps|To385|Torbjorn Ekrem||BOLD:AEB7793
Tanytarsus miriforceps|To384|Torbjorn Ekrem||BOLD:AAH9841
Tanytarsus miriforceps|Finnmark744|Elisabeth Stur||BOLD:AAH9841
Tanytarsus miriforceps|Finnmark446|Elisabeth Stur||BOLD:AAH9841
Tanytarsus niger|ZMUO.024623|Lauri Paasivirta||BOLD:AAV7095
Tanytarsus niger|ZMUO.024624|Lauri Paasivirta||BOLD:AAV7095
Tanytarsus niger|To443|Torbjorn Ekrem||BOLD:AAV7095
Tanytarsus niger|Finnmark424|Elisabeth Stur||BOLD:AAV7096
Tanytarsus smolandicus|ZMUO.026125|Lauri Paasivirta||BOLD:ADF1590
Tanytarsus smolandicus|ZMUO.026126|Lauri Paasivirta||BOLD:ADF1590
Tanytarsus mendax|NHRS-BYWS000000538|Yngve Brodin|morphology|BOLD:ADF1590
Corynocera ambigua|ZMUO.024281|Lauri Paasivirta||BOLD:ACR5987
Corynocera ambigua|ZMUO.024280|Lauri Paasivirta||BOLD:ACR5987
Tanytarsus|CCDB22603-A07|Sofia Wiedenbrug||BOLD:ACQ4006
Tanytarsus sinuatus|ES354|Elisabeth Stur
Tanytarsus sinuatus|ZSM34343-E07|Sofia Wiedenbrug|Morphology|BOLD:ACK3645
Tanytarsus sinuatus|ZSM34343-D07|Sofia Wiedenbrug|Morphology|BOLD:ACK3645
Tanytarsus sinuatus|ZSM34342-H03|Sofia Wiedenbrug|BIN-taxonomy|BOLD:ACK3645

Tanytarsus sinuatus|ZSM34343-E07|Sofia Wiedenbrug|Morphology|BOLD:ACK3645
Tanytarsus sinuatus|ZSM34343-D07|Sofia Wiedenbrug|Morphology|BOLD:ACK3645
Tanytarsus sinuatus|ZSM34342-H03|Sofia Wiedenbrug|BIN-taxonomy|BOLD:ACK3645
Tanytarsus sinuatus|CCDB24229-E08|Sofia Wiedenbrug|BIN Taxonomy Match|BOLD:ACK3645
Tanytarsus sinuatus|CCDB24229-E02|Sofia Wiedenbrug|BIN Taxonomy Match|BOLD:ACK3645
Tanytarsus sinuatus|ZSM34343-D03|Sofia Wiedenbrug|Morphology|BOLD:ACK3645
Tanytarsus sinuatus|CCDB24229-E09|Sofia Wiedenbrug|BIN Taxonomy Match|BOLD:ACK3645
Tanytarsus sinuatus|CCDB24228-A10|Sofia Wiedenbrug|BIN Taxonomy Match|BOLD:ACK3645
Tanytarsus sinuatus|CCDB24228-A09|Sofia Wiedenbrug|BIN Taxonomy Match|BOLD:ACK3645
Tanytarsus sinuatus|ES351|Xiaolong Lin|Morphology & BIN match|BOLD:ACK3645
Tanytarsus sinuatus|ES377|Xiaolong Lin|Morphology & BIN match|BOLD:ACK3645
Tanytarsus sinuatus|ES386|Xiaolong Lin|Morphology & BIN match|BOLD:ACK3645
Tanytarsus sinuatus|ES350|Xiaolong Lin|Morphology & BIN match|BOLD:ACK3645
Tanytarsus sinuatus|ES352|Xiaolong Lin|Morphology & BIN match|BOLD:ACK3645
Tanytarsus sinuatus|ES355|Xiaolong Lin|Morphology & BIN match|BOLD:ACK3645
Tanytarsus sinuatus|ES387|Xiaolong Lin|Morphology & BIN match|BOLD:ACK3645
Tanytarsus gracilentus|SOD03|Sondre Dahle|Morphology|BOLD:AAU0267
Tanytarsus gracilentus|To58|Torbjorn Ekrem||BOLD:AAU0267
Tanytarsus gracilentus|To57|Torbjorn Ekrem||BOLD:AAU0267
Tanytarsus gracilentus|NHRS-BYWS000001029|Yngve Brodin|morphology|BOLD:AAU0267
Tanytarsus gracilentus|NHRS-BYWS000000131|Yngve Brodin|morphology|BOLD:AAU0267
Tanytarsus gracilentus|NHRS-BYWS000000143|Yngve Brodin|morphology|BOLD:AAU0267
Tanytarsus gracilentus|NHRS-BYWS000000483|Yngve Brodin|morphology|BOLD:AAU0267
Tanytarsus gracilentus|NHRS-BYWS000000402|Yngve Brodin|morphology|BOLD:AAU0267
Tanytarsus gracilentus|NHRS-BYWS000000130|Yngve Brodin|morphology|BOLD:AAU0267
Tanytarsus gracilentus|NHRS-BYWS000000129|Yngve Brodin|morphology|BOLD:AAU0267
Tanytarsus gracilentus|ZMUO.026189|Lauri Paasivirta||BOLD:AAU0267
Tanytarsus gracilentus|ZMUO.026190|Lauri Paasivirta||BOLD:AAU0267
Tanytarsus gracilentus|NHRS-BYWS000000465|Yngve Brodin|morphology|BOLD:AAU0267
Tanytarsus gracilentus|NHRS-BYWS000001826|Yngve Brodin||BOLD:AAU0267
Tanytarsus gracilentus|Finnmark409|Elisabeth Stur||BOLD:AAU0266
Tanytarsus gracilentus|Finnmark632|Torbjorn Ekrem||BOLD:AAU0266
Tanytarsus medius|BIOUG36655-A05|Kate Perez|BIN Taxonomy Match (Jul 2018)|BOLD:ACG9929
Tanytarsus medius|XL137|Xiaolong Lin|Morphology|BOLD:ACG9929
Tanytarsus medius|ZMUO.025087|Lauri Paasivirta||BOLD:ACG9929
Tanytarsus medius|ZMUO.025086|Lauri Paasivirta||BOLD:ACG9929
Tanytarsus medius|CH-eik124|Elisabeth Stur||BOLD:ACG9929
Tanytarsus ejuncidus|ZSM-DIP-33146-D12|Caroline Chimeno|BIN Taxonomy Match (Jan 2022)|BOLD...
Tanytarsus ejuncidus|ZSM-DIP-33146-D11|Caroline Chimeno|BIN Taxonomy Match (Jan 2022)|BOLD...
Tanytarsus ejuncidus|ZSM-DIP-33145-H09|Caroline Chimeno|BIN Taxonomy Match (Jan 2022)|BOLD...
Tanytarsus ejuncidus|ZSM-DIP-33144-H04|Caroline Chimeno|BIN Taxonomy Match (Jan 2022)|BOLD...
Tanytarsus ejuncidus|XL138|Xiaolong Lin|Morphology|BOLD:AAW1102
Tanytarsus ejuncidus|ZSM-DIP-33144-B10|Caroline Chimeno|BIN Taxonomy Match (Jan 2022)|BOLD...
Tanytarsus ejuncidus|ZSM-DIP-33137-G11|Caroline Chimeno|BIN Taxonomy Match (Jan 2022)|BOLD...
Tanytarsus ejuncidus|CH-OSF38|Torbjorn Ekrem||BOLD:AAW1102
Tanytarsus ejuncidus|ZSM-DIP-33129-A04|Caroline Chimeno|BIN Taxonomy Match (Jan 2022)|BOLD...
Tanytarsus ejuncidus|BIOUG16358-F01|Kate Perez|BIN Taxonomy Match|BOLD:AAW1102
Tanytarsus lestagei|ZMUO.025164|Lauri Paasivirta||BOLD:ACD1309
Tanytarsus dispar|TRD-CH242|Torbjorn Ekrem|morphology|BOLD:ACD1309
Tanytarsus dispar|TRD-CH223|Torbjorn Ekrem|morphology|BOLD:ACD1309
Tanytarsus dispar|TRD-CH244|Torbjorn Ekrem|morphology|BOLD:ACD1309
Tanytarsus lestagei|XL177|Xiaolong Lin|Morphology|BOLD:ACR5689
Tanytarsus lestagei|XL143|Xiaolong Lin|Morphology|BOLD:AAP5876
Tanytarsus lestagei|TRD-CH383|Elisabeth Stur|morphology|BOLD:AAP5876
Tanytarsus lestagei|TRD-CH152|Elisabeth Stur|morphology|BOLD:AAP5876
Tanytarsus lestagei|TRD-CH382|Elisabeth Stur|morphology|BOLD:AAP5876
Tanytarsus telmaticus|ZMUO.026199|Lauri Paasivirta||BOLD:AAP5876
Tanytarsus lestagei|XL144|Xiaolong Lin|Morphology|BOLD:AAP5876
Tanytarsus lestagei|Finnmark182|Torbjorn Ekrem||BOLD:AAP5876
Tanytarsus telmaticus|ZMUO.024946|Lauri Paasivirta||BOLD:ACI5769
Tanytarsus telmaticus|ZMUO.024945|Lauri Paasivirta||BOLD:ACI5769
Tanytarsus lestagei|ZMUO.025165|Lauri Paasivirta||BOLD:ADI2276
Tanytarsus lestagei|ZMUO.024894|Lauri Paasivirta||BOLD:ADI2278
Tanytarsus palmeni|TRD-CH279|Torbjorn Ekrem|morphology|BOLD:ACX5198
Tanytarsus telmaticus|NO36|Torbjorn Ekrem||BOLD:AAF0834
Tanytarsus longitarsis|XL88|Xiaolong Lin|Morphology|BOLD:ACR5860
Tanytarsus longitarsis|XL98|Xiaolong Lin|Morphology|BOLD:AAU4445
Tanytarsus telmaticus|NHRS-BYWS000000996|Yngve Brodin|morphology|BOLD:AAU4445
Tanytarsus telmaticus|NHRS-BYWS000000449|Yngve Brodin|morphology|BOLD:AAU4445
Tanytarsus telmaticus|NHRS-BYWS000000446|Yngve Brodin|morphology|BOLD:AAU4445
Tanytarsus telmaticus|NHRS-BYWS000000448|Yngve Brodin|morphology|BOLD:AAU4445
Tanytarsus telmaticus|NHRS-BYWS000000977|Yngve Brodin|morphology|BOLD:AAU4445
Tanytarsus longitarsis|XL97|Xiaolong Lin|Morphology|BOLD:AAU4445
Tanytarsus cf. longitarsis|To75|Torbjorn Ekrem||BOLD:AAU4445
Tanytarsus telmaticus|NO38|Torbjorn Ekrem||BOLD:AAF0835
Tanytarsus telmaticus|NHRS-BYWS000001019|Yngve Brodin|morphology|BOLD:AAF0835
Tanytarsus telmaticus|Finnmark593|Elisabeth Stur||BOLD:AAF0835
Tanytarsus thomasi|BIOUG16432-H03|Xiaolong Lin|BOLD ID Engine Manual|BOLD:ACQ9930
Tanytarsus thomasi|Finnmark666|Xiaolong Lin|Morphology & BIN match|BOLD:AAB9122
Tanytarsus brundini|BIOUG04296-D07|Kate Perez|BIN Taxonomy Match|BOLD:AAB9122
Tanytarsus brundini|BIOUG04114-F02|Kate Perez|BIN Taxonomy Match|BOLD:AAB9122
Tanytarsus brundini|BIOUG04114-E02|Kate Perez|BIN Taxonomy Match|BOLD:AAB9122
Tanytarsus curticornis|ZMUO.024979|Lauri Paasivirta||BOLD:AAH9823
Tanytarsus curticornis|BIOUG04433-G02|Kate Perez|BIN Taxonomy Match|BOLD:AAH9823
Tanytarsus curticornis|XL99|Xiaolong Lin|Morphology|BOLD:AAH9823
Tanytarsus curticornis|To82|Torbjorn Ekrem||BOLD:AAH9823
Tanytarsus quadridentatus|ZMUO.029807|Marko Mutanen||BOLD:ADI4263
Tanytarsus|ZSM-DIP-33142-C11|Caroline Chimeno|BIN Taxonomy Match (Jan 2022)|BOLD:ACF7553
Tanytarsus heusdensis|BIOUG36655-C07|Kate Perez|BIN Taxonomy Match (Jun 2019)|BOLD:ACF7553
Tanytarsus heusdensis|BC-ZSM-DIP-22551-F12|Dieter Doczkal||BOLD:ACF7553
Tanytarsus reei|XL180|Xiaolong Lin|Morphology|BOLD:ACF7553
Tanytarsus heusdensis|ZFMK-TIS-2534215|Simone Chapuis||BOLD:ACF7553
Tanytarsus heusdensis|BIOUG05396-D10|Kate Perez|BIN Taxonomy Match|BOLD:ACF7553
Tanytarsus heusdensis|BIOUG16528-H09|Kate Perez|BIN Taxonomy Match|BOLD:ACF7553
Tanytarsus|ZSM-DIP-33149-E09|Caroline Chimeno|BIN Taxonomy Match (Jan 2022)|BOLD:ACF7553
Tanytarsus|ZSM-DIP-33147-D12|Caroline Chimeno|BIN Taxonomy Match (Jan 2022)|BOLD:ACF7553
Tanytarsus|ZSM-DIP-33142-A06|Caroline Chimeno|BIN Taxonomy Match (Jan 2022)|BOLD:ACF7553
Tanytarsus heusdensis|BIOUG16616-G05|Kate Perez|BIN Taxonomy Match|BOLD:ACF7553
Tanytarsus sp. BOLD:ACF7553|BIOUG16358-C05|Kate Perez|BIN Taxonomy Match|BOLD:ACF7553
Paratanytarsus tenellulus|ZMUO.025081|Lauri Paasivirta||BOLD:ACO9916
Paratanytarsus tenellulus|ZMUO.024985|Lauri Paasivirta||BOLD:ACO9916
Paratanytarsus tenellulus|NHRS-BYWS000000332|Yngve Brodin|morphology|BOLD:ACO9916
Paratanytarsus natvigi|NHRS-BYWS000000263|Yngve Brodin|morphology|BOLD:ADK4927

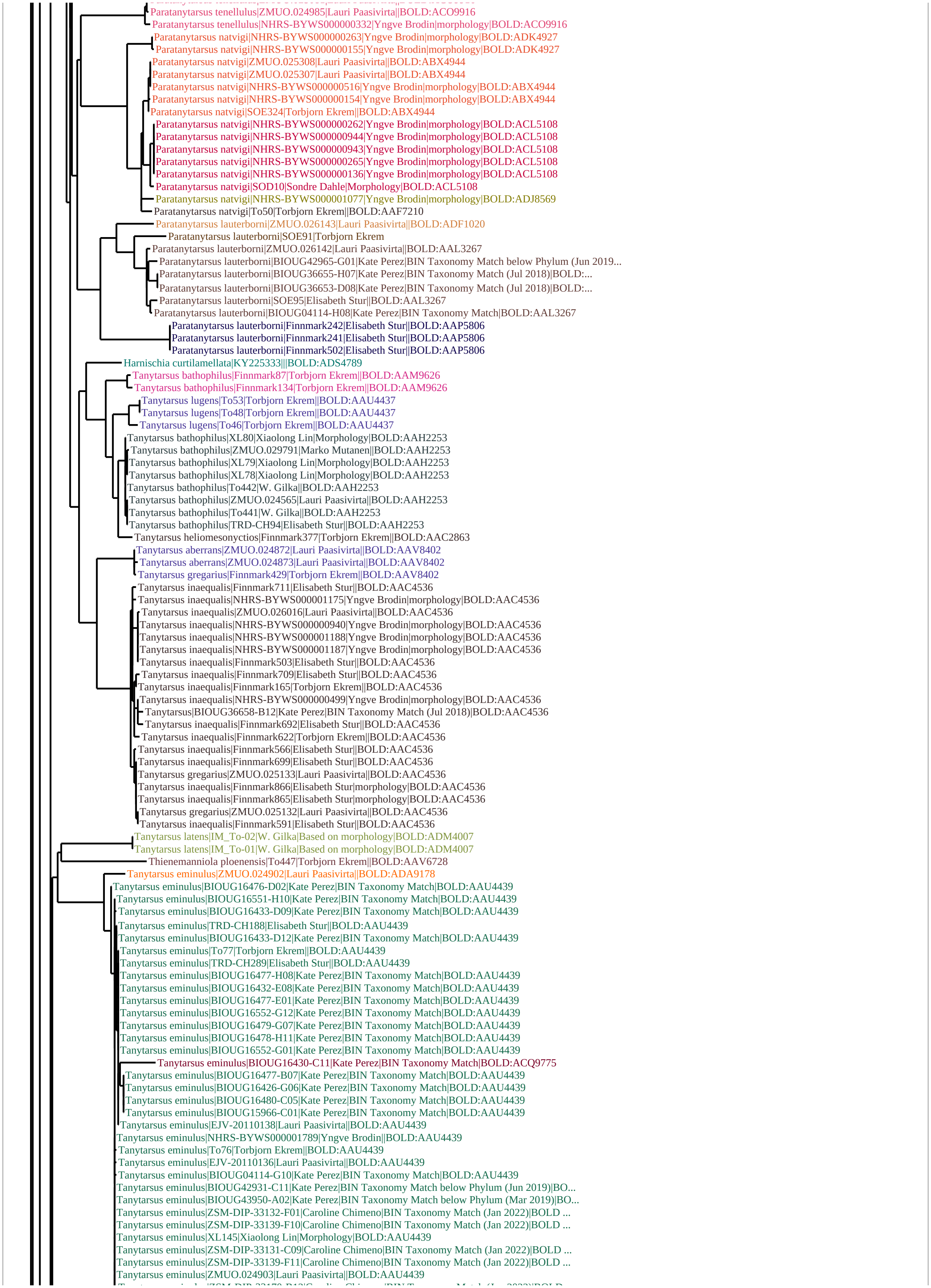
Paratanytarsus tenellulus|ZMUO.024985|Lauri Paasivirta||BOLD:ACO9916
Paratanytarsus tenellulus|NHRS-BYWS000000332|Yngve Brodin|morphology|BOLD:ACO9916
Paratanytarsus natvigi|NHRS-BYWS000000263|Yngve Brodin|morphology|BOLD:ADK4927
Paratanytarsus natvigi|NHRS-BYWS000000155|Yngve Brodin|morphology|BOLD:ADK4927
Paratanytarsus natvigi|ZMUO.025308|Lauri Paasivirta||BOLD:ABX4944
Paratanytarsus natvigi|ZMUO.025307|Lauri Paasivirta||BOLD:ABX4944
Paratanytarsus natvigi|NHRS-BYWS000000516|Yngve Brodin|morphology|BOLD:ABX4944
Paratanytarsus natvigi|NHRS-BYWS000000154|Yngve Brodin|morphology|BOLD:ABX4944
Paratanytarsus natvigi|SOE324|Torbjorn Ekrem||BOLD:ABX4944
Paratanytarsus natvigi|NHRS-BYWS000000262|Yngve Brodin|morphology|BOLD:ACL5108
Paratanytarsus natvigi|NHRS-BYWS000000944|Yngve Brodin|morphology|BOLD:ACL5108
Paratanytarsus natvigi|NHRS-BYWS000000943|Yngve Brodin|morphology|BOLD:ACL5108
Paratanytarsus natvigi|NHRS-BYWS000000265|Yngve Brodin|morphology|BOLD:ACL5108
Paratanytarsus natvigi|NHRS-BYWS000000136|Yngve Brodin|morphology|BOLD:ACL5108
Paratanytarsus natvigi|SOD10|Sondre Dahle|Morphology|BOLD:ACL5108
Paratanytarsus natvigi|NHRS-BYWS000001077|Yngve Brodin|morphology|BOLD:ADJ8569
Paratanytarsus natvigi|To50|Torbjorn Ekrem||BOLD:AAF7210
Paratanytarsus lauterborni|ZMUO.026143|Lauri Paasivirta||BOLD:ADF1020
Paratanytarsus lauterborni|SOE91|Torbjorn Ekrem
Paratanytarsus lauterborni|ZMUO.026142|Lauri Paasivirta||BOLD:AAL3267
Paratanytarsus lauterborni|BIOUG42965-G01|Kate Perez|BIN Taxonomy Match below Phylum (Jun 2019...
Paratanytarsus lauterborni|BIOUG36655-H07|Kate Perez|BIN Taxonomy Match (Jul 2018)|BOLD:...
Paratanytarsus lauterborni|BIOUG36653-D08|Kate Perez|BIN Taxonomy Match (Jul 2018)|BOLD:...
Paratanytarsus lauterborni|SOE95|Elisabeth Stur||BOLD:AAL3267
Paratanytarsus lauterborni|BIOUG04114-H08|Kate Perez|BIN Taxonomy Match|BOLD:AAL3267
Paratanytarsus lauterborni|Finnmark242|Elisabeth Stur||BOLD:AAP5806
Paratanytarsus lauterborni|Finnmark241|Elisabeth Stur||BOLD:AAP5806
Paratanytarsus lauterborni|Finnmark502|Elisabeth Stur||BOLD:AAP5806
Harnischia curtilamellata|KY225333|||BOLD:ADS4789
Tanytarsus bathophilus|Finnmark87|Torbjorn Ekrem||BOLD:AAM9626
Tanytarsus bathophilus|Finnmark134|Torbjorn Ekrem||BOLD:AAM9626
Tanytarsus lugens|To53|Torbjorn Ekrem||BOLD:AAU4437
Tanytarsus lugens|To48|Torbjorn Ekrem||BOLD:AAU4437
Tanytarsus lugens|To46|Torbjorn Ekrem||BOLD:AAU4437
Tanytarsus bathophilus|XL80|Xiaolong Lin|Morphology|BOLD:AAH2253
Tanytarsus bathophilus|ZMUO.029791|Marko Mutanen||BOLD:AAH2253
Tanytarsus bathophilus|XL79|Xiaolong Lin|Morphology|BOLD:AAH2253
Tanytarsus bathophilus|XL78|Xiaolong Lin|Morphology|BOLD:AAH2253
Tanytarsus bathophilus|To442|W. Gilka||BOLD:AAH2253
Tanytarsus bathophilus|ZMUO.024565|Lauri Paasivirta||BOLD:AAH2253
Tanytarsus bathophilus|To441|W. Gilka||BOLD:AAH2253
Tanytarsus bathophilus|TRD-CH94|Elisabeth Stur||BOLD:AAH2253
Tanytarsus heliomesonyctios|Finnmark377|Torbjorn Ekrem||BOLD:AAC2863
Tanytarsus aberrans|ZMUO.024872|Lauri Paasivirta||BOLD:AAV8402
Tanytarsus aberrans|ZMUO.024873|Lauri Paasivirta||BOLD:AAV8402
Tanytarsus gregarius|Finnmark429|Torbjorn Ekrem||BOLD:AAV8402
Tanytarsus inaequalis|Finnmark711|Elisabeth Stur||BOLD:AAC4536
Tanytarsus inaequalis|NHRS-BYWS000001175|Yngve Brodin|morphology|BOLD:AAC4536
Tanytarsus inaequalis|ZMUO.026016|Lauri Paasivirta||BOLD:AAC4536
Tanytarsus inaequalis|NHRS-BYWS000000940|Yngve Brodin|morphology|BOLD:AAC4536
Tanytarsus inaequalis|NHRS-BYWS000001188|Yngve Brodin|morphology|BOLD:AAC4536
Tanytarsus inaequalis|NHRS-BYWS000001187|Yngve Brodin|morphology|BOLD:AAC4536
Tanytarsus inaequalis|Finnmark503|Elisabeth Stur||BOLD:AAC4536
Tanytarsus inaequalis|Finnmark709|Elisabeth Stur||BOLD:AAC4536
Tanytarsus inaequalis|Finnmark165|Torbjorn Ekrem||BOLD:AAC4536
Tanytarsus inaequalis|NHRS-BYWS000000499|Yngve Brodin|morphology|BOLD:AAC4536
Tanytarsus|BIOUG36658-B12|Kate Perez|BIN Taxonomy Match (Jul 2018)|BOLD:AAC4536
Tanytarsus inaequalis|Finnmark692|Elisabeth Stur||BOLD:AAC4536
Tanytarsus inaequalis|Finnmark622|Torbjorn Ekrem||BOLD:AAC4536
Tanytarsus inaequalis|Finnmark566|Elisabeth Stur||BOLD:AAC4536
Tanytarsus inaequalis|Finnmark699|Elisabeth Stur||BOLD:AAC4536
Tanytarsus gregarius|ZMUO.025133|Lauri Paasivirta||BOLD:AAC4536
Tanytarsus inaequalis|Finnmark866|Elisabeth Stur|morphology|BOLD:AAC4536
Tanytarsus inaequalis|Finnmark865|Elisabeth Stur|morphology|BOLD:AAC4536
Tanytarsus gregarius|ZMUO.025132|Lauri Paasivirta||BOLD:AAC4536
Tanytarsus inaequalis|Finnmark591|Elisabeth Stur||BOLD:AAC4536
Tanytarsus latens|IM_To-02|W. Gilka|Based on morphology|BOLD:ADM4007
Tanytarsus latens|IM_To-01|W. Gilka|Based on morphology|BOLD:ADM4007
Thienemanniola ploenensis|To447|Torbjorn Ekrem||BOLD:AAV6728
Tanytarsus eminulus|ZMUO.024902|Lauri Paasivirta||BOLD:ADA9178
Tanytarsus eminulus|BIOUG16476-D02|Kate Perez|BIN Taxonomy Match|BOLD:AAU4439
Tanytarsus eminulus|BIOUG16551-H10|Kate Perez|BIN Taxonomy Match|BOLD:AAU4439
Tanytarsus eminulus|BIOUG16433-D09|Kate Perez|BIN Taxonomy Match|BOLD:AAU4439
Tanytarsus eminulus|TRD-CH188|Elisabeth Stur||BOLD:AAU4439
Tanytarsus eminulus|BIOUG16433-D12|Kate Perez|BIN Taxonomy Match|BOLD:AAU4439
Tanytarsus eminulus|To77|Torbjorn Ekrem||BOLD:AAU4439
Tanytarsus eminulus|TRD-CH289|Elisabeth Stur||BOLD:AAU4439
Tanytarsus eminulus|BIOUG16477-H08|Kate Perez|BIN Taxonomy Match|BOLD:AAU4439
Tanytarsus eminulus|BIOUG16432-E08|Kate Perez|BIN Taxonomy Match|BOLD:AAU4439
Tanytarsus eminulus|BIOUG16477-E01|Kate Perez|BIN Taxonomy Match|BOLD:AAU4439
Tanytarsus eminulus|BIOUG16552-G12|Kate Perez|BIN Taxonomy Match|BOLD:AAU4439
Tanytarsus eminulus|BIOUG16479-G07|Kate Perez|BIN Taxonomy Match|BOLD:AAU4439
Tanytarsus eminulus|BIOUG16478-H11|Kate Perez|BIN Taxonomy Match|BOLD:AAU4439
Tanytarsus eminulus|BIOUG16552-G01|Kate Perez|BIN Taxonomy Match|BOLD:AAU4439
Tanytarsus eminulus|BIOUG16430-C11|Kate Perez|BIN Taxonomy Match|BOLD:ACQ9775
Tanytarsus eminulus|BIOUG16477-B07|Kate Perez|BIN Taxonomy Match|BOLD:AAU4439
Tanytarsus eminulus|BIOUG16426-G06|Kate Perez|BIN Taxonomy Match|BOLD:AAU4439
Tanytarsus eminulus|BIOUG16480-C05|Kate Perez|BIN Taxonomy Match|BOLD:AAU4439
Tanytarsus eminulus|BIOUG15966-C01|Kate Perez|BIN Taxonomy Match|BOLD:AAU4439
Tanytarsus eminulus|EJV-20110138|Lauri Paasivirta||BOLD:AAU4439
Tanytarsus eminulus|NHRS-BYWS000001789|Yngve Brodin||BOLD:AAU4439
Tanytarsus eminulus|To76|Torbjorn Ekrem||BOLD:AAU4439
Tanytarsus eminulus|EJV-20110136|Lauri Paasivirta||BOLD:AAU4439
Tanytarsus eminulus|BIOUG04114-G10|Kate Perez|BIN Taxonomy Match|BOLD:AAU4439
Tanytarsus eminulus|BIOUG42931-C11|Kate Perez|BIN Taxonomy Match below Phylum (Jun 2019)|BO...
Tanytarsus eminulus|BIOUG43950-A02|Kate Perez|BIN Taxonomy Match below Phylum (Mar 2019)|BO...
Tanytarsus eminulus|ZSM-DIP-33132-F01|Caroline Chimeno|BIN Taxonomy Match (Jan 2022)|BOLD ...
Tanytarsus eminulus|ZSM-DIP-33139-F10|Caroline Chimeno|BIN Taxonomy Match (Jan 2022)|BOLD ...
Tanytarsus eminulus|XL145|Xiaolong Lin|Morphology|BOLD:AAU4439
Tanytarsus eminulus|ZSM-DIP-33131-C09|Caroline Chimeno|BIN Taxonomy Match (Jan 2022)|BOLD ...
Tanytarsus eminulus|ZSM-DIP-33139-F11|Caroline Chimeno|BIN Taxonomy Match (Jan 2022)|BOLD ...
Tanytarsus eminulus|ZMUO.024903|Lauri Paasivirta||BOLD:AAU4439

Tanytarsus eminulus|ZSM-DIP-33131-C09|Caroline Chimeno|BIN Taxonomy Match (Jan 2022)|BOLD ...
Tanytarsus eminulus|ZSM-DIP-33139-F11|Caroline Chimeno|BIN Taxonomy Match (Jan 2022)|BOLD ...
Tanytarsus eminulus|ZMUO.024903|Lauri Paasivirta||BOLD:AAU4439
Tanytarsus eminulus|ZSM-DIP-33170-B12|Caroline Chimeno|BIN Taxonomy Match (Jan 2022)|BOLD ...
Tanytarsus eminulus|ZSM-DIP-33142-A05|Caroline Chimeno|BIN Taxonomy Match (Jan 2022)|BOLD ...
Tanytarsus eminulus|BIOUG16476-D06|Kate Perez|BIN Taxonomy Match|BOLD:AAU4439
Tanytarsus eminulus|BIOUG16480-E04|Kate Perez|BIN Taxonomy Match|BOLD:AAU4439
Tanytarsus eminulus|BIOUG16435-G10|Kate Perez|BIN Taxonomy Match|BOLD:AAU4439
Tanytarsus eminulus|BIOUG16430-E04|Kate Perez|BIN Taxonomy Match|BOLD:AAU4439
Tanytarsus eminulus|BIOUG16432-A07|Kate Perez|BIN Taxonomy Match|BOLD:AAU4439
Tanytarsus eminulus|BIOUG16431-C04|Kate Perez|BIN Taxonomy Match|BOLD:AAU4439
Tanytarsus eminulus|BIOUG16432-G09|Kate Perez|BIN Taxonomy Match|BOLD:AAU4439
Tanytarsus eminulus|BIOUG16476-E03|Kate Perez|BIN Taxonomy Match|BOLD:AAU4439
Tanytarsus eminulus|BIOUG16432-D02|Kate Perez|BIN Taxonomy Match|BOLD:AAU4439
Tanytarsus eminulus|BIOUG16434-E03|Kate Perez|BIN Taxonomy Match|BOLD:AAU4439
Tanytarsus eminulus|BIOUG16434-D06|Kate Perez|BIN Taxonomy Match|BOLD:AAU4439
Tanytarsus eminulus|BIOUG16557-G10|Kate Perez|BIN Taxonomy Match|BOLD:AAU4439
Tanytarsus eminulus|BIOUG16431-C10|Kate Perez|BIN Taxonomy Match|BOLD:AAU4439
Tanytarsus eminulus|BIOUG16476-F02|Kate Perez|BIN Taxonomy Match|BOLD:AAU4439
Tanytarsus eminulus|BIOUG16478-E02|Kate Perez|BIN Taxonomy Match|BOLD:AAU4439
Tanytarsus eminulus|BIOUG16436-C05|Kate Perez|BIN Taxonomy Match|BOLD:AAU4439
Tanytarsus eminulus|BIOUG16430-E11|Kate Perez|BIN Taxonomy Match|BOLD:AAU4439
Tanytarsus eminulus|BIOUG16433-C08|Kate Perez|BIN Taxonomy Match|BOLD:AAU4439
Tanytarsus eminulus|BIOUG16433-D11|Kate Perez|BIN Taxonomy Match|BOLD:AAU4439
Tanytarsus eminulus|BIOUG16480-G10|Kate Perez|BIN Taxonomy Match|BOLD:AAU4439
Tanytarsus eminulus|BIOUG16436-H10|Kate Perez|BIN Taxonomy Match|BOLD:AAU4439
Tanytarsus eminulus|BIOUG16436-C11|Kate Perez|BIN Taxonomy Match|BOLD:AAU4439
Tanytarsus eminulus|BIOUG16436-B10|Kate Perez|BIN Taxonomy Match|BOLD:AAU4439
Tanytarsus eminulus|BIOUG16436-A04|Kate Perez|BIN Taxonomy Match|BOLD:AAU4439
Tanytarsus eminulus|BIOUG16432-C12|Kate Perez|BIN Taxonomy Match|BOLD:AAU4439
Tanytarsus eminulus|BIOUG16479-E07|Kate Perez|BIN Taxonomy Match|BOLD:AAU4439
Tanytarsus eminulus|BIOUG16597-B01|Kate Perez|BIN Taxonomy Match|BOLD:AAU4439
Tanytarsus eminulus|BIOUG16552-A04|Kate Perez|BIN Taxonomy Match|BOLD:AAU4439
Tanytarsus eminulus|BIOUG16551-H09|Kate Perez|BIN Taxonomy Match|BOLD:AAU4439
Tanytarsus eminulus|BIOUG16555-H07|Kate Perez|BIN Taxonomy Match|BOLD:AAU4439
Tanytarsus eminulus|BIOUG16479-G12|Kate Perez|BIN Taxonomy Match|BOLD:AAU4439
Tanytarsus eminulus|BIOUG16431-E08|Kate Perez|BIN Taxonomy Match|BOLD:AAU4439
Tanytarsus eminulus|BIOUG16432-F02|Kate Perez|BIN Taxonomy Match|BOLD:AAU4439
Tanytarsus eminulus|BIOUG16436-G03|Kate Perez|BIN Taxonomy Match|BOLD:AAU4439
Tanytarsus eminulus|BIOUG16480-D08|Kate Perez|BIN Taxonomy Match|BOLD:AAU4439
Tanytarsus eminulus|BIOUG16436-H11|Kate Perez|BIN Taxonomy Match|BOLD:AAU4439
Tanytarsus eminulus|BIOUG16476-D08|Kate Perez|BIN Taxonomy Match|BOLD:AAU4439
Tanytarsus eminulus|BIOUG16479-A12|Kate Perez|BIN Taxonomy Match|BOLD:AAU4439
Tanytarsus eminulus|BIOUG16476-F05|Kate Perez|BIN Taxonomy Match|BOLD:AAU4439
Tanytarsus eminulus|BIOUG16430-A09|Kate Perez|BIN Taxonomy Match|BOLD:AAU4439
Tanytarsus eminulus|BIOUG16431-B08|Kate Perez|BIN Taxonomy Match|BOLD:AAU4439
Tanytarsus eminulus|BIOUG16431-C03|Kate Perez|BIN Taxonomy Match|BOLD:AAU4439
Tanytarsus eminulus|BIOUG16432-H01|Kate Perez|BIN Taxonomy Match|BOLD:AAU4439
Tanytarsus eminulus|BIOUG36776-H05|Kate Perez|BIN Taxonomy Match (Jul 2018)|BOLD:AAU4439
Tanytarsus eminulus|BIOUG16432-B12|Kate Perez|BIN Taxonomy Match|BOLD:AAU4439
Tanytarsus eminulus|BIOUG16434-G12|Kate Perez|BIN Taxonomy Match|BOLD:AAU4439
Tanytarsus eminulus|BIOUG16479-F03|Kate Perez|BIN Taxonomy Match|BOLD:AAU4439
Tanytarsus eminulus|BIOUG16432-G10|Kate Perez|BIN Taxonomy Match|BOLD:AAU4439
Tanytarsus eminulus|BIOUG16476-C08|Kate Perez|BIN Taxonomy Match|BOLD:AAU4439
Tanytarsus eminulus|BIOUG16476-E08|Kate Perez|BIN Taxonomy Match|BOLD:AAU4439
Tanytarsus eminulus|BIOUG16476-B01|Kate Perez|BIN Taxonomy Match|BOLD:AAU4439
Tanytarsus eminulus|BIOUG16478-A11|Kate Perez|BIN Taxonomy Match|BOLD:AAU4439
Tanytarsus eminulus|BIOUG16432-G02|Kate Perez|BIN Taxonomy Match|BOLD:AAU4439
Tanytarsus eminulus|BIOUG16435-D11|Kate Perez|BIN Taxonomy Match|BOLD:AAU4439
Tanytarsus eminulus|BIOUG16478-F01|Kate Perez|BIN Taxonomy Match|BOLD:AAU4439
Tanytarsus eminulus|BIOUG16430-A12|Kate Perez|BIN Taxonomy Match
Tanytarsus eminulus|BIOUG16434-D11|Kate Perez|BIN Taxonomy Match|BOLD:AAU4439
Tanytarsus eminulus|BIOUG16552-A10|Kate Perez|BIN Taxonomy Match|BOLD:AAU4439
Tanytarsus eminulus|BIOUG16551-D10|Kate Perez|BIN Taxonomy Match|BOLD:AAU4439
Tanytarsus eminulus|BIOUG16552-F07|Kate Perez|BIN Taxonomy Match|BOLD:AAU4439
Tanytarsus eminulus|BIOUG16552-D02|Kate Perez|BIN Taxonomy Match|BOLD:AAU4439
Tanytarsus eminulus|BIOUG16552-B06|Kate Perez|BIN Taxonomy Match|BOLD:AAU4439
Tanytarsus eminulus|BIOUG16552-H04|Kate Perez|BIN Taxonomy Match|BOLD:AAU4439
Tanytarsus eminulus|EJV-20110135|Lauri Paasivirta||BOLD:AAU4439
Neostempellina thienemanni|CCDB21606-F01|Sofia Wiedenbrug||BOLD:AAX6525
Neostempellina thienemanni|CCDB21606-E09|Sofia Wiedenbrug||BOLD:AAX6525
Neostempellina thienemanni|CCDB21606-E10|Sofia Wiedenbrug||BOLD:AAX6525
Neostempellina thienemanni|CCDB21606-E06|Sofia Wiedenbrug||BOLD:AAX6525
Neostempellina thienemanni|CCDB21606-E08|Sofia Wiedenbrug||BOLD:AAX6525
Neostempellina thienemanni|To332|Torbjorn Ekrem||BOLD:AAX6525
Constempellina brevicosta|ZMUO.026222|Lauri Paasivirta||BOLD:AAP5885
Constempellina brevicosta|NHRS-BYWS000001097|Yngve Brodin|morphology|BOLD:AAP5885
Constempellina brevicosta|NHRS-BYWS000001098|Yngve Brodin|morphology|BOLD:AAP5885
Constempellina brevicosta|ZMUO.026221|Lauri Paasivirta||BOLD:AAP5885
Constempellina|Finnmark180|Torbjorn Ekrem|morphology|BOLD:AAP5885
Stempellina sp. 1ES|ATNA552|Elisabeth Stur||BOLD:AAH2999
Stempellina sp. 1ES|ATNA522|Elisabeth Stur||BOLD:AAH2999
Stempellina sp. 1ES|ATNA523|Elisabeth Stur||BOLD:AAH2999
Stempellina sp. 1ES|ATNA521|Elisabeth Stur||BOLD:AAH2999
Stempellina sp. 1ES|ATNA519|Elisabeth Stur||BOLD:AAH2999
Stempellina sp. 1ES|EBAI-Ch167|Elisabeth Stur|morphology|BOLD:AAH2999
Stempellina sp. 1ES|EBAI-Ch166|Elisabeth Stur|morphology|BOLD:AAH2999
Stempellina sp. 1ES|EBAI-Ch252|Elisabeth Stur|morphology|BOLD:AAH2999
Stempellina sp. 1ES|EBAI-Ch168|Elisabeth Stur|morphology|BOLD:AAH2999
Stempellina sp. 1ES|ATNA520|Elisabeth Stur||BOLD:AAH2999
Stempellina sp. 1ES|ATNA186|Elisabeth Stur||BOLD:AAH2999
Constempellina brevicosta|Finnmark431|Elisabeth Stur||BOLD:ACS1632
Constempellina brevicosta|ZMUO.024300|Lauri Paasivirta||BOLD:ACS1634
Constempellina brevicosta|ZMUO.024299|Lauri Paasivirta||BOLD:ACS1634
Constempellina brevicosta|Finnmark124|Elisabeth Stur||BOLD:ACS1634
Constempellina brevicosta|Finnmark372|Elisabeth Stur||BOLD:ACS1634
Constempellina brevicosta|To136|Torbjorn Ekrem||BOLD:ACS1633
Constempellina brevicosta|Finnmark117|Elisabeth Stur||BOLD:ACS1633
Constempellina brevicosta|Finnmark84|Elisabeth Stur||BOLD:ACS1633
Constempellina brevicosta|Finnmark80|Torbjorn Ekrem||BOLD:ACS1633
Constempellina brevicosta|CH-OSF142|Elisabeth Stur||BOLD:ACS1633
Constempellina brevicosta|CH-OSF143|Elisabeth Stur||BOLD:ACS1633
Constempellina brevicosta|Finnmark793|Elisabeth Stur||BOLD:ACS1633

Constempellina brevicosta|CH-OSF142|Elisabeth Stur||BOLD:ACS1633
Constempellina brevicosta|CH-OSF143|Elisabeth Stur||BOLD:ACS1633
Constempellina brevicosta|Finnmark793|Elisabeth Stur||BOLD:ACS1633
Constempellina brevicosta|Finnmark81|Elisabeth Stur||BOLD:ACS1633
Constempellina brevicosta|Finnmark123|Elisabeth Stur||BOLD:ACS1633
Constempellina brevicosta|Finnmark371|Elisabeth Stur||BOLD:ACS1633
Paratanytarsus brevicalcar|ZMUO.025229|Lauri Paasivirta||BOLD:AAX1215
Paratanytarsus brevicalcar|ZMUO.025228|Lauri Paasivirta||BOLD:AAX1215
Paratanytarsus|ZSM-DIP-33129-C03|Caroline Chimeno|BIN Taxonomy Match (Jan 2022)|BOLD:AAX...
Paratanytarsus brevicalcar|ZMUO.026343|Lauri Paasivirta||BOLD:ADF1942
Micropsectra klinki|ZSM-DIP-33139-G02|Caroline Chimeno|BIN Taxonomy Match (Jan 2022)|BOLD ...
Micropsectra klinki|ZSM-DIP-33139-G03|Caroline Chimeno|BIN Taxonomy Match (Jan 2022)|BOLD ...
Micropsectra klinki|ZMUO.029790|Marko Mutanen||BOLD:AAW0057
Micropsectra klinki|BIOUG36624-H01|Kate Perez|BIN Taxonomy Match (Jul 2018)|BOLD:AAW0057
Micropsectra klinki|CH-OSF18|Elisabeth Stur||BOLD:AAW0057
Micropsectra logani|TRD-CH276|Elisabeth Stur||BOLD:AAB3857
Micropsectra logani|BIOUG16259-F07|Kate Perez|BIN Taxonomy Match|BOLD:AAB3857
Micropsectra logani|Finnmark555|Elisabeth Stur||BOLD:AAB3857
Micropsectra logani|PK-190-38|Petra Kranzfelder|morphology
Micropsectra logani|PK-190-39|Petra Kranzfelder|morphology
Micropsectra logani|PK-188-2|Petra Kranzfelder|morphology|BOLD:AAB3857
Micropsectra logani|BIOUG15538-D01|Kate Perez|BIN Taxonomy Match|BOLD:AAB3857
Micropsectra logani|PK-191-20|Petra Kranzfelder|morphology|BOLD:AAB3857
Micropsectra logani|PK-191-22|Petra Kranzfelder|morphology|BOLD:AAB3857
Micropsectra logani|PK-191-23|Petra Kranzfelder|morphology|BOLD:AAB3857
Micropsectra logani|PK-192-18|Petra Kranzfelder|morphology|BOLD:AAB3857
Micropsectra logani|PK-192-20|Petra Kranzfelder|morphology|BOLD:AAB3857
Micropsectra logani|BIOUG16328-B03|Kate Perez|BIN Taxonomy Match|BOLD:AAB3857
Micropsectra logani|TRD-CH401|Elisabeth Stur|morphology|BOLD:AAB3857
Micropsectra logani|BIOUG16625-A03|Kate Perez|BIN Taxonomy Match|BOLD:AAB3857
Micropsectra logani|BIOUG16328-G02|Kate Perez|BIN Taxonomy Match|BOLD:AAB3857
Micropsectra logani|BIOUG16331-A12|Kate Perez|BIN Taxonomy Match|BOLD:AAB3857
Micropsectra logani|BIOUG16261-A01|Kate Perez|BIN Taxonomy Match|BOLD:AAB3857
Micropsectra logani|BIOUG17025-C08|Kate Perez|BIN Taxonomy Match|BOLD:AAB3857
Micropsectra logani|BIOUG16329-A09|Kate Perez|BIN Taxonomy Match|BOLD:AAB3857
Micropsectra logani|BIOUG16329-A12|Kate Perez|BIN Taxonomy Match|BOLD:AAB3857
Micropsectra logani|BIOUG16478-F11|Kate Perez|BIN Taxonomy Match|BOLD:AAB3857
Micropsectra logani|BIOUG16332-E05|Kate Perez|BIN Taxonomy Match|BOLD:AAB3857
Micropsectra logani|BIOUG16479-A05|Kate Perez|BIN Taxonomy Match|BOLD:AAB3857
Micropsectra logani|BIOUG16435-C02|Kate Perez|BIN Taxonomy Match|BOLD:AAB3857
Micropsectra logani|BIOUG16626-F04|Kate Perez|BIN Taxonomy Match|BOLD:AAB3857
Micropsectra logani|BIOUG16297-E01|Kate Perez|BIN Taxonomy Match|BOLD:AAB3857
Micropsectra logani|BIOUG16332-D03|Kate Perez|BIN Taxonomy Match|BOLD:AAB3857
Micropsectra logani|BIOUG15963-D04|Kate Perez|BIN Taxonomy Match|BOLD:AAB3857
Micropsectra logani|BIOUG15465-E07|Kate Perez|BIN Taxonomy Match|BOLD:AAB3857
Micropsectra logani|Finnmark42|Elisabeth Stur||BOLD:AAB3857
Micropsectra logani|ZMUO.024584|Lauri Paasivirta||BOLD:AAB3857
Micropsectra logani|EBAI-Ch013|Elisabeth Stur|morphology|BOLD:AAB3857
Micropsectra logani|ATNA17|Elisabeth Stur||BOLD:AAB3857
Micropsectra logani|BIOUG16299-B04|Kate Perez|BIN Taxonomy Match|BOLD:AAB3857
Micropsectra logani|BIOUG16333-G11|Kate Perez|BIN Taxonomy Match|BOLD:AAB3857
Micropsectra logani|TRD-CH165|Elisabeth Stur||BOLD:AAB3857
Micropsectra logani|PK-192-17|Petra Kranzfelder|morphology|BOLD:AAB3857
Micropsectra logani|PK-189-2|Petra Kranzfelder|morphology|BOLD:AAB3857
Micropsectra logani|ZSM-DIP-33141-E08|Caroline Chimeno|BIN Taxonomy Match (Jan 2022)|BOLD ...
Micropsectra logani|ATNA165|Elisabeth Stur||BOLD:AAB3857
Micropsectra logani|ATNA177|Elisabeth Stur||BOLD:AAB3857
Micropsectra logani|ATNA411|Elisabeth Stur||BOLD:AAB3857
Micropsectra logani|ZMUO.026270|Lauri Paasivirta||BOLD:AAB3857
Micropsectra logani|ATNA27|Elisabeth Stur||BOLD:AAB3857
Micropsectra logani|BIOUG15545-D12|Kate Perez|BIN Taxonomy Match|BOLD:AAB3857
Micropsectra logani|ATNA36|Elisabeth Stur||BOLD:AAB3857
Micropsectra logani|EBAI-Ch014|Elisabeth Stur|morphology|BOLD:AAB3857
Micropsectra logani|BIOUG15654-H02|Kate Perez|BIN Taxonomy Match|BOLD:AAB3857
Micropsectra logani|BIOUG16331-B08|Kate Perez|BIN Taxonomy Match|BOLD:AAB3857
Micropsectra logani|BIOUG16260-G10|Kate Perez|BIN Taxonomy Match|BOLD:AAB3857
Micropsectra logani|BIOUG16333-B02|Kate Perez|BIN Taxonomy Match|BOLD:AAB3857
Micropsectra logani|ATNA22|Elisabeth Stur||BOLD:AAB3857
Micropsectra logani|ATNA121|Elisabeth Stur||BOLD:AAB3857
Micropsectra logani|ATNA35|Elisabeth Stur||BOLD:AAB3857
Micropsectra logani|ATNA34|Elisabeth Stur||BOLD:AAB3857
Micropsectra logani|ATNA183|Elisabeth Stur||BOLD:AAB3857
Micropsectra logani|BIOUG16328-A02|Kate Perez|BIN Taxonomy Match|BOLD:AAB3857
Micropsectra logani|BIOUG15537-E02|Kate Perez|BIN Taxonomy Match|BOLD:AAB3857
Micropsectra logani|ZMUO.024585|Lauri Paasivirta||BOLD:AAB3857
Micropsectra logani|To33|Torbjorn Ekrem||BOLD:AAB3857
Micropsectra logani|PK-191-14|Petra Kranzfelder|morphology|BOLD:AAB3857
Micropsectra logani|PK-192-16|Petra Kranzfelder|morphology|BOLD:AAB3857
Micropsectra logani|PK-190-36|Petra Kranzfelder|morphology|BOLD:AAB3857
Micropsectra logani|BIOUG16331-D05|Kate Perez|BIN Taxonomy Match|BOLD:AAB3857
Micropsectra logani|BIOUG16298-A02|Kate Perez|BIN Taxonomy Match|BOLD:AAB3857
Micropsectra logani|BIOUG16257-F10|Kate Perez|BIN Taxonomy Match|BOLD:AAB3857
Micropsectra logani|BIOUG16297-B12|Kate Perez|BIN Taxonomy Match|BOLD:AAB3857
Micropsectra logani|BIOUG16259-B01|Kate Perez|BIN Taxonomy Match|BOLD:AAB3857
Micropsectra logani|BIOUG16256-D10|Kate Perez|BIN Taxonomy Match|BOLD:AAB3857
Micropsectra logani|BIOUG16256-E10|Kate Perez|BIN Taxonomy Match|BOLD:AAB3857
Micropsectra logani|BIOUG16259-A04|Kate Perez|BIN Taxonomy Match|BOLD:AAB3857
Micropsectra logani|BIOUG16260-A08|Kate Perez|BIN Taxonomy Match|BOLD:AAB3857
Micropsectra logani|BIOUG16296-D11|Kate Perez|BIN Taxonomy Match|BOLD:AAB3857
Micropsectra logani|BIOUG16627-A12|Kate Perez|BIN Taxonomy Match|BOLD:AAB3857
Micropsectra logani|BIOUG16631-G06|Kate Perez|BIN Taxonomy Match|BOLD:AAB3857
Micropsectra logani|BIOUG16296-F07|Kate Perez|BIN Taxonomy Match|BOLD:AAB3857
Micropsectra logani|BIOUG16330-C06|Kate Perez|BIN Taxonomy Match|BOLD:AAB3857
Micropsectra logani|BIOUG16332-F04|Kate Perez|BIN Taxonomy Match|BOLD:AAB3857
Micropsectra logani|BIOUG16332-B05|Kate Perez|BIN Taxonomy Match|BOLD:AAB3857
Micropsectra logani|Finnmark09|Elisabeth Stur||BOLD:AAB3857
Micropsectra logani|Finnmark132|Elisabeth Stur||BOLD:AAB3857
Micropsectra logani|PK-188-1|Petra Kranzfelder|morphology|BOLD:AAB3857
Micropsectra logani|BIOUG16299-E10|Kate Perez|BIN Taxonomy Match|BOLD:AAB3857
Micropsectra logani|Finnmark378|Elisabeth Stur||BOLD:AAB3857
Micropsectra pallidula|ZSM-DIP-33137-A04|Caroline Chimeno|BIN Taxonomy Match (Jan 2022)|BOL...
Micropsectra pallidula|ZSM-DIP-33144-F08|Caroline Chimeno|BIN Taxonomy Match (Jan 2022)|BOL...
Micropsectra pallidula|ZSM-DIP-33146-E08|Caroline Chimeno|BIN Taxonomy Match (Jan 2022)|BOL...

Micropsectra pallidula|ZSM-DIP-33137-A04|Caroline Chimeno|BIN Taxonomy Match (Jan 2022)|BOL...
Micropsectra pallidula|ZSM-DIP-33144-F08|Caroline Chimeno|BIN Taxonomy Match (Jan 2022)|BOL...
Micropsectra pallidula|ZSM-DIP-33146-E08|Caroline Chimeno|BIN Taxonomy Match (Jan 2022)|BOL...
Micropsectra pallidula|CH-eik25|Elisabeth Stur||BOLD:AEE6875
Micropsectra pallidula|ZSM-DIP-33129-F12|Caroline Chimeno|BIN Taxonomy Match (Jan 2022)|BOL...
Micropsectra pallidula|ZMUO.024673|Lauri Paasivirta||BOLD:AEE6875
Micropsectra pallidula|BIOUG07505-D05|Kate Perez|BIN Taxonomy Match|BOLD:AEE6875
Micropsectra pallidula|ZSM-DIP-33136-E03|Caroline Chimeno|BIN Taxonomy Match (Jan 2022)|BOL...
Micropsectra pallidula|ZSM-DIP-33140-C02|Caroline Chimeno|BIN Taxonomy Match (Jan 2022)|BOL...
Micropsectra pallidula|ZSM-DIP-33172-G07|Caroline Chimeno|BIN Taxonomy Match (Jan 2022)|BOL...
Micropsectra pallidula|ZSM-DIP-33128-E06|Caroline Chimeno|BIN Taxonomy Match (Jan 2022)|BOL...
Micropsectra pallidula|ZSM-DIP-33128-E07|Caroline Chimeno|BIN Taxonomy Match (Jan 2022)|BOL...
Micropsectra pallidula|ZSM-DIP-33146-E07|Caroline Chimeno|BIN Taxonomy Match (Jan 2022)|BOL...
Micropsectra pallidula|ZSM-DIP-33129-F10|Caroline Chimeno|BIN Taxonomy Match (Jan 2022)|BOL...
Micropsectra pallidula|To74|Torbjorn Ekrem||BOLD:AEE6875
Micropsectra pallidula|To34|Torbjorn Ekrem||BOLD:AEE6875
Micropsectra pallidula|SOE135|Elisabeth Stur||BOLD:AEE6875
Micropsectra pallidula|SOE136|Elisabeth Stur||BOLD:AEE6875
Micropsectra pallidula|SOE144|Elisabeth Stur||BOLD:AEE6875
Micropsectra pallidula|ZMUO.024672|Lauri Paasivirta||BOLD:AEE6875
Micropsectra pallidula|SOE190|Elisabeth Stur||BOLD:AEE6875
Micropsectra pallidula|FN808218|||BOLD:AEE6875
Micropsectra pallidula|CH-OSF05|Elisabeth Stur||BOLD:AEE6875
Micropsectra pallidula|ZSM34343-G12|Sofia Wiedenbrug|Morphology|BOLD:AEE6875
Micropsectra pallidula|BIOUG16784-B03|Kate Perez|BIN Taxonomy Match|BOLD:AEE6875
Micropsectra pallidula|CCDB24039-F11|Sofia Wiedenbrug|BIN Taxonomy Match|BOLD:AEE6875
Micropsectra pallidula|BIOUG16776-D04|Kate Perez|BIN Taxonomy Match|BOLD:AEE6875
Micropsectra pallidula|ZSM34343-H09|Sofia Wiedenbrug|Morphology|BOLD:AAC7552
Micropsectra pallidula|ES240|Elisabeth Stur||BOLD:AEE6875
Micropsectra pallidula|SOE382|Elisabeth Stur||BOLD:AEE6875
Micropsectra pallidula|BIOUG08138-E09|Kate Perez|BIN Taxonomy Match|BOLD:AEE6875
Micropsectra pallidula|Finnmark346|Elisabeth Stur||BOLD:AEE6875
Micropsectra cf. roseiventris|CCDB24183-G01|Sofia Wiedenbrug|Morphology|BOLD:AAF7001
Micropsectra roseiventris|To84|Torbjorn Ekrem||BOLD:AAF7001
Micropsectra roseiventris|ZSM-DIP-33170-E11|Caroline Chimeno|BIN Taxonomy Match (Jan 2022)|...
Micropsectra roseiventris|CCDB24183-H07|Sofia Wiedenbrug|BIN-taxonomy|BOLD:AAF7002
Micropsectra roseiventris|BIOUG08253-E04|Kate Perez|BIN Taxonomy Match|BOLD:AAF7002
Micropsectra roseiventris|CCDB24183-B07|Sofia Wiedenbrug|Morphology|BOLD:AAF7002
Micropsectra roseiventris|ZMUO.024516|Lauri Paasivirta||BOLD:AAF7002
Micropsectra roseiventris|CH-OSF16|Elisabeth Stur||BOLD:AAF7002
Micropsectra roseiventris|CH-OSF178|Elisabeth Stur||BOLD:AAF7002
Micropsectra roseiventris|Finnmark658|Elisabeth Stur||BOLD:AAF7002
Micropsectra roseiventris|Finnmark655|Elisabeth Stur||BOLD:AAF7002
Micropsectra roseiventris|CH-OSF179|Elisabeth Stur||BOLD:AAF7002
Micropsectra roseiventris|ATNA46|Elisabeth Stur||BOLD:AAF7002
Micropsectra roseiventris|ZMUO.024515|Lauri Paasivirta||BOLD:AAF7002
Micropsectra roseiventris|CCDB24183-H04|Sofia Wiedenbrug|Morphology|BOLD:AAF7002
Micropsectra roseiventris|Finnmark656|Elisabeth Stur||BOLD:AAF7002
Micropsectra roseiventris|Finnmark74|Elisabeth Stur||BOLD:AAF7002
Micropsectra roseiventris|Finnmark528|Elisabeth Stur||BOLD:AAF7002
Micropsectra cf. longicrista|CCDB22605-F10|Sofia Wiedenbrug||BOLD:ACT9203
Micropsectra cf. longicrista|CCDB22605-F12|Sofia Wiedenbrug||BOLD:ACT9203
Micropsectra cf. longicrista|CCDB22605-F08|Sofia Wiedenbrug||BOLD:ACT9203
Micropsectra cf. longicrista|CCDB22605-F11|Sofia Wiedenbrug||BOLD:ACT9203
Micropsectra cf. longicrista|CCDB22605-F09|Sofia Wiedenbrug||BOLD:ACT9203
Micropsectra sp. 5SW|CCDB24183-G03|Sofia Wiedenbrug|Morphology|BOLD:ADC3829
Micropsectra sp. 5SW|CCDB24183-G02|Sofia Wiedenbrug|Morphology|BOLD:ADC3829
Micropsectra sp. 5SW|CCDB24183-H05|Sofia Wiedenbrug|Morphology|BOLD:ADC3829
Micropsectra sp. 5SW|CCDB24183-C07|Sofia Wiedenbrug|Morphology|BOLD:ADC3829
Micropsectra appendica|BIOUG07265-F11|Kate Perez|BIN Taxonomy Match|BOLD:AAE0593
Micropsectra appendica|BIOUG43948-A09|Kate Perez|BIN Taxonomy Match below Phylum (Jan 2019)...
Micropsectra appendica|BIOUG08133-E01|Kate Perez|BIN Taxonomy Match|BOLD:AAE0593
Micropsectra appendica|ATNA49|Elisabeth Stur||BOLD:AAE0593
Micropsectra appendica|BIOUG07509-F09|Kate Perez|BIN Taxonomy Match|BOLD:AAE0593
Micropsectra appendica|BIOUG07262-F06|Kate Perez|BIN Taxonomy Match|BOLD:AAE0593
Micropsectra appendica|BIOUG07563-A04|Kate Perez|BIN Taxonomy Match|BOLD:AAE0593
Micropsectra appendica|ATNA317|Elisabeth Stur||BOLD:AAE0593
Micropsectra appendica|CCDB24183-D02|Sofia Wiedenbrug|Morphology|BOLD:AAE0593
Micropsectra appendica|ZMUO.026310|Lauri Paasivirta||BOLD:AAE0593
Micropsectra appendica|ZMUO.026311|Lauri Paasivirta||BOLD:AAE0593
Micropsectra appendica|BIOUG08220-H04|Kate Perez|BIN Taxonomy Match|BOLD:AAE0593
Micropsectra appendica|BIOUG06049-E01|Kate Perez|BIN Taxonomy Match|BOLD:AAE0593
Micropsectra appendica|To133|Torbjorn Ekrem||BOLD:AAE0593
Micropsectra appendica|To132|Torbjorn Ekrem||BOLD:AAE0593
Tanytarsini|ZFMK-TIS-2534213|||BOLD:AAD4167
Micropsectra atrofasciata|ZMUO.024558|Lauri Paasivirta||BOLD:AAD4167
Micropsectra atrofasciata|ZSM-DIP-33138-C12|Caroline Chimeno|BIN Taxonomy Match (Jan 2022)|...
Micropsectra atrofasciata|GBOL 01212|Jerome Moriniere||BOLD:AAD4167
Micropsectra atrofasciata|ZMUO.024762|Lauri Paasivirta||BOLD:AAD4167
Micropsectra atrofasciata|ZMUO.024761|Lauri Paasivirta||BOLD:AAD4167
Micropsectra atrofasciata|To152|Elisabeth Stur||BOLD:AAD4167
Micropsectra atrofasciata|ZSM-DIP-33143-E11|Caroline Chimeno|BIN Taxonomy Match (Jan 2022)|...
Micropsectra sp. 5ES|Finnmark244|Torbjorn Ekrem||BOLD:AAI1530
Micropsectra atrofasciata|ZSM-DIP-33172-B05|Caroline Chimeno|BIN Taxonomy Match (Jan 2022)|...
Micropsectra sp. 5ES|TRD-CH154|Elisabeth Stur|morphology|BOLD:AAI1530
Micropsectra sp. 5ES|TRD-CH355|Elisabeth Stur|morphology|BOLD:AAI1530
Micropsectra atrofasciata|ZSM-DIP-33172-B01|Caroline Chimeno|BIN Taxonomy Match (Jan 2022)|...
Micropsectra sp. 5ES|BC-ZSM-DIP-22551-E10|Dieter Doczkal||BOLD:AAI1530
Micropsectra sp. 5ES|BIOUG17440-F03|Kate Perez|BIN Taxonomy Match|BOLD:AAI1530
Micropsectra sp. 5ES|EBAI-Ch011|Elisabeth Stur|morphology|BOLD:AAI1530
Micropsectra sp. 5ES|ATNA319|Elisabeth Stur||BOLD:AAI1530
Micropsectra sp. 5ES|Finnmark499|Elisabeth Stur||BOLD:AAI1530
Micropsectra sp. 5ES|ATNA323|Elisabeth Stur||BOLD:AAI1530
Micropsectra sp. 5ES|ATNA322|Elisabeth Stur||BOLD:AAI1530
Micropsectra sp. 5ES|ATNA321|Elisabeth Stur||BOLD:AAI1530
Micropsectra sp. 5ES|ATNA20|Elisabeth Stur||BOLD:AAI1530
Micropsectra atrofasciata|ZSM-DIP-33141-C03|Caroline Chimeno|BIN Taxonomy Match (Jan 2022)|...
Micropsectra sp. 5ES|Finnmark835|Elisabeth Stur||BOLD:AAI1530
Micropsectra sp. 5ES|ATNA320|Elisabeth Stur||BOLD:AAI1530
Micropsectra sp. 5ES|Finnmark494|Elisabeth Stur||BOLD:AAI1530
Micropsectra sp. 5ES|EBAI-Ch010|Elisabeth Stur|morphology|BOLD:AAI1530
Micropsectra sp. 5ES|ATNA318|Elisabeth Stur||BOLD:AAI1530
Micropsectra atrofasciata|ZSM-DIP-33143-E10|Caroline Chimeno|BIN Taxonomy Match (Jan 2022)|

[illegible]
Micropsectra sp. 5ES|EBAI-Ch010|Elisabeth Stur|morphology|BOLD:AAI1530
Micropsectra sp. 5ES|ATNA318|Elisabeth Stur||BOLD:AAI1530
Micropsectra atrofasciata|ZSM-DIP-33143-E10|Caroline Chimeno|BIN Taxonomy Match (Jan 2022)|...
Micropsectra sp. 5ES|TRD-CH380|Elisabeth Stur|morphology|BOLD:AAI1530
Micropsectra|BIOUG16298-H01|Kate Perez|BIN Taxonomy Match|BOLD:AAI1530
Micropsectra|BIOUG42764-C08|Kate Perez|BIN Taxonomy Match below Phylum (Jun 2019)|BOLD:A...
Micropsectra atrofasciata|ZSM-DIP-33131-H09|Caroline Chimeno|BIN Taxonomy Match (Jan 2022)|...
Micropsectra sp. 5ES|CH-OSF51|Elisabeth Stur||BOLD:AAI1530
Micropsectra sp. 5ES|CH-OSF119|Elisabeth Stur||BOLD:AAI1530
Micropsectra sp. 5ES|CH-OSF101|Elisabeth Stur||BOLD:AAI1530
Micropsectra sp. 5ES|BIOUG17096-D12|Kate Perez|BIN Taxonomy Match|BOLD:AAI1530
Micropsectra sp. 5ES|CH-OSF45|Elisabeth Stur||BOLD:AAI1530
Micropsectra sp. 5ES|CH-OSF19|Elisabeth Stur||BOLD:AAI1530
Micropsectra sp. 5ES|ATNA517|Elisabeth Stur||BOLD:AAI1530
Micropsectra atrofasciata|ZSM-DIP-33148-F10|Caroline Chimeno|BIN Taxonomy Match (Jan 2022)|...
Micropsectra atrofasciata|ZSM-DIP-33141-C04|Caroline Chimeno|BIN Taxonomy Match (Jan 2022)|...
Micropsectra atrofasciata|ZSM-DIP-33172-C10|Caroline Chimeno|BIN Taxonomy Match (Jan 2022)|...
Micropsectra sp. 5ES|Finnmark837|Elisabeth Stur||BOLD:AAI1530
Micropsectra sp. 5ES|ATNA53|Elisabeth Stur||BOLD:AAI1530
Micropsectra atrofasciata|ZMUO.024557|Lauri Paasivirta||BOLD:AAI1530
Micropsectra sp. 5ES|PK-189-1|Elisabeth Stur|BIN Taxonomy Match?|BOLD:AAI1530
Micropsectra sp. 5ES|CCDB24228-F09|Sofia Wiedenbrug|BIN Taxonomy Match|BOLD:AAI1530
Micropsectra sp. 5ES|CCDB24228-E06|Sofia Wiedenbrug|BIN Taxonomy Match|BOLD:AAI1530
Micropsectra|BIOUG15905-H11|Kate Perez|BIN Taxonomy Match|BOLD:AAI1530
Micropsectra sp. 5ES|Finnmark500|Elisabeth Stur||BOLD:AAI1530
Micropsectra sp. 5ES|Finnmark245|Torbjorn Ekrem||BOLD:AAI1530
Micropsectra sp. 5ES|Finnmark148|Elisabeth Stur||BOLD:AAI1530
Micropsectra sp. 5ES|Finnmark448|Elisabeth Stur||BOLD:AAI1530
Micropsectra calcifontis|ZMUO.026347|Lauri Paasivirta||BOLD:ADF0014
Micropsectra calcifontis|ZMUO.026346|Lauri Paasivirta||BOLD:ACR4872
Micropsectra|CCDB24228-E07|Sofia Wiedenbrug|BIN Taxonomy Match|BOLD:ACU4582
Micropsectra|BC-ZSM-DIP-22488-F08|Dieter Doczkal||BOLD:ACU4582
Micropsectra|CCDB24228-C06|Sofia Wiedenbrug|BIN Taxonomy Match|BOLD:ACU4582
Micropsectra|CCDB24037-C05|Sofia Wiedenbrug|BIN Taxonomy Match|BOLD:ACU4582
Micropsectra|BC-ZSM-DIP-22551-C04|Dieter Doczkal||BOLD:ACU4582
Micropsectra oberaarensis|To349|Torbjorn Ekrem||BOLD:AAG0291
Micropsectra oberaarensis|To336|Torbjorn Ekrem||BOLD:AAG0291
Micropsectra seguyi|To334|Elisabeth Stur||BOLD:AAE8101
Micropsectra seguyi|To333|Elisabeth Stur||BOLD:AAE8101
Micropsectra sp. To391|ZSM34342-E12|Sofia Wiedenbrug|BIN-taxonomy|BOLD:ACB3649
Micropsectra attenuata|ZSM-DIP-33141-G12|Caroline Chimeno|BIN Taxonomy Match (Jan 2022)|BOL...
Micropsectra attenuata|ES80|Elisabeth Stur||BOLD:AAC7793
Micropsectra attenuata|SOE244|Elisabeth Stur||BOLD:AAC7793
Micropsectra attenuata|SOE224|Elisabeth Stur||BOLD:AAC7793
Micropsectra attenuata|To302|Torbjorn Ekrem||BOLD:AAC7793
Micropsectra attenuata|To131|Torbjorn Ekrem||BOLD:AAC7793
Micropsectra attenuata|ES78|Torbjorn Ekrem||BOLD:AAC7793
Micropsectra attenuata|To109|Elisabeth Stur||BOLD:AAC7793
Micropsectra lacustris|Aly02|Alyssa M. Anderson||BOLD:AAE8086
Micropsectra lacustris|Finnmark836|Elisabeth Stur||BOLD:AAE8086
Micropsectra lacustris|ATNA308|Elisabeth Stur||BOLD:AAE8086
Micropsectra lacustris|EBAI-Ch005|Elisabeth Stur|morphology|BOLD:AAE8086
Micropsectra lacustris|EBAI-Ch003|Elisabeth Stur|morphology|BOLD:AAE8086
Micropsectra lacustris|Finnmark761|Elisabeth Stur||BOLD:AAE8086
Micropsectra lacustris|Finnmark157|Elisabeth Stur||BOLD:AAE8086
Micropsectra lacustris|ATNA309|Elisabeth Stur||BOLD:AAE8086
Micropsectra lacustris|Aly01|Alyssa M. Anderson||BOLD:AAE8086
Micropsectra lacustris|Finnmark149|Elisabeth Stur||BOLD:AAE8086
Micropsectra lacustris|ATNA120|Elisabeth Stur||BOLD:AAE8086
Micropsectra lacustris|EBAI-Ch002|Elisabeth Stur|morphology|BOLD:AAE8086
Micropsectra lacustris|EBAI-Ch004|Elisabeth Stur|morphology|BOLD:AAE8086
Micropsectra lacustris|EBAI-Ch006|Elisabeth Stur|morphology|BOLD:AAE8086
Micropsectra lacustris|EBAI-Ch007|Elisabeth Stur|morphology|BOLD:AAE8086
Micropsectra lacustris|EBAI-Ch008|Elisabeth Stur|morphology|BOLD:AAE8086
Micropsectra lacustris|EBAI-Ch009|Elisabeth Stur|morphology|BOLD:AAE8086
Micropsectra lacustris|EBAI-Ch001|Elisabeth Stur|morphology|BOLD:AAE8086
Micropsectra lacustris|EBAI-Ch177|Elisabeth Stur|morphology|BOLD:AAE8086
Micropsectra lacustris|EBAI-Ch248|Elisabeth Stur||BOLD:AAE8086
Micropsectra lacustris|ATNA30|Elisabeth Stur||BOLD:AAE8086
Micropsectra lacustris|ATNA409|Elisabeth Stur||BOLD:AAE8086
Micropsectra styriaca|ZMUO.025829|Lauri Paasivirta||BOLD:ADF0546
Micropsectra styriaca|ZMUO.024693|Lauri Paasivirta||BOLD:ADF0731
Micropsectra styriaca|ZMUO.024692|Lauri Paasivirta||BOLD:ADF0731
Micropsectra styriaca|ZMUO.025828|Lauri Paasivirta||BOLD:AAV4675
Micropsectra styriaca|CH-OSF181|Elisabeth Stur||BOLD:AAV4675
Micropsectra|CCDB24228-H10|Sofia Wiedenbrug|BIN Taxonomy Match|BOLD:ACU5456
Micropsectra|CCDB24228-H11|Sofia Wiedenbrug|BIN Taxonomy Match|BOLD:ACU5456
Micropsectra|CCDB24228-H09|Sofia Wiedenbrug|BIN Taxonomy Match|BOLD:ACU5456
Micropsectra acuta|ES286|Torbjorn Ekrem||BOLD:AAK3960
Rheotanytarsus|BIOUG16614-G11|Kate Perez|BOLD ID Engine Manual|BOLD:ACR3994
Rheotanytarsus|BIOUG16612-H06|Kate Perez|BOLD ID Engine Manual|BOLD:ACR3994
Rheotanytarsus|BIOUG16614-D07|Kate Perez|BOLD ID Engine Manual|BOLD:ACR3994
Rheotanytarsus|BIOUG16647-D12|Kate Perez|BOLD ID Engine Manual|BOLD:ACR3994
Rheotanytarsus|BIOUG16619-D05|Kate Perez|BIN Taxonomy Match|BOLD:ACR3994
Rheotanytarsus|BIOUG16609-E09|Kate Perez|BOLD ID Engine Manual|BOLD:ACR3994
Rheotanytarsus|BIOUG16614-B12|Kate Perez|BOLD ID Engine Manual|BOLD:ACR3994
Rheotanytarsus|BIOUG15977-B06|Kate Perez|BOLD ID Engine Manual|BOLD:ACR3994
Rheotanytarsus|BIOUG16526-A05|Kate Perez|BOLD ID Engine Manual|BOLD:ACR3994
Rheotanytarsus|BIOUG16619-B11|Kate Perez|BIN Taxonomy Match|BOLD:ACR3994
Rheotanytarsus|BIOUG17224-G12|Kate Perez|BOLD ID Engine Manual|BOLD:ACR3994
Rheotanytarsus|BIOUG16613-F11|Kate Perez|BOLD ID Engine Manual|BOLD:ACR3994
Rheotanytarsus erignus|BIOUG16620-F04|Kate Perez|BIN Taxonomy Match (Feb 2020)|BOLD:ACR3994
Rheotanytarsus|BIOUG16619-C09|Kate Perez|BIN Taxonomy Match|BOLD:ACR3994
Rheotanytarsus|BIOUG16269-H03|Kate Perez|BOLD ID Engine Manual|BOLD:ACR3994
Rheotanytarsus|BIOUG16647-B02|Kate Perez|BOLD ID Engine Manual|BOLD:ACR3994
Rheotanytarsus|BIOUG16527-D07|Kate Perez|BOLD ID Engine Manual|BOLD:ACR3994
Rheotanytarsus erignus|BIOUG16620-D05|Kate Perez|BIN Taxonomy Match (Feb 2020)|BOLD:ACR3994
Rheotanytarsus|BIOUG16620-E10|Kate Perez|BIN Taxonomy Match|BOLD:ACR3994
Rheotanytarsus curtistylus|MZ004967|||BOLD:ACR3994
Rheotanytarsus|BIOUG17225-E07|Kate Perez|BOLD ID Engine Manual|BOLD:ACR3994
Rheotanytarsus|BIOUG16614-B04|Kate Perez|BOLD ID Engine Manual|BOLD:ACR3994
Rheotanytarsus|BIOUG16736-H03|Kate Perez|BOLD ID Engine Manual|BOLD:ACR3994
[illegible]

Rheotanytarsus|BIOUG17225-E07|Kate Perez|BOLD ID Engine Manual|BOLD:ACR3994
Rheotanytarsus|BIOUG16614-B04|Kate Perez|BOLD ID Engine Manual|BOLD:ACR3994
Rheotanytarsus|BIOUG16736-H03|Kate Perez|BOLD ID Engine Manual|BOLD:ACR3994
Rheotanytarsus|BIOUG36680-F05|Kate Perez|BIN Taxonomy Match (May 2018)|BOLD:ACR3994
Rheotanytarsus|BIOUG16612-B05|Kate Perez|BOLD ID Engine Manual|BOLD:ACR3994
Rheotanytarsus|BIOUG16620-H07|Kate Perez|BIN Taxonomy Match|BOLD:ACR3994
Rheotanytarsus|BIOUG16530-H09|Kate Perez|BOLD ID Engine Manual|BOLD:ACR3994
Rheotanytarsus|BIOUG16527-G10|Kate Perez|BOLD ID Engine Manual|BOLD:ACR3994
Rheotanytarsus|BIOUG16527-B04|Kate Perez|BOLD ID Engine Manual|BOLD:ACR3994
Rheotanytarsus|BIOUG16358-C06|Kate Perez|BOLD ID Engine Manual|BOLD:ACR3994
Rheotanytarsus|BIOUG16612-H05|Kate Perez|BOLD ID Engine Manual|BOLD:ACR3994
Rheotanytarsus|BIOUG16618-E01|Kate Perez|BOLD ID Engine Manual|BOLD:ACR3994
Rheotanytarsus|BIOUG17095-H05|Kate Perez|BOLD ID Engine Manual|BOLD:ACR3994
Rheotanytarsus|BIOUG16647-A12|Kate Perez|BOLD ID Engine Manual|BOLD:ACR3994
Rheotanytarsus|BIOUG16620-C12|Kate Perez|BIN Taxonomy Match|BOLD:ACR3994
Rheotanytarsus|BIOUG16360-D10|Kate Perez|BOLD ID Engine Manual|BOLD:ACR3994
Rheotanytarsus|BIOUG16530-F02|Kate Perez|BOLD ID Engine Manual|BOLD:ACR3994
Micropsectra pharetrophora|ES47|Elisabeth Stur||BOLD:AAE0131
Micropsectra pharetrophora|To12|Elisabeth Stur||BOLD:AAE0131
Micropsectra pharetrophora|To11|Elisabeth Stur||BOLD:AAE0131
Micropsectra cf. bavarica|To350|Torbjorn Ekrem||BOLD:AAX4341
Micropsectra sp. 3SW|CCDB21606-A11|Sofia Wiedenbrug||BOLD:ACT7402
Micropsectra sp. 2SW|CCDB21606-A04|Sofia Wiedenbrug||BOLD:ACT8179
Micropsectra sp. 2SW|ZSM34343-B01|Sofia Wiedenbrug|Morphology|BOLD:ACT8179
Micropsectra sp. 2SW|ZSM34342-F12|Sofia Wiedenbrug|Morphology|BOLD:ACT8179
Micropsectra sp. 2SW|BC-ZSM-DIP-22488-C11|Dieter Doczkal||BOLD:ACT8179
Micropsectra sp. 2SW|ZSM34342-E10|Sofia Wiedenbrug|Morphology|BOLD:ACT8179
Micropsectra sp. 2SW|CCDB24038-F01|Sofia Wiedenbrug|BIN Taxonomy Match|BOLD:ACT8179
Micropsectra sp. 4SW|ZSM34342-F11|Sofia Wiedenbrug|Morphology|BOLD:AAB9933
Micropsectra sofiae|SOE257|Torbjorn Ekrem||BOLD:AAB9933
Micropsectra sofiae|SOE221|Elisabeth Stur||BOLD:AAB9933
Micropsectra sofiae|SOE205|Elisabeth Stur||BOLD:AAB9933
Micropsectra sofiae|SOE12|Elisabeth Stur||BOLD:AAB9933
Micropsectra sofiae|SOE11|Elisabeth Stur||BOLD:AAB9933
Micropsectra sofiae|SOE238|Elisabeth Stur||BOLD:AAB9933
Micropsectra sofiae|ZMUO.026209|Lauri Paasivirta||BOLD:AAB9933
Micropsectra sofiae|ZMUO.026208|Lauri Paasivirta||BOLD:AAB9933
Micropsectra sofiae|ATNA29|Elisabeth Stur||BOLD:AAB9933
Micropsectra sofiae|To166|Elisabeth Stur||BOLD:AAB9933
Micropsectra sofiae|To145|Elisabeth Stur||BOLD:AAB9932
Micropsectra sofiae|To92|Elisabeth Stur||BOLD:AAB9932
Micropsectra schrankelae|SOE384|Elisabeth Stur||BOLD:AAC4138
Micropsectra schrankelae|SOE197|Torbjorn Ekrem||BOLD:AAC4138
Micropsectra schrankelae|To47|Elisabeth Stur||BOLD:AAC4138
Micropsectra schrankelae|To108|Elisabeth Stur||BOLD:AAC4138
Micropsectra schrankelae|To44|Elisabeth Stur||BOLD:AAC4138
Micropsectra schrankelae|To43|Elisabeth Stur||BOLD:AAC4138
Micropsectra schrankelae|To42|Elisabeth Stur||BOLD:AAC4138
Chironomidae|ZSM-DIP-33172-B07|Valerie Levesque-Beaudin|BIN Taxonomy Match (Sep 2021)|BOLD...
Chironomidae|ZSM-DIP-33148-F09|Valerie Levesque-Beaudin|BOLD ID Engine Manual (Sep 2021)|BO...
Chironomidae|ZSM-DIP-33149-G02|Valerie Levesque-Beaudin|BIN Taxonomy Match (Sep 2021)|BOLD...
Chironomidae|ZSM-DIP-33172-C09|Valerie Levesque-Beaudin|BIN Taxonomy Match (Sep 2021)|BOLD...
Chironomidae|ZSM-DIP-33172-B03|Valerie Levesque-Beaudin|BIN Taxonomy Match (Sep 2021)|BOLD...
Micropsectra borealis|NO42|Torbjorn Ekrem||BOLD:AAV1958
Micropsectra borealis|NO41|Torbjorn Ekrem||BOLD:AAV1958
Chironomidae|ZSM-DIP-33139-D11|Valerie Levesque-Beaudin|BIN Taxonomy Match (Sep 2021)|BOLD...
Chironomidae|ZSM-DIP-33133-H05|Valerie Levesque-Beaudin|BOLD ID Engine Manual (Sep 2021)|BO...
Micropsectra lindrothi|ZMUO.025296|Lauri Paasivirta||BOLD:AAD1527
Micropsectra lindrothi|NHRS-BYWS000001014|Yngve Brodin|morphology|BOLD:AAD1527
Micropsectra lindrothi|To361|Torbjorn Ekrem||BOLD:AAD1527
Micropsectra|BIOUG05141-D09|Kate Perez|BIN Taxonomy Match|BOLD:ACF2339
Micropsectra uliginosa|NO39|Torbjorn Ekrem||BOLD:AAX4333
Micropsectra chionophila|SOE04|Torbjorn Ekrem||BOLD:AAE7044
Micropsectra chionophila|BIOUG15571-E06|Kate Perez|BIN Taxonomy Match|BOLD:AAE7044
Micropsectra chionophila|To55|Torbjorn Ekrem||BOLD:AAE7044
Micropsectra chionophila|To40|Torbjorn Ekrem||BOLD:AAE7044
Micropsectra chionophila|To39|Torbjorn Ekrem||BOLD:AAE7044
Micropsectra nana|ZMUO.024959|Lauri Paasivirta||BOLD:ADA5812
Micropsectra nana|ZMUO.024958|Lauri Paasivirta||BOLD:ADA5812
Micropsectra|BIOUG05101-A09|Kate Perez|BIN Taxonomy Match|BOLD:ACE0523
Chironomidae|BIOUG05220-A06|Meredith Miller|Tree-based Identification (Feb 2019)
Micropsectra nana|To135|Elisabeth Stur||BOLD:AAE7238
Micropsectra nana|SOE383|Elisabeth Stur||BOLD:AAE7238
Micropsectra nana|SOD04|Sondre Dahle|Morphology|BOLD:AAE7238
Micropsectra nana|BIOUG05219-A06|Kate Perez|BIN Taxonomy Match|BOLD:AAE7238
Micropsectra nana|BIOUG05149-B12|Kate Perez|BIN Taxonomy Match|BOLD:AAE7238
Micropsectra nana|BIOUG05945-H06|Kate Perez|BIN Taxonomy Match|BOLD:AAE7238
Micropsectra nana|BIOUG05778-H09|Kate Perez|BIN Taxonomy Match|BOLD:AAE7238
Micropsectra nana|Finnmark690|Elisabeth Stur||BOLD:AAE7238
Micropsectra nana|CH-eik129|Elisabeth Stur||BOLD:AAE7238
Micropsectra nana|TRD-CH63|Elisabeth Stur||BOLD:AAE7238
Micropsectra nana|ATNA563|Elisabeth Stur||BOLD:AAE7238
Micropsectra nana|ZMUO.024660|Lauri Paasivirta||BOLD:AAE7238
Micropsectra nana|CCDB21605-B04|Sofia Wiedenbrug||BOLD:AAE7238
Micropsectra nana|BIOUG15592-B04|Kate Perez|BIN Taxonomy Match|BOLD:AAE7238
Micropsectra nana|BIOUG05216-H09|Kate Perez|BIN Taxonomy Match|BOLD:AAE7238
Micropsectra nana|BIOUG05108-G01|Kate Perez|BIN Taxonomy Match|BOLD:AAE7238
Micropsectra nana|BIOUG05149-F11|Kate Perez|BIN Taxonomy Match|BOLD:AAE7238
Micropsectra nana|BIOUG05149-D08|Kate Perez|BIN Taxonomy Match|BOLD:AAE7238
Micropsectra nana|BIOUG05215-F02|Kate Perez|BIN Taxonomy Match|BOLD:AAE7238
Micropsectra nana|BIOUG05483-C11|Kate Perez|BIN Taxonomy Match|BOLD:AAE7238
Micropsectra nana|CCDB24183-H08|Sofia Wiedenbrug|BIN-taxonomy|BOLD:AAE7238
Micropsectra nana|BIOUG15800-A09|Kate Perez|BIN Taxonomy Match|BOLD:AAE7238
Micropsectra nana|ATNA25|Elisabeth Stur||BOLD:AAE7238
Micropsectra nana|TRD-CH128|Elisabeth Stur||BOLD:AAE7238
Micropsectra nana|BIOUG06054-E09|Kate Perez|BIN Taxonomy Match|BOLD:AAE7238
Micropsectra nana|BIOUG05395-F09|Kate Perez|BIN Taxonomy Match|BOLD:AAE7238
Micropsectra nana|CH-eik83|Elisabeth Stur||BOLD:AAE7238
Micropsectra nana|ZMUO.024661|Lauri Paasivirta||BOLD:AAE7238
Micropsectra nana|BIOUG05395-E04|Kate Perez|BIN Taxonomy Match|BOLD:AAE7238
Micropsectra nana|BIOUG05216-E08|Kate Perez|BIN Taxonomy Match|BOLD:AAE7238
Micropsectra nana|BIOUG05216-G10|Kate Perez|BIN Taxonomy Match|BOLD:AAE7238
Micropsectra nana|BIOUG05396-E10|Kate Perez|BIN Taxonomy Match|BOLD:AAE7238

Micropsectra nana|BIOUG05216-E08|Kate Perez|BIN Taxonomy Match|BOLD:AAE7238
Micropsectra nana|BIOUG05216-G10|Kate Perez|BIN Taxonomy Match|BOLD:AAE7238
Micropsectra nana|BIOUG05396-E10|Kate Perez|BIN Taxonomy Match|BOLD:AAE7238
Micropsectra nana|BIOUG05485-F09|Kate Perez|BIN Taxonomy Match|BOLD:AAE7238
Micropsectra nana|BIOUG05215-A05|Kate Perez|BIN Taxonomy Match|BOLD:AAE7238
Micropsectra nana|BIOUG05216-C09|Kate Perez|BIN Taxonomy Match|BOLD:AAE7238
Micropsectra nana|BIOUG05484-H11|Kate Perez|BIN Taxonomy Match|BOLD:AAE7238
Micropsectra nana|BIOUG05215-H05|Kate Perez|BIN Taxonomy Match|BOLD:AAE7238
Micropsectra nana|BIOUG06052-G08|Kate Perez|BIN Taxonomy Match|BOLD:AAE7238
Micropsectra nana|BIOUG05216-F12|Kate Perez|BIN Taxonomy Match|BOLD:AAE7238
Micropsectra nana|CH-eik76|Elisabeth Stur||BOLD:AAE7238
Micropsectra nana|BIOUG05218-G03|Kate Perez|BIN Taxonomy Match|BOLD:AAE7238
Micropsectra nana|BIOUG05101-B04|Kate Perez|BIN Taxonomy Match|BOLD:AAE7238
Micropsectra nana|BIOUG06053-D03|Kate Perez|BIN Taxonomy Match|BOLD:AAE7238
Micropsectra nana|BIOUG05218-G06|Kate Perez|BIN Taxonomy Match|BOLD:AAE7238
Micropsectra nana|BIOUG05219-G10|Kate Perez|BIN Taxonomy Match|BOLD:AAE7238
Micropsectra nana|BIOUG05215-G01|Kate Perez|BIN Taxonomy Match|BOLD:AAE7238
Micropsectra nana|BIOUG05218-C11|Kate Perez|BIN Taxonomy Match|BOLD:AAE7238
Micropsectra nana|BIOUG05217-H03|Kate Perez|BIN Taxonomy Match|BOLD:AAE7238
Micropsectra nana|BIOUG06052-H06|Kate Perez|BIN Taxonomy Match|BOLD:AAE7238
Micropsectra nana|BIOUG05484-D09|Kate Perez|BIN Taxonomy Match|BOLD:AAE7238
Micropsectra nana|BC-ZSM-DIP-22551-E01|Dieter Doczkal||BOLD:AAE7238
Micropsectra nana|BIOUG05486-E08|Kate Perez|BIN Taxonomy Match|BOLD:AAE7238
Micropsectra nana|BIOUG08180-G08|Kate Perez|BIN Taxonomy Match|BOLD:AAE7238
Micropsectra nana|BIOUG06087-H01|Kate Perez|BIN Taxonomy Match|BOLD:AAE7238
Micropsectra nana|BIOUG05267-D12|Kate Perez|BIN Taxonomy Match|BOLD:AAE7238
Micropsectra nana|BIOUG06094-G08|Kate Perez|BIN Taxonomy Match|BOLD:AAE7238
Micropsectra nana|CCDB24183-F07|Sofia Wiedenbrug|Morphology|BOLD:AAE7238
Micropsectra nana|BIOUG05219-F06|Kate Perez|BIN Taxonomy Match|BOLD:AAE7238
Micropsectra nana|BIOUG05947-A01|Kate Perez|BIN Taxonomy Match|BOLD:AAE7238
Micropsectra lindebergi|ZMUO.024605|Lauri Paasivirta||BOLD:ABY9720
Micropsectra insignilobus|ZMUO.024309|Lauri Paasivirta||BOLD:ABY9720
Micropsectra insignilobus|ZMUO.024308|Lauri Paasivirta||BOLD:ABY9720
Micropsectra lindebergi|ZMUO.024512|Lauri Paasivirta||BOLD:ABY9720
Micropsectra lindebergi|ZMUO.024511|Lauri Paasivirta||BOLD:ABY9720
Micropsectra lindebergi|ZMUO.024604|Lauri Paasivirta||BOLD:ABY9720
Micropsectra insignilobus|Finnmark68|Elisabeth Stur||BOLD:ABY9720
Micropsectra insignilobus|ZMUO.024286|Lauri Paasivirta||BOLD:ABY9720
Micropsectra insignilobus|Finnmark524|Elisabeth Stur||BOLD:ABY9720
Micropsectra insignilobus|ZMUO.024287|Lauri Paasivirta||BOLD:ACF1868
Micropsectra insignilobus|Finnmark682|Elisabeth Stur||BOLD:ACF1868
Micropsectra insignilobus|TRD-CH2|Elisabeth Stur||BOLD:AAB9837
Micropsectra insignilobus|To31|Torbjorn Ekrem||BOLD:AAB9837
Micropsectra insignilobus|Finnmark376|Elisabeth Stur||BOLD:ADT7628
Micropsectra notescens|To352|Torbjorn Ekrem||BOLD:AAC4099
Micropsectra notescens|To351|Torbjorn Ekrem||BOLD:AAC4099
Micropsectra notescens|To315|Elisabeth Stur||BOLD:AAC4099
Micropsectra apposita|ZMUO.024288|Lauri Paasivirta||BOLD:AAC7823
Micropsectra contracta|TRD-CH257|Elisabeth Stur||BOLD:AAC7823
Micropsectra contracta|To358|Torbjorn Ekrem||BOLD:AAC7823
Micropsectra|ZSM-DIP-33143-B05|Caroline Chimeno|BIN Taxonomy Match (Jan 2022)|BOLD:AAC7823
Micropsectra|ZSM-DIP-33134-B04|Caroline Chimeno|BIN Taxonomy Match (Jan 2022)|BOLD:AAC7823
Micropsectra contracta|GBOL11553|Sofia Wiedenbrug|Morphology|BOLD:AAC7823
Micropsectra|ZSM-DIP-33129-C11|Caroline Chimeno|BIN Taxonomy Match (Jan 2022)|BOLD:AAC7823
Micropsectra junci|NHRS-BYWS000000370|Yngve Brodin|morphology|BOLD:AAC7823
Micropsectra contracta|SOE283|Torbjorn Ekrem||BOLD:AAC7823
Micropsectra contracta|SOE50|Elisabeth Stur||BOLD:AAC7823
Micropsectra|ZSM-DIP-33172-F03|Caroline Chimeno|BIN Taxonomy Match (Jan 2022)|BOLD:AAC7823
Micropsectra|ZSM-DIP-33130-G12|Caroline Chimeno|BIN Taxonomy Match (Jan 2022)|BOLD:AAC7823
Micropsectra|ZSM-DIP-33130-F08|Caroline Chimeno|BIN Taxonomy Match (Jan 2022)|BOLD:AAC7823
Micropsectra|ZSM-DIP-33130-F07|Caroline Chimeno|BIN Taxonomy Match (Jan 2022)|BOLD:AAC7823
Micropsectra|ZSM-DIP-33130-H01|Caroline Chimeno|BIN Taxonomy Match (Jan 2022)|BOLD:AAC7823
Micropsectra|ZSM-DIP-33143-B04|Caroline Chimeno|BIN Taxonomy Match (Jan 2022)|BOLD:AAC7823
Micropsectra|ZSM-DIP-33143-C03|Caroline Chimeno|BIN Taxonomy Match (Jan 2022)|BOLD:AAC7823
Micropsectra|ZSM-DIP-33143-C04|Caroline Chimeno|BIN Taxonomy Match (Jan 2022)|BOLD:AAC7823
Micropsectra|ZSM-DIP-33143-C05|Caroline Chimeno|BIN Taxonomy Match (Jan 2022)|BOLD:AAC7823
Micropsectra|ZSM-DIP-33135-G01|Caroline Chimeno|BIN Taxonomy Match (Jan 2022)|BOLD:AAC7823
Micropsectra|ZSM-DIP-33135-G02|Caroline Chimeno|BIN Taxonomy Match (Jan 2022)|BOLD:AAC7823
Micropsectra|ZSM-DIP-33145-D01|Caroline Chimeno|BIN Taxonomy Match (Jan 2022)|BOLD:AAC7823
Micropsectra|ZSM-DIP-33170-A07|Caroline Chimeno|BIN Taxonomy Match (Jan 2022)|BOLD:AAC7823
Micropsectra|ZSM-DIP-33170-A08|Caroline Chimeno|BIN Taxonomy Match (Jan 2022)|BOLD:AAC7823
Micropsectra|ZSM-DIP-33149-C07|Caroline Chimeno|BIN Taxonomy Match (Jan 2022)|BOLD:AAC7823
Micropsectra|ZSM-DIP-33134-B05|Caroline Chimeno|BIN Taxonomy Match (Jan 2022)|BOLD:AAC7823
Micropsectra|ZSM-DIP-33170-A01|Caroline Chimeno|BIN Taxonomy Match (Jan 2022)|BOLD:AAC7823
Micropsectra contracta|Finnmark14|Elisabeth Stur||BOLD:AAC7823
Micropsectra apposita|ZMUO.024289|Lauri Paasivirta||BOLD:AAC7823
Micropsectra contracta|TRD-CH256|Elisabeth Stur||BOLD:AAC7823
Micropsectra contracta|BIOUG16331-D11|Kate Perez|BIN Taxonomy Match|BOLD:AAC7823
Micropsectra contracta|BIOUG16257-D11|Kate Perez|BIN Taxonomy Match|BOLD:AAC7823
Micropsectra contracta|CH-OSF46|Elisabeth Stur||BOLD:AAC7823
Micropsectra contracta|Finnmark10|Elisabeth Stur||BOLD:AAC7823
Micropsectra contracta|To356|Torbjorn Ekrem||BOLD:AAC7823
Micropsectra contracta|To357|Torbjorn Ekrem||BOLD:AAC7823
Micropsectra|ZSM-DIP-33140-H08|Caroline Chimeno|BIN Taxonomy Match (Jan 2022)|BOLD:AAC7823
Micropsectra contracta|To06|Torbjorn Ekrem||BOLD:AAC7823
Micropsectra|BIOUG36819-E02|Kate Perez|BIN Taxonomy Match (Jul 2018)|BOLD:AAC4098
Micropsectra|ZSM-DIP-33170-C08|Caroline Chimeno|BIN Taxonomy Match (Jan 2022)|BOLD:AAC4098
Micropsectra notescens|To353|Torbjorn Ekrem||BOLD:AAC4098
Micropsectra|ZSM-DIP-33138-C07|Caroline Chimeno|BIN Taxonomy Match (Jan 2022)|BOLD:AAC4098
Micropsectra|BIOUG42659-C12|Kate Perez|BIN Taxonomy Match below Phylum (Jun 2019)|BOLD:A...
Micropsectra|BIOUG43948-D09|Kate Perez|BIN Taxonomy Match below Phylum (Jan 2019)|BOLD:A...
Micropsectra|ZSM-DIP-33129-E09|Caroline Chimeno|BIN Taxonomy Match (Jan 2022)|BOLD:AAC4098
Micropsectra notescens|ZMUO.024827|Lauri Paasivirta||BOLD:AAC4098
Micropsectra notescens|CH-OSF120|Elisabeth Stur||BOLD:AAC4098
Micropsectra notescens|CH-OSF121|Elisabeth Stur||BOLD:AAC4098
Micropsectra notescens|ZMUO.024298|Lauri Paasivirta||BOLD:AAC4098
Micropsectra apposita|ZMUO.024310|Lauri Paasivirta||BOLD:AAC4098
Micropsectra notescens|ZMUO.024828|Lauri Paasivirta||BOLD:AAC4098
Micropsectra notescens|CH-OSF52|Elisabeth Stur||BOLD:AAC4098
Micropsectra notescens|CH-OSF47|Elisabeth Stur||BOLD:AAC4098
Micropsectra notescens|BIOUG05108-G03|Kate Perez|BIN Taxonomy Match|BOLD:AAC4098
Micropsectra notescens|To354|Elisabeth Stur||BOLD:AAC4098
Micropsectra notescens|To324|Elisabeth Stur||BOLD:AAC4098

[illegible]
Micropsectra notescens|BIOUG05108-G03|Kate Perez|BIN Taxonomy Match|BOLD:AAC4098
Micropsectra notescens|To354|Elisabeth Stur||BOLD:AAC4098
Micropsectra notescens|To324|Elisabeth Stur||BOLD:AAC4098
Micropsectra recurvata|TRD-CH1|Elisabeth Stur||BOLD:AAC4510
Micropsectra recurvata|SOE45|Torbjorn Ekrem||BOLD:AAC4510
Micropsectra recurvata|SOE254|Elisabeth Stur||BOLD:AAC4510
Micropsectra recurvata|NHRS-BYWS000001156|Yngve Brodin|morphology|BOLD:AAC4510
Micropsectra recurvata|SOE130|Elisabeth Stur||BOLD:AAC4510
Micropsectra recurvata|SOE129|Elisabeth Stur||BOLD:AAC4510
Micropsectra recurvata|ATNA1|Elisabeth Stur||BOLD:AAC4510
Micropsectra recurvata|CH-OSF188|Elisabeth Stur||BOLD:AAC4510
Micropsectra recurvata|Finnmark236|Elisabeth Stur||BOLD:AAC4510
Micropsectra recurvata|ZMUO.024526|Lauri Paasivirta||BOLD:AAC4510
Micropsectra recurvata|CCDB24183-B09|Sofia Wiedenbrug|Morphology|BOLD:AAC4510
Micropsectra recurvata|CCDB24183-B06|Sofia Wiedenbrug|Morphology|BOLD:AAC4510
Micropsectra recurvata|ZMUO.024525|Lauri Paasivirta||BOLD:AAC4510
Micropsectra recurvata|CCDB24183-B11|Sofia Wiedenbrug|BIN-taxonomy|BOLD:AAC4510
Micropsectra recurvata|ATNA316|Elisabeth Stur||BOLD:AAC4510
Micropsectra recurvata|SOE274|Elisabeth Stur||BOLD:AAC4510
Micropsectra recurvata|To38|Torbjorn Ekrem||BOLD:AAC4510
Chironomidae|ZSM-DIP-33139-C08|Caroline Chimeno|BIN Taxonomy Match (Jan 2022)|BOLD:ADS3982
Chironomidae|ZSM-DIP-33128-E09|Valerie Levesque-Beaudin|BOLD ID Engine Manual (Sep 2021)|BO...
Chironomidae|ZSM-DIP-33172-G06|Valerie Levesque-Beaudin|BIN Taxonomy Match (Sep 2021)|BOLD...
Chironomidae|ZSM-DIP-33128-E08|Valerie Levesque-Beaudin|BIN Taxonomy Match (Sep 2021)|BOLD...
Chironomidae|ZSM-DIP-33128-E11|Valerie Levesque-Beaudin|BIN Taxonomy Match (Sep 2021)|BOLD...
Chironomidae|ZSM-DIP-33129-D08|Valerie Levesque-Beaudin|BIN Taxonomy Match (Sep 2021)|BOLD...
Chironomidae|ZSM-DIP-33172-G05|Valerie Levesque-Beaudin|BIN Taxonomy Match (Sep 2021)|BOLD...
Micropsectra junci|ZSM-DIP-33170-E12|Caroline Chimeno|BIN Taxonomy Match (Jan 2022)|BOLD ...
Micropsectra junci|BIOUG04217-C07|Kate Perez|BIN Taxonomy Match|BOLD:AAA9962
Micropsectra junci|ZSM-DIP-33133-H06|Caroline Chimeno|BIN Taxonomy Match (Jan 2022)|BOLD ...
Micropsectra junci|SOE122|Torbjorn Ekrem||BOLD:AAA9962
Micropsectra junci|SOE119|Torbjorn Ekrem||BOLD:AAA9962
Micropsectra junci|SOE53|Torbjorn Ekrem||BOLD:AAA9962
Micropsectra junci|SOE46|Torbjorn Ekrem||BOLD:AAA9962
Micropsectra junci|To54|Torbjorn Ekrem||BOLD:AAA9962
Micropsectra junci|ATNA42|Elisabeth Stur||BOLD:ABZ3274
Micropsectra junci|SOE379|Torbjorn Ekrem||BOLD:ACE7506
Micropsectra junci|SOE381|Torbjorn Ekrem||BOLD:ACE7506
Micropsectra junci|SOE123|Torbjorn Ekrem||BOLD:ACE7506
Micropsectra junci|SOE444|Torbjorn Ekrem||BOLD:ACE7506
Micropsectra junci|SOE120|Torbjorn Ekrem||BOLD:ACE7506
Micropsectra junci|SOE282|Torbjorn Ekrem||BOLD:ACE7506
Micropsectra junci|SOE51|Elisabeth Stur||BOLD:ACE7506
Micropsectra junci|ATNA164|Elisabeth Stur||BOLD:ACE7506
Micropsectra junci|ATNA24|Elisabeth Stur||BOLD:ACE7506
Micropsectra junci|ATNA324|Elisabeth Stur||BOLD:ACE7506
Micropsectra junci|ATNA31|Elisabeth Stur||BOLD:ACE7506
Micropsectra junci|ATNA51|Elisabeth Stur||BOLD:ACE7506
Micropsectra junci|ATNA50|Elisabeth Stur||BOLD:ACE7506
Micropsectra junci|ATNA45|Elisabeth Stur||BOLD:ACE7506
Micropsectra junci|ATNA39|Elisabeth Stur||BOLD:ACE7506
Micropsectra junci|ATNA40|Elisabeth Stur||BOLD:ACE7506
Micropsectra junci|ATNA33|Elisabeth Stur||BOLD:ACE7506
Micropsectra junci|ATNA32|Elisabeth Stur||BOLD:ACE7506
Micropsectra junci|ZSM-DIP-33140-B10|Caroline Chimeno|BIN Taxonomy Match (Jan 2022)|BOLD ...
Chironomidae|CCDB22603-E07|Sofia Wiedenbrug|no morphological identification - introduction of int...
Chironomidae|CCDB22603-E11|Sofia Wiedenbrug|no morphological identification - introduction of int...
Chironomidae|CCDB22603-E08|Sofia Wiedenbrug|no morphological identification - introduction of int...
Chironomidae|CCDB22603-E12|Sofia Wiedenbrug|no morphological identification - introduction of int...
Chironomidae|CCDB22603-E10|Sofia Wiedenbrug|no morphological identification - introduction of int...
Chironomidae|CCDB22603-E06|Sofia Wiedenbrug|no morphological identification - introduction of int...
Micropsectra junci|CCDB24183-C03|Sofia Wiedenbrug|BIN-taxonomy|BOLD:AAA9963
Micropsectra junci|CCDB24183-B10|Sofia Wiedenbrug|BIN-taxonomy|BOLD:AAA9963
Micropsectra junci|CCDB21606-A07|Sofia Wiedenbrug||BOLD:AAA9963
Micropsectra junci|CCDB21606-G10|Sofia Wiedenbrug||BOLD:AAA9963
Micropsectra junci|CCDB21606-C02|Sofia Wiedenbrug||BOLD:AAA9963
Micropsectra junci|CCDB21606-H03|Sofia Wiedenbrug||BOLD:AAA9963
Micropsectra junci|CCDB21606-C01|Sofia Wiedenbrug||BOLD:AAA9963
Micropsectra junci|CCDB21606-B11|Sofia Wiedenbrug||BOLD:AAA9963
Micropsectra junci|ZMUO.024555|Lauri Paasivirta||BOLD:AAA9963
Micropsectra junci|CCDB24183-B08|Sofia Wiedenbrug|Morphology|BOLD:AAA9963
Micropsectra junci|ZMUO.024556|Lauri Paasivirta||BOLD:AAA9963
Micropsectra junci|CH-OSF17|Elisabeth Stur||BOLD:AAA9963
Micropsectra junci|CCDB24183-H06|Sofia Wiedenbrug|BIN-taxonomy|BOLD:AAA9963
Micropsectra junci|BIOUG05142-E05|Kate Perez|BIN Taxonomy Match|BOLD:AAA9963
Micropsectra junci|CH-OSF53|Elisabeth Stur||BOLD:AAA9963
Micropsectra junci|To157|Torbjorn Ekrem||BOLD:AAA9963
Micropsectra junci|To154|Torbjorn Ekrem||BOLD:AAA9963
Micropsectra junci|CCDB24183-B05|Sofia Wiedenbrug|Morphology|BOLD:AAA9963
Micropsectra junci|BIOUG04433-E06|Kate Perez|BIN Taxonomy Match|BOLD:AAA9963
Micropsectra junci|ATNA38|Elisabeth Stur||BOLD:AAA9963
Micropsectra junci|Finnmark349|Elisabeth Stur||BOLD:AAA9963
Micropsectra junci|SOE445|Torbjorn Ekrem||BOLD:AAA9963
Micropsectra junci|SOE127|Torbjorn Ekrem||BOLD:AAA9963
Micropsectra junci|SOE118|Torbjorn Ekrem||BOLD:AAA9963
Micropsectra junci|SOE121|Torbjorn Ekrem||BOLD:AAA9963
Micropsectra junci|EBAI-Ch012|Elisabeth Stur|morphology|BOLD:AAA9963
Micropsectra junci|Finnmark640|Elisabeth Stur||BOLD:AAA9963
Paratanytarsus|BIOUG55123-A05|Kate Perez|BIN Taxonomy Match (Oct 2020)|BOLD:AAD1485
Paratanytarsus|BIOUG55153-H06|Kate Perez|BIN Taxonomy Match (Oct 2020)|BOLD:AAD1485
Paratanytarsus|BIOUG36655-F07|Kate Perez|BIN Taxonomy Match (Jul 2018)|BOLD:AAD1485
Paratanytarsus grimmii|To18|Torbjorn Ekrem||BOLD:AAD1485
Paratanytarsus laccophilus|TRD-CH241|Elisabeth Stur||BOLD:AAC8842
Paratanytarsus laccophilus|ZMUO.025368|Lauri Paasivirta||BOLD:AAC8842
Paratanytarsus laccophilus|ZSM-DIP-33142-A10|Caroline Chimeno|BIN Taxonomy Match (Jan 2022)...
Paratanytarsus laccophilus|TRD-CH23|Elisabeth Stur||BOLD:AAC8842
Paratanytarsus laccophilus|CCDB21606-G04|Sofia Wiedenbrug||BOLD:AAC8842
Paratanytarsus laccophilus|ZSM-DIP-33172-E12|Caroline Chimeno|BIN Taxonomy Match (Jan 2022)...
Paratanytarsus laccophilus|ZMUO.025369|Lauri Paasivirta||BOLD:AAC8842
Paratanytarsus laccophilus|TRD-CH363|Elisabeth Stur|morphology|BOLD:AAC8842
Paratanytarsus laccophilus|TRD-CH339|Elisabeth Stur|morphology|BOLD:AAC8842
Paratanytarsus laccophilus|RIN_CH79|Mona Renate Saursaunet|BOLD Identification engine|BOLD:AAC...
[illegible]

Paratanytarsus laccophilus|TRD-CH363|Elisabeth Stur|morphology|BOLD:AAC8842
Paratanytarsus laccophilus|TRD-CH339|Elisabeth Stur|morphology|BOLD:AAC8842
Paratanytarsus laccophilus|RIN_CH79|Mona Renate Saursaunet|BOLD Identification engine|BOLD:AAC...
Paratanytarsus laccophilus|RIN_CH78|Mona Renate Saursaunet|BOLD Identification engine|BOLD:AAC...
Paratanytarsus laccophilus|NHRS-BYWS000001083|Yngve Brodin|morphology|BOLD:AAC8842
Paratanytarsus laccophilus|TRD-CH337|Elisabeth Stur|morphology|BOLD:AAC8842
Paratanytarsus laccophilus|TRD-CH314|Elisabeth Stur||BOLD:AAC8842
Paratanytarsus laccophilus|TRD-CH224|Elisabeth Stur||BOLD:AAC8842
Paratanytarsus laccophilus|TRD-CH311|Elisabeth Stur||BOLD:AAC8842
Paratanytarsus laccophilus|TRD-CH37|Elisabeth Stur||BOLD:AAC8842
Paratanytarsus laccophilus|TRD-CH303|Elisabeth Stur||BOLD:AAC8842
Paratanytarsus laccophilus|TRD-CH390|Elisabeth Stur|morphology|BOLD:AAC8842
Paratanytarsus laccophilus|RIN_CH81|Mona Renate Saursaunet|BOLD Identification engine|BOLD:AAC...
Paratanytarsus laccophilus|TRD-CH21|Elisabeth Stur||BOLD:AAC8842
Paratanytarsus laccophilus|Finnmark685|Elisabeth Stur||BOLD:AAC8842
Paratanytarsus laccophilus|Finnmark168|Elisabeth Stur||BOLD:AAC8842
Paratanytarsus laccophilus|CCDB21606-B07|Sofia Wiedenbrug||BOLD:AAC8842
Paratanytarsus laccophilus|CCDB21606-B10|Sofia Wiedenbrug||BOLD:AAC8842
Paratanytarsus laccophilus|EJV-20110031|Lauri Paasivirta||BOLD:AAC8842
Paratanytarsus laccophilus|EJV-20110034|Lauri Paasivirta||BOLD:AAC8842
Paratanytarsus laccophilus|EJV-20110033|Lauri Paasivirta||BOLD:AAC8842
Paratanytarsus laccophilus|EJV-20110032|Lauri Paasivirta||BOLD:AAC8842
Paratanytarsus laccophilus|EJV-20110030|Lauri Paasivirta||BOLD:AAC8842
Paratanytarsus abiskoensis|Finnmark112|Elisabeth Stur||BOLD:AAE3677
Paratanytarsus laetipes|ZSM-DIP-33170-D11|Caroline Chimeno|BIN Taxonomy Match (Jan 2022)|BO...
Paratanytarsus laetipes|ZSM-DIP-33149-E06|Caroline Chimeno|BIN Taxonomy Match (Jan 2022)|BO...
Paratanytarsus laetipes|BIOUG36677-E04|Kate Perez|BIN Taxonomy Match (May 2018)|BOLD:AAD...
Paratanytarsus laetipes|NHRS-BYWS000000414|Yngve Brodin|morphology|BOLD:ADJ5814
Paratanytarsus sp. 2ES|Finnmark625|Elisabeth Stur
Paratanytarsus laetipes|ZMUO.025215|Lauri Paasivirta||BOLD:ADA3838
Paratanytarsus laetipes|ZMUO.025214|Lauri Paasivirta||BOLD:ADT6819
Paratanytarsus sp. 2ES|Finnmark624|Elisabeth Stur||BOLD:AAV6211
Paratanytarsus hyperboreus|Finnmark704|Elisabeth Stur||BOLD:AAP3292
Paratanytarsus hyperboreus|Finnmark702|Elisabeth Stur||BOLD:AAP3292
Paratanytarsus hyperboreus|ZMUO.026253|Lauri Paasivirta||BOLD:AAP3292
Paratanytarsus hyperboreus|ZMUO.026254|Lauri Paasivirta||BOLD:AAP3292
Paratanytarsus hyperboreus|Finnmark565|Torbjorn Ekrem||BOLD:AAP3292
Paratanytarsus austriacus|TRD-CH42|Elisabeth Stur||BOLD:ACM5899
Paratanytarsus austriacus|ZMUO.024707|Lauri Paasivirta||BOLD:ACM5899
Paratanytarsus austriacus|ZMUO.024553|Lauri Paasivirta||BOLD:ACM5899
Paratanytarsus austriacus|ZMUO.024554|Lauri Paasivirta||BOLD:ACM5899
Paratanytarsus austriacus|ZMUO.024706|Lauri Paasivirta||BOLD:ACM5899
Paratanytarsus austriacus|BIOUG36655-A12|Kate Perez|BIN Taxonomy Match (Jul 2018)|BOLD:A...
Paratanytarsus austriacus|BIOUG15536-G05|Kate Perez|BIN Taxonomy Match|BOLD:ACM5899
Paratanytarsus austriacus|ZSM-DIP-33142-H07|Caroline Chimeno|BIN Taxonomy Match (Jan 2022)|...
Paratanytarsus austriacus|ATNA326|Elisabeth Stur||BOLD:AAA7953
Paratanytarsus austriacus|ATNA325|Elisabeth Stur||BOLD:AAA7953
Paratanytarsus austriacus|SOE02|Elisabeth Stur||BOLD:AAA7953
Paratanytarsus austriacus|SOE01|Torbjorn Ekrem||BOLD:AAA7953
Paratanytarsus hyperboreus|To45|Torbjorn Ekrem||BOLD:AAE7221
Paratanytarsus hyperboreus|ATNA26|Elisabeth Stur||BOLD:AAE7221
Paratanytarsus hyperboreus|Finnmark339|Elisabeth Stur||BOLD:AAE7221
Paratanytarsus hyperboreus|ATNA48|Elisabeth Stur||BOLD:AAE7221
Paratanytarsus hyperboreus|Finnmark338|Elisabeth Stur||BOLD:AAE7221
Paratanytarsus bituberculatus|ZSM-DIP-33145-A02|Caroline Chimeno|BIN Taxonomy Match (Jan 2022...
Paratanytarsus bituberculatus|BIOUG36854-F06|Kate Perez|BIN Taxonomy Match (Jul 2018)|BOLD...
Paratanytarsus bituberculatus|ZSM-DIP-33142-A11|Caroline Chimeno|BIN Taxonomy Match (Jan 2022...
Paratanytarsus bituberculatus|TRD-CH359|Elisabeth Stur|morphology|BOLD:AEE6413
Paratanytarsus tenuis|ZMUO.024649|Lauri Paasivirta||BOLD:ADA8414
Paratanytarsus tenuis|ZMUO.024822|Lauri Paasivirta||BOLD:ADA8414
Paratanytarsus tenuis|ZMUO.024821|Lauri Paasivirta||BOLD:ADA8414
Paratanytarsus tenuis|ZMUO.024648|Lauri Paasivirta||BOLD:ADA8414
Paratanytarsus tenuis|To63|Torbjorn Ekrem||BOLD:AAE3684
Paratanytarsus tenuis|To61|Torbjorn Ekrem||BOLD:AAE3684
Paratanytarsus|BIOUG36726-A06|Kate Perez|BIN Taxonomy Match (May 2018)|BOLD:AAE3684
Paratanytarsus|BIOUG36601-D08|Kate Perez|BIN Taxonomy Match (May 2018)|BOLD:AAE3684
Paratanytarsus tenuis|SOD07|Sondre Dahle|Morphology|BOLD:AAE3684
Paratanytarsus tenuis|SOD06|Sondre Dahle|Morphology|BOLD:AAE3684
Paratanytarsus tenuis|Finnmark419|Elisabeth Stur||BOLD:AAE3684
Paratanytarsus sp. 6TE|To363|Torbjorn Ekrem||BOLD:AAE3674
Paratanytarsus setosimanus|To360|Torbjorn Ekrem||BOLD:AAC4205
Paratanytarsus setosimanus|To362|Torbjorn Ekrem||BOLD:AAC4205
Paratanytarsus setosimanus|To359|Torbjorn Ekrem||BOLD:AAC4205
Paratanytarsus sp. 1ES|Finnmark404|Elisabeth Stur||BOLD:ADY7766
Paratanytarsus penicillatus|ZMUO.026173|Lauri Paasivirta||BOLD:ADF1666
Paratanytarsus penicillatus|ZMUO.026172|Lauri Paasivirta||BOLD:ADF1666
Paratanytarsus sp. 9TE|Finnmark738|Torbjorn Ekrem||BOLD:AAM5371
Paratanytarsus sp. 9TE|Finnmark111|Elisabeth Stur||BOLD:AAM5371
Paratanytarsus sp. 9TE|Finnmark238|Elisabeth Stur||BOLD:AAP6582
Paratanytarsus sp. 9TE|Finnmark237|Elisabeth Stur||BOLD:AAP6582
Paratanytarsus sp. 9TE|Finnmark501|Elisabeth Stur||BOLD:AAP6582
Paratanytarsus sp. 9TE|Finnmark627|Elisabeth Stur||BOLD:AAP6582
Paratanytarsus penicillatus|NO02|Elisabeth Stur||BOLD:AAF7209
Paratanytarsus penicillatus|SOD02|Sondre Dahle|Morphology|BOLD:AAF7209
Paratanytarsus penicillatus|SOE44|Elisabeth Stur||BOLD:AAF7209
Paratanytarsus penicillatus|NO03|Elisabeth Stur||BOLD:AAF7209
Paratanytarsus penicillatus|SOD09|Sondre Dahle|Morphology|BOLD:AAF7209
Paratanytarsus penicillatus|Finnmark82|Elisabeth Stur||BOLD:AAF7209
Paratanytarsus penicillatus|Finnmark455|Elisabeth Stur||BOLD:AAF7209
Paratanytarsus penicillatus|Finnmark447|Elisabeth Stur||BOLD:AAF7209
Paratanytarsus penicillatus|SOD08|Sondre Dahle|Morphology|BOLD:AAF7209
Paratanytarsus penicillatus|SOD01|Sondre Dahle|Morphology|BOLD:AAF7209
Paratanytarsus penicillatus|ZMUO.024307|Lauri Paasivirta||BOLD:AAF7209
Paratanytarsus penicillatus|SOD11|Sondre Dahle|Morphology|BOLD:AAF7209
Paratanytarsus penicillatus|Finnmark83|Elisabeth Stur||BOLD:AAF7209
Paratanytarsus penicillatus|Finnmark335|Elisabeth Stur||BOLD:AAF7209
Chironomidae|ZSM-DIP-33147-E10|Caroline Chimeno|BIN Taxonomy Match (Jan 2022)|BOLD:ACY6070
Paratanytarsus dissimilis|BIOUG42746-A12|Kate Perez|BIN Taxonomy Match below Phylum (Jun 2019...
Paratanytarsus dissimilis|ZSM-DIP-33149-F01|Caroline Chimeno|BIN Taxonomy Match (Jan 2022)|...
Paratanytarsus dissimilis|ZSM-DIP-33129-B05|Caroline Chimeno|BIN Taxonomy Match (Jan 2022)|...
Paratanytarsus dissimilis|ZSM-DIP-33139-A08|Caroline Chimeno|BIN Taxonomy Match (Jan 2022)|...
Paratanytarsus dissimilis|ZSM-DIP-33139-A07|Caroline Chimeno|BIN Taxonomy Match (Jan 2022)|...
Paratanytarsus dissimilis|ZSM-DIP-33137-H03|Caroline Chimeno|BIN Taxonomy Match (Jan 2022)|...

Paratanytarsus dissimilis|ZSM-DIP-33139-A08|Caroline Chimeno|BIN Taxonomy Match (Jan 2022)|...
Paratanytarsus dissimilis|ZSM-DIP-33139-A07|Caroline Chimeno|BIN Taxonomy Match (Jan 2022)|...
Paratanytarsus dissimilis|ZSM-DIP-33137-H03|Caroline Chimeno|BIN Taxonomy Match (Jan 2022)|...
Paratanytarsus dissimilis|ZSM-DIP-33134-E08|Caroline Chimeno|BIN Taxonomy Match (Jan 2022)|...
Paratanytarsus dissimilis|ZSM-DIP-33134-E09|Caroline Chimeno|BIN Taxonomy Match (Jan 2022)|...
Paratanytarsus dissimilis|NHRS-BYWS000000266|Yngve Brodin|morphology|BOLD:AAW4635
Tanytarsus pallidicornis|PP0261|Lauri Paasivirta||BOLD:AAW4635
Paratanytarsus dissimilis|ZMUO.024725|Lauri Paasivirta||BOLD:AAW4635
Paratanytarsus dissimilis|ZMUO.024724|Lauri Paasivirta||BOLD:AAW4635
Chironomidae|ZSM-DIP-33134-E11|Caroline Chimeno|BIN Taxonomy Match (Jan 2022)|BOLD:AAW4635
Paratanytarsus dissimilis|EJV-20110060|Lauri Paasivirta||BOLD:AAW4635
Paratanytarsus dissimilis|EJV-20110058|Lauri Paasivirta||BOLD:AAW4635
Paratanytarsus dissimilis|EJV-20110059|Lauri Paasivirta||BOLD:AAW4635
Paratanytarsus dissimilis|EJV-20110061|Lauri Paasivirta||BOLD:AAW4635
Paratanytarsus dissimilis|EJV-20110057|Lauri Paasivirta||BOLD:AAW4635
Paratanytarsus inopertus|NHRS-BYWS000000550|Yngve Brodin|morphology|BOLD:AAU1200
Paratanytarsus inopertus|NHRS-BYWS000001807|Yngve Brodin||BOLD:AAU1200
Paratanytarsus inopertus|NHRS-BYWS000000110|Yngve Brodin|morphology|BOLD:AAU1200
Paratanytarsus inopertus|NHRS-BYWS000000279|Yngve Brodin|morphology|BOLD:AAU1200
Paratanytarsus inopertus|NHRS-BYWS000001065|Yngve Brodin|morphology|BOLD:AAU1200
Paratanytarsus inopertus|ZMUO.024816|Lauri Paasivirta||BOLD:AAU1200
Paratanytarsus inopertus|ZMUO.024815|Lauri Paasivirta||BOLD:AAU1200
Chironominae|BIOUG36735-G02|Kate Perez|BIN Taxonomy Match (May 2018)|BOLD:AAU1200
Chironominae|BIOUG36992-H06|Kate Perez|BIN Taxonomy Match (Jul 2018)|BOLD:AAU1200
Paratanytarsus inopertus|NHRS-BYWS000001754|Yngve Brodin||BOLD:AAU1200
Paratanytarsus inopertus|NHRS-BYWS000001805|Yngve Brodin||BOLD:AAU1200
Paratanytarsus inopertus|NHRS-BYWS000001806|Yngve Brodin||BOLD:AAU1200
Paratanytarsus inopertus|NHRS-BYWS000001820|Yngve Brodin||BOLD:AAU1200
Paratanytarsus inopertus|NHRS-BYWS000001824|Yngve Brodin||BOLD:AAU1200
Paratanytarsus inopertus|NHRS-BYWS000000420|Yngve Brodin|morphology|BOLD:AAU1200
Paratanytarsus inopertus|NHRS-BYWS000000460|Yngve Brodin|morphology|BOLD:AAU1200
Paratanytarsus inopertus|NHRS-BYWS000000178|Yngve Brodin|morphology|BOLD:AAU1200
Paratanytarsus inopertus|NHRS-BYWS000000195|Yngve Brodin|morphology|BOLD:AAU1200
Paratanytarsus inopertus|NHRS-BYWS000000196|Yngve Brodin|morphology|BOLD:AAU1200
Paratanytarsus inopertus|NHRS-BYWS000000206|Yngve Brodin|morphology|BOLD:AAU1200
Paratanytarsus inopertus|NHRS-BYWS000000228|Yngve Brodin|morphology|BOLD:AAU1200
Paratanytarsus inopertus|NHRS-BYWS000000300|Yngve Brodin|morphology|BOLD:AAU1200
Paratanytarsus inopertus|NHRS-BYWS000000383|Yngve Brodin|morphology|BOLD:AAU1200
Paratanytarsus inopertus|NHRS-BYWS000001046|Yngve Brodin|morphology|BOLD:AAU1200
Paratanytarsus inopertus|NHRS-BYWS000001752|Yngve Brodin||BOLD:AAU1200
Chironomus plumosus|EJV-20110249|Lauri Paasivirta||BOLD:AAU1200
Polypedilum scalaenum|ZSM-DIP-33131-E02|Caroline Chimeno|BIN Taxonomy Match (Jan 2022)|BOLD...
Chironomidae|BIOUG36677-D07|Kate Perez|BIN Taxonomy Match (May 2018)|BOLD:ACP4052
Polypedilum scalaenum|NHRS-BYWS000001118|Yngve Brodin|morphology|BOLD:ADK1584
Polypedilum scalaenum|NHRS-BYWS000001119|Yngve Brodin|morphology|BOLD:ADK1584
Phaenopsectra flavipes|ES19|Elisabeth Stur||BOLD:AAI4472
Phaenopsectra flavipes|ES20|Elisabeth Stur||BOLD:AAI4472
Phaenopsectra flavipes|ZSM-DIP-33129-A01|Caroline Chimeno|BIN Taxonomy Match (Jan 2022)|BOL...
Phaenopsectra flavipes|ZMUO.024236|Lauri Paasivirta||BOLD:AAU2481
Phaenopsectra flavipes|ZSM-DIP-33129-B01|Caroline Chimeno|BIN Taxonomy Match (Jan 2022)|BOL...
Phaenopsectra flavipes|TRD-CH172|Elisabeth Stur||BOLD:AAU2481
Phaenopsectra flavipes|TRD-CH319|Elisabeth Stur||BOLD:AAU2481
Phaenopsectra flavipes|TRD-CH318|Elisabeth Stur||BOLD:AAU2481
Phaenopsectra flavipes|TRD-CH136|Elisabeth Stur|morphology|BOLD:AAU2481
Phaenopsectra flavipes|ZSM-DIP-33145-F09|Caroline Chimeno|BIN Taxonomy Match (Jan 2022)|BOL...
Phaenopsectra flavipes|TRD-CH346|Elisabeth Stur|morphology|BOLD:AAU2481
Phaenopsectra flavipes|NHRS-BYWS000001843|Yngve Brodin||BOLD:AAU2481
Phaenopsectra flavipes|NHRS-BYWS000000427|Yngve Brodin|morphology|BOLD:AAU2481
Phaenopsectra flavipes|ZMUO.024237|Lauri Paasivirta||BOLD:AAU2481
Phaenopsectra flavipes|ZSM-DIP-33129-E05|Caroline Chimeno|BIN Taxonomy Match (Jan 2022)|BOL...
Phaenopsectra flavipes|NHRS-BYWS000001832|Yngve Brodin||BOLD:AAU2481
Phaenopsectra flavipes|EJV-20110081|Lauri Paasivirta||BOLD:AAU2481
Phaenopsectra flavipes|EJV-20110078|Lauri Paasivirta||BOLD:AAU2481
Phaenopsectra flavipes|ZSM-DIP-33136-G01|Caroline Chimeno|BIN Taxonomy Match (Jan 2022)|BOL...
Phaenopsectra flavipes|TRD-CH186|Elisabeth Stur||BOLD:AAU2481
Phaenopsectra flavipes|EJV-20110082|Lauri Paasivirta||BOLD:AAU2481
Phaenopsectra flavipes|EJV-20110083|Lauri Paasivirta||BOLD:AAU2481
Phaenopsectra flavipes|NHRS-BYWS000000125|Yngve Brodin|morphology|BOLD:AAU2481
Phaenopsectra flavipes|NHRS-BYWS000001136|Yngve Brodin|morphology|BOLD:AAU2481
Phaenopsectra flavipes|TRD-CH361|Elisabeth Stur|morphology|BOLD:AAU2481
Phaenopsectra flavipes|EJV-20110079|Lauri Paasivirta||BOLD:AAU2481
Phaenopsectra flavipes|EJV-20110080|Lauri Paasivirta||BOLD:AAU2481
Polypedilum quadriguttatum|NHRS-BYWS000000919|Yngve Brodin|morphology|BOLD:ACU9154
Polypedilum quadriguttatum|NHRS-BYWS000000918|Yngve Brodin|morphology|BOLD:ACU9154
Polypedilum simulans|Finnmark734|Elisabeth Stur||BOLD:AAV2668
Polypedilum quadriguttatum|ZMUO.025246|Lauri Paasivirta||BOLD:AAE4099
Polypedilum quadriguttatum|ZMUO.025245|Lauri Paasivirta||BOLD:AAE4099
Polypedilum|ZSM-DIP-33137-H04|Caroline Chimeno|BIN Taxonomy Match (Jan 2022)|BOLD:AAE4099
Polypedilum quadriguttatum|NHRS-BYWS000001799|Yngve Brodin||BOLD:AAE4099
Polypedilum scalaenum|ZMUO.025314|Lauri Paasivirta||BOLD:ADF1782
Polypedilum scalaenum|ZMUO.025313|Lauri Paasivirta||BOLD:ADF1782
Polypedilum asoprimum|song189|xxx Chao|Morphology|BOLD:AAY3408
Polypedilum scalaenum|EJV-20110211|Lauri Paasivirta||BOLD:AAY3408
Polypedilum tritum|ZMUO.024812|Lauri Paasivirta||BOLD:ADJ5715
Polypedilum tritum|ZMUO.024811|Lauri Paasivirta||BOLD:ADJ5715
Polypedilum tritum|ZMUO.024258|Lauri Paasivirta||BOLD:ADJ5715
Polypedilum tritum|BIOUG36658-H06|Kate Perez|BIN Taxonomy Match (Jul 2018)|BOLD:ADJ5715
Polypedilum uncinatum|NHRS-BYWS000000556|Yngve Brodin|morphology|BOLD:ADJ5716
Polypedilum uncinatum|NHRS-BYWS000000555|Yngve Brodin|morphology|BOLD:ADJ5716
Polypedilum uncinatum|NHRS-BYWS000000554|Yngve Brodin|morphology|BOLD:ADJ5716
Polypedilum uncinatum|NHRS-BYWS000000553|Yngve Brodin|morphology|BOLD:ADJ5716
Polypedilum uncinatum|NO37|Torbjorn Ekrem||BOLD:AAF0341
Polypedilum uncinatum|NO35|Torbjorn Ekrem||BOLD:AAF0341
Polypedilum uncinatum|NO34|Torbjorn Ekrem||BOLD:AAF0341
Polypedilum uncinatum|NO33|Torbjorn Ekrem||BOLD:AAF0341
Polypedilum tetracrenatum|ZMUO.025137|Lauri Paasivirta||BOLD:ADA7305
Polypedilum tetracrenatum|ZMUO.025136|Lauri Paasivirta||BOLD:ADA7305
Polypedilum|ZSM-DIP-33134-E07|Caroline Chimeno|BIN Taxonomy Match (Jan 2022)|BOLD:ACV3478
Polypedilum tridens|CH141|||BOLD:ACV3478
Polypedilum bicrenatum|ZMUO.025352|Lauri Paasivirta||BOLD:ACV3478
Polypedilum bicrenatum|NHRS-BYWS000000993|Yngve Brodin|morphology|BOLD:ACV3478
Polypedilum bicrenatum|NHRS-BYWS000001120|Yngve Brodin|morphology|BOLD:ACV3478
Polypedilum tridens|song193|xxx Chao|Morphology|BOLD:ACV3478

Polypedilum bicrenatum|NHRS-BYWS000000993|Yngve Brodin|morphology|BOLD:ACV3478
Polypedilum bicrenatum|NHRS-BYWS000001120|Yngve Brodin|morphology|BOLD:ACV3478
Polypedilum tridens|song193|xxx Chao|Morphology|BOLD:ACV3478
Polypedilum tridens|song192|xxx Chao|Morphology|BOLD:ACV3478
Polypedilum bicrenatum|ZMUO.025351|Lauri Paasivirta||BOLD:ACV3478
Polypedilum|BIOUG36779-A10|Kate Perez|BIN Taxonomy Match (May 2018)|BOLD:ACV3478
Polypedilum bicrenatum|NHRS-BYWS000000506|Yngve Brodin|morphology|BOLD:ACV3478
Polypedilum bicrenatum|NHRS-BYWS000000291|Yngve Brodin|morphology|BOLD:ACV3478
Polypedilum bicrenatum|NHRS-BYWS000000442|Yngve Brodin|morphology|BOLD:ACV3478
Polypedilum bicrenatum|NHRS-BYWS000000404|Yngve Brodin|morphology|BOLD:ACV3478
Polypedilum trigonus|ZMUO.025346|Lauri Paasivirta||BOLD:AAV3587
Polypedilum trigonus|ZMUO.025345|Lauri Paasivirta||BOLD:AAV3587
Polypedilum trigonus|NHRS-BYWS000000557|Yngve Brodin|morphology|BOLD:AAV3587
Polypedilum trigonus|NHRS-BYWS000000558|Yngve Brodin|morphology|BOLD:AAV3587
Polypedilum trigonus|NHRS-BYWS000000459|Yngve Brodin|morphology|BOLD:AAV3587
Polypedilum sp. 3TE|CH-OSF171|Torbjorn Ekrem||BOLD:AAV3587
Polypedilum sordens|BIOUG36931-G07|Kate Perez|BIN Taxonomy Match (May 2018)|BOLD:ADF3485
Polypedilum sordens|NHRS-BYWS000001073|Yngve Brodin|morphology|BOLD:ACY3855
Polypedilum sordens|NHRS-BYWS000000325|Yngve Brodin|morphology|BOLD:ACY3855
Polypedilum sordens|CH153|||BOLD:ACY3855
Polypedilum sordens|NHRS-BYWS000000324|Yngve Brodin|morphology|BOLD:ACY3855
Polypedilum sordens|NHRS-BYWS000001058|Yngve Brodin|morphology|BOLD:ACY3854
Polypedilum sordens|ZMUO.024938|Lauri Paasivirta||BOLD:ACY3854
Polypedilum sordens|ZMUO.024937|Lauri Paasivirta||BOLD:ACY3854
Polypedilum sordens|TRD-CH434|Elisabeth Stur|morphology|BOLD:ACY3854
Polypedilum sordens|NHRS-BYWS000001049|Yngve Brodin|morphology|BOLD:ACY3854
Polypedilum sordens|song190|xxx Chao|Morphology|BOLD:ACY3854
Polypedilum sordens|CHAO48|xxx Chao|Morphology|BOLD:ACY3854
Polypedilum sordens|CHAO47|xxx Chao|Morphology|BOLD:ACY3854
Paratendipes albimanus|BIOUG55145-G09|Stefan Schmidt|BIN Taxonomy Match (Sep 2020)|BOLD:...
Paratendipes albimanus|ZSM-DIP-33133-B04|Caroline Chimeno|BIN Taxonomy Match (Jan 2022)|BOL...
Paratendipes albimanus|NHRS-BYWS000000351|Yngve Brodin|morphology|BOLD:AAO1037
Paratendipes albimanus|NHRS-BYWS000000503|Yngve Brodin|morphology|BOLD:AAO1037
Paratendipes albimanus|ZSM-DIP-33131-D01|Caroline Chimeno|BIN Taxonomy Match (Jan 2022)|BOL...
Paratendipes albimanus|NHRS-BYWS000000441|Yngve Brodin|morphology|BOLD:AAO1037
Paratendipes albimanus|BIOUG55115-E06|Stefan Schmidt|BIN Taxonomy Match (Sep 2020)|BOLD:...
Paratendipes albimanus|CH-eik22|Elisabeth Stur||BOLD:AAO1037
Paratendipes albimanus|ZSM-DIP-33145-G07|Caroline Chimeno|BIN Taxonomy Match (Jan 2022)|BOL...
Paratendipes albimanus|BIOUG55119-D03|Stefan Schmidt|BIN Taxonomy Match (Sep 2020)|BOLD:...
Paratendipes albimanus|ZSM-DIP-33146-E10|Caroline Chimeno|BIN Taxonomy Match (Jan 2022)|BOL...
Paratendipes albimanus|CCDB22604-G04|Sofia Wiedenbrug||BOLD:AAO1037
Paratendipes albimanus|BIOUG42670-B07|Kate Perez|BIN Taxonomy Match below Phylum (Jun 2019)...
Paratendipes albimanus|BIOUG42704-G01|Kate Perez|BIN Taxonomy Match below Phylum (Jun 2019)...
Paratendipes albimanus|BIOUG42687-D09|Kate Perez|BIN Taxonomy Match below Phylum (Jun 2019)...
Paratendipes albimanus|BIOUG55115-D11|Stefan Schmidt|BIN Taxonomy Match (Sep 2020)|BOLD:...
Paratendipes albimanus|ZSM-DIP-33144-F10|Caroline Chimeno|BIN Taxonomy Match (Jan 2022)|BOL...
Paratendipes albimanus|BIOUG17096-B10|Kate Perez|BIN Taxonomy Match|BOLD:AAO1037
Paratendipes albimanus|ZSM-DIP-33147-F12|Caroline Chimeno|BIN Taxonomy Match (Jan 2022)|BOL...
Paratendipes albimanus|BIOUG42656-B02|Kate Perez|BIN Taxonomy Match below Phylum (Jun 2019)...
Paratendipes albimanus|ZSM-DIP-33144-F11|Caroline Chimeno|BIN Taxonomy Match (Jan 2022)|BOL...
Paratendipes albimanus|CCDB24228-G10|Sofia Wiedenbrug|BIN Taxonomy Match|BOLD:AAO1037
Paratendipes albimanus|BIOUG42752-H01|Kate Perez|BIN Taxonomy Match below Phylum (Jun 2019)...
Paratendipes albimanus|BIOUG55115-A04|Stefan Schmidt|BIN Taxonomy Match (Sep 2020)|BOLD:...
Paratendipes albimanus|BIOUG55127-E12|Stefan Schmidt|BIN Taxonomy Match (Sep 2020)|BOLD:...
Paratendipes albimanus|NHRS-BYWS000000398|Yngve Brodin|morphology|BOLD:AAO1037
Paratendipes albimanus|NHRS-BYWS000000505|Yngve Brodin|morphology|BOLD:AAO1037
Paratendipes albimanus|BIOUG55117-D09|Stefan Schmidt|BIN Taxonomy Match (Sep 2020)|BOLD:...
Paratendipes albimanus|BIOUG55126-E07|Stefan Schmidt|BIN Taxonomy Match (Sep 2020)|BOLD:...
Paratendipes albimanus|BIOUG55118-A08|Stefan Schmidt|BIN Taxonomy Match (Sep 2020)|BOLD:...
Paratendipes albimanus|BIOUG55115-E02|Stefan Schmidt|BIN Taxonomy Match (Sep 2020)|BOLD:...
Paratendipes albimanus|BIOUG55112-E02|Stefan Schmidt|BIN Taxonomy Match (Sep 2020)|BOLD:...
Paratendipes albimanus|BIOUG55119-A06|Stefan Schmidt|BIN Taxonomy Match (Sep 2020)|BOLD:...
Paratendipes albimanus|BIOUG55151-F09|Stefan Schmidt|BIN Taxonomy Match (Sep 2020)|BOLD:...
Paratendipes albimanus|BIOUG55144-E06|Stefan Schmidt|BIN Taxonomy Match (Sep 2020)|BOLD:...
Paratendipes albimanus|BIOUG55153-E10|Stefan Schmidt|BIN Taxonomy Match (Sep 2020)|BOLD:...
Paratendipes albimanus|BIOUG42737-B07|Kate Perez|BIN Taxonomy Match below Phylum (Jun 2019)...
Paratendipes albimanus|ZSM-DIP-33142-A08|Caroline Chimeno|BIN Taxonomy Match (Jan 2022)|BOL...
Paratendipes albimanus|BIOUG42781-G09|Kate Perez|BIN Taxonomy Match below Phylum (Jun 2019)...
Paratendipes albimanus|ZSM-DIP-33144-H07|Caroline Chimeno|BIN Taxonomy Match (Jan 2022)|BOL...
Paratendipes albimanus|ZSM-DIP-33146-A01|Caroline Chimeno|BIN Taxonomy Match (Jan 2022)|BOL...
Paratendipes albimanus|ZSM-DIP-33147-E08|Caroline Chimeno|BIN Taxonomy Match (Jan 2022)|BOL...
Paratendipes albimanus|ZSM-DIP-33147-E09|Caroline Chimeno|BIN Taxonomy Match (Jan 2022)|BOL...
Paratendipes albimanus|ZSM-DIP-33131-H02|Caroline Chimeno|BIN Taxonomy Match (Jan 2022)|BOL...
Paratendipes albimanus|ZSM-DIP-33170-B05|Caroline Chimeno|BIN Taxonomy Match (Jan 2022)|BOL...
Paratendipes albimanus|BIOUG42742-F05|Kate Perez|BIN Taxonomy Match below Phylum (Jun 2019)...
Paratendipes albimanus|ZSM-DIP-33170-D09|Caroline Chimeno|BIN Taxonomy Match (Jan 2022)|BOL...
Paratendipes albimanus|ZSM-DIP-33146-D06|Caroline Chimeno|BIN Taxonomy Match (Jan 2022)|BOL...
Paratendipes albimanus|ZSM-DIP-33134-G12|Caroline Chimeno|BIN Taxonomy Match (Jan 2022)|BOL...
Paratendipes albimanus|BC-ZSM-DIP-22491-H05|Dieter Doczkal||BOLD:AAO1037
Paratendipes albimanus|BIOUG17305-H05|Kate Perez|BIN Taxonomy Match|BOLD:AAO1037
Paratendipes albimanus|BIOUG17305-C08|Kate Perez|BIN Taxonomy Match|BOLD:AAO1037
Paratendipes albimanus|ZSM-DIP-33143-A09|Caroline Chimeno|BIN Taxonomy Match (Jan 2022)|BOL...
Paratendipes albimanus|BIOUG17095-E01|Kate Perez|BIN Taxonomy Match|BOLD:AAO1037
Paratendipes albimanus|BIOUG42749-A09|Kate Perez|BIN Taxonomy Match below Phylum (Jun 2019)...
Paratendipes albimanus|ZSM-DIP-33144-E11|Caroline Chimeno|BIN Taxonomy Match (Jan 2022)|BOL...
Paratendipes albimanus|BIOUG17303-G01|Kate Perez|BIN Taxonomy Match|BOLD:AAO1037
Paratendipes albimanus|BIOUG42739-H10|Kate Perez|BIN Taxonomy Match below Phylum (Jun 2019)...
Paratendipes albimanus|ZSM-DIP-33145-G09|Caroline Chimeno|BIN Taxonomy Match (Jan 2022)|BOL...
Paratendipes albimanus|BIOUG16647-C07|Kate Perez|BIN Taxonomy Match|BOLD:AAO1037
Paratendipes albimanus|BIOUG16199-F08|Kate Perez|BIN Taxonomy Match|BOLD:AAO1037
Paratendipes albimanus|TRD-CH110|Elisabeth Stur||BOLD:AAO1037
Paratendipes albimanus|BIOUG16296-B11|Kate Perez|BIN Taxonomy Match|BOLD:AAO1037
Paratendipes albimanus|BIOUG16259-G04|Kate Perez|BIN Taxonomy Match|BOLD:AAO1037
Paratendipes albimanus|BIOUG16632-G04|Kate Perez|BIN Taxonomy Match|BOLD:AAO1037
Paratendipes albimanus|ZSM-DIP-33135-G05|Caroline Chimeno|BIN Taxonomy Match (Jan 2022)|BOL...
Paratendipes albimanus|BIOUG15967-B05|Kate Perez|BIN Taxonomy Match|BOLD:AAO1037
Paratendipes albimanus|BIOUG16299-F03|Kate Perez|BIN Taxonomy Match|BOLD:AAO1037
Paratendipes albimanus|BIOUG16257-F12|Kate Perez|BIN Taxonomy Match|BOLD:AAO1037
Paratendipes albimanus|BIOUG15954-E04|Kate Perez|BIN Taxonomy Match|BOLD:AAO1037
Paratendipes albimanus|BIOUG15950-F01|Kate Perez|BIN Taxonomy Match|BOLD:AAO1037
Paratendipes albimanus|BIOUG16261-D04|Kate Perez|BIN Taxonomy Match|BOLD:AAO1037
Paratendipes albimanus|BIOUG15953-C05|Kate Perez|BIN Taxonomy Match|BOLD:AAO1037
Paratendipes albimanus|BIOUG15952-F06|Kate Perez|BIN Taxonomy Match|BOLD:AAO1037

Paratendipes albimanus|BIOUG15950-F01|Kate Perez|BIN Taxonomy Match|BOLD:AAO1037
Paratendipes albimanus|BIOUG16261-D04|Kate Perez|BIN Taxonomy Match|BOLD:AAO1037
Paratendipes albimanus|BIOUG15953-C05|Kate Perez|BIN Taxonomy Match|BOLD:AAO1037
Paratendipes albimanus|BIOUG15952-F06|Kate Perez|BIN Taxonomy Match|BOLD:AAO1037
Paratendipes albimanus|BIOUG44442-C01|Kate Perez|BIN Taxonomy Match below Phylum (Mar 2019)...
Paratendipes albimanus|ZSM-DIP-33146-A02|Caroline Chimeno|BIN Taxonomy Match (Jan 2022)|BOL...
Paratendipes albimanus|BIOUG15964-A02|Kate Perez|BIN Taxonomy Match|BOLD:AAO1037
Paratendipes albimanus|BIOUG16257-G11|Kate Perez|BIN Taxonomy Match|BOLD:AAO1037
Paratendipes albimanus|NHRS-BYWS000000254|Yngve Brodin|morphology|BOLD:AAO1037
Paratendipes albimanus|BIOUG16198-E05|Kate Perez|BIN Taxonomy Match|BOLD:AAO1037
Paratendipes albimanus|BIOUG15950-G02|Kate Perez|BIN Taxonomy Match|BOLD:AAO1037
Paratendipes albimanus|BIOUG15947-D10|Kate Perez|BIN Taxonomy Match|BOLD:AAO1037
Paratendipes albimanus|BIOUG15951-F06|Kate Perez|BIN Taxonomy Match|BOLD:AAO1037
Paratendipes albimanus|BIOUG15963-E11|Kate Perez|BIN Taxonomy Match|BOLD:AAO1037
Paratendipes albimanus|BIOUG15953-E04|Kate Perez|BIN Taxonomy Match|BOLD:AAO1037
Paratendipes albimanus|BIOUG15950-D05|Kate Perez|BIN Taxonomy Match|BOLD:AAO1037
Paratendipes albimanus|BIOUG15951-B08|Kate Perez|BIN Taxonomy Match|BOLD:AAO1037
Paratendipes albimanus|BIOUG15951-E08|Kate Perez|BIN Taxonomy Match|BOLD:AAO1037
Paratendipes albimanus|BIOUG15967-B10|Kate Perez|BIN Taxonomy Match|BOLD:AAO1037
Paratendipes albimanus|BIOUG16258-H06|Kate Perez|BIN Taxonomy Match|BOLD:AAO1037
Paratendipes albimanus|BIOUG16299-G05|Kate Perez|BIN Taxonomy Match|BOLD:AAO1037
Paratendipes albimanus|EJV-20110099|Lauri Paasivirta||BOLD:AAO1037
Paratendipes albimanus|CH-eik128|Elisabeth Stur||BOLD:AAO1037
Paratendipes albimanus|TRD-CH320|Elisabeth Stur||BOLD:AAO1037
Paratendipes albimanus|ZMUO.025201|Lauri Paasivirta||BOLD:AAO1037
Paratendipes albimanus|ZMUO.025200|Lauri Paasivirta||BOLD:AAO1037
Paratendipes albimanus|BIOUG16631-G07|Kate Perez|BIN Taxonomy Match|BOLD:AAO1037
Paratendipes albimanus|BIOUG15905-B09|Kate Perez|BIN Taxonomy Match|BOLD:AAO1037
Paratendipes albimanus|EJV-20110097|Lauri Paasivirta||BOLD:AAO1037
Paratendipes albimanus|EJV-20110100|Lauri Paasivirta||BOLD:AAO1037
Paratendipes albimanus|EJV-20110101|Lauri Paasivirta||BOLD:AAO1037
Paratendipes albimanus|ZSM-DIP-33172-F02|Caroline Chimeno|BIN Taxonomy Match (Jan 2022)|BOL...
Paratendipes albimanus|ZSM-DIP-33142-A07|Caroline Chimeno|BIN Taxonomy Match (Jan 2022)|BOL...
Paratendipes albimanus|ZSM-DIP-33144-C12|Caroline Chimeno|BIN Taxonomy Match (Jan 2022)|BOL...
Paratendipes albimanus|ZSM-DIP-33137-G04|Caroline Chimeno|BIN Taxonomy Match (Jan 2022)|BOL...
Paratendipes albimanus|ZSM-DIP-33149-E01|Caroline Chimeno|BIN Taxonomy Match (Jan 2022)|BOL...
Paratendipes albimanus|ZSM-DIP-33149-G11|Caroline Chimeno|BIN Taxonomy Match (Jan 2022)|BOL...
Paratendipes albimanus|ZSM-DIP-33172-F01|Caroline Chimeno|BIN Taxonomy Match (Jan 2022)|BOL...
Paratendipes albimanus|EJV-20110095|Lauri Paasivirta||BOLD:AAO1037
Paratendipes albimanus|EJV-20110094|Lauri Paasivirta||BOLD:AAO1037
Paratendipes albimanus|EJV-20110096|Lauri Paasivirta||BOLD:AAO1037
Paratendipes albimanus|BIOUG16257-D12|Kate Perez|BIN Taxonomy Match|BOLD:AAO1037
Paratendipes albimanus|BIOUG16261-H04|Kate Perez|BIN Taxonomy Match|BOLD:AAO1037
Paratendipes albimanus|BIOUG16198-F08|Kate Perez|BIN Taxonomy Match|BOLD:AAO1037
Paratendipes albimanus|BIOUG16198-D06|Kate Perez|BIN Taxonomy Match|BOLD:AAO1037
Paratendipes albimanus|BIOUG16198-A02|Kate Perez|BIN Taxonomy Match|BOLD:AAO1037
Paratendipes albimanus|BIOUG16330-A07|Kate Perez|BIN Taxonomy Match|BOLD:AAO1037
Paratendipes albimanus|BIOUG15963-C08|Kate Perez|BIN Taxonomy Match|BOLD:AAO1037
Paratendipes albimanus|BIOUG15954-H08|Kate Perez|BIN Taxonomy Match|BOLD:AAO1037
Paratendipes albimanus|BIOUG15948-H08|Kate Perez|BIN Taxonomy Match|BOLD:AAO1037
Paratendipes albimanus|BIOUG15948-G12|Kate Perez|BIN Taxonomy Match|BOLD:AAO1037
Paratendipes albimanus|BIOUG15948-E07|Kate Perez|BIN Taxonomy Match|BOLD:AAO1037
Paratendipes albimanus|BIOUG15948-B03|Kate Perez|BIN Taxonomy Match|BOLD:AAO1037
Paratendipes albimanus|BIOUG15947-B09|Kate Perez|BIN Taxonomy Match|BOLD:AAO1037
Paratendipes albimanus|BIOUG15905-F03|Kate Perez|BIN Taxonomy Match|BOLD:AAO1037
Paratendipes albimanus|BIOUG15965-E10|Kate Perez|BIN Taxonomy Match|BOLD:AAO1037
Paratendipes albimanus|BIOUG16198-G04|Kate Perez|BIN Taxonomy Match|BOLD:AAO1037
Paratendipes albimanus|BIOUG15964-D01|Kate Perez|BIN Taxonomy Match|BOLD:AAO1037
Paratendipes albimanus|BIOUG15967-A05|Kate Perez|BIN Taxonomy Match|BOLD:AAO1037
Paratendipes albimanus|BIOUG15966-D06|Kate Perez|BIN Taxonomy Match|BOLD:AAO1037
Paratendipes albimanus|BIOUG15964-G01|Kate Perez|BIN Taxonomy Match|BOLD:AAO1037
Paratendipes albimanus|BIOUG15964-H07|Kate Perez|BIN Taxonomy Match|BOLD:AAO1037
Paratendipes albimanus|BIOUG15964-H08|Kate Perez|BIN Taxonomy Match|BOLD:AAO1037
Paratendipes albimanus|BIOUG15965-H04|Kate Perez|BIN Taxonomy Match|BOLD:AAO1037
Paratendipes albimanus|BIOUG16256-E06|Kate Perez|BIN Taxonomy Match|BOLD:AAO1037
Paratendipes albimanus|BIOUG16331-E11|Kate Perez|BIN Taxonomy Match|BOLD:AAO1037
Paratendipes albimanus|BIOUG15905-A06|Kate Perez|BIN Taxonomy Match|BOLD:AAO1037
Paratendipes albimanus|BIOUG16530-G01|Kate Perez|BIN Taxonomy Match|BOLD:AAO1037
Paratendipes albimanus|BIOUG15950-F02|Kate Perez|BIN Taxonomy Match|BOLD:AAO1037
Paratendipes albimanus|BIOUG15953-B02|Kate Perez|BIN Taxonomy Match|BOLD:AAO1037
Paratendipes albimanus|BIOUG15952-D12|Kate Perez|BIN Taxonomy Match|BOLD:AAO1037
Paratendipes albimanus|BIOUG15950-E12|Kate Perez|BIN Taxonomy Match|BOLD:AAO1037
Paratendipes albimanus|BIOUG15954-B10|Kate Perez|BIN Taxonomy Match|BOLD:AAO1037
Paratendipes albimanus|BIOUG15905-G11|Kate Perez|BIN Taxonomy Match|BOLD:AAO1037
Paratendipes albimanus|BIOUG15963-H10|Kate Perez|BIN Taxonomy Match|BOLD:AAO1037
Paratendipes albimanus|BIOUG16297-G11|Kate Perez|BIN Taxonomy Match|BOLD:AAO1037
Paratendipes albimanus|BIOUG15905-D07|Kate Perez|BIN Taxonomy Match|BOLD:AAO1037
Paratendipes albimanus|BIOUG15948-A08|Kate Perez|BIN Taxonomy Match|BOLD:AAO1037
Paratendipes albimanus|BIOUG15948-H04|Kate Perez|BIN Taxonomy Match|BOLD:AAO1037
Paratendipes albimanus|BIOUG15967-D01|Kate Perez|BIN Taxonomy Match|BOLD:AAO1037
Paratendipes albimanus|BIOUG15965-A05|Kate Perez|BIN Taxonomy Match|BOLD:AAO1037
Paratendipes albimanus|BIOUG15905-D03|Kate Perez|BIN Taxonomy Match|BOLD:AAO1037
Paratendipes albimanus|BIOUG15905-D05|Kate Perez|BIN Taxonomy Match|BOLD:AAO1037
Paratendipes albimanus|BIOUG15948-D09|Kate Perez|BIN Taxonomy Match|BOLD:AAO1037
Paratendipes albimanus|BIOUG15954-C06|Kate Perez|BIN Taxonomy Match|BOLD:AAO1037
Paratendipes albimanus|BIOUG15966-G12|Kate Perez|BIN Taxonomy Match|BOLD:AAO1037
Paratendipes albimanus|BIOUG15967-D02|Kate Perez|BIN Taxonomy Match|BOLD:AAO1037
Paratendipes albimanus|BIOUG15967-H07|Kate Perez|BIN Taxonomy Match|BOLD:AAO1037
Paratendipes albimanus|BIOUG15951-H03|Kate Perez|BIN Taxonomy Match|BOLD:AAO1037
Paratendipes albimanus|BIOUG16330-E01|Kate Perez|BIN Taxonomy Match|BOLD:AAO1037
Paratendipes albimanus|BIOUG15900-H04|Kate Perez|BIN Taxonomy Match|BOLD:AAO1037
Paratendipes albimanus|BIOUG15952-B01|Kate Perez|BIN Taxonomy Match|BOLD:AAO1037
Paratendipes albimanus|BIOUG15967-G12|Kate Perez|BIN Taxonomy Match|BOLD:AAO1037
Paratendipes albimanus|BIOUG15950-G06|Kate Perez|BIN Taxonomy Match|BOLD:AAO1037
Paratendipes albimanus|BIOUG15953-G10|Kate Perez|BIN Taxonomy Match|BOLD:AAO1037
Paratendipes albimanus|BIOUG15950-C08|Kate Perez|BIN Taxonomy Match|BOLD:AAO1037
Paratendipes albimanus|BIOUG15948-G04|Kate Perez|BIN Taxonomy Match|BOLD:AAO1037
Paratendipes albimanus|BIOUG15952-D01|Kate Perez|BIN Taxonomy Match|BOLD:AAO1037
Paratendipes albimanus|BIOUG15950-E08|Kate Perez|BIN Taxonomy Match|BOLD:AAO1037
Paratendipes albimanus|BIOUG15952-C08|Kate Perez|BIN Taxonomy Match|BOLD:AAO1037
Paratendipes albimanus|EJV-20110098|Lauri Paasivirta||BOLD:AAO1037
Paratendipes albimanus|BIOUG15966-F05|Kate Perez|BIN Taxonomy Match|BOLD:AAO1037
Tribelos intextum|ZMUO.024856|Lauri Paasivirta||BOLD:AAV3590
[illegible]

Paratendipes albimanus|EJV-20110098|Lauri Paasivirta||BOLD:AAO1037
Paratendipes albimanus|BIOUG15966-F05|Kate Perez|BIN Taxonomy Match|BOLD:AAO1037
Tribelos intextum|ZMUO.024856|Lauri Paasivirta||BOLD:AAV3590
Tribelos intextum|ZMUO.024855|Lauri Paasivirta||BOLD:AAV3590
Tribelos intextum|CHNY03|Yngve Brodin||BOLD:AAV3590
Tribelos intextum|NHRS-BYWS000001059|Yngve Brodin|morphology|BOLD:AAV3590
Tribelos intextum|CH-OSF147|Elisabeth Stur||BOLD:AAV3590
Polypedilum tritum|ZMUO.024259|Lauri Paasivirta||BOLD:ADA7118
Polypedilum sp. 6TE|TRD-CH230|Elisabeth Stur|morphology|BOLD:AAW4670
Polypedilum sp. 6TE|TRD-CH20|Elisabeth Stur|morphology|BOLD:AAW4670
Polypedilum sp. 6TE|TRD-CH13|Elisabeth Stur|morphology|BOLD:AAW4670
Polypedilum sp. 6TE|TRD-CH22|Elisabeth Stur|morphology|BOLD:AAW4670
Polypedilum sp. 6TE|RIN_CH05|Elisabeth Stur|morphology|BOLD:AAW4670
Polypedilum sp. 6TE|RIN_CH10|Elisabeth Stur|morphology|BOLD:AAW4670
Polypedilum tritum|NHRS-BYWS000000583|Yngve Brodin|morphology|BOLD:AAW4670
Polypedilum tritum|EJV-20110024|Lauri Paasivirta||BOLD:AAW4670
Polypedilum tritum|EJV-20110022|Lauri Paasivirta||BOLD:AAW4670
Polypedilum tritum|EJV-20110023|Lauri Paasivirta||BOLD:AAW4670
Polypedilum aegyptium|ZMUO.025389|Lauri Paasivirta||BOLD:ADE9660
Polypedilum aegyptium|ZMUO.025388|Lauri Paasivirta||BOLD:ADE9660
Polypedilum amoenum|ZMUO.026129|Lauri Paasivirta||BOLD:ADF1247
Polypedilum amoenum|ZMUO.026128|Lauri Paasivirta||BOLD:ADF1247
Polypedilum amoenum|PP0267|Lauri Paasivirta||BOLD:ADF1247
Heterotrissocladius grimshawi|KY225361
Sergentia psiloptera|KY225371|||BOLD:AAP6561
Sergentia psiloptera|KY225372|||BOLD:AAP6561
Sergentia sp. 2TE|CH-OSF146|Torbjorn Ekrem||BOLD:AAP6561
Sergentia prima|ZMUO.029773|Marko Mutanen||BOLD:AAP6561
Sergentia coracina|ZMUO.024633|Lauri Paasivirta||BOLD:AAP6561
Sergentia sp. 2TE|TRD-CH287|Elisabeth Stur||BOLD:AAP6561
Sergentia sp. 2TE|TRD-CH280|Elisabeth Stur||BOLD:AAP6561
Sergentia sp. 2TE|Finnmark226|Torbjorn Ekrem||BOLD:AAP6561
Sergentia sp. 1TE|TRD-CH259|Elisabeth Stur||BOLD:AAM9441
Sergentia baueri|ZSM-DIP-33142-D06|Caroline Chimeno|BIN Taxonomy Match (Jan 2022)|BOLD:A...
Sergentia sp. 1TE|TRD-CH167|Elisabeth Stur||BOLD:AAM9441
Sergentia baueri|ZMUO.029820|Marko Mutanen||BOLD:AAM9441
Sergentia baueri|ZMUO.025949|Lauri Paasivirta||BOLD:AAM9441
Sergentia sp. 1TE|Finnmark11|Torbjorn Ekrem||BOLD:AAM9441
Sergentia sp. 1TE|Finnmark541|Torbjorn Ekrem||BOLD:AAM9441
Sergentia prima|NO05|Torbjorn Ekrem||BOLD:AAJ7711
Sergentia baueri|NHRS-BYWS000001154|Yngve Brodin|morphology|BOLD:AAJ7711
Sergentia prima|ZMUO.024293|Lauri Paasivirta||BOLD:AAJ7711
Sergentia prima|ZMUO.024292|Lauri Paasivirta||BOLD:AAJ7711
Sergentia|BIOUG43948-G02|Kate Perez|BIN Taxonomy Match below Phylum (Jan 2019)|BOLD:AAJ7711
Sergentia prima|Finnmark13|Elisabeth Stur||BOLD:AAJ7711
Sergentia prima|ZMUO.024626|Lauri Paasivirta||BOLD:AAJ7711
Sergentia baueri|NHRS-BYWS000001169|Yngve Brodin|morphology|BOLD:AAJ7711
Sergentia|ZMUO.026214|Lauri Paasivirta||BOLD:AAJ7711
Sergentia prima|ZMUO.024625|Lauri Paasivirta||BOLD:AAJ7711
Sergentia prima|TRD-CH127|Elisabeth Stur||BOLD:AAJ7711
Sergentia coracina|NHRS-BYWS000001153|Yngve Brodin|morphology|BOLD:AAH9836
Sergentia coracina|NO06|Torbjorn Ekrem||BOLD:AAH9836
Sergentia coracina|NO04|Torbjorn Ekrem||BOLD:AAH9836
Sergentia coracina|TRD-CH126|Elisabeth Stur||BOLD:AAH9836
Sergentia coracina|Finnmark525|Torbjorn Ekrem||BOLD:AAH9836
Chironomidae|BIOUG42958-G02|Kate Perez|BIN Taxonomy Match below Phylum (Jul 2019)|BOLD:A...
Sergentia sp. 3TE|Finnmark387|Torbjorn Ekrem||BOLD:AAV2350
Synendotendipes lepidus|ZMUO.024240|Lauri Paasivirta||BOLD:ACT5753
Synendotendipes impar|CCDB21606-E01|Sofia Wiedenbrug||BOLD:ACT5753
Synendotendipes impar|CCDB21606-C12|Sofia Wiedenbrug||BOLD:ACT5753
Synendotendipes sp. 1ES|TRD-CH341|Elisabeth Stur|morphology|BOLD:ACT5753
Synendotendipes luski|Finnmark665|Torbjorn Ekrem||BOLD:AAV3585
Synendotendipes dispar|NHRS-BYWS000001158|Yngve Brodin|morphology|BOLD:AEL5382
Synendotendipes luski|Finnmark807|Torbjorn Ekrem||BOLD:AAV3585
Synendotendipes luski|Finnmark663|Torbjorn Ekrem||BOLD:AAV3585
Synendotendipes lepidus|NHRS-BYWS000001084|Yngve Brodin|morphology|BOLD:AAV3351
Synendotendipes impar|ZMUO.025385|Lauri Paasivirta||BOLD:AAV3351
Synendotendipes impar|ZMUO.025384|Lauri Paasivirta||BOLD:AAV3351
Synendotendipes lepidus|NHRS-BYWS000000312|Yngve Brodin|morphology|BOLD:AAV3351
Synendotendipes impar|ZMUO.024694|Lauri Paasivirta||BOLD:AAV3351
Synendotendipes impar|RIN_CH91|Mona Renate Saursaunet|BOLD Identification engine|BOLD:AAV3351
Synendotendipes lepidus|NHRS-BYWS000000535|Yngve Brodin|morphology|BOLD:AAV3351
Synendotendipes impar|RIN_CH20|Mona Renate Saursaunet|BOLD Identification engine|BOLD:AAV3351
Synendotendipes impar|Finnmark742|Torbjorn Ekrem||BOLD:AAV3351
Synendotendipes impar|RIN_CH47|Mona Renate Saursaunet|BOLD Identification engine|BOLD:AAV3351
Synendotendipes impar|RIN_CH18|Mona Renate Saursaunet|BOLD Identification engine|BOLD:AAV3351
Synendotendipes impar|RIN_CH12|Mona Renate Saursaunet|BOLD Identification engine|BOLD:AAV3351
Synendotendipes impar|RIN_CH39|Mona Renate Saursaunet|BOLD Identification engine|BOLD:AAV3351
Synendotendipes impar|RIN_CH48|Mona Renate Saursaunet|BOLD Identification engine|BOLD:AAV3351
Synendotendipes impar|RIN_CH49|Mona Renate Saursaunet|BOLD Identification engine|BOLD:AAV3351
Synendotendipes impar|RIN_CH61|Mona Renate Saursaunet|BOLD Identification engine|BOLD:AAV3351
Synendotendipes impar|RIN_CH82|Mona Renate Saursaunet|BOLD Identification engine|BOLD:AAV3351
Synendotendipes impar|TRD-CH31|Elisabeth Stur||BOLD:AAV3351
Synendotendipes impar|TRD-CH206|Elisabeth Stur||BOLD:AAV3351
Synendotendipes impar|TRD-CH342|Elisabeth Stur|morphology|BOLD:AAV3351
Synendotendipes impar|RIN_CH19|Mona Renate Saursaunet|BOLD Identification engine|BOLD:AAV3351
Synendotendipes impar|TRD-CH344|Elisabeth Stur|morphology|BOLD:AAV3351
Synendotendipes impar|TRD-CH210|Elisabeth Stur||BOLD:AAV3351
Synendotendipes impar|TRD-CH345|Elisabeth Stur|morphology
Synendotendipes impar|CH-OSF170|Torbjorn Ekrem||BOLD:AAV3351
Synendotendipes impar|TRD-CH30|Elisabeth Stur||BOLD:AAV3351
Synendotendipes impar|Finnmark445|Torbjorn Ekrem||BOLD:AAV3351
Synendotendipes impar|ZMUO.024695|Lauri Paasivirta||BOLD:AAV3351
Synendotendipes impar|RIN_CH64|Mona Renate Saursaunet|BOLD Identification engine|BOLD:AAV3351
Synendotendipes impar|EJV-20110016|Lauri Paasivirta||BOLD:AAV3351
Chironomidae|ZSM-DIP-33147-C03|Valerie Levesque-Beaudin|BIN Taxonomy Match (Nov 2021)|BOLD...
Polypedilum pedestre|TRD-CH182|Elisabeth Stur||BOLD:ACT6930
Polypedilum|CH-OSF176|Torbjorn Ekrem||BOLD:AAV3588
Polypedilum nubeculosum|ZSM-DIP-33128-B06|Caroline Chimeno|BIN Taxonomy Match (Jan 2022)|BO...
Polypedilum nubeculosum|ZSM-DIP-33144-E12|Caroline Chimeno|BIN Taxonomy Match (Jan 2022)|BO...
Polypedilum nubeculosum|ZSM-DIP-33144-C10|Caroline Chimeno|BIN Taxonomy Match (Jan 2022)|BO...
Polypedilum nubeculosum|ZSM-DIP-33142-G10|Caroline Chimeno|BIN Taxonomy Match (Jan 2022)|BO...
Polypedilum nubeculosum|ZSM-DIP-33142-G07|Caroline Chimeno|BIN Taxonomy Match (Jan 2022)|BO...

Polypedilum nubeculosum|ZSM-DIP-33144-C10|Caroline Chimeno|BIN Taxonomy Match (Jan 2022)|BO...
Polypedilum nubeculosum|ZSM-DIP-33142-G10|Caroline Chimeno|BIN Taxonomy Match (Jan 2022)|BO...
Polypedilum nubeculosum|ZSM-DIP-33142-G07|Caroline Chimeno|BIN Taxonomy Match (Jan 2022)|BO...
Polypedilum nubeculosum|BIOUG36761-C09|Kate Perez|BIN Taxonomy Match (Jul 2018)|BOLD:AER...
Polypedilum nubeculosum|ZSM-DIP-33134-E02|Caroline Chimeno|BIN Taxonomy Match (Jan 2022)|BO...
Polypedilum nubeculosum|NHRS-BYWS000001185|Yngve Brodin|morphology|BOLD:AER2796
Polypedilum nubeculosum|ZMUO.024549|Lauri Paasivirta||BOLD:AAY3395
Polypedilum nubeculosum|ZMUO.024550|Lauri Paasivirta||BOLD:AAY3395
Polypedilum nubeculosum|NHRS-BYWS000001176|Yngve Brodin|morphology|BOLD:AAY3395
Polypedilum nubeculosum|BIOUG55120-G01|Kate Perez|BIN Taxonomy Match (Oct 2020)|BOLD:AAY...
Polypedilum nubeculosum|NHRS-BYWS000001022|Yngve Brodin|morphology|BOLD:AAY3395
Polypedilum nubeculosum|song191|xxx Chao|Morphology|BOLD:AAY3395
Polypedilum nubeculosum|BIOUG36734-H11|Kate Perez|BIN Taxonomy Match (May 2018)|BOLD:AAY...
Polypedilum nubeculosum|BIOUG37052-D04|Kate Perez|BIN Taxonomy Match (Jul 2018)|BOLD:AAY...
Polypedilum nubeculosum|NHRS-BYWS000000906|Yngve Brodin|morphology|BOLD:AAY3395
Polypedilum nubeculosum|NHRS-BYWS000001844|Yngve Brodin||BOLD:AAY3395
Polypedilum albicorne|EBAI-Ch210|Elisabeth Stur|morphology
Polypedilum albicorne|TRD-CH330|Elisabeth Stur||BOLD:AAL0178
Polypedilum albicorne|ZMUO.024908|Lauri Paasivirta||BOLD:AAL0178
Polypedilum albicorne|ZMUO.024909|Lauri Paasivirta||BOLD:AAL0178
Polypedilum albicorne|BIOUG16601-A12|Kate Perez|BIN Taxonomy Match|BOLD:AAL0178
Polypedilum albicorne|BIOUG15905-H06|Kate Perez|BIN Taxonomy Match|BOLD:AAL0178
Polypedilum albicorne|BIOUG16597-B04|Kate Perez|BIN Taxonomy Match|BOLD:AAL0178
Polypedilum albicorne|BIOUG15947-A05|Kate Perez|BIN Taxonomy Match|BOLD:AAL0178
Polypedilum albicorne|BIOUG16198-B07|Kate Perez|BIN Taxonomy Match|BOLD:AAL0178
Polypedilum albicorne|BIOUG15964-G05|Kate Perez|BIN Taxonomy Match|BOLD:AAL0178
Polypedilum albicorne|BIOUG15951-C01|Kate Perez|BIN Taxonomy Match|BOLD:AAL0178
Polypedilum albicorne|BIOUG15953-C09|Kate Perez|BIN Taxonomy Match|BOLD:AAL0178
Polypedilum albicorne|BIOUG16476-G02|Kate Perez|BIN Taxonomy Match|BOLD:AAL0178
Polypedilum albicorne|BIOUG16436-B08|Kate Perez|BIN Taxonomy Match|BOLD:AAL0178
Polypedilum albicorne|BIOUG15953-C08|Kate Perez|BIN Taxonomy Match|BOLD:AAL0178
Polypedilum albicorne|BIOUG15950-E04|Kate Perez|BIN Taxonomy Match|BOLD:AAL0178
Polypedilum albicorne|BIOUG15950-C04|Kate Perez|BIN Taxonomy Match|BOLD:AAL0178
Polypedilum albicorne|BIOUG15900-G06|Kate Perez|BIN Taxonomy Match|BOLD:AAL0178
Polypedilum albicorne|Finnmark178|Elisabeth Stur||BOLD:AAL0178
Polypedilum albicorne|ES75|Elisabeth Stur||BOLD:AAL0178
Polypedilum albicorne|ZSM-DIP-33147-B12|Caroline Chimeno|BIN Taxonomy Match (Jan 2022)|BOLD...
Polypedilum albicorne|ZSM-DIP-33142-F09|Caroline Chimeno|BIN Taxonomy Match (Jan 2022)|BOLD...
Polypedilum albicorne|ZSM-DIP-33130-C11|Caroline Chimeno|BIN Taxonomy Match (Jan 2022)|BOLD...
Polypedilum albicorne|ATNA535|Elisabeth Stur||BOLD:AAL0178
Polypedilum albicorne|BIOUG16198-B04|Kate Perez|BIN Taxonomy Match|BOLD:AAL0178
Polypedilum albicorne|BIOUG16596-G07|Kate Perez|BIN Taxonomy Match|BOLD:AAL0178
Polypedilum albicorne|BIOUG16198-D05|Kate Perez|BIN Taxonomy Match|BOLD:AAL0178
Polypedilum albicorne|BIOUG15905-F02|Kate Perez|BIN Taxonomy Match|BOLD:AAL0178
Polypedilum albicorne|BIOUG16198-G02|Kate Perez|BIN Taxonomy Match|BOLD:AAL0178
Polypedilum albicorne|BIOUG15964-A06|Kate Perez|BIN Taxonomy Match|BOLD:AAL0178
Polypedilum albicorne|BIOUG16430-E07|Kate Perez|BIN Taxonomy Match|BOLD:AAL0178
Polypedilum albicorne|BIOUG15952-B03|Kate Perez|BIN Taxonomy Match|BOLD:AAL0178
Polypedilum albicorne|BIOUG16297-E07|Kate Perez|BIN Taxonomy Match|BOLD:AAL0178
Polypedilum albicorne|BIOUG16205-C07|Kate Perez|BIN Taxonomy Match|BOLD:AAL0178
Polypedilum albicorne|BIOUG15954-F08|Kate Perez|BIN Taxonomy Match|BOLD:AAL0178
Polypedilum albicorne|BIOUG15948-A02|Kate Perez|BIN Taxonomy Match|BOLD:AAL0178
Polypedilum albicorne|ZSM-DIP-33147-B08|Caroline Chimeno|BIN Taxonomy Match (Jan 2022)|BOLD...
Polypedilum albicorne|TRD-CH192|Elisabeth Stur||BOLD:AAL0178
Polypedilum albicorne|Finnmark889|Elisabeth Stur||BOLD:AAL0178
Polypedilum albicorne|EBAI-Ch188|Elisabeth Stur|morphology|BOLD:AAL0178
Polypedilum albicorne|BIOUG15905-D09|Kate Perez|BIN Taxonomy Match|BOLD:AAL0178
Polypedilum albicorne|BIOUG16597-A09|Kate Perez|BIN Taxonomy Match|BOLD:AAL0178
Polypedilum albicorne|BIOUG15954-G03|Kate Perez|BIN Taxonomy Match|BOLD:AAL0178
Polypedilum albicorne|BIOUG15947-F11|Kate Perez|BIN Taxonomy Match|BOLD:AAL0178
Polypedilum albicorne|BIOUG15963-F04|Kate Perez|BIN Taxonomy Match|BOLD:AAL0178
Polypedilum albicorne|BIOUG15949-A09|Kate Perez|BIN Taxonomy Match|BOLD:AAL0178
Polypedilum albicorne|BIOUG15954-B08|Kate Perez|BIN Taxonomy Match|BOLD:AAL0178
Polypedilum albicorne|BIOUG16198-E02|Kate Perez|BIN Taxonomy Match|BOLD:AAL0178
Polypedilum albicorne|BIOUG16207-E10|Kate Perez|BIN Taxonomy Match|BOLD:AAL0178
Polypedilum albicorne|BIOUG16205-A07|Kate Perez|BIN Taxonomy Match|BOLD:AAL0178
Polypedilum albicorne|BIOUG15950-D09|Kate Perez|BIN Taxonomy Match|BOLD:AAL0178
Polypedilum albicorne|BIOUG15967-B07|Kate Perez|BIN Taxonomy Match|BOLD:AAL0178
Polypedilum albicorne|BIOUG15963-C03|Kate Perez|BIN Taxonomy Match|BOLD:AAL0178
Polypedilum albicorne|BIOUG15950-A02|Kate Perez|BIN Taxonomy Match|BOLD:AAL0178
Polypedilum albicorne|BIOUG16259-F01|Kate Perez|BIN Taxonomy Match|BOLD:AAL0178
Polypedilum albicorne|BIOUG15951-B04|Kate Perez|BIN Taxonomy Match|BOLD:AAL0178
Polypedilum albicorne|BIOUG16596-B11|Kate Perez|BIN Taxonomy Match|BOLD:AAL0178
Polypedilum albicorne|BIOUG15953-G02|Kate Perez|BIN Taxonomy Match|BOLD:AAL0178
Polypedilum albicorne|BIOUG15951-F09|Kate Perez|BIN Taxonomy Match|BOLD:AAL0178
Polypedilum albicorne|BIOUG15900-G03|Kate Perez|BIN Taxonomy Match|BOLD:AAL0178
Polypedilum albicorne|ATNA551|Elisabeth Stur||BOLD:AAL0178
Polypedilum albicorne|ATNA534|Elisabeth Stur||BOLD:AAL0178
Polypedilum albicorne|BIOUG08127-E12|Kate Perez|BIN Taxonomy Match|BOLD:AAL0178
Polypedilum albicorne|BIOUG05899-B02|Kate Perez|BIN Taxonomy Match|BOLD:AAL0178
Polypedilum albicorne|BIOUG07807-H06|Kate Perez|BIN Taxonomy Match|BOLD:AAL0178
Polypedilum albicorne|TRD-CH329|Elisabeth Stur||BOLD:AAL0178
Polypedilum albicorne|CCDB21606-G05|Sofia Wiedenbrug||BOLD:AAL0178
Polypedilum albicorne|BIOUG16198-A11|Kate Perez|BIN Taxonomy Match|BOLD:AAL0178
Polypedilum albicorne|BIOUG15966-H02|Kate Perez|BIN Taxonomy Match|BOLD:AAL0178
Polypedilum albicorne|BIOUG15954-F11|Kate Perez|BIN Taxonomy Match|BOLD:AAL0178
Polypedilum albicorne|BIOUG15951-H11|Kate Perez|BIN Taxonomy Match|BOLD:AAL0178
Polypedilum albicorne|BIOUG15964-E11|Kate Perez|BIN Taxonomy Match|BOLD:AAL0178
Polypedilum albicorne|BIOUG16198-B10|Kate Perez|BIN Taxonomy Match|BOLD:AAL0178
Polypedilum albicorne|BIOUG16596-C07|Kate Perez|BIN Taxonomy Match|BOLD:AAL0178
Polypedilum albicorne|BIOUG16526-D06|Kate Perez|BIN Taxonomy Match|BOLD:AAL0178
Polypedilum albicorne|BIOUG08257-F09|Kate Perez|BIN Taxonomy Match|BOLD:AAL0178
Polypedilum albicorne|BIOUG15966-H06|Kate Perez|BIN Taxonomy Match|BOLD:AAL0178
Polypedilum albicorne|BIOUG15954-D10|Kate Perez|BIN Taxonomy Match|BOLD:AAL0178
Polypedilum albicorne|BIOUG15952-A02|Kate Perez|BIN Taxonomy Match|BOLD:AAL0178
Polypedilum albicorne|BIOUG16328-C05|Kate Perez|BIN Taxonomy Match|BOLD:AAL0178
Polypedilum albicorne|BIOUG15952-H04|Kate Perez|BIN Taxonomy Match|BOLD:AAL0178
Polypedilum albicorne|BIOUG15951-B09|Kate Perez|BIN Taxonomy Match|BOLD:AAL0178
Polypedilum albicorne|BIOUG15951-H06|Kate Perez|BIN Taxonomy Match|BOLD:AAL0178
Polypedilum albicorne|BIOUG08314-D01|Kate Perez|BIN Taxonomy Match|BOLD:AAL0178
Tribelos|ZMUO.026011|Lauri Paasivirta||BOLD:ADF1087
Tribelos|ZMUO.026012|Lauri Paasivirta||BOLD:ADF1087
Endochironomus albipennis|NHRS-BYWS000000534|Yngve Brodin|morphology|BOLD:ACD8095

Tribelos|ZMUO.026011|Lauri Paasivirta||BOLD:ADF1087
Tribelos|ZMUO.026012|Lauri Paasivirta||BOLD:ADF1087
Endochironomus albipennis|NHRS-BYWS000000534|Yngve Brodin|morphology|BOLD:ACD8095
Endochironomus tendens|ZMUO.024255|Lauri Paasivirta||BOLD:ACD8095
Endochironomus tendens|ZMUO.024931|Lauri Paasivirta||BOLD:ACD8095
Endochironomus tendens|ZMUO.024932|Lauri Paasivirta||BOLD:ACD8095
Endochironomus tendens|ZMUO.024254|Lauri Paasivirta||BOLD:ACD8095
Endochironomus tendens|ZMUO.024245|Lauri Paasivirta||BOLD:ACD8095
Endochironomus tendens|ZMUO.024224|Lauri Paasivirta||BOLD:ACD8095
Endochironomus albipennis|NHRS-BYWS000001030|Yngve Brodin|morphology|BOLD:AEB2387
Endochironomus stackelbergi|ZMUO.024810|Lauri Paasivirta||BOLD:AAW5643
Endochironomus stackelbergi|ZMUO.024809|Lauri Paasivirta||BOLD:AAW5643
Endochironomus albipennis|KY225348|||BOLD:AAW5643
Endochironomus tendens|EJV-20110113|Lauri Paasivirta||BOLD:AAW5643
Endochironomus tendens|NHRS-BYWS000000384|Yngve Brodin|morphology|BOLD:AAW5643
Endochironomus tendens|NHRS-BYWS000001186|Yngve Brodin|morphology|BOLD:AAW5643
Endochironomus albipennis|EJV-20110231|Lauri Paasivirta||BOLD:AAW5643
Endochironomus tendens|NHRS-BYWS000001141|Yngve Brodin|morphology|BOLD:AAW5643
Endochironomus tendens|NHRS-BYWS000000969|Yngve Brodin|morphology|BOLD:AAW5643
Endochironomus tendens|NHRS-BYWS000001005|Yngve Brodin|morphology|BOLD:AAW5643
Endochironomus tendens|NHRS-BYWS000001006|Yngve Brodin|morphology|BOLD:AAW5643
Endochironomus tendens|NHRS-BYWS000001007|Yngve Brodin|morphology|BOLD:AAW5643
Endochironomus tendens|NHRS-BYWS000001023|Yngve Brodin|morphology|BOLD:AAW5643
Endochironomus tendens|NHRS-BYWS000001042|Yngve Brodin|morphology|BOLD:AAW5643
Endochironomus albipennis|ZMUO.024745|Lauri Paasivirta||BOLD:AAW5643
Endochironomus albipennis|ZMUO.024744|Lauri Paasivirta||BOLD:AAW5643
Endochironomus albipennis|EJV-20110230|Lauri Paasivirta||BOLD:AAW5643
Polypedilum cultellatum|ZMUO.024261|Lauri Paasivirta||BOLD:AAQ1361
Polypedilum cultellatum|ZMUO.024260|Lauri Paasivirta||BOLD:AAQ1361
Polypedilum cultellatum|ZSM-DIP-33147-C07|Caroline Chimeno|BIN Taxonomy Match (Jan 2022)|BO...
Polypedilum cultellatum|song202|xxx Chao|Morphology|BOLD:AAQ1361
Polypedilum cultellatum|ZSM-DIP-33137-G09|Caroline Chimeno|BIN Taxonomy Match (Jan 2022)|BO...
Polypedilum cultellatum|EJV-20110158|Lauri Paasivirta||BOLD:AAQ1361
Polypedilum cultellatum|EJV-20110157|Lauri Paasivirta||BOLD:AAQ1361
Paralauterborniella nigrohalteralis|NHRS-BYWS000001009|Yngve Brodin|morphology|BOLD:ADA8916
Paralauterborniella nigrohalteralis|ZMUO.024971|Lauri Paasivirta||BOLD:ADA8916
Paralauterborniella nigrohalteralis|NHRS-BYWS000001124|Yngve Brodin|morphology|BOLD:ADA8916
Polypedilum acutum|PP0276|Lauri Paasivirta||BOLD:ADZ1216
Lauterborniella agrayloides|ZMUO.025330|Lauri Paasivirta||BOLD:ADF0433
Lauterborniella agrayloides|ZMUO.025329|Lauri Paasivirta||BOLD:ADF0433
Lauterborniella agrayloides|NHRS-BYWS000000239|Yngve Brodin|morphology|BOLD:ADF0433
Lauterborniella agrayloides|CH-OSF185|Elisabeth Stur||BOLD:AAV2669
Microtendipes rydalensis|ZMUO.025111|Lauri Paasivirta||BOLD:ADA9434
Microtendipes rydalensis|ZMUO.025110|Lauri Paasivirta||BOLD:ADA9434
Zavreliella marmorata|ZMUO.025391|Lauri Paasivirta||BOLD:ADF0299
Zavreliella marmorata|ZMUO.025390|Lauri Paasivirta||BOLD:ADF0299
Phaenopsectra|BIOUG16647-C10|Kate Perez|BOLD ID Engine Manual|BOLD:ACR1613
Microtendipes brevitarsis|NHRS-BYWS000001161|Yngve Brodin|morphology|BOLD:AAG5479
Microtendipes sp. 1ES|TRD-CH96|Elisabeth Stur|morphology|BOLD:AAG5479
Microtendipes britteni|CCDB22605-G10|Sofia Wiedenbrug||BOLD:ACT8650
Microtendipes britteni|CCDB22605-G09|Sofia Wiedenbrug||BOLD:ACT8650
Microtendipes britteni|ZSM-DIP-33143-A01|Caroline Chimeno|BIN Taxonomy Match (Jan 2022)|BOL...
Microtendipes britteni|ZSM-DIP-33142-D03|Caroline Chimeno|BIN Taxonomy Match (Jan 2022)|BOL...
Microtendipes britteni|ZSM-DIP-33142-D02|Caroline Chimeno|BIN Taxonomy Match (Jan 2022)|BOL...
Microtendipes|BIOUG17262-E12|Kate Perez|BOLD ID Engine Manual|BOLD:ACR0670
Microtendipes|BIOUG16526-E02|Kate Perez|BOLD ID Engine Manual|BOLD:ACR0670
Microtendipes confinis|ZSM-DIP-33149-C06|Caroline Chimeno|BIN Taxonomy Match (Jan 2022)|BOL...
Microtendipes confinis|ZSM-DIP-33170-B09|Caroline Chimeno|BIN Taxonomy Match (Jan 2022)|BOL...
Microtendipes|BIOUG17380-E12|Kate Perez|BOLD ID Engine Manual|BOLD:ACQ9190
Microtendipes|BIOUG17137-C09|Kate Perez|BOLD ID Engine Manual|BOLD:ACQ9190
Microtendipes|BIOUG17376-H09|Kate Perez|BOLD ID Engine Manual|BOLD:ACQ9190
Microtendipes confinis|ZMUO.026182|Lauri Paasivirta||BOLD:ACQ9190
Microtendipes confinis|ZMUO.026181|Lauri Paasivirta||BOLD:ACQ9190
Microtendipes|BIOUG16706-F11|Kate Perez|BOLD ID Engine Manual|BOLD:ACQ9190
Microtendipes confinis|NHRS-BYWS000000905|Yngve Brodin|morphology|BOLD:ACQ9190
Microtendipes confinis|NHRS-BYWS000000500|Yngve Brodin|morphology|BOLD:ACQ9190
Microtendipes confinis|NHRS-BYWS000000361|Yngve Brodin|morphology|BOLD:ACQ9190
Microtendipes confinis|NHRS-BYWS000000343|Yngve Brodin|morphology|BOLD:ACQ9190
Microtendipes confinis|NHRS-BYWS000000342|Yngve Brodin|morphology|BOLD:ACQ9190
Microtendipes|BIOUG16525-A08|Kate Perez|BOLD ID Engine Manual|BOLD:ACQ9190
Microtendipes pedellus|CCDB22605-G07|Sofia Wiedenbrug||BOLD:ACR0263
Microtendipes pedellus|ZSM-DIP-33144-G03|Caroline Chimeno|BIN Taxonomy Match (Jan 2022)|BOL...
Microtendipes pedellus|ZSM-DIP-33134-G04|Caroline Chimeno|BIN Taxonomy Match (Jan 2022)|BOL...
Microtendipes pedellus|ZSM-DIP-33140-H10|Caroline Chimeno|BIN Taxonomy Match (Jan 2022)|BOL...
Microtendipes pedellus|ZSM-DIP-33142-H11|Caroline Chimeno|BIN Taxonomy Match (Jan 2022)|BOL...
Microtendipes|ZSM-DIP-33141-A03|Caroline Chimeno|BIN Taxonomy Match (Jan 2022)|BOLD:ACR0263
Microtendipes pedellus|ZSM-DIP-33142-H10|Caroline Chimeno|BIN Taxonomy Match (Jan 2022)|BOL...
Microtendipes pedellus|ZSM-DIP-33141-H10|Caroline Chimeno|BIN Taxonomy Match (Jan 2022)|BOL...
Microtendipes pedellus|ZSM-DIP-33170-B04|Caroline Chimeno|BIN Taxonomy Match (Jan 2022)|BOL...
Microtendipes pedellus|ZSM-DIP-33144-G02|Caroline Chimeno|BIN Taxonomy Match (Jan 2022)|BOL...
Microtendipes pedellus|ZSM-DIP-33131-G12|Caroline Chimeno|BIN Taxonomy Match (Jan 2022)|BOL...
Microtendipes pedellus|ZSM-DIP-33133-A02|Caroline Chimeno|BIN Taxonomy Match (Jan 2022)|BOL...
Microtendipes pedellus|BIOUG44041-H07|Kate Perez|BIN Taxonomy Match (Jun 2019)|BOLD:ACR0263
Microtendipes cf. pedellus|NHRS-BYWS000000536|Yngve Brodin|morphology|BOLD:ACR0263
Microtendipes pedellus|ZSM-DIP-33144-G04|Caroline Chimeno|BIN Taxonomy Match (Jan 2022)|BOL...
Microtendipes|ZSM-DIP-33141-A04|Caroline Chimeno|BIN Taxonomy Match (Jan 2022)|BOLD:ACR0263
Microtendipes pedellus|ZSM-DIP-33131-C10|Caroline Chimeno|BIN Taxonomy Match (Jan 2022)|BOL...
Microtendipes pedellus|ZMUO.024243|Lauri Paasivirta||BOLD:ACR0263
Microtendipes pedellus|ZMUO.024242|Lauri Paasivirta||BOLD:ACR0263
Microtendipes pedellus|BIOUG16614-H07|Kate Perez|BOLD ID Engine Manual|BOLD:ACR0263
Microtendipes nigellus|TRD-CH79|Elisabeth Stur||BOLD:AAP6560
Microtendipes nigellus|Finnmark143|Elisabeth Stur||BOLD:AAP6560
Microtendipes chloris|Finnmark416|Torbjorn Ekrem||BOLD:AEL5025
Microtendipes chloris|ZMUO.024318|Lauri Paasivirta||BOLD:ACY5270
Microtendipes chloris|ZMUO.024317|Lauri Paasivirta||BOLD:ACY5270
Microtendipes chloris|KY225368|||BOLD:ACY5270
Microtendipes pedellus|NHRS-BYWS000000121|Yngve Brodin|morphology|BOLD:ACY5270
Microtendipes pedellus|NHRS-BYWS000000120|Yngve Brodin|morphology|BOLD:ACY5270
Microtendipes pedellus|NHRS-BYWS000000119|Yngve Brodin|morphology|BOLD:ACY5270
Microtendipes|ZSM-DIP-33133-E04|Caroline Chimeno|BIN Taxonomy Match (Jan 2022)|BOLD:ACY5270
Microtendipes|BIOUG36853-F08|Kate Perez|BIN Taxonomy Match (Jul 2018)|BOLD:ACY5270
Microtendipes|BIOUG36625-H03|Kate Perez|BIN Taxonomy Match (Jul 2018)|BOLD:ACY5270
Microtendipes sp. RA6_2|KY225366|||BOLD:ACY5270

Microtendipes|ZSM-DIP-33153-E04|Caroline Chimeno|BIN Taxonomy Match (Jan 2022)|BOLD:ACY5270
Microtendipes|BIOUG36853-F08|Kate Perez|BIN Taxonomy Match (Jul 2018)|BOLD:ACY5270
Microtendipes|BIOUG36625-H03|Kate Perez|BIN Taxonomy Match (Jul 2018)|BOLD:ACY5270
Microtendipes sp. PA6_2|KY225366|||BOLD:ACY5270
Microtendipes sp. PA6|KY225367|||BOLD:ACY5270
Microtendipes sp. PA3|KY225364|||BOLD:ACY5270
Microtendipes sp. PA5|KY225365|||BOLD:ACY5270
Microtendipes brevitarsis|Finnmark654|Elisabeth Stur||BOLD:AAM6832
Microtendipes brevitarsis|TRD-CH262|Elisabeth Stur||BOLD:AAM6832
Microtendipes brevitarsis|TRD-CH78|Elisabeth Stur||BOLD:AAM6832
Microtendipes brevitarsis|Finnmark52|Torbjorn Ekrem||BOLD:AAM6832
Microtendipes brevitarsis|ZMUO.024611|Lauri Paasivirta||BOLD:AAM6832
Microtendipes brevitarsis|ZMUO.024610|Lauri Paasivirta||BOLD:AAM6832
Microtendipes brevitarsis|TRD-CH300|Elisabeth Stur||BOLD:AAM6832
Microtendipes brevitarsis|TRD-CH264|Elisabeth Stur||BOLD:AAM6832
Microtendipes brevitarsis|TRD-CH263|Elisabeth Stur||BOLD:AAM6832
Microtendipes brevitarsis|TRD-CH426|Elisabeth Stur|morphology|BOLD:AAM6832
Microtendipes brevitarsis|TRD-CH425|Elisabeth Stur|morphology|BOLD:AAM6832
Microtendipes brevitarsis|TRD-CH424|Elisabeth Stur|morphology|BOLD:AAM6832
Microtendipes brevitarsis|TRD-CH92|Elisabeth Stur||BOLD:AAM6832
Microtendipes brevitarsis|Finnmark78|Elisabeth Stur||BOLD:AAM6832
Microtendipes brevitarsis|Finnmark343|Elisabeth Stur||BOLD:AAM6832
Microtendipes pedellus|NO 85|Elisabeth Stur||BOLD:ABY3509
Microtendipes pedellus|TRD-CH80|Elisabeth Stur||BOLD:ABY3509
Microtendipes pedellus|EJV-20110045|Lauri Paasivirta||BOLD:ABY3509
Microtendipes pedellus|EJV-20110040|Lauri Paasivirta||BOLD:ABY3509
Microtendipes pedellus|EJV-20110042|Lauri Paasivirta||BOLD:ABY3509
Microtendipes pedellus|EJV-20110043|Lauri Paasivirta||BOLD:ABY3509
Microtendipes pedellus|EJV-20110044|Lauri Paasivirta||BOLD:ABY3509
Microtendipes pedellus|EJV-20110046|Lauri Paasivirta||BOLD:ABY3509
Microtendipes pedellus|NHRS-BYWS000001818|Yngve Brodin||BOLD:ABY3509
Microtendipes pedellus|NHRS-BYWS000001850|Yngve Brodin||BOLD:ABY3509
Microtendipes cf. chloris|GBOL03729|Sofia Wiedenbrug|Morphology
Microtendipes pedellus|CH-OSF74|Elisabeth Stur||BOLD:ABY3509
Microtendipes pedellus|EJV-20110041|Lauri Paasivirta||BOLD:ABY3509
Microtendipes pedellus|EJV-20110039|Lauri Paasivirta||BOLD:ABY3509
Microtendipes pedellus|EJV-20110038|Lauri Paasivirta||BOLD:ABY3509
Microtendipes pedellus|EJV-20110037|Lauri Paasivirta||BOLD:ABY3509
Microtendipes cf. chloris|GBOL03727|Sofia Wiedenbrug|Morphology|BOLD:ABY3509
Microtendipes pedellus|NO 83|Elisabeth Stur||BOLD:ABY3509
Polypedilum tuberculum|TRD-CH364|Elisabeth Stur|morphology|BOLD:ACS2704
Phaenopsectra punctipes|NHRS-BYWS000000186|Yngve Brodin|morphology|BOLD:ACM9259
Polypedilum laetum|ZMUO.026239|Lauri Paasivirta||BOLD:ADD3129
Polypedilum laetum|ZMUO.026238|Lauri Paasivirta||BOLD:ADD3129
Polypedilum laetum|ZSM-DIP-33147-A07|Caroline Chimeno|BIN Taxonomy Match (Jan 2022)|BOLD ...
Polypedilum laetum|ZSM-DIP-33143-H06|Caroline Chimeno|BIN Taxonomy Match (Jan 2022)|BOLD ...
Polypedilum laetum|ZSM-DIP-33149-G01|Caroline Chimeno|BIN Taxonomy Match (Jan 2022)|BOLD ...
Polypedilum laetum|ZSM-DIP-33142-G02|Caroline Chimeno|BIN Taxonomy Match (Jan 2022)|BOLD ...
Polypedilum|ZSM-DIP-33144-A01|Caroline Chimeno|BIN Taxonomy Match (Jan 2022)|BOLD:ACP9495
Polypedilum|BIOUG16620-F03|Kate Perez|BIN Taxonomy Match|BOLD:ACP9495
Polypedilum|BIOUG16298-B04|Kate Perez|BIN Taxonomy Match (Aug 2017)|BOLD:ACP9495
Polypedilum cultellatum|TRD-CH310|Elisabeth Stur||BOLD:ACX5929
Polypedilum cultellatum|Finnmark592|Torbjorn Ekrem||BOLD:AAH7761
Phaenopsectra punctipes|ZMUO.024253|Lauri Paasivirta||BOLD:ADA9022
Phaenopsectra punctipes|ZMUO.024252|Lauri Paasivirta||BOLD:ADA9022
Phaenopsectra punctipes|ZMUO.024808|Lauri Paasivirta||BOLD:AAQ2147
Phaenopsectra punctipes|ZMUO.024807|Lauri Paasivirta||BOLD:AAQ2147
Phaenopsectra punctipes|Finnmark456|Elisabeth Stur||BOLD:AAQ2147
Phaenopsectra punctipes|CCDB21606-B05|Sofia Wiedenbrug||BOLD:AAQ2147
Phaenopsectra punctipes|TRD-CH347|Elisabeth Stur|morphology|BOLD:AAQ2147
Phaenopsectra punctipes|Finnmark589|Elisabeth Stur||BOLD:AAQ2147
Omisus caledonicus|ZMUO.024942|Lauri Paasivirta||BOLD:ADA8363
Omisus caledonicus|ZMUO.024941|Lauri Paasivirta||BOLD:ADA8363
Polypedilum nubens|GBOL02189|Martin Spies||BOLD:ACN9463
Polypedilum pullum|SOE161|Torbjorn Ekrem||BOLD:AAH7744
Polypedilum pullum|BIOUG43948-F07|Kate Perez|BIN Taxonomy Match below Phylum (Jan 2019)|BOL...
Polypedilum pullum|CH-OSF153|Elisabeth Stur||BOLD:AAH7744
Polypedilum pullum|SOE162|Torbjorn Ekrem||BOLD:AAH7744
Polypedilum sp. 4TE|TRD-CH274|Elisabeth Stur|morphology|BOLD:ACX4956
Polypedilum pullum|ZMUO.029789|Marko Mutanen||BOLD:ACX4956
Polypedilum pullum|ZMUO.024671|Lauri Paasivirta||BOLD:ACX4956
Polypedilum pullum|ZMUO.024670|Lauri Paasivirta||BOLD:ACX4956
Polypedilum pullum|ZMUO.024857|Lauri Paasivirta||BOLD:ACX4956
Polypedilum pullum|ZMUO.024858|Lauri Paasivirta||BOLD:ACX4956
Polypedilum sp. 4TE|TRD-CH271|Elisabeth Stur|morphology|BOLD:ACX4956
Polypedilum sp. 4TE|TRD-CH270|Elisabeth Stur|morphology|BOLD:ACX4956
Polypedilum albinodus|TRD-CH328|Elisabeth Stur||BOLD:AAM9576
Polypedilum albinodus|TRD-CH327|Elisabeth Stur||BOLD:AAM9576
Polypedilum albinodus|Finnmark121|Torbjorn Ekrem||BOLD:AAM9576
Polypedilum|BIOUG15948-D02|Kate Perez|BIN Taxonomy Match|BOLD:AAM9576
Polypedilum pullum|ZMUO.024790|Lauri Paasivirta||BOLD:AAM9576
Polypedilum|BIOUG15947-C07|Kate Perez|BIN Taxonomy Match|BOLD:AAM9576
Polypedilum|BIOUG44442-D01|Kate Perez|BIN Taxonomy Match below Phylum (Mar 2019)|BOLD:AA...
Polypedilum|BIOUG16257-E03|Kate Perez|BIN Taxonomy Match|BOLD:AAM9576
Polypedilum|BIOUG16430-E10|Kate Perez|BIN Taxonomy Match|BOLD:AAM9576
Polypedilum|BIOUG15953-B09|Kate Perez|BIN Taxonomy Match|BOLD:AAM9576
Polypedilum|BIOUG16261-A04|Kate Perez|BIN Taxonomy Match|BOLD:AAM9576
Polypedilum|BIOUG15966-B06|Kate Perez|BIN Taxonomy Match|BOLD:AAM9576
Polypedilum|BIOUG15952-E09|Kate Perez|BIN Taxonomy Match|BOLD:AAM9576
Polypedilum|BIOUG15950-C10|Kate Perez|BIN Taxonomy Match|BOLD:AAM9576
Polypedilum|BIOUG15950-D03|Kate Perez|BIN Taxonomy Match|BOLD:AAM9576
Polypedilum|BIOUG15949-F10|Kate Perez|BIN Taxonomy Match|BOLD:AAM9576
Polypedilum|BIOUG15905-E12|Kate Perez|BIN Taxonomy Match|BOLD:AAM9576
Polypedilum pullum|EJV-20110219|Lauri Paasivirta||BOLD:AAM9576
Polypedilum pullum|ZMUO.024791|Lauri Paasivirta||BOLD:AAW4728
Polypedilum sp. 5TE|NO 82|Elisabeth Stur|morphology|BOLD:AAW4728
Polypedilum pullum|EJV-20110218|Lauri Paasivirta||BOLD:AAW4728
Synendotendipes lepidus|ZMUO.024241|Lauri Paasivirta||BOLD:ACX4393
Synendotendipes lepidus|TRD-CH305|Elisabeth Stur||BOLD:ACX4393
Synendotendipes dispar|ZMUO.026141|Lauri Paasivirta||BOLD:ABW6010
Synendotendipes dispar|ZMUO.026140|Lauri Paasivirta||BOLD:ABW6010
Chironomidae|BI2019_B04|Bart Peeters|BOLD ID-Engine|BOLD:ABW6010
Chironomidae|BI2019_K01|Knut Andreas Eikland|BOLD ID-Engine|BOLD:ABW6010
[illegible]

Synendotendipes dispar|ZMUO.026140|Lauri Paasivirta||BOLD:ABW6010
Chironomidae|BI2019_B04|Bart Peeters|BOLD ID-Engine|BOLD:ABW6010
Chironomidae|BI2019_K01|Knut Andreas Eikland|BOLD ID-Engine|BOLD:ABW6010
Synendotendipes dispar|TRD-CH306|Elisabeth Stur||BOLD:ABW6010
Chironominae|BI2019_E04|Erlend I. F. Fossen|BOLD ID-Engine|BOLD:ABW6010
Stictochironomus sticticus|To49|Torbjorn Ekrem||BOLD:AAP3558
Stictochironomus sticticus|TRD-CH281|Elisabeth Stur||BOLD:AAP3558
Stictochironomus sticticus|Finnmark202|Torbjorn Ekrem||BOLD:AAP3558
Stictochironomus pictulus|ZMUO.025173|Lauri Paasivirta||BOLD:ADE9888
Stictochironomus pictulus|ZMUO.025172|Lauri Paasivirta||BOLD:ADE9888
Stictochironomus sticticus|ZMUO.024599|Lauri Paasivirta||BOLD:AAM9673
Stictochironomus sticticus|ZMUO.024598|Lauri Paasivirta||BOLD:AAM9673
Stictochironomus sticticus|ZMUO.024316|Lauri Paasivirta||BOLD:AAM9673
Stictochironomus sticticus|ZMUO.024315|Lauri Paasivirta||BOLD:AAM9673
Stictochironomus sp. 3TE|Finnmark51|Elisabeth Stur|Morphology|BOLD:AAM9673
Stictochironomus pictulus|NHRS-BYWS000001113|Yngve Brodin|morphology|BOLD:ACO4514
Stictochironomus maculipennis|ATNA568|Elisabeth Stur||BOLD:ACO4514
Stictochironomus maculipennis|ATNA569|Elisabeth Stur||BOLD:ACO4514
Stictochironomus maculipennis|TRD-CH113|Elisabeth Stur||BOLD:ACO4514
Stictochironomus pictulus|NHRS-BYWS000001114|Yngve Brodin|morphology|BOLD:ACO4514
Stictochironomus maculipennis|TRD-CH321|Elisabeth Stur||BOLD:ACO4514
Stictochironomus maculipennis|TRD-CH322|Elisabeth Stur||BOLD:ACO4514
Stictochironomus maculipennis|TRD-CH95|Elisabeth Stur||BOLD:ACO4514
Stictochironomus rosenschoeldi|ZMUO.024304|Lauri Paasivirta||BOLD:AEM3172
Stictochironomus rosenschoeldi|ZMUO.024303|Lauri Paasivirta||BOLD:AEM3172
Stictochironomus rosenschoeldi|Finnmark653|Torbjorn Ekrem||BOLD:AAV3350
Stictochironomus rosenschoeldi|ZMUO.024596|Lauri Paasivirta||BOLD:AAV3350
Stictochironomus rosenschoeldi|ZMUO.024597|Lauri Paasivirta||BOLD:AAV3350
Stictochironomus rosenschoeldi|Finnmark527|Torbjorn Ekrem||BOLD:AAV3350
Stictochironomus pictulus|ZMUO.024721|Lauri Paasivirta||BOLD:ADA9115
Stictochironomus pictulus|ZMUO.024720|Lauri Paasivirta||BOLD:ADA9115
Stictochironomus sp. 2TE|Finnmark108|Elisabeth Stur||BOLD:AAM6830
Stictochironomus sp. 2TE|Finnmark782|Torbjorn Ekrem||BOLD:AAM6830
Stictochironomus sp. 2TE|Finnmark781|Elisabeth Stur||BOLD:AAM6830
Stictochironomus sp. 2TE|Finnmark385|Torbjorn Ekrem||BOLD:AAM6830
Polypedilum|BIOUG16529-H09|Kate Perez|BOLD ID Engine Manual|BOLD:ACR0701
Polypedilum|BIOUG16529-D06|Kate Perez|BOLD ID Engine Manual|BOLD:ACR0701
Polypedilum convictum|CCDB22605-E04|Sofia Wiedenbrug||BOLD:ACT9278
Polypedilum convictum|ZSM-DIP-33137-G10|Caroline Chimeno|BIN Taxonomy Match (Jan 2022)|BOLD...
Polypedilum convictum|ZMUO.024911|Lauri Paasivirta||BOLD:AAW4661
Polypedilum convictum|ZMUO.024910|Lauri Paasivirta||BOLD:AAW4661
Polypedilum convictum|ZSM-DIP-33134-G08|Caroline Chimeno|BIN Taxonomy Match (Jan 2022)|BOLD...
Polypedilum convictum|BIOUG16529-F11|Kate Perez|BIN Taxonomy Match|BOLD:AAW4661
Polypedilum convictum|BIOUG16529-B04|Kate Perez|BIN Taxonomy Match|BOLD:AAW4661
Polypedilum convictum|TRD-CH404|Elisabeth Stur|morphology|BOLD:AAW4661
Polypedilum convictum|TRD-CH193|Elisabeth Stur|Morphology|BOLD:AAW4661
Polypedilum convictum|BIOUG16270-D10|Kate Perez|BIN Taxonomy Match|BOLD:AAW4661
Polypedilum convictum|EJV-20110156|Lauri Paasivirta||BOLD:AAW4661
Tanytarsus pseudoheusdensis|ES79|Xiaolong Lin|Morphology|BOLD:ACX8267
Tanytarsus pseudoheusdensis|ES77|Xiaolong Lin|Morphology|BOLD:ACX8267
Tanytarsus anderseni|Finnmark110|Elisabeth Stur||BOLD:AAM9625
Tanytarsus anderseni|Finnmark142|Elisabeth Stur||BOLD:AEC7035
Tanytarsus anderseni|Finnmark146|Elisabeth Stur||BOLD:AEC7035
Tanytarsus anderseni|Finnmark380|Elisabeth Stur||BOLD:AEC7035
Polypedilum arundineti|Finnmark170|Torbjorn Ekrem||BOLD:AAP6452
Polypedilum arundineti|NO 86|Elisabeth Stur||BOLD:AAP6452
Polypedilum arundineti|TRD-CH432|Elisabeth Stur|morphology|BOLD:AAP6452
Polypedilum arundineti|CH142|||BOLD:AAP6452
Polypedilum arundineti|ZMUO.024263|Lauri Paasivirta||BOLD:AAP6452
Polypedilum arundineti|ZMUO.024262|Lauri Paasivirta||BOLD:AAP6452
Polypedilum arundineti|CH143|||BOLD:AAP6452
Polypedilum arundineti|TRD-CH433|Elisabeth Stur|morphology|BOLD:AAP6452
Polypedilum arundineti|RIN_CH24|Mona Renate Saursaunet|BOLD Identification engine|BOLD:AAP6452
Polypedilum arundineti|NO 81|Elisabeth Stur||BOLD:AAP6452
Nilothauma brayi|ZMUO.025387|Lauri Paasivirta||BOLD:ADF1066
Nilothauma brayi|ZMUO.025386|Lauri Paasivirta||BOLD:ADF1066
Polypedilum octopunctatum|CCDB21605-H10|Sofia Wiedenbrug||BOLD:ACT2194
Tanytarsus pallidicornis|ZSM-DIP-33145-F11|Caroline Chimeno|BIN Taxonomy Match (Jan 2022)|B...
Tanytarsus pallidicornis|BIOUG55125-E05|Kate Perez|BIN Taxonomy Match (Oct 2020)|BOLD:AC...
Tanytarsus pallidicornis|ZSM-DIP-33138-C11|Caroline Chimeno|BIN Taxonomy Match (Jan 2022)|B...
Tanytarsus pallidicornis|ZSM-DIP-33142-G08|Caroline Chimeno|BIN Taxonomy Match (Jan 2022)|B...
Tanytarsus pallidicornis|ZSM-DIP-33142-G03|Caroline Chimeno|BIN Taxonomy Match (Jan 2022)|B...
Tanytarsus pallidicornis|ZSM-DIP-33129-A08|Caroline Chimeno|BIN Taxonomy Match (Jan 2022)|B...
Tanytarsus pallidicornis|ZSM-DIP-33129-A09|Caroline Chimeno|BIN Taxonomy Match (Jan 2022)|B...
Tanytarsus pallidicornis|ZSM-DIP-33145-B10|Caroline Chimeno|BIN Taxonomy Match (Jan 2022)|B...
Tanytarsus pallidicornis|ZSM-DIP-33145-B11|Caroline Chimeno|BIN Taxonomy Match (Jan 2022)|B...
Tanytarsus pallidicornis|BIOUG36658-B06|Kate Perez|BIN Taxonomy Match (Jul 2018)|BOLD:AC...
Tanytarsus pallidicornis|BIOUG36625-F12|Kate Perez|BIN Taxonomy Match (Jul 2018)|BOLD:AC...
Tanytarsus pallidicornis|BIOUG36680-D03|Kate Perez|BIN Taxonomy Match (May 2018)|BOLD:AC...
Tanytarsus pallidicornis|BIOUG36680-E12|Kate Perez|BIN Taxonomy Match (May 2018)|BOLD:AC...
Tanytarsus pallidicornis|BIOUG36625-D06|Kate Perez|BIN Taxonomy Match (Jul 2018)|BOLD:AC...
Tanytarsus pallidicornis|TRD-CH153|Elisabeth Stur|morphology|BOLD:ACR3318
Tanytarsus pallidicornis|TRD-CH150|Torbjorn Ekrem|morphology|BOLD:ACR3318
Pagastiella orophila|ZMUO.025134|Lauri Paasivirta||BOLD:AAV6386
Pagastiella orophila|ZMUO.025135|Lauri Paasivirta||BOLD:AAV6386
Pagastiella orophila|Finnmark668|Elisabeth Stur||BOLD:AAV6386
Pagastiella orophila|NHRS-BYWS000000405|Yngve Brodin|morphology|BOLD:AAV6386
Pagastiella orophila|Finnmark683|Elisabeth Stur||BOLD:AAV6386
Pagastiella orophila|Finnmark451|Elisabeth Stur|Morphology|BOLD:AAV6386
Pagastiella orophila|Finnmark449|Elisabeth Stur|Morphology|BOLD:AAV6386
Pagastiella orophila|Finnmark348|Elisabeth Stur|Morphology|BOLD:AAV6386
Pagastiella orophila|Finnmark347|Elisabeth Stur|Morphology|BOLD:AAV6386
Pagastiella orophila|TRD-CH62|Elisabeth Stur|Morphology|BOLD:AAV6386
Pagastiella orophila|Finnmark420|Elisabeth Stur||BOLD:AAV6386
Pagastiella orophila|NO 87|Elisabeth Stur||BOLD:AAV6386
Xenochironomus xenolabis|ZMUO.025365|Lauri Paasivirta||BOLD:ACD6724
Cyphomella cornea|ZSM-DIP-33149-F12|Caroline Chimeno|BIN Taxonomy Match (Jan 2022)|BOLD:...
Cyphomella cornea|ZSM-DIP-33147-A06|Paul Hebert|Barcode Match|BOLD:AAI9470
Cyphomella cornea|ZSM-DIP-33147-A04|Paul Hebert|Barcode Match|BOLD:AAI9470
Ablabesmyia monilis|KY225347|||BOLD:AAW4818
Xenochironomus xenolabis|EJV-20110160|Lauri Paasivirta||BOLD:AAW4818
Xenochironomus xenolabis|NHRS-BYWS000000496|Yngve Brodin|morphology|BOLD:AAW4818
Xenochironomus xenolabis|NHRS-BYWS000000938|Yngve Brodin|morphology|BOLD:AAW4818

Xenochironomus xenolabis|EJV-20110160|Lauri Paasivirta||BOLD:AAW4818
Xenochironomus xenolabis|NHRS-BYWS000000496|Yngve Brodin|morphology|BOLD:AAW4818
Xenochironomus xenolabis|NHRS-BYWS000000938|Yngve Brodin|morphology|BOLD:AAW4818
Xenochironomus xenolabis|NHRS-BYWS000000939|Yngve Brodin|morphology|BOLD:AAW4818
Xenochironomus xenolabis|NHRS-BYWS000000960|Yngve Brodin|morphology|BOLD:AAW4818
Xenochironomus xenolabis|ZMUO.025364|Lauri Paasivirta||BOLD:AAW4818
Xenochironomus xenolabis|EJV-20110159|Lauri Paasivirta||BOLD:AAW4818
Kiefferulus tendipediformis|NHRS-BYWS000000315|Yngve Brodin|morphology|BOLD:AAW4677
Kiefferulus tendipediformis|NHRS-BYWS000000316|Yngve Brodin|morphology|BOLD:AAW4677
Kiefferulus tendipediformis|ZSM-DIP-33144-C09|Caroline Chimeno|BIN Taxonomy Match (Jan 2022 ...
Kiefferulus tendipediformis|CH-OSF79|Elisabeth Stur||BOLD:AAW4677
Kiefferulus tendipediformis|NHRS-BYWS000000579|Yngve Brodin|morphology|BOLD:AAW4677
Kiefferulus tendipediformis|NHRS-BYWS000001031|Yngve Brodin|morphology|BOLD:AAW4677
Kiefferulus tendipediformis|ZMUO.025207|Lauri Paasivirta||BOLD:AAW4677
Kiefferulus tendipediformis|NHRS-BYWS000000578|Yngve Brodin|morphology|BOLD:AAW4677
Kiefferulus tendipediformis|EJV-20110025|Lauri Paasivirta||BOLD:AAW4677
Kiefferulus tendipediformis|EJV-20110036|Lauri Paasivirta||BOLD:AAW4677
Kiefferulus tendipediformis|EJV-20110035|Lauri Paasivirta||BOLD:AAW4677
Kiefferulus tendipediformis|EJV-20110027|Lauri Paasivirta||BOLD:AAW4677
Kiefferulus tendipediformis|EJV-20110029|Lauri Paasivirta||BOLD:AAW4677
Kiefferulus tendipediformis|EJV-20110028|Lauri Paasivirta||BOLD:AAW4677
Kiefferulus tendipediformis|EJV-20110026|Lauri Paasivirta||BOLD:AAW4677
Demeijerea rufipes|ZMUO.026188|Lauri Paasivirta||BOLD:ADE9782
Demeijerea rufipes|NHRS-BYWS000001101|Yngve Brodin|morphology|BOLD:ADE9782
Demeijerea rufipes|ZMUO.026187|Lauri Paasivirta||BOLD:ADE9782
Demeijerea rufipes|NHRS-BYWS000001003|Yngve Brodin|morphology|BOLD:ADE9782
Chironominae|CCDB24229-H02|Sofia Wiedenbrug|BIN Taxonomy Match|BOLD:ACU4989
Chironominae|BIOUG05945-E06|Kate Perez|BIN Taxonomy Match|BOLD:ACG4027
Saetheria|EBAI-Ch243|Elisabeth Stur|morphology|BOLD:ADF3948
Saetheria|EBAI-Ch244|Elisabeth Stur|morphology|BOLD:ADF3948
Saetheria|EBAI-Ch242|Elisabeth Stur|morphology|BOLD:ADF3948
Saetheria|EBAI-Ch175|Elisabeth Stur|morphology|BOLD:ADF3948
Saetheria|EBAI-Ch174|Elisabeth Stur|morphology|BOLD:ADF3948
Saetheria tylus|Finnmark733|Torbjorn Ekrem||BOLD:AAV6406
Paracladopelma nigritulum|ZMUO.024718|Lauri Paasivirta||BOLD:ADA5651
Paracladopelma winnelli|Finnmark727|Torbjorn Ekrem||BOLD:AAK4963
Paracladopelma winnelli|Finnmark35|Torbjorn Ekrem||BOLD:AAM6831
Glyptotendipes scirpi|ZMUO.024948|Lauri Paasivirta||BOLD:ACD5799
Glyptotendipes scirpi|ZMUO.024947|Lauri Paasivirta||BOLD:ACD5799
Glyptotendipes imbecillis|NHRS-BYWS000000990|Yngve Brodin|morphology|BOLD:ADK5078
Glyptotendipes imbecillis|NHRS-BYWS000001085|Yngve Brodin|morphology|BOLD:ADK5078
Glyptotendipes imbecillis|ZMUO.026131|Lauri Paasivirta||BOLD:ACD5349
Glyptotendipes imbecillis|ZMUO.026130|Lauri Paasivirta||BOLD:ACD5349
Glyptotendipes imbecillis|NHRS-BYWS000001067|Yngve Brodin|morphology|BOLD:ACD5349
Paracladopelma camptolabis|ZMUO.024638|Lauri Paasivirta||BOLD:ACT5340
Paracladopelma camptolabis|ZSM-DIP-33128-H07|Caroline Chimeno|BIN Taxonomy Match (Jan 2022)...
Paracladopelma|PK-186-33|Petra Kranzfelder|morphology|BOLD:ACT5340
Paracladopelma|PK-186-32|Petra Kranzfelder|morphology|BOLD:ACT5340
Paracladopelma camptolabis|TRD-CH369|Elisabeth Stur|morphology|BOLD:ACT5340
Dicrotendipes nervosus|IM_SSL205V_30|Piotr Gadawski|Morphology|BOLD:AAU1021
Dicrotendipes nervosus|DIC_LOB_IM_SSL205V_30|Piotr Gadawski|Morphology|BOLD:AAU1021
Dicrotendipes nervosus|LA_LSLD5_95|Piotr Gadawski||BOLD:AAU1021
Dicrotendipes nervosus|LA_LSLD1_123|Piotr Gadawski||BOLD:AAU1021
Dicrotendipes nervosus|PU_LSLP7_294|Piotr Gadawski||BOLD:AAU1021
Dicrotendipes nervosus|DIC_NER_IM_VR9X14_39|Piotr Gadawski|Morphology|BOLD:AAU1021
Dicrotendipes nervosus|LA_LSLP5_166|Piotr Gadawski||BOLD:AAU1021
Dicrotendipes nervosus|LA_LSLD5_302|Piotr Gadawski||BOLD:AAU1021
Dicrotendipes nervosus|LA_LSLD1_124|Piotr Gadawski||BOLD:AAU1021
Dicrotendipes nervosus|LA_LSLP8_111|Piotr Gadawski||BOLD:AAU1021
Dicrotendipes nervosus|BIOUG52512-D12|Kate Perez|BIN Taxonomy Match (Mar 2020)|BOLD:AAU1021
Dicrotendipes nervosus|LA_LSLD1_8|Piotr Gadawski||BOLD:AAU1021
Dicrotendipes|ZSM-DIP-33129-A02|Caroline Chimeno|BIN Taxonomy Match (Jan 2022)|BOLD:AAU1021
Dicrotendipes|ZSM-DIP-33128-H08|Caroline Chimeno|BIN Taxonomy Match (Jan 2022)|BOLD:AAU1021
Dicrotendipes|ZSM-DIP-33134-E04|Caroline Chimeno|BIN Taxonomy Match (Jan 2022)|BOLD:AAU1021
Dicrotendipes nervosus|BIOUG36735-D03|Kate Perez|BIN Taxonomy Match (May 2018)|BOLD:AAU1021
Dicrotendipes nervosus|EJV-20110117|Lauri Paasivirta||BOLD:AAU1021
Dicrotendipes nervosus|EJV-20110114|Lauri Paasivirta||BOLD:AAU1021
Dicrotendipes nervosus|BIOUG42780-E07|Kate Perez|BIN Taxonomy Match below Phylum (Jun 2019)...
Dicrotendipes|ZSM-DIP-33170-D04|Caroline Chimeno|BIN Taxonomy Match (Jan 2022)|BOLD:AAU1021
Dicrotendipes nervosus|NHRS-BYWS000000298|Yngve Brodin|morphology|BOLD:AAU1021
Dicrotendipes nervosus|NHRS-BYWS000000126|Yngve Brodin|morphology|BOLD:AAU1021
Dicrotendipes nervosus|NHRS-BYWS000000127|Yngve Brodin|morphology|BOLD:AAU1021
Dicrotendipes nervosus|NHRS-BYWS000001026|Yngve Brodin|morphology|BOLD:AAU1021
Dicrotendipes nervosus|NHRS-BYWS000001875|Yngve Brodin||BOLD:AAU1021
Dicrotendipes nervosus|EJV-20110116|Lauri Paasivirta||BOLD:AAU1021
Dicrotendipes nervosus|NHRS-BYWS000001819|Yngve Brodin||BOLD:AAU1021
Dicrotendipes nervosus|NHRS-BYWS000001792|Yngve Brodin||BOLD:AAU1021
Dicrotendipes nervosus|BIOUG36776-A03|Kate Perez|BIN Taxonomy Match (Jul 2018)|BOLD:AAU1021
Dicrotendipes nervosus|BIOUG36653-C09|Kate Perez|BIN Taxonomy Match (Jul 2018)|BOLD:AAU1021
Dicrotendipes nervosus|EJV-20110115|Lauri Paasivirta||BOLD:AAU1021
Dicrotendipes nervosus|NHRS-BYWS000000447|Yngve Brodin|morphology|BOLD:AAU1021
Dicrotendipes nervosus|NHRS-BYWS000001874|Yngve Brodin||BOLD:AAU1021
Dicrotendipes nervosus|NHRS-BYWS000001848|Yngve Brodin||BOLD:AAU1021
Dicrotendipes nervosus|NHRS-BYWS000001793|Yngve Brodin||BOLD:AAU1021
Dicrotendipes nervosus|NHRS-BYWS000000237|Yngve Brodin|morphology|BOLD:AAU1021
Dicrotendipes nervosus|NHRS-BYWS000000910|Yngve Brodin|morphology|BOLD:AAU1021
Glyptotendipes|ZSM-DIP-33135-A01|Caroline Chimeno|BIN Taxonomy Match (Jan 2022)|BOLD:ACD...
Chironomidae|KY225337
Glyptotendipes nr. paripes|CH152|||BOLD:ACD4470
Glyptotendipes pallens|NHRS-BYWS000001001|Yngve Brodin|morphology|BOLD:ACD4470
Glyptotendipes pallens|NHRS-BYWS000000927|Yngve Brodin|morphology|BOLD:ACD4470
Glyptotendipes pallens|NHRS-BYWS000000245|Yngve Brodin|morphology|BOLD:ACD4470
Glyptotendipes pallens|NHRS-BYWS000000246|Yngve Brodin|morphology|BOLD:ACD4470
Glyptotendipes nr. paripes|ZMUO.024764|Lauri Paasivirta||BOLD:ACD4470
Glyptotendipes pallens|NHRS-BYWS000001002|Yngve Brodin|morphology|BOLD:ACD4470
Glyptotendipes pallens|NHRS-BYWS000000247|Yngve Brodin|morphology|BOLD:ACD4470
Glyptotendipes nr. paripes|ZMUO.024763|Lauri Paasivirta||BOLD:ACD4470
Glyptotendipes pallens|ZMUO.024984|Lauri Paasivirta||BOLD:ACD4470
Glyptotendipes pallens|NHRS-BYWS000000527|Yngve Brodin|morphology|BOLD:ACD4470
Glyptotendipes pallens|ZMUO.024747|Lauri Paasivirta||BOLD:ACD4470
Glyptotendipes pallens|ZMUO.024983|Lauri Paasivirta||BOLD:ACD4470
Glyptotendipes pallens|ZMUO.024746|Lauri Paasivirta||BOLD:ACD4470
Dicrotendipes lobiger|Finnmark442|Torbjorn Ekrem||BOLD:AAM6004

[illegible]
Glyptotendipes pallens|ZMUO.024983|Lauri Paasivirta||BOLD:ACD4470
Glyptotendipes pallens|ZMUO.024746|Lauri Paasivirta||BOLD:ACD4470
Dicrotendipes lobiger|Finnmark442|Torbjorn Ekrem||BOLD:AAM6004
Dicrotendipes lobiger|Finnmark136|Torbjorn Ekrem||BOLD:AAM6004
Dicrotendipes lobiger|DIC_LOB_IM_SSL205V_25|Piotr Gadawski|Morphology|BOLD:AAW4705
Dicrotendipes lobiger|DIC_LOB_IM_PL7VII14_161|Piotr Gadawski|Morphology|BOLD:AAW4705
Dicrotendipes lobiger|ZMUO.024823|Lauri Paasivirta||BOLD:AAW4705
Dicrotendipes lobiger|EJV-20110229|Lauri Paasivirta||BOLD:AAW4705
Dicrotendipes lobiger|EJV-20110228|Lauri Paasivirta||BOLD:AAW4705
Dicrotendipes lobiger|EJV-20110227|Lauri Paasivirta||BOLD:AAW4705
Dicrotendipes lobiger|RIN_CH84|Mona Renate Saursaunet|BOLD Identification engine|BOLD:AAW4705
Dicrotendipes lobiger|BIOUG55153-D03|Renee Miskie|BIN Taxonomy Match (Jan 2021)|BOLD:AAW...
Dicrotendipes lobiger|ZMUO.024824|Lauri Paasivirta||BOLD:AAW4705
Dicrotendipes lobiger|ZSM-DIP-33133-A03|Caroline Chimeno|BIN Taxonomy Match (Jan 2022)|BOLD...
Dicrotendipes lobiger|TRD-CH25|Elisabeth Stur||BOLD:AAW4705
Dicrotendipes lobiger|TRD-CH26|Elisabeth Stur||BOLD:AAW4705
Dicrotendipes lobiger|RIN_CH28|Mona Renate Saursaunet|BOLD Identification engine|BOLD:AAW4705
Dicrotendipes lobiger|TRD-CH27|Elisabeth Stur||BOLD:AAW4705
Dicrotendipes lobiger|RIN_CH89|Mona Renate Saursaunet|BOLD Identification engine|BOLD:AAW4705
Dicrotendipes lobiger|RIN_CH85|Mona Renate Saursaunet|BOLD Identification engine|BOLD:AAW4705
Glyptotendipes aequalis|ZMUO.024751|Lauri Paasivirta||BOLD:ADA6897
Glyptotendipes aequalis|ZMUO.024750|Lauri Paasivirta||BOLD:ADA6897
Demicryptochironomus vulneratus|ZMUO.025107|Lauri Paasivirta||BOLD:ADA8775
Demicryptochironomus vulneratus|ZMUO.025106|Lauri Paasivirta||BOLD:ADA8775
Demicryptochironomus vulneratus|NHRS-BYWS000001115|Yngve Brodin|morphology|BOLD:ADA8775
Demicryptochironomus cf. evgenii|Finnmark730|Torbjorn Ekrem||BOLD:AAL1205
Demicryptochironomus cf. evgenii|Finnmark724|Torbjorn Ekrem||BOLD:AAL1205
Parachironomus siljanensis|NHRS-BYWS000000956|Yngve Brodin|morphology|BOLD:AAP6556
Parachironomus siljanensis|Finnmark222|Torbjorn Ekrem||BOLD:AAP6556
Parachironomus siljanensis|Finnmark261|Torbjorn Ekrem||BOLD:AAP6556
Parachironomus varus|EJV-20110125|Lauri Paasivirta||BOLD:AAP6556
Parachironomus siljanensis|Finnmark221|Torbjorn Ekrem||BOLD:AAP6556
Parachironomus siljanensis|NHRS-BYWS000001812|Torbjorn Ekrem||BOLD:AAP6556
Parachironomus siljanensis|NHRS-BYWS000000955|Yngve Brodin|morphology|BOLD:AAP6556
Parachironomus gracilior|ZSM-DIP-33129-A11|Caroline Chimeno|BIN Taxonomy Match (Jan 2022)|B...
Parachironomus gracilior|NHRS-BYWS000001174|Yngve Brodin|morphology|BOLD:AAX2700
Parachironomus gracilior|ZSM-DIP-33129-A03|Caroline Chimeno|BIN Taxonomy Match (Jan 2022)|B...
Parachironomus gracilior|NHRS-BYWS000000576|Yngve Brodin|morphology|BOLD:AAX2700
Parachironomus|BIOUG36677-G10|Kate Perez|BIN Taxonomy Match (May 2018)|BOLD:AAX2700
Parachironomus gracilior|ZSM-DIP-33129-A10|Caroline Chimeno|BIN Taxonomy Match (Jan 2022)|B...
Parachironomus gracilior|ZMUO.024743|Lauri Paasivirta||BOLD:AAX2700
Parachironomus gracilior|ZMUO.024742|Lauri Paasivirta||BOLD:AAX2700
Parachironomus gracilior|ZSM-DIP-33139-F09|Caroline Chimeno|BIN Taxonomy Match (Jan 2022)|B...
Parachironomus gracilior|NHRS-BYWS000000105|Yngve Brodin|morphology|BOLD:AAX2700
Parachironomus gracilior|BIOUG14950-A11|Kate Perez|BIN Taxonomy Match|BOLD:AAX2700
Parachironomus gracilior|NHRS-BYWS000001043|Yngve Brodin|morphology|BOLD:AAX2700
Parachironomus gracilior|NHRS-BYWS000000209|Yngve Brodin|morphology|BOLD:AAX2700
Parachironomus gracilior|NHRS-BYWS000000210|Yngve Brodin|morphology|BOLD:AAX2700
Parachironomus gracilior|NHRS-BYWS000000103|Yngve Brodin|morphology|BOLD:AAX2700
Parachironomus gracilior|EJV-20110246|Lauri Paasivirta||BOLD:AAX2700
Parachironomus biannulatus|ZMUO.025185|Lauri Paasivirta||BOLD:ADA8425
Parachironomus biannulatus|ZMUO.025184|Lauri Paasivirta||BOLD:ADA8425
Parachironomus varus|PP0266|Lauri Paasivirta||BOLD:ADZ0189
Parachironomus paradigitalis|ZMUO.024885|Lauri Paasivirta||BOLD:ADA6315
Parachironomus paradigitalis|ZMUO.024884|Lauri Paasivirta||BOLD:ADA6315
Parachironomus parilis|ZMUO.025093|Lauri Paasivirta||BOLD:ADA8129
Parachironomus parilis|NHRS-BYWS000000531|Yngve Brodin|morphology|BOLD:AAW4848
Parachironomus parilis|RIN_CH59|Mona Renate Saursaunet|BOLD Identification engine|BOLD:AAW4848
Parachironomus parilis|ZMUO.025092|Lauri Paasivirta||BOLD:AAW4848
Parachironomus parilis|RIN_CH87|Mona Renate Saursaunet|BOLD Identification engine|BOLD:AAW4848
Parachironomus parilis|BIOUG14950-G07|Kate Perez|BIN Taxonomy Match|BOLD:AAW4848
Parachironomus parilis|TRD-CH226|Elisabeth Stur||BOLD:AAW4848
Parachironomus parilis|EJV-20110235|Lauri Paasivirta||BOLD:AAW4848
Parachironomus parilis|EJV-20110234|Lauri Paasivirta||BOLD:AAW4848
Parachironomus parilis|EJV-20110233|Lauri Paasivirta||BOLD:AAW4848
Paracladopelma camptolabis|BIOUG06062-D01|Kate Perez|BIN Taxonomy Match|BOLD:ACG6668
Paracladopelma undine|Finnmark185|Torbjorn Ekrem||BOLD:AAP6209
Paracladopelma undine|BIOUG16198-F03|Kate Perez|BIN Taxonomy Match|BOLD:AAP6209
Cryptochironomus obreptans|ZMUO.026144|Lauri Paasivirta||BOLD:ADE9664
Cryptochironomus albofasciatus|ZMUO.025213|Lauri Paasivirta||BOLD:ADE9664
Cryptochironomus albofasciatus|NHRS-BYWS000001066|Yngve Brodin|morphology|BOLD:ADE9664
Cryptochironomus albofasciatus|NHRS-BYWS000000495|Yngve Brodin|morphology|BOLD:ADK3172
Cryptochironomus albofasciatus|ZMUO.025212|Lauri Paasivirta||BOLD:ADF0941
Cryptochironomus obreptans|ZMUO.026145|Lauri Paasivirta||BOLD:ADF0940
Cryptochironomus supplicans|ZMUO.025357|Lauri Paasivirta||BOLD:ADF0940
Cryptochironomus supplicans|ZMUO.025356|Lauri Paasivirta||BOLD:ADF0940
Cryptochironomus supplicans|NHRS-BYWS000000494|Yngve Brodin|morphology|BOLD:ADF0940
Cryptochironomus supplicans|NHRS-BYWS000000487|Yngve Brodin|morphology|BOLD:ADF0940
Cryptochironomus supplicans|NHRS-BYWS000000348|Yngve Brodin|morphology|BOLD:ADF0940
Cryptochironomus supplicans|NHRS-BYWS000000283|Yngve Brodin|morphology|BOLD:ADF0940
Cryptochironomus supplicans|NHRS-BYWS000000399|Yngve Brodin|morphology|BOLD:AAW4936
Cryptochironomus supplicans|EJV-20110071|Lauri Paasivirta||BOLD:AAW4936
Cryptochironomus obreptans|KY225346|||BOLD:AAW4936
Cryptochironomus supplicans|NHRS-BYWS000000250|Yngve Brodin|morphology|BOLD:AAW4936
Cryptochironomus supplicans|NHRS-BYWS000000102|Yngve Brodin|morphology|BOLD:AAW4936
Cryptochironomus supplicans|EJV-20110068|Lauri Paasivirta||BOLD:AAW4936
Cryptochironomus supplicans|EJV-20110069|Lauri Paasivirta||BOLD:AAW4936
Demicryptochironomus vulneratus|PP0273|Lauri Paasivirta||BOLD:ACQ8800
Chironominae|BIOUG16476-E06|Kate Perez|BOLD ID Engine Manual|BOLD:ACQ8800
Chironominae|BIOUG16433-G07|Kate Perez|BOLD ID Engine Manual|BOLD:ACQ8800
Demicryptochironomus vulneratus|BIOUG16430-A01|Elisabeth Stur|BOLD ID |BOLD:ACQ8800
Chironominae|BIOUG16551-E02|Kate Perez|BOLD ID Engine Manual|BOLD:ACQ8800
Glyptotendipes signatus|NHRS-BYWS000000975|Yngve Brodin|morphology|BOLD:ADA3607
Glyptotendipes signatus|ZMUO.025252|Lauri Paasivirta||BOLD:ADA3607
Glyptotendipes signatus|ZMUO.025251|Lauri Paasivirta||BOLD:ADA3607
Dicrotendipes|CCDB22603-B02|Sofia Wiedenbrug||BOLD:ACQ7103
Dicrotendipes pulsus|CCDB21606-B02|Sofia Wiedenbrug||BOLD:ACT5783
Dicrotendipes pulsus|CCDB21606-B04|Sofia Wiedenbrug||BOLD:ACT5783
Dicrotendipes pulsus|NHRS-BYWS000001822|Yngve Brodin||BOLD:ABZ6609
Dicrotendipes modestus|CH-OSF149|Elisabeth Stur||BOLD:ABZ6609
Dicrotendipes pulsus|NHRS-BYWS000000362|Yngve Brodin|morphology|BOLD:AAM6003
Dicrotendipes pulsus|NHRS-BYWS000000347|Yngve Brodin|morphology|BOLD:AAM6003
[illegible]

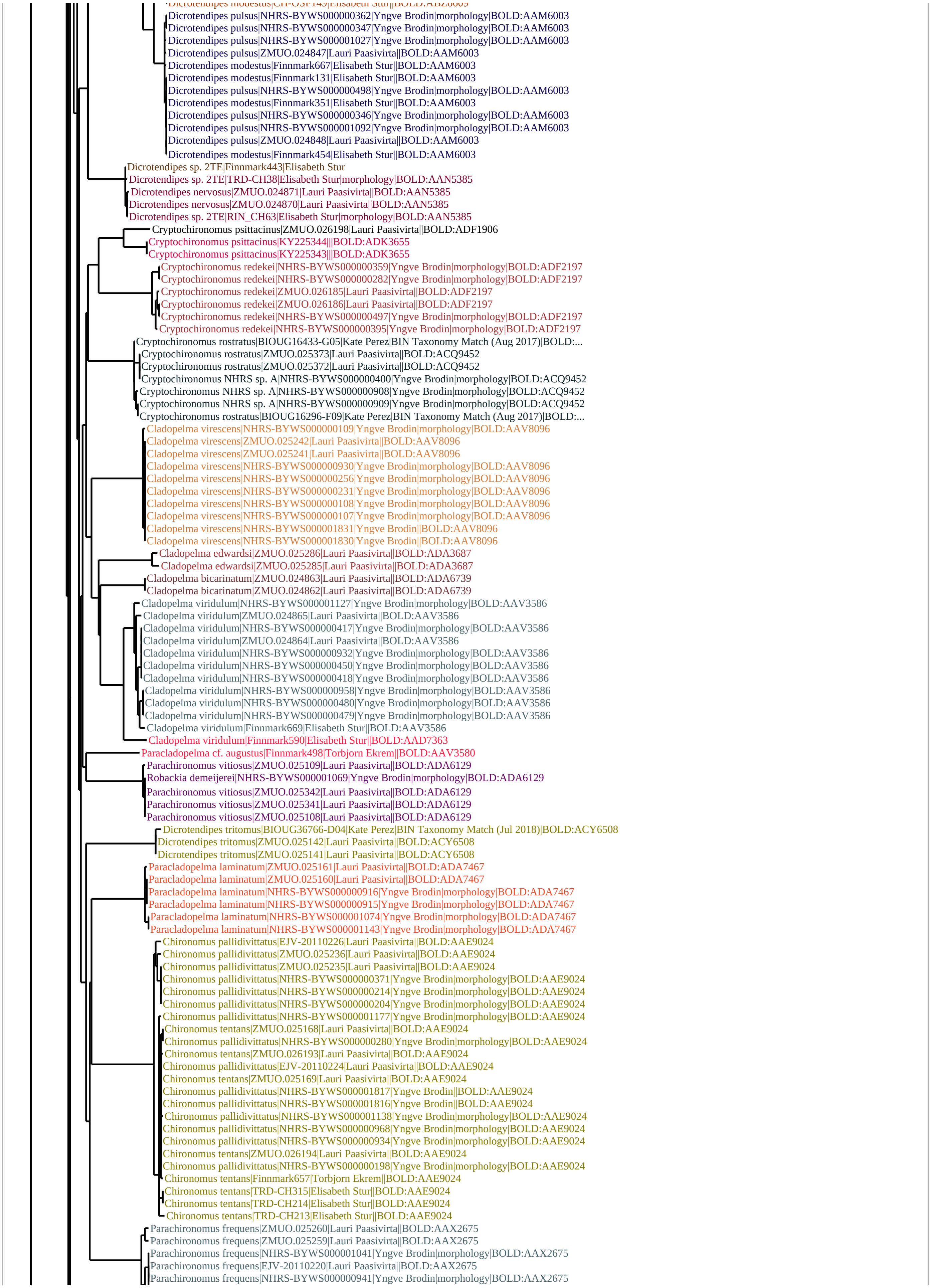
Dicrotendipes modestus|CH-OSF149|Elisabeth Stur||BOLD:ABZ6609
Dicrotendipes pulsus|NHRS-BYWS000000362|Yngve Brodin|morphology|BOLD:AAM6003
Dicrotendipes pulsus|NHRS-BYWS000000347|Yngve Brodin|morphology|BOLD:AAM6003
Dicrotendipes pulsus|NHRS-BYWS000001027|Yngve Brodin|morphology|BOLD:AAM6003
Dicrotendipes pulsus|ZMUO.024847|Lauri Paasivirta||BOLD:AAM6003
Dicrotendipes modestus|Finnmark667|Elisabeth Stur||BOLD:AAM6003
Dicrotendipes modestus|Finnmark131|Elisabeth Stur||BOLD:AAM6003
Dicrotendipes pulsus|NHRS-BYWS000000498|Yngve Brodin|morphology|BOLD:AAM6003
Dicrotendipes modestus|Finnmark351|Elisabeth Stur||BOLD:AAM6003
Dicrotendipes pulsus|NHRS-BYWS000000346|Yngve Brodin|morphology|BOLD:AAM6003
Dicrotendipes pulsus|NHRS-BYWS000001092|Yngve Brodin|morphology|BOLD:AAM6003
Dicrotendipes pulsus|ZMUO.024848|Lauri Paasivirta||BOLD:AAM6003
Dicrotendipes modestus|Finnmark454|Elisabeth Stur||BOLD:AAM6003
Dicrotendipes sp. 2TE|Finnmark443|Elisabeth Stur
Dicrotendipes sp. 2TE|TRD-CH38|Elisabeth Stur|morphology|BOLD:AAN5385
Dicrotendipes nervosus|ZMUO.024871|Lauri Paasivirta||BOLD:AAN5385
Dicrotendipes nervosus|ZMUO.024870|Lauri Paasivirta||BOLD:AAN5385
Dicrotendipes sp. 2TE|RIN_CH63|Elisabeth Stur|morphology|BOLD:AAN5385
Cryptochironomus psittacinus|ZMUO.026198|Lauri Paasivirta||BOLD:ADF1906
Cryptochironomus psittacinus|KY225344|||BOLD:ADK3655
Cryptochironomus psittacinus|KY225343|||BOLD:ADK3655
Cryptochironomus redekei|NHRS-BYWS000000359|Yngve Brodin|morphology|BOLD:ADF2197
Cryptochironomus redekei|NHRS-BYWS000000282|Yngve Brodin|morphology|BOLD:ADF2197
Cryptochironomus redekei|ZMUO.026185|Lauri Paasivirta||BOLD:ADF2197
Cryptochironomus redekei|ZMUO.026186|Lauri Paasivirta||BOLD:ADF2197
Cryptochironomus redekei|NHRS-BYWS000000497|Yngve Brodin|morphology|BOLD:ADF2197
Cryptochironomus redekei|NHRS-BYWS000000395|Yngve Brodin|morphology|BOLD:ADF2197
Cryptochironomus rostratus|BIOUG16433-G05|Kate Perez|BIN Taxonomy Match (Aug 2017)|BOLD:...
Cryptochironomus rostratus|ZMUO.025373|Lauri Paasivirta||BOLD:ACQ9452
Cryptochironomus rostratus|ZMUO.025372|Lauri Paasivirta||BOLD:ACQ9452
Cryptochironomus NHRS sp. A|NHRS-BYWS000000400|Yngve Brodin|morphology|BOLD:ACQ9452
Cryptochironomus NHRS sp. A|NHRS-BYWS000000908|Yngve Brodin|morphology|BOLD:ACQ9452
Cryptochironomus NHRS sp. A|NHRS-BYWS000000909|Yngve Brodin|morphology|BOLD:ACQ9452
Cryptochironomus rostratus|BIOUG16296-F09|Kate Perez|BIN Taxonomy Match (Aug 2017)|BOLD:...
Cladopelma virescens|NHRS-BYWS000000109|Yngve Brodin|morphology|BOLD:AAV8096
Cladopelma virescens|ZMUO.025242|Lauri Paasivirta||BOLD:AAV8096
Cladopelma virescens|ZMUO.025241|Lauri Paasivirta||BOLD:AAV8096
Cladopelma virescens|NHRS-BYWS000000930|Yngve Brodin|morphology|BOLD:AAV8096
Cladopelma virescens|NHRS-BYWS000000256|Yngve Brodin|morphology|BOLD:AAV8096
Cladopelma virescens|NHRS-BYWS000000231|Yngve Brodin|morphology|BOLD:AAV8096
Cladopelma virescens|NHRS-BYWS000000108|Yngve Brodin|morphology|BOLD:AAV8096
Cladopelma virescens|NHRS-BYWS000000107|Yngve Brodin|morphology|BOLD:AAV8096
Cladopelma virescens|NHRS-BYWS000001831|Yngve Brodin||BOLD:AAV8096
Cladopelma virescens|NHRS-BYWS000001830|Yngve Brodin||BOLD:AAV8096
Cladopelma edwardsi|ZMUO.025286|Lauri Paasivirta||BOLD:ADA3687
Cladopelma edwardsi|ZMUO.025285|Lauri Paasivirta||BOLD:ADA3687
Cladopelma bicarinatum|ZMUO.024863|Lauri Paasivirta||BOLD:ADA6739
Cladopelma bicarinatum|ZMUO.024862|Lauri Paasivirta||BOLD:ADA6739
Cladopelma viridulum|NHRS-BYWS000001127|Yngve Brodin|morphology|BOLD:AAV3586
Cladopelma viridulum|ZMUO.024865|Lauri Paasivirta||BOLD:AAV3586
Cladopelma viridulum|NHRS-BYWS000000417|Yngve Brodin|morphology|BOLD:AAV3586
Cladopelma viridulum|ZMUO.024864|Lauri Paasivirta||BOLD:AAV3586
Cladopelma viridulum|NHRS-BYWS000000932|Yngve Brodin|morphology|BOLD:AAV3586
Cladopelma viridulum|NHRS-BYWS000000450|Yngve Brodin|morphology|BOLD:AAV3586
Cladopelma viridulum|NHRS-BYWS000000418|Yngve Brodin|morphology|BOLD:AAV3586
Cladopelma viridulum|NHRS-BYWS000000958|Yngve Brodin|morphology|BOLD:AAV3586
Cladopelma viridulum|NHRS-BYWS000000480|Yngve Brodin|morphology|BOLD:AAV3586
Cladopelma viridulum|NHRS-BYWS000000479|Yngve Brodin|morphology|BOLD:AAV3586
Cladopelma viridulum|Finnmark669|Elisabeth Stur||BOLD:AAV3586
Cladopelma viridulum|Finnmark590|Elisabeth Stur||BOLD:AAD7363
Paracladopelma cf. augustus|Finnmark498|Torbjorn Ekrem||BOLD:AAV3580
Parachironomus vitiosus|ZMUO.025109|Lauri Paasivirta||BOLD:ADA6129
Robackia demeijerei|NHRS-BYWS000001069|Yngve Brodin|morphology|BOLD:ADA6129
Parachironomus vitiosus|ZMUO.025342|Lauri Paasivirta||BOLD:ADA6129
Parachironomus vitiosus|ZMUO.025341|Lauri Paasivirta||BOLD:ADA6129
Parachironomus vitiosus|ZMUO.025108|Lauri Paasivirta||BOLD:ADA6129
Dicrotendipes tritomus|BIOUG36766-D04|Kate Perez|BIN Taxonomy Match (Jul 2018)|BOLD:ACY6508
Dicrotendipes tritomus|ZMUO.025142|Lauri Paasivirta||BOLD:ACY6508
Dicrotendipes tritomus|ZMUO.025141|Lauri Paasivirta||BOLD:ACY6508
Paracladopelma laminatum|ZMUO.025161|Lauri Paasivirta||BOLD:ADA7467
Paracladopelma laminatum|ZMUO.025160|Lauri Paasivirta||BOLD:ADA7467
Paracladopelma laminatum|NHRS-BYWS000000916|Yngve Brodin|morphology|BOLD:ADA7467
Paracladopelma laminatum|NHRS-BYWS000000915|Yngve Brodin|morphology|BOLD:ADA7467
Paracladopelma laminatum|NHRS-BYWS000001074|Yngve Brodin|morphology|BOLD:ADA7467
Paracladopelma laminatum|NHRS-BYWS000001143|Yngve Brodin|morphology|BOLD:ADA7467
Chironomus pallidivittatus|EJV-20110226|Lauri Paasivirta||BOLD:AAE9024
Chironomus pallidivittatus|ZMUO.025236|Lauri Paasivirta||BOLD:AAE9024
Chironomus pallidivittatus|ZMUO.025235|Lauri Paasivirta||BOLD:AAE9024
Chironomus pallidivittatus|NHRS-BYWS000000371|Yngve Brodin|morphology|BOLD:AAE9024
Chironomus pallidivittatus|NHRS-BYWS000000214|Yngve Brodin|morphology|BOLD:AAE9024
Chironomus pallidivittatus|NHRS-BYWS000000204|Yngve Brodin|morphology|BOLD:AAE9024
Chironomus pallidivittatus|NHRS-BYWS000001177|Yngve Brodin|morphology|BOLD:AAE9024
Chironomus tentans|ZMUO.025168|Lauri Paasivirta||BOLD:AAE9024
Chironomus pallidivittatus|NHRS-BYWS000000280|Yngve Brodin|morphology|BOLD:AAE9024
Chironomus tentans|ZMUO.026193|Lauri Paasivirta||BOLD:AAE9024
Chironomus pallidivittatus|EJV-20110224|Lauri Paasivirta||BOLD:AAE9024
Chironomus tentans|ZMUO.025169|Lauri Paasivirta||BOLD:AAE9024
Chironomus pallidivittatus|NHRS-BYWS000001817|Yngve Brodin||BOLD:AAE9024
Chironomus pallidivittatus|NHRS-BYWS000001816|Yngve Brodin||BOLD:AAE9024
Chironomus pallidivittatus|NHRS-BYWS000001138|Yngve Brodin|morphology|BOLD:AAE9024
Chironomus pallidivittatus|NHRS-BYWS000000968|Yngve Brodin|morphology|BOLD:AAE9024
Chironomus pallidivittatus|NHRS-BYWS000000934|Yngve Brodin|morphology|BOLD:AAE9024
Chironomus tentans|ZMUO.026194|Lauri Paasivirta||BOLD:AAE9024
Chironomus pallidivittatus|NHRS-BYWS000000198|Yngve Brodin|morphology|BOLD:AAE9024
Chironomus tentans|Finnmark657|Torbjorn Ekrem||BOLD:AAE9024
Chironomus tentans|TRD-CH315|Elisabeth Stur||BOLD:AAE9024
Chironomus tentans|TRD-CH214|Elisabeth Stur||BOLD:AAE9024
Chironomus tentans|TRD-CH213|Elisabeth Stur||BOLD:AAE9024
Parachironomus frequens|ZMUO.025260|Lauri Paasivirta||BOLD:AAX2675
Parachironomus frequens|ZMUO.025259|Lauri Paasivirta||BOLD:AAX2675
Parachironomus frequens|NHRS-BYWS000001041|Yngve Brodin|morphology|BOLD:AAX2675
Parachironomus frequens|EJV-20110220|Lauri Paasivirta||BOLD:AAX2675
Parachironomus frequens|NHRS-BYWS000000941|Yngve Brodin|morphology|BOLD:AAX2675

Parachironomus frequens|NHRS-BYWS000001041|Yngve Brodin|morphology|BOLD:AAX2675
Parachironomus frequens|EJV-20110220|Lauri Paasivirta||BOLD:AAX2675
Parachironomus frequens|NHRS-BYWS000000941|Yngve Brodin|morphology|BOLD:AAX2675
Parachironomus frequens|ZSM-DIP-33139-F07|Caroline Chimeno|BIN Taxonomy Match (Jan 2022)|BO...
Parachironomus frequens|NHRS-BYWS000001040|Yngve Brodin|morphology|BOLD:AAX2675
Cricotopus pilicauda|ZMUO.026157|Lauri Paasivirta||BOLD:ACR2474
Paracladopelma|BIOUG17224-F05|Valerie Levesque-Beaudin|Digital Morphology|BOLD:ACR2474
Cryptotendipes pflugfelderi|ZMUO.026234|Lauri Paasivirta||BOLD:ADF1721
Harnischia curtilamellata|ZMUO.024934|Lauri Paasivirta||BOLD:AAY2074
Harnischia curtilamellata|ZSM-DIP-33149-B08|Caroline Chimeno|BIN Taxonomy Match (Jan 2022)|...
Harnischia curtilamellata|ZMUO.024933|Lauri Paasivirta||BOLD:AAY2074
Harnischia curtilamellata|ZSM-DIP-33133-A04|Caroline Chimeno|BIN Taxonomy Match (Jan 2022)|...
Harnischia curtilamellata|ZSM-DIP-33133-A05|Caroline Chimeno|BIN Taxonomy Match (Jan 2022)|...
Harnischia curtilamellata|ZSM-DIP-33135-A04|Caroline Chimeno|BIN Taxonomy Match (Jan 2022)|...
Harnischia curtilamellata|ZSM-DIP-33133-G01|Caroline Chimeno|BIN Taxonomy Match (Jan 2022)|...
Harnischia curtilamellata|EJV-20110217|Lauri Paasivirta||BOLD:AAY2074
Microchironomus tener|NHRS-BYWS000000929|Yngve Brodin|morphology|BOLD:AAX4146
Microchironomus tener|ZMUO.025225|Lauri Paasivirta||BOLD:AAX4146
Microchironomus tener|ZMUO.025224|Lauri Paasivirta||BOLD:AAX4146
Microchironomus tener|NHRS-BYWS000000191|Yngve Brodin|morphology|BOLD:AAX4146
Microchironomus tener|NHRS-BYWS000000207|Yngve Brodin|morphology|BOLD:AAX4146
Microchironomus tener|NHRS-BYWS000000106|Yngve Brodin|morphology|BOLD:AAX4146
Microchironomus tener|NHRS-BYWS000000255|Yngve Brodin|morphology|BOLD:AAX4146
Microchironomus tener|NHRS-BYWS000000104|Yngve Brodin|morphology|BOLD:AAX4146
Microchironomus tener|NHRS-BYWS000000224|Yngve Brodin|morphology|BOLD:AAX4146
Microchironomus tener|NHRS-BYWS000001847|Yngve Brodin||BOLD:AAX4146
Paracladopelma|CCDB22605E03|Sofia Wiedenbrug||BOLD:ACT9115
Paracladopelma|ZSM-DIP-33129-F04|Caroline Chimeno|BIN Taxonomy Match (Jan 2022)|BOLD:ACT...
Paracladopelma|ZSM-DIP-33149-G12|Caroline Chimeno|BIN Taxonomy Match (Jan 2022)|BOLD:ACT...
Paracladopelma|ZSM-DIP-33147-D08|Caroline Chimeno|BIN Taxonomy Match (Jan 2022)|BOLD:ACT...
Paracladopelma|ZSM-DIP-33137-G02|Caroline Chimeno|BIN Taxonomy Match (Jan 2022)|BOLD:ACT...
Paracladopelma|ZSM-DIP-33144-E06|Caroline Chimeno|BIN Taxonomy Match (Jan 2022)|BOLD:ACT...
Paracladopelma|ZSM-DIP-33146-E04|Caroline Chimeno|BIN Taxonomy Match (Jan 2022)|BOLD:ACT...
Paracladopelma|ZSM-DIP-33147-C04|Caroline Chimeno|BIN Taxonomy Match (Jan 2022)|BOLD:ACT...
Paracladopelma|ZSM-DIP-33147-D11|Caroline Chimeno|BIN Taxonomy Match (Jan 2022)|BOLD:ACT...
Paracladopelma|CCDB22605-E02|Sofia Wiedenbrug||BOLD:ACT9115
Paracladopelma|BIOUG55103-F04|Stefan Schmidt|BIN Taxonomy Match (Sep 2020)|BOLD:ACT9115
Paracladopelma|BIOUG42719-C09|Kate Perez|BIN Taxonomy Match below Phylum (Jun 2019)|BOLD ...
Paracladopelma|BIOUG55197-G04|Stefan Schmidt|BIN Taxonomy Match (Sep 2020)|BOLD:ACT9115
Dicrotendipes notatus|ZMUO.025240|Lauri Paasivirta||BOLD:ADA4878
Dicrotendipes notatus|ZMUO.025239|Lauri Paasivirta||BOLD:ADA4878
Dicrotendipes pulsus|DIC_PUL_IM_SSL205V_37|Piotr Gadawski|Morphology|BOLD:ADA4878
Dicrotendipes pulsus|DIC_LOB_IM_PL7V14_122|Piotr Gadawski|Morphology|BOLD:ADA4878
Dicrotendipes pulsus|DIC_PUL_IM_PL7V14_116|Piotr Gadawski|Morphology|BOLD:ADA4878
Dicrotendipes|IM_SKD2_30|Piotr Gadawski|Morphology|BOLD:ADA4878
Dicrotendipes pulsus|BIOUG52512-E09|Kate Perez|BIN Taxonomy Match (Mar 2020)|BOLD:ADA4878
Parachironomus subalpinus|TRD-CH157|Elisabeth Stur|morphology|BOLD:AAP6557
Parachironomus subalpinus|Finnmark225|Torbjorn Ekrem||BOLD:AAP6557
Parachironomus subalpinus|Finnmark224|Torbjorn Ekrem||BOLD:AAP6557
Parachironomus subalpinus|ZMUO.025159|Lauri Paasivirta||BOLD:AAP6557
Parachironomus tenuicaudatus|BIOUG36762-D08|Kate Perez|BIN Taxonomy Match (May 2018)|BOLD ...
Parachironomus tenuicaudatus|ZMUO.025379|Lauri Paasivirta||BOLD:ACE3297
Parachironomus tenuicaudatus|ZMUO.025378|Lauri Paasivirta||BOLD:ACE3297
Parachironomus tenuicaudatus|CH-eik19|Elisabeth Stur||BOLD:ACE3297
Chironomus transvaalensis|LA_LSLRZ9_227|Piotr Gadawski|Morphology|BOLD:AAW3995
Chironomus transvaalensis|LA_LSLRZ9_98|Piotr Gadawski||BOLD:AAW3995
Chironomus transvaalensis|LA_LSLRZ9_232|Piotr Gadawski|Morphology|BOLD:AAW3995
Chironomus transvaalensis|LA_LSLRZ9_224|Piotr Gadawski|Morphology|BOLD:AAW3995
Chironomus transvaalensis|LA_LSLRZ9_59|Piotr Gadawski||BOLD:AAW3995
Chironomus transvaalensis|LA_LSLRZ9_100|Piotr Gadawski||BOLD:AAW3995
Chironomus|ZSM-DIP-33128-C01|Caroline Chimeno|BIN Taxonomy Match (Jan 2022)|BOLD:ACO0599
Chironomus|ZSM-DIP-33145-B07|Caroline Chimeno|BIN Taxonomy Match (Jan 2022)|BOLD:ACO0599
Chironomus|ZSM-DIP-33147-F07|Caroline Chimeno|BIN Taxonomy Match (Jan 2022)|BOLD:ACO0599
Chironomus|ZSM-DIP-33140-H11|Caroline Chimeno|BIN Taxonomy Match (Jan 2022)|BOLD:ACO0599
Chironomus|ZSM-DIP-33144-C08|Caroline Chimeno|BIN Taxonomy Match (Jan 2022)|BOLD:ACO0599
Chironomus|ZSM-DIP-33144-G10|Caroline Chimeno|BIN Taxonomy Match (Jan 2022)|BOLD:ACO0599
Chironomus|ZSM-DIP-33129-F07|Caroline Chimeno|BIN Taxonomy Match (Jan 2022)|BOLD:ACO0599
Chironomus|ZSM-DIP-33139-A01|Caroline Chimeno|BIN Taxonomy Match (Jan 2022)|BOLD:ACO0599
Chironomus|ZSM-DIP-33138-B12|Caroline Chimeno|BIN Taxonomy Match (Jan 2022)|BOLD:ACO0599
Chironomus|ZSM-DIP-33138-B10|Caroline Chimeno|BIN Taxonomy Match (Jan 2022)|BOLD:ACO0599
Chironomus|ZSM-DIP-33137-G01|Caroline Chimeno|BIN Taxonomy Match (Jan 2022)|BOLD:ACO0599
Chironomus|ZSM-DIP-33135-A05|Caroline Chimeno|BIN Taxonomy Match (Jan 2022)|BOLD:ACO0599
Chironomus|ZSM-DIP-33142-G04|Caroline Chimeno|BIN Taxonomy Match (Jan 2022)|BOLD:ACO0599
Chironomus|ZSM-DIP-33133-F12|Caroline Chimeno|BIN Taxonomy Match (Jan 2022)
Cryptotendipes usmaensis|ZMUO.025254|Lauri Paasivirta||BOLD:ADA4237
Cryptotendipes usmaensis|ZMUO.025253|Lauri Paasivirta||BOLD:ADA4237
Cryptotendipes usmaensis|ZSM-DIP-33133-G07|Caroline Chimeno|BIN Taxonomy Match (Jan 2022)|B...
Cryptotendipes usmaensis|ZSM-DIP-33133-A08|Caroline Chimeno|BIN Taxonomy Match (Jan 2022)|B...
Cryptotendipes usmaensis|ZSM-DIP-33133-G06|Caroline Chimeno|BIN Taxonomy Match (Jan 2022)|B...
Chironomus|ZMUO.025350|Lauri Paasivirta||BOLD:ADF0267
Chironomus|ZMUO.025349|Lauri Paasivirta||BOLD:ADF0267
Glyptotendipes cauliginellus|ZMUO.024247|Lauri Paasivirta||BOLD:ACE3296
Glyptotendipes cauliginellus|CH-eik21|Elisabeth Stur||BOLD:ACE3296
Glyptotendipes cauliginellus|CH-eik20|Elisabeth Stur||BOLD:ACE3296
Glyptotendipes cauliginellus|CH-eik125|Elisabeth Stur||BOLD:ACE3296
Glyptotendipes cauliginellus|CH-eik53|Elisabeth Stur||BOLD:ACE3296
Glyptotendipes cauliginellus|CH-eik23|Elisabeth Stur||BOLD:ACE3296
Chironomus mendax|ZSM-DIP-33145-E05|Caroline Chimeno|BIN Taxonomy Match (Jan 2022)|BOLD:...
Chironomus mendax|BIOUG15951-F08|Kate Perez|BIN Taxonomy Match (Aug 2017)|BOLD:ACQ8317
Chironomus mendax|ZMUO.025125|Lauri Paasivirta||BOLD:ACQ8317
Chironomus mendax|ZMUO.025124|Lauri Paasivirta||BOLD:ACQ8317
Chironomus storai|ZMUO.026020|Lauri Paasivirta||BOLD:ACR8183
Chironomus storai|ZMUO.024868|Lauri Paasivirta||BOLD:ACR8183
Chironomus storai|Finnmark662|Torbjorn Ekrem||BOLD:AAV3584
Chironomus storai|ZMUO.026021|Lauri Paasivirta||BOLD:AAV3584
Chironomus storai|Finnmark453|Torbjorn Ekrem||BOLD:AAV3584
Chironomus|Ya6-06187La1_11M|Henk Valenduuk||BOLD:ACG9844
Chironomus macani|CH-eik126|Elisabeth Stur||BOLD:ACG9844
Chironomus macani|ZMUO.025395|Lauri Paasivirta||BOLD:ACG9844
Fleuria lacustris|GBOL00762|Martin Spies|morpho-taxonomy|BOLD:ACB2983
Fleuria lacustris|GBOL00003|Martin Spies|morpho-taxonomy|BOLD:ACB2983
Benthalia carbonaria|ZMUO.025322|Lauri Paasivirta||BOLD:AAW3454
Benthalia carbonaria|LA_LSLRZ17_42|Piotr Gadawski||BOLD:ADF1214

Fleuria lacustris|GBOL00003|Martin Spies|morpho-taxonomy|BOLD:ACB2983
Benthalia carbonaria|ZMUO.025322|Lauri Paasivirta||BOLD:AAW3454
Benthalia carbonaria|LA_LSLRZ17_42|Piotr Gadawski||BOLD:ADF1214
Benthalia carbonaria|LA_LSLD1_204|Piotr Gadawski||BOLD:ADF1214
Benthalia carbonaria|LA_LSLD3_283|Piotr Gadawski||BOLD:ADF1214
Benthalia carbonaria|LA_LSLD1_259|Piotr Gadawski||BOLD:ADF1214
Benthalia carbonaria|LA_LSLZ14_21|Piotr Gadawski||BOLD:ADF1214
Benthalia carbonaria|LA_LSLD1_141|Piotr Gadawski||BOLD:ADF1214
Benthalia carbonaria|LA_LSLD1_256|Piotr Gadawski||BOLD:ADF1214
Benthalia carbonaria|ZMUO.025321|Lauri Paasivirta||BOLD:ADF1214
Benthalia carbonaria|LA_LSLD1_257|Piotr Gadawski||BOLD:ADF1214
Benthalia carbonaria|LA_LSLZ14_22|Piotr Gadawski||BOLD:ADF1214
Benthalia carbonaria|LA_LSLD1_142|Piotr Gadawski||BOLD:ADF1214
Benthalia carbonaria|LA_LSLD1_255|Piotr Gadawski||BOLD:ADF1214
Benthalia carbonaria|BIOUG42676-E08|Kate Perez|BIN Taxonomy Match below Phylum (Jul 2019)|B...
Benthalia dissidens|mes11|Henk Valenduuk||BOLD:ADF1214
Chironomus salinarius|NO70|Elisabeth Stur
Chironomus salinarius|NO72|Elisabeth Stur
Chironomus salinarius|NHRS-BYWS000001783|Yngve Brodin||BOLD:AAN5317
Chironomus salinarius|NHRS-BYWS000001146|Yngve Brodin|morphology|BOLD:AAN5317
Chironomus salinarius|NHRS-BYWS000001782|Yngve Brodin||BOLD:AAN5317
Chironomus|ZSM-DIP-33134-G06|Caroline Chimeno|BIN Taxonomy Match (Jan 2022)|BOLD:ACG9999
Chironomus|ZSM-DIP-33147-D10|Caroline Chimeno|BIN Taxonomy Match (Jan 2022)|BOLD:ACG9999
Chironomus|BIOUG42727-H03|Kate Perez|BIN Taxonomy Match below Phylum (Jun 2019)|BOLD:ACG...
Chironomus|ZSM-DIP-33129-F05|Caroline Chimeno|BIN Taxonomy Match (Jan 2022)|BOLD:ACG9999
Chironomus|ZSM-DIP-33170-D03|Caroline Chimeno|BIN Taxonomy Match (Jan 2022)|BOLD:ACG9999
Chironomus|ZSM-DIP-33146-E03|Caroline Chimeno|BIN Taxonomy Match (Jan 2022)|BOLD:ACG9999
Chironomus|ZSM-DIP-33170-D01|Caroline Chimeno|BIN Taxonomy Match (Jan 2022)|BOLD:ACG9999
Chironomus dorsalis|ZMUO.025090|Lauri Paasivirta||BOLD:ACG9999
Chironomus dorsalis|ZMUO.025091|Lauri Paasivirta||BOLD:ACG9999
Chironomus pseudomendax|RIN_CH86|Elisabeth Stur|morphology|BOLD:ACG9999
Chironomus pseudomendax|CH-eik127|Elisabeth Stur||BOLD:ACG9999
Chironomus|ZSM-DIP-33149-E03|Caroline Chimeno|BIN Taxonomy Match (Jan 2022)|BOLD:ACG9999
Chironomus|ZSM-DIP-33145-H05|Caroline Chimeno|BIN Taxonomy Match (Jan 2022)|BOLD:ACG9999
Chironomus|BIOUG42691-B03|Kate Perez|BIN Taxonomy Match below Phylum (Jun 2019)|BOLD:ACG...
Chironomus storai|ZMUO.024869|Lauri Paasivirta||BOLD:ADA6298
Chironomus cf. mendax|CH-OSF96|Torbjorn Ekrem||BOLD:AAP5147
Chironomus cf. mendax|TRD-CH423|Elisabeth Stur|morphology|BOLD:AAP5147
Chironomus cf. mendax|TRD-CH298|Elisabeth Stur||BOLD:AAP5147
Chironomus cf. mendax|TRD-CH229|Elisabeth Stur||BOLD:AAP5147
Chironomus sp. 7TE|CH-OSF48|Torbjorn Ekrem||BOLD:AAW0807
Chironomus nr. pseudothummi|ZMUO.024913|Lauri Paasivirta||BOLD:AAW0807
Chironomus nr. pseudothummi|ZMUO.024912|Lauri Paasivirta||BOLD:AAW0807
Chironomus nr. piger|ZMUO.025234|Lauri Paasivirta||BOLD:AER6112
Chironomus nr. pseudothummi|ZMUO.025190|Lauri Paasivirta||BOLD:AER6112
Chironomus nr. pseudothummi|ZMUO.024540|Lauri Paasivirta||BOLD:AER6112
Chironomus pseudothummi|LA_LSLRZ9_240|Piotr Gadawski|Morphology|BOLD:AER6112
Chironomus pseudothummi|LA_LSLRZ9_237|Piotr Gadawski|Morphology|BOLD:AER6112
Chironomus|ZSM-DIP-33141-D06|Caroline Chimeno|BIN Taxonomy Match (Jan 2022)|BOLD:AAU4044
Chironomus nr. pseudothummi|ZMUO.024814|Lauri Paasivirta||BOLD:AAU4044
Chironomus nr. pseudothummi|ZMUO.025191|Lauri Paasivirta||BOLD:AAU4044
Chironomus pseudothummi|NHRS-BYWS000000991|Yngve Brodin|morphology|BOLD:AAU4044
Chironomus|ZMUO.026116|Lauri Paasivirta||BOLD:AAU4044
Chironomus pseudothummi-agg.2|EJV-20110240|Lauri Paasivirta||BOLD:AAU4044
Chironomus pseudothummi|NHRS-BYWS000000409|Yngve Brodin|morphology|BOLD:AAU4044
Chironomus pseudothummi|NHRS-BYWS000000314|Yngve Brodin|morphology|BOLD:AAU4044
Chironomus pseudothummi-agg.1|EJV-20110003|Lauri Paasivirta||BOLD:AAU4044
Chironomus pseudothummi-agg.1|EJV-20110002|Lauri Paasivirta||BOLD:AAU4044
Chironomus pseudothummi|NHRS-BYWS000000931|Yngve Brodin|morphology|BOLD:AAU4044
Chironomus pseudothummi|NHRS-BYWS000000545|Yngve Brodin|morphology|BOLD:AAU4044
Chironomus pseudothummi|NHRS-BYWS000000981|Yngve Brodin|morphology|BOLD:AAU4044
Chironomus pseudothummi|NHRS-BYWS000000970|Yngve Brodin|morphology|BOLD:AAU4044
Chironomus pseudothummi|NHRS-BYWS000000313|Yngve Brodin|morphology|BOLD:AAU4044
Chironomus pseudothummi|NHRS-BYWS000000982|Yngve Brodin|morphology|BOLD:AAU4044
Chironomus pseudothummi-agg.2|EJV-20110239|Lauri Paasivirta||BOLD:AAU4044
Chironomus pseudothummi-agg.2|EJV-20110243|Lauri Paasivirta||BOLD:AAU4044
Chironomus pseudothummi|NHRS-BYWS000001062|Yngve Brodin|morphology|BOLD:AAU4044
Chironomus|ZMUO.026117|Lauri Paasivirta||BOLD:AAU4044
Chironomus pseudothummi|NHRS-BYWS000001142|Yngve Brodin|morphology|BOLD:AAU4044
Chironomus pseudothummi|NHRS-BYWS000001860|Yngve Brodin||BOLD:AAU4044
Chironomus pseudothummi|NHRS-BYWS000001865|Yngve Brodin||BOLD:AAU4044
Chironomus pseudothummi-agg.1|EJV-20110009|Lauri Paasivirta||BOLD:AAU4044
Chironomus pseudothummi-agg.1|EJV-20110007|Lauri Paasivirta||BOLD:AAU4044
Chironomus pseudothummi-agg.1|EJV-20110010|Lauri Paasivirta||BOLD:AAU4044
Chironomus pseudothummi-agg.2|EJV-20110242|Lauri Paasivirta||BOLD:AAU4044
Chironomus pseudothummi|NHRS-BYWS000001033|Yngve Brodin|morphology|BOLD:AAU4044
Chironomus pseudothummi-agg.2|EJV-20110241|Lauri Paasivirta||BOLD:AAU4044
Chironomus pseudothummi-agg.1|EJV-20110008|Lauri Paasivirta||BOLD:AAU4044
Chironomus pseudothummi-agg.1|EJV-20110005|Lauri Paasivirta||BOLD:AAU4044
Chironomus pseudothummi-agg.1|EJV-20110004|Lauri Paasivirta||BOLD:AAU4044
Chironomus pseudothummi-agg.1|EJV-20110001|Lauri Paasivirta||BOLD:AAU4044
Chironomus riparius|LN894552|||BOLD:AAA7263
Chironomus riparius|LN894371|||BOLD:AAA7263
Chironomus riparius|LN894358|||BOLD:AAA7263
Chironomus riparius|LN894359|||BOLD:AAA7263
Chironomus riparius|LN894369|||BOLD:AAA7263
Chironomus riparius|LN894289|||BOLD:AAA7263
Chironomus nr. sollicitus|ZMUO.025402|Lauri Paasivirta||BOLD:AAA7263
Chironomus nr. sollicitus|ZMUO.024543|Lauri Paasivirta||BOLD:AAA7263
Chironomus riparius|LN894215|||BOLD:AAA7263
Chironomus riparius|LN894503|||BOLD:AAA7263
Chironomus riparius|ZSM-DIP-33144-G11|Caroline Chimeno|BIN Taxonomy Match (Jan 2022)|BOLD ...
Chironomus riparius|LN894313|||BOLD:AAA7263
Chironomus riparius|LN894316|||BOLD:AAA7263
Chironomus riparius|LN894300|||BOLD:AAA7263
Chironomus riparius|LN894220|||BOLD:AAA7263
Chironomus riparius|LN894265|||BOLD:AAA7263
Chironomus riparius|LN894283|||BOLD:AAA7263
Chironomus riparius|LN894247|||BOLD:AAA7263
Chironomus riparius|LN894290|||BOLD:AAA7263
Chironomus riparius|LN894339|||BOLD:AAA7263
Chironomus riparius|LN894276|||BOLD:AAA7263
Chironomus riparius|LN894534|||BOLD:AAA7263

[illegible]
Chironomus riparius|LN894339|||BOLD:AAA7263
Chironomus riparius|LN894276|||BOLD:AAA7263
Chironomus riparius|LN894534|||BOLD:AAA7263
Chironomus riparius|LN894268|||BOLD:AAA7263
Chironomus riparius|LN894259|||BOLD:AAA7263
Chironomus riparius|LN894336|||BOLD:AAA7263
Chironomus riparius|LN894291|||BOLD:AAA7263
Chironomus riparius|LN894277|||BOLD:AAA7263
Chironomus riparius|LN894349|||BOLD:AAA7263
Chironomus riparius|LN894547|||BOLD:AAA7263
Chironomus riparius|LN894548|||BOLD:AAA7263
Chironomus riparius|LN894515|||BOLD:AAA7263
Chironomus riparius|LN894345|||BOLD:AAA7263
Chironomus riparius|LN894517|||BOLD:AAA7263
Chironomus riparius|LN894502|||BOLD:AAA7263
Chironomus riparius|LN894232|||BOLD:AAA7263
Chironomus riparius|LN894527|||BOLD:AAA7263
Chironomus riparius|LN894526|||BOLD:AAA7263
Chironomus riparius|LN894227|||BOLD:AAA7263
Chironomus riparius|LN894518|||BOLD:AAA7263
Chironomus riparius|LN894356|||BOLD:AAA7263
Chironomus riparius|LN894248|||BOLD:AAA7263
Chironomus riparius|LA_LSLRZ9_238|Piotr Gadawski|Morphology|BOLD:AAA7263
Chironomus riparius|LN894546|||BOLD:AAA7263
Chironomus riparius|LN894522|||BOLD:AAA7263
Chironomus riparius|LN894520|||BOLD:AAA7263
Chironomus riparius|LN894535|||BOLD:AAA7263
Chironomus riparius|LN894528|||BOLD:AAA7263
Chironomus riparius|LN894530|||BOLD:AAA7263
Chironomus riparius|LN894536|||BOLD:AAA7263
Chironomus riparius|LN894544|||BOLD:AAA7263
Chironomus riparius|LN894543|||BOLD:AAA7263
Chironomus riparius|LN894531|||BOLD:AAA7263
Chironomus riparius|LN894523|||BOLD:AAA7263
Chironomus riparius|LN894533|||BOLD:AAA7263
Chironomus riparius|LN894506|||BOLD:AAA7263
Chironomus riparius|LN894524|||BOLD:AAA7263
Chironomus riparius|LN894538|||BOLD:AAA7263
Chironomus riparius|LN894508|||BOLD:AAA7263
Chironomus riparius|LN894551|||BOLD:AAA7263
Chironomus riparius|LN894519|||BOLD:AAA7263
Chironomus riparius|LN894529|||BOLD:AAA7263
Chironomus riparius|LN894507|||BOLD:AAA7263
Chironomus riparius|LN894513|||BOLD:AAA7263
Chironomus riparius|LN894511|||BOLD:AAA7263
Chironomus riparius|LN894509|||BOLD:AAA7263
Chironomus riparius|LN894504|||BOLD:AAA7263
Chironomus riparius|LN894521|||BOLD:AAA7263
Chironomus riparius|LN894505|||BOLD:AAA7263
Chironomus riparius|LN894540|||BOLD:AAA7263
Chironomus riparius|LN894217|||BOLD:AAA7263
Chironomus riparius|LN894204|||BOLD:AAA7263
Chironomus riparius|LN894311|||BOLD:AAA7263
Chironomus riparius|LN894229|||BOLD:AAA7263
Chironomus riparius|LN894199|||BOLD:AAA7263
Chironomus riparius|LN894223|||BOLD:AAA7263
Chironomus riparius|LN894252|||BOLD:AAA7263
Chironomus riparius|LN894350|||BOLD:AAA7263
Chironomus riparius|LN894271|||BOLD:AAA7263
Chironomus riparius|LN894272|||BOLD:AAA7263
Chironomus riparius|LN894282|||BOLD:AAA7263
Chironomus riparius|LN894304|||BOLD:AAA7263
Chironomus riparius|LN894317|||BOLD:AAA7263
Chironomus riparius|LN894318|||BOLD:AAA7263
Chironomus riparius|LN894340|||BOLD:AAA7263
Chironomus riparius|LN894308|||BOLD:AAA7263
Chironomus riparius|LN894342|||BOLD:AAA7263
Chironomus riparius|LN894210|||BOLD:AAA7263
Chironomus riparius|LN894487|||BOLD:AAA7263
Chironomus riparius|LN894312|||BOLD:AAA7263
Chironomus riparius|LN894233|||BOLD:AAA7263
Chironomus riparius|LN894305|||BOLD:AAA7263
Chironomus riparius|LN894203|||BOLD:AAA7263
Chironomus riparius|LN894542|||BOLD:AAA7263
Chironomus riparius|LN894510|||BOLD:AAA7263
Chironomus riparius|LN894525|||BOLD:AAA7263
Chironomus riparius|LN894539|||BOLD:AAA7263
Chironomus riparius|LN894545|||BOLD:AAA7263
Chironomus riparius|LN894337|||BOLD:AAA7263
Chironomus riparius|LN894333|||BOLD:AAA7263
Chironomus riparius|LN894261|||BOLD:AAA7263
Chironomus riparius|LN894246|||BOLD:AAA7263
Chironomus riparius|LN894328|||BOLD:AAA7263
Chironomus riparius|LN894208|||BOLD:AAA7263
Chironomus riparius|LN894224|||BOLD:AAA7263
Chironomus riparius|LN894255|||BOLD:AAA7263
Chironomus riparius|LN894250|||BOLD:AAA7263
Chironomus riparius|LN894216|||BOLD:AAA7263
Chironomus riparius|LN894279|||BOLD:AAA7263
Chironomus riparius|LN894256|||BOLD:AAA7263
Chironomus riparius|LN894267|||BOLD:AAA7263
Chironomus riparius|LN894325|||BOLD:AAA7263
Chironomus riparius|LN894297|||BOLD:AAA7263
Chironomus riparius|LN894218|||BOLD:AAA7263
Chironomus riparius|LN894260|||BOLD:AAA7263
Chironomus riparius|LN894275|||BOLD:AAA7263
Chironomus riparius|LN894293|||BOLD:AAA7263
Chironomus riparius|LN894238|||BOLD:AAA7263
Chironomus riparius|LN894273|||BOLD:AAA7263
Chironomus riparius|LN894288|||BOLD:AAA7263
Chironomus riparius|LN894344|||BOLD:AAA7263
Chironomus riparius|LN894205|||BOLD:AAA7263
[illegible]

Chironomus riparius|LN894288|||BOLD:AAA7263
Chironomus riparius|LN894344|||BOLD:AAA7263
Chironomus riparius|LN894205|||BOLD:AAA7263
Chironomus riparius|LN894284|||BOLD:AAA7263
Chironomus riparius|LN894264|||BOLD:AAA7263
Chironomus riparius|LN894280|||BOLD:AAA7263
Chironomus riparius|LN894287|||BOLD:AAA7263
Chironomus riparius|LN894299|||BOLD:AAA7263
Chironomus riparius|LN894206|||BOLD:AAA7263
Chironomus riparius|LN894207|||BOLD:AAA7263
Chironomus riparius|LN894355|||BOLD:AAA7263
Chironomus riparius|LN894301|||BOLD:AAA7263
Chironomus riparius|LN894286|||BOLD:AAA7263
Chironomus riparius|LN894296|||BOLD:AAA7263
Chironomus riparius|LN894258|||BOLD:AAA7263
Chironomus riparius|LN894234|||BOLD:AAA7263
Chironomus riparius|LN894249|||BOLD:AAA7263
Chironomus riparius|LN894263|||BOLD:AAA7263
Chironomus riparius|LN894351|||BOLD:AAA7263
Chironomus riparius|LN894354|||BOLD:AAA7263
Chironomus riparius|LN894334|||BOLD:AAA7263
Chironomus riparius|LN894347|||BOLD:AAA7263
Chironomus riparius|LN894219|||BOLD:AAA7263
Chironomus riparius|LN894240|||BOLD:AAA7263
Chironomus riparius|LN894353|||BOLD:AAA7263
Chironomus riparius|LN894251|||BOLD:AAA7263
Chironomus riparius|LN894292|||BOLD:AAA7263
Chironomus riparius|LN894352|||BOLD:AAA7263
Chironomus riparius|LN894330|||BOLD:AAA7263
Chironomus riparius|LN894329|||BOLD:AAA7263
Chironomus riparius|LN894270|||BOLD:AAA7263
Chironomus riparius|LN894236|||BOLD:AAA7263
Chironomus riparius|LN894306|||BOLD:AAA7263
Chironomus riparius|LN894237|||BOLD:AAA7263
Chironomus riparius|LN894321|||BOLD:AAA7263
Chironomus riparius|LN894346|||BOLD:AAA7263
Chironomus riparius|LN894200|||BOLD:AAA7263
Chironomus riparius|LN894285|||BOLD:AAA7263
Chironomus riparius|LN894231|||BOLD:AAA7263
Chironomus riparius|LN894222|||BOLD:AAA7263
Chironomus riparius|LN894326|||BOLD:AAA7263
Chironomus riparius|LN894314|||BOLD:AAA7263
Chironomus riparius|LN894307|||BOLD:AAA7263
Chironomus riparius|LN894338|||BOLD:AAA7263
Chironomus riparius|LN894324|||BOLD:AAA7263
Chironomus riparius|LN894243|||BOLD:AAA7263
Chironomus riparius|LN894241|||BOLD:AAA7263
Chironomus riparius|LN894332|||BOLD:AAA7263
Chironomus riparius|LN894490|||BOLD:AAA7263
Chironomus riparius|LN894492|||BOLD:AAA7263
Chironomus riparius|LN894496|||BOLD:AAA7263
Chironomus riparius|LN894488|||BOLD:AAA7263
Chironomus riparius|LN894489|||BOLD:AAA7263
Chironomus riparius|LN894491|||BOLD:AAA7263
Chironomus riparius|LN894494|||BOLD:AAA7263
Chironomus riparius|LN894493|||BOLD:AAA7263
Chironomus riparius|LN894495|||BOLD:AAA7263
Chironomus riparius|LN894357|||BOLD:AAA7263
Chironomus riparius|LN894209|||BOLD:AAA7263
Chironomus riparius|LN894361|||BOLD:AAA7263
Chironomus riparius|LN894382|||BOLD:AAA7263
Chironomus riparius|LN894367|||BOLD:AAA7263
Chironomus riparius|LN894363|||BOLD:AAA7263
Chironomus riparius|LN894360|||BOLD:AAA7263
Chironomus riparius|LN894365|||BOLD:AAA7263
Chironomus riparius|LN894366|||BOLD:AAA7263
Chironomus riparius|LN894364|||BOLD:AAA7263
Chironomus riparius|LN894362|||BOLD:AAA7263
Chironomus riparius|LN894514|||BOLD:AAA7263
Chironomus riparius|LN894410
Chironomus riparius|LN894373|||BOLD:AAA7263
Chironomus riparius|LN894376|||BOLD:AAA7263
Chironomus riparius|LN894374|||BOLD:AAA7263
Chironomus riparius|LN894375|||BOLD:AAA7263
Chironomus riparius|LN894377|||BOLD:AAA7263
Chironomus riparius|LN894372|||BOLD:AAA7263
Chironomus riparius|LN894380|||BOLD:AAA7263
Chironomus riparius|LN894381|||BOLD:AAA7263
Chironomus riparius|LN894378|||BOLD:AAA7263
Chironomus riparius|LN894379|||BOLD:AAA7263
Chironomus riparius|LN894402|||BOLD:AAA7263
Chironomus riparius|LN894386|||BOLD:AAA7263
Chironomus riparius|LN894417|||BOLD:AAA7263
Chironomus riparius|LN894414|||BOLD:AAA7263
Chironomus riparius|LN894383|||BOLD:AAA7263
Chironomus riparius|LN894400|||BOLD:AAA7263
Chironomus riparius|LN894449|||BOLD:AAA7263
Chironomus riparius|LN894441|||BOLD:AAA7263
Chironomus riparius|LN894454|||BOLD:AAA7263
Chironomus riparius|LN894408|||BOLD:AAA7263
Chironomus riparius|LN894419|||BOLD:AAA7263
Chironomus riparius|LN894418|||BOLD:AAA7263
Chironomus riparius|LN894390|||BOLD:AAA7263
Chironomus riparius|LN894431|||BOLD:AAA7263
Chironomus riparius|LN894469|||BOLD:AAA7263
Chironomus riparius|LN894486|||BOLD:AAA7263
Chironomus riparius|LN894416|||BOLD:AAA7263
Chironomus riparius|LN894389|||BOLD:AAA7263
Chironomus riparius|LN894481|||BOLD:AAA7263
Chironomus riparius|LN894413|||BOLD:AAA7263
Chironomus riparius|LN894480|||BOLD:AAA7263
Chironomus riparius|LN894479|||BOLD:AAA7263
Chironomus riparius|LN894434|||BOLD:AAA7263

Chironomus riparius|LN894480|||BOLD:AAA7263
Chironomus riparius|LN894479|||BOLD:AAA7263
Chironomus riparius|LN894434|||BOLD:AAA7263
Chironomus riparius|LN894438|||BOLD:AAA7263
Chironomus riparius|LN894420|||BOLD:AAA7263
Chironomus riparius|LN894470|||BOLD:AAA7263
Chironomus riparius|LN894436|||BOLD:AAA7263
Chironomus riparius|LN894407|||BOLD:AAA7263
Chironomus riparius|LN894447|||BOLD:AAA7263
Chironomus riparius|LN894399|||BOLD:AAA7263
Chironomus riparius|LN894482|||BOLD:AAA7263
Chironomus riparius|LN894435|||BOLD:AAA7263
Chironomus riparius|LN894477|||BOLD:AAA7263
Chironomus riparius|LN894478|||BOLD:AAA7263
Chironomus riparius|LN894473|||BOLD:AAA7263
Chironomus riparius|LN894462|||BOLD:AAA7263
Chironomus riparius|LN894459|||BOLD:AAA7263
Chironomus riparius|LN894391|||BOLD:AAA7263
Chironomus riparius|LN894615|||BOLD:AAA7263
Chironomus riparius|LN894439|||BOLD:AAA7263
Chironomus riparius|LN894394|||BOLD:AAA7263
Chironomus riparius|LN894397|||BOLD:AAA7263
Chironomus riparius|LN894451|||BOLD:AAA7263
Chironomus riparius|LN894424|||BOLD:AAA7263
Chironomus riparius|LN894442|||BOLD:AAA7263
Chironomus riparius|LN894483|||BOLD:AAA7263
Chironomus riparius|LN894392|||BOLD:AAA7263
Chironomus riparius|LN894384|||BOLD:AAA7263
Chironomus riparius|LN894458|||BOLD:AAA7263
Chironomus riparius|LN894444|||BOLD:AAA7263
Chironomus riparius|LN894395|||BOLD:AAA7263
Chironomus riparius|LN894445|||BOLD:AAA7263
Chironomus riparius|LN894426|||BOLD:AAA7263
Chironomus riparius|LN894461|||BOLD:AAA7263
Chironomus riparius|LN894425|||BOLD:AAA7263
Chironomus riparius|LN894466|||BOLD:AAA7263
Chironomus riparius|LN894393|||BOLD:AAA7263
Chironomus riparius|LN894422|||BOLD:AAA7263
Chironomus riparius|LN894421|||BOLD:AAA7263
Chironomus riparius|LN894415|||BOLD:AAA7263
Chironomus riparius|LN894460|||BOLD:AAA7263
Chironomus riparius|LN894443|||BOLD:AAA7263
Chironomus riparius|LN894409|||BOLD:AAA7263
Chironomus riparius|LN894468|||BOLD:AAA7263
Chironomus riparius|LN894465|||BOLD:AAA7263
Chironomus riparius|LN894406|||BOLD:AAA7263
Chironomus riparius|LN894404|||BOLD:AAA7263
Chironomus riparius|LN894455|||BOLD:AAA7263
Chironomus riparius|LN894471|||BOLD:AAA7263
Chironomus riparius|LN894463|||BOLD:AAA7263
Chironomus riparius|LN894485|||BOLD:AAA7263
Chironomus riparius|LN894427|||BOLD:AAA7263
Chironomus riparius|LN894472|||BOLD:AAA7263
Chironomus riparius|LN894610|||BOLD:AAA7263
Chironomus riparius|LN894602|||BOLD:AAA7263
Chironomus riparius|LN894596|||BOLD:AAA7263
Chironomus riparius|LN894618|||BOLD:AAA7263
Chironomus riparius|LN894609|||BOLD:AAA7263
Chironomus riparius|LN894612|||BOLD:AAA7263
Chironomus riparius|LN894598|||BOLD:AAA7263
Chironomus riparius|LN894619|||BOLD:AAA7263
Chironomus riparius|LN894606|||BOLD:AAA7263
Chironomus riparius|LN894605|||BOLD:AAA7263
Chironomus riparius|LN894617|||BOLD:AAA7263
Chironomus riparius|LN894608|||BOLD:AAA7263
Chironomus riparius|LN894437|||BOLD:AAA7263
Chironomus riparius|LN894474|||BOLD:AAA7263
Chironomus riparius|LN894467|||BOLD:AAA7263
Chironomus riparius|LN894411|||BOLD:AAA7263
Chironomus riparius|LN894396|||BOLD:AAA7263
Chironomus riparius|LN894388|||BOLD:AAA7263
Chironomus riparius|LN894405|||BOLD:AAA7263
Chironomus riparius|LN894448|||BOLD:AAA7263
Chironomus riparius|LN894412|||BOLD:AAA7263
Chironomus riparius|LN894453|||BOLD:AAA7263
Chironomus riparius|LN894440|||BOLD:AAA7263
Chironomus riparius|LN894456|||BOLD:AAA7263
Chironomus riparius|LN894446|||BOLD:AAA7263
Chironomus riparius|LN894432|||BOLD:AAA7263
Chironomus riparius|LN894387|||BOLD:AAA7263
Chironomus riparius|LN894600|||BOLD:AAA7263
Chironomus riparius|LN894403|||BOLD:AAA7263
Chironomus riparius|LN894398|||BOLD:AAA7263
Chironomus riparius|LN894604|||BOLD:AAA7263
Chironomus riparius|LN894607|||BOLD:AAA7263
Chironomus riparius|LN894601|||BOLD:AAA7263
Chironomus riparius|LN894611|||BOLD:AAA7263
Chironomus riparius|LN894616|||BOLD:AAA7263
Chironomus riparius|LN894599|||BOLD:AAA7263
Chironomus riparius|LN894450|||BOLD:AAA7263
Chironomus riparius|LN894428|||BOLD:AAA7263
Chironomus riparius|LN894401|||BOLD:AAA7263
Chironomus riparius|LN894475|||BOLD:AAA7263
Chironomus riparius|LN894464|||BOLD:AAA7263
Chironomus riparius|LN894430|||BOLD:AAA7263
Chironomus riparius|LN894385|||BOLD:AAA7263
Chironomus riparius|LN894423|||BOLD:AAA7263
Chironomus riparius|LN894429|||BOLD:AAA7263
Chironomus riparius|LN894613|||BOLD:AAA7263
Chironomus riparius|LN894457|||BOLD:AAA7263
Chironomus riparius|LN894452|||BOLD:AAA7263
Chironomus riparius|LN894476|||BOLD:AAA7263
Chironomus riparius|LN894484|||BOLD:AAA7263

[illegible]
Chironomus riparius|LN894452|||BOLD:AAA7263
Chironomus riparius|LN894476|||BOLD:AAA7263
Chironomus riparius|LN894484|||BOLD:AAA7263
Chironomus riparius|LN894595|||BOLD:AAA7263
Chironomus riparius|LN894597|||BOLD:AAA7263
Chironomus riparius|LN894603|||BOLD:AAA7263
Chironomus riparius|LN894614|||BOLD:AAA7263
Chironomus riparius|LN894620|||BOLD:AAA7263
Chironomus riparius|LN894433|||BOLD:AAA7263
Chironomus riparius|LN894623|||BOLD:AAA7263
Chironomus riparius|LN894626|||BOLD:AAA7263
Chironomus riparius|LN894624|||BOLD:AAA7263
Chironomus riparius|LN894627
Chironomus riparius|LN894368|||BOLD:AAA7263
Chironomus riparius|LN894622|||BOLD:AAA7263
Chironomus riparius|LN894621|||BOLD:AAA7263
Chironomus riparius|LN894625|||BOLD:AAA7263
Chironomus riparius|LN894303|||BOLD:AAA7263
Chironomus|BIOUG36853-C03|Kate Perez|BIN Taxonomy Match (Jul 2018)|BOLD:AAA7263
Chironomus riparius|LN894497|||BOLD:AAA7263
Chironomus riparius|LN894498|||BOLD:AAA7263
Chironomus riparius|LN894499|||BOLD:AAA7263
Chironomus riparius|LN894500|||BOLD:AAA7263
Chironomus riparius|LN894501|||BOLD:AAA7263
Chironomus riparius|LN894516|||BOLD:AAA7263
Chironomus riparius|LN894550|||BOLD:AAA7263
Chironomus riparius|LN894549|||BOLD:AAA7263
Chironomus riparius|LN894541|||BOLD:AAA7263
Chironomus riparius|LN894274|||BOLD:AAA7263
Chironomus riparius|LN894537|||BOLD:AAA7263
Chironomus riparius|LN894532|||BOLD:AAA7263
Chironomus riparius|LN894322|||BOLD:AAA7263
Chironomus riparius|LN894512|||BOLD:AAA7263
Chironomus riparius|LN894198|||BOLD:AAA7263
Chironomus riparius|LN894315|||BOLD:AAA7263
Chironomus riparius|LN894257|||BOLD:AAA7263
Chironomus riparius|LN894320|||BOLD:AAA7263
Chironomus riparius|LN894298|||BOLD:AAA7263
Chironomus riparius|LN894244|||BOLD:AAA7263
Chironomus riparius|LN894254|||BOLD:AAA7263
Chironomus riparius|LN894341|||BOLD:AAA7263
Chironomus riparius|LN894370|||BOLD:AAA7263
Chironomus riparius|LN894211|||BOLD:AAA7263
Chironomus riparius|LN894221|||BOLD:AAA7263
Chironomus riparius|LN894319|||BOLD:AAA7263
Chironomus riparius|LN894269|||BOLD:AAA7263
Chironomus riparius|LN894294|||BOLD:AAA7263
Chironomus riparius|LN894213|||BOLD:AAA7263
Chironomus riparius|LN894201|||BOLD:AAA7263
Chironomus riparius|LN894226|||BOLD:AAA7263
Chironomus riparius|LN894343|||BOLD:AAA7263
Chironomus riparius|LN894212|||BOLD:AAA7263
Chironomus riparius|LN894266|||BOLD:AAA7263
Chironomus riparius|LN894239|||BOLD:AAA7263
Chironomus riparius|LN894310|||BOLD:AAA7263
Chironomus riparius|LN894235|||BOLD:AAA7263
Chironomus riparius|LN894202|||BOLD:AAA7263
Chironomus riparius|LN894245|||BOLD:AAA7263
Chironomus riparius|LN894327|||BOLD:AAA7263
Chironomus riparius|LN894262|||BOLD:AAA7263
Chironomus riparius|LN894295|||BOLD:AAA7263
Chironomus riparius|LN894323|||BOLD:AAA7263
Chironomus riparius|LN894335|||BOLD:AAA7263
Chironomus riparius|LN894302|||BOLD:AAA7263
Chironomus riparius|LN894253|||BOLD:AAA7263
Chironomus riparius|LN894230|||BOLD:AAA7263
Chironomus riparius|LN894242|||BOLD:AAA7263
Chironomus riparius|LN894228|||BOLD:AAA7263
Chironomus riparius|LN894348|||BOLD:AAA7263
Chironomus riparius|LN894309|||BOLD:AAA7263
Chironomus riparius|LN894331|||BOLD:AAA7263
Chironomus riparius|LN894214|||BOLD:AAA7263
Chironomus riparius|LN894225|||BOLD:AAA7263
Chironomus riparius|LN894278|||BOLD:AAA7263
Chironomus riparius|LN894281|||BOLD:AAA7263
Chironomus riparius|OL911048|||BOLD:AAA7263
Chironomus|4035755211|||BOLD:AAJ4282
Chironomus luridus|LA_LSLRZ9_57|Piotr Gadawski||BOLD:AAJ4282
Chironomus luridus|LA_LSLRZ9 _97|Piotr Gadawski||BOLD:AAJ4282
Chironomus luridus|LA_LSLRZ9_58|Piotr Gadawski||BOLD:AAJ4282
Chironomus luridus|LA_LSLRZ9_226|Piotr Gadawski|Morphology|BOLD:AAJ4282
Chironomus luridus|LA_LSLRZ9_99|Piotr Gadawski||BOLD:AAJ4282
Chironomus luridus|LA_ASL2_35|Piotr Gadawski||BOLD:AAJ4282
Chironomus luridus|LA_LSLRZ9_223|Piotr Gadawski|Morphology|BOLD:AAJ4282
Chironomus luridus|RIN_CH54|Mona Renate Saursaunet|BOLD Identification engine|BOLD:AAJ4282
Chironomus luridus|LA_LSLRZ9_230|Piotr Gadawski|Morphology|BOLD:AAJ4282
Chironomus luridus|LA_LSLRZ9_239|Piotr Gadawski|Morphology|BOLD:AAJ4282
Chironomus luridus|LA_LSLRZ9_221|Piotr Gadawski|Morphology|BOLD:AAJ4282
Chironomus luridus|TRD-CH379|Elisabeth Stur|morphology|BOLD:AAJ4282
Chironomus luridus|TRD-CH358|Elisabeth Stur|morphology|BOLD:AAJ4282
Chironomus luridus|LA_LSLRZ9_241|Piotr Gadawski|Morphology|BOLD:AAJ4282
Chironomus luridus|TRD-CH376|Elisabeth Stur|BOLD Identification Engine|BOLD:AAJ4282
Chironomus luridus|RIN_CH38||BOLD Identification engine|BOLD:AAJ4282
Chironomus luridus|RIN_CH53|Elisabeth Stur|BOLD Identification engine|BOLD:AAJ4282
Chironomus luridus|BIOUG16626-H10|Kate Perez|BIN Taxonomy Match|BOLD:AAJ4282
Chironomus luridus|TRD-CH155|Elisabeth Stur|morphology|BOLD:AAJ4282
Chironomus|BIOUG55199-D06|Stefan Schmidt|BIN Taxonomy Match (Sep 2020)|BOLD:AAJ4282
Chironomus|BIOUG55421-B05|Stefan Schmidt|BIN Taxonomy Match (Sep 2020)|BOLD:AAJ4282
Chironomus luridus|LA_LSLRZ9_225|Piotr Gadawski|Morphology|BOLD:AAJ4282
Chironomus nr. pseudothummi|ZMUO.024748|Lauri Paasivirta||BOLD:AAJ4282
Chironomus nr. riparius|ZMUO.025192|Lauri Paasivirta||BOLD:AAJ4282
Chironomus nr. riparius|ZMUO.024539|Lauri Paasivirta||BOLD:AAJ4282
[illegible]

Chironomus nr. pseudothummi|ZMUO.024748|Lauri Paasivirta||BOLD:AAJ4282
Chironomus nr. riparius|ZMUO.025192|Lauri Paasivirta||BOLD:AAJ4282
Chironomus nr. riparius|ZMUO.024539|Lauri Paasivirta||BOLD:AAJ4282
Chironomus nr. riparius|ZMUO.024906|Lauri Paasivirta||BOLD:AAJ4282
Chironomus nr. riparius|ZMUO.024918|Lauri Paasivirta||BOLD:AAJ4282
Chironomus nr. riparius|ZMUO.024907|Lauri Paasivirta||BOLD:AAJ4282
Chironomus|ZSM-DIP-33129-D07|Caroline Chimeno|BIN Taxonomy Match (Jan 2022)|BOLD:AAJ4282
Chironomus|BIOUG42967-A06|Kate Perez|BIN Taxonomy Match below Phylum (Jun 2019)|BOLD:AAJ...
Chironomus|ZSM-DIP-33142-G06|Caroline Chimeno|BIN Taxonomy Match (Jan 2022)|BOLD:AAJ4282
Chironomus|ZSM-DIP-33133-A01|Caroline Chimeno|BIN Taxonomy Match (Jan 2022)|BOLD:AAJ4282
Chironomus|ZSM-DIP-33128-G11|Caroline Chimeno|BIN Taxonomy Match (Jan 2022)|BOLD:AAJ4282
Chironomus|BIOUG42718-F02|Kate Perez|BIN Taxonomy Match below Phylum (Jun 2019)|BOLD:AAJ...
Chironomus|BIOUG42948-B09|Kate Perez|BIN Taxonomy Match below Phylum (Jun 2019)|BOLD:AAJ...
Chironomus|ZSM-DIP-33145-G01|Caroline Chimeno|BIN Taxonomy Match (Jan 2022)|BOLD:AAJ4282
Chironomus|ZSM-DIP-33145-H06|Caroline Chimeno|BIN Taxonomy Match (Jan 2022)|BOLD:AAJ4282
Chironomus|ZSM-DIP-33149-E02|Caroline Chimeno|BIN Taxonomy Match (Jan 2022)|BOLD:AAJ4282
Chironomus|ZSM-DIP-33129-F09|Caroline Chimeno|BIN Taxonomy Match (Jan 2022)|BOLD:AAJ4282
Chironomus|BIOUG42939-A04|Kate Perez|BIN Taxonomy Match below Phylum (Jun 2019)|BOLD:AAJ...
Chironomus luridus|LA_ASL1_31|Piotr Gadawski||BOLD:AAJ4282
Chironomus nr. riparius|ZMUO.024538|Lauri Paasivirta||BOLD:AAJ4282
Chironomus|BIOUG42723-A11|Kate Perez|BIN Taxonomy Match below Phylum (Jun 2019)|BOLD:AAJ...
Chironomus|ZSM-DIP-33144-F12|Caroline Chimeno|BIN Taxonomy Match (Jan 2022)|BOLD:AAJ4282
Chironomus|ZSM-DIP-33144-E08|Caroline Chimeno|BIN Taxonomy Match (Jan 2022)|BOLD:AAJ4282
Chironomus|ZSM-DIP-33144-E07|Caroline Chimeno|BIN Taxonomy Match (Jan 2022)|BOLD:AAJ4282
Chironomus|ZSM-DIP-33137-F12|Caroline Chimeno|BIN Taxonomy Match (Jan 2022)|BOLD:AAJ4282
Chironomus nr. riparius|ZMUO.025193|Lauri Paasivirta||BOLD:AAJ4282
Chironomus aprilinus|NHRS-BYWS000000438|Yngve Brodin|morphology
Chironomus aprilinus|NHRS-BYWS000001779|Yngve Brodin||BOLD:AAF3277
Chironomus aprilinus|ZMUO.026122|Lauri Paasivirta||BOLD:AAF3277
Chironomus aprilinus|NHRS-BYWS000001781|Yngve Brodin||BOLD:AAF3277
Chironomus aprilinus|NHRS-BYWS000000475|Yngve Brodin|morphology|BOLD:AAF3277
Chironomus aprilinus|NHRS-BYWS000001780|Yngve Brodin||BOLD:AAF3277
Chironomus aprilinus|EJV-20110245|Lauri Paasivirta||BOLD:AAF3277
Chironomus aprilinus|NHRS-BYWS000000954|Yngve Brodin|morphology|BOLD:AAF3277
Chironomus aprilinus|NHRS-BYWS000000235|Yngve Brodin|morphology|BOLD:AAF3277
Chironomus aprilinus|NHRS-BYWS000000901|Yngve Brodin|morphology|BOLD:AAF3277
Chironomus aprilinus|NHRS-BYWS000000353|Yngve Brodin|morphology|BOLD:AAF3277
Chironomus aprilinus|NHRS-BYWS000000175|Yngve Brodin|morphology|BOLD:AAF3277
Chironomus aprilinus|NHRS-BYWS000001064|Yngve Brodin|morphology|BOLD:AAF3277
Chironomus aprilinus|NHRS-BYWS000000276|Yngve Brodin|morphology|BOLD:AAF3277
Chironomus aprilinus|NHRS-BYWS000000236|Yngve Brodin|morphology|BOLD:AAF3277
Chironomus aprilinus|NHRS-BYWS000000176|Yngve Brodin|morphology|BOLD:AAF3277
Chironomus nr. pseudothummi|ZMUO.025359|Lauri Paasivirta||BOLD:AAF3277
Chironomus nr. pseudothummi|ZMUO.025358|Lauri Paasivirta||BOLD:AAF3277
Chironomus aprilinus|ZMUO.026121|Lauri Paasivirta||BOLD:AAF3277
Chironomus aprilinus|NHRS-BYWS000000416|Yngve Brodin|morphology|BOLD:AAF3277
Chironomus aprilinus|NHRS-BYWS000000174|Yngve Brodin|morphology|BOLD:AAF3277
Chironomus sollicitus|ATNA78|Elisabeth Stur||BOLD:AAI4306
Chironomus sp. 1TE|Finnmark340|Torbjorn Ekrem||BOLD:AAC0592
Chironomus riparius|NHRS-BYWS000001160|Yngve Brodin|morphology|BOLD:AAI4307
Chironomus riparius|NHRS-BYWS000001159|Yngve Brodin|morphology|BOLD:AAI4307
Chironomus sp. 10TE|ATNA340|Torbjorn Ekrem||BOLD:AAI4307
Chironomus sp. 10TE|CH-OSF180|Torbjorn Ekrem||BOLD:AAI4307
Chironomus nr. sollicitus|ZMUO.024594|Lauri Paasivirta||BOLD:AAS1281
Chironomus melanescens|Finnmark688|Torbjorn Ekrem||BOLD:AAS1281
Chironomus|ZSM-DIP-33128-E05|Caroline Chimeno|BIN Taxonomy Match (Jan 2022)|BOLD:AAS1281
Chironomus nr. sollicitus|ZMUO.024595|Lauri Paasivirta||BOLD:AAS1281
Chironomus nr. sollicitus|ZMUO.024888|Lauri Paasivirta||BOLD:AAS1281
Chironomus cf. sollicitus|NHRS-BYWS000001082|Yngve Brodin|morphology|BOLD:AAI4299
Chironomus|SOE263|Torbjorn Ekrem||BOLD:AAI4299
Chironomus|RIN_CH46|Mona Renate Saursaunet|BOLD Identification engine|BOLD:AAI4299
Chironomus|RIN_CH52|Mona Renate Saursaunet|BOLD Identification engine|BOLD:AAI4299
Chironomus|BIOUG42931-H04|Kate Perez|BIN Taxonomy Match below Phylum (Jun 2019)|BOLD:AAI...
Chironomus|BIOUG42927-G11|Kate Perez|BIN Taxonomy Match below Phylum (Jun 2019)|BOLD:AAI...
Chironomus|BIOUG42923-C07|Kate Perez|BIN Taxonomy Match below Phylum (Jun 2019)|BOLD:AAI...
Chironomus|ZSM-DIP-33139-A02|Caroline Chimeno|BIN Taxonomy Match (Jan 2022)|BOLD:AAW4001
Chironomus|ZSM-DIP-33146-D03|Caroline Chimeno|BIN Taxonomy Match (Jan 2022)|BOLD:AAW4001
Chironomus alpestris|ZSM34342-E09|Martin Spies|Morphology|BOLD:AAW4001
Chironomus alpestris|ZSM34342-D01|Martin Spies|Morphology|BOLD:AAW4001
Chironomus|ZSM-DIP-33139-A03|Caroline Chimeno|BIN Taxonomy Match (Jan 2022)|BOLD:AAW4001
Chironomus dorsalis|BIOUG42691-G06|Kate Perez|BIN Taxonomy Match below Phylum (Jun 2019)|BO...
Chironomus dorsalis|BIOUG42691-A02|Kate Perez|BIN Taxonomy Match below Phylum (Jun 2019)|BO...
Chironomus pseudothummi|CH-OSF190|Torbjorn Ekrem||BOLD:ABZ0429
Chironomus NHRS sp. A|NHRS-BYWS000001130|Yngve Brodin|morphology|BOLD:ABZ0429
Chironomus nr. pseudothummi|ZMUO.024813|Lauri Paasivirta||BOLD:ABZ0429
Chironomus nr. pseudothummi|ZMUO.024749|Lauri Paasivirta||BOLD:ABZ0429
Chironomus pseudothummi|CH-OSF36|Torbjorn Ekrem||BOLD:ABZ0429
Chironomus nr. pseudothummi|ZMUO.024949|Lauri Paasivirta||BOLD:ADL5630
Chironomus sp. TE11|Finnmark434|Torbjorn Ekrem||BOLD:ADL5630
Chironomus sp. TE11|Finnmark725|Torbjorn Ekrem||BOLD:ADL5630
Chironomus sp. TE11|Finnmark578|Torbjorn Ekrem||BOLD:ADL5630
Chironomus sp. TE11|TRD-CH299|Elisabeth Stur||BOLD:ADL5630
Chironomus nr. pseudothummi|ZMUO.024950|Lauri Paasivirta||BOLD:ADL5630
Chironomus sp. TE11|TRD-CH378|Elisabeth Stur|morphology|BOLD:ADL5630
Chironomus sp. TE11|CH-OSF86|Torbjorn Ekrem||BOLD:ADL5630
Chironomus sp. TE11|Finnmark166|Torbjorn Ekrem||BOLD:ADL5630
Chironomus sp. TE11|ATNA339|Torbjorn Ekrem||BOLD:ADL5630
Chironomus sp. TE11|TRD-CH228|Elisabeth Stur||BOLD:ADL5630
Chironomus sp. TE11|Finnmark304|Torbjorn Ekrem||BOLD:ADL5630
Chironomus|ZSM-DIP-33138-C01|Caroline Chimeno|BIN Taxonomy Match (Jan 2022)|BOLD:ADL5630
Chironomus|BIOUG55415-G10|Stefan Schmidt|BIN Taxonomy Match (Sep 2020)|BOLD:ADL5630
Chironomus|BIOUG42736-A09|Kate Perez|BIN Taxonomy Match below Phylum (Jul 2019)|BOLD:ADL...
Chironomus|BIOUG42941-A08|Kate Perez|BIN Taxonomy Match below Phylum (Jul 2019)|BOLD:ADL...
Chironomus|BIOUG42762-F07|Kate Perez|BIN Taxonomy Match below Phylum (Jul 2019)|BOLD:ADL...
Chironomus sp. 6TE|Finnmark357|Torbjorn Ekrem||BOLD:AAV3581
Chironomus heteropilicornis|TRD-CH266|Elisabeth Stur||BOLD:ACX5781
Chironomus heteropilicornis|TRD-CH269|Elisabeth Stur||BOLD:ACX5781
Chironomus pilicornis|NHRS-BYWS000001079|Yngve Brodin|morphology|BOLD:ACX5781
Chironomus heteropilicornis|TRD-CH268|Elisabeth Stur||BOLD:ACX5781
Chironomus heteropilicornis|TRD-CH267|Elisabeth Stur||BOLD:ACX5781
Chironomus pilicornis|NHRS-BYWS000001080|Yngve Brodin|morphology|BOLD:ACX5781
Chironomus heteropilicornis|TRD-CH410|Elisabeth Stur|morphology|BOLD:ACX5781
Chironomus riihimakiensis|ZMUO.024922|Lauri Paasivirta||BOLD:AAW3994

Chironomus pilicornis|NHRS-BYWS000001080|Yngve Brodin|morphology|BOLD:ACX5781
Chironomus heteropilicornis|TRD-CH410|Elisabeth Stur|morphology|BOLD:ACX5781
Chironomus riihimakiensis|ZMUO.024922|Lauri Paasivirta||BOLD:AAW3994
Chironomus cf. tenuistylus|PK-186-18|Petra Kranzfelder|morphology|BOLD:AAW3994
Chironomus cf. tenuistylus|PK-186-17|Petra Kranzfelder|morphology|BOLD:AAW3994
Chironomus cf. tenuistylus|PK-186-16|Petra Kranzfelder|morphology|BOLD:AAW3994
Chironomus cf. tenuistylus|PK-186-15|Petra Kranzfelder|morphology|BOLD:AAW3994
Chironomus cf. tenuistylus|PK-186-14|Petra Kranzfelder|morphology|BOLD:AAW3994
Chironomus cf. tenuistylus|PK-186-13|Petra Kranzfelder|morphology|BOLD:AAW3994
Chironomus cf. tenuistylus|PK-186-11|Petra Kranzfelder|morphology|BOLD:AAW3994
Chironomus|PK-186-8|Petra Kranzfelder|morphology|BOLD:ACT6965
Chironomus|PK-186-9|Petra Kranzfelder|morphology|BOLD:ACT6965
Chironomus cf. venustus|Finnmark259|Torbjorn Ekrem||BOLD:AAV3582
Chironomus hyperboreus|Finnmark38|Torbjorn Ekrem||BOLD:AAC0596
Chironomus hyperboreus|TRD-CH258|Elisabeth Stur||BOLD:AAC0596
Chironomus hyperboreus|Finnmark664|Elisabeth Stur||BOLD:AAC0596
Chironomus islandicus|Finnmark36|Elisabeth Stur||BOLD:AAI4308
Chironomus islandicus|Finnmark526|Elisabeth Stur||BOLD:AAI4308
Chironomus islandicus|Finnmark868|Elisabeth Stur|morphology|BOLD:AAI4308
Chironomus islandicus|Finnmark37|Elisabeth Stur||BOLD:AAI4308
Chironomus islandicus|Finnmark743|Elisabeth Stur||BOLD:AAI4308
Chironomus islandicus|Finnmark652|Elisabeth Stur||BOLD:AAI4308
Chironomus islandicus|Finnmark53|Elisabeth Stur||BOLD:AAI4308
Chironomus islandicus|TRD-CH356|Elisabeth Stur|morphology|BOLD:AAI4308
Chironomus sp. 14TE|Finnmark869|Elisabeth Stur|morphology|BOLD:ACY4329
Limnophyes edwardsi|ZMUO.025972|Lauri Paasivirta||BOLD:ADE9709
Chironomus|ZMUO.025968|Lauri Paasivirta||BOLD:ADE9709
Chironomus|ZMUO.025967|Lauri Paasivirta||BOLD:ADE9709
Chironomus riihimakiensis|ZMUO.024921|Lauri Paasivirta||BOLD:ADA8845
Chironomus nr. longistylus|ZMUO.024803|Lauri Paasivirta||BOLD:ADA8845
Chironomus sp. 8TE|TRD-CH286|Elisabeth Stur||BOLD:ADA8845
Chironomus sp. 8TE|Finnmark631|Torbjorn Ekrem||BOLD:AAC0595
Chironomus sp. 8TE|Finnmark444|Torbjorn Ekrem||BOLD:AAC0595
Chironomus sp. 8TE|Finnmark661|Torbjorn Ekrem||BOLD:AAC0595
Chironomus sp. 8TE|Finnmark223|Torbjorn Ekrem||BOLD:AAC0595
Chironomus|ZMUO.024799|Lauri Paasivirta||BOLD:AAW4009
Chironomus cingulatus|ZMUO.024541|Lauri Paasivirta||BOLD:AAW4009
Chironomus cingulatus|CCDB21605-H05|Martin Spies|Morphology|BOLD:AAW4009
Chironomus agilis|ZMUO.029800|Marko Mutanen||BOLD:AAW4009
Chironomus obtusidens|TRD-CH207|Elisabeth Stur||BOLD:ACT6966
Chironomus|ZMUO.025129|Lauri Paasivirta||BOLD:AAI4297
Chironomus|ZMUO.025128|Lauri Paasivirta||BOLD:AAI4297
Chironomus longistylus|ATNA76|Elisabeth Stur||BOLD:AAI4304
Chironomus longistylus|ATNA341|Elisabeth Stur||BOLD:AAI4304
Chironomus longistylus|ATNA79|Elisabeth Stur||BOLD:AAI4305
Chironomus longistylus|ATNA77|Elisabeth Stur||BOLD:AAI4305
Chironomus longistylus|ATNA343|Elisabeth Stur||BOLD:AAI4305
Chironomus longistylus|ATNA342|Elisabeth Stur||BOLD:AAI4305
Chironomus acerbus|Finnmark601|Torbjorn Ekrem||BOLD:AAQ2148
Chironomus|ZMUO.025195|Lauri Paasivirta||BOLD:AAC0594
Chironomus|ZMUO.025194|Lauri Paasivirta||BOLD:AAC0594
Chironomus sp. 3TE|Finnmark308|Torbjorn Ekrem||BOLD:AAJ4142
Chironomus sp. 3TE|Finnmark307|Torbjorn Ekrem||BOLD:AAJ4142
Chironomus sp. 3TE|Finnmark306|Torbjorn Ekrem||BOLD:AAJ4142
Chironomus anthracinus|ZMUO.025126|Lauri Paasivirta||BOLD:AAM5953
Chironomus anthracinus|ZMUO.024616|Lauri Paasivirta||BOLD:AAM5953
Chironomus sp. 5TE|Finnmark49|Torbjorn Ekrem||BOLD:AAM5953
Chironomus anthracinus|ZMUO.025127|Lauri Paasivirta||BOLD:AAM5953
Chironomus sp. 5TE|Finnmark383|Torbjorn Ekrem||BOLD:AAM5953
Chironomus sp. PA15|KY225338|||BOLD:ACO8871
Chironomus anthracinus|KY225342
Chironomus anthracinus|ZMUO.024617|Lauri Paasivirta||BOLD:ACO8871
Chironomus anthracinus|KY225340|||BOLD:ACO8871
Chironomus anthracinus|KY225341|||BOLD:ACO8871
Chironomus melanotus|ZMUO.024957|Lauri Paasivirta||BOLD:AAM5952
Chironomus sp. 4TE|ATNA566|Elisabeth Stur||BOLD:AAM5952
Chironomus sp. 4TE|CH-OSF97|Torbjorn Ekrem||BOLD:AAM5952
Chironomus sp. 4TE|ATNA577|Elisabeth Stur|morphology|BOLD:AAM5952
Chironomus sp. 4TE|Finnmark126|Torbjorn Ekrem||BOLD:AAM5952
Chironomus sp. 4TE|TRD-CH377|Elisabeth Stur|morphology|BOLD:AAM5952
Chironomus sp. 4TE|TRD-CH227|Elisabeth Stur||BOLD:AAM5952
Chironomus sp. 4TE|TRD-CH181|Elisabeth Stur||BOLD:AAM5952
Chironomus melanotus|NHRS-BYWS000001081|Yngve Brodin|morphology|BOLD:AAW4000
Chironomus nr. longistylus|ZMUO.024802|Lauri Paasivirta||BOLD:ADA6115
Chironomus melanotus|ZMUO.024867|Lauri Paasivirta||BOLD:ADA6115
Chironomus melanotus|ZMUO.024866|Lauri Paasivirta||BOLD:ADA6115
Chironomus|ZMUO.024798|Lauri Paasivirta||BOLD:AAW4002
Chironomus cingulatus|ZMUO.025209|Lauri Paasivirta||BOLD:AAW4002
Chironomus cingulatus|NHRS-BYWS000001068|Yngve Brodin|morphology|BOLD:AAW4002
Chironomus cingulatus|BIOUG42683-A12|Kate Perez|BIN Taxonomy Match below Phylum (Jun 2019)|...
Chironomus cingulatus|BIOUG42929-C11|Kate Perez|BIN Taxonomy Match below Phylum (Jun 2019)|...
Chironomus cingulatus|NHRS-BYWS000001168|Yngve Brodin|morphology|BOLD:AAW4002
Chironomus cingulatus|ZMUO.024542|Lauri Paasivirta||BOLD:AAW4002
Chironomus cingulatus|BIOUG42760-C06|Kate Perez|BIN Taxonomy Match below Phylum (Jun 2019)|...
Chironomus cingulatus|BIOUG42742-C12|Kate Perez|BIN Taxonomy Match below Phylum (Jun 2019)|...
Chironomus cingulatus|BIOUG42686-E11|Kate Perez|BIN Taxonomy Match below Phylum (Jun 2019)|...
Chironomus cingulatus|BIOUG42754-H02|Kate Perez|BIN Taxonomy Match below Phylum (Jun 2019)|...
Chironomus cingulatus|BIOUG42755-A11|Kate Perez|BIN Taxonomy Match below Phylum (Jun 2019)|...
Chironomus cingulatus|BIOUG42724-F08|Kate Perez|BIN Taxonomy Match below Phylum (Jun 2019)|...
Chironomus lacunarius|CCDB21606-H09|Martin Spies|Morphology|BOLD:AAW4002
Chironomus lacunarius|ZSM34343-E02|Martin Spies|Morphology|BOLD:AAW4002
Chironomus lacunarius|ZSM34343-D01|Martin Spies|Morphology|BOLD:AAW4002
Chironomus lacunarius|CCDB21606-H10|Martin Spies|Morphology|BOLD:AAW4002
Chironomus lacunarius|ZSM34343-C09|Martin Spies|Morphology|BOLD:AAW4002
Chironomus annularius|NHRS-BYWS000000234|Yngve Brodin|morphology|BOLD:AAU4047
Chironomus annularius|NHRS-BYWS000000935|Yngve Brodin|morphology|BOLD:AAU4047
Chironomus annularius|NHRS-BYWS000001139|Yngve Brodin|morphology|BOLD:AAU4047
Chironomus annularius|EJV-20110247|Lauri Paasivirta||BOLD:AAU4047
Chironomus annularius|ZMUO.025401|Lauri Paasivirta||BOLD:AAU4047
Chironomus annularius|NHRS-BYWS000001140|Yngve Brodin|morphology|BOLD:AAU4047
Chironomus annularius|NHRS-BYWS000001133|Yngve Brodin|morphology|BOLD:AAU4047
Chironomus annularius|NHRS-BYWS000001862|Yngve Brodin||BOLD:AAU4047
Chironomus annularius|ZMUO.025400|Lauri Paasivirta||BOLD:AAU4047

Chironomus annularius|NHRS-BYWS000001133|Yngve Brodin|morphology|BOLD:AAU4047
Chironomus annularius|NHRS-BYWS000001862|Yngve Brodin||BOLD:AAU4047
Chironomus annularius|ZMUO.025400|Lauri Paasivirta||BOLD:AAU4047
Chironomus annularius|NHRS-BYWS000001012|Yngve Brodin|morphology|BOLD:AAU4046
Chironomus melanotus|ZMUO.024544|Lauri Paasivirta||BOLD:AAU4046
Chironomus annularius|ZMUO.026134|Lauri Paasivirta||BOLD:AAU4046
Chironomus annularius|NHRS-BYWS000001861|Yngve Brodin||BOLD:AAU4046
Chironomus melanotus|ZMUO.025403|Lauri Paasivirta||BOLD:AAU4046
Chironomus annularius|NHRS-BYWS000001166|Yngve Brodin|morphology|BOLD:AAU4046
Chironomus annularius|ZMUO.026135|Lauri Paasivirta||BOLD:AAU4046
Chironomus annularius|NHRS-BYWS000001032|Yngve Brodin|morphology|BOLD:AAU4046
Chironomus annularius|NHRS-BYWS000001013|Yngve Brodin|morphology|BOLD:AAU4046
Chironomus annularius|NHRS-BYWS000000577|Yngve Brodin|morphology|BOLD:AAU4046
Chironomus annularius|NHRS-BYWS000000118|Yngve Brodin|morphology|BOLD:AAU4046
Chironomus entis|ZMUO.024546|Lauri Paasivirta||BOLD:AAJ4161
Chironomus|ZMUO.026229|Lauri Paasivirta||BOLD:AAJ4161
Chironomus cf. nudiventris|NHRS-BYWS000001004|Yngve Brodin|morphology|BOLD:AAW3990
Chironomus plumosus|ZMUO.024737|Lauri Paasivirta||BOLD:AAW3990
Chironomus|ZMUO.025265|Lauri Paasivirta||BOLD:ADF1572
Chironomus plumosus|ZMUO.025175|Lauri Paasivirta||BOLD:ADF1572
Chironomus|ZMUO.025266|Lauri Paasivirta||BOLD:ADF1572
Chironomus plumosus|ZMUO.025174|Lauri Paasivirta||BOLD:ADF1572
Chironomus plumosus|ZMUO.024736|Lauri Paasivirta||BOLD:ADA8508
Chironomus entis|ZMUO.024820|Lauri Paasivirta||BOLD:ACB4515
Chironomus entis|ZMUO.024819|Lauri Paasivirta||BOLD:ACB4515
Chironomus entis|ZMUO.024657|Lauri Paasivirta||BOLD:ACB4515
Chironomus entis-t.|ZMUO.029776|Marko Mutanen||BOLD:ACB4515
Chironomus tentans|KY225373|||BOLD:AAU2239
Chironomus tentans|KY225374|||BOLD:AAU2239
Chironomus plumosus|NHRS-BYWS000000980|Yngve Brodin|morphology|BOLD:AAU2239
Chironomus plumosus|NHRS-BYWS000000281|Yngve Brodin|morphology|BOLD:AAU2239
Chironomus plumosus|NHRS-BYWS000000979|Yngve Brodin|morphology|BOLD:AAU2239
Chironomus|ZMUO.026230|Lauri Paasivirta||BOLD:AAU2239
Chironomus plumosus|NHRS-BYWS000000213|Yngve Brodin|morphology|BOLD:AAU2239
Chironomus plumosus|NHRS-BYWS000000212|Yngve Brodin|morphology|BOLD:AAU2239
Chironomus plumosus|NHRS-BYWS000000354|Yngve Brodin|morphology|BOLD:AAU2239
Chironomus plumosus|NHRS-BYWS000000101|Yngve Brodin|morphology|BOLD:AAU2239
Chironomus plumosus|NHRS-BYWS000001833|Yngve Brodin||BOLD:AAU2239
Chironomus plumosus|KY225375|||BOLD:AAU2239
Chironomus plumosus|NHRS-BYWS000001000|Yngve Brodin|morphology|BOLD:AAU2239
Chironomus plumosus|CCDB22605-H01|Martin Spies|Morphology|BOLD:AAU2239
Chironomus plumosus|NHRS-BYWS000000526|Yngve Brodin|morphology|BOLD:AAU2239
Chironomus plumosus|NHRS-BYWS000001814|Yngve Brodin||BOLD:AAU2239
Chironomus plumosus|NHRS-BYWS000001834|Yngve Brodin||BOLD:AAU2239
Chironomus|ZMUO.025361|Lauri Paasivirta||BOLD:AAU2239
Chironomus|ZMUO.025360|Lauri Paasivirta||BOLD:AAU2239
Chironomus nuditarsis|KY225345|||BOLD:ACD8415
Chironomus NHRS sp. B|NHRS-BYWS000000116|Yngve Brodin|morphology|BOLD:ACD8415
Glyptotendipes lobiferus|TRD-CH343|Elisabeth Stur|morphology|BOLD:AAF8348
Glyptotendipes lobiferus|TRD-CH28|Elisabeth Stur||BOLD:AAF8348
Glyptotendipes cauliginellus|NHRS-BYWS000001018|Yngve Brodin|morphology|BOLD:AAF8348
Glyptotendipes cauliginellus|EJV-20110011|Lauri Paasivirta||BOLD:AAF8348
Glyptotendipes cauliginellus|ZMUO.024246|Lauri Paasivirta||BOLD:AAF8348
Glyptotendipes cauliginellus|NHRS-BYWS000000967|Yngve Brodin|morphology|BOLD:AAF8348
Glyptotendipes cauliginellus|NHRS-BYWS000000476|Yngve Brodin|morphology|BOLD:AAF8348
Glyptotendipes cauliginellus|EJV-20110015|Lauri Paasivirta||BOLD:AAF8348
Glyptotendipes cauliginellus|NHRS-BYWS000000340|Yngve Brodin|morphology|BOLD:AAF8348
Glyptotendipes cauliginellus|EJV-20110013|Lauri Paasivirta||BOLD:AAF8348
Glyptotendipes cauliginellus|EJV-20110012|Lauri Paasivirta||BOLD:AAF8348
Glyptotendipes cauliginellus|EJV-20110014|Lauri Paasivirta||BOLD:AAF8348
Glyptotendipes cauliginellus|NHRS-BYWS000000311|Yngve Brodin|morphology|BOLD:AAF8348
Glyptotendipes cauliginellus|NHRS-BYWS000000310|Yngve Brodin|morphology|BOLD:AAF8348
Harnischia fuscimana|ZMUO.025211|Lauri Paasivirta||BOLD:AAW5694
Harnischia fuscimana|ZMUO.025210|Lauri Paasivirta||BOLD:AAW5694
Harnischia fuscimana|EJV-20110154|Lauri Paasivirta||BOLD:AAW5694
Harnischia fuscimana|EJV-20110153|Lauri Paasivirta||BOLD:AAW5694
Parachironomus monochromus|IM_VI6V14_297|Piotr Gadawski|Morphology|BOLD:AED9752
Parachironomus monochromus|PAR_MON_IM_PL7VII14_162|Piotr Gadawski|Morphology|BOLD:ACY5073
Parachironomus monochromus|PAR_MON_IM_VR9X14_106|Piotr Gadawski|Morphology|BOLD:ACY5073
Parachironomus monochromus|PAR_MON_IM_VI8X14_59|Piotr Gadawski|Morphology|BOLD:ACY5073
Parachironomus monochromus|PAR_MON_IM_VI6V14_127|Piotr Gadawski|Morphology|BOLD:ACY5073
Parachironomus monochromus|IM_PL7V14_278|Piotr Gadawski|Morphology|BOLD:ACY5073
Parachironomus monochromus|BIOUG55112-A05|Stefan Schmidt|BIN Taxonomy Match (Sep 2020)|BOLD...
Parachironomus monochromus|BIOUG55199-D01|Stefan Schmidt|BIN Taxonomy Match (Sep 2020)|BOLD...
Parachironomus monochromus|BIOUG42697-F08|Kate Perez|BIN Taxonomy Match below Phylum (Jun 2019...
Parachironomus monochromus|BIOUG42740-C03|Kate Perez|BIN Taxonomy Match below Phylum (Jun 2019...
Parachironomus monochromus|BIOUG55189-B01|Stefan Schmidt|BIN Taxonomy Match (Sep 2020)|BOLD...
Parachironomus monochromus|BIOUG55188-C02|Stefan Schmidt|BIN Taxonomy Match (Sep 2020)|BOLD...
Parachironomus monochromus|BIOUG42786-F08|Kate Perez|BIN Taxonomy Match below Phylum (Jun 2019...
Parachironomus monochromus|BIOUG55147-C10|Stefan Schmidt|BIN Taxonomy Match (Sep 2020)|BOLD...
Parachironomus monochromus|BIOUG55193-E07|Stefan Schmidt|BIN Taxonomy Match (Sep 2020)|BOLD...
Parachironomus monochromus|ZMUO.025334|Lauri Paasivirta||BOLD:ACY5073
Parachironomus monochromus|ZMUO.025333|Lauri Paasivirta||BOLD:ACY5073
Parachironomus|ZSM-DIP-33149-H01|Caroline Chimeno|BIN Taxonomy Match (Jan 2022)|BOLD:ACY...
Parachironomus monochromus|BIOUG55417-A08|Stefan Schmidt|BIN Taxonomy Match (Sep 2020)|BOLD...
Parachironomus|ZSM-DIP-33149-E07|Caroline Chimeno|BIN Taxonomy Match (Jan 2022)|BOLD:ACY...
Einfeldia pagana|CCDB21606-B03|Sofia Wiedenbrug||BOLD:AAP6213
Einfeldia pagana|NHRS-BYWS000000974|Yngve Brodin|morphology|BOLD:AAP6213
Einfeldia pagana|NHRS-BYWS000000124|Yngve Brodin|morphology|BOLD:AAP6213
Einfeldia pagana|NHRS-BYWS000000123|Yngve Brodin|morphology|BOLD:AAP6213
Einfeldia pagana|NHRS-BYWS000000122|Yngve Brodin|morphology|BOLD:AAP6213
Einfeldia pagana|ZMUO.025238|Lauri Paasivirta||BOLD:AAP6213
Einfeldia synchrona|TRD-CH357|Elisabeth Stur|morphology|BOLD:AAP6213
Einfeldia pagana|ZMUO.025237|Lauri Paasivirta||BOLD:AAP6213
Einfeldia synchrona|TRD-CH29|Elisabeth Stur||BOLD:AAP6213
Einfeldia synchrona|TRD-CH24|Elisabeth Stur||BOLD:AAP6213
Einfeldia synchrona|Finnmark169|Elisabeth Stur||BOLD:AAP6213
Glyptotendipes paripes|ZMUO.024982|Lauri Paasivirta||BOLD:AAC0597
Glyptotendipes paripes|ZMUO.024981|Lauri Paasivirta||BOLD:AAC0597
Glyptotendipes barbipes|NHRS-BYWS000001053|Yngve Brodin|morphology|BOLD:AAY1045
Glyptotendipes barbipes|NHRS-BYWS000000953|Yngve Brodin|morphology|BOLD:AAY1045
Glyptotendipes barbipes|NHRS-BYWS000001787|Yngve Brodin||BOLD:AAY1045
Glyptotendipes barbipes|NHRS-BYWS000000369|Yngve Brodin|morphology|BOLD:AAY1045

Glyptotendipes barbipes|NHRS-BYWS[illegible]|Yngve Brodin|morphology|BOLD:AAY1045
Glyptotendipes barbipes|NHRS-BYWS000000953|Yngve Brodin|morphology|BOLD:AAY1045
Glyptotendipes barbipes|NHRS-BYWS000001787|Yngve Brodin||BOLD:AAY1045
Glyptotendipes barbipes|NHRS-BYWS000000369|Yngve Brodin|morphology|BOLD:AAY1045
Glyptotendipes barbipes|ZMUO.025272|Lauri Paasivirta||BOLD:AAY1045
Glyptotendipes barbipes|ZMUO.025271|Lauri Paasivirta||BOLD:AAY1045
Glyptotendipes barbipes|NHRS-BYWS000000360|Yngve Brodin|morphology|BOLD:AAY1045
Glyptotendipes barbipes|NHRS-BYWS000000464|Yngve Brodin|morphology|BOLD:AAY1045
Glyptotendipes barbipes|NHRS-BYWS000001181|Yngve Brodin|morphology|BOLD:AAY1045
Parachironomus digitalis|BIOUG16257-C05|Kate Perez|BIN Taxonomy Match|BOLD:AAP6555
Parachironomus digitalis|BIOUG16299-A02|Kate Perez|BIN Taxonomy Match|BOLD:AAP6555
Parachironomus digitalis|BIOUG16329-D01|Kate Perez|BIN Taxonomy Match|BOLD:AAP6555
Parachironomus digitalis|EJV-20110145|Lauri Paasivirta||BOLD:AAP6555
Parachironomus digitalis|ZMUO.024794|Lauri Paasivirta||BOLD:AAP6555
Parachironomus digitalis|ZSM-DIP-33139-F06|Caroline Chimeno|BIN Taxonomy Match (Jan 2022)|B...
Parachironomus digitalis|ZSM-DIP-33139-F08|Caroline Chimeno|BIN Taxonomy Match (Jan 2022)|B...
Parachironomus digitalis|Finnmark176|Torbjorn Ekrem||BOLD:AAP6555
Parachironomus digitalis|ZMUO.024795|Lauri Paasivirta||BOLD:AAP6555
Parachironomus digitalis|NHRS-BYWS000000138|Yngve Brodin|morphology|BOLD:AAP6555
Parachironomus digitalis|BIOUG16260-G04|Kate Perez|BIN Taxonomy Match|BOLD:AAP6555
Parachironomus digitalis|BIOUG16259-E09|Kate Perez|BIN Taxonomy Match|BOLD:AAP6555
Parachironomus digitalis|BIOUG16258-D10|Kate Perez|BIN Taxonomy Match|BOLD:AAP6555
Limnophyes minimus|BIOUG07930-G03|Kate Perez|BIN Taxonomy Match|BOLD:AAA8204
Limnophyes minimus|ZSM-DIP-33131-E05|Caroline Chimeno|BIN Taxonomy Match (Jan 2022)|BOLD ...
Limnophyes minimus|BIOUG15808-F05|Kate Perez|BIN Taxonomy Match|BOLD:AAA8204
Limnophyes minimus|BIOUG15537-F05|Kate Perez|BIN Taxonomy Match|BOLD:AAA8204
Limnophyes minimus|BIOUG42932-A03|Kate Perez|BIN Taxonomy Match below Phylum (Jun 2019)|BOL...
Limnophyes minimus|BIOUG15963-D06|Kate Perez|BIN Taxonomy Match|BOLD:AAA8204
Limnophyes minimus|BIOUG08129-A07|Kate Perez|BIN Taxonomy Match|BOLD:AAA8204
Limnophyes minimus|BIOUG07509-E06|Kate Perez|BIN Taxonomy Match|BOLD:AAA8204
Limnophyes minimus|BIOUG15977-A08|Kate Perez|BIN Taxonomy Match|BOLD:AAA8204
Limnophyes minimus|BIOUG15794-D11|Kate Perez|BIN Taxonomy Match|BOLD:AAA8204
Limnophyes minimus|BIOUG15806-B06|Kate Perez|BIN Taxonomy Match|BOLD:AAA8204
Limnophyes minimus|BIOUG07944-D04|Kate Perez|BIN Taxonomy Match|BOLD:AAA8204
Limnophyes minimus|BIOUG07566-D12|Kate Perez|BIN Taxonomy Match|BOLD:AAA8204
Limnophyes minimus|BIOUG15542-F12|Kate Perez|BIN Taxonomy Match|BOLD:AAA8204
Limnophyes minimus|BIOUG16594-F08|Kate Perez|BIN Taxonomy Match|BOLD:AAA8204
Limnophyes minimus|BIOUG36726-H06|Kate Perez|BIN Taxonomy Match (May 2018)|BOLD:AAA8204
Limnophyes minimus|BIOUG15544-C04|Kate Perez|BIN Taxonomy Match|BOLD:AAA8204
Limnophyes minimus|BIOUG08255-G04|Kate Perez|BIN Taxonomy Match|BOLD:AAA8204
Limnophyes minimus|BIOUG06100-G04|Kate Perez|BIN Taxonomy Match|BOLD:AAA8204
Limnophyes minimus|BIOUG05947-B08|Kate Perez|BIN Taxonomy Match|BOLD:AAA8204
Limnophyes minimus|BIOUG06065-H03|Kate Perez|BIN Taxonomy Match|BOLD:AAA8204
Limnophyes minimus|BIOUG15951-A06|Kate Perez|BIN Taxonomy Match|BOLD:AAA8204
Limnophyes minimus|BIOUG06054-A10|Kate Perez|BIN Taxonomy Match|BOLD:AAA8204
Limnophyes minimus|BIOUG06054-H04|Kate Perez|BIN Taxonomy Match|BOLD:AAA8204
Limnophyes minimus|BIOUG15538-A01|Kate Perez|BIN Taxonomy Match|BOLD:AAA8204
Limnophyes minimus|BIOUG15539-F01|Kate Perez|BIN Taxonomy Match|BOLD:AAA8204
Limnophyes minimus|BIOUG05900-E07|Kate Perez|BIN Taxonomy Match|BOLD:AAA8204
Limnophyes minimus|BIOUG05900-G01|Kate Perez|BIN Taxonomy Match|BOLD:AAA8204
Limnophyes minimus|BIOUG15458-A06|Kate Perez|BIN Taxonomy Match|BOLD:AAA8204
Limnophyes minimus|BIOUG06053-E09|Kate Perez|BIN Taxonomy Match|BOLD:AAA8204
Limnophyes minimus|BIOUG05780-A03|Kate Perez|BIN Taxonomy Match|BOLD:AAA8204
Limnophyes minimus|BIOUG05944-F03|Kate Perez|BIN Taxonomy Match|BOLD:AAA8204
Limnophyes minimus|BIOUG05945-B05|Kate Perez|BIN Taxonomy Match|BOLD:AAA8204
Limnophyes minimus|BIOUG15539-C12|Kate Perez|BIN Taxonomy Match|BOLD:AAA8204
Limnophyes minimus|BIOUG07405-B12|Kate Perez|BIN Taxonomy Match|BOLD:AAA8204
Limnophyes minimus|CCDB24038-A06|Sofia Wiedenbrug|BIN Taxonomy Match|BOLD:AAA8204
Limnophyes minimus|BIOUG15543-G12|Kate Perez|BIN Taxonomy Match|BOLD:AAA8204
Limnophyes minimus|BIOUG15561-C12|Kate Perez|BIN Taxonomy Match|BOLD:AAA8204
Limnophyes minimus|BIOUG15561-H04|Kate Perez|BIN Taxonomy Match|BOLD:AAA8204
Limnophyes minimus|BIOUG15795-F03|Kate Perez|BIN Taxonomy Match|BOLD:AAA8204
Limnophyes minimus|BIOUG15795-F11|Kate Perez|BIN Taxonomy Match|BOLD:AAA8204
Limnophyes minimus|BIOUG16596-A10|Kate Perez|BIN Taxonomy Match|BOLD:AAA8204
Limnophyes minimus|BIOUG16597-D07|Kate Perez|BIN Taxonomy Match|BOLD:AAA8204
Limnophyes minimus|BIOUG15651-A09|Kate Perez|BIN Taxonomy Match|BOLD:AAA8204
Limnophyes minimus|CCDB24183-H10|Sofia Wiedenbrug|BIN-taxonomy|BOLD:AAA8204
Limnophyes minimus|BIOUG08319-H05|Kate Perez|BIN Taxonomy Match|BOLD:AAA8204
Limnophyes minimus|BIOUG15977-D05|Kate Perez|BIN Taxonomy Match|BOLD:AAA8204
Limnophyes minimus|BIOUG15464-E04|Kate Perez|BIN Taxonomy Match|BOLD:AAA8204
Limnophyes minimus|BIOUG15544-G06|Kate Perez|BIN Taxonomy Match|BOLD:AAA8204
Limnophyes minimus|BIOUG05900-D03|Kate Perez|BIN Taxonomy Match|BOLD:AAA8204
Limnophyes minimus|BIOUG16214-F12|Kate Perez|BIN Taxonomy Match|BOLD:AAA8204
Limnophyes minimus|BIOUG04437-H01|Kate Perez|BIN Taxonomy Match|BOLD:AAA8204
Limnophyes minimus|BIOUG04437-D11|Kate Perez|BIN Taxonomy Match|BOLD:AAA8204
Limnophyes minimus|BIOUG06053-G06|Kate Perez|BIN Taxonomy Match|BOLD:AAA8204
Limnophyes minimus|BIOUG05944-A12|Kate Perez|BIN Taxonomy Match|BOLD:AAA8204
Limnophyes minimus|ZSM-DIP-33148-B09|Caroline Chimeno|BIN Taxonomy Match (Jan 2022)|BOLD ...
Limnophyes minimus|ZSM-DIP-33148-B11|Caroline Chimeno|BIN Taxonomy Match (Jan 2022)|BOLD ...
Limnophyes minimus|BIOUG42946-G01|Kate Perez|BIN Taxonomy Match below Phylum (Jun 2019)|BOL...
Limnophyes minimus|BIOUG08259-F11|Kate Perez|BIN Taxonomy Match|BOLD:AAA8204
Limnophyes minimus|BIOUG15455-F09|Kate Perez|BIN Taxonomy Match|BOLD:AAA8204
Limnophyes minimus|BIOUG15456-B05|Kate Perez|BIN Taxonomy Match|BOLD:AAA8204
Limnophyes minimus|BIOUG15761-E10|Kate Perez|BIN Taxonomy Match|BOLD:AAA8204
Limnophyes minimus|BIOUG05396-H07|Kate Perez|BIN Taxonomy Match|BOLD:AAA8204
Limnophyes minimus|BIOUG15765-D05|Kate Perez|BIN Taxonomy Match|BOLD:AAA8204
Limnophyes minimus|BIOUG15799-C06|Kate Perez|BIN Taxonomy Match|BOLD:AAA8204
Limnophyes minimus|BIOUG05778-C09|Kate Perez|BIN Taxonomy Match|BOLD:AAA8204
Limnophyes minimus|BIOUG15543-A12|Kate Perez|BIN Taxonomy Match|BOLD:AAA8204
Limnophyes minimus|BIOUG08127-F05|Kate Perez|BIN Taxonomy Match|BOLD:AAA8204
Limnophyes minimus|BIOUG15791-H05|Kate Perez|BIN Taxonomy Match|BOLD:AAA8204
Limnophyes minimus|BIOUG16208-C04|Kate Perez|BIN Taxonomy Match|BOLD:AAA8204
Limnophyes minimus|BIOUG15792-F08|Kate Perez|BIN Taxonomy Match|BOLD:AAA8204
Limnophyes minimus|BIOUG15807-F04|Kate Perez|BIN Taxonomy Match|BOLD:AAA8204
Limnophyes minimus|BIOUG15808-C08|Kate Perez|BIN Taxonomy Match|BOLD:AAA8204
Limnophyes minimus|BIOUG15808-E06|Kate Perez|BIN Taxonomy Match|BOLD:AAA8204
Limnophyes minimus|BIOUG15808-G09|Kate Perez|BIN Taxonomy Match|BOLD:AAA8204
Limnophyes minimus|BIOUG16551-E04|Kate Perez|BIN Taxonomy Match|BOLD:AAA8204
Limnophyes minimus|BIOUG16214-E09|Kate Perez|BIN Taxonomy Match|BOLD:AAA8204
Limnophyes minimus|BIOUG15454-D02|Kate Perez|BIN Taxonomy Match|BOLD:AAA8204
Limnophyes minimus|BIOUG07755-C08|Kate Perez|BIN Taxonomy Match|BOLD:AAA8204
Limnophyes minimus|BIOUG16596-F12|Kate Perez|BIN Taxonomy Match|BOLD:AAA8204
Limnophyes minimus|BIOUG16597-E07|Kate Perez|BIN Taxonomy Match|BOLD:AAA8204
[illegible]

Limnophyes minimus|BIOUG07755-C08|Kate Perez|BIN Taxonomy Match|BOLD:AAA8204
Limnophyes minimus|BIOUG16596-F12|Kate Perez|BIN Taxonomy Match|BOLD:AAA8204
Limnophyes minimus|BIOUG16597-E07|Kate Perez|BIN Taxonomy Match|BOLD:AAA8204
Limnophyes minimus|BIOUG07758-E05|Kate Perez|BIN Taxonomy Match|BOLD:AAA8204
Limnophyes minimus|BIOUG07414-H04|Kate Perez|BIN Taxonomy Match|BOLD:AAA8204
Limnophyes minimus|BIOUG15855-G04|Kate Perez|BIN Taxonomy Match|BOLD:AAA8204
Limnophyes minimus|BIOUG15455-G02|Kate Perez|BIN Taxonomy Match|BOLD:AAA8204
Limnophyes minimus|BIOUG07410-E11|Kate Perez|BIN Taxonomy Match|BOLD:AAA8204
Limnophyes minimus|BIOUG15185-D06|Kate Perez|BIN Taxonomy Match|BOLD:AAA8204
Limnophyes minimus|BIOUG06280-F10|Kate Perez|BIN Taxonomy Match|BOLD:AAA8204
Limnophyes minimus|BIOUG05945-E05|Kate Perez|BIN Taxonomy Match|BOLD:AAA8204
Limnophyes minimus|BIOUG16594-F07|Kate Perez|BIN Taxonomy Match|BOLD:AAA8204
Limnophyes minimus|BIOUG08174-B02|Kate Perez|BIN Taxonomy Match|BOLD:AAA8204
Limnophyes minimus|BIOUG15538-D04|Kate Perez|BIN Taxonomy Match|BOLD:AAA8204
Limnophyes minimus|BIOUG05110-H02|Kate Perez|BIN Taxonomy Match|BOLD:AAA8204
Limnophyes minimus|BIOUG05141-C05|Kate Perez|BIN Taxonomy Match|BOLD:AAA8204
Limnophyes minimus|BIOUG05141-E02|Kate Perez|BIN Taxonomy Match|BOLD:AAA8204
Limnophyes minimus|BIOUG04114-C10|Kate Perez|BIN Taxonomy Match|BOLD:AAA8204
Limnophyes minimus|BIOUG04366-B08|Kate Perez|BIN Taxonomy Match|BOLD:AAA8204
Limnophyes minimus|BIOUG05109-D03|Kate Perez|BIN Taxonomy Match|BOLD:AAA8204
Limnophyes minimus|BIOUG15542-D10|Kate Perez|BIN Taxonomy Match|BOLD:AAA8204
Limnophyes minimus|BIOUG05144-D02|Kate Perez|BIN Taxonomy Match|BOLD:AAA8204
Limnophyes minimus|BIOUG04366-G07|Kate Perez|BIN Taxonomy Match|BOLD:AAA8204
Limnophyes minimus|BIOUG05945-E09|Kate Perez|BIN Taxonomy Match|BOLD:AAA8204
Limnophyes minimus|CCDB24183-G09|Sofia Wiedenbrug|BIN-taxonomy|BOLD:AAA8204
Limnophyes minimus|CCDB24229-B01|Sofia Wiedenbrug|BIN Taxonomy Match|BOLD:AAA8204
Limnophyes minimus|BIOUG16552-A12|Kate Perez|BIN Taxonomy Match|BOLD:AAA8204
Limnophyes minimus|BIOUG16596-F01|Kate Perez|BIN Taxonomy Match|BOLD:AAA8204
Limnophyes minimus|BIOUG16597-E11|Kate Perez|BIN Taxonomy Match|BOLD:AAA8204
Limnophyes minimus|BIOUG05141-D10|Kate Perez|BIN Taxonomy Match|BOLD:AAA8204
Limnophyes minimus|BIOUG04212-D05|Kate Perez|BIN Taxonomy Match|BOLD:AAA8204
Limnophyes minimus|BIOUG04366-G01|Kate Perez|BIN Taxonomy Match|BOLD:AAA8204
Limnophyes minimus|BIOUG04153-G06|Kate Perez|BIN Taxonomy Match|BOLD:AAA8204
Limnophyes minimus|BIOUG04155-E04|Kate Perez|BIN Taxonomy Match|BOLD:AAA8204
Limnophyes minimus|BIOUG15853-C01|Kate Perez|BIN Taxonomy Match|BOLD:AAA8204
Limnophyes minimus|BIOUG04153-D03|Kate Perez|BIN Taxonomy Match|BOLD:AAA8204
Limnophyes minimus|ZMUO.024265|Lauri Paasivirta||BOLD:AAA8204
Limnophyes minimus|BIOUG08260-D12|Kate Perez|BIN Taxonomy Match|BOLD:AAA8204
Limnophyes minimus|BIOUG15458-D05|Kate Perez|BIN Taxonomy Match|BOLD:AAA8204
Limnophyes minimus|BIOUG16597-F03|Kate Perez|BIN Taxonomy Match|BOLD:AAA8204
Limnophyes minimus|BIOUG15720-C11|Kate Perez|BIN Taxonomy Match|BOLD:AAA8204
Limnophyes minimus|BIOUG15795-A02|Kate Perez|BIN Taxonomy Match|BOLD:AAA8204
Limnophyes minimus|BIOUG15795-D11|Kate Perez|BIN Taxonomy Match|BOLD:AAA8204
Limnophyes minimus|BIOUG15796-B06|Kate Perez|BIN Taxonomy Match|BOLD:AAA8204
Limnophyes minimus|BIOUG16596-E10|Kate Perez|BIN Taxonomy Match|BOLD:AAA8204
Limnophyes minimus|BIOUG16597-E06|Kate Perez|BIN Taxonomy Match|BOLD:AAA8204
Limnophyes minimus|BIOUG17066-D10|Kate Perez|BIN Taxonomy Match|BOLD:AAA8204
Limnophyes minimus|BIOUG15763-B12|Kate Perez|BIN Taxonomy Match|BOLD:AAA8204
Limnophyes minimus|BIOUG15763-C06|Kate Perez|BIN Taxonomy Match|BOLD:AAA8204
Limnophyes minimus|BIOUG15763-D07|Kate Perez|BIN Taxonomy Match|BOLD:AAA8204
Limnophyes minimus|BIOUG08316-D05|Kate Perez|BIN Taxonomy Match|BOLD:AAA8204
Limnophyes minimus|BIOUG15718-H06|Kate Perez|BIN Taxonomy Match|BOLD:AAA8204
Limnophyes minimus|BIOUG15720-G10|Kate Perez|BIN Taxonomy Match|BOLD:AAA8204
Limnophyes minimus|BIOUG15762-F05|Kate Perez|BIN Taxonomy Match|BOLD:AAA8204
Limnophyes minimus|BIOUG15765-B09|Kate Perez|BIN Taxonomy Match|BOLD:AAA8204
Limnophyes minimus|BIOUG15766-G07|Kate Perez|BIN Taxonomy Match|BOLD:AAA8204
Limnophyes minimus|BIOUG08098-F10|Kate Perez|BIN Taxonomy Match|BOLD:AAA8204
Limnophyes minimus|BIOUG15541-G02|Kate Perez|BIN Taxonomy Match|BOLD:AAA8204
Limnophyes minimus|BIOUG05778-C05|Kate Perez|BIN Taxonomy Match|BOLD:AAA8204
Limnophyes minimus|BIOUG15540-G12|Kate Perez|BIN Taxonomy Match|BOLD:AAA8204
Limnophyes minimus|BIOUG15465-A12|Kate Perez|BIN Taxonomy Match|BOLD:AAA8204
Limnophyes minimus|BIOUG08132-G06|Kate Perez|BIN Taxonomy Match|BOLD:AAA8204
Limnophyes minimus|BIOUG08134-E05|Kate Perez|BIN Taxonomy Match|BOLD:AAA8204
Limnophyes minimus|BIOUG15795-B11|Kate Perez|BIN Taxonomy Match|BOLD:AAA8204
Limnophyes minimus|BIOUG15796-F04|Kate Perez|BIN Taxonomy Match|BOLD:AAA8204
Limnophyes minimus|BIOUG15797-C11|Kate Perez|BIN Taxonomy Match|BOLD:AAA8204
Limnophyes minimus|BIOUG16596-G09|Kate Perez|BIN Taxonomy Match|BOLD:AAA8204
Limnophyes minimus|BIOUG07928-H01|Kate Perez|BIN Taxonomy Match|BOLD:AAA8204
Limnophyes minimus|BIOUG15766-G08|Kate Perez|BIN Taxonomy Match|BOLD:AAA8204
Limnophyes minimus|BIOUG16598-B03|Kate Perez|BIN Taxonomy Match|BOLD:AAA8204
Limnophyes minimus|BIOUG07566-A02|Kate Perez|BIN Taxonomy Match|BOLD:AAA8204
Limnophyes minimus|BIOUG07566-B09|Kate Perez|BIN Taxonomy Match|BOLD:AAA8204
Limnophyes minimus|BIOUG07563-B08|Kate Perez|BIN Taxonomy Match|BOLD:AAA8204
Limnophyes minimus|BIOUG07760-B10|Kate Perez|BIN Taxonomy Match|BOLD:AAA8204
Limnophyes minimus|BIOUG15653-B12|Kate Perez|BIN Taxonomy Match|BOLD:AAA8204
Limnophyes minimus|BIOUG07410-E01|Kate Perez|BIN Taxonomy Match|BOLD:AAA8204
Limnophyes minimus|BIOUG16426-G05|Kate Perez|BIN Taxonomy Match|BOLD:AAA8204
Limnophyes minimus|BIOUG16433-H05|Kate Perez|BIN Taxonomy Match|BOLD:AAA8204
Limnophyes minimus|BIOUG16434-A05|Kate Perez|BIN Taxonomy Match|BOLD:AAA8204
Limnophyes minimus|BIOUG16435-E05|Kate Perez|BIN Taxonomy Match|BOLD:AAA8204
Limnophyes minimus|BIOUG16431-G12|Kate Perez|BIN Taxonomy Match|BOLD:AAA8204
Limnophyes minimus|BIOUG16432-G06|Kate Perez|BIN Taxonomy Match|BOLD:AAA8204
Limnophyes minimus|BIOUG16432-H10|Kate Perez|BIN Taxonomy Match|BOLD:AAA8204
Limnophyes minimus|BIOUG16477-F02|Kate Perez|BIN Taxonomy Match|BOLD:AAA8204
Limnophyes minimus|BIOUG16708-H11|Kate Perez|BIN Taxonomy Match|BOLD:AAA8204
Limnophyes minimus|BIOUG16626-A04|Kate Perez|BIN Taxonomy Match|BOLD:AAA8204
Limnophyes minimus|BIOUG16996-C08|Kate Perez|BIN Taxonomy Match|BOLD:AAA8204
Limnophyes minimus|BIOUG06053-E08|Kate Perez|BIN Taxonomy Match|BOLD:AAA8204
Limnophyes minimus|ZSM34343-F08|Sofia Wiedenbrug|Morphology|BOLD:AAA8204
Limnophyes minimus|BIOUG15769-E08|Kate Perez|BIN Taxonomy Match|BOLD:AAA8204
Limnophyes minimus|BIOUG15947-E02|Kate Perez|BIN Taxonomy Match|BOLD:AAA8204
Limnophyes minimus|BIOUG08127-F12|Kate Perez|BIN Taxonomy Match|BOLD:AAA8204
Limnophyes minimus|BIOUG15966-B10|Kate Perez|BIN Taxonomy Match|BOLD:AAA8204
Limnophyes minimus|BIOUG08125-C08|Kate Perez|BIN Taxonomy Match|BOLD:AAA8204
Limnophyes minimus|BIOUG06443-G11|Kate Perez|BIN Taxonomy Match|BOLD:AAA8204
Limnophyes minimus|BIOUG16436-A07|Kate Perez|BIN Taxonomy Match|BOLD:AAA8204
Limnophyes minimus|BIOUG15651-A08|Kate Perez|BIN Taxonomy Match|BOLD:AAA8204
Limnophyes minimus|BIOUG43081-E10|Kate Perez|BIN Taxonomy Match below Phylum (Jun 2019)|BOL...
Limnophyes minimus|BIOUG07757-G11|Kate Perez|BIN Taxonomy Match|BOLD:AAA8204
Limnophyes minimus|ZSM-DIP-33137-F02|Caroline Chimeno|BIN Taxonomy Match (Jan 2022)|BOLD ...
Limnophyes minimus|BIOUG07757-B03|Kate Perez|BIN Taxonomy Match|BOLD:AAA8204
Limnophyes minimus|BIOUG07504-C02|Kate Perez|BIN Taxonomy Match|BOLD:AAA8204
Limnophyes minimus|BIOUG04089-A08|Kate Perez|BIN Taxonomy Match|BOLD:AAA8204

Limnophyes minimus|BIOUG07757-B03|Kate Perez|BIN Taxonomy Match|BOLD:AAA8204
Limnophyes minimus|BIOUG07504-C02|Kate Perez|BIN Taxonomy Match|BOLD:AAA8204
Limnophyes minimus|BIOUG04089-A08|Kate Perez|BIN Taxonomy Match|BOLD:AAA8204
Limnophyes minimus|BIOUG05110-B05|Kate Perez|BIN Taxonomy Match|BOLD:AAA8204
Limnophyes minimus|BIOUG05110-G05|Kate Perez|BIN Taxonomy Match|BOLD:AAA8204
Limnophyes minimus|BIOUG06090-F12|Kate Perez|BIN Taxonomy Match|BOLD:AAA8204
Limnophyes minimus|BIOUG04090-E11|Kate Perez|BIN Taxonomy Match|BOLD:AAA8204
Limnophyes minimus|BIOUG05110-A04|Kate Perez|BIN Taxonomy Match|BOLD:AAA8204
Limnophyes minimus|BIOUG16601-C04|Kate Perez|BIN Taxonomy Match|BOLD:AAA8204
Limnophyes minimus|BIOUG06093-G06|Kate Perez|BIN Taxonomy Match|BOLD:AAA8204
Limnophyes minimus|BIOUG05144-B06|Kate Perez|BIN Taxonomy Match|BOLD:AAA8204
Limnophyes minimus|BIOUG04155-G06|Kate Perez|BIN Taxonomy Match|BOLD:AAA8204
Limnophyes minimus|BIOUG04155-G12|Kate Perez|BIN Taxonomy Match|BOLD:AAA8204
Limnophyes minimus|BIOUG15853-C10|Kate Perez|BIN Taxonomy Match|BOLD:AAA8204
Limnophyes minimus|BIOUG15763-D10|Kate Perez|BIN Taxonomy Match|BOLD:AAA8204
Limnophyes minimus|BIOUG15763-G01|Kate Perez|BIN Taxonomy Match|BOLD:AAA8204
Limnophyes minimus|BIOUG15764-A02|Kate Perez|BIN Taxonomy Match|BOLD:AAA8204
Limnophyes minimus|BIOUG15764-A03|Kate Perez|BIN Taxonomy Match|BOLD:AAA8204
Limnophyes minimus|BIOUG15764-C01|Kate Perez|BIN Taxonomy Match|BOLD:AAA8204
Limnophyes minimus|BIOUG15764-F11|Kate Perez|BIN Taxonomy Match|BOLD:AAA8204
Limnophyes minimus|BIOUG15764-G03|Kate Perez|BIN Taxonomy Match|BOLD:AAA8204
Limnophyes minimus|BIOUG15718-F12|Kate Perez|BIN Taxonomy Match|BOLD:AAA8204
Limnophyes minimus|BIOUG15719-A04|Kate Perez|BIN Taxonomy Match|BOLD:AAA8204
Limnophyes minimus|BIOUG15719-E08|Kate Perez|BIN Taxonomy Match|BOLD:AAA8204
Limnophyes minimus|BIOUG15719-F02|Kate Perez|BIN Taxonomy Match|BOLD:AAA8204
Limnophyes minimus|BIOUG15719-F04|Kate Perez|BIN Taxonomy Match|BOLD:AAA8204
Limnophyes minimus|BIOUG15719-G12|Kate Perez|BIN Taxonomy Match|BOLD:AAA8204
Limnophyes minimus|BIOUG15720-G09|Kate Perez|BIN Taxonomy Match|BOLD:AAA8204
Limnophyes minimus|BIOUG15761-F11|Kate Perez|BIN Taxonomy Match|BOLD:AAA8204
Limnophyes minimus|BIOUG15762-G03|Kate Perez|BIN Taxonomy Match|BOLD:AAA8204
Limnophyes minimus|BIOUG15764-H03|Kate Perez|BIN Taxonomy Match|BOLD:AAA8204
Limnophyes minimus|BIOUG15765-G07|Kate Perez|BIN Taxonomy Match|BOLD:AAA8204
Limnophyes minimus|BIOUG15765-G08|Kate Perez|BIN Taxonomy Match|BOLD:AAA8204
Limnophyes minimus|BIOUG08259-D05|Kate Perez|BIN Taxonomy Match|BOLD:AAA8204
Limnophyes minimus|BIOUG15769-D02|Kate Perez|BIN Taxonomy Match|BOLD:AAA8204
Limnophyes minimus|BIOUG07647-B11|Kate Perez|BIN Taxonomy Match|BOLD:AAA8204
Limnophyes minimus|BIOUG08095-H08|Kate Perez|BIN Taxonomy Match|BOLD:AAA8204
Limnophyes minimus|BIOUG15949-C06|Kate Perez|BIN Taxonomy Match|BOLD:AAA8204
Limnophyes minimus|BIOUG08126-D09|Kate Perez|BIN Taxonomy Match|BOLD:AAA8204
Limnophyes minimus|BIOUG05776-F04|Kate Perez|BIN Taxonomy Match|BOLD:AAA8204
Limnophyes minimus|BIOUG05776-F06|Kate Perez|BIN Taxonomy Match|BOLD:AAA8204
Limnophyes minimus|BIOUG05776-G04|Kate Perez|BIN Taxonomy Match|BOLD:AAA8204
Limnophyes minimus|BIOUG08138-E06|Kate Perez|BIN Taxonomy Match|BOLD:AAA8204
Limnophyes minimus|BIOUG08130-E02|Kate Perez|BIN Taxonomy Match|BOLD:AAA8204
Limnophyes minimus|BIOUG08136-C10|Kate Perez|BIN Taxonomy Match|BOLD:AAA8204
Limnophyes minimus|BIOUG08137-C04|Kate Perez|BIN Taxonomy Match|BOLD:AAA8204
Limnophyes minimus|BIOUG08137-E09|Kate Perez|BIN Taxonomy Match|BOLD:AAA8204
Limnophyes minimus|BIOUG15806-B07|Kate Perez|BIN Taxonomy Match|BOLD:AAA8204
Limnophyes minimus|BIOUG15564-B12|Kate Perez|BIN Taxonomy Match|BOLD:AAA8204
Limnophyes minimus|BIOUG16207-G04|Kate Perez|BIN Taxonomy Match|BOLD:AAA8204
Limnophyes minimus|BIOUG08256-C06|Kate Perez|BIN Taxonomy Match|BOLD:AAA8204
Limnophyes minimus|BIOUG15794-B10|Kate Perez|BIN Taxonomy Match|BOLD:AAA8204
Limnophyes minimus|BIOUG15797-C01|Kate Perez|BIN Taxonomy Match|BOLD:AAA8204
Limnophyes minimus|BIOUG15798-B09|Kate Perez|BIN Taxonomy Match|BOLD:AAA8204
Limnophyes minimus|BIOUG15807-E12|Kate Perez|BIN Taxonomy Match|BOLD:AAA8204
Limnophyes minimus|BIOUG15765-A03|Kate Perez|BIN Taxonomy Match|BOLD:AAA8204
Limnophyes minimus|BIOUG15808-B10|Kate Perez|BIN Taxonomy Match|BOLD:AAA8204
Limnophyes minimus|BIOUG15809-C10|Kate Perez|BIN Taxonomy Match|BOLD:AAA8204
Limnophyes minimus|BIOUG15809-E10|Kate Perez|BIN Taxonomy Match|BOLD:AAA8204
Limnophyes minimus|BIOUG15809-F07|Kate Perez|BIN Taxonomy Match|BOLD:AAA8204
Limnophyes minimus|BIOUG07753-F07|Kate Perez|BIN Taxonomy Match|BOLD:AAA8204
Limnophyes minimus|BIOUG08367-C01|Kate Perez|BIN Taxonomy Match|BOLD:AAA8204
Limnophyes minimus|BIOUG07753-D09|Kate Perez|BIN Taxonomy Match|BOLD:AAA8204
Limnophyes minimus|BIOUG07754-C04|Kate Perez|BIN Taxonomy Match|BOLD:AAA8204
Limnophyes minimus|BIOUG07755-B04|Kate Perez|BIN Taxonomy Match|BOLD:AAA8204
Limnophyes minimus|BIOUG07757-B09|Kate Perez|BIN Taxonomy Match|BOLD:AAA8204
Limnophyes minimus|BIOUG07760-D12|Kate Perez|BIN Taxonomy Match|BOLD:AAA8204
Limnophyes minimus|BIOUG07758-B06|Kate Perez|BIN Taxonomy Match|BOLD:AAA8204
Limnophyes minimus|BIOUG07758-G01|Kate Perez|BIN Taxonomy Match|BOLD:AAA8204
Limnophyes minimus|BIOUG07758-H04|Kate Perez|BIN Taxonomy Match|BOLD:AAA8204
Limnophyes minimus|BIOUG07759-E05|Kate Perez|BIN Taxonomy Match|BOLD:AAA8204
Limnophyes minimus|BIOUG07563-E10|Kate Perez|BIN Taxonomy Match|BOLD:AAA8204
Limnophyes minimus|BIOUG15454-C05|Kate Perez|BIN Taxonomy Match|BOLD:AAA8204
Limnophyes minimus|BIOUG07569-A05|Kate Perez|BIN Taxonomy Match|BOLD:AAA8204
Limnophyes minimus|BIOUG07757-G09|Kate Perez|BIN Taxonomy Match|BOLD:AAA8204
Limnophyes minimus|BIOUG07759-B09|Kate Perez|BIN Taxonomy Match|BOLD:AAA8204
Limnophyes minimus|BIOUG15459-F08|Kate Perez|BIN Taxonomy Match|BOLD:AAA8204
Limnophyes minimus|BIOUG15460-D02|Kate Perez|BIN Taxonomy Match|BOLD:AAA8204
Limnophyes minimus|BIOUG07759-H07|Kate Perez|BIN Taxonomy Match|BOLD:AAA8204
Limnophyes minimus|BIOUG07760-D02|Kate Perez|BIN Taxonomy Match|BOLD:AAA8204
Limnophyes minimus|BIOUG07760-E07|Kate Perez|BIN Taxonomy Match|BOLD:AAA8204
Limnophyes minimus|BIOUG05486-G04|Kate Perez|BIN Taxonomy Match|BOLD:AAA8204
Limnophyes minimus|BIOUG08094-E05|Kate Perez|BIN Taxonomy Match|BOLD:AAA8204
Limnophyes minimus|BIOUG15855-E03|Kate Perez|BIN Taxonomy Match|BOLD:AAA8204
Limnophyes minimus|BIOUG15855-H01|Kate Perez|BIN Taxonomy Match|BOLD:AAA8204
Limnophyes minimus|BIOUG08095-A12|Kate Perez|BIN Taxonomy Match|BOLD:AAA8204
Limnophyes minimus|BIOUG15455-A06|Kate Perez|BIN Taxonomy Match|BOLD:AAA8204
Limnophyes minimus|BIOUG15455-H06|Kate Perez|BIN Taxonomy Match|BOLD:AAA8204
Limnophyes minimus|BIOUG15456-B11|Kate Perez|BIN Taxonomy Match|BOLD:AAA8204
Limnophyes minimus|BIOUG08211-B11|Kate Perez|BIN Taxonomy Match|BOLD:AAA8204
Limnophyes minimus|BIOUG15187-D01|Kate Perez|BIN Taxonomy Match|BOLD:AAA8204
Limnophyes minimus|BIOUG07409-G07|Kate Perez|BIN Taxonomy Match|BOLD:AAA8204
Limnophyes minimus|BIOUG07568-B12|Kate Perez|BIN Taxonomy Match|BOLD:AAA8204
Limnophyes minimus|BIOUG07414-A10|Kate Perez|BIN Taxonomy Match|BOLD:AAA8204
Limnophyes minimus|BIOUG07414-D10|Kate Perez|BIN Taxonomy Match|BOLD:AAA8204
Limnophyes minimus|BIOUG16430-D09|Kate Perez|BIN Taxonomy Match|BOLD:AAA8204
Limnophyes minimus|BIOUG16431-H11|Kate Perez|BIN Taxonomy Match|BOLD:AAA8204
Limnophyes minimus|BIOUG16733-D05|Kate Perez|BIN Taxonomy Match|BOLD:AAA8204
Limnophyes minimus|BIOUG16736-E06|Kate Perez|BIN Taxonomy Match|BOLD:AAA8204
Limnophyes minimus|BIOUG36655-C08|Kate Perez|BIN Taxonomy Match (Jul 2018)|BOLD:AAA8204
Limnophyes minimus|BIOUG36678-E03|Kate Perez|BIN Taxonomy Match (Jul 2018)|BOLD:AAA8204
Limnophyes minimus|BIOUG36677-D03|Kate Perez|BIN Taxonomy Match (May 2018)|BOLD:AAA8204
Limnophyes minimus|BIOUG36776-G01|Kate Perez|BIN Taxonomy Match (Jul 2018)|BOLD:AAA8204

[illegible]
Limnophyes minimus|BIOUG36678-E03|Kate Perez|BIN Taxonomy Match (Jul 2018)|BOLD:AAA8204
Limnophyes minimus|BIOUG36677-D03|Kate Perez|BIN Taxonomy Match (May 2018)|BOLD:AAA8204
Limnophyes minimus|BIOUG36776-G01|Kate Perez|BIN Taxonomy Match (Jul 2018)|BOLD:AAA8204
Limnophyes minimus|BIOUG36779-A01|Kate Perez|BIN Taxonomy Match (May 2018)|BOLD:AAA8204
Limnophyes minimus|BIOUG15792-B09|Kate Perez|BIN Taxonomy Match|BOLD:AAA8204
Limnophyes minimus|BIOUG42758-B03|Kate Perez|BIN Taxonomy Match below Phylum (Jun 2019)|BOL...
Limnophyes minimus|BIOUG16597-C04|Kate Perez|BIN Taxonomy Match|BOLD:AAA8204
Limnophyes minimus|BIOUG07567-G01|Kate Perez|BIN Taxonomy Match|BOLD:AAA8204
Limnophyes minimus|BIOUG08320-C12|Kate Perez|BIN Taxonomy Match|BOLD:AAA8204
Limnophyes minimus|BIOUG15457-F10|Kate Perez|BIN Taxonomy Match|BOLD:AAA8204
Limnophyes minimus|BIOUG07928-C05|Kate Perez|BIN Taxonomy Match|BOLD:AAA8204
Limnophyes minimus|BIOUG06208-C08|Kate Perez|BIN Taxonomy Match|BOLD:AAA8204
Limnophyes minimus|BIOUG06208-D09|Kate Perez|BIN Taxonomy Match|BOLD:AAA8204
Limnophyes minimus|BIOUG06208-H03|Kate Perez|BIN Taxonomy Match|BOLD:AAA8204
Limnophyes minimus|BIOUG04437-C04|Kate Perez|BIN Taxonomy Match|BOLD:AAA8204
Limnophyes minimus|BIOUG04795-F06|Kate Perez|BIN Taxonomy Match|BOLD:AAA8204
Limnophyes minimus|BIOUG05947-D09|Kate Perez|BIN Taxonomy Match|BOLD:AAA8204
Limnophyes minimus|BIOUG05107-B02|Kate Perez|BIN Taxonomy Match|BOLD:AAA8204
Limnophyes minimus|BIOUG08320-G10|Kate Perez|BIN Taxonomy Match|BOLD:AAA8204
Limnophyes minimus|BIOUG05102-F09|Kate Perez|BIN Taxonomy Match|BOLD:AAA8204
Limnophyes minimus|BIOUG05103-C11|Kate Perez|BIN Taxonomy Match|BOLD:AAA8204
Limnophyes minimus|BIOUG06053-G03|Kate Perez|BIN Taxonomy Match|BOLD:AAA8204
Limnophyes minimus|BIOUG05105-E02|Kate Perez|BIN Taxonomy Match|BOLD:AAA8204
Limnophyes minimus|BIOUG15464-H09|Kate Perez|BIN Taxonomy Match|BOLD:AAA8204
Limnophyes minimus|BIOUG06058-B10|Kate Perez|BIN Taxonomy Match|BOLD:AAA8204
Limnophyes minimus|BIOUG06062-D02|Kate Perez|BIN Taxonomy Match|BOLD:AAA8204
Limnophyes minimus|BIOUG04090-D08|Kate Perez|BIN Taxonomy Match|BOLD:AAA8204
Limnophyes minimus|BIOUG05109-H03|Kate Perez|BIN Taxonomy Match|BOLD:AAA8204
Limnophyes minimus|BIOUG04085-F03|Kate Perez|BIN Taxonomy Match|BOLD:AAA8204
Limnophyes minimus|BIOUG05141-E11|Kate Perez|BIN Taxonomy Match|BOLD:AAA8204
Limnophyes minimus|BIOUG05141-H01|Kate Perez|BIN Taxonomy Match|BOLD:AAA8204
Limnophyes minimus|BIOUG04212-A07|Kate Perez|BIN Taxonomy Match|BOLD:AAA8204
Limnophyes minimus|BIOUG04212-E03|Kate Perez|BIN Taxonomy Match|BOLD:AAA8204
Limnophyes minimus|BIOUG04216-C05|Kate Perez|BIN Taxonomy Match|BOLD:AAA8204
Limnophyes minimus|BIOUG04085-B10|Kate Perez|BIN Taxonomy Match|BOLD:AAA8204
Limnophyes minimus|BIOUG04089-G03|Kate Perez|BIN Taxonomy Match|BOLD:AAA8204
Limnophyes minimus|BIOUG04114-G04|Kate Perez|BIN Taxonomy Match|BOLD:AAA8204
Limnophyes minimus|BIOUG05483-E02|Kate Perez|BIN Taxonomy Match|BOLD:AAA8204
Limnophyes minimus|BIOUG05144-C05|Kate Perez|BIN Taxonomy Match|BOLD:AAA8204
Limnophyes minimus|BIOUG04261-B03|Kate Perez|BIN Taxonomy Match|BOLD:AAA8204
Limnophyes minimus|BIOUG04261-B04|Kate Perez|BIN Taxonomy Match|BOLD:AAA8204
Limnophyes minimus|BIOUG04261-B10|Kate Perez|BIN Taxonomy Match|BOLD:AAA8204
Limnophyes minimus|BIOUG04090-E06|Kate Perez|BIN Taxonomy Match|BOLD:AAA8204
Limnophyes minimus|BIOUG16214-D12|Kate Perez|BIN Taxonomy Match|BOLD:AAA8204
Limnophyes minimus|BIOUG04261-A01|Kate Perez|BIN Taxonomy Match|BOLD:AAA8204
Limnophyes minimus|BIOUG04261-A04|Kate Perez|BIN Taxonomy Match|BOLD:AAA8204
Limnophyes minimus|BIOUG04269-D05|Kate Perez|BIN Taxonomy Match|BOLD:AAA8204
Limnophyes minimus|BIOUG04269-H09|Kate Perez|BIN Taxonomy Match|BOLD:AAA8204
Limnophyes minimus|BIOUG04269-B04|Kate Perez|BIN Taxonomy Match|BOLD:AAA8204
Limnophyes minimus|BIOUG06058-E12|Kate Perez|BIN Taxonomy Match|BOLD:AAA8204
Limnophyes minimus|BIOUG05109-A08|Kate Perez|BIN Taxonomy Match|BOLD:AAA8204
Limnophyes minimus|BIOUG05109-D08|Kate Perez|BIN Taxonomy Match|BOLD:AAA8204
Limnophyes minimus|BIOUG04119-F07|Kate Perez|BIN Taxonomy Match|BOLD:AAA8204
Limnophyes minimus|BIOUG04119-H08|Kate Perez|BIN Taxonomy Match|BOLD:AAA8204
Limnophyes minimus|BIOUG05219-E11|Kate Perez|BIN Taxonomy Match|BOLD:AAA8204
Limnophyes minimus|BIOUG05219-H02|Kate Perez|BIN Taxonomy Match|BOLD:AAA8204
Limnophyes minimus|BIOUG05144-B04|Kate Perez|BIN Taxonomy Match|BOLD:AAA8204
Limnophyes minimus|BIOUG05144-B10|Kate Perez|BIN Taxonomy Match|BOLD:AAA8204
Limnophyes minimus|BIOUG05144-C07|Kate Perez|BIN Taxonomy Match|BOLD:AAA8204
Limnophyes minimus|BIOUG05144-C09|Kate Perez|BIN Taxonomy Match|BOLD:AAA8204
Limnophyes minimus|BIOUG05144-G04|Kate Perez|BIN Taxonomy Match|BOLD:AAA8204
Limnophyes minimus|BIOUG05144-H05|Kate Perez|BIN Taxonomy Match|BOLD:AAA8204
Limnophyes minimus|BIOUG04212-F08|Kate Perez|BIN Taxonomy Match|BOLD:AAA8204
Limnophyes minimus|BIOUG04296-F01|Kate Perez|BIN Taxonomy Match|BOLD:AAA8204
Limnophyes minimus|BIOUG05144-A05|Kate Perez|BIN Taxonomy Match|BOLD:AAA8204
Limnophyes minimus|BIOUG05144-A06|Kate Perez|BIN Taxonomy Match|BOLD:AAA8204
Limnophyes minimus|BIOUG05144-B11|Kate Perez|BIN Taxonomy Match|BOLD:AAA8204
Limnophyes minimus|BIOUG05144-D03|Kate Perez|BIN Taxonomy Match|BOLD:AAA8204
Limnophyes minimus|BIOUG04261-A03|Kate Perez|BIN Taxonomy Match|BOLD:AAA8204
Limnophyes minimus|BIOUG05101-F08|Kate Perez|BIN Taxonomy Match|BOLD:AAA8204
Limnophyes minimus|BIOUG05215-B07|Kate Perez|BIN Taxonomy Match|BOLD:AAA8204
Limnophyes minimus|BIOUG05216-A12|Kate Perez|BIN Taxonomy Match|BOLD:AAA8204
Limnophyes minimus|BIOUG05216-E06|Kate Perez|BIN Taxonomy Match|BOLD:AAA8204
Limnophyes minimus|BIOUG05216-G01|Kate Perez|BIN Taxonomy Match|BOLD:AAA8204
Limnophyes minimus|BIOUG05217-B01|Kate Perez|BIN Taxonomy Match|BOLD:AAA8204
Limnophyes minimus|BIOUG05217-C10|Kate Perez|BIN Taxonomy Match|BOLD:AAA8204
Limnophyes minimus|BIOUG05217-G05|Kate Perez|BIN Taxonomy Match|BOLD:AAA8204
Limnophyes minimus|BIOUG15852-F07|Kate Perez|BIN Taxonomy Match|BOLD:AAA8204
Limnophyes minimus|BIOUG15853-A08|Kate Perez|BIN Taxonomy Match|BOLD:AAA8204
Limnophyes minimus|BIOUG15853-F04|Kate Perez|BIN Taxonomy Match|BOLD:AAA8204
Limnophyes minimus|BIOUG04437-G04|Kate Perez|BIN Taxonomy Match|BOLD:AAA8204
Limnophyes minimus|BIOUG04437-H02|Kate Perez|BIN Taxonomy Match|BOLD:AAA8204
Limnophyes minimus|BIOUG04437-C07|Kate Perez|BIN Taxonomy Match|BOLD:AAA8204
Limnophyes minimus|BIOUG07805-A11|Kate Perez|BIN Taxonomy Match|BOLD:AAA8204
Limnophyes minimus|BIOUG07805-C02|Kate Perez|BIN Taxonomy Match|BOLD:AAA8204
Limnophyes minimus|BIOUG07805-G11|Kate Perez|BIN Taxonomy Match|BOLD:AAA8204
Limnophyes minimus|BIOUG07805-H06|Kate Perez|BIN Taxonomy Match|BOLD:AAA8204
Limnophyes minimus|BIOUG04217-D12|Kate Perez|BIN Taxonomy Match|BOLD:AAA8204
Limnophyes minimus|BIOUG04217-E12|Kate Perez|BIN Taxonomy Match|BOLD:AAA8204
Limnophyes minimus|BIOUG04217-G02|Kate Perez|BIN Taxonomy Match|BOLD:AAA8204
Limnophyes minimus|BIOUG04217-G05|Kate Perez|BIN Taxonomy Match|BOLD:AAA8204
Limnophyes minimus|BIOUG36603-H05|Kate Perez|BIN Taxonomy Match (Jan 2018)|BOLD:AAA8204
Limnophyes minimus|BIOUG08174-E11|Kate Perez|BIN Taxonomy Match|BOLD:AAA8204
Limnophyes minimus|BIOUG08209-D05|Kate Perez|BIN Taxonomy Match|BOLD:AAA8204
Limnophyes minimus|BIOUG15764-A10|Kate Perez|BIN Taxonomy Match|BOLD:AAA8204
Limnophyes minimus|BIOUG15762-F11|Kate Perez|BIN Taxonomy Match|BOLD:AAA8204
Limnophyes minimus|BIOUG15794-A12|Kate Perez|BIN Taxonomy Match|BOLD:AAA8204
Limnophyes minimus|BIOUG07753-G06|Kate Perez|BIN Taxonomy Match|BOLD:AAA8204
Limnophyes minimus|BIOUG15455-G11|Kate Perez|BIN Taxonomy Match|BOLD:AAA8204
Limnophyes minimus|ZSM-DIP-33135-F09|Caroline Chimeno|BIN Taxonomy Match (Jan 2022)|BOLD ...
Limnophyes minimus|BIOUG42965-G02|Kate Perez|BIN Taxonomy Match below Phylum (Jun 2019)|BOL...
Limnophyes minimus|ZSM34343-F12|Sofia Wiedenbrug|Morphology|BOLD:AAA8204
[illegible]

Limnophyes minimus|ZSM-DIP-33135-F09|Caroline Chimeno|BIN Taxonomy Match (Jan 2022)|BOLD ...
Limnophyes minimus|BIOUG42965-G02|Kate Perez|BIN Taxonomy Match below Phylum (Jun 2019)|BOL...
Limnophyes minimus|ZSM34343-F12|Sofia Wiedenbrug|Morphology|BOLD:AAA8204
Limnophyes minimus|BIOUG15764-H09|Kate Perez|BIN Taxonomy Match|BOLD:AAA8204
Limnophyes minimus|BIOUG07509-E05|Kate Perez|BIN Taxonomy Match|BOLD:AAA8204
Limnophyes minimus|BIOUG07410-C04|Kate Perez|BIN Taxonomy Match|BOLD:AAA8204
Limnophyes minimus|BIOUG05486-F10|Kate Perez|BIN Taxonomy Match|BOLD:AAA8204
Limnophyes minimus|BIOUG07414-E11|Kate Perez|BIN Taxonomy Match|BOLD:AAA8204
Limnophyes minimus|BIOUG04437-E01|Kate Perez|BIN Taxonomy Match|BOLD:AAA8204
Limnophyes minimus|BIOUG36684-H11|Kate Perez|BIN Taxonomy Match (May 2018)|BOLD:AAA8204
Limnophyes minimus|BIOUG07808-H06|Kate Perez|BIN Taxonomy Match|BOLD:AAA8204
Limnophyes minimus|BIOUG15764-E09|Kate Perez|BIN Taxonomy Match|BOLD:AAA8204
Limnophyes minimus|BIOUG16398-B12|Kate Perez|BIN Taxonomy Match|BOLD:AAA8204
Limnophyes minimus|BIOUG15536-A06|Kate Perez|BIN Taxonomy Match|BOLD:AAA8204
Limnophyes minimus|BIOUG07405-F09|Kate Perez|BIN Taxonomy Match|BOLD:AAA8204
Limnophyes minimus|BIOUG07809-A11|Kate Perez|BIN Taxonomy Match|BOLD:AAA8204
Limnophyes minimus|BIOUG15766-H02|Kate Perez|BIN Taxonomy Match|BOLD:AAA8204
Limnophyes minimus|BIOUG08095-A02|Kate Perez|BIN Taxonomy Match|BOLD:AAA8204
Limnophyes minimus|BIOUG05777-D06|Kate Perez|BIN Taxonomy Match|BOLD:AAA8204
Limnophyes minimus|BIOUG07568-D01|Kate Perez|BIN Taxonomy Match|BOLD:AAA8204
Limnophyes minimus|BIOUG07410-B08|Kate Perez|BIN Taxonomy Match|BOLD:AAA8204
Limnophyes minimus|BIOUG16625-A02|Kate Perez|BIN Taxonomy Match|BOLD:AAA8204
Limnophyes minimus|BIOUG15718-C10|Kate Perez|BIN Taxonomy Match|BOLD:AAA8204
Limnophyes minimus|BIOUG15539-E11|Kate Perez|BIN Taxonomy Match|BOLD:AAA8204
Limnophyes minimus|BIOUG05900-C08|Kate Perez|BIN Taxonomy Match|BOLD:AAA8204
Limnophyes minimus|BIOUG05485-E07|Kate Perez|BIN Taxonomy Match|BOLD:AAA8204
Limnophyes minimus|BIOUG06065-F12|Kate Perez|BIN Taxonomy Match|BOLD:AAA8204
Limnophyes minimus|BIOUG05395-A07|Kate Perez|BIN Taxonomy Match|BOLD:AAA8204
Limnophyes minimus|BIOUG04433-C06|Kate Perez|BIN Taxonomy Match|BOLD:AAA8204
Limnophyes minimus|BIOUG05217-C06|Kate Perez|BIN Taxonomy Match|BOLD:AAA8204
Limnophyes minimus|BIOUG04437-E03|Kate Perez|BIN Taxonomy Match|BOLD:AAA8204
Limnophyes minimus|BIOUG15766-E08|Kate Perez|BIN Taxonomy Match|BOLD:AAA8204
Limnophyes minimus|BIOUG15769-C04|Kate Perez|BIN Taxonomy Match|BOLD:AAA8204
Limnophyes minimus|BIOUG15769-D08|Kate Perez|BIN Taxonomy Match|BOLD:AAA8204
Limnophyes minimus|BIOUG15537-G02|Kate Perez|BIN Taxonomy Match|BOLD:AAA8204
Limnophyes minimus|BIOUG15795-D06|Kate Perez|BIN Taxonomy Match|BOLD:AAA8204
Limnophyes minimus|BIOUG15795-H07|Kate Perez|BIN Taxonomy Match|BOLD:AAA8204
Limnophyes minimus|BIOUG16596-F10|Kate Perez|BIN Taxonomy Match|BOLD:AAA8204
Limnophyes minimus|BIOUG16596-H02|Kate Perez|BIN Taxonomy Match|BOLD:AAA8204
Limnophyes minimus|BIOUG36987-G09|Kate Perez|BIN Taxonomy Match (Jul 2018)|BOLD:AAA8204
Limnophyes minimus|BIOUG55189-F11|Stefan Schmidt|BIN Taxonomy Match (Sep 2020)|BOLD:AAA8204
Limnophyes minimus|CH-OSF44|Elisabeth Stur||BOLD:AAA8204
Limnophyes minimus|ZSM-DIP-33135-H11|Caroline Chimeno|BIN Taxonomy Match (Jan 2022)|BOLD ...
Limnophyes minimus|ZSM-DIP-33135-H10|Caroline Chimeno|BIN Taxonomy Match (Jan 2022)|BOLD ...
Limnophyes minimus|ZSM-DIP-33135-H09|Caroline Chimeno|BIN Taxonomy Match (Jan 2022)|BOLD ...
Limnophyes minimus|ZSM-DIP-33135-H08|Caroline Chimeno|BIN Taxonomy Match (Jan 2022)|BOLD ...
Limnophyes minimus|ZSM-DIP-33135-H05|Caroline Chimeno|BIN Taxonomy Match (Jan 2022)|BOLD ...
Limnophyes minimus|ZSM-DIP-33135-H04|Caroline Chimeno|BIN Taxonomy Match (Jan 2022)|BOLD ...
Limnophyes minimus|ZSM-DIP-33135-G12|Caroline Chimeno|BIN Taxonomy Match (Jan 2022)|BOLD ...
Limnophyes minimus|ZSM-DIP-33135-F10|Caroline Chimeno|BIN Taxonomy Match (Jan 2022)|BOLD ...
Limnophyes minimus|ZSM-DIP-33130-A11|Caroline Chimeno|BIN Taxonomy Match (Jan 2022)|BOLD ...
Limnophyes minimus|ZSM-DIP-33144-B04|Caroline Chimeno|BIN Taxonomy Match (Jan 2022)|BOLD ...
Limnophyes minimus|ZSM-DIP-33144-B03|Caroline Chimeno|BIN Taxonomy Match (Jan 2022)|BOLD ...
Limnophyes minimus|ZSM-DIP-33137-D09|Caroline Chimeno|BIN Taxonomy Match (Jan 2022)|BOLD ...
Limnophyes minimus|BIOUG42961-H06|Kate Perez|BIN Taxonomy Match below Phylum (Jun 2019)|BOL...
Limnophyes minimus|BIOUG42961-F06|Kate Perez|BIN Taxonomy Match below Phylum (Jun 2019)|BOL...
Limnophyes minimus|BIOUG42961-E06|Kate Perez|BIN Taxonomy Match below Phylum (Jun 2019)|BOL...
Limnophyes minimus|BIOUG42961-D12|Kate Perez|BIN Taxonomy Match below Phylum (Jun 2019)|BOL...
Limnophyes minimus|BIOUG07810-B07|Kate Perez|BIN Taxonomy Match|BOLD:AAA8204
Limnophyes minimus|BIOUG15455-C10|Kate Perez|BIN Taxonomy Match|BOLD:AAA8204
Limnophyes minimus|BIOUG15895-G05|Kate Perez|BIN Taxonomy Match|BOLD:AAA8204
Limnophyes minimus|BIOUG15895-E08|Kate Perez|BIN Taxonomy Match|BOLD:AAA8204
Limnophyes minimus|ZSM-DIP-33140-D12|Caroline Chimeno|BIN Taxonomy Match (Jan 2022)|BOLD ...
Limnophyes minimus|ZSM-DIP-33138-H09|Caroline Chimeno|BIN Taxonomy Match (Jan 2022)|BOLD ...
Limnophyes minimus|ZSM-DIP-33138-H03|Caroline Chimeno|BIN Taxonomy Match (Jan 2022)|BOLD ...
Limnophyes minimus|ZSM-DIP-33138-G07|Caroline Chimeno|BIN Taxonomy Match (Jan 2022)|BOLD ...
Limnophyes minimus|ZSM-DIP-33146-F04|Caroline Chimeno|BIN Taxonomy Match (Jan 2022)|BOLD ...
Limnophyes minimus|ZSM-DIP-33146-B11|Caroline Chimeno|BIN Taxonomy Match (Jan 2022)|BOLD ...
Limnophyes minimus|ZSM-DIP-33138-B03|Caroline Chimeno|BIN Taxonomy Match (Jan 2022)|BOLD ...
Limnophyes minimus|ZSM-DIP-33138-B02|Caroline Chimeno|BIN Taxonomy Match (Jan 2022)|BOLD ...
Limnophyes minimus|ZSM-DIP-33137-D07|Caroline Chimeno|BIN Taxonomy Match (Jan 2022)|BOLD ...
Limnophyes minimus|ZSM-DIP-33137-D06|Caroline Chimeno|BIN Taxonomy Match (Jan 2022)|BOLD ...
Limnophyes minimus|ZSM-DIP-33137-D04|Caroline Chimeno|BIN Taxonomy Match (Jan 2022)|BOLD ...
Limnophyes minimus|ZSM-DIP-33137-D02|Caroline Chimeno|BIN Taxonomy Match (Jan 2022)|BOLD ...
Limnophyes minimus|BIOUG36677-C06|Kate Perez|BIN Taxonomy Match (May 2018)|BOLD:AAA8204
Limnophyes minimus|BIOUG07263-C12|Kate Perez|BIN Taxonomy Match|BOLD:AAA8204
Limnophyes minimus|BIOUG04437-E04|Kate Perez|BIN Taxonomy Match|BOLD:AAA8204
Limnophyes minimus|BIOUG05490-H03|Kate Perez|BIN Taxonomy Match|BOLD:AAA8204
Limnophyes minimus|BIOUG16597-C02|Kate Perez|BIN Taxonomy Match|BOLD:AAA8204
Limnophyes minimus|BIOUG16596-D05|Kate Perez|BIN Taxonomy Match|BOLD:AAA8204
Limnophyes minimus|BIOUG15806-D02|Kate Perez|BIN Taxonomy Match|BOLD:AAA8204
Limnophyes minimus|BIOUG15799-B12|Kate Perez|BIN Taxonomy Match|BOLD:AAA8204
Limnophyes minimus|BIOUG07505-D09|Kate Perez|BIN Taxonomy Match|BOLD:AAA8204
Limnophyes minimus|BIOUG04089-D09|Kate Perez|BIN Taxonomy Match|BOLD:AAA8204
Limnophyes minimus|BIOUG54161-A04|Stefan Schmidt|BIN Taxonomy Match (Sep 2020)|BOLD:AAA8204
Limnophyes minimus|BIOUG42966-D10|Kate Perez|BIN Taxonomy Match below Phylum (Jun 2019)|BOL...
Limnophyes minimus|CH-eik05|Elisabeth Stur||BOLD:AAA8204
Limnophyes minimus|Finnmark07|Elisabeth Stur||BOLD:AAA8204
Limnophyes minimus|ZSM-DIP-33132-E12|Caroline Chimeno|BIN Taxonomy Match (Jan 2022)|BOLD ...
Limnophyes minimus|ZSM-DIP-33132-D10|Caroline Chimeno|BIN Taxonomy Match (Jan 2022)|BOLD ...
Limnophyes minimus|BIOUG55147-F05|Stefan Schmidt|BIN Taxonomy Match (Sep 2020)|BOLD:AAA8204
Limnophyes minimus|BIOUG42695-E12|Kate Perez|BIN Taxonomy Match below Phylum (Jun 2019)|BOL...
Limnophyes minimus|BIOUG15565-D03|Kate Perez|BIN Taxonomy Match|BOLD:AAA8204
Limnophyes minimus|BIOUG15465-A06|Kate Perez|BIN Taxonomy Match|BOLD:AAA8204
Limnophyes minimus|BIOUG08318-E05|Kate Perez|BIN Taxonomy Match|BOLD:AAA8204
Limnophyes minimus|BIOUG15766-D08|Kate Perez|BIN Taxonomy Match|BOLD:AAA8204
Limnophyes minimus|BIOUG07755-B10|Kate Perez|BIN Taxonomy Match|BOLD:AAA8204
Limnophyes minimus|BIOUG07753-D12|Kate Perez|BIN Taxonomy Match|BOLD:AAA8204
Limnophyes minimus|BIOUG07753-D01|Kate Perez|BIN Taxonomy Match|BOLD:AAA8204
Limnophyes minimus|BIOUG07753-A10|Kate Perez|BIN Taxonomy Match|BOLD:AAA8204
Limnophyes minimus|BIOUG54164-B04|Stefan Schmidt|BIN Taxonomy Match (Sep 2020)|BOLD:AAA8204
Limnophyes minimus|BIOUG42968-D08|Kate Perez|BIN Taxonomy Match below Phylum (Jun 2019)|BOL...
Limnophyes minimus|DIPNO-0040|Kate Perez|BIN Taxonomy Match (Jun 2019)|BOLD:AAA8204

Limnophyes minimus|BIOUG54164-B04|Stefan Schmidt|BIN Taxonomy Match (Sep 2020)|BOLD:AAA8204
Limnophyes minimus|BIOUG42968-D08|Kate Perez|BIN Taxonomy Match below Phylum (Jun 2019)|BOL...
Limnophyes minimus|DIPNO-0040|Kate Perez|BIN Taxonomy Match (Jun 2019)|BOLD:AAA8204
Limnophyes minimus|CH-eik113|Elisabeth Stur||BOLD:AAA8204
Limnophyes minimus|BIOUG07755-H04|Kate Perez|BIN Taxonomy Match|BOLD:AAA8204
Limnophyes minimus|BIOUG07945-D10|Kate Perez|BIN Taxonomy Match|BOLD:AAA8204
Limnophyes minimus|BIOUG08140-C10|Kate Perez|BIN Taxonomy Match|BOLD:AAA8204
Limnophyes minimus|BIOUG07754-G08|Kate Perez|BIN Taxonomy Match|BOLD:AAA8204
Limnophyes minimus|ZSM-DIP-33143-B11|Caroline Chimeno|BIN Taxonomy Match (Jan 2022)|BOLD ...
Limnophyes minimus|ZSM-DIP-33143-A05|Caroline Chimeno|BIN Taxonomy Match (Jan 2022)|BOLD ...
Limnophyes minimus|BIOUG16434-B10|Kate Perez|BIN Taxonomy Match|BOLD:AAA8204
Limnophyes minimus|CH-OSF43|Elisabeth Stur||BOLD:AAA8204
Limnophyes minimus|BIOUG15600-F09|Kate Perez|BIN Taxonomy Match|BOLD:AAA8204
Limnophyes minimus|BIOUG16436-D11|Kate Perez|BIN Taxonomy Match|BOLD:AAA8204
Limnophyes minimus|RIN_CH06|Elisabeth Stur|morphology|BOLD:AAA8204
Limnophyes minimus|CH-OSF152|Elisabeth Stur||BOLD:AAA8204
Limnophyes minimus|CH-OSF127|Elisabeth Stur||BOLD:AAA8204
Limnophyes minimus|CH-OSF126|Elisabeth Stur||BOLD:AAA8204
Limnophyes minimus|BIOUG15947-C08|Kate Perez|BIN Taxonomy Match|BOLD:AAA8204
Limnophyes minimus|BIOUG15809-F02|Kate Perez|BIN Taxonomy Match|BOLD:AAA8204
Limnophyes minimus|BIOUG15809-B05|Kate Perez|BIN Taxonomy Match|BOLD:AAA8204
Limnophyes minimus|BIOUG15808-H02|Kate Perez|BIN Taxonomy Match|BOLD:AAA8204
Limnophyes minimus|BIOUG16596-G10|Kate Perez|BIN Taxonomy Match|BOLD:AAA8204
Limnophyes minimus|BIOUG15765-B06|Kate Perez|BIN Taxonomy Match|BOLD:AAA8204
Limnophyes minimus|BIOUG15798-B12|Kate Perez|BIN Taxonomy Match|BOLD:AAA8204
Limnophyes minimus|BIOUG15455-B09|Kate Perez|BIN Taxonomy Match|BOLD:AAA8204
Limnophyes minimus|BIOUG16551-H05|Kate Perez|BIN Taxonomy Match|BOLD:AAA8204
Limnophyes minimus|BIOUG15809-D06|Kate Perez|BIN Taxonomy Match|BOLD:AAA8204
Limnophyes minimus|ZSM-DIP-33130-F05|Caroline Chimeno|BIN Taxonomy Match (Jan 2022)|BOLD ...
Limnophyes minimus|ZSM-DIP-33130-D07|Caroline Chimeno|BIN Taxonomy Match (Jan 2022)|BOLD ...
Limnophyes minimus|ZSM-DIP-33134-C06|Caroline Chimeno|BIN Taxonomy Match (Jan 2022)|BOLD ...
Limnophyes minimus|ZSM-DIP-33134-B07|Caroline Chimeno|BIN Taxonomy Match (Jan 2022)|BOLD ...
Limnophyes minimus|BIOUG15765-A10|Kate Perez|BIN Taxonomy Match|BOLD:AAA8204
Limnophyes minimus|BIOUG15809-A12|Kate Perez|BIN Taxonomy Match|BOLD:AAA8204
Limnophyes minimus|BIOUG15807-E05|Kate Perez|BIN Taxonomy Match|BOLD:AAA8204
Limnophyes minimus|BIOUG15797-B11|Kate Perez|BIN Taxonomy Match|BOLD:AAA8204
Limnophyes minimus|BIOUG36678-B12|Kate Perez|BIN Taxonomy Match (Jul 2018)|BOLD:AAA8204
Limnophyes minimus|BIOUG36737-A04|Kate Perez|BIN Taxonomy Match (May 2018)|BOLD:AAA8204
Limnophyes minimus|BIOUG42681-D04|Kate Perez|BIN Taxonomy Match below Phylum (Jun 2019)|BOL...
Limnophyes minimus|BIOUG42681-B11|Kate Perez|BIN Taxonomy Match below Phylum (Jun 2019)|BOL...
Limnophyes minimus|BIOUG43079-C06|Kate Perez|BIN Taxonomy Match below Phylum (Jun 2019)|BOL...
Limnophyes minimus|BIOUG43079-B08|Kate Perez|BIN Taxonomy Match below Phylum (Jun 2019)|BOL...
Limnophyes minimus|BIOUG15762-D03|Kate Perez|BIN Taxonomy Match|BOLD:AAA8204
Limnophyes minimus|BIOUG15762-C06|Kate Perez|BIN Taxonomy Match|BOLD:AAA8204
Limnophyes minimus|BIOUG07569-G03|Kate Perez|BIN Taxonomy Match|BOLD:AAA8204
Limnophyes minimus|BIOUG15460-F10|Kate Perez|BIN Taxonomy Match|BOLD:AAA8204
Limnophyes minimus|BIOUG07760-B11|Kate Perez|BIN Taxonomy Match|BOLD:AAA8204
Limnophyes minimus|BIOUG07759-F06|Kate Perez|BIN Taxonomy Match|BOLD:AAA8204
Limnophyes minimus|BIOUG07565-A10|Kate Perez|BIN Taxonomy Match|BOLD:AAA8204
Limnophyes minimus|BIOUG08315-G08|Kate Perez|BIN Taxonomy Match|BOLD:AAA8204
Limnophyes minimus|BIOUG07760-A08|Kate Perez|BIN Taxonomy Match|BOLD:AAA8204
Limnophyes minimus|BIOUG07758-F11|Kate Perez|BIN Taxonomy Match|BOLD:AAA8204
Limnophyes minimus|BIOUG16597-G09|Kate Perez|BIN Taxonomy Match|BOLD:AAA8204
Limnophyes minimus|BIOUG16597-F09|Kate Perez|BIN Taxonomy Match|BOLD:AAA8204
Limnophyes minimus|BIOUG08138-C03|Kate Perez|BIN Taxonomy Match|BOLD:AAA8204
Limnophyes minimus|BIOUG08137-H02|Kate Perez|BIN Taxonomy Match|BOLD:AAA8204
Limnophyes minimus|BIOUG15806-C10|Kate Perez|BIN Taxonomy Match|BOLD:AAA8204
Limnophyes minimus|BIOUG15800-A02|Kate Perez|BIN Taxonomy Match|BOLD:AAA8204
Limnophyes minimus|BIOUG15964-G03|Kate Perez|BIN Taxonomy Match|BOLD:AAA8204
Limnophyes minimus|BIOUG15807-F02|Kate Perez|BIN Taxonomy Match|BOLD:AAA8204
Limnophyes minimus|BIOUG06056-G09|Kate Perez|BIN Taxonomy Match|BOLD:AAA8204
Limnophyes minimus|BIOUG06047-A07|Kate Perez|BIN Taxonomy Match|BOLD:AAA8204
Limnophyes minimus|BIOUG16596-D04|Kate Perez|BIN Taxonomy Match|BOLD:AAA8204
Limnophyes minimus|BIOUG16594-D09|Kate Perez|BIN Taxonomy Match|BOLD:AAA8204
Limnophyes minimus|ZSM-DIP-33143-A04|Caroline Chimeno|BIN Taxonomy Match (Jan 2022)|BOLD ...
Limnophyes minimus|ZSM-DIP-33143-A03|Caroline Chimeno|BIN Taxonomy Match (Jan 2022)|BOLD ...
Limnophyes minimus|BIOUG42788-E03|Kate Perez|BIN Taxonomy Match below Phylum (Jun 2019)|BOL...
Limnophyes minimus|ZSM-DIP-33142-F02|Caroline Chimeno|BIN Taxonomy Match (Jan 2022)|BOLD ...
Limnophyes minimus|ZSM-DIP-33142-D04|Caroline Chimeno|BIN Taxonomy Match (Jan 2022)|BOLD ...
Limnophyes minimus|ZSM-DIP-33142-C10|Caroline Chimeno|BIN Taxonomy Match (Jan 2022)|BOLD ...
Limnophyes minimus|ZSM-DIP-33142-C08|Caroline Chimeno|BIN Taxonomy Match (Jan 2022)|BOLD ...
Limnophyes minimus|ZSM-DIP-33142-B04|Caroline Chimeno|BIN Taxonomy Match (Jan 2022)|BOLD ...
Limnophyes minimus|ZSM-DIP-33134-D10|Caroline Chimeno|BIN Taxonomy Match (Jan 2022)|BOLD ...
Limnophyes minimus|ZSM-DIP-33134-D03|Caroline Chimeno|BIN Taxonomy Match (Jan 2022)|BOLD ...
Limnophyes minimus|ZSM-DIP-33132-D06|Caroline Chimeno|BIN Taxonomy Match (Jan 2022)|BOLD ...
Limnophyes minimus|ZSM-DIP-33132-C09|Caroline Chimeno|BIN Taxonomy Match (Jan 2022)|BOLD ...
Limnophyes minimus|BIOUG16596-D08|Kate Perez|BIN Taxonomy Match|BOLD:AAA8204
Limnophyes minimus|BIOUG16594-E08|Kate Perez|BIN Taxonomy Match|BOLD:AAA8204
Limnophyes minimus|ZSM-DIP-33135-F07|Caroline Chimeno|BIN Taxonomy Match (Jan 2022)|BOLD ...
Limnophyes minimus|ZSM-DIP-33135-F05|Caroline Chimeno|BIN Taxonomy Match (Jan 2022)|BOLD ...
Limnophyes minimus|ZSM-DIP-33143-H01|Caroline Chimeno|BIN Taxonomy Match (Jan 2022)|BOLD ...
Limnophyes minimus|ZSM-DIP-33143-G10|Caroline Chimeno|BIN Taxonomy Match (Jan 2022)|BOLD ...
Limnophyes minimus|BIOUG16626-D03|Kate Perez|BIN Taxonomy Match|BOLD:AAA8204
Limnophyes minimus|BIOUG36846-E04|Kate Perez|BIN Taxonomy Match (Jul 2018)|BOLD:AAA8204
Limnophyes minimus|ZSM-DIP-33132-A05|Caroline Chimeno|BIN Taxonomy Match (Jan 2022)|BOLD ...
Limnophyes minimus|ZSM-DIP-33131-H11|Caroline Chimeno|BIN Taxonomy Match (Jan 2022)|BOLD ...
Limnophyes minimus|BIOUG55425-B06|Stefan Schmidt|BIN Taxonomy Match (Sep 2020)|BOLD:AAA8204
Limnophyes minimus|BIOUG43080-H10|Kate Perez|BIN Taxonomy Match below Phylum (Jun 2019)|BOL...
Limnophyes minimus|BIOUG07756-C05|Kate Perez|BIN Taxonomy Match|BOLD:AAA8204
Limnophyes minimus|BIOUG07755-F12|Kate Perez|BIN Taxonomy Match|BOLD:AAA8204
Limnophyes minimus|BIOUG15765-D04|Kate Perez|BIN Taxonomy Match|BOLD:AAA8204
Limnophyes minimus|BIOUG15765-C07|Kate Perez|BIN Taxonomy Match|BOLD:AAA8204
Limnophyes minimus|BIOUG15765-A11|Kate Perez|BIN Taxonomy Match|BOLD:AAA8204
Limnophyes minimus|BIOUG15765-A09|Kate Perez|BIN Taxonomy Match|BOLD:AAA8204
Limnophyes minimus|ZSM-DIP-33144-E03|Caroline Chimeno|BIN Taxonomy Match (Jan 2022)|BOLD ...
Limnophyes minimus|ZSM-DIP-33141-A02|Caroline Chimeno|BIN Taxonomy Match (Jan 2022)|BOLD ...
Limnophyes minimus|ZSM-DIP-33141-A01|Caroline Chimeno|BIN Taxonomy Match (Jan 2022)|BOLD ...
Limnophyes minimus|ZSM-DIP-33140-E01|Caroline Chimeno|BIN Taxonomy Match (Jan 2022)|BOLD ...
Limnophyes minimus|BIOUG15762-G11|Kate Perez|BIN Taxonomy Match|BOLD:AAA8204
Limnophyes minimus|BIOUG15762-F08|Kate Perez|BIN Taxonomy Match|BOLD:AAA8204
Limnophyes minimus|BIOUG15465-H09|Kate Perez|BIN Taxonomy Match|BOLD:AAA8204
Limnophyes minimus|BIOUG15465-B03|Kate Perez|BIN Taxonomy Match|BOLD:AAA8204
Limnophyes minimus|BIOUG15765-C03|Kate Perez|BIN Taxonomy Match|BOLD:AAA8204

Limnophyes minimus|BIOUG15465-H09|Kate Perez|BIN Taxonomy Match|BOLD:AAA8204
Limnophyes minimus|BIOUG15465-B03|Kate Perez|BIN Taxonomy Match|BOLD:AAA8204
Limnophyes minimus|BIOUG15765-C03|Kate Perez|BIN Taxonomy Match|BOLD:AAA8204
Limnophyes minimus|BIOUG15762-D07|Kate Perez|BIN Taxonomy Match|BOLD:AAA8204
Limnophyes minimus|ZSM-DIP-33141-H07|Caroline Chimeno|BIN Taxonomy Match (Jan 2022)|BOLD ...
Limnophyes minimus|ZSM-DIP-33141-G06|Caroline Chimeno|BIN Taxonomy Match (Jan 2022)|BOLD ...
Limnophyes minimus|ZSM-DIP-33141-G04|Caroline Chimeno|BIN Taxonomy Match (Jan 2022)|BOLD ...
Limnophyes minimus|ZSM-DIP-33141-G03|Caroline Chimeno|BIN Taxonomy Match (Jan 2022)|BOLD ...
Limnophyes minimus|ZSM-DIP-33141-F08|Caroline Chimeno|BIN Taxonomy Match (Jan 2022)|BOLD ...
Limnophyes minimus|ZSM-DIP-33141-F07|Caroline Chimeno|BIN Taxonomy Match (Jan 2022)|BOLD ...
Limnophyes minimus|ZSM-DIP-33141-F03|Caroline Chimeno|BIN Taxonomy Match (Jan 2022)|BOLD ...
Limnophyes minimus|ZSM-DIP-33141-F02|Caroline Chimeno|BIN Taxonomy Match (Jan 2022)|BOLD ...
Limnophyes minimus|BIOUG42965-D11|Kate Perez|BIN Taxonomy Match below Phylum (Jun 2019)|BOL...
Limnophyes minimus|BIOUG42964-A07|Kate Perez|BIN Taxonomy Match below Phylum (Jun 2019)|BOL...
Limnophyes minimus|BIOUG42963-G12|Kate Perez|BIN Taxonomy Match below Phylum (Jun 2019)|BOL...
Limnophyes minimus|BIOUG42963-G07|Kate Perez|BIN Taxonomy Match below Phylum (Jun 2019)|BOL...
Limnophyes minimus|BIOUG42944-E02|Kate Perez|BIN Taxonomy Match below Phylum (Jun 2019)|BOL...
Limnophyes minimus|ZSM-DIP-33134-D08|Caroline Chimeno|BIN Taxonomy Match (Jan 2022)|BOLD ...
Limnophyes minimus|BIOUG42930-G07|Kate Perez|BIN Taxonomy Match below Phylum (Jun 2019)|BOL...
Limnophyes minimus|BIOUG42927-C12|Kate Perez|BIN Taxonomy Match below Phylum (Jun 2019)|BOL...
Limnophyes minimus|BIOUG42950-D10|Kate Perez|BIN Taxonomy Match below Phylum (Jun 2019)|BOL...
Limnophyes minimus|BIOUG42764-C12|Kate Perez|BIN Taxonomy Match below Phylum (Jun 2019)|BOL...
Limnophyes minimus|BIOUG42938-C05|Kate Perez|BIN Taxonomy Match below Phylum (Jun 2019)|BOL...
Limnophyes minimus|BIOUG42915-D08|Kate Perez|BIN Taxonomy Match below Phylum (Jun 2019)|BOL...
Limnophyes minimus|ZSM-DIP-33131-F06|Caroline Chimeno|BIN Taxonomy Match (Jan 2022)|BOLD ...
Limnophyes minimus|BIOUG42969-G01|Kate Perez|BIN Taxonomy Match below Phylum (Jun 2019)|BOL...
Limnophyes minimus|BIOUG42755-D04|Kate Perez|BIN Taxonomy Match below Phylum (Jun 2019)|BOL...
Limnophyes minimus|BIOUG07506-F08|Kate Perez|BIN Taxonomy Match|BOLD:AAA8204
Limnophyes minimus|BIOUG15807-H08|Kate Perez|BIN Taxonomy Match|BOLD:AAA8204
Limnophyes minimus|BIOUG15793-G04|Kate Perez|BIN Taxonomy Match|BOLD:AAA8204
Limnophyes minimus|BIOUG43080-H06|Kate Perez|BIN Taxonomy Match below Phylum (Jun 2019)|BOL...
Limnophyes minimus|BIOUG43080-B09|Kate Perez|BIN Taxonomy Match below Phylum (Jun 2019)|BOL...
Limnophyes minimus|BIOUG43079-G11|Kate Perez|BIN Taxonomy Match below Phylum (Jun 2019)|BOL...
Limnophyes minimus|BIOUG43079-E08|Kate Perez|BIN Taxonomy Match below Phylum (Jun 2019)|BOL...
Limnophyes minimus|ZSM-DIP-33172-F05|Caroline Chimeno|BIN Taxonomy Match (Jan 2022)|BOLD ...
Limnophyes minimus|BIOUG43076-C07|Kate Perez|BIN Taxonomy Match below Phylum (Jun 2019)|BOL...
Limnophyes minimus|BIOUG43075-C07|Kate Perez|BIN Taxonomy Match below Phylum (Jun 2019)|BOL...
Limnophyes minimus|BIOUG43074-G07|Kate Perez|BIN Taxonomy Match below Phylum (Jun 2019)|BOL...
Limnophyes minimus|BIOUG15762-A11|Kate Perez|BIN Taxonomy Match|BOLD:AAA8204
Limnophyes minimus|BIOUG15762-A03|Kate Perez|BIN Taxonomy Match|BOLD:AAA8204
Limnophyes minimus|BIOUG42664-E05|Kate Perez|BIN Taxonomy Match below Phylum (Jun 2019)|BOL...
Limnophyes minimus|BIOUG42662-F12|Kate Perez|BIN Taxonomy Match below Phylum (Jun 2019)|BOL...
Limnophyes minimus|BIOUG15791-F06|Kate Perez|BIN Taxonomy Match|BOLD:AAA8204
Limnophyes minimus|BIOUG15791-F04|Kate Perez|BIN Taxonomy Match|BOLD:AAA8204
Limnophyes minimus|CH-OSF28|Elisabeth Stur||BOLD:AAA8204
Limnophyes minimus|CH-OSF27|Elisabeth Stur||BOLD:AAA8204
Limnophyes minimus|CH-eik98|Elisabeth Stur||BOLD:AAA8204
Limnophyes minimus|CH-eik02|Elisabeth Stur||BOLD:AAA8204
Limnophyes minimus|BIOUG15806-H01|Kate Perez|BIN Taxonomy Match|BOLD:AAA8204
Limnophyes minimus|BIOUG15800-A10|Kate Perez|BIN Taxonomy Match|BOLD:AAA8204
Limnophyes minimus|BIOUG15799-E03|Kate Perez|BIN Taxonomy Match|BOLD:AAA8204
Limnophyes minimus|BIOUG15966-F06|Kate Perez|BIN Taxonomy Match|BOLD:AAA8204
Limnophyes minimus|BIOUG15761-C04|Kate Perez|BIN Taxonomy Match|BOLD:AAA8204
Limnophyes minimus|BIOUG15761-B05|Kate Perez|BIN Taxonomy Match|BOLD:AAA8204
Limnophyes minimus|BIOUG08136-B07|Kate Perez|BIN Taxonomy Match|BOLD:AAA8204
Limnophyes minimus|BIOUG15762-A08|Kate Perez|BIN Taxonomy Match|BOLD:AAA8204
Limnophyes minimus|BIOUG05778-F09|Kate Perez|BIN Taxonomy Match|BOLD:AAA8204
Limnophyes minimus|BIOUG15720-D05|Kate Perez|BIN Taxonomy Match|BOLD:AAA8204
Limnophyes minimus|BIOUG15795-B03|Kate Perez|BIN Taxonomy Match|BOLD:AAA8204
Limnophyes minimus|BIOUG15793-F06|Kate Perez|BIN Taxonomy Match|BOLD:AAA8204
Limnophyes minimus|BIOUG15793-D08|Kate Perez|BIN Taxonomy Match|BOLD:AAA8204
Limnophyes minimus|BIOUG15792-B02|Kate Perez|BIN Taxonomy Match|BOLD:AAA8204
Limnophyes minimus|BIOUG15791-H08|Kate Perez|BIN Taxonomy Match|BOLD:AAA8204
Limnophyes minimus|BIOUG15791-F03|Kate Perez|BIN Taxonomy Match|BOLD:AAA8204
Limnophyes minimus|BIOUG07753-B09|Kate Perez|BIN Taxonomy Match|BOLD:AAA8204
Limnophyes minimus|BIOUG07753-A12|Kate Perez|BIN Taxonomy Match|BOLD:AAA8204
Limnophyes minimus|BIOUG15793-E12|Kate Perez|BIN Taxonomy Match|BOLD:AAA8204
Limnophyes minimus|BIOUG15807-A04|Kate Perez|BIN Taxonomy Match|BOLD:AAA8204
Limnophyes minimus|BIOUG15465-F09|Kate Perez|BIN Taxonomy Match|BOLD:AAA8204
Limnophyes minimus|BIOUG15719-H08|Kate Perez|BIN Taxonomy Match|BOLD:AAA8204
Limnophyes minimus|ZSM-DIP-33146-B10|Caroline Chimeno|BIN Taxonomy Match (Jan 2022)|BOLD ...
Limnophyes minimus|ZSM-DIP-33146-A05|Caroline Chimeno|BIN Taxonomy Match (Jan 2022)|BOLD ...
Limnophyes minimus|ZSM-DIP-33137-F07|Caroline Chimeno|BIN Taxonomy Match (Jan 2022)|BOLD ...
Limnophyes minimus|ZSM-DIP-33137-F06|Caroline Chimeno|BIN Taxonomy Match (Jan 2022)|BOLD ...
Limnophyes minimus|ZSM-DIP-33145-A10|Caroline Chimeno|BIN Taxonomy Match (Jan 2022)|BOLD ...
Limnophyes minimus|ZSM-DIP-33129-B08|Caroline Chimeno|BIN Taxonomy Match (Jan 2022)|BOLD ...
Limnophyes minimus|ZSM-DIP-33137-E12|Caroline Chimeno|BIN Taxonomy Match (Jan 2022)|BOLD ...
Limnophyes minimus|ZSM-DIP-33137-E07|Caroline Chimeno|BIN Taxonomy Match (Jan 2022)|BOLD ...
Limnophyes minimus|ZSM-DIP-33133-H08|Caroline Chimeno|BIN Taxonomy Match (Jan 2022)|BOLD ...
Limnophyes minimus|ZSM-DIP-33133-E02|Caroline Chimeno|BIN Taxonomy Match (Jan 2022)|BOLD ...
Limnophyes minimus|ZSM-DIP-33133-D11|Caroline Chimeno|BIN Taxonomy Match (Jan 2022)|BOLD ...
Limnophyes minimus|ZSM-DIP-33133-D03|Caroline Chimeno|BIN Taxonomy Match (Jan 2022)|BOLD ...
Limnophyes minimus|BIOUG15855-D05|Kate Perez|BIN Taxonomy Match|BOLD:AAA8204
Limnophyes minimus|BIOUG15460-H02|Kate Perez|BIN Taxonomy Match|BOLD:AAA8204
Limnophyes minimus|ZSM-DIP-33142-B02|Caroline Chimeno|BIN Taxonomy Match (Jan 2022)|BOLD ...
Limnophyes minimus|ZSM-DIP-33141-H08|Caroline Chimeno|BIN Taxonomy Match (Jan 2022)|BOLD ...
Limnophyes minimus|ZSM-DIP-33131-E10|Caroline Chimeno|BIN Taxonomy Match (Jan 2022)|BOLD ...
Limnophyes minimus|ZSM-DIP-33131-C12|Caroline Chimeno|BIN Taxonomy Match (Jan 2022)|BOLD ...
Limnophyes minimus|ZSM-DIP-33143-A07|Caroline Chimeno|BIN Taxonomy Match (Jan 2022)|BOLD ...
Limnophyes minimus|BIOUG42676-D12|Kate Perez|BIN Taxonomy Match below Phylum (Jun 2019)|BOL...
Limnophyes minimus|CH-eik136|Elisabeth Stur||BOLD:AAA8204
Limnophyes minimus|CH-eik132|Elisabeth Stur||BOLD:AAA8204
Limnophyes minimus|ZSM-DIP-33133-H10|Caroline Chimeno|BIN Taxonomy Match (Jan 2022)|BOLD ...
Limnophyes minimus|BIOUG42948-C07|Kate Perez|BIN Taxonomy Match below Phylum (Jun 2019)|BOL...
Limnophyes minimus|BIOUG08094-B01|Kate Perez|BIN Taxonomy Match|BOLD:AAA8204
Limnophyes minimus|BIOUG05897-A09|Kate Perez|BIN Taxonomy Match|BOLD:AAA8204
Limnophyes minimus|BIOUG15460-A03|Kate Perez|BIN Taxonomy Match|BOLD:AAA8204
Limnophyes minimus|BIOUG15459-C12|Kate Perez|BIN Taxonomy Match|BOLD:AAA8204
Limnophyes minimus|BIOUG15795-B04|Kate Perez|BIN Taxonomy Match|BOLD:AAA8204
Limnophyes minimus|BIOUG15793-B09|Kate Perez|BIN Taxonomy Match|BOLD:AAA8204
Limnophyes minimus|BIOUG42653-A02|Kate Perez|BIN Taxonomy Match below Phylum (Jun 2019)|BOL...
Limnophyes minimus|BIOUG42948-F02|Kate Perez|BIN Taxonomy Match below Phylum (Jun 2019)|BOL...
Limnophyes minimus|BIOUG42914-D03|Kate Perez|BIN Taxonomy Match below Phylum (Jun 2019)|BOL...

Limnophyes minimus|BIOUG[illegible]|Kate Perez|BIN Taxonomy Match|BOLD:AAA8204
Limnophyes minimus|BIOUG42653-A02|Kate Perez|BIN Taxonomy Match below Phylum (Jun 2019)|BOL...
Limnophyes minimus|BIOUG42948-F02|Kate Perez|BIN Taxonomy Match below Phylum (Jun 2019)|BOL...
Limnophyes minimus|BIOUG42914-D03|Kate Perez|BIN Taxonomy Match below Phylum (Jun 2019)|BOL...
Limnophyes minimus|BIOUG42756-F08|Kate Perez|BIN Taxonomy Match below Phylum (Jun 2019)|BOL...
Limnophyes minimus|BIOUG55196-B06|Stefan Schmidt|BIN Taxonomy Match (Sep 2020)|BOLD:AAA8204
Limnophyes minimus|BIOUG42664-G12|Kate Perez|BIN Taxonomy Match below Phylum (Jun 2019)|BOL...
Limnophyes minimus|BIOUG42918-D12|Kate Perez|BIN Taxonomy Match below Phylum (Jun 2019)|BOL...
Limnophyes minimus|BIOUG42917-H09|Kate Perez|BIN Taxonomy Match below Phylum (Jun 2019)|BOL...
Limnophyes minimus|BIOUG42760-G05|Kate Perez|BIN Taxonomy Match below Phylum (Jun 2019)|BOL...
Limnophyes minimus|BIOUG42921-D03|Kate Perez|BIN Taxonomy Match below Phylum (Jun 2019)|BOL...
Limnophyes minimus|BIOUG42920-A12|Kate Perez|BIN Taxonomy Match below Phylum (Jun 2019)|BOL...
Limnophyes minimus|BIOUG42757-G03|Kate Perez|BIN Taxonomy Match below Phylum (Jun 2019)|BOL...
Limnophyes minimus|BIOUG36681-C12|Kate Perez|BIN Taxonomy Match (Jul 2018)|BOLD:AAA8204
Limnophyes minimus|BIOUG07758-E11|Kate Perez|BIN Taxonomy Match|BOLD:AAA8204
Limnophyes minimus|BIOUG08140-H06|Kate Perez|BIN Taxonomy Match|BOLD:AAA8204
Limnophyes minimus|BIOUG08136-C06|Kate Perez|BIN Taxonomy Match|BOLD:AAA8204
Limnophyes minimus|BIOUG05778-F10|Kate Perez|BIN Taxonomy Match|BOLD:AAA8204
Limnophyes minimus|BIOUG05777-F09|Kate Perez|BIN Taxonomy Match|BOLD:AAA8204
Limnophyes minimus|BIOUG15948-F11|Kate Perez|BIN Taxonomy Match|BOLD:AAA8204
Limnophyes minimus|BIOUG15948-D03|Kate Perez|BIN Taxonomy Match|BOLD:AAA8204
Limnophyes minimus|BIOUG08314-E02|Kate Perez|BIN Taxonomy Match|BOLD:AAA8204
Limnophyes minimus|BIOUG15761-A06|Kate Perez|BIN Taxonomy Match|BOLD:AAA8204
Limnophyes minimus|BIOUG15719-H04|Kate Perez|BIN Taxonomy Match|BOLD:AAA8204
Limnophyes minimus|BIOUG15719-H01|Kate Perez|BIN Taxonomy Match|BOLD:AAA8204
Limnophyes minimus|BIOUG15719-F06|Kate Perez|BIN Taxonomy Match|BOLD:AAA8204
Limnophyes minimus|BIOUG08316-H08|Kate Perez|BIN Taxonomy Match|BOLD:AAA8204
Limnophyes minimus|BIOUG15763-H08|Kate Perez|BIN Taxonomy Match|BOLD:AAA8204
Limnophyes minimus|BIOUG15763-B02|Kate Perez|BIN Taxonomy Match|BOLD:AAA8204
Limnophyes minimus|BIOUG06054-E05|Kate Perez|BIN Taxonomy Match|BOLD:AAA8204
Limnophyes minimus|BIOUG05104-E08|Kate Perez|BIN Taxonomy Match|BOLD:AAA8204
Limnophyes minimus|ZSM-DIP-33130-B06|Caroline Chimeno|BIN Taxonomy Match (Jan 2022)|BOLD ...
Limnophyes minimus|ZSM-DIP-33130-B04|Caroline Chimeno|BIN Taxonomy Match (Jan 2022)|BOLD ...
Limnophyes minimus|BIOUG42960-G12|Kate Perez|BIN Taxonomy Match below Phylum (Jun 2019)|BOL...
Limnophyes minimus|BIOUG42959-H03|Kate Perez|BIN Taxonomy Match below Phylum (Jun 2019)|BOL...
Limnophyes minimus|BIOUG43074-D07|Kate Perez|BIN Taxonomy Match below Phylum (Jun 2019)|BOL...
Limnophyes minimus|BIOUG42966-D05|Kate Perez|BIN Taxonomy Match below Phylum (Jun 2019)|BOL...
Limnophyes minimus|BIOUG42933-B11|Kate Perez|BIN Taxonomy Match below Phylum (Jun 2019)|BOL...
Limnophyes minimus|BIOUG42933-B07|Kate Perez|BIN Taxonomy Match below Phylum (Jun 2019)|BOL...
Limnophyes minimus|BIOUG15761-H04|Kate Perez|BIN Taxonomy Match|BOLD:AAA8204
Limnophyes minimus|BIOUG15761-E03|Kate Perez|BIN Taxonomy Match|BOLD:AAA8204
Limnophyes minimus|BIOUG08316-G01|Kate Perez|BIN Taxonomy Match|BOLD:AAA8204
Limnophyes minimus|BIOUG15763-H07|Kate Perez|BIN Taxonomy Match|BOLD:AAA8204
Limnophyes minimus|BIOUG15764-B11|Kate Perez|BIN Taxonomy Match|BOLD:AAA8204
Limnophyes minimus|BIOUG15718-B07|Kate Perez|BIN Taxonomy Match|BOLD:AAA8204
Limnophyes minimus|BIOUG07945-A02|Kate Perez|BIN Taxonomy Match|BOLD:AAA8204
Limnophyes minimus|BIOUG08136-B05|Kate Perez|BIN Taxonomy Match|BOLD:AAA8204
Limnophyes minimus|BIOUG05776-B07|Kate Perez|BIN Taxonomy Match|BOLD:AAA8204
Limnophyes minimus|BIOUG08097-H01|Kate Perez|BIN Taxonomy Match|BOLD:AAA8204
Limnophyes minimus|ZSM-DIP-33135-C11|Caroline Chimeno|BIN Taxonomy Match (Jan 2022)|BOLD ...
Limnophyes minimus|BIOUG42707-E10|Kate Perez|BIN Taxonomy Match below Phylum (Jun 2019)|BOL...
Limnophyes minimus|BIOUG42706-A09|Kate Perez|BIN Taxonomy Match below Phylum (Jun 2019)|BOL...
Limnophyes minimus|BIOUG55111-A03|Stefan Schmidt|BIN Taxonomy Match (Sep 2020)|BOLD:AAA8204
Limnophyes minimus|BIOUG42705-B03|Kate Perez|BIN Taxonomy Match below Phylum (Jun 2019)|BOL...
Limnophyes minimus|BIOUG55413-G02|Stefan Schmidt|BIN Taxonomy Match (Sep 2020)|BOLD:AAA8204
Limnophyes minimus|BIOUG42660-A11|Kate Perez|BIN Taxonomy Match below Phylum (Jun 2019)|BOL...
Limnophyes minimus|ZSM-DIP-33133-E01|Caroline Chimeno|BIN Taxonomy Match (Jan 2022)|BOLD ...
Limnophyes minimus|ZSM-DIP-33131-F12|Caroline Chimeno|BIN Taxonomy Match (Jan 2022)|BOLD ...
Limnophyes minimus|ZSM-DIP-33131-F10|Caroline Chimeno|BIN Taxonomy Match (Jan 2022)|BOLD ...
Limnophyes minimus|ZSM-DIP-33131-F09|Caroline Chimeno|BIN Taxonomy Match (Jan 2022)|BOLD ...
Limnophyes minimus|ZSM-DIP-33131-F04|Caroline Chimeno|BIN Taxonomy Match (Jan 2022)|BOLD ...
Limnophyes minimus|BIOUG42927-C07|Kate Perez|BIN Taxonomy Match below Phylum (Jun 2019)|BOL...
Limnophyes minimus|BIOUG42759-C04|Kate Perez|BIN Taxonomy Match below Phylum (Jun 2019)|BOL...
Limnophyes minimus|BIOUG42939-D06|Kate Perez|BIN Taxonomy Match below Phylum (Jun 2019)|BOL...
Limnophyes minimus|BIOUG42938-H01|Kate Perez|BIN Taxonomy Match below Phylum (Jun 2019)|BOL...
Limnophyes minimus|BIOUG15459-B09|Kate Perez|BIN Taxonomy Match|BOLD:AAA8204
Limnophyes minimus|BIOUG07647-F01|Kate Perez|BIN Taxonomy Match|BOLD:AAA8204
Limnophyes minimus|BIOUG42663-A01|Kate Perez|BIN Taxonomy Match below Phylum (Jun 2019)|BOL...
Limnophyes minimus|BIOUG42931-E09|Kate Perez|BIN Taxonomy Match below Phylum (Jun 2019)|BOL...
Limnophyes minimus|BIOUG08317-E07|Kate Perez|BIN Taxonomy Match|BOLD:AAA8204
Limnophyes minimus|BIOUG08317-B02|Kate Perez|BIN Taxonomy Match|BOLD:AAA8204
Limnophyes minimus|ZSM-DIP-33131-C11|Caroline Chimeno|BIN Taxonomy Match (Jan 2022)|BOLD ...
Limnophyes minimus|ZSM-DIP-33130-H09|Caroline Chimeno|BIN Taxonomy Match (Jan 2022)|BOLD ...
Limnophyes minimus|ZSM-DIP-33130-G02|Caroline Chimeno|BIN Taxonomy Match (Jan 2022)|BOLD ...
Limnophyes minimus|ZSM-DIP-33130-F11|Caroline Chimeno|BIN Taxonomy Match (Jan 2022)|BOLD ...
Limnophyes minimus|CH-eik105|Elisabeth Stur||BOLD:AAA8204
Limnophyes minimus|CH-eik104|Elisabeth Stur||BOLD:AAA8204
Limnophyes minimus|CH-eik88|Elisabeth Stur||BOLD:AAA8204
Limnophyes minimus|CH-eik85|Elisabeth Stur||BOLD:AAA8204
Limnophyes minimus|BIOUG42664-F05|Kate Perez|BIN Taxonomy Match below Phylum (Jun 2019)|BOL...
Limnophyes minimus|BIOUG42664-E08|Kate Perez|BIN Taxonomy Match below Phylum (Jun 2019)|BOL...
Limnophyes minimus|BIOUG42916-A09|Kate Perez|BIN Taxonomy Match below Phylum (Jun 2019)|BOL...
Limnophyes minimus|BIOUG42755-D07|Kate Perez|BIN Taxonomy Match below Phylum (Jun 2019)|BOL...
Limnophyes minimus|BIOUG43077-G07|Kate Perez|BIN Taxonomy Match below Phylum (Jun 2019)|BOL...
Limnophyes minimus|BIOUG43077-A08|Kate Perez|BIN Taxonomy Match below Phylum (Jun 2019)|BOL...
Limnophyes minimus|ATNA257|Elisabeth Stur||BOLD:AAA8204
Limnophyes minimus|ATNA244|Elisabeth Stur||BOLD:AAA8204
Limnophyes minimus|BIOUG55125-A05|Stefan Schmidt|BIN Taxonomy Match (Sep 2020)|BOLD:AAA8204
Limnophyes minimus|BIOUG43078-B04|Kate Perez|BIN Taxonomy Match below Phylum (Jun 2019)|BOL...
Limnophyes minimus|CH-eik10|Elisabeth Stur||BOLD:AAA8204
Limnophyes minimus|CH-eik06|Elisabeth Stur||BOLD:AAA8204
Limnophyes minimus|BIOUG43076-H08|Kate Perez|BIN Taxonomy Match below Phylum (Jun 2019)|BOL...
Limnophyes minimus|ZSM-DIP-33141-B09|Caroline Chimeno|BIN Taxonomy Match (Jan 2022)|BOLD ...
Limnophyes minimus|ATNA216|Elisabeth Stur||BOLD:AAA8204
Limnophyes minimus|TRD-CH57|Elisabeth Stur||BOLD:AAA8204
Limnophyes minimus|BIOUG06052-A08|Kate Perez|BIN Taxonomy Match|BOLD:AAA8204
Limnophyes minimus|BIOUG06056-H03|Kate Perez|BIN Taxonomy Match|BOLD:AAA8204
Limnophyes minimus|BIOUG15720-D10|Kate Perez|BIN Taxonomy Match|BOLD:AAA8204
Limnophyes minimus|BIOUG15720-C10|Kate Perez|BIN Taxonomy Match|BOLD:AAA8204
Limnophyes minimus|BIOUG15763-F09|Kate Perez|BIN Taxonomy Match|BOLD:AAA8204
Limnophyes minimus|BIOUG15763-B07|Kate Perez|BIN Taxonomy Match|BOLD:AAA8204
Limnophyes minimus|ZSM-DIP-33141-D04|Caroline Chimeno|BIN Taxonomy Match (Jan 2022)|BOLD ...
Limnophyes minimus|ZSM-DIP-33141-C07|Caroline Chimeno|BIN Taxonomy Match (Jan 2022)|BOLD ...
[illegible]

Limnophyes minimus|BIOUG15763-B07|Kate Perez|BIN Taxonomy Match|BOLD:AAA8204
Limnophyes minimus|ZSM-DIP-33141-D04|Caroline Chimeno|BIN Taxonomy Match (Jan 2022)|BOLD ...
Limnophyes minimus|ZSM-DIP-33141-C07|Caroline Chimeno|BIN Taxonomy Match (Jan 2022)|BOLD ...
Limnophyes minimus|ZSM-DIP-33141-B10|Caroline Chimeno|BIN Taxonomy Match (Jan 2022)|BOLD ...
Limnophyes minimus|ZSM-DIP-33131-G11|Caroline Chimeno|BIN Taxonomy Match (Jan 2022)|BOLD ...
Limnophyes minimus|BIOUG42670-E08|Kate Perez|BIN Taxonomy Match below Phylum (Jun 2019)|BOL...
Limnophyes minimus|BIOUG55199-D12|Stefan Schmidt|BIN Taxonomy Match (Sep 2020)|BOLD:AAA8204
Limnophyes minimus|BIOUG42741-B12|Kate Perez|BIN Taxonomy Match below Phylum (Jun 2019)|BOL...
Limnophyes minimus|BIOUG42667-D01|Kate Perez|BIN Taxonomy Match below Phylum (Jun 2019)|BOL...
Limnophyes minimus|BIOUG15763-B04|Kate Perez|BIN Taxonomy Match|BOLD:AAA8204
Limnophyes minimus|BIOUG07759-E07|Kate Perez|BIN Taxonomy Match|BOLD:AAA8204
Limnophyes minimus|CCDB24183-E01|Sofia Wiedenbrug|BIN-taxonomy|BOLD:AAA8204
Limnophyes minimus|BIOUG15456-D09|Kate Perez|BIN Taxonomy Match|BOLD:AAA8204
Limnophyes minimus|BIOUG15762-C03|Kate Perez|BIN Taxonomy Match|BOLD:AAA8204
Limnophyes minimus|BIOUG15761-H09|Kate Perez|BIN Taxonomy Match|BOLD:AAA8204
Limnophyes minimus|BIOUG15720-D08|Kate Perez|BIN Taxonomy Match|BOLD:AAA8204
Limnophyes minimus|BIOUG15720-C02|Kate Perez|BIN Taxonomy Match|BOLD:AAA8204
Limnophyes minimus|ZSM-DIP-33136-E10|Caroline Chimeno|BIN Taxonomy Match (Jan 2022)|BOLD ...
Limnophyes minimus|ZSM-DIP-33136-C09|Caroline Chimeno|BIN Taxonomy Match (Jan 2022)|BOLD ...
Limnophyes minimus|ZSM-DIP-33136-C08|Caroline Chimeno|BIN Taxonomy Match (Jan 2022)|BOLD ...
Limnophyes minimus|ZSM-DIP-33136-B02|Caroline Chimeno|BIN Taxonomy Match (Jan 2022)|BOLD ...
Limnophyes minimus|ZSM-DIP-33172-F10|Caroline Chimeno|BIN Taxonomy Match (Jan 2022)|BOLD ...
Limnophyes minimus|ZSM-DIP-33144-G08|Caroline Chimeno|BIN Taxonomy Match (Jan 2022)|BOLD ...
Limnophyes minimus|ZSM-DIP-33136-B01|Caroline Chimeno|BIN Taxonomy Match (Jan 2022)|BOLD ...
Limnophyes minimus|ZSM-DIP-33136-A12|Caroline Chimeno|BIN Taxonomy Match (Jan 2022)|BOLD ...
Limnophyes minimus|ZSM-DIP-33172-H08|Caroline Chimeno|BIN Taxonomy Match (Jan 2022)|BOLD ...
Limnophyes minimus|ZSM-DIP-33172-H05|Caroline Chimeno|BIN Taxonomy Match (Jan 2022)|BOLD ...
Limnophyes minimus|ZSM-DIP-33136-F08|Caroline Chimeno|BIN Taxonomy Match (Jan 2022)|BOLD ...
Limnophyes minimus|ZSM-DIP-33136-F06|Caroline Chimeno|BIN Taxonomy Match (Jan 2022)|BOLD ...
Limnophyes minimus|CH-eik91|Elisabeth Stur||BOLD:AAA8204
Limnophyes minimus|Finnmark393|Elisabeth Stur||BOLD:AAA8204
Limnophyes minimus|ZSM-DIP-33145-H01|Caroline Chimeno|BIN Taxonomy Match (Jan 2022)|BOLD ...
Limnophyes minimus|ZSM-DIP-33145-E03|Caroline Chimeno|BIN Taxonomy Match (Jan 2022)|BOLD ...
Limnophyes minimus|ZSM-DIP-33144-G07|Caroline Chimeno|BIN Taxonomy Match (Jan 2022)|BOLD ...
Limnophyes minimus|ZSM-DIP-33144-G06|Caroline Chimeno|BIN Taxonomy Match (Jan 2022)|BOLD ...
Limnophyes minimus|ZSM-DIP-33147-B11|Caroline Chimeno|BIN Taxonomy Match (Jan 2022)|BOLD ...
Limnophyes minimus|ZSM-DIP-33147-A02|Caroline Chimeno|BIN Taxonomy Match (Jan 2022)|BOLD ...
Limnophyes minimus|ZSM-DIP-33137-F10|Caroline Chimeno|BIN Taxonomy Match (Jan 2022)|BOLD ...
Limnophyes minimus|ZSM-DIP-33137-F09|Caroline Chimeno|BIN Taxonomy Match (Jan 2022)|BOLD ...
Limnophyes minimus|ZSM-DIP-33148-C08|Caroline Chimeno|BIN Taxonomy Match (Jan 2022)|BOLD ...
Limnophyes minimus|ZSM-DIP-33137-F11|Caroline Chimeno|BIN Taxonomy Match (Jan 2022)|BOLD ...
Limnophyes minimus|ZSM-DIP-33138-A09|Caroline Chimeno|BIN Taxonomy Match (Jan 2022)|BOLD ...
Limnophyes minimus|ZSM-DIP-33148-C09|Caroline Chimeno|BIN Taxonomy Match (Jan 2022)|BOLD ...
Limnophyes minimus|ZSM-DIP-33138-A12|Caroline Chimeno|BIN Taxonomy Match (Jan 2022)|BOLD ...
Limnophyes minimus|ZSM-DIP-33138-A11|Caroline Chimeno|BIN Taxonomy Match (Jan 2022)|BOLD ...
Limnophyes minimus|BIOUG06052-E06|Kate Perez|BIN Taxonomy Match|BOLD:AAA8204
Limnophyes minimus|BIOUG06052-C03|Kate Perez|BIN Taxonomy Match|BOLD:AAA8204
Limnophyes minimus|BIOUG15464-B08|Kate Perez|BIN Taxonomy Match|BOLD:AAA8204
Limnophyes minimus|BIOUG06052-G01|Kate Perez|BIN Taxonomy Match|BOLD:AAA8204
Limnophyes minimus|BIOUG05900-C06|Kate Perez|BIN Taxonomy Match|BOLD:AAA8204
Limnophyes minimus|BIOUG15464-H02|Kate Perez|BIN Taxonomy Match|BOLD:AAA8204
Limnophyes minimus|BIOUG04366-H10|Kate Perez|BIN Taxonomy Match|BOLD:AAA8204
Limnophyes minimus|BIOUG05485-B08|Kate Perez|BIN Taxonomy Match|BOLD:AAA8204
Limnophyes minimus|BIOUG06051-B02|Kate Perez|BIN Taxonomy Match|BOLD:AAA8204
Limnophyes minimus|BIOUG05149-H10|Kate Perez|BIN Taxonomy Match|BOLD:AAA8204
Limnophyes minimus|BIOUG05900-E06|Kate Perez|BIN Taxonomy Match|BOLD:AAA8204
Limnophyes minimus|BIOUG05900-D07|Kate Perez|BIN Taxonomy Match|BOLD:AAA8204
Limnophyes minimus|BIOUG06052-F08|Kate Perez|BIN Taxonomy Match|BOLD:AAA8204
Limnophyes minimus|BIOUG05141-H09|Kate Perez|BIN Taxonomy Match|BOLD:AAA8204
Limnophyes minimus|BIOUG05149-F06|Kate Perez|BIN Taxonomy Match|BOLD:AAA8204
Limnophyes minimus|BIOUG04155-H02|Kate Perez|BIN Taxonomy Match|BOLD:AAA8204
Limnophyes minimus|BIOUG06051-H04|Kate Perez|BIN Taxonomy Match|BOLD:AAA8204
Limnophyes minimus|BIOUG06051-E06|Kate Perez|BIN Taxonomy Match|BOLD:AAA8204
Limnophyes minimus|BIOUG04433-H01|Kate Perez|BIN Taxonomy Match|BOLD:AAA8204
Limnophyes minimus|BIOUG06052-A01|Kate Perez|BIN Taxonomy Match|BOLD:AAA8204
Limnophyes minimus|BIOUG06210-B11|Kate Perez|BIN Taxonomy Match|BOLD:AAA8204
Limnophyes minimus|BIOUG06210-A07|Kate Perez|BIN Taxonomy Match|BOLD:AAA8204
Limnophyes minimus|BIOUG05780-E11|Kate Perez|BIN Taxonomy Match|BOLD:AAA8204
Limnophyes minimus|BIOUG16712-C05|Kate Perez|BIN Taxonomy Match|BOLD:AAA8204
Limnophyes minimus|BIOUG06055-A06|Kate Perez|BIN Taxonomy Match|BOLD:AAA8204
Limnophyes minimus|BIOUG15463-E01|Kate Perez|BIN Taxonomy Match|BOLD:AAA8204
Limnophyes minimus|BIOUG05141-B12|Kate Perez|BIN Taxonomy Match|BOLD:AAA8204
Limnophyes minimus|BIOUG15593-D10|Kate Perez|BIN Taxonomy Match|BOLD:AAA8204
Limnophyes minimus|BIOUG08319-G11|Kate Perez|BIN Taxonomy Match|BOLD:AAA8204
Limnophyes minimus|CCDB24183-G06|Sofia Wiedenbrug|Morphology|BOLD:AAA8204
Limnophyes minimus|BIOUG16480-C12|Kate Perez|BIN Taxonomy Match|BOLD:AAA8204
Limnophyes minimus|BIOUG15764-H02|Kate Perez|BIN Taxonomy Match|BOLD:AAA8204
Limnophyes minimus|BIOUG15766-E07|Kate Perez|BIN Taxonomy Match|BOLD:AAA8204
Limnophyes minimus|BIOUG15544-A04|Kate Perez|BIN Taxonomy Match|BOLD:AAA8204
Limnophyes minimus|BIOUG06050-G09|Kate Perez|BIN Taxonomy Match|BOLD:AAA8204
Limnophyes minimus|BIOUG05395-D12|Kate Perez|BIN Taxonomy Match|BOLD:AAA8204
Limnophyes minimus|BIOUG16476-C02|Kate Perez|BIN Taxonomy Match|BOLD:AAA8204
Limnophyes minimus|BIOUG16710-G04|Kate Perez|BIN Taxonomy Match|BOLD:AAA8204
Limnophyes minimus|BIOUG04155-H01|Kate Perez|BIN Taxonomy Match|BOLD:AAA8204
Limnophyes minimus|BIOUG16551-F02|Kate Perez|BIN Taxonomy Match|BOLD:AAA8204
Limnophyes minimus|BIOUG16432-F07|Kate Perez|BIN Taxonomy Match|BOLD:AAA8204
Limnophyes minimus|BIOUG15793-E05|Kate Perez|BIN Taxonomy Match|BOLD:AAA8204
Limnophyes minimus|BIOUG04795-C05|Kate Perez|BIN Taxonomy Match|BOLD:AAA8204
Limnophyes minimus|BIOUG05944-A11|Kate Perez|BIN Taxonomy Match|BOLD:AAA8204
Limnophyes minimus|BIOUG36736-E07|Kate Perez|BIN Taxonomy Match (May 2018)|BOLD:AAA8204
Limnophyes minimus|BIOUG06052-D11|Kate Perez|BIN Taxonomy Match|BOLD:AAA8204
Limnophyes minimus|BIOUG07505-D11|Kate Perez|BIN Taxonomy Match|BOLD:AAA8204
Limnophyes minimus|BIOUG06056-D09|Kate Perez|BIN Taxonomy Match|BOLD:AAA8204
Limnophyes minimus|BIOUG04437-G03|Kate Perez|BIN Taxonomy Match|BOLD:AAA8204
Limnophyes minimus|BIOUG05946-B05|Kate Perez|BIN Taxonomy Match|BOLD:AAA8204
Limnophyes minimus|BIOUG55190-E11|Stefan Schmidt|BIN Taxonomy Match (Sep 2020)|BOLD:AAA8204
Limnophyes minimus|BIOUG36841-D06|Kate Perez|BIN Taxonomy Match (Jul 2018)|BOLD:AAA8204
Limnophyes minimus|BIOUG04437-D08|Kate Perez|BIN Taxonomy Match|BOLD:AAA8204
Limnophyes minimus|BIOUG04212-D06|Kate Perez|BIN Taxonomy Match|BOLD:AAA8204
Limnophyes minimus|BIOUG05900-E10|Kate Perez|BIN Taxonomy Match|BOLD:AAA8204
Limnophyes minimus|BIOUG06055-A10|Kate Perez|BIN Taxonomy Match|BOLD:AAA8204
Limnophyes minimus|BIOUG05399-D04|Kate Perez|BIN Taxonomy Match|BOLD:AAA8204
Limnophyes minimus|ZSM34343-G05|Sofia Wiedenbrug|Morphology|BOLD:AAA8204

Limnophyes minimus|BIOUG06055-A10|Kate Perez|BIN Taxonomy Match|BOLD:AAA8204
Limnophyes minimus|BIOUG05399-D04|Kate Perez|BIN Taxonomy Match|BOLD:AAA8204
Limnophyes minimus|ZSM34343-G05|Sofia Wiedenbrug|Morphology|BOLD:AAA8204
Limnophyes minimus|BIOUG15458-A12|Kate Perez|BIN Taxonomy Match|BOLD:AAA8204
Limnophyes minimus|BIOUG04296-C04|Kate Perez|BIN Taxonomy Match|BOLD:AAA8204
Limnophyes minimus|BIOUG15458-D06|Kate Perez|BIN Taxonomy Match|BOLD:AAA8204
Limnophyes minimus|BIOUG15538-C05|Kate Perez|BIN Taxonomy Match|BOLD:AAA8204
Limnophyes minimus|BIOUG06210-F08|Kate Perez|BIN Taxonomy Match|BOLD:AAA8204
Limnophyes minimus|BIOUG05946-A10|Kate Perez|BIN Taxonomy Match|BOLD:AAA8204
Limnophyes minimus|CCDB22605-E12|Sofia Wiedenbrug||BOLD:AAA8204
Limnophyes minimus|BIOUG05144-H04|Kate Perez|BIN Taxonomy Match|BOLD:AAA8204
Limnophyes minimus|BIOUG04296-E06|Kate Perez|BIN Taxonomy Match|BOLD:AAA8204
Limnophyes minimus|BIOUG04089-A07|Kate Perez|BIN Taxonomy Match|BOLD:AAA8204
Limnophyes minimus|BIOUG08131-C12|Kate Perez|BIN Taxonomy Match|BOLD:AAA8204
Limnophyes minimus|BIOUG04296-E11|Kate Perez|BIN Taxonomy Match|BOLD:AAA8204
Limnophyes minimus|BIOUG15852-C07|Kate Perez|BIN Taxonomy Match|BOLD:AAA8204
Limnophyes minimus|BIOUG16433-E02|Kate Perez|BIN Taxonomy Match|BOLD:AAA8204
Limnophyes minimus|BIOUG06095-D03|Kate Perez|BIN Taxonomy Match|BOLD:AAA8204
Limnophyes minimus|ZSM-DIP-33131-F07|Caroline Chimeno|BIN Taxonomy Match (Jan 2022)|BOLD ...
Limnophyes minimus|BIOUG05105-E03|Kate Perez|BIN Taxonomy Match|BOLD:AAA8204
Limnophyes minimus|BIOUG42964-C03|Kate Perez|BIN Taxonomy Match below Phylum (Jun 2019)|BOL...
Limnophyes minimus|BIOUG04437-C11|Kate Perez|BIN Taxonomy Match|BOLD:AAA8204
Limnophyes minimus|BIOUG05395-F11|Kate Perez|BIN Taxonomy Match|BOLD:AAA8204
Limnophyes minimus|BIOUG04261-D07|Kate Perez|BIN Taxonomy Match|BOLD:AAA8204
Limnophyes minimus|BIOUG15808-A03|Kate Perez|BIN Taxonomy Match|BOLD:AAA8204
Limnophyes minimus|BIOUG16597-D01|Kate Perez|BIN Taxonomy Match|BOLD:AAA8204
Limnophyes minimus|ATNA420|Elisabeth Stur||BOLD:AAA8204
Limnophyes minimus|BIOUG16218-E08|Kate Perez|BIN Taxonomy Match|BOLD:AAA8204
Limnophyes minimus|BIOUG15806-F11|Kate Perez|BIN Taxonomy Match|BOLD:AAA8204
Limnophyes minimus|BIOUG15798-H01|Kate Perez|BIN Taxonomy Match|BOLD:AAA8204
Limnophyes minimus|BIOUG04433-D06|Kate Perez|BIN Taxonomy Match|BOLD:AAA8204
Limnophyes minimus|NHRS-BYWS000000903|Yngve Brodin|morphology|BOLD:AAA8204
Limnophyes minimus|BIOUG04216-A11|Kate Perez|BIN Taxonomy Match|BOLD:AAA8204
Limnophyes minimus|BIOUG05219-B11|Kate Perez|BIN Taxonomy Match|BOLD:AAA8204
Limnophyes minimus|BIOUG15765-B01|Kate Perez|BIN Taxonomy Match|BOLD:AAA8204
Limnophyes minimus|BIOUG16622-G07|Kate Perez|BIN Taxonomy Match|BOLD:AAA8204
Limnophyes minimus|BIOUG36725-A01|Kate Perez|BIN Taxonomy Match (May 2018)|BOLD:AAA8204
Limnophyes minimus|BIOUG08097-B12|Kate Perez|BIN Taxonomy Match|BOLD:AAA8204
Limnophyes minimus|BIOUG16597-G07|Kate Perez|BIN Taxonomy Match|BOLD:AAA8204
Limnophyes minimus|CCDB22605-D11|Sofia Wiedenbrug||BOLD:AAA8204
Limnophyes minimus|ZSM-DIP-33138-A08|Caroline Chimeno|BIN Taxonomy Match (Jan 2022)|BOLD ...
Limnophyes minimus|BIOUG05778-H02|Kate Perez|BIN Taxonomy Match|BOLD:AAA8204
Limnophyes minimus|BIOUG16205-A10|Kate Perez|BIN Taxonomy Match|BOLD:AAA8204
Limnophyes minimus|BIOUG06056-F12|Kate Perez|BIN Taxonomy Match|BOLD:AAA8204
Limnophyes minimus|BIOUG15761-H11|Kate Perez|BIN Taxonomy Match|BOLD:AAA8204
Limnophyes minimus|BIOUG15899-A04|Kate Perez|BIN Taxonomy Match|BOLD:AAA8204
Limnophyes minimus|BIOUG08369-A07|Kate Perez|BIN Taxonomy Match|BOLD:AAA8204
Limnophyes minimus|BIOUG15763-C01|Kate Perez|BIN Taxonomy Match|BOLD:AAA8204
Limnophyes minimus|BIOUG15895-F08|Kate Perez|BIN Taxonomy Match|BOLD:AAA8204
Limnophyes minimus|BIOUG15855-H07|Kate Perez|BIN Taxonomy Match|BOLD:AAA8204
Limnophyes minimus|BIOUG17178-D02|Kate Perez|BIN Taxonomy Match|BOLD:AAA8204
Limnophyes minimus|BIOUG07567-B03|Kate Perez|BIN Taxonomy Match|BOLD:AAA8204
Limnophyes minimus|BIOUG15797-H03|Kate Perez|BIN Taxonomy Match|BOLD:AAA8204
Limnophyes minimus|BIOUG15718-F08|Kate Perez|BIN Taxonomy Match|BOLD:AAA8204
Limnophyes minimus|BIOUG16434-C07|Kate Perez|BIN Taxonomy Match|BOLD:AAA8204
Limnophyes minimus|BIOUG15762-H08|Kate Perez|BIN Taxonomy Match|BOLD:AAA8204
Limnophyes minimus|BIOUG07565-E10|Kate Perez|BIN Taxonomy Match|BOLD:AAA8204
Limnophyes minimus|BIOUG15541-H07|Kate Perez|BIN Taxonomy Match|BOLD:AAA8204
Limnophyes minimus|BIOUG16712-H02|Kate Perez|BIN Taxonomy Match|BOLD:AAA8204
Limnophyes minimus|BIOUG07929-C12|Kate Perez|BIN Taxonomy Match|BOLD:AAA8204
Limnophyes minimus|BIOUG07564-C04|Kate Perez|BIN Taxonomy Match|BOLD:AAA8204
Limnophyes minimus|BIOUG08174-C07|Kate Perez|BIN Taxonomy Match|BOLD:AAA8204
Limnophyes minimus|BIOUG15794-E03|Kate Perez|BIN Taxonomy Match|BOLD:AAA8204
Limnophyes minimus|BIOUG07944-A01|Kate Perez|BIN Taxonomy Match|BOLD:AAA8204
Limnophyes minimus|BIOUG15796-B10|Kate Perez|BIN Taxonomy Match|BOLD:AAA8204
Limnophyes minimus|BIOUG15674-C07|Kate Perez|BIN Taxonomy Match|BOLD:AAA8204
Limnophyes minimus|BIOUG07809-B09|Kate Perez|BIN Taxonomy Match|BOLD:AAA8204
Limnophyes minimus|BIOUG08367-E12|Kate Perez|BIN Taxonomy Match|BOLD:AAA8204
Limnophyes minimus|BIOUG08362-A08|Kate Perez|BIN Taxonomy Match|BOLD:AAA8204
Limnophyes minimus|BIOUG08097-F12|Kate Perez|BIN Taxonomy Match|BOLD:AAA8204
Limnophyes minimus|BIOUG07809-D10|Kate Perez|BIN Taxonomy Match|BOLD:AAA8204
Limnophyes minimus|BIOUG15457-E12|Kate Perez|BIN Taxonomy Match|BOLD:AAA8204
Limnophyes minimus|BIOUG16433-F12|Kate Perez|BIN Taxonomy Match|BOLD:AAA8204
Limnophyes minimus|BIOUG43079-B09|Kate Perez|BIN Taxonomy Match below Phylum (Jun 2019)|BOL...
Limnophyes minimus|BIOUG05778-D04|Kate Perez|BIN Taxonomy Match|BOLD:AAA8204
Limnophyes minimus|BIOUG15538-A05|Kate Perez|BIN Taxonomy Match|BOLD:AAA8204
Limnophyes minimus|BIOUG05267-C03|Kate Perez|BIN Taxonomy Match|BOLD:AAA8204
Limnophyes minimus|BIOUG42961-C03|Kate Perez|BIN Taxonomy Match below Phylum (Jun 2019)|BOL...
Limnophyes minimus|BIOUG15541-D07|Kate Perez|BIN Taxonomy Match|BOLD:AAA8204
Limnophyes minimus|BIOUG07566-B10|Kate Perez|BIN Taxonomy Match|BOLD:AAA8204
Limnophyes minimus|BIOUG07929-B11|Kate Perez|BIN Taxonomy Match|BOLD:AAA8204
Limnophyes minimus|BIOUG07847-E08|Kate Perez|BIN Taxonomy Match|BOLD:AAA8204
Limnophyes minimus|BIOUG15806-H06|Kate Perez|BIN Taxonomy Match|BOLD:AAA8204
Limnophyes minimus|BIOUG07408-F07|Kate Perez|BIN Taxonomy Match|BOLD:AAA8204
Limnophyes minimus|BIOUG16434-G01|Kate Perez|BIN Taxonomy Match|BOLD:AAA8204
Limnophyes minimus|BIOUG06051-C09|Kate Perez|BIN Taxonomy Match|BOLD:AAA8204
Limnophyes minimus|ZSM-DIP-33143-A10|Caroline Chimeno|BIN Taxonomy Match (Jan 2022)|BOLD ...
Limnophyes minimus|BIOUG15652-A03|Kate Perez|BIN Taxonomy Match|BOLD:AAA8204
Limnophyes minimus|BIOUG07406-F04|Kate Perez|BIN Taxonomy Match|BOLD:AAA8204
Limnophyes minimus|BIOUG16479-C04|Kate Perez|Tree based identification|BOLD:AAA8204
Limnophyes minimus|CCDB22605-F07|Sofia Wiedenbrug||BOLD:AAA8204
Limnophyes minimus|BIOUG15899-B05|Kate Perez|BIN Taxonomy Match|BOLD:AAA8204
Limnophyes minimus|BIOUG07646-C06|Kate Perez|BIN Taxonomy Match|BOLD:AAA8204
Limnophyes minimus|BIOUG16597-E03|Kate Perez|BIN Taxonomy Match|BOLD:AAA8204
Limnophyes minimus|BIOUG05141-D03|Kate Perez|BIN Taxonomy Match|BOLD:AAA8204
Limnophyes minimus|BIOUG15543-A09|Kate Perez|BIN Taxonomy Match|BOLD:AAA8204
Limnophyes minimus|BIOUG16476-E12|Kate Perez|BIN Taxonomy Match|BOLD:AAA8204
Limnophyes minimus|BIOUG15536-B12|Kate Perez|BIN Taxonomy Match|BOLD:AAA8204
Limnophyes minimus|ZSM-DIP-33146-F11|Caroline Chimeno|BIN Taxonomy Match (Jan 2022)|BOLD ...
Limnophyes minimus|BIOUG07646-D10|Kate Perez|BIN Taxonomy Match|BOLD:AAA8204
Limnophyes minimus|BIOUG07415-F01|Kate Perez|BIN Taxonomy Match|BOLD:AAA8204
Limnophyes minimus|BIOUG15595-D07|Kate Perez|BIN Taxonomy Match|BOLD:AAA8204
Limnophyes minimus|BIOUG07414-B07|Kate Perez|BIN Taxonomy Match|BOLD:AAA8204

Limnophyes minimus|BIOUG07415-F01|Kate Perez|BIN Taxonomy Match|BOLD:AAA8204
Limnophyes minimus|BIOUG15595-D07|Kate Perez|BIN Taxonomy Match|BOLD:AAA8204
Limnophyes minimus|BIOUG07414-B07|Kate Perez|BIN Taxonomy Match|BOLD:AAA8204
Limnophyes minimus|CH-OSF67|Elisabeth Stur||BOLD:AAA8204
Limnophyes minimus|BIOUG08180-A08|Kate Perez|BIN Taxonomy Match|BOLD:AAA8204
Limnophyes minimus|BIOUG15762-G09|Kate Perez|BIN Taxonomy Match|BOLD:AAA8204
Limnophyes minimus|BIOUG15540-D10|Kate Perez|BIN Taxonomy Match|BOLD:AAA8204
Limnophyes minimus|BIOUG15764-A11|Kate Perez|BIN Taxonomy Match|BOLD:AAA8204
Limnophyes minimus|BIOUG15794-H04|Kate Perez|BIN Taxonomy Match|BOLD:AAA8204
Limnophyes minimus|BIOUG15794-F09|Kate Perez|BIN Taxonomy Match|BOLD:AAA8204
Limnophyes minimus|BIOUG07563-G08|Kate Perez|BIN Taxonomy Match|BOLD:AAA8204
Limnophyes minimus|BIOUG08125-C09|Kate Perez|BIN Taxonomy Match|BOLD:AAA8204
Limnophyes minimus|BIOUG15899-B02|Kate Perez|BIN Taxonomy Match|BOLD:AAA8204
Limnophyes minimus|BIOUG16270-E08|Kate Perez|BIN Taxonomy Match|BOLD:AAA8204
Limnophyes minimus|BIOUG15765-H01|Kate Perez|BIN Taxonomy Match|BOLD:AAA8204
Limnophyes minimus|BIOUG08255-H03|Kate Perez|BIN Taxonomy Match|BOLD:AAA8204
Limnophyes minimus|BIOUG15457-C02|Kate Perez|BIN Taxonomy Match|BOLD:AAA8204
Limnophyes minimus|BIOUG15895-A12|Kate Perez|BIN Taxonomy Match|BOLD:AAA8204
Limnophyes minimus|BIOUG08094-C12|Kate Perez|BIN Taxonomy Match|BOLD:AAA8204
Limnophyes minimus|BIOUG08260-E05|Kate Perez|BIN Taxonomy Match|BOLD:AAA8204
Limnophyes minimus|BIOUG15977-F04|Kate Perez|BIN Taxonomy Match|BOLD:AAA8204
Limnophyes minimus|BIOUG15465-B07|Kate Perez|BIN Taxonomy Match|BOLD:AAA8204
Limnophyes minimus|BIOUG16435-C08|Kate Perez|BIN Taxonomy Match|BOLD:AAA8204
Limnophyes minimus|BIOUG08097-G07|Kate Perez|BIN Taxonomy Match|BOLD:AAA8204
Limnophyes minimus|BIOUG07568-E01|Kate Perez|BIN Taxonomy Match|BOLD:AAA8204
Limnophyes minimus|BIOUG07566-G01|Kate Perez|BIN Taxonomy Match|BOLD:AAA8204
Limnophyes minimus|BIOUG36684-F03|Kate Perez|BIN Taxonomy Match (May 2018)|BOLD:AAA8204
Limnophyes minimus|BIOUG36726-G09|Kate Perez|BIN Taxonomy Match (May 2018)|BOLD:AAA8204
Limnophyes minimus|BIOUG15964-E08|Kate Perez|BIN Taxonomy Match|BOLD:AAA8204
Limnophyes minimus|BIOUG16597-F05|Kate Perez|BIN Taxonomy Match|BOLD:AAA8204
Limnophyes minimus|BIOUG07565-A01|Kate Perez|BIN Taxonomy Match|BOLD:AAA8204
Limnophyes minimus|BIOUG07945-H06|Kate Perez|BIN Taxonomy Match|BOLD:AAA8204
Limnophyes minimus|BIOUG15652-F02|Kate Perez|BIN Taxonomy Match|BOLD:AAA8204
Limnophyes minimus|BIOUG15596-C08|Kate Perez|BIN Taxonomy Match|BOLD:AAA8204
Limnophyes minimus|BIOUG07564-C09|Kate Perez|BIN Taxonomy Match|BOLD:AAA8204
Limnophyes minimus|BIOUG07929-G06|Kate Perez|BIN Taxonomy Match|BOLD:AAA8204
Limnophyes minimus|BIOUG04437-C10|Kate Perez|BIN Taxonomy Match|BOLD:AAA8204
Limnophyes minimus|BIOUG15761-D04|Kate Perez|BIN Taxonomy Match|BOLD:AAA8204
Limnophyes minimus|BIOUG15459-A02|Kate Perez|BIN Taxonomy Match|BOLD:AAA8204
Limnophyes minimus|BIOUG36762-E07|Kate Perez|BIN Taxonomy Match (May 2018)|BOLD:AAA8204
Limnophyes minimus|BIOUG42687-D04|Kate Perez|BIN Taxonomy Match below Phylum (Jun 2019)|BOL...
Limnophyes minimus|BIOUG16478-E09|Kate Perez|BIN Taxonomy Match|BOLD:AAA8204
Limnophyes minimus|BIOUG15762-G01|Kate Perez|BIN Taxonomy Match|BOLD:AAA8204
Limnophyes minimus|BIOUG15674-F10|Kate Perez|BIN Taxonomy Match|BOLD:AAA8204
Limnophyes minimus|BIOUG42919-H09|Kate Perez|BIN Taxonomy Match below Phylum (Jun 2019)|BOL...
Limnophyes minimus|BIOUG42759-H06|Kate Perez|BIN Taxonomy Match below Phylum (Jun 2019)|BOL...
Limnophyes minimus|BIOUG42675-D02|Kate Perez|BIN Taxonomy Match below Phylum (Jun 2019)|BOL...
Limnophyes minimus|BIOUG42680-A09|Kate Perez|BIN Taxonomy Match below Phylum (Jun 2019)|BOL...
Limnophyes minimus|BIOUG42964-F09|Kate Perez|BIN Taxonomy Match below Phylum (Jun 2019)|BOL...
Limnophyes minimus|BIOUG42966-C10|Kate Perez|BIN Taxonomy Match below Phylum (Jun 2019)|BOL...
Limnophyes minimus|ZSM-DIP-33141-H05|Caroline Chimeno|BIN Taxonomy Match (Jan 2022)|BOLD ...
Limnophyes minimus|ZSM-DIP-33137-E08|Caroline Chimeno|BIN Taxonomy Match (Jan 2022)|BOLD ...
Limnophyes minimus|BIOUG42921-F04|Kate Perez|BIN Taxonomy Match below Phylum (Jun 2019)|BOL...
Limnophyes minimus|BIOUG42965-B04|Kate Perez|BIN Taxonomy Match below Phylum (Jun 2019)|BOL...
Limnophyes minimus|ZSM-DIP-33137-D05|Caroline Chimeno|BIN Taxonomy Match (Jan 2022)|BOLD ...
Limnophyes minimus|BIOUG55414-E08|Stefan Schmidt|BIN Taxonomy Match (Sep 2020)|BOLD:AAA8204
Limnophyes minimus|ZSM-DIP-33131-C04|Caroline Chimeno|BIN Taxonomy Match (Jan 2022)|BOLD ...
Limnophyes minimus|BIOUG07415-D10|Kate Perez|BIN Taxonomy Match|BOLD:AAA8204
Limnophyes minimus|BIOUG16596-D03|Kate Perez|BIN Taxonomy Match|BOLD:AAA8204
Limnophyes minimus|BIOUG16626-C01|Kate Perez|BIN Taxonomy Match|BOLD:AAA8204
Limnophyes minimus|ZSM-DIP-33143-G12|Caroline Chimeno|BIN Taxonomy Match (Jan 2022)|BOLD ...
Limnophyes minimus|BIOUG16476-F09|Kate Perez|BIN Taxonomy Match|BOLD:AAA8204
Limnophyes minimus|BIOUG15809-G09|Kate Perez|BIN Taxonomy Match|BOLD:AAA8204
Limnophyes minimus|BIOUG15720-H09|Kate Perez|BIN Taxonomy Match|BOLD:AAA8204
Limnophyes minimus|BIOUG15720-B07|Kate Perez|BIN Taxonomy Match|BOLD:AAA8204
Limnophyes minimus|BIOUG08132-A12|Kate Perez|BIN Taxonomy Match|BOLD:AAA8204
Limnophyes minimus|BIOUG07758-C10|Kate Perez|BIN Taxonomy Match|BOLD:AAA8204
Limnophyes minimus|BIOUG36841-D05|Kate Perez|BIN Taxonomy Match (Jul 2018)|BOLD:AAA8204
Limnophyes minimus|BIOUG16214-B07|Kate Perez|BIN Taxonomy Match|BOLD:AAA8204
Limnophyes minimus|BIOUG05778-D07|Kate Perez|BIN Taxonomy Match|BOLD:AAA8204
Limnophyes minimus|BIOUG15455-B07|Kate Perez|BIN Taxonomy Match|BOLD:AAA8204
Limnophyes minimus|BIOUG42700-G08|Kate Perez|BIN Taxonomy Match below Phylum (Jun 2019)|BOL...
Limnophyes minimus|ZSM-DIP-33132-H10|Caroline Chimeno|BIN Taxonomy Match (Jan 2022)|BOLD ...
Limnophyes minimus|BIOUG04155-F06|Kate Perez|BIN Taxonomy Match|BOLD:AAA8204
Limnophyes minimus|BIOUG04085-C05|Kate Perez|BIN Taxonomy Match|BOLD:AAA8204
Limnophyes minimus|BIOUG05946-C08|Kate Perez|BIN Taxonomy Match|BOLD:AAA8204
Limnophyes minimus|BIOUG04433-C11|Kate Perez|BIN Taxonomy Match|BOLD:AAA8204
Limnophyes minimus|BIOUG04362-C06|Kate Perez|BIN Taxonomy Match|BOLD:AAA8204
Limnophyes minimus|BIOUG04296-E05|Kate Perez|BIN Taxonomy Match|BOLD:AAA8204
Limnophyes minimus|BIOUG04153-A10|Kate Perez|BIN Taxonomy Match|BOLD:AAA8204
Limnophyes minimus|BIOUG04090-D06|Kate Perez|BIN Taxonomy Match|BOLD:AAA8204
Limnophyes minimus|BIOUG04216-E09|Kate Perez|BIN Taxonomy Match|BOLD:AAA8204
Limnophyes minimus|BIOUG15806-G08|Kate Perez|BIN Taxonomy Match|BOLD:AAA8204
Limnophyes minimus|BIOUG15762-H09|Kate Perez|BIN Taxonomy Match|BOLD:AAA8204
Limnophyes minimus|BIOUG15766-A05|Kate Perez|BIN Taxonomy Match|BOLD:AAA8204
Limnophyes minimus|BIOUG15536-D06|Kate Perez|BIN Taxonomy Match|BOLD:AAA8204
Limnophyes minimus|ZSM34343-F07|Sofia Wiedenbrug|Morphology|BOLD:AAA8204
Limnophyes minimus|ZSM34343-F11|Sofia Wiedenbrug|Morphology|BOLD:AAA8204
Limnophyes minimus|BIOUG43077-D02|Kate Perez|BIN Taxonomy Match below Phylum (Jun 2019)|BOL...
Limnophyes minimus|BIOUG15718-F02|Kate Perez|BIN Taxonomy Match|BOLD:AAA8204
Limnophyes minimus|BIOUG07929-E03|Kate Perez|BIN Taxonomy Match|BOLD:AAA8204
Limnophyes minimus|BIOUG07755-A10|Kate Perez|BIN Taxonomy Match|BOLD:AAA8204
Limnophyes minimus|BIOUG15650-F11|Kate Perez|BIN Taxonomy Match|BOLD:AAA8204
Limnophyes minimus|CCDB21606-E11|Sofia Wiedenbrug||BOLD:AAA8204
Limnophyes minimus|BIOUG42970-H03|Kate Perez|BIN Taxonomy Match below Phylum (Jun 2019)|BOL...
Limnophyes minimus|BIOUG05399-C05|Kate Perez|BIN Taxonomy Match|BOLD:AAA8204
Limnophyes minimus|BIOUG08259-B12|Kate Perez|BIN Taxonomy Match|BOLD:AAA8204
Limnophyes minimus|BIOUG55191-H10|Stefan Schmidt|BIN Taxonomy Match (Sep 2020)|BOLD:AAA8204
Limnophyes minimus|BIOUG15647-D05|Kate Perez|BIN Taxonomy Match|BOLD:AAA8204
Limnophyes minimus|BIOUG15458-G04|Kate Perez|BIN Taxonomy Match|BOLD:AAA8204
Limnophyes minimus|BIOUG05219-E04|Kate Perez|BIN Taxonomy Match|BOLD:AAA8204
Limnophyes minimus|BIOUG15542-B10|Kate Perez|BIN Taxonomy Match|BOLD:AAA8204
Limnophyes minimus|BIOUG15899-A07|Kate Perez|BIN Taxonomy Match|BOLD:AAA8204

Limnophyes minimus|BIOUG15458-G04|Kate Perez|BIN Taxonomy Match|BOLD:AAA8204
Limnophyes minimus|BIOUG05219-E04|Kate Perez|BIN Taxonomy Match|BOLD:AAA8204
Limnophyes minimus|BIOUG15542-B10|Kate Perez|BIN Taxonomy Match|BOLD:AAA8204
Limnophyes minimus|BIOUG15899-A07|Kate Perez|BIN Taxonomy Match|BOLD:AAA8204
Limnophyes minimus|BIOUG15458-B10|Kate Perez|BIN Taxonomy Match|BOLD:AAA8204
Limnophyes minimus|BIOUG42668-E09|Kate Perez|BIN Taxonomy Match below Phylum (Jun 2019)|BOL...
Limnophyes minimus|BIOUG06053-C05|Kate Perez|BIN Taxonomy Match|BOLD:AAA8204
Limnophyes minimus|BIOUG05218-H09|Kate Perez|BIN Taxonomy Match|BOLD:AAA8204
Limnophyes minimus|BIOUG05110-B04|Kate Perez|BIN Taxonomy Match|BOLD:AAA8204
Limnophyes minimus|BIOUG05218-H05|Kate Perez|BIN Taxonomy Match|BOLD:AAA8204
Limnophyes minimus|BIOUG06055-E06|Kate Perez|BIN Taxonomy Match|BOLD:AAA8204
Limnophyes minimus|BIOUG07407-A03|Kate Perez|BIN Taxonomy Match|BOLD:AAA8204
Limnophyes minimus|BIOUG07757-A01|Kate Perez|BIN Taxonomy Match|BOLD:AAA8204
Limnophyes minimus|ZSM-DIP-33143-B10|Caroline Chimeno|BIN Taxonomy Match (Jan 2022)|BOLD ...
Limnophyes minimus|BIOUG07413-B08|Kate Perez|BIN Taxonomy Match|BOLD:AAA8204
Limnophyes minimus|BIOUG07945-B05|Kate Perez|BIN Taxonomy Match|BOLD:AAA8204
Limnophyes minimus|BIOUG08092-F08|Kate Perez|BIN Taxonomy Match|BOLD:AAA8204
Limnophyes minimus|ZSM-DIP-33142-B01|Caroline Chimeno|BIN Taxonomy Match (Jan 2022)|BOLD ...
Limnophyes minimus|BIOUG07414-B12|Kate Perez|BIN Taxonomy Match|BOLD:AAA8204
Limnophyes minimus|BIOUG08315-C02|Kate Perez|BIN Taxonomy Match|BOLD:AAA8204
Limnophyes minimus|ZSM-DIP-33172-F06|Caroline Chimeno|BIN Taxonomy Match (Jan 2022)|BOLD ...
Limnophyes minimus|ZSM-DIP-33130-D06|Caroline Chimeno|BIN Taxonomy Match (Jan 2022)|BOLD ...
Limnophyes minimus|ZSM-DIP-33137-E06|Caroline Chimeno|BIN Taxonomy Match (Jan 2022)|BOLD ...
Limnophyes minimus|BIOUG42751-B02|Kate Perez|BIN Taxonomy Match below Phylum (Jun 2019)|BOL...
Limnophyes minimus|BIOUG07805-F10|Kate Perez|BIN Taxonomy Match|BOLD:AAA8204
Limnophyes minimus|BIOUG15651-F04|Kate Perez|BIN Taxonomy Match|BOLD:AAA8204
Limnophyes minimus|BIOUG07755-B08|Kate Perez|BIN Taxonomy Match|BOLD:AAA8204
Limnophyes minimus|BIOUG15565-B03|Kate Perez|BIN Taxonomy Match|BOLD:AAA8204
Limnophyes minimus|BIOUG15540-D06|Kate Perez|BIN Taxonomy Match|BOLD:AAA8204
Limnophyes minimus|BIOUG06056-F11|Kate Perez|BIN Taxonomy Match|BOLD:AAA8204
Limnophyes minimus|BIOUG05218-F07|Kate Perez|BIN Taxonomy Match|BOLD:AAA8204
Limnophyes minimus|BIOUG15457-H07|Kate Perez|Tree based identification|BOLD:AAA8204
Limnophyes minimus|BIOUG07565-B08|Kate Perez|BIN Taxonomy Match|BOLD:AAA8204
Limnophyes minimus|BIOUG07415-C04|Sofia Wiedenbrug|Morphology
Chironomidae|BIOUG05269-B06|Meredith Miller|Tree-based Identification (Feb 2019)
Limnophyes minimus|BIOUG05269-A08|Kate Perez|BIN Taxonomy Match|BOLD:AAA8204
Limnophyes minimus|BIOUG16480-E11|Kate Perez|BIN Taxonomy Match|BOLD:AAA8204
Limnophyes minimus|BIOUG15855-C12|Kate Perez|BIN Taxonomy Match|BOLD:AAA8204
Limnophyes minimus|ZSM34343-G02|Sofia Wiedenbrug|Morphology|BOLD:AAA8204
Limnophyes minimus|BIOUG15793-E07|Kate Perez|BIN Taxonomy Match|BOLD:AAA8204
Limnophyes minimus|BIOUG15184-G11|Kate Perez|BIN Taxonomy Match|BOLD:AAA8204
Limnophyes minimus|BIOUG15540-B12|Kate Perez|BIN Taxonomy Match|BOLD:AAA8204
Limnophyes minimus|CH-OSF11|Elisabeth Stur||BOLD:AAA8204
Limnophyes minimus|ZSM-DIP-33132-D01|Caroline Chimeno|BIN Taxonomy Match (Jan 2022)|BOLD ...
Limnophyes minimus|ZSM-DIP-33170-G04|Caroline Chimeno|BIN Taxonomy Match (Jan 2022)|BOLD ...
Limnophyes minimus|BIOUG06056-F09|Kate Perez|BIN Taxonomy Match|BOLD:AAA8204
Limnophyes minimus|BIOUG04437-D12|Kate Perez|BIN Taxonomy Match|BOLD:AAA8204
Limnophyes minimus|BIOUG05109-G09|Kate Perez|BIN Taxonomy Match|BOLD:AAA8204
Limnophyes minimus|BIOUG07805-H03|Kate Perez|BIN Taxonomy Match|BOLD:AAA8204
Limnophyes minimus|BIOUG16627-B09|Kate Perez|BIN Taxonomy Match|BOLD:AAA8204
Limnophyes minimus|BIOUG05266-D11|Kate Perez|BIN Taxonomy Match|BOLD:AAA8204
Limnophyes minimus|BIOUG08131-F11|Kate Perez|BIN Taxonomy Match|BOLD:AAA8204
Limnophyes minimus|BIOUG07647-G09|Kate Perez|BIN Taxonomy Match|BOLD:AAA8204
Limnophyes minimus|BIOUG07759-B12|Kate Perez|BIN Taxonomy Match|BOLD:AAA8204
Limnophyes minimus|BIOUG15766-C01|Kate Perez|BIN Taxonomy Match|BOLD:AAA8204
Limnophyes minimus|BIOUG42761-G06|Kate Perez|BIN Taxonomy Match below Phylum (Jun 2019)|BOL...
Limnophyes minimus|BIOUG15766-A06|Kate Perez|BIN Taxonomy Match|BOLD:AAA8204
Limnophyes minimus|CCDB21606-E12|Sofia Wiedenbrug||BOLD:AAA8204
Limnophyes minimus|BIOUG42966-B05|Kate Perez|BIN Taxonomy Match below Phylum (Jun 2019)|BOL...
Limnophyes minimus|BIOUG15718-B04|Kate Perez|BIN Taxonomy Match|BOLD:AAA8204
Limnophyes minimus|BIOUG08174-F09|Kate Perez|BIN Taxonomy Match|BOLD:AAA8204
Limnophyes minimus|Finnmark647|Elisabeth Stur||BOLD:AAA8204
Limnophyes minimus|ZSM-DIP-33131-F05|Caroline Chimeno|BIN Taxonomy Match (Jan 2022)|BOLD ...
Limnophyes minimus|BIOUG42960-A02|Kate Perez|BIN Taxonomy Match below Phylum (Jun 2019)|BOL...
Limnophyes minimus|CH-eik29|Elisabeth Stur||BOLD:AAA8204
Limnophyes minimus|CH-eik14|Elisabeth Stur||BOLD:AAA8204
Limnophyes minimus|NHRS-BYWS000000152|Yngve Brodin|morphology|BOLD:AAA8204
Limnophyes minimus|CH-eik30|Elisabeth Stur||BOLD:AAA8204
Limnophyes minimus|NHRS-BYWS000000560|Yngve Brodin|morphology|BOLD:AAA8204
Limnophyes minimus|NHRS-BYWS000000187|Yngve Brodin|morphology|BOLD:AAA8204
Limnophyes minimus|CCDB24183-G10|Sofia Wiedenbrug|Morphology|BOLD:AAA8204
Limnophyes minimus|CCDB24183-B01|Sofia Wiedenbrug|Morphology|BOLD:AAA8204
Limnophyes minimus|ZSM-DIP-33141-C06|Caroline Chimeno|BIN Taxonomy Match (Jan 2022)|BOLD ...
Limnophyes minimus|ZSM-DIP-33131-G05|Caroline Chimeno|BIN Taxonomy Match (Jan 2022)|BOLD ...
Limnophyes minimus|BIOUG54162-C10|Stefan Schmidt|BIN Taxonomy Match (Sep 2020)|BOLD:AAA8204
Limnophyes minimus|CCDB24183-H03|Sofia Wiedenbrug|BIN-taxonomy|BOLD:AAA8204
Limnophyes minimus|ZSM-DIP-33138-H05|Caroline Chimeno|BIN Taxonomy Match (Jan 2022)|BOLD ...
Limnophyes minimus|ZSM-DIP-33138-B01|Caroline Chimeno|BIN Taxonomy Match (Jan 2022)|BOLD ...
Limnophyes minimus|NHRS-BYWS000000232|Yngve Brodin|morphology|BOLD:AAA8204
Limnophyes minimus|ZSM-DIP-33140-D10|Caroline Chimeno|BIN Taxonomy Match (Jan 2022)|BOLD ...
Limnophyes minimus|ZMUO.024691|Lauri Paasivirta||BOLD:AAA8204
Limnophyes minimus|ZMUO.024690|Lauri Paasivirta||BOLD:AAA8204
Limnophyes minimus|GBOL11560|Sofia Wiedenbrug||BOLD:AAA8204
Limnophyes minimus|ZMUO.024266|Lauri Paasivirta||BOLD:AAA8204
Limnophyes minimus|CCDB22605-D10|Sofia Wiedenbrug||BOLD:AAA8204
Limnophyes minimus|CCDB22605-D08|Sofia Wiedenbrug||BOLD:AAA8204
Limnophyes minimus|ATNA388|Elisabeth Stur||BOLD:AAA8204
Limnophyes minimus|RIN_CH94|Mona Renate Saursaunet|BOLD Identification engine|BOLD:AAA8204
Limnophyes minimus|CCDB22605-E11|Sofia Wiedenbrug||BOLD:AAA8204
Limnophyes minimus|BIOUG05141-E08|Kate Perez|BIN Taxonomy Match|BOLD:AAA8204
Limnophyes minimus|BIOUG16597-F11|Kate Perez|BIN Taxonomy Match|BOLD:AAA8204
Limnophyes minimus|BIOUG15764-C09|Kate Perez|BIN Taxonomy Match|BOLD:AAA8204
Limnophyes minimus|BIOUG16328-C04|Kate Perez|BIN Taxonomy Match|BOLD:AAA8204
Limnophyes minimus|BIOUG15853-E03|Kate Perez|BIN Taxonomy Match|BOLD:AAA8204
Limnophyes minimus|BIOUG15720-D12|Kate Perez|BIN Taxonomy Match|BOLD:AAA8204
Limnophyes minimus|BIOUG15762-E04|Kate Perez|BIN Taxonomy Match|BOLD:AAA8204
Limnophyes minimus|BIOUG16597-E01|Kate Perez|BIN Taxonomy Match|BOLD:AAA8204
Limnophyes minimus|BIOUG15764-B03|Kate Perez|BIN Taxonomy Match|BOLD:AAA8204
Limnophyes minimus|BIOUG15948-A05|Kate Perez|BIN Taxonomy Match|BOLD:AAA8204
Limnophyes minimus|BIOUG16298-E05|Kate Perez|BIN Taxonomy Match|BOLD:AAA8204
Limnophyes minimus|BIOUG15563-C10|Kate Perez|BIN Taxonomy Match|BOLD:AAA8204
Limnophyes minimus|BIOUG16436-H08|Kate Perez|BIN Taxonomy Match|BOLD:AAA8204
Limnophyes minimus|BIOUG15458-E04|Kate Perez|BIN Taxonomy Match|BOLD:AAA8204
[illegible]

Limnophyes minimus|BIOUG15563-C10|Kate Perez|BIN Taxonomy Match|BOLD:AAA8204
Limnophyes minimus|BIOUG16436-H08|Kate Perez|BIN Taxonomy Match|BOLD:AAA8204
Limnophyes minimus|BIOUG15458-E04|Kate Perez|BIN Taxonomy Match|BOLD:AAA8204
Limnophyes minimus|BIOUG15806-D07|Kate Perez|BIN Taxonomy Match|BOLD:AAA8204
Limnophyes minimus|BIOUG15718-A11|Kate Perez|BIN Taxonomy Match|BOLD:AAA8204
Limnophyes minimus|BIOUG55165-D02|Stefan Schmidt|BIN Taxonomy Match (Sep 2020)|BOLD:AAA8204
Limnophyes minimus|ZSM-DIP-33170-G05|Caroline Chimeno|BIN Taxonomy Match (Jan 2022)|BOLD ...
Limnophyes minimus|ZSM-DIP-33133-D12|Caroline Chimeno|BIN Taxonomy Match (Jan 2022)|BOLD ...
Limnophyes minimus|BIOUG16596-H11|Kate Perez|BIN Taxonomy Match|BOLD:AAA8204
Limnophyes minimus|BIOUG15653-F08|Kate Perez|BIN Taxonomy Match|BOLD:AAA8204
Limnophyes minimus|BIOUG05778-B05|Kate Perez|BIN Taxonomy Match|BOLD:AAA8204
Limnophyes minimus|BIOUG05400-E02|Kate Perez|BIN Taxonomy Match|BOLD:AAA8204
Limnophyes minimus|BIOUG07567-H02|Kate Perez|BIN Taxonomy Match|BOLD:AAA8204
Limnophyes minimus|BIOUG05104-F12|Kate Perez|BIN Taxonomy Match|BOLD:AAA8204
Limnophyes minimus|BIOUG07568-H08|Kate Perez|BIN Taxonomy Match|BOLD:AAA8204
Limnophyes minimus|ZSM-DIP-33133-H01|Caroline Chimeno|BIN Taxonomy Match (Jan 2022)|BOLD ...
Limnophyes minimus|BIOUG04212-B11|Kate Perez|BIN Taxonomy Match|BOLD:AAA8204
Limnophyes minimus|BIOUG04437-H03|Kate Perez|BIN Taxonomy Match|BOLD:AAA8204
Limnophyes minimus|BIOUG06053-A02|Kate Perez|BIN Taxonomy Match|BOLD:AAA8204
Limnophyes minimus|BIOUG07930-A10|Kate Perez|BIN Taxonomy Match|BOLD:AAA8204
Limnophyes minimus|BIOUG04269-D02|Kate Perez|BIN Taxonomy Match|BOLD:AAA8204
Limnophyes minimus|BIOUG04212-G01|Kate Perez|BIN Taxonomy Match|BOLD:AAA8204
Limnophyes minimus|BIOUG15952-A01|Kate Perez|Tree based identification|BOLD:AAA8204
Limnophyes minimus|ZSM-DIP-33132-G12|Caroline Chimeno|BIN Taxonomy Match (Jan 2022)|BOLD ...
Limnophyes minimus|ZSM-DIP-33137-E10|Caroline Chimeno|BIN Taxonomy Match (Jan 2022)|BOLD ...
Limnophyes minimus|BIOUG06058-H11|Kate Perez|BIN Taxonomy Match|BOLD:AAA8204
Limnophyes minimus|BIOUG06516-D11|Kate Perez|BIN Taxonomy Match|BOLD:AAA8204
Limnophyes minimus|BIOUG07413-F11|Kate Perez|BIN Taxonomy Match|BOLD:AAA8204
Limnophyes minimus|BIOUG08179-C12|Kate Perez|BIN Taxonomy Match|BOLD:AAA8204
Limnophyes minimus|BIOUG15769-D06|Kate Perez|BIN Taxonomy Match|BOLD:AAA8204
Limnophyes minimus|CCDB22605-D07|Sofia Wiedenbrug||BOLD:AAA8204
Limnophyes minimus|ZSM-DIP-33149-D09|Caroline Chimeno|BIN Taxonomy Match (Jan 2022)|BOLD ...
Limnophyes minimus|BIOUG42945-F04|Kate Perez|BIN Taxonomy Match below Phylum (Jun 2019)|BOL...
Limnophyes minimus|BIOUG42749-A12|Kate Perez|BIN Taxonomy Match below Phylum (Jun 2019)|BOL...
Limnophyes minimus|BIOUG15598-G11|Kate Perez|BIN Taxonomy Match|BOLD:AAA8204
Limnophyes minimus|BIOUG04261-B12|Kate Perez|BIN Taxonomy Match|BOLD:AAA8204
Limnophyes minimus|ZSM-DIP-33133-E03|Caroline Chimeno|BIN Taxonomy Match (Jan 2022)|BOLD ...
Limnophyes minimus|BIOUG15764-H05|Kate Perez|BIN Taxonomy Match|BOLD:AAA8204
Limnophyes minimus|ZSM-DIP-33172-F04|Caroline Chimeno|BIN Taxonomy Match (Jan 2022)|BOLD ...
Limnophyes minimus|ZSM-DIP-33136-E11|Caroline Chimeno|BIN Taxonomy Match (Jan 2022)|BOLD ...
Limnophyes minimus|BIOUG15795-A08|Kate Perez|Tree based identification|BOLD:AAA8204
Limnophyes minimus|CCDB21606-F03|Sofia Wiedenbrug||BOLD:AAA8204
Limnophyes minimus|ZSM-DIP-33143-G07|Caroline Chimeno|BIN Taxonomy Match (Jan 2022)|BOLD ...
Limnophyes minimus|CH-eik16|Elisabeth Stur||BOLD:AAA8204
Limnophyes minimus|BIOUG15794-B05|Kate Perez|BIN Taxonomy Match|BOLD:AAA8204
Limnophyes minimus|BIOUG06052-F02|Kate Perez|BIN Taxonomy Match|BOLD:AAA8204
Limnophyes minimus|BIOUG16597-C01|Kate Perez|BIN Taxonomy Match|BOLD:AAA8204
Limnophyes minimus|BIOUG15761-B03|Kate Perez|BIN Taxonomy Match|BOLD:AAA8204
Limnophyes minimus|CCDB22605-E10|Sofia Wiedenbrug||BOLD:AAA8204
Limnophyes minimus|ZSM-DIP-33170-G03|Caroline Chimeno|BIN Taxonomy Match (Jan 2022)|BOLD ...
Limnophyes minimus|BIOUG42742-G09|Kate Perez|BIN Taxonomy Match below Phylum (Jun 2019)|BOL...
Limnophyes minimus|BIOUG42753-A05|Kate Perez|BIN Taxonomy Match below Phylum (Jun 2019)|BOL...
Limnophyes minimus|ZSM-DIP-33170-H03|Caroline Chimeno|BIN Taxonomy Match (Jan 2022)|BOLD ...
Limnophyes minimus|BIOUG08095-G07|Kate Perez|BIN Taxonomy Match|BOLD:AAA8204
Limnophyes minimus|BIOUG55416-F02|Stefan Schmidt|BIN Taxonomy Match (Sep 2020)|BOLD:AAA8204
Limnophyes minimus|ZSM-DIP-33170-H01|Caroline Chimeno|BIN Taxonomy Match (Jan 2022)|BOLD ...
Limnophyes minimus|BIOUG42747-H06|Kate Perez|BIN Taxonomy Match below Phylum (Jun 2019)|BOL...
Limnophyes minimus|BIOUG42753-E01|Kate Perez|BIN Taxonomy Match below Phylum (Jun 2019)|BOL...
Limnophyes minimus|BIOUG43080-D04|Kate Perez|BIN Taxonomy Match below Phylum (Jun 2019)|BOL...
Limnophyes minimus|BIOUG08125-E05|Kate Perez|BIN Taxonomy Match|BOLD:AAA8204
Limnophyes minimus|ZSM-DIP-33136-F10|Caroline Chimeno|BIN Taxonomy Match (Jan 2022)|BOLD ...
Limnophyes minimus|BIOUG43078-A08|Kate Perez|BIN Taxonomy Match below Phylum (Jun 2019)|BOL...
Limnophyes minimus|BIOUG42748-F09|Kate Perez|BIN Taxonomy Match below Phylum (Jun 2019)|BOL...
Limnophyes minimus|GBOL11511|Sofia Wiedenbrug||BOLD:AAA8204
Limnophyes minimus|BIOUG07810-E08|Kate Perez|BIN Taxonomy Match|BOLD:AAA8204
Limnophyes minimus|BIOUG04216-A01|Kate Perez|BIN Taxonomy Match|BOLD:AAA8204
Limnophyes minimus|BIOUG07413-E10|Kate Perez|BIN Taxonomy Match|BOLD:AAA8204
Limnophyes minimus|BIOUG16205-D07|Kate Perez|BIN Taxonomy Match|BOLD:AAA8204
Limnophyes minimus|BIOUG15809-E02|Kate Perez|BIN Taxonomy Match|BOLD:AAA8204
Limnophyes minimus|BIOUG07569-G07|Kate Perez|BIN Taxonomy Match|BOLD:AAA8204
Limnophyes minimus|BIOUG15809-F03|Kate Perez|Tree based identification|BOLD:AAA8204
Limnophyes minimus|BIOUG15720-E10|Kate Perez|BIN Taxonomy Match|BOLD:AAA8204
Limnophyes minimus|ZSM-DIP-33149-A11|Caroline Chimeno|BIN Taxonomy Match (Jan 2022)|BOLD ...
Limnophyes minimus|ZSM-DIP-33136-F05|Caroline Chimeno|BIN Taxonomy Match (Jan 2022)|BOLD ...
Limnophyes minimus|BIOUG42679-G09|Kate Perez|BIN Taxonomy Match below Phylum (Jun 2019)|BOL...
Limnophyes minimus|BIOUG07418-D01|Kate Perez|BIN Taxonomy Match|BOLD:AAA8204
Limnophyes minimus|BIOUG42922-G02|Kate Perez|BIN Taxonomy Match below Phylum (Jun 2019)|BOL...
Limnophyes minimus|ZSM-DIP-33138-A10|Caroline Chimeno|BIN Taxonomy Match (Jan 2022)|BOLD ...
Limnophyes minimus|BIOUG43077-F01|Kate Perez|BIN Taxonomy Match below Phylum (Jun 2019)|BOL...
Limnophyes minimus|BIOUG07568-C06|Kate Perez|BIN Taxonomy Match|BOLD:AAA8204
Limnophyes minimus|BIOUG07413-D08|Kate Perez|BIN Taxonomy Match|BOLD:AAA8204
Limnophyes minimus|BIOUG07568-F11|Kate Perez|BIN Taxonomy Match|BOLD:AAA8204
Limnophyes minimus|BIOUG05144-E08|Kate Perez|BIN Taxonomy Match|BOLD:AAA8204
Limnophyes minimus|BIOUG04090-F09|Kate Perez|BIN Taxonomy Match|BOLD:AAA8204
Limnophyes minimus|BIOUG07805-D12|Kate Perez|BIN Taxonomy Match|BOLD:AAA8204
Limnophyes minimus|BIOUG07756-A12|Kate Perez|BIN Taxonomy Match|BOLD:AAA8204
Limnophyes minimus|BIOUG08258-A09|Kate Perez|BIN Taxonomy Match|BOLD:AAA8204
Limnophyes minimus|ZSM-DIP-33140-F06|Caroline Chimeno|BIN Taxonomy Match (Jan 2022)|BOLD ...
Limnophyes minimus|ZSM-DIP-33149-C10|Caroline Chimeno|BIN Taxonomy Match (Jan 2022)|BOLD ...
Limnophyes minimus|BIOUG42741-B09|Kate Perez|BIN Taxonomy Match below Phylum (Jun 2019)|BOL...
Limnophyes minimus|BIOUG42748-H02|Kate Perez|BIN Taxonomy Match below Phylum (Jun 2019)|BOL...
Limnophyes minimus|BIOUG42746-H09|Kate Perez|BIN Taxonomy Match below Phylum (Jun 2019)|BOL...
Limnophyes minimus|ZSM-DIP-33143-B12|Caroline Chimeno|BIN Taxonomy Match (Jan 2022)|BOLD ...
Limnophyes minimus|ZSM-DIP-33143-C01|Caroline Chimeno|BIN Taxonomy Match (Jan 2022)|BOLD ...
Limnophyes minimus|BIOUG15454-G04|Kate Perez|BIN Taxonomy Match|BOLD:AAA8204
Limnophyes minimus|BIOUG15459-D02|Kate Perez|BIN Taxonomy Match|BOLD:AAA8204
Limnophyes minimus|BIOUG42746-D04|Kate Perez|BIN Taxonomy Match below Phylum (Jun 2019)|BOL...
Limnophyes minimus|ZSM-DIP-33135-H02|Caroline Chimeno|BIN Taxonomy Match (Jan 2022)|BOLD ...
Limnophyes minimus|ZSM-DIP-33130-H04|Caroline Chimeno|BIN Taxonomy Match (Jan 2022)|BOLD ...
Limnophyes minimus|ZSM-DIP-33137-C06|Caroline Chimeno|BIN Taxonomy Match (Jan 2022)|BOLD ...
Limnophyes minimus|BIOUG42965-E06|Kate Perez|BIN Taxonomy Match below Phylum (Jun 2019)|BOL...
Limnophyes minimus|CH-OSF108|Elisabeth Stur||BOLD:AAA8204
Limnophyes minimus|BIOUG16625-G12|Kate Perez|BIN Taxonomy Match|BOLD:AAA8204

Limnophyes minimus|BIOUG42965-E06|Kate Perez|BIN Taxonomy Match below Phylum (Jun 2019)|BOL...
Limnophyes minimus|CH-OSF108|Elisabeth Stur||BOLD:AAA8204
Limnophyes minimus|BIOUG16625-G12|Kate Perez|BIN Taxonomy Match|BOLD:AAA8204
Limnophyes minimus|BIOUG15895-E10|Kate Perez|BIN Taxonomy Match|BOLD:AAA8204
Limnophyes minimus|BIOUG15808-A02|Kate Perez|BIN Taxonomy Match|BOLD:AAA8204
Limnophyes minimus|BIOUG15895-A11|Kate Perez|BIN Taxonomy Match|BOLD:AAA8204
Limnophyes minimus|BIOUG16597-D06|Kate Perez|BIN Taxonomy Match|BOLD:AAA8204
Limnophyes minimus|BIOUG15454-B12|Kate Perez|BIN Taxonomy Match|BOLD:AAA8204
Limnophyes minimus|BIOUG17224-D03|Kate Perez|BIN Taxonomy Match|BOLD:AAA8204
Limnophyes minimus|BIOUG15538-H09|Kate Perez|BIN Taxonomy Match|BOLD:AAA8204
Limnophyes minimus|BIOUG15720-F11|Kate Perez|BIN Taxonomy Match|BOLD:AAA8204
Limnophyes minimus|BIOUG15719-A11|Kate Perez|BIN Taxonomy Match|BOLD:AAA8204
Limnophyes minimus|BIOUG15763-H04|Kate Perez|BIN Taxonomy Match|BOLD:AAA8204
Limnophyes minimus|BIOUG42754-D03|Kate Perez|BIN Taxonomy Match below Phylum (Jun 2019)|BOL...
Limnophyes minimus|BIOUG43074-E01|Kate Perez|BIN Taxonomy Match below Phylum (Jun 2019)|BOL...
Limnophyes minimus|ZSM-DIP-33135-F02|Caroline Chimeno|BIN Taxonomy Match (Jan 2022)|BOLD ...
Limnophyes minimus|BIOUG42960-H08|Kate Perez|BIN Taxonomy Match below Phylum (Jun 2019)|BOL...
Limnophyes minimus|BIOUG42938-D11|Kate Perez|BIN Taxonomy Match below Phylum (Jun 2019)|BOL...
Limnophyes minimus|ZSM-DIP-33130-B05|Caroline Chimeno|BIN Taxonomy Match (Jan 2022)|BOLD ...
Limnophyes minimus|ZSM-DIP-33131-G01|Caroline Chimeno|BIN Taxonomy Match (Jan 2022)|BOLD ...
Limnophyes minimus|BIOUG42667-A10|Kate Perez|BIN Taxonomy Match below Phylum (Jun 2019)|BOL...
Limnophyes minimus|BIOUG55199-D11|Stefan Schmidt|BIN Taxonomy Match (Sep 2020)|BOLD:AAA8204
Limnophyes minimus|ZSM-DIP-33134-D05|Caroline Chimeno|BIN Taxonomy Match (Jan 2022)|BOLD ...
Limnophyes minimus|ZSM-DIP-33142-C09|Caroline Chimeno|BIN Taxonomy Match (Jan 2022)|BOLD ...
Limnophyes minimus|BIOUG42677-F04|Kate Perez|BIN Taxonomy Match below Phylum (Jun 2019)|BOL...
Limnophyes minimus|BIOUG42916-D10|Kate Perez|BIN Taxonomy Match below Phylum (Jun 2019)|BOL...
Limnophyes minimus|ZSM-DIP-33132-C08|Caroline Chimeno|BIN Taxonomy Match (Jan 2022)|BOLD ...
Limnophyes minimus|BIOUG42653-F12|Kate Perez|BIN Taxonomy Match below Phylum (Jun 2019)|BOL...
Limnophyes minimus|Finnmark297|Elisabeth Stur||BOLD:AAA8204
Limnophyes minimus|BIOUG42748-E10|Kate Perez|BIN Taxonomy Match below Phylum (Jun 2019)|BOL...
Limnophyes minimus|BIOUG42743-E06|Kate Perez|BIN Taxonomy Match below Phylum (Jun 2019)|BOL...
Limnophyes minimus|BIOUG07758-H06|Kate Perez|BIN Taxonomy Match|BOLD:AAA8204
Limnophyes minimus|ZSM-DIP-33143-A08|Caroline Chimeno|BIN Taxonomy Match (Jan 2022)|BOLD ...
Limnophyes minimus|ZSM-DIP-33172-H09|Caroline Chimeno|BIN Taxonomy Match (Jan 2022)|BOLD ...
Limnophyes minimus|BIOUG07805-C04|Kate Perez|BIN Taxonomy Match|BOLD:AAA8204
Limnophyes minimus|BIOUG05144-D07|Kate Perez|BIN Taxonomy Match|BOLD:AAA8204
Limnophyes minimus|BIOUG07753-G11|Kate Perez|BIN Taxonomy Match|BOLD:AAA8204
Limnophyes minimus|BIOUG05105-G09|Kate Perez|BIN Taxonomy Match|BOLD:AAA8204
Limnophyes minimus|BIOUG42755-C01|Kate Perez|BIN Taxonomy Match below Phylum (Jun 2019)|BOL...
Limnophyes minimus|BIOUG42654-F10|Kate Perez|BIN Taxonomy Match below Phylum (Jun 2019)|BOL...
Limnophyes minimus|ZSM-DIP-33131-F08|Caroline Chimeno|BIN Taxonomy Match (Jan 2022)|BOLD ...
Limnophyes minimus|BIOUG07809-H03|Kate Perez|BIN Taxonomy Match|BOLD:AAA8204
Limnophyes minimus|BIOUG07809-H07|Kate Perez|BIN Taxonomy Match|BOLD:AAA8204
Chironomidae|BIOUG07809-D07|Meredith Miller|Tree-based Identification (Feb 2019)
Limnophyes minimus|BIOUG07809-B06|Kate Perez|BIN Taxonomy Match|BOLD:AAA8204
Limnophyes minimus|BIOUG07808-F05|Kate Perez|BIN Taxonomy Match|BOLD:AAA8204
Limnophyes minimus|BIOUG07806-E08|Kate Perez|BIN Taxonomy Match|BOLD:AAA8204
Limnophyes minimus|BIOUG07808-E07|Kate Perez|BIN Taxonomy Match|BOLD:AAA8204
Limnophyes minimus|BIOUG04155-F05|Kate Perez|BIN Taxonomy Match|BOLD:AAA8204
Limnophyes minimus|BIOUG07809-A09|Kate Perez|BIN Taxonomy Match|BOLD:AAA8204
Limnophyes minimus|BIOUG05944-F07|Kate Perez|BIN Taxonomy Match|BOLD:AAA8204
Limnophyes minimus|BIOUG15719-D03|Kate Perez|BIN Taxonomy Match|BOLD:AAA8204
Limnophyes minimus|ZSM-DIP-33137-E03|Caroline Chimeno|BIN Taxonomy Match (Jan 2022)|BOLD ...
Limnophyes minimus|ZSM-DIP-33141-D01|Caroline Chimeno|BIN Taxonomy Match (Jan 2022)|BOLD ...
Limnophyes minimus|BIOUG42741-D05|Kate Perez|BIN Taxonomy Match below Phylum (Jun 2019)|BOL...
Limnophyes minimus|BIOUG42656-A08|Kate Perez|BIN Taxonomy Match below Phylum (Jun 2019)|BOL...
Limnophyes minimus|BIOUG07754-B04|Kate Perez|BIN Taxonomy Match|BOLD:AAA8204
Limnophyes minimus|CCDB21606-F04|Sofia Wiedenbrug||BOLD:AAA8204
Limnophyes minimus|CCDB21606-F02|Sofia Wiedenbrug||BOLD:AAA8204
Limnophyes minimus|BIOUG36653-H08|Kate Perez|BIN Taxonomy Match (Jul 2018)|BOLD:AAA8204
Limnophyes minimus|BIOUG36676-F06|Kate Perez|BIN Taxonomy Match (Jul 2018)|BOLD:AAA8204
Limnophyes minimus|BIOUG36678-A06|Kate Perez|BIN Taxonomy Match (Jul 2018)|BOLD:AAA8204
Limnophyes minimus|BIOUG36677-B07|Kate Perez|BIN Taxonomy Match (May 2018)|BOLD:AAA8204
Limnophyes minimus|BIOUG36676-E06|Kate Perez|BIN Taxonomy Match (Jul 2018)|BOLD:AAA8204
Limnophyes minimus|BIOUG36733-D02|Kate Perez|BIN Taxonomy Match (May 2018)|BOLD:AAA8204
Limnophyes minimus|Finnmark495|Elisabeth Stur||BOLD:AAA8204
Limnophyes minimus|BIOUG15794-E10|Kate Perez|BIN Taxonomy Match|BOLD:AAA8204
Limnophyes minimus|Finnmark628|Elisabeth Stur||BOLD:AAA8204
Limnophyes|24373-G10|Mikko J Tiusanen|Tree based identification|BOLD:ACU5176
Limnophyes|24464-B08|Mikko J Tiusanen|Tree based identification|BOLD:ACU5176
Limnophyes|24372-C12|Mikko J Tiusanen|Tree based identification|BOLD:ACU5176
Limnophyes|24465-F09|Mikko J Tiusanen|Tree based identification|BOLD:ACU5176
Limnophyes|24372-B03|Mikko J Tiusanen|Tree based identification|BOLD:ACU5176
Limnophyes|24372-D05|Mikko J Tiusanen|Tree based identification|BOLD:ACU5176
Limnophyes|24464-C05|Mikko J Tiusanen|Tree based identification|BOLD:ACU5176
Limnophyes|24501-E12|Mikko J Tiusanen|Tree based identification|BOLD:ACU5176
Limnophyes natalensis|Finnmark411|Elisabeth Stur||BOLD:AAT9715
Limnophyes minimus|Finnmark92|Elisabeth Stur||BOLD:AAM5218
Limnophyes minimus|Finnmark88|Elisabeth Stur||BOLD:AAM5218
Chironomidae|BIOUG07808-A04|Meredith Miller|Tree-based Identification (Feb 2019)
Chironomidae|BIOUG07809-A06|Meredith Miller|Digital Morphology (Feb 2019)
Limnophyes|BIOUG05395-H05|Kate Perez|BIN Taxonomy Match|BOLD:ACJ3327
Limnophyes|BIOUG08097-D11|Kate Perez|BIN Taxonomy Match|BOLD:ACJ3327
Limnophyes|BIOUG08129-C04|Kate Perez|BIN Taxonomy Match|BOLD:ACJ3327
Limnophyes|BIOUG08098-B08|Kate Perez|BIN Taxonomy Match|BOLD:ACJ3327
Limnophyes|BIOUG06052-E07|Kate Perez|BIN Taxonomy Match|BOLD:ACJ3327
Limnophyes natalensis|ATNA495|Elisabeth Stur||BOLD:AAB7361
Limnophyes natalensis|ATNA253|Elisabeth Stur||BOLD:AAB7361
Limnophyes natalensis|SOE111
Limnophyes natalensis|24528-G12|Kate Perez|BIN Taxonomy Match|BOLD:ACT1270
Limnophyes natalensis|CCDB24183-H09|Sofia Wiedenbrug|Morphology
Limnophyes natalensis|CCDB21605-F06|Sofia Wiedenbrug||BOLD:ACT1270
Limnophyes|Finnmark257|Elisabeth Stur
Limnophyes natalensis|ZSM-DIP-33136-E12|Caroline Chimeno|BIN Taxonomy Match (Jan 2022)|BOLD...
Limnophyes natalensis|TRD-CH17|Elisabeth Stur||BOLD:AAB7361
Limnophyes natalensis|SOE155|Elisabeth Stur||BOLD:AAB7361
Limnophyes natalensis|SOE191|Elisabeth Stur||BOLD:AAB7361
Limnophyes natalensis|SOE195|Elisabeth Stur||BOLD:AAB7361
Limnophyes natalensis|ATNA307|Elisabeth Stur||BOLD:AAB7361
Limnophyes natalensis|TRD-CH56|Elisabeth Stur||BOLD:AAB7361
Limnophyes natalensis|CH-eik67|Elisabeth Stur||BOLD:AAB7361
Limnophyes natalensis|ZSM-DIP-33141-H09|Caroline Chimeno|BIN Taxonomy Match (Jan 2022)|BOLD...
Limnophyes natalensis|ZSM-DIP-33131-H07|Caroline Chimeno|BIN Taxonomy Match (Jan 2022)|BOLD...

[illegible]
Limnophyes natalensis|CH-eik67|Elisabeth Stur||BOLD:AAB7361
Limnophyes natalensis|ZSM-DIP-33141-H09|Caroline Chimeno|BIN Taxonomy Match (Jan 2022)|BOLD...
Limnophyes natalensis|ZSM-DIP-33131-H07|Caroline Chimeno|BIN Taxonomy Match (Jan 2022)|BOLD...
Limnophyes natalensis|BIOUG05149-E01|Kate Perez|BIN Taxonomy Match|BOLD:AAB7361
Limnophyes natalensis|CCDB21605-F07|Sofia Wiedenbrug||BOLD:AAB7361
Limnophyes natalensis|SOE180|Elisabeth Stur||BOLD:AAB7361
Limnophyes natalensis|ATNA496|Elisabeth Stur||BOLD:AAB7361
Limnophyes natalensis|Finnmark612|Elisabeth Stur||BOLD:AAB7361
Limnophyes natalensis|SOE469|Elisabeth Stur||BOLD:AAB7361
Limnophyes natalensis|ATNA263|Elisabeth Stur||BOLD:AAB7361
Limnophyes natalensis|ZMUO.024679|Lauri Paasivirta||BOLD:AAB7361
Limnophyes natalensis|ZSM-DIP-33141-G07|Caroline Chimeno|BIN Taxonomy Match (Jan 2022)|BOLD...
Limnophyes natalensis|ZSM-DIP-33134-F12|Caroline Chimeno|BIN Taxonomy Match (Jan 2022)|BOLD...
Limnophyes natalensis|ZSM-DIP-33134-F10|Caroline Chimeno|BIN Taxonomy Match (Jan 2022)|BOLD...
Limnophyes natalensis|SOE198|Elisabeth Stur||BOLD:AAB7361
Limnophyes natalensis|ZSM-DIP-33172-A06|Caroline Chimeno|BIN Taxonomy Match (Jan 2022)|BOLD...
Limnophyes natalensis|SOE481|Elisabeth Stur||BOLD:AAB7361
Limnophyes natalensis|ZMUO.024678|Lauri Paasivirta||BOLD:AAB7361
Limnophyes natalensis|TRD-CH19|Elisabeth Stur||BOLD:AAB7361
Limnophyes natalensis|ZSM-DIP-33143-D10|Caroline Chimeno|BIN Taxonomy Match (Jan 2022)|BOLD...
Limnophyes natalensis|CH-eik07|Elisabeth Stur||BOLD:AAB7361
Limnophyes natalensis|SOE480|Elisabeth Stur
Limnophyes natalensis|SOE467|Elisabeth Stur
Limnophyes natalensis|SOE479|Elisabeth Stur
Limnophyes natalensis|SOE477|Elisabeth Stur
Limnophyes natalensis|SOE422|Elisabeth Stur
Limnophyes natalensis|Finnmark320|Elisabeth Stur||BOLD:AAB7361
Limnophyes|ZSM-DIP-33148-B10|Caroline Chimeno|BIN Taxonomy Match (Jan 2022)|BOLD:ACO9143
Limnophyes|ZSM-DIP-33131-D05|Caroline Chimeno|BIN Taxonomy Match (Jan 2022)|BOLD:ACO9143
Limnophyes|CCDB24228-H03|Sofia Wiedenbrug|BIN Taxonomy Match|BOLD:ACO9143
Limnophyes|CCDB24039-C01|Sofia Wiedenbrug|BIN Taxonomy Match|BOLD:ACO9143
Limnophyes sp. 2ES|SOE192|Elisabeth Stur||BOLD:AAJ2170
Limnophyes sp. 2ES|SOE351|Elisabeth Stur||BOLD:AAJ2170
Limnophyes|BIOUG16333-B06|Kate Perez|BIN Taxonomy Match|BOLD:AAJ2170
Limnophyes|BIOUG55197-C12|Stefan Schmidt|BIN Taxonomy Match (Sep 2020)|BOLD:ABU5525
Limnophyes|BIOUG42745-A08|Kate Perez|BIN Taxonomy Match below Phylum (Jun 2019)|BOLD:ABU...
Limnophyes|BIOUG42952-F08|Kate Perez|BIN Taxonomy Match below Phylum (Jun 2019)|BOLD:ABU...
Limnophyes|BIOUG55126-B03|Stefan Schmidt|BIN Taxonomy Match (Sep 2020)|BOLD:ABU5525
Limnophyes|BIOUG42760-B04|Kate Perez|BIN Taxonomy Match below Phylum (Jun 2019)|BOLD:ABU...
Limnophyes|BIOUG42763-C09|Kate Perez|BIN Taxonomy Match below Phylum (Jun 2019)|BOLD:ABU...
Limnophyes sp. 14ES|BIOUG16478-C07|Kate Perez|BIN Taxonomy Match|BOLD:ABU5525
Limnophyes sp. 14ES|BIOUG16396-B02|Kate Perez|BIN Taxonomy Match|BOLD:ABU5525
Limnophyes sp. 14ES|CH-eik17|Elisabeth Stur||BOLD:ABU5525
Limnophyes|BIOUG42739-C08|Kate Perez|BIN Taxonomy Match below Phylum (Jun 2019)|BOLD:ABU...
Limnophyes|BIOUG42667-F08|Kate Perez|BIN Taxonomy Match below Phylum (Jun 2019)|BOLD:ABU...
Limnophyes|BIOUG42701-E04|Kate Perez|BIN Taxonomy Match below Phylum (Jun 2019)|BOLD:ABU...
Limnophyes|BIOUG42918-A03|Kate Perez|BIN Taxonomy Match below Phylum (Jun 2019)|BOLD:ABU...
Limnophyes sp. 14ES|BIOUG15895-F03|Kate Perez|BIN Taxonomy Match|BOLD:ABU5525
Limnophyes sp. 14ES|CH-eik12|Elisabeth Stur||BOLD:ABU5525
Limnophyes sp. 14ES|BIOUG16333-C06|Kate Perez|BIN Taxonomy Match|BOLD:ABU5525
Limnophyes sp. 14ES|BIOUG15539-G09|Kate Perez|BIN Taxonomy Match|BOLD:ABU5525
Limnophyes|BIOUG55188-C08|Stefan Schmidt|BIN Taxonomy Match (Sep 2020)|BOLD:ABU5525
Limnophyes|BIOUG55196-C12|Stefan Schmidt|BIN Taxonomy Match (Sep 2020)|BOLD:ABU5525
Limnophyes|BIOUG55190-H02|Stefan Schmidt|BIN Taxonomy Match (Sep 2020)|BOLD:ABU5525
Limnophyes|BIOUG55414-A01|Stefan Schmidt|BIN Taxonomy Match (Sep 2020)|BOLD:ABU5525
Limnophyes|BIOUG55190-H09|Stefan Schmidt|BIN Taxonomy Match (Sep 2020)|BOLD:ABU5525
Limnophyes|BIOUG55414-A05|Stefan Schmidt|BIN Taxonomy Match (Sep 2020)|BOLD:ABU5525
Limnophyes|BIOUG55124-C04|Stefan Schmidt|BIN Taxonomy Match (Sep 2020)|BOLD:ABU5525
Limnophyes|BIOUG55423-G01|Stefan Schmidt|BIN Taxonomy Match (Sep 2020)|BOLD:ABU5525
Limnophyes|BIOUG55426-E02|Stefan Schmidt|BIN Taxonomy Match (Sep 2020)|BOLD:ABU5525
Limnophyes|BIOUG55426-C04|Stefan Schmidt|BIN Taxonomy Match (Sep 2020)|BOLD:ABU5525
Limnophyes|BIOUG55427-B11|Stefan Schmidt|BIN Taxonomy Match (Sep 2020)|BOLD:ABU5525
Limnophyes|BIOUG55427-A06|Stefan Schmidt|BIN Taxonomy Match (Sep 2020)|BOLD:ABU5525
Limnophyes|BIOUG55436-F05|Stefan Schmidt|BIN Taxonomy Match (Sep 2020)|BOLD:ABU5525
Limnophyes|BIOUG55436-B02|Stefan Schmidt|BIN Taxonomy Match (Sep 2020)|BOLD:ABU5525
Limnophyes|BIOUG55150-D06|Stefan Schmidt|BIN Taxonomy Match (Sep 2020)|BOLD:ABU5525
Limnophyes|BIOUG42707-G12|Kate Perez|BIN Taxonomy Match below Phylum (Jun 2019)|BOLD:ABU...
Limnophyes|BIOUG42735-F09|Kate Perez|BIN Taxonomy Match below Phylum (Jun 2019)|BOLD:ABU...
Limnophyes|BIOUG55165-D03|Stefan Schmidt|BIN Taxonomy Match (Sep 2020)|BOLD:ABU5525
Limnophyes|BIOUG42914-D07|Kate Perez|BIN Taxonomy Match below Phylum (Jun 2019)|BOLD:ABU...
Limnophyes|BIOUG42951-G11|Kate Perez|BIN Taxonomy Match below Phylum (Jun 2019)|BOLD:ABU...
Limnophyes|BIOUG42685-D11|Kate Perez|BIN Taxonomy Match below Phylum (Jun 2019)|BOLD:ABU...
Limnophyes|BIOUG43074-B07|Kate Perez|BIN Taxonomy Match below Phylum (Jun 2019)|BOLD:ABU...
Limnophyes|BIOUG55127-B07|Stefan Schmidt|BIN Taxonomy Match (Sep 2020)|BOLD:ABU5525
Limnophyes|BIOUG55126-F07|Stefan Schmidt|BIN Taxonomy Match (Sep 2020)|BOLD:ABU5525
Limnophyes|BIOUG55124-G06|Stefan Schmidt|BIN Taxonomy Match (Sep 2020)|BOLD:ABU5525
Limnophyes|BIOUG55120-E02|Stefan Schmidt|BIN Taxonomy Match (Sep 2020)|BOLD:ABU5525
Limnophyes|BIOUG55119-H04|Stefan Schmidt|BIN Taxonomy Match (Sep 2020)|BOLD:ABU5525
Limnophyes|BIOUG43078-A10|Kate Perez|BIN Taxonomy Match below Phylum (Jun 2019)|BOLD:ABU...
Limnophyes|BIOUG43078-A03|Kate Perez|BIN Taxonomy Match below Phylum (Jun 2019)|BOLD:ABU...
Limnophyes|BIOUG55421-H06|Stefan Schmidt|BIN Taxonomy Match (Sep 2020)|BOLD:ABU5525
Limnophyes|BIOUG54164-G06|Stefan Schmidt|BIN Taxonomy Match (Sep 2020)|BOLD:ABU5525
Limnophyes|BIOUG42674-D04|Kate Perez|BIN Taxonomy Match below Phylum (Jun 2019)|BOLD:ABU...
Limnophyes|BIOUG55200-E05|Stefan Schmidt|BIN Taxonomy Match (Sep 2020)|BOLD:ABU5525
Limnophyes|BIOUG42966-D08|Kate Perez|BIN Taxonomy Match below Phylum (Jun 2019)|BOLD:ABU...
Limnophyes|BIOUG55200-B05|Stefan Schmidt|BIN Taxonomy Match (Sep 2020)|BOLD:ABU5525
Limnophyes|BIOUG55199-G12|Stefan Schmidt|BIN Taxonomy Match (Sep 2020)|BOLD:ABU5525
Limnophyes|BIOUG55198-B09|Stefan Schmidt|BIN Taxonomy Match (Sep 2020)|BOLD:ABU5525
Limnophyes|BIOUG42668-D02|Kate Perez|BIN Taxonomy Match below Phylum (Jun 2019)|BOLD:ABU...
Limnophyes|BIOUG55197-H02|Stefan Schmidt|BIN Taxonomy Match (Sep 2020)|BOLD:ABU5525
Limnophyes|BIOUG55197-D10|Stefan Schmidt|BIN Taxonomy Match (Sep 2020)|BOLD:ABU5525
Limnophyes|BIOUG55197-B10|Stefan Schmidt|BIN Taxonomy Match (Sep 2020)|BOLD:ABU5525
Limnophyes|BIOUG55197-A08|Stefan Schmidt|BIN Taxonomy Match (Sep 2020)|BOLD:ABU5525
Limnophyes|BIOUG55196-E06|Stefan Schmidt|BIN Taxonomy Match (Sep 2020)|BOLD:ABU5525
Limnophyes|BIOUG55195-G02|Stefan Schmidt|BIN Taxonomy Match (Sep 2020)|BOLD:ABU5525
Limnophyes|BIOUG42673-D05|Kate Perez|BIN Taxonomy Match below Phylum (Jun 2019)|BOLD:ABU...
Limnophyes|BIOUG55200-F08|Stefan Schmidt|BIN Taxonomy Match (Sep 2020)|BOLD:ABU5525
Limnophyes|BIOUG42959-D09|Kate Perez|BIN Taxonomy Match below Phylum (Jun 2019)|BOLD:ABU...
Limnophyes|BIOUG55192-G07|Stefan Schmidt|BIN Taxonomy Match (Sep 2020)|BOLD:ABU5525
Limnophyes|BIOUG55413-C09|Stefan Schmidt|BIN Taxonomy Match (Sep 2020)|BOLD:ABU5525
Limnophyes|BIOUG42659-B07|Kate Perez|BIN Taxonomy Match below Phylum (Jun 2019)|BOLD:ABU...
Limnophyes|BIOUG42945-C06|Kate Perez|BIN Taxonomy Match below Phylum (Jun 2019)|BOLD:ABU...
[illegible]

Limnophyes|BIOUG55413-C09|Stefan Schmidt|BIN Taxonomy Match (Sep 2020)|BOLD:ABU5525
Limnophyes|BIOUG42659-B07|Kate Perez|BIN Taxonomy Match below Phylum (Jun 2019)|BOLD:ABU...
Limnophyes|BIOUG42945-C06|Kate Perez|BIN Taxonomy Match below Phylum (Jun 2019)|BOLD:ABU...
Limnophyes|BIOUG55193-B01|Stefan Schmidt|BIN Taxonomy Match (Sep 2020)|BOLD:ABU5525
Limnophyes|BIOUG42941-H11|Kate Perez|BIN Taxonomy Match below Phylum (Jun 2019)|BOLD:ABU...
Limnophyes|BIOUG42941-C10|Kate Perez|BIN Taxonomy Match below Phylum (Jun 2019)|BOLD:ABU...
Limnophyes|BIOUG42941-B07|Kate Perez|BIN Taxonomy Match below Phylum (Jun 2019)|BOLD:ABU...
Limnophyes|BIOUG42940-G09|Kate Perez|BIN Taxonomy Match below Phylum (Jun 2019)|BOLD:ABU...
Limnophyes|BIOUG55414-E03|Stefan Schmidt|BIN Taxonomy Match (Sep 2020)|BOLD:ABU5525
Limnophyes|BIOUG55414-A11|Stefan Schmidt|BIN Taxonomy Match (Sep 2020)|BOLD:ABU5525
Limnophyes|BIOUG42700-C01|Kate Perez|BIN Taxonomy Match below Phylum (Jun 2019)|BOLD:ABU...
Limnophyes|BIOUG55105-A05|Stefan Schmidt|BIN Taxonomy Match (Sep 2020)|BOLD:ABU5525
Limnophyes|BIOUG55192-E09|Stefan Schmidt|BIN Taxonomy Match (Sep 2020)|BOLD:ABU5525
Limnophyes|BIOUG42967-B04|Kate Perez|BIN Taxonomy Match below Phylum (Jun 2019)|BOLD:ABU...
Limnophyes|BIOUG42703-H10|Kate Perez|BIN Taxonomy Match below Phylum (Jun 2019)|BOLD:ABU...
Limnophyes|BIOUG55415-B03|Stefan Schmidt|BIN Taxonomy Match (Sep 2020)|BOLD:ABU5525
Limnophyes|BIOUG42963-E07|Kate Perez|BIN Taxonomy Match below Phylum (Jun 2019)|BOLD:ABU...
Limnophyes|BIOUG42962-F12|Kate Perez|BIN Taxonomy Match below Phylum (Jun 2019)|BOLD:ABU...
Limnophyes|BIOUG42966-A01|Kate Perez|BIN Taxonomy Match below Phylum (Jun 2019)|BOLD:ABU...
Limnophyes|BIOUG42964-G05|Kate Perez|BIN Taxonomy Match below Phylum (Jun 2019)|BOLD:ABU...
Limnophyes|BIOUG42943-E07|Kate Perez|BIN Taxonomy Match below Phylum (Jun 2019)|BOLD:ABU...
Limnophyes|ZSM-DIP-33129-D09|Caroline Chimeno|BIN Taxonomy Match (Jan 2022)|BOLD:ABU5525
Limnophyes|BIOUG55428-B01|Stefan Schmidt|BIN Taxonomy Match (Sep 2020)|BOLD:ABU5525
Limnophyes|BIOUG55427-C01|Stefan Schmidt|BIN Taxonomy Match (Sep 2020)|BOLD:ABU5525
Limnophyes|BIOUG55424-H05|Stefan Schmidt|BIN Taxonomy Match (Sep 2020)|BOLD:ABU5525
Limnophyes|BIOUG55424-D11|Stefan Schmidt|BIN Taxonomy Match (Sep 2020)|BOLD:ABU5525
Limnophyes|BIOUG55424-B09|Stefan Schmidt|BIN Taxonomy Match (Sep 2020)|BOLD:ABU5525
Limnophyes|BIOUG55424-A02|Stefan Schmidt|BIN Taxonomy Match (Sep 2020)|BOLD:ABU5525
Limnophyes|BIOUG55423-D03|Stefan Schmidt|BIN Taxonomy Match (Sep 2020)|BOLD:ABU5525
Limnophyes|BIOUG55422-F05|Stefan Schmidt|BIN Taxonomy Match (Sep 2020)|BOLD:ABU5525
Limnophyes|BIOUG55425-D12|Stefan Schmidt|BIN Taxonomy Match (Sep 2020)|BOLD:ABU5525
Limnophyes|BIOUG55425-B09|Stefan Schmidt|BIN Taxonomy Match (Sep 2020)|BOLD:ABU5525
Limnophyes|BIOUG55195-F11|Stefan Schmidt|BIN Taxonomy Match (Sep 2020)|BOLD:ABU5525
Limnophyes|BIOUG55195-E10|Stefan Schmidt|BIN Taxonomy Match (Sep 2020)|BOLD:ABU5525
Limnophyes|BIOUG55194-A08|Stefan Schmidt|BIN Taxonomy Match (Sep 2020)|BOLD:ABU5525
Limnophyes|BIOUG55193-F06|Stefan Schmidt|BIN Taxonomy Match (Sep 2020)|BOLD:ABU5525
Limnophyes|BIOUG42663-E07|Kate Perez|BIN Taxonomy Match below Phylum (Jun 2019)|BOLD:ABU...
Limnophyes|BIOUG55192-F01|Stefan Schmidt|BIN Taxonomy Match (Sep 2020)|BOLD:ABU5525
Limnophyes|BIOUG55187-D06|Stefan Schmidt|BIN Taxonomy Match (Sep 2020)|BOLD:ABU5525
Limnophyes|BIOUG42942-B07|Kate Perez|BIN Taxonomy Match below Phylum (Jun 2019)|BOLD:ABU...
Limnophyes|BIOUG42955-A02|Kate Perez|BIN Taxonomy Match below Phylum (Jun 2019)|BOLD:ABU...
Limnophyes|BIOUG42954-H07|Kate Perez|BIN Taxonomy Match below Phylum (Jun 2019)|BOLD:ABU...
Limnophyes|BIOUG42954-A06|Kate Perez|BIN Taxonomy Match below Phylum (Jun 2019)|BOLD:ABU...
Limnophyes|BIOUG42952-G03|Kate Perez|BIN Taxonomy Match below Phylum (Jun 2019)|BOLD:ABU...
Limnophyes|BIOUG42950-F09|Kate Perez|BIN Taxonomy Match below Phylum (Jun 2019)|BOLD:ABU...
Limnophyes|BIOUG42949-H08|Kate Perez|BIN Taxonomy Match below Phylum (Jun 2019)|BOLD:ABU...
Limnophyes|BIOUG42944-E11|Kate Perez|BIN Taxonomy Match below Phylum (Jun 2019)|BOLD:ABU...
Limnophyes|BIOUG55188-D05|Stefan Schmidt|BIN Taxonomy Match (Sep 2020)|BOLD:ABU5525
Limnophyes|BIOUG42740-C02|Kate Perez|BIN Taxonomy Match below Phylum (Jun 2019)|BOLD:ABU...
Limnophyes|BIOUG55152-C05|Stefan Schmidt|BIN Taxonomy Match (Sep 2020)|BOLD:ABU5525
Limnophyes|BIOUG42949-D10|Kate Perez|BIN Taxonomy Match below Phylum (Jun 2019)|BOLD:ABU...
Limnophyes|BIOUG42949-D07|Kate Perez|BIN Taxonomy Match below Phylum (Jun 2019)|BOLD:ABU...
Limnophyes|BIOUG42949-B12|Kate Perez|BIN Taxonomy Match below Phylum (Jun 2019)|BOLD:ABU...
Limnophyes|BIOUG42949-A07|Kate Perez|BIN Taxonomy Match below Phylum (Jun 2019)|BOLD:ABU...
Limnophyes|BIOUG42948-H09|Kate Perez|BIN Taxonomy Match below Phylum (Jun 2019)|BOLD:ABU...
Limnophyes|BIOUG42948-D03|Kate Perez|BIN Taxonomy Match below Phylum (Jun 2019)|BOLD:ABU...
Limnophyes|BIOUG42940-B05|Kate Perez|BIN Taxonomy Match below Phylum (Jun 2019)|BOLD:ABU...
Limnophyes|BIOUG42662-H10|Kate Perez|BIN Taxonomy Match below Phylum (Jun 2019)|BOLD:ABU...
Limnophyes|BIOUG42969-E12|Kate Perez|BIN Taxonomy Match below Phylum (Jun 2019)|BOLD:ABU...
Limnophyes|BIOUG42968-E05|Kate Perez|BIN Taxonomy Match below Phylum (Jun 2019)|BOLD:ABU...
Limnophyes|BIOUG42930-F11|Kate Perez|BIN Taxonomy Match below Phylum (Jun 2019)|BOLD:ABU...
Limnophyes|BIOUG42943-B09|Kate Perez|BIN Taxonomy Match below Phylum (Jun 2019)|BOLD:ABU...
Limnophyes|BIOUG42942-H11|Kate Perez|BIN Taxonomy Match below Phylum (Jun 2019)|BOLD:ABU...
Limnophyes|BIOUG42759-E03|Kate Perez|BIN Taxonomy Match below Phylum (Jun 2019)|BOLD:ABU...
Limnophyes|BIOUG42939-F03|Kate Perez|BIN Taxonomy Match below Phylum (Jun 2019)|BOLD:ABU...
Limnophyes|BIOUG42938-H06|Kate Perez|BIN Taxonomy Match below Phylum (Jun 2019)|BOLD:ABU...
Limnophyes|BIOUG42938-E10|Kate Perez|BIN Taxonomy Match below Phylum (Jun 2019)|BOLD:ABU...
Limnophyes|BIOUG42938-E08|Kate Perez|BIN Taxonomy Match below Phylum (Jun 2019)|BOLD:ABU...
Limnophyes|BIOUG42942-F02|Kate Perez|BIN Taxonomy Match below Phylum (Jun 2019)|BOLD:ABU...
Limnophyes|BIOUG42942-C04|Kate Perez|BIN Taxonomy Match below Phylum (Jun 2019)|BOLD:ABU...
Limnophyes|BIOUG42938-D01|Kate Perez|BIN Taxonomy Match below Phylum (Jun 2019)|BOLD:ABU...
Limnophyes|BIOUG42748-G07|Kate Perez|BIN Taxonomy Match below Phylum (Jun 2019)|BOLD:ABU...
Limnophyes|BIOUG42749-E02|Kate Perez|BIN Taxonomy Match below Phylum (Jun 2019)|BOLD:ABU...
Limnophyes|BIOUG42752-G12|Kate Perez|BIN Taxonomy Match below Phylum (Jun 2019)|BOLD:ABU...
Limnophyes|BIOUG42753-E07|Kate Perez|BIN Taxonomy Match below Phylum (Jun 2019)|BOLD:ABU...
Limnophyes|BIOUG55191-A07|Stefan Schmidt|BIN Taxonomy Match (Sep 2020)|BOLD:ABU5525
Limnophyes|BIOUG55191-E06|Stefan Schmidt|BIN Taxonomy Match (Sep 2020)|BOLD:ABU5525
Limnophyes|BIOUG55191-F10|Stefan Schmidt|BIN Taxonomy Match (Sep 2020)|BOLD:ABU5525
Limnophyes|BIOUG42718-H07|Kate Perez|BIN Taxonomy Match below Phylum (Jun 2019)|BOLD:ABU...
Limnophyes|BIOUG42744-A08|Kate Perez|BIN Taxonomy Match below Phylum (Jun 2019)|BOLD:ABU...
Limnophyes|BIOUG42744-E07|Kate Perez|BIN Taxonomy Match below Phylum (Jun 2019)|BOLD:ABU...
Limnophyes|BIOUG42672-F05|Kate Perez|BIN Taxonomy Match below Phylum (Jun 2019)|BOLD:ABU...
Limnophyes|BIOUG42739-F07|Kate Perez|BIN Taxonomy Match below Phylum (Jun 2019)|BOLD:ABU...
Limnophyes|BIOUG42751-G04|Kate Perez|BIN Taxonomy Match below Phylum (Jun 2019)|BOLD:ABU...
Limnophyes|BIOUG42946-F10|Kate Perez|BIN Taxonomy Match below Phylum (Jun 2019)|BOLD:ABU...
Limnophyes sp. 14ES|BIOUG16329-E10|Kate Perez|BIN Taxonomy Match|BOLD:ABU5525
Limnophyes|BIOUG42939-G02|Kate Perez|BIN Taxonomy Match below Phylum (Jun 2019)|BOLD:ABU...
Limnophyes|BIOUG42968-D06|Kate Perez|BIN Taxonomy Match below Phylum (Jun 2019)|BOLD:ABU...
Limnophyes|BIOUG42748-H06|Kate Perez|BIN Taxonomy Match below Phylum (Jun 2019)|BOLD:ABU...
Limnophyes|BIOUG42914-H05|Kate Perez|BIN Taxonomy Match below Phylum (Jun 2019)|BOLD:ABU...
Limnophyes|BIOUG55192-E10|Stefan Schmidt|BIN Taxonomy Match (Sep 2020)|BOLD:ABU5525
Limnophyes|BIOUG42939-C04|Kate Perez|BIN Taxonomy Match below Phylum (Jun 2019)|BOLD:ABU...
Limnophyes|BIOUG42942-F09|Kate Perez|BIN Taxonomy Match below Phylum (Jun 2019)|BOLD:ABU...
Limnophyes|BIOUG42944-B07|Kate Perez|BIN Taxonomy Match below Phylum (Jun 2019)|BOLD:ABU...
Limnophyes|BIOUG42715-A03|Kate Perez|BIN Taxonomy Match below Phylum (Jun 2019)|BOLD:ABU...
Limnophyes|BIOUG55145-G04|Stefan Schmidt|BIN Taxonomy Match (Sep 2020)|BOLD:ABU5525
Limnophyes|BIOUG42740-C07|Kate Perez|BIN Taxonomy Match below Phylum (Jun 2019)|BOLD:ABU...
Limnophyes|BIOUG42943-A04|Kate Perez|BIN Taxonomy Match below Phylum (Jun 2019)|BOLD:ABU...
Limnophyes|BIOUG42746-G04|Kate Perez|BIN Taxonomy Match below Phylum (Jun 2019)|BOLD:ABU...
Limnophyes|BIOUG42916-D05|Kate Perez|BIN Taxonomy Match below Phylum (Jun 2019)|BOLD:ABU...
Limnophyes|BIOUG42704-C10|Kate Perez|BIN Taxonomy Match below Phylum (Jun 2019)|BOLD:ABU...
Limnophyes|BIOUG42967-F04|Kate Perez|BIN Taxonomy Match below Phylum (Jun 2019)|BOLD:ABU...
Limnophyes|BIOUG42939-C06|Kate Perez|BIN Taxonomy Match below Phylum (Jun 2019)|BOLD:ABU...

Limnophyes|BIOUG42704-C10|Kate Perez|BIN Taxonomy Match below Phylum (Jun 2019)|BOLD:ABU...
Limnophyes|BIOUG42967-F04|Kate Perez|BIN Taxonomy Match below Phylum (Jun 2019)|BOLD:ABU...
Limnophyes|BIOUG42939-C06|Kate Perez|BIN Taxonomy Match below Phylum (Jun 2019)|BOLD:ABU...
Limnophyes|BIOUG42671-B02|Kate Perez|BIN Taxonomy Match below Phylum (Jun 2019)|BOLD:ABU...
Limnophyes|BIOUG42943-F10|Kate Perez|BIN Taxonomy Match below Phylum (Jun 2019)|BOLD:ABU...
Limnophyes|BIOUG42970-D12|Kate Perez|BIN Taxonomy Match below Phylum (Jun 2019)|BOLD:ABU...
Limnophyes|BIOUG42948-F09|Kate Perez|BIN Taxonomy Match below Phylum (Jun 2019)|BOLD:ABU...
Limnophyes|BIOUG42941-E12|Kate Perez|BIN Taxonomy Match below Phylum (Jun 2019)|BOLD:ABU...
Limnophyes|BIOUG42706-B07|Kate Perez|BIN Taxonomy Match below Phylum (Jun 2019)|BOLD:ABU...
Limnophyes|BIOUG55416-E05|Stefan Schmidt|BIN Taxonomy Match (Sep 2020)|BOLD:ABU5525
Limnophyes sp. 14ES|BIOUG15948-F10|Kate Perez|BIN Taxonomy Match|BOLD:ABU5525
Limnophyes|BIOUG55416-E09|Stefan Schmidt|BIN Taxonomy Match (Sep 2020)|BOLD:ABU5525
Limnophyes|BIOUG55436-F09|Stefan Schmidt|BIN Taxonomy Match (Sep 2020)|BOLD:ABU5525
Limnophyes|BIOUG42748-H10|Kate Perez|BIN Taxonomy Match below Phylum (Jun 2019)|BOLD:ABU...
Limnophyes|BIOUG42278-H09|Kate Perez|BIN Taxonomy Match below Phylum (Jun 2019)|BOLD:ABU...
Limnophyes|BIOUG42676-G07|Kate Perez|BIN Taxonomy Match below Phylum (Jun 2019)|BOLD:ABU...
Limnophyes|BIOUG42941-H10|Kate Perez|BIN Taxonomy Match below Phylum (Jun 2019)|BOLD:ABU...
Limnophyes|BIOUG42955-C06|Kate Perez|BIN Taxonomy Match below Phylum (Jun 2019)|BOLD:ABU...
Limnophyes|BIOUG42927-G05|Kate Perez|BIN Taxonomy Match below Phylum (Jun 2019)|BOLD:ABU...
Limnophyes|BIOUG42943-C06|Kate Perez|BIN Taxonomy Match below Phylum (Jun 2019)|BOLD:ABU...
Limnophyes sp. 14ES|BIOUG17064-G05|Kate Perez|BIN Taxonomy Match|BOLD:ABU5525
Limnophyes sp. 14ES|BIOUG15797-C03|Kate Perez|BIN Taxonomy Match|BOLD:ABU5525
Limnophyes|BIOUG42946-G12|Kate Perez|BIN Taxonomy Match below Phylum (Jun 2019)|BOLD:ABU...
Limnophyes|BIOUG42670-H11|Kate Perez|BIN Taxonomy Match below Phylum (Jun 2019)|BOLD:ABU...
Limnophyes sp. 14ES|BIOUG16776-E10|Kate Perez|BIN Taxonomy Match|BOLD:ABU5525
Limnophyes sp. 14ES|BIOUG16354-E05|Kate Perez|BIN Taxonomy Match|BOLD:ABU5525
Limnophyes sp. 14ES|BIOUG16552-C10|Kate Perez|BIN Taxonomy Match|BOLD:ABU5525
Limnophyes sp. 14ES|BIOUG15948-E03|Kate Perez|BIN Taxonomy Match|BOLD:ABU5525
Limnophyes NHRS sp. A|NHRS-BYWS000000339|Yngve Brodin|morphology|BOLD:ADJ7032
Limnophyes|BIOUG16354-E12|Kate Perez|BOLD ID Engine Manual|BOLD:ACR0830
Limnophyes|BIOUG16529-A12|Kate Perez|BOLD ID Engine Manual|BOLD:ACR0830
Limnophyes|ZSM-DIP-33172-A08|Caroline Chimeno|BIN Taxonomy Match (Jan 2022)|BOLD:AAB1806
Limnophyes|ZSM-DIP-33170-G07|Caroline Chimeno|BIN Taxonomy Match (Jan 2022)|BOLD:AAB1806
Limnophyes|ZSM-DIP-33170-G06|Caroline Chimeno|BIN Taxonomy Match (Jan 2022)|BOLD:AAB1806
Limnophyes|ZSM-DIP-33148-C10|Caroline Chimeno|BIN Taxonomy Match (Jan 2022)|BOLD:AAB1806
Limnophyes|ZSM-DIP-33172-A10|Caroline Chimeno|BIN Taxonomy Match (Jan 2022)|BOLD:AAB1806
Limnophyes|ZSM-DIP-33130-E08|Caroline Chimeno|BIN Taxonomy Match (Jan 2022)|BOLD:AAB1806
Limnophyes NHRS sp. B|NHRS-BYWS000000451|Yngve Brodin|morphology|BOLD:AAB1806
Limnophyes sp. 3ES|SOE234|Elisabeth Stur||BOLD:AAB1806
Limnophyes sp. 3ES|SOE350|Elisabeth Stur||BOLD:AAB1806
Limnophyes sp. 3ES|SOE448|Elisabeth Stur||BOLD:AAB1806
Limnophyes sp. 3ES|SOE236|Elisabeth Stur||BOLD:AAB1806
Limnophyes sp. 3ES|CH-eik100|Elisabeth Stur||BOLD:AAB1806
Limnophyes sp. 3ES|SOE470|Elisabeth Stur||BOLD:AAB1806
Limnophyes sp. 3ES|SOE454|Elisabeth Stur||BOLD:AAB1806
Limnophyes sp. 3ES|SOE58|Elisabeth Stur||BOLD:AAB1806
Limnophyes sp. 3ES|SOE142|Elisabeth Stur||BOLD:AAB1806
Limnophyes sp. 3ES|SOE311|Elisabeth Stur||BOLD:AAB1806
Limnophyes sp. 3ES|SOE326|Elisabeth Stur||BOLD:AAB1806
Limnophyes sp. 3ES|SOE451|Elisabeth Stur||BOLD:AAB1806
Limnophyes sp. 3ES|SOE482|Elisabeth Stur||BOLD:AAB1806
Limnophyes sp. 3ES|SOE447|Elisabeth Stur||BOLD:AAB1806
Limnophyes sp. 8ES|CCDB22605-D09|Sofia Wiedenbrug||BOLD:AAW1297
Limnophyes sp. 8ES|ZSM-DIP-33138-H07|Caroline Chimeno|BIN Taxonomy Match (Jan 2022)|BOLD ...
Limnophyes sp. 8ES|ZSM-DIP-33137-E02|Caroline Chimeno|BIN Taxonomy Match (Jan 2022)|BOLD ...
Limnophyes|ZSM-DIP-33135-F01|Caroline Chimeno|BIN Taxonomy Match (Jan 2022)|BOLD:AAW1297
Limnophyes|BIOUG42677-F01|Kate Perez|BIN Taxonomy Match below Phylum (Jun 2019)|BOLD:AAW...
Limnophyes|BIOUG42750-C04|Kate Perez|BIN Taxonomy Match below Phylum (Jun 2019)|BOLD:AAW...
Limnophyes sp. 8ES|ZSM-DIP-33136-D04|Caroline Chimeno|BIN Taxonomy Match (Jan 2022)|BOLD ...
Limnophyes|ZSM-DIP-33136-C12|Caroline Chimeno|BIN Taxonomy Match (Jan 2022)|BOLD:AAW1297
Limnophyes sp. 8ES|ZSM-DIP-33136-C06|Caroline Chimeno|BIN Taxonomy Match (Jan 2022)|BOLD ...
Limnophyes sp. 8ES|ZSM-DIP-33131-H05|Caroline Chimeno|BIN Taxonomy Match (Jan 2022)|BOLD ...
Limnophyes sp. 8ES|ZSM-DIP-33131-H06|Caroline Chimeno|BIN Taxonomy Match (Jan 2022)|BOLD ...
Limnophyes|BIOUG55428-F07|Stefan Schmidt|BIN Taxonomy Match (Sep 2020)|BOLD:AAW1297
Limnophyes|BIOUG42667-B09|Kate Perez|BIN Taxonomy Match below Phylum (Jun 2019)|BOLD:AAW...
Limnophyes|BIOUG42927-B06|Kate Perez|BIN Taxonomy Match below Phylum (Jun 2019)|BOLD:AAW...
Limnophyes sp. 8ES|BIOUG05107-A12|Kate Perez|BIN Taxonomy Match|BOLD:AAW1297
Limnophyes|ZSM-DIP-33137-E09|Caroline Chimeno|BIN Taxonomy Match (Jan 2022)|BOLD:AAW1297
Limnophyes sp. 8ES|ZSM-DIP-33138-H06|Caroline Chimeno|BIN Taxonomy Match (Jan 2022)|BOLD ...
Limnophyes|ZSM-DIP-33137-E01|Caroline Chimeno|BIN Taxonomy Match (Jan 2022)|BOLD:AAW1297
Limnophyes|ZSM-DIP-33136-C11|Caroline Chimeno|BIN Taxonomy Match (Jan 2022)|BOLD:AAW1297
Limnophyes|ZSM-DIP-33136-C07|Caroline Chimeno|BIN Taxonomy Match (Jan 2022)|BOLD:AAW1297
Limnophyes|BIOUG42709-B10|Kate Perez|BIN Taxonomy Match below Phylum (Jun 2019)|BOLD:AAW...
Limnophyes sp. 8ES|ZSM-DIP-33137-D08|Caroline Chimeno|BIN Taxonomy Match (Jan 2022)|BOLD ...
Limnophyes sp. 8ES|ZSM-DIP-33141-H06|Caroline Chimeno|BIN Taxonomy Match (Jan 2022)|BOLD ...
Limnophyes|ZSM-DIP-33136-C10|Caroline Chimeno|BIN Taxonomy Match (Jan 2022)|BOLD:AAW1297
Limnophyes sp. 8ES|ZSM-DIP-33136-D03|Caroline Chimeno|BIN Taxonomy Match (Jan 2022)|BOLD ...
Limnophyes|ZSM-DIP-33136-D05|Caroline Chimeno|BIN Taxonomy Match (Jan 2022)|BOLD:AAW1297
Limnophyes sp. 8ES|ZSM-DIP-33136-F02|Caroline Chimeno|BIN Taxonomy Match (Jan 2022)|BOLD ...
Limnophyes sp. 8ES|ZSM-DIP-33136-F12|Caroline Chimeno|BIN Taxonomy Match (Jan 2022)|BOLD ...
Limnophyes|ZSM-DIP-33137-E05|Caroline Chimeno|BIN Taxonomy Match (Jan 2022)|BOLD:AAW1297
Limnophyes|BIOUG42946-C11|Kate Perez|BIN Taxonomy Match below Phylum (Jun 2019)|BOLD:AAW...
Limnophyes|BIOUG42679-C11|Kate Perez|BIN Taxonomy Match below Phylum (Jun 2019)|BOLD:AAW...
Limnophyes|ZSM-DIP-33135-F11|Caroline Chimeno|BIN Taxonomy Match (Jan 2022)|BOLD:AAW1297
Limnophyes sp. 8ES|ZSM-DIP-33130-C05|Caroline Chimeno|BIN Taxonomy Match (Jan 2022)|BOLD ...
Limnophyes sp. 8ES|BIOUG05103-F10|Kate Perez|BIN Taxonomy Match|BOLD:AAW1297
Limnophyes sp. 8ES|BIOUG06095-F10|Kate Perez|BIN Taxonomy Match|BOLD:AAW1297
Limnophyes sp. 8ES|CH-OSF55|Elisabeth Stur||BOLD:AAW1297
Limnophyes sp. 8ES|BIOUG05947-F05|Kate Perez|BIN Taxonomy Match|BOLD:AAW1297
Limnophyes sp. 8ES|BIOUG07407-D12|Kate Perez|BIN Taxonomy Match|BOLD:AAW1297
Limnophyes sp. 8ES|BIOUG07405-F10|Kate Perez|BIN Taxonomy Match|BOLD:AAW1297
Limnophyes sp. 8ES|ZSM-DIP-33131-H10|Caroline Chimeno|BIN Taxonomy Match (Jan 2022)|BOLD ...
Limnophyes sp. 8ES|BIOUG07755-C03|Kate Perez|BIN Taxonomy Match|BOLD:AAW1297
Limnophyes sp. 8ES|BIOUG06209-G09|Kate Perez|BIN Taxonomy Match|BOLD:AAW1297
Chironomidae|BIOUG04269-A03|Kate Perez|BIN Taxonomy Match|BOLD:ACC1276
Limnophyes sp. 2SW|BIOUG07566-A09|Sofia Wiedenbrug||BOLD:ACG4398
Limnophyes sp. 2SW|BIOUG07808-B01|Sofia Wiedenbrug|BIN Taxonomy Match|BOLD:ACG4398
Limnophyes sp. 2SW|BIOUG07809-F10|Sofia Wiedenbrug|BIN Taxonomy Match|BOLD:ACG4398
Limnophyes sp. 2SW|BIOUG07808-F11|Sofia Wiedenbrug|BIN Taxonomy Match|BOLD:ACG4398
Limnophyes sp. 2SW|BIOUG07566-E10|Sofia Wiedenbrug||BOLD:ACG4398
Limnophyes sp. 2SW|BIOUG07568-G02|Sofia Wiedenbrug||BOLD:ACG4398
Limnophyes sp. 2SW|BIOUG07568-C11|Sofia Wiedenbrug||BOLD:ACG4398
Limnophyes sp. 2SW|BIOUG07407-A11|Sofia Wiedenbrug||BOLD:ACG4398

[illegible]
Limnophyes sp. 2SW|BIOUG07568-G02|Sofia Wiedenbrug||BOLD:ACG4398
Limnophyes sp. 2SW|BIOUG07568-C11|Sofia Wiedenbrug||BOLD:ACG4398
Limnophyes sp. 2SW|BIOUG07407-A11|Sofia Wiedenbrug||BOLD:ACG4398
Limnophyes sp. 2SW|BIOUG07810-D09|Sofia Wiedenbrug||BOLD:ACG4398
Limnophyes sp. 2SW|BIOUG07929-G10|Sofia Wiedenbrug||BOLD:ACG4398
Limnophyes sp. 2SW|BIOUG07410-H04|Sofia Wiedenbrug||BOLD:ACG4398
Limnophyes sp. 2SW|BIOUG07930-C10|Sofia Wiedenbrug||BOLD:ACG4398
Limnophyes sp. 2SW|BIOUG06280-G08|Sofia Wiedenbrug||BOLD:ACG4398
Limnophyes sp. 2SW|BIOUG08135-G04|Sofia Wiedenbrug||BOLD:ACG4398
Limnophyes sp. 2SW|BIOUG08174-B11|Sofia Wiedenbrug||BOLD:ACG4398
Limnophyes sp. 2SW|BIOUG07568-E09|Sofia Wiedenbrug||BOLD:ACG4398
Limnophyes sp. 2SW|BIOUG07810-D05|Sofia Wiedenbrug||BOLD:ACG4398
Limnophyes sp. 2SW|BIOUG07418-D12|Sofia Wiedenbrug||BOLD:ACG4398
Limnophyes sp. 2SW|BIOUG06065-B06|Sofia Wiedenbrug||BOLD:ACG4398
Limnophyes sp. 2SW|BIOUG07410-F11|Sofia Wiedenbrug||BOLD:ACG4398
Limnophyes sp. 2SW|BIOUG07929-H05|Sofia Wiedenbrug||BOLD:ACG4398
Limnophyes sp. 2SW|BIOUG07503-D05|Sofia Wiedenbrug|BIN Taxonomy Match|BOLD:ACG4398
Limnophyes sp. 2SW|BIOUG07930-D03|Sofia Wiedenbrug||BOLD:ACG4398
Limnophyes sp. 2SW|BIOUG07405-C07|Sofia Wiedenbrug||BOLD:ACG4398
Limnophyes sp. 2SW|BIOUG06052-E12|Sofia Wiedenbrug||BOLD:ACG4398
Limnophyes sp. 2SW|BIOUG05946-E03|Sofia Wiedenbrug||BOLD:ACG4398
Limnophyes sp. 2SW|BIOUG07805-C03|Sofia Wiedenbrug|BOLD ID Engine Manual|BOLD:ACG4398
Limnophyes sp. 2SW|BIOUG06054-H01|Sofia Wiedenbrug||BOLD:ACG4398
Limnophyes sp. 2SW|BIOUG06094-C01|Sofia Wiedenbrug||BOLD:ACG4398
Limnophyes sp. 2SW|BIOUG08180-F04|Sofia Wiedenbrug||BOLD:ACG4398
Limnophyes sp. 2SW|BIOUG07563-G11|Sofia Wiedenbrug||BOLD:ACG4398
Limnophyes sp. 2SW|BIOUG06090-C04|Sofia Wiedenbrug||BOLD:ACG4398
Limnophyes sp. 2SW|BIOUG07407-A12|Sofia Wiedenbrug||BOLD:ACG4398
Limnophyes sp. 2SW|BIOUG06274-C03|Sofia Wiedenbrug|BOLD ID Engine Manual|BOLD:ACG4398
Limnophyes sp. 2SW|BIOUG07760-E10|Sofia Wiedenbrug||BOLD:ACG4398
Limnophyes sp. 2SW|BIOUG07754-A06|Sofia Wiedenbrug||BOLD:ACG4398
Limnophyes sp. 2SW|BIOUG07755-E06|Sofia Wiedenbrug||BOLD:ACG4398
Limnophyes sp. 2SW|BIOUG07757-C06|Sofia Wiedenbrug||BOLD:ACG4398
Limnophyes sp. 2SW|BIOUG07757-E02|Sofia Wiedenbrug||BOLD:ACG4398
Limnophyes sp. 2SW|BIOUG07563-H04|Sofia Wiedenbrug||BOLD:ACG4398
Limnophyes sp. 2SW|BIOUG07758-F07|Sofia Wiedenbrug||BOLD:ACG4398
Limnophyes sp. 2SW|BIOUG07759-G10|Sofia Wiedenbrug||BOLD:ACG4398
Limnophyes sp. 2SW|BIOUG07759-A05|Sofia Wiedenbrug||BOLD:ACG4398
Limnophyes sp. 2SW|BIOUG07944-C08|Sofia Wiedenbrug||BOLD:ACG4398
Limnophyes sp. 2SW|BIOUG07759-G06|Sofia Wiedenbrug||BOLD:ACG4398
Limnophyes sp. 2SW|BIOUG07755-A11|Sofia Wiedenbrug||BOLD:ACG4398
Limnophyes sp. 2SW|BIOUG07757-B06|Sofia Wiedenbrug||BOLD:ACG4398
Limnophyes sp. 2SW|BIOUG07810-B06|Sofia Wiedenbrug||BOLD:ACG4398
Limnophyes sp. 2SW|BIOUG07810-G12|Sofia Wiedenbrug||BOLD:ACG4398
Limnophyes sp. 2SW|BIOUG08180-C07|Sofia Wiedenbrug||BOLD:ACG4398
Limnophyes sp. 2SW|BIOUG07754-B05|Sofia Wiedenbrug||BOLD:ACG4398
Limnophyes sp. 2SW|BIOUG08140-D09|Sofia Wiedenbrug||BOLD:ACG4398
Limnophyes sp. 2SW|BIOUG06517-G06|Sofia Wiedenbrug||BOLD:ACG4398
Limnophyes sp. 2SW|BIOUG08137-G03|Sofia Wiedenbrug||BOLD:ACG4398
Limnophyes sp. 2SW|BIOUG06087-A06|Sofia Wiedenbrug||BOLD:ACG4398
Limnophyes sp. 2SW|BIOUG06209-E10|Sofia Wiedenbrug||BOLD:ACG4398
Limnophyes sp. 2SW|BIOUG07928-E04|Sofia Wiedenbrug||BOLD:ACG4398
Limnophyes sp. 2SW|BIOUG07567-G12|Sofia Wiedenbrug||BOLD:ACG4398
Limnophyes sp. 2SW|BIOUG07808-H08|Sofia Wiedenbrug|BIN Taxonomy Match|BOLD:ACG4398
Limnophyes sp. 2SW|BIOUG07808-D02|Sofia Wiedenbrug|BIN Taxonomy Match|BOLD:ACG4398
Limnophyes sp. 2SW|BIOUG07565-D02|Sofia Wiedenbrug|BOLD ID Engine Manual|BOLD:ACG4398
Limnophyes sp. 2SW|BIOUG07808-A01|Sofia Wiedenbrug|BOLD ID Engine Manual|BOLD:ACG4398
Limnophyes sp. 2SW|BIOUG07809-G09|Sofia Wiedenbrug|BIN Taxonomy Match|BOLD:ACG4398
Limnophyes sp. 2SW|BIOUG07407-A05|Sofia Wiedenbrug|BOLD ID Engine Manual|BOLD:ACG4398
Limnophyes sp. 2SW|BIOUG07646-B09|Sofia Wiedenbrug|BIN Taxonomy Match|BOLD:ACG4398
Limnophyes sp. 2SW|BIOUG07808-G09|Sofia Wiedenbrug|BIN Taxonomy Match|BOLD:ACG4398
Limnophyes sp. 2SW|BIOUG06052-D02|Sofia Wiedenbrug||BOLD:ACG4398
Limnophyes sp. 2SW|BIOUG07567-C04|Sofia Wiedenbrug||BOLD:ACG4398
Limnophyes sp. 2SW|BIOUG07757-F03|Sofia Wiedenbrug||BOLD:ACG4398
Limnophyes sp. 2SW|BIOUG07418-D04|Sofia Wiedenbrug|BOLD ID Engine Manual|BOLD:ACG4398
Limnophyes sp. 2SW|BIOUG07410-C11|Sofia Wiedenbrug||BOLD:ACG4398
Limnophyes sp. 2SW|BIOUG07757-B07|Sofia Wiedenbrug||BOLD:ACG4398
Limnophyes sp. 2SW|BIOUG07930-B04|Sofia Wiedenbrug||BOLD:ACG4398
Limnophyes sp. 2SW|BIOUG07415-H06|Sofia Wiedenbrug|BIN Taxonomy Match|BOLD:ACG4398
Limnophyes sp. 2SW|BIOUG07405-B10|Sofia Wiedenbrug||BOLD:ACG4398
Limnophyes sp. 2SW|BIOUG06210-D02|Sofia Wiedenbrug|BIN Taxonomy Match|BOLD:ACG4398
Limnophyes sp. 2SW|BIOUG07805-H04|Sofia Wiedenbrug|BOLD ID Engine Manual|BOLD:ACG4398
Limnophyes sp. 2SW|BIOUG05780-F01|Sofia Wiedenbrug||BOLD:ACG4398
Limnophyes sp. 2SW|BIOUG06093-C11|Sofia Wiedenbrug||BOLD:ACG4398
Limnophyes sp. 2SW|BIOUG05777-H11|Sofia Wiedenbrug||BOLD:ACG4398
Limnophyes sp. 2SW|BIOUG08136-H11|Sofia Wiedenbrug||BOLD:ACG4398
Limnophyes sp. 2SW|BIOUG07759-A01|Sofia Wiedenbrug||BOLD:ACG4398
Limnophyes sp. 2SW|BIOUG07755-C09|Sofia Wiedenbrug||BOLD:ACG4398
Limnophyes sp. 2SW|BIOUG08132-G09|Sofia Wiedenbrug||BOLD:ACG4398
Limnophyes sp. 2SW|BIOUG08128-D02|Sofia Wiedenbrug||BOLD:ACG4398
Limnophyes sp. 2SW|BIOUG08318-D11|Sofia Wiedenbrug||BOLD:ACG4398
Limnophyes sp. 2SW|BIOUG07410-C03|Sofia Wiedenbrug||BOLD:ACG4398
Limnophyes sp. 2SW|BIOUG07944-D12|Sofia Wiedenbrug||BOLD:ACG4398
Limnophyes sp. 2SW|BIOUG07569-H09|Sofia Wiedenbrug||BOLD:ACG4398
Limnophyes sp. 2SW|BIOUG06090-D10|Sofia Wiedenbrug||BOLD:ACG4398
Limnophyes sp. 2SW|BIOUG05944-G07|Sofia Wiedenbrug||BOLD:ACG4398
Limnophyes sp. 2SW|BIOUG07944-E04|Sofia Wiedenbrug||BOLD:ACG4398
Limnophyes sp. 2SW|BIOUG07810-E11|Sofia Wiedenbrug||BOLD:ACG4398
Limnophyes sp. 2SW|BIOUG08128-F05|Sofia Wiedenbrug||BOLD:ACG4398
Limnophyes sp. 2SW|BIOUG07647-A01|Sofia Wiedenbrug||BOLD:ACG4398
Limnophyes sp. 2SW|BIOUG07565-B10|Sofia Wiedenbrug||BOLD:ACG4398
Limnophyes sp. 2SW|BIOUG07809-A12|Sofia Wiedenbrug|BIN Taxonomy Match|BOLD:ACG4398
Limnophyes sp. 2SW|BIOUG07809-B08|Sofia Wiedenbrug|BIN Taxonomy Match|BOLD:ACG4398
Limnophyes sp. 2SW|BIOUG07809-G10|Sofia Wiedenbrug|BIN Taxonomy Match|BOLD:ACG4398
Limnophyes sp. 2SW|BIOUG07646-H09|Sofia Wiedenbrug|BOLD ID Engine Manual|BOLD:ACG4398
Limnophyes sp. 2SW|BIOUG07646-E12|Sofia Wiedenbrug|BIN Taxonomy Match|BOLD:ACG4398
Limnophyes sp. 2SW|BIOUG07567-F06|Sofia Wiedenbrug|BOLD ID Engine Manual|BOLD:ACG4398
Limnophyes sp. 2SW|BIOUG07415-C03|Sofia Wiedenbrug|BOLD ID Engine Manual|BOLD:ACG4398
Limnophyes sp. 2SW|BIOUG07808-C04|Sofia Wiedenbrug|BOLD ID Engine Manual|BOLD:ACG4398
Limnophyes sp. 2SW|BIOUG07646-A06|Sofia Wiedenbrug|BOLD ID Engine Manual|BOLD:ACG4398
Limnophyes sp. 2SW|BIOUG06210-D06|Sofia Wiedenbrug||BOLD:ACG4398
Limnophyes sp. 2SW|BIOUG07646-G06|Sofia Wiedenbrug|BOLD ID Engine Manual|BOLD:ACG4398
Limnophyes sp. 2SW|BIOUG07566-C01|Sofia Wiedenbrug|BOLD ID Engine Manual|BOLD:ACG4398

Limnophyes sp. 2SW|BIOUG07646-A06|Sofia Wiedenbrug|BOLD ID Engine Manual|BOLD:ACG4398
Limnophyes sp. 2SW|BIOUG06210-D06|Sofia Wiedenbrug||BOLD:ACG4398
Limnophyes sp. 2SW|BIOUG07646-G06|Sofia Wiedenbrug|BOLD ID Engine Manual|BOLD:ACG4398
Limnophyes sp. 2SW|BIOUG07566-C01|Sofia Wiedenbrug|BOLD ID Engine Manual|BOLD:ACG4398
Limnophyes sp. 2SW|BIOUG07565-D06|Sofia Wiedenbrug||BOLD:ACG4398
Limnophyes sp. 2SW|BIOUG07565-H07|Sofia Wiedenbrug||BOLD:ACG4398
Limnophyes sp. 2SW|BIOUG06100-C03|Sofia Wiedenbrug|BOLD ID Engine Manual|BOLD:ACG4398
Limnophyes sp. 2SW|BIOUG07928-C03|Sofia Wiedenbrug||BOLD:ACG4398
Limnophyes sp. 2SW|BIOUG07809-H08|Sofia Wiedenbrug|BIN Taxonomy Match|BOLD:ACG4398
Limnophyes sp. 2SW|BIOUG08370-D04|Sofia Wiedenbrug||BOLD:ACG4398
Limnophyes sp. 2SW|BIOUG06053-C11|Sofia Wiedenbrug||BOLD:ACG4398
Limnophyes sp. 2SW|BIOUG07753-F01|Sofia Wiedenbrug||BOLD:ACG4398
Limnophyes sp. 2SW|BIOUG06090-B06|Sofia Wiedenbrug||BOLD:ACG4398
Limnophyes sp. 2SW|BIOUG07754-F04|Sofia Wiedenbrug||BOLD:ACG4398
Limnophyes sp. 2SW|BIOUG05777-E10|Sofia Wiedenbrug||BOLD:ACG4398
Limnophyes sp. 2SW|BIOUG07930-E04|Sofia Wiedenbrug||BOLD:ACG4398
Limnophyes sp. 2SW|BIOUG07566-D11|Sofia Wiedenbrug||BOLD:ACG4398
Limnophyes sp. 2SW|BIOUG06208-D01|Sofia Wiedenbrug||BOLD:ACG4398
Limnophyes sp. 2SW|BIOUG07566-C04|Sofia Wiedenbrug||BOLD:ACG4398
Limnophyes sp. 2SW|BIOUG06095-G07|Sofia Wiedenbrug||BOLD:ACG4398
Limnophyes sp. 2SW|BIOUG07569-H03|Sofia Wiedenbrug||BOLD:ACG4398
Limnophyes sp. 2SW|BIOUG07929-C09|Sofia Wiedenbrug||BOLD:ACG4398
Limnophyes sp. 2SW|BIOUG06450-C08|Sofia Wiedenbrug||BOLD:ACG4398
Limnophyes sp. 2SW|BIOUG06210-E08|Sofia Wiedenbrug|BIN Taxonomy Match|BOLD:ACG4398
Limnophyes sp. 2SW|BIOUG07928-F02|Sofia Wiedenbrug||BOLD:ACG4398
Limnophyes sp. 2SW|BIOUG07929-C08|Sofia Wiedenbrug||BOLD:ACG4398
Limnophyes sp. 2SW|BIOUG08180-E02|Sofia Wiedenbrug||BOLD:ACG4398
Limnophyes sp. 2SW|BIOUG07929-F08|Sofia Wiedenbrug||BOLD:ACG4398
Limnophyes sp. 2SW|BIOUG07568-A10|Sofia Wiedenbrug||BOLD:ACG4398
Limnophyes sp. 2SW|BIOUG07567-A05|Sofia Wiedenbrug||BOLD:ACG4398
Limnophyes sp. 2SW|BIOUG07410-G06|Sofia Wiedenbrug||BOLD:ACG4398
Limnophyes sp. 2SW|BIOUG07808-H04|Sofia Wiedenbrug|BIN Taxonomy Match|BOLD:ACG4398
Limnophyes sp. 2SW|BIOUG07413-C09|Sofia Wiedenbrug||BOLD:ACG4398
Limnophyes sp. 2SW|BIOUG07945-C08|Sofia Wiedenbrug||BOLD:ACG4398
Limnophyes sp. 2SW|BIOUG05776-A03|Sofia Wiedenbrug||BOLD:ACG4398
Limnophyes sp. 2SW|BIOUG08138-D10|Sofia Wiedenbrug||BOLD:ACG4398
Limnophyes sp. 2SW|BIOUG08097-D05|Sofia Wiedenbrug||BOLD:ACG4398
Limnophyes sp. 2SW|BIOUG08132-F04|Sofia Wiedenbrug||BOLD:ACG4398
Limnophyes sp. 2SW|BIOUG07930-E11|Sofia Wiedenbrug||BOLD:ACG4398
Limnophyes sp. 2SW|BIOUG07755-H09|Sofia Wiedenbrug||BOLD:ACG4398
Limnophyes sp. 2SW|BIOUG07759-D08|Sofia Wiedenbrug||BOLD:ACG4398
Limnophyes sp. 2SW|BIOUG07568-H11|Sofia Wiedenbrug||BOLD:ACG4398
Limnophyes sp. 2SW|BIOUG07756-G08|Sofia Wiedenbrug||BOLD:ACG4398
Limnophyes sp. 2SW|BIOUG08130-A12|Sofia Wiedenbrug||BOLD:ACG4398
Limnophyes sp. 2SW|BIOUG07566-A03|Sofia Wiedenbrug||BOLD:ACG4398
Limnophyes sp. 2SW|BIOUG08125-F07|Sofia Wiedenbrug||BOLD:ACG4398
Limnophyes sp. 2SW|BIOUG06450-A07|Sofia Wiedenbrug||BOLD:ACG4398
Limnophyes sp. 2SW|BIOUG07410-G08|Sofia Wiedenbrug||BOLD:ACG4398
Limnophyes sp. 2SW|BIOUG07563-A12|Sofia Wiedenbrug||BOLD:ACG4398
Limnophyes sp. 2SW|BIOUG07753-B02|Sofia Wiedenbrug||BOLD:ACG4398
Limnophyes sp. 2SW|BIOUG08133-G12|Sofia Wiedenbrug||BOLD:ACG4398
Limnophyes sp. 2SW|BIOUG07414-G03|Sofia Wiedenbrug||BOLD:ACG4398
Limnophyes sp. 2SW|BIOUG07414-B04|Sofia Wiedenbrug||BOLD:ACG4398
Limnophyes sp. 2SW|BIOUG07414-E02|Sofia Wiedenbrug||BOLD:ACG4398
Limnophyes sp. 2SW|BIOUG08129-G10|Sofia Wiedenbrug||BOLD:ACG4398
Limnophyes sp. 2SW|BIOUG07753-D07|Sofia Wiedenbrug||BOLD:ACG4398
Limnophyes sp. 2SW|BIOUG06093-H08|Sofia Wiedenbrug||BOLD:ACG4398
Limnophyes sp. 2SW|BIOUG08092-B12|Sofia Wiedenbrug||BOLD:ACG4398
Limnophyes sp. 2SW|BIOUG08174-D04|Sofia Wiedenbrug||BOLD:ACG4398
Limnophyes sp. 2SW|BIOUG06516-B10|Sofia Wiedenbrug||BOLD:ACG4398
Limnophyes sp. 2SW|BIOUG07928-H05|Sofia Wiedenbrug||BOLD:ACG4398
Limnophyes sp. 2SW|BIOUG07568-D04|Sofia Wiedenbrug||BOLD:ACG4398
Limnophyes sp. 2SW|BIOUG07569-D05|Sofia Wiedenbrug||BOLD:ACG4398
Limnophyes sp. 2SW|BIOUG07408-A10|Sofia Wiedenbrug||BOLD:ACG4398
Limnophyes sp. 2SW|BIOUG07408-G11|Sofia Wiedenbrug||BOLD:ACG4398
Limnophyes sp. 2SW|BIOUG07409-G08|Sofia Wiedenbrug||BOLD:ACG4398
Limnophyes sp. 2SW|BIOUG07808-A07|Sofia Wiedenbrug|BIN Taxonomy Match|BOLD:ACG4398
Limnophyes sp. 2SW|BIOUG07809-F07|Sofia Wiedenbrug|BIN Taxonomy Match|BOLD:ACG4398
Limnophyes sp. 2SW|BIOUG07809-H05|Sofia Wiedenbrug|BIN Taxonomy Match|BOLD:ACG4398
Limnophyes sp. 2SW|BIOUG07809-H06|Sofia Wiedenbrug|BIN Taxonomy Match|BOLD:ACG4398
Limnophyes sp. 2SW|BIOUG07413-C04|Sofia Wiedenbrug||BOLD:ACG4398
Limnophyes sp. 2SW|BIOUG06052-D06|Sofia Wiedenbrug||BOLD:ACG4398
Limnophyes sp. 2SW|BIOUG07408-D09|Sofia Wiedenbrug||BOLD:ACG4398
Limnophyes sp. 2SW|BIOUG07944-H08|Sofia Wiedenbrug||BOLD:ACG4398
Limnophyes sp. 2SW|BIOUG08129-C06|Sofia Wiedenbrug||BOLD:ACG4398
Limnophyes sp. 2SW|BIOUG06052-C07|Sofia Wiedenbrug||BOLD:ACG4398
Limnophyes sp. 2SW|BIOUG08092-B11|Sofia Wiedenbrug||BOLD:ACG4398
Limnophyes sp. 2SW|BIOUG07755-E08|Sofia Wiedenbrug||BOLD:ACG4398
Limnophyes sp. 2SW|BIOUG07565-D08|Sofia Wiedenbrug||BOLD:ACG4398
Limnophyes sp. 2SW|BIOUG07565-A04|Sofia Wiedenbrug||BOLD:ACG4398
Limnophyes sp. 2SW|BIOUG07410-G03|Sofia Wiedenbrug||BOLD:ACG4398
Limnophyes sp. 2SW|BIOUG07414-G09|Sofia Wiedenbrug||BOLD:ACG4398
Limnophyes sp. 2SW|BIOUG07414-G07|Sofia Wiedenbrug||BOLD:ACG4398
Limnophyes sp. 2SW|BIOUG07510-E07|Sofia Wiedenbrug||BOLD:ACG4398
Limnophyes sp. 2SW|BIOUG07510-C02|Sofia Wiedenbrug||BOLD:ACG4398
Limnophyes sp. 2SW|BIOUG07509-B08|Sofia Wiedenbrug||BOLD:ACG4398
Limnophyes sp. 2SW|BIOUG08174-F05|Sofia Wiedenbrug||BOLD:ACG4398
Limnophyes sp. 2SW|BIOUG07753-D04|Sofia Wiedenbrug||BOLD:ACG4398
Limnophyes sp. 2SW|BIOUG07753-E09|Sofia Wiedenbrug||BOLD:ACG4398
Limnophyes sp. 2SW|BIOUG07928-E10|Sofia Wiedenbrug||BOLD:ACG4398
Limnophyes sp. 2SW|BIOUG07809-G02|Sofia Wiedenbrug|BIN Taxonomy Match|BOLD:ACG4398
Limnophyes sp. 2SW|BIOUG07568-G10|Sofia Wiedenbrug||BOLD:ACG4398
Limnophyes sp. 2SW|BIOUG07569-A08|Sofia Wiedenbrug||BOLD:ACG4398
Limnophyes sp. 2SW|BIOUG07569-A11|Sofia Wiedenbrug||BOLD:ACG4398
Limnophyes sp. 2SW|BIOUG07569-C05|Sofia Wiedenbrug||BOLD:ACG4398
Limnophyes sp. 2SW|BIOUG07569-E07|Sofia Wiedenbrug||BOLD:ACG4398
Limnophyes sp. 2SW|BIOUG07413-F09|Sofia Wiedenbrug||BOLD:ACG4398
Limnophyes sp. 2SW|CCDB24038-C08|Sofia Wiedenbrug|BIN Taxonomy Match|BOLD:ACG4398
Limnophyes sp. 2SW|BIOUG06093-D09|Sofia Wiedenbrug||BOLD:ACG4398
Limnophyes sp. 2SW|BIOUG07504-B07|Sofia Wiedenbrug|BIN Taxonomy Match|BOLD:ACG4398
Limnophyes sp. 2SW|BIOUG07415-E06|Sofia Wiedenbrug|BIN Taxonomy Match|BOLD:ACG4398
Limnophyes sp. 2SW|BIOUG07415-A12|Sofia Wiedenbrug|BIN Taxonomy Match|BOLD:ACG4398
Limnophyes sp. 2SW|BIOUG07415-D06|Sofia Wiedenbrug|BIN Taxonomy Match|BOLD:ACG4398
[illegible]

Limnophyes sp. 2SW|BIOUG07415-E06|Sofia Wiedenbrug|BIN Taxonomy Match|BOLD:ACG4398
Limnophyes sp. 2SW|BIOUG07415-A12|Sofia Wiedenbrug|BIN Taxonomy Match|BOLD:ACG4398
Limnophyes sp. 2SW|BIOUG07415-D06|Sofia Wiedenbrug|BIN Taxonomy Match|BOLD:ACG4398
Limnophyes sp. 2SW|BIOUG07415-E04|Sofia Wiedenbrug|BIN Taxonomy Match|BOLD:ACG4398
Limnophyes sp. 2SW|BIOUG07568-F02|Sofia Wiedenbrug||BOLD:ACG4398
Limnophyes sp. 2SW|BIOUG07414-E09|Sofia Wiedenbrug||BOLD:ACG4398
Limnophyes sp. 2SW|BIOUG07413-C01|Sofia Wiedenbrug||BOLD:ACG4398
Limnophyes sp. 2SW|BIOUG07414-C12|Sofia Wiedenbrug||BOLD:ACG4398
Limnophyes sp. 2SW|BIOUG06088-D02|Sofia Wiedenbrug||BOLD:ACG4398
Limnophyes sp. 2SW|BIOUG06056-F06|Sofia Wiedenbrug||BOLD:ACG4398
Limnophyes sp. 2SW|BIOUG06051-B12|Sofia Wiedenbrug||BOLD:ACG4398
Limnophyes sp. 2SW|BIOUG08127-H06|Sofia Wiedenbrug||BOLD:ACG4398
Limnophyes sp. 2SW|BIOUG06094-G11|Sofia Wiedenbrug||BOLD:ACG4398
Limnophyes sp. 2SW|BIOUG06095-C10|Sofia Wiedenbrug||BOLD:ACG4398
Limnophyes sp. 2SW|BIOUG06095-D09|Sofia Wiedenbrug||BOLD:ACG4398
Limnophyes sp. 2SW|BIOUG06096-B05|Sofia Wiedenbrug||BOLD:ACG4398
Limnophyes sp. 2SW|BIOUG07408-F03|Sofia Wiedenbrug||BOLD:ACG4398
Limnophyes sp. 2SW|BIOUG05780-F08|Sofia Wiedenbrug||BOLD:ACG4398
Limnophyes sp. 2SW|BIOUG07929-F07|Sofia Wiedenbrug||BOLD:ACG4398
Limnophyes sp. 2SW|BIOUG05945-C09|Sofia Wiedenbrug||BOLD:ACG4398
Limnophyes sp. 2SW|BIOUG07757-G08|Sofia Wiedenbrug||BOLD:ACG4398
Limnophyes sp. 2SW|BIOUG07754-D10|Sofia Wiedenbrug||BOLD:ACG4398
Limnophyes sp. 2SW|BIOUG07418-F11|Sofia Wiedenbrug|BIN Taxonomy Match|BOLD:ACG4398
Limnophyes sp. 2SW|BIOUG06051-C10|Sofia Wiedenbrug||BOLD:ACG4398
Limnophyes sp. 2SW|BIOUG06056-H11|Sofia Wiedenbrug||BOLD:ACG4398
Limnophyes sp. 2SW|BIOUG06096-E10|Sofia Wiedenbrug||BOLD:ACG4398
Limnophyes sp. 2SW|BIOUG07929-C11|Sofia Wiedenbrug||BOLD:ACG4398
Limnophyes sp. 2SW|BIOUG08135-G01|Sofia Wiedenbrug||BOLD:ACG4398
Limnophyes sp. 2SW|BIOUG07944-C05|Sofia Wiedenbrug||BOLD:ACG4398
Limnophyes sp. 2SW|BIOUG07944-E11|Sofia Wiedenbrug||BOLD:ACG4398
Limnophyes sp. 2SW|BIOUG06210-G01|Sofia Wiedenbrug|BIN Taxonomy Match|BOLD:ACG4398
Limnophyes sp. 2SW|BIOUG05944-A07|Sofia Wiedenbrug||BOLD:ACG4398
Limnophyes sp. 2SW|BIOUG06058-A09|Sofia Wiedenbrug||BOLD:ACG4398
Limnophyes sp. 2SW|BIOUG06096-D07|Sofia Wiedenbrug||BOLD:ACG4398
Limnophyes sp. 2SW|BIOUG06065-F09|Sofia Wiedenbrug||BOLD:ACG4398
Limnophyes sp. 2SW|BIOUG07503-G12|Sofia Wiedenbrug|BIN Taxonomy Match|BOLD:ACG4398
Limnophyes sp. 2SW|BIOUG06056-F08|Sofia Wiedenbrug||BOLD:ACG4398
Limnophyes sp. 2SW|BIOUG05947-H07|Sofia Wiedenbrug||BOLD:ACG4398
Limnophyes sp. 2SW|BIOUG06092-C12|Sofia Wiedenbrug||BOLD:ACG4398
Limnophyes sp. 2SW|BIOUG07415-H07|Sofia Wiedenbrug|BIN Taxonomy Match|BOLD:ACG4398
Limnophyes sp. 2SW|BIOUG06055-D09|Sofia Wiedenbrug||BOLD:ACG4398
Limnophyes sp. 2SW|BIOUG05780-D11|Sofia Wiedenbrug||BOLD:ACG4398
Limnophyes sp. 2SW|BIOUG05944-C12|Sofia Wiedenbrug||BOLD:ACG4398
Limnophyes sp. 2SW|BIOUG05944-D09|Sofia Wiedenbrug||BOLD:ACG4398
Limnophyes sp. 2SW|BIOUG07407-D08|Sofia Wiedenbrug||BOLD:ACG4398
Limnophyes sp. 2SW|BIOUG07407-C06|Sofia Wiedenbrug||BOLD:ACG4398
Limnophyes sp. 2SW|BIOUG06210-A01|Sofia Wiedenbrug||BOLD:ACG4398
Limnophyes sp. 2SW|BIOUG06210-H06|Sofia Wiedenbrug|BIN Taxonomy Match|BOLD:ACG4398
Limnophyes sp. 2SW|BIOUG07408-D02|Sofia Wiedenbrug||BOLD:ACG4398
Limnophyes sp. 2SW|BIOUG05944-H10|Sofia Wiedenbrug||BOLD:ACG4398
Limnophyes sp. 2SW|BIOUG06049-A12|Sofia Wiedenbrug||BOLD:ACG4398
Limnophyes sp. 2SW|BIOUG06054-G10|Sofia Wiedenbrug||BOLD:ACG4398
Limnophyes sp. 2SW|BIOUG06058-E04|Sofia Wiedenbrug||BOLD:ACG4398
Limnophyes sp. 2SW|BIOUG07414-G05|Sofia Wiedenbrug||BOLD:ACG4398
Limnophyes sp. 2SW|BIOUG06054-F02|Sofia Wiedenbrug||BOLD:ACG4398
Limnophyes sp. 2SW|BIOUG06096-C12|Sofia Wiedenbrug||BOLD:ACG4398
Limnophyes sp. 2SW|BIOUG07415-C11|Sofia Wiedenbrug|BIN Taxonomy Match|BOLD:ACG4398
Limnophyes sp. 2SW|BIOUG06054-A07|Sofia Wiedenbrug||BOLD:ACG4398
Limnophyes sp. 2SW|BIOUG06210-C09|Sofia Wiedenbrug|BIN Taxonomy Match|BOLD:ACG4398
Limnophyes sp. 2SW|BIOUG06208-G05|Sofia Wiedenbrug||BOLD:ACG4398
Limnophyes sp. 2SW|BIOUG08320-F04|Sofia Wiedenbrug||BOLD:ACG4398
Limnophyes sp. 2SW|BIOUG06054-G08|Sofia Wiedenbrug||BOLD:ACG4398
Limnophyes sp. 2SW|BIOUG06094-C10|Sofia Wiedenbrug||BOLD:ACG4398
Limnophyes sp. 2SW|BIOUG07805-A05|Sofia Wiedenbrug|BIN Taxonomy Match|BOLD:ACG4398
Limnophyes sp. 2SW|BIOUG07805-D08|Sofia Wiedenbrug|BOLD ID Engine Manual|BOLD:ACG4398
Limnophyes sp. 2SW|BIOUG07805-F02|Sofia Wiedenbrug|BOLD ID Engine Manual|BOLD:ACG4398
Limnophyes sp. 2SW|BIOUG07805-H10|Sofia Wiedenbrug|BIN Taxonomy Match|BOLD:ACG4398
Limnophyes sp. 2SW|BIOUG05780-G06|Sofia Wiedenbrug||BOLD:ACG4398
Limnophyes sp. 2SW|BIOUG05944-H09|Sofia Wiedenbrug||BOLD:ACG4398
Limnophyes sp. 2SW|BIOUG06095-A09|Sofia Wiedenbrug||BOLD:ACG4398
Limnophyes sp. 2SW|BIOUG05777-B01|Sofia Wiedenbrug||BOLD:ACG4398
Limnophyes sp. 2SW|BIOUG05777-E08|Sofia Wiedenbrug||BOLD:ACG4398
Limnophyes sp. 2SW|CCDB21606-G03|Sofia Wiedenbrug||BOLD:ACG4398
Limnophyes sp. 2SW|BIOUG07407-A10|Sofia Wiedenbrug||BOLD:ACG4398
Limnophyes sp. 2SW|BIOUG06093-B02|Sofia Wiedenbrug||BOLD:ACG4398
Limnophyes sp. 2SW|BIOUG05780-A04|Sofia Wiedenbrug||BOLD:ACG4398
Limnophyes sp. 2SW|BIOUG05945-D01|Sofia Wiedenbrug||BOLD:ACG4398
Limnophyes sp. 2SW|BIOUG07405-H06|Sofia Wiedenbrug||BOLD:ACG4398
Limnophyes sp. 2SW|BIOUG07406-A08|Sofia Wiedenbrug||BOLD:ACG4398
Limnophyes sp. 2SW|BIOUG07406-G04|Sofia Wiedenbrug||BOLD:ACG4398
Limnophyes sp. 2SW|BIOUG06094-D10|Sofia Wiedenbrug||BOLD:ACG4398
Limnophyes sp. 2SW|BIOUG07929-E01|Sofia Wiedenbrug||BOLD:ACG4398
Limnophyes sp. 2SW|BIOUG05777-G03|Sofia Wiedenbrug||BOLD:ACG4398
Limnophyes sp. 2SW|BIOUG07757-F11|Sofia Wiedenbrug||BOLD:ACG4398
Limnophyes sp. 2SW|BIOUG07759-A09|Sofia Wiedenbrug||BOLD:ACG4398
Limnophyes sp. 2SW|BIOUG07563-B03|Sofia Wiedenbrug||BOLD:ACG4398
Limnophyes sp. 2SW|BIOUG05900-E11|Sofia Wiedenbrug||BOLD:ACG4398
Limnophyes sp. 2SW|BIOUG07759-B02|Sofia Wiedenbrug||BOLD:ACG4398
Limnophyes sp. 2SW|BIOUG07563-A08|Sofia Wiedenbrug||BOLD:ACG4398
Limnophyes sp. 2SW|BIOUG07754-H08|Sofia Wiedenbrug||BOLD:ACG4398
Limnophyes sp. 2SW|BIOUG08135-B11|Sofia Wiedenbrug||BOLD:ACG4398
Limnophyes sp. 2SW|BIOUG08140-G11|Sofia Wiedenbrug||BOLD:ACG4398
Limnophyes sp. 2SW|BIOUG07758-D10|Sofia Wiedenbrug||BOLD:ACG4398
Limnophyes sp. 2SW|BIOUG07415-F02|Sofia Wiedenbrug|BOLD ID Engine Manual|BOLD:ACG4398
Limnophyes sp. 2SW|BIOUG07758-E10|Sofia Wiedenbrug||BOLD:ACG4398
Limnophyes sp. 2SW|BIOUG07409-A03|Sofia Wiedenbrug||BOLD:ACG4398
Limnophyes sp. 2SW|BIOUG07563-G05|Sofia Wiedenbrug||BOLD:ACG4398
Limnophyes sp. 2SW|BIOUG06516-C11|Sofia Wiedenbrug||BOLD:ACG4398
Limnophyes sp. 2SW|BIOUG07755-D06|Sofia Wiedenbrug||BOLD:ACG4398
Limnophyes sp. 2SW|BIOUG07755-B09|Sofia Wiedenbrug||BOLD:ACG4398
Limnophyes sp. 2SW|BIOUG07754-A09|Sofia Wiedenbrug||BOLD:ACG4398
Limnophyes sp. 2SW|BIOUG07754-E02|Sofia Wiedenbrug||BOLD:ACG4398
Limnophyes sp. 2SW|BIOUG07754-B11|Sofia Wiedenbrug||BOLD:ACG4398

Limnophyes sp. 2SW|BIOUG07754-A09|Sofia Wiedenbrug||BOLD:ACG4398
Limnophyes sp. 2SW|BIOUG07754-E02|Sofia Wiedenbrug||BOLD:ACG4398
Limnophyes sp. 2SW|BIOUG07754-B11|Sofia Wiedenbrug||BOLD:ACG4398
Limnophyes sp. 2SW|BIOUG07754-A11|Sofia Wiedenbrug||BOLD:ACG4398
Limnophyes sp. 2SW|BIOUG08369-G11|Sofia Wiedenbrug||BOLD:ACG4398
Limnophyes sp. 2SW|BIOUG05776-D11|Sofia Wiedenbrug||BOLD:ACG4398
Limnophyes sp. 2SW|BIOUG08130-A11|Sofia Wiedenbrug||BOLD:ACG4398
Limnophyes sp. 2SW|BIOUG08127-B02|Sofia Wiedenbrug||BOLD:ACG4398
Limnophyes sp. 2SW|BIOUG07760-D10|Sofia Wiedenbrug||BOLD:ACG4398
Limnophyes sp. 2SW|BIOUG07755-E09|Sofia Wiedenbrug||BOLD:ACG4398
Limnophyes sp. 2SW|BIOUG07757-C09|Sofia Wiedenbrug||BOLD:ACG4398
Limnophyes sp. 2SW|BIOUG07760-F08|Sofia Wiedenbrug||BOLD:ACG4398
Limnophyes sp. 2SW|BIOUG07758-B07|Sofia Wiedenbrug||BOLD:ACG4398
Limnophyes sp. 2SW|BIOUG07757-D05|Sofia Wiedenbrug||BOLD:ACG4398
Limnophyes sp. 2SW|BIOUG07758-H08|Sofia Wiedenbrug||BOLD:ACG4398
Limnophyes sp. 2SW|BIOUG07759-B07|Sofia Wiedenbrug||BOLD:ACG4398
Limnophyes sp. 2SW|BIOUG07759-C03|Sofia Wiedenbrug||BOLD:ACG4398
Limnophyes sp. 2SW|BIOUG07563-B09|Sofia Wiedenbrug||BOLD:ACG4398
Limnophyes sp. 2SW|BIOUG07647-H11|Sofia Wiedenbrug||BOLD:ACG4398
Limnophyes sp. 2SW|BIOUG07760-B07|Sofia Wiedenbrug||BOLD:ACG4398
Limnophyes sp. 2SW|BIOUG07810-G01|Sofia Wiedenbrug||BOLD:ACG4398
Limnophyes sp. 2SW|BIOUG06441-H11|Sofia Wiedenbrug||BOLD:ACG4398
Limnophyes sp. 2SW|BIOUG07758-D04|Sofia Wiedenbrug||BOLD:ACG4398
Limnophyes sp. 2SW|BIOUG07755-C04|Sofia Wiedenbrug||BOLD:ACG4398
Limnophyes sp. 2SW|BIOUG08095-F03|Sofia Wiedenbrug||BOLD:ACG4398
Limnophyes sp. 2SW|BIOUG08369-G02|Sofia Wiedenbrug||BOLD:ACG4398
Limnophyes sp. 2SW|BIOUG07759-B04|Sofia Wiedenbrug||BOLD:ACG4398
Limnophyes sp. 2SW|BIOUG08098-E07|Sofia Wiedenbrug||BOLD:ACG4398
Limnophyes sp. 2SW|BIOUG07754-A01|Sofia Wiedenbrug||BOLD:ACG4398
Limnophyes sp. 2SW|BIOUG07754-G04|Sofia Wiedenbrug||BOLD:ACG4398
Limnophyes sp. 2SW|BIOUG07810-D08|Sofia Wiedenbrug||BOLD:ACG4398
Limnophyes sp. 2SW|BIOUG08130-F03|Sofia Wiedenbrug||BOLD:ACG4398
Limnophyes sp. 2SW|BIOUG08135-B06|Sofia Wiedenbrug||BOLD:ACG4398
Limnophyes sp. 2SW|BIOUG08135-G07|Sofia Wiedenbrug||BOLD:ACG4398
Limnophyes sp. 2SW|BIOUG07930-G02|Sofia Wiedenbrug||BOLD:ACG4398
Limnophyes sp. 2SW|BIOUG06443-D01|Sofia Wiedenbrug||BOLD:ACG4398
Limnophyes sp. 2SW|BIOUG08139-A11|Sofia Wiedenbrug||BOLD:ACG4398
Limnophyes sp. 2SW|BIOUG06516-C10|Sofia Wiedenbrug||BOLD:ACG4398
Limnophyes sp. 2SW|BIOUG07753-B06|Sofia Wiedenbrug||BOLD:ACG4398
Limnophyes sp. 2SW|BIOUG07753-C06|Sofia Wiedenbrug||BOLD:ACG4398
Limnophyes sp. 2SW|BIOUG07753-E05|Sofia Wiedenbrug||BOLD:ACG4398
Limnophyes sp. 2SW|BIOUG07754-H11|Sofia Wiedenbrug||BOLD:ACG4398
Limnophyes sp. 2SW|BIOUG07755-C05|Sofia Wiedenbrug||BOLD:ACG4398
Limnophyes sp. 2SW|BIOUG07755-F08|Sofia Wiedenbrug||BOLD:ACG4398
Limnophyes sp. 2SW|BIOUG07760-G11|Sofia Wiedenbrug||BOLD:ACG4398
Limnophyes sp. 2SW|BIOUG07760-G12|Sofia Wiedenbrug||BOLD:ACG4398
Limnophyes sp. 2SW|BIOUG07757-E08|Sofia Wiedenbrug||BOLD:ACG4398
Limnophyes sp. 2SW|BIOUG07563-G04|Sofia Wiedenbrug||BOLD:ACG4398
Limnophyes sp. 2SW|BIOUG07757-F04|Sofia Wiedenbrug||BOLD:ACG4398
Limnophyes sp. 2SW|BIOUG07758-E03|Sofia Wiedenbrug||BOLD:ACG4398
Limnophyes sp. 2SW|BIOUG07754-G02|Sofia Wiedenbrug||BOLD:ACG4398
Limnophyes sp. 2SW|BIOUG07755-F09|Sofia Wiedenbrug||BOLD:ACG4398
Limnophyes sp. 2SW|BIOUG06517-G10|Sofia Wiedenbrug||BOLD:ACG4398
Limnophyes sp. 2SW|BIOUG07759-E06|Sofia Wiedenbrug||BOLD:ACG4398
Limnophyes sp. 2SW|BIOUG07759-G12|Sofia Wiedenbrug||BOLD:ACG4398
Limnophyes sp. 2SW|BIOUG07810-D06|Sofia Wiedenbrug||BOLD:ACG4398
Limnophyes sp. 2SW|BIOUG07755-F03|Sofia Wiedenbrug||BOLD:ACG4398
Limnophyes sp. 2SW|BIOUG07759-H05|Sofia Wiedenbrug||BOLD:ACG4398
Limnophyes sp. 2SW|BIOUG08133-A08|Sofia Wiedenbrug||BOLD:ACG4398
Limnophyes sp. 2SW|BIOUG07754-G07|Sofia Wiedenbrug||BOLD:ACG4398
Limnophyes sp. 2SW|BIOUG07810-B12|Sofia Wiedenbrug||BOLD:ACG4398
Limnophyes sp. 2SW|BIOUG07569-F12|Sofia Wiedenbrug||BOLD:ACG4398
Limnophyes sp. 2SW|BIOUG07928-H02|Sofia Wiedenbrug||BOLD:ACG4398
Limnophyes sp. 2SW|BIOUG08097-C09|Sofia Wiedenbrug||BOLD:ACG4398
Limnophyes sp. 2SW|BIOUG07647-F02|Sofia Wiedenbrug||BOLD:ACG4398
Limnophyes sp. 2SW|BIOUG07759-F12|Sofia Wiedenbrug||BOLD:ACG4398
Limnophyes sp. 2SW|BIOUG07647-C03|Sofia Wiedenbrug||BOLD:ACG4398
Limnophyes sp. 2SW|BIOUG06100-F01|Sofia Wiedenbrug||BOLD:ACG4398
Limnophyes sp. 2SW|BIOUG06100-D04|Sofia Wiedenbrug||BOLD:ACG4398
Limnophyes sp. 2SW|BIOUG06100-B08|Sofia Wiedenbrug||BOLD:ACG4398
Limnophyes sp. 5SW|CCDB21606-C11|Sofia Wiedenbrug||BOLD:ACT5786
Limnophyes sp. 6ES|CCDB24037-G11|Sofia Wiedenbrug|BIN Taxonomy Match|BOLD:AAI1341
Limnophyes sp. 6ES|CCDB24183-C06|Sofia Wiedenbrug|Morphology|BOLD:AAI1341
Limnophyes sp. 6ES|ES195|Elisabeth Stur||BOLD:AAI1341
Limnophyes|24371-H05|Mikko J Tiusanen|Tree based identification|BOLD:ACU4591
Limnophyes|23878-G06|Mikko J Tiusanen|Tree based identification|BOLD:ACU4591
Limnophyes|24354-C09|Mikko J Tiusanen|Tree based identification|BOLD:ACU4591
Limnophyes|24372-F04|Mikko J Tiusanen|Tree based identification|BOLD:ACU4591
Limnophyes|24371-D07|Mikko J Tiusanen|Tree based identification|BOLD:ACU4591
Limnophyes|24501-H03|Mikko J Tiusanen|Tree based identification|BOLD:ACU4591
Limnophyes minimus|24530-B06|Kate Perez|BIN Taxonomy Match|BOLD:AAA8202
Limnophyes minimus|24406-D08|Kate Perez|BIN Taxonomy Match|BOLD:AAA8202
Limnophyes minimus|24405-A06|Kate Perez|BIN Taxonomy Match|BOLD:AAA8202
Limnophyes minimus|24464-B10|Kate Perez|BIN Taxonomy Match|BOLD:AAA8202
Limnophyes minimus|24372-C11|Kate Perez|BIN Taxonomy Match|BOLD:AAA8202
Limnophyes minimus|ZSM-DIP-33142-E10|Caroline Chimeno|BIN Taxonomy Match (Jan 2022)|BOLD ...
Limnophyes minimus|SOE471|Elisabeth Stur
Limnophyes sp. 7SW|BIOUG07945-G05|Kate Perez|BIN Taxonomy Match|BOLD:ACG3403
Limnophyes sp. 7SW|BIOUG07414-A06|Kate Perez|BIN Taxonomy Match|BOLD:ACG3403
Limnophyes sp. 7SW|BIOUG07414-D06|Sofia Wiedenbrug|BIN Taxonomy Match|BOLD:ACG3403
Limnophyes sp. 7SW|BIOUG07754-A07|Kate Perez|BIN Taxonomy Match|BOLD:ACG3403
Limnophyes sp. 7SW|BIOUG06055-H06|Sofia Wiedenbrug|BIN Taxonomy Match|BOLD:ACG3403
Limnophyes sp. 7SW|BIOUG05220-C09|Kate Perez|BIN Taxonomy Match|BOLD:ACG3403
Limnophyes sp. 7ES|BIOUG15762-E08|Elisabeth Stur|BIN Taxonomy Match|BOLD:ACG3403
Limnophyes spinigus|CCDB22605-C01|Sofia Wiedenbrug||BOLD:ACT8740
Limnophyes spinigus|ZSM-DIP-33130-D04|Caroline Chimeno|BIN Taxonomy Match (Jan 2022)|BOLD ...
Limnophyes spinigus|CCDB22605-C04|Sofia Wiedenbrug||BOLD:ACT8740
Limnophyes spinigus|CCDB22605-C02|Sofia Wiedenbrug||BOLD:ACT8740
Limnophyes spinigus|ZSM-DIP-33142-F05|Caroline Chimeno|BIN Taxonomy Match (Jan 2022)|BOLD ...
Limnophyes spinigus|ZMUO.024739|Lauri Paasivirta||BOLD:AAB1812
Limnophyes madeirae|Finnmark485|Elisabeth Stur||BOLD:AAB1812
Metriocnemus eurynotus|ZMUO.024684|Lauri Paasivirta||BOLD:ADA7069
Metriocnemus eurynotus|ZMUO.024685|Lauri Paasivirta||BOLD:ADA7069

[illegible]
Limnophyes madeirae|Finnmark485|Elisabeth Stur||BOLD:AAB1812
Metriocnemus eurynotus|ZMUO.024684|Lauri Paasivirta||BOLD:ADA7069
Metriocnemus eurynotus|ZMUO.024685|Lauri Paasivirta||BOLD:ADA7069
Metriocnemus NHRS sp. A|NHRS-BYWS000001054|Yngve Brodin|morphology|BOLD:ADA7069
Metriocnemus brusti|Finnmark633|Elisabeth Stur||BOLD:AAA9438
Metriocnemus sp. 3SW|ZSM34342-G12|Sofia Wiedenbrug|Morphology|BOLD:AAV4654
Metriocnemus sp. 3SW|CCDB24229-E12|Sofia Wiedenbrug|BIN Taxonomy Match|BOLD:AAV4654
Metriocnemus sp. 3SW|ZSM34342-H09|Sofia Wiedenbrug|Morphology|BOLD:AAV4654
Metriocnemus sp. 3SW|ZSM34342-H10|Sofia Wiedenbrug|Morphology|BOLD:AAV4654
Metriocnemus sp. 3SW|ZSM34342-H11|Sofia Wiedenbrug|Morphology|BOLD:AAV4654
Metriocnemus sp. 3SW|ZSM34342-H07|Sofia Wiedenbrug|Morphology|BOLD:AAV4654
Metriocnemus sp. 3SW|ZSM34342-G11|Sofia Wiedenbrug|Morphology|BOLD:AAV4654
Metriocnemus sp. 1ES|Finnmark286|Elisabeth Stur||BOLD:AAV4654
Orthocladiinae|Finnmark613|Elisabeth Stur||BOLD:AAQ2645
Smittia nudipennis|CH-OSF184|Elisabeth Stur||BOLD:AAJ3812
Smittia nudipennis|SOE243|Elisabeth Stur||BOLD:AAJ3812
Smittia nudipennis|SOE430|Elisabeth Stur||BOLD:AAJ3812
Smittia sp. 3SW|CCDB22605-E07|Sofia Wiedenbrug||BOLD:ACU0162
Smittia edwardsi|ZMUO.025316|Lauri Paasivirta||BOLD:ACU0162
Smittia edwardsi|ZMUO.025315|Lauri Paasivirta||BOLD:ACU0162
Smittia|ZMUO.026341|Lauri Paasivirta||BOLD:ACC3486
Smittia|ZMUO.026340|Lauri Paasivirta||BOLD:ACC3486
Smittia|BIOUG04089-A06|Kate Perez|BIN Taxonomy Match (Aug 2017)|BOLD:ACC3486
Smittia|ZSM-DIP-33132-D04|Caroline Chimeno|BIN Taxonomy Match (Jan 2022)|BOLD:ACU4226
Smittia|ZSM-DIP-33132-D08|Caroline Chimeno|BIN Taxonomy Match (Jan 2022)|BOLD:ACU4226
Smittia|ZSM-DIP-33132-C10|Caroline Chimeno|BIN Taxonomy Match (Jan 2022)|BOLD:ACU4226
Smittia|ZSM-DIP-33132-C11|Caroline Chimeno|BIN Taxonomy Match (Jan 2022)|BOLD:ACU4226
Smittia|ZSM-DIP-33132-D03|Caroline Chimeno|BIN Taxonomy Match (Jan 2022)|BOLD:ACU4226
Smittia|CCDB24228-G04|Sofia Wiedenbrug|BIN Taxonomy Match|BOLD:ACU4226
Chironomidae|ZSM-DIP-33143-G02|Valerie Levesque-Beaudin|BOLD ID Engine Manual (Sep 2021)|BO...
Chironomidae|ZSM-DIP-33147-C01|Valerie Levesque-Beaudin|BIN Taxonomy Match (Sep 2021)|BOLD...
Smittia paranudipennis|ZMUO.024965|Lauri Paasivirta||BOLD:ADA5855
Smittia paranudipennis|ZMUO.024964|Lauri Paasivirta||BOLD:ADA5855
Smittia paranudipennis|BIOUG15800-G11|Kate Perez|BIN Taxonomy Match|BOLD:ACK3746
Smittia paranudipennis|BIOUG16626-B04|Kate Perez|BIN Taxonomy Match|BOLD:ACK3746
Smittia paranudipennis|BIOUG15593-D04|Kate Perez|BIN Taxonomy Match|BOLD:ACK3746
Smittia paranudipennis|BIOUG16208-A04|Kate Perez|BIN Taxonomy Match|BOLD:ACK3746
Smittia paranudipennis|BIOUG15853-D09|Kate Perez|BIN Taxonomy Match|BOLD:ACK3746
Smittia paranudipennis|BIOUG15808-B03|Kate Perez|BIN Taxonomy Match|BOLD:ACK3746
Smittia paranudipennis|TRD-CH36|Elisabeth Stur||BOLD:ACK3746
Smittia paranudipennis|BIOUG15796-C05|Kate Perez|BIN Taxonomy Match|BOLD:ACK3746
Smittia paranudipennis|BIOUG15852-D10|Kate Perez|BIN Taxonomy Match|BOLD:ACK3746
Smittia paranudipennis|BIOUG15652-G05|Kate Perez|BIN Taxonomy Match|BOLD:ACK3746
Smittia paranudipennis|BIOUG15899-A03|Kate Perez|BIN Taxonomy Match|BOLD:ACK3746
Smittia paranudipennis|BIOUG15798-A01|Kate Perez|BIN Taxonomy Match|BOLD:ACK3746
Smittia paranudipennis|TRD-CH14|Elisabeth Stur||BOLD:ACK3746
Smittia paranudipennis|BIOUG15674-A02|Kate Perez|BIN Taxonomy Match|BOLD:ACK3746
Smittia paranudipennis|BIOUG15765-F11|Kate Perez|BIN Taxonomy Match|BOLD:ACK3746
Smittia paranudipennis|BIOUG15762-C09|Kate Perez|BIN Taxonomy Match|BOLD:ACK3746
Smittia paranudipennis|BIOUG15718-G02|Kate Perez|BIN Taxonomy Match|BOLD:ACK3746
Smittia|ZSM34343-B02|Sofia Wiedenbrug|Morphology|BOLD:AAP5942
Smittia sp. 10ES|BIOUG15797-E03|Elisabeth Stur|BIN Taxonomy Match|BOLD:AAP5942
Smittia sp. 10ES|BIOUG15808-H06|Elisabeth Stur|BIN Taxonomy Match|BOLD:AAP5942
Smittia sp. 10ES|BIOUG15853-E10|Elisabeth Stur|BIN Taxonomy Match|BOLD:AAP5942
Smittia sp. 10ES|BIOUG15800-A11|Elisabeth Stur|BIN Taxonomy Match|BOLD:AAP5942
Smittia sp. 10ES|Finnmark162|Elisabeth Stur||BOLD:AAP5942
Smittia sp. 10ES|Finnmark160|Elisabeth Stur||BOLD:AAP5942
Smittia sp. 10ES|Finnmark615|Elisabeth Stur||BOLD:AAP5942
Smittia sp. 10ES|CCDB22605-C05|Sofia Wiedenbrug||BOLD:AAP5942
Smittia|ZSM-DIP-33170-A06|Caroline Chimeno|BIN Taxonomy Match (Jan 2022)|BOLD:AAP5942
Smittia|ZSM-DIP-33170-A04|Caroline Chimeno|BIN Taxonomy Match (Jan 2022)|BOLD:AAP5942
Smittia|ZSM-DIP-33146-G12|Caroline Chimeno|BIN Taxonomy Match (Jan 2022)|BOLD:AAP5942
Smittia|GBOL11507|Sofia Wiedenbrug||BOLD:AAP5942
Smittia|ZSM-DIP-33172-F07|Caroline Chimeno|BIN Taxonomy Match (Jan 2022)|BOLD:AAP5942
Smittia|ZSM-DIP-33172-F08|Caroline Chimeno|BIN Taxonomy Match (Jan 2022)|BOLD:AAP5942
Smittia|ZSM-DIP-33170-A05|Caroline Chimeno|BIN Taxonomy Match (Jan 2022)|BOLD:AAP5942
Smittia|ZSM-DIP-33145-B04|Caroline Chimeno|BIN Taxonomy Match (Jan 2022)|BOLD:AAP5942
Smittia|ZSM-DIP-33145-B05|Caroline Chimeno|BIN Taxonomy Match (Jan 2022)|BOLD:AAP5942
Smittia|ZSM-DIP-33145-B03|Caroline Chimeno|BIN Taxonomy Match (Jan 2022)|BOLD:AAP5942
Smittia|ZSM-DIP-33170-A03|Caroline Chimeno|BIN Taxonomy Match (Jan 2022)|BOLD:AAP5942
Smittia|ZSM-DIP-33141-G05|Caroline Chimeno|BIN Taxonomy Match (Jan 2022)|BOLD:AAP5942
Smittia|ZSM-DIP-33146-H03|Caroline Chimeno|BIN Taxonomy Match (Jan 2022)|BOLD:AAP5942
Smittia sp. 10ES|CH-OSF166|Elisabeth Stur||BOLD:AAP5942
Smittia sp. 10ES|Finnmark611|Elisabeth Stur||BOLD:AAP5942
Psectrotanypus varius|BIOUG55199-C08|Stefan Schmidt|BIN Taxonomy Match (Sep 2020)|BOLD:A...
Psectrotanypus varius|CCDB21606-H08|Sofia Wiedenbrug||BOLD:ACK3819
Psectrotanypus varius|ZMUO.024547|Lauri Paasivirta||BOLD:ACK3819
Psectrotanypus varius|CCDB24183-G04|Sofia Wiedenbrug|Morphology|BOLD:ACK3819
Psectrotanypus varius|BIOUG42694-E06|Kate Perez|BIN Taxonomy Match below Phylum (Jun 2019)|...
Psectrotanypus varius|ZMUO.024548|Lauri Paasivirta||BOLD:ACK3819
Psectrotanypus varius|TRD-CH47|Elisabeth Stur||BOLD:ACK3819
Psectrotanypus varius|RIN_CH31|Elisabeth Stur|morphology|BOLD:ACK3819
Psectrotanypus varius|TRD-CH208|Elisabeth Stur||BOLD:ACK3819
Psectrotanypus varius|TRD-CH34|Elisabeth Stur||BOLD:ACK3819
Psectrotanypus varius|TRD-CH33|Elisabeth Stur||BOLD:ACK3819
Chironomidae|BIOUG55192-B01|Kate Perez|BIN Taxonomy Match (Oct 2020)|BOLD:ACN5653
Metriocnemus caudigus|ZMUO.024719|Lauri Paasivirta||BOLD:AEN0348
Metriocnemus caudigus|CCDB24183-C05|Sofia Wiedenbrug|Morphology|BOLD:AAH3792
Metriocnemus caudigus|ES35|Elisabeth Stur||BOLD:AAH3792
Metriocnemus caudigus|ES34|Elisabeth Stur||BOLD:AAH3792
Metriocnemus caudigus|BIOUG05142-E07|Kate Perez|BIN Taxonomy Match|BOLD:AAH3792
Bryophaenocladius flavoscutellatus|BIOUG17178-D09|Kate Perez|BIN Taxonomy Match|BOLD:AAW1344
Bryophaenocladius flavoscutellatus|ZSM34341-C10|Sofia Wiedenbrug|Morphology|BOLD:AAW1344
Bryophaenocladius flavoscutellatus|CCDB24228-D02|Sofia Wiedenbrug|BIN Taxonomy Match|BOLD:AAW1344
Bryophaenocladius flavoscutellatus|BIOUG17119-G06|Kate Perez|BIN Taxonomy Match|BOLD:AAW1344
Bryophaenocladius flavoscutellatus|BIOUG17114-B02|Kate Perez|BIN Taxonomy Match|BOLD:AAW1344
Bryophaenocladius flavoscutellatus|BIOUG17137-D11|Kate Perez|BIN Taxonomy Match|BOLD:AAW1344
Bryophaenocladius flavoscutellatus|CCDB24228-F04|Sofia Wiedenbrug|BIN Taxonomy Match|BOLD:AAW1344
Bryophaenocladius flavoscutellatus|BIOUG17114-D08|Kate Perez|BIN Taxonomy Match|BOLD:AAW1344
Bryophaenocladius flavoscutellatus|BIOUG17120-C03|Kate Perez|BIN Taxonomy Match|BOLD:AAW1344
Bryophaenocladius flavoscutellatus|CCDB24228-F06|Sofia Wiedenbrug|BIN Taxonomy Match|BOLD:AAW1344
Bryophaenocladius flavoscutellatus|BIOUG42709-H01|Kate Perez|BIN Taxonomy Match below Phylum (...
[illegible]

Bryophaenocladius flavoscutellatus|BIOUG17120-C03|Kate Perez|BIN Taxonomy Match|BOLD:AAW1344
Bryophaenocladius flavoscutellatus|CCDB24228-F06|Sofia Wiedenbrug|BIN Taxonomy Match|BOLD:AAW1344
Bryophaenocladius flavoscutellatus|BIOUG42709-H01|Kate Perez|BIN Taxonomy Match below Phylum (...
Bryophaenocladius flavoscutellatus|ZSM-DIP-33132-A04|Caroline Chimeno|BIN Taxonomy Match (Jan ...
Bryophaenocladius flavoscutellatus|BIOUG42669-E03|Kate Perez|BIN Taxonomy Match below Phylum (...
Bryophaenocladius flavoscutellatus|BIOUG42927-A09|Kate Perez|BIN Taxonomy Match below Phylum (...
Bryophaenocladius flavoscutellatus|ZSM-DIP-33138-H08|Caroline Chimeno|BIN Taxonomy Match (Jan ...
Bryophaenocladius flavoscutellatus|BIOUG42678-D09|Kate Perez|BIN Taxonomy Match below Phylum (...
Bryophaenocladius flavoscutellatus|BIOUG55195-F12|Stefan Schmidt|BIN Taxonomy Match (Sep 2020...
Bryophaenocladius flavoscutellatus|BIOUG42713-A09|Kate Perez|BIN Taxonomy Match below Phylum (...
Bryophaenocladius flavoscutellatus|BIOUG42709-G02|Kate Perez|BIN Taxonomy Match below Phylum (...
Bryophaenocladius flavoscutellatus|BIOUG42659-A04|Kate Perez|BIN Taxonomy Match below Phylum (...
Bryophaenocladius flavoscutellatus|BIOUG42657-D08|Kate Perez|BIN Taxonomy Match below Phylum (...
Bryophaenocladius flavoscutellatus|ZSM-DIP-33136-A03|Caroline Chimeno|BIN Taxonomy Match (Jan ...
Bryophaenocladius flavoscutellatus|BIOUG55120-D11|Stefan Schmidt|BIN Taxonomy Match (Sep 2020...
Bryophaenocladius flavoscutellatus|BIOUG42915-E04|Kate Perez|BIN Taxonomy Match below Phylum (...
Bryophaenocladius flavoscutellatus|ZSM-DIP-33136-A04|Caroline Chimeno|BIN Taxonomy Match (Jan ...
Bryophaenocladius flavoscutellatus|ZSM-DIP-33136-A05|Caroline Chimeno|BIN Taxonomy Match (Jan ...
Bryophaenocladius flavoscutellatus|ZSM-DIP-33136-A06|Caroline Chimeno|BIN Taxonomy Match (Jan ...
Bryophaenocladius flavoscutellatus|ZSM-DIP-33147-H07|Caroline Chimeno|BIN Taxonomy Match (Jan ...
Bryophaenocladius flavoscutellatus|ZSM-DIP-33147-H08|Caroline Chimeno|BIN Taxonomy Match (Jan ...
Bryophaenocladius flavoscutellatus|ZSM-DIP-33147-H09|Caroline Chimeno|BIN Taxonomy Match (Jan ...
Bryophaenocladius flavoscutellatus|ZSM-DIP-33138-F08|Caroline Chimeno|BIN Taxonomy Match (Jan ...
Bryophaenocladius flavoscutellatus|BIOUG42741-E03|Kate Perez|BIN Taxonomy Match below Phylum (...
Bryophaenocladius flavoscutellatus|BIOUG42744-G11|Kate Perez|BIN Taxonomy Match below Phylum (...
Bryophaenocladius flavoscutellatus|BIOUG42702-F12|Kate Perez|BIN Taxonomy Match below Phylum (...
Bryophaenocladius flavoscutellatus|BIOUG42745-G10|Kate Perez|BIN Taxonomy Match below Phylum (...
Bryophaenocladius flavoscutellatus|BIOUG42660-C12|Kate Perez|BIN Taxonomy Match below Phylum (...
Bryophaenocladius flavoscutellatus|BIOUG42764-H07|Kate Perez|BIN Taxonomy Match below Phylum (...
Bryophaenocladius flavoscutellatus|BIOUG42926-A10|Kate Perez|BIN Taxonomy Match below Phylum (...
Bryophaenocladius flavoscutellatus|ZSM34341-D02|Sofia Wiedenbrug|Morphology|BOLD:AAW1344
Bryophaenocladius flavoscutellatus|BIOUG17117-F07|Kate Perez|BIN Taxonomy Match|BOLD:AAW1344
Bryophaenocladius flavoscutellatus|BIOUG17114-F02|Kate Perez|BIN Taxonomy Match|BOLD:AAW1344
Bryophaenocladius flavoscutellatus|ZSM34341-D05|Sofia Wiedenbrug|Morphology|BOLD:AAW1344
Bryophaenocladius flavoscutellatus|BIOUG17095-E04|Kate Perez|BIN Taxonomy Match|BOLD:AAW1344
Bryophaenocladius flavoscutellatus|BIOUG17095-E02|Kate Perez|BIN Taxonomy Match|BOLD:AAW1344
Bryophaenocladius flavoscutellatus|ZSM34341-C12|Sofia Wiedenbrug|Morphology|BOLD:AAW1344
Bryophaenocladius flavoscutellatus|BIOUG17225-G05|Kate Perez|BIN Taxonomy Match|BOLD:AAW1344
Bryophaenocladius flavoscutellatus|ZSM-DIP-33129-D03|Caroline Chimeno|BIN Taxonomy Match (Jan ...
Bryophaenocladius flavoscutellatus|BIOUG42663-B06|Kate Perez|BIN Taxonomy Match below Phylum (...
Bryophaenocladius flavoscutellatus|BIOUG42741-H05|Kate Perez|BIN Taxonomy Match below Phylum (...
Bryophaenocladius flavoscutellatus|ZSM-DIP-33132-B09|Caroline Chimeno|BIN Taxonomy Match (Jan ...
Bryophaenocladius flavoscutellatus|ZSM-DIP-33132-B11|Caroline Chimeno|BIN Taxonomy Match (Jan ...
Bryophaenocladius flavoscutellatus|ZSM-DIP-33138-H02|Caroline Chimeno|BIN Taxonomy Match (Jan ...
Bryophaenocladius flavoscutellatus|ZSM-DIP-33129-B12|Caroline Chimeno|BIN Taxonomy Match (Jan ...
Bryophaenocladius flavoscutellatus|ZSM-DIP-33129-D02|Caroline Chimeno|BIN Taxonomy Match (Jan ...
Bryophaenocladius flavoscutellatus|BIOUG42655-C12|Kate Perez|BIN Taxonomy Match below Phylum (...
Bryophaenocladius flavoscutellatus|BIOUG42654-E08|Kate Perez|BIN Taxonomy Match below Phylum (...
Bryophaenocladius flavoscutellatus|BIOUG42764-F02|Kate Perez|BIN Taxonomy Match below Phylum (...
Bryophaenocladius flavoscutellatus|BIOUG17096-E05|Kate Perez|BIN Taxonomy Match|BOLD:AAW1344
Bryophaenocladius flavoscutellatus|CH-OSF83|Elisabeth Stur||BOLD:AAW1344
Cricotopus magus|Finnmark797|G. A. Halvorsen||BOLD:ACD1875
Cricotopus magus|Finnmark795|G. A. Halvorsen||BOLD:ACD1875
Smittia|ZSM34343-G06|Sofia Wiedenbrug|Morphology
Smittia sp. 27ES|24371-A12|Kate Perez|BIN Taxonomy Match|BOLD:ACA0411
Smittia sp. 27ES|24354-C01|Kate Perez|BIN Taxonomy Match|BOLD:ACA0411
Smittia|ZFMK-TIS-32896|Niklas Beckers||BOLD:ACA0411
Smittia sp. 27ES|24336-D08|Kate Perez|BIN Taxonomy Match|BOLD:ACA0411
Smittia sp. 27ES|24512-A11|Kate Perez|BIN Taxonomy Match|BOLD:ACA0411
Smittia sp. 27ES|24373-G05|Kate Perez|BIN Taxonomy Match|BOLD:ACA0411
Smittia sp. 27ES|24373-D02|Kate Perez|BIN Taxonomy Match|BOLD:ACA0411
Smittia|CCDB22603-E02|Sofia Wiedenbrug||BOLD:ACQ2283
Smittia|24354-F07|Kate Perez|BIN Taxonomy Match|BOLD:ACO4167
Smittia|24465-F03|Kate Perez|BIN Taxonomy Match|BOLD:ACO4167
Smittia|24418-E09|Kate Perez|BIN Taxonomy Match|BOLD:ACO4167
Smittia|25132-F09|Kate Perez|BOLD ID Engine Manual (Apr 2018)|BOLD:ACO4167
Smittia|ES384|Elisabeth Stur||BOLD:ACO4167
Chironomidae|CCDB22605-A04|Sofia Wiedenbrug|no morphological identification - introduction of int...
Smittia|24372-E09|Kate Perez|BIN Taxonomy Match|BOLD:AAG3901
Smittia|SOE317|Elisabeth Stur||BOLD:AAG3901
Smittia|SOE316|Elisabeth Stur||BOLD:AAG3901
Orthocladiinae|24465-B11|Kate Perez|BIN Taxonomy Match|BOLD:AAU6766
Orthocladiinae|23878-H02|Kate Perez|BIN Taxonomy Match|BOLD:AAU6766
Smittia sp. 9ES|BIOUG15539-F10|Elisabeth Stur|BIN Taxonomy Match|BOLD:AAM7061
Smittia sp. 9ES|BIOUG15457-D03|Elisabeth Stur|BIN Taxonomy Match|BOLD:AAM7061
Smittia sp. 9ES|24474-A04|Kate Perez|BIN Taxonomy Match|BOLD:AAM7061
Smittia sp. 9ES|BIOUG15454-F01|Elisabeth Stur|BIN Taxonomy Match|BOLD:AAM7061
Smittia sp. 9ES|BIOUG15455-D03|Elisabeth Stur|BIN Taxonomy Match|BOLD:AAM7061
Smittia sp. 9ES|BIOUG15456-A05|Elisabeth Stur|BIN Taxonomy Match|BOLD:AAM7061
Smittia sp. 9ES|BIOUG15184-H08|Elisabeth Stur|BIN Taxonomy Match|BOLD:AAM7061
Smittia sp. 9ES|BIOUG15455-F07|Elisabeth Stur|BIN Taxonomy Match|BOLD:AAM7061
Smittia sp. 9ES|BIOUG15185-B12|Elisabeth Stur|BIN Taxonomy Match|BOLD:AAM7061
Smittia sp. 9ES|BIOUG15561-E01|Elisabeth Stur|BIN Taxonomy Match|BOLD:AAM7061
Smittia sp. 9ES|BIOUG15539-B10|Elisabeth Stur|BIN Taxonomy Match|BOLD:AAM7061
Smittia sp. 9ES|BIOUG15537-E04|Elisabeth Stur|BIN Taxonomy Match|BOLD:AAM7061
Smittia sp. 9ES|Finnmark98|Elisabeth Stur||BOLD:AAM7061
Smittia sp. 9ES|Finnmark552|Elisabeth Stur
Psectrocladius fennicus|ZMUO.026284|Lauri Paasivirta||BOLD:ADC5003
Psectrocladius fennicus|ZMUO.026283|Lauri Paasivirta||BOLD:ADC5003
Psectrocladius sp. 1SW|CCDB24183-C10|Sofia Wiedenbrug|Morphology|BOLD:ADC5003
Cricotopus tibialis|ZMUO.025183|Lauri Paasivirta||BOLD:AAB7294
Cricotopus tibialis|ZMUO.025083|Lauri Paasivirta||BOLD:AAB7294
Cricotopus tibialis|BIOUG43946-C09|Kate Perez|BIN Taxonomy Match below Phylum (Jan 2019)|BO...
Cricotopus tibialis|ZMUO.025182|Lauri Paasivirta||BOLD:AAB7294
Cricotopus tibialis|ZMUO.025082|Lauri Paasivirta||BOLD:AAB7294
Cricotopus|ZMUO.025927|Lauri Paasivirta||BOLD:AAB7294
Cricotopus tibialis|ATNA288|Elisabeth Stur||BOLD:AAB7294
Cricotopus tibialis|ATNA258|Elisabeth Stur||BOLD:AAB7294
Cricotopus tibialis|ES375|Elisabeth Stur||BOLD:AAB7294
Cricotopus tibialis|ATNA303|Elisabeth Stur||BOLD:AAB7294
Cricotopus tibialis|CH-eik55|Elisabeth Stur||BOLD:AAB7294
Cricotopus tibialis|TRD-CH302|Elisabeth Stur||BOLD:AAB7294
Cricotopus tibialis|ATNA270|Elisabeth Stur||BOLD:AAB7294

Cricotopus tibialis|CH-eik55|Elisabeth Stur||BOLD:AAB7294
Cricotopus tibialis|TRD-CH302|Elisabeth Stur||BOLD:AAB7294
Cricotopus tibialis|ATNA270|Elisabeth Stur||BOLD:AAB7294
Cricotopus tibialis|NO57|Elisabeth Stur||BOLD:AAB7294
Cricotopus tibialis|Finnmark513|Elisabeth Stur||BOLD:AAB7294
Cricotopus villosus|Finnmark474|Elisabeth Stur|Microscope|BOLD:ABA8476
Cricotopus villosus|Finnmark469|Elisabeth Stur|Microscope|BOLD:ABA8476
Cricotopus sp. 26ES|Finnmark255|Elisabeth Stur|Microscope|BOLD:ABX2654
Cricotopus pilosellus|Finnmark133|Torbjorn Ekrem||BOLD:AAM6022
Cricotopus pilosellus|TRD-CH245|Elisabeth Stur||BOLD:AAG1016
Cricotopus pilosellus|ZMUO.024620|Lauri Paasivirta||BOLD:AAG1016
Cricotopus pilosellus|ZMUO.029777|Marko Mutanen||BOLD:AAG1016
Cricotopus polaris|ZMUO.024759|Lauri Paasivirta||BOLD:AAG1016
Cricotopus pilosellus|ZMUO.024619|Lauri Paasivirta||BOLD:AAG1016
Cricotopus polaris|ZMUO.024760|Lauri Paasivirta||BOLD:AAG1016
Cricotopus pilosellus|Finnmark167|Elisabeth Stur||BOLD:AAG1016
Cricotopus pilosellus|Finnmark473|Elisabeth Stur||BOLD:AAG1016
Cricotopus pilosellus|Finnmark603|Elisabeth Stur||BOLD:AAG1016
Cricotopus sp. 2ES|ATNA271|Elisabeth Stur||BOLD:AAI6025
Cricotopus sp. 2ES|Finnmark85|Elisabeth Stur||BOLD:AAI6025
Cricotopus sp. 2ES|Finnmark341|Elisabeth Stur||BOLD:AAI6025
Cricotopus polaris|BIOUG15948-G01|Kate Perez|BIN Taxonomy Match|BOLD:ABA3997
Cricotopus polaris|Finnmark723|Elisabeth Stur
Cricotopus|TRD-CH143|Elisabeth Stur|morphology|BOLD:ABA3997
Cricotopus polaris|Finnmark127|Elisabeth Stur||BOLD:ABA3997
Cricotopus polaris|NO59|Elisabeth Stur||BOLD:ABA3997
Cricotopus|TRD-CH146|Elisabeth Stur|morphology|BOLD:ABA3997
Cricotopus|TRD-CH147|Elisabeth Stur|morphology|BOLD:ABA3997
Cricotopus polaris|BIOUG16256-B10|Kate Perez|BIN Taxonomy Match|BOLD:ABA3997
Cricotopus polaris|BIOUG16256-F05|Kate Perez|BIN Taxonomy Match|BOLD:ABA3997
Cricotopus|TRD-CH174|Elisabeth Stur||BOLD:ABA3997
Cricotopus polaris|NO58|Elisabeth Stur||BOLD:ABA3997
Cricotopus polaris|Finnmark471|Susan E. Gresens||BOLD:AAV1706
Cricotopus polaris|Finnmark477|Elisabeth Stur||BOLD:AAV1706
Cricotopus polaris|Finnmark511|Elisabeth Stur
Cricotopus polaris|Finnmark470|Elisabeth Stur
Cricotopus|TRD-CH145|Elisabeth Stur|morphology|BOLD:ACR2284
Cricotopus|TRD-CH144|Elisabeth Stur|morphology|BOLD:ACR2284
Cricotopus polaris|Finnmark581|Elisabeth Stur||BOLD:ACG9064
Chironomidae|24415-C01|Mikko J Tiusanen|Tree based identification|BOLD:ACU4101
Georthocladius|Koz1236|Renee Miskie|BIN Taxonomy Match (May 2019)|BOLD:ACD9509
Georthocladius|BIOUG05104-F02|Kate Perez|BIN Taxonomy Match|BOLD:ACD9509
Georthocladius|BIOUG05141-F12|Kate Perez|BIN Taxonomy Match|BOLD:ACD9509
Georthocladius|BIOUG05108-A11|Kate Perez|BIN Taxonomy Match|BOLD:ACD9509
Georthocladius|BIOUG05101-F05|Kate Perez|BIN Taxonomy Match|BOLD:ACD9509
Georthocladius|BIOUG05105-D06|Kate Perez|BIN Taxonomy Match|BOLD:ACD9509
Georthocladius|BIOUG05105-B10|Kate Perez|BIN Taxonomy Match|BOLD:ACD9509
Georthocladius|BIOUG05104-D02|Kate Perez|BIN Taxonomy Match|BOLD:ACD9509
Georthocladius|BIOUG05103-A08|Kate Perez|BIN Taxonomy Match|BOLD:ACD9509
Georthocladius|BIOUG05143-G08|Kate Perez|BIN Taxonomy Match|BOLD:ACD9509
Georthocladius|BIOUG05101-F04|Kate Perez|BIN Taxonomy Match|BOLD:ACD9509
Georthocladius|BIOUG05105-F01|Kate Perez|BIN Taxonomy Match|BOLD:ACD9509
Georthocladius|BIOUG05141-C11|Kate Perez|BIN Taxonomy Match|BOLD:ACD9509
Georthocladius|BIOUG05102-A01|Kate Perez|BIN Taxonomy Match|BOLD:ACD9509
Georthocladius|BIOUG05101-B07|Kate Perez|BIN Taxonomy Match|BOLD:ACD9509
Georthocladius|BIOUG05141-B07|Kate Perez|BIN Taxonomy Match|BOLD:ACD9509
Georthocladius|BIOUG05104-B06|Kate Perez|BIN Taxonomy Match|BOLD:ACD9509
Georthocladius|BIOUG05102-H01|Kate Perez|BIN Taxonomy Match|BOLD:ACD9509
Georthocladius|BIOUG05102-A03|Kate Perez|BIN Taxonomy Match|BOLD:ACD9509
Georthocladius|BIOUG04795-H07|Kate Perez|BIN Taxonomy Match|BOLD:ACD9509
Georthocladius|BIOUG05106-H05|Kate Perez|BIN Taxonomy Match|BOLD:ACD9509
Georthocladius|BIOUG05106-D04|Kate Perez|BIN Taxonomy Match|BOLD:ACD9509
Chironomidae|BIOUG05109-C10|Meredith Miller|Tree-based Identification (Feb 2019)
Georthocladius|BIOUG05108-G09|Kate Perez|BIN Taxonomy Match|BOLD:ACD9509
Georthocladius|BIOUG05109-B08|Kate Perez|BIN Taxonomy Match|BOLD:ACD9509
Georthocladius|BIOUG05109-H04|Kate Perez|BIN Taxonomy Match|BOLD:ACD9509
Georthocladius|BIOUG05103-A10|Kate Perez|BIN Taxonomy Match|BOLD:ACD9509
Georthocladius|BIOUG05109-C06|Kate Perez|BIN Taxonomy Match|BOLD:ACD9509
Georthocladius|CCDB24183-A06|Sofia Wiedenbrug|Morphology|BOLD:ACD9509
Georthocladius|BIOUG05142-G05|Kate Perez|BIN Taxonomy Match|BOLD:ACD9509
Georthocladius|BIOUG05143-H02|Kate Perez|BIN Taxonomy Match|BOLD:ACD9509
Georthocladius|ZMUO.024531|Lauri Paasivirta||BOLD:ACD9509
Georthocladius|ZMUO.024530|Lauri Paasivirta||BOLD:ACD9509
Georthocladius|BIOUG05103-B03|Kate Perez|BIN Taxonomy Match|BOLD:ACD9509
Georthocladius|BIOUG05109-D01|Kate Perez|BIN Taxonomy Match|BOLD:ACD9509
Georthocladius|BIOUG05103-A07|Kate Perez|BIN Taxonomy Match|BOLD:ACD9509
Georthocladius|BIOUG05109-D12|Kate Perez|BIN Taxonomy Match|BOLD:ACD9509
Georthocladius|BIOUG04795-C04|Kate Perez|BIN Taxonomy Match|BOLD:ACD9509
Georthocladius|BIOUG05103-A12|Kate Perez|BIN Taxonomy Match|BOLD:ACD9509
Georthocladius|BIOUG05209-H07|Kate Perez|BIN Taxonomy Match|BOLD:ACD9509
Georthocladius|BIOUG04795-C03|Kate Perez|BIN Taxonomy Match|BOLD:ACD9509
Georthocladius|BIOUG05142-A11|Kate Perez|BIN Taxonomy Match|BOLD:ACD9509
Georthocladius|BIOUG05105-F08|Kate Perez|BIN Taxonomy Match|BOLD:ACD9509
Georthocladius|BIOUG05141-A12|Kate Perez|BIN Taxonomy Match|BOLD:ACD9509
Georthocladius|BIOUG05108-C09|Kate Perez|BIN Taxonomy Match|BOLD:ACD9509
Georthocladius|BIOUG05141-D05|Kate Perez|BIN Taxonomy Match|BOLD:ACD9509
Georthocladius|BIOUG05108-C06|Kate Perez|BIN Taxonomy Match|BOLD:ACD9509
Georthocladius|BIOUG05108-D08|Kate Perez|BIN Taxonomy Match|BOLD:ACD9509
Georthocladius|BIOUG05109-E11|Kate Perez|BIN Taxonomy Match|BOLD:ACD9509
Georthocladius|BIOUG05141-C03|Kate Perez|BIN Taxonomy Match|BOLD:ACD9509
Georthocladius|BIOUG05108-B01|Kate Perez|BIN Taxonomy Match|BOLD:ACD9509
Georthocladius|BIOUG05108-G02|Kate Perez|BIN Taxonomy Match|BOLD:ACD9509
Georthocladius|BIOUG05109-A01|Kate Perez|BIN Taxonomy Match|BOLD:ACD9509
Georthocladius|BIOUG05109-C05|Kate Perez|BIN Taxonomy Match|BOLD:ACD9509
Georthocladius|BIOUG05106-C10|Kate Perez|BIN Taxonomy Match|BOLD:ACD9509
Georthocladius|BIOUG05107-F07|Kate Perez|BIN Taxonomy Match|BOLD:ACD9509
Georthocladius|BIOUG05107-E11|Kate Perez|BIN Taxonomy Match|BOLD:ACD9509
Georthocladius|BIOUG05107-H02|Kate Perez|BIN Taxonomy Match|BOLD:ACD9509
Georthocladius|BIOUG05102-H06|Kate Perez|BIN Taxonomy Match|BOLD:ACD9509
Georthocladius|BIOUG05108-F05|Kate Perez|BIN Taxonomy Match|BOLD:ACD9509
Georthocladius|BIOUG05109-A09|Kate Perez|BIN Taxonomy Match|BOLD:ACD9509
Georthocladius|BIOUG05109-C09|Kate Perez|BIN Taxonomy Match|BOLD:ACD9509
Georthocladius|BIOUG05109-G11|Kate Perez|BIN Taxonomy Match|BOLD:ACD9509

Georthocladius|BIOUG05109-A09|Kate Perez|BIN Taxonomy Match|BOLD:ACD9509
Georthocladius|BIOUG05109-C09|Kate Perez|BIN Taxonomy Match|BOLD:ACD9509
Georthocladius|BIOUG05109-G11|Kate Perez|BIN Taxonomy Match|BOLD:ACD9509
Georthocladius|BIOUG05141-A04|Kate Perez|BIN Taxonomy Match|BOLD:ACD9509
Georthocladius|BIOUG05105-C01|Kate Perez|BIN Taxonomy Match|BOLD:ACD9509
Georthocladius|BIOUG05143-G02|Kate Perez|BIN Taxonomy Match|BOLD:ACD9509
Georthocladius|BIOUG05143-E05|Kate Perez|BIN Taxonomy Match|BOLD:ACD9509
Georthocladius|BIOUG05110-B03|Kate Perez|BIN Taxonomy Match|BOLD:ACD9509
Georthocladius|BIOUG05110-A10|Kate Perez|BIN Taxonomy Match|BOLD:ACD9509
Georthocladius|BIOUG05110-C04|Kate Perez|BIN Taxonomy Match|BOLD:ACD9509
Georthocladius|BIOUG05110-H11|Kate Perez|BIN Taxonomy Match|BOLD:ACD9509
Georthocladius|BIOUG04795-B12|Kate Perez|BIN Taxonomy Match|BOLD:ACD9509
Georthocladius|BIOUG05104-A05|Kate Perez|BIN Taxonomy Match|BOLD:ACD9509
Georthocladius|BIOUG05142-D12|Kate Perez|BIN Taxonomy Match|BOLD:ACD9509
Georthocladius|BIOUG05107-G01|Kate Perez|BIN Taxonomy Match|BOLD:ACD9509
Georthocladius|BIOUG05104-G03|Kate Perez|BIN Taxonomy Match|BOLD:ACD9509
Georthocladius|BIOUG05104-E02|Kate Perez|BIN Taxonomy Match|BOLD:ACD9509
Georthocladius|BIOUG05104-D11|Kate Perez|BIN Taxonomy Match|BOLD:ACD9509
Georthocladius|BIOUG05104-D07|Kate Perez|BIN Taxonomy Match|BOLD:ACD9509
Georthocladius|BIOUG05104-B12|Kate Perez|BIN Taxonomy Match|BOLD:ACD9509
Georthocladius|BIOUG05104-A07|Kate Perez|BIN Taxonomy Match|BOLD:ACD9509
Georthocladius|BIOUG04795-D06|Kate Perez|BIN Taxonomy Match|BOLD:ACD9509
Georthocladius|BIOUG04795-C08|Kate Perez|BIN Taxonomy Match|BOLD:ACD9509
Georthocladius|BIOUG05105-D10|Kate Perez|BIN Taxonomy Match|BOLD:ACD9509
Georthocladius|BIOUG05105-D02|Kate Perez|BIN Taxonomy Match|BOLD:ACD9509
Georthocladius|BIOUG05105-C05|Kate Perez|BIN Taxonomy Match|BOLD:ACD9509
Georthocladius|BIOUG05105-B07|Kate Perez|BIN Taxonomy Match|BOLD:ACD9509
Georthocladius|BIOUG05103-H08|Kate Perez|BIN Taxonomy Match|BOLD:ACD9509
Georthocladius|BIOUG05103-D03|Kate Perez|BIN Taxonomy Match|BOLD:ACD9509
Georthocladius|BIOUG05102-H11|Kate Perez|BIN Taxonomy Match|BOLD:ACD9509
Georthocladius|BIOUG05102-E05|Kate Perez|BIN Taxonomy Match|BOLD:ACD9509
Georthocladius|BIOUG05102-E03|Kate Perez|BIN Taxonomy Match|BOLD:ACD9509
Georthocladius|BIOUG05101-F07|Kate Perez|BIN Taxonomy Match|BOLD:ACD9509
Georthocladius|BIOUG05101-E11|Kate Perez|BIN Taxonomy Match|BOLD:ACD9509
Georthocladius|BIOUG05101-E04|Kate Perez|BIN Taxonomy Match|BOLD:ACD9509
Georthocladius|BIOUG05101-A01|Kate Perez|BIN Taxonomy Match|BOLD:ACD9509
Georthocladius|BIOUG04795-H11|Kate Perez|BIN Taxonomy Match|BOLD:ACD9509
Georthocladius|BIOUG05105-B02|Kate Perez|BIN Taxonomy Match|BOLD:ACD9509
Georthocladius|BIOUG05103-H10|Kate Perez|BIN Taxonomy Match|BOLD:ACD9509
Georthocladius|BIOUG04795-H05|Kate Perez|BIN Taxonomy Match|BOLD:ACD9509
Georthocladius|BIOUG04795-D12|Kate Perez|BIN Taxonomy Match|BOLD:ACD9509
Georthocladius|BIOUG04795-D09|Kate Perez|BIN Taxonomy Match|BOLD:ACD9509
Georthocladius|BIOUG05107-D04|Kate Perez|BIN Taxonomy Match|BOLD:ACD9509
Georthocladius|BIOUG05107-C12|Kate Perez|BIN Taxonomy Match|BOLD:ACD9509
Georthocladius|BIOUG05107-A05|Kate Perez|BIN Taxonomy Match|BOLD:ACD9509
Georthocladius|BIOUG05106-H02|Kate Perez|BIN Taxonomy Match|BOLD:ACD9509
Georthocladius|BIOUG05106-G06|Kate Perez|BIN Taxonomy Match|BOLD:ACD9509
Georthocladius|BIOUG05106-F06|Kate Perez|BIN Taxonomy Match|BOLD:ACD9509
Georthocladius|BIOUG05106-C09|Kate Perez|BIN Taxonomy Match|BOLD:ACD9509
Georthocladius|BIOUG05106-A11|Kate Perez|BIN Taxonomy Match|BOLD:ACD9509
Georthocladius|BIOUG05105-H06|Kate Perez|BIN Taxonomy Match|BOLD:ACD9509
Georthocladius|BIOUG05107-D12|Kate Perez|BIN Taxonomy Match|BOLD:ACD9509
Georthocladius|BIOUG05107-D09|Kate Perez|BIN Taxonomy Match|BOLD:ACD9509
Georthocladius|BIOUG05105-F11|Kate Perez|BIN Taxonomy Match|BOLD:ACD9509
Georthocladius|BIOUG05102-C07|Kate Perez|BIN Taxonomy Match|BOLD:ACD9509
Rheocricotopus atripes|ZSM-DIP-33137-B10|Caroline Chimeno|BIN Taxonomy Match (Jan 2022)|BOL...
Rheocricotopus atripes|CCDB21605-F02|Sofia Wiedenbrug||BOLD:AAD5902
Rheocricotopus atripes|ZSM-DIP-33141-H03|Caroline Chimeno|BIN Taxonomy Match (Jan 2022)|BOL...
Rheocricotopus atripes|ZSM-DIP-33137-B09|Caroline Chimeno|BIN Taxonomy Match (Jan 2022)|BOL...
Rheocricotopus atripes|ZSM-DIP-33139-D07|Caroline Chimeno|BIN Taxonomy Match (Jan 2022)|BOL...
Rheocricotopus atripes|ZSM-DIP-33139-D09|Caroline Chimeno|BIN Taxonomy Match (Jan 2022)|BOL...
Rheocricotopus atripes|ZSM-DIP-33139-H05|Caroline Chimeno|BIN Taxonomy Match (Jan 2022)|BOL...
Rheocricotopus atripes|GBOL11520|Sofia Wiedenbrug|Morphology|BOLD:AAD5902
Rheocricotopus atripes|GBOL11519|Sofia Wiedenbrug|Morphology|BOLD:AAD5902
Rheocricotopus atripes|CCDB21605-B06|Sofia Wiedenbrug||BOLD:AAD5902
Rheocricotopus atripes|CCDB21605-B08|Sofia Wiedenbrug||BOLD:AAD5902
Rheocricotopus atripes|GBOL11518|Sofia Wiedenbrug|Morphology|BOLD:AAD5902
Rheocricotopus atripes|ZSM-DIP-33131-G02|Caroline Chimeno|BIN Taxonomy Match (Jan 2022)|BOL...
Rheocricotopus atripes|ZSM-DIP-33137-A05|Caroline Chimeno|BIN Taxonomy Match (Jan 2022)|BOL...
Rheocricotopus atripes|ZSM-DIP-33148-A03|Caroline Chimeno|BIN Taxonomy Match (Jan 2022)|BOL...
Rheocricotopus atripes|ZSM-DIP-33139-D05|Caroline Chimeno|BIN Taxonomy Match (Jan 2022)|BOL...
Rheocricotopus atripes|ZSM-DIP-33139-D06|Caroline Chimeno|BIN Taxonomy Match (Jan 2022)|BOL...
Rheocricotopus atripes|ZSM-DIP-33139-E08|Caroline Chimeno|BIN Taxonomy Match (Jan 2022)|BOL...
Rheocricotopus atripes|ZSM-DIP-33140-C12|Caroline Chimeno|BIN Taxonomy Match (Jan 2022)|BOL...
Rheocricotopus atripes|ZSM-DIP-33140-D01|Caroline Chimeno|BIN Taxonomy Match (Jan 2022)|BOL...
Rheocricotopus atripes|GBOL11522|Sofia Wiedenbrug|BIN-taxonomy|BOLD:AAD5902
Rheocricotopus atripes|ZSM-DIP-33139-D08|Caroline Chimeno|BIN Taxonomy Match (Jan 2022)|BOL...
Rheocricotopus atripes|ZSM-DIP-33137-B07|Caroline Chimeno|BIN Taxonomy Match (Jan 2022)|BOL...
Rheocricotopus atripes|ZSM-DIP-33148-A04|Caroline Chimeno|BIN Taxonomy Match (Jan 2022)|BOL...
Rheocricotopus atripes|ZSM-DIP-33140-C01|Caroline Chimeno|BIN Taxonomy Match (Jan 2022)
Rheocricotopus atripes|ZSM-DIP-33137-B08|Caroline Chimeno|BIN Taxonomy Match (Jan 2022)|BOL...
Rheocricotopus atripes|GBOL03745|Sofia Wiedenbrug||BOLD:AAD5902
Rheocricotopus atripes|CH-OSF154|Elisabeth Stur||BOLD:AAD5902
Rheocricotopus atripes|ZMUO.024838|Lauri Paasivirta||BOLD:AAD5902
Rheocricotopus atripes|ZMUO.024837|Lauri Paasivirta||BOLD:AAD5902
Rheocricotopus atripes|SOE98|Elisabeth Stur||BOLD:AAD5902
Rheocricotopus atripes|SOE134|Elisabeth Stur||BOLD:AAD5902
Rheocricotopus atripes|SOE259|Elisabeth Stur||BOLD:AAD5902
Rheocricotopus atripes|SOE219|Elisabeth Stur||BOLD:AAD5902
Rheocricotopus atripes|SOE176|Elisabeth Stur||BOLD:AAD5902
Rheocricotopus atripes|Finnmark607|Elisabeth Stur||BOLD:AAD5902
Nanocladius balticus|ZMUO.025301|Lauri Paasivirta||BOLD:ADA3217
Nanocladius nr. balticus|ZMUO.024639|Lauri Paasivirta||BOLD:ADA3217
Orthocladiinae|BIOUG07760-F05|Kate Perez|BIN Taxonomy Match|BOLD:AAD9250
Pseudorthocladius filiformis|ZSM-DIP-33134-A08|Caroline Chimeno|BIN Taxonomy Match (Jan 2022 ...
Pseudorthocladius filiformis|ZSM-DIP-33148-A08|Caroline Chimeno|BIN Taxonomy Match (Jan 2022 ...
Pseudorthocladius filiformis|ZSM-DIP-33134-A09|Caroline Chimeno|BIN Taxonomy Match (Jan 2022 ...
Pseudorthocladius filiformis|ZSM-DIP-33134-A03|Caroline Chimeno|BIN Taxonomy Match (Jan 2022 ...
Pseudorthocladius filiformis|ZSM-DIP-33134-A10|Caroline Chimeno|BIN Taxonomy Match (Jan 2022 ...
Pseudorthocladius filiformis|ZSM-DIP-33148-A10|Caroline Chimeno|BIN Taxonomy Match (Jan 2022 ...
Pseudorthocladius filiformis|ZSM-DIP-33148-A11|Caroline Chimeno|BIN Taxonomy Match (Jan 2022 ...
Pseudorthocladius filiformis|ZSM-DIP-33170-F04|Caroline Chimeno|BIN Taxonomy Match (Jan 2022 ...
[illegible]

Pseudorthocladius filiformis|ZSM-DIP-33148-A10|Caroline Chimeno|BIN Taxonomy Match (Jan 2022 ...
Pseudorthocladius filiformis|ZSM-DIP-33148-A11|Caroline Chimeno|BIN Taxonomy Match (Jan 2022 ...
Pseudorthocladius filiformis|ZSM-DIP-33170-F04|Caroline Chimeno|BIN Taxonomy Match (Jan 2022 ...
Pseudorthocladius filiformis|ZSM-DIP-33170-F07|Caroline Chimeno|BIN Taxonomy Match (Jan 2022 ...
Pseudorthocladius filiformis|ZSM-DIP-33170-G08|Caroline Chimeno|BIN Taxonomy Match (Jan 2022 ...
Pseudorthocladius filiformis|ZSM-DIP-33170-G09|Caroline Chimeno|BIN Taxonomy Match (Jan 2022 ...
Pseudorthocladius filiformis|ZSM-DIP-33170-G10|Caroline Chimeno|BIN Taxonomy Match (Jan 2022 ...
Pseudorthocladius filiformis|ZSM-DIP-33170-H07|Caroline Chimeno|BIN Taxonomy Match (Jan 2022 ...
Pseudorthocladius filiformis|ZSM-DIP-33170-H09|Caroline Chimeno|BIN Taxonomy Match (Jan 2022 ...
Orthocladiinae|BIOUG07757-B01|Kate Perez|BIN Taxonomy Match|BOLD:AAD9250
Pseudorthocladius filiformis|ZSM-DIP-33148-A06|Caroline Chimeno|BIN Taxonomy Match (Jan 2022 ...
Orthocladiinae|BIOUG07565-D04|Kate Perez|BIN Taxonomy Match|BOLD:AAD9250
Orthocladiinae|BIOUG07566-G11|Kate Perez|BIN Taxonomy Match|BOLD:AAD9250
Pseudorthocladius filiformis|ZSM-DIP-33148-A05|Caroline Chimeno|BIN Taxonomy Match (Jan 2022 ...
Pseudorthocladius filiformis|ZSM-DIP-33148-H01|Caroline Chimeno|BIN Taxonomy Match (Jan 2022 ...
Pseudorthocladius filiformis|BIOUG07415-B12|Kate Perez|BIN Taxonomy Match (May 2020)|BOLD ...
Pseudorthocladius filiformis|SOE97|Elisabeth Stur||BOLD:AAD9250
Pseudorthocladius filiformis|SOE429|Elisabeth Stur||BOLD:AAD9250
Orthocladiinae|BIOUG07753-G03|Kate Perez|BIN Taxonomy Match|BOLD:AAD9250
Orthocladiinae|BIOUG07755-E05|Kate Perez|BIN Taxonomy Match|BOLD:AAD9250
Orthocladiinae|BIOUG07568-D02|Kate Perez|BIN Taxonomy Match|BOLD:AAD9250
Orthocladiinae|BIOUG07568-F01|Kate Perez|BIN Taxonomy Match|BOLD:AAD9250
Orthocladiinae|BIOUG07807-G09|Kate Perez|BIN Taxonomy Match|BOLD:AAD9250
Orthocladiinae|BIOUG07509-H08|Kate Perez|BIN Taxonomy Match|BOLD:AAD9250
Orthocladiinae|BIOUG07503-A04|Kate Perez|BIN Taxonomy Match|BOLD:AAD9250
Pseudorthocladius filiformis|SOE21|Elisabeth Stur||BOLD:AAD9250
Pseudorthocladius filiformis|SOE242|Elisabeth Stur||BOLD:AAD9250
Pseudorthocladius filiformis|SOE419|Elisabeth Stur||BOLD:AAD9250
Pseudorthocladius cf. curtistylus|BIOUG06088-D07|Kate Perez|BIN Taxonomy Match|BOLD:ACG6805
Pseudorthocladius cf. curtistylus|BIOUG07757-D04|Kate Perez|BIN Taxonomy Match|BOLD:ACG6805
Pseudorthocladius cf. curtistylus|BIOUG07413-F08|Kate Perez|BIN Taxonomy Match|BOLD:ACG6805
Pseudorthocladius cf. curtistylus|ZSM-DIP-33136-A07|Caroline Chimeno|BIN Taxonomy Match (Jan 2...
Pseudorthocladius cf. curtistylus|BIOUG07647-G01|Kate Perez|BIN Taxonomy Match|BOLD:ACG6805
Pseudorthocladius cf. curtistylus|BIOUG07415-C07|Kate Perez|BIN Taxonomy Match|BOLD:ACG6805
Pseudorthocladius cf. curtistylus|ZSM-DIP-33133-H02|Caroline Chimeno|BIN Taxonomy Match (Jan 2...
Pseudorthocladius cf. curtistylus|ZSM-DIP-33145-F04|Caroline Chimeno|BIN Taxonomy Match (Jan 2...
Pseudorthocladius cf. curtistylus|ZSM-DIP-33145-F05|Caroline Chimeno|BIN Taxonomy Match (Jan 2...
Pseudorthocladius cf. curtistylus|ZSM-DIP-33146-A09|Caroline Chimeno|BIN Taxonomy Match (Jan 2...
Pseudorthocladius cf. curtistylus|ZSM-DIP-33148-D07|Caroline Chimeno|BIN Taxonomy Match (Jan 2...
Pseudorthocladius cf. curtistylus|ZSM-DIP-33148-D08|Caroline Chimeno|BIN Taxonomy Match (Jan 2...
Pseudorthocladius cf. curtistylus|ZSM-DIP-33149-A10|Caroline Chimeno|BIN Taxonomy Match (Jan 2...
Pseudorthocladius cf. curtistylus|BIOUG07808-G10|Kate Perez|BIN Taxonomy Match|BOLD:ACG6805
Pseudorthocladius cf. curtistylus|BIOUG07758-D12|Kate Perez|BIN Taxonomy Match|BOLD:ACG6805
Pseudorthocladius cf. curtistylus|BIOUG07757-G06|Kate Perez|BIN Taxonomy Match|BOLD:ACG6805
Pseudorthocladius cf. curtistylus|BIOUG08097-B11|Kate Perez|BIN Taxonomy Match|BOLD:ACG6805
Pseudorthocladius cf. curtistylus|BIOUG07808-D11|Kate Perez|BIN Taxonomy Match|BOLD:ACG6805
Pseudorthocladius cf. curtistylus|BIOUG07567-F02|Kate Perez|BIN Taxonomy Match|BOLD:ACG6805
Pseudorthocladius cf. curtistylus|BIOUG07756-F07|Kate Perez|BIN Taxonomy Match|BOLD:ACG6805
Pseudorthocladius cf. curtistylus|BIOUG07754-A12|Kate Perez|BIN Taxonomy Match|BOLD:ACG6805
Pseudorthocladius cf. curtistylus|BIOUG06094-B04|Kate Perez|BIN Taxonomy Match|BOLD:ACG6805
Pseudorthocladius cf. curtistylus|ZSM-DIP-33149-A08|Caroline Chimeno|BIN Taxonomy Match (Jan 2...
Pseudorthocladius cf. curtistylus|BIOUG07759-F08|Kate Perez|BIN Taxonomy Match|BOLD:ACG6805
Pseudorthocladius cf. curtistylus|BIOUG07753-G04|Kate Perez|BIN Taxonomy Match|BOLD:ACG6805
Pseudorthocladius cf. curtistylus|BIOUG07567-A10|Kate Perez|BIN Taxonomy Match|BOLD:ACG6805
Pseudorthocladius cf. curtistylus|BIOUG07945-C06|Kate Perez|BIN Taxonomy Match|BOLD:ACG6805
Pseudorthocladius cf. curtistylus|BIOUG08138-H02|Kate Perez|BIN Taxonomy Match|BOLD:ACG6805
Pseudorthocladius filiformis|ZMUO.025119|Lauri Paasivirta||BOLD:AAD9251
Pseudorthocladius filiformis|SOE304|Elisabeth Stur||BOLD:AAD9251
Pseudorthocladius filiformis|SOE378|Elisabeth Stur||BOLD:AAD9251
Pseudorthocladius filiformis|ZMUO.025118|Lauri Paasivirta||BOLD:AAD9251
Pseudorthocladius filiformis|BIOUG07808-F07|Kate Perez|BIN Taxonomy Match|BOLD:AAD9251
Nanocladius balticus|ZMUO.025302|Lauri Paasivirta||BOLD:ADA3218
Parakiefferiella coronata|ZMUO.024940|Lauri Paasivirta||BOLD:ADA6291
Parakiefferiella coronata|ZMUO.025839|Lauri Paasivirta||BOLD:ADA6291
Parakiefferiella coronata|ZMUO.025838|Lauri Paasivirta||BOLD:ADA6291
Parakiefferiella coronata|ZMUO.024939|Lauri Paasivirta||BOLD:ADA6291
Parakiefferiella coronata|Finnmark678|Elisabeth Stur||BOLD:AAV2834
Orthocladiinae|GBOL03742|Sofia Wiedenbrug
Chironomidae|CCDB21605-E01|Sofia Wiedenbrug
Pseudorthocladius ABV3504|CCDB21605-E09|Sofia Wiedenbrug||BOLD:ABV3504
Pseudorthocladius ABV3504|CCDB21605-E02|Sofia Wiedenbrug||BOLD:ABV3504
Pseudorthocladius cf. curtistylus|TRD-CH195|Elisabeth Stur||BOLD:ABV3504
Pseudorthocladius cf. curtistylus|BIOUG16256-C06|Elisabeth Stur|BIN Taxonomy Match|BOLD:ABV3504
Pseudorthocladius|BIOUG36840-F12|Kate Perez|BIN Taxonomy Match (Jul 2018)|BOLD:ABV3504
Pseudorthocladius|BIOUG42927-E12|Kate Perez|BIN Taxonomy Match below Phylum (Jun 2019)|BOLD...
Pseudorthocladius cf. curtistylus|CH-OSF42|Elisabeth Stur||BOLD:ABV3504
Pseudorthocladius|BIOUG36841-D02|Kate Perez|BIN Taxonomy Match (Jul 2018)|BOLD:ABV3504
Pseudorthocladius|BIOUG42754-C09|Kate Perez|BIN Taxonomy Match below Phylum (Jun 2019)|BOLD...
Pseudorthocladius|BIOUG42733-G02|Kate Perez|BIN Taxonomy Match below Phylum (Jun 2019)|BOLD...
Pseudorthocladius ABV3504|BIOUG07757-A06|Kate Perez|BIN Taxonomy Match|BOLD:ABV3504
Pseudorthocladius ABV3504|BIOUG07944-H01|Kate Perez|BIN Taxonomy Match|BOLD:ABV3504
Pseudorthocladius|BIOUG07646-E06|Kate Perez|BIN Taxonomy Match|BOLD:ABV3504
Pseudorthocladius curtistylus|ZSM-DIP-33170-F05|Caroline Chimeno|BIN Taxonomy Match (Jan 2022...
Pseudorthocladius curtistylus|ZSM-DIP-33148-A07|Caroline Chimeno|BIN Taxonomy Match (Jan 2022...
Pseudorthocladius cf. curtistylus|TRD-CH235|Elisabeth Stur||BOLD:ABV3504
Pseudorthocladius curtistylus|NHRS-BYWS000000559|Yngve Brodin|morphology|BOLD:ABV3504
Pseudorthocladius curtistylus|NHRS-BYWS000000462|Yngve Brodin|morphology|BOLD:ABV3504
Pseudorthocladius cf. curtistylus|CH-eik65|Elisabeth Stur||BOLD:ABV3504
Pseudorthocladius cf. curtistylus|CH-eik62|Elisabeth Stur||BOLD:ABV3504
Pseudorthocladius|ZSM-DIP-33148-B03|Caroline Chimeno|BIN Taxonomy Match (Jan 2022)|BOLD:...
Pseudorthocladius|ZSM-DIP-33139-G12|Caroline Chimeno|BIN Taxonomy Match (Jan 2022)|BOLD:...
Pseudorthocladius curtistylus|NHRS-BYWS000000461|Yngve Brodin|morphology|BOLD:ABV3504
Pseudorthocladius curtistylus|ZMUO.024943|Lauri Paasivirta||BOLD:ABV3504
Pseudorthocladius ABV3504|BIOUG07406-C08|Kate Perez|BIN Taxonomy Match|BOLD:ABV3504
Pseudorthocladius|BIOUG36992-G03|Kate Perez|BIN Taxonomy Match (Jul 2018)|BOLD:ABV3504
Pseudorthocladius cf. curtistylus|CH-eik89|Elisabeth Stur||BOLD:ABV3504
Pseudorthocladius cf. curtistylus|CH-eik112|Elisabeth Stur||BOLD:ABV3504
Pseudorthocladius curtistylus|NHRS-BYWS000000933|Yngve Brodin|morphology|BOLD:ABV3504
Pseudorthocladius curtistylus|ZSM-DIP-33148-A12|Caroline Chimeno|BIN Taxonomy Match (Jan 2022...
Pseudorthocladius curtistylus|ZSM-DIP-33170-F09|Caroline Chimeno|BIN Taxonomy Match (Jan 2022...
Pseudorthocladius ABV3504|BIOUG08129-B02|Kate Perez|BIN Taxonomy Match|BOLD:ABV3504
Pseudorthocladius cf. curtistylus|BIOUG15793-E09|Elisabeth Stur|BIN Taxonomy Match|BOLD:ABV3504
Pseudorthocladius ABV3504|BIOUG07809-F01|Kate Perez|BIN Taxonomy Match|BOLD:ABV3504
[illegible]

Pseudorthocladius ABV3504|BIOUG08129-B02|Kate Perez|BIN Taxonomy Match|BOLD:ABV3504
Pseudorthocladius cf. curtistylus|BIOUG15793-E09|Elisabeth Stur|BIN Taxonomy Match|BOLD:ABV3504
Pseudorthocladius ABV3504|BIOUG07809-F01|Kate Perez|BIN Taxonomy Match|BOLD:ABV3504
Pseudorthocladius ABV3504|BIOUG08126-D08|Kate Perez|BIN Taxonomy Match|BOLD:ABV3504
Pseudorthocladius curtistylus|ZSM-DIP-33170-H05|Caroline Chimeno|BIN Taxonomy Match (Jan 2022...
Pseudorthocladius|BIOUG36762-A12|Kate Perez|BIN Taxonomy Match (May 2018)|BOLD:ABV3504
Pseudorthocladius ABV3504|BIOUG07930-A12|Kate Perez|BIN Taxonomy Match|BOLD:ABV3504
Orthocladiinae|BIOUG36682-A02|Kate Perez|BIN Taxonomy Match (Jul 2018)|BOLD:ACF9345
Allocladius nanseni|Finnmark439|Elisabeth Stur||BOLD:AAL1593
Nanocladius distinctus|NHRS-BYWS000000923|Yngve Brodin|morphology|BOLD:ACC9943
Psectrocladius oxyura|ZSM-DIP-33129-D12|Valerie Levesque-Beaudin|BOLD ID Engine Manual (Sep 20...
Psectrocladius limbatellus|NHRS-BYWS000001163|Yngve Brodin|morphology|BOLD:AEG1130
Psectrocladius limbatellus|NHRS-BYWS000000435|Yngve Brodin|morphology|BOLD:AEG1130
Psectrocladius limbatellus|NHRS-BYWS000001802|Yngve Brodin||BOLD:AEG1130
Psectrocladius limbatellus|NHRS-BYWS000000964|Yngve Brodin|morphology|BOLD:AAU0273
Psectrocladius ventricosus|ZMUO.025354|Lauri Paasivirta||BOLD:AAU0273
Psectrocladius limbatellus|NHRS-BYWS000000220|Yngve Brodin|morphology|BOLD:AAU0273
Psectrocladius limbatellus|NHRS-BYWS000000965|Yngve Brodin|morphology|BOLD:AAU0273
Psectrocladius limbatellus|NHRS-BYWS000000575|Yngve Brodin|morphology|BOLD:AAU0273
Psectrocladius limbatellus|NHRS-BYWS000000165|Yngve Brodin|morphology|BOLD:AAU0273
Psectrocladius limbatellus|NHRS-BYWS000001863|Yngve Brodin||BOLD:AAU0273
Psectrocladius limbatellus|NHRS-BYWS000000222|Yngve Brodin|morphology|BOLD:AAU0273
Psectrocladius limbatellus|NHRS-BYWS000000221|Yngve Brodin|morphology|BOLD:AAU0273
Psectrocladius limbatellus|NHRS-BYWS000000385|Yngve Brodin|morphology|BOLD:AAU0273
Psectrocladius limbatellus|NHRS-BYWS000000338|Yngve Brodin|morphology|BOLD:AAU0273
Psectrocladius limbatellus|NHRS-BYWS000000412|Yngve Brodin|morphology|BOLD:AAU0273
Psectrocladius limbatellus|NHRS-BYWS000001745|Yngve Brodin||BOLD:AAU0273
Psectrocladius limbatellus|NHRS-BYWS000001744|Yngve Brodin||BOLD:AAU0273
Psectrocladius limbatellus|NHRS-BYWS000001036|Yngve Brodin|morphology|BOLD:AAU0273
Psectrocladius limbatellus|NHRS-BYWS000001871|Yngve Brodin||BOLD:AAU0273
Psectrocladius limbatellus|NHRS-BYWS000001743|Yngve Brodin||BOLD:AAU0273
Psectrocladius limbatellus|NHRS-BYWS000000373|Yngve Brodin|morphology|BOLD:AAU0273
Psectrocladius limbatellus|NHRS-BYWS000000226|Yngve Brodin|morphology|BOLD:AAU0273
Psectrocladius ventricosus|ZMUO.025353|Lauri Paasivirta||BOLD:AAU0273
Psectrocladius limbatellus|NHRS-BYWS000000374|Yngve Brodin|morphology|BOLD:AAU0273
Psectrocladius limbatellus|NHRS-BYWS000000219|Yngve Brodin|morphology|BOLD:AAU0273
Psectrocladius ventricosus|EJV-20110232|Lauri Paasivirta||BOLD:AAU0273
Psectrocladius|ZMUO.026019|Lauri Paasivirta||BOLD:AAD0483
Psectrocladius|ZMUO.026018|Lauri Paasivirta||BOLD:AAD0483
Psectrocladius barbimanus|Finnmark113|Elisabeth Stur||BOLD:AAD0483
Psectrocladius barbimanus|NHRS-BYWS000000502|Yngve Brodin|morphology|BOLD:AAU6335
Psectrocladius barbimanus|NHRS-BYWS000001858|Yngve Brodin||BOLD:AAU6335
Rheocricotopus chapmani|24465-B05|Kate Perez|BIN Taxonomy Match|BOLD:AAI0295
Rheocricotopus chapmani|ZMUO.026092|Lauri Paasivirta||BOLD:AAI0295
Rheocricotopus chapmani|24373-E06|Kate Perez|BIN Taxonomy Match|BOLD:AAI0295
Rheocricotopus chapmani|24354-B10|Kate Perez|BIN Taxonomy Match|BOLD:AAI0295
Rheocricotopus chapmani|ATNA294|Elisabeth Stur||BOLD:AAI0295
Rheocricotopus chapmani|ATNA262|Elisabeth Stur||BOLD:AAI0295
Rheocricotopus nr. unidentatus|ZMUO.024793|Lauri Paasivirta||BOLD:ADA5961
Rheocricotopus|ZMUO.025951|Lauri Paasivirta||BOLD:ADA5962
Rheocricotopus nr. unidentatus|ZMUO.024792|Lauri Paasivirta||BOLD:ADA5962
Rheocricotopus|ZMUO.025950|Lauri Paasivirta||BOLD:ADA5962
Rheocricotopus|ZSM-DIP-33137-B06|Caroline Chimeno|BIN Taxonomy Match (Jan 2022)|BOLD:ADY...
Rheocricotopus sp. 3ES|BC-ZSM-DIP-22488-E11|Dieter Doczkal||BOLD:ADY9601
Rheocricotopus sp. 3ES|CCDB24183-F12|Sofia Wiedenbrug|Morphology|BOLD:AEE5575
Rheocricotopus sp. 3ES|TRD-CH273|Elisabeth Stur||BOLD:AAB3290
Rheocricotopus sp. 3ES|EBAI-Ch076|Elisabeth Stur|morphology|BOLD:AAB3290
Rheocricotopus sp. 3ES|EBAI-Ch079|Elisabeth Stur|morphology|BOLD:AAB3290
Rheocricotopus sp. 3ES|EBAI-Ch077|Elisabeth Stur|morphology|BOLD:AAB3290
Rheocricotopus sp. 3ES|EBAI-Ch081|Elisabeth Stur|morphology|BOLD:AAB3290
Rheocricotopus sp. 3ES|EBAI-Ch080|Elisabeth Stur|morphology|BOLD:AAB3290
Rheocricotopus sp. 3ES|ATNA467|Elisabeth Stur||BOLD:AAB3290
Rheocricotopus sp. 3ES|SOE327|Torbjorn Ekrem||BOLD:AAB3290
Rheocricotopus effusus|ZMUO.024723|Lauri Paasivirta||BOLD:AAB3288
Chironomidae|CCDB22605-B10|Sofia Wiedenbrug|no morphological identification - introduction of int...
Chironomidae|GBOL11540|Sofia Wiedenbrug|no morphological identification - introduction of interim...
Chironomidae|CCDB21606-A09|Sofia Wiedenbrug|no morphological identification - introduction of int...
Rheocricotopus effusus|ZMUO.024722|Lauri Paasivirta||BOLD:ACH2822
Rheocricotopus effusus|ZSM-DIP-33139-E10|Caroline Chimeno|BIN Taxonomy Match (Jan 2022)|BOL...
Rheocricotopus effusus|ZSM-DIP-33137-B03|Caroline Chimeno|BIN Taxonomy Match (Jan 2022)|BOL...
Rheocricotopus effusus|ZSM-DIP-33131-G07|Caroline Chimeno|BIN Taxonomy Match (Jan 2022)|BOL...
Chironomidae|BC-ZSM-DIP-22488-D05|Dieter Doczkal|no morphological identification - introduction o...
Chironomidae|CCDB24228-D10|Sofia Wiedenbrug|no morphological identification - introduction of int...
Chironomidae|CCDB24038-H06|Sofia Wiedenbrug|no morphological identification - introduction of int...
Chironomidae|CCDB24038-H05|Sofia Wiedenbrug|no morphological identification - introduction of int...
Chironomidae|CCDB24038-G10|Sofia Wiedenbrug|no morphological identification - introduction of int...
Chironomidae|BIOUG07406-H06|Kate Perez|no morphological identification - introduction of interim ...
Rheocricotopus effusus|ZSM-DIP-33131-G06|Caroline Chimeno|BIN Taxonomy Match (Jan 2022)|BOL...
Rheocricotopus effusus|BIOUG05897-H09|Kate Perez|BIN Taxonomy Match|BOLD:AAB3288
Chironomidae|BIOUG07807-D08|Meredith Miller|Tree-based Identification (Feb 2019)
Rheocricotopus effusus|Finnmark147|Elisabeth Stur||BOLD:AAB3288
Rheocricotopus effusus|SOE458|Torbjorn Ekrem||BOLD:AAB3288
Rheocricotopus effusus|CCDB24183-G12|Sofia Wiedenbrug|Morphology|BOLD:AAB3288
Rheocricotopus effusus|ES108|Elisabeth Stur||BOLD:AAB3288
Rheocricotopus effusus|ES12|Elisabeth Stur||BOLD:AAB3288
Rheocricotopus effusus|ES11|Elisabeth Stur||BOLD:AAB3288
Rheocricotopus effusus|SOE456|Torbjorn Ekrem||BOLD:AAB3288
Rheocricotopus effusus|SOE399|Torbjorn Ekrem||BOLD:AAB3288
Rheocricotopus effusus|ES110|Elisabeth Stur||BOLD:AAB3288
Rheocricotopus effusus|SOE410|Torbjorn Ekrem||BOLD:AAB3288
Rheocricotopus effusus|SOE405|Torbjorn Ekrem||BOLD:AAB3288
Rheocricotopus effusus|SOE396|Torbjorn Ekrem||BOLD:AAB3288
Rheocricotopus effusus|SOE474|Torbjorn Ekrem||BOLD:AAB3288
Rheocricotopus effusus|ATNA148|Elisabeth Stur||BOLD:AAB3288
Rheocricotopus effusus|SOE179|Elisabeth Stur||BOLD:AAB3288
Rheocricotopus effusus|SOE199|Elisabeth Stur||BOLD:AAB3288
Rheocricotopus effusus|SOE149|Elisabeth Stur||BOLD:AAB3288
Rheocricotopus effusus|SOE77|Elisabeth Stur||BOLD:AAB3288
Rheocricotopus effusus|SOE308|Torbjorn Ekrem||BOLD:AAB3288
Rheocricotopus effusus|SOE148|Elisabeth Stur||BOLD:AAB3288
Rheocricotopus effusus|SOE251|Elisabeth Stur||BOLD:AAB3288
Rheocricotopus effusus|EBAI-Ch078|Elisabeth Stur|morphology|BOLD:AAB3288
Rheocricotopus effusus|ATNA347|Elisabeth Stur||BOLD:AAB3288

Rheocricotopus effusus|SOE251|Elisabeth Stur||BOLD:AAB3288
Rheocricotopus effusus|EBAI-Ch078|Elisabeth Stur|morphology|BOLD:AAB3288
Rheocricotopus effusus|ATNA347|Elisabeth Stur||BOLD:AAB3288
Rheocricotopus effusus|SOE409|Elisabeth Stur||BOLD:AAB3288
Rheocricotopus effusus|SOE404|Torbjorn Ekrem||BOLD:AAB3288
Rheocricotopus effusus|SOE395|Torbjorn Ekrem||BOLD:AAB3288
Rheocricotopus effusus|ATNA477|Elisabeth Stur||BOLD:AAB3288
Rheocricotopus sp. 2ES|Finnmark466|Elisabeth Stur||BOLD:AAV2321
Orthocladiinae|BIOUG15654-E04|Kate Perez|BOLD ID Engine Manual|BOLD:ACL2710
Orthocladiinae|BIOUG16626-E09|Kate Perez|BOLD ID Engine Manual|BOLD:ACL2710
Orthocladiinae|BIOUG36844-B04|Kate Perez|BIN Taxonomy Match (Jul 2018)|BOLD:ACL2710
Orthocladiinae|BIOUG16434-G06|Kate Perez|BOLD ID Engine Manual|BOLD:ACL2710
Orthocladiinae|BIOUG15457-D02|Kate Perez|BOLD ID Engine Manual|BOLD:ACL2710
Orthocladiinae|BIOUG16551-D09|Kate Perez|BOLD ID Engine Manual|BOLD:ACL2710
Chironomidae|BIOUG43081-F01|Kate Perez|BIN Taxonomy Match (Jul 2019)|BOLD:ADU7549
Chironomidae|BIOUG43080-C10|Kate Perez|BIN Taxonomy Match (Jul 2019)|BOLD:ADU7549
Chironomidae|BIOUG42966-H06|Kate Perez|BIN Taxonomy Match (Jul 2019)|BOLD:ADU7549
Chironomidae|BIOUG42966-B06|Kate Perez|BIN Taxonomy Match (Jul 2019)|BOLD:ADU7549
Chironomidae|BIOUG42964-C02|Kate Perez|BIN Taxonomy Match (Jul 2019)|BOLD:ADU7549
Chironomidae|BIOUG42959-F03|Kate Perez|BIN Taxonomy Match (Jul 2019)|BOLD:ADU7549
Chironomidae|BIOUG43076-H01|Kate Perez|BIN Taxonomy Match (Jul 2019)|BOLD:ADU7549
Chironomidae|BIOUG55104-E02|Stefan Schmidt|BIN Taxonomy Match (Sep 2020)|BOLD:ADU7549
Smittia edwardsi|NO26|Torbjorn Ekrem||BOLD:AAF4817
Smittia edwardsi|BIOUG43075-C09|Kate Perez|BIN Taxonomy Match below Phylum (Jul 2019)|BOLD...
Smittia edwardsi|CH-OSF150|Elisabeth Stur||BOLD:ADV7271
Smittia edwardsi|ZMUO.024591|Lauri Paasivirta||BOLD:ADV7271
Smittia edwardsi|ZSM-DIP-33131-E06|Caroline Chimeno|BIN Taxonomy Match (Jan 2022)|BOLD:A...
Smittia edwardsi|ZSM-DIP-33170-C12|Caroline Chimeno|BIN Taxonomy Match (Jan 2022)|BOLD:A...
Smittia edwardsi|BC-ZSM-DIP-22551-F06|Dieter Doczkal||BOLD:ADV7271
Smittia edwardsi|BIOUG42965-A07|Kate Perez|BIN Taxonomy Match below Phylum (Jul 2019)|BOLD...
Smittia edwardsi|BIOUG42961-B11|Kate Perez|BIN Taxonomy Match below Phylum (Jul 2019)|BOLD...
Smittia edwardsi|BIOUG42960-F11|Kate Perez|BIN Taxonomy Match below Phylum (Jul 2019)|BOLD...
Smittia edwardsi|BIOUG42960-D02|Kate Perez|BIN Taxonomy Match below Phylum (Jul 2019)|BOLD...
Smittia edwardsi|NHRS-BYWS000001017|Yngve Brodin|morphology|BOLD:ADV7271
Smittia edwardsi|ZMUO.024590|Lauri Paasivirta||BOLD:ADV7271
Smittia edwardsi|ZSM-DIP-33131-D04|Caroline Chimeno|BIN Taxonomy Match (Jan 2022)|BOLD:A...
Smittia edwardsi|NHRS-BYWS000001056|Yngve Brodin|morphology|BOLD:ADV7271
Smittia edwardsi|NHRS-BYWS000000966|Yngve Brodin|morphology|BOLD:ADV7271
Smittia edwardsi|TRD-CH415|Elisabeth Stur|morphology|BOLD:ADV7271
Smittia edwardsi|ATNA578|Elisabeth Stur|morphology|BOLD:ADV7271
Smittia edwardsi|BIOUG16436-A05|Kate Perez|BIN Taxonomy Match|BOLD:ADV7271
Smittia edwardsi|BIOUG15456-B04|Kate Perez|BIN Taxonomy Match|BOLD:ADV7271
Chironomidae|24371-D06|Mikko J Tiusanen|Tree based identification|BOLD:ACU5354
Chironomidae|24373-C05|Mikko J Tiusanen|Tree based identification|BOLD:ACU5354
Chironomidae|24371-D09|Mikko J Tiusanen|Tree based identification|BOLD:ACU5354
Chironomidae|24465-D04|Mikko J Tiusanen|Tree based identification|BOLD:ACU5354
Chironomidae|24373-F11|Mikko J Tiusanen|Tree based identification|BOLD:ACU5354
Chironomidae|24354-E10|Mikko J Tiusanen|Tree based identification|BOLD:ACU5354
Chironomidae|24354-B02|Mikko J Tiusanen|Tree based identification|BOLD:ACU5354
Chironomidae|23878-H05|Mikko J Tiusanen|Tree based identification|BOLD:ACU5354
Chironomidae|24354-B04|Mikko J Tiusanen|Tree based identification|BOLD:ACU5354
Chironomidae|24354-B08|Mikko J Tiusanen|Tree based identification|BOLD:ACU5354
Chironomidae|23878-F03|Mikko J Tiusanen|Tree based identification|BOLD:ACU5354
Chironomidae|23975-B05|Mikko J Tiusanen|Tree based identification|BOLD:ACU5354
Chironomidae|24373-F08|Mikko J Tiusanen|Tree based identification|BOLD:ACU5354
Chironomidae|24373-G04|Mikko J Tiusanen|Tree based identification|BOLD:ACU5354
Chironomidae|24373-A04|Mikko J Tiusanen|Tree based identification|BOLD:ACU5354
Chironomidae|24372-F07|Mikko J Tiusanen|Tree based identification|BOLD:ACU5354
Chironomidae|24372-C05|Mikko J Tiusanen|Tree based identification|BOLD:ACU5354
Chironomidae|24372-C04|Mikko J Tiusanen|Tree based identification|BOLD:ACU5354
Chironomidae|24464-D05|Mikko J Tiusanen|Tree based identification|BOLD:ACU5354
Chironomidae|24371-C06|Mikko J Tiusanen|Tree based identification|BOLD:ACU5354
Chironomidae|24372-H11|Mikko J Tiusanen|Tree based identification|BOLD:ACU5354
Chironomidae|24354-E02|Mikko J Tiusanen|Tree based identification|BOLD:ACU5354
Chironomidae|24371-A07|Mikko J Tiusanen|Tree based identification|BOLD:ACU5354
Chironomidae|24465-F01|Mikko J Tiusanen|Tree based identification|BOLD:ACU5354
Chironomidae|24465-E09|Mikko J Tiusanen|Tree based identification|BOLD:ACU5354
Chironomidae|23975-A11|Mikko J Tiusanen|Tree based identification|BOLD:ACU5354
Chironomidae|23878-G08|Mikko J Tiusanen|Tree based identification|BOLD:ACU5354
Chironomidae|23878-F04|Mikko J Tiusanen|Tree based identification|BOLD:ACU5354
Chironomidae|25131-F04|Kate Perez|BOLD ID Engine Manual (Apr 2018)|BOLD:ACU5354
Chironomidae|25130-G04|Kate Perez|BOLD ID Engine Manual (Apr 2018)|BOLD:ACU5353
Chironomidae|23878-F07|Mikko J Tiusanen|Tree based identification|BOLD:ACU5353
Chironomidae|24354-E12|Mikko J Tiusanen|Tree based identification|BOLD:ACU5353
Chironomidae|25128-B06|Kate Perez|BIN Taxonomy Match (Mar 2018)|BOLD:ACU5353
Chironomidae|25131-G06|Kate Perez|BOLD ID Engine Manual (Apr 2018)|BOLD:ACU5353
Chironomidae|25130-C01|Kate Perez|BOLD ID Engine Manual (Apr 2018)|BOLD:ACU5353
Chironomidae|25128-H07|Kate Perez|BIN Taxonomy Match (Mar 2018)|BOLD:ACU5353
Chironomidae|25131-C07|Kate Perez|BOLD ID Engine Manual (Apr 2018)|BOLD:ACU5353
Chironomidae|25130-H01|Kate Perez|BOLD ID Engine Manual (Apr 2018)|BOLD:ACU5353
Chironomidae|25131-F09|Kate Perez|BOLD ID Engine Manual (Apr 2018)|BOLD:ACU5353
Chironomidae|25130-B01|Kate Perez|BOLD ID Engine Manual (Apr 2018)|BOLD:ACU5353
Chironomidae|25132-D08|Kate Perez|BOLD ID Engine Manual (Apr 2018)|BOLD:ACU5353
Chironomidae|25130-E02|Kate Perez|BOLD ID Engine Manual (Apr 2018)|BOLD:ACU5353
Chironomidae|24465-G04|Mikko J Tiusanen|Tree based identification|BOLD:ACU5353
Chironomidae|25128-C07|Kate Perez|BIN Taxonomy Match (Mar 2018)|BOLD:ACU5353
Chironomidae|25131-E07|Kate Perez|BOLD ID Engine Manual (Apr 2018)|BOLD:ACU5353
Chironomidae|25128-A10|Kate Perez|BIN Taxonomy Match (Mar 2018)|BOLD:ACU5353
Chironomidae|25129-H03|Kate Perez|BIN Taxonomy Match (Mar 2018)|BOLD:ACU5353
Chironomidae|25132-D12|Kate Perez|BOLD ID Engine Manual (Apr 2018)|BOLD:ACU5353
Chironomidae|25132-H09|Kate Perez|BOLD ID Engine Manual (Apr 2018)|BOLD:ACU5353
Chironomidae|25130-E09|Kate Perez|BOLD ID Engine Manual (Apr 2018)|BOLD:ACU5353
Chironomidae|25129-B11|Kate Perez|BIN Taxonomy Match (Mar 2018)|BOLD:ACU5353
Chironomidae|25128-D05|Kate Perez|BIN Taxonomy Match (Mar 2018)|BOLD:ACU5353
Chironomidae|25129-C04|Kate Perez|BIN Taxonomy Match (Mar 2018)|BOLD:ACU5353
Chironomidae|25129-G11|Kate Perez|BIN Taxonomy Match (Mar 2018)|BOLD:ACU5353
Chironomidae|25128-G04|Kate Perez|BIN Taxonomy Match (Mar 2018)|BOLD:ACU5353
Smittia edwardsi|ATNA237|Elisabeth Stur||BOLD:AAF4818
Smittia edwardsi|ATNA238|Elisabeth Stur||BOLD:ACN5734
Smittia edwardsi|ATNA392|Elisabeth Stur
Chironomidae|BIOUG08132-G04|Kate Perez|no morphological identification - introduction of interim ...
Chironomidae|BIOUG08132-E07|Kate Perez|no morphological identification - introduction of interim ...
Chironomidae|BIOUG05109-D05|Kate Perez|no morphological identification - introduction of interim [illegible]

[illegible]
Chironomidae|BIOUG08132-G04|Kate Perez|no morphological identification - introduction of interim ...
Chironomidae|BIOUG08132-E07|Kate Perez|no morphological identification - introduction of interim ...
Chironomidae|BIOUG05109-D05|Kate Perez|no morphological identification - introduction of interim ...
Smittia sp. 16ES|Finnmark465|Elisabeth Stur||BOLD:AAB0375
Smittia sp. 16ES|BIOUG16551-D05|Elisabeth Stur|Tree based identification
Smittia sp. 16ES|Finnmark464|Elisabeth Stur||BOLD:AAB0375
Smittia sp. 16ES|Finnmark536|Elisabeth Stur||BOLD:AAB0375
Smittia sp. 16ES|TRD-CH132|Elisabeth Stur||BOLD:AAB0375
Smittia sp. 16ES|CH-eik61|Elisabeth Stur||BOLD:AAB0375
Smittia sp. 16ES|Finnmark63|Elisabeth Stur||BOLD:AAB0375
Smittia sp. 16ES|TRD-CH65|Elisabeth Stur||BOLD:AAB0375
Smittia sp. 16ES|TRD-CH352|Elisabeth Stur|morphology|BOLD:AAB0375
Smittia sp. 16ES|BIOUG15648-F11|Kate Perez|BIN Taxonomy Match|BOLD:AAB0375
Smittia sp. 16ES|BIOUG16433-A10|Kate Perez|BIN Taxonomy Match|BOLD:AAB0375
Smittia sp. 16ES|BIOUG16476-D04|Kate Perez|BIN Taxonomy Match|BOLD:AAB0375
Smittia sp. 16ES|BIOUG15563-A08|Kate Perez|BIN Taxonomy Match|BOLD:AAB0375
Smittia sp. 16ES|BIOUG15544-D04|Kate Perez|BIN Taxonomy Match|BOLD:AAB0375
Smittia sp. 16ES|24373-A02|Kate Perez|BIN Taxonomy Match|BOLD:AAB0375
Smittia sp. 16ES|BIOUG15718-F11|Kate Perez|BIN Taxonomy Match|BOLD:AAB0375
Smittia sp. 16ES|BIOUG15949-D06|Kate Perez|BIN Taxonomy Match|BOLD:AAB0375
Smittia sp. 16ES|BIOUG15807-B12|Kate Perez|BIN Taxonomy Match|BOLD:AAB0375
Smittia sp. 16ES|BIOUG15807-C12|Kate Perez|BIN Taxonomy Match|BOLD:AAB0375
Smittia sp. 16ES|BIOUG15791-H04|Kate Perez|BIN Taxonomy Match|BOLD:AAB0375
Smittia sp. 16ES|BIOUG15965-B10|Kate Perez|BIN Taxonomy Match|BOLD:AAB0375
Smittia sp. 16ES|BIOUG15460-H06|Kate Perez|BIN Taxonomy Match|BOLD:AAB0375
Smittia sp. 16ES|BIOUG15855-D09|Kate Perez|BIN Taxonomy Match|BOLD:AAB0375
Smittia sp. 16ES|BIOUG15460-G07|Kate Perez|BIN Taxonomy Match|BOLD:AAB0375
Smittia sp. 16ES|BIOUG15954-E06|Kate Perez|BIN Taxonomy Match|BOLD:AAB0375
Smittia sp. 16ES|BIOUG15905-C08|Kate Perez|BIN Taxonomy Match|BOLD:AAB0375
Smittia sp. 16ES|BIOUG15561-G02|Kate Perez|BIN Taxonomy Match|BOLD:AAB0375
Smittia sp. 16ES|BIOUG16328-G11|Kate Perez|BIN Taxonomy Match|BOLD:AAB0375
Smittia sp. 16ES|BIOUG16426-G11|Kate Perez|BIN Taxonomy Match|BOLD:AAB0375
Smittia sp. 16ES|NO15|Elisabeth Stur
Smittia sp. 16ES|Finnmark617|Elisabeth Stur
Psectrocladius zetterstedti|ZMUO.024618|Lauri Paasivirta||BOLD:ADA8696
Psectrocladius sp. 6TE|Finnmark99|Torbjorn Ekrem||BOLD:AAM9561
Psectrocladius sp. 6TE|Finnmark96|Torbjorn Ekrem||BOLD:AAM9561
Psectrocladius sp. 6TE|TRD-CH67|Elisabeth Stur||BOLD:AAM9561
Psectrocladius sp. 6TE|NO 98|Elisabeth Stur||BOLD:AAM9561
Chironomidae|BI2019_V06||BOLD ID Engine
Orthocladiinae|BI2019_E02|Erlend I. F. Fossen|BOLD ID-Engine|BOLD:ABW5949
Nanocladius dichromus|ZMUO.025310|Lauri Paasivirta||BOLD:AAC3042
Nanocladius dichromus|ZMUO.025309|Lauri Paasivirta||BOLD:AAC3042
Nanocladius minimus|Finnmark840|Elisabeth Stur||BOLD:ACM4858
Nanocladius dichromus|ZMUO.024587|Lauri Paasivirta||BOLD:ADA6426
Nanocladius dichromus|ZMUO.024586|Lauri Paasivirta||BOLD:ADA6426
Nanocladius dichromus|ZMUO.025255|Lauri Paasivirta||BOLD:ADA5095
Nanocladius dichromus|ZMUO.025256|Lauri Paasivirta||BOLD:ADA5095
Nanocladius dichromus|NHRS-BYWS000000267|Yngve Brodin|morphology|BOLD:ADA5095
Nanocladius dichromus|NHRS-BYWS000000294|Yngve Brodin|morphology|BOLD:ADJ9286
Nanocladius dichromus|NHRS-BYWS000000408|Yngve Brodin|morphology
Nanocladius dichromus|NHRS-BYWS000000543|Yngve Brodin|morphology|BOLD:ADJ9287
Nanocladius dichromus|NHRS-BYWS000001180|Yngve Brodin|morphology|BOLD:ADJ9285
Nanocladius dichromus|EJV-20110075|Lauri Paasivirta||BOLD:AAW4696
Nanocladius dichromus|EJV-20110076|Lauri Paasivirta||BOLD:AAW4696
Nanocladius dichromus|EJV-20110077|Lauri Paasivirta||BOLD:AAW4696
Nanocladius dichromus|EJV-20110074|Lauri Paasivirta||BOLD:AAW4696
Nanocladius dichromus|EJV-20110073|Lauri Paasivirta||BOLD:AAW4696
Nanocladius dichromus|EJV-20110072|Lauri Paasivirta||BOLD:AAW4696
Nanocladius dichromus|NHRS-BYWS000000258|Yngve Brodin|morphology|BOLD:AAU3477
Nanocladius distinctus|Finnmark483|Elisabeth Stur||BOLD:AAU3477
Nanocladius distinctus|Finnmark468|Elisabeth Stur||BOLD:AAU3477
Psectrocladius|ZMUO.025908|Lauri Paasivirta||BOLD:AAH8154
Psectrocladius limbatellus|ZMUO.026295|Lauri Paasivirta||BOLD:AAH8154
Psectrocladius|ZMUO.025909|Lauri Paasivirta||BOLD:AAH8154
Psectrocladius oligosetus|SOE372|Torbjorn Ekrem||BOLD:AAH8154
Psectrocladius limbatellus|ZMUO.026296|Lauri Paasivirta||BOLD:AAH8154
Psectrocladius|ZMUO.026281|Lauri Paasivirta||BOLD:AAH8154
Psectrocladius oligosetus|NO01|Torbjorn Ekrem||BOLD:AAH8154
Psectrocladius oligosetus|Finnmark114|Elisabeth Stur||BOLD:AAH8154
Psectrocladius oligosetus|Finnmark342|Elisabeth Stur||BOLD:AAH8154
Psectrocladius fennicus|Finnmark93|Torbjorn Ekrem||BOLD:AAM9562
Psectrocladius fennicus|NHRS-BYWS000001121|Yngve Brodin|morphology|BOLD:AAM9562
Psectrocladius fennicus|Finnmark583|Torbjorn Ekrem||BOLD:AAM9562
Psectrocladius schlienzi|TRD-CH45|Elisabeth Stur||BOLD:AAU6327
Psectrocladius schlienzi|RIN_CH67|Elisabeth Stur|BOLD Identification engine|BOLD:AAU6327
Psectrocladius schlienzi|RIN_CH35|Elisabeth Stur|BOLD Identification engine|BOLD:AAU6327
Psectrocladius schlienzi|RIN_CH62|Elisabeth Stur|BOLD Identification engine|BOLD:AAU6327
Psectrocladius schlienzi|TRD-CH50|Elisabeth Stur|morphology|BOLD:AAU6327
Psectrocladius schlienzi|BIOUG42784-C03|Kate Perez|BIN Taxonomy Match below Phylum (Jun 2019 ...
Psectrocladius schlienzi|BIOUG42694-F12|Kate Perez|BIN Taxonomy Match below Phylum (Jun 2019 ...
Psectrocladius schlienzi|BIOUG42672-D11|Kate Perez|BIN Taxonomy Match below Phylum (Jun 2019 ...
Psectrocladius schlienzi|BIOUG55122-B06|Stefan Schmidt|BIN Taxonomy Match (Sep 2020)|BOLD ...
Psectrocladius schlienzi|BIOUG42692-D10|Kate Perez|BIN Taxonomy Match below Phylum (Jun 2019 ...
Psectrocladius schlienzi|BIOUG42698-D11|Kate Perez|BIN Taxonomy Match below Phylum (Jun 2019 ...
Psectrocladius schlienzi|BIOUG42725-G03|Kate Perez|BIN Taxonomy Match below Phylum (Jun 2019 ...
Psectrocladius schlienzi|BIOUG42729-G12|Kate Perez|BIN Taxonomy Match below Phylum (Jun 2019 ...
Psectrocladius schlienzi|BIOUG42731-E07|Kate Perez|BIN Taxonomy Match below Phylum (Jun 2019 ...
Psectrocladius schlienzi|BIOUG42735-F01|Kate Perez|BIN Taxonomy Match below Phylum (Jun 2019 ...
Psectrocladius schlienzi|BIOUG42788-B08|Kate Perez|BIN Taxonomy Match below Phylum (Jun 2019 ...
Psectrocladius schlienzi|BIOUG55191-A12|Stefan Schmidt|BIN Taxonomy Match (Sep 2020)|BOLD ...
Psectrocladius schlienzi|BIOUG55189-H09|Stefan Schmidt|BIN Taxonomy Match (Sep 2020)|BOLD ...
Psectrocladius schlienzi|BIOUG42681-E05|Kate Perez|BIN Taxonomy Match below Phylum (Jun 2019 ...
Psectrocladius schlienzi|BIOUG55199-A01|Stefan Schmidt|BIN Taxonomy Match (Sep 2020)|BOLD ...
Psectrocladius schlienzi|BIOUG55112-F12|Stefan Schmidt|BIN Taxonomy Match (Sep 2020)|BOLD ...
Psectrocladius schlienzi|NHRS-BYWS000001873|Yngve Brodin||BOLD:AAU6327
Psectrocladius schlienzi|NHRS-BYWS000001872|Yngve Brodin||BOLD:AAU6327
Psectrocladius cf. limbatellus|Finnmark636|Torbjorn Ekrem||BOLD:AAG5500
Psectrocladius cf. limbatellus|Finnmark331|Torbjorn Ekrem||BOLD:AAG5500
Orthocladiinae|BIOUG05483-F11|Jerome Moriniere|BIN Taxonomy Match|BOLD:ACF7860
Eukiefferiella coerulescens|CCDB21606-F09|Sofia Wiedenbrug||BOLD:ACT7412
Eukiefferiella|CCDB24037-C12|Sofia Wiedenbrug|BIN Taxonomy Match|BOLD:ACU4437
[illegible]

Orthocladiinae|BIOUG05483-F11|Jerome Moriniere|BIN Taxonomy Match|BOLD:ACF7860
Eukiefferiella coerulescens|CCDB21606-F09|Sofia Wiedenbrug||BOLD:ACT7412
Eukiefferiella|CCDB24037-C12|Sofia Wiedenbrug|BIN Taxonomy Match|BOLD:ACU4437
Pseudorthocladius macrovirgatus|ZSM-DIP-33148-D12|Caroline Chimeno|BIN Taxonomy Match (Jan 202...
Pseudorthocladius macrovirgatus|ZSM-DIP-33148-D10|Caroline Chimeno|BIN Taxonomy Match (Jan 202...
Pseudorthocladius macrovirgatus|ZSM-DIP-33146-B03|Caroline Chimeno|BIN Taxonomy Match (Jan 202...
Pseudorthocladius macrovirgatus|ZSM-DIP-33146-B02|Caroline Chimeno|BIN Taxonomy Match (Jan 202...
Pseudorthocladius macrovirgatus|ZSM-DIP-33146-B01|Caroline Chimeno|BIN Taxonomy Match (Jan 202...
Pseudorthocladius macrovirgatus|ZSM-DIP-33146-A11|Caroline Chimeno|BIN Taxonomy Match (Jan 202...
Pseudorthocladius macrovirgatus|ZSM-DIP-33146-A10|Caroline Chimeno|BIN Taxonomy Match (Jan 202...
Pseudorthocladius macrovirgatus|ZSM-DIP-33148-E03|Caroline Chimeno|BIN Taxonomy Match (Jan 202...
Pseudorthocladius macrovirgatus|ZSM-DIP-33148-E01|Caroline Chimeno|BIN Taxonomy Match (Jan 202...
Pseudorthocladius macrovirgatus|ZSM-DIP-33145-F03|Caroline Chimeno|BIN Taxonomy Match (Jan 202...
Pseudorthocladius|BIOUG05946-D02|Kate Perez|BIN Taxonomy Match|BOLD:ACG2966
Pseudorthocladius|BIOUG05459-F12|Kate Perez|BIN Taxonomy Match|BOLD:ACF9730
Chaetocladius|BIOUG06068-E06|Kate Perez|BIN Taxonomy Match|BOLD:ACG4647
Chaetocladius|ZSM-DIP-33172-F09|Caroline Chimeno|BIN Taxonomy Match (Jan 2022)|BOLD:ACG4647
Chaetocladius|ZSM-DIP-33148-H08|Caroline Chimeno|BIN Taxonomy Match (Jan 2022)|BOLD:ACG4647
Chaetocladius|GBOL11503|Sofia Wiedenbrug||BOLD:ACG4647
Chaetocladius|BIOUG05946-A12|Kate Perez|BIN Taxonomy Match|BOLD:ACG4647
Chaetocladius|BIOUG05900-A07|Kate Perez|BIN Taxonomy Match|BOLD:ACG4647
Chaetocladius|BIOUG05900-A02|Kate Perez|BIN Taxonomy Match|BOLD:ACG4647
Chaetocladius|BIOUG05899-F12|Kate Perez|BIN Taxonomy Match|BOLD:ACG4647
Metriocnemus fuscipes|ZSM34343-G08|Sofia Wiedenbrug|Morphology|BOLD:AAI1573
Metriocnemus fuscipes|CCDB24038-E11|Sofia Wiedenbrug|BIN Taxonomy Match|BOLD:AAI1573
Metriocnemus fuscipes|GBOL11555|Sofia Wiedenbrug||BOLD:AAI1573
Metriocnemus fuscipes|GBOL11556|Sofia Wiedenbrug|Morphology|BOLD:AAI1573
Metriocnemus fuscipes|ZSM-DIP-33134-F03|Caroline Chimeno|BIN Taxonomy Match (Jan 2022)|BOLD...
Metriocnemus fuscipes|BIOUG16779-F11|Kate Perez|BIN Taxonomy Match|BOLD:AAI1573
Metriocnemus fuscipes|BIOUG16778-E11|Kate Perez|BIN Taxonomy Match|BOLD:AAI1573
Metriocnemus fuscipes|BIOUG16853-C01|Kate Perez|BIN Taxonomy Match|BOLD:AAI1573
Metriocnemus fuscipes|BIOUG16893-E09|Kate Perez|BIN Taxonomy Match|BOLD:AAI1573
Metriocnemus fuscipes|BIOUG16892-F09|Kate Perez|BIN Taxonomy Match|BOLD:AAI1573
Metriocnemus fuscipes|BIOUG42663-F12|Kate Perez|BIN Taxonomy Match below Phylum (Jun 2019)|...
Metriocnemus fuscipes|BIOUG16855-G03|Kate Perez|BIN Taxonomy Match|BOLD:AAI1573
Metriocnemus fuscipes|ZSM-DIP-33149-H09|Caroline Chimeno|BIN Taxonomy Match (Jan 2022)|BOLD...
Metriocnemus fuscipes|ZSM34341-G12|Sofia Wiedenbrug|Morphology|BOLD:AAI1573
Metriocnemus fuscipes|BIOUG16857-B01|Kate Perez|BIN Taxonomy Match|BOLD:AAI1573
Metriocnemus fuscipes|BIOUG16892-H01|Kate Perez|BIN Taxonomy Match|BOLD:AAI1573
Metriocnemus fuscipes|BIOUG16855-G06|Kate Perez|BIN Taxonomy Match|BOLD:AAI1573
Metriocnemus fuscipes|BIOUG16857-H02|Kate Perez|BIN Taxonomy Match|BOLD:AAI1573
Metriocnemus fuscipes|BIOUG16892-A04|Kate Perez|BIN Taxonomy Match|BOLD:AAI1573
Metriocnemus fuscipes|BIOUG16855-A03|Kate Perez|BIN Taxonomy Match|BOLD:AAI1573
Metriocnemus fuscipes|BIOUG16721-G03|Kate Perez|BIN Taxonomy Match|BOLD:AAI1573
Metriocnemus fuscipes|BIOUG16856-H05|Kate Perez|BIN Taxonomy Match|BOLD:AAI1573
Metriocnemus fuscipes|BIOUG16783-A03|Kate Perez|BIN Taxonomy Match|BOLD:AAI1573
Metriocnemus fuscipes|BIOUG15852-G05|Kate Perez|BIN Taxonomy Match|BOLD:AAI1573
Metriocnemus fuscipes|GBOL11498|Sofia Wiedenbrug|Morphology|BOLD:AAI1573
Metriocnemus fuscipes|CH-OSF117|Elisabeth Stur||BOLD:AAI1573
Metriocnemus fuscipes|BIOUG15458-A07|Kate Perez|BIN Taxonomy Match|BOLD:AAI1573
Metriocnemus fuscipes|TRD-CH119|Elisabeth Stur||BOLD:AAI1573
Metriocnemus fuscipes|TRD-CH35|Elisabeth Stur||BOLD:AAI1573
Metriocnemus fuscipes|ZMUO.025088|Lauri Paasivirta||BOLD:AAI1573
Metriocnemus fuscipes|BIOUG15458-C02|Kate Perez|BIN Taxonomy Match|BOLD:AAI1573
Metriocnemus fuscipes|BIOUG15460-H01|Kate Perez|BIN Taxonomy Match|BOLD:AAI1573
Metriocnemus fuscipes|BIOUG15184-G02|Kate Perez|BIN Taxonomy Match|BOLD:AAI1573
Metriocnemus fuscipes|BIOUG15457-D05|Kate Perez|BIN Taxonomy Match|BOLD:AAI1573
Metriocnemus fuscipes|BIOUG15183-H06|Kate Perez|BIN Taxonomy Match|BOLD:AAI1573
Metriocnemus fuscipes|BIOUG15543-E09|Kate Perez|BIN Taxonomy Match|BOLD:AAI1573
Metriocnemus fuscipes|BIOUG15184-C10|Kate Perez|BIN Taxonomy Match|BOLD:AAI1573
Metriocnemus fuscipes|BIOUG16625-C08|Kate Perez|BIN Taxonomy Match|BOLD:AAI1573
Metriocnemus fuscipes|BIOUG15184-A08|Kate Perez|BIN Taxonomy Match|BOLD:AAI1573
Metriocnemus fuscipes|BIOUG15457-D09|Kate Perez|BIN Taxonomy Match|BOLD:AAI1573
Metriocnemus fuscipes|BIOUG15183-C10|Kate Perez|BIN Taxonomy Match|BOLD:AAI1573
Metriocnemus fuscipes|BIOUG15183-F09|Kate Perez|BIN Taxonomy Match|BOLD:AAI1573
Metriocnemus fuscipes|BIOUG15459-A08|Kate Perez|BIN Taxonomy Match|BOLD:AAI1573
Metriocnemus fuscipes|BIOUG15459-C03|Kate Perez|BIN Taxonomy Match|BOLD:AAI1573
Metriocnemus fuscipes|BIOUG15460-B08|Kate Perez|BIN Taxonomy Match|BOLD:AAI1573
Metriocnemus fuscipes|BIOUG17025-F05|Kate Perez|BIN Taxonomy Match|BOLD:AAI1573
Metriocnemus fuscipes|BIOUG15544-B09|Kate Perez|BIN Taxonomy Match|BOLD:AAI1573
Metriocnemus fuscipes|BIOUG15536-C09|Kate Perez|BIN Taxonomy Match|BOLD:AAI1573
Metriocnemus fuscipes|BIOUG15948-C12|Kate Perez|BIN Taxonomy Match|BOLD:AAI1573
Metriocnemus fuscipes|ZSM-DIP-33134-F04|Caroline Chimeno|BIN Taxonomy Match (Jan 2022)|BOLD...
Metriocnemus fuscipes|NHRS-BYWS000000565|Yngve Brodin|morphology|BOLD:AAI1573
Metriocnemus fuscipes|BIOUG16892-G09|Kate Perez|BIN Taxonomy Match|BOLD:AAI1573
Metriocnemus fuscipes|ATNA536|Elisabeth Stur||BOLD:AAI1573
Metriocnemus fuscipes|BIOUG15592-E02|Kate Perez|BIN Taxonomy Match|BOLD:AAI1573
Metriocnemus fuscipes|NHRS-BYWS000000137|Yngve Brodin|morphology|BOLD:AAI1573
Metriocnemus fuscipes|ZMUO.025089|Lauri Paasivirta||BOLD:AAI1573
Metriocnemus fuscipes|BIOUG04261-D06|Kate Perez|BIN Taxonomy Match|BOLD:AAI1573
Metriocnemus fuscipes|BIOUG16893-D04|Kate Perez|BIN Taxonomy Match|BOLD:AAI1573
Metriocnemus fuscipes|BIOUG16856-H04|Kate Perez|BIN Taxonomy Match|BOLD:AAI1573
Metriocnemus fuscipes|BIOUG17117-H09|Kate Perez|BIN Taxonomy Match|BOLD:AAI1573
Metriocnemus fuscipes|BIOUG15458-G12|Kate Perez|BIN Taxonomy Match|BOLD:AAI1573
Metriocnemus fuscipes|BIOUG16435-B06|Kate Perez|BIN Taxonomy Match|BOLD:AAI1573
Metriocnemus fuscipes|BIOUG15855-A04|Kate Perez|BIN Taxonomy Match|BOLD:AAI1573
Metriocnemus fuscipes|BIOUG15184-H09|Kate Perez|BIN Taxonomy Match|BOLD:AAI1573
Metriocnemus fuscipes|BIOUG16893-H01|Kate Perez|BIN Taxonomy Match|BOLD:AAI1573
Metriocnemus fuscipes|RIN_CH80|Mona Renate Saursaunet|BOLD Identification engine|BOLD:AAI1573
Metriocnemus fuscipes|BIOUG15563-A10|Kate Perez|BIN Taxonomy Match|BOLD:AAI1573
Metriocnemus fuscipes|BIOUG15459-D12|Kate Perez|BIN Taxonomy Match|BOLD:AAI1573
Metriocnemus fuscipes|BIOUG15454-G08|Kate Perez|BIN Taxonomy Match|BOLD:AAI1573
Metriocnemus fuscipes|BIOUG15201-H08|Kate Perez|BIN Taxonomy Match|BOLD:AAI1573
Metriocnemus fuscipes|BIOUG15460-D08|Kate Perez|BIN Taxonomy Match|BOLD:AAI1573
Metriocnemus fuscipes|BIOUG15459-C02|Kate Perez|BIN Taxonomy Match|BOLD:AAI1573
Metriocnemus fuscipes|BIOUG15809-B02|Kate Perez|BIN Taxonomy Match|BOLD:AAI1573
Metriocnemus fuscipes|BIOUG15537-D04|Kate Perez|BIN Taxonomy Match|BOLD:AAI1573
Metriocnemus fuscipes|Finnmark545|Elisabeth Stur||BOLD:AAI1573
Metriocnemus fuscipes|CH-eik54|Elisabeth Stur||BOLD:AAD0838
Metriocnemus fuscipes|ATNA198|Elisabeth Stur||BOLD:AAD0838
Metriocnemus fuscipes|ATNA232|Elisabeth Stur||BOLD:AAD0838
Metriocnemus fuscipes|Finnmark479|Elisabeth Stur||BOLD:AAD0838
Guttipelopia guttipennis|ZMUO.025094|Lauri Paasivirta||BOLD:AAW5657

Metriocnemus fuscipes|ATNA232|Elisabeth Stur||BOLD:AAD0838
Metriocnemus fuscipes|Finnmark479|Elisabeth Stur||BOLD:AAD0838
Guttipelopia guttipennis|ZMUO.025094|Lauri Paasivirta||BOLD:AAW5657
Guttipelopia guttipennis|TRD-CH366|Elisabeth Stur|morphology|BOLD:AAW5657
Guttipelopia guttipennis|TRD-CH365|Elisabeth Stur|morphology|BOLD:AAW5657
Guttipelopia guttipennis|TRD-CH6|Elisabeth Stur||BOLD:AAW5657
Guttipelopia guttipennis|RIN_CH76|Mona Renate Saursaunet|BOLD Identification engine|BOLD:AAW5657
Guttipelopia guttipennis|RIN_CH75|Mona Renate Saursaunet|BOLD Identification engine|BOLD:AAW5657
Guttipelopia guttipennis|RIN_CH74|Mona Renate Saursaunet|BOLD Identification engine|BOLD:AAW5657
Guttipelopia guttipennis|RIN_CH73|Mona Renate Saursaunet|BOLD Identification engine|BOLD:AAW5657
Guttipelopia guttipennis|RIN_CH33|Mona Renate Saursaunet|BOLD Identification engine|BOLD:AAW5657
Guttipelopia guttipennis|TRD-CH32|Elisabeth Stur||BOLD:AAW5657
Guttipelopia guttipennis|ZMUO.025095|Lauri Paasivirta||BOLD:AAW5657
Guttipelopia guttipennis|EJV-20110020|Lauri Paasivirta||BOLD:AAW5657
Guttipelopia guttipennis|EJV-20110018|Lauri Paasivirta||BOLD:AAW5657
Chironomidae|BIOUG42676-A12|Kate Perez|BOLD ID Engine Manual below Phylum (Jul 2019)|BOLD ...
Rheopelopia ornata|ZMUO.025205|Lauri Paasivirta||BOLD:ADA5174
Rheopelopia ornata|ZMUO.025204|Lauri Paasivirta||BOLD:ADA4590
Xenopelopia falcigera|NHRS-BYWS000000329|Yngve Brodin|morphology|BOLD:ADJ7967
Xenopelopia falcigera|NHRS-BYWS000000328|Yngve Brodin|morphology|BOLD:ADJ7967
Xenopelopia nigricans|ZMUO.024659|Lauri Paasivirta||BOLD:ADA6313
Xenopelopia nigricans|ZSM-DIP-33139-H02|Caroline Chimeno|BIN Taxonomy Match (Jan 2022)|BOLD...
Xenopelopia nigricans|ZMUO.024658|Lauri Paasivirta||BOLD:ADA6313
Xenopelopia nigricans|NHRS-BYWS000000455|Yngve Brodin|morphology|BOLD:ADA6313
Krenopelopia|Finnmark703|Elisabeth Stur||BOLD:AAC9199
Krenopelopia|CCDB22605-A10|Sofia Wiedenbrug|BIN Taxonomy Match|BOLD:AAZ1682
Krenopelopia sp.|ZSM-DIP-33149-F11|Caroline Chimeno|BIN Taxonomy Match (Jan 2022)|BOLD:A...
Krenopelopia sp.|ES295|Elisabeth Stur||BOLD:AAZ1682
Krenopelopia|CCDB21606-G01|Sofia Wiedenbrug|BIN Taxonomy Match|BOLD:ACT5787
Krenopelopia|CH-eik26|Elisabeth Stur||BOLD:AAI2213
Krenopelopia|ZSM34341-D10|Sofia Wiedenbrug|Morphology|BOLD:AAI2213
Krenopelopia|BIOUG05898-E07|Kate Perez|BIN Taxonomy Match|BOLD:AAI2213
Krenopelopia sp.|SOE158|Elisabeth Stur||BOLD:AAI2213
Krenopelopia sp.|SOE151|Elisabeth Stur||BOLD:AAI2213
Krenopelopia|CH-eik134|Elisabeth Stur||BOLD:AAI2213
Krenopelopia sp.|SOE157|Elisabeth Stur||BOLD:AAI2213
Krenopelopia|ATNA332|Elisabeth Stur||BOLD:AAI2213
Krenopelopia|CH-eik27|Elisabeth Stur||BOLD:AAI2213
Krenopelopia|CCDB24183-D05|Sofia Wiedenbrug|Morphology|BOLD:AAI2213
Krenopelopia|CCDB21606-E05|Sofia Wiedenbrug|BIN Taxonomy Match|BOLD:AAI2213
Krenopelopia binotata|ZSM-DIP-33138-E10|Caroline Chimeno|BIN Taxonomy Match (Jan 2022)|BOLD...
Krenopelopia binotata|ZMUO.025847|Lauri Paasivirta||BOLD:AAI2213
Krenopelopia binotata|ZMUO.025848|Lauri Paasivirta||BOLD:AAI2213
Krenopelopia binotata|ZSM-DIP-33143-D05|Caroline Chimeno|BIN Taxonomy Match (Jan 2022)|BOLD...
Krenopelopia binotata|ZSM-DIP-33137-A03|Caroline Chimeno|BIN Taxonomy Match (Jan 2022)|BOLD...
Krenopelopia|ATNA337|Elisabeth Stur||BOLD:AAI2213
Krenopelopia|BIOUG05217-F06|Kate Perez|BIN Taxonomy Match|BOLD:AAI2213
Krenopelopia|BIOUG36655-A02|Kate Perez|BIN Taxonomy Match (Jul 2018)|BOLD:AAI2213
Krenopelopia|BIOUG36932-A12|Kate Perez|BIN Taxonomy Match (Jul 2018)|BOLD:AAI2213
Krenopelopia|BIOUG36775-H11|Kate Perez|BIN Taxonomy Match (Jul 2018)|BOLD:AAI2213
Krenopelopia|CCDB24228-C07|Sofia Wiedenbrug|BIN Taxonomy Match|BOLD:AAI2213
Krenopelopia|CCDB24228-H08|Sofia Wiedenbrug|BIN Taxonomy Match|BOLD:AAI2213
Krenopelopia sp.|SOE42|Elisabeth Stur
Krenopelopia|CCDB22603-E01|Sofia Wiedenbrug||BOLD:ACQ3154
Krenopelopia sp.|SOE487|Torbjorn Ekrem
Krenopelopia|BIOUG05776-H05|Kate Perez|BIN Taxonomy Match|BOLD:AAI2213
Krenopelopia|BIOUG06051-E03|Kate Perez|BIN Taxonomy Match|BOLD:AAI2213
Krenopelopia|BIOUG06055-H11|Kate Perez|BIN Taxonomy Match|BOLD:AAI2213
Krenopelopia sp.|ZSM34342-E11|Sofia Wiedenbrug|Morphology|BOLD:AAC9198
Krenopelopia sp.|CCDB24183-H02|Sofia Wiedenbrug|BIN-taxonomy|BOLD:AAC9198
Krenopelopia sp.|CCDB24183-C04|Sofia Wiedenbrug|Morphology|BOLD:AAC9198
Krenopelopia|CH-eik106|Elisabeth Stur||BOLD:AAC9198
Krenopelopia|ATNA336|Elisabeth Stur||BOLD:AAC9198
Krenopelopia sp.|CCDB24183-B12|Sofia Wiedenbrug|BIN-taxonomy|BOLD:AAC9198
Krenopelopia sp.|CCDB24183-H01|Sofia Wiedenbrug|Morphology|BOLD:AAC9198
Krenopelopia|ATNA557|Elisabeth Stur||BOLD:AAC9198
Krenopelopia sp.|CCDB21606-H04|Sofia Wiedenbrug|BIN Taxonomy Match|BOLD:AAC9198
Krenopelopia sp.|CCDB21606-H07|Sofia Wiedenbrug|BIN Taxonomy Match|BOLD:AAC9198
Krenopelopia sp.|SOE82|Torbjorn Ekrem||BOLD:AAC9198
Krenopelopia|CH-OSF34|Torbjorn Ekrem||BOLD:AAC9198
Krenopelopia|ATNA73|Elisabeth Stur||BOLD:AAC9198
Krenopelopia binotata|ZMUO.024796|Lauri Paasivirta||BOLD:AAC9198
Krenopelopia binotata|ZMUO.024797|Lauri Paasivirta||BOLD:AAC9198
Krenopelopia sp.|BIOUG05898-E11|Kate Perez|BIN Taxonomy Match|BOLD:AAC9198
Krenopelopia sp.|BIOUG06053-D01|Kate Perez|BIN Taxonomy Match|BOLD:AAC9198
Krenopelopia sp.|BIOUG06052-B01|Kate Perez|BIN Taxonomy Match|BOLD:AAC9198
Krenopelopia sp.|BIOUG06051-H10|Kate Perez|BIN Taxonomy Match|BOLD:AAC9198
Krenopelopia sp.|ES76|Elisabeth Stur||BOLD:AAC9196
Krenopelopia|BIOUG07509-H03|Kate Perez|BIN Taxonomy Match|BOLD:AAC9196
Krenopelopia|BIOUG06052-F05|Kate Perez|BIN Taxonomy Match|BOLD:AAC9196
Krenopelopia sp.|SOE216|Elisabeth Stur||BOLD:AAC9197
Krenopelopia|CH-OSF173|Elisabeth Stur||BOLD:AAC9197
Krenopelopia sp.|SOE17|Elisabeth Stur||BOLD:AAC9197
Krenopelopia|BIOUG05947-B07|Kate Perez|BIN Taxonomy Match|BOLD:AAC9197
Trissopelopia longimanus|ZMUO.025295|Lauri Paasivirta||BOLD:AAW7942
Trissopelopia longimanus|ZMUO.025294|Lauri Paasivirta||BOLD:AAW7942
Trissopelopia longimanus|BIOUG43948-A02|Kate Perez|BIN Taxonomy Match below Phylum (Jan 2019 ...
Trissopelopia longimanus|Finnmark783|Elisabeth Stur||BOLD:AAW7942
Trissopelopia longimanus|SOE273|Torbjorn Ekrem||BOLD:AAW7942
Trissopelopia cf. flavida|Finnmark784|Elisabeth Stur||BOLD:AAE7957
Trissopelopia cf. flavida|SOE385|Elisabeth Stur||BOLD:AAE7957
Trissopelopia cf. flavida|ES199|Elisabeth Stur||BOLD:AAE7957
Trissopelopia cf. flavida|Finnmark888|Elisabeth Stur||BOLD:AAE7957
Trissopelopia cf. flavida|ES200|Elisabeth Stur||BOLD:AAE7957
Trissopelopia cf. flavida|SOE268|Elisabeth Stur||BOLD:AAE7957
Arctopelopia griseipennis|ZMUO.026161|Lauri Paasivirta||BOLD:ACQ1819
Arctopelopia griseipennis|ZMUO.026160|Lauri Paasivirta||BOLD:ACQ1819
Arctopelopia griseipennis|NHRS-BYWS000001179|Yngve Brodin|morphology|BOLD:ACQ1819
Arctopelopia griseipennis|NHRS-BYWS000001173|Yngve Brodin|morphology|BOLD:ACQ1819
Arctopelopia griseipennis|NHRS-BYWS000000128|Yngve Brodin|morphology|BOLD:ACQ1819
Tanypodinae|GBOL03710|Jerome Moriniere|BIN Taxonomy Match|BOLD:ACQ1819
Chironomidae|ZSM-DIP-33145-G05|Valerie Levesque-Beaudin|BOLD ID Engine Manual (Sep 2021)|BO...
Chironomidae|ZSM-DIP-33128-H04|Valerie Levesque-Beaudin|BIN Taxonomy Match (Sep 2021)|BOLD...

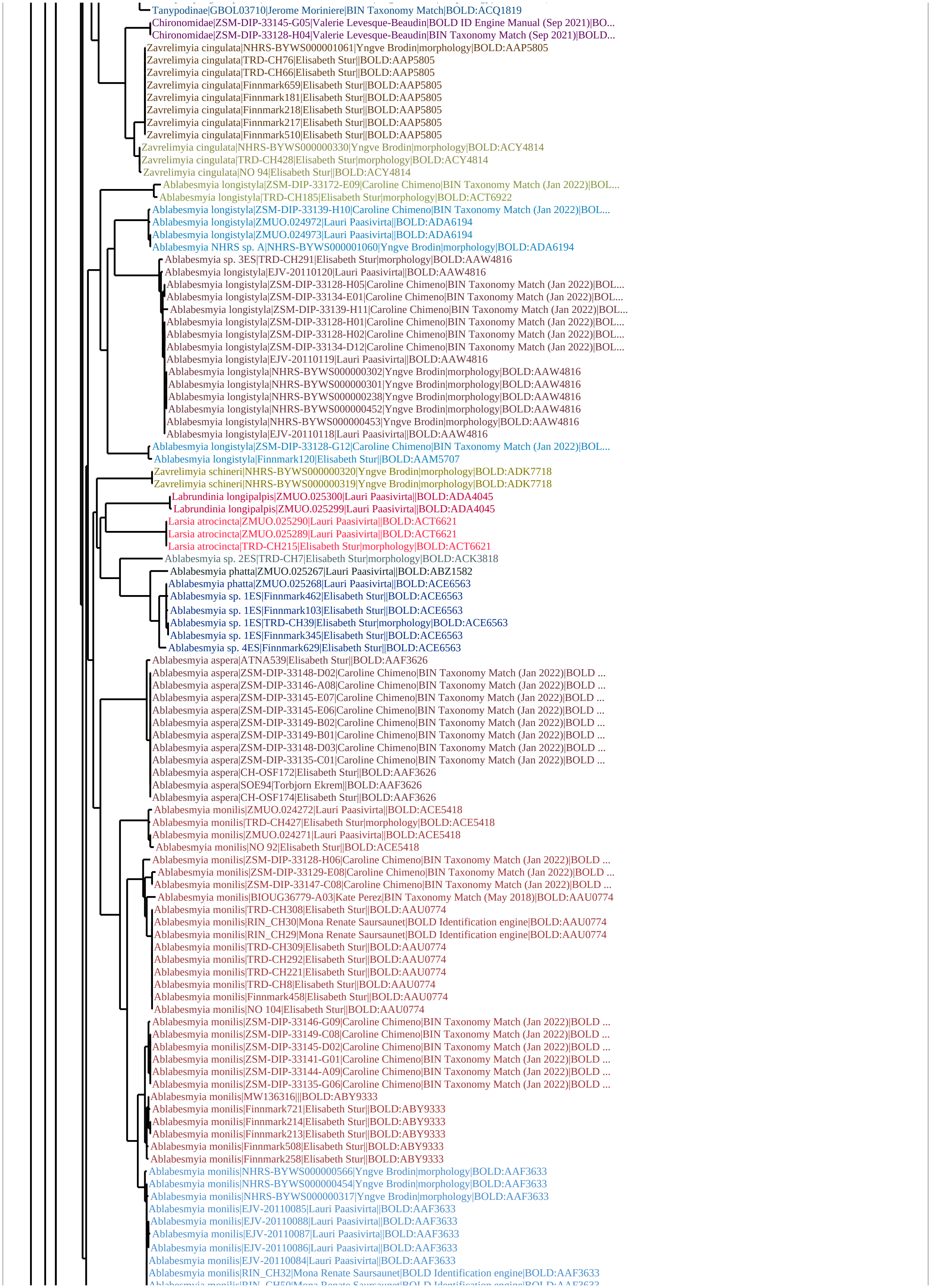
Tanypodinae|GBOL03710|Jerome Moriniere|BIN Taxonomy Match|BOLD:ACQ1819
Chironomidae|ZSM-DIP-33145-G05|Valerie Levesque-Beaudin|BOLD ID Engine Manual (Sep 2021)|BO...
Chironomidae|ZSM-DIP-33128-H04|Valerie Levesque-Beaudin|BIN Taxonomy Match (Sep 2021)|BOLD...
Zavrelimyia cingulata|NHRS-BYWS000001061|Yngve Brodin|morphology|BOLD:AAP5805
Zavrelimyia cingulata|TRD-CH76|Elisabeth Stur||BOLD:AAP5805
Zavrelimyia cingulata|TRD-CH66|Elisabeth Stur||BOLD:AAP5805
Zavrelimyia cingulata|Finnmark659|Elisabeth Stur||BOLD:AAP5805
Zavrelimyia cingulata|Finnmark181|Elisabeth Stur||BOLD:AAP5805
Zavrelimyia cingulata|Finnmark218|Elisabeth Stur||BOLD:AAP5805
Zavrelimyia cingulata|Finnmark217|Elisabeth Stur||BOLD:AAP5805
Zavrelimyia cingulata|Finnmark510|Elisabeth Stur||BOLD:AAP5805
Zavrelimyia cingulata|NHRS-BYWS000000330|Yngve Brodin|morphology|BOLD:ACY4814
Zavrelimyia cingulata|TRD-CH428|Elisabeth Stur|morphology|BOLD:ACY4814
Zavrelimyia cingulata|NO 94|Elisabeth Stur||BOLD:ACY4814
Ablabesmyia longistyla|ZSM-DIP-33172-E09|Caroline Chimeno|BIN Taxonomy Match (Jan 2022)|BOL...
Ablabesmyia longistyla|TRD-CH185|Elisabeth Stur|morphology|BOLD:ACT6922
Ablabesmyia longistyla|ZSM-DIP-33139-H10|Caroline Chimeno|BIN Taxonomy Match (Jan 2022)|BOL...
Ablabesmyia longistyla|ZMUO.024972|Lauri Paasivirta||BOLD:ADA6194
Ablabesmyia longistyla|ZMUO.024973|Lauri Paasivirta||BOLD:ADA6194
Ablabesmyia NHRS sp. A|NHRS-BYWS000001060|Yngve Brodin|morphology|BOLD:ADA6194
Ablabesmyia sp. 3ES|TRD-CH291|Elisabeth Stur|morphology|BOLD:AAW4816
Ablabesmyia longistyla|EJV-20110120|Lauri Paasivirta||BOLD:AAW4816
Ablabesmyia longistyla|ZSM-DIP-33128-H05|Caroline Chimeno|BIN Taxonomy Match (Jan 2022)|BOL...
Ablabesmyia longistyla|ZSM-DIP-33134-E01|Caroline Chimeno|BIN Taxonomy Match (Jan 2022)|BOL...
Ablabesmyia longistyla|ZSM-DIP-33139-H11|Caroline Chimeno|BIN Taxonomy Match (Jan 2022)|BOL...
Ablabesmyia longistyla|ZSM-DIP-33128-H01|Caroline Chimeno|BIN Taxonomy Match (Jan 2022)|BOL...
Ablabesmyia longistyla|ZSM-DIP-33128-H02|Caroline Chimeno|BIN Taxonomy Match (Jan 2022)|BOL...
Ablabesmyia longistyla|ZSM-DIP-33134-D12|Caroline Chimeno|BIN Taxonomy Match (Jan 2022)|BOL...
Ablabesmyia longistyla|EJV-20110119|Lauri Paasivirta||BOLD:AAW4816
Ablabesmyia longistyla|NHRS-BYWS000000302|Yngve Brodin|morphology|BOLD:AAW4816
Ablabesmyia longistyla|NHRS-BYWS000000301|Yngve Brodin|morphology|BOLD:AAW4816
Ablabesmyia longistyla|NHRS-BYWS000000238|Yngve Brodin|morphology|BOLD:AAW4816
Ablabesmyia longistyla|NHRS-BYWS000000452|Yngve Brodin|morphology|BOLD:AAW4816
Ablabesmyia longistyla|NHRS-BYWS000000453|Yngve Brodin|morphology|BOLD:AAW4816
Ablabesmyia longistyla|EJV-20110118|Lauri Paasivirta||BOLD:AAW4816
Ablabesmyia longistyla|ZSM-DIP-33128-G12|Caroline Chimeno|BIN Taxonomy Match (Jan 2022)|BOL...
Ablabesmyia longistyla|Finnmark120|Elisabeth Stur||BOLD:AAM5707
Zavrelimyia schineri|NHRS-BYWS000000320|Yngve Brodin|morphology|BOLD:ADK7718
Zavrelimyia schineri|NHRS-BYWS000000319|Yngve Brodin|morphology|BOLD:ADK7718
Labrundinia longipalpis|ZMUO.025300|Lauri Paasivirta||BOLD:ADA4045
Labrundinia longipalpis|ZMUO.025299|Lauri Paasivirta||BOLD:ADA4045
Larsia atrocincta|ZMUO.025290|Lauri Paasivirta||BOLD:ACT6621
Larsia atrocincta|ZMUO.025289|Lauri Paasivirta||BOLD:ACT6621
Larsia atrocincta|TRD-CH215|Elisabeth Stur|morphology|BOLD:ACT6621
Ablabesmyia sp. 2ES|TRD-CH7|Elisabeth Stur|morphology|BOLD:ACK3818
Ablabesmyia phatta|ZMUO.025267|Lauri Paasivirta||BOLD:ABZ1582
Ablabesmyia phatta|ZMUO.025268|Lauri Paasivirta||BOLD:ACE6563
Ablabesmyia sp. 1ES|Finnmark462|Elisabeth Stur||BOLD:ACE6563
Ablabesmyia sp. 1ES|Finnmark103|Elisabeth Stur||BOLD:ACE6563
Ablabesmyia sp. 1ES|TRD-CH39|Elisabeth Stur|morphology|BOLD:ACE6563
Ablabesmyia sp. 1ES|Finnmark345|Elisabeth Stur||BOLD:ACE6563
Ablabesmyia sp. 4ES|Finnmark629|Elisabeth Stur||BOLD:ACE6563
Ablabesmyia aspera|ATNA539|Elisabeth Stur||BOLD:AAF3626
Ablabesmyia aspera|ZSM-DIP-33148-D02|Caroline Chimeno|BIN Taxonomy Match (Jan 2022)|BOLD ...
Ablabesmyia aspera|ZSM-DIP-33146-A08|Caroline Chimeno|BIN Taxonomy Match (Jan 2022)|BOLD ...
Ablabesmyia aspera|ZSM-DIP-33145-E07|Caroline Chimeno|BIN Taxonomy Match (Jan 2022)|BOLD ...
Ablabesmyia aspera|ZSM-DIP-33145-E06|Caroline Chimeno|BIN Taxonomy Match (Jan 2022)|BOLD ...
Ablabesmyia aspera|ZSM-DIP-33149-B02|Caroline Chimeno|BIN Taxonomy Match (Jan 2022)|BOLD ...
Ablabesmyia aspera|ZSM-DIP-33149-B01|Caroline Chimeno|BIN Taxonomy Match (Jan 2022)|BOLD ...
Ablabesmyia aspera|ZSM-DIP-33148-D03|Caroline Chimeno|BIN Taxonomy Match (Jan 2022)|BOLD ...
Ablabesmyia aspera|ZSM-DIP-33135-C01|Caroline Chimeno|BIN Taxonomy Match (Jan 2022)|BOLD ...
Ablabesmyia aspera|CH-OSF172|Elisabeth Stur||BOLD:AAF3626
Ablabesmyia aspera|SOE94|Torbjorn Ekrem||BOLD:AAF3626
Ablabesmyia aspera|CH-OSF174|Elisabeth Stur||BOLD:AAF3626
Ablabesmyia monilis|ZMUO.024272|Lauri Paasivirta||BOLD:ACE5418
Ablabesmyia monilis|TRD-CH427|Elisabeth Stur|morphology|BOLD:ACE5418
Ablabesmyia monilis|ZMUO.024271|Lauri Paasivirta||BOLD:ACE5418
Ablabesmyia monilis|NO 92|Elisabeth Stur||BOLD:ACE5418
Ablabesmyia monilis|ZSM-DIP-33128-H06|Caroline Chimeno|BIN Taxonomy Match (Jan 2022)|BOLD ...
Ablabesmyia monilis|ZSM-DIP-33129-E08|Caroline Chimeno|BIN Taxonomy Match (Jan 2022)|BOLD ...
Ablabesmyia monilis|ZSM-DIP-33147-C08|Caroline Chimeno|BIN Taxonomy Match (Jan 2022)|BOLD ...
Ablabesmyia monilis|BIOUG36779-A03|Kate Perez|BIN Taxonomy Match (May 2018)|BOLD:AAU0774
Ablabesmyia monilis|TRD-CH308|Elisabeth Stur||BOLD:AAU0774
Ablabesmyia monilis|RIN_CH30|Mona Renate Saursaunet|BOLD Identification engine|BOLD:AAU0774
Ablabesmyia monilis|RIN_CH29|Mona Renate Saursaunet|BOLD Identification engine|BOLD:AAU0774
Ablabesmyia monilis|TRD-CH309|Elisabeth Stur||BOLD:AAU0774
Ablabesmyia monilis|TRD-CH292|Elisabeth Stur||BOLD:AAU0774
Ablabesmyia monilis|TRD-CH221|Elisabeth Stur||BOLD:AAU0774
Ablabesmyia monilis|TRD-CH8|Elisabeth Stur||BOLD:AAU0774
Ablabesmyia monilis|Finnmark458|Elisabeth Stur||BOLD:AAU0774
Ablabesmyia monilis|NO 104|Elisabeth Stur||BOLD:AAU0774
Ablabesmyia monilis|ZSM-DIP-33146-G09|Caroline Chimeno|BIN Taxonomy Match (Jan 2022)|BOLD ...
Ablabesmyia monilis|ZSM-DIP-33149-C08|Caroline Chimeno|BIN Taxonomy Match (Jan 2022)|BOLD ...
Ablabesmyia monilis|ZSM-DIP-33145-D02|Caroline Chimeno|BIN Taxonomy Match (Jan 2022)|BOLD ...
Ablabesmyia monilis|ZSM-DIP-33141-G01|Caroline Chimeno|BIN Taxonomy Match (Jan 2022)|BOLD ...
Ablabesmyia monilis|ZSM-DIP-33144-A09|Caroline Chimeno|BIN Taxonomy Match (Jan 2022)|BOLD ...
Ablabesmyia monilis|ZSM-DIP-33135-G06|Caroline Chimeno|BIN Taxonomy Match (Jan 2022)|BOLD ...
Ablabesmyia monilis|MW136316|||BOLD:ABY9333
Ablabesmyia monilis|Finnmark721|Elisabeth Stur||BOLD:ABY9333
Ablabesmyia monilis|Finnmark214|Elisabeth Stur||BOLD:ABY9333
Ablabesmyia monilis|Finnmark213|Elisabeth Stur||BOLD:ABY9333
Ablabesmyia monilis|Finnmark508|Elisabeth Stur||BOLD:ABY9333
Ablabesmyia monilis|Finnmark258|Elisabeth Stur||BOLD:ABY9333
Ablabesmyia monilis|NHRS-BYWS000000566|Yngve Brodin|morphology|BOLD:AAF3633
Ablabesmyia monilis|NHRS-BYWS000000454|Yngve Brodin|morphology|BOLD:AAF3633
Ablabesmyia monilis|NHRS-BYWS000000317|Yngve Brodin|morphology|BOLD:AAF3633
Ablabesmyia monilis|EJV-20110085|Lauri Paasivirta||BOLD:AAF3633
Ablabesmyia monilis|EJV-20110088|Lauri Paasivirta||BOLD:AAF3633
Ablabesmyia monilis|EJV-20110087|Lauri Paasivirta||BOLD:AAF3633
Ablabesmyia monilis|EJV-20110086|Lauri Paasivirta||BOLD:AAF3633
Ablabesmyia monilis|EJV-20110084|Lauri Paasivirta||BOLD:AAF3633
Ablabesmyia monilis|RIN_CH32|Mona Renate Saursaunet|BOLD Identification engine|BOLD:AAF3633

Ablabesmyia monilis|EJV-2011008[illegible]|Lauri Paasivirta||BOLD:AAF3633
Ablabesmyia monilis|EJV-20110084|Lauri Paasivirta||BOLD:AAF3633
Ablabesmyia monilis|RIN_CH32|Mona Renate Saursaunet|BOLD Identification engine|BOLD:AAF3633
Ablabesmyia monilis|RIN_CH50|Mona Renate Saursaunet|BOLD Identification engine|BOLD:AAF3633
Ablabesmyia monilis|ATNA330|Elisabeth Stur||BOLD:AAF3633
Ablabesmyia monilis|TRD-CH158|Elisabeth Stur|morphology|BOLD:AAF3633
Ablabesmyia monilis|NHRS-BYWS000000445|Yngve Brodin|morphology|BOLD:AAF3633
Ablabesmyia monilis|NHRS-BYWS000000318|Yngve Brodin|morphology|BOLD:AAF3633
Ablabesmyia monilis|ATNA538|Elisabeth Stur||BOLD:AAF3633
Ablabesmyia monilis|NHRS-BYWS000000190|Yngve Brodin|morphology|BOLD:AAF3633
Ablabesmyia monilis|RIN_CH34|Mona Renate Saursaunet|BOLD Identification engine|BOLD:AAF3633
Ablabesmyia monilis|RIN_CH15|Mona Renate Saursaunet|BOLD Identification engine|BOLD:AAF3633
Ablabesmyia monilis|TRD-CH325|Elisabeth Stur||BOLD:AAF3633
Ablabesmyia monilis|SOE9|Elisabeth Stur||BOLD:AAF3633
Ablabesmyia monilis|RIN_CH13|Mona Renate Saursaunet|BOLD Identification engine|BOLD:AAF3633
Ablabesmyia monilis|SOE10|Elisabeth Stur||BOLD:AAF3633
Ablabesmyia monilis|Finnmark568|Elisabeth Stur||BOLD:AAF3633
Telopelopia fascigera|ZMUO.026154|Lauri Paasivirta||BOLD:ADF0409
Telopelopia fascigera|ZMUO.026153|Lauri Paasivirta||BOLD:ADF0409
Rheopelopia sp. 1ES|ATNA371|Elisabeth Stur||BOLD:AAG2945
Rheopelopia sp. 1ES|TRD-CH324|Elisabeth Stur||BOLD:AAG2945
Rheopelopia sp. 1ES|BIOUG16328-C02|Kate Perez|BIN Taxonomy Match|BOLD:AAG2945
Rheopelopia sp. 1ES|BIOUG16435-D10|Kate Perez|BIN Taxonomy Match|BOLD:AAG2945
Rheopelopia sp. 1ES|BIOUG16258-C10|Kate Perez|BIN Taxonomy Match|BOLD:AAG2945
Rheopelopia sp. 1ES|BIOUG16477-E09|Kate Perez|BIN Taxonomy Match|BOLD:AAG2945
Rheopelopia sp. 1ES|BIOUG15953-E06|Elisabeth Stur|BIN Taxonomy Match|BOLD:AAG2945
Rheopelopia sp. 1ES|BIOUG15952-E01|Kate Perez|BIN Taxonomy Match|BOLD:AAG2945
Rheopelopia sp. 1ES|BIOUG15952-C03|Kate Perez|BIN Taxonomy Match|BOLD:AAG2945
Rheopelopia sp. 1ES|BIOUG15949-B04|Kate Perez|BIN Taxonomy Match|BOLD:AAG2945
Rheopelopia sp. 1ES|Finnmark270|Elisabeth Stur||BOLD:AAG2945
Telmatopelopia nemorum|ZMUO.024805|Lauri Paasivirta||BOLD:ADA5560
Telmatopelopia nemorum|ZMUO.024806|Lauri Paasivirta||BOLD:ADA5560
Telmatopelopia nemorum|NHRS-BYWS000001131|Yngve Brodin|morphology|BOLD:ADA5560
Chironomidae|ZSM-DIP-33129-F06|Valerie Levesque-Beaudin|BIN Taxonomy Match (Sep 2021)|BOLD...
Chironomidae|ZSM-DIP-33129-E11|Valerie Levesque-Beaudin|BIN Taxonomy Match (Sep 2021)|BOLD...
Chironomidae|ZSM-DIP-33149-E04|Valerie Levesque-Beaudin|BIN Taxonomy Match (Sep 2021)|BOLD...
Chironomidae|ZSM-DIP-33144-E10|Valerie Levesque-Beaudin|BIN Taxonomy Match (Sep 2021)|BOLD...
Chironomidae|ZSM-DIP-33139-A04|Valerie Levesque-Beaudin|BIN Taxonomy Match (Sep 2021)|BOLD...
Chironomidae|ZSM-DIP-33144-G12|Valerie Levesque-Beaudin|BIN Taxonomy Match (Sep 2021)|BOLD...
Chironomidae|ZSM-DIP-33149-E05|Valerie Levesque-Beaudin|BIN Taxonomy Match (Sep 2021)|BOLD...
Chironomidae|ZSM-DIP-33147-D06|Valerie Levesque-Beaudin|BIN Taxonomy Match (Sep 2021)|BOLD...
Chironomidae|ZSM-DIP-33147-D05|Valerie Levesque-Beaudin|BIN Taxonomy Match (Sep 2021)|BOLD...
Chironomidae|ZSM-DIP-33144-E05|Valerie Levesque-Beaudin|BIN Taxonomy Match (Sep 2021)|BOLD...
Chironomidae|ZSM-DIP-33128-A04|Valerie Levesque-Beaudin|BIN Taxonomy Match (Sep 2021)|BOLD...
Chironomidae|ZSM-DIP-33144-E04|Valerie Levesque-Beaudin|BIN Taxonomy Match (Sep 2021)|BOLD...
Chironomidae|ZSM-DIP-33147-F03|Valerie Levesque-Beaudin|BIN Taxonomy Match (Sep 2021)|BOLD...
Chironomidae|ZSM-DIP-33144-B11|Valerie Levesque-Beaudin|BOLD ID Engine Manual (Sep 2021)|BO...
Zavrelimyia nr. barbatipes|ZMUO.024601|Lauri Paasivirta||BOLD:ACM5292
Zavrelimyia nr. barbatipes|ZMUO.024600|Lauri Paasivirta||BOLD:ACM5292
Zavrelimyia nr. melanura|ZMUO.024893|Lauri Paasivirta||BOLD:ACM5292
Zavrelimyia sp. 3ES|TRD-CH75|Elisabeth Stur|morphology|BOLD:ACM5292
Zavrelimyia nr. barbatipes|ZMUO.024891|Lauri Paasivirta||BOLD:AAV2699
Zavrelimyia nr. barbatipes|ZSM-DIP-33172-E08|Caroline Chimeno|BIN Taxonomy Match (Jan 2022)...
Zavrelimyia|ZSM-DIP-33172-E07|Caroline Chimeno|BIN Taxonomy Match (Jan 2022)|BOLD:AAV2699
Zavrelimyia nr. barbatipes|ZSM-DIP-33172-E06|Caroline Chimeno|BIN Taxonomy Match (Jan 2022)...
Zavrelimyia sp. 1ES|TRD-CH393|Elisabeth Stur|morphology|BOLD:AAV2699
Zavrelimyia|BIOUG43949-H04|Kate Perez|BIN Taxonomy Match below Phylum (Jan 2019)|BOLD:AA...
Zavrelimyia sp. 1ES|Finnmark754|G. A. Halvorsen||BOLD:AAV2699
Zavrelimyia sp. 1ES|CH-OSF116|Elisabeth Stur||BOLD:AAV2699
Zavrelimyia sp. 2ES|SOE272|Elisabeth Stur||BOLD:AAG2011
Zavrelimyia nr. barbatipes|ZMUO.024890|Lauri Paasivirta||BOLD:AAG2011
Zavrelimyia sp. 2ES|CH-OSF163|Elisabeth Stur||BOLD:AAG2011
Zavrelimyia sp. 2ES|SOE271|Elisabeth Stur||BOLD:AAG2011
Zavrelimyia melanura|ZSM-DIP-33149-D02|Caroline Chimeno|BIN Taxonomy Match (Jan 2022)|BOLD...
Zavrelimyia melanura|ZSM-DIP-33130-F06|Caroline Chimeno|BIN Taxonomy Match (Jan 2022)|BOLD...
Zavrelimyia melanura|ZSM-DIP-33145-A08|Caroline Chimeno|BIN Taxonomy Match (Jan 2022)|BOLD...
Zavrelimyia melanura|ZSM-DIP-33143-B07|Caroline Chimeno|BIN Taxonomy Match (Jan 2022)|BOLD...
Zavrelimyia nr. melanura|ZMUO.024892|Lauri Paasivirta||BOLD:AAD8015
Zavrelimyia nr. melanura|ZMUO.024786|Lauri Paasivirta||BOLD:AAD8015
Zavrelimyia nr. melanura|ZMUO.024787|Lauri Paasivirta||BOLD:AAD8015
Zavrelimyia melanura|ZSM-DIP-33130-H03|Caroline Chimeno|BIN Taxonomy Match (Jan 2022)|BOLD...
Zavrelimyia melanura|ZSM-DIP-33130-H02|Caroline Chimeno|BIN Taxonomy Match (Jan 2022)|BOLD...
Zavrelimyia melanura|ZSM-DIP-33145-D03|Caroline Chimeno|BIN Taxonomy Match (Jan 2022)|BOLD...
Zavrelimyia melanura|ZSM-DIP-33141-G02|Caroline Chimeno|BIN Taxonomy Match (Jan 2022)|BOLD...
Zavrelimyia melanura|ZSM-DIP-33135-G08|Caroline Chimeno|BIN Taxonomy Match (Jan 2022)|BOLD...
Zavrelimyia melanura|ZSM-DIP-33143-B08|Caroline Chimeno|BIN Taxonomy Match (Jan 2022)|BOLD...
Zavrelimyia melanura|SOE83|Elisabeth Stur||BOLD:AAD8015
Zavrelimyia melanura|ATNA327|Elisabeth Stur||BOLD:AAD8015
Zavrelimyia melanura|SOE269|Elisabeth Stur||BOLD:AAD8015
Zavrelimyia|ZMUO.025935|Lauri Paasivirta||BOLD:AAD8015
Zavrelimyia|ZMUO.025934|Lauri Paasivirta||BOLD:AAD8015
Zavrelimyia melanura|SOE93|Elisabeth Stur||BOLD:AAD8015
Zavrelimyia melanura|SOE245|Elisabeth Stur||BOLD:AAD8015
Zavrelimyia melanura|ATNA74|Elisabeth Stur||BOLD:AAD8015
Pentaneurella katterjokki|ES147|Elisabeth Stur
Pentaneurella katterjokki|ZMUO.025857|Lauri Paasivirta||BOLD:AAC3939
Pentaneurella katterjokki|ATNA72|Elisabeth Stur||BOLD:AAC3939
Pentaneurella katterjokki|ATNA335|Elisabeth Stur||BOLD:AAC3939
Pentaneurella katterjokki|ES46|Elisabeth Stur||BOLD:AAC3939
Pentaneurella katterjokki|ES82|Elisabeth Stur||BOLD:AAC3939
Pentaneurella katterjokki|EBAI-Ch066|Elisabeth Stur|morphology|BOLD:AAC3939
Pentaneurella katterjokki|ZMUO.025856|Lauri Paasivirta||BOLD:AAC3939
Pentaneurella katterjokki|ATNA122|Elisabeth Stur||BOLD:AAC3939
Pentaneurella katterjokki|EBAI-Ch122|Elisabeth Stur|morphology|BOLD:AAC3939
Pentaneurella katterjokki|ATNA338|Elisabeth Stur||BOLD:AAC3939
Pentaneurella katterjokki|ATNA333|Elisabeth Stur||BOLD:AAC3939
Pentaneurella katterjokki|ATNA331|Elisabeth Stur||BOLD:AAC3939
Pentaneurella katterjokki|ATNA329|Elisabeth Stur||BOLD:AAC3939
Pentaneurella katterjokki|ATNA328|Elisabeth Stur||BOLD:AAC3939
Pentaneurella katterjokki|ATNA334|Elisabeth Stur||BOLD:AAC3939
Pentaneurella katterjokki|ATNA71|Elisabeth Stur||BOLD:AAC3939
Zavrelimyia cf. divisa|TRD-CH370|Elisabeth Stur|morphology|BOLD:ACT8467
Zavrelimyia cf. divisa|TRD-CH184|Elisabeth Stur|morphology|BOLD:ACT8467

Pentaneurella katterjokki|ATNA71|Elisabeth Stur||BOLD:AAC3939
Zavrelimyia cf. divisa|TRD-CH370|Elisabeth Stur|morphology|BOLD:ACT8467
Zavrelimyia cf. divisa|TRD-CH184|Elisabeth Stur|morphology|BOLD:ACT8467
Zavrelimyia divisa|CH-OSF100|Elisabeth Stur||BOLD:AAP7146
Zavrelimyia divisa|Finnmark891|Elisabeth Stur||BOLD:AAP7146
Zavrelimyia divisa|CH-eik117|Elisabeth Stur||BOLD:AAP7146
Zavrelimyia divisa|Finnmark161|Elisabeth Stur||BOLD:AAP7146
Monopelopia tenuicalcar|ZMUO.025318|Lauri Paasivirta||BOLD:ACO6911
Monopelopia tenuicalcar|ZMUO.025317|Lauri Paasivirta||BOLD:ACO6911
Monopelopia tenuicalcar|NHRS-BYWS000000331|Yngve Brodin|morphology|BOLD:ACO6911
Monopelopia tenuicalcar|ZSM-DIP-33128-H09|Caroline Chimeno|BIN Taxonomy Match (Jan 2022)|BO...
Monopelopia tenuicalcar|ZSM-DIP-33148-D04|Caroline Chimeno|BIN Taxonomy Match (Jan 2022)|BO...
Monopelopia tenuicalcar|ZSM-DIP-33135-D11|Caroline Chimeno|BIN Taxonomy Match (Jan 2022)|BO...
Monopelopia tenuicalcar|ZSM-DIP-33145-E09|Caroline Chimeno|BIN Taxonomy Match (Jan 2022)|BO...
Monopelopia tenuicalcar|ZSM-DIP-33149-B04|Caroline Chimeno|BIN Taxonomy Match (Jan 2022)|BO...
Monopelopia tenuicalcar|ZSM-DIP-33145-E10|Caroline Chimeno|BIN Taxonomy Match (Jan 2022)|BO...
Monopelopia tenuicalcar|ZSM-DIP-33145-E11|Caroline Chimeno|BIN Taxonomy Match (Jan 2022)|BO...
Monopelopia tenuicalcar|ZSM-DIP-33149-A02|Caroline Chimeno|BIN Taxonomy Match (Jan 2022)|BO...
Monopelopia tenuicalcar|ZSM-DIP-33135-D12|Caroline Chimeno|BIN Taxonomy Match (Jan 2022)|BO...
Monopelopia tenuicalcar|ZSM-DIP-33135-D10|Caroline Chimeno|BIN Taxonomy Match (Jan 2022)|BO...
Monopelopia tenuicalcar|ZSM-DIP-33148-D05|Caroline Chimeno|BIN Taxonomy Match (Jan 2022)|BO...
Monopelopia tenuicalcar|ZSM-DIP-33148-D06|Caroline Chimeno|BIN Taxonomy Match (Jan 2022)|BO...
Monopelopia tenuicalcar|ZSM-DIP-33149-A04|Caroline Chimeno|BIN Taxonomy Match (Jan 2022)|BO...
Monopelopia tenuicalcar|ZSM-DIP-33149-A05|Caroline Chimeno|BIN Taxonomy Match (Jan 2022)|BO...
Monopelopia tenuicalcar|ZSM-DIP-33149-A03|Caroline Chimeno|BIN Taxonomy Match (Jan 2022)|BO...
Monopelopia tenuicalcar|ZSM-DIP-33149-A01|Caroline Chimeno|BIN Taxonomy Match (Jan 2022)|BO...
Monopelopia tenuicalcar|ZSM-DIP-33145-E08|Caroline Chimeno|BIN Taxonomy Match (Jan 2022)|BO...
Monopelopia tenuicalcar|ZSM-DIP-33149-A06|Caroline Chimeno|BIN Taxonomy Match (Jan 2022)|BO...
Monopelopia tenuicalcar|ZSM-DIP-33135-D09|Caroline Chimeno|BIN Taxonomy Match (Jan 2022)|BO...
Monopelopia tenuicalcar|CCDB24183-A10|Sofia Wiedenbrug|BIN-taxonomy|BOLD:AAM6277
Monopelopia tenuicalcar|NO 95|Elisabeth Stur||BOLD:AAM6277
Rheopelopia maculipennis|ZMUO.025288|Lauri Paasivirta||BOLD:ADA3165
Rheopelopia maculipennis|ZMUO.025287|Lauri Paasivirta||BOLD:ADA3165
Rheopelopia maculipennis|TRD-CH179|Elisabeth Stur||BOLD:AAW5040
Rheopelopia maculipennis|BIOUG16432-H11|Kate Perez|BIN Taxonomy Match|BOLD:AAW5040
Rheopelopia maculipennis|BIOUG16436-G07|Kate Perez|BIN Taxonomy Match|BOLD:AAW5040
Rheopelopia maculipennis|BIOUG16435-F05|Kate Perez|BIN Taxonomy Match|BOLD:AAW5040
Rheopelopia maculipennis|BIOUG16477-E05|Kate Perez|BIN Taxonomy Match|BOLD:AAW5040
Rheopelopia maculipennis|BIOUG16476-C11|Kate Perez|BIN Taxonomy Match|BOLD:AAW5040
Rheopelopia maculipennis|BIOUG16434-A01|Kate Perez|BIN Taxonomy Match|BOLD:AAW5040
Rheopelopia maculipennis|BIOUG16597-G12|Kate Perez|BIN Taxonomy Match|BOLD:AAW5040
Rheopelopia maculipennis|ZSM-DIP-33142-G05|Caroline Chimeno|BIN Taxonomy Match (Jan 2022)|B...
Rheopelopia maculipennis|ZMUO.026392|Marko Mutanen||BOLD:AAW5040
Rheopelopia maculipennis|ZMUO.026393|Marko Mutanen||BOLD:AAW5040
Rheopelopia maculipennis|EJV-20110171|Lauri Paasivirta||BOLD:AAW5040
Rheopelopia maculipennis|EJV-20110170|Lauri Paasivirta||BOLD:AAW5040
Conchapelopia pallens|BIOUG55416-D04|Kate Perez|BIN Taxonomy Match (Oct 2020)|BOLD:AAC4803
Conchapelopia|Finnmark697|Elisabeth Stur||BOLD:AAV2694
Chironomidae|CCDB24228-G09|Jerome Moriniere|BIN Taxonomy Match|BOLD:ACU4667
Chironomidae|CCDB24228-H07|Jerome Moriniere|BIN Taxonomy Match|BOLD:ACU4667
Chironomidae|CCDB24228-F08|Jerome Moriniere|BIN Taxonomy Match|BOLD:ACU4667
Conchapelopia pallidula|ZSM-DIP-33144-F01|Caroline Chimeno|BIN Taxonomy Match (Jan 2022)|BO...
Conchapelopia|BIOUG15951-D07|Kate Perez|BOLD ID Engine Manual|BOLD:ACQ3496
Conchapelopia pallidula|PK-194-5|Elisabeth Stur|morphology|BOLD:ACQ3496
Conchapelopia|BIOUG16198-H10|Kate Perez|BOLD ID Engine Manual|BOLD:ACQ3496
Conchapelopia|BIOUG16296-H08|Kate Perez|BIN Taxonomy Match|BOLD:AAW5799
Conchapelopia hittmairorum|ZMUO.024781|Lauri Paasivirta||BOLD:AAW5799
Conchapelopia|BIOUG16332-G01|Kate Perez|BIN Taxonomy Match|BOLD:AAW5799
Conchapelopia|BIOUG17264-H07|Kate Perez|BIN Taxonomy Match|BOLD:AAW5799
Conchapelopia hittmairorum|ZMUO.024780|Lauri Paasivirta||BOLD:AAW5799
Conchapelopia hittmairorum|EJV-20110172|Lauri Paasivirta||BOLD:AAW5799
Conchapelopia melanops|ZMUO.024774|Lauri Paasivirta||BOLD:AAP5886
Conchapelopia melanops|BIOUG36681-H01|Kate Perez|BIN Taxonomy Match (Jul 2018)|BOLD:AAP5886
Conchapelopia melanops|BIOUG16205-D01|Kate Perez|BIN Taxonomy Match|BOLD:AAP5886
Conchapelopia melanops|ZSM-DIP-33139-F04|Caroline Chimeno|BIN Taxonomy Match (Jan 2022)|BOL...
Conchapelopia melanops|ZSM-DIP-33145-H08|Caroline Chimeno|BIN Taxonomy Match (Jan 2022)|BOL...
Conchapelopia melanops|Finnmark215|Elisabeth Stur||BOLD:AAP5886
Conchapelopia melanops|Finnmark216|Elisabeth Stur||BOLD:AAP5886
Conchapelopia melanops|Finnmark719|Elisabeth Stur||BOLD:AAP5886
Conchapelopia melanops|TRD-CH367|Elisabeth Stur|morphology|BOLD:AAP5886
Conchapelopia melanops|TRD-CH171|Elisabeth Stur||BOLD:AAP5886
Conchapelopia melanops|BIOUG43950-C03|Kate Perez|BIN Taxonomy Match below Phylum (Jan 2019)...
Conchapelopia melanops|ZMUO.024773|Lauri Paasivirta||BOLD:AAP5886
Conchapelopia melanops|ZSM-DIP-33170-B07|Caroline Chimeno|BIN Taxonomy Match (Jan 2022)|BOL...
Conchapelopia melanops|BIOUG15966-H09|Kate Perez|BIN Taxonomy Match|BOLD:AAP5886
Conchapelopia melanops|BIOUG36776-B08|Kate Perez|BIN Taxonomy Match (Jul 2018)|BOLD:AAP5886
Conchapelopia melanops|BIOUG36933-A07|Kate Perez|BIN Taxonomy Match (Jul 2018)|BOLD:AAP5886
Conchapelopia melanops|ZSM-DIP-33139-F05|Caroline Chimeno|BIN Taxonomy Match (Jan 2022)|BOL...
Conchapelopia melanops|EJV-20110173|Lauri Paasivirta||BOLD:AAP5886
Conchapelopia melanops|Finnmark264|Elisabeth Stur||BOLD:AAP5886
Thienemannimyia tripunctata|ZMUO.026152|Lauri Paasivirta||BOLD:ADE9889
Thienemannimyia tripunctata|ZMUO.026151|Lauri Paasivirta||BOLD:ADE9889
Thienemannimyia tripunctata|ZSM-DIP-33147-D07|Caroline Chimeno|BIN Taxonomy Match (Jan 2022 ...
Thienemannimyia tripunctata|ZSM-DIP-33143-A02|Caroline Chimeno|BIN Taxonomy Match (Jan 2022 ...
Arctopelopia barbitarsis|TRD-CH74|Elisabeth Stur||BOLD:AAM5765
Arctopelopia barbitarsis|TRD-CH77|Elisabeth Stur||BOLD:AAM5765
Arctopelopia barbitarsis|TRD-CH73|Elisabeth Stur||BOLD:AAM5765
Arctopelopia barbitarsis|ZMUO.024608|Lauri Paasivirta||BOLD:AAM5765
Arctopelopia barbitarsis|Finnmark34|Elisabeth Stur||BOLD:AAM5765
Arctopelopia barbitarsis|Finnmark722|Elisabeth Stur||BOLD:AAM5765
Arctopelopia barbitarsis|Finnmark660|Elisabeth Stur||BOLD:AAM5765
Arctopelopia barbitarsis|BIOUG43948-E08|Kate Perez|BIN Taxonomy Match below Phylum (Jan 2019 ...
Arctopelopia barbitarsis|ATNA567|Elisabeth Stur||BOLD:AAM5765
Arctopelopia barbitarsis|TRD-CH98|Elisabeth Stur||BOLD:AAM5765
Arctopelopia barbitarsis|ZMUO.024609|Lauri Paasivirta||BOLD:AAM5765
Arctopelopia barbitarsis|Finnmark122|Elisabeth Stur||BOLD:AAM5765
Arctopelopia barbitarsis|Finnmark388|Elisabeth Stur||BOLD:AAM5765
Arctopelopia barbitarsis|Finnmark353|Elisabeth Stur||BOLD:AAM5765
Thienemannimyia northumbrica|BIOUG42703-G07|Kate Perez|BIN Taxonomy Match below Phylum (Jun 20...
Thienemannimyia northumbrica|BIOUG16614-F01|Kate Perez|BIN Taxonomy Match|BOLD:AAV2698
Thienemannimyia northumbrica|ZSM-DIP-33137-A02|Caroline Chimeno|BIN Taxonomy Match (Jan 2022 ...
Thienemannimyia northumbrica|CH-OSF114|Elisabeth Stur||BOLD:AAV2698
Thienemannimyia northumbrica|CH-OSF40|Elisabeth Stur||BOLD:AAV2698

Thienemannimyia northumbrica|ZSM-DIP-33137-A02|Caroline Chimeno|BIN Taxonomy Match (Jan 2022 ...
Thienemannimyia northumbrica|CH-OSF114|Elisabeth Stur||BOLD:AAV2698
Thienemannimyia northumbrica|CH-OSF40|Elisabeth Stur||BOLD:AAV2698
Thienemannimyia fusciceps|ZMUO.026184|Lauri Paasivirta||BOLD:AAP7147
Thienemannimyia fusciceps|ZMUO.026183|Lauri Paasivirta||BOLD:AAP7147
Thienemannimyia fusciceps|Finnmark174|Elisabeth Stur||BOLD:AAP7147
Thienemannimyia fusciceps|Finnmark509|Elisabeth Stur||BOLD:AAP7147
Thienemannimyia fusciceps|Finnmark260|Elisabeth Stur||BOLD:AAP7147
Thienemannimyia carnea|ZMUO.025166|Lauri Paasivirta||BOLD:ABZ4646
Thienemannimyia carnea|BIOUG16257-F04|Kate Perez|BIN Taxonomy Match|BOLD:ABZ4646
Thienemannimyia carnea|ZMUO.025167|Lauri Paasivirta||BOLD:ABZ4646
Thienemannimyia carnea|ZSM-DIP-33170-B08|Caroline Chimeno|BIN Taxonomy Match (Jan 2022)|BOL...
Thienemannimyia carnea|TRD-CH176|Elisabeth Stur||BOLD:ABZ4646
Thienemannimyia carnea|BIOUG15966-D04|Kate Perez|BIN Taxonomy Match|BOLD:ABZ4646
Thienemannimyia carnea|BIOUG15954-A10|Kate Perez|BIN Taxonomy Match
Thienemannimyia carnea|BIOUG16330-D06|Kate Perez|BIN Taxonomy Match|BOLD:ABZ4646
Thienemannimyia carnea|TRD-CH326|Elisabeth Stur||BOLD:ABZ4646
Thienemannimyia carnea|BIOUG16552-B11|Kate Perez|BIN Taxonomy Match
Thienemannimyia carnea|TRD-CH417|Elisabeth Stur|morphology|BOLD:ABZ4646
Thienemannimyia carnea|BIOUG15953-A12|Kate Perez|BIN Taxonomy Match
Thienemannimyia carnea|BIOUG16431-F02|Kate Perez|BIN Taxonomy Match
Thienemannimyia carnea|BIOUG16477-A06|Kate Perez|BIN Taxonomy Match|BOLD:ABZ4646
Thienemannimyia carnea|BIOUG16436-D07|Kate Perez|BIN Taxonomy Match|BOLD:ABZ4646
Thienemannimyia carnea|BIOUG15963-A12|Kate Perez|BIN Taxonomy Match|BOLD:ABZ4646
Thienemannimyia carnea|BIOUG15949-E04|Kate Perez|BIN Taxonomy Match|BOLD:ABZ4646
Thienemannimyia carnea|BIOUG15947-D05|Kate Perez|BIN Taxonomy Match|BOLD:ABZ4646
Thienemannimyia carnea|BIOUG15947-A01|Kate Perez|BIN Taxonomy Match|BOLD:ABZ4646
Thienemannimyia carnea|ZMUO.026164|Lauri Paasivirta||BOLD:ABZ4646
Thienemannimyia carnea|ZMUO.026163|Lauri Paasivirta||BOLD:ABZ4646
Thienemannimyia carnea|EJV-20110167|Lauri Paasivirta||BOLD:ABZ4646
Thienemannimyia carnea|EJV-20110166|Lauri Paasivirta||BOLD:ABZ4646
Thienemannimyia sp. 1ES|EBAI-Ch165|Elisabeth Stur|morphology|BOLD:AAG2944
Thienemannimyia sp. 1ES|ATNA540|Elisabeth Stur||BOLD:AAG2944
Thienemannimyia sp. 1ES|ATNA556|Elisabeth Stur||BOLD:AAG2944
Thienemannimyia sp. 1ES|BIOUG16396-F11|Kate Perez|BIN Taxonomy Match|BOLD:AAG2944
Thienemannimyia sp. 1ES|EBAI-Ch164|Elisabeth Stur|morphology
Thienemannimyia sp. 1ES|ATNA541|Elisabeth Stur||BOLD:AAG2944
Thienemannimyia sp. 1ES|ATNA182|Elisabeth Stur||BOLD:AAG2944
Thienemannimyia sp. 1ES|EBAI-Ch163|Elisabeth Stur|morphology
Thienemannimyia sp. 1ES|BIOUG15949-A06|Kate Perez|BIN Taxonomy Match|BOLD:AAG2944
Thienemannimyia sp. 1ES|Finnmark579|Elisabeth Stur||BOLD:AAG2944
Chironomidae|CCDB24228-G08||BIN Taxonomy Match|BOLD:ACY4074
Chironomidae|ZSM-DIP-33143-E08|Valerie Levesque-Beaudin|BOLD ID Engine Manual (Sep 2021)|BO...
Chironomidae|ZSM-DIP-33143-H10|Valerie Levesque-Beaudin|BIN Taxonomy Match (Sep 2021)|BOLD...
Chironomidae|ZSM-DIP-33146-H09|Valerie Levesque-Beaudin|BIN Taxonomy Match (Sep 2021)|BOLD...
Chironomidae|ZSM-DIP-33147-A09|Valerie Levesque-Beaudin|BIN Taxonomy Match (Sep 2021)|BOLD...
Chironomidae|ZSM-DIP-33149-F06|Valerie Levesque-Beaudin|BIN Taxonomy Match (Sep 2021)|BOLD...
Chironomidae|ZSM-DIP-33147-B05|Valerie Levesque-Beaudin|BIN Taxonomy Match (Sep 2021)|BOLD...
Chironomidae|ZSM-DIP-33147-A10|Valerie Levesque-Beaudin|BIN Taxonomy Match (Sep 2021)|BOLD...
Chironomidae|ZSM-DIP-33143-E07|Valerie Levesque-Beaudin|BIN Taxonomy Match (Sep 2021)|BOLD...
Chironomidae|ZSM-DIP-33143-E06|Valerie Levesque-Beaudin|BIN Taxonomy Match (Sep 2021)|BOLD...
Thienemannimyia lentiginosa|NHRS-BYWS000000937|Yngve Brodin|morphology|BOLD:AAV2695
Thienemannimyia lentiginosa|NHRS-BYWS000000936|Yngve Brodin|morphology|BOLD:AAV2695
Thienemannimyia lentiginosa|ZMUO.024976|Lauri Paasivirta||BOLD:AAV2695
Thienemannimyia lentiginosa|ZMUO.024975|Lauri Paasivirta||BOLD:AAV2695
Thienemannimyia lentiginosa|NHRS-BYWS000000401|Yngve Brodin|morphology|BOLD:AAV2695
Thienemannimyia lentiginosa|Finnmark707|Elisabeth Stur||BOLD:AAV2695
Tanypus sp. ZS18|MW308702|||BOLD:ADC6439
Orthocladiinae|BIOUG16558-A09|Elisabeth Stur|morphology|BOLD:ACR3118
Parakiefferiella sp. 3SW|BIOUG05144-G01|Sofia Wiedenbrug|BIN Taxonomy Match|BOLD:ACE0462
Parakiefferiella sp. 3SW|BIOUG05141-D02|Sofia Wiedenbrug|BIN Taxonomy Match|BOLD:ACE0462
Parakiefferiella sp. 3SW|BIOUG05144-G06|Sofia Wiedenbrug|BIN Taxonomy Match|BOLD:ACE0462
Parakiefferiella sp. 3SW|BIOUG05141-C12|Sofia Wiedenbrug|BIN Taxonomy Match|BOLD:ACE0462
Parakiefferiella sp. 3SW|BIOUG04795-G09|Sofia Wiedenbrug|BIN Taxonomy Match|BOLD:ACE0462
Parakiefferiella sp. 3SW|BIOUG04795-E05|Sofia Wiedenbrug|BIN Taxonomy Match|BOLD:ACE0462
Epoicocladius ephemerae|ZMUO.025140|Lauri Paasivirta||BOLD:ACR1208
Chironomidae|BIOUG43946-C08|Kate Perez|BIN Taxonomy Match below Phylum (Jan 2019)|BOLD:A...
Potthastia gaedii|Finnmark885|Elisabeth Stur||BOLD:ADJ6393
Smittia|24465-A08|Kate Perez|BIN Taxonomy Match|BOLD:ABA7010
Chironomidae|24372-A03|Mikko J Tiusanen|Tree based identification|BOLD:ACU6172
Orthocladiinae|BIOUG15545-C09|Kate Perez|BOLD ID Engine Manual|BOLD:ACP5848
Orthocladiinae|BIOUG15454-C06|Kate Perez|BOLD ID Engine Manual|BOLD:ACP5848
Orthocladiinae|BIOUG15537-D10|Kate Perez|BOLD ID Engine Manual|BOLD:ACP5848
Chironomidae|BIOUG17114-C07|Jerome Moriniere|BIN Taxonomy Match|BOLD:ACR3552
Chironomidae|BIOUG16994-C03|Jerome Moriniere|BIN Taxonomy Match|BOLD:ACR3552
Chironomidae|BIOUG16900-G11|Jerome Moriniere|BIN Taxonomy Match|BOLD:ACR3552
Cricotopus albiforceps|ZMUO.024712|Lauri Paasivirta||BOLD:AAW5785
Cricotopus albiforceps|ZMUO.024713|Lauri Paasivirta||BOLD:AAW5785
Cricotopus albiforceps|ZSM-DIP-33146-F01|Caroline Chimeno|BIN Taxonomy Match (Jan 2022)|BOL...
Cricotopus albiforceps|ZSM-DIP-33132-E09|Caroline Chimeno|BIN Taxonomy Match (Jan 2022)|BOL...
Cricotopus albiforceps|EJV-20110222|Lauri Paasivirta||BOLD:AAW5785
Parakiefferiella finnmarkica|ZMUO.025281|Lauri Paasivirta||BOLD:ACQ7422
Parakiefferiella cf. finnmarkica|TRD-CH386|Elisabeth Stur|morphology|BOLD:ACQ7422
Parakiefferiella cf. finnmarkica|BIOUG16208-C06|Elisabeth Stur|BOLD ID Engine Manual|BOLD:ACQ7422
Parakiefferiella finnmarkica|ZMUO.025282|Lauri Paasivirta||BOLD:ACS0073
Parakiefferiella smolandica|TRD-CH106|Elisabeth Stur||BOLD:AAM9698
Parakiefferiella smolandica|ZMUO.024669|Lauri Paasivirta||BOLD:AAM9698
Parakiefferiella smolandica|ZMUO.024628|Lauri Paasivirta||BOLD:AAM9698
Parakiefferiella smolandica|ZMUO.024668|Lauri Paasivirta||BOLD:AAM9698
Parakiefferiella smolandica|ZMUO.024627|Lauri Paasivirta||BOLD:AAM9698
Parakiefferiella smolandica|Finnmark798|Elisabeth Stur||BOLD:AAM9698
Parakiefferiella smolandica|Finnmark41|Elisabeth Stur||BOLD:AAM9698
Parakiefferiella cf. fennica|Finnmark421|Elisabeth Stur||BOLD:AAV6224
Parakiefferiella cf. fennica|Finnmark265|Elisabeth Stur||BOLD:AAV6224
Nanocladius rectinervis|CH-OSF41|Elisabeth Stur||BOLD:AAW0928
Nanocladius rectinervis|Finnmark811|Elisabeth Stur||BOLD:AAW0928
Nanocladius rectinervis|Finnmark812|Elisabeth Stur||BOLD:AAW0928
Nanocladius rectinervis|EJV-20110190|Lauri Paasivirta||BOLD:AAW0928
Nanocladius rectinervis|EJV-20110189|Lauri Paasivirta||BOLD:AAW0928
Nanocladius rectinervis|EJV-20110188|Lauri Paasivirta||BOLD:AAW0928
Smittia leucopogon|ZMUO.024756|Lauri Paasivirta||BOLD:ABW7981
Smittia|ZSM-DIP-33128-G06|Caroline Chimeno|BIN Taxonomy Match (Jan 2022)|BOLD:ACU9413
Thienemannia gracei|CCDB24037-B02|Sofia Wiedenbrug|BIN Taxonomy Match|BOLD:AAW0581

Smittia leucopogon|ZMUO.024756|Lauri Paasivirta||BOLD:ABW7981
Smittia|ZSM-DIP-33128-G06|Caroline Chimeno|BIN Taxonomy Match (Jan 2022)|BOLD:ACU9413
Thienemannia gracei|CCDB24037-B02|Sofia Wiedenbrug|BIN Taxonomy Match|BOLD:AAW0581
Thienemannia gracei|BIOUG16777-G01|Kate Perez|BIN Taxonomy Match|BOLD:AAW0581
Thienemannia gracei|ZSM-DIP-33133-D02|Caroline Chimeno|BIN Taxonomy Match (Jan 2022)|BOLD ...
Thienemannia gracei|ZSM-DIP-33142-F01|Caroline Chimeno|BIN Taxonomy Match (Jan 2022)|BOLD ...
Thienemannia gracei|BC-ZSM-DIP-22488-G09|Dieter Doczkal||BOLD:AAW0581
Thienemannia gracei|BC-ZSM-DIP-22488-F02|Dieter Doczkal||BOLD:AAW0581
Thienemannia gracei|BIOUG15456-A06|Kate Perez|BIN Taxonomy Match|BOLD:AAW0581
Thienemannia gracei|CH-OSF66|Elisabeth Stur||BOLD:AAW0581
Thienemannia fulvofasciata|ZSM34341-F08|Sofia Wiedenbrug|Morphology|BOLD:AAI0863
Thienemannia fulvofasciata|ZSM34342-D07|Sofia Wiedenbrug|Morphology|BOLD:AAI0863
Thienemannia fulvofasciata|CCDB24038-F02|Sofia Wiedenbrug|BIN Taxonomy Match|BOLD:AAI0863
Thienemannia fulvofasciata|ES109|Elisabeth Stur||BOLD:AAI0863
Thienemannia fulvofasciata|ES65|Elisabeth Stur||BOLD:AAI0863
Smittia|CCDB24039-H01|Sofia Wiedenbrug|BIN Taxonomy Match|BOLD:ACR0543
Smittia cf. stercoraria|TRD-CH418|Elisabeth Stur|morphology|BOLD:ACR0543
Smittia cf. stercoraria|BIOUG16712-H07|Elisabeth Stur|morphology|BOLD:ACR0543
Chironomidae|BIOUG16776-D12|Kate Perez|no morphological identification - introduction of interim ...
Smittia|ZSM-DIP-33133-F02|Caroline Chimeno|BIN Taxonomy Match (Jan 2022)|BOLD:ACR0048
Chironomidae|CCDB22605-D01|Sofia Wiedenbrug|no morphological identification - introduction of int...
Chironomidae|BIOUG16785-F03|Kate Perez|no morphological identification - introduction of interim ...
Chironomidae|BIOUG16778-F11|Kate Perez|no morphological identification - introduction of interim ...
Smittia|ZSM-DIP-33131-A05|Caroline Chimeno|BIN Taxonomy Match (Jan 2022)|BOLD:ACR0048
Chironomidae|BIOUG16740-F12|Kate Perez|no morphological identification - introduction of interim ...
Chironomidae|BIOUG16779-E01|Kate Perez|no morphological identification - introduction of interim ...
Chironomidae|BIOUG16739-F11|Kate Perez|no morphological identification - introduction of interim ...
Chironomidae|BIOUG16779-H02|Kate Perez|no morphological identification - introduction of interim ...
Chironomidae|BIOUG16854-A12|Kate Perez|no morphological identification - introduction of interim ...
Chironomidae|BIOUG16785-B08|Kate Perez|no morphological identification - introduction of interim ...
Chironomidae|BIOUG16739-B11|Kate Perez|no morphological identification - introduction of interim ...
Chironomidae|BIOUG16779-H08|Kate Perez|no morphological identification - introduction of interim ...
Chironomidae|BIOUG16779-C07|Kate Perez|no morphological identification - introduction of interim ...
Chironomidae|BIOUG16782-E11|Kate Perez|no morphological identification - introduction of interim ...
Smittia|ZSM-DIP-33146-G01|Caroline Chimeno|BIN Taxonomy Match (Jan 2022)|BOLD:ACR0048
Chironomidae|BIOUG16785-A09|Kate Perez|no morphological identification - introduction of interim ...
Chironomidae|BIOUG16738-G03|Kate Perez|no morphological identification - introduction of interim ...
Chironomidae|BIOUG16776-C04|Kate Perez|no morphological identification - introduction of interim ...
Chironomidae|BIOUG16778-D11|Kate Perez|no morphological identification - introduction of interim ...
Chironomidae|BIOUG16738-E02|Kate Perez|no morphological identification - introduction of interim ...
Chironomidae|BIOUG16784-A12|Kate Perez|no morphological identification - introduction of interim ...
Chironomidae|BIOUG16776-D11|Kate Perez|no morphological identification - introduction of interim ...
Chironomidae|BIOUG16892-C05|Kate Perez|no morphological identification - introduction of interim ...
Chironomidae|BIOUG16783-B11|Kate Perez|no morphological identification - introduction of interim ...
Chironomidae|CCDB22605-D03|Sofia Wiedenbrug|no morphological identification - introduction of int...
Chironomidae|BIOUG16778-C04|Kate Perez|no morphological identification - introduction of interim ...
Chironomidae|BIOUG16779-G10|Kate Perez|no morphological identification - introduction of interim ...
Chironomidae|BIOUG16783-F11|Kate Perez|no morphological identification - introduction of interim ...
Chironomidae|BIOUG16736-C06|Kate Perez|no morphological identification - introduction of interim ...
Chironomidae|BIOUG16854-F09|Kate Perez|no morphological identification - introduction of interim ...
Chironomidae|BIOUG16785-F02|Kate Perez|no morphological identification - introduction of interim ...
Chironomidae|BIOUG16782-H05|Kate Perez|no morphological identification - introduction of interim ...
Chironomidae|BIOUG16739-H06|Kate Perez|no morphological identification - introduction of interim ...
Chironomidae|BIOUG16739-G03|Kate Perez|no morphological identification - introduction of interim ...
Smittia|ZSM-DIP-33131-C06|Caroline Chimeno|BIN Taxonomy Match (Jan 2022)|BOLD:ACR0048
Smittia|BIOUG16857-D01|Kate Perez|BIN Taxonomy Match|BOLD:ACE0351
Smittia|BIOUG16893-B07|Kate Perez|BIN Taxonomy Match|BOLD:ACE0351
Smittia|BIOUG16857-H07|Kate Perez|BIN Taxonomy Match|BOLD:ACE0351
Smittia|BIOUG16893-F12|Kate Perez|BIN Taxonomy Match|BOLD:ACE0351
Smittia|BIOUG16784-G07|Kate Perez|BIN Taxonomy Match|BOLD:ACE0351
Smittia cf. stercoraria|ZSM-DIP-33128-C10|Caroline Chimeno|BIN Taxonomy Match (Jan 2022)|BO...
Smittia cf. stercoraria|ZSM-DIP-33128-C05|Caroline Chimeno|BIN Taxonomy Match (Jan 2022)|BO...
Smittia cf. stercoraria|ZSM-DIP-33141-B11|Caroline Chimeno|BIN Taxonomy Match (Jan 2022)|BO...
Smittia|BIOUG43079-H08|Kate Perez|BIN Taxonomy Match below Phylum (Jul 2019)|BOLD:ACE0351
Smittia|BIOUG16856-E08|Kate Perez|BIN Taxonomy Match|BOLD:ACE0351
Smittia|BIOUG16856-H10|Kate Perez|BIN Taxonomy Match|BOLD:ACE0351
Smittia|BIOUG16738-E12|Kate Perez|BIN Taxonomy Match|BOLD:ACE0351
Smittia|BIOUG55424-C03|Stefan Schmidt|BIN Taxonomy Match (Sep 2020)|BOLD:ACE0351
Smittia|CCDB22604-F05|Sofia Wiedenbrug||BOLD:ACE0351
Smittia|BIOUG16721-A08|Kate Perez|BIN Taxonomy Match|BOLD:ACE0351
Smittia|BIOUG16721-A05|Kate Perez|BIN Taxonomy Match|BOLD:ACE0351
Smittia|BIOUG16776-C07|Kate Perez|BIN Taxonomy Match|BOLD:ACE0351
Smittia|BIOUG16738-D09|Kate Perez|BIN Taxonomy Match|BOLD:ACE0351
Smittia|BIOUG16778-E01|Kate Perez|BIN Taxonomy Match|BOLD:ACE0351
Smittia|BIOUG16893-B04|Kate Perez|BIN Taxonomy Match|BOLD:ACE0351
Smittia|BIOUG16892-H03|Kate Perez|BIN Taxonomy Match|BOLD:ACE0351
Smittia|BIOUG16892-H05|Kate Perez|BIN Taxonomy Match|BOLD:ACE0351
Smittia|BIOUG16856-E12|Kate Perez|BIN Taxonomy Match|BOLD:ACE0351
Smittia|BIOUG16856-D06|Kate Perez|BIN Taxonomy Match|BOLD:ACE0351
Smittia|BIOUG16892-B03|Kate Perez|BIN Taxonomy Match|BOLD:ACE0351
Smittia|BIOUG16721-B01|Kate Perez|BIN Taxonomy Match|BOLD:ACE0351
Smittia|BIOUG16784-D10|Kate Perez|BIN Taxonomy Match|BOLD:ACE0351
Smittia|BIOUG16783-B12|Kate Perez|BIN Taxonomy Match|BOLD:ACE0351
Smittia cf. stercoraria|BIOUG17117-H05|Kate Perez|BIN Taxonomy Match|BOLD:ACE0351
Smittia cf. stercoraria|BIOUG16855-E01|Kate Perez|BIN Taxonomy Match|BOLD:ACE0351
Smittia cf. stercoraria|ZSM-DIP-33136-C04|Caroline Chimeno|BIN Taxonomy Match (Jan 2022)|BO...
Smittia cf. stercoraria|BIOUG16854-G07|Kate Perez|BIN Taxonomy Match|BOLD:ACE0351
Smittia cf. stercoraria|BIOUG16892-B09|Kate Perez|BIN Taxonomy Match|BOLD:ACE0351
Smittia cf. stercoraria|BIOUG16854-C05|Kate Perez|BIN Taxonomy Match|BOLD:ACE0351
Smittia cf. stercoraria|BIOUG16784-G09|Kate Perez|BIN Taxonomy Match|BOLD:ACE0351
Smittia cf. stercoraria|BIOUG16892-C12|Kate Perez|BIN Taxonomy Match|BOLD:ACE0351
Smittia cf. stercoraria|BIOUG16859-H09|Kate Perez|BIN Taxonomy Match|BOLD:ACE0351
Smittia cf. stercoraria|BIOUG16785-H05|Kate Perez|BIN Taxonomy Match|BOLD:ACE0351
Smittia cf. stercoraria|BIOUG16784-E12|Kate Perez|BIN Taxonomy Match|BOLD:ACE0351
Smittia cf. stercoraria|CCDB22604-F08|Sofia Wiedenbrug||BOLD:ACE0351
Smittia|BIOUG55423-F05|Stefan Schmidt|BIN Taxonomy Match (Sep 2020)|BOLD:ACE0351
Smittia cf. stercoraria|ZSM-DIP-33128-G09|Caroline Chimeno|BIN Taxonomy Match (Jan 2022)|BO...
Smittia cf. stercoraria|ZSM-DIP-33128-G05|Caroline Chimeno|BIN Taxonomy Match (Jan 2022)|BO...
Smittia cf. stercoraria|ZSM-DIP-33136-F04|Caroline Chimeno|BIN Taxonomy Match (Jan 2022)|BO...
Smittia cf. stercoraria|ZSM-DIP-33132-E07|Caroline Chimeno|BIN Taxonomy Match (Jan 2022)|BO...
Smittia|BIOUG55424-H10|Stefan Schmidt|BIN Taxonomy Match (Sep 2020)|BOLD:ACE0351
Smittia cf. stercoraria|ZSM-DIP-33135-F04|Caroline Chimeno|BIN Taxonomy Match (Jan 2022)|BO...
Smittia cf. stercoraria|ZSM-DIP-33131-D09|Caroline Chimeno|BIN Taxonomy Match (Jan 2022)|BO...

Smittia cf. stercoraria|ZSM-DIP-33132-E07|Caroline Chimeno|BIN Taxonomy Match (Jan 2022)|BO...
Smittia|BIOUG55424-H10|Stefan Schmidt|BIN Taxonomy Match (Sep 2020)|BOLD:ACE0351
Smittia cf. stercoraria|ZSM-DIP-33135-F04|Caroline Chimeno|BIN Taxonomy Match (Jan 2022)|BO...
Smittia cf. stercoraria|ZSM-DIP-33131-D09|Caroline Chimeno|BIN Taxonomy Match (Jan 2022)|BO...
Smittia cf. stercoraria|ZSM-DIP-33139-B10|Caroline Chimeno|BIN Taxonomy Match (Jan 2022)|BO...
Smittia cf. stercoraria|ZSM-DIP-33141-B12|Caroline Chimeno|BIN Taxonomy Match (Jan 2022)|BO...
Smittia cf. stercoraria|ZSM-DIP-33130-C07|Caroline Chimeno|BIN Taxonomy Match (Jan 2022)|BO...
Smittia cf. stercoraria|ZSM-DIP-33135-F03|Caroline Chimeno|BIN Taxonomy Match (Jan 2022)|BO...
Smittia cf. stercoraria|ZSM-DIP-33135-F06|Caroline Chimeno|BIN Taxonomy Match (Jan 2022)|BO...
Smittia cf. stercoraria|ZSM-DIP-33141-A12|Caroline Chimeno|BIN Taxonomy Match (Jan 2022)|BO...
Smittia|BIOUG42959-B11|Kate Perez|BIN Taxonomy Match below Phylum (Jul 2019)|BOLD:ACE0351
Smittia|BIOUG55423-D05|Stefan Schmidt|BIN Taxonomy Match (Sep 2020)|BOLD:ACE0351
Smittia|BIOUG55425-A12|Stefan Schmidt|BIN Taxonomy Match (Sep 2020)|BOLD:ACE0351
Smittia|BIOUG55426-C08|Stefan Schmidt|BIN Taxonomy Match (Sep 2020)|BOLD:ACE0351
Smittia|BIOUG55428-D08|Stefan Schmidt|BIN Taxonomy Match (Sep 2020)|BOLD:ACE0351
Smittia|BIOUG55436-F11|Stefan Schmidt|BIN Taxonomy Match (Sep 2020)|BOLD:ACE0351
Smittia cf. stercoraria|ZSM-DIP-33132-H07|Caroline Chimeno|BIN Taxonomy Match (Jan 2022)|BO...
Smittia cf. stercoraria|ZSM-DIP-33133-D04|Caroline Chimeno|BIN Taxonomy Match (Jan 2022)|BO...
Smittia cf. stercoraria|ZSM-DIP-33133-D05|Caroline Chimeno|BIN Taxonomy Match (Jan 2022)|BO...
Smittia cf. stercoraria|ZSM-DIP-33133-D06|Caroline Chimeno|BIN Taxonomy Match (Jan 2022)|BO...
Smittia cf. stercoraria|ZSM-DIP-33137-C11|Caroline Chimeno|BIN Taxonomy Match (Jan 2022)|BO...
Smittia cf. stercoraria|ZSM-DIP-33144-C03|Caroline Chimeno|BIN Taxonomy Match (Jan 2022)|BO...
Smittia cf. stercoraria|ZSM-DIP-33141-B01|Caroline Chimeno|BIN Taxonomy Match (Jan 2022)|BO...
Smittia cf. stercoraria|ZSM-DIP-33141-B02|Caroline Chimeno|BIN Taxonomy Match (Jan 2022)|BO...
Smittia cf. stercoraria|ZSM-DIP-33143-D03|Caroline Chimeno|BIN Taxonomy Match (Jan 2022)|BO...
Smittia cf. stercoraria|ZSM-DIP-33135-F08|Caroline Chimeno|BIN Taxonomy Match (Jan 2022)|BO...
Smittia cf. stercoraria|ZSM-DIP-33136-C02|Caroline Chimeno|BIN Taxonomy Match (Jan 2022)|BO...
Smittia cf. stercoraria|ZSM-DIP-33136-C03|Caroline Chimeno|BIN Taxonomy Match (Jan 2022)|BO...
Smittia cf. stercoraria|ZSM-DIP-33136-F01|Caroline Chimeno|BIN Taxonomy Match (Jan 2022)|BO...
Smittia cf. stercoraria|ZSM-DIP-33136-H10|Caroline Chimeno|BIN Taxonomy Match (Jan 2022)|BO...
Smittia cf. stercoraria|ZSM-DIP-33139-B11|Caroline Chimeno|BIN Taxonomy Match (Jan 2022)|BO...
Smittia cf. stercoraria|ZSM-DIP-33140-D04|Caroline Chimeno|BIN Taxonomy Match (Jan 2022)|BO...
Smittia cf. stercoraria|ZSM-DIP-33141-A11|Caroline Chimeno|BIN Taxonomy Match (Jan 2022)|BO...
Smittia cf. stercoraria|ZSM-DIP-33172-H04|Caroline Chimeno|BIN Taxonomy Match (Jan 2022)|BO...
Smittia cf. stercoraria|ZSM-DIP-33128-C11|Caroline Chimeno|BIN Taxonomy Match (Jan 2022)|BO...
Smittia cf. stercoraria|ZSM-DIP-33146-C11|Caroline Chimeno|BIN Taxonomy Match (Jan 2022)|BO...
Smittia cf. stercoraria|ZSM-DIP-33146-D02|Caroline Chimeno|BIN Taxonomy Match (Jan 2022)|BO...
Smittia|BIOUG55428-B07|Stefan Schmidt|BIN Taxonomy Match (Sep 2020)|BOLD:ACE0351
Smittia cf. stercoraria|ZSM-DIP-33128-G04|Caroline Chimeno|BIN Taxonomy Match (Jan 2022)|BO...
Smittia|BIOUG55428-E05|Stefan Schmidt|BIN Taxonomy Match (Sep 2020)|BOLD:ACE0351
Smittia cf. stercoraria|CCDB22604-F10|Sofia Wiedenbrug||BOLD:ACE0351
Smittia cf. stercoraria|ZSM-DIP-33139-B09|Caroline Chimeno|BIN Taxonomy Match (Jan 2022)|BO...
Smittia cf. stercoraria|ZSM-DIP-33140-D07|Caroline Chimeno|BIN Taxonomy Match (Jan 2022)|BO...
Smittia cf. stercoraria|CCDB22604-F06|Sofia Wiedenbrug||BOLD:ACE0351
Smittia cf. stercoraria|ZSM-DIP-33141-A10|Caroline Chimeno|BIN Taxonomy Match (Jan 2022)|BO...
Smittia cf. stercoraria|ZSM-DIP-33136-C01|Caroline Chimeno|BIN Taxonomy Match (Jan 2022)|BO...
Smittia|BIOUG55423-F12|Stefan Schmidt|BIN Taxonomy Match (Sep 2020)|BOLD:ACE0351
Smittia cf. stercoraria|CCDB24183E03|Sofia Wiedenbrug|Morphology|BOLD:ACE0351
Smittia cf. stercoraria|BIOUG05105-B08|Kate Perez|BIN Taxonomy Match|BOLD:ACE0351
Smittia|BIOUG42967-B07|Kate Perez|BIN Taxonomy Match below Phylum (Jun 2019)|BOLD:AAG1020
Smittia sp. 23ES|BIOUG37052-F06|Kate Perez|BIN Taxonomy Match (Jan 2019)|BOLD:AAH9641
Smittia sp. 23ES|BIOUG37182-C04|Kate Perez|BIN Taxonomy Match (Jan 2019)|BOLD:AAH9641
Smittia aterrima|ZMUO.026167|Lauri Paasivirta||BOLD:AAH9641
Smittia sp. 23ES|BIOUG16777-E12|Kate Perez|BIN Taxonomy Match|BOLD:AAH9641
Smittia sp. 23ES|Finnmark73|Elisabeth Stur||BOLD:AAH9641
Smittia|BIOUG36625-H06|Kate Perez|BIN Taxonomy Match (Jul 2018)|BOLD:AAN5355
Smittia|BIOUG36625-G03|Kate Perez|BIN Taxonomy Match (Jul 2018)|BOLD:AAN5355
Smittia|BIOUG36625-F09|Kate Perez|BIN Taxonomy Match (Jul 2018)|BOLD:AAN5355
Smittia|BIOUG36625-G07|Kate Perez|BIN Taxonomy Match (Jul 2018)|BOLD:AAN5355
Smittia|BIOUG36625-E07|Kate Perez|BIN Taxonomy Match (Jul 2018)|BOLD:AAN5355
Smittia|BIOUG36624-G11|Kate Perez|BIN Taxonomy Match (Jul 2018)|BOLD:AAN5355
Smittia|BIOUG36624-G10|Kate Perez|BIN Taxonomy Match (Jul 2018)|BOLD:AAN5355
Smittia cf. stercoraria|BIOUG14592-G07|Elisabeth Stur|BIN Taxonomy Match|BOLD:AAN5355
Chironomidae|CCDB22604-F09|Sofia Wiedenbrug|no morphological identification - introduction of int...
Smittia cf. stercoraria|ZSM-DIP-33139-B06|Caroline Chimeno|BIN Taxonomy Match (Jan 2022)|BO...
Smittia stercoraria|ZSM-DIP-33146-D10|Caroline Chimeno|BIN Taxonomy Match (Jan 2022)|BOLD ...
Smittia cf. stercoraria|ZSM-DIP-33139-B05|Caroline Chimeno|BIN Taxonomy Match (Jan 2022)|BO...
Smittia cf. stercoraria|ZSM-DIP-33139-B04|Caroline Chimeno|BIN Taxonomy Match (Jan 2022)|BO...
Smittia stercoraria|ZSM-DIP-33135-H03|Caroline Chimeno|BIN Taxonomy Match (Jan 2022)|BOLD ...
Smittia cf. stercoraria|ZSM-DIP-33134-F05|Caroline Chimeno|BIN Taxonomy Match (Jan 2022)|BO...
Smittia|BIOUG42933-E06|Kate Perez|BIN Taxonomy Match below Phylum (Jun 2019)|BOLD:AAN5355
Smittia|BIOUG36625-B08|Kate Perez|BIN Taxonomy Match (Jul 2018)|BOLD:AAN5355
Smittia|BIOUG36624-G08|Kate Perez|BIN Taxonomy Match (Jul 2018)|BOLD:AAN5355
Smittia|ZSM-DIP-33133-E10|Caroline Chimeno|BIN Taxonomy Match (Jan 2022)|BOLD:AAN5355
Smittia|ZSM-DIP-33133-D09|Caroline Chimeno|BIN Taxonomy Match (Jan 2022)|BOLD:AAN5355
Smittia|ZSM-DIP-33140-C04|Caroline Chimeno|BIN Taxonomy Match (Jan 2022)|BOLD:AAN5355
Smittia|BIOUG36625-C11|Kate Perez|BIN Taxonomy Match (Jul 2018)|BOLD:AAN5355
Smittia|BIOUG36625-D09|Kate Perez|BIN Taxonomy Match (Jul 2018)|BOLD:AAN5355
Smittia|BIOUG36625-G08|Kate Perez|BIN Taxonomy Match (Jul 2018)|BOLD:AAN5355
Smittia|BIOUG36625-G12|Kate Perez|BIN Taxonomy Match (Jul 2018)|BOLD:AAN5355
Smittia|BIOUG36625-E04|Kate Perez|BIN Taxonomy Match (Jul 2018)|BOLD:AAN5355
Smittia cf. stercoraria|BIOUG15456-G03|Elisabeth Stur|BIN Taxonomy Match|BOLD:AAN5355
Smittia cf. stercoraria|BIOUG15460-C04|Elisabeth Stur|BIN Taxonomy Match|BOLD:AAN5355
Smittia stercoraria|ZMUO.025335|Lauri Paasivirta||BOLD:AAN5355
Smittia|BIOUG36625-F06|Kate Perez|BIN Taxonomy Match (Jul 2018)|BOLD:AAN5355
Chironomidae|BIOUG16892-A06|Kate Perez|no morphological identification - introduction of interim ...
Smittia|BIOUG36625-F02|Kate Perez|BIN Taxonomy Match (Jul 2018)|BOLD:AAN5355
Smittia|BIOUG36624-G05|Kate Perez|BIN Taxonomy Match (Jul 2018)|BOLD:AAN5355
Smittia cf. stercoraria|TRD-CH121|Elisabeth Stur||BOLD:AAN5355
Smittia|BIOUG36625-F05|Kate Perez|BIN Taxonomy Match (Jul 2018)|BOLD:AAN5358
Smittia cf. stercoraria|TRD-CH82|Elisabeth Stur||BOLD:AAN5358
Smittia|BIOUG36627-E06|Kate Perez|BIN Taxonomy Match (Jul 2018)|BOLD:AAN5358
Smittia|BIOUG36650-A06|Kate Perez|BIN Taxonomy Match (Jul 2018)|BOLD:AAN5358
Smittia|BIOUG36625-D05|Kate Perez|BIN Taxonomy Match (Jul 2018)|BOLD:AAN5358
Smittia|BIOUG36625-G04|Kate Perez|BIN Taxonomy Match (Jul 2018)|BOLD:AAN5358
Smittia|BIOUG36625-C08|Kate Perez|BIN Taxonomy Match (Jul 2018)|BOLD:AAN5358
Smittia|BIOUG36625-C10|Kate Perez|BIN Taxonomy Match (Jul 2018)|BOLD:AAN5358
Smittia|BIOUG36625-D11|Kate Perez|BIN Taxonomy Match (Jul 2018)|BOLD:AAN5358
Smittia|BIOUG36625-H04|Kate Perez|BIN Taxonomy Match (Jul 2018)|BOLD:AAN5358
Smittia|BIOUG36625-C01|Kate Perez|BIN Taxonomy Match (Jul 2018)|BOLD:AAN5358
Smittia|BIOUG36625-H05|Kate Perez|BIN Taxonomy Match (Jul 2018)|BOLD:AAN5358
Smittia|BIOUG36778-B01|Kate Perez|BIN Taxonomy Match (Jul 2018)|BOLD:AAN5358
Smittia|BIOUG36624-H02|Kate Perez|BIN Taxonomy Match (Jul 2018)|BOLD:AAN5358
[illegible]

Smittia|BIOUG36625-H05|Kate Perez|BIN Taxonomy Match (Jul 2018)|BOLD:AAN5358
Smittia|BIOUG36778-B01|Kate Perez|BIN Taxonomy Match (Jul 2018)|BOLD:AAN5358
Smittia|BIOUG36624-H02|Kate Perez|BIN Taxonomy Match (Jul 2018)|BOLD:AAN5358
Smittia|BIOUG36625-B11|Kate Perez|BIN Taxonomy Match (Jul 2018)|BOLD:AAN5358
Smittia|BIOUG36625-D10|Kate Perez|BIN Taxonomy Match (Jul 2018)|BOLD:AAN5358
Smittia|BIOUG36625-E09|Kate Perez|BIN Taxonomy Match (Jul 2018)|BOLD:AAN5358
Smittia|BIOUG36625-F10|Kate Perez|BIN Taxonomy Match (Jul 2018)|BOLD:AAN5358
Smittia stercoraria|ZMUO.025336|Lauri Paasivirta||BOLD:AAN5358
Smittia|BIOUG36625-F03|Kate Perez|BIN Taxonomy Match (Jul 2018)|BOLD:AAN5358
Smittia|BIOUG36625-B03|Kate Perez|BIN Taxonomy Match (Jul 2018)|BOLD:AAN5358
Smittia|BIOUG43080-C12|Kate Perez|BIN Taxonomy Match below Phylum (Jun 2019)|BOLD:AAN5358
Smittia|ZSM-DIP-33136-B10|Caroline Chimeno|BIN Taxonomy Match (Jan 2022)|BOLD:AAN5358
Smittia|BIOUG36677-A10|Kate Perez|BIN Taxonomy Match (May 2018)|BOLD:AAN5358
Smittia|BIOUG36625-B07|Kate Perez|BIN Taxonomy Match (Jul 2018)|BOLD:AAN5358
Smittia|BIOUG36624-G06|Kate Perez|BIN Taxonomy Match (Jul 2018)|BOLD:AAN5358
Smittia cf. stercoraria|Finnmark298|Elisabeth Stur||BOLD:AAN5358
Smittia|ZSM34343-E12|Sofia Wiedenbrug|Morphology|BOLD:ADC3582
Smittia|ZSM34343-E10|Sofia Wiedenbrug|Morphology|BOLD:ADC3582
Smittia sp. 20ES|ATNA384|Elisabeth Stur||BOLD:AAG3907
Smittia sp. 13ES|ATNA391|Elisabeth Stur||BOLD:AAG3906
Smittia sp. 13ES|ATNA381|Elisabeth Stur||BOLD:AAG3906
Smittia sp. 13ES|ATNA479|Elisabeth Stur||BOLD:AAG3906
Smittia sp. 13ES|Finnmark438|Elisabeth Stur||BOLD:AAG3906
Bryophaenocladius nigrus|ZSM-DIP-33134-H02|Caroline Chimeno|BIN Taxonomy Match (Jan 2022)|B...
Bryophaenocladius nigrus|ZSM-DIP-33134-H03|Caroline Chimeno|BIN Taxonomy Match (Jan 2022)|B...
Bryophaenocladius nigrus|BIOUG55187-B12|Stefan Schmidt|BIN Taxonomy Match (Sep 2020)|BOLD ...
Bryophaenocladius nigrus|BIOUG42719-C07|Kate Perez|BIN Taxonomy Match below Phylum (Jun 2019 ...
Bryophaenocladius nigrus|BIOUG36847-B04|Kate Perez|BIN Taxonomy Match (Jun 2019)|BOLD:AC...
Bryophaenocladius nigrus|BIOUG36844-H04|Kate Perez|BIN Taxonomy Match (Jun 2019)|BOLD:AC...
Bryophaenocladius nigrus|BIOUG36777-C04|Kate Perez|BIN Taxonomy Match (Jun 2019)|BOLD:AC...
Bryophaenocladius nigrus|BIOUG36787-E12|Kate Perez|BIN Taxonomy Match (Jun 2019)|BOLD:AC...
Bryophaenocladius nigrus|ZSM-DIP-33134-H06|Caroline Chimeno|BIN Taxonomy Match (Jan 2022)|B...
Bryophaenocladius nigrus|BIOUG55179-E12|Stefan Schmidt|BIN Taxonomy Match (Sep 2020)|BOLD ...
Chironomidae|BIOUG16736-H02|Jerome Moriniere|BIN Taxonomy Match|BOLD:ACR0396
Bryophaenocladius nigrus|BIOUG36776-E02|Kate Perez|BIN Taxonomy Match (Jun 2019)|BOLD:AC...
Bryophaenocladius nigrus|ZMUO.026127|Lauri Paasivirta||BOLD:ACR0396
Bryophaenocladius nigrus|ZSM-DIP-33134-H11|Caroline Chimeno|BIN Taxonomy Match (Jan 2022)|B...
Bryophaenocladius nigrus|ZSM-DIP-33142-A02|Caroline Chimeno|BIN Taxonomy Match (Jan 2022)|B...
Bryophaenocladius nigrus|ZSM-DIP-33134-H09|Caroline Chimeno|BIN Taxonomy Match (Jan 2022)|B...
Chironomidae|BIOUG16354-H11|Jerome Moriniere|BIN Taxonomy Match|BOLD:ACR0396
Smittia|ATNA553|Elisabeth Stur||BOLD:ACD2297
Smittia aterrima|Finnmark323|Elisabeth Stur||BOLD:AAV3380
Smittia aterrima|ATNA305|Elisabeth Stur||BOLD:AAE1362
Smittia aterrima|ATNA205|Elisabeth Stur||BOLD:AAE1362
Smittia aterrima|ATNA204|Elisabeth Stur||BOLD:AAE1362
Smittia aterrima|Finnmark605|Elisabeth Stur||BOLD:AAE1362
Smittia|CCDB24038-C05|Sofia Wiedenbrug|BIN Taxonomy Match|BOLD:ACF7218
Smittia|CCDB24038-A05|Sofia Wiedenbrug|BIN Taxonomy Match|BOLD:ACF7218
Smittia|BIOUG05217-B03|Kate Perez|BIN Taxonomy Match|BOLD:ACF7218
Smittia|BIOUG05149-G08|Kate Perez|BIN Taxonomy Match|BOLD:ACF7218
Smittia|BIOUG55104-C01|Kate Perez|BIN Taxonomy Match (Oct 2020)|BOLD:ACF7218
Smittia|ZSM-DIP-33139-B02|Caroline Chimeno|BIN Taxonomy Match (Jan 2022)|BOLD:ACF7218
Smittia|ZSM-DIP-33143-A06|Caroline Chimeno|BIN Taxonomy Match (Jan 2022)|BOLD:ACF7218
Smittia|CCDB24039-H07|Sofia Wiedenbrug|BIN Taxonomy Match|BOLD:ACF7218
Smittia|BIOUG16625-E05|Kate Perez|BIN Taxonomy Match|BOLD:ACF7218
Smittia|BIOUG15538-B11|Kate Perez|BIN Taxonomy Match|BOLD:ACF7218
Smittia sp. 21ES|CCDB24228-G02|Sofia Wiedenbrug|BIN Taxonomy Match|BOLD:AAG3903
Smittia sp. 21ES|ATNA277|Elisabeth Stur||BOLD:AAG3903
Smittia sp. 21ES|ATNA437|Elisabeth Stur||BOLD:AAG3903
Smittia sp. 14ES|Finnmark03|Elisabeth Stur||BOLD:AAM7064
Smittia sp. 14ES|BIOUG07505-D01|Kate Perez|BIN Taxonomy Match|BOLD:ACW5117
Smittia sp. 14ES|Finnmark61|Elisabeth Stur||BOLD:ACW5117
Smittia sp. 14ES|GBOL11558|Sofia Wiedenbrug||BOLD:ACW5117
Smittia leucopogon|ZMUO.024757|Lauri Paasivirta||BOLD:ACW5117
Smittia|BIOUG42959-A11|Kate Perez|BIN Taxonomy Match below Phylum (Jun 2019)|BOLD:ACW5117
Smittia|BIOUG42963-H06|Kate Perez|BIN Taxonomy Match below Phylum (Jun 2019)|BOLD:ACW5117
Smittia|BIOUG42964-B09|Kate Perez|BIN Taxonomy Match below Phylum (Jun 2019)|BOLD:ACW5117
Smittia|BIOUG42971-C04|Kate Perez|BIN Taxonomy Match below Phylum (Jun 2019)|BOLD:ACW5117
Smittia|BIOUG43074-C06|Kate Perez|BIN Taxonomy Match below Phylum (Jun 2019)|BOLD:ACW5117
Smittia sp. 14ES|BIOUG06093-D03|Kate Perez|BIN Taxonomy Match|BOLD:ACW5117
Smittia sp. 14ES|BIOUG06094-E02|Kate Perez|BIN Taxonomy Match|BOLD:ACW5117
Smittia sp. 14ES|BIOUG08094-A05|Kate Perez|BIN Taxonomy Match|BOLD:ACW5117
Smittia sp. 14ES|BIOUG06450-A08|Kate Perez|BIN Taxonomy Match|BOLD:ACW5117
Smittia sp. 14ES|BIOUG06209-G03|Kate Perez|BIN Taxonomy Match|BOLD:ACW5117
Cardiocladius capucinus|ZMUO.026218|Lauri Paasivirta||BOLD:AAI3695
Cardiocladius capucinus|ZMUO.026217|Lauri Paasivirta||BOLD:AAI3695
Cardiocladius capucinus|EBAI-Ch115|Elisabeth Stur|BOLD Identification Engine|BOLD:AAI3695
Cardiocladius capucinus|ATNA466|Elisabeth Stur||BOLD:AAI3695
Smittia aterrima|ATNA278|Elisabeth Stur||BOLD:AAE1363
Smittia aterrima|ATNA249|Elisabeth Stur||BOLD:AAE1363
Smittia aterrima|ATNA478|Elisabeth Stur||BOLD:AAE1363
Smittia aterrima|Finnmark551|Elisabeth Stur||BOLD:AAE1363
Smittia aterrima|Finnmark546|Elisabeth Stur||BOLD:AAE1363
Smittia aterrima|Finnmark490|Elisabeth Stur||BOLD:AAE1363
Smittia aterrima|Finnmark317|Elisabeth Stur||BOLD:AAE1363
Smittia aterrima|ZMUO.026168|Lauri Paasivirta||BOLD:AAG3905
Smittia aterrima|ATNA380|Elisabeth Stur||BOLD:AAG3905
Smittia aterrima|Finnmark70|Elisabeth Stur||BOLD:AAG3905
Smittia aterrima|Finnmark302|Elisabeth Stur||BOLD:AAG3905
Smittia scutellosetosa|BIOUG36678-C08|Kate Perez|BIN Taxonomy Match (Jul 2018)|BOLD:ACO4993
Smittia scutellosetosa|TRD-CH90|Elisabeth Stur||BOLD:ACO4993
Smittia scutellosetosa|TRD-CH89|Elisabeth Stur||BOLD:ACO4993
Smittia scutellosetosa|TRD-CH88|Elisabeth Stur||BOLD:ACO4993
Metriocnemus albolineatus|SOE348|Elisabeth Stur||BOLD:AAC6644
Metriocnemus albolineatus|CCDB24183-F06|Sofia Wiedenbrug|BIN-taxonomy|BOLD:AAC6643
Metriocnemus albolineatus|CCDB24183-F05|Sofia Wiedenbrug|Morphology|BOLD:AAC6643
Metriocnemus albolineatus|BIOUG15544-E08|Kate Perez|BIN Taxonomy Match|BOLD:AAC6643
Metriocnemus albolineatus|ZSM-DIP-33170-F03|Caroline Chimeno|BIN Taxonomy Match (Jan 2022)|...
Metriocnemus albolineatus|ZSM-DIP-33138-F10|Caroline Chimeno|BIN Taxonomy Match (Jan 2022)|...
Metriocnemus albolineatus|BIOUG15536-E06|Kate Perez|BIN Taxonomy Match|BOLD:AAC6643
Metriocnemus albolineatus|SOE369|Elisabeth Stur||BOLD:AAC6643
Metriocnemus albolineatus|ZMUO.024570|Lauri Paasivirta||BOLD:AAC6643
Metriocnemus albolineatus|CH-OSF118|Elisabeth Stur||BOLD:AAC6643

Metriocnemus albolineatus|SOE369|Elisabeth Stur||BOLD:AAC6643
Metriocnemus albolineatus|ZMUO.024570|Lauri Paasivirta||BOLD:AAC6643
Metriocnemus albolineatus|CH-OSF118|Elisabeth Stur||BOLD:AAC6643
Metriocnemus albolineatus|SOE374|Elisabeth Stur||BOLD:AAC6643
Metriocnemus albolineatus|SOE466|Elisabeth Stur||BOLD:AAC6643
Metriocnemus albolineatus|SOE52|Elisabeth Stur||BOLD:AAC6643
Metriocnemus albolineatus|SOE365|Elisabeth Stur||BOLD:AAC6643
Metriocnemus albolineatus|CH-eik11|Elisabeth Stur||BOLD:AAC6643
Metriocnemus albolineatus|CH-eik15|Elisabeth Stur||BOLD:AAC6643
Metriocnemus albolineatus|GBOL11502|Sofia Wiedenbrug|Morphology|BOLD:AAC6643
Metriocnemus albolineatus|ZSM-DIP-33143-D07|Caroline Chimeno|BIN Taxonomy Match (Jan 2022)|...
Metriocnemus albolineatus|ZSM-DIP-33134-F06|Caroline Chimeno|BIN Taxonomy Match (Jan 2022)|...
Metriocnemus albolineatus|ZSM-DIP-33146-F08|Caroline Chimeno|BIN Taxonomy Match (Jan 2022)|...
Metriocnemus albolineatus|ZSM-DIP-33170-G11|Caroline Chimeno|BIN Taxonomy Match (Jan 2022)|...
Metriocnemus albolineatus|ZSM-DIP-33137-D01|Caroline Chimeno|BIN Taxonomy Match (Jan 2022)|...
Metriocnemus albolineatus|ZSM34343-C08|Sofia Wiedenbrug|Morphology|BOLD:AAC6643
Metriocnemus albolineatus|BIOUG16430-D02|Kate Perez|BIN Taxonomy Match|BOLD:AAC6643
Metriocnemus albolineatus|CH-eik81|Elisabeth Stur||BOLD:AAC6643
Metriocnemus albolineatus|CH-eik60|Elisabeth Stur||BOLD:AAC6643
Metriocnemus albolineatus|CH-eik57|Elisabeth Stur||BOLD:AAC6643
Metriocnemus albolineatus|CH-eik82|Elisabeth Stur||BOLD:AAC6643
Metriocnemus albolineatus|CH-eik59|Elisabeth Stur||BOLD:AAC6643
Metriocnemus albolineatus|GBOL11504|Sofia Wiedenbrug|Morphology|BOLD:AAC6643
Metriocnemus albolineatus|CCDB21605-G04|Sofia Wiedenbrug||BOLD:AAC6643
Metriocnemus albolineatus|ZSM-DIP-33170-F01|Caroline Chimeno|BIN Taxonomy Match (Jan 2022)|...
Metriocnemus albolineatus|CCDB21605-B12|Sofia Wiedenbrug||BOLD:AAC6643
Metriocnemus albolineatus|ZSM-DIP-33149-H08|Caroline Chimeno|BIN Taxonomy Match (Jan 2022)|...
Metriocnemus albolineatus|BIOUG05143-D11|Kate Perez|BIN Taxonomy Match|BOLD:AAC6643
Metriocnemus albolineatus|BIOUG05105-H07|Kate Perez|BIN Taxonomy Match|BOLD:AAC6643
Metriocnemus albolineatus|SOE353|Elisabeth Stur||BOLD:AAC6643
Metriocnemus albolineatus|SOE346|Elisabeth Stur||BOLD:AAC6643
Metriocnemus albolineatus|SOE376|Elisabeth Stur||BOLD:AAC6643
Metriocnemus albolineatus|SOE344|Elisabeth Stur||BOLD:AAC6643
Metriocnemus albolineatus|BIOUG42721-D08|Kate Perez|BIN Taxonomy Match below Phylum (Jun 2019...
Metriocnemus albolineatus|BIOUG43081-H02|Kate Perez|BIN Taxonomy Match below Phylum (Jun 2019...
Metriocnemus albolineatus|ZMUO.024571|Lauri Paasivirta||BOLD:AAC6643
Metriocnemus albolineatus|Finnmark301|Elisabeth Stur||BOLD:AAC6643
Metriocnemus albolineatus|Finnmark97|Elisabeth Stur||BOLD:AAC6643
Metriocnemus albolineatus|TRD-CH43|Elisabeth Stur||BOLD:AAC6643
Metriocnemus albolineatus|TRD-CH59|Elisabeth Stur||BOLD:AAC6643
Metriocnemus albolineatus|TRD-CH412|Elisabeth Stur|morphology|BOLD:AAC6643
Metriocnemus albolineatus|BIOUG16632-C02|Kate Perez|BIN Taxonomy Match|BOLD:AAC6643
Metriocnemus albolineatus|BIOUG15458-D10|Kate Perez|BIN Taxonomy Match|BOLD:AAC6643
Metriocnemus albolineatus|BIOUG15457-D04|Kate Perez|BIN Taxonomy Match|BOLD:AAC6643
Metriocnemus albolineatus|TRD-CH237|Elisabeth Stur||BOLD:AAC6643
Metriocnemus albolineatus|BIOUG15545-B08|Kate Perez|BIN Taxonomy Match|BOLD:AAC6643
Metriocnemus albolineatus|BIOUG15454-D12|Kate Perez|BIN Taxonomy Match|BOLD:AAC6643
Metriocnemus albolineatus|BIOUG15797-D10|Kate Perez|BIN Taxonomy Match|BOLD:AAC6643
Metriocnemus albolineatus|BIOUG15719-A10|Kate Perez|BIN Taxonomy Match|BOLD:AAC6643
Metriocnemus albolineatus|BIOUG15459-A06|Kate Perez|BIN Taxonomy Match|BOLD:AAC6643
Metriocnemus albolineatus|BIOUG15460-E12|Kate Perez|BIN Taxonomy Match|BOLD:AAC6643
Metriocnemus albolineatus|BIOUG15201-G05|Kate Perez|BIN Taxonomy Match|BOLD:AAC6643
Metriocnemus albolineatus|BIOUG15455-A09|Kate Perez|BIN Taxonomy Match|BOLD:AAC6643
Metriocnemus albolineatus|BIOUG17064-E07|Kate Perez|BIN Taxonomy Match|BOLD:AAC6643
Metriocnemus albolineatus|BIOUG17065-D08|Kate Perez|BIN Taxonomy Match|BOLD:AAC6643
Metriocnemus albolineatus|BIOUG17065-D10|Kate Perez|BIN Taxonomy Match|BOLD:AAC6643
Metriocnemus albolineatus|BIOUG15184-H06|Kate Perez|BIN Taxonomy Match|BOLD:AAC6643
Metriocnemus albolineatus|BIOUG15458-B02|Kate Perez|BIN Taxonomy Match|BOLD:AAC6643
Metriocnemus albolineatus|BIOUG16434-A08|Kate Perez|BIN Taxonomy Match|BOLD:AAC6643
Metriocnemus albolineatus|BIOUG15456-H03|Kate Perez|BIN Taxonomy Match|BOLD:AAC6643
Metriocnemus albolineatus|BIOUG15675-F11|Kate Perez|BIN Taxonomy Match|BOLD:AAC6643
Metriocnemus albolineatus|BIOUG15460-H10|Kate Perez|BIN Taxonomy Match|BOLD:AAC6643
Metriocnemus albolineatus|BIOUG15455-F01|Kate Perez|BIN Taxonomy Match|BOLD:AAC6643
Metriocnemus albolineatus|BIOUG16625-H09|Kate Perez|BIN Taxonomy Match|BOLD:AAC6643
Metriocnemus albolineatus|BIOUG15454-F03|Kate Perez|BIN Taxonomy Match|BOLD:AAC6643
Metriocnemus albolineatus|BIOUG16480-F08|Kate Perez|BIN Taxonomy Match|BOLD:AAC6643
Metriocnemus albolineatus|BIOUG15542-C11|Kate Perez|BIN Taxonomy Match|BOLD:AAC6643
Metriocnemus albolineatus|BIOUG15950-D04|Kate Perez|BIN Taxonomy Match|BOLD:AAC6643
Metriocnemus albolineatus|BIOUG15718-A02|Kate Perez|BIN Taxonomy Match|BOLD:AAC6643
Metriocnemus albolineatus|NHRS-BYWS000000572|Yngve Brodin|morphology|BOLD:AAC6643
Metriocnemus albolineatus|Finnmark295|Elisabeth Stur||BOLD:AAC6643
Bryophaenocladius subparallelus|TRD-CH400|Elisabeth Stur|morphology|BOLD:ACU4987
Bryophaenocladius subparallelus|ZSM-DIP-33149-G04|Caroline Chimeno|BIN Taxonomy Match (Jan 202...
Bryophaenocladius subparallelus|ZSM-DIP-33141-F04|Caroline Chimeno|BIN Taxonomy Match (Jan 202...
Bryophaenocladius subparallelus|ZSM-DIP-33134-D04|Caroline Chimeno|BIN Taxonomy Match (Jan 202...
Bryophaenocladius subparallelus|ZSM-DIP-33132-C06|Caroline Chimeno|BIN Taxonomy Match (Jan 202...
Paraphaenocladius|CCDB24228-F05|Sofia Wiedenbrug|BIN Taxonomy Match|BOLD:ACU4987
Bryophaenocladius subparallelus|ZSM-DIP-33143-G08|Caroline Chimeno|BIN Taxonomy Match (Jan 202...
Bryophaenocladius subparallelus|NHRS-BYWS000000426|Yngve Brodin|morphology|BOLD:ACU4987
Bryophaenocladius subparallelus|NHRS-BYWS000001102|Yngve Brodin|morphology|BOLD:ACU4987
Bryophaenocladius subparallelus|NHRS-BYWS000001103|Yngve Brodin|morphology|BOLD:ACU4987
Bryophaenocladius subparallelus|NHRS-BYWS000000425|Yngve Brodin|morphology|BOLD:ACU4987
Smittia|CH-OSF80|Elisabeth Stur||BOLD:ABA7011
Tvetenia bavarica|Finnmark776|G. A. Halvorsen||BOLD:AAD2063
Tvetenia bavarica|EBAI-Ch108|Elisabeth Stur|BOLD Identification Engine|BOLD:AAD2063
Tvetenia bavarica|ZMUO.026114|Lauri Paasivirta||BOLD:AAD2063
Tvetenia bavarica|EBAI-Ch107|Elisabeth Stur|BOLD Identification Engine|BOLD:AAD2063
Tvetenia bavarica|ATNA5|Elisabeth Stur||BOLD:AAD2063
Tvetenia bavarica|SOE241|Elisabeth Stur||BOLD:AAD2063
Tvetenia bavarica|EBAI-Ch143|Elisabeth Stur|BOLD Identification Engine|BOLD:AAD2063
Tvetenia bavarica|EBAI-Ch207|Elisabeth Stur|morphology|BOLD:AAD2063
Tvetenia bavarica|BIOUG17023-A07|Kate Perez|BIN Taxonomy Match|BOLD:AAD2063
Tvetenia bavarica|BIOUG17023-E11|Kate Perez|BIN Taxonomy Match|BOLD:AAD2063
Tvetenia bavarica|CH-OSF61|Elisabeth Stur||BOLD:AAD2063
Tvetenia bavarica|ATNA488|Elisabeth Stur||BOLD:AAD2063
Tvetenia bavarica|ATNA207|Elisabeth Stur||BOLD:AAD2063
Tvetenia bavarica|ATNA281|Elisabeth Stur||BOLD:AAD2063
Tvetenia bavarica|EBAI-Ch144|Elisabeth Stur|BOLD Identification Engine|BOLD:AAD2063
Tvetenia bavarica|EBAI-Ch105|Elisabeth Stur|BOLD Identification Engine|BOLD:AAD2063
Tvetenia bavarica|EBAI-Ch138|Elisabeth Stur|BOLD Identification Engine|BOLD:AAD2063
Tvetenia bavarica|EBAI-Ch103|Elisabeth Stur|morphology|BOLD:AAD2063
Tvetenia bavarica|ATNA353|Elisabeth Stur||BOLD:AAD2063
Tvetenia bavarica|EBAI-Ch208|Elisabeth Stur|morphology

[illegible]
Tvetenia bavarica|EBAI-Ch103|Elisabeth Stur|morphology|BOLD:AAD2063
Tvetenia bavarica|ATNA353|Elisabeth Stur||BOLD:AAD2063
Tvetenia bavarica|EBAI-Ch208|Elisabeth Stur|morphology
Tvetenia bavarica|ATNA487|Elisabeth Stur||BOLD:AAD2063
Tvetenia bavarica|ZSM34343-A05|Sofia Wiedenbrug|Morphology|BOLD:AAD2063
Tvetenia bavarica|CCDB24039-G09|Sofia Wiedenbrug|BIN Taxonomy Match|BOLD:AAD2063
Tvetenia|ZSM34342-E02|Sofia Wiedenbrug|Morphology|BOLD:AAD2063
Tvetenia bavarica|CCDB24037-A05|Sofia Wiedenbrug|BIN Taxonomy Match|BOLD:AAD2063
Tvetenia bavarica|CCDB24037-H02|Sofia Wiedenbrug|BIN Taxonomy Match|BOLD:AAD2063
Tvetenia bavarica|BC-ZSM-DIP-22491-H01|Dieter Doczkal||BOLD:AAD2063
Tvetenia bavarica|CCDB24037-F07|Sofia Wiedenbrug|BIN Taxonomy Match|BOLD:AAD2063
Tvetenia bavarica|CCDB24229-A03|Sofia Wiedenbrug|BIN Taxonomy Match|BOLD:AAD2063
Tvetenia bavarica|CCDB24039-A02|Sofia Wiedenbrug|BIN Taxonomy Match|BOLD:AAD2063
Tvetenia bavarica|CCDB24037-E11|Sofia Wiedenbrug|BIN Taxonomy Match|BOLD:AAD2063
Tvetenia bavarica|CCDB24039-H03|Sofia Wiedenbrug|BIN Taxonomy Match|BOLD:AAD2063
Tvetenia bavarica|CCDB24039-G12|Sofia Wiedenbrug|BIN Taxonomy Match|BOLD:AAD2063
Tvetenia bavarica|CCDB24037-E01|Sofia Wiedenbrug|BIN Taxonomy Match|BOLD:AAD2063
Tvetenia bavarica|CCDB24037-E03|Sofia Wiedenbrug||BOLD:AAD2063
Tvetenia bavarica|CCDB24037-E10|Sofia Wiedenbrug|BIN Taxonomy Match|BOLD:AAD2063
Tvetenia bavarica|CCDB24037-E08|Sofia Wiedenbrug|BIN Taxonomy Match|BOLD:AAD2063
Tvetenia bavarica|CCDB24229-F12|Sofia Wiedenbrug|BIN Taxonomy Match|BOLD:AAD2063
Tvetenia bavarica|CCDB24037-B04|Sofia Wiedenbrug|BIN Taxonomy Match|BOLD:AAD2063
Tvetenia bavarica|BC-ZSM-DIP-22491-G09|Dieter Doczkal||BOLD:AAD2063
Tvetenia|ZSM34343-B09|Sofia Wiedenbrug|Morphology|BOLD:AAD2063
Tvetenia bavarica|CCDB24039-A04|Sofia Wiedenbrug|BIN Taxonomy Match|BOLD:AAD2063
Tvetenia bavarica|CCDB24038-H04|Sofia Wiedenbrug|BIN Taxonomy Match|BOLD:AAD2063
Tvetenia bavarica|CCDB24039-G11|Sofia Wiedenbrug|BIN Taxonomy Match|BOLD:AAD2063
Tvetenia bavarica|ZSM34343-H04|Sofia Wiedenbrug|Morphology|BOLD:AAD2063
Tvetenia bavarica|CCDB24037-B05|Sofia Wiedenbrug|BIN Taxonomy Match|BOLD:AAD2063
Tvetenia bavarica|CCDB24037-B03|Sofia Wiedenbrug|BIN Taxonomy Match|BOLD:AAD2063
Tvetenia bavarica|BC-ZSM-DIP-22551-E02|Dieter Doczkal||BOLD:AAD2063
Tvetenia bavarica|BC-ZSM-DIP-22491-D06|Dieter Doczkal||BOLD:AAD2063
Tvetenia bavarica|ES39|Elisabeth Stur||BOLD:AAD2063
Tvetenia bavarica|CCDB24039-H11|Sofia Wiedenbrug|BIN Taxonomy Match|BOLD:AAD2063
Tvetenia bavarica|CCDB21606-F06|Sofia Wiedenbrug||BOLD:AAD2063
Tvetenia bavarica|CCDB24039-C07|Sofia Wiedenbrug|BIN Taxonomy Match|BOLD:AAD2063
Tvetenia bavarica|BC-ZSM-DIP-22551-E08|Dieter Doczkal||BOLD:AAD2063
Tvetenia bavarica|CCDB24038-C11|Sofia Wiedenbrug|BIN Taxonomy Match|BOLD:AAD2063
Tvetenia bavarica|CCDB24038-C10|Sofia Wiedenbrug|BIN Taxonomy Match|BOLD:AAD2063
Tvetenia bavarica|CCDB24037-H03|Sofia Wiedenbrug|BIN Taxonomy Match|BOLD:AAD2063
Tvetenia bavarica|CCDB24037-E09|Sofia Wiedenbrug|BIN Taxonomy Match|BOLD:AAD2063
Tvetenia bavarica|CCDB24039-D07|Sofia Wiedenbrug|BIN Taxonomy Match|BOLD:AAD2063
Tvetenia bavarica|ES175|Elisabeth Stur||BOLD:AAD2063
Tvetenia bavarica|ES16|Elisabeth Stur||BOLD:AAD2063
Tvetenia bavarica|ZMUO.024836|Lauri Paasivirta||BOLD:AAD2063
Tvetenia bavarica|ZMUO.024835|Lauri Paasivirta||BOLD:AAD2063
Tvetenia bavarica|Finnmark788|Elisabeth Stur||BOLD:AAD2063
Tvetenia bavarica|ZMUO.025853|Lauri Paasivirta||BOLD:AAD2063
Tvetenia bavarica|ZMUO.025852|Lauri Paasivirta||BOLD:AAD2063
Tvetenia bavarica|Finnmark548|Elisabeth Stur||BOLD:AAD2063
Orthocladius sp. 9TE|ATNA315|Torbjorn Ekrem||BOLD:AAD4029
Orthocladius sp. 9TE|Finnmark355|Torbjorn Ekrem||BOLD:AAD4029
Orthocladius sp. 9TE|ATNA442|Torbjorn Ekrem||BOLD:AAD4029
Orthocladius sp. 9TE|ATNA313|Elisabeth Stur||BOLD:AAD4029
Orthocladius sp. 9TE|ATNA229|Elisabeth Stur||BOLD:AAD4029
Orthocladius sp. 9TE|ATNA441|Torbjorn Ekrem||BOLD:AAD4029
Orthocladius sp. 9TE|ATNA438|Torbjorn Ekrem||BOLD:AAD4029
Orthocladius sp. 9TE|ATNA443|Torbjorn Ekrem||BOLD:AAD4029
Orthocladius sp. 9TE|Finnmark642|Torbjorn Ekrem||BOLD:AAD4029
Orthocladius gelidorum|ATNA424|Torbjorn Ekrem||BOLD:AAI3489
Orthocladius gelidorum|ATNA434|Elisabeth Stur||BOLD:AAI3489
Orthocladius gelidorum|ES389|Elisabeth Stur||BOLD:AAI3489
Orthocladius gelidorum|ATNA418|Torbjorn Ekrem||BOLD:AAI3489
Orthocladius gelidorum|ATNA473|Elisabeth Stur||BOLD:AAI3489
Orthocladius gelidorum|ATNA433|Elisabeth Stur||BOLD:AAI3489
Orthocladius gelidorum|ATNA419|Torbjorn Ekrem||BOLD:AAI3489
Orthocladius gelidorum|Finnmark608|Torbjorn Ekrem||BOLD:AAI3489
Orthocladius sp. 6TE|ATNA439|Torbjorn Ekrem||BOLD:AAI3490
Orthocladius olivaceus|ATNA314|Torbjorn Ekrem||BOLD:AAI3491
Orthocladius olivaceus|ATNA444|Torbjorn Ekrem||BOLD:AAI3491
Orthocladius olivaceus|ATNA440|Torbjorn Ekrem||BOLD:AAI3491
Orthocladius sp. 8TE|ATNA300|Torbjorn Ekrem||BOLD:AAI3488
Orthocladius sp. 8TE|ATNA299|Torbjorn Ekrem||BOLD:AAI3488
Orthocladius sp. 7TE|Finnmark406|Torbjorn Ekrem||BOLD:AAV5076
Orthocladius gelidorum|ZMUO.026015|Lauri Paasivirta||BOLD:ADF2399
Orthocladius gelidorum|ZMUO.029781|Marko Mutanen||BOLD:ADF2399
Orthocladius|ZMUO.025842|Lauri Paasivirta||BOLD:ADF2399
Orthocladius|ZMUO.025911|Lauri Paasivirta||BOLD:ADF2399
Orthocladius|ZMUO.025910|Lauri Paasivirta||BOLD:ADF2399
Orthocladius cf. sp. 2TE|BIOUG16713-C08|Elisabeth Stur|BIN Taxonomy Match|BOLD:ACE0526
Orthocladius cf. sp. 2TE|BIOUG15963-F10|Elisabeth Stur|BIN Taxonomy Match|BOLD:ACE0526
Orthocladius cf. sp. 2TE|BIOUG17064-C07|Elisabeth Stur|BIN Taxonomy Match|BOLD:ACE0526
Orthocladius cf. sp. 2TE|TRD-CH331|Elisabeth Stur|morphology|BOLD:ACE0526
Orthocladius olivaceus|NHRS-BYWS000001100|Yngve Brodin|morphology|BOLD:ACE0526
Orthocladius olivaceus|NHRS-BYWS000001099|Yngve Brodin|morphology|BOLD:ACE0526
Orthocladius olivaceus|NHRS-BYWS000000166|Yngve Brodin|morphology|BOLD:ACE0526
Orthocladius cf. sp. 2TE|TRD-CH164|Elisabeth Stur|morphology|BOLD:ACE0526
Orthocladius cf. sp. 2TE|BIOUG15953-H01|Elisabeth Stur|BIN Taxonomy Match|BOLD:ACE0526
Orthocladius cf. sp. 2TE|TRD-CH142|Elisabeth Stur|morphology|BOLD:ACE0526
Orthocladius|BIOUG04795-G11|Kate Perez|BIN Taxonomy Match|BOLD:ACE0526
Orthocladius olivaceus|ZMUO.026178|Lauri Paasivirta||BOLD:AER2991
Orthocladius sp. 2TE|Finnmark634|Torbjorn Ekrem||BOLD:AER2991
Orthocladius priomixtus|ZMUO.026293|Lauri Paasivirta||BOLD:AAD4028
Orthocladius subletteorum|CCDB24183-F10|Sofia Wiedenbrug|Morphology|BOLD:AAD4028
Orthocladius subletteorum|ATNA377|Torbjorn Ekrem||BOLD:AAD4028
Orthocladius priomixtus|ZMUO.026294|Lauri Paasivirta||BOLD:AAD4028
Orthocladius subletteorum|SOE38|Torbjorn Ekrem||BOLD:AAD4028
Orthocladius subletteorum|SOE37|Torbjorn Ekrem||BOLD:AAD4028
Orthocladius subletteorum|Finnmark596|Torbjorn Ekrem||BOLD:AAD4028
Chironomidae|CCDB24228-F03||BIN Taxonomy Match
Orthocladius|BC-ZSM-DIP-22551-E04|Dieter Doczkal||BOLD:ACX2763
Orthocladius|BC-ZSM-DIP-22551-D07|Dieter Doczkal||BOLD:ACX2369
[illegible]

Chironomidae|CCDB24228-F03||BIN Taxonomy Match
Orthocladius|BC-ZSM-DIP-22551-E04|Dieter Doczkal||BOLD:ACX2763
Orthocladius|BC-ZSM-DIP-22551-D07|Dieter Doczkal||BOLD:ACX2369
Orthocladius|BC-ZSM-DIP-22551-D01|Dieter Doczkal||BOLD:ACX2369
Orthocladius|CCDB24038-H09|Sofia Wiedenbrug|BIN Taxonomy Match|BOLD:ACU4613
Orthocladius musester|ZMUO.025249|Lauri Paasivirta||BOLD:ACY5137
Orthocladius musester|ZMUO.025250|Lauri Paasivirta||BOLD:ACY5137
Orthocladius fuscimanus|PP0265|Lauri Paasivirta||BOLD:ACY5137
Orthocladius|BC-ZSM-DIP-22491-H04|Dieter Doczkal||BOLD:ACX2009
Orthocladius|BC-ZSM-DIP-22488-F05|Dieter Doczkal||BOLD:ACU4932
Orthocladius|CCDB24038-H07|Sofia Wiedenbrug|BIN Taxonomy Match|BOLD:ACU4932
Orthocladius|BIOUG16332-F06|Elisabeth Stur|morphology|BOLD:ACP7936
Orthocladius fuscimanus|BC-ZSM-DIP-22551-E11|Dieter Doczkal||BOLD:ACX3046
Orthocladius fuscimanus|BC-ZSM-DIP-22488-G11|Dieter Doczkal||BOLD:AAV5075
Orthocladius fuscimanus|CCDB24038-G08|Sofia Wiedenbrug|BIN Taxonomy Match|BOLD:AAV5075
Orthocladius fuscimanus|Finnmark333|Torbjorn Ekrem||BOLD:AAV5075
Gymnometriocnemus marionensis|Finnmark06|Elisabeth Stur|Morphology|BOLD:AAM7065
Gymnometriocnemus subnudus|BIOUG06210-D04|Kate Perez|BIN Taxonomy Match|BOLD:AAI6451
Gymnometriocnemus subnudus|BIOUG05484-C11|Kate Perez|BIN Taxonomy Match|BOLD:AAI6451
Chironomidae|BIOUG05269-C05|Meredith Miller|Tree-based Identification (Feb 2019)
Gymnometriocnemus subnudus|BIOUG05267-C05|Kate Perez|BIN Taxonomy Match|BOLD:AAI6451
Gymnometriocnemus subnudus|BIOUG05144-F01|Kate Perez|BIN Taxonomy Match|BOLD:AAI6451
Gymnometriocnemus subnudus|BIOUG05219-D09|Kate Perez|BIN Taxonomy Match|BOLD:AAI6451
Gymnometriocnemus subnudus|BIOUG05219-C05|Kate Perez|BIN Taxonomy Match|BOLD:AAI6451
Gymnometriocnemus subnudus|BIOUG05219-F11|Kate Perez|BIN Taxonomy Match|BOLD:AAI6451
Gymnometriocnemus subnudus|BIOUG05483-A07|Kate Perez|BIN Taxonomy Match|BOLD:AAI6451
Gymnometriocnemus subnudus|CH-OSF123|Elisabeth Stur||BOLD:AAI6451
Gymnometriocnemus subnudus|BIOUG05215-D07|Kate Perez|BIN Taxonomy Match|BOLD:AAI6451
Gymnometriocnemus subnudus|BIOUG05218-D02|Kate Perez|BIN Taxonomy Match|BOLD:AAI6451
Gymnometriocnemus subnudus|BIOUG05267-B03|Kate Perez|BIN Taxonomy Match|BOLD:AAI6451
Gymnometriocnemus subnudus|CH-OSF135|Elisabeth Stur||BOLD:AAI6451
Gymnometriocnemus subnudus|BIOUG05149-D12|Kate Perez|BIN Taxonomy Match|BOLD:AAI6451
Gymnometriocnemus subnudus|BIOUG06058-B11|Kate Perez|BIN Taxonomy Match|BOLD:AAI6451
Gymnometriocnemus subnudus|BIOUG05141-G01|Kate Perez|BIN Taxonomy Match|BOLD:AAI6451
Gymnometriocnemus subnudus|BIOUG05218-H03|Kate Perez|BIN Taxonomy Match|BOLD:AAI6451
Gymnometriocnemus subnudus|BIOUG05219-G09|Kate Perez|BIN Taxonomy Match|BOLD:AAI6451
Gymnometriocnemus subnudus|BIOUG05215-C06|Kate Perez|BIN Taxonomy Match|BOLD:AAI6451
Gymnometriocnemus subnudus|BIOUG05216-B01|Kate Perez|BIN Taxonomy Match|BOLD:AAI6451
Gymnometriocnemus subnudus|BIOUG05216-B08|Kate Perez|BIN Taxonomy Match|BOLD:AAI6451
Gymnometriocnemus subnudus|BIOUG05216-D02|Kate Perez|BIN Taxonomy Match|BOLD:AAI6451
Gymnometriocnemus subnudus|BIOUG05216-G05|Kate Perez|BIN Taxonomy Match|BOLD:AAI6451
Gymnometriocnemus subnudus|BIOUG05217-E03|Kate Perez|BIN Taxonomy Match|BOLD:AAI6451
Gymnometriocnemus subnudus|BIOUG05219-F07|Kate Perez|BIN Taxonomy Match|BOLD:AAI6451
Gymnometriocnemus subnudus|BIOUG05217-H06|Kate Perez|BIN Taxonomy Match|BOLD:AAI6451
Gymnometriocnemus subnudus|BIOUG05216-E04|Kate Perez|BIN Taxonomy Match|BOLD:AAI6451
Gymnometriocnemus subnudus|BIOUG05219-B01|Kate Perez|BIN Taxonomy Match|BOLD:AAI6451
Gymnometriocnemus subnudus|BIOUG05486-C06|Kate Perez|BIN Taxonomy Match|BOLD:AAI6451
Gymnometriocnemus subnudus|BIOUG05395-G08|Kate Perez|BIN Taxonomy Match|BOLD:AAI6451
Gymnometriocnemus subnudus|BIOUG05945-B09|Kate Perez|BIN Taxonomy Match|BOLD:AAI6451
Gymnometriocnemus subnudus|BIOUG05485-C05|Kate Perez|BIN Taxonomy Match|BOLD:AAI6451
Gymnometriocnemus subnudus|BIOUG05396-B10|Kate Perez|BIN Taxonomy Match|BOLD:AAI6451
Gymnometriocnemus subnudus|BIOUG05396-H08|Kate Perez|BIN Taxonomy Match|BOLD:AAI6451
Gymnometriocnemus subnudus|BIOUG06055-F11|Kate Perez|BIN Taxonomy Match|BOLD:AAI6451
Gymnometriocnemus subnudus|BIOUG05219-B02|Kate Perez|BIN Taxonomy Match|BOLD:AAI6451
Gymnometriocnemus subnudus|BIOUG05149-G02|Kate Perez|BIN Taxonomy Match|BOLD:AAI6451
Gymnometriocnemus subnudus|BIOUG05218-G11|Kate Perez|BIN Taxonomy Match|BOLD:AAI6451
Gymnometriocnemus subnudus|BIOUG05149-C12|Kate Perez|BIN Taxonomy Match|BOLD:AAI6451
Gymnometriocnemus subnudus|BIOUG05396-D02|Kate Perez|BIN Taxonomy Match|BOLD:AAI6451
Gymnometriocnemus subnudus|BIOUG05396-G08|Kate Perez|BIN Taxonomy Match|BOLD:AAI6451
Gymnometriocnemus subnudus|CH-OSF88|Elisabeth Stur||BOLD:AAI6451
Gymnometriocnemus subnudus|BIOUG15597-C05|Kate Perez|BIN Taxonomy Match|BOLD:AAI6451
Gymnometriocnemus subnudus|BIOUG05483-D12|Kate Perez|BIN Taxonomy Match|BOLD:AAI6451
Gymnometriocnemus subnudus|BIOUG05400-D09|Kate Perez|BIN Taxonomy Match|BOLD:AAI6451
Gymnometriocnemus subnudus|BIOUG15543-E03|Kate Perez|BIN Taxonomy Match|BOLD:AAI6451
Gymnometriocnemus subnudus|BIOUG05486-D02|Kate Perez|BIN Taxonomy Match|BOLD:AAI6451
Gymnometriocnemus subnudus|BIOUG05220-A04|Kate Perez|BIN Taxonomy Match|BOLD:AAI6451
Gymnometriocnemus subnudus|BIOUG15539-B06|Kate Perez|BIN Taxonomy Match|BOLD:AAI6451
Gymnometriocnemus subnudus|BIOUG06058-A08|Kate Perez|BIN Taxonomy Match|BOLD:AAI6451
Gymnometriocnemus subnudus|BIOUG06054-D05|Kate Perez|BIN Taxonomy Match|BOLD:AAI6451
Gymnometriocnemus subnudus|BIOUG06047-A04|Kate Perez|BIN Taxonomy Match|BOLD:AAI6451
Gymnometriocnemus subnudus|ZMUO.024701|Lauri Paasivirta||BOLD:AAI6451
Gymnometriocnemus subnudus|BIOUG05109-E12|Kate Perez|BIN Taxonomy Match|BOLD:AAI6451
Gymnometriocnemus subnudus|BIOUG05218-G02|Kate Perez|BIN Taxonomy Match|BOLD:AAI6451
Gymnometriocnemus subnudus|BIOUG05219-E12|Kate Perez|BIN Taxonomy Match|BOLD:AAI6451
Gymnometriocnemus subnudus|BIOUG05215-E12|Kate Perez|BIN Taxonomy Match|BOLD:AAI6451
Gymnometriocnemus subnudus|BIOUG05400-D10|Kate Perez|BIN Taxonomy Match|BOLD:AAI6451
Gymnometriocnemus subnudus|BIOUG05102-C12|Kate Perez|BIN Taxonomy Match|BOLD:AAI6451
Gymnometriocnemus subnudus|BIOUG05105-A03|Kate Perez|BIN Taxonomy Match|BOLD:AAI6451
Gymnometriocnemus subnudus|BIOUG05105-D11|Kate Perez|BIN Taxonomy Match|BOLD:AAI6451
Gymnometriocnemus subnudus|BIOUG05217-H05|Kate Perez|BIN Taxonomy Match|BOLD:AAI6451
Gymnometriocnemus subnudus|BIOUG05217-E04|Kate Perez|BIN Taxonomy Match|BOLD:AAI6451
Gymnometriocnemus subnudus|BIOUG05218-H01|Kate Perez|BIN Taxonomy Match|BOLD:AAI6451
Gymnometriocnemus subnudus|BIOUG05218-H07|Kate Perez|BIN Taxonomy Match|BOLD:AAI6451
Gymnometriocnemus subnudus|BIOUG05219-B08|Kate Perez|BIN Taxonomy Match|BOLD:AAI6451
Gymnometriocnemus subnudus|BIOUG05217-G06|Kate Perez|BIN Taxonomy Match|BOLD:AAI6451
Gymnometriocnemus subnudus|BIOUG05217-G12|Kate Perez|BIN Taxonomy Match|BOLD:AAI6451
Gymnometriocnemus subnudus|BIOUG05102-A08|Kate Perez|BIN Taxonomy Match|BOLD:AAI6451
Gymnometriocnemus subnudus|BIOUG05215-G04|Kate Perez|BIN Taxonomy Match|BOLD:AAI6451
Gymnometriocnemus subnudus|BIOUG05216-C01|Kate Perez|BIN Taxonomy Match|BOLD:AAI6451
Gymnometriocnemus subnudus|BIOUG05216-G03|Kate Perez|BIN Taxonomy Match|BOLD:AAI6451
Gymnometriocnemus subnudus|BIOUG05216-G06|Kate Perez|BIN Taxonomy Match|BOLD:AAI6451
Gymnometriocnemus subnudus|BIOUG05216-H04|Kate Perez|BIN Taxonomy Match|BOLD:AAI6451
Gymnometriocnemus subnudus|BIOUG05219-E06|Kate Perez|BIN Taxonomy Match|BOLD:AAI6451
Gymnometriocnemus subnudus|BIOUG05944-E12|Kate Perez|BIN Taxonomy Match|BOLD:AAI6451
Gymnometriocnemus subnudus|BIOUG05947-F10|Kate Perez|BIN Taxonomy Match|BOLD:AAI6451
Gymnometriocnemus subnudus|BIOUG06054-H05|Kate Perez|BIN Taxonomy Match|BOLD:AAI6451
Gymnometriocnemus subnudus|BIOUG05105-D07|Kate Perez|BIN Taxonomy Match|BOLD:AAI6451
Gymnometriocnemus subnudus|BIOUG05217-G10|Kate Perez|BIN Taxonomy Match|BOLD:AAI6451
Gymnometriocnemus subnudus|BIOUG05946-H09|Kate Perez|BIN Taxonomy Match|BOLD:AAI6451
Gymnometriocnemus subnudus|BIOUG05215-B05|Kate Perez|BIN Taxonomy Match|BOLD:AAI6451
Gymnometriocnemus subnudus|BIOUG05780-E06|Kate Perez|BIN Taxonomy Match|BOLD:AAI6451
Gymnometriocnemus subnudus|BIOUG05900-G08|Kate Perez|BIN Taxonomy Match|BOLD:AAI6451
Gymnometriocnemus subnudus|BIOUG05149-B01|Kate Perez|BIN Taxonomy Match|BOLD:AAI6451

Gymnometriocnemus subnudus|BIOUG05780-E06|Kate Perez|BIN Taxonomy Match|BOLD:AAI6451
Gymnometriocnemus subnudus|BIOUG05900-G08|Kate Perez|BIN Taxonomy Match|BOLD:AAI6451
Gymnometriocnemus subnudus|BIOUG05149-B01|Kate Perez|BIN Taxonomy Match|BOLD:AAI6451
Gymnometriocnemus subnudus|BIOUG05149-C01|Kate Perez|BIN Taxonomy Match|BOLD:AAI6451
Gymnometriocnemus subnudus|BIOUG05149-G09|Kate Perez|BIN Taxonomy Match|BOLD:AAI6451
Gymnometriocnemus subnudus|BIOUG05780-G05|Kate Perez|BIN Taxonomy Match|BOLD:AAI6451
Gymnometriocnemus subnudus|BIOUG05483-B09|Kate Perez|BIN Taxonomy Match|BOLD:AAI6451
Gymnometriocnemus subnudus|BIOUG05141-A09|Kate Perez|BIN Taxonomy Match|BOLD:AAI6451
Gymnometriocnemus subnudus|BIOUG05483-A06|Kate Perez|BIN Taxonomy Match|BOLD:AAI6451
Gymnometriocnemus subnudus|BIOUG05944-A06|Kate Perez|BIN Taxonomy Match|BOLD:AAI6451
Gymnometriocnemus subnudus|BIOUG05400-D11|Kate Perez|BIN Taxonomy Match|BOLD:AAI6451
Gymnometriocnemus subnudus|BIOUG05220-C02|Kate Perez|BIN Taxonomy Match|BOLD:AAI6451
Gymnometriocnemus subnudus|BIOUG05897-D05|Kate Perez|BIN Taxonomy Match|BOLD:AAI6451
Gymnometriocnemus subnudus|BIOUG05267-B01|Kate Perez|BIN Taxonomy Match|BOLD:AAI6451
Gymnometriocnemus subnudus|BIOUG05486-G03|Kate Perez|BIN Taxonomy Match|BOLD:AAI6451
Gymnometriocnemus subnudus|BIOUG05220-F03|Kate Perez|BIN Taxonomy Match|BOLD:AAI6451
Gymnometriocnemus subnudus|BIOUG05220-B11|Kate Perez|BIN Taxonomy Match|BOLD:AAI6451
Gymnometriocnemus subnudus|BIOUG05947-H03|Kate Perez|BIN Taxonomy Match|BOLD:AAI6451
Gymnometriocnemus subnudus|BIOUG05267-G04|Kate Perez|BIN Taxonomy Match|BOLD:AAI6451
Gymnometriocnemus subnudus|BIOUG05484-D04|Kate Perez|BIN Taxonomy Match|BOLD:AAI6451
Gymnometriocnemus subnudus|BIOUG05267-B11|Kate Perez|BIN Taxonomy Match|BOLD:AAI6451
Gymnometriocnemus subnudus|BIOUG05267-E03|Kate Perez|BIN Taxonomy Match|BOLD:AAI6451
Gymnometriocnemus subnudus|BIOUG05149-G07|Kate Perez|BIN Taxonomy Match|BOLD:AAI6451
Gymnometriocnemus subnudus|ZMUO.024700|Lauri Paasivirta||BOLD:AAI6451
Gymnometriocnemus subnudus|BIOUG05217-A12|Kate Perez|BIN Taxonomy Match|BOLD:AAI6451
Gymnometriocnemus subnudus|BIOUG15591-E05|Kate Perez|BIN Taxonomy Match|BOLD:AAI6451
Gymnometriocnemus subnudus|BIOUG15540-D07|Kate Perez|BIN Taxonomy Match|BOLD:AAI6451
Gymnometriocnemus subnudus|BIOUG05220-D06|Kate Perez|BIN Taxonomy Match|BOLD:AAI6451
Gymnometriocnemus subnudus|BIOUG15763-F08|Kate Perez|BIN Taxonomy Match|BOLD:AAI6451
Gymnometriocnemus subnudus|BIOUG05486-F09|Kate Perez|BIN Taxonomy Match|BOLD:AAI6451
Gymnometriocnemus subnudus|BIOUG05215-G08|Kate Perez|BIN Taxonomy Match|BOLD:AAI6451
Gymnometriocnemus subnudus|BIOUG05215-F11|Kate Perez|BIN Taxonomy Match|BOLD:AAI6451
Gymnometriocnemus subnudus|BIOUG05215-F12|Kate Perez|BIN Taxonomy Match|BOLD:AAI6451
Gymnometriocnemus subnudus|BIOUG05215-B09|Kate Perez|BIN Taxonomy Match|BOLD:AAI6451
Gymnometriocnemus subnudus|BIOUG05216-G11|Kate Perez|BIN Taxonomy Match|BOLD:AAI6451
Gymnometriocnemus subnudus|BIOUG06056-B04|Kate Perez|BIN Taxonomy Match|BOLD:AAI6451
Gymnometriocnemus subnudus|BIOUG15800-D02|Kate Perez|BIN Taxonomy Match|BOLD:AAI6451
Gymnometriocnemus subnudus|BIOUG15808-E05|Kate Perez|BIN Taxonomy Match|BOLD:AAI6451
Gymnometriocnemus subnudus|BIOUG05267-A07|Kate Perez|BIN Taxonomy Match|BOLD:AAI6451
Gymnometriocnemus subnudus|BIOUG05220-B09|Kate Perez|BIN Taxonomy Match|BOLD:AAI6451
Gymnometriocnemus subnudus|BIOUG05219-B09|Kate Perez|BIN Taxonomy Match|BOLD:AAI6451
Gymnometriocnemus subnudus|BIOUG05216-B02|Kate Perez|BIN Taxonomy Match|BOLD:AAI6451
Gymnometriocnemus subnudus|ATNA398|Elisabeth Stur||BOLD:AAI6451
Gymnometriocnemus subnudus|BIOUG05216-G02|Kate Perez|BIN Taxonomy Match|BOLD:AAI6451
Gymnometriocnemus subnudus|BIOUG06058-H02|Kate Perez|BIN Taxonomy Match|BOLD:AAI6451
Gymnometriocnemus subnudus|BIOUG05219-E10|Kate Perez|BIN Taxonomy Match|BOLD:AAI6451
Gymnometriocnemus subnudus|BIOUG05219-D11|Kate Perez|BIN Taxonomy Match|BOLD:AAI6451
Gymnometriocnemus subnudus|BIOUG05219-C10|Kate Perez|BIN Taxonomy Match|BOLD:AAI6451
Gymnometriocnemus subnudus|BIOUG05218-G09|Kate Perez|BIN Taxonomy Match|BOLD:AAI6451
Gymnometriocnemus subnudus|BIOUG06062-G03|Kate Perez|BIN Taxonomy Match|BOLD:AAI6451
Gymnometriocnemus subnudus|BIOUG05105-F05|Kate Perez|BIN Taxonomy Match|BOLD:AAI6451
Gymnometriocnemus subnudus|BIOUG05215-D02|Kate Perez|BIN Taxonomy Match|BOLD:AAI6451
Gymnometriocnemus subnudus|BIOUG05149-F07|Kate Perez|BIN Taxonomy Match|BOLD:AAI6451
Gymnometriocnemus subnudus|BIOUG04795-B10|Kate Perez|BIN Taxonomy Match|BOLD:AAI6451
Gymnometriocnemus subnudus|BIOUG05107-C01|Kate Perez|BIN Taxonomy Match|BOLD:AAI6451
Gymnometriocnemus subnudus|BIOUG05218-G08|Kate Perez|BIN Taxonomy Match|BOLD:AAI6451
Gymnometriocnemus subnudus|BIOUG05219-C01|Kate Perez|BIN Taxonomy Match|BOLD:AAI6451
Gymnometriocnemus subnudus|BIOUG05215-H01|Kate Perez|BIN Taxonomy Match|BOLD:AAI6451
Gymnometriocnemus subnudus|BIOUG05215-F10|Kate Perez|BIN Taxonomy Match|BOLD:AAI6451
Gymnometriocnemus subnudus|BIOUG05216-A11|Kate Perez|BIN Taxonomy Match|BOLD:AAI6451
Gymnometriocnemus subnudus|BIOUG05216-C11|Kate Perez|BIN Taxonomy Match|BOLD:AAI6451
Gymnometriocnemus subnudus|BIOUG05216-D08|Kate Perez|BIN Taxonomy Match|BOLD:AAI6451
Gymnometriocnemus subnudus|BIOUG05216-E05|Kate Perez|BIN Taxonomy Match|BOLD:AAI6451
Gymnometriocnemus subnudus|BIOUG05216-F04|Kate Perez|BIN Taxonomy Match|BOLD:AAI6451
Gymnometriocnemus subnudus|BIOUG05217-B08|Kate Perez|BIN Taxonomy Match|BOLD:AAI6451
Gymnometriocnemus subnudus|BIOUG05217-D03|Kate Perez|BIN Taxonomy Match|BOLD:AAI6451
Gymnometriocnemus subnudus|BIOUG05219-E05|Kate Perez|BIN Taxonomy Match|BOLD:AAI6451
Gymnometriocnemus subnudus|BIOUG05944-E11|Kate Perez|BIN Taxonomy Match|BOLD:AAI6451
Gymnometriocnemus subnudus|BIOUG05483-H03|Kate Perez|BIN Taxonomy Match|BOLD:AAI6451
Gymnometriocnemus subnudus|BIOUG05219-C08|Kate Perez|BIN Taxonomy Match|BOLD:AAI6451
Gymnometriocnemus subnudus|BIOUG05216-D09|Kate Perez|BIN Taxonomy Match|BOLD:AAI6451
Gymnometriocnemus subnudus|BIOUG05217-A04|Kate Perez|BIN Taxonomy Match|BOLD:AAI6451
Gymnometriocnemus subnudus|BIOUG05217-C11|Kate Perez|BIN Taxonomy Match|BOLD:AAI6451
Gymnometriocnemus subnudus|BIOUG05219-F05|Kate Perez|BIN Taxonomy Match|BOLD:AAI6451
Gymnometriocnemus subnudus|BIOUG05219-C06|Kate Perez|BIN Taxonomy Match|BOLD:AAI6451
Gymnometriocnemus subnudus|BIOUG05219-H01|Kate Perez|BIN Taxonomy Match|BOLD:AAI6451
Gymnometriocnemus subnudus|BIOUG06056-E05|Kate Perez|BIN Taxonomy Match|BOLD:AAI6451
Gymnometriocnemus subnudus|BIOUG06053-G02|Kate Perez|BIN Taxonomy Match|BOLD:AAI6451
Gymnometriocnemus subnudus|BIOUG05219-B07|Kate Perez|BIN Taxonomy Match|BOLD:AAI6451
Gymnometriocnemus subnudus|BIOUG05215-B08|Kate Perez|BIN Taxonomy Match|BOLD:AAI6451
Gymnometriocnemus subnudus|BIOUG05395-G07|Kate Perez|BIN Taxonomy Match|BOLD:AAI6451
Gymnometriocnemus subnudus|BIOUG05945-F01|Kate Perez|BIN Taxonomy Match|BOLD:AAI6451
Gymnometriocnemus subnudus|BIOUG06051-D10|Kate Perez|BIN Taxonomy Match|BOLD:AAI6451
Gymnometriocnemus subnudus|BIOUG06062-F03|Kate Perez|BIN Taxonomy Match|BOLD:AAI6451
Gymnometriocnemus subnudus|BIOUG05149-G05|Kate Perez|BIN Taxonomy Match|BOLD:AAI6451
Gymnometriocnemus subnudus|BIOUG05149-G12|Kate Perez|BIN Taxonomy Match|BOLD:AAI6451
Gymnometriocnemus subnudus|BIOUG05149-C11|Kate Perez|BIN Taxonomy Match|BOLD:AAI6451
Gymnometriocnemus subnudus|BIOUG05396-H01|Kate Perez|BIN Taxonomy Match|BOLD:AAI6451
Gymnometriocnemus subnudus|BIOUG05399-D07|Kate Perez|BIN Taxonomy Match|BOLD:AAI6451
Gymnometriocnemus subnudus|BIOUG06055-E03|Kate Perez|BIN Taxonomy Match|BOLD:AAI6451
Gymnometriocnemus subnudus|BIOUG05484-F12|Kate Perez|BIN Taxonomy Match|BOLD:AAI6451
Gymnometriocnemus subnudus|BIOUG05777-E12|Kate Perez|BIN Taxonomy Match|BOLD:AAI6451
Gymnometriocnemus subnudus|BIOUG05778-H07|Kate Perez|BIN Taxonomy Match|BOLD:AAI6451
Gymnometriocnemus subnudus|BIOUG05486-B12|Kate Perez|BIN Taxonomy Match|BOLD:AAI6451
Gymnometriocnemus subnudus|BIOUG05267-A10|Kate Perez|BIN Taxonomy Match|BOLD:AAI6451
Gymnometriocnemus subnudus|BIOUG05486-F01|Kate Perez|BIN Taxonomy Match|BOLD:AAI6451
Gymnometriocnemus subnudus|BIOUG05267-F05|Kate Perez|BIN Taxonomy Match|BOLD:AAI6451
Gymnometriocnemus subnudus|BIOUG05267-E04|Kate Perez|BIN Taxonomy Match|BOLD:AAI6451
Gymnometriocnemus subnudus|BIOUG05267-C02|Kate Perez|BIN Taxonomy Match|BOLD:AAI6451
Gymnometriocnemus subnudus|BIOUG05777-D12|Kate Perez|BIN Taxonomy Match|BOLD:AAI6451
Gymnometriocnemus subnudus|BIOUG05396-E07|Kate Perez|BIN Taxonomy Match|BOLD:AAI6451
Gymnometriocnemus subnudus|BIOUG05220-D03|Kate Perez|BIN Taxonomy Match|BOLD:AAI6451
Gymnometriocnemus subnudus|ATNA280|Elisabeth Stur||BOLD:AAI6451

Gymnometriocnemus subnudus|BIOUG05396-E07|Kate Perez|BIN Taxonomy Match|BOLD:AAI6451
Gymnometriocnemus subnudus|BIOUG05220-D03|Kate Perez|BIN Taxonomy Match|BOLD:AAI6451
Gymnometriocnemus subnudus|ATNA280|Elisabeth Stur||BOLD:AAI6451
Gymnometriocnemus subnudus|BIOUG05947-G11|Kate Perez|BIN Taxonomy Match|BOLD:AAI6451
Gymnometriocnemus subnudus|BIOUG05219-A07|Kate Perez|BIN Taxonomy Match|BOLD:AAI6451
Gymnometriocnemus subnudus|BIOUG05141-F03|Kate Perez|BIN Taxonomy Match|BOLD:AAI6451
Gymnometriocnemus subnudus|BIOUG05142-C10|Kate Perez|BIN Taxonomy Match|BOLD:AAI6451
Gymnometriocnemus subnudus|BIOUG05219-F08|Kate Perez|BIN Taxonomy Match|BOLD:AAI6451
Gymnometriocnemus subnudus|BIOUG05219-H03|Kate Perez|BIN Taxonomy Match|BOLD:AAI6451
Gymnometriocnemus subnudus|BIOUG06062-H06|Kate Perez|BIN Taxonomy Match|BOLD:AAI6451
Gymnometriocnemus subnudus|BIOUG05108-H06|Kate Perez|BIN Taxonomy Match|BOLD:AAI6451
Gymnometriocnemus subnudus|BIOUG05149-G04|Kate Perez|BIN Taxonomy Match|BOLD:AAI6451
Gymnometriocnemus subnudus|BIOUG05217-G07|Kate Perez|BIN Taxonomy Match|BOLD:AAI6451
Gymnometriocnemus subnudus|BIOUG05215-E09|Kate Perez|BIN Taxonomy Match|BOLD:AAI6451
Gymnometriocnemus subnudus|BIOUG05216-C05|Kate Perez|BIN Taxonomy Match|BOLD:AAI6451
Gymnometriocnemus subnudus|BIOUG05216-D03|Kate Perez|BIN Taxonomy Match|BOLD:AAI6451
Gymnometriocnemus subnudus|BIOUG05216-E12|Kate Perez|BIN Taxonomy Match|BOLD:AAI6451
Gymnometriocnemus subnudus|BIOUG05216-H07|Kate Perez|BIN Taxonomy Match|BOLD:AAI6451
Gymnometriocnemus subnudus|BIOUG05218-D06|Kate Perez|BIN Taxonomy Match|BOLD:AAI6451
Gymnometriocnemus subnudus|BIOUG06053-G07|Kate Perez|BIN Taxonomy Match|BOLD:AAI6451
Gymnometriocnemus subnudus|BIOUG06062-B08|Kate Perez|BIN Taxonomy Match|BOLD:AAI6451
Gymnometriocnemus subnudus|BIOUG06055-F02|Kate Perez|BIN Taxonomy Match|BOLD:AAI6451
Gymnometriocnemus subnudus|BIOUG05485-G12|Kate Perez|BIN Taxonomy Match|BOLD:AAI6451
Gymnometriocnemus subnudus|BIOUG05218-F12|Kate Perez|BIN Taxonomy Match|BOLD:AAI6451
Gymnometriocnemus subnudus|BIOUG05219-C03|Kate Perez|BIN Taxonomy Match|BOLD:AAI6451
Gymnometriocnemus subnudus|BIOUG05215-F06|Kate Perez|BIN Taxonomy Match|BOLD:AAI6451
Gymnometriocnemus subnudus|BIOUG06051-F04|Kate Perez|BIN Taxonomy Match|BOLD:AAI6451
Gymnometriocnemus subnudus|BIOUG05396-H10|Kate Perez|BIN Taxonomy Match|BOLD:AAI6451
Gymnometriocnemus subnudus|BIOUG05486-A01|Kate Perez|BIN Taxonomy Match|BOLD:AAI6451
Gymnometriocnemus subnudus|BIOUG05215-E11|Kate Perez|BIN Taxonomy Match|BOLD:AAI6451
Gymnometriocnemus subnudus|BIOUG05778-H01|Kate Perez|BIN Taxonomy Match|BOLD:AAI6451
Gymnometriocnemus subnudus|BIOUG05778-A01|Kate Perez|BIN Taxonomy Match|BOLD:AAI6451
Gymnometriocnemus subnudus|BIOUG06056-H09|Kate Perez|BIN Taxonomy Match|BOLD:AAI6451
Gymnometriocnemus subnudus|BIOUG06052-A02|Kate Perez|BIN Taxonomy Match|BOLD:AAI6451
Gymnometriocnemus subnudus|BIOUG05947-F07|Kate Perez|BIN Taxonomy Match|BOLD:AAI6451
Gymnometriocnemus subnudus|BIOUG05947-A02|Kate Perez|BIN Taxonomy Match|BOLD:AAI6451
Tavastia alticrista|CCDB21606-H02|Sofia Wiedenbrug||BOLD:ACT6409
Tavastia alticrista|CCDB24183-D12|Sofia Wiedenbrug|Morphology|BOLD:ACT6409
Tavastia alticrista|CCDB24183-D07|Sofia Wiedenbrug|Bin-match|BOLD:ACT6409
Tavastia|SOE323|Elisabeth Stur||BOLD:AAH2947
Tavastia|BIOUG15562-E12|Kate Perez|BIN Taxonomy Match|BOLD:AAH2947
Tavastia|SOE325|Elisabeth Stur||BOLD:AAH2947
Tavastia|BIOUG05144-E03|Kate Perez|BIN Taxonomy Match|BOLD:AAH2947
Tavastia|BIOUG05141-A08|Kate Perez|BIN Taxonomy Match|BOLD:AAH2947
Tavastia|BIOUG05149-A01|Kate Perez|BIN Taxonomy Match|BOLD:AAH2947
Tavastia|BIOUG05149-A02|Kate Perez|BIN Taxonomy Match|BOLD:AAH2947
Tavastia|BIOUG05149-A04|Kate Perez|BIN Taxonomy Match|BOLD:AAH2947
Tavastia|BIOUG15464-D05|Kate Perez|BIN Taxonomy Match|BOLD:AAH2947
Tavastia|BIOUG15459-B07|Kate Perez|BIN Taxonomy Match|BOLD:AAH2947
Tavastia|BIOUG15545-G12|Kate Perez|BIN Taxonomy Match|BOLD:AAH2947
Tavastia|BIOUG15545-D07|Kate Perez|BIN Taxonomy Match|BOLD:AAH2947
Tavastia|BIOUG15541-D04|Kate Perez|BIN Taxonomy Match|BOLD:AAH2947
Tavastia|BIOUG05102-F01|Kate Perez|BIN Taxonomy Match|BOLD:AAH2947
Chironomidae|BIOUG07809-G07|Meredith Miller|Tree-based Identification (Feb 2019)
Gymnometriocnemus pallidus|BIOUG07806-G07|Kate Perez|BIN Taxonomy Match|BOLD:AAG6457
Gymnometriocnemus pallidus|BIOUG07808-B03|Kate Perez|BIN Taxonomy Match|BOLD:AAG6457
Gymnometriocnemus pallidus|BIOUG06209-F12|Kate Perez|BIN Taxonomy Match|BOLD:AAG6457
Gymnometriocnemus pallidus|BIOUG05945-H09|Kate Perez|BIN Taxonomy Match|BOLD:AAG6457
Gymnometriocnemus pallidus|BIOUG06096-E08|Kate Perez|BIN Taxonomy Match|BOLD:AAG6457
Gymnometriocnemus pallidus|BIOUG06053-A07|Kate Perez|BIN Taxonomy Match|BOLD:AAG6457
Gymnometriocnemus pallidus|BIOUG06058-G04|Kate Perez|BIN Taxonomy Match|BOLD:AAG6457
Gymnometriocnemus pallidus|BIOUG05945-D09|Kate Perez|BIN Taxonomy Match|BOLD:AAG6457
Gymnometriocnemus pallidus|BIOUG06275-G05|Kate Perez|BIN Taxonomy Match|BOLD:AAG6457
Gymnometriocnemus pallidus|BIOUG05396-D01|Kate Perez|BIN Taxonomy Match|BOLD:AAG6457
Gymnometriocnemus pallidus|BIOUG07568-B08|Kate Perez|BIN Taxonomy Match|BOLD:AAG6457
Gymnometriocnemus pallidus|BIOUG06093-E07|Kate Perez|BIN Taxonomy Match|BOLD:AAG6457
Gymnometriocnemus pallidus|BIOUG06087-C08|Kate Perez|BIN Taxonomy Match|BOLD:AAG6457
Gymnometriocnemus pallidus|CH-OSF167|Elisabeth Stur|Morphology|BOLD:AAG6457
Gymnometriocnemus pallidus|CH-OSF168|Elisabeth Stur|Morphology|BOLD:AAG6457
Gymnometriocnemus pallidus|CH-eik47|Elisabeth Stur|Morphology|BOLD:AAG6457
Gymnometriocnemus pallidus|CH-eik63|Elisabeth Stur|Morphology|BOLD:AAG6457
Gymnometriocnemus pallidus|CH-eik64|Elisabeth Stur|Morphology|BOLD:AAG6457
Chironomidae|DIPNO-0041|||BOLD:AAG6457
Gymnometriocnemus pallidus|CH-eik131|Elisabeth Stur|Morphology|BOLD:AAG6457
Gymnometriocnemus pallidus|BIOUG07413-D01|Kate Perez|BIN Taxonomy Match|BOLD:AAG6457
Gymnometriocnemus pallidus|BIOUG05780-G08|Kate Perez|BIN Taxonomy Match|BOLD:AAG6457
Gymnometriocnemus pallidus|BIOUG06100-E06|Kate Perez|BIN Taxonomy Match|BOLD:AAG6457
Gymnometriocnemus pallidus|BIOUG06056-B11|Kate Perez|BIN Taxonomy Match|BOLD:AAG6457
Gymnometriocnemus pallidus|BIOUG06056-G01|Kate Perez|BIN Taxonomy Match|BOLD:AAG6457
Gymnometriocnemus pallidus|BIOUG06095-A12|Kate Perez|BIN Taxonomy Match|BOLD:AAG6457
Gymnometriocnemus pallidus|BIOUG06053-H07|Kate Perez|BIN Taxonomy Match|BOLD:AAG6457
Gymnometriocnemus pallidus|BIOUG07503-D11|Kate Perez|BIN Taxonomy Match|BOLD:AAG6457
Gymnometriocnemus pallidus|BIOUG07415-B01|Kate Perez|BIN Taxonomy Match|BOLD:AAG6457
Gymnometriocnemus pallidus|BIOUG07415-G11|Kate Perez|BIN Taxonomy Match|BOLD:AAG6457
Gymnometriocnemus pallidus|BIOUG06090-D01|Kate Perez|BIN Taxonomy Match|BOLD:AAG6457
Gymnometriocnemus pallidus|BIOUG06062-G10|Kate Perez|BIN Taxonomy Match|BOLD:AAG6457
Gymnometriocnemus pallidus|BIOUG06086-G05|Kate Perez|BIN Taxonomy Match|BOLD:AAG6457
Gymnometriocnemus pallidus|BIOUG06086-G01|Kate Perez|BIN Taxonomy Match|BOLD:AAG6457
Gymnometriocnemus pallidus|BIOUG06065-B04|Kate Perez|BIN Taxonomy Match|BOLD:AAG6457
Gymnometriocnemus pallidus|BIOUG05900-C03|Kate Perez|BIN Taxonomy Match|BOLD:AAG6457
Gymnometriocnemus pallidus|BIOUG06062-D07|Kate Perez|BIN Taxonomy Match|BOLD:AAG6457
Gymnometriocnemus pallidus|BIOUG06054-H11|Kate Perez|BIN Taxonomy Match|BOLD:AAG6457
Gymnometriocnemus pallidus|BIOUG06054-A09|Kate Perez|BIN Taxonomy Match|BOLD:AAG6457
Gymnometriocnemus pallidus|BIOUG06053-F02|Kate Perez|BIN Taxonomy Match|BOLD:AAG6457
Gymnometriocnemus pallidus|BIOUG06053-D07|Kate Perez|BIN Taxonomy Match|BOLD:AAG6457
Gymnometriocnemus pallidus|BIOUG05947-D06|Kate Perez|BIN Taxonomy Match|BOLD:AAG6457
Gymnometriocnemus pallidus|BIOUG06208-G04|Kate Perez|BIN Taxonomy Match|BOLD:AAG6457
Gymnometriocnemus pallidus|BIOUG06208-F08|Kate Perez|BIN Taxonomy Match|BOLD:AAG6457
Gymnometriocnemus pallidus|BIOUG06208-E10|Kate Perez|BIN Taxonomy Match|BOLD:AAG6457
Gymnometriocnemus pallidus|BIOUG06208-E03|Kate Perez|BIN Taxonomy Match|BOLD:AAG6457
Gymnometriocnemus pallidus|BIOUG06094-H01|Kate Perez|BIN Taxonomy Match|BOLD:AAG6457
Gymnometriocnemus pallidus|BIOUG06090-F11|Kate Perez|BIN Taxonomy Match|BOLD:AAG6457
Gymnometriocnemus pallidus|BIOUG06208-F01|Kate Perez|BIN Taxonomy Match|BOLD:AAG6457

Gymnometriocnemus pallidus|BIOUG06208-E03|Kate Perez|BIN Taxonomy Match|BOLD:AAG6457
Gymnometriocnemus pallidus|BIOUG06094-H01|Kate Perez|BIN Taxonomy Match|BOLD:AAG6457
Gymnometriocnemus pallidus|BIOUG06090-F11|Kate Perez|BIN Taxonomy Match|BOLD:AAG6457
Gymnometriocnemus pallidus|BIOUG06208-E01|Kate Perez|BIN Taxonomy Match|BOLD:AAG6457
Gymnometriocnemus pallidus|BIOUG06100-B11|Kate Perez|BIN Taxonomy Match|BOLD:AAG6457
Gymnometriocnemus pallidus|BIOUG06096-F11|Kate Perez|BIN Taxonomy Match|BOLD:AAG6457
Gymnometriocnemus pallidus|BIOUG06095-E06|Kate Perez|BIN Taxonomy Match|BOLD:AAG6457
Gymnometriocnemus pallidus|BIOUG06054-B09|Kate Perez|BIN Taxonomy Match|BOLD:AAG6457
Gymnometriocnemus pallidus|BIOUG07805-B12|Kate Perez|BIN Taxonomy Match|BOLD:AAG6457
Gymnometriocnemus pallidus|BIOUG07805-D09|Kate Perez|BIN Taxonomy Match|BOLD:AAG6457
Gymnometriocnemus pallidus|BIOUG06055-E04|Kate Perez|BIN Taxonomy Match|BOLD:AAG6457
Gymnometriocnemus pallidus|BIOUG06088-B07|Kate Perez|BIN Taxonomy Match|BOLD:AAG6457
Gymnometriocnemus pallidus|BIOUG06058-F01|Kate Perez|BIN Taxonomy Match|BOLD:AAG6457
Gymnometriocnemus pallidus|BIOUG06049-F10|Kate Perez|BIN Taxonomy Match|BOLD:AAG6457
Gymnometriocnemus pallidus|BIOUG05945-D10|Kate Perez|BIN Taxonomy Match|BOLD:AAG6457
Gymnometriocnemus pallidus|BIOUG05945-F10|Kate Perez|BIN Taxonomy Match|BOLD:AAG6457
Gymnometriocnemus pallidus|BIOUG07415-H08|Kate Perez|BIN Taxonomy Match|BOLD:AAG6457
Gymnometriocnemus pallidus|BIOUG06054-C10|Kate Perez|BIN Taxonomy Match|BOLD:AAG6457
Gymnometriocnemus pallidus|BIOUG06093-D12|Kate Perez|BIN Taxonomy Match|BOLD:AAG6457
Gymnometriocnemus pallidus|BIOUG05780-H01|Kate Perez|BIN Taxonomy Match|BOLD:AAG6457
Gymnometriocnemus pallidus|BIOUG05944-C11|Kate Perez|BIN Taxonomy Match|BOLD:AAG6457
Gymnometriocnemus pallidus|BIOUG06055-E11|Kate Perez|BIN Taxonomy Match|BOLD:AAG6457
Gymnometriocnemus pallidus|BIOUG06053-H03|Kate Perez|BIN Taxonomy Match|BOLD:AAG6457
Gymnometriocnemus pallidus|BIOUG05900-F10|Kate Perez|BIN Taxonomy Match|BOLD:AAG6457
Gymnometriocnemus pallidus|BIOUG06065-C02|Kate Perez|BIN Taxonomy Match|BOLD:AAG6457
Gymnometriocnemus pallidus|BIOUG06087-E11|Kate Perez|BIN Taxonomy Match|BOLD:AAG6457
Gymnometriocnemus pallidus|BIOUG06090-C10|Kate Perez|BIN Taxonomy Match|BOLD:AAG6457
Gymnometriocnemus pallidus|BIOUG06092-B03|Kate Perez|BIN Taxonomy Match|BOLD:AAG6457
Gymnometriocnemus pallidus|BIOUG06092-B06|Kate Perez|BIN Taxonomy Match|BOLD:AAG6457
Gymnometriocnemus pallidus|BIOUG06092-D06|Kate Perez|BIN Taxonomy Match|BOLD:AAG6457
Gymnometriocnemus pallidus|BIOUG06092-F04|Kate Perez|BIN Taxonomy Match|BOLD:AAG6457
Gymnometriocnemus pallidus|BIOUG06054-D09|Kate Perez|BIN Taxonomy Match|BOLD:AAG6457
Gymnometriocnemus pallidus|BIOUG05459-F09|Kate Perez|BIN Taxonomy Match|BOLD:AAG6457
Gymnometriocnemus pallidus|BIOUG06056-B10|Kate Perez|BIN Taxonomy Match|BOLD:AAG6457
Gymnometriocnemus pallidus|BIOUG06053-C10|Kate Perez|BIN Taxonomy Match|BOLD:AAG6457
Gymnometriocnemus pallidus|BIOUG06058-F05|Kate Perez|BIN Taxonomy Match|BOLD:AAG6457
Gymnometriocnemus pallidus|BIOUG06053-B03|Kate Perez|BIN Taxonomy Match|BOLD:AAG6457
Gymnometriocnemus pallidus|BIOUG06054-F07|Kate Perez|BIN Taxonomy Match|BOLD:AAG6457
Gymnometriocnemus pallidus|BIOUG06049-F06|Kate Perez|BIN Taxonomy Match|BOLD:AAG6457
Gymnometriocnemus pallidus|BIOUG06053-F08|Kate Perez|BIN Taxonomy Match|BOLD:AAG6457
Gymnometriocnemus pallidus|BIOUG06054-B01|Kate Perez|BIN Taxonomy Match|BOLD:AAG6457
Gymnometriocnemus pallidus|BIOUG05780-F10|Kate Perez|BIN Taxonomy Match|BOLD:AAG6457
Gymnometriocnemus pallidus|BIOUG05944-C09|Kate Perez|BIN Taxonomy Match|BOLD:AAG6457
Gymnometriocnemus pallidus|BIOUG06051-G10|Kate Perez|BIN Taxonomy Match|BOLD:AAG6457
Gymnometriocnemus pallidus|BIOUG06051-G08|Kate Perez|BIN Taxonomy Match|BOLD:AAG6457
Gymnometriocnemus pallidus|BIOUG06056-G05|Kate Perez|BIN Taxonomy Match|BOLD:AAG6457
Gymnometriocnemus pallidus|BIOUG06051-G12|Kate Perez|BIN Taxonomy Match|BOLD:AAG6457
Gymnometriocnemus pallidus|BIOUG06050-G12|Kate Perez|BIN Taxonomy Match|BOLD:AAG6457
Gymnometriocnemus pallidus|BIOUG06050-B05|Kate Perez|BIN Taxonomy Match|BOLD:AAG6457
Gymnometriocnemus pallidus|BIOUG06051-F05|Kate Perez|BIN Taxonomy Match|BOLD:AAG6457
Gymnometriocnemus pallidus|BIOUG06051-C12|Kate Perez|BIN Taxonomy Match|BOLD:AAG6457
Gymnometriocnemus pallidus|BIOUG06052-F07|Kate Perez|BIN Taxonomy Match|BOLD:AAG6457
Gymnometriocnemus pallidus|BIOUG06052-E11|Kate Perez|BIN Taxonomy Match|BOLD:AAG6457
Gymnometriocnemus pallidus|BIOUG05900-D08|Kate Perez|BIN Taxonomy Match|BOLD:AAG6457
Gymnometriocnemus pallidus|BIOUG06053-H04|Kate Perez|BIN Taxonomy Match|BOLD:AAG6457
Gymnometriocnemus pallidus|BIOUG05900-H01|Kate Perez|BIN Taxonomy Match|BOLD:AAG6457
Gymnometriocnemus pallidus|BIOUG06093-G10|Kate Perez|BIN Taxonomy Match|BOLD:AAG6457
Gymnometriocnemus pallidus|BIOUG06094-C05|Kate Perez|BIN Taxonomy Match|BOLD:AAG6457
Gymnometriocnemus pallidus|BIOUG06052-C01|Kate Perez|BIN Taxonomy Match|BOLD:AAG6457
Gymnometriocnemus pallidus|BIOUG06053-F07|Kate Perez|BIN Taxonomy Match|BOLD:AAG6457
Gymnometriocnemus pallidus|BIOUG05780-G10|Kate Perez|BIN Taxonomy Match|BOLD:AAG6457
Gymnometriocnemus pallidus|BIOUG05780-H07|Kate Perez|BIN Taxonomy Match|BOLD:AAG6457
Gymnometriocnemus pallidus|BIOUG05944-B08|Kate Perez|BIN Taxonomy Match|BOLD:AAG6457
Gymnometriocnemus pallidus|BIOUG05946-G04|Kate Perez|BIN Taxonomy Match|BOLD:AAG6457
Gymnometriocnemus pallidus|BIOUG06056-H10|Kate Perez|BIN Taxonomy Match|BOLD:AAG6457
Gymnometriocnemus pallidus|BIOUG07408-D06|Kate Perez|BIN Taxonomy Match|BOLD:AAG6457
Gymnometriocnemus pallidus|BIOUG05946-D08|Kate Perez|BIN Taxonomy Match|BOLD:AAG6457
Gymnometriocnemus pallidus|BIOUG05900-A12|Kate Perez|BIN Taxonomy Match|BOLD:AAG6457
Gymnometriocnemus pallidus|BIOUG05780-C08|Kate Perez|BIN Taxonomy Match|BOLD:AAG6457
Gymnometriocnemus pallidus|BIOUG07568-G12|Kate Perez|BIN Taxonomy Match|BOLD:AAG6457
Gymnometriocnemus pallidus|BIOUG06096-E05|Kate Perez|BIN Taxonomy Match|BOLD:AAG6457
Gymnometriocnemus pallidus|BIOUG06088-F12|Kate Perez|BIN Taxonomy Match|BOLD:AAG6457
Gymnometriocnemus pallidus|BIOUG08094-H06|Kate Perez|BIN Taxonomy Match|BOLD:AAG6457
Gymnometriocnemus pallidus|BIOUG06450-F12|Kate Perez|BIN Taxonomy Match|BOLD:AAG6457
Gymnometriocnemus pallidus|BIOUG05780-D08|Kate Perez|BIN Taxonomy Match|BOLD:AAG6457
Gymnometriocnemus pallidus|BIOUG07753-F08|Kate Perez|BIN Taxonomy Match|BOLD:AAG6457
Gymnometriocnemus pallidus|BIOUG05780-G01|Kate Perez|BIN Taxonomy Match|BOLD:AAG6457
Gymnometriocnemus pallidus|BIOUG05944-F04|Kate Perez|BIN Taxonomy Match|BOLD:AAG6457
Gymnometriocnemus pallidus|BIOUG05945-E08|Kate Perez|BIN Taxonomy Match|BOLD:AAG6457
Gymnometriocnemus pallidus|BIOUG07930-F11|Kate Perez|BIN Taxonomy Match|BOLD:AAG6457
Gymnometriocnemus pallidus|BIOUG05778-A03|Kate Perez|BIN Taxonomy Match|BOLD:AAG6457
Gymnometriocnemus pallidus|BIOUG05778-B02|Kate Perez|BIN Taxonomy Match|BOLD:AAG6457
Gymnometriocnemus pallidus|BIOUG05778-D01|Kate Perez|BIN Taxonomy Match|BOLD:AAG6457
Gymnometriocnemus pallidus|BIOUG07944-D11|Kate Perez|BIN Taxonomy Match|BOLD:AAG6457
Gymnometriocnemus pallidus|BIOUG05778-F01|Kate Perez|BIN Taxonomy Match|BOLD:AAG6457
Gymnometriocnemus pallidus|BIOUG07753-E03|Kate Perez|BIN Taxonomy Match|BOLD:AAG6457
Gymnometriocnemus pallidus|BIOUG07757-B02|Kate Perez|BIN Taxonomy Match|BOLD:AAG6457
Gymnometriocnemus pallidus|BIOUG06441-E09|Kate Perez|BIN Taxonomy Match|BOLD:AAG6457
Gymnometriocnemus pallidus|BIOUG07410-E10|Kate Perez|BIN Taxonomy Match|BOLD:AAG6457
Gymnometriocnemus pallidus|BIOUG05897-B01|Kate Perez|BIN Taxonomy Match|BOLD:AAG6457
Gymnometriocnemus pallidus|BIOUG07758-H07|Kate Perez|BIN Taxonomy Match|BOLD:AAG6457
Gymnometriocnemus pallidus|BIOUG07758-C05|Kate Perez|BIN Taxonomy Match|BOLD:AAG6457
Gymnometriocnemus pallidus|BIOUG07757-E05|Kate Perez|BIN Taxonomy Match|BOLD:AAG6457
Gymnometriocnemus pallidus|BIOUG07760-H03|Kate Perez|BIN Taxonomy Match|BOLD:AAG6457
Gymnometriocnemus pallidus|BIOUG07755-B07|Kate Perez|BIN Taxonomy Match|BOLD:AAG6457
Gymnometriocnemus pallidus|BIOUG07753-B08|Kate Perez|BIN Taxonomy Match|BOLD:AAG6457
Gymnometriocnemus pallidus|BIOUG07753-F06|Kate Perez|BIN Taxonomy Match|BOLD:AAG6457
Gymnometriocnemus pallidus|BIOUG08135-D10|Kate Perez|BIN Taxonomy Match|BOLD:AAG6457
Gymnometriocnemus pallidus|BIOUG08135-C08|Kate Perez|BIN Taxonomy Match|BOLD:AAG6457
Gymnometriocnemus pallidus|BIOUG05778-G10|Kate Perez|BIN Taxonomy Match|BOLD:AAG6457
Gymnometriocnemus pallidus|BIOUG05778-G07|Kate Perez|BIN Taxonomy Match|BOLD:AAG6457
Gymnometriocnemus pallidus|BIOUG05778-E01|Kate Perez|BIN Taxonomy Match|BOLD:AAG6457
Gymnometriocnemus pallidus|BIOUG05777-G07|Kate Perez|BIN Taxonomy Match|BOLD:AAG6457
[illegible]

Gymnometriocnemus pallidus|BIOUG05778-G07|Kate Perez|BIN Taxonomy Match|BOLD:AAG6457
Gymnometriocnemus pallidus|BIOUG05778-E01|Kate Perez|BIN Taxonomy Match|BOLD:AAG6457
Gymnometriocnemus pallidus|BIOUG05777-G07|Kate Perez|BIN Taxonomy Match|BOLD:AAG6457
Gymnometriocnemus pallidus|BIOUG05777-B06|Kate Perez|BIN Taxonomy Match|BOLD:AAG6457
Gymnometriocnemus pallidus|BIOUG05777-B02|Kate Perez|BIN Taxonomy Match|BOLD:AAG6457
Gymnometriocnemus pallidus|BIOUG05776-F11|Kate Perez|BIN Taxonomy Match|BOLD:AAG6457
Gymnometriocnemus pallidus|BIOUG05776-F08|Kate Perez|BIN Taxonomy Match|BOLD:AAG6457
Gymnometriocnemus pallidus|BIOUG07810-H10|Kate Perez|BIN Taxonomy Match|BOLD:AAG6457
Gymnometriocnemus pallidus|BIOUG05776-B01|Kate Perez|BIN Taxonomy Match|BOLD:AAG6457
Gymnometriocnemus pallidus|BIOUG07756-G09|Kate Perez|BIN Taxonomy Match|BOLD:AAG6457
Gymnometriocnemus pallidus|BIOUG07759-A08|Kate Perez|BIN Taxonomy Match|BOLD:AAG6457
Gymnometriocnemus pallidus|BIOUG08174-E04|Kate Perez|BIN Taxonomy Match|BOLD:AAG6457
Gymnometriocnemus pallidus|BIOUG08133-E10|Kate Perez|BIN Taxonomy Match|BOLD:AAG6457
Gymnometriocnemus pallidus|BIOUG07945-A06|Kate Perez|BIN Taxonomy Match|BOLD:AAG6457
Gymnometriocnemus pallidus|BIOUG07944-C06|Kate Perez|BIN Taxonomy Match|BOLD:AAG6457
Gymnometriocnemus pallidus|BIOUG05486-C09|Kate Perez|BIN Taxonomy Match|BOLD:AAG6457
Gymnometriocnemus pallidus|BIOUG07413-E01|Kate Perez|BIN Taxonomy Match|BOLD:AAG6457
Gymnometriocnemus pallidus|BIOUG07760-A01|Kate Perez|BIN Taxonomy Match|BOLD:AAG6457
Gymnometriocnemus pallidus|BIOUG07647-A11|Kate Perez|BIN Taxonomy Match|BOLD:AAG6457
Gymnometriocnemus pallidus|BIOUG08098-G10|Kate Perez|BIN Taxonomy Match|BOLD:AAG6457
Gymnometriocnemus pallidus|BIOUG05777-A01|Kate Perez|BIN Taxonomy Match|BOLD:AAG6457
Gymnometriocnemus pallidus|BIOUG07759-H01|Kate Perez|BIN Taxonomy Match|BOLD:AAG6457
Gymnometriocnemus pallidus|BIOUG07760-B06|Kate Perez|BIN Taxonomy Match|BOLD:AAG6457
Gymnometriocnemus pallidus|BIOUG06518-A07|Kate Perez|BIN Taxonomy Match|BOLD:AAG6457
Gymnometriocnemus pallidus|BIOUG05898-H11|Kate Perez|BIN Taxonomy Match|BOLD:AAG6457
Gymnometriocnemus pallidus|BIOUG06442-B09|Kate Perez|BIN Taxonomy Match|BOLD:AAG6457
Gymnometriocnemus pallidus|BIOUG05776-B03|Kate Perez|BIN Taxonomy Match|BOLD:AAG6457
Gymnometriocnemus pallidus|BIOUG07509-D02|Kate Perez|BIN Taxonomy Match|BOLD:AAG6457
Gymnometriocnemus pallidus|BIOUG06450-H02|Kate Perez|BIN Taxonomy Match|BOLD:AAG6457
Gymnometriocnemus pallidus|BIOUG08209-H05|Kate Perez|BIN Taxonomy Match|BOLD:AAG6457
Gymnometriocnemus pallidus|BIOUG08134-C07|Kate Perez|BIN Taxonomy Match|BOLD:AAG6457
Gymnometriocnemus pallidus|BIOUG06446-F09|Kate Perez|BIN Taxonomy Match|BOLD:AAG6457
Gymnometriocnemus pallidus|BIOUG07759-E08|Kate Perez|BIN Taxonomy Match|BOLD:AAG6457
Gymnometriocnemus pallidus|BIOUG07929-G12|Kate Perez|BIN Taxonomy Match|BOLD:AAG6457
Gymnometriocnemus pallidus|BIOUG07755-H10|Kate Perez|BIN Taxonomy Match|BOLD:AAG6457
Gymnometriocnemus pallidus|BIOUG07929-H11|Kate Perez|BIN Taxonomy Match|BOLD:AAG6457
Gymnometriocnemus pallidus|BIOUG07930-D08|Kate Perez|BIN Taxonomy Match|BOLD:AAG6457
Gymnometriocnemus pallidus|BIOUG05486-E11|Kate Perez|BIN Taxonomy Match|BOLD:AAG6457
Gymnometriocnemus pallidus|BIOUG08180-F05|Kate Perez|BIN Taxonomy Match|BOLD:AAG6457
Gymnometriocnemus pallidus|BIOUG06450-A09|Kate Perez|BIN Taxonomy Match|BOLD:AAG6457
Gymnometriocnemus pallidus|BIOUG07754-G03|Kate Perez|BIN Taxonomy Match|BOLD:AAG6457
Gymnometriocnemus pallidus|BIOUG05778-G03|Kate Perez|BIN Taxonomy Match|BOLD:AAG6457
Gymnometriocnemus pallidus|BIOUG05776-E09|Kate Perez|BIN Taxonomy Match|BOLD:AAG6457
Gymnometriocnemus pallidus|BIOUG05776-D04|Kate Perez|BIN Taxonomy Match|BOLD:AAG6457
Gymnometriocnemus pallidus|BIOUG06065-E08|Kate Perez|BIN Taxonomy Match|BOLD:AAG6457
Gymnometriocnemus pallidus|BIOUG06051-F11|Kate Perez|BIN Taxonomy Match|BOLD:AAG6457
Gymnometriocnemus pallidus|BIOUG06100-A09|Kate Perez|BIN Taxonomy Match|BOLD:AAG6457
Gymnometriocnemus pallidus|BIOUG06055-H02|Kate Perez|BIN Taxonomy Match|BOLD:AAG6457
Gymnometriocnemus pallidus|BIOUG06055-E01|Kate Perez|BIN Taxonomy Match|BOLD:AAG6457
Gymnometriocnemus pallidus|BIOUG05947-G12|Kate Perez|BIN Taxonomy Match|BOLD:AAG6457
Gymnometriocnemus pallidus|BIOUG06047-D07|Kate Perez|BIN Taxonomy Match|BOLD:AAG6457
Gymnometriocnemus|BIOUG05105-E11|Kate Perez|BIN Taxonomy Match|BOLD:ACE0146
Gymnometriocnemus|BIOUG05103-E11|Kate Perez|BIN Taxonomy Match|BOLD:ACE0146
Gymnometriocnemus|BIOUG05142-E08|Kate Perez|BIN Taxonomy Match|BOLD:ACE0146
Gymnometriocnemus|BIOUG05141-H03|Kate Perez|BIN Taxonomy Match|BOLD:ACE0146
Gymnometriocnemus|BIOUG05103-E05|Kate Perez|BIN Taxonomy Match|BOLD:ACE0146
Gymnometriocnemus|BIOUG05141-A07|Kate Perez|BIN Taxonomy Match|BOLD:ACE0146
Gymnometriocnemus|BIOUG05144-H01|Kate Perez|BIN Taxonomy Match|BOLD:ACE0146
Gymnometriocnemus|BIOUG05109-E10|Kate Perez|BIN Taxonomy Match|BOLD:ACE0146
Gymnometriocnemus|BIOUG05141-B01|Kate Perez|BIN Taxonomy Match|BOLD:ACE0146
Gymnometriocnemus|BIOUG05110-F04|Kate Perez|BIN Taxonomy Match|BOLD:ACE0146
Gymnometriocnemus|BIOUG05105-A05|Kate Perez|BIN Taxonomy Match|BOLD:ACE0146
Gymnometriocnemus|BIOUG15561-E12|Kate Perez|BIN Taxonomy Match|BOLD:ACE0146
Gymnometriocnemus|BIOUG15539-C11|Kate Perez|BIN Taxonomy Match|BOLD:ACE0146
Gymnometriocnemus|BIOUG05144-B02|Kate Perez|BIN Taxonomy Match|BOLD:ACE0146
Gymnometriocnemus|BIOUG05144-B08|Kate Perez|BIN Taxonomy Match|BOLD:ACE0146
Gymnometriocnemus|BIOUG15654-C11|Kate Perez|BIN Taxonomy Match|BOLD:ACE0146
Gymnometriocnemus|BIOUG05101-C09|Kate Perez|BIN Taxonomy Match|BOLD:ACE0146
Gymnometriocnemus brevitarsis|BIOUG15595-G03|Elisabeth Stur|BIN Taxonomy Match|BOLD:AAI1980
Gymnometriocnemus brevitarsis|CH-OSF33|Elisabeth Stur|Morphology|BOLD:AAI1980
Gymnometriocnemus brevitarsis|BIOUG15456-C02|Elisabeth Stur|BIN Taxonomy Match|BOLD:AAI1980
Gymnometriocnemus brevitarsis|SOE128|Elisabeth Stur|Morphology|BOLD:AAI1980
Gymnometriocnemus brevitarsis|BIOUG15543-C12|Elisabeth Stur|BIN Taxonomy Match|BOLD:AAI1980
Gymnometriocnemus|24376-C11|Kate Perez|BIN Taxonomy Match|BOLD:AAE3567
Gymnometriocnemus|24374-E04|Kate Perez|BIN Taxonomy Match|BOLD:AAE3567
Gymnometriocnemus kamimegavirgus|BIOUG05109-D06|Kate Perez|BIN Taxonomy Match|BOLD:AAE3567
Gymnometriocnemus|24375-D06|Kate Perez|BIN Taxonomy Match|BOLD:AAE3567
Gymnometriocnemus|24374-C07|Kate Perez|BIN Taxonomy Match|BOLD:AAE3567
Gymnometriocnemus|24376-H01|Kate Perez|BIN Taxonomy Match|BOLD:AAE3567
Gymnometriocnemus kamimegavirgus|BIOUG05215-B10|Kate Perez|BIN Taxonomy Match|BOLD:AAE3567
Gymnometriocnemus kamimegavirgus|BIOUG05215-G09|Kate Perez|BIN Taxonomy Match|BOLD:AAE3567
Gymnometriocnemus kamimegavirgus|BIOUG05215-C08|Kate Perez|BIN Taxonomy Match|BOLD:AAE3567
Gymnometriocnemus kamimegavirgus|BIOUG05144-H07|Kate Perez|BIN Taxonomy Match|BOLD:AAE3567
Gymnometriocnemus kamimegavirgus|BIOUG05219-A12|Kate Perez|BIN Taxonomy Match|BOLD:AAE3567
Gymnometriocnemus kamimegavirgus|BIOUG05109-A04|Kate Perez|BIN Taxonomy Match|BOLD:AAE3567
Gymnometriocnemus volitans|BIOUG04269-C12|Kate Perez|BIN Taxonomy Match|BOLD:AAE3567
Gymnometriocnemus kamimegavirgus|BIOUG05141-H05|Kate Perez|BIN Taxonomy Match|BOLD:AAE3567
Gymnometriocnemus kamimegavirgus|BIOUG05103-E10|Kate Perez|BIN Taxonomy Match|BOLD:AAE3567
Gymnometriocnemus kamimegavirgus|BIOUG05101-E03|Kate Perez|BIN Taxonomy Match|BOLD:AAE3567
Gymnometriocnemus|24376-C03|Kate Perez|BIN Taxonomy Match|BOLD:AAE3567
Gymnometriocnemus|24376-E05|Kate Perez|BIN Taxonomy Match|BOLD:AAE3567
Gymnometriocnemus kamimegavirgus|BIOUG15545-A09|Elisabeth Stur|BIN Taxonomy Match|BOLD:AAE3567
Gymnometriocnemus kamimegavirgus|BIOUG15681-H10|Elisabeth Stur|BIN Taxonomy Match|BOLD:AAE3567
Gymnometriocnemus|24407-E09|Kate Perez|BIN Taxonomy Match|BOLD:AAE3567
Gymnometriocnemus|24407-G06|Kate Perez|BIN Taxonomy Match|BOLD:AAE3567
Gymnometriocnemus|24376-D04|Kate Perez|BIN Taxonomy Match|BOLD:AAE3567
Gymnometriocnemus|24407-G12|Kate Perez|BIN Taxonomy Match|BOLD:AAE3567
Gymnometriocnemus|24375-B12|Kate Perez|BIN Taxonomy Match|BOLD:AAE3567
Gymnometriocnemus kamimegavirgus|BIOUG15457-B06|Elisabeth Stur|BIN Taxonomy Match|BOLD:AAE3567
Gymnometriocnemus|24374-E05|Kate Perez|BIN Taxonomy Match|BOLD:AAE3567
Gymnometriocnemus|24375-A06|Kate Perez|BIN Taxonomy Match|BOLD:AAE3567
Gymnometriocnemus kamimegavirgus|BIOUG15456-G02|Elisabeth Stur|BIN Taxonomy Match|BOLD:AAE3567
Gymnometriocnemus|24374-D07|Kate Perez|BIN Taxonomy Match|BOLD:AAE3567

Gymnometriocnemus|24375-A06|Kate Perez|BIN Taxonomy Match|BOLD:AAE3567
Gymnometriocnemus kamimegavirgus|BIOUG15456-G02|Elisabeth Stur|BIN Taxonomy Match|BOLD:AAE3567
Gymnometriocnemus|24374-D07|Kate Perez|BIN Taxonomy Match|BOLD:AAE3567
Gymnometriocnemus kamimegavirgus|BIOUG15537-E01|Elisabeth Stur|BIN Taxonomy Match|BOLD:AAE3567
Gymnometriocnemus|24376-A09|Kate Perez|BIN Taxonomy Match|BOLD:AAE3567
Gymnometriocnemus volitans|BIOUG04261-D02|Kate Perez|BIN Taxonomy Match|BOLD:AAE3567
Gymnometriocnemus volitans|BIOUG04261-F10|Kate Perez|BIN Taxonomy Match|BOLD:AAE3567
Gymnometriocnemus|24376-F06|Kate Perez|BIN Taxonomy Match|BOLD:AAE3567
Gymnometriocnemus|24374-D11|Kate Perez|BIN Taxonomy Match|BOLD:AAE3567
Gymnometriocnemus|24407-E04|Kate Perez|BIN Taxonomy Match|BOLD:AAE3567
Gymnometriocnemus|24468-D09|Kate Perez|BIN Taxonomy Match|BOLD:AAE3567
Gymnometriocnemus|24537-C05|Kate Perez|BIN Taxonomy Match|BOLD:AAE3567
Gymnometriocnemus kamimegavirgus|BIOUG15565-D02|Elisabeth Stur|BIN Taxonomy Match|BOLD:AAE3567
Gymnometriocnemus kamimegavirgus|BIOUG15460-E07|Elisabeth Stur|BIN Taxonomy Match|BOLD:AAE3567
Gymnometriocnemus kamimegavirgus|BIOUG15455-B06|Elisabeth Stur|BIN Taxonomy Match|BOLD:AAE3567
Gymnometriocnemus|24376-E02|Kate Perez|BIN Taxonomy Match|BOLD:AAE3567
Gymnometriocnemus kamimegavirgus|BIOUG15460-B02|Elisabeth Stur|BIN Taxonomy Match|BOLD:AAE3567
Gymnometriocnemus kamimegavirgus|BIOUG15454-C03|Elisabeth Stur|BIN Taxonomy Match|BOLD:AAE3567
Gymnometriocnemus kamimegavirgus|BIOUG15543-E02|Elisabeth Stur|BIN Taxonomy Match|BOLD:AAE3567
Gymnometriocnemus kamimegavirgus|BIOUG15540-D02|Elisabeth Stur|BIN Taxonomy Match|BOLD:AAE3567
Gymnometriocnemus kamimegavirgus|BIOUG15538-G06|Elisabeth Stur|BIN Taxonomy Match|BOLD:AAE3567
Gymnometriocnemus kamimegavirgus|Finnmark04|Elisabeth Stur|Morphology|BOLD:AAE3567
Gymnometriocnemus|24374-F07|Kate Perez|BIN Taxonomy Match|BOLD:AAE3567
Gymnometriocnemus|24375-C09|Kate Perez|BIN Taxonomy Match|BOLD:AAE3567
Gymnometriocnemus|24374-D05|Kate Perez|BIN Taxonomy Match|BOLD:AAE3567
Gymnometriocnemus|24407-F02|Kate Perez|BIN Taxonomy Match|BOLD:AAE3567
Gymnometriocnemus|24407-G03|Kate Perez|BIN Taxonomy Match|BOLD:AAE3567
Gymnometriocnemus kamimegavirgus|BIOUG15538-G05|Elisabeth Stur|BIN Taxonomy Match|BOLD:AAE3567
Gymnometriocnemus|24374-A08|Kate Perez|BIN Taxonomy Match|BOLD:AAE3567
Gymnometriocnemus|24374-E03|Kate Perez|BIN Taxonomy Match|BOLD:AAE3567
Gymnometriocnemus|24374-C06|Kate Perez|BIN Taxonomy Match|BOLD:AAE3567
Gymnometriocnemus kamimegavirgus|ATNA203|Elisabeth Stur|Morphology|BOLD:AAE3567
Gymnometriocnemus kamimegavirgus|TRD-CH55|Elisabeth Stur|Morphology|BOLD:AAE3567
Gymnometriocnemus kamimegavirgus|Finnmark76|Elisabeth Stur|Morphology|BOLD:AAE3567
Gymnometriocnemus kamimegavirgus|NO30|Elisabeth Stur|Morphology|BOLD:AAE3567
Gymnometriocnemus|24375-H06|Kate Perez|BIN Taxonomy Match|BOLD:AAE3567
Gymnometriocnemus kamimegavirgus|BIOUG15563-F04|Elisabeth Stur|BIN Taxonomy Match|BOLD:AAE3567
Gymnometriocnemus|24376-A10|Kate Perez|BIN Taxonomy Match|BOLD:AAE3567
Gymnometriocnemus|24468-F06|Kate Perez|BIN Taxonomy Match|BOLD:AAE3567
Gymnometriocnemus|24375-B11|Kate Perez|BIN Taxonomy Match|BOLD:AAE3567
Gymnometriocnemus|24407-F08|Kate Perez|BIN Taxonomy Match|BOLD:AAE3567
Gymnometriocnemus|24374-A01|Kate Perez|BIN Taxonomy Match|BOLD:AAE3567
Gymnometriocnemus|24375-H04|Kate Perez|BIN Taxonomy Match|BOLD:AAE3567
Gymnometriocnemus|24376-D02|Kate Perez|BIN Taxonomy Match|BOLD:AAE3567
Gymnometriocnemus|24376-F04|Kate Perez|BIN Taxonomy Match|BOLD:AAE3567
Gymnometriocnemus|24374-C02|Kate Perez|BIN Taxonomy Match|BOLD:AAE3567
Gymnometriocnemus|24374-H09|Kate Perez|BIN Taxonomy Match|BOLD:AAE3567
Gymnometriocnemus kamimegavirgus|BIOUG15459-E10|Elisabeth Stur|BIN Taxonomy Match|BOLD:AAE3567
Gymnometriocnemus kamimegavirgus|BIOUG15454-B01|Elisabeth Stur|BIN Taxonomy Match|BOLD:AAE3567
Gymnometriocnemus kamimegavirgus|BIOUG15561-E03|Elisabeth Stur|BIN Taxonomy Match|BOLD:AAE3567
Gymnometriocnemus kamimegavirgus|BIOUG15201-H10|Elisabeth Stur|BIN Taxonomy Match|BOLD:AAE3567
Gymnometriocnemus kamimegavirgus|BIOUG15455-G03|Elisabeth Stur|BIN Taxonomy Match|BOLD:AAE3567
Gymnometriocnemus|24407-H06|Kate Perez|BIN Taxonomy Match|BOLD:AAE3567
Gymnometriocnemus|24375-B01|Kate Perez|BIN Taxonomy Match|BOLD:AAE3567
Gymnometriocnemus|24376-G02|Kate Perez|BIN Taxonomy Match|BOLD:AAE3567
Gymnometriocnemus kamimegavirgus|BIOUG07569-C01|Kate Perez|BIN Taxonomy Match|BOLD:AAE3567
Gymnometriocnemus|24374-G12|Kate Perez|BIN Taxonomy Match|BOLD:AAE3567
Gymnometriocnemus|24376-F09|Kate Perez|BIN Taxonomy Match|BOLD:AAE3567
Gymnometriocnemus kamimegavirgus|BIOUG05141-D07|Kate Perez|BIN Taxonomy Match|BOLD:AAE3567
Gymnometriocnemus kamimegavirgus|BIOUG15564-G02|Elisabeth Stur|BIN Taxonomy Match|BOLD:AAE3567
Gymnometriocnemus|24375-B06|Kate Perez|BIN Taxonomy Match|BOLD:AAE3567
Gymnometriocnemus kamimegavirgus|BIOUG15562-C08|Elisabeth Stur|BIN Taxonomy Match|BOLD:AAE3567
Gymnometriocnemus kamimegavirgus|BIOUG05215-C03|Kate Perez|BIN Taxonomy Match|BOLD:AAE3567
Gymnometriocnemus kamimegavirgus|BIOUG05219-A03|Kate Perez|BIN Taxonomy Match|BOLD:AAE3567
Gymnometriocnemus kamimegavirgus|BIOUG05219-B05|Kate Perez|BIN Taxonomy Match|BOLD:AAE3567
Gymnometriocnemus kamimegavirgus|BIOUG05144-B09|Kate Perez|BIN Taxonomy Match|BOLD:AAE3567
Gymnometriocnemus kamimegavirgus|BIOUG05215-D01|Kate Perez|BIN Taxonomy Match|BOLD:AAE3567
Gymnometriocnemus kamimegavirgus|BIOUG05217-A03|Kate Perez|BIN Taxonomy Match|BOLD:AAE3567
Gymnometriocnemus kamimegavirgus|BIOUG05217-A06|Kate Perez|BIN Taxonomy Match|BOLD:AAE3567
Gymnometriocnemus kamimegavirgus|BIOUG05217-D10|Kate Perez|BIN Taxonomy Match|BOLD:AAE3567
Gymnometriocnemus kamimegavirgus|BIOUG05109-A03|Kate Perez|BIN Taxonomy Match|BOLD:AAE3567
Gymnometriocnemus kamimegavirgus|BIOUG05109-B09|Kate Perez|BIN Taxonomy Match|BOLD:AAE3567
Gymnometriocnemus kamimegavirgus|BIOUG05109-D07|Kate Perez|BIN Taxonomy Match|BOLD:AAE3567
Gymnometriocnemus kamimegavirgus|BIOUG05141-C09|Kate Perez|BIN Taxonomy Match|BOLD:AAE3567
Gymnometriocnemus kamimegavirgus|BIOUG15458-D09|Elisabeth Stur|BIN Taxonomy Match|BOLD:AAE3567
Gymnometriocnemus kamimegavirgus|BIOUG05105-E12|Kate Perez|BIN Taxonomy Match|BOLD:AAE3567
Gymnometriocnemus kamimegavirgus|BIOUG15541-G03|Elisabeth Stur|BIN Taxonomy Match|BOLD:AAE3567
Gymnometriocnemus kamimegavirgus|BIOUG15565-A12|Elisabeth Stur|BIN Taxonomy Match|BOLD:AAE3567
Gymnometriocnemus kamimegavirgus|BIOUG15460-H04|Elisabeth Stur|BIN Taxonomy Match|BOLD:AAE3567
Gymnometriocnemus kamimegavirgus|BIOUG15459-H08|Elisabeth Stur|BIN Taxonomy Match|BOLD:AAE3567
Gymnometriocnemus|24407-D07|Kate Perez|BIN Taxonomy Match|BOLD:AAE3567
Gymnometriocnemus|24407-F07|Kate Perez|BIN Taxonomy Match|BOLD:AAE3567
Gymnometriocnemus|24374-A07|Kate Perez|BIN Taxonomy Match|BOLD:AAE3567
Gymnometriocnemus kamimegavirgus|BIOUG15539-G11|Elisabeth Stur|BIN Taxonomy Match|BOLD:AAE3567
Gymnometriocnemus|24407-G05|Kate Perez|BIN Taxonomy Match|BOLD:AAE3567
Gymnometriocnemus|24376-D08|Kate Perez|BIN Taxonomy Match|BOLD:AAE3567
Gymnometriocnemus kamimegavirgus|BIOUG15536-E03|Elisabeth Stur|BIN Taxonomy Match|BOLD:AAE3567
Gymnometriocnemus kamimegavirgus|BIOUG05101-D10|Kate Perez|BIN Taxonomy Match|BOLD:AAE3567
Gymnometriocnemus kamimegavirgus|BIOUG04795-F04|Kate Perez|BIN Taxonomy Match|BOLD:AAE3567
Gymnometriocnemus kamimegavirgus|BIOUG05220-B10|Kate Perez|BIN Taxonomy Match|BOLD:AAE3567
Gymnometriocnemus kamimegavirgus|BIOUG05267-D01|Kate Perez|BIN Taxonomy Match|BOLD:AAE3567
Gymnometriocnemus kamimegavirgus|BIOUG15457-C11|Elisabeth Stur|BIN Taxonomy Match|BOLD:AAE3567
Gymnometriocnemus|24374-C05|Kate Perez|BIN Taxonomy Match|BOLD:AAE3567
Gymnometriocnemus|24376-E11|Kate Perez|BIN Taxonomy Match|BOLD:AAE3567
Gymnometriocnemus kamimegavirgus|BIOUG05109-A06|Kate Perez|BIN Taxonomy Match|BOLD:AAE3567
Gymnometriocnemus kamimegavirgus|BIOUG05105-B06|Kate Perez|BIN Taxonomy Match|BOLD:AAE3567
Gymnometriocnemus kamimegavirgus|BIOUG05144-F11|Kate Perez|BIN Taxonomy Match|BOLD:AAE3567
Gymnometriocnemus kamimegavirgus|BIOUG15454-H05|Elisabeth Stur|BIN Taxonomy Match|BOLD:AAE3567
Gymnometriocnemus|24376-C06|Kate Perez|BIN Taxonomy Match|BOLD:AAE3567
Gymnometriocnemus|24378-B10|Kate Perez|BIN Taxonomy Match|BOLD:AAE3567
Gymnometriocnemus|24407-F05|Kate Perez|BIN Taxonomy Match|BOLD:AAE3567
Gymnometriocnemus kamimegavirgus|BIOUG15565-A10|Elisabeth Stur|BIN Taxonomy Match|BOLD:AAE3567
Gymnometriocnemus kamimegavirgus|BIOUG05220-B03|Kate Perez|BIN Taxonomy Match|BOLD:AAE3567

[illegible]
Gymnometriocnemus|24407-F05|Kate Perez|BIN Taxonomy Match|BOLD:AAE3567
Gymnometriocnemus kamimegavirgus|BIOUG15565-A10|Elisabeth Stur|BIN Taxonomy Match|BOLD:AAE3567
Gymnometriocnemus kamimegavirgus|BIOUG05220-B03|Kate Perez|BIN Taxonomy Match|BOLD:AAE3567
Gymnometriocnemus kamimegavirgus|CH-OSF131|Elisabeth Stur|Morphology|BOLD:AAE3567
Gymnometriocnemus kamimegavirgus|BIOUG05267-D10|Kate Perez|BIN Taxonomy Match|BOLD:AAE3567
Gymnometriocnemus kamimegavirgus|SOE437|Elisabeth Stur|Morphology|BOLD:AAE3567
Gymnometriocnemus kamimegavirgus|CH-OSF30|Elisabeth Stur|Morphology|BOLD:AAE3567
Gymnometriocnemus kamimegavirgus|ATNA267|Elisabeth Stur|Morphology|BOLD:AAE3567
Gymnometriocnemus kamimegavirgus|ATNA269|Elisabeth Stur|Morphology|BOLD:AAE3567
Gymnometriocnemus kamimegavirgus|ZFMK-TIS-33185|Niklas Beckers||BOLD:AAE3567
Gymnometriocnemus volitans|ZMUO.024294|Lauri Paasivirta||BOLD:AAE3567
Gymnometriocnemus volitans|ZMUO.024295|Lauri Paasivirta||BOLD:AAE3567
Gymnometriocnemus kamimegavirgus|BIOUG05109-C11|Kate Perez|BIN Taxonomy Match|BOLD:AAE3567
Gymnometriocnemus|24375-G11|Kate Perez|BIN Taxonomy Match|BOLD:AAE3567
Gymnometriocnemus|24375-A10|Kate Perez|BIN Taxonomy Match|BOLD:AAE3567
Gymnometriocnemus kamimegavirgus|BIOUG05106-C12|Kate Perez|BIN Taxonomy Match|BOLD:AAE3567
Gymnometriocnemus kamimegavirgus|BIOUG05106-F05|Kate Perez|BIN Taxonomy Match|BOLD:AAE3567
Gymnometriocnemus kamimegavirgus|BIOUG05107-A04|Kate Perez|BIN Taxonomy Match|BOLD:AAE3567
Gymnometriocnemus kamimegavirgus|BIOUG05107-B03|Kate Perez|BIN Taxonomy Match|BOLD:AAE3567
Gymnometriocnemus kamimegavirgus|BIOUG05107-B04|Kate Perez|BIN Taxonomy Match|BOLD:AAE3567
Gymnometriocnemus kamimegavirgus|BIOUG04795-D10|Kate Perez|BIN Taxonomy Match|BOLD:AAE3567
Gymnometriocnemus kamimegavirgus|BIOUG04795-E03|Kate Perez|BIN Taxonomy Match|BOLD:AAE3567
Gymnometriocnemus kamimegavirgus|BIOUG04795-H06|Kate Perez|BIN Taxonomy Match|BOLD:AAE3567
Gymnometriocnemus kamimegavirgus|BIOUG05101-C12|Kate Perez|BIN Taxonomy Match|BOLD:AAE3567
Gymnometriocnemus kamimegavirgus|BIOUG05101-H09|Kate Perez|BIN Taxonomy Match|BOLD:AAE3567
Gymnometriocnemus kamimegavirgus|BIOUG05102-F12|Kate Perez|BIN Taxonomy Match|BOLD:AAE3567
Gymnometriocnemus kamimegavirgus|BIOUG05103-F05|Kate Perez|BIN Taxonomy Match|BOLD:AAE3567
Gymnometriocnemus kamimegavirgus|BIOUG05103-G12|Kate Perez|BIN Taxonomy Match|BOLD:AAE3567
Gymnometriocnemus kamimegavirgus|BIOUG05103-H05|Kate Perez|BIN Taxonomy Match|BOLD:AAE3567
Gymnometriocnemus kamimegavirgus|BIOUG05105-A08|Kate Perez|BIN Taxonomy Match|BOLD:AAE3567
Gymnometriocnemus kamimegavirgus|BIOUG05105-B05|Kate Perez|BIN Taxonomy Match|BOLD:AAE3567
Gymnometriocnemus kamimegavirgus|BIOUG05105-D09|Kate Perez|BIN Taxonomy Match|BOLD:AAE3567
Gymnometriocnemus kamimegavirgus|BIOUG05105-E01|Kate Perez|BIN Taxonomy Match|BOLD:AAE3567
Gymnometriocnemus kamimegavirgus|BIOUG05107-G03|Kate Perez|BIN Taxonomy Match|BOLD:AAE3567
Gymnometriocnemus kamimegavirgus|BIOUG05108-C08|Kate Perez|BIN Taxonomy Match|BOLD:AAE3567
Gymnometriocnemus kamimegavirgus|BIOUG05108-E01|Kate Perez|BIN Taxonomy Match|BOLD:AAE3567
Gymnometriocnemus kamimegavirgus|BIOUG05103-C06|Kate Perez|BIN Taxonomy Match|BOLD:AAE3567
Gymnometriocnemus kamimegavirgus|BIOUG05109-H01|Kate Perez|BIN Taxonomy Match|BOLD:AAE3567
Gymnometriocnemus kamimegavirgus|BIOUG05141-D04|Kate Perez|BIN Taxonomy Match|BOLD:AAE3567
Gymnometriocnemus kamimegavirgus|BIOUG05141-G10|Kate Perez|BIN Taxonomy Match|BOLD:AAE3567
Gymnometriocnemus kamimegavirgus|BIOUG05217-E11|Kate Perez|BIN Taxonomy Match|BOLD:AAE3567
Gymnometriocnemus kamimegavirgus|BIOUG05216-B10|Kate Perez|BIN Taxonomy Match|BOLD:AAE3567
Gymnometriocnemus kamimegavirgus|BIOUG05103-C12|Kate Perez|BIN Taxonomy Match|BOLD:AAE3567
Gymnometriocnemus volitans|BIOUG04261-D03|Kate Perez|BIN Taxonomy Match|BOLD:AAE3567
Gymnometriocnemus kamimegavirgus|BIOUG05144-G09|Kate Perez|BIN Taxonomy Match|BOLD:AAE3567
Gymnometriocnemus kamimegavirgus|BIOUG05218-G05|Kate Perez|BIN Taxonomy Match|BOLD:AAE3567
Gymnometriocnemus kamimegavirgus|BIOUG05218-G07|Kate Perez|BIN Taxonomy Match|BOLD:AAE3567
Gymnometriocnemus kamimegavirgus|BIOUG05218-H02|Kate Perez|BIN Taxonomy Match|BOLD:AAE3567
Gymnometriocnemus kamimegavirgus|BIOUG05218-H10|Kate Perez|BIN Taxonomy Match|BOLD:AAE3567
Gymnometriocnemus kamimegavirgus|BIOUG05219-A10|Kate Perez|BIN Taxonomy Match|BOLD:AAE3567
Gymnometriocnemus kamimegavirgus|BIOUG05219-C04|Kate Perez|BIN Taxonomy Match|BOLD:AAE3567
Gymnometriocnemus kamimegavirgus|BIOUG05219-G04|Kate Perez|BIN Taxonomy Match|BOLD:AAE3567
Gymnometriocnemus kamimegavirgus|BIOUG05219-G12|Kate Perez|BIN Taxonomy Match|BOLD:AAE3567
Gymnometriocnemus kamimegavirgus|BIOUG05144-A11|Kate Perez|BIN Taxonomy Match|BOLD:AAE3567
Gymnometriocnemus kamimegavirgus|BIOUG05144-B01|Kate Perez|BIN Taxonomy Match|BOLD:AAE3567
Gymnometriocnemus kamimegavirgus|BIOUG05144-E12|Kate Perez|BIN Taxonomy Match|BOLD:AAE3567
Gymnometriocnemus kamimegavirgus|BIOUG05144-F04|Kate Perez|BIN Taxonomy Match|BOLD:AAE3567
Gymnometriocnemus kamimegavirgus|BIOUG05144-H08|Kate Perez|BIN Taxonomy Match|BOLD:AAE3567
Gymnometriocnemus kamimegavirgus|BIOUG05144-F12|Kate Perez|BIN Taxonomy Match|BOLD:AAE3567
Gymnometriocnemus kamimegavirgus|BIOUG05144-G10|Kate Perez|BIN Taxonomy Match|BOLD:AAE3567
Gymnometriocnemus kamimegavirgus|BIOUG05217-G08|Kate Perez|BIN Taxonomy Match|BOLD:AAE3567
Gymnometriocnemus kamimegavirgus|BIOUG05144-C11|Kate Perez|BIN Taxonomy Match|BOLD:AAE3567
Gymnometriocnemus kamimegavirgus|BIOUG05144-D06|Kate Perez|BIN Taxonomy Match|BOLD:AAE3567
Gymnometriocnemus kamimegavirgus|BIOUG05216-B09|Kate Perez|BIN Taxonomy Match|BOLD:AAE3567
Gymnometriocnemus kamimegavirgus|BIOUG05216-C03|Kate Perez|BIN Taxonomy Match|BOLD:AAE3567
Gymnometriocnemus kamimegavirgus|BIOUG05216-E11|Kate Perez|BIN Taxonomy Match|BOLD:AAE3567
Gymnometriocnemus kamimegavirgus|BIOUG05216-F07|Kate Perez|BIN Taxonomy Match|BOLD:AAE3567
Gymnometriocnemus kamimegavirgus|BIOUG05216-F11|Kate Perez|BIN Taxonomy Match|BOLD:AAE3567
Gymnometriocnemus kamimegavirgus|BIOUG05217-A07|Kate Perez|BIN Taxonomy Match|BOLD:AAE3567
Gymnometriocnemus kamimegavirgus|BIOUG05217-C03|Kate Perez|BIN Taxonomy Match|BOLD:AAE3567
Gymnometriocnemus kamimegavirgus|BIOUG05217-C07|Kate Perez|BIN Taxonomy Match|BOLD:AAE3567
Gymnometriocnemus kamimegavirgus|BIOUG05217-F05|Kate Perez|BIN Taxonomy Match|BOLD:AAE3567
Gymnometriocnemus kamimegavirgus|BIOUG05217-G01|Kate Perez|BIN Taxonomy Match|BOLD:AAE3567
Gymnometriocnemus kamimegavirgus|BIOUG04795-G07|Kate Perez|BIN Taxonomy Match|BOLD:AAE3567
Gymnometriocnemus kamimegavirgus|BIOUG05103-E12|Kate Perez|BIN Taxonomy Match|BOLD:AAE3567
Gymnometriocnemus kamimegavirgus|BIOUG05107-C10|Kate Perez|BIN Taxonomy Match|BOLD:AAE3567
Gymnometriocnemus kamimegavirgus|BIOUG05946-H05|Kate Perez|BIN Taxonomy Match|BOLD:AAE3567
Gymnometriocnemus kamimegavirgus|BIOUG05109-H11|Kate Perez|BIN Taxonomy Match|BOLD:AAE3567
Gymnometriocnemus kamimegavirgus|BIOUG05141-F08|Kate Perez|BIN Taxonomy Match|BOLD:AAE3567
Gymnometriocnemus kamimegavirgus|BIOUG05485-H01|Kate Perez|BIN Taxonomy Match|BOLD:AAE3567
Gymnometriocnemus kamimegavirgus|BIOUG05110-H06|Kate Perez|BIN Taxonomy Match|BOLD:AAE3567
Gymnometriocnemus kamimegavirgus|BIOUG05110-A03|Kate Perez|BIN Taxonomy Match|BOLD:AAE3567
Gymnometriocnemus kamimegavirgus|BIOUG05109-D10|Kate Perez|BIN Taxonomy Match|BOLD:AAE3567
Gymnometriocnemus kamimegavirgus|BIOUG05141-H06|Kate Perez|BIN Taxonomy Match|BOLD:AAE3567
Gymnometriocnemus kamimegavirgus|BIOUG05109-E06|Kate Perez|BIN Taxonomy Match|BOLD:AAE3567
Gymnometriocnemus kamimegavirgus|BIOUG05110-A07|Kate Perez|BIN Taxonomy Match|BOLD:AAE3567
Gymnometriocnemus kamimegavirgus|BIOUG05141-F05|Kate Perez|BIN Taxonomy Match|BOLD:AAE3567
Gymnometriocnemus kamimegavirgus|BIOUG05141-G07|Kate Perez|BIN Taxonomy Match|BOLD:AAE3567
Gymnometriocnemus kamimegavirgus|BIOUG05109-E09|Kate Perez|BIN Taxonomy Match|BOLD:AAE3567
Gymnometriocnemus kamimegavirgus|BIOUG05219-H09|Kate Perez|BIN Taxonomy Match|BOLD:AAE3567
Gymnometriocnemus kamimegavirgus|BIOUG05104-F09|Kate Perez|BIN Taxonomy Match|BOLD:AAE3567
Gymnometriocnemus kamimegavirgus|BIOUG05104-E07|Kate Perez|BIN Taxonomy Match|BOLD:AAE3567
Gymnometriocnemus kamimegavirgus|CCDB24183-D10|Sofia Wiedenbrug|Morphology|BOLD:AAE3567
Gymnometriocnemus kamimegavirgus|BIOUG05216-C08|Kate Perez|BIN Taxonomy Match|BOLD:AAE3567
Gymnometriocnemus kamimegavirgus|BIOUG05142-C04|Kate Perez|BIN Taxonomy Match|BOLD:AAE3567
Gymnometriocnemus kamimegavirgus|BIOUG05218-H11|Kate Perez|BIN Taxonomy Match|BOLD:AAE3567
Gymnometriocnemus kamimegavirgus|BIOUG05215-G07|Kate Perez|BIN Taxonomy Match|BOLD:AAE3567
Gymnometriocnemus kamimegavirgus|BIOUG04795-G03|Kate Perez|BIN Taxonomy Match|BOLD:AAE3567
Gymnometriocnemus kamimegavirgus|BIOUG05104-G08|Kate Perez|BIN Taxonomy Match|BOLD:AAE3567
Gymnometriocnemus kamimegavirgus|BIOUG05215-H04|Kate Perez|BIN Taxonomy Match|BOLD:AAE3567
Gymnometriocnemus kamimegavirgus|BIOUG05142-A02|Kate Perez|BIN Taxonomy Match|BOLD:AAE3567
Gymnometriocnemus kamimegavirgus|BIOUG05144-E04|Kate Perez|BIN Taxonomy Match|BOLD:AAE3567
Gymnometriocnemus kamimegavirgus|BIOUG05217-H01|Kate Perez|BIN Taxonomy Match|BOLD:AAE3567

Gymnometriocnemus kamimegavirgus|BIOUG05215-H04|Kate Perez|BIN Taxonomy Match|BOLD:AAE3567
Gymnometriocnemus kamimegavirgus|BIOUG05142-A02|Kate Perez|BIN Taxonomy Match|BOLD:AAE3567
Gymnometriocnemus kamimegavirgus|BIOUG05144-E04|Kate Perez|BIN Taxonomy Match|BOLD:AAE3567
Gymnometriocnemus kamimegavirgus|BIOUG05217-H01|Kate Perez|BIN Taxonomy Match|BOLD:AAE3567
Gymnometriocnemus|24376-C07|Kate Perez|BIN Taxonomy Match|BOLD:AAE3567
Gymnometriocnemus kamimegavirgus|BIOUG05149-C02|Kate Perez|BIN Taxonomy Match|BOLD:AAE3567
Gymnometriocnemus kamimegavirgus|BIOUG05149-G03|Kate Perez|BIN Taxonomy Match|BOLD:AAE3567
Gymnometriocnemus kamimegavirgus|BIOUG05101-C07|Kate Perez|BIN Taxonomy Match|BOLD:AAE3567
Gymnometriocnemus kamimegavirgus|BIOUG05399-F06|Kate Perez|BIN Taxonomy Match|BOLD:AAE3567
Gymnometriocnemus kamimegavirgus|CH-OSF32|Elisabeth Stur|Morphology|BOLD:AAE3567
Gymnometriocnemus|24376-D01|Kate Perez|BIN Taxonomy Match|BOLD:AAE3567
Gymnometriocnemus kamimegavirgus|BIOUG15460-A06|Elisabeth Stur|BIN Taxonomy Match|BOLD:AAE3567
Gymnometriocnemus kamimegavirgus|BIOUG15187-B12|Elisabeth Stur|BIN Taxonomy Match|BOLD:AAE3567
Gymnometriocnemus kamimegavirgus|CH-OSF29|Elisabeth Stur|Morphology|BOLD:AAE3567
Gymnometriocnemus kamimegavirgus|BIOUG05141-F04|Kate Perez|BIN Taxonomy Match|BOLD:AAE3567
Chironomidae|BIOUG05269-B10|Meredith Miller|Tree-based Identification (Feb 2019)
Chironomidae|BIOUG05269-H07|Meredith Miller|Tree-based Identification (Feb 2019)
Gymnometriocnemus kamimegavirgus|Finnmark522|Elisabeth Stur|Morphology|BOLD:AAE3567
Gymnometriocnemus|24537-G01|Kate Perez|BIN Taxonomy Match|BOLD:AAE3567
Gymnometriocnemus|24375-D07|Kate Perez|BIN Taxonomy Match|BOLD:AAE3567
Gymnometriocnemus kamimegavirgus|BIOUG15454-H07|Elisabeth Stur|BIN Taxonomy Match|BOLD:AAE3567
Gymnometriocnemus|24376-B02|Kate Perez|BIN Taxonomy Match|BOLD:AAE3567
Gymnometriocnemus|24376-C08|Kate Perez|BIN Taxonomy Match|BOLD:AAE3567
Gymnometriocnemus|24355-B10|Kate Perez|BIN Taxonomy Match|BOLD:AAE3567
Gymnometriocnemus|24376-G06|Kate Perez|BIN Taxonomy Match|BOLD:AAE3567
Gymnometriocnemus|24374-D09|Kate Perez|BIN Taxonomy Match|BOLD:AAE3567
Gymnometriocnemus volitans|BIOUG04085-D04|Kate Perez|BIN Taxonomy Match|BOLD:AAE3567
Gymnometriocnemus kamimegavirgus|BIOUG15457-F03|Elisabeth Stur|BIN Taxonomy Match|BOLD:AAE3567
Gymnometriocnemus kamimegavirgus|BIOUG15456-B02|Elisabeth Stur|BIN Taxonomy Match|BOLD:AAE3567
Gymnometriocnemus|24374-E02|Kate Perez|BIN Taxonomy Match|BOLD:AAE3567
Gymnometriocnemus|24375-G02|Kate Perez|BIN Taxonomy Match|BOLD:AAE3567
Gymnometriocnemus|24407-D08|Kate Perez|BIN Taxonomy Match|BOLD:AAE3567
Gymnometriocnemus|24407-E12|Kate Perez|BIN Taxonomy Match|BOLD:AAE3567
Gymnometriocnemus|24374-B08|Kate Perez|BIN Taxonomy Match|BOLD:AAE3567
Gymnometriocnemus|24374-E10|Kate Perez|BIN Taxonomy Match|BOLD:AAE3567
Gymnometriocnemus|24375-B10|Kate Perez|BIN Taxonomy Match|BOLD:AAE3567
Gymnometriocnemus|24374-A04|Kate Perez|BIN Taxonomy Match|BOLD:AAE3567
Gymnometriocnemus|24538-B11|Kate Perez|BIN Taxonomy Match|BOLD:AAE3567
Gymnometriocnemus|24376-F02|Kate Perez|BIN Taxonomy Match|BOLD:AAE3567
Gymnometriocnemus|24374-H10|Kate Perez|BIN Taxonomy Match|BOLD:AAE3567
Gymnometriocnemus kamimegavirgus|BIOUG05215-C02|Kate Perez|BIN Taxonomy Match|BOLD:AAE3567
Gymnometriocnemus|24374-B04|Kate Perez|BIN Taxonomy Match|BOLD:AAE3567
Gymnometriocnemus|24375-C08|Kate Perez|BIN Taxonomy Match|BOLD:AAE3567
Gymnometriocnemus|24374-E11|Kate Perez|BIN Taxonomy Match|BOLD:AAE3567
Gymnometriocnemus kamimegavirgus|BIOUG15464-H11|Elisabeth Stur|BIN Taxonomy Match|BOLD:AAE3567
Gymnometriocnemus|24375-G12|Kate Perez|BIN Taxonomy Match|BOLD:AAE3567
Gymnometriocnemus|24375-D04|Kate Perez|BIN Taxonomy Match|BOLD:AAE3567
Gymnometriocnemus kamimegavirgus|BIOUG05218-G12|Kate Perez|BIN Taxonomy Match|BOLD:AAE3567
Gymnometriocnemus volitans|BIOUG04261-C11|Kate Perez|BIN Taxonomy Match|BOLD:AAE3567
Gymnometriocnemus volitans|BIOUG04085-E07|Kate Perez|BIN Taxonomy Match|BOLD:AAE3567
Gymnometriocnemus|24374-D02|Kate Perez|BIN Taxonomy Match|BOLD:AAE3567
Gymnometriocnemus|24467-B10|Kate Perez|BIN Taxonomy Match|BOLD:AAE3567
Gymnometriocnemus volitans|ZMUO.024521|Lauri Paasivirta||BOLD:AAE3567
Gymnometriocnemus volitans|ZMUO.024522|Lauri Paasivirta||BOLD:AAE3567
Gymnometriocnemus|24376-A04|Kate Perez|BIN Taxonomy Match|BOLD:AAE3567
Gymnometriocnemus volitans|BIOUG04261-C05|Kate Perez|BIN Taxonomy Match|BOLD:AAE3567
Gymnometriocnemus volitans|BIOUG04261-F02|Kate Perez|BIN Taxonomy Match|BOLD:AAE3567
Gymnometriocnemus volitans|BIOUG04261-F05|Kate Perez|BIN Taxonomy Match|BOLD:AAE3567
Gymnometriocnemus|24540-D06|Kate Perez|BIN Taxonomy Match|BOLD:AAE3567
Gymnometriocnemus kamimegavirgus|BIOUG15458-D07|Elisabeth Stur|BIN Taxonomy Match|BOLD:AAE3567
Gymnometriocnemus kamimegavirgus|BIOUG15184-A02|Elisabeth Stur|BIN Taxonomy Match|BOLD:AAE3567
Gymnometriocnemus|24374-D06|Kate Perez|BIN Taxonomy Match|BOLD:AAE3567
Gymnometriocnemus|24469-A08|Kate Perez|BIN Taxonomy Match|BOLD:AAE3567
Gymnometriocnemus|24374-G01|Kate Perez|BIN Taxonomy Match|BOLD:AAE3567
Gymnometriocnemus|24374-D10|Kate Perez|BIN Taxonomy Match|BOLD:AAE3567
Gymnometriocnemus kamimegavirgus|BIOUG15457-A09|Elisabeth Stur|BIN Taxonomy Match|BOLD:AAE3567
Gymnometriocnemus kamimegavirgus|BIOUG15675-D02|Elisabeth Stur|BIN Taxonomy Match|BOLD:AAE3567
Gymnometriocnemus kamimegavirgus|BIOUG15459-E12|Elisabeth Stur|BIN Taxonomy Match|BOLD:AAE3567
Gymnometriocnemus kamimegavirgus|BIOUG15455-C06|Elisabeth Stur|BIN Taxonomy Match|BOLD:AAE3567
Gymnometriocnemus kamimegavirgus|BIOUG15458-G03|Elisabeth Stur|BIN Taxonomy Match|BOLD:AAE3567
Gymnometriocnemus kamimegavirgus|BIOUG15541-B03|Elisabeth Stur|BIN Taxonomy Match|BOLD:AAE3567
Gymnometriocnemus kamimegavirgus|BIOUG15464-H01|Elisabeth Stur|BIN Taxonomy Match|BOLD:AAE3567
Gymnometriocnemus kamimegavirgus|Finnmark370|Elisabeth Stur|Morphology|BOLD:AAE3567
Gymnometriocnemus kamimegavirgus|BIOUG05267-G05|Kate Perez|BIN Taxonomy Match|BOLD:AAE3567
Gymnometriocnemus kamimegavirgus|BIOUG05486-D04|Kate Perez|BIN Taxonomy Match|BOLD:AAE3567
Gymnometriocnemus kamimegavirgus|BIOUG05215-F09|Kate Perez|BIN Taxonomy Match|BOLD:AAE3567
Gymnometriocnemus kamimegavirgus|BIOUG05141-G02|Kate Perez|BIN Taxonomy Match|BOLD:AAE3567
Gymnometriocnemus kamimegavirgus|BIOUG05219-H04|Kate Perez|BIN Taxonomy Match|BOLD:AAE3567
Gymnometriocnemus kamimegavirgus|BIOUG05215-E02|Kate Perez|BIN Taxonomy Match|BOLD:AAE3567
Gymnometriocnemus kamimegavirgus|BIOUG05267-E01|Kate Perez|BIN Taxonomy Match|BOLD:AAE3567
Gymnometriocnemus kamimegavirgus|BIOUG05267-C12|Kate Perez|BIN Taxonomy Match|BOLD:AAE3567
Gymnometriocnemus kamimegavirgus|BIOUG05217-A11|Kate Perez|BIN Taxonomy Match|BOLD:AAE3567
Gymnometriocnemus kamimegavirgus|BIOUG05216-C10|Kate Perez|BIN Taxonomy Match|BOLD:AAE3567
Gymnometriocnemus kamimegavirgus|BIOUG05216-C06|Kate Perez|BIN Taxonomy Match|BOLD:AAE3567
Gymnometriocnemus kamimegavirgus|BIOUG05216-E01|Kate Perez|BIN Taxonomy Match|BOLD:AAE3567
Gymnometriocnemus kamimegavirgus|BIOUG05216-D04|Kate Perez|BIN Taxonomy Match|BOLD:AAE3567
Gymnometriocnemus kamimegavirgus|BIOUG05216-F01|Kate Perez|BIN Taxonomy Match|BOLD:AAE3567
Gymnometriocnemus kamimegavirgus|BIOUG05216-E07|Kate Perez|BIN Taxonomy Match|BOLD:AAE3567
Gymnometriocnemus kamimegavirgus|BIOUG05219-H08|Kate Perez|BIN Taxonomy Match|BOLD:AAE3567
Gymnometriocnemus kamimegavirgus|BIOUG05219-H06|Kate Perez|BIN Taxonomy Match|BOLD:AAE3567
Gymnometriocnemus kamimegavirgus|BIOUG05218-F08|Kate Perez|BIN Taxonomy Match|BOLD:AAE3567
Gymnometriocnemus kamimegavirgus|BIOUG05141-G04|Kate Perez|BIN Taxonomy Match|BOLD:AAE3567
Gymnometriocnemus kamimegavirgus|BIOUG05141-C02|Kate Perez|BIN Taxonomy Match|BOLD:AAE3567
Gymnometriocnemus kamimegavirgus|BIOUG05141-A01|Kate Perez|BIN Taxonomy Match|BOLD:AAE3567
Gymnometriocnemus kamimegavirgus|BIOUG05110-F11|Kate Perez|BIN Taxonomy Match|BOLD:AAE3567
Gymnometriocnemus kamimegavirgus|BIOUG05144-E01|Kate Perez|BIN Taxonomy Match|BOLD:AAE3567
Gymnometriocnemus kamimegavirgus|BIOUG05105-C03|Kate Perez|BIN Taxonomy Match|BOLD:AAE3567
Gymnometriocnemus kamimegavirgus|BIOUG05105-B01|Kate Perez|BIN Taxonomy Match|BOLD:AAE3567
Gymnometriocnemus kamimegavirgus|BIOUG05103-H03|Kate Perez|BIN Taxonomy Match|BOLD:AAE3567
Gymnometriocnemus kamimegavirgus|BIOUG05103-F01|Kate Perez|BIN Taxonomy Match|BOLD:AAE3567
Gymnometriocnemus kamimegavirgus|BIOUG05103-B04|Kate Perez|BIN Taxonomy Match|BOLD:AAE3567
Gymnometriocnemus kamimegavirgus|BIOUG05102-E11|Kate Perez|BIN Taxonomy Match|BOLD:AAE3567
Gymnometriocnemus kamimegavirgus|BIOUG05101-H03|Kate Perez|BIN Taxonomy Match|BOLD:AAE3567

Gymnometriocnemus kamimegavirgus|BIOUG05103-B04|Kate Perez|BIN Taxonomy Match|BOLD:AAE3567
Gymnometriocnemus kamimegavirgus|BIOUG05102-E11|Kate Perez|BIN Taxonomy Match|BOLD:AAE3567
Gymnometriocnemus kamimegavirgus|BIOUG05101-H03|Kate Perez|BIN Taxonomy Match|BOLD:AAE3567
Gymnometriocnemus kamimegavirgus|BIOUG05101-G11|Kate Perez|BIN Taxonomy Match|BOLD:AAE3567
Gymnometriocnemus kamimegavirgus|BIOUG04795-H10|Kate Perez|BIN Taxonomy Match|BOLD:AAE3567
Gymnometriocnemus kamimegavirgus|BIOUG04795-G04|Kate Perez|BIN Taxonomy Match|BOLD:AAE3567
Gymnometriocnemus kamimegavirgus|BIOUG05144-C03|Kate Perez|BIN Taxonomy Match|BOLD:AAE3567
Gymnometriocnemus kamimegavirgus|BIOUG05217-H02|Kate Perez|BIN Taxonomy Match|BOLD:AAE3567
Gymnometriocnemus kamimegavirgus|BIOUG05144-C12|Kate Perez|BIN Taxonomy Match|BOLD:AAE3567
Gymnometriocnemus kamimegavirgus|BIOUG05144-C06|Kate Perez|BIN Taxonomy Match|BOLD:AAE3567
Gymnometriocnemus kamimegavirgus|BIOUG05209-C12|Kate Perez|BIN Taxonomy Match|BOLD:AAE3567
Gymnometriocnemus kamimegavirgus|BIOUG05101-H08|Kate Perez|BIN Taxonomy Match|BOLD:AAE3567
Gymnometriocnemus kamimegavirgus|BIOUG05216-A08|Kate Perez|BIN Taxonomy Match|BOLD:AAE3567
Gymnometriocnemus kamimegavirgus|BIOUG05215-G10|Kate Perez|BIN Taxonomy Match|BOLD:AAE3567
Gymnometriocnemus kamimegavirgus|BIOUG05103-D08|Kate Perez|BIN Taxonomy Match|BOLD:AAE3567
Gymnometriocnemus kamimegavirgus|BIOUG05103-D06|Kate Perez|BIN Taxonomy Match|BOLD:AAE3567
Gymnometriocnemus kamimegavirgus|BIOUG05103-D02|Kate Perez|BIN Taxonomy Match|BOLD:AAE3567
Gymnometriocnemus kamimegavirgus|BIOUG05103-C08|Kate Perez|BIN Taxonomy Match|BOLD:AAE3567
Gymnometriocnemus kamimegavirgus|BIOUG05107-D03|Kate Perez|BIN Taxonomy Match|BOLD:AAE3567
Gymnometriocnemus kamimegavirgus|BIOUG05106-G02|Kate Perez|BIN Taxonomy Match|BOLD:AAE3567
Gymnometriocnemus kamimegavirgus|BIOUG05105-G08|Kate Perez|BIN Taxonomy Match|BOLD:AAE3567
Gymnometriocnemus kamimegavirgus|BIOUG05105-F10|Kate Perez|BIN Taxonomy Match|BOLD:AAE3567
Gymnometriocnemus kamimegavirgus|BIOUG05218-H08|Kate Perez|BIN Taxonomy Match|BOLD:AAE3567
Gymnometriocnemus kamimegavirgus|BIOUG05218-F11|Kate Perez|BIN Taxonomy Match|BOLD:AAE3567
Gymnometriocnemus kamimegavirgus|BIOUG05219-G08|Kate Perez|BIN Taxonomy Match|BOLD:AAE3567
Gymnometriocnemus kamimegavirgus|BIOUG05219-D06|Kate Perez|BIN Taxonomy Match|BOLD:AAE3567
Gymnometriocnemus kamimegavirgus|BIOUG05219-D03|Kate Perez|BIN Taxonomy Match|BOLD:AAE3567
Gymnometriocnemus kamimegavirgus|BIOUG05219-D02|Kate Perez|BIN Taxonomy Match|BOLD:AAE3567
Gymnometriocnemus kamimegavirgus|BIOUG05144-G03|Kate Perez|BIN Taxonomy Match|BOLD:AAE3567
Gymnometriocnemus kamimegavirgus|BIOUG05144-F10|Kate Perez|BIN Taxonomy Match|BOLD:AAE3567
Gymnometriocnemus kamimegavirgus|BIOUG05144-F08|Kate Perez|BIN Taxonomy Match|BOLD:AAE3567
Gymnometriocnemus kamimegavirgus|BIOUG05144-C08|Kate Perez|BIN Taxonomy Match|BOLD:AAE3567
Gymnometriocnemus kamimegavirgus|BIOUG05216-H10|Kate Perez|BIN Taxonomy Match|BOLD:AAE3567
Gymnometriocnemus kamimegavirgus|BIOUG05216-F02|Kate Perez|BIN Taxonomy Match|BOLD:AAE3567
Gymnometriocnemus kamimegavirgus|BIOUG05144-B12|Kate Perez|BIN Taxonomy Match|BOLD:AAE3567
Gymnometriocnemus kamimegavirgus|BIOUG05144-B07|Kate Perez|BIN Taxonomy Match|BOLD:AAE3567
Gymnometriocnemus kamimegavirgus|BIOUG05149-H08|Kate Perez|BIN Taxonomy Match|BOLD:AAE3567
Gymnometriocnemus kamimegavirgus|BIOUG05144-G07|Kate Perez|BIN Taxonomy Match|BOLD:AAE3567
Gymnometriocnemus kamimegavirgus|BIOUG05144-H09|Kate Perez|BIN Taxonomy Match|BOLD:AAE3567
Gymnometriocnemus kamimegavirgus|BIOUG05144-G05|Kate Perez|BIN Taxonomy Match|BOLD:AAE3567
Gymnometriocnemus kamimegavirgus|BIOUG05106-G01|Kate Perez|BIN Taxonomy Match|BOLD:AAE3567
Gymnometriocnemus kamimegavirgus|BIOUG05106-D09|Kate Perez|BIN Taxonomy Match|BOLD:AAE3567
Gymnometriocnemus kamimegavirgus|BIOUG05217-F01|Kate Perez|BIN Taxonomy Match|BOLD:AAE3567
Gymnometriocnemus kamimegavirgus|BIOUG05217-B10|Kate Perez|BIN Taxonomy Match|BOLD:AAE3567
Gymnometriocnemus kamimegavirgus|BIOUG04795-G02|Kate Perez|BIN Taxonomy Match|BOLD:AAE3567
Gymnometriocnemus kamimegavirgus|BIOUG05217-G03|Kate Perez|BIN Taxonomy Match|BOLD:AAE3567
Gymnometriocnemus kamimegavirgus|BIOUG05217-E07|Kate Perez|BIN Taxonomy Match|BOLD:AAE3567
Gymnometriocnemus kamimegavirgus|BIOUG04795-H09|Kate Perez|BIN Taxonomy Match|BOLD:AAE3567
Gymnometriocnemus kamimegavirgus|BIOUG05219-G01|Kate Perez|BIN Taxonomy Match|BOLD:AAE3567
Gymnometriocnemus kamimegavirgus|BIOUG05219-E07|Kate Perez|BIN Taxonomy Match|BOLD:AAE3567
Gymnometriocnemus kamimegavirgus|BIOUG05102-B11|Kate Perez|BIN Taxonomy Match|BOLD:AAE3567
Gymnometriocnemus kamimegavirgus|BIOUG05219-G05|Kate Perez|BIN Taxonomy Match|BOLD:AAE3567
Gymnometriocnemus kamimegavirgus|BIOUG05102-B10|Kate Perez|BIN Taxonomy Match|BOLD:AAE3567
Gymnometriocnemus kamimegavirgus|BIOUG05103-F12|Kate Perez|BIN Taxonomy Match|BOLD:AAE3567
Gymnometriocnemus kamimegavirgus|BIOUG05218-H06|Kate Perez|BIN Taxonomy Match|BOLD:AAE3567
Gymnometriocnemus kamimegavirgus|BIOUG05105-H05|Kate Perez|BIN Taxonomy Match|BOLD:AAE3567
Gymnometriocnemus kamimegavirgus|BIOUG04795-C12|Kate Perez|BIN Taxonomy Match|BOLD:AAE3567
Gymnometriocnemus kamimegavirgus|BIOUG05141-E09|Kate Perez|BIN Taxonomy Match|BOLD:AAE3567
Gymnometriocnemus kamimegavirgus|BIOUG05109-C08|Kate Perez|BIN Taxonomy Match|BOLD:AAE3567
Gymnometriocnemus kamimegavirgus|BIOUG05110-D11|Kate Perez|BIN Taxonomy Match|BOLD:AAE3567
Gymnometriocnemus kamimegavirgus|BIOUG05110-G12|Kate Perez|BIN Taxonomy Match|BOLD:AAE3567
Gymnometriocnemus kamimegavirgus|BIOUG05141-A03|Kate Perez|BIN Taxonomy Match|BOLD:AAE3567
Gymnometriocnemus kamimegavirgus|BIOUG05141-B08|Kate Perez|BIN Taxonomy Match|BOLD:AAE3567
Gymnometriocnemus kamimegavirgus|BIOUG05141-C01|Kate Perez|BIN Taxonomy Match|BOLD:AAE3567
Gymnometriocnemus kamimegavirgus|BIOUG05141-D11|Kate Perez|BIN Taxonomy Match|BOLD:AAE3567
Gymnometriocnemus kamimegavirgus|BIOUG05141-F06|Kate Perez|BIN Taxonomy Match|BOLD:AAE3567
Gymnometriocnemus kamimegavirgus|BIOUG05219-A02|Kate Perez|BIN Taxonomy Match|BOLD:AAE3567
Gymnometriocnemus kamimegavirgus|BIOUG05219-C11|Kate Perez|BIN Taxonomy Match|BOLD:AAE3567
Gymnometriocnemus kamimegavirgus|BIOUG05219-D01|Kate Perez|BIN Taxonomy Match|BOLD:AAE3567
Gymnometriocnemus kamimegavirgus|BIOUG05110-G11|Kate Perez|BIN Taxonomy Match|BOLD:AAE3567
Gymnometriocnemus kamimegavirgus|BIOUG05141-B05|Kate Perez|BIN Taxonomy Match|BOLD:AAE3567
Gymnometriocnemus kamimegavirgus|BIOUG05267-C01|Kate Perez|BIN Taxonomy Match|BOLD:AAE3567
Gymnometriocnemus kamimegavirgus|BIOUG05220-A07|Kate Perez|BIN Taxonomy Match|BOLD:AAE3567
Gymnometriocnemus kamimegavirgus|BIOUG05106-D01|Kate Perez|BIN Taxonomy Match|BOLD:AAE3567
Gymnometriocnemus kamimegavirgus|BIOUG05104-D05|Kate Perez|BIN Taxonomy Match|BOLD:AAE3567
Gymnometriocnemus kamimegavirgus|BIOUG05144-H03|Kate Perez|BIN Taxonomy Match|BOLD:AAE3567
Gymnometriocnemus kamimegavirgus|BIOUG05144-E05|Kate Perez|BIN Taxonomy Match|BOLD:AAE3567
Gymnometriocnemus kamimegavirgus|BIOUG05219-D07|Kate Perez|BIN Taxonomy Match|BOLD:AAE3567
Gymnometriocnemus kamimegavirgus|BIOUG05102-G01|Kate Perez|BIN Taxonomy Match|BOLD:AAE3567
Gymnometriocnemus kamimegavirgus|BIOUG05141-H10|Kate Perez|BIN Taxonomy Match|BOLD:AAE3567
Gymnometriocnemus kamimegavirgus|BIOUG05215-H10|Kate Perez|BIN Taxonomy Match|BOLD:AAE3567
Gymnometriocnemus kamimegavirgus|BIOUG05144-D10|Kate Perez|BIN Taxonomy Match|BOLD:AAE3567
Gymnometriocnemus kamimegavirgus|BIOUG05215-D03|Kate Perez|BIN Taxonomy Match|BOLD:AAE3567
Gymnometriocnemus kamimegavirgus|BIOUG05215-H08|Kate Perez|BIN Taxonomy Match|BOLD:AAE3567
Gymnometriocnemus kamimegavirgus|BIOUG05141-B04|Kate Perez|BIN Taxonomy Match|BOLD:AAE3567
Gymnometriocnemus kamimegavirgus|BIOUG05110-F02|Kate Perez|BIN Taxonomy Match|BOLD:AAE3567
Gymnometriocnemus kamimegavirgus|BIOUG05149-E08|Kate Perez|BIN Taxonomy Match|BOLD:AAE3567
Gymnometriocnemus kamimegavirgus|BIOUG05149-E09|Kate Perez|BIN Taxonomy Match|BOLD:AAE3567
Gymnometriocnemus kamimegavirgus|BIOUG05149-F03|Kate Perez|BIN Taxonomy Match|BOLD:AAE3567
Gymnometriocnemus kamimegavirgus|BIOUG05220-B07|Kate Perez|BIN Taxonomy Match|BOLD:AAE3567
Gymnometriocnemus kamimegavirgus|BIOUG05267-F12|Sofia Wiedenbrug|BIN Taxonomy Match|BOLD:AAE3567
Gymnometriocnemus kamimegavirgus|BIOUG05269-C12|Sofia Wiedenbrug||BOLD:AAE3567
Gymnometriocnemus|25128-D11|Kate Perez|BIN Taxonomy Match (Mar 2018)|BOLD:AAI1981
Gymnometriocnemus|23878-G09|Kate Perez|BIN Taxonomy Match|BOLD:AAI1981
Gymnometriocnemus|SOE315|Torbjorn Ekrem||BOLD:AAI1981
Gymnometriocnemus brumalis|Finnmark75|Elisabeth Stur||BOLD:AAM5243
Gymnometriocnemus brumalis|ZMUO.024514|Lauri Paasivirta||BOLD:AAW0095
Gymnometriocnemus brumalis|ZMUO.024513|Lauri Paasivirta||BOLD:AAW0095
Gymnometriocnemus brumalis|CH-OSF70|Elisabeth Stur||BOLD:AAW0095
Gymnometriocnemus brumalis|CH-OSF31|Elisabeth Stur||BOLD:AAW0095
Gymnometriocnemus brumalis|CH-OSF14|Elisabeth Stur||BOLD:AAW0095
Gymnometriocnemus brumalis|CH-OSF13|Elisabeth Stur||BOLD:AAW0095
Gymnometriocnemus|BIOUG04269-D06|Kate Perez|BIN Taxonomy Match|BOLD:AAU6760

Gymnometriocnemus brumalis|CH-OSF14|Elisabeth Stur||BOLD:AAW0095
Gymnometriocnemus brumalis|CH-OSF13|Elisabeth Stur||BOLD:AAW0095
Gymnometriocnemus|BIOUG04269-D06|Kate Perez|BIN Taxonomy Match|BOLD:AAU6760
Gymnometriocnemus|BIOUG04261-B09|Kate Perez|BIN Taxonomy Match|BOLD:AAU6760
Gymnometriocnemus brumalis|BIOUG04261-E12|Kate Perez|BIN Taxonomy Match|BOLD:ACC1684
Gymnometriocnemus brumalis|Finnmark58|Elisabeth Stur||BOLD:AAL3408
Gymnometriocnemus brumalis|SOE452|Elisabeth Stur
Gymnometriocnemus brumalis|NO31|||BOLD:AAL3408
Gymnometriocnemus brumalis|Finnmark484|Elisabeth Stur||BOLD:AAL3408
Gymnometriocnemus brumalis|BIOUG04269-A09|Kate Perez|BIN Taxonomy Match|BOLD:AAL3408
Gymnometriocnemus brumalis|Finnmark324|Elisabeth Stur||BOLD:AAL3408
Gymnometriocnemus brumalis|ZSM-DIP-33130-G03|Caroline Chimeno|BIN Taxonomy Match (Jan 2022)...
Gymnometriocnemus brumalis|ZSM-DIP-33143-C02|Caroline Chimeno|BIN Taxonomy Match (Jan 2022)...
Gymnometriocnemus brumalis|ZSM-DIP-33130-F12|Caroline Chimeno|BIN Taxonomy Match (Jan 2022)...
Gymnometriocnemus brumalis|ZSM-DIP-33132-H11|Caroline Chimeno|BIN Taxonomy Match (Jan 2022)...
Chironomidae|CCDB24037-H01||BIN Taxonomy Match
Gymnometriocnemus brumalis|CCDB24037-H05|Sofia Wiedenbrug|BIN Taxonomy Match|BOLD:ACD4501
Gymnometriocnemus brumalis|ZSM34342-C03|Sofia Wiedenbrug|Morphology|BOLD:ACD4501
Gymnometriocnemus brumalis|ZSM34342-C11|Sofia Wiedenbrug|Morphology|BOLD:ACD4501
Gymnometriocnemus brumalis|CCDB24037-G12|Sofia Wiedenbrug|BIN Taxonomy Match|BOLD:ACD4501
Gymnometriocnemus brumalis|ZSM-DIP-33145-A12|Caroline Chimeno|BIN Taxonomy Match (Jan 2022)...
Gymnometriocnemus brumalis|BIOUG16777-A12|Renee Miskie|BIN Taxonomy Match (Oct 2021)
Gymnometriocnemus brumalis|BIOUG16785-D09|Kate Perez|BOLD ID Engine Manual|BOLD:ACD4501
Gymnometriocnemus brumalis|BIOUG16784-E06|Kate Perez|BOLD ID Engine Manual|BOLD:ACD4501
Gymnometriocnemus brumalis|BIOUG16740-H08|Kate Perez|BOLD ID Engine Manual|BOLD:ACD4501
Gymnometriocnemus brumalis|BIOUG16776-F10|Kate Perez|BOLD ID Engine Manual|BOLD:ACD4501
Gymnometriocnemus brumalis|BIOUG16784-A09|Kate Perez|BOLD ID Engine Manual|BOLD:ACD4501
Gymnometriocnemus brumalis|BIOUG16784-C02|Kate Perez|BOLD ID Engine Manual|BOLD:ACD4501
Gymnometriocnemus brumalis|BIOUG16740-A07|Kate Perez|BOLD ID Engine Manual|BOLD:ACD4501
Gymnometriocnemus brumalis|BIOUG16738-C07|Kate Perez|BOLD ID Engine Manual|BOLD:ACD4501
Gymnometriocnemus brumalis|BIOUG16738-D07|Kate Perez|BOLD ID Engine Manual|BOLD:ACD4501
Gymnometriocnemus brumalis|BIOUG16738-F04|Kate Perez|BOLD ID Engine Manual|BOLD:ACD4501
Gymnometriocnemus brumalis|BIOUG16740-H07|Kate Perez|BOLD ID Engine Manual|BOLD:ACD4501
Gymnometriocnemus brumalis|BIOUG16720-E11|Kate Perez|BOLD ID Engine Manual|BOLD:ACD4501
Gymnometriocnemus brumalis|BIOUG16720-G01|Kate Perez|BOLD ID Engine Manual|BOLD:ACD4501
Gymnometriocnemus brumalis|BIOUG16775-H02|Kate Perez|BOLD ID Engine Manual|BOLD:ACD4501
Gymnometriocnemus brumalis|BIOUG16784-C05|Kate Perez|BOLD ID Engine Manual|BOLD:ACD4501
Gymnometriocnemus brumalis|BIOUG16775-H07|Kate Perez|BOLD ID Engine Manual|BOLD:ACD4501
Gymnometriocnemus brumalis|BIOUG16779-H06|Kate Perez|BOLD ID Engine Manual|BOLD:ACD4501
Gymnometriocnemus brumalis|BIOUG16778-G04|Kate Perez|BOLD ID Engine Manual|BOLD:ACD4501
Gymnometriocnemus brumalis|BIOUG16776-G03|Kate Perez|BOLD ID Engine Manual|BOLD:ACD4501
Gymnometriocnemus brumalis|BIOUG16779-C05|Kate Perez|BOLD ID Engine Manual|BOLD:ACD4501
Gymnometriocnemus brumalis|BIOUG16721-F06|Kate Perez|BOLD ID Engine Manual|BOLD:ACD4501
Gymnometriocnemus brumalis|BIOUG16775-G06|Kate Perez|BOLD ID Engine Manual|BOLD:ACD4501
Gymnometriocnemus brumalis|BIOUG16739-G06|Kate Perez|BOLD ID Engine Manual|BOLD:ACD4501
Gymnometriocnemus brumalis|BIOUG16782-A11|Kate Perez|BOLD ID Engine Manual|BOLD:ACD4501
Gymnometriocnemus brumalis|BIOUG16720-B08|Kate Perez|BOLD ID Engine Manual|BOLD:ACD4501
Gymnometriocnemus brumalis|BIOUG16785-E07|Kate Perez|BOLD ID Engine Manual|BOLD:ACD4501
Gymnometriocnemus brumalis|BIOUG16777-B06|Kate Perez|BOLD ID Engine Manual|BOLD:ACD4501
Gymnometriocnemus brumalis|BIOUG16739-B02|Kate Perez|BOLD ID Engine Manual|BOLD:ACD4501
Gymnometriocnemus brumalis|ZSM-DIP-33132-G04|Caroline Chimeno|BIN Taxonomy Match (Jan 2022)...
Gymnometriocnemus brumalis|CCDB22605-F06|Sofia Wiedenbrug||BOLD:ACD4501
Gymnometriocnemus brumalis|ZSM-DIP-33143-D02|Caroline Chimeno|BIN Taxonomy Match (Jan 2022)...
Gymnometriocnemus brumalis|ZSM-DIP-33132-G03|Caroline Chimeno|BIN Taxonomy Match (Jan 2022)...
Gymnometriocnemus brumalis|ZSM-DIP-33132-G01|Caroline Chimeno|BIN Taxonomy Match (Jan 2022)...
Gymnometriocnemus brumalis|ZSM-DIP-33131-A03|Caroline Chimeno|BIN Taxonomy Match (Jan 2022)...
Gymnometriocnemus brumalis|ZSM-DIP-33132-F12|Caroline Chimeno|BIN Taxonomy Match (Jan 2022)...
Gymnometriocnemus brumalis|ZSM-DIP-33132-G02|Caroline Chimeno|BIN Taxonomy Match (Jan 2022)...
Gymnometriocnemus brumalis|ZSM-DIP-33132-G06|Caroline Chimeno|BIN Taxonomy Match (Jan 2022)...
Gymnometriocnemus brumalis|ZSM-DIP-33132-G07|Caroline Chimeno|BIN Taxonomy Match (Jan 2022)...
Gymnometriocnemus brumalis|ZSM-DIP-33132-G09|Caroline Chimeno|BIN Taxonomy Match (Jan 2022)...
Gymnometriocnemus brumalis|ZSM-DIP-33130-B08|Caroline Chimeno|BIN Taxonomy Match (Jan 2022)...
Gymnometriocnemus brumalis|ZSM-DIP-33131-A02|Caroline Chimeno|BIN Taxonomy Match (Jan 2022)...
Gymnometriocnemus brumalis|ZSM-DIP-33131-B09|Caroline Chimeno|BIN Taxonomy Match (Jan 2022)...
Gymnometriocnemus brumalis|ZSM-DIP-33131-B10|Caroline Chimeno|BIN Taxonomy Match (Jan 2022)...
Gymnometriocnemus brumalis|ZSM-DIP-33141-C08|Caroline Chimeno|BIN Taxonomy Match (Jan 2022)...
Gymnometriocnemus brumalis|ZSM-DIP-33145-C11|Caroline Chimeno|BIN Taxonomy Match (Jan 2022)...
Gymnometriocnemus brumalis|ZSM-DIP-33145-C12|Caroline Chimeno|BIN Taxonomy Match (Jan 2022)...
Gymnometriocnemus brumalis|ZSM-DIP-33131-A06|Caroline Chimeno|BIN Taxonomy Match (Jan 2022)...
Gymnometriocnemus brumalis|CCDB22605-D12|Sofia Wiedenbrug||BOLD:ACD4501
Gymnometriocnemus brumalis|ZSM-DIP-33131-B08|Caroline Chimeno|BIN Taxonomy Match (Jan 2022)...
Gymnometriocnemus brumalis|ZSM-DIP-33130-F10|Caroline Chimeno|BIN Taxonomy Match (Jan 2022)...
Gymnometriocnemus autumnalis|Finnmark201|Elisabeth Stur|Morphology|BOLD:AAM3286
Gymnometriocnemus autumnalis|ATNA481|Elisabeth Stur|Morphology|BOLD:AAM3286
Gymnometriocnemus autumnalis|Finnmark563|Elisabeth Stur|Morphology|BOLD:AAM3286
Gymnometriocnemus autumnalis|Finnmark282|Elisabeth Stur|Morphology
Metriocnemus|ZMUO.025825|Lauri Paasivirta||BOLD:ACC5504
Tvetenia verralli|ZMUO.025946|Lauri Paasivirta||BOLD:AAW8775
Tvetenia tshernovskii|Finnmark817|Elisabeth Stur||BOLD:AAW8775
Tvetenia tshernovskii|Finnmark823|Elisabeth Stur||BOLD:AAW8775
Tvetenia tshernovskii|Finnmark809|Elisabeth Stur||BOLD:AAW8775
Tvetenia tshernovskii|Finnmark808|Elisabeth Stur||BOLD:AAW8775
Tvetenia verralli|ZMUO.025945|Lauri Paasivirta||BOLD:AAW8775
Tvetenia discoloripes|EJV-20110214|Lauri Paasivirta||BOLD:AAW8775
Tavastia yggdrasilia|ZMUO.025179|Lauri Paasivirta||BOLD:ADA7473
Tavastia yggdrasilia|ZMUO.025178|Lauri Paasivirta||BOLD:ADA7473
Orthocladius cf. abiskoensis|Finnmark712|Torbjorn Ekrem||BOLD:AAV2830
Orthocladius cf. abiskoensis|Finnmark714|Torbjorn Ekrem||BOLD:AAV2831
Orthocladius cf. abiskoensis|Finnmark426|Torbjorn Ekrem||BOLD:AAV2831
Chaetocladius minutissimus|ES264|Elisabeth Stur||BOLD:AAW6731
Chaetocladius perennis|BIOUG15952-C10|Elisabeth Stur|BIN Taxonomy Match|BOLD:ACF6903
Chaetocladius aff. perennis|ZSM34342-A11|Sofia Wiedenbrug|Morphology|BOLD:ACF6903
Chaetocladius perennis|CCDB24038-B02|Jerome Moriniere|BIN Taxonomy Match|BOLD:ACF6903
Chaetocladius perennis|BIOUG16721-A12|Jerome Moriniere|BIN Taxonomy Match|BOLD:ACF6903
Chaetocladius perennis|BC-ZSM-DIP-22491-F01|Dieter Doczkal|BIN Taxonomy Match|BOLD:ACF6903
Chaetocladius perennis|ZSM-DIP-33130-E06|Caroline Chimeno|BIN Taxonomy Match (Jan 2022)|BOL...
Chaetocladius perennis|BIOUG16785-C12|Jerome Moriniere|BIN Taxonomy Match|BOLD:ACF6903
Chaetocladius perennis|BC-ZSM-DIP-22491-F05|Dieter Doczkal|BIN Taxonomy Match|BOLD:ACF6903
Chaetocladius perennis|BIOUG16721-F03|Jerome Moriniere|BIN Taxonomy Match|BOLD:ACF6903
Chaetocladius perennis|BC-ZSM-DIP-22491-D02|Dieter Doczkal|BIN Taxonomy Match|BOLD:ACF6903
Chaetocladius perennis|ZSM-DIP-33136-B09|Caroline Chimeno|BIN Taxonomy Match (Jan 2022)|BOL...
Chaetocladius perennis|BIOUG16853-E08|Jerome Moriniere|BIN Taxonomy Match|BOLD:ACF6903
Chaetocladius perennis|CCDB24038-F04|Jerome Moriniere|BIN Taxonomy Match|BOLD:ACF6903

Chaetocladius perennis|BC-ZSM-DIP-22491-D02|Dieter Doczkal|BIN Taxonomy Match|BOLD:ACF6903
Chaetocladius perennis|ZSM-DIP-33136-B09|Caroline Chimeno|BIN Taxonomy Match (Jan 2022)|BOL...
Chaetocladius perennis|BIOUG16853-E08|Jerome Moriniere|BIN Taxonomy Match|BOLD:ACF6903
Chaetocladius perennis|CCDB24038-E04|Jerome Moriniere|BIN Taxonomy Match|BOLD:ACF6903
Chaetocladius perennis|CCDB24039-G05|Jerome Moriniere|BIN Taxonomy Match|BOLD:ACF6903
Chaetocladius perennis|BC-ZSM-DIP-22551-F08|Dieter Doczkal|BIN Taxonomy Match|BOLD:ACF6903
Chaetocladius perennis|BC-ZSM-DIP-22491-E09|Dieter Doczkal|BIN Taxonomy Match|BOLD:ACF6903
Chaetocladius perennis|CCDB24038-B01|Jerome Moriniere|BIN Taxonomy Match|BOLD:ACF6903
Chaetocladius perennis|CCDB24039-F12|Jerome Moriniere|BIN Taxonomy Match|BOLD:ACF6903
Chaetocladius perennis|BC-ZSM-DIP-22491-F03|Dieter Doczkal|BIN Taxonomy Match|BOLD:ACF6903
Chaetocladius perennis|BC-ZSM-DIP-22488-E07|Dieter Doczkal|BIN Taxonomy Match|BOLD:ACF6903
Chaetocladius perennis|BIOUG16739-E09|Jerome Moriniere|BIN Taxonomy Match|BOLD:ACF6903
Chaetocladius perennis|CCDB24038-D03|Jerome Moriniere|BIN Taxonomy Match|BOLD:ACF6903
Chaetocladius perennis|BIOUG16261-C06|Elisabeth Stur|BIN Taxonomy Match|BOLD:ACF6903
Chaetocladius perennis|BIOUG15454-C12|Elisabeth Stur|BIN Taxonomy Match|BOLD:ACF6903
Chaetocladius aff. perennis|ZSM34342-B11|Sofia Wiedenbrug|Morphology|BOLD:ACF6903
Chaetocladius perennis|BIOUG15459-G12|Kate Perez|BIN Taxonomy Match|BOLD:ACF6903
Chaetocladius perennis|TRD-CH115|Elisabeth Stur|morphology|BOLD:ACF6903
Chaetocladius perennis|BIOUG17025-G05|Elisabeth Stur|BIN Taxonomy Match|BOLD:ACF6903
Chaetocladius perennis|TRD-CH87|Elisabeth Stur|morphology|BOLD:ACF6903
Chaetocladius perennis|TRD-CH120|Elisabeth Stur||BOLD:ACF6903
Chaetocladius perennis|ZMUO.024519|Lauri Paasivirta||BOLD:ACF6903
Chaetocladius perennis|BIOUG14592-A03|Kate Perez|BIN Taxonomy Match|BOLD:ACF6903
Chaetocladius perennis|ZMUO.024520|Lauri Paasivirta||BOLD:ACF6903
Chaetocladius perennis|BIOUG16720-G05|Jerome Moriniere|BIN Taxonomy Match|BOLD:ACF6903
Chaetocladius perennis|BIOUG16739-A11|Jerome Moriniere|BIN Taxonomy Match|BOLD:ACF6903
Chaetocladius perennis|BIOUG16721-E03|Jerome Moriniere|BIN Taxonomy Match|BOLD:ACF6903
Chaetocladius perennis|BIOUG14592-G08|Elisabeth Stur|BIN Taxonomy Match|BOLD:ACF6903
Chaetocladius perennis|BIOUG17025-F01|Elisabeth Stur|BIN Taxonomy Match|BOLD:ACF6903
Chaetocladius perennis|CH-OSF07|Elisabeth Stur||BOLD:ACF6903
Chaetocladius perennis|BIOUG14592-A08|Elisabeth Stur|BIN Taxonomy Match|BOLD:ACF6903
Chaetocladius perennis|BIOUG16333-E01|Elisabeth Stur|BIN Taxonomy Match|BOLD:ACF6903
Chaetocladius perennis|BIOUG14592-A07|Elisabeth Stur|BIN Taxonomy Match|BOLD:ACF6903
Chaetocladius perennis|BIOUG15762-B04|Kate Perez|BIN Taxonomy Match (Jun 2019)|BOLD:ACF6903
Chaetocladius perennis|BIOUG17025-C03|Elisabeth Stur|BIN Taxonomy Match|BOLD:ACF6903
Chaetocladius perennis|BIOUG15766-C03|Elisabeth Stur|BIN Taxonomy Match|BOLD:ACF6903
Chaetocladius perennis|BIOUG14592-A04|Elisabeth Stur|BIN Taxonomy Match|BOLD:ACF6903
Chaetocladius perennis|BC-ZSM-DIP-22491-H10|Dieter Doczkal|BIN Taxonomy Match|BOLD:ACF6903
Chaetocladius perennis|BIOUG17064-H06|Elisabeth Stur|BIN Taxonomy Match|BOLD:ACF6903
Chaetocladius perennis|BIOUG15761-B01|Elisabeth Stur|BIN Taxonomy Match|BOLD:ACF6903
Chaetocladius tenuistylus|ZMUO.026268|Lauri Paasivirta||BOLD:AAN5376
Chaetocladius tenuistylus|ZMUO.026267|Lauri Paasivirta||BOLD:AAN5376
Chaetocladius tenuistylus|Finnmark274|Elisabeth Stur||BOLD:AAN5376
Rheosmittia spinicornis|ZMUO.024825|Lauri Paasivirta||BOLD:ACM1900
Rheosmittia|ATNA582|Elisabeth Stur|morphology|BOLD:ACT6262
Rheosmittia spinicornis|ZMUO.024826|Lauri Paasivirta||BOLD:ACT6262
Rheosmittia|EBAI-Ch263|Elisabeth Stur|morphology|BOLD:ACT6262
Rheosmittia sp. 1ES|TRD-CH407|Elisabeth Stur|morphology|BOLD:ACT6262
Rheosmittia sp. 1ES|TRD-CH197|Elisabeth Stur|morphology|BOLD:ACT6262
Heterotanytarsus apicalis|ZMUO.024278|Lauri Paasivirta||BOLD:AAB8663
Heterotanytarsus apicalis|ZMUO.024277|Lauri Paasivirta||BOLD:AAB8663
Heterotanytarsus apicalis|Finnmark651|Elisabeth Stur||BOLD:AAB8663
Heterotanytarsus apicalis|Finnmark48|Elisabeth Stur||BOLD:AAB8663
Heterotanytarsus apicalis|Finnmark535|Elisabeth Stur||BOLD:AAB8663
Heterotanytarsus apicalis|Finnmark520|Elisabeth Stur||BOLD:AAB8663
Heterotanytarsus apicalis|Finnmark428|Elisabeth Stur||BOLD:AAB8663
Heterotanytarsus apicalis|SOE411|Elisabeth Stur||BOLD:AAB8663
Heterotanytarsus apicalis|TRD-CH103|Elisabeth Stur||BOLD:AAB8663
Heterotanytarsus apicalis|Finnmark59|Elisabeth Stur||BOLD:AAB8663
Heterotanytarsus apicalis|Finnmark537|Elisabeth Stur||BOLD:AAB8663
Heterotanytarsus apicalis|SOE174|Elisabeth Stur||BOLD:AAB8663
Heterotanytarsus apicalis|Finnmark205|Elisabeth Stur||BOLD:AAB8663
Heterotanytarsus apicalis|SOE223|Elisabeth Stur||BOLD:AAB8663
Heterotanytarsus apicalis|SOE209|Elisabeth Stur||BOLD:AAB8663
Heterotanytarsus apicalis|SOE258|Elisabeth Stur||BOLD:AAB8663
Heterotanytarsus apicalis|SOE261|Elisabeth Stur||BOLD:AAB8663
Heterotanytarsus apicalis|SOE295|Elisabeth Stur||BOLD:AAB8663
Heterotanytarsus apicalis|SOE361|Elisabeth Stur||BOLD:AAB8663
Heterotanytarsus apicalis|SOE362|Elisabeth Stur||BOLD:AAB8663
Heterotanytarsus apicalis|SOE363|Elisabeth Stur||BOLD:AAB8663
Heterotanytarsus apicalis|Finnmark832|Elisabeth Stur||BOLD:AAB8663
Heterotanytarsus apicalis|SOE413|Elisabeth Stur||BOLD:AAB8663
Heterotanytarsus apicalis|SOE427|Elisabeth Stur||BOLD:AAB8663
Heterotanytarsus apicalis|TRD-CH278|Elisabeth Stur||BOLD:AAB8663
Heterotanytarsus apicalis|NHRS-BYWS000001170|Yngve Brodin|morphology|BOLD:AAB8663
Heterotanytarsus apicalis|ZMUO.024843|Lauri Paasivirta||BOLD:AAB8663
Heterotanytarsus apicalis|SOE412|Elisabeth Stur||BOLD:AAB8663
Heterotanytarsus apicalis|SOE294|Elisabeth Stur||BOLD:AAB8663
Heterotanytarsus apicalis|SOE211|Elisabeth Stur||BOLD:AAB8663
Heterotanytarsus apicalis|SOE204|Elisabeth Stur||BOLD:AAB8663
Heterotanytarsus apicalis|TRD-CH102|Elisabeth Stur||BOLD:AAB8663
Heterotanytarsus apicalis|Finnmark646|Elisabeth Stur||BOLD:AAB8663
Stackelbergina praeclara|Finnmark677|Elisabeth Stur||BOLD:AAV2833
Aagaardia protensa|ZMUO.024920|Lauri Paasivirta||BOLD:ACC0018
Aagaardia|BIOUG04153-D02|Kate Perez|BIN Taxonomy Match|BOLD:ACC0018
Aagaardia protensa|NO 101|Elisabeth Stur||BOLD:ACC0018
Rheocricotopus|GBOL11569|Sofia Wiedenbrug||BOLD:ACT8739
Rheocricotopus|CCDB22605-C11|Sofia Wiedenbrug||BOLD:ACT8739
Rheocricotopus|GBOL11570|Sofia Wiedenbrug||BOLD:ACT8739
Rheocricotopus|CCDB22605-C12|Sofia Wiedenbrug||BOLD:ACT8739
Rheocricotopus|CCDB22605-C10|Sofia Wiedenbrug||BOLD:ACT8739
Rheocricotopus|CCDB22605-C09|Sofia Wiedenbrug||BOLD:ACT8739
Rheocricotopus|CCDB22605-C08|Sofia Wiedenbrug||BOLD:ACT8739
Rheocricotopus|CCDB22605-C06|Sofia Wiedenbrug||BOLD:ACT8739
Krenosmittia halvorseni|EBAI-Ch245|Elisabeth Stur|morphology
Krenosmittia halvorseni|ATNA400|Elisabeth Stur||BOLD:AAE5796
Krenosmittia halvorseni|ATNA234|Elisabeth Stur||BOLD:AAE5796
Krenosmittia halvorseni|ATNA399|Elisabeth Stur||BOLD:AAE5796
Krenosmittia halvorseni|ATNA254|Elisabeth Stur||BOLD:AAE5796
Krenosmittia sp. 1ES|Finnmark191|Elisabeth Stur||BOLD:AAP6931
Krenosmittia halvorseni|ZMUO.025831|Lauri Paasivirta||BOLD:AAP6931
Krenosmittia sp. 1ES|ATNA525|Elisabeth Stur||BOLD:AAP6931
Krenosmittia sp. 1ES|ATNA524|Elisabeth Stur||BOLD:AAP6931
[illegible]

Krenosmittia halvorseni|ZMUO.025831|Lauri Paasivirta||BOLD:AAP6931
Krenosmittia sp. 1ES|ATNA525|Elisabeth Stur||BOLD:AAP6931
Krenosmittia sp. 1ES|ATNA524|Elisabeth Stur||BOLD:AAP6931
Krenosmittia sp. 1ES|ATNA554|Elisabeth Stur||BOLD:AAP6931
Krenosmittia sp. 1ES|ATNA532|Elisabeth Stur||BOLD:AAP6931
Krenosmittia sp. 1ES|Finnmark619|Elisabeth Stur||BOLD:AAP6931
Lappokiefferiella sp. 1ES|Finnmark871|Elisabeth Stur|morphology|BOLD:AAU5379
Lappokiefferiella sp. 1ES|Finnmark870|Elisabeth Stur|morphology|BOLD:AAU5379
Lappokiefferiella sp. 1ES|Finnmark375|Elisabeth Stur||BOLD:AAU5379
Lappokiefferiella sp. 1ES|Finnmark374|Elisabeth Stur||BOLD:AAU5379
Lappokiefferiella sp. 1ES|Finnmark328|Elisabeth Stur||BOLD:AAU5379
Lappokiefferiella sp. 1ES|Finnmark327|Elisabeth Stur
Chironomidae|CCDB24229-F01|Jerome Moriniere|BIN Taxonomy Match|BOLD:ACU4857
Chironomidae|CCDB24228-D09|Jerome Moriniere|BIN Taxonomy Match|BOLD:ACU4988
Chironomidae|CCDB24228-H04|Jerome Moriniere|BIN Taxonomy Match|BOLD:ACU4855
Chironomidae|ZSM-DIP-33142-H09|Caroline Chimeno|BIN Taxonomy Match (Jan 2022)|BOLD:ACU4855
Chironomidae|ZSM-DIP-33149-C04|Caroline Chimeno|BIN Taxonomy Match (Jan 2022)|BOLD:ACU4855
Chironomidae|ZSM-DIP-33146-C04|Caroline Chimeno|BIN Taxonomy Match (Jan 2022)|BOLD:ACU4855
Chironomidae|ZSM-DIP-33143-F06|Caroline Chimeno|BIN Taxonomy Match (Jan 2022)|BOLD:ACU4855
Chironomidae|ZSM-DIP-33142-E07|Caroline Chimeno|BIN Taxonomy Match (Jan 2022)|BOLD:ACU4855
Chironomidae|CCDB24228-H02|Jerome Moriniere|BIN Taxonomy Match|BOLD:ACU4855
Chironomidae|CCDB24228-H01|Jerome Moriniere|BIN Taxonomy Match|BOLD:ACU4855
Chironomidae|ZSM-DIP-33131-B03|Caroline Chimeno|BIN Taxonomy Match (Jan 2022)|BOLD:ACU4855
Chironomidae|ZSM-DIP-33131-B01|Caroline Chimeno|BIN Taxonomy Match (Jan 2022)|BOLD:ACU4855
Chironomidae|ZSM-DIP-33132-E04|Caroline Chimeno|BIN Taxonomy Match (Jan 2022)|BOLD:ACU4855
Chironomidae|ZSM-DIP-33132-E02|Caroline Chimeno|BIN Taxonomy Match (Jan 2022)|BOLD:ACU4855
Chironomidae|ZSM-DIP-33132-E01|Caroline Chimeno|BIN Taxonomy Match (Jan 2022)|BOLD:ACU4855
Chironomidae|ZSM-DIP-33132-D11|Caroline Chimeno|BIN Taxonomy Match (Jan 2022)|BOLD:ACU4855
Chironomidae|ZSM-DIP-33132-C03|Caroline Chimeno|BIN Taxonomy Match (Jan 2022)|BOLD:ACU4855
Chironomidae|ZSM-DIP-33132-C02|Caroline Chimeno|BIN Taxonomy Match (Jan 2022)|BOLD:ACU4855
Chironomidae|ZSM-DIP-33131-B06|Caroline Chimeno|BIN Taxonomy Match (Jan 2022)|BOLD:ACU4855
Chironomidae|ZSM-DIP-33131-B05|Caroline Chimeno|BIN Taxonomy Match (Jan 2022)|BOLD:ACU4855
Chironomidae|ZSM-DIP-33132-E05|Caroline Chimeno|BIN Taxonomy Match (Jan 2022)|BOLD:ACU4855
Acricotopus lucens|NHRS-BYWS000000411|Yngve Brodin|morphology|BOLD:ACK6975
Acricotopus lucens|NHRS-BYWS000000299|Yngve Brodin|morphology|BOLD:ACK6975
Acricotopus lucens|NHRS-BYWS000000305|Yngve Brodin|morphology|BOLD:ACK6975
Acricotopus lucens|NHRS-BYWS000001055|Yngve Brodin|morphology|BOLD:ACK6975
Acricotopus lucens|NHRS-BYWS000000290|Yngve Brodin|morphology|BOLD:ACK6975
Acricotopus lucens|NHRS-BYWS000000392|Yngve Brodin|morphology|BOLD:ACK6975
Acricotopus lucens|ZMUO.025199|Lauri Paasivirta||BOLD:ACK6975
Acricotopus lucens|ZMUO.025943|Lauri Paasivirta||BOLD:ACK6975
Acricotopus lucens|ZMUO.025942|Lauri Paasivirta||BOLD:ACK6975
Acricotopus lucens|ZMUO.025198|Lauri Paasivirta||BOLD:ACK6975
Acricotopus lucens|RIN_CH42|Elisabeth Stur|BOLD Identification engine|BOLD:ACK6975
Acricotopus lucens|RIN_CH43|Elisabeth Stur|BOLD Identification engine|BOLD:ACK6975
Acricotopus lucens|TRD-CH44|Elisabeth Stur|Morphology|BOLD:ACK6975
Acricotopus lucens|RIN_CH40|Elisabeth Stur|BOLD Identification engine|BOLD:ACK6975
Pseudorthocladius|CH-OSF183|Elisabeth Stur||BOLD:AAV2836
Pseudorthocladius curtistylus|24467-D11|Kate Perez|BIN Taxonomy Match|BOLD:AAF7013
Pseudorthocladius curtistylus|24530-D12|Kate Perez|BIN Taxonomy Match|BOLD:AAF7013
Pseudorthocladius curtistylus|24489-H02|Kate Perez|BIN Taxonomy Match|BOLD:AAF7013
Pseudorthocladius curtistylus|24376-C05|Kate Perez|BIN Taxonomy Match|BOLD:AAF7013
Pseudorthocladius curtistylus|24469-G04|Kate Perez|BIN Taxonomy Match|BOLD:AAF7013
Pseudorthocladius curtistylus|24536-E12|Kate Perez|BIN Taxonomy Match|BOLD:AAF7013
Pseudorthocladius curtistylus|ATNA274|Elisabeth Stur||BOLD:AAF7013
Pseudorthocladius curtistylus|ATNA236|Elisabeth Stur||BOLD:AAF7013
Pseudorthocladius curtistylus|24468-B11|Kate Perez|BIN Taxonomy Match|BOLD:AAF7013
Pseudorthocladius curtistylus|24354-B01|Kate Perez|BIN Taxonomy Match|BOLD:AAF7013
Pseudorthocladius curtistylus|23878-G02|Kate Perez|BIN Taxonomy Match|BOLD:AAF7013
Pseudorthocladius curtistylus|24354-H04|Kate Perez|BIN Taxonomy Match|BOLD:AAF7013
Pseudorthocladius curtistylus|24354-H10|Kate Perez|BIN Taxonomy Match|BOLD:AAF7013
Pseudorthocladius curtistylus|24465-D10|Kate Perez|BIN Taxonomy Match|BOLD:AAF7013
Pseudorthocladius curtistylus|23976-D09|Kate Perez|BIN Taxonomy Match|BOLD:AAF7013
Pseudorthocladius curtistylus|24371-F08|Kate Perez|BIN Taxonomy Match|BOLD:AAF7013
Pseudorthocladius curtistylus|24406-C12|Kate Perez|BIN Taxonomy Match|BOLD:AAF7013
Pseudorthocladius curtistylus|24505-F05|Kate Perez|BIN Taxonomy Match|BOLD:AAF7013
Pseudorthocladius curtistylus|24468-F10|Kate Perez|BIN Taxonomy Match|BOLD:AAF7013
Pseudorthocladius curtistylus|24538-A08|Kate Perez|BIN Taxonomy Match|BOLD:AAF7013
Pseudorthocladius curtistylus|ATNA432|Elisabeth Stur||BOLD:AAF7013
Pseudorthocladius curtistylus|ATNA285|Elisabeth Stur||BOLD:AAF7013
Pseudorthocladius curtistylus|Finnmark337|Elisabeth Stur||BOLD:AAF7013
Paraphaenocladius impensus|ZSM-DIP-33138-G06|Caroline Chimeno|BIN Taxonomy Match (Jan 2022)...
Chironomidae|CCDB21605-C07|Sofia Wiedenbrug|no morphological identification - introduction of int...
Paraphaenocladius impensus|SOE329|Elisabeth Stur||BOLD:AAC4199
Paraphaenocladius impensus|SOE196|Elisabeth Stur||BOLD:AAC4199
Paraphaenocladius impensus|24373-D11|Kate Perez|BIN Taxonomy Match|BOLD:AAQ2297
Paraphaenocladius impensus|24354-D11|Kate Perez|BIN Taxonomy Match|BOLD:AAQ2297
Paraphaenocladius impensus|24465-E10|Kate Perez|BIN Taxonomy Match|BOLD:AAQ2297
Paraphaenocladius impensus|23975-A12|Kate Perez|BIN Taxonomy Match|BOLD:AAQ2297
Paraphaenocladius impensus|24464-A09|Kate Perez|BIN Taxonomy Match|BOLD:AAQ2297
Paraphaenocladius impensus|ATNA389|Elisabeth Stur||BOLD:AAQ2297
Paraphaenocladius impensus|Finnmark598|Elisabeth Stur||BOLD:AAQ2297
Chironomidae|ZSM-DIP-33149-F10|Valerie Levesque-Beaudin|BIN Taxonomy Match (Sep 2021)|BOLD...
Chironomidae|ZSM-DIP-33149-B12|Valerie Levesque-Beaudin|BOLD ID Engine Manual (Sep 2021)|BO...
Heterotrissocladius sp. 1SW|CCDB22605-A12|Sofia Wiedenbrug||BOLD:ACT8639
Heterotrissocladius sp. 1SW|ZSM-DIP-33132-G10|Caroline Chimeno|BIN Taxonomy Match (Jan 2022 ...
Heterotrissocladius zierli|CCDB21606-F11|Sofia Wiedenbrug||BOLD:AAX0835
Heterotrissocladius zierli|ES09|Elisabeth Stur||BOLD:AAX0835
Paratrissocladius excerptus|CCDB22605-G12|Sofia Wiedenbrug||BOLD:ACR9184
Paratrissocladius excerptus|CCDB22605-G11|Sofia Wiedenbrug||BOLD:ACR9184
Paratrissocladius excerptus|CCDB22604-E10|Sofia Wiedenbrug||BOLD:ACR9184
Paratrissocladius excerptus|CCDB22604-E09|Sofia Wiedenbrug||BOLD:ACR9184
Paratrissocladius excerptus|ZSM-DIP-33146-A03|Caroline Chimeno|BIN Taxonomy Match (Jan 2022 ...
Paratrissocladius excerptus|ZSM-DIP-33142-B06|Caroline Chimeno|BIN Taxonomy Match (Jan 2022 ...
Chironomidae|BIOUG15071-G06|Kate Perez|BOLD ID Engine Manual|BOLD:ACP2523
Heterotrissocladius subpilosus|TRD-CH159|Elisabeth Stur||BOLD:ACK3624
Heterotrissocladius subpilosus|TRD-CH131|Elisabeth Stur||BOLD:ACK3624
Heterotrissocladius subpilosus|ZMUO.024533|Lauri Paasivirta||BOLD:ACK3624
Heterotrissocladius subpilosus|ZMUO.024532|Lauri Paasivirta||BOLD:ACK3624
Heterotrissocladius subpilosus|TRD-CH160|Elisabeth Stur||BOLD:ACK3624
Heterotrissocladius subpilosus|TRD-CH3|Elisabeth Stur||BOLD:ACK3624
Heterotrissocladius subpilosus|TRD-CH93|Elisabeth Stur||BOLD:ACK3624
Heterotrissocladius marcidus|ZMUO.025933|Lauri Paasivirta||BOLD:ACY4623

Heterotrissocladius subpilosus|TRD-CH3|Elisabeth Stur||BOLD:ACK3624
Heterotrissocladius subpilosus|TRD-CH93|Elisabeth Stur||BOLD:ACK3624
Heterotrissocladius marcidus|ZMUO.025933|Lauri Paasivirta||BOLD:ACY4623
Heterotrissocladius marcidus|ZMUO.025932|Lauri Paasivirta||BOLD:ACY4623
Heterotrissocladius marcidus|CCDB22603-H03|Sofia Wiedenbrug||BOLD:ACQ0651
Heterotrissocladius marcidus|CCDB22603-H01|Sofia Wiedenbrug||BOLD:ACQ0651
Heterotrissocladius marcidus|CCDB22603-F05|Sofia Wiedenbrug||BOLD:ACQ0651
Heterotrissocladius marcidus|CCDB22603-F12|Sofia Wiedenbrug||BOLD:ACQ0651
Heterotrissocladius marcidus|CCDB22603-H02|Sofia Wiedenbrug||BOLD:ACQ0651
Heterotrissocladius marcidus|CCDB22603-F01|Sofia Wiedenbrug||BOLD:ACQ0651
Heterotrissocladius marcidus|CCDB22603-D11|Sofia Wiedenbrug||BOLD:ACQ0651
Heterotrissocladius marcidus|CCDB22603-D12|Sofia Wiedenbrug||BOLD:ACQ0651
Heterotrissocladius marcidus|ZMUO.024311|Lauri Paasivirta||BOLD:AAF2163
Heterotrissocladius marcidus|BIOUG04217-H08|Kate Perez|BIN Taxonomy Match|BOLD:AAF2163
Heterotrissocladius marcidus|BIOUG04217-E04|Kate Perez|BIN Taxonomy Match|BOLD:AAF2163
Heterotrissocladius|ZSM-DIP-33128-F03|Caroline Chimeno|BIN Taxonomy Match (Jan 2022)|BOLD ...
Heterotrissocladius|ZSM-DIP-33172-G10|Caroline Chimeno|BIN Taxonomy Match (Jan 2022)|BOLD ...
Heterotrissocladius|ZSM-DIP-33172-G09|Caroline Chimeno|BIN Taxonomy Match (Jan 2022)|BOLD ...
Heterotrissocladius|ZSM-DIP-33140-B12|Caroline Chimeno|BIN Taxonomy Match (Jan 2022)|BOLD ...
Heterotrissocladius|ZSM-DIP-33136-E02|Caroline Chimeno|BIN Taxonomy Match (Jan 2022)|BOLD ...
Heterotrissocladius|ZSM-DIP-33136-B05|Caroline Chimeno|BIN Taxonomy Match (Jan 2022)|BOLD ...
Heterotrissocladius|ZSM-DIP-33136-B08|Caroline Chimeno|BIN Taxonomy Match (Jan 2022)|BOLD ...
Heterotrissocladius|ZSM-DIP-33135-E12|Caroline Chimeno|BIN Taxonomy Match (Jan 2022)|BOLD ...
Heterotrissocladius|ZSM-DIP-33129-F11|Caroline Chimeno|BIN Taxonomy Match (Jan 2022)|BOLD ...
Heterotrissocladius|BIOUG36678-A08|Kate Perez|BIN Taxonomy Match (Jul 2018)|BOLD:AAF2163
Heterotrissocladius marcidus|BIOUG16432-H02|Kate Perez|BIN Taxonomy Match|BOLD:AAF2163
Heterotrissocladius marcidus|BIOUG15456-A02|Kate Perez|BIN Taxonomy Match|BOLD:AAF2163
Heterotrissocladius marcidus|Finnmark208|Elisabeth Stur||BOLD:AAF2163
Heterotrissocladius marcidus|Finnmark22|Elisabeth Stur||BOLD:AAF2163
Heterotrissocladius marcidus|Finnmark701|Elisabeth Stur||BOLD:AAF2163
Heterotrissocladius marcidus|BIOUG15455-B10|Kate Perez|BIN Taxonomy Match|BOLD:AAF2163
Chironomidae|BIOUG05143-F03|Kate Perez|no morphological identification - introduction of interim ...
Heterotrissocladius marcidus|CH-OSF21|Elisabeth Stur||BOLD:AAF2163
Heterotrissocladius|BIOUG36678-C03|Kate Perez|BIN Taxonomy Match (Jul 2018)|BOLD:AAF2163
Heterotrissocladius marcidus|Finnmark54|Elisabeth Stur||BOLD:AAF2163
Heterotrissocladius marcidus|Finnmark198|Elisabeth Stur||BOLD:AAF2163
Heterotrissocladius marcidus|TRD-CH4|Elisabeth Stur||BOLD:AAF2163
Heterotrissocladius marcidus|ATNA454|Elisabeth Stur||BOLD:AAF2163
Heterotrissocladius marcidus|ATNA455|Elisabeth Stur||BOLD:AAF2163
Heterotrissocladius marcidus|ATNA197|Elisabeth Stur||BOLD:AAF2163
Heterotrissocladius marcidus|TRD-CH371|Elisabeth Stur|morphology|BOLD:AAF2163
Heterotrissocladius grimshawi|NHRS-BYWS000001155|Yngve Brodin|morphology|BOLD:AAF2163
Heterotrissocladius|BIOUG43948-D03|Kate Perez|BIN Taxonomy Match below Phylum (Jan 2019)|BO...
Heterotrissocladius marcidus|ZMUO.024312|Lauri Paasivirta||BOLD:AAF2163
Heterotrissocladius marcidus|BIOUG17022-H03|Kate Perez|BIN Taxonomy Match|BOLD:AAF2163
Heterotrissocladius marcidus|BIOUG15454-C08|Kate Perez|BIN Taxonomy Match|BOLD:AAF2163
Heterotrissocladius marcidus|BIOUG15185-C08|Kate Perez|BIN Taxonomy Match|BOLD:AAF2163
Heterotrissocladius marcidus|BIOUG15455-D12|Kate Perez|BIN Taxonomy Match|BOLD:AAF2163
Heterotrissocladius marcidus|BIOUG16431-E04|Kate Perez|BIN Taxonomy Match|BOLD:AAF2163
Heterotrissocladius marcidus|BIOUG15459-F07|Kate Perez|BIN Taxonomy Match|BOLD:AAF2163
Heterotrissocladius marcidus|BIOUG16431-D03|Kate Perez|BIN Taxonomy Match|BOLD:AAF2163
Heterotrissocladius marcidus|BIOUG15183-G05|Kate Perez|BIN Taxonomy Match|BOLD:AAF2163
Heterotrissocladius marcidus|BIOUG15464-D03|Kate Perez|BIN Taxonomy Match|BOLD:AAF2163
Heterotrissocladius marcidus|Finnmark497|Elisabeth Stur||BOLD:AAF2163
Heterotrissocladius|ZSM-DIP-33130-F09|Caroline Chimeno|BIN Taxonomy Match (Jan 2022)|BOLD ...
Heterotrissocladius grimshawi|ZMUO.024314|Lauri Paasivirta||BOLD:AAI8458
Heterotrissocladius grimshawi|Finnmark55|Elisabeth Stur||BOLD:AAI8458
Heterotrissocladius grimshawi|ZMUO.024313|Lauri Paasivirta||BOLD:AAI8458
Heterotrissocladius grimshawi|Finnmark796|Elisabeth Stur||BOLD:AAI8458
Heterotrissocladius brundini|NHRS-BYWS000001171|Yngve Brodin|morphology|BOLD:AAI8458
Heterotrissocladius grimshawi|Finnmark533|Elisabeth Stur||BOLD:AAI8458
Heterotrissocladius grimshawi|Finnmark531|Elisabeth Stur||BOLD:AAI8458
Heterotrissocladius grimshawi|Finnmark403|Elisabeth Stur||BOLD:AAI8458
Heterotrissocladius grimshawi|Finnmark399|Elisabeth Stur||BOLD:AAI8458
Heterotrissocladius grimshawi|Finnmark381|Elisabeth Stur||BOLD:AAI8458
Heterotrissocladius grimshawi|Finnmark648|Elisabeth Stur||BOLD:AAI8458
Paraphaenocladius impensus|ZSM-DIP-33145-A03|Caroline Chimeno|BIN Taxonomy Match (Jan 2022)...
Paraphaenocladius impensus|TRD-CH392|Elisabeth Stur|morphology|BOLD:ACN9514
Paraphaenocladius impensus|TRD-CH295|Elisabeth Stur||BOLD:ACN9514
Paraphaenocladius impensus|ZSM-DIP-33144-A04|Caroline Chimeno|BIN Taxonomy Match (Jan 2022)...
Paraphaenocladius impensus|BIOUG16476-F06|Kate Perez|BIN Taxonomy Match|BOLD:ACN9514
Chironomidae|CCDB22605-E05|Sofia Wiedenbrug|no morphological identification - introduction of int...
Paraphaenocladius impensus|BIOUG16430-G11|Kate Perez|BIN Taxonomy Match|BOLD:ACN9514
Paraphaenocladius impensus|ZSM-DIP-33147-G04|Caroline Chimeno|BIN Taxonomy Match (Jan 2022)...
Paraphaenocladius impensus|ZSM-DIP-33147-G05|Caroline Chimeno|BIN Taxonomy Match (Jan 2022)...
Paraphaenocladius impensus|NHRS-BYWS000001089|Yngve Brodin|morphology|BOLD:ACN9514
Paraphaenocladius impensus|NHRS-BYWS000001150|Yngve Brodin|morphology|BOLD:ACN9514
Paraphaenocladius impensus|ZSM-DIP-33138-G11|Caroline Chimeno|BIN Taxonomy Match (Jan 2022)...
Paraphaenocladius impensus|NHRS-BYWS000001135|Yngve Brodin|morphology|BOLD:ACN9514
Paraphaenocladius impensus|CH-eik97|Elisabeth Stur||BOLD:ACN9514
Paraphaenocladius impensus|ZMUO.024644|Lauri Paasivirta||BOLD:ACN9514
Paraphaenocladius impensus|NHRS-BYWS000001090|Yngve Brodin|morphology|BOLD:ACN9514
Paraphaenocladius impensus|ZSM-DIP-33139-A09|Caroline Chimeno|BIN Taxonomy Match (Jan 2022)...
Paraphaenocladius impensus|ZMUO.024645|Lauri Paasivirta||BOLD:ACN9514
Paraphaenocladius impensus|ZSM-DIP-33147-G07|Caroline Chimeno|BIN Taxonomy Match (Jan 2022)...
Paraphaenocladius impensus|ZSM-DIP-33145-C10|Caroline Chimeno|BIN Taxonomy Match (Jan 2022)...
Paraphaenocladius impensus|NHRS-BYWS000000997|Yngve Brodin|morphology|BOLD:ACN9514
Paraphaenocladius impensus|CH-eik18|Elisabeth Stur||BOLD:ACN9514
Paraphaenocladius impensus|Finnmark584|Elisabeth Stur||BOLD:ACN9514
Paraphaenocladius impensus|ZSM34342-F03|Sofia Wiedenbrug|Morphology|BOLD:ACT5784
Paraphaenocladius impensus|ZSM34343-B03|Sofia Wiedenbrug|BIN-taxonomy|BOLD:ACT5784
Paraphaenocladius impensus|ZSM34342-F06|Sofia Wiedenbrug|Morphology|BOLD:ACT5784
Paraphaenocladius impensus|CCDB24228-A04|Sofia Wiedenbrug|BIN Taxonomy Match|BOLD:ACT5784
Paraphaenocladius impensus|CCDB21606-B01|Sofia Wiedenbrug||BOLD:ACT5784
Paraphaenocladius impensus|CCDB21605-C09|Sofia Wiedenbrug||BOLD:ACT2714
Paraphaenocladius impensus|BC-ZSM-DIP-22551-E05|Dieter Doczkal||BOLD:ACT2714
Paraphaenocladius impensus|CCDB24038-H10|Sofia Wiedenbrug|BIN Taxonomy Match|BOLD:ACT2714
Paraphaenocladius impensus|CCDB24228-G05|Sofia Wiedenbrug|BIN Taxonomy Match|BOLD:ACU4175
Paraphaenocladius impensus|CCDB24039-G10|Sofia Wiedenbrug|BIN Taxonomy Match|BOLD:ACU4175
Paraphaenocladius impensus|CCDB24038-F05|Sofia Wiedenbrug|BIN Taxonomy Match|BOLD:ACU4175
Paraphaenocladius impensus|BC-ZSM-DIP-22551-E03|Sofia Wiedenbrug|BIN-taxonomy|BOLD:ACU4175
Paraphaenocladius impensus|ZSM34341-B12|Sofia Wiedenbrug|Morphology|BOLD:ACU4175
Paraphaenocladius impensus|CCDB24039-F06|Sofia Wiedenbrug|BIN Taxonomy Match|BOLD:ACU4175

Paraphaenocladius impensus|[illegible]|Sofia Wiedenbrug|BIN Taxonomy Match|BOLD:ACU4175
Paraphaenocladius impensus|BC-ZSM-DIP-22551-E03|Sofia Wiedenbrug|BIN-taxonomy|BOLD:ACU4175
Paraphaenocladius impensus|ZSM34341-B12|Sofia Wiedenbrug|Morphology|BOLD:ACU4175
Paraphaenocladius impensus|CCDB24039-E06|Sofia Wiedenbrug|BIN Taxonomy Match|BOLD:ACU4175
Paraphaenocladius impensus|CCDB24229-A04|Sofia Wiedenbrug|BIN Taxonomy Match|BOLD:ACU4175
Paraphaenocladius|ZSM34342-G10|Jerome Moriniere|BIN Taxonomy Match|BOLD:ACU4110
Paraphaenocladius|CCDB24229-G09|Jerome Moriniere|BIN Taxonomy Match|BOLD:ACU4110
Paraphaenocladius|CCDB24037-B07|Jerome Moriniere|BIN Taxonomy Match|BOLD:ACU4110
Paraphaenocladius|CCDB24037-C08|Jerome Moriniere|BIN Taxonomy Match|BOLD:ACU4110
Paraphaenocladius|CCDB24039-E05|Jerome Moriniere|BIN Taxonomy Match|BOLD:ACU4110
Paraphaenocladius|CCDB24039-E04|Jerome Moriniere|BIN Taxonomy Match|BOLD:ACU4110
Paraphaenocladius|CCDB24228-B07|Jerome Moriniere|BIN Taxonomy Match|BOLD:ACU4110
Paraphaenocladius|CCDB24037-C11|Jerome Moriniere|BIN Taxonomy Match|BOLD:ACU4110
Paraphaenocladius|CCDB24229-F08|Jerome Moriniere|BIN Taxonomy Match|BOLD:ACU4110
Paraphaenocladius|CCDB24037-C09|Jerome Moriniere|BIN Taxonomy Match|BOLD:ACU4110
Paraphaenocladius|BC-ZSM-DIP-22491-H07|Dieter Doczkal|BIN Taxonomy Match|BOLD:ACU4110
Paraphaenocladius impensus|SOE449|Torbjorn Ekrem
Paraphaenocladius impensus|ZSM-DIP-33140-D05|Caroline Chimeno|BIN Taxonomy Match (Jan 2022)...
Paraphaenocladius impensus|ZSM-DIP-33140-C07|Caroline Chimeno|BIN Taxonomy Match (Jan 2022)...
Paraphaenocladius impensus|ZSM-DIP-33140-C06|Caroline Chimeno|BIN Taxonomy Match (Jan 2022)...
Paraphaenocladius impensus|ZSM-DIP-33133-H04|Caroline Chimeno|BIN Taxonomy Match (Jan 2022)...
Paraphaenocladius impensus|SOE150|Elisabeth Stur||BOLD:AAC4198
Paraphaenocladius impensus|SOE146|Elisabeth Stur||BOLD:AAC4198
Paraphaenocladius impensus|SOE434|Torbjorn Ekrem||BOLD:AAC4198
Paraphaenocladius impensus|BIOUG16479-C09|Kate Perez|BIN Taxonomy Match|BOLD:AAC4200
Paraphaenocladius impensus|CCDB24038-H11|Sofia Wiedenbrug|BIN Taxonomy Match|BOLD:AAC4200
Paraphaenocladius impensus|CCDB24037-H04|Sofia Wiedenbrug|BIN Taxonomy Match|BOLD:AAC4200
Paraphaenocladius impensus|BIOUG16431-G04|Kate Perez|BIN Taxonomy Match|BOLD:AAC4200
Paraphaenocladius impensus|ZSM-DIP-33143-D08|Caroline Chimeno|BIN Taxonomy Match (Jan 2022)...
Paraphaenocladius impensus|ZSM-DIP-33132-H08|Caroline Chimeno|BIN Taxonomy Match (Jan 2022)...
Paraphaenocladius impensus|ZSM-DIP-33146-G05|Caroline Chimeno|BIN Taxonomy Match (Jan 2022)...
Paraphaenocladius impensus|ZSM-DIP-33141-H02|Caroline Chimeno|BIN Taxonomy Match (Jan 2022)...
Paraphaenocladius impensus|ZSM-DIP-33132-H09|Caroline Chimeno|BIN Taxonomy Match (Jan 2022)...
Paraphaenocladius impensus|ZSM-DIP-33134-D02|Caroline Chimeno|BIN Taxonomy Match (Jan 2022)...
Paraphaenocladius impensus|ZSM-DIP-33136-A08|Caroline Chimeno|BIN Taxonomy Match (Jan 2022)...
Paraphaenocladius impensus|ZSM-DIP-33148-A09|Caroline Chimeno|BIN Taxonomy Match (Jan 2022)...
Paraphaenocladius impensus|ZSM-DIP-33146-G04|Caroline Chimeno|BIN Taxonomy Match (Jan 2022)...
Paraphaenocladius impensus|BIOUG16994-A08|Kate Perez|BIN Taxonomy Match|BOLD:AAC4200
Paraphaenocladius impensus|BIOUG15978-F06|Kate Perez|BIN Taxonomy Match|BOLD:AAC4200
Paraphaenocladius impensus|BIOUG15977-H02|Kate Perez|BIN Taxonomy Match|BOLD:AAC4200
Paraphaenocladius impensus|ZSM-DIP-33141-H04|Caroline Chimeno|BIN Taxonomy Match (Jan 2022)...
Paraphaenocladius impensus|ZSM-DIP-33145-A11|Caroline Chimeno|BIN Taxonomy Match (Jan 2022)...
Paraphaenocladius impensus|SOE392|Torbjorn Ekrem||BOLD:AAC4200
Paraphaenocladius impensus|CCDB24183-H11|Sofia Wiedenbrug|Morphology|BOLD:AAC4200
Paraphaenocladius impensus|BIOUG55199-C10|Kate Perez|BIN Taxonomy Match (Oct 2020)|BOLD:...
Paraphaenocladius impensus|BIOUG55194-E05|Kate Perez|BIN Taxonomy Match (Oct 2020)|BOLD:...
Paraphaenocladius impensus|BIOUG15977-A03|Kate Perez|BIN Taxonomy Match|BOLD:AAC4200
Paraphaenocladius impensus|CH-OSF133|Elisabeth Stur||BOLD:AAC4200
Paraphaenocladius impensus|CH-OSF112|Elisabeth Stur||BOLD:AAC4200
Paraphaenocladius impensus|BIOUG55189-F01|Kate Perez|BIN Taxonomy Match (Oct 2020)|BOLD:...
Paraphaenocladius impensus|BIOUG16900-A05|Kate Perez|BIN Taxonomy Match|BOLD:AAC4200
Paraphaenocladius impensus|BIOUG17265-A02|Kate Perez|BIN Taxonomy Match|BOLD:AAC4200
Paraphaenocladius impensus|BIOUG16733-F11|Kate Perez|BIN Taxonomy Match|BOLD:AAC4200
Paraphaenocladius impensus|ZSM-DIP-33138-G10|Caroline Chimeno|BIN Taxonomy Match (Jan 2022)...
Paraphaenocladius impensus|ZSM-DIP-33170-G12|Caroline Chimeno|BIN Taxonomy Match (Jan 2022)...
Paraphaenocladius impensus|ZSM-DIP-33138-G05|Caroline Chimeno|BIN Taxonomy Match (Jan 2022)...
Paraphaenocladius impensus|ZSM-DIP-33136-E07|Caroline Chimeno|BIN Taxonomy Match (Jan 2022)...
Paraphaenocladius impensus|ZSM-DIP-33128-E12|Caroline Chimeno|BIN Taxonomy Match (Jan 2022)...
Paraphaenocladius impensus|ZSM-DIP-33170-H04|Caroline Chimeno|BIN Taxonomy Match (Jan 2022)...
Paraphaenocladius impensus|BIOUG16620-F09|Kate Perez|BIN Taxonomy Match|BOLD:AAC4200
Paraphaenocladius impensus|CH-OSF54|Elisabeth Stur||BOLD:AAC4200
Paraphaenocladius impensus|BIOUG16526-D04|Kate Perez|BIN Taxonomy Match|BOLD:AAC4200
Paraphaenocladius|24468-E07|Kate Perez|BIN Taxonomy Match|BOLD:ACF8768
Paraphaenocladius|24378-F09|Kate Perez|BIN Taxonomy Match|BOLD:ACF8768
Paraphaenocladius|24405-E11|Kate Perez|BIN Taxonomy Match|BOLD:ACF8768
Paraphaenocladius|24372-B04|Kate Perez|BIN Taxonomy Match|BOLD:ACF8768
Paraphaenocladius|24474-E07|Kate Perez|BIN Taxonomy Match|BOLD:ACF8768
Paraphaenocladius|BIOUG05219-A08|Kate Perez|BIN Taxonomy Match|BOLD:ACF8768
Chironomidae|BIOUG04366-G11|Kate Perez|BIN Taxonomy Match|BOLD:ACC6402
Paraphaenocladius sp. 4ES|Finnmark369|Elisabeth Stur||BOLD:AAL7381
Paraphaenocladius|24406-E10|Kate Perez|BIN Taxonomy Match|BOLD:AAU6742
Paraphaenocladius|24466-A02|Kate Perez|BIN Taxonomy Match|BOLD:AAU6742
Paraphaenocladius|24404-B02|Kate Perez|BIN Taxonomy Match|BOLD:AAU6742
Paraphaenocladius|24538-A03|Kate Perez|BIN Taxonomy Match|BOLD:AAU6742
Paraphaenocladius impensus|24465-G02|Kate Perez|BIN Taxonomy Match|BOLD:AAC4201
Paraphaenocladius impensus|24371-E03|Kate Perez|BIN Taxonomy Match|BOLD:AAC4201
Paraphaenocladius impensus|ATNA295|Elisabeth Stur||BOLD:AAC4201
Paraphaenocladius impensus|Finnmark334|Elisabeth Stur||BOLD:AAC4201
Parachaetocladius abnobaeus|ZMUO.025952|Lauri Paasivirta||BOLD:ACF6302
Parachaetocladius abnobaeus|BIOUG05143-D05|Kate Perez|BIN Taxonomy Match|BOLD:ACF6302
Chaetocladius dissipatus|SOE133|Elisabeth Stur||BOLD:AAE1188
Chaetocladius dissipatus|ATNA356|Elisabeth Stur||BOLD:AAE1188
Chaetocladius dissipatus|ATNA302|Elisabeth Stur||BOLD:AAE1188
Chaetocladius dissipatus|ATNA230|Elisabeth Stur||BOLD:AAE1188
Chaetocladius dissipatus|ATNA510|Elisabeth Stur||BOLD:AAE1188
Chaetocladius dissipatus|ATNA472|Elisabeth Stur||BOLD:AAE1188
Prodiamesa olivacea|ZMUO.025891|Lauri Paasivirta||BOLD:AAD7458
Prodiamesa olivacea|ZMUO.025890|Lauri Paasivirta||BOLD:AAD7458
Prodiamesa olivacea|ZSM-DIP-33132-F02|Caroline Chimeno|BIN Taxonomy Match (Jan 2022)|BOLD ...
Prodiamesa olivacea|ZMUO.024967|Lauri Paasivirta||BOLD:AAD7458
Prodiamesa olivacea|ZMUO.024966|Lauri Paasivirta||BOLD:AAD7458
Prodiamesa olivacea|BIOUG16710-G08|Kate Perez|BIN Taxonomy Match|BOLD:AAD7458
Prodiamesa olivacea|CCDB21605-G01|Sofia Wiedenbrug||BOLD:AAD7458
Prodiamesa olivacea|BIOUG16627-A08|Kate Perez|BIN Taxonomy Match|BOLD:AAD7458
Prodiamesa olivacea|BIOUG05146-B09|Kate Perez|BIN Taxonomy Match|BOLD:AAD7458
Prodiamesa olivacea|SOE72|Elisabeth Stur||BOLD:AAD7458
Prodiamesa olivacea|SOE165|Elisabeth Stur||BOLD:AAD7458
Prodiamesa olivacea|NO08|Elisabeth Stur||BOLD:AAD7458
Prodiamesa olivacea|ZFMK-TIS-2534199|||BOLD:AAD7458
Prodiamesa olivacea|ZFMK-TIS-2534191|||BOLD:AAD7458
Prodiamesa olivacea|ZFMK-TIS-2534187|||BOLD:AAD7458
Prodiamesa olivacea|ZFMK-TIS-2534158|||BOLD:AAD7458
Prodiamesa olivacea|SOE73|Elisabeth Stur||BOLD:AAD7458
Prodiamesa olivacea|TRD-CH216|Elisabeth Stur||BOLD:AAD7458

Prodiamesa olivacea|ZFMK-TIS-2534187|||BOLD:AAD7458
Prodiamesa olivacea|ZFMK-TIS-2534158|||BOLD:AAD7458
Prodiamesa olivacea|SOE73|Elisabeth Stur||BOLD:AAD7458
Prodiamesa olivacea|TRD-CH316|Elisabeth Stur||BOLD:AAD7458
Prodiamesa olivacea|TRD-CH265|Elisabeth Stur||BOLD:AAD7458
Prodiamesa olivacea|BIOUG15855-B03|Kate Perez|BIN Taxonomy Match|BOLD:AAD7458
Prodiamesa olivacea|NO07|Elisabeth Stur||BOLD:AAD7458
Prodiamesa olivacea|BIOUG17022-D12|Kate Perez|BIN Taxonomy Match|BOLD:AAD7458
Prodiamesa olivacea|BIOUG16626-B08|Kate Perez|BIN Taxonomy Match|BOLD:AAD7458
Prodiamesa olivacea|PK-192-70|Petra Kranzfelder|morphology|BOLD:AAD7458
Prodiamesa olivacea|BIOUG17064-A08|Kate Perez|BIN Taxonomy Match|BOLD:AAD7458
Prodiamesa olivacea|PK-192-62|Petra Kranzfelder|morphology|BOLD:AAD7458
Prodiamesa olivacea|BIOUG16477-E03|Kate Perez|BIN Taxonomy Match|BOLD:AAD7458
Prodiamesa olivacea|BIOUG16296-A01|Kate Perez|BIN Taxonomy Match|BOLD:AAD7458
Prodiamesa olivacea|BIOUG15594-F11|Kate Perez|BIN Taxonomy Match|BOLD:AAD7458
Prodiamesa olivacea|TRD-CH111|Elisabeth Stur||BOLD:AAD7458
Prodiamesa olivacea|SOE69|Elisabeth Stur||BOLD:AAD7458
Prodiamesa olivacea|TRD-CH166|Elisabeth Stur||BOLD:AAD7458
Prodiamesa olivacea|TRD-CH283|Elisabeth Stur||BOLD:AAD7458
Prodiamesa olivacea|BIOUG16626-G12|Kate Perez|BIN Taxonomy Match|BOLD:AAD7458
Prodiamesa olivacea|NO48|Elisabeth Stur||BOLD:AAD7458
Orthocladiinae|CCDB24037-E04|Sofia Wiedenbrug|BIN Taxonomy Match|BOLD:AAF7012
Pseudorthocladius curtistylus|CCDB22603-G07|Sofia Wiedenbrug||BOLD:ACQ3153
Pseudorthocladius curtistylus|ES40|Elisabeth Stur||BOLD:AAF7012
Parametriocnemus boreoalpinus|EBAI-Ch267|Elisabeth Stur|morphology|BOLD:AAL7338
Parametriocnemus boreoalpinus|ATNA494|Elisabeth Stur||BOLD:AAL7338
Parametriocnemus boreoalpinus|Finnmark890|Elisabeth Stur||BOLD:AAL7338
Parametriocnemus boreoalpinus|ATNA555|Elisabeth Stur||BOLD:AAL7338
Parametriocnemus boreoalpinus|EBAI-Ch238|Elisabeth Stur|morphology|BOLD:AAL7338
Parametriocnemus boreoalpinus|Finnmark597|Elisabeth Stur||BOLD:AAL7338
Pseudorthocladius pilosipennis|BIOUG05217-H10|Kate Perez|BIN Taxonomy Match|BOLD:AAL1600
Pseudorthocladius pilosipennis|BIOUG05207-B05|Kate Perez|BIN Taxonomy Match|BOLD:AAL1600
Pseudorthocladius pilosipennis|BIOUG05217-H11|Kate Perez|BIN Taxonomy Match|BOLD:AAL1600
Pseudorthocladius pilosipennis|CCDB24183-A03|Sofia Wiedenbrug|Morphology|BOLD:AAL1600
Pseudorthocladius pilosipennis|ZMUO.024968|Lauri Paasivirta||BOLD:AAL1600
Pseudorthocladius pilosipennis|ZMUO.026352|Lauri Paasivirta||BOLD:AAL1600
Pseudorthocladius pilosipennis|24416-B02|Kate Perez|BIN Taxonomy Match|BOLD:AAL1600
Pseudorthocladius pilosipennis|ZMUO.026353|Lauri Paasivirta||BOLD:AAL1600
Pseudorthocladius pilosipennis|24528-E11|Kate Perez|BIN Taxonomy Match|BOLD:AAL1600
Pseudorthocladius pilosipennis|ATNA357|Elisabeth Stur||BOLD:AAL1600
Pseudorthocladius pilosipennis|BIOUG05215-E06|Kate Perez|BIN Taxonomy Match|BOLD:AAL1600
Pseudorthocladius pilosipennis|BIOUG05220-H10|Kate Perez|BIN Taxonomy Match|BOLD:AAL1600
Rheocricotopus reduncus|ZMUO.025974|Lauri Paasivirta||BOLD:AAM7075
Rheocricotopus reduncus|Finnmark89|Elisabeth Stur||BOLD:AAM7075
Paraphaenocladius ACQ5239|CCDB22603-H07|Sofia Wiedenbrug||BOLD:ACQ5239
Paraphaenocladius ACQ5239|CCDB22603-A06|Sofia Wiedenbrug||BOLD:ACQ5239
Paraphaenocladius exagitans|CCDB21606-D01|Sofia Wiedenbrug||BOLD:ACT8523
Paraphaenocladius exagitans|CCDB21606-D02|Sofia Wiedenbrug||BOLD:ACT8523
Paraphaenocladius exagitans|CCDB24228-D05|Sofia Wiedenbrug|BIN Taxonomy Match|BOLD:ACT8523
Paraphaenocladius exagitans|CCDB21606-A10|Sofia Wiedenbrug||BOLD:ACT8523
Paraphaenocladius exagitans|CCDB24228-A03|Sofia Wiedenbrug|BIN Taxonomy Match|BOLD:ACT8523
Paraphaenocladius exagitans|CCDB24228-B08|Sofia Wiedenbrug|BIN Taxonomy Match|BOLD:ACT8523
Paraphaenocladius exagitans|CCDB24228-E12|Sofia Wiedenbrug|BIN Taxonomy Match|BOLD:ACT8523
Paraphaenocladius exagitans|CCDB24037-C10|Sofia Wiedenbrug|BIN Taxonomy Match|BOLD:ACT8523
Paraphaenocladius exagitans|ZSM34342-G07|Sofia Wiedenbrug|Morphology|BOLD:ACT8523
Paraphaenocladius exagitans|BC-ZSM-DIP-22488-H10|Dieter Doczkal||BOLD:ACT8523
Paraphaenocladius exagitans|CCDB24038-G06|Sofia Wiedenbrug|BIN Taxonomy Match|BOLD:ACT8523
Paraphaenocladius exagitans|BC-ZSM-DIP-22491-E05|Dieter Doczkal||BOLD:ACT8523
Paraphaenocladius|CCDB24038-F04|Sofia Wiedenbrug|BIN Taxonomy Match|BOLD:ACX1756
Paraphaenocladius|BC-ZSM-DIP-22491-D04|Dieter Doczkal||BOLD:ACX1756
Paraphaenocladius exagitans|BIOUG05215-C12|Kate Perez|BIN Taxonomy Match|BOLD:AAE3719
Paraphaenocladius exagitans|CCDB24183-B04|Sofia Wiedenbrug|Morphology|BOLD:AAE3719
Paraphaenocladius exagitans|CH-OSF132|Elisabeth Stur||BOLD:AAE3719
Paraphaenocladius exagitans|CH-OSF57|Elisabeth Stur||BOLD:AAE3719
Paraphaenocladius exagitans|ZMUO.024841|Lauri Paasivirta||BOLD:ACQ4724
Paraphaenocladius exagitans|BIOUG05216-A04|Kate Perez|BIN Taxonomy Match|BOLD:ACQ4724
Paraphaenocladius exagitans|BIOUG05207-E07|Kate Perez|BIN Taxonomy Match|BOLD:ACQ4724
Paraphaenocladius exagitans|BIOUG05141-H02|Kate Perez|BIN Taxonomy Match|BOLD:ACQ4724
Paraphaenocladius exagitans|SOE57|Elisabeth Stur||BOLD:ACQ4724
Paraphaenocladius exagitans|ATNA296|Elisabeth Stur||BOLD:ACQ4724
Paraphaenocladius exagitans|ZSM-DIP-33140-C11|Caroline Chimeno|BIN Taxonomy Match (Jan 2022 ...
Paraphaenocladius exagitans|ZSM-DIP-33140-D08|Caroline Chimeno|BIN Taxonomy Match (Jan 2022 ...
Paraphaenocladius exagitans|ZMUO.024842|Lauri Paasivirta||BOLD:ACQ4724
Paraphaenocladius exagitans|ZSM-DIP-33131-G04|Caroline Chimeno|BIN Taxonomy Match (Jan 2022 ...
Paraphaenocladius exagitans|ZSM-DIP-33131-G03|Caroline Chimeno|BIN Taxonomy Match (Jan 2022 ...
Paraphaenocladius exagitans|ZSM-DIP-33131-G09|Caroline Chimeno|BIN Taxonomy Match (Jan 2022 ...
Paraphaenocladius exagitans|ZSM-DIP-33137-B11|Caroline Chimeno|BIN Taxonomy Match (Jan 2022 ...
Paraphaenocladius exagitans|ZSM-DIP-33137-B12|Caroline Chimeno|BIN Taxonomy Match (Jan 2022 ...
Paraphaenocladius exagitans|ZSM-DIP-33139-D10|Caroline Chimeno|BIN Taxonomy Match (Jan 2022 ...
Paraphaenocladius exagitans|ZSM-DIP-33139-E02|Caroline Chimeno|BIN Taxonomy Match (Jan 2022 ...
Paraphaenocladius exagitans|ZSM-DIP-33139-E03|Caroline Chimeno|BIN Taxonomy Match (Jan 2022 ...
Paraphaenocladius exagitans|ZSM-DIP-33139-E04|Caroline Chimeno|BIN Taxonomy Match (Jan 2022 ...
Paraphaenocladius exagitans|ZSM-DIP-33139-E05|Caroline Chimeno|BIN Taxonomy Match (Jan 2022 ...
Paraphaenocladius exagitans|ZSM-DIP-33139-E06|Caroline Chimeno|BIN Taxonomy Match (Jan 2022 ...
Paraphaenocladius exagitans|ZSM-DIP-33139-E07|Caroline Chimeno|BIN Taxonomy Match (Jan 2022 ...
Paraphaenocladius exagitans|ZSM-DIP-33140-C10|Caroline Chimeno|BIN Taxonomy Match (Jan 2022 ...
Paraphaenocladius exagitans|ZSM-DIP-33131-G08|Caroline Chimeno|BIN Taxonomy Match (Jan 2022 ...
Paraphaenocladius exagitans|SOE322|Torbjorn Ekrem||BOLD:ACQ4724
Paraphaenocladius exagitans|ZSM-DIP-33134-E03|Caroline Chimeno|BIN Taxonomy Match (Jan 2022 ...
Paraphaenocladius exagitans|NO24|Elisabeth Stur||BOLD:ACQ4724
Paraphaenocladius exagitans|SOE140|Elisabeth Stur||BOLD:ACQ4724
Paraphaenocladius exagitans|BIOUG05106-B07|Kate Perez|BIN Taxonomy Match|BOLD:ACQ4724
Heterotrissocladius marcidus|ZMUO.029823|Marko Mutanen||BOLD:ADI3620
Heterotrissocladius sp. 2SW|GBOL11554|Sofia Wiedenbrug|bin taxonomy|BOLD:ACT0982
Heterotrissocladius sp. 2SW|CCDB21605-G03|Sofia Wiedenbrug||BOLD:ACT0982
Paraphaenocladius sp. 2ES|Finnmark319|Elisabeth Stur||BOLD:AAV6218
Paraphaenocladius sp. 2ES|Finnmark316|Elisabeth Stur||BOLD:AAV6218
Paraphaenocladius sp. 2ES|Finnmark315|Elisabeth Stur||BOLD:AAV6218
Bryophaenocladius|CCDB24229-A07|Sofia Wiedenbrug|BIN Taxonomy Match|BOLD:ACR1329
Bryophaenocladius|BIOUG43077-F06|Kate Perez|BIN Taxonomy Match below Phylum (Jun 2019)|BOLD...
Bryophaenocladius|BIOUG16709-B05|Kate Perez|BOLD ID Engine Manual|BOLD:ACR1329
Bryophaenocladius|BIOUG42942-C09|Kate Perez|BIN Taxonomy Match below Phylum (Jun 2019)|BOLD...
Bryophaenocladius|CCDB24228-D06|Sofia Wiedenbrug|BIN Taxonomy Match|BOLD:ACR1329

Bryophaenocladius|BIOUG16709-B05|Kate Perez|BOLD ID Engine Manual|BOLD:ACR1329
Bryophaenocladius|BIOUG42942-C09|Kate Perez|BIN Taxonomy Match below Phylum (Jun 2019)|BOLD...
Bryophaenocladius|CCDB24228-D06|Sofia Wiedenbrug|BIN Taxonomy Match|BOLD:ACR1329
Bryophaenocladius|BIOUG42930-E04|Kate Perez|BIN Taxonomy Match below Phylum (Jun 2019)|BOLD...
Bryophaenocladius|BIOUG42938-F10|Kate Perez|BIN Taxonomy Match below Phylum (Jun 2019)|BOLD...
Bryophaenocladius|BC-ZSM-DIP-22551-F10|Dieter Doczkal|BIN Taxonomy Match|BOLD:ACU4527
Bryophaenocladius|CCDB24038-G04|Jerome Moriniere|BIN Taxonomy Match|BOLD:ACU4527
Bryophaenocladius|BC-ZSM-DIP-22491-E03|Dieter Doczkal|BIN Taxonomy Match|BOLD:ACU4527
Bryophaenocladius|CCDB24228-E10|Sofia Wiedenbrug|BIN Taxonomy Match|BOLD:ACU4852
Bryophaenocladius|CCDB24228-D07|Sofia Wiedenbrug|BIN Taxonomy Match|BOLD:ACU4852
Bryophaenocladius|CCDB24228-D04|Sofia Wiedenbrug|BIN Taxonomy Match|BOLD:ACU4852
Bryophaenocladius|ZSM34343-D11|Sofia Wiedenbrug|Morphology|BOLD:ACU4086
Bryophaenocladius|ZSM34341-E11|Sofia Wiedenbrug|Morphology|BOLD:ACU4086
Bryophaenocladius|CCDB24228-D03|Sofia Wiedenbrug|BIN Taxonomy Match|BOLD:ACU4086
Bryophaenocladius|CCDB24228-C05|Sofia Wiedenbrug|BIN Taxonomy Match|BOLD:ACU4086
Bryophaenocladius|CCDB24228-C04|Sofia Wiedenbrug|BIN Taxonomy Match|BOLD:ACU4086
Chironomidae|ZSM-DIP-33145-H11|Caroline Chimeno|BIN Taxonomy Match (Jan 2022)|BOLD:ACY8737
Chironomidae|ZSM-DIP-33139-A12|Caroline Chimeno|BIN Taxonomy Match (Jan 2022)|BOLD:ACY8737
Chironomidae|ZSM-DIP-33149-E08|Caroline Chimeno|BIN Taxonomy Match (Jan 2022)|BOLD:ACY8737
Chironomidae|ZSM-DIP-33170-E08|Caroline Chimeno|BIN Taxonomy Match (Jan 2022)|BOLD:ACY8737
Chironomidae|ZSM-DIP-33147-E06|Caroline Chimeno|BIN Taxonomy Match (Jan 2022)|BOLD:ACY8737
Chironomidae|ZSM-DIP-33146-D01|Caroline Chimeno|BIN Taxonomy Match (Jan 2022)|BOLD:ACY8737
Chironomidae|ZSM-DIP-33145-G04|Caroline Chimeno|BIN Taxonomy Match (Jan 2022)|BOLD:ACY8737
Chironomidae|ZSM-DIP-33145-G03|Caroline Chimeno|BIN Taxonomy Match (Jan 2022)|BOLD:ACY8737
Chironomidae|ZSM-DIP-33144-F04|Caroline Chimeno|BIN Taxonomy Match (Jan 2022)|BOLD:ACY8737
Chironomidae|ZSM-DIP-33139-A11|Caroline Chimeno|BIN Taxonomy Match (Jan 2022)|BOLD:ACY8737
Chironomidae|ZSM-DIP-33133-F05|Caroline Chimeno|BIN Taxonomy Match (Jan 2022)|BOLD:ACY8737
Chironomidae|ZSM-DIP-33170-D08|Caroline Chimeno|BIN Taxonomy Match (Jan 2022)|BOLD:ACY8737
Chironomidae|ZSM-DIP-33138-D04|Caroline Chimeno|BIN Taxonomy Match (Jan 2022)|BOLD:ACY8737
Chironomidae|BIOUG55195-G04|Stefan Schmidt|BIN Taxonomy Match (Sep 2020)|BOLD:ACY8737
Chironomidae|BIOUG42914-E06|Kate Perez|BIN Taxonomy Match below Phylum (Jun 2019)|BOLD:A...
Chironomidae|BIOUG42723-C10|Kate Perez|BIN Taxonomy Match below Phylum (Jun 2019)|BOLD:A...
Chironomidae|BIOUG42677-D09|Kate Perez|BIN Taxonomy Match below Phylum (Jun 2019)|BOLD:A...
Chironomidae|BIOUG42670-E03|Kate Perez|BIN Taxonomy Match below Phylum (Jun 2019)|BOLD:A...
Chironomidae|BIOUG42665-F01|Kate Perez|BIN Taxonomy Match below Phylum (Jun 2019)|BOLD:A...
Chironomidae|BIOUG42764-E04|Kate Perez|BIN Taxonomy Match below Phylum (Jun 2019)|BOLD:A...
Chironomidae|BIOUG42667-B06|Kate Perez|BIN Taxonomy Match below Phylum (Jun 2019)|BOLD:A...
Chironomidae|BIOUG42759-D06|Kate Perez|BIN Taxonomy Match below Phylum (Jun 2019)|BOLD:A...
Chironomidae|BIOUG42919-F02|Kate Perez|BIN Taxonomy Match below Phylum (Jun 2019)|BOLD:A...
Chironomidae|BIOUG42918-F02|Kate Perez|BIN Taxonomy Match below Phylum (Jun 2019)|BOLD:A...
Chironomidae|BIOUG37052-C09|Meredith Miller|Tree-based Identification (Jan 2019)|BOLD:AD...
Chironomidae|BIOUG36761-E04|Meredith Miller|Tree-based Identification (Jan 2019)|BOLD:AD...
Chironomidae|BIOUG16703-E02|Jerome Moriniere|BIN Taxonomy Match|BOLD:ACR1708
Chironomidae|ZSM-DIP-33134-H07|Caroline Chimeno|BIN Taxonomy Match (Jan 2022)|BOLD:ACR0876
Chironomidae|BIOUG36828-F02|Kate Perez|BIN Taxonomy Match (Jul 2018)|BOLD:ACQ8502
Chironomidae|BIOUG36820-E10|Kate Perez|BIN Taxonomy Match (Jul 2018)|BOLD:ACQ8502
Chironomidae|BIOUG36853-H11|Kate Perez|BIN Taxonomy Match (Jul 2018)|BOLD:ACQ8502
Chironomidae|BIOUG36854-H09|Kate Perez|BIN Taxonomy Match (Jul 2018)|BOLD:ACQ8502
Chironomidae|BIOUG36777-E06|Kate Perez|BIN Taxonomy Match (Jul 2018)|BOLD:ACQ8502
Chironomidae|BIOUG36932-B01|Kate Perez|BIN Taxonomy Match (Jul 2018)|BOLD:ACQ8502
Chironomidae|BIOUG36761-E03|Kate Perez|BIN Taxonomy Match (Jul 2018)|BOLD:ACQ8502
Chironomidae|BIOUG36762-E10|Kate Perez|BIN Taxonomy Match (May 2018)|BOLD:ACQ8502
Chironomidae|BIOUG36828-A05|Kate Perez|BIN Taxonomy Match (Jul 2018)|BOLD:ACQ8502
Chironomidae|BIOUG36827-G05|Kate Perez|BIN Taxonomy Match (Jul 2018)|BOLD:ACQ8502
Chironomidae|BIOUG36828-B11|Kate Perez|BIN Taxonomy Match (Jul 2018)|BOLD:ACQ8502
Chironomidae|BIOUG16733-C11|Jerome Moriniere|BIN Taxonomy Match|BOLD:ACQ8502
Chironomidae|BIOUG16709-C04|Jerome Moriniere|BIN Taxonomy Match|BOLD:ACQ8502
Chironomidae|BIOUG43078-H09|Kate Perez|BIN Taxonomy Match below Phylum (Jun 2019)|BOLD:A...
Chironomidae|BIOUG36761-G11|Kate Perez|BIN Taxonomy Match (Jul 2018)|BOLD:ACQ8502
Chironomidae|BIOUG36845-F03|Kate Perez|BIN Taxonomy Match (Jul 2018)|BOLD:ACQ8502
Chironomidae|BIOUG36828-E05|Kate Perez|BIN Taxonomy Match (Jul 2018)|BOLD:ACQ8502
Chironomidae|BIOUG16270-H01|Jerome Moriniere|BIN Taxonomy Match|BOLD:ACQ8502
Chironomidae|BIOUG17119-F11|Jerome Moriniere|BIN Taxonomy Match|BOLD:ACR0876
Chironomidae|BIOUG16893-E03|Jerome Moriniere|BIN Taxonomy Match|BOLD:ACR0876
Chironomidae|BIOUG16736-D07|Jerome Moriniere|BIN Taxonomy Match|BOLD:ACR0876
Chironomidae|BIOUG16710-F11|Meredith Miller|Tree-based Identification (Feb 2019)|BOLD:AC...
Chironomidae|BIOUG36762-G05|Kate Perez|BIN Taxonomy Match (May 2018)|BOLD:ACR0876
Chironomidae|BIOUG36785-G03|Kate Perez|BIN Taxonomy Match (Jul 2018)|BOLD:ACR0876
Chironomidae|BIOUG36828-C07|Kate Perez|BIN Taxonomy Match (Jul 2018)|BOLD:ACR0876
Chironomidae|BIOUG37052-G05|Kate Perez|BIN Taxonomy Match (Jul 2018)|BOLD:ACR0876
Chironomidae|BIOUG36854-B06|Kate Perez|BIN Taxonomy Match (Jul 2018)|BOLD:ACR0876
Chironomidae|BIOUG16708-E06|Jerome Moriniere|BIN Taxonomy Match|BOLD:ACR0876
Chironomidae|BIOUG16735-B11|Jerome Moriniere|BIN Taxonomy Match|BOLD:ACR0876
Chironomidae|BIOUG16735-D04|Jerome Moriniere|BIN Taxonomy Match|BOLD:ACR0876
Chironomidae|BIOUG42966-A03|Kate Perez|BIN Taxonomy Match below Phylum (Jun 2019)|BOLD:A...
Chironomidae|ZSM-DIP-33133-B03|Caroline Chimeno|BIN Taxonomy Match (Jan 2022)|BOLD:ACR0876
Chironomidae|ZSM-DIP-33134-H10|Caroline Chimeno|BIN Taxonomy Match (Jan 2022)|BOLD:ACR0876
Chironomidae|ZSM-DIP-33129-C01|Caroline Chimeno|BIN Taxonomy Match (Jan 2022)|BOLD:ACR0876
Chironomidae|BIOUG36787-H06|Kate Perez|BIN Taxonomy Match (Jul 2018)|BOLD:ACR0876
Chironomidae|BIOUG36820-H10|Kate Perez|BIN Taxonomy Match (Jul 2018)|BOLD:ACR0876
Chironomidae|BIOUG36786-A06|Kate Perez|BIN Taxonomy Match (Jul 2018)|BOLD:ACR0876
Chironomidae|BIOUG36847-C04|Kate Perez|BIN Taxonomy Match (Jul 2018)|BOLD:ACR0876
Chironomidae|BIOUG36853-F12|Kate Perez|BIN Taxonomy Match (Jul 2018)|BOLD:ACR0876
Chironomidae|BIOUG36678-F08|Kate Perez|BIN Taxonomy Match (Jul 2018)|BOLD:ACR0876
Chironomidae|BIOUG37052-B09|Kate Perez|BIN Taxonomy Match (Jul 2018)|BOLD:ACR0876
Chironomidae|BIOUG16709-F09|Jerome Moriniere|BIN Taxonomy Match|BOLD:ACR0876
Chironomidae|BIOUG36828-H03|Kate Perez|BIN Taxonomy Match (Jul 2018)|BOLD:ACR0876
Chironomidae|BIOUG36820-B05|Kate Perez|BIN Taxonomy Match (Jul 2018)|BOLD:ACR0876
Chironomidae|BIOUG36788-D01|Kate Perez|BIN Taxonomy Match (Jul 2018)|BOLD:ACR0876
Chironomidae|BIOUG36846-A11|Kate Perez|BIN Taxonomy Match (Jul 2018)|BOLD:ACR0876
Chironomidae|BIOUG36828-B05|Kate Perez|BIN Taxonomy Match (Jul 2018)|BOLD:ACR0876
Chironomidae|BIOUG36679-G09|Kate Perez|BIN Taxonomy Match (Jul 2018)|BOLD:ACR0876
Chironomidae|BIOUG36765-H05|Kate Perez|BIN Taxonomy Match (May 2018)|BOLD:ACR0876
Chironomidae|BIOUG16708-A12|Kate Perez|Tree base identification
Chironomidae|ZSM-DIP-33134-H05|Caroline Chimeno|BIN Taxonomy Match (Jan 2022)|BOLD:ACR0876
Chironomidae|BIOUG16612-C03|Jerome Moriniere|BIN Taxonomy Match|BOLD:ACR0876
Chironomidae|BIOUG36787-F10|Kate Perez|BIN Taxonomy Match (Jul 2018)|BOLD:ACR0361
Chironomidae|BIOUG36786-E09|Kate Perez|BIN Taxonomy Match (Jul 2018)|BOLD:ACR0361
Chironomidae|BIOUG17444-A11|Jerome Moriniere|BIN Taxonomy Match|BOLD:ACR0361
Chironomidae|BIOUG17114-G06|Jerome Moriniere|BIN Taxonomy Match|BOLD:ACR0361
Chironomidae|BIOUG36828-E07|Kate Perez|BIN Taxonomy Match (Jul 2018)|BOLD:ACR0361
Chironomidae|BIOUG16733-D08|Jerome Moriniere|BIN Taxonomy Match|BOLD:ACR0361
Chironomidae|BIOUG16529-B09|Jerome Moriniere|BIN Taxonomy Match|BOLD:ACR0361

Chironomidae|BIOUG36828-E07|Kate Perez|BIN Taxonomy Match (Jul 2018)|BOLD:ACR0361
Chironomidae|BIOUG16733-D08|Jerome Moriniere|BIN Taxonomy Match|BOLD:ACR0361
Chironomidae|BIOUG16529-B09|Jerome Moriniere|BIN Taxonomy Match|BOLD:ACR0361
Chironomidae|BIOUG16527-E12|Jerome Moriniere|BIN Taxonomy Match|BOLD:ACR0361
Smittia|CCDB24229-A10|Sofia Wiedenbrug|BIN Taxonomy Match|BOLD:ACU5497
Smittia|CCDB24229-A11|Sofia Wiedenbrug|BIN Taxonomy Match|BOLD:ACU5497
Smittia|CCDB24228-E01|Sofia Wiedenbrug|BIN Taxonomy Match|BOLD:ACU5497
Smittia|CCDB24039-A03|Sofia Wiedenbrug|BIN Taxonomy Match|BOLD:ACU5497
Eukiefferiella claripennis|MZ004968|||BOLD:AEK7845
Smittia|BC-ZSM-DIP-22488-H04|Dieter Doczkal|BIN Taxonomy Match|BOLD:ACX2764
Eukiefferiella coerulescens|ES57|Elisabeth Stur||BOLD:AAY3165
Orthocladiinae|BIOUG08131-F09|Kate Perez|BIN Taxonomy Match|BOLD:ACJ3228
Psectrocladius calcaratus|TRD-CH107|Elisabeth Stur||BOLD:AAM7062
Psectrocladius calcaratus|Finnmark676|Torbjorn Ekrem||BOLD:AAM7062
Psectrocladius calcaratus|Finnmark246|Torbjorn Ekrem||BOLD:AAM7062
Psectrocladius calcaratus|Finnmark104|Torbjorn Ekrem||BOLD:AAM7062
Psectrocladius calcaratus|Finnmark478|Torbjorn Ekrem||BOLD:AAM7062
Bryophaenocladius xanthogyne|ZSM-DIP-33131-D06|Caroline Chimeno|BIN Taxonomy Match (Jan 2022 ...
Smittia|CCDB21605-H07|Sofia Wiedenbrug||BOLD:ACC0349
Bryophaenocladius xanthogyne|ZMUO.024733|Lauri Paasivirta||BOLD:ACC0349
Bryophaenocladius xanthogyne|ZMUO.024732|Lauri Paasivirta||BOLD:ACC0349
Bryophaenocladius nr. xanthogyne|ZMUO.024852|Lauri Paasivirta||BOLD:ACC0349
Orthocladiinae|BIOUG04269-E09|Kate Perez|BIN Taxonomy Match (Aug 2017)|BOLD:ACC0349
Smittia|BIOUG16997-A02|Kate Perez|BOLD ID Engine Manual|BOLD:ACC0349
Orthocladiinae|BIOUG04261-H04|Kate Perez|BIN Taxonomy Match (Aug 2017)|BOLD:ACC0349
Orthocladiinae|BIOUG04090-B11|Kate Perez|BIN Taxonomy Match (Aug 2017)|BOLD:ACC0349
Bryophaenocladius nr. xanthogyne|ZMUO.024851|Lauri Paasivirta||BOLD:ACC0349
Orthocladiinae|BIOUG04090-B02|Kate Perez|BIN Taxonomy Match (Aug 2017)|BOLD:ACC0349
Orthocladiinae|BIOUG04089-E11|Kate Perez|BIN Taxonomy Match (Aug 2017)|BOLD:ACC0349
Smittia|ZSM-DIP-33138-G03|Caroline Chimeno|BIN Taxonomy Match (Jan 2022)|BOLD:ACR1218
Smittia|BIOUG17117-D08|Kate Perez|BOLD ID Engine Manual|BOLD:ACR1218
Smittia|ZSM-DIP-33132-B10|Caroline Chimeno|BIN Taxonomy Match (Jan 2022)|BOLD:ACR1218
Smittia|BIOUG17117-C12|Kate Perez|BOLD ID Engine Manual|BOLD:ACR1218
Smittia|ZSM-DIP-33132-B12|Caroline Chimeno|BIN Taxonomy Match (Jan 2022)|BOLD:ACR1218
Smittia|BIOUG16996-A10|Kate Perez|BOLD ID Engine Manual|BOLD:ACR1218
Smittia|ZSM-DIP-33138-H11|Caroline Chimeno|BIN Taxonomy Match (Jan 2022)|BOLD:ACR1218
Smittia|BIOUG17114-H03|Kate Perez|BOLD ID Engine Manual|BOLD:ACR1218
Smittia|BIOUG16996-H08|Kate Perez|BOLD ID Engine Manual|BOLD:ACR1218
Smittia|ZSM-DIP-33142-B11|Caroline Chimeno|BIN Taxonomy Match (Jan 2022)|BOLD:ACR1218
Smittia|BIOUG17114-G09|Kate Perez|BOLD ID Engine Manual|BOLD:ACR1218
Smittia|BIOUG17117-E10|Kate Perez|BOLD ID Engine Manual|BOLD:ACR1218
Smittia|BIOUG17114-E12|Kate Perez|BOLD ID Engine Manual|BOLD:ACR1218
Smittia|BIOUG17119-F09|Kate Perez|BOLD ID Engine Manual|BOLD:ACR1218
Smittia|BIOUG17120-D08|Kate Perez|BOLD ID Engine Manual|BOLD:ACR1218
Smittia|BIOUG17136-C03|Kate Perez|BOLD ID Engine Manual|BOLD:ACR1218
Smittia|BIOUG17117-H04|Kate Perez|BOLD ID Engine Manual|BOLD:ACR1218
Smittia|BIOUG16997-C11|Kate Perez|BOLD ID Engine Manual|BOLD:ACR1218
Smittia|BIOUG16900-A01|Kate Perez|BOLD ID Engine Manual|BOLD:ACR1218
Smittia|BIOUG17114-G02|Kate Perez|BOLD ID Engine Manual|BOLD:ACR1218
Smittia|BIOUG16996-G07|Kate Perez|BOLD ID Engine Manual|BOLD:ACR1218
Smittia|BIOUG16994-H01|Kate Perez|BOLD ID Engine Manual|BOLD:ACR1218
Smittia|BIOUG17099-G10|Kate Perez|BOLD ID Engine Manual|BOLD:ACR1218
Smittia|ZSM-DIP-33138-H04|Caroline Chimeno|BIN Taxonomy Match (Jan 2022)|BOLD:ACR1218
Smittia|ZSM-DIP-33142-C01|Caroline Chimeno|BIN Taxonomy Match (Jan 2022)|BOLD:ACR1218
Smittia|ZSM-DIP-33138-G04|Caroline Chimeno|BIN Taxonomy Match (Jan 2022)|BOLD:ACR1218
Smittia|ZSM-DIP-33132-C01|Caroline Chimeno|BIN Taxonomy Match (Jan 2022)|BOLD:ACR1218
Halocladius varians|NHRS-BYWS000000278|Yngve Brodin|morphology|BOLD:AAV7022
Halocladius varians|NHRS-BYWS000001148|Yngve Brodin|morphology|BOLD:AAV7022
Halocladius varians|NHRS-BYWS000001147|Yngve Brodin|morphology|BOLD:AAV7022
Halocladius varians|NHRS-BYWS000001786|Yngve Brodin||BOLD:AAV7022
Bryophaenocladius xanthogyne|ZMUO.024534|Lauri Paasivirta||BOLD:ADA7427
Bryophaenocladius xanthogyne|BIOUG36676-E09|Kate Perez|BIN Taxonomy Match (Jul 2018)|BOLD ...
Bryophaenocladius xanthogyne|BIOUG36678-E05|Kate Perez|BIN Taxonomy Match (Jul 2018)|BOLD ...
Bryophaenocladius xanthogyne|BIOUG36677-A04|Kate Perez|BIN Taxonomy Match (May 2018)|BOLD ...
Bryophaenocladius xanthogyne|ZSM-DIP-33130-B01|Caroline Chimeno|BIN Taxonomy Match (Jan 2022 ...
Bryophaenocladius xanthogyne|BIOUG36678-B04|Kate Perez|BIN Taxonomy Match (Jul 2018)|BOLD ...
Bryophaenocladius xanthogyne|BIOUG36677-A12|Kate Perez|BIN Taxonomy Match (May 2018)|BOLD ...
Bryophaenocladius xanthogyne|ZSM-DIP-33130-B03|Caroline Chimeno|BIN Taxonomy Match (Jan 2022 ...
Bryophaenocladius xanthogyne|ZMUO.024535|Lauri Paasivirta||BOLD:ADA7427
Bryophaenocladius xanthogyne|ZSM-DIP-33130-B02|Caroline Chimeno|BIN Taxonomy Match (Jan 2022 ...
Bryophaenocladius sp. 11ES|Finnmark360|Elisabeth Stur||BOLD:AAW7326
Eukiefferiella tirolensis|CCDB21606-G06|Sofia Wiedenbrug||BOLD:ACT5781
Eukiefferiella tirolensis|CCDB24037-G02|Sofia Wiedenbrug|BIN Taxonomy Match|BOLD:ACT5781
Eukiefferiella|ZSM34343-F04|Jerome Moriniere|BIN Taxonomy Match|BOLD:ACX2540
Eukiefferiella|BC-ZSM-DIP-22488-H11|Dieter Doczkal|BIN Taxonomy Match|BOLD:ACX2540
Chironomidae|CCDB22603-F02|Sofia Wiedenbrug
Eukiefferiella|CCDB22603-E03|Sofia Wiedenbrug||BOLD:ACQ3155
Eukiefferiella|BC-ZSM-DIP-22488-D04|Dieter Doczkal||BOLD:ACU4068
Eukiefferiella|BC-ZSM-DIP-22488-C10|Dieter Doczkal||BOLD:ACU4068
Eukiefferiella|BC-ZSM-DIP-22488-D08|Dieter Doczkal||BOLD:ACU4068
Eukiefferiella|BC-ZSM-DIP-22488-D02|Dieter Doczkal||BOLD:ACU4068
Eukiefferiella|CCDB24038-G05|Sofia Wiedenbrug|BIN Taxonomy Match|BOLD:ACU4068
Eukiefferiella|CCDB24038-G07|Sofia Wiedenbrug|BIN Taxonomy Match|BOLD:ACU4068
Eukiefferiella|BC-ZSM-DIP-22488-E12|Dieter Doczkal||BOLD:ACU4068
Eukiefferiella|CCDB24038-H02|Sofia Wiedenbrug|BIN Taxonomy Match|BOLD:ACU4068
Eukiefferiella|CCDB24038-E09|Sofia Wiedenbrug|BIN Taxonomy Match|BOLD:ACU4068
Eukiefferiella|BC-ZSM-DIP-22488-D06|Dieter Doczkal||BOLD:ACU4068
Eukiefferiella|CCDB24038-H03|Sofia Wiedenbrug|BIN Taxonomy Match|BOLD:ACU4068
Eukiefferiella|CCDB24037-E02|Sofia Wiedenbrug|BIN Taxonomy Match|BOLD:ACU4068
Eukiefferiella|CH-OSF73|Elisabeth Stur||BOLD:ADS5020
Eukiefferiella brevicalcar|BIOUG15537-C08|Kate Perez|BIN Taxonomy Match|BOLD:AAY3164
Eukiefferiella brevicalcar|BIOUG15545-G07|Kate Perez|BIN Taxonomy Match|BOLD:AAY3164
Eukiefferiella brevicalcar|BIOUG15561-B01|Kate Perez|BIN Taxonomy Match|BOLD:AAY3164
Eukiefferiella brevicalcar|BIOUG15950-A04|Kate Perez|BIN Taxonomy Match|BOLD:AAY3164
Eukiefferiella brevicalcar|ZMUO.024874|Lauri Paasivirta||BOLD:AAY3164
Eukiefferiella brevicalcar|ZMUO.024875|Lauri Paasivirta||BOLD:AAY3164
Eukiefferiella brevicalcar|BIOUG15465-A10|Kate Perez|BIN Taxonomy Match|BOLD:AAY3164
Eukiefferiella brevicalcar|BIOUG15562-D03|Kate Perez|BIN Taxonomy Match|BOLD:AAY3164
Eukiefferiella brevicalcar|EBAI-Ch222|Elisabeth Stur|morphology|BOLD:AAY3164
Eukiefferiella brevicalcar|EBAI-Ch221|Elisabeth Stur|morphology|BOLD:AAY3164
Eukiefferiella brevicalcar|EBAI-Ch219|Elisabeth Stur|morphology|BOLD:AAY3164
Eukiefferiella brevicalcar|EBAI-Ch218|Elisabeth Stur|morphology|BOLD:AAY3164
Eukiefferiella brevicalcar|EBAI-Ch180|Elisabeth Stur|morphology|BOLD:AAY3164

[illegible]
Eukiefferiella brevicalcar|EBAI-Ch219|Elisabeth Stur|morphology|BOLD:AAY3164
Eukiefferiella brevicalcar|EBAI-Ch218|Elisabeth Stur|morphology|BOLD:AAY3164
Eukiefferiella brevicalcar|EBAI-Ch180|Elisabeth Stur|morphology|BOLD:AAY3164
Eukiefferiella brevicalcar|Finnmark896|G. A. Halvorsen||BOLD:AAY3164
Eukiefferiella brevicalcar|ATNA222|Elisabeth Stur||BOLD:AAY3164
Eukiefferiella brevicalcar|CH-OSF12|Elisabeth Stur||BOLD:AAY3164
Eukiefferiella brevicalcar|Finnmark643|Elisabeth Stur||BOLD:AAY3164
Chironomidae|BIOUG17120-A04|Jerome Moriniere|BIN Taxonomy Match|BOLD:ACR3254
Chironomidae|BIOUG17380-C10|Jerome Moriniere|BIN Taxonomy Match|BOLD:ACR2744
Chironomidae|BIOUG17379-G12|Jerome Moriniere|BIN Taxonomy Match|BOLD:ACR2744
Chironomidae|BIOUG17303-C03|Jerome Moriniere|BIN Taxonomy Match|BOLD:ACR2744
Chironomidae|BIOUG55144-A07|Kate Perez|BIN Taxonomy Match (Oct 2020)|BOLD:ACR2744
Chironomidae|BIOUG17376-E09|Jerome Moriniere|BIN Taxonomy Match|BOLD:ACR2744
Chironomidae|BIOUG17178-B06|Jerome Moriniere|BIN Taxonomy Match|BOLD:ACR2744
Chironomidae|BIOUG17137-G12|Jerome Moriniere|BIN Taxonomy Match|BOLD:ACR2744
Chironomidae|BIOUG17437-A10|Jerome Moriniere|BIN Taxonomy Match|BOLD:ACR2744
Chironomidae|BIOUG17380-E07|Jerome Moriniere|BIN Taxonomy Match|BOLD:ACR2744
Bryophaenocladius|BIOUG42731-H11|Kate Perez|BIN Taxonomy Match below Phylum (Jun 2019)|BOLD...
Bryophaenocladius|BIOUG42689-B10|Kate Perez|BIN Taxonomy Match below Phylum (Jun 2019)|BOLD...
Bryophaenocladius|BIOUG42685-D09|Kate Perez|BIN Taxonomy Match below Phylum (Jun 2019)|BOLD...
Bryophaenocladius scanicus|TRD-CH196|Elisabeth Stur||BOLD:ACT7634
Bryophaenocladius scanicus|ZMUO.024961|Lauri Paasivirta||BOLD:ACT7634
Bryophaenocladius|BIOUG55195-D11|Stefan Schmidt|BIN Taxonomy Match (Sep 2020)|BOLD:ACT7634
Bryophaenocladius nidorum|ZMUO.025293|Lauri Paasivirta||BOLD:ACT7634
Bryophaenocladius nidorum|ZMUO.025292|Lauri Paasivirta||BOLD:ACT7634
Bryophaenocladius nidorum|ZMUO.025176|Lauri Paasivirta||BOLD:ACT7634
Bryophaenocladius|BIOUG42679-H09|Kate Perez|BIN Taxonomy Match below Phylum (Jun 2019)|BOLD...
Bryophaenocladius|BIOUG42687-D08|Kate Perez|BIN Taxonomy Match below Phylum (Jun 2019)|BOLD...
Bryophaenocladius nidorum|ZMUO.025177|Lauri Paasivirta||BOLD:ACT7634
Bryophaenocladius|BIOUG36680-H03|Kate Perez|BIN Taxonomy Match (May 2018)|BOLD:ACT7634
Bryophaenocladius|BIOUG42713-C02|Kate Perez|BIN Taxonomy Match below Phylum (Jun 2019)|BOLD...
Bryophaenocladius|BIOUG42923-B06|Kate Perez|BIN Taxonomy Match below Phylum (Jun 2019)|BOLD...
Bryophaenocladius|BIOUG42920-D04|Kate Perez|BIN Taxonomy Match below Phylum (Jun 2019)|BOLD...
Chironomidae|ZSM-DIP-33145-D07|Valerie Levesque-Beaudin|BIN Taxonomy Match (Sep 2021)|BOLD...
Chironomidae|ZSM-DIP-33170-A12|Valerie Levesque-Beaudin|BIN Taxonomy Match (Sep 2021)|BOLD...
Chironomidae|ZSM-DIP-33149-D05|Valerie Levesque-Beaudin|BIN Taxonomy Match (Sep 2021)|BOLD...
Chironomidae|ZSM-DIP-33149-D10|Valerie Levesque-Beaudin|BIN Taxonomy Match (Sep 2021)|BOLD...
Chironomidae|ZSM-DIP-33130-F04|Valerie Levesque-Beaudin|BIN Taxonomy Match (Sep 2021)|BOLD...
Chironomidae|ZSM-DIP-33141-E05|Valerie Levesque-Beaudin|BIN Taxonomy Match (Sep 2021)|BOLD...
Chironomidae|ZSM-DIP-33141-E04|Valerie Levesque-Beaudin|BIN Taxonomy Match (Sep 2021)|BOLD...
Chironomidae|ZSM-DIP-33144-B01|Valerie Levesque-Beaudin|BIN Taxonomy Match (Sep 2021)|BOLD...
Chironomidae|ZSM-DIP-33145-D06|Valerie Levesque-Beaudin|BIN Taxonomy Match (Sep 2021)|BOLD...
Chironomidae|ZSM-DIP-33145-D10|Valerie Levesque-Beaudin|BIN Taxonomy Match (Sep 2021)|BOLD...
Chironomidae|ZSM-DIP-33141-E10|Valerie Levesque-Beaudin|BIN Taxonomy Match (Sep 2021)|BOLD...
Chironomidae|ZSM-DIP-33130-F03|Valerie Levesque-Beaudin|BIN Taxonomy Match (Sep 2021)|BOLD...
Chironomidae|ZSM-DIP-33170-B02|Valerie Levesque-Beaudin|BIN Taxonomy Match (Sep 2021)|BOLD...
Chironomidae|ZSM-DIP-33149-D07|Valerie Levesque-Beaudin|BIN Taxonomy Match (Sep 2021)|BOLD...
Chironomidae|ZSM-DIP-33149-C12|Valerie Levesque-Beaudin|BIN Taxonomy Match (Sep 2021)|BOLD...
Chironomidae|ZSM-DIP-33145-E01|Valerie Levesque-Beaudin|BIN Taxonomy Match (Sep 2021)|BOLD...
Chironomidae|ZSM-DIP-33145-E02|Valerie Levesque-Beaudin|BIN Taxonomy Match (Sep 2021)|BOLD...
Chironomidae|ZSM-DIP-33149-D08|Valerie Levesque-Beaudin|BIN Taxonomy Match (Sep 2021)|BOLD...
Chironomidae|ZSM-DIP-33141-E12|Valerie Levesque-Beaudin|BIN Taxonomy Match (Sep 2021)|BOLD...
Chironomidae|ZSM-DIP-33149-D04|Valerie Levesque-Beaudin|BIN Taxonomy Match (Sep 2021)|BOLD...
Chironomidae|ZSM-DIP-33144-A11|Valerie Levesque-Beaudin|BIN Taxonomy Match (Sep 2021)|BOLD...
Chironomidae|ZSM-DIP-33149-D11|Valerie Levesque-Beaudin|BIN Taxonomy Match (Sep 2021)|BOLD...
Chironomidae|ZSM-DIP-33143-G11|Valerie Levesque-Beaudin|BIN Taxonomy Match (Sep 2021)|BOLD...
Chironomidae|ZSM-DIP-33145-D12|Valerie Levesque-Beaudin|BIN Taxonomy Match (Sep 2021)|BOLD...
Chironomidae|ZSM-DIP-33145-D08|Valerie Levesque-Beaudin|BIN Taxonomy Match (Sep 2021)|BOLD...
Chironomidae|ZSM-DIP-33135-G11|Valerie Levesque-Beaudin|BIN Taxonomy Match (Sep 2021)|BOLD...
Chironomidae|ZSM-DIP-33144-B08|Valerie Levesque-Beaudin|BIN Taxonomy Match (Sep 2021)|BOLD...
Chironomidae|ZSM-DIP-33149-C11|Valerie Levesque-Beaudin|BIN Taxonomy Match (Sep 2021)|BOLD...
Chironomidae|ZSM-DIP-33145-D11|Valerie Levesque-Beaudin|BIN Taxonomy Match (Sep 2021)|BOLD...
Chironomidae|ZSM-DIP-33130-G09|Valerie Levesque-Beaudin|BIN Taxonomy Match (Sep 2021)|BOLD...
Chironomidae|ZSM-DIP-33144-B02|Valerie Levesque-Beaudin|BIN Taxonomy Match (Sep 2021)|BOLD...
Chironomidae|ZSM-DIP-33170-B01|Valerie Levesque-Beaudin|BIN Taxonomy Match (Sep 2021)|BOLD...
Chironomidae|ZSM-DIP-33134-B08|Valerie Levesque-Beaudin|BIN Taxonomy Match (Sep 2021)|BOLD...
Chironomidae|ZSM-DIP-33149-D06|Valerie Levesque-Beaudin|BIN Taxonomy Match (Sep 2021)|BOLD...
Chironomidae|ZSM-DIP-33144-B09|Valerie Levesque-Beaudin|BIN Taxonomy Match (Sep 2021)|BOLD...
Chironomidae|ZSM-DIP-33134-B06|Valerie Levesque-Beaudin|BIN Taxonomy Match (Sep 2021)|BOLD...
Chironomidae|ZSM-DIP-33129-C09|Caroline Chimeno|BIN Taxonomy Match (Jan 2022)|BOLD:ACR3766
Chironomidae|ZSM-DIP-33136-H03|Caroline Chimeno|BIN Taxonomy Match (Jan 2022)|BOLD:ACR3766
Orthocladiinae|MEBFL12||BOLD ID Engine|BOLD:ACR3766
Chironomidae|BIOUG16705-A09|Jerome Moriniere|BIN Taxonomy Match|BOLD:ACR3766
Chironomidae|BIOUG16705-C10|Jerome Moriniere|BIN Taxonomy Match|BOLD:ACR3766
Chironomidae|BIOUG16705-G11|Jerome Moriniere|BIN Taxonomy Match|BOLD:ACR3766
Chironomidae|BIOUG16706-C10|Jerome Moriniere|BIN Taxonomy Match|BOLD:ACR3766
Chironomidae|BIOUG16734-C10|Jerome Moriniere|BIN Taxonomy Match|BOLD:ACR3766
Chironomidae|BIOUG16735-A11|Jerome Moriniere|BIN Taxonomy Match|BOLD:ACR3766
Chironomidae|BIOUG17376-E07|Jerome Moriniere|BIN Taxonomy Match|BOLD:ACR3766
Chironomidae|BIOUG17376-B10|Jerome Moriniere|BIN Taxonomy Match|BOLD:ACR3766
Chironomidae|BIOUG16710-H02|Jerome Moriniere|BIN Taxonomy Match|BOLD:ACR3766
Chironomidae|BIOUG16709-H07|Jerome Moriniere|BIN Taxonomy Match|BOLD:ACR3766
Chironomidae|BIOUG16705-H08|Jerome Moriniere|BIN Taxonomy Match|BOLD:ACR3766
Chironomidae|BIOUG17444-F06|Jerome Moriniere|BIN Taxonomy Match|BOLD:ACR3766
Bryophaenocladius propinquus|ZSM-DIP-33146-F10|Caroline Chimeno|BIN Taxonomy Match (Jan 2022 ...
Bryophaenocladius propinquus|ZSM-DIP-33137-F04|Caroline Chimeno|BIN Taxonomy Match (Jan 2022 ...
Bryophaenocladius propinquus|NHRS-BYWS000000334|Yngve Brodin|morphology|BOLD:ADF0610
Bryophaenocladius propinquus|ZSM-DIP-33130-A05|Caroline Chimeno|BIN Taxonomy Match (Jan 2022 ...
Bryophaenocladius propinquus|NHRS-BYWS000000924|Yngve Brodin|morphology|BOLD:ADF0610
Bryophaenocladius|ZMUO.026223|Lauri Paasivirta||BOLD:ADF0610
Bryophaenocladius|ZMUO.026224|Lauri Paasivirta||BOLD:ADF0610
Bryophaenocladius propinquus|ZSM-DIP-33137-F05|Caroline Chimeno|BIN Taxonomy Match (Jan 2022 ...
Bryophaenocladius propinquus|ZSM-DIP-33141-F06|Caroline Chimeno|BIN Taxonomy Match (Jan 2022 ...
Bryophaenocladius propinquus|ZSM-DIP-33142-D05|Caroline Chimeno|BIN Taxonomy Match (Jan 2022 ...
Bryophaenocladius propinquus|ZSM-DIP-33137-F01|Caroline Chimeno|BIN Taxonomy Match (Jan 2022 ...
Bryophaenocladius propinquus|ZSM-DIP-33137-F03|Caroline Chimeno|BIN Taxonomy Match (Jan 2022 ...
Bryophaenocladius propinquus|ZSM-DIP-33146-F09|Caroline Chimeno|BIN Taxonomy Match (Jan 2022 ...
Bryophaenocladius propinquus|BIOUG36931-G05|Kate Perez|BIN Taxonomy Match (May 2018)|BOLD ...
Bryophaenocladius propinquus|BIOUG36987-B03|Kate Perez|BIN Taxonomy Match (Jul 2018)|BOLD ...
Bryophaenocladius propinquus|ZSM-DIP-33131-E04|Caroline Chimeno|BIN Taxonomy Match (Jan 2022 ...
Bryophaenocladius propinquus|ZSM-DIP-33134-H08|Caroline Chimeno|BIN Taxonomy Match (Jan 2022 ...
Bryophaenocladius propinquus|ZSM-DIP-33134-H04|Caroline Chimeno|BIN Taxonomy Match (Jan 2022 ...
[illegible]

Bryophaenocladius propinquus|ZSM-DIP-33131-E04|Caroline Chimeno|BIN Taxonomy Match (Jan 2022 ...
Bryophaenocladius propinquus|ZSM-DIP-33134-H08|Caroline Chimeno|BIN Taxonomy Match (Jan 2022 ...
Bryophaenocladius propinquus|ZSM-DIP-33134-H04|Caroline Chimeno|BIN Taxonomy Match (Jan 2022 ...
Bryophaenocladius sp. 3ES|Finnmark64|Elisabeth Stur||BOLD:AAM7074
Bryophaenocladius|CCDB24039-H05|Sofia Wiedenbrug|BIN Taxonomy Match|BOLD:ACU5428
Bryophaenocladius|CCDB24039-H04|Sofia Wiedenbrug|BIN Taxonomy Match|BOLD:ACU5428
Bryophaenocladius cf. akiensis|ZSM-DIP-33142-A12|Caroline Chimeno|BIN Taxonomy Match (Jan 2022...
Bryophaenocladius cf. akiensis|BIOUG17305-D01|Sofia Wiedenbrug|BIN Taxonomy Match|BOLD:ACR3672
Bryophaenocladius cf. akiensis|BIOUG17224-F09|Sofia Wiedenbrug|BIN Taxonomy Match|BOLD:ACR3672
Bryophaenocladius cf. akiensis|ZSM34341-B10|Sofia Wiedenbrug|BIN Taxonomy Match|BOLD:ACR3672
Bryophaenocladius cf. akiensis|ZSM34341-B07|Sofia Wiedenbrug|Morphology|BOLD:ACR3672
Bryophaenocladius cf. akiensis|ZSM34341-C02|Sofia Wiedenbrug|BIN Taxonomy Match|BOLD:ACR3672
Bryophaenocladius cf. akiensis|BIOUG17303-E03|Sofia Wiedenbrug|BIN Taxonomy Match|BOLD:ACR3672
Bryophaenocladius cf. akiensis|ZSM34341-C07|Sofia Wiedenbrug|BIN Taxonomy Match|BOLD:ACR3672
Bryophaenocladius cf. akiensis|ZSM34341-B11|Sofia Wiedenbrug|BIN Taxonomy Match|BOLD:ACR3672
Bryophaenocladius cf. akiensis|ZSM-DIP-33142-B03|Caroline Chimeno|BIN Taxonomy Match (Jan 2022...
Bryophaenocladius cf. akiensis|ZSM34341-B09|Sofia Wiedenbrug|BIN Taxonomy Match|BOLD:ACR3672
Bryophaenocladius cf. akiensis|ZSM34341-C08|Sofia Wiedenbrug|BIN Taxonomy Match|BOLD:ACR3672
Bryophaenocladius cf. akiensis|ZSM34341-B06|Sofia Wiedenbrug|Morphology|BOLD:ACR3672
Bryophaenocladius cf. akiensis|ZSM34341-C04|Sofia Wiedenbrug|Morphology|BOLD:ACR3672
Bryophaenocladius cf. akiensis|ZSM34341-B04|Sofia Wiedenbrug|Morphology|BOLD:ACR3672
Bryophaenocladius cf. akiensis|ZSM34341-B08|Sofia Wiedenbrug|BIN Taxonomy Match|BOLD:ACR3672
Bryophaenocladius cf. akiensis|CCDB24228-D01|Sofia Wiedenbrug|BIN Taxonomy Match|BOLD:ACR3672
Bryophaenocladius aestivus|ZMUO.024578|Lauri Paasivirta||BOLD:AAZ9340
Bryophaenocladius aestivus|ZMUO.024579|Lauri Paasivirta||BOLD:AAZ9340
Bryophaenocladius aestivus|BIOUG37182-E08|Kate Perez|BIN Taxonomy Match (Jul 2018)|BOLD:...
Bryophaenocladius aestivus|BIOUG36762-G12|Kate Perez|BIN Taxonomy Match (May 2018)|BOLD:...
Bryophaenocladius aestivus|BIOUG36845-G03|Kate Perez|BIN Taxonomy Match (Jul 2018)|BOLD:...
Bryophaenocladius aestivus|BIOUG16736-E10|Kate Perez|BIN Taxonomy Match|BOLD:AAZ9340
Bryophaenocladius aestivus|CH-OSF158|Elisabeth Stur||BOLD:AAZ9340
Bryophaenocladius aestivus|CH-OSF157|Elisabeth Stur||BOLD:AAZ9340
Chironomidae|BIOUG36725-D04|Kate Perez|BIN Taxonomy Match (Jan 2019)|BOLD:ADM2958
Chironomidae|BIOUG36724-F05|Meredith Miller|Tree-based Identification (Jan 2019)|BOLD:AD...
Chironomidae|ZSM-DIP-33138-A05|Caroline Chimeno|BIN Taxonomy Match (Jan 2022)|BOLD:ADM2958
Bryophaenocladius|ZSM-DIP-33138-F11|Caroline Chimeno|BIN Taxonomy Match (Jan 2022)|BOLD:...
Bryophaenocladius|ZSM-DIP-33138-B08|Caroline Chimeno|BIN Taxonomy Match (Jan 2022)|BOLD:...
Bryophaenocladius|ZSM-DIP-33129-B10|Caroline Chimeno|BIN Taxonomy Match (Jan 2022)|BOLD:...
Bryophaenocladius|ZSM-DIP-33138-B04|Caroline Chimeno|BIN Taxonomy Match (Jan 2022)|BOLD:...
Bryophaenocladius|ZSM-DIP-33138-B06|Caroline Chimeno|BIN Taxonomy Match (Jan 2022)|BOLD:...
Bryophaenocladius|ZSM-DIP-33138-A06|Caroline Chimeno|BIN Taxonomy Match (Jan 2022)|BOLD:...
Bryophaenocladius|BIOUG16997-B01|Kate Perez|BOLD ID Engine Manual|BOLD:ACR3707
Bryophaenocladius sp. 4ES|CH-eik38|Elisabeth Stur||BOLD:AAW1343
Bryophaenocladius|BIOUG42673-C12|Kate Perez|BIN Taxonomy Match below Phylum (Jun 2019)|BOLD...
Bryophaenocladius|ZSM-DIP-33138-D11|Caroline Chimeno|BIN Taxonomy Match (Jan 2022)|BOLD:...
Bryophaenocladius|ZSM-DIP-33140-G12|Caroline Chimeno|BIN Taxonomy Match (Jan 2022)|BOLD:...
Bryophaenocladius sp. 4ES|BIOUG16734-H04|Kate Perez|BIN Taxonomy Match|BOLD:AAW1343
Bryophaenocladius sp. 4ES|BIOUG17303-G08|Kate Perez|BIN Taxonomy Match|BOLD:AAW1343
Bryophaenocladius sp. 4ES|BIOUG16736-B02|Kate Perez|BIN Taxonomy Match|BOLD:AAW1343
Bryophaenocladius sp. 4ES|BIOUG16706-A08|Kate Perez|BIN Taxonomy Match|BOLD:AAW1343
Bryophaenocladius|BIOUG55421-D03|Stefan Schmidt|BIN Taxonomy Match (Sep 2020)|BOLD:AAW1343
Bryophaenocladius|BIOUG42948-E05|Kate Perez|BIN Taxonomy Match below Phylum (Jun 2019)|BOLD...
Bryophaenocladius|BIOUG42948-E01|Kate Perez|BIN Taxonomy Match below Phylum (Jun 2019)|BOLD...
Bryophaenocladius|ZSM-DIP-33133-G08|Caroline Chimeno|BIN Taxonomy Match (Jan 2022)|BOLD:...
Bryophaenocladius|ZSM-DIP-33142-H01|Caroline Chimeno|BIN Taxonomy Match (Jan 2022)|BOLD:...
Bryophaenocladius|ZSM-DIP-33137-H02|Caroline Chimeno|BIN Taxonomy Match (Jan 2022)|BOLD:...
Bryophaenocladius|ZSM-DIP-33144-F05|Caroline Chimeno|BIN Taxonomy Match (Jan 2022)|BOLD:...
Bryophaenocladius sp. 4ES|ZSM-DIP-33138-D02|Caroline Chimeno|BIN Taxonomy Match (Jan 2022)|...
Bryophaenocladius|ZSM-DIP-33142-H04|Caroline Chimeno|BIN Taxonomy Match (Jan 2022)|BOLD:...
Bryophaenocladius|BIOUG42948-F03|Kate Perez|BIN Taxonomy Match below Phylum (Jun 2019)|BOLD...
Bryophaenocladius|ZSM-DIP-33149-F03|Caroline Chimeno|BIN Taxonomy Match (Jan 2022)|BOLD:...
Bryophaenocladius|ZSM-DIP-33136-G11|Caroline Chimeno|BIN Taxonomy Match (Jan 2022)|BOLD:...
Bryophaenocladius|ZSM-DIP-33138-E04|Caroline Chimeno|BIN Taxonomy Match (Jan 2022)|BOLD:...
Bryophaenocladius|ZSM-DIP-33138-E06|Caroline Chimeno|BIN Taxonomy Match (Jan 2022)|BOLD:...
Bryophaenocladius|ZSM-DIP-33140-G04|Caroline Chimeno|BIN Taxonomy Match (Jan 2022)|BOLD:...
Bryophaenocladius|ZSM-DIP-33145-G06|Caroline Chimeno|BIN Taxonomy Match (Jan 2022)|BOLD:...
Bryophaenocladius|ZSM-DIP-33145-H03|Caroline Chimeno|BIN Taxonomy Match (Jan 2022)|BOLD:...
Bryophaenocladius|ZSM-DIP-33147-G02|Caroline Chimeno|BIN Taxonomy Match (Jan 2022)|BOLD:...
Bryophaenocladius|ZSM-DIP-33147-D01|Caroline Chimeno|BIN Taxonomy Match (Jan 2022)|BOLD:...
Bryophaenocladius|ZSM-DIP-33140-H01|Caroline Chimeno|BIN Taxonomy Match (Jan 2022)|BOLD:...
Bryophaenocladius|ZSM-DIP-33140-G05|Caroline Chimeno|BIN Taxonomy Match (Jan 2022)|BOLD:...
Bryophaenocladius|ZSM-DIP-33140-G03|Caroline Chimeno|BIN Taxonomy Match (Jan 2022)|BOLD:...
Bryophaenocladius|ZSM-DIP-33140-G07|Caroline Chimeno|BIN Taxonomy Match (Jan 2022)|BOLD:...
Bryophaenocladius|ZSM-DIP-33140-G06|Caroline Chimeno|BIN Taxonomy Match (Jan 2022)|BOLD:...
Bryophaenocladius|ZSM-DIP-33140-G02|Caroline Chimeno|BIN Taxonomy Match (Jan 2022)|BOLD:...
Bryophaenocladius|ZSM-DIP-33140-G01|Caroline Chimeno|BIN Taxonomy Match (Jan 2022)|BOLD:...
Bryophaenocladius|ZSM-DIP-33140-F12|Caroline Chimeno|BIN Taxonomy Match (Jan 2022)|BOLD:...
Bryophaenocladius|ZSM-DIP-33140-F10|Caroline Chimeno|BIN Taxonomy Match (Jan 2022)|BOLD:...
Bryophaenocladius sp. 4ES|ZSM-DIP-33139-B12|Caroline Chimeno|BIN Taxonomy Match (Jan 2022)|...
Bryophaenocladius|ZSM-DIP-33139-B08|Caroline Chimeno|BIN Taxonomy Match (Jan 2022)|BOLD:...
Bryophaenocladius|ZSM-DIP-33139-B07|Caroline Chimeno|BIN Taxonomy Match (Jan 2022)|BOLD:...
Bryophaenocladius|ZSM-DIP-33138-D12|Caroline Chimeno|BIN Taxonomy Match (Jan 2022)|BOLD:...
Bryophaenocladius|ZSM-DIP-33138-D01|Caroline Chimeno|BIN Taxonomy Match (Jan 2022)|BOLD:...
Bryophaenocladius|ZSM-DIP-33136-H01|Caroline Chimeno|BIN Taxonomy Match (Jan 2022)|BOLD:...
Bryophaenocladius|ZSM-DIP-33136-G06|Caroline Chimeno|BIN Taxonomy Match (Jan 2022)|BOLD:...
Bryophaenocladius|ZSM-DIP-33135-B08|Caroline Chimeno|BIN Taxonomy Match (Jan 2022)|BOLD:...
Bryophaenocladius|ZSM-DIP-33135-A11|Caroline Chimeno|BIN Taxonomy Match (Jan 2022)|BOLD:...
Bryophaenocladius|ZSM-DIP-33135-A10|Caroline Chimeno|BIN Taxonomy Match (Jan 2022)|BOLD:...
Bryophaenocladius|ZSM-DIP-33142-H05|Caroline Chimeno|BIN Taxonomy Match (Jan 2022)|BOLD:...
Bryophaenocladius|ZSM-DIP-33142-G12|Caroline Chimeno|BIN Taxonomy Match (Jan 2022)|BOLD:...
Bryophaenocladius|ZSM-DIP-33142-G11|Caroline Chimeno|BIN Taxonomy Match (Jan 2022)|BOLD:...
Bryophaenocladius|ZSM-DIP-33133-G09|Caroline Chimeno|BIN Taxonomy Match (Jan 2022)|BOLD:...
Bryophaenocladius|ZSM-DIP-33133-C03|Caroline Chimeno|BIN Taxonomy Match (Jan 2022)|BOLD:...
Bryophaenocladius|ZSM-DIP-33133-B01|Caroline Chimeno|BIN Taxonomy Match (Jan 2022)|BOLD:...
Bryophaenocladius sp. 4ES|ZSM-DIP-33138-D09|Caroline Chimeno|BIN Taxonomy Match (Jan 2022)|...
Bryophaenocladius|ZSM-DIP-33137-H06|Caroline Chimeno|BIN Taxonomy Match (Jan 2022)|BOLD:...
Bryophaenocladius|ZSM-DIP-33135-B03|Caroline Chimeno|BIN Taxonomy Match (Jan 2022)|BOLD:...
Bryophaenocladius|ZSM-DIP-33135-A12|Caroline Chimeno|BIN Taxonomy Match (Jan 2022)|BOLD:...
Bryophaenocladius|ZSM-DIP-33140-H02|Caroline Chimeno|BIN Taxonomy Match (Jan 2022)|BOLD:...
Bryophaenocladius|ZSM-DIP-33140-G11|Caroline Chimeno|BIN Taxonomy Match (Jan 2022)|BOLD:...
Bryophaenocladius|ZSM-DIP-33140-G09|Caroline Chimeno|BIN Taxonomy Match (Jan 2022)|BOLD:...
Bryophaenocladius|ZSM-DIP-33140-G08|Caroline Chimeno|BIN Taxonomy Match (Jan 2022)|BOLD:...
Bryophaenocladius|ZSM-DIP-33128-B09|Caroline Chimeno|BIN Taxonomy Match (Jan 2022)|BOLD:...
Bryophaenocladius|ZSM-DIP-33128-B08|Caroline Chimeno|BIN Taxonomy Match (Jan 2022)|BOLD:...

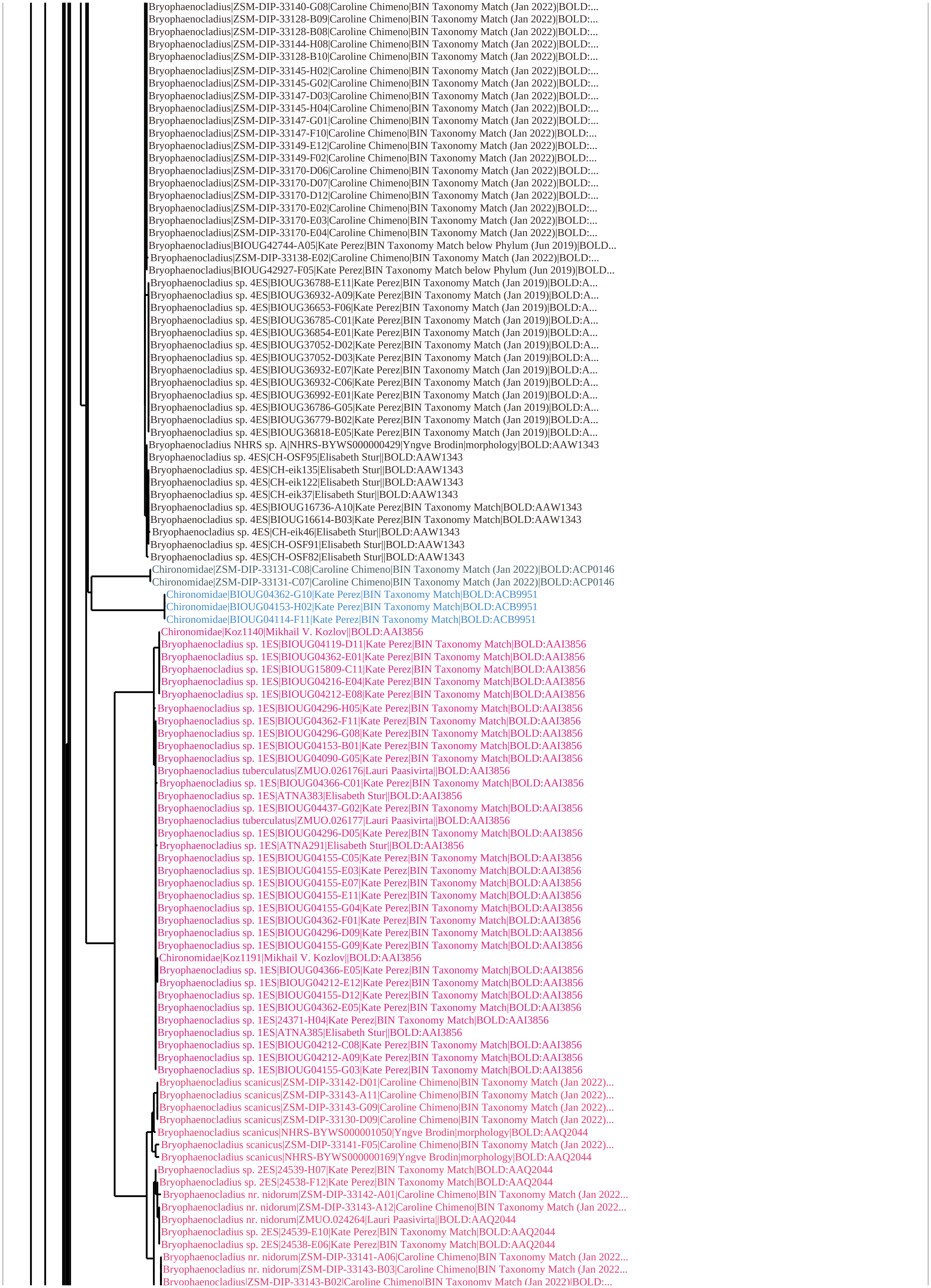
Bryophaenocladius|ZSM-DIP-33140-G08|Caroline Chimeno|BIN Taxonomy Match (Jan 2022)|BOLD:...
Bryophaenocladius|ZSM-DIP-33128-B09|Caroline Chimeno|BIN Taxonomy Match (Jan 2022)|BOLD:...
Bryophaenocladius|ZSM-DIP-33128-B08|Caroline Chimeno|BIN Taxonomy Match (Jan 2022)|BOLD:...
Bryophaenocladius|ZSM-DIP-33144-H08|Caroline Chimeno|BIN Taxonomy Match (Jan 2022)|BOLD:...
Bryophaenocladius|ZSM-DIP-33128-B10|Caroline Chimeno|BIN Taxonomy Match (Jan 2022)|BOLD:...
Bryophaenocladius|ZSM-DIP-33145-H02|Caroline Chimeno|BIN Taxonomy Match (Jan 2022)|BOLD:...
Bryophaenocladius|ZSM-DIP-33145-G02|Caroline Chimeno|BIN Taxonomy Match (Jan 2022)|BOLD:...
Bryophaenocladius|ZSM-DIP-33147-D03|Caroline Chimeno|BIN Taxonomy Match (Jan 2022)|BOLD:...
Bryophaenocladius|ZSM-DIP-33145-H04|Caroline Chimeno|BIN Taxonomy Match (Jan 2022)|BOLD:...
Bryophaenocladius|ZSM-DIP-33147-G01|Caroline Chimeno|BIN Taxonomy Match (Jan 2022)|BOLD:...
Bryophaenocladius|ZSM-DIP-33147-F10|Caroline Chimeno|BIN Taxonomy Match (Jan 2022)|BOLD:...
Bryophaenocladius|ZSM-DIP-33149-E12|Caroline Chimeno|BIN Taxonomy Match (Jan 2022)|BOLD:...
Bryophaenocladius|ZSM-DIP-33149-F02|Caroline Chimeno|BIN Taxonomy Match (Jan 2022)|BOLD:...
Bryophaenocladius|ZSM-DIP-33170-D06|Caroline Chimeno|BIN Taxonomy Match (Jan 2022)|BOLD:...
Bryophaenocladius|ZSM-DIP-33170-D07|Caroline Chimeno|BIN Taxonomy Match (Jan 2022)|BOLD:...
Bryophaenocladius|ZSM-DIP-33170-D12|Caroline Chimeno|BIN Taxonomy Match (Jan 2022)|BOLD:...
Bryophaenocladius|ZSM-DIP-33170-E02|Caroline Chimeno|BIN Taxonomy Match (Jan 2022)|BOLD:...
Bryophaenocladius|ZSM-DIP-33170-E03|Caroline Chimeno|BIN Taxonomy Match (Jan 2022)|BOLD:...
Bryophaenocladius|ZSM-DIP-33170-E04|Caroline Chimeno|BIN Taxonomy Match (Jan 2022)|BOLD:...
Bryophaenocladius|BIOUG42744-A05|Kate Perez|BIN Taxonomy Match below Phylum (Jun 2019)|BOLD...
Bryophaenocladius|ZSM-DIP-33138-E02|Caroline Chimeno|BIN Taxonomy Match (Jan 2022)|BOLD:...
Bryophaenocladius|BIOUG42927-F05|Kate Perez|BIN Taxonomy Match below Phylum (Jun 2019)|BOLD...
Bryophaenocladius sp. 4ES|BIOUG36788-E11|Kate Perez|BIN Taxonomy Match (Jan 2019)|BOLD:A...
Bryophaenocladius sp. 4ES|BIOUG36932-A09|Kate Perez|BIN Taxonomy Match (Jan 2019)|BOLD:A...
Bryophaenocladius sp. 4ES|BIOUG36653-F06|Kate Perez|BIN Taxonomy Match (Jan 2019)|BOLD:A...
Bryophaenocladius sp. 4ES|BIOUG36785-C01|Kate Perez|BIN Taxonomy Match (Jan 2019)|BOLD:A...
Bryophaenocladius sp. 4ES|BIOUG36854-E01|Kate Perez|BIN Taxonomy Match (Jan 2019)|BOLD:A...
Bryophaenocladius sp. 4ES|BIOUG37052-D02|Kate Perez|BIN Taxonomy Match (Jan 2019)|BOLD:A...
Bryophaenocladius sp. 4ES|BIOUG37052-D03|Kate Perez|BIN Taxonomy Match (Jan 2019)|BOLD:A...
Bryophaenocladius sp. 4ES|BIOUG36932-E07|Kate Perez|BIN Taxonomy Match (Jan 2019)|BOLD:A...
Bryophaenocladius sp. 4ES|BIOUG36932-C06|Kate Perez|BIN Taxonomy Match (Jan 2019)|BOLD:A...
Bryophaenocladius sp. 4ES|BIOUG36992-E01|Kate Perez|BIN Taxonomy Match (Jan 2019)|BOLD:A...
Bryophaenocladius sp. 4ES|BIOUG36786-G05|Kate Perez|BIN Taxonomy Match (Jan 2019)|BOLD:A...
Bryophaenocladius sp. 4ES|BIOUG36779-B02|Kate Perez|BIN Taxonomy Match (Jan 2019)|BOLD:A...
Bryophaenocladius sp. 4ES|BIOUG36818-E05|Kate Perez|BIN Taxonomy Match (Jan 2019)|BOLD:A...
Bryophaenocladius NHRS sp. A|NHRS-BYWS000000429|Yngve Brodin|morphology|BOLD:AAW1343
Bryophaenocladius sp. 4ES|CH-OSF95|Elisabeth Stur||BOLD:AAW1343
Bryophaenocladius sp. 4ES|CH-eik135|Elisabeth Stur||BOLD:AAW1343
Bryophaenocladius sp. 4ES|CH-eik122|Elisabeth Stur||BOLD:AAW1343
Bryophaenocladius sp. 4ES|CH-eik37|Elisabeth Stur||BOLD:AAW1343
Bryophaenocladius sp. 4ES|BIOUG16736-A10|Kate Perez|BIN Taxonomy Match|BOLD:AAW1343
Bryophaenocladius sp. 4ES|BIOUG16614-B03|Kate Perez|BIN Taxonomy Match|BOLD:AAW1343
Bryophaenocladius sp. 4ES|CH-eik46|Elisabeth Stur||BOLD:AAW1343
Bryophaenocladius sp. 4ES|CH-OSF91|Elisabeth Stur||BOLD:AAW1343
Bryophaenocladius sp. 4ES|CH-OSF82|Elisabeth Stur||BOLD:AAW1343
Chironomidae|ZSM-DIP-33131-C08|Caroline Chimeno|BIN Taxonomy Match (Jan 2022)|BOLD:ACP0146
Chironomidae|ZSM-DIP-33131-C07|Caroline Chimeno|BIN Taxonomy Match (Jan 2022)|BOLD:ACP0146
Chironomidae|BIOUG04362-G10|Kate Perez|BIN Taxonomy Match|BOLD:ACB9951
Chironomidae|BIOUG04153-H02|Kate Perez|BIN Taxonomy Match|BOLD:ACB9951
Chironomidae|BIOUG04114-F11|Kate Perez|BIN Taxonomy Match|BOLD:ACB9951
Chironomidae|Koz1140|Mikhail V. Kozlov||BOLD:AAI3856
Bryophaenocladius sp. 1ES|BIOUG04119-D11|Kate Perez|BIN Taxonomy Match|BOLD:AAI3856
Bryophaenocladius sp. 1ES|BIOUG04362-E01|Kate Perez|BIN Taxonomy Match|BOLD:AAI3856
Bryophaenocladius sp. 1ES|BIOUG15809-C11|Kate Perez|BIN Taxonomy Match|BOLD:AAI3856
Bryophaenocladius sp. 1ES|BIOUG04216-E04|Kate Perez|BIN Taxonomy Match|BOLD:AAI3856
Bryophaenocladius sp. 1ES|BIOUG04212-E08|Kate Perez|BIN Taxonomy Match|BOLD:AAI3856
Bryophaenocladius sp. 1ES|BIOUG04296-H05|Kate Perez|BIN Taxonomy Match|BOLD:AAI3856
Bryophaenocladius sp. 1ES|BIOUG04362-F11|Kate Perez|BIN Taxonomy Match|BOLD:AAI3856
Bryophaenocladius sp. 1ES|BIOUG04296-G08|Kate Perez|BIN Taxonomy Match|BOLD:AAI3856
Bryophaenocladius sp. 1ES|BIOUG04153-B01|Kate Perez|BIN Taxonomy Match|BOLD:AAI3856
Bryophaenocladius sp. 1ES|BIOUG04090-G05|Kate Perez|BIN Taxonomy Match|BOLD:AAI3856
Bryophaenocladius tuberculatus|ZMUO.026176|Lauri Paasivirta||BOLD:AAI3856
Bryophaenocladius sp. 1ES|BIOUG04366-C01|Kate Perez|BIN Taxonomy Match|BOLD:AAI3856
Bryophaenocladius sp. 1ES|ATNA383|Elisabeth Stur||BOLD:AAI3856
Bryophaenocladius sp. 1ES|BIOUG04437-G02|Kate Perez|BIN Taxonomy Match|BOLD:AAI3856
Bryophaenocladius tuberculatus|ZMUO.026177|Lauri Paasivirta||BOLD:AAI3856
Bryophaenocladius sp. 1ES|BIOUG04296-D05|Kate Perez|BIN Taxonomy Match|BOLD:AAI3856
Bryophaenocladius sp. 1ES|ATNA291|Elisabeth Stur||BOLD:AAI3856
Bryophaenocladius sp. 1ES|BIOUG04155-C05|Kate Perez|BIN Taxonomy Match|BOLD:AAI3856
Bryophaenocladius sp. 1ES|BIOUG04155-E03|Kate Perez|BIN Taxonomy Match|BOLD:AAI3856
Bryophaenocladius sp. 1ES|BIOUG04155-E07|Kate Perez|BIN Taxonomy Match|BOLD:AAI3856
Bryophaenocladius sp. 1ES|BIOUG04155-E11|Kate Perez|BIN Taxonomy Match|BOLD:AAI3856
Bryophaenocladius sp. 1ES|BIOUG04155-G04|Kate Perez|BIN Taxonomy Match|BOLD:AAI3856
Bryophaenocladius sp. 1ES|BIOUG04362-F01|Kate Perez|BIN Taxonomy Match|BOLD:AAI3856
Bryophaenocladius sp. 1ES|BIOUG04296-D09|Kate Perez|BIN Taxonomy Match|BOLD:AAI3856
Bryophaenocladius sp. 1ES|BIOUG04155-G09|Kate Perez|BIN Taxonomy Match|BOLD:AAI3856
Chironomidae|Koz1191|Mikhail V. Kozlov||BOLD:AAI3856
Bryophaenocladius sp. 1ES|BIOUG04366-E05|Kate Perez|BIN Taxonomy Match|BOLD:AAI3856
Bryophaenocladius sp. 1ES|BIOUG04212-E12|Kate Perez|BIN Taxonomy Match|BOLD:AAI3856
Bryophaenocladius sp. 1ES|BIOUG04155-D12|Kate Perez|BIN Taxonomy Match|BOLD:AAI3856
Bryophaenocladius sp. 1ES|BIOUG04362-E05|Kate Perez|BIN Taxonomy Match|BOLD:AAI3856
Bryophaenocladius sp. 1ES|24371-H04|Kate Perez|BIN Taxonomy Match|BOLD:AAI3856
Bryophaenocladius sp. 1ES|ATNA385|Elisabeth Stur||BOLD:AAI3856
Bryophaenocladius sp. 1ES|BIOUG04212-C08|Kate Perez|BIN Taxonomy Match|BOLD:AAI3856
Bryophaenocladius sp. 1ES|BIOUG04212-A09|Kate Perez|BIN Taxonomy Match|BOLD:AAI3856
Bryophaenocladius sp. 1ES|BIOUG04155-G03|Kate Perez|BIN Taxonomy Match|BOLD:AAI3856
Bryophaenocladius scanicus|ZSM-DIP-33142-D01|Caroline Chimeno|BIN Taxonomy Match (Jan 2022)...
Bryophaenocladius scanicus|ZSM-DIP-33143-A11|Caroline Chimeno|BIN Taxonomy Match (Jan 2022)...
Bryophaenocladius scanicus|ZSM-DIP-33143-G09|Caroline Chimeno|BIN Taxonomy Match (Jan 2022)...
Bryophaenocladius scanicus|ZSM-DIP-33130-D09|Caroline Chimeno|BIN Taxonomy Match (Jan 2022)...
Bryophaenocladius scanicus|NHRS-BYWS000001050|Yngve Brodin|morphology|BOLD:AAQ2044
Bryophaenocladius scanicus|ZSM-DIP-33141-F05|Caroline Chimeno|BIN Taxonomy Match (Jan 2022)...
Bryophaenocladius scanicus|NHRS-BYWS000000169|Yngve Brodin|morphology|BOLD:AAQ2044
Bryophaenocladius sp. 2ES|24539-H07|Kate Perez|BIN Taxonomy Match|BOLD:AAQ2044
Bryophaenocladius sp. 2ES|24538-F12|Kate Perez|BIN Taxonomy Match|BOLD:AAQ2044
Bryophaenocladius nr. nidorum|ZSM-DIP-33142-A01|Caroline Chimeno|BIN Taxonomy Match (Jan 2022...
Bryophaenocladius nr. nidorum|ZSM-DIP-33143-A12|Caroline Chimeno|BIN Taxonomy Match (Jan 2022...
Bryophaenocladius nr. nidorum|ZMUO.024264|Lauri Paasivirta||BOLD:AAQ2044
Bryophaenocladius sp. 2ES|24539-E10|Kate Perez|BIN Taxonomy Match|BOLD:AAQ2044
Bryophaenocladius sp. 2ES|24538-E06|Kate Perez|BIN Taxonomy Match|BOLD:AAQ2044
Bryophaenocladius nr. nidorum|ZSM-DIP-33141-A06|Caroline Chimeno|BIN Taxonomy Match (Jan 2022...
Bryophaenocladius nr. nidorum|ZSM-DIP-33143-B03|Caroline Chimeno|BIN Taxonomy Match (Jan 2022...
Bryophaenocladius|ZSM-DIP-33143-B02|Caroline Chimeno|BIN Taxonomy Match (Jan 2022)|BOLD:...

Bryophaenocladius nr. nidorum|ZSM-DIP-33141-A06|Caroline Chimeno|BIN Taxonomy Match (Jan 2022...
Bryophaenocladius nr. nidorum|ZSM-DIP-33143-B03|Caroline Chimeno|BIN Taxonomy Match (Jan 2022...
Bryophaenocladius|ZSM-DIP-33143-B02|Caroline Chimeno|BIN Taxonomy Match (Jan 2022)|BOLD:...
Bryophaenocladius|ZSM-DIP-33143-B01|Caroline Chimeno|BIN Taxonomy Match (Jan 2022)|BOLD:...
Bryophaenocladius nr. nidorum|ZSM-DIP-33142-C12|Caroline Chimeno|BIN Taxonomy Match (Jan 2022...
Bryophaenocladius nr. nidorum|ZSM-DIP-33142-C06|Caroline Chimeno|BIN Taxonomy Match (Jan 2022...
Bryophaenocladius nr. nidorum|ZSM-DIP-33142-B12|Caroline Chimeno|BIN Taxonomy Match (Jan 2022...
Bryophaenocladius sp. 2ES|24537-B08|Kate Perez|BIN Taxonomy Match|BOLD:AAQ2044
Bryophaenocladius sp. 2ES|Finnmark606|Elisabeth Stur||BOLD:AAQ2044
Chironomidae|BIOUG42721-H04|Valerie Levesque-Beaudin|Morphology (Jun 2021)|BOLD:ADW6866
Orthocladius holsatus|PP0278|Lauri Paasivirta||BOLD:ADY7865
Orthocladius sp. 17ES|TRD-CH49|Elisabeth Stur|morphology|BOLD:ACK6243
Orthocladius sp. 17ES|RIN_CH65|Elisabeth Stur|morphology|BOLD:ACK6243
Psectrocladius psilopterus|ZMUO.026225|Lauri Paasivirta||BOLD:AAX8186
Psectrocladius octomaculatus|NHRS-BYWS000001164|Yngve Brodin|morphology|BOLD:AAX8186
Psectrocladius octomaculatus|NHRS-BYWS000001851|Yngve Brodin||BOLD:AAX8186
Psectrocladius psilopterus|ZMUO.026226|Lauri Paasivirta||BOLD:AAX8186
Psectrocladius|BIOUG43949-A04|Kate Perez|BIN Taxonomy Match below Phylum (Jan 2019)|BOLD ...
Psectrocladius psilopterus|NO61|Elisabeth Stur||BOLD:AAX8186
Psectrocladius limbatellus|ZMUO.025143|Lauri Paasivirta||BOLD:AAK9986
Psectrocladius sordidellus|ZMUO.024630|Lauri Paasivirta||BOLD:AAK9986
Psectrocladius limbatellus|ZMUO.025144|Lauri Paasivirta||BOLD:AAK9986
Psectrocladius sordidellus|ZMUO.024629|Lauri Paasivirta||BOLD:AAK9986
Psectrocladius sp. 4TE|Finnmark195|Torbjorn Ekrem||BOLD:AAK9986
Psectrocladius sp. 7TE|Finnmark670|Torbjorn Ekrem||BOLD:AAL9179
Psectrocladius sp. 7TE|Finnmark86|Torbjorn Ekrem||BOLD:AAL9179
Psectrocladius sp. 7TE|Finnmark432|Torbjorn Ekrem||BOLD:AAL9179
Psectrocladius sp. 7TE|Finnmark398|Torbjorn Ekrem||BOLD:AAL9179
Psectrocladius sp. 7TE|Finnmark145|Torbjorn Ekrem||BOLD:AAL9179
Psectrocladius oxyura|NHRS-BYWS000001122|Yngve Brodin|morphology|BOLD:AAL9179
Psectrocladius sp. 7TE|Finnmark91|Torbjorn Ekrem||BOLD:AAL9179
Psectrocladius sp. 7TE|Finnmark39|Torbjorn Ekrem||BOLD:AAL9179
Psectrocladius oxyura|ZMUO.029793|Marko Mutanen||BOLD:AAL9179
Psectrocladius sp. 7TE|Finnmark532|Torbjorn Ekrem||BOLD:AAL9179
Psectrocladius sp. 7TE|Finnmark650|Torbjorn Ekrem||BOLD:AAL9179
Psectrocladius platypus|ES385|Elisabeth Stur||BOLD:AAG5499
Psectrocladius platypus|Finnmark637|Torbjorn Ekrem||BOLD:AAG5499
Psectrocladius obvius|ZMUO.026202|Lauri Paasivirta||BOLD:AAG5499
Psectrocladius platypus|TRD-CH398|Elisabeth Stur|morphology|BOLD:AAG5499
Psectrocladius platypus|Finnmark279|Torbjorn Ekrem||BOLD:AAG5499
Bryophaenocladius|ZSM-DIP-33145-C05|Caroline Chimeno|BIN Taxonomy Match (Jan 2022)|BOLD:...
Bryophaenocladius|ZSM-DIP-33140-F11|Caroline Chimeno|BIN Taxonomy Match (Jan 2022)|BOLD:...
Bryophaenocladius|ZSM-DIP-33139-B01|Caroline Chimeno|BIN Taxonomy Match (Jan 2022)|BOLD:...
Bryophaenocladius|ZSM-DIP-33144-C02|Caroline Chimeno|BIN Taxonomy Match (Jan 2022)|BOLD:...
Chironomidae|ZSM-DIP-33170-E01|Valerie Levesque-Beaudin|BIN Taxonomy Match (Sep 2021)|BOLD...
Chironomidae|ZSM-DIP-33149-F04|Valerie Levesque-Beaudin|BIN Taxonomy Match (Sep 2021)|BOLD...
Chironomidae|ZSM-DIP-33147-D04|Valerie Levesque-Beaudin|BOLD ID Engine Manual (Sep 2021)|BO...
Chironomidae|ZSM-DIP-33146-F02|Valerie Levesque-Beaudin|BIN Taxonomy Match (Sep 2021)|BOLD...
Chironomidae|ZSM-DIP-33136-G05|Valerie Levesque-Beaudin|BIN Taxonomy Match (Sep 2021)|BOLD...
Chironomidae|ZSM-DIP-33133-F09|Valerie Levesque-Beaudin|BOLD ID Engine Manual (Sep 2021)|BO...
Chironomidae|ZSM-DIP-33146-F07|Valerie Levesque-Beaudin|BIN Taxonomy Match (Sep 2021)|BOLD...
Chironomidae|ZSM-DIP-33146-F06|Valerie Levesque-Beaudin|BIN Taxonomy Match (Sep 2021)|BOLD...
Chironomidae|ZSM-DIP-33147-G03|Valerie Levesque-Beaudin|BIN Taxonomy Match (Sep 2021)|BOLD...
Chironomidae|ZSM-DIP-33136-H11|Valerie Levesque-Beaudin|BIN Taxonomy Match (Sep 2021)|BOLD...
Chironomidae|ZSM-DIP-33144-D04|Valerie Levesque-Beaudin|BIN Taxonomy Match (Sep 2021)|BOLD...
Chironomidae|ZSM-DIP-33144-C06|Valerie Levesque-Beaudin|BIN Taxonomy Match (Sep 2021)|BOLD...
Chironomidae|ZSM-DIP-33144-C05|Valerie Levesque-Beaudin|BIN Taxonomy Match (Sep 2021)|BOLD...
Chironomidae|ZSM-DIP-33144-C04|Valerie Levesque-Beaudin|BIN Taxonomy Match (Sep 2021)|BOLD...
Chironomidae|ZSM-DIP-33129-E01|Valerie Levesque-Beaudin|BIN Taxonomy Match (Sep 2021)|BOLD...
Chironomidae|ZSM-DIP-33147-F11|Valerie Levesque-Beaudin|BIN Taxonomy Match (Sep 2021)|BOLD...
Chironomidae|ZSM-DIP-33133-A09|Valerie Levesque-Beaudin|BIN Taxonomy Match (Sep 2021)|BOLD...
Bryophaenocladius|BIOUG42664-E06|Kate Perez|BIN Taxonomy Match below Phylum (Jun 2019)|BOLD...
Bryophaenocladius sp. 8ES|BIOUG36658-H08|Kate Perez|BIN Taxonomy Match (Jan 2019)|BOLD:A...
Bryophaenocladius|BIOUG55187-G11|Stefan Schmidt|BIN Taxonomy Match (Sep 2020)|BOLD:AAG1021
Bryophaenocladius sp. 8ES|BIOUG17094-F01|Kate Perez|BIN Taxonomy Match|BOLD:AAG1021
Bryophaenocladius sp. 8ES|BIOUG17096-A07|Kate Perez|BIN Taxonomy Match|BOLD:AAG1021
Bryophaenocladius sp. 8ES|BIOUG16707-E09|Kate Perez|BIN Taxonomy Match|BOLD:AAG1021
Bryophaenocladius sp. 8ES|BIOUG16996-B10|Kate Perez|BIN Taxonomy Match|BOLD:AAG1021
Bryophaenocladius sp. 8ES|BIOUG36680-E05|Kate Perez|BIN Taxonomy Match (May 2018)|BOLD:A...
Bryophaenocladius sp. 8ES|BIOUG36854-C01|Kate Perez|BIN Taxonomy Match (Jan 2019)|BOLD:A...
Bryophaenocladius sp. 8ES|BIOUG36775-D01|Kate Perez|BIN Taxonomy Match (Jan 2019)|BOLD:A...
Bryophaenocladius sp. 8ES|BIOUG36760-G03|Kate Perez|BIN Taxonomy Match (Jan 2019)|BOLD:A...
Bryophaenocladius sp. 8ES|BIOUG17095-B07|Kate Perez|BIN Taxonomy Match|BOLD:AAG1021
Bryophaenocladius sp. 8ES|BIOUG36776-A06|Kate Perez|BIN Taxonomy Match (Jan 2019)|BOLD:A...
Bryophaenocladius sp. 8ES|BIOUG16893-G11|Kate Perez|BIN Taxonomy Match|BOLD:AAG1021
Bryophaenocladius sp. 8ES|BIOUG36655-F10|Kate Perez|BIN Taxonomy Match (Jan 2019)|BOLD:A...
Bryophaenocladius sp. 8ES|BIOUG16893-H05|Kate Perez|BIN Taxonomy Match|BOLD:AAG1021
Bryophaenocladius sp. 8ES|BIOUG16892-F08|Kate Perez|BIN Taxonomy Match|BOLD:AAG1021
Bryophaenocladius sp. 8ES|BIOUG36847-D02|Kate Perez|BIN Taxonomy Match (Jan 2019)|BOLD:A...
Bryophaenocladius sp. 8ES|BIOUG36625-B12|Kate Perez|BIN Taxonomy Match (Jan 2019)|BOLD:A...
Bryophaenocladius sp. 8ES|BIOUG17094-D07|Kate Perez|BIN Taxonomy Match|BOLD:AAG1021
Chironomidae|ZSM-DIP-33131-C02|Valerie Levesque-Beaudin|BOLD ID Engine Manual (Sep 2021)|BO...
Chironomidae|ZSM-DIP-33132-F11|Valerie Levesque-Beaudin|BIN Taxonomy Match (Sep 2021)|BOLD...
Orthocladiinae|BIOUG15806-B09|Elisabeth Stur|morphology|BOLD:ACP9498
Chironomidae|ZSM-DIP-33143-C09|Valerie Levesque-Beaudin|BOLD ID Engine Manual (Sep 2021)|BO...
Chironomidae|ZSM-DIP-33143-C10|Valerie Levesque-Beaudin|BIN Taxonomy Match (Sep 2021)|BOLD...
Chironomidae|ZSM-DIP-33143-C06|Valerie Levesque-Beaudin|BIN Taxonomy Match (Sep 2021)|BOLD...
Bryophaenocladius subvernalis|ZMUO.024784|Lauri Paasivirta||BOLD:AAW0929
Bryophaenocladius subvernalis|ZMUO.024785|Lauri Paasivirta||BOLD:AAW0929
Bryophaenocladius sp. 6ES|CH-OSF81|Elisabeth Stur||BOLD:AAW0929
Bryophaenocladius sp. 6ES|BIOUG15592-D04|Kate Perez|BIN Taxonomy Match|BOLD:AAW0929
Bryophaenocladius|BIOUG17065-D01|Elisabeth Stur|morphology|BOLD:ACR0319
Bryophaenocladius|BIOUG17025-F10|Elisabeth Stur|morphology|BOLD:ACR0319
Bryophaenocladius|BIOUG17022-D11|Elisabeth Stur|morphology|BOLD:ACR0319
Bryophaenocladius|BIOUG16712-D12|Kate Perez|BOLD ID Engine Manual|BOLD:ACR0319
Metriocnemus|GBOL11557|Sofia Wiedenbrug||BOLD:ACV9941
Metriocnemus|ZSM-DIP-33131-A11|Caroline Chimeno|BIN Taxonomy Match (Jan 2022)|BOLD:ACV9941
Chironomidae|ZSM-DIP-33145-C02|Valerie Levesque-Beaudin|BIN Taxonomy Match (Sep 2021)|BOLD...
Chironomidae|ZSM-DIP-33145-C01|Valerie Levesque-Beaudin|BOLD ID Engine Manual (Sep 2021)|BO...
Bryophaenocladius flexidens|ZMUO.026110|Lauri Paasivirta||BOLD:ACC7917
Bryophaenocladius flexidens|BIOUG04437-E05|Kate Perez|BIN Taxonomy Match (Aug 2017)|BOLD ...
Bryophaenocladius flexidens|BIOUG04437-F07|Kate Perez|BIN Taxonomy Match (Aug 2017)|BOLD ...
Bryophaenocladius flexidens|ZSM-DIP-33141-B03|Caroline Chimeno|BIN Taxonomy Match (Jan 2022...

Bryophaenocladius flexidens|ZMUO.[illegible]|Lauri Paasivirta||BOLD:[illegible]
Bryophaenocladius flexidens|BIOUG04437-E05|Kate Perez|BIN Taxonomy Match (Aug 2017)|BOLD ...
Bryophaenocladius flexidens|BIOUG04437-F07|Kate Perez|BIN Taxonomy Match (Aug 2017)|BOLD ...
Bryophaenocladius flexidens|ZSM-DIP-33141-B03|Caroline Chimeno|BIN Taxonomy Match (Jan 2022 ...
Bryophaenocladius flexidens|ZSM-DIP-33146-B04|Caroline Chimeno|BIN Taxonomy Match (Jan 2022 ...
Bryophaenocladius flexidens|ZSM-DIP-33128-D06|Caroline Chimeno|BIN Taxonomy Match (Jan 2022 ...
Bryophaenocladius flexidens|ZSM-DIP-33128-D05|Caroline Chimeno|BIN Taxonomy Match (Jan 2022 ...
Bryophaenocladius flexidens|ZSM-DIP-33144-E02|Caroline Chimeno|BIN Taxonomy Match (Jan 2022 ...
Bryophaenocladius flexidens|ZSM-DIP-33146-B07|Caroline Chimeno|BIN Taxonomy Match (Jan 2022 ...
Bryophaenocladius flexidens|ZSM-DIP-33146-B06|Caroline Chimeno|BIN Taxonomy Match (Jan 2022 ...
Bryophaenocladius flexidens|ZSM-DIP-33141-B04|Caroline Chimeno|BIN Taxonomy Match (Jan 2022 ...
Bryophaenocladius flexidens|ATNA448|Elisabeth Stur||BOLD:AAE8516
Bryophaenocladius flexidens|ATNA483|Elisabeth Stur||BOLD:AAE8516
Bryophaenocladius flexidens|ATNA447|Elisabeth Stur||BOLD:AAE8516
Bryophaenocladius flexidens|ATNA482|Elisabeth Stur||BOLD:AAE8516
Bryophaenocladius|BIOUG16718-H10|Kate Perez|BOLD ID Engine Manual|BOLD:ACR2905
Bryophaenocladius flexidens|BIOUG16632-B07|Kate Perez|BIN Taxonomy Match|BOLD:AAE8515
Bryophaenocladius flexidens|BIOUG17022-F02|Kate Perez|BIN Taxonomy Match|BOLD:AAE8515
Bryophaenocladius flexidens|BIOUG17023-C06|Kate Perez|BIN Taxonomy Match|BOLD:AAE8515
Bryophaenocladius flexidens|BIOUG17064-C03|Kate Perez|BIN Taxonomy Match|BOLD:AAE8515
Bryophaenocladius flexidens|ATNA350|Elisabeth Stur||BOLD:AAE8515
Bryophaenocladius flexidens|ATNA349|Elisabeth Stur||BOLD:AAE8515
Bryophaenocladius flexidens|BIOUG17023-C08|Kate Perez|BIN Taxonomy Match|BOLD:AAO6000
Bryophaenocladius flexidens|ATNA351|Elisabeth Stur||BOLD:AAO6000
Bryophaenocladius flexidens|BIOUG17022-H09|Kate Perez|BIN Taxonomy Match|BOLD:AAO6000
Bryophaenocladius flexidens|BIOUG16630-H08|Kate Perez|BIN Taxonomy Match|BOLD:AAO6000
Bryophaenocladius flexidens|BIOUG17022-G10|Kate Perez|BIN Taxonomy Match|BOLD:AAO6000
Bryophaenocladius flexidens|BIOUG17025-F04|Kate Perez|BIN Taxonomy Match|BOLD:AAO6000
Bryophaenocladius flexidens|BIOUG16718-G05|Kate Perez|BIN Taxonomy Match|BOLD:AAO6000
Bryophaenocladius flexidens|Finnmark277|Elisabeth Stur||BOLD:AAO6000
Cricotopus cylindraceus|ZMUO.025231|Lauri Paasivirta||BOLD:ADA4272
Cricotopus cylindraceus|ZMUO.025230|Lauri Paasivirta||BOLD:ADA4272
Cricotopus caducus|NHRS-BYWS000001091|Yngve Brodin|morphology|BOLD:ADF1693
Cricotopus caducus|NHRS-BYWS000001088|Yngve Brodin|morphology|BOLD:ADF1693
Cricotopus caducus|ZMUO.025397|Lauri Paasivirta||BOLD:ADF1693
Cricotopus caducus|NHRS-BYWS000000952|Yngve Brodin|morphology|BOLD:ADF1693
Cricotopus caducus|ZMUO.025396|Lauri Paasivirta||BOLD:ADF1693
Cricotopus caducus|NHRS-BYWS000000484|Yngve Brodin|morphology|BOLD:ADF1693
Cricotopus caducus|NHRS-BYWS000000423|Yngve Brodin|morphology|BOLD:ADF1693
Cricotopus caducus|NHRS-BYWS000000422|Yngve Brodin|morphology|BOLD:ADF1693
Cricotopus caducus|NHRS-BYWS000000421|Yngve Brodin|morphology|BOLD:ADF1693
Cricotopus coronatus|Finnmark476|Susan E. Gresens||BOLD:AAV1708
Cricotopus coronatus|Finnmark475|Susan E. Gresens||BOLD:AAV1708
Chironomidae|23878-H07|Mikko J Tiusanen|Tree based identification|BOLD:ACU5263
Cricotopus patens|ZMUO.025150|Lauri Paasivirta||BOLD:ADA5928
Cricotopus patens|ZMUO.025149|Lauri Paasivirta||BOLD:ADA5928
Cricotopus patens|Finnmark467|Susan E. Gresens||BOLD:AAV1705
Cricotopus skirwithensis|BIOUG16551-D07|Kate Perez|BIN Taxonomy Match|BOLD:AAM7071
Cricotopus skirwithensis|BIOUG16434-D09|Kate Perez|BIN Taxonomy Match|BOLD:AAM7071
Cricotopus skirwithensis|BIOUG16430-B12|Kate Perez|BIN Taxonomy Match|BOLD:AAM7071
Cricotopus skirwithensis|BIOUG15536-C05|Kate Perez|BIN Taxonomy Match|BOLD:AAM7071
Cricotopus skirwithensis|BIOUG16331-B12|Kate Perez|BIN Taxonomy Match|BOLD:AAM7071
Cricotopus skirwithensis|BIOUG16257-B04|Kate Perez|BIN Taxonomy Match|BOLD:AAM7071
Cricotopus skirwithensis|BIOUG16434-D02|Kate Perez|BIN Taxonomy Match|BOLD:AAM7071
Cricotopus skirwithensis|BIOUG16477-E08|Kate Perez|BIN Taxonomy Match|BOLD:AAM7071
Cricotopus skirwithensis|BIOUG16435-A05|Kate Perez|BIN Taxonomy Match|BOLD:AAM7071
Cricotopus skirwithensis|BIOUG16330-A04|Kate Perez|BIN Taxonomy Match|BOLD:AAM7071
Cricotopus skirwithensis|BIOUG16328-D11|Kate Perez|BIN Taxonomy Match|BOLD:AAM7071
Cricotopus skirwithensis|BIOUG16258-B04|Kate Perez|BIN Taxonomy Match|BOLD:AAM7071
Cricotopus skirwithensis|BIOUG16434-D10|Kate Perez|BIN Taxonomy Match|BOLD:AAM7071
Cricotopus skirwithensis|BIOUG16551-D08|Kate Perez|BIN Taxonomy Match|BOLD:AAM7071
Cricotopus skirwithensis|BIOUG16331-A04|Kate Perez|BIN Taxonomy Match|BOLD:AAM7071
Cricotopus skirwithensis|BIOUG16296-C11|Kate Perez|BIN Taxonomy Match
Cricotopus skirwithensis|BIOUG16551-E09|Kate Perez|BIN Taxonomy Match|BOLD:AAM7071
Cricotopus skirwithensis|BIOUG16477-B06|Kate Perez|BIN Taxonomy Match|BOLD:AAM7071
Cricotopus skirwithensis|BIOUG16296-F08|Kate Perez|BIN Taxonomy Match|BOLD:AAM7071
Cricotopus skirwithensis|BIOUG16434-A06|Kate Perez|BIN Taxonomy Match|BOLD:AAM7071
Cricotopus skirwithensis|BIOUG16479-F09|Kate Perez|BIN Taxonomy Match|BOLD:AAM7071
Cricotopus skirwithensis|BIOUG16435-C04|Kate Perez|BIN Taxonomy Match|BOLD:AAM7071
Cricotopus skirwithensis|BIOUG16479-G05|Kate Perez|BIN Taxonomy Match|BOLD:AAM7071
Cricotopus skirwithensis|BIOUG16435-H05|Kate Perez|BIN Taxonomy Match|BOLD:AAM7071
Cricotopus skirwithensis|BIOUG16478-D07|Kate Perez|BIN Taxonomy Match|BOLD:AAM7071
Cricotopus skirwithensis|BIOUG16551-G04|Kate Perez|BIN Taxonomy Match|BOLD:AAM7071
Cricotopus skirwithensis|BIOUG16260-H01|Kate Perez|BIN Taxonomy Match|BOLD:AAM7071
Cricotopus skirwithensis|BIOUG16330-E06|Kate Perez|BIN Taxonomy Match|BOLD:AAM7071
Cricotopus skirwithensis|BIOUG16258-E05|Kate Perez|BIN Taxonomy Match|BOLD:AAM7071
Cricotopus skirwithensis|BIOUG16426-H02|Kate Perez|BIN Taxonomy Match|BOLD:AAM7071
Cricotopus skirwithensis|BIOUG16433-E05|Kate Perez|BIN Taxonomy Match|BOLD:AAM7071
Cricotopus skirwithensis|BIOUG16430-G02|Kate Perez|BIN Taxonomy Match|BOLD:AAM7071
Cricotopus skirwithensis|BIOUG16478-H05|Kate Perez|BIN Taxonomy Match|BOLD:AAM7071
Cricotopus skirwithensis|BIOUG16432-E06|Kate Perez|BIN Taxonomy Match|BOLD:AAM7071
Cricotopus skirwithensis|BIOUG16329-C05|Kate Perez|BIN Taxonomy Match|BOLD:AAM7071
Cricotopus skirwithensis|BIOUG16433-C09|Kate Perez|BIN Taxonomy Match|BOLD:AAM7071
Cricotopus skirwithensis|BIOUG16432-D07|Kate Perez|BIN Taxonomy Match|BOLD:AAM7071
Cricotopus skirwithensis|BIOUG16329-H07|Kate Perez|BIN Taxonomy Match|BOLD:AAM7071
Cricotopus skirwithensis|BIOUG16478-D10|Kate Perez|BIN Taxonomy Match|BOLD:AAM7071
Cricotopus skirwithensis|BIOUG16328-C06|Kate Perez|BIN Taxonomy Match|BOLD:AAM7071
Cricotopus skirwithensis|BIOUG16261-E10|Kate Perez|BIN Taxonomy Match|BOLD:AAM7071
Cricotopus skirwithensis|BIOUG16296-C06|Kate Perez|BIN Taxonomy Match|BOLD:AAM7071
Cricotopus skirwithensis|BIOUG16478-B12|Kate Perez|BIN Taxonomy Match|BOLD:AAM7071
Cricotopus skirwithensis|BIOUG16479-B08|Kate Perez|BIN Taxonomy Match|BOLD:AAM7071
Cricotopus skirwithensis|BIOUG16435-G01|Kate Perez|BIN Taxonomy Match|BOLD:AAM7071
Cricotopus skirwithensis|BIOUG16436-H02|Kate Perez|BIN Taxonomy Match|BOLD:AAM7071
Cricotopus skirwithensis|BIOUG16396-E03|Kate Perez|BIN Taxonomy Match|BOLD:AAM7071
Cricotopus skirwithensis|BIOUG16433-F04|Kate Perez|BIN Taxonomy Match|BOLD:AAM7071
Cricotopus skirwithensis|BIOUG16432-E09|Kate Perez|BIN Taxonomy Match|BOLD:AAM7071
Cricotopus skirwithensis|BIOUG16430-F12|Kate Perez|BIN Taxonomy Match|BOLD:AAM7071
Cricotopus skirwithensis|BIOUG16436-C03|Kate Perez|BIN Taxonomy Match|BOLD:AAM7071
Cricotopus skirwithensis|BIOUG16433-A11|Kate Perez|BIN Taxonomy Match|BOLD:AAM7071
Cricotopus skirwithensis|BIOUG16478-E03|Kate Perez|BIN Taxonomy Match|BOLD:AAM7071
Cricotopus skirwithensis|BIOUG15592-D11|Kate Perez|BIN Taxonomy Match|BOLD:AAM7071
Cricotopus skirwithensis|BIOUG16476-B06|Kate Perez|BIN Taxonomy Match|BOLD:AAM7071
Cricotopus skirwithensis|BIOUG16476-C05|Kate Perez|BIN Taxonomy Match|BOLD:AAM7071
[illegible]

Cricotopus skirwithensis|BIOUG15592-D11|Kate Perez|BIN Taxonomy Match|BOLD:AAM7071
Cricotopus skirwithensis|BIOUG16476-B06|Kate Perez|BIN Taxonomy Match|BOLD:AAM7071
Cricotopus skirwithensis|BIOUG16476-C05|Kate Perez|BIN Taxonomy Match|BOLD:AAM7071
Cricotopus skirwithensis|BIOUG16431-H04|Kate Perez|BIN Taxonomy Match|BOLD:AAM7071
Cricotopus skirwithensis|BIOUG16328-E01|Kate Perez|BIN Taxonomy Match|BOLD:AAM7071
Cricotopus skirwithensis|BIOUG16333-C10|Kate Perez|BIN Taxonomy Match|BOLD:AAM7071
Cricotopus skirwithensis|BIOUG16426-F12|Kate Perez|BIN Taxonomy Match|BOLD:AAM7071
Cricotopus skirwithensis|BIOUG16331-E07|Kate Perez|BIN Taxonomy Match|BOLD:AAM7071
Cricotopus skirwithensis|BIOUG16476-C04|Kate Perez|BIN Taxonomy Match|BOLD:AAM7071
Cricotopus skirwithensis|BIOUG16476-A08|Kate Perez|BIN Taxonomy Match|BOLD:AAM7071
Cricotopus skirwithensis|BIOUG15653-C06|Kate Perez|BIN Taxonomy Match|BOLD:AAM7071
Cricotopus skirwithensis|Finnmark31|Elisabeth Stur||BOLD:AAM7071
Cricotopus skirwithensis|BIOUG16259-G12|Kate Perez|BIN Taxonomy Match|BOLD:AAM7071
Cricotopus skirwithensis|BIOUG16552-E04|Kate Perez|BIN Taxonomy Match|BOLD:AAM7071
Cricotopus skirwithensis|BIOUG16477-C04|Kate Perez|BIN Taxonomy Match|BOLD:AAM7071
Cricotopus skirwithensis|BIOUG16479-C03|Kate Perez|BIN Taxonomy Match|BOLD:AAM7071
Cricotopus skirwithensis|Finnmark799|Elisabeth Stur||BOLD:AAM7071
Cricotopus skirwithensis|BIOUG16434-D12|Kate Perez|BIN Taxonomy Match|BOLD:AAM7071
Cricotopus skirwithensis|BIOUG16261-F07|Kate Perez|BIN Taxonomy Match|BOLD:AAM7071
Cricotopus skirwithensis|BIOUG16329-D05|Kate Perez|BIN Taxonomy Match|BOLD:AAM7071
Cricotopus skirwithensis|BIOUG16476-E01|Kate Perez|BIN Taxonomy Match|BOLD:AAM7071
Cricotopus skirwithensis|BIOUG16258-B02|Kate Perez|BIN Taxonomy Match|BOLD:AAM7071
Cricotopus skirwithensis|BIOUG16479-B01|Kate Perez|BIN Taxonomy Match|BOLD:AAM7071
Cricotopus skirwithensis|BIOUG16261-H01|Kate Perez|BIN Taxonomy Match|BOLD:AAM7071
Cricotopus skirwithensis|BIOUG16435-B04|Kate Perez|BIN Taxonomy Match|BOLD:AAM7071
Cricotopus skirwithensis|BIOUG16258-A07|Kate Perez|BIN Taxonomy Match|BOLD:AAM7071
Cricotopus skirwithensis|BIOUG15599-D02|Kate Perez|BIN Taxonomy Match|BOLD:AAM7071
Cricotopus skirwithensis|BIOUG16551-E11|Kate Perez|BIN Taxonomy Match|BOLD:AAM7071
Cricotopus skirwithensis|BIOUG16396-C04|Kate Perez|BIN Taxonomy Match|BOLD:AAM7071
Cricotopus skirwithensis|BIOUG16435-A09|Kate Perez|BIN Taxonomy Match|BOLD:AAM7071
Cricotopus skirwithensis|BIOUG16430-H05|Kate Perez|BIN Taxonomy Match|BOLD:AAM7071
Cricotopus skirwithensis|BIOUG16433-D04|Kate Perez|BIN Taxonomy Match|BOLD:AAM7071
Cricotopus skirwithensis|BIOUG16551-D12|Kate Perez|BIN Taxonomy Match|BOLD:AAM7071
Cricotopus skirwithensis|BIOUG16434-D08|Kate Perez|BIN Taxonomy Match|BOLD:AAM7071
Cricotopus skirwithensis|BIOUG16436-D06|Kate Perez|BIN Taxonomy Match|BOLD:AAM7071
Cricotopus skirwithensis|BIOUG16299-F12|Kate Perez|BIN Taxonomy Match|BOLD:AAM7071
Cricotopus skirwithensis|BIOUG16476-E09|Kate Perez|BIN Taxonomy Match|BOLD:AAM7071
Cricotopus skirwithensis|BIOUG16331-F12|Kate Perez|BIN Taxonomy Match|BOLD:AAM7071
Cricotopus skirwithensis|BIOUG16434-F02|Kate Perez|BIN Taxonomy Match|BOLD:AAM7071
Cricotopus skirwithensis|BIOUG16431-G06|Kate Perez|BIN Taxonomy Match|BOLD:AAM7071
Cricotopus skirwithensis|BIOUG16436-B11|Kate Perez|BIN Taxonomy Match|BOLD:AAM7071
Cricotopus skirwithensis|BIOUG16430-A11|Kate Perez|BIN Taxonomy Match|BOLD:AAM7071
Cricotopus skirwithensis|BIOUG16436-E04|Kate Perez|BIN Taxonomy Match|BOLD:AAM7071
Cricotopus skirwithensis|BIOUG16436-E07|Kate Perez|BIN Taxonomy Match|BOLD:AAM7071
Cricotopus skirwithensis|BIOUG16477-D11|Kate Perez|BIN Taxonomy Match|BOLD:AAM7071
Cricotopus skirwithensis|BIOUG16431-D12|Kate Perez|BIN Taxonomy Match|BOLD:AAM7071
Cricotopus skirwithensis|BIOUG16434-C11|Kate Perez|BIN Taxonomy Match|BOLD:AAM7071
Cricotopus skirwithensis|BIOUG16260-A02|Kate Perez|BIN Taxonomy Match|BOLD:AAM7071
Cricotopus skirwithensis|BIOUG16479-H07|Kate Perez|BIN Taxonomy Match|BOLD:AAM7071
Cricotopus skirwithensis|BIOUG16480-F11|Kate Perez|BIN Taxonomy Match|BOLD:AAM7071
Cricotopus skirwithensis|BIOUG16478-G10|Kate Perez|BIN Taxonomy Match|BOLD:AAM7071
Cricotopus skirwithensis|BIOUG16328-F05|Kate Perez|BIN Taxonomy Match|BOLD:AAM7071
Cricotopus skirwithensis|BIOUG16552-B03|Kate Perez|BIN Taxonomy Match|BOLD:AAM7071
Cricotopus skirwithensis|BIOUG16333-G04|Kate Perez|BIN Taxonomy Match|BOLD:AAM7071
Cricotopus skirwithensis|BIOUG16332-B07|Kate Perez|BIN Taxonomy Match|BOLD:AAM7071
Cricotopus skirwithensis|BIOUG16328-B01|Kate Perez|BIN Taxonomy Match|BOLD:AAM7071
Cricotopus skirwithensis|BIOUG15653-G04|Kate Perez|BIN Taxonomy Match|BOLD:AAM7071
Cricotopus skirwithensis|BIOUG16431-D11|Kate Perez|BIN Taxonomy Match|BOLD:AAM7071
Cricotopus skirwithensis|BIOUG16476-F10|Kate Perez|BIN Taxonomy Match|BOLD:AAM7071
Cricotopus skirwithensis|BIOUG16331-C04|Kate Perez|BIN Taxonomy Match|BOLD:AAM7071
Cricotopus skirwithensis|BIOUG15654-F02|Kate Perez|BIN Taxonomy Match|BOLD:AAM7071
Cricotopus skirwithensis|BIOUG16435-F04|Kate Perez|BIN Taxonomy Match|BOLD:AAM7071
Cricotopus skirwithensis|BIOUG16433-D07|Kate Perez|BIN Taxonomy Match|BOLD:AAM7071
Cricotopus skirwithensis|BIOUG16478-B09|Kate Perez|BIN Taxonomy Match|BOLD:AAM7071
Cricotopus skirwithensis|BIOUG16433-H07|Kate Perez|BIN Taxonomy Match|BOLD:AAM7071
Cricotopus skirwithensis|BIOUG16430-F09|Kate Perez|BIN Taxonomy Match|BOLD:AAM7071
Cricotopus skirwithensis|BIOUG16331-H02|Kate Perez|BIN Taxonomy Match|BOLD:AAM7071
Cricotopus skirwithensis|BIOUG16476-G07|Kate Perez|BIN Taxonomy Match|BOLD:AAM7071
Cricotopus skirwithensis|BIOUG16257-G04|Kate Perez|BIN Taxonomy Match|BOLD:AAM7071
Cricotopus skirwithensis|BIOUG16434-F06|Kate Perez|BIN Taxonomy Match|BOLD:AAM7071
Cricotopus skirwithensis|BIOUG16432-C04|Kate Perez|BIN Taxonomy Match|BOLD:AAM7071
Cricotopus skirwithensis|BIOUG16434-G09|Kate Perez|BIN Taxonomy Match|BOLD:AAM7071
Cricotopus skirwithensis|BIOUG16552-D04|Kate Perez|BIN Taxonomy Match|BOLD:AAM7071
Cricotopus skirwithensis|BIOUG16551-H02|Kate Perez|BIN Taxonomy Match|BOLD:AAM7071
Cricotopus skirwithensis|BIOUG16260-G05|Kate Perez|BIN Taxonomy Match|BOLD:AAM7071
Cricotopus skirwithensis|BIOUG16431-F09|Kate Perez|BIN Taxonomy Match|BOLD:AAM7071
Cricotopus skirwithensis|BIOUG16258-G03|Kate Perez|BIN Taxonomy Match|BOLD:AAM7071
Cricotopus skirwithensis|BIOUG16431-B10|Kate Perez|BIN Taxonomy Match|BOLD:AAM7071
Cricotopus skirwithensis|BIOUG16479-G09|Kate Perez|BIN Taxonomy Match|BOLD:AAM7071
Cricotopus skirwithensis|BIOUG16480-G02|Kate Perez|BIN Taxonomy Match|BOLD:AAM7071
Cricotopus skirwithensis|BIOUG16258-D11|Kate Perez|BIN Taxonomy Match|BOLD:AAM7071
Cricotopus skirwithensis|BIOUG16478-C08|Kate Perez|BIN Taxonomy Match|BOLD:AAM7071
Cricotopus skirwithensis|BIOUG16436-A12|Kate Perez|BIN Taxonomy Match|BOLD:AAM7071
Cricotopus skirwithensis|BIOUG16297-B01|Kate Perez|BIN Taxonomy Match|BOLD:AAM7071
Cricotopus skirwithensis|BIOUG16396-C07|Kate Perez|BIN Taxonomy Match|BOLD:AAM7071
Cricotopus skirwithensis|BIOUG16478-A05|Kate Perez|BIN Taxonomy Match|BOLD:AAM7071
Cricotopus skirwithensis|BIOUG16433-A08|Kate Perez|BIN Taxonomy Match|BOLD:AAM7071
Cricotopus skirwithensis|BIOUG16430-C10|Kate Perez|BIN Taxonomy Match|BOLD:AAM7071
Cricotopus skirwithensis|BIOUG16299-E11|Kate Perez|BIN Taxonomy Match|BOLD:AAM7071
Cricotopus skirwithensis|BIOUG16552-F11|Kate Perez|BIN Taxonomy Match|BOLD:AAM7071
Cricotopus skirwithensis|BIOUG16331-H06|Kate Perez|BIN Taxonomy Match|BOLD:AAM7071
Cricotopus skirwithensis|BIOUG16434-A11|Kate Perez|BIN Taxonomy Match|BOLD:AAM7071
Cricotopus skirwithensis|BIOUG16479-H01|Kate Perez|BIN Taxonomy Match|BOLD:AAM7071
Cricotopus skirwithensis|BIOUG16431-B02|Kate Perez|BIN Taxonomy Match|BOLD:AAM7071
Cricotopus skirwithensis|BIOUG16298-E02|Kate Perez|BIN Taxonomy Match|BOLD:AAM7071
Cricotopus skirwithensis|BIOUG16436-B05|Kate Perez|BIN Taxonomy Match|BOLD:AAM7071
Cricotopus skirwithensis|BIOUG16476-B07|Kate Perez|BIN Taxonomy Match|BOLD:AAM7071
Cricotopus skirwithensis|BIOUG16478-G01|Kate Perez|BIN Taxonomy Match|BOLD:AAM7071
Cricotopus skirwithensis|BIOUG16480-B12|Kate Perez|BIN Taxonomy Match|BOLD:AAM7071
Cricotopus skirwithensis|BIOUG15592-G06|Kate Perez|BIN Taxonomy Match|BOLD:AAM7071
Cricotopus skirwithensis|BIOUG16479-H04|Kate Perez|BIN Taxonomy Match|BOLD:AAM7071
Cricotopus skirwithensis|BIOUG16297-E12|Elisabeth Stur|Tree based identification|BOLD:AAM7071
Cricotopus skirwithensis|BIOUG16329-D07|Kate Perez|BIN Taxonomy Match|BOLD:AAM7071

Cricotopus skirwithensis|BIOUG16479-H04|Kate Perez|BIN Taxonomy Match|BOLD:AAM7071
Cricotopus skirwithensis|BIOUG16297-E12|Elisabeth Stur|Tree based identification|BOLD:AAM7071
Cricotopus skirwithensis|BIOUG16329-D07|Kate Perez|BIN Taxonomy Match|BOLD:AAM7071
Cricotopus skirwithensis|BIOUG16552-G08|Kate Perez|BIN Taxonomy Match|BOLD:AAM7071
Cricotopus skirwithensis|BIOUG16259-D07|Kate Perez|BIN Taxonomy Match|BOLD:AAM7071
Cricotopus skirwithensis|BIOUG16552-C09|Kate Perez|BIN Taxonomy Match|BOLD:AAM7071
Cricotopus skirwithensis|BIOUG16332-H05|Kate Perez|BIN Taxonomy Match|BOLD:AAM7071
Cricotopus skirwithensis|BIOUG16328-E03|Kate Perez|BIN Taxonomy Match|BOLD:AAM7071
Cricotopus skirwithensis|BIOUG16434-H09|Kate Perez|BIN Taxonomy Match|BOLD:AAM7071
Cricotopus skirwithensis|BIOUG16298-A11|Kate Perez|BIN Taxonomy Match|BOLD:AAM7071
Cricotopus skirwithensis|BIOUG16328-D07|Kate Perez|BIN Taxonomy Match|BOLD:AAM7071
Cricotopus skirwithensis|BIOUG16426-H07|Kate Perez|BIN Taxonomy Match|BOLD:AAM7071
Cricotopus skirwithensis|BIOUG16299-F11|Kate Perez|BIN Taxonomy Match|BOLD:AAM7071
Cricotopus skirwithensis|BIOUG16476-C03|Kate Perez|BIN Taxonomy Match|BOLD:AAM7071
Cricotopus skirwithensis|BIOUG16477-G12|Kate Perez|BIN Taxonomy Match|BOLD:AAM7071
Cricotopus skirwithensis|BIOUG16434-D05|Kate Perez|BIN Taxonomy Match|BOLD:AAM7071
Cricotopus skirwithensis|BIOUG16476-A12|Kate Perez|BIN Taxonomy Match|BOLD:AAM7071
Cricotopus skirwithensis|BIOUG16328-B07|Kate Perez|BIN Taxonomy Match|BOLD:AAM7071
Cricotopus skirwithensis|BIOUG16298-F06|Kate Perez|BIN Taxonomy Match|BOLD:AAM7071
Cricotopus skirwithensis|BIOUG16552-G04|Kate Perez|BIN Taxonomy Match|BOLD:AAM7071
Cricotopus skirwithensis|BIOUG16261-A07|Kate Perez|BIN Taxonomy Match|BOLD:AAM7071
Cricotopus skirwithensis|BIOUG16256-E09|Kate Perez|BIN Taxonomy Match|BOLD:AAM7071
Cricotopus skirwithensis|BIOUG16476-H01|Kate Perez|BIN Taxonomy Match|BOLD:AAM7071
Cricotopus skirwithensis|BIOUG16436-C10|Kate Perez|BIN Taxonomy Match|BOLD:AAM7071
Cricotopus skirwithensis|BIOUG16431-F05|Kate Perez|BIN Taxonomy Match|BOLD:AAM7071
Cricotopus skirwithensis|BIOUG16396-A12|Kate Perez|BIN Taxonomy Match|BOLD:AAM7071
Cricotopus skirwithensis|BIOUG16257-B01|Kate Perez|BIN Taxonomy Match|BOLD:AAM7071
Cricotopus skirwithensis|BIOUG16552-E07|Kate Perez|BIN Taxonomy Match|BOLD:AAM7071
Cricotopus skirwithensis|BIOUG16435-F01|Kate Perez|BIN Taxonomy Match|BOLD:AAM7071
Cricotopus skirwithensis|BIOUG16480-A04|Kate Perez|BIN Taxonomy Match|BOLD:AAM7071
Cricotopus skirwithensis|BIOUG16259-D10|Kate Perez|BIN Taxonomy Match|BOLD:AAM7071
Cricotopus skirwithensis|BIOUG15543-A05|Kate Perez|BIN Taxonomy Match|BOLD:AAM7071
Cricotopus skirwithensis|BIOUG16480-H04|Kate Perez|BIN Taxonomy Match|BOLD:AAM7071
Cricotopus skirwithensis|BIOUG16430-E06|Kate Perez|BIN Taxonomy Match|BOLD:AAM7071
Cricotopus skirwithensis|BIOUG16436-A01|Kate Perez|BIN Taxonomy Match|BOLD:AAM7071
Cricotopus skirwithensis|BIOUG16476-A04|Kate Perez|BIN Taxonomy Match|BOLD:AAM7071
Cricotopus skirwithensis|BIOUG16261-F11|Kate Perez|BIN Taxonomy Match|BOLD:AAM7071
Cricotopus skirwithensis|BIOUG15539-D08|Kate Perez|BIN Taxonomy Match|BOLD:AAM7071
Cricotopus skirwithensis|BIOUG16329-A11|Kate Perez|BIN Taxonomy Match|BOLD:AAM7071
Cricotopus skirwithensis|BIOUG16297-H11|Kate Perez|BIN Taxonomy Match|BOLD:AAM7071
Cricotopus skirwithensis|BIOUG16330-D02|Kate Perez|BIN Taxonomy Match|BOLD:AAM7071
Cricotopus skirwithensis|BIOUG16329-A03|Kate Perez|BIN Taxonomy Match|BOLD:AAM7071
Cricotopus skirwithensis|BIOUG16436-A02|Kate Perez|BIN Taxonomy Match|BOLD:AAM7071
Cricotopus skirwithensis|BIOUG16330-C08|Kate Perez|BIN Taxonomy Match|BOLD:AAM7071
Cricotopus skirwithensis|BIOUG16333-E10|Kate Perez|BIN Taxonomy Match|BOLD:AAM7071
Cricotopus skirwithensis|BIOUG16551-G06|Kate Perez|BIN Taxonomy Match|BOLD:AAM7071
Cricotopus skirwithensis|BIOUG16257-B07|Kate Perez|BIN Taxonomy Match|BOLD:AAM7071
Cricotopus skirwithensis|BIOUG16332-B10|Kate Perez|BIN Taxonomy Match|BOLD:AAM7071
Cricotopus skirwithensis|BIOUG16434-C10|Kate Perez|BIN Taxonomy Match|BOLD:AAM7071
Cricotopus skirwithensis|BIOUG16477-F08|Kate Perez|BIN Taxonomy Match|BOLD:AAM7071
Cricotopus skirwithensis|BIOUG16261-C08|Kate Perez|BIN Taxonomy Match|BOLD:AAM7071
Cricotopus skirwithensis|BIOUG16430-E12|Kate Perez|BIN Taxonomy Match|BOLD:AAM7071
Cricotopus skirwithensis|BIOUG16430-D10|Kate Perez|BIN Taxonomy Match|BOLD:AAM7071
Cricotopus skirwithensis|BIOUG16296-G10|Kate Perez|BIN Taxonomy Match|BOLD:AAM7071
Cricotopus skirwithensis|BIOUG16331-D08|Kate Perez|BIN Taxonomy Match|BOLD:AAM7071
Cricotopus skirwithensis|BIOUG16433-H03|Kate Perez|BIN Taxonomy Match|BOLD:AAM7071
Cricotopus skirwithensis|BIOUG16476-D05|Kate Perez|BIN Taxonomy Match|BOLD:AAM7071
Cricotopus skirwithensis|BIOUG16426-G03|Kate Perez|BIN Taxonomy Match|BOLD:AAM7071
Cricotopus skirwithensis|BIOUG16477-E04|Kate Perez|BIN Taxonomy Match|BOLD:AAM7071
Cricotopus skirwithensis|BIOUG16432-A08|Kate Perez|BIN Taxonomy Match|BOLD:AAM7071
Cricotopus skirwithensis|BIOUG16430-D03|Kate Perez|BIN Taxonomy Match|BOLD:AAM7071
Cricotopus skirwithensis|BIOUG16432-E03|Kate Perez|BIN Taxonomy Match|BOLD:AAM7071
Cricotopus skirwithensis|BIOUG15537-H11|Kate Perez|BIN Taxonomy Match|BOLD:AAM7071
Cricotopus skirwithensis|BIOUG16256-C01|Kate Perez|BIN Taxonomy Match|BOLD:AAM7071
Cricotopus skirwithensis|BIOUG16626-D08|Kate Perez|BIN Taxonomy Match|BOLD:AAM7071
Cricotopus skirwithensis|BIOUG16296-E10|Kate Perez|BIN Taxonomy Match|BOLD:AAM7071
Cricotopus skirwithensis|BIOUG16435-D09|Kate Perez|BIN Taxonomy Match|BOLD:AAM7071
Cricotopus skirwithensis|BIOUG16432-C10|Kate Perez|BIN Taxonomy Match|BOLD:AAM7071
Cricotopus skirwithensis|BIOUG16261-F09|Kate Perez|BIN Taxonomy Match|BOLD:AAM7071
Cricotopus skirwithensis|BIOUG16332-A07|Kate Perez|BIN Taxonomy Match|BOLD:AAM7071
Cricotopus skirwithensis|BIOUG16551-F12|Kate Perez|BIN Taxonomy Match|BOLD:AAM7071
Cricotopus skirwithensis|BIOUG16431-A07|Kate Perez|BIN Taxonomy Match|BOLD:AAM7071
Cricotopus skirwithensis|BIOUG16297-A11|Kate Perez|BIN Taxonomy Match|BOLD:AAM7071
Cricotopus skirwithensis|BIOUG16297-A12|Kate Perez|BIN Taxonomy Match|BOLD:AAM7071
Cricotopus skirwithensis|BIOUG16477-F03|Kate Perez|BIN Taxonomy Match|BOLD:AAM7071
Cricotopus skirwithensis|BIOUG16435-A03|Kate Perez|BIN Taxonomy Match|BOLD:AAM7071
Cricotopus skirwithensis|BIOUG16479-B10|Kate Perez|BIN Taxonomy Match|BOLD:AAM7071
Cricotopus skirwithensis|BIOUG16552-H06|Kate Perez|BIN Taxonomy Match|BOLD:AAM7071
Cricotopus skirwithensis|BIOUG16259-B02|Kate Perez|BIN Taxonomy Match|BOLD:AAM7071
Cricotopus skirwithensis|BIOUG16329-H10|Kate Perez|BIN Taxonomy Match|BOLD:AAM7071
Cricotopus skirwithensis|BIOUG16435-E11|Kate Perez|BIN Taxonomy Match|BOLD:AAM7071
Cricotopus skirwithensis|BIOUG16435-G12|Kate Perez|BIN Taxonomy Match|BOLD:AAM7071
Cricotopus skirwithensis|BIOUG16479-E04|Kate Perez|BIN Taxonomy Match|BOLD:AAM7071
Cricotopus skirwithensis|BIOUG16430-E03|Kate Perez|BIN Taxonomy Match|BOLD:AAM7071
Cricotopus skirwithensis|BIOUG15674-E03|Kate Perez|BIN Taxonomy Match|BOLD:AAM7071
Cricotopus skirwithensis|BIOUG16478-H08|Kate Perez|BIN Taxonomy Match|BOLD:AAM7071
Cricotopus skirwithensis|BIOUG16296-A05|Kate Perez|BIN Taxonomy Match|BOLD:AAM7071
Cricotopus skirwithensis|BIOUG16331-F09|Kate Perez|BIN Taxonomy Match|BOLD:AAM7071
Cricotopus skirwithensis|BIOUG16332-F12|Kate Perez|BIN Taxonomy Match|BOLD:AAM7071
Cricotopus skirwithensis|BIOUG16333-A11|Kate Perez|BIN Taxonomy Match|BOLD:AAM7071
Cricotopus skirwithensis|BIOUG16259-F05|Kate Perez|BIN Taxonomy Match|BOLD:AAM7071
Cricotopus skirwithensis|BIOUG16296-F11|Kate Perez|BIN Taxonomy Match|BOLD:AAM7071
Cricotopus skirwithensis|BIOUG16435-E08|Kate Perez|BIN Taxonomy Match|BOLD:AAM7071
Cricotopus skirwithensis|BIOUG16479-C01|Kate Perez|BIN Taxonomy Match|BOLD:AAM7071
Cricotopus skirwithensis|BIOUG16432-A05|Kate Perez|BIN Taxonomy Match|BOLD:AAM7071
Cricotopus skirwithensis|BIOUG16436-E03|Kate Perez|BIN Taxonomy Match|BOLD:AAM7071
Cricotopus skirwithensis|BIOUG16477-G09|Kate Perez|BIN Taxonomy Match|BOLD:AAM7071
Cricotopus skirwithensis|BIOUG16426-G10|Kate Perez|BIN Taxonomy Match|BOLD:AAM7071
Cricotopus skirwithensis|BIOUG15653-G10|Kate Perez|BIN Taxonomy Match|BOLD:AAM7071
Cricotopus skirwithensis|BIOUG16435-F07|Kate Perez|BIN Taxonomy Match|BOLD:AAM7071
Cricotopus skirwithensis|BIOUG16435-E12|Kate Perez|BIN Taxonomy Match|BOLD:AAM7071
Cricotopus skirwithensis|BIOUG16430-C07|Kate Perez|BIN Taxonomy Match|BOLD:AAM7071
Cricotopus skirwithensis|BIOUG16431-A02|Kate Perez|BIN Taxonomy Match|BOLD:AAM7071

[illegible]
Cricotopus skirwithensis|BIOUG16435-E12|Kate Perez|BIN Taxonomy Match|BOLD:AAM7071
Cricotopus skirwithensis|BIOUG16430-C07|Kate Perez|BIN Taxonomy Match|BOLD:AAM7071
Cricotopus skirwithensis|BIOUG16431-A02|Kate Perez|BIN Taxonomy Match|BOLD:AAM7071
Cricotopus skirwithensis|BIOUG16432-B03|Kate Perez|BIN Taxonomy Match|BOLD:AAM7071
Cricotopus skirwithensis|BIOUG16432-F11|Kate Perez|BIN Taxonomy Match|BOLD:AAM7071
Cricotopus skirwithensis|BIOUG16432-G08|Kate Perez|BIN Taxonomy Match|BOLD:AAM7071
Cricotopus skirwithensis|BIOUG16432-H08|Kate Perez|BIN Taxonomy Match|BOLD:AAM7071
Cricotopus skirwithensis|BIOUG16433-A05|Kate Perez|BIN Taxonomy Match|BOLD:AAM7071
Cricotopus skirwithensis|BIOUG16477-D10|Kate Perez|BIN Taxonomy Match|BOLD:AAM7071
Cricotopus skirwithensis|BIOUG16432-F08|Kate Perez|BIN Taxonomy Match|BOLD:AAM7071
Cricotopus skirwithensis|BIOUG16432-C11|Kate Perez|BIN Taxonomy Match|BOLD:AAM7071
Cricotopus skirwithensis|BIOUG16551-D03|Kate Perez|BIN Taxonomy Match|BOLD:AAM7071
Cricotopus skirwithensis|BIOUG16257-C11|Kate Perez|BIN Taxonomy Match|BOLD:AAM7071
Cricotopus skirwithensis|BIOUG16257-G12|Kate Perez|BIN Taxonomy Match|BOLD:AAM7071
Cricotopus skirwithensis|BIOUG16434-C08|Kate Perez|BIN Taxonomy Match|BOLD:AAM7071
Cricotopus skirwithensis|BIOUG16478-B02|Kate Perez|BIN Taxonomy Match|BOLD:AAM7071
Cricotopus skirwithensis|BIOUG16478-G02|Kate Perez|BIN Taxonomy Match|BOLD:AAM7071
Cricotopus skirwithensis|BIOUG16476-H05|Kate Perez|BIN Taxonomy Match|BOLD:AAM7071
Cricotopus skirwithensis|BIOUG16434-C03|Kate Perez|BIN Taxonomy Match|BOLD:AAM7071
Cricotopus skirwithensis|BIOUG16433-F02|Kate Perez|BIN Taxonomy Match|BOLD:AAM7071
Cricotopus skirwithensis|BIOUG16432-A10|Kate Perez|BIN Taxonomy Match|BOLD:AAM7071
Cricotopus skirwithensis|BIOUG16431-D09|Kate Perez|BIN Taxonomy Match|BOLD:AAM7071
Cricotopus skirwithensis|BIOUG16552-B10|Kate Perez|BIN Taxonomy Match|BOLD:AAM7071
Cricotopus skirwithensis|BIOUG16299-H06|Kate Perez|BIN Taxonomy Match|BOLD:AAM7071
Cricotopus skirwithensis|BIOUG16552-F05|Kate Perez|BIN Taxonomy Match|BOLD:AAM7071
Cricotopus skirwithensis|BIOUG16258-H04|Kate Perez|BIN Taxonomy Match|BOLD:AAM7071
Cricotopus skirwithensis|BIOUG16433-E06|Kate Perez|BIN Taxonomy Match|BOLD:AAM7071
Cricotopus skirwithensis|BIOUG16435-D01|Kate Perez|BIN Taxonomy Match|BOLD:AAM7071
Cricotopus skirwithensis|BIOUG16478-B01|Kate Perez|BIN Taxonomy Match|BOLD:AAM7071
Cricotopus skirwithensis|BIOUG16478-B05|Kate Perez|BIN Taxonomy Match|BOLD:AAM7071
Cricotopus skirwithensis|BIOUG16479-G08|Kate Perez|BIN Taxonomy Match|BOLD:AAM7071
Cricotopus skirwithensis|BIOUG16258-C01|Kate Perez|BIN Taxonomy Match|BOLD:AAM7071
Cricotopus skirwithensis|BIOUG16552-E05|Kate Perez|BIN Taxonomy Match|BOLD:AAM7071
Cricotopus skirwithensis|BIOUG16261-G12|Kate Perez|BIN Taxonomy Match|BOLD:AAM7071
Cricotopus skirwithensis|BIOUG16261-G06|Kate Perez|BIN Taxonomy Match|BOLD:AAM7071
Cricotopus skirwithensis|BIOUG16297-H03|Kate Perez|BIN Taxonomy Match|BOLD:AAM7071
Cricotopus skirwithensis|BIOUG16297-B08|Kate Perez|BIN Taxonomy Match|BOLD:AAM7071
Cricotopus skirwithensis|BIOUG16433-G03|Kate Perez|BIN Taxonomy Match|BOLD:AAM7071
Cricotopus skirwithensis|BIOUG16479-B11|Kate Perez|BIN Taxonomy Match|BOLD:AAM7071
Cricotopus skirwithensis|BIOUG16430-F04|Kate Perez|BIN Taxonomy Match|BOLD:AAM7071
Cricotopus skirwithensis|BIOUG16259-D08|Kate Perez|BIN Taxonomy Match|BOLD:AAM7071
Cricotopus skirwithensis|BIOUG16261-B03|Kate Perez|BIN Taxonomy Match|BOLD:AAM7071
Cricotopus skirwithensis|BIOUG16434-B09|Kate Perez|BIN Taxonomy Match|BOLD:AAM7071
Cricotopus skirwithensis|BIOUG16479-D07|Kate Perez|BIN Taxonomy Match|BOLD:AAM7071
Cricotopus skirwithensis|BIOUG16432-A02|Kate Perez|BIN Taxonomy Match|BOLD:AAM7071
Cricotopus skirwithensis|BIOUG16433-D01|Kate Perez|BIN Taxonomy Match|BOLD:AAM7071
Cricotopus skirwithensis|BIOUG16477-B01|Kate Perez|BIN Taxonomy Match|BOLD:AAM7071
Cricotopus skirwithensis|BIOUG16478-C10|Kate Perez|BIN Taxonomy Match|BOLD:AAM7071
Cricotopus skirwithensis|BIOUG16432-B02|Kate Perez|BIN Taxonomy Match|BOLD:AAM7071
Cricotopus skirwithensis|BIOUG16298-E08|Kate Perez|BIN Taxonomy Match|BOLD:AAM7071
Cricotopus skirwithensis|BIOUG16396-C08|Kate Perez|BIN Taxonomy Match|BOLD:AAM7071
Cricotopus skirwithensis|BIOUG16434-E02|Kate Perez|BIN Taxonomy Match|BOLD:AAM7071
Cricotopus skirwithensis|BIOUG16431-D04|Kate Perez|BIN Taxonomy Match|BOLD:AAM7071
Cricotopus skirwithensis|BIOUG16328-C08|Kate Perez|BIN Taxonomy Match|BOLD:AAM7071
Cricotopus skirwithensis|BIOUG16434-G05|Kate Perez|BIN Taxonomy Match|BOLD:AAM7071
Cricotopus skirwithensis|BIOUG16328-F11|Kate Perez|BIN Taxonomy Match|BOLD:AAM7071
Cricotopus skirwithensis|BIOUG16555-H06|Kate Perez|BIN Taxonomy Match|BOLD:AAM7071
Cricotopus skirwithensis|BIOUG16298-D01|Kate Perez|BIN Taxonomy Match|BOLD:AAM7071
Cricotopus skirwithensis|BIOUG15654-C05|Kate Perez|BIN Taxonomy Match|BOLD:AAM7071
Cricotopus skirwithensis|BIOUG15592-A03|Kate Perez|BIN Taxonomy Match|BOLD:AAM7071
Cricotopus skirwithensis|BIOUG16436-H09|Kate Perez|BIN Taxonomy Match|BOLD:AAM7071
Cricotopus skirwithensis|BIOUG16435-G05|Kate Perez|BIN Taxonomy Match|BOLD:AAM7071
Cricotopus skirwithensis|BIOUG16330-D12|Kate Perez|BIN Taxonomy Match|BOLD:AAM7071
Cricotopus skirwithensis|BIOUG16398-D12|Kate Perez|BIN Taxonomy Match|BOLD:AAM7071
Cricotopus skirwithensis|BIOUG15537-G08|Kate Perez|BIN Taxonomy Match|BOLD:AAM7071
Cricotopus skirwithensis|BIOUG16436-G11|Kate Perez|BIN Taxonomy Match|BOLD:AAM7071
Cricotopus skirwithensis|BIOUG16299-D09|Kate Perez|BIN Taxonomy Match|BOLD:AAM7071
Cricotopus skirwithensis|BIOUG16329-G05|Kate Perez|BIN Taxonomy Match|BOLD:AAM7071
Cricotopus skirwithensis|BIOUG16476-A09|Kate Perez|BIN Taxonomy Match|BOLD:AAM7071
Cricotopus skirwithensis|BIOUG16435-G07|Kate Perez|BIN Taxonomy Match|BOLD:AAM7071
Cricotopus skirwithensis|BIOUG16328-A10|Kate Perez|BIN Taxonomy Match|BOLD:AAM7071
Cricotopus skirwithensis|BIOUG16480-H08|Kate Perez|BIN Taxonomy Match|BOLD:AAM7071
Cricotopus skirwithensis|BIOUG16436-E06|Kate Perez|BIN Taxonomy Match|BOLD:AAM7071
Cricotopus skirwithensis|BIOUG16476-A07|Kate Perez|BIN Taxonomy Match|BOLD:AAM7071
Cricotopus skirwithensis|BIOUG16436-H07|Kate Perez|BIN Taxonomy Match|BOLD:AAM7071
Cricotopus skirwithensis|BIOUG16432-D10|Kate Perez|BIN Taxonomy Match|BOLD:AAM7071
Cricotopus skirwithensis|BIOUG16329-A08|Kate Perez|BIN Taxonomy Match|BOLD:AAM7071
Cricotopus skirwithensis|BIOUG16478-G08|Kate Perez|BIN Taxonomy Match|BOLD:AAM7071
Cricotopus skirwithensis|BIOUG16297-F06|Kate Perez|BIN Taxonomy Match|BOLD:AAM7071
Cricotopus skirwithensis|BIOUG16480-E01|Kate Perez|BIN Taxonomy Match|BOLD:AAM7071
Cricotopus skirwithensis|BIOUG16436-B02|Kate Perez|BIN Taxonomy Match|BOLD:AAM7071
Cricotopus skirwithensis|BIOUG16479-C05|Kate Perez|BIN Taxonomy Match|BOLD:AAM7071
Cricotopus skirwithensis|BIOUG16436-D03|Kate Perez|BIN Taxonomy Match|BOLD:AAM7071
Cricotopus skirwithensis|BIOUG16329-G10|Kate Perez|BIN Taxonomy Match|BOLD:AAM7071
Cricotopus skirwithensis|BIOUG16296-E12|Kate Perez|BIN Taxonomy Match|BOLD:AAM7071
Cricotopus skirwithensis|BIOUG16435-B11|Kate Perez|BIN Taxonomy Match|BOLD:AAM7071
Cricotopus skirwithensis|BIOUG16480-D04|Kate Perez|BIN Taxonomy Match|BOLD:AAM7071
Cricotopus skirwithensis|BIOUG16430-B04|Kate Perez|BIN Taxonomy Match|BOLD:AAM7071
Cricotopus skirwithensis|BIOUG15464-G02|Kate Perez|BIN Taxonomy Match|BOLD:AAM7071
Cricotopus skirwithensis|BIOUG16430-D07|Kate Perez|BIN Taxonomy Match|BOLD:AAM7071
Cricotopus skirwithensis|BIOUG16432-B04|Kate Perez|BIN Taxonomy Match|BOLD:AAM7071
Cricotopus skirwithensis|BIOUG16430-C09|Kate Perez|BIN Taxonomy Match|BOLD:AAM7071
Cricotopus skirwithensis|BIOUG15542-A09|Kate Perez|BIN Taxonomy Match|BOLD:AAM7071
Cricotopus skirwithensis|BIOUG16257-D08|Kate Perez|BIN Taxonomy Match|BOLD:AAM7071
Cricotopus skirwithensis|BIOUG16331-A03|Kate Perez|BIN Taxonomy Match|BOLD:AAM7071
Cricotopus skirwithensis|BIOUG16260-G07|Kate Perez|BIN Taxonomy Match|BOLD:AAM7071
Cricotopus skirwithensis|BIOUG16260-F04|Kate Perez|BIN Taxonomy Match|BOLD:AAM7071
Cricotopus skirwithensis|BIOUG16258-C12|Kate Perez|BIN Taxonomy Match|BOLD:AAM7071
Cricotopus skirwithensis|BIOUG16330-C05|Kate Perez|BIN Taxonomy Match|BOLD:AAM7071
Cricotopus skirwithensis|BIOUG16430-G04|Kate Perez|BIN Taxonomy Match|BOLD:AAM7071
Cricotopus skirwithensis|BIOUG16430-C04|Kate Perez|BIN Taxonomy Match|BOLD:AAM7071
Cricotopus skirwithensis|BIOUG16434-G07|Kate Perez|BIN Taxonomy Match|BOLD:AAM7071
[illegible]

Cricotopus skirwithensis|BIOUG16430-G04|Kate Perez|BIN Taxonomy Match|BOLD:AAM7071
Cricotopus skirwithensis|BIOUG16430-C04|Kate Perez|BIN Taxonomy Match|BOLD:AAM7071
Cricotopus skirwithensis|BIOUG16434-G07|Kate Perez|BIN Taxonomy Match|BOLD:AAM7071
Cricotopus skirwithensis|BIOUG16433-F07|Kate Perez|BIN Taxonomy Match|BOLD:AAM7071
Cricotopus skirwithensis|BIOUG16435-B08|Kate Perez|BIN Taxonomy Match|BOLD:AAM7071
Cricotopus skirwithensis|BIOUG16435-A06|Kate Perez|BIN Taxonomy Match|BOLD:AAM7071
Cricotopus skirwithensis|BIOUG16430-A05|Kate Perez|BIN Taxonomy Match|BOLD:AAM7071
Cricotopus skirwithensis|BIOUG16261-H11|Kate Perez|BIN Taxonomy Match|BOLD:AAM7071
Cricotopus skirwithensis|BIOUG16330-B08|Kate Perez|BIN Taxonomy Match|BOLD:AAM7071
Cricotopus skirwithensis|BIOUG16330-A05|Kate Perez|BIN Taxonomy Match|BOLD:AAM7071
Cricotopus skirwithensis|BIOUG16257-E01|Kate Perez|BIN Taxonomy Match|BOLD:AAM7071
Cricotopus skirwithensis|BIOUG16257-C03|Kate Perez|BIN Taxonomy Match|BOLD:AAM7071
Cricotopus skirwithensis|BIOUG16551-E01|Kate Perez|BIN Taxonomy Match|BOLD:AAM7071
Cricotopus skirwithensis|BIOUG16552-C01|Kate Perez|BIN Taxonomy Match|BOLD:AAM7071
Cricotopus skirwithensis|BIOUG16332-G04|Kate Perez|BIN Taxonomy Match|BOLD:AAM7071
Cricotopus skirwithensis|BIOUG15541-F07|Kate Perez|BIN Taxonomy Match|BOLD:AAM7071
Cricotopus skirwithensis|BIOUG16431-F08|Kate Perez|BIN Taxonomy Match|BOLD:AAM7071
Cricotopus skirwithensis|BIOUG16255-D10|Kate Perez|BIN Taxonomy Match|BOLD:AAM7071
Cricotopus skirwithensis|BIOUG16333-G06|Kate Perez|BIN Taxonomy Match|BOLD:AAM7071
Cricotopus skirwithensis|BIOUG16431-E01|Kate Perez|BIN Taxonomy Match|BOLD:AAM7071
Cricotopus skirwithensis|BIOUG16297-F08|Kate Perez|BIN Taxonomy Match|BOLD:AAM7071
Cricotopus skirwithensis|BIOUG16328-G04|Kate Perez|BIN Taxonomy Match|BOLD:AAM7071
Cricotopus skirwithensis|BIOUG16299-G09|Kate Perez|BIN Taxonomy Match|BOLD:AAM7071
Cricotopus skirwithensis|BIOUG16551-E08|Kate Perez|BIN Taxonomy Match|BOLD:AAM7071
Cricotopus skirwithensis|BIOUG16256-D01|Kate Perez|BIN Taxonomy Match|BOLD:AAM7071
Cricotopus skirwithensis|BIOUG16260-A11|Kate Perez|BIN Taxonomy Match|BOLD:AAM7071
Cricotopus skirwithensis|BIOUG15541-G07|Kate Perez|BIN Taxonomy Match|BOLD:AAM7071
Cricotopus skirwithensis|BIOUG16480-F01|Kate Perez|BIN Taxonomy Match|BOLD:AAM7071
Cricotopus skirwithensis|BIOUG16260-A04|Kate Perez|BIN Taxonomy Match|BOLD:AAM7071
Cricotopus skirwithensis|BIOUG16432-G04|Kate Perez|BIN Taxonomy Match|BOLD:AAM7071
Cricotopus skirwithensis|BIOUG16431-E09|Kate Perez|BIN Taxonomy Match|BOLD:AAM7071
Cricotopus skirwithensis|BIOUG16552-D01|Kate Perez|BIN Taxonomy Match|BOLD:AAM7071
Cricotopus skirwithensis|BIOUG16435-B09|Kate Perez|BIN Taxonomy Match|BOLD:AAM7071
Cricotopus skirwithensis|BIOUG16396-D06|Kate Perez|BIN Taxonomy Match|BOLD:AAM7071
Cricotopus skirwithensis|BIOUG16435-H01|Kate Perez|BIN Taxonomy Match|BOLD:AAM7071
Cricotopus skirwithensis|BIOUG16477-G11|Kate Perez|BIN Taxonomy Match|BOLD:AAM7071
Cricotopus skirwithensis|BIOUG16480-C09|Kate Perez|BIN Taxonomy Match|BOLD:AAM7071
Cricotopus skirwithensis|BIOUG16476-F03|Kate Perez|BIN Taxonomy Match|BOLD:AAM7071
Cricotopus skirwithensis|BIOUG16479-C08|Kate Perez|BIN Taxonomy Match|BOLD:AAM7071
Cricotopus skirwithensis|BIOUG16480-C08|Kate Perez|BIN Taxonomy Match|BOLD:AAM7071
Cricotopus skirwithensis|BIOUG16258-B06|Kate Perez|BIN Taxonomy Match|BOLD:AAM7071
Cricotopus skirwithensis|BIOUG16434-B04|Kate Perez|BIN Taxonomy Match|BOLD:AAM7071
Cricotopus skirwithensis|BIOUG16478-G05|Kate Perez|BIN Taxonomy Match|BOLD:AAM7071
Cricotopus skirwithensis|BIOUG16477-F10|Kate Perez|BIN Taxonomy Match|BOLD:AAM7071
Cricotopus skirwithensis|BIOUG15674-E10|Kate Perez|BIN Taxonomy Match|BOLD:AAM7071
Cricotopus skirwithensis|BIOUG16433-H08|Kate Perez|BIN Taxonomy Match|BOLD:AAM7071
Cricotopus skirwithensis|BIOUG16258-C07|Kate Perez|BIN Taxonomy Match|BOLD:AAM7071
Cricotopus skirwithensis|BIOUG15544-D12|Kate Perez|BIN Taxonomy Match|BOLD:AAM7071
Cricotopus skirwithensis|BIOUG16299-D12|Kate Perez|BIN Taxonomy Match|BOLD:AAM7071
Cricotopus skirwithensis|BIOUG16592-C03|Kate Perez|BIN Taxonomy Match|BOLD:AAM7071
Cricotopus skirwithensis|BIOUG16479-B06|Kate Perez|BIN Taxonomy Match|BOLD:AAM7071
Cricotopus skirwithensis|BIOUG16329-G03|Kate Perez|BIN Taxonomy Match|BOLD:AAM7071
Cricotopus skirwithensis|BIOUG16297-H10|Kate Perez|BIN Taxonomy Match|BOLD:AAM7071
Cricotopus skirwithensis|BIOUG15766-F02|Kate Perez|BIN Taxonomy Match|BOLD:AAM7071
Chironomidae|ZSM-DIP-33136-D09|Caroline Chimeno|BIN Taxonomy Match (Jan 2022)|BOLD:ACU5356
Chironomidae|ZSM-DIP-33140-B02|Caroline Chimeno|BIN Taxonomy Match (Jan 2022)|BOLD:ACU5356
Chironomidae|BC-ZSM-DIP-22551-F03|Dieter Doczkal|BIN Taxonomy Match|BOLD:ACU5356
Chironomidae|CCDB24037-A03|Jerome Moriniere|BIN Taxonomy Match|BOLD:ACU5356
Cricotopus|CCDB24039-D09|Sofia Wiedenbrug|BIN Taxonomy Match|BOLD:ACU4640
Cricotopus|CCDB24039-D02|Sofia Wiedenbrug|BIN Taxonomy Match|BOLD:ACU4640
Cricotopus skirwithensis|ZMUO.025893|Lauri Paasivirta||BOLD:ACK7136
Cricotopus skirwithensis|ZMUO.024834|Lauri Paasivirta||BOLD:ACK7136
Cricotopus skirwithensis|ZMUO.025892|Lauri Paasivirta||BOLD:ACK7136
Cricotopus skirwithensis|ZMUO.024833|Lauri Paasivirta||BOLD:ACK7136
Cricotopus nivalis|BIOUG07503-C02|Kate Perez|BIN Taxonomy Match|BOLD:ACK7136
Cricotopus nivalis|CCDB24228-A05|Sofia Wiedenbrug|BIN Taxonomy Match|BOLD:AAB5829
Cricotopus nivalis|CCDB24039-F02|Sofia Wiedenbrug|BIN Taxonomy Match|BOLD:AAB5829
Cricotopus nivalis|ES37|Torbjorn Ekrem||BOLD:AAB5829
Cricotopus nivalis|ES36|Torbjorn Ekrem||BOLD:AAB5829
Cricotopus nivalis|ATNA272|Torbjorn Ekrem||BOLD:AAB5829
Cricotopus nivalis|ATNA283|Torbjorn Ekrem||BOLD:AAB5829
Cricotopus nivalis|ATNA301|Torbjorn Ekrem||BOLD:AAB5829
Cricotopus nivalis|SOE403|Torbjorn Ekrem||BOLD:AAB5829
Cricotopus nivalis|SOE186|Torbjorn Ekrem||BOLD:AAB5829
Cricotopus nivalis|SOE255|Torbjorn Ekrem||BOLD:AAB5829
Cricotopus nivalis|BC-ZSM-DIP-22551-C12|Dieter Doczkal||BOLD:AAB5829
Cricotopus nivalis|SOE229|Torbjorn Ekrem||BOLD:AAB5829
Cricotopus nivalis|ATNA273|Torbjorn Ekrem||BOLD:AAB5829
Cricotopus nivalis|SOE253|Torbjorn Ekrem||BOLD:AAB5829
Cricotopus nivalis|SOE230|Torbjorn Ekrem||BOLD:AAB5829
Cricotopus nivalis|SOE213|Torbjorn Ekrem||BOLD:AAB5829
Cricotopus nivalis|SOE207|Torbjorn Ekrem||BOLD:AAB5829
Cricotopus nivalis|SOE426|Torbjorn Ekrem||BOLD:AAB5829
Cricotopus nivalis|SOE402|Torbjorn Ekrem||BOLD:AAB5829
Cricotopus nivalis|SOE101|Torbjorn Ekrem||BOLD:AAB5829
Cricotopus nivalis|ATNA289|Torbjorn Ekrem||BOLD:AAB5829
Cricotopus nivalis|SOE355|Torbjorn Ekrem||BOLD:AAB5829
Cricotopus nivalis|SOE100|Torbjorn Ekrem||BOLD:AAB5829
Cricotopus osellai|ATNA480|Elisabeth Stur||BOLD:AAD1506
Cricotopus osellai|ES102|Torbjorn Ekrem||BOLD:AAD1506
Cricotopus osellai|SOE132|Torbjorn Ekrem||BOLD:AAD1506
Cricotopus osellai|SOE124|Torbjorn Ekrem||BOLD:AAD1506
Cricotopus osellai|ES101|Torbjorn Ekrem||BOLD:AAD1506
Cricotopus osellai|SOE212|Torbjorn Ekrem||BOLD:AAD1506
Cricotopus osellai|ATNA352|Torbjorn Ekrem||BOLD:AAD1506
Cricotopus osellai|BIOUG16711-H04|Kate Perez|BIN Taxonomy Match|BOLD:AAD1506
Cricotopus osellai|ATNA475|Elisabeth Stur||BOLD:AAD1506
Cricotopus osellai|ATNA171|Torbjorn Ekrem||BOLD:AAD1506
Cricotopus osellai|ATNA159|Torbjorn Ekrem||BOLD:AAD1506
Cricotopus skirwithensis|ZMUO.026325|Lauri Paasivirta||BOLD:AAD1506
Cricotopus osellai|BIOUG17022-H11|Kate Perez|BIN Taxonomy Match|BOLD:AAD1506
Cricotopus osellai|BIOUG15185-E09|Kate Perez|BIN Taxonomy Match|BOLD:AAD1506
Cricotopus osellai|Finnmark609|Elisabeth Stur||BOLD:AAD1506

Cricotopus osellai|BIOUG17022-H11|Kate Perez|BIN Taxonomy Match|BOLD:AAD1506
Cricotopus osellai|BIOUG15185-E09|Kate Perez|BIN Taxonomy Match|BOLD:AAD1506
Cricotopus osellai|Finnmark609|Elisabeth Stur||BOLD:AAD1506
Procladius sp. PROC_SIM7_1|KY225357
Nilotanypus dubius|ZMUO.025203|Lauri Paasivirta||BOLD:ADA3224
Nilotanypus dubius|ZMUO.025202|Lauri Paasivirta||BOLD:ADA3224
Nilotanypus dubius|EJV-20110183|Lauri Paasivirta||BOLD:AAW4683
Nilotanypus dubius|EJV-20110182|Lauri Paasivirta||BOLD:AAW4683
Nilotanypus dubius|EJV-20110184|Lauri Paasivirta||BOLD:AAW4682
Nilotanypus dubius|EJV-20110181|Lauri Paasivirta||BOLD:AAW4682
Tvetenia calvescens|EBAI-Ch099|Elisabeth Stur|morphology|BOLD:AAU0004
Tvetenia calvescens|CH-OSF129|Elisabeth Stur||BOLD:AAU0004
Tvetenia calvescens|EBAI-Ch100|Elisabeth Stur|morphology|BOLD:AAU0004
Tvetenia calvescens|EBAI-Ch101|Elisabeth Stur|morphology|BOLD:AAU0004
Tvetenia calvescens|CH-OSF128|Elisabeth Stur||BOLD:AAU0004
Tvetenia calvescens|CH-OSF60|Elisabeth Stur||BOLD:AAU0004
Tvetenia calvescens|Finnmark873|Elisabeth Stur|morphology|BOLD:AAU0004
Tvetenia calvescens|Finnmark775|G. A. Halvorsen||BOLD:AAU0004
Tvetenia calvescens|Finnmark519|Elisabeth Stur||BOLD:AAU0004
Tvetenia calvescens|ZSM-DIP-33147-A01|Caroline Chimeno|BIN Taxonomy Match (Jan 2022)|BOLD ...
Orthocladiinae|BIOUG16360-H01|Kate Perez|BIN Taxonomy Match|BOLD:AAG1011
Tvetenia calvescens|PK-192-66|Petra Kranzfelder|morphology|BOLD:AAG1011
Tvetenia calvescens|BIOUG15592-A02|Kate Perez|BIN Taxonomy Match|BOLD:AAG1011
Tvetenia calvescens|BIOUG15852-G10|Kate Perez|BIN Taxonomy Match|BOLD:AAG1011
Tvetenia calvescens|BIOUG16631-H06|Kate Perez|BIN Taxonomy Match|BOLD:AAG1011
Tvetenia calvescens|BIOUG16632-H06|Kate Perez|BIN Taxonomy Match|BOLD:AAG1011
Tvetenia calvescens|PK-192-108|Petra Kranzfelder|morphology|BOLD:AAG1011
Tvetenia calvescens|BIOUG16631-A04|Kate Perez|BIN Taxonomy Match|BOLD:AAG1011
Tvetenia calvescens|PK-192-128|Petra Kranzfelder|morphology|BOLD:AAG1011
Tvetenia calvescens|BIOUG15792-D09|Kate Perez|BIN Taxonomy Match|BOLD:AAG1011
Tvetenia calvescens|BIOUG16713-D02|Kate Perez|BIN Taxonomy Match|BOLD:AAG1011
Tvetenia calvescens|BIOUG16330-C04|Kate Perez|BIN Taxonomy Match|BOLD:AAG1011
Tvetenia calvescens|BIOUG16331-C06|Kate Perez|BIN Taxonomy Match|BOLD:AAG1011
Tvetenia calvescens|BIOUG16331-G08|Kate Perez|BIN Taxonomy Match|BOLD:AAG1011
Tvetenia calvescens|BIOUG16260-B09|Kate Perez|BIN Taxonomy Match|BOLD:AAG1011
Tvetenia calvescens|BIOUG16712-F09|Kate Perez|BIN Taxonomy Match|BOLD:AAG1011
Tvetenia calvescens|BIOUG15564-D05|Kate Perez|BIN Taxonomy Match|BOLD:AAG1011
Tvetenia calvescens|BIOUG16296-D05|Kate Perez|BIN Taxonomy Match|BOLD:AAG1011
Tvetenia calvescens|BIOUG16711-E05|Kate Perez|BIN Taxonomy Match|BOLD:AAG1011
Tvetenia calvescens|BIOUG16718-F01|Kate Perez|BIN Taxonomy Match|BOLD:AAG1011
Tvetenia calvescens|BIOUG16430-H10|Kate Perez|BIN Taxonomy Match|BOLD:AAG1011
Tvetenia calvescens|BIOUG15675-C03|Kate Perez|BIN Taxonomy Match|BOLD:AAG1011
Tvetenia calvescens|BIOUG16630-G12|Kate Perez|BIN Taxonomy Match|BOLD:AAG1011
Tvetenia calvescens|BIOUG16597-D09|Kate Perez|BIN Taxonomy Match|BOLD:AAG1011
Tvetenia calvescens|BIOUG16332-F01|Kate Perez|BIN Taxonomy Match|BOLD:AAG1011
Tvetenia calvescens|BIOUG16396-D11|Kate Perez|BIN Taxonomy Match|BOLD:AAG1011
Tvetenia calvescens|BIOUG15762-G10|Kate Perez|BIN Taxonomy Match|BOLD:AAG1011
Tvetenia calvescens|BIOUG16297-E04|Kate Perez|BIN Taxonomy Match|BOLD:AAG1011
Tvetenia calvescens|BIOUG16328-F10|Kate Perez|BIN Taxonomy Match|BOLD:AAG1011
Tvetenia calvescens|BIOUG15597-A05|Kate Perez|BIN Taxonomy Match|BOLD:AAG1011
Tvetenia calvescens|BIOUG15649-C08|Kate Perez|BIN Taxonomy Match|BOLD:AAG1011
Tvetenia calvescens|BIOUG15648-G03|Kate Perez|BIN Taxonomy Match|BOLD:AAG1011
Tvetenia calvescens|BIOUG16713-E05|Kate Perez|BIN Taxonomy Match|BOLD:AAG1011
Tvetenia calvescens|BIOUG16477-A11|Kate Perez|BIN Taxonomy Match|BOLD:AAG1011
Tvetenia calvescens|BIOUG16711-E10|Kate Perez|BIN Taxonomy Match|BOLD:AAG1011
Tvetenia calvescens|BIOUG16632-G07|Kate Perez|BIN Taxonomy Match|BOLD:AAG1011
Tvetenia calvescens|BIOUG16432-G05|Kate Perez|BIN Taxonomy Match|BOLD:AAG1011
Tvetenia calvescens|BIOUG16430-B10|Kate Perez|BIN Taxonomy Match|BOLD:AAG1011
Tvetenia calvescens|BIOUG15806-D05|Kate Perez|BIN Taxonomy Match|BOLD:AAG1011
Tvetenia calvescens|BIOUG16331-A02|Kate Perez|BIN Taxonomy Match|BOLD:AAG1011
Tvetenia calvescens|BIOUG15681-G02|Kate Perez|BIN Taxonomy Match|BOLD:AAG1011
Tvetenia calvescens|BIOUG15674-H01|Kate Perez|BIN Taxonomy Match|BOLD:AAG1011
Tvetenia calvescens|BIOUG15852-B09|Kate Perez|BIN Taxonomy Match|BOLD:AAG1011
Tvetenia calvescens|BIOUG16632-G10|Kate Perez|BIN Taxonomy Match|BOLD:AAG1011
Tvetenia calvescens|BIOUG16476-H08|Kate Perez|BIN Taxonomy Match|BOLD:AAG1011
Tvetenia calvescens|BIOUG16630-G08|Kate Perez|BIN Taxonomy Match|BOLD:AAG1011
Tvetenia calvescens|BIOUG16632-E07|Kate Perez|BIN Taxonomy Match|BOLD:AAG1011
Tvetenia calvescens|BIOUG16592-B06|Kate Perez|BIN Taxonomy Match|BOLD:AAG1011
Tvetenia calvescens|BIOUG15853-D02|Kate Perez|BIN Taxonomy Match|BOLD:AAG1011
Tvetenia calvescens|BIOUG16435-H03|Kate Perez|BIN Taxonomy Match|BOLD:AAG1011
Tvetenia calvescens|BIOUG16631-F03|Kate Perez|BIN Taxonomy Match|BOLD:AAG1011
Tvetenia calvescens|BIOUG16631-B02|Kate Perez|BIN Taxonomy Match|BOLD:AAG1011
Tvetenia calvescens|BIOUG16713-E04|Kate Perez|BIN Taxonomy Match|BOLD:AAG1011
Tvetenia calvescens|BIOUG16630-H11|Kate Perez|BIN Taxonomy Match|BOLD:AAG1011
Tvetenia calvescens|BIOUG15852-D03|Kate Perez|BIN Taxonomy Match|BOLD:AAG1011
Tvetenia calvescens|BIOUG16552-E02|Kate Perez|BIN Taxonomy Match|BOLD:AAG1011
Tvetenia calvescens|BIOUG15538-C08|Kate Perez|BIN Taxonomy Match|BOLD:AAG1011
Tvetenia calvescens|BIOUG16631-H07|Kate Perez|BIN Taxonomy Match|BOLD:AAG1011
Tvetenia calvescens|BIOUG15592-B08|Kate Perez|BIN Taxonomy Match|BOLD:AAG1011
Tvetenia calvescens|ATNA206|Elisabeth Stur||BOLD:AAG1011
Tvetenia calvescens|BIOUG16631-D10|Kate Perez|BIN Taxonomy Match|BOLD:AAG1011
Tvetenia calvescens|BIOUG15648-A11|Kate Perez|BIN Taxonomy Match|BOLD:AAG1011
Tvetenia calvescens|PK-192-116|Petra Kranzfelder|morphology|BOLD:AAG1011
Tvetenia calvescens|BIOUG16436-H04|Kate Perez|BIN Taxonomy Match|BOLD:AAG1011
Tvetenia calvescens|BIOUG16435-E04|Kate Perez|BIN Taxonomy Match|BOLD:AAG1011
Tvetenia calvescens|BIOUG16329-F08|Kate Perez|BIN Taxonomy Match|BOLD:AAG1011
Tvetenia calvescens|BIOUG16557-F08|Kate Perez|BIN Taxonomy Match|BOLD:AAG1011
Tvetenia calvescens|BIOUG16597-B05|Kate Perez|BIN Taxonomy Match|BOLD:AAG1011
Tvetenia calvescens|BIOUG16478-C03|Kate Perez|BIN Taxonomy Match|BOLD:AAG1011
Tvetenia calvescens|BIOUG15807-B09|Kate Perez|BIN Taxonomy Match|BOLD:AAG1011
Tvetenia calvescens|BIOUG16332-F11|Kate Perez|BIN Taxonomy Match|BOLD:AAG1011
Tvetenia calvescens|BIOUG16333-A09|Kate Perez|BIN Taxonomy Match|BOLD:AAG1011
Tvetenia calvescens|BIOUG15808-B09|Kate Perez|BIN Taxonomy Match|BOLD:AAG1011
Tvetenia calvescens|BIOUG15809-A09|Kate Perez|BIN Taxonomy Match|BOLD:AAG1011
Tvetenia calvescens|BIOUG16330-H03|Kate Perez|BIN Taxonomy Match|BOLD:AAG1011
Tvetenia calvescens|BIOUG16711-E07|Kate Perez|BIN Taxonomy Match|BOLD:AAG1011
Tvetenia calvescens|BIOUG16711-E08|Kate Perez|BIN Taxonomy Match|BOLD:AAG1011
Tvetenia calvescens|BIOUG17023-C10|Kate Perez|BIN Taxonomy Match|BOLD:AAG1011
Tvetenia calvescens|BIOUG16631-C12|Kate Perez|BIN Taxonomy Match|BOLD:AAG1011
Tvetenia calvescens|BIOUG17022-F08|Kate Perez|BIN Taxonomy Match|BOLD:AAG1011
Tvetenia calvescens|PK-192-115|Petra Kranzfelder|morphology
Tvetenia calvescens|BIOUG16597-C07|Kate Perez|BIN Taxonomy Match|BOLD:AAG1011
Tvetenia calvescens|BIOUG16396-C03|Kate Perez|BIN Taxonomy Match|BOLD:AAG1011

Tvetenia calvescens|PK-192-115|Petra Kranzfelder|morphology
Tvetenia calvescens|BIOUG16597-C07|Kate Perez|BIN Taxonomy Match|BOLD:AAG1011
Tvetenia calvescens|BIOUG16396-C03|Kate Perez|BIN Taxonomy Match|BOLD:AAG1011
Tvetenia calvescens|BIOUG16713-G05|Kate Perez|BIN Taxonomy Match|BOLD:AAG1011
Tvetenia calvescens|BIOUG16712-F06|Kate Perez|BIN Taxonomy Match|BOLD:AAG1011
Tvetenia calvescens|BIOUG17022-C05|Kate Perez|BIN Taxonomy Match|BOLD:AAG1011
Tvetenia calvescens|BIOUG16626-D05|Kate Perez|BIN Taxonomy Match|BOLD:AAG1011
Tvetenia calvescens|BIOUG16630-A01|Kate Perez|BIN Taxonomy Match|BOLD:AAG1011
Tvetenia calvescens|BIOUG16631-E10|Kate Perez|BIN Taxonomy Match|BOLD:AAG1011
Tvetenia calvescens|BIOUG16631-A12|Kate Perez|BIN Taxonomy Match|BOLD:AAG1011
Tvetenia calvescens|BIOUG16627-A10|Kate Perez|BIN Taxonomy Match|BOLD:AAG1011
Tvetenia calvescens|BIOUG16257-C06|Kate Perez|BIN Taxonomy Match|BOLD:AAG1011
Tvetenia calvescens|BIOUG16552-A06|Kate Perez|BIN Taxonomy Match|BOLD:AAG1011
Tvetenia calvescens|BIOUG15599-B06|Kate Perez|BIN Taxonomy Match|BOLD:AAG1011
Tvetenia calvescens|BIOUG16594-G10|Kate Perez|BIN Taxonomy Match|BOLD:AAG1011
Tvetenia calvescens|BIOUG16296-G01|Kate Perez|BIN Taxonomy Match|BOLD:AAG1011
Tvetenia calvescens|BIOUG15855-A01|Kate Perez|BIN Taxonomy Match|BOLD:AAG1011
Tvetenia calvescens|BIOUG16711-D08|Kate Perez|BIN Taxonomy Match|BOLD:AAG1011
Tvetenia calvescens|BIOUG16627-A09|Kate Perez|BIN Taxonomy Match|BOLD:AAG1011
Tvetenia calvescens|BIOUG16332-G11|Kate Perez|BIN Taxonomy Match|BOLD:AAG1011
Tvetenia calvescens|BIOUG15797-F11|Kate Perez|BIN Taxonomy Match|BOLD:AAG1011
Tvetenia calvescens|BIOUG16297-H07|Kate Perez|BIN Taxonomy Match|BOLD:AAG1011
Tvetenia calvescens|BIOUG16477-A08|Kate Perez|BIN Taxonomy Match|BOLD:AAG1011
Tvetenia calvescens|BIOUG15796-G01|Kate Perez|BIN Taxonomy Match|BOLD:AAG1011
Tvetenia calvescens|BIOUG16431-G01|Kate Perez|BIN Taxonomy Match|BOLD:AAG1011
Tvetenia calvescens|BIOUG16712-A09|Kate Perez|BIN Taxonomy Match|BOLD:AAG1011
Tvetenia calvescens|BIOUG16333-E05|Kate Perez|BIN Taxonomy Match|BOLD:AAG1011
Tvetenia calvescens|BIOUG16479-H11|Kate Perez|BIN Taxonomy Match|BOLD:AAG1011
Tvetenia calvescens|BIOUG16433-B03|Kate Perez|BIN Taxonomy Match|BOLD:AAG1011
Tvetenia calvescens|BIOUG16631-F02|Kate Perez|BIN Taxonomy Match|BOLD:AAG1011
Tvetenia calvescens|BIOUG16622-H04|Kate Perez|BIN Taxonomy Match|BOLD:AAG1011
Tvetenia calvescens|BIOUG15796-A08|Kate Perez|BIN Taxonomy Match|BOLD:AAG1011
Tvetenia calvescens|BIOUG16631-H05|Kate Perez|BIN Taxonomy Match|BOLD:AAG1011
Tvetenia calvescens|BIOUG16436-C02|Kate Perez|BIN Taxonomy Match|BOLD:AAG1011
Tvetenia calvescens|BIOUG16631-E08|Kate Perez|BIN Taxonomy Match|BOLD:AAG1011
Tvetenia calvescens|BIOUG16632-C12|Kate Perez|BIN Taxonomy Match|BOLD:AAG1011
Tvetenia calvescens|BIOUG17022-E07|Kate Perez|BIN Taxonomy Match|BOLD:AAG1011
Tvetenia calvescens|BIOUG16598-A11|Kate Perez|BIN Taxonomy Match|BOLD:AAG1011
Tvetenia calvescens|BIOUG16480-G03|Kate Perez|BIN Taxonomy Match|BOLD:AAG1011
Tvetenia calvescens|BIOUG16328-A08|Kate Perez|BIN Taxonomy Match|BOLD:AAG1011
Tvetenia calvescens|BIOUG15653-G03|Kate Perez|BIN Taxonomy Match|BOLD:AAG1011
Tvetenia calvescens|BIOUG15598-D11|Kate Perez|BIN Taxonomy Match|BOLD:AAG1011
Tvetenia calvescens|BIOUG15655-F04|Kate Perez|BIN Taxonomy Match|BOLD:AAG1011
Tvetenia calvescens|BIOUG16632-C05|Kate Perez|BIN Taxonomy Match|BOLD:AAG1011
Tvetenia calvescens|BIOUG16712-A06|Kate Perez|BIN Taxonomy Match|BOLD:AAG1011
Tvetenia calvescens|BIOUG16477-E02|Kate Perez|BIN Taxonomy Match|BOLD:AAG1011
Tvetenia calvescens|BIOUG16626-C02|Kate Perez|BIN Taxonomy Match|BOLD:AAG1011
Tvetenia calvescens|BIOUG16435-G08|Kate Perez|BIN Taxonomy Match|BOLD:AAG1011
Tvetenia calvescens|BIOUG16299-H08|Kate Perez|BIN Taxonomy Match|BOLD:AAG1011
Tvetenia calvescens|BIOUG16557-G01|Kate Perez|BIN Taxonomy Match|BOLD:AAG1011
Tvetenia calvescens|BIOUG15807-A08|Kate Perez|BIN Taxonomy Match|BOLD:AAG1011
Tvetenia calvescens|BIOUG15806-B12|Kate Perez|BIN Taxonomy Match|BOLD:AAG1011
Tvetenia calvescens|BIOUG16331-G11|Kate Perez|BIN Taxonomy Match|BOLD:AAG1011
Tvetenia calvescens|BIOUG16431-H09|Kate Perez|BIN Taxonomy Match|BOLD:AAG1011
Tvetenia calvescens|BIOUG15855-D12|Kate Perez|BIN Taxonomy Match|BOLD:AAG1011
Tvetenia calvescens|BIOUG15855-G01|Kate Perez|BIN Taxonomy Match|BOLD:AAG1011
Tvetenia calvescens|BIOUG15676-A05|Kate Perez|BIN Taxonomy Match|BOLD:AAG1011
Tvetenia calvescens|BIOUG16331-E04|Kate Perez|BIN Taxonomy Match|BOLD:AAG1011
Tvetenia calvescens|BIOUG16296-C01|Kate Perez|BIN Taxonomy Match|BOLD:AAG1011
Tvetenia calvescens|BIOUG16594-H06|Kate Perez|BIN Taxonomy Match|BOLD:AAG1011
Tvetenia calvescens|BIOUG16594-G07|Kate Perez|BIN Taxonomy Match|BOLD:AAG1011
Tvetenia calvescens|BIOUG16551-H11|Kate Perez|BIN Taxonomy Match|BOLD:AAG1011
Tvetenia calvescens|BIOUG15808-F12|Kate Perez|BIN Taxonomy Match|BOLD:AAG1011
Tvetenia calvescens|BIOUG15795-E11|Kate Perez|BIN Taxonomy Match|BOLD:AAG1011
Tvetenia calvescens|BIOUG15793-E02|Kate Perez|BIN Taxonomy Match|BOLD:AAG1011
Tvetenia calvescens|BIOUG15792-G11|Kate Perez|BIN Taxonomy Match|BOLD:AAG1011
Tvetenia calvescens|BIOUG15792-E12|Kate Perez|BIN Taxonomy Match|BOLD:AAG1011
Tvetenia calvescens|BIOUG15792-E02|Kate Perez|BIN Taxonomy Match|BOLD:AAG1011
Tvetenia calvescens|BIOUG16333-D04|Kate Perez|BIN Taxonomy Match|BOLD:AAG1011
Tvetenia calvescens|BIOUG15800-B10|Kate Perez|BIN Taxonomy Match|BOLD:AAG1011
Tvetenia calvescens|BIOUG15799-B09|Kate Perez|BIN Taxonomy Match|BOLD:AAG1011
Tvetenia calvescens|BIOUG15798-G03|Kate Perez|BIN Taxonomy Match|BOLD:AAG1011
Tvetenia calvescens|BIOUG16332-A04|Kate Perez|BIN Taxonomy Match|BOLD:AAG1011
Tvetenia calvescens|BIOUG16551-H03|Kate Perez|BIN Taxonomy Match|BOLD:AAG1011
Tvetenia calvescens|BIOUG16396-C02|Kate Perez|BIN Taxonomy Match|BOLD:AAG1011
Tvetenia calvescens|BIOUG15806-F01|Kate Perez|BIN Taxonomy Match|BOLD:AAG1011
Tvetenia calvescens|BIOUG15797-F09|Kate Perez|BIN Taxonomy Match|BOLD:AAG1011
Tvetenia calvescens|BIOUG15765-F08|Kate Perez|BIN Taxonomy Match|BOLD:AAG1011
Tvetenia calvescens|BIOUG15762-B09|Kate Perez|BIN Taxonomy Match|BOLD:AAG1011
Tvetenia calvescens|BIOUG16711-E09|Kate Perez|BIN Taxonomy Match|BOLD:AAG1011
Tvetenia calvescens|BIOUG16331-G10|Kate Perez|BIN Taxonomy Match|BOLD:AAG1011
Tvetenia calvescens|BIOUG16333-B01|Kate Perez|BIN Taxonomy Match|BOLD:AAG1011
Tvetenia calvescens|BIOUG16631-G11|Kate Perez|BIN Taxonomy Match|BOLD:AAG1011
Tvetenia calvescens|BIOUG15596-C06|Kate Perez|BIN Taxonomy Match|BOLD:AAG1011
Tvetenia calvescens|BIOUG16479-E12|Kate Perez|BIN Taxonomy Match|BOLD:AAG1011
Tvetenia calvescens|BIOUG16713-D08|Kate Perez|BIN Taxonomy Match|BOLD:AAG1011
Tvetenia calvescens|BIOUG16632-B06|Kate Perez|BIN Taxonomy Match|BOLD:AAG1011
Tvetenia calvescens|BIOUG16551-G12|Kate Perez|BIN Taxonomy Match|BOLD:AAG1011
Tvetenia calvescens|BIOUG16713-F11|Kate Perez|BIN Taxonomy Match|BOLD:AAG1011
Tvetenia calvescens|BIOUG15792-A05|Kate Perez|BIN Taxonomy Match|BOLD:AAG1011
Tvetenia calvescens|BIOUG16713-A04|Kate Perez|BIN Taxonomy Match|BOLD:AAG1011
Tvetenia calvescens|BIOUG16713-B04|Kate Perez|BIN Taxonomy Match|BOLD:AAG1011
Tvetenia calvescens|BIOUG16711-C03|Kate Perez|BIN Taxonomy Match|BOLD:AAG1011
Tvetenia calvescens|BIOUG16630-G09|Kate Perez|BIN Taxonomy Match|BOLD:AAG1011
Tvetenia calvescens|BIOUG16713-H10|Kate Perez|BIN Taxonomy Match|BOLD:AAG1011
Tvetenia calvescens|BIOUG16626-A06|Kate Perez|BIN Taxonomy Match|BOLD:AAG1011
Tvetenia calvescens|BIOUG17025-C06|Kate Perez|BIN Taxonomy Match|BOLD:AAG1011
Tvetenia calvescens|BIOUG15792-C07|Kate Perez|BIN Taxonomy Match|BOLD:AAG1011
Tvetenia calvescens|BIOUG17023-A11|Kate Perez|BIN Taxonomy Match|BOLD:AAG1011
Tvetenia calvescens|BIOUG15720-F02|Kate Perez|BIN Taxonomy Match|BOLD:AAG1011
Tvetenia calvescens|Finnmark56|Elisabeth Stur||BOLD:AAG1011
Tvetenia calvescens|Finnmark557|Elisabeth Stur||BOLD:AAG1011
Tvetenia calvescens|Finnmark275|Elisabeth Stur||BOLD:AAG1011

[illegible]
Tvetenia calvescens|Finnmark56|Elisabeth Stur||BOLD:AAG1011
Tvetenia calvescens|Finnmark557|Elisabeth Stur||BOLD:AAG1011
Tvetenia calvescens|Finnmark275|Elisabeth Stur||BOLD:AAG1011
Psectrocladius platypus|ZMUO.025887|Lauri Paasivirta||BOLD:AAF6405
Psectrocladius platypus|ZMUO.025886|Lauri Paasivirta||BOLD:AAF6405
Psectrocladius conjungens|SOE40|Torbjorn Ekrem||BOLD:AAF6405
Psectrocladius conjungens|SOE39|Torbjorn Ekrem||BOLD:AAF6405
Psectrocladius sp. 08TE|Finnmark710|Torbjorn Ekrem|morphology|BOLD:AAV2884
Chironomidae|24405-G09|Mikko J Tiusanen|Tree based identification|BOLD:ACU5253
Tavastia|24512-H05|Kate Perez|BIN Taxonomy Match|BOLD:AAH2946
Tavastia|24505-B07|Mikko J Tiusanen|Tree based identification|BOLD:AAH2946
Tavastia|SOE318|Elisabeth Stur||BOLD:AAH2946
Mesocricotopus thienemanni|ZMUO.024282|Lauri Paasivirta||BOLD:AAP6934
Mesocricotopus thienemanni|Finnmark251|Elisabeth Stur||BOLD:AAP6934
Trissocladius brevipalpis|ZMUO.024664|Lauri Paasivirta||BOLD:ADA6853
Trissocladius brevipalpis|ZMUO.029783|Marko Mutanen||BOLD:ADA6853
Psectrocladius sp. 5TE|Finnmark715|Torbjorn Ekrem||BOLD:AAV2832
Psectrocladius sp. 5TE|Finnmark358|Torbjorn Ekrem||BOLD:AAV2832
Parakiefferiella|BIOUG16551-G07|Elisabeth Stur|BOLD ID Engine Manual|BOLD:ACP8764
Parakiefferiella|BIOUG15855-D06|Elisabeth Stur|BOLD ID Engine Manual|BOLD:ACP8764
Parakiefferiella|BIOUG15964-C04|Elisabeth Stur|BOLD ID Engine Manual|BOLD:ACP8764
Parakiefferiella|BIOUG16396-D10|Elisabeth Stur|BOLD ID Engine Manual|BOLD:ACP8764
Parakiefferiella|BIOUG16478-G09|Elisabeth Stur|BOLD ID Engine Manual|BOLD:ACP8764
Parakiefferiella|BIOUG16205-B12|Elisabeth Stur|BOLD ID Engine Manual|BOLD:ACP8764
Parakiefferiella|BIOUG16557-H07|Elisabeth Stur|BOLD ID Engine Manual|BOLD:ACP8764
Parakiefferiella|BIOUG16626-E04|Elisabeth Stur|BOLD ID Engine Manual|BOLD:ACP8764
Parakiefferiella|BIOUG15793-G05|Elisabeth Stur|BOLD ID Engine Manual|BOLD:ACP8764
Parakiefferiella|BIOUG16436-B12|Elisabeth Stur|BOLD ID Engine Manual|BOLD:ACP8764
Parakiefferiella|BIOUG16557-G02|Elisabeth Stur|BOLD ID Engine Manual|BOLD:ACP8764
Parakiefferiella|BIOUG16552-C11|Elisabeth Stur|BOLD ID Engine Manual|BOLD:ACP8764
Parakiefferiella|BIOUG15967-F07|Elisabeth Stur|BOLD ID Engine Manual|BOLD:ACP8764
Parakiefferiella|BIOUG16477-G08|Elisabeth Stur|morphology|BOLD:ACP9703
Parakiefferiella|BIOUG15809-F11|Elisabeth Stur|morphology|BOLD:ACP9703
Parakiefferiella|BIOUG15895-F02|Elisabeth Stur|morphology|BOLD:ACP9703
Parakiefferiella|BIOUG16478-A06|Elisabeth Stur|BOLD ID Engine Manual|BOLD:ACP9703
Parakiefferiella|BIOUG16431-D02|Elisabeth Stur|morphology|BOLD:ACP9703
Parakiefferiella|BIOUG15855-F05|Elisabeth Stur|morphology|BOLD:ACP9703
Parakiefferiella|BIOUG16552-G06|Elisabeth Stur|morphology|BOLD:ACP9703
Parakiefferiella|BIOUG16480-D06|Elisabeth Stur|BOLD ID Engine Manual|BOLD:ACQ6468
Parakiefferiella|BIOUG15967-D05|Elisabeth Stur|BOLD ID Engine Manual|BOLD:ACQ6468
Chironomidae|BIOUG16479-C10|Kate Perez|BOLD ID Engine Manual|BOLD:ACP6740
Chironomidae|BIOUG15808-H07|Kate Perez|Tree based identification
Chironomidae|BIOUG16552-F09|Kate Perez|BOLD ID Engine Manual|BOLD:ACP6740
Chironomidae|BIOUG15895-E04|Kate Perez|BOLD ID Engine Manual|BOLD:ACP6740
Chironomidae|BIOUG16552-H10|Kate Perez|BOLD ID Engine Manual|BOLD:ACP6740
Chironomidae|BIOUG15855-C03|Kate Perez|BOLD ID Engine Manual|BOLD:ACP6740
Chironomidae|BIOUG16435-B12|Kate Perez|BOLD ID Engine Manual|BOLD:ACP6740
Chironomidae|BIOUG15852-E07|Kate Perez|BOLD ID Engine Manual|BOLD:ACP6740
Chironomidae|BIOUG15852-H04|Kate Perez|BOLD ID Engine Manual|BOLD:ACP6740
Chironomidae|BIOUG16476-G04|Kate Perez|BOLD ID Engine Manual|BOLD:ACP6740
Chironomidae|BIOUG15852-G06|Kate Perez|BOLD ID Engine Manual|BOLD:ACP6740
Chironomidae|BIOUG16436-F11|Kate Perez|BOLD ID Engine Manual|BOLD:ACP6740
Chironomidae|BIOUG16557-H10|Kate Perez|BOLD ID Engine Manual|BOLD:ACP6740
Chironomidae|BIOUG16431-A11|Kate Perez|BOLD ID Engine Manual|BOLD:ACP6740
Chironomidae|BIOUG15895-C10|Kate Perez|BOLD ID Engine Manual|BOLD:ACP6740
Chironomidae|BIOUG16332-D04|Kate Perez|BOLD ID Engine Manual|BOLD:ACP6740
Chironomidae|BIOUG16207-F03|Kate Perez|BOLD ID Engine Manual|BOLD:ACP6740
Chironomidae|BIOUG16208-A03|Kate Perez|BOLD ID Engine Manual|BOLD:ACP6740
Chironomidae|BIOUG16208-C03|Kate Perez|BOLD ID Engine Manual|BOLD:ACP6740
Chironomidae|BIOUG16432-F05|Kate Perez|BOLD ID Engine Manual|BOLD:ACP6740
Chironomidae|BIOUG16552-F01|Kate Perez|BOLD ID Engine Manual|BOLD:ACP6740
Chironomidae|BIOUG15795-C09|Kate Perez|BOLD ID Engine Manual|BOLD:ACP6740
Chironomidae|BIOUG15797-F08|Kate Perez|BOLD ID Engine Manual|BOLD:ACP6740
Chironomidae|BIOUG16552-A11|Kate Perez|BOLD ID Engine Manual|BOLD:ACP6740
Chironomidae|BIOUG16396-E04|Kate Perez|BOLD ID Engine Manual|BOLD:ACP6740
Chironomidae|BIOUG16433-G11|Kate Perez|BOLD ID Engine Manual|BOLD:ACP6740
Chironomidae|BIOUG16398-G08|Kate Perez|BOLD ID Engine Manual|BOLD:ACP6740
Chironomidae|BIOUG16552-H09|Kate Perez|BOLD ID Engine Manual|BOLD:ACP6740
Chironomidae|BIOUG15795-D10|Kate Perez|BOLD ID Engine Manual|BOLD:ACP6740
Chironomidae|BIOUG16205-E07|Kate Perez|BOLD ID Engine Manual|BOLD:ACP6740
Chironomidae|BIOUG16557-H09|Kate Perez|BOLD ID Engine Manual|BOLD:ACP6740
Chironomidae|BIOUG16552-B01|Kate Perez|BOLD ID Engine Manual|BOLD:ACP6740
Chironomidae|BIOUG16432-D04|Kate Perez|BOLD ID Engine Manual|BOLD:ACP6740
Chironomidae|BIOUG16476-G10|Kate Perez|BOLD ID Engine Manual|BOLD:ACP6740
Chironomidae|BIOUG15794-F12|Kate Perez|BOLD ID Engine Manual|BOLD:ACP6740
Chironomidae|BIOUG16259-F10|Kate Perez|BOLD ID Engine Manual|BOLD:ACP6740
Chironomidae|BIOUG15899-C09|Kate Perez|BOLD ID Engine Manual|BOLD:ACP6740
Chironomidae|BIOUG16433-G01|Kate Perez|BOLD ID Engine Manual|BOLD:ACP6740
Chironomidae|BIOUG16557-G05|Kate Perez|BOLD ID Engine Manual|BOLD:ACP6740
Chironomidae|BIOUG16436-A11|Kate Perez|BOLD ID Engine Manual|BOLD:ACP6740
Chironomidae|BIOUG15795-H02|Kate Perez|BOLD ID Engine Manual|BOLD:ACP6740
Chironomidae|BIOUG16592-B10|Kate Perez|BOLD ID Engine Manual|BOLD:ACP6740
Chironomidae|BIOUG16431-G03|Kate Perez|BOLD ID Engine Manual|BOLD:ACP6740
Chironomidae|BIOUG16432-D03|Kate Perez|BOLD ID Engine Manual|BOLD:ACP6740
Chironomidae|BIOUG16476-A10|Kate Perez|BOLD ID Engine Manual|BOLD:ACP6740
Chironomidae|BIOUG16431-D05|Kate Perez|BOLD ID Engine Manual|BOLD:ACP6740
Chironomidae|BIOUG16477-H03|Kate Perez|BOLD ID Engine Manual|BOLD:ACP6740
Chironomidae|BIOUG16435-D03|Kate Perez|BOLD ID Engine Manual|BOLD:ACP6740
Chironomidae|BIOUG16598-B02|Kate Perez|BOLD ID Engine Manual|BOLD:ACP6740
Chironomidae|BIOUG16205-B09|Kate Perez|BOLD ID Engine Manual|BOLD:ACP6740
Chironomidae|BIOUG16592-B09|Kate Perez|BOLD ID Engine Manual|BOLD:ACP6740
Chironomidae|BIOUG16592-C02|Kate Perez|BOLD ID Engine Manual|BOLD:ACP6740
Chironomidae|BIOUG16557-H03|Kate Perez|BOLD ID Engine Manual|BOLD:ACP6740
Chironomidae|BIOUG16557-F07|Kate Perez|BOLD ID Engine Manual|BOLD:ACP6740
Chironomidae|BIOUG16555-G11|Kate Perez|BOLD ID Engine Manual|BOLD:ACP6740
Chironomidae|BIOUG15806-A12|Kate Perez|BOLD ID Engine Manual|BOLD:ACP6740
Chironomidae|BIOUG16552-B08|Kate Perez|BOLD ID Engine Manual|BOLD:ACP6740
Chironomidae|BIOUG15798-F01|Kate Perez|BOLD ID Engine Manual|BOLD:ACP6740
Chironomidae|BIOUG15800-D05|Kate Perez|BOLD ID Engine Manual|BOLD:ACP6740
Parakiefferiella|BIOUG15761-B09|Elisabeth Stur|morphology|BOLD:ACP6740
Halocladius fucicola|NHRS-BYWS000000485|Yngve Brodin|morphology|BOLD:ADJ6113
Halocladius fucicola|NHRS-BYWS000000474|Yngve Brodin|morphology|BOLD:ADJ6113
[illegible]

Parakiefferiella|BIOUG15761-B09|Elisabeth Stur|morphology|BOLD:ACP6740
Halocladius fucicola|NHRS-BYWS000000485|Yngve Brodin|morphology|BOLD:ADJ6113
Halocladius fucicola|NHRS-BYWS000000474|Yngve Brodin|morphology|BOLD:ADJ6113
Halocladius fucicola|NHRS-BYWS000000473|Yngve Brodin|morphology|BOLD:ADJ6113
Halocladius fucicola|NHRS-BYWS000000472|Yngve Brodin|morphology|BOLD:ADJ6113
Halocladius variabilis|NHRS-BYWS000000482|Yngve Brodin|morphology|BOLD:AAB8751
Halocladius variabilis|CH-OSF160|Elisabeth Stur||BOLD:AAB8751
Halocladius variabilis|NHRS-BYWS000000468|Yngve Brodin|morphology|BOLD:AAB8751
Halocladius variabilis|CH-OSF94|Elisabeth Stur||BOLD:AAB8751
Halocladius variabilis|NHRS-BYWS000000172|Yngve Brodin|morphology|BOLD:AAB8751
Halocladius variabilis|NHRS-BYWS000000424|Yngve Brodin|morphology|BOLD:AAB8751
Halocladius variabilis|CH-OSF92|Elisabeth Stur||BOLD:AAB8751
Halocladius variabilis|CH-eik94|Elisabeth Stur||BOLD:AAB8751
Halocladius variabilis|CH-OSF76|Elisabeth Stur||BOLD:AAB8751
Halocladius variabilis|NHRS-BYWS000000308|Yngve Brodin|morphology|BOLD:AAB8751
Halocladius variabilis|NHRS-BYWS000000481|Yngve Brodin|morphology|BOLD:AAB8751
Halocladius variabilis|NHRS-BYWS000001768|Yngve Brodin||BOLD:AAB8751
Halocladius variabilis|Finnmark67|Elisabeth Stur||BOLD:AAB8751
Halocladius variabilis|CH-OSF93|Elisabeth Stur||BOLD:AAB8751
Halocladius variabilis|TRD-CH332|Elisabeth Stur||BOLD:AAB8751
Halocladius variabilis|RIN_CH02|Mona Renate Saursaunet|BOLD Identification engine|BOLD:AAB8751
Halocladius variabilis|NHRS-BYWS000000179|Yngve Brodin|morphology|BOLD:AAB8751
Halocladius variabilis|Finnmark435|Elisabeth Stur||BOLD:AAB8751
Halocladius variabilis|Finnmark94|Elisabeth Stur||BOLD:AAB8751
Halocladius variabilis|CH-eik107|Elisabeth Stur||BOLD:AAB8751
Halocladius variabilis|ZMUO.025243|Lauri Paasivirta||BOLD:AAB8751
Halocladius variabilis|CH-eik84|Elisabeth Stur||BOLD:AAB8751
Halocladius variabilis|CH-eik99|Elisabeth Stur||BOLD:AAB8751
Halocladius variabilis|CH-eik95|Elisabeth Stur||BOLD:AAB8751
Halocladius variabilis|Finnmark694|Elisabeth Stur||BOLD:AAB8751
Halocladius variabilis|CH-OSF161|Elisabeth Stur||BOLD:AAB8751
Halocladius variabilis|CH-eik96|Elisabeth Stur||BOLD:AAB8751
Halocladius variabilis|NHRS-BYWS000000410|Yngve Brodin|morphology|BOLD:AAB8751
Halocladius variabilis|TRD-CH163|Elisabeth Stur||BOLD:AAB8751
Halocladius variabilis|ZMUO.025244|Lauri Paasivirta||BOLD:AAB8751
Halocladius variabilis|TRD-CH162|Elisabeth Stur
Halocladius variabilis|BIOUG16260-G01|Kate Perez|BIN Taxonomy Match|BOLD:AAB8751
Halocladius variabilis|CH-OSF77|Elisabeth Stur||BOLD:AAB8751
Halocladius variabilis|NHRS-BYWS000001849|Yngve Brodin||BOLD:AAB8751
Halocladius variabilis|Finnmark329|Elisabeth Stur||BOLD:AAB8751
Halocladius variabilis|Finnmark311|Elisabeth Stur||BOLD:AAB8751
Halocladius variabilis|Finnmark638|Elisabeth Stur||BOLD:AAB8751
Halocladius variabilis|NHRS-BYWS000000173|Yngve Brodin|morphology|BOLD:AAB8751
Halocladius variabilis|Finnmark407|Elisabeth Stur||BOLD:AAB8751
Halocladius variabilis|NHRS-BYWS000001769|Yngve Brodin||BOLD:AAB8751
Halocladius variabilis|Finnmark294|Elisabeth Stur||BOLD:AAB8751
Nilotanypus|BIOUG07413-G10|Valerie Levesque-Beaudin||BOLD:ACI6308
Nilotanypus|BIOUG07807-H04|Kate Perez|BIN Taxonomy Match|BOLD:ACI6308
Nilotanypus|BIOUG08125-C07|Valerie Levesque-Beaudin||BOLD:ACI6308
Nilotanypus dubius|EBAI-Ch162|Elisabeth Stur|morphology|BOLD:AAP5790
Nilotanypus dubius|TRD-Cer195|Elisabeth Stur||BOLD:AAP5790
Nilotanypus dubius|EBAI-Ch161|Elisabeth Stur|morphology|BOLD:AAP5790
Nilotanypus dubius|EBAI-Ch246|Elisabeth Stur|morphology|BOLD:AAP5790
Nilotanypus dubius|TRD-Cer210|Elisabeth Stur||BOLD:AAP5790
Nilotanypus dubius|TRD-Cer209|Elisabeth Stur||BOLD:AAP5790
Nilotanypus dubius|TRD-Cer208|Elisabeth Stur||BOLD:AAP5790
Nilotanypus dubius|Finnmark877|G. A. Halvorsen||BOLD:AAP5790
Nilotanypus dubius|Finnmark210|Elisabeth Stur||BOLD:AAP5790
Nilotanypus dubius|Finnmark209|Elisabeth Stur||BOLD:AAP5790
Nilotanypus dubius|Finnmark173|Elisabeth Stur||BOLD:AAP5790
Nilotanypus dubius|Finnmark256|Elisabeth Stur||BOLD:AAP5790
Synorthocladius|BIOUG17112-D12|Kate Perez|BOLD ID Engine Manual|BOLD:ACR6475
Synorthocladius semivirens|EJV-20110200|Lauri Paasivirta||BOLD:ACM5335
Chironomidae|BI2019_K05|Knut Andreas Eikland|BOLD ID-Engine
Synorthocladius semivirens|CH-OSF140|Elisabeth Stur||BOLD:ACM5335
Synorthocladius semivirens|CH-OSF139|Elisabeth Stur||BOLD:ACM5335
Synorthocladius semivirens|EJV-20110197|Lauri Paasivirta||BOLD:ACM5335
Synorthocladius semivirens|ZMUO.024607|Lauri Paasivirta||BOLD:ACM5335
Synorthocladius semivirens|NHRS-BYWS000000150|Yngve Brodin|morphology|BOLD:ACM5335
Synorthocladius semivirens|EJV-20110199|Lauri Paasivirta||BOLD:ACM5335
Synorthocladius semivirens|EJV-20110198|Lauri Paasivirta||BOLD:ACM5335
Synorthocladius semivirens|BIOUG42738-B04|Kate Perez|BIN Taxonomy Match below Phylum (Jun 2019...
Synorthocladius semivirens|Finnmark847|Elisabeth Stur||BOLD:AAK6026
Synorthocladius semivirens|NHRS-BYWS000000926|Yngve Brodin|morphology|BOLD:AAK6026
Synorthocladius semivirens|BIOUG16330-E04|Kate Perez|BIN Taxonomy Match|BOLD:AAK6026
Synorthocladius semivirens|BIOUG16299-G07|Kate Perez|BIN Taxonomy Match|BOLD:AAK6026
Synorthocladius semivirens|BIOUG16329-F10|Kate Perez|BIN Taxonomy Match|BOLD:AAK6026
Synorthocladius semivirens|ZMUO.024606|Lauri Paasivirta||BOLD:AAK6026
Synorthocladius semivirens|BIOUG16398-C02|Kate Perez|BIN Taxonomy Match|BOLD:AAK6026
Synorthocladius semivirens|SOE200|Elisabeth Stur||BOLD:AAK6026
Synorthocladius semivirens|BIOUG15964-F02|Kate Perez|BIN Taxonomy Match|BOLD:AAK6026
Synorthocladius semivirens|BIOUG16596-F02|Kate Perez|BIN Taxonomy Match|BOLD:AAK6026
Synorthocladius semivirens|BIOUG16299-H07|Kate Perez|BIN Taxonomy Match|BOLD:AAK6026
Synorthocladius semivirens|BIOUG16434-C06|Kate Perez|BIN Taxonomy Match|BOLD:AAK6026
Synorthocladius semivirens|BIOUG16328-H08|Kate Perez|BIN Taxonomy Match|BOLD:AAK6026
Synorthocladius semivirens|BIOUG16529-D10|Kate Perez|BIN Taxonomy Match|BOLD:AAK6026
Chironomidae|BIOUG55148-C02|Renee Miskie|BIN Taxonomy Match (Jan 2021)|BOLD:ADZ8727
Chironomidae|BIOUG07418-F08|Meredith Miller|Tree-based Identification (Feb 2019)
Synorthocladius semivirens|Finnmark825|Elisabeth Stur||BOLD:ACM5930
Synorthocladius semivirens|Finnmark827|Elisabeth Stur||BOLD:ACM5930
Synorthocladius semivirens|Finnmark831|Elisabeth Stur||BOLD:ACM5930
Synorthocladius semivirens|Finnmark824|Elisabeth Stur||BOLD:ACM5930
Synorthocladius semivirens|Finnmark810|Elisabeth Stur||BOLD:ACM5930
Synorthocladius semivirens|Finnmark267|Elisabeth Stur||BOLD:ACM5930
Cricotopus triannulatus|BIOUG55112-F11|Kate Perez|BIN Taxonomy Match (Oct 2020)|BOLD:ACR...
Cricotopus triannulatus|NHRS-BYWS000000570|Yngve Brodin|morphology|BOLD:ACR4519
Cricotopus triannulatus|NHRS-BYWS000000486|Yngve Brodin|morphology|BOLD:ACR4519
Cricotopus triannulatus|NHRS-BYWS000000322|Yngve Brodin|morphology|BOLD:ACR4519
Cricotopus triannulatus|NHRS-BYWS000000471|Yngve Brodin|morphology|BOLD:ACR4519
Cricotopus triannulatus|EJV-20110193|Lauri Paasivirta||BOLD:ACR4519
Cricotopus triannulatus|NHRS-BYWS000000163|Yngve Brodin|morphology|BOLD:ACR4519
Cricotopus triannulatus|ZMUO.024689|Lauri Paasivirta||BOLD:ACR4519
Cricotopus triannulatus|ES396|Elisabeth Stur|Morphology|BOLD:ACR4519

Cricotopus triannulatus|NHRS-BYWS000000163|Yngve Brodin|morphology|BOLD:ACR4519
Cricotopus triannulatus|ZMUO.024689|Lauri Paasivirta||BOLD:ACR4519
Cricotopus triannulatus|ES396|Elisabeth Stur|Morphology|BOLD:ACR4519
Cricotopus triannulatus|ZSM-DIP-33140-H06|Caroline Chimeno|BIN Taxonomy Match (Jan 2022)|BO...
Cricotopus triannulatus|BIOUG16620-G09|Kate Perez|BIN Taxonomy Match|BOLD:ACR4519
Cricotopus triannulatus|NHRS-BYWS000000948|Yngve Brodin|morphology|BOLD:ACR4519
Cricotopus triannulatus|NHRS-BYWS000000540|Yngve Brodin|morphology|BOLD:ACR4519
Cricotopus triannulatus|ZMUO.024688|Lauri Paasivirta||BOLD:ACR4519
Cricotopus triannulatus|EJV-20110191|Lauri Paasivirta||BOLD:ACR4519
Chironomidae|CCDB24038-F08|Jerome Moriniere|BIN Taxonomy Match|BOLD:ACU4522
Chironomidae|CCDB24037-A09|Jerome Moriniere|BIN Taxonomy Match|BOLD:ACU4522
Smittia pratorum|ZSM-DIP-33141-E03|Caroline Chimeno|BIN Taxonomy Match (Jan 2022)|BOLD:A...
Smittia pratorum|ZSM-DIP-33147-G12|Caroline Chimeno|BIN Taxonomy Match (Jan 2022)|BOLD:A...
Smittia pratorum|ZSM-DIP-33141-E02|Caroline Chimeno|BIN Taxonomy Match (Jan 2022)|BOLD:A...
Smittia pratorum|ZSM-DIP-33141-D12|Caroline Chimeno|BIN Taxonomy Match (Jan 2022)|BOLD:A...
Smittia pratorum|ZSM-DIP-33141-D11|Caroline Chimeno|BIN Taxonomy Match (Jan 2022)|BOLD:A...
Smittia pratorum|ZSM-DIP-33134-F07|Caroline Chimeno|BIN Taxonomy Match (Jan 2022)|BOLD:A...
Smittia pratorum|ZSM-DIP-33134-F09|Caroline Chimeno|BIN Taxonomy Match (Jan 2022)|BOLD:A...
Smittia pratorum|ZSM-DIP-33136-E04|Caroline Chimeno|BIN Taxonomy Match (Jan 2022)|BOLD:A...
Smittia pratorum|ZSM-DIP-33141-E01|Caroline Chimeno|BIN Taxonomy Match (Jan 2022)|BOLD:A...
Smittia pratorum|ZSM-DIP-33134-F08|Caroline Chimeno|BIN Taxonomy Match (Jan 2022)|BOLD:A...
Smittia pratorum|ZSM-DIP-33140-D09|Caroline Chimeno|BIN Taxonomy Match (Jan 2022)|BOLD:A...
Smittia pratorum|TRD-CH48|Elisabeth Stur|morphology|BOLD:ACM5388
Smittia sp. 8ES|BIOUG16734-F04|Kate Perez|BIN Taxonomy Match|BOLD:ACP4736
Smittia sp. 8ES|BIOUG16855-C04|Kate Perez|BIN Taxonomy Match|BOLD:ACP4736
Smittia sp. 8ES|BIOUG16859-F12|Kate Perez|BIN Taxonomy Match|BOLD:ACP4736
Smittia|BIOUG42961-B12|Kate Perez|BIN Taxonomy Match below Phylum (Jun 2019)|BOLD:ACP4736
Smittia sp. 8ES|BIOUG17444-B04|Kate Perez|BIN Taxonomy Match|BOLD:ACP4736
Smittia|BIOUG43078-H02|Kate Perez|BIN Taxonomy Match below Phylum (Jun 2019)|BOLD:ACP4736
Smittia|BIOUG43077-B09|Kate Perez|BIN Taxonomy Match below Phylum (Jun 2019)|BOLD:ACP4736
Smittia sp. 8ES|BIOUG16351-E10|Kate Perez|BIN Taxonomy Match|BOLD:ACP4736
Smittia sp. 8ES|BIOUG15648-F08|Kate Perez|BIN Taxonomy Match|BOLD:ACP4736
Smittia sp. 8ES|BIOUG15649-F08|Kate Perez|BIN Taxonomy Match|BOLD:ACP4736
Smittia|BIOUG43077-D10|Kate Perez|BIN Taxonomy Match below Phylum (Jun 2019)|BOLD:ACP4736
Smittia|BIOUG43083-D07|Kate Perez|BIN Taxonomy Match below Phylum (May 2019)|BOLD:ACP4736
Smittia|BIOUG43074-F05|Kate Perez|BIN Taxonomy Match below Phylum (Jun 2019)|BOLD:ACP4736
Smittia sp. 8ES|BIOUG05144-A08|Kate Perez|BIN Taxonomy Match|BOLD:ACP4736
Smittia sp. 8ES|BIOUG17022-E02|Kate Perez|BIN Taxonomy Match|BOLD:ACP4736
Smittia sp. 8ES|BIOUG16358-A08|Kate Perez|BIN Taxonomy Match|BOLD:ACP4736
Smittia sp. 8ES|BIOUG17064-A12|Kate Perez|BIN Taxonomy Match|BOLD:ACP4736
Smittia sp. 8ES|BIOUG17064-C10|Kate Perez|BIN Taxonomy Match|BOLD:ACP4736
Smittia sp. 8ES|BIOUG17064-G12|Kate Perez|BIN Taxonomy Match|BOLD:ACP4736
Smittia sp. 8ES|BIOUG17025-F02|Kate Perez|BIN Taxonomy Match|BOLD:ACP4736
Smittia sp. 8ES|BIOUG17064-B10|Kate Perez|BIN Taxonomy Match|BOLD:ACP4736
Smittia|BIOUG36841-D09|Kate Perez|BIN Taxonomy Match (Jul 2018)|BOLD:ACP4736
Smittia sp. 8ES|BIOUG16713-D06|Kate Perez|BIN Taxonomy Match|BOLD:ACP4736
Smittia sp. 8ES|BIOUG16480-C11|Kate Perez|BIN Taxonomy Match|BOLD:ACP4736
Smittia|BIOUG42972-C06|Kate Perez|BIN Taxonomy Match below Phylum (Jun 2019)|BOLD:ACP4736
Smittia sp. 8ES|BIOUG16479-F08|Kate Perez|BIN Taxonomy Match|BOLD:ACP4736
Smittia sp. 8ES|BIOUG16431-C05|Kate Perez|BIN Taxonomy Match|BOLD:ACP4736
Smittia sp. 8ES|BIOUG15807-E06|Kate Perez|BIN Taxonomy Match|BOLD:ACP4736
Smittia sp. 8ES|BIOUG17064-F07|Kate Perez|BIN Taxonomy Match|BOLD:ACP4736
Smittia sp. 8ES|BIOUG17065-B02|Kate Perez|BIN Taxonomy Match|BOLD:ACP4736
Smittia sp. 8ES|BIOUG17066-A12|Kate Perez|BIN Taxonomy Match|BOLD:ACP4736
Smittia sp. 8ES|BIOUG16360-G09|Kate Perez|BIN Taxonomy Match|BOLD:ACP4736
Smittia sp. 8ES|BIOUG16351-F05|Kate Perez|BIN Taxonomy Match|BOLD:ACP4736
Smittia sp. 8ES|BIOUG16736-H06|Kate Perez|BIN Taxonomy Match|BOLD:ACP4736
Smittia|BIOUG36776-E08|Kate Perez|BIN Taxonomy Match (Jul 2018)|BOLD:ACP4736
Smittia|BIOUG36932-E12|Kate Perez|BIN Taxonomy Match (Jul 2018)|BOLD:ACP4736
Smittia|BIOUG36992-H05|Kate Perez|BIN Taxonomy Match (Jul 2018)|BOLD:ACP4736
Smittia sp. 8ES|BIOUG16707-C06|Kate Perez|BIN Taxonomy Match|BOLD:ACP4736
Smittia sp. 8ES|BIOUG15949-H11|Kate Perez|BIN Taxonomy Match|BOLD:ACP4736
Smittia sp. 8ES|BIOUG15809-C01|Kate Perez|BIN Taxonomy Match|BOLD:ACP4736
Smittia sp. 8ES|BIOUG17094-B04|Kate Perez|BIN Taxonomy Match|BOLD:ACP4736
Smittia sp. 8ES|BIOUG16736-C04|Kate Perez|BIN Taxonomy Match|BOLD:ACP4736
Smittia sp. 8ES|BIOUG16778-E02|Kate Perez|BIN Taxonomy Match|BOLD:ACP4736
Smittia sp. 8ES|BIOUG17064-F09|Kate Perez|BIN Taxonomy Match|BOLD:ACP4736
Smittia sp. 8ES|BIOUG16779-C01|Kate Perez|BIN Taxonomy Match|BOLD:ACP4736
Smittia sp. 8ES|BIOUG16705-B11|Kate Perez|BIN Taxonomy Match|BOLD:ACP4736
Smittia sp. 8ES|BIOUG15456-C10|Kate Perez|BIN Taxonomy Match|BOLD:ACP4736
Smittia sp. 8ES|BIOUG17095-H09|Kate Perez|BIN Taxonomy Match|BOLD:ACP4736
Smittia sp. 8ES|BIOUG04212-F07|Kate Perez|BIN Taxonomy Match|BOLD:ACP4736
Smittia sp. 8ES|BIOUG16705-D07|Kate Perez|BIN Taxonomy Match|BOLD:ACP4736
Smittia sp. 8ES|BIOUG06062-C02|Kate Perez|BIN Taxonomy Match|BOLD:ACP4736
Smittia sp. 8ES|BIOUG04089-E12|Kate Perez|BIN Taxonomy Match|BOLD:ACP4736
Smittia sp. 8ES|BIOUG04362-C04|Kate Perez|BIN Taxonomy Match|BOLD:ACP4736
Smittia|BIOUG42961-G06|Kate Perez|BIN Taxonomy Match below Phylum (Jun 2019)|BOLD:ACP4736
Smittia|BIOUG42961-C05|Kate Perez|BIN Taxonomy Match below Phylum (Jun 2019)|BOLD:ACP4736
Smittia|BIOUG42963-A02|Kate Perez|BIN Taxonomy Match below Phylum (Jun 2019)|BOLD:ACP4736
Smittia|BIOUG42962-B10|Kate Perez|BIN Taxonomy Match below Phylum (Jun 2019)|BOLD:ACP4736
Smittia|BIOUG43077-F05|Kate Perez|BIN Taxonomy Match below Phylum (Jun 2019)|BOLD:ACP4736
Smittia|BIOUG43077-E02|Kate Perez|BIN Taxonomy Match below Phylum (Jun 2019)|BOLD:ACP4736
Smittia|BIOUG43078-F02|Kate Perez|BIN Taxonomy Match below Phylum (Jun 2019)|BOLD:ACP4736
Smittia|BIOUG43078-E07|Kate Perez|BIN Taxonomy Match below Phylum (Jun 2019)|BOLD:ACP4736
Smittia|BIOUG55105-F06|Stefan Schmidt|BIN Taxonomy Match (Sep 2020)|BOLD:ACP4736
Smittia|BIOUG42940-G02|Kate Perez|BIN Taxonomy Match below Phylum (Jun 2019)|BOLD:ACP4736
Smittia|BIOUG42959-G09|Kate Perez|BIN Taxonomy Match below Phylum (Jun 2019)|BOLD:ACP4736
Smittia|BIOUG42959-F02|Kate Perez|BIN Taxonomy Match below Phylum (Jun 2019)|BOLD:ACP4736
Smittia|BIOUG42713-A12|Kate Perez|BIN Taxonomy Match below Phylum (Jun 2019)|BOLD:ACP4736
Smittia|BIOUG55103-A10|Stefan Schmidt|BIN Taxonomy Match (Sep 2020)|BOLD:ACP4736
Smittia|BIOUG42964-F02|Kate Perez|BIN Taxonomy Match below Phylum (Jun 2019)|BOLD:ACP4736
Smittia|BIOUG42963-D09|Kate Perez|BIN Taxonomy Match below Phylum (Jun 2019)|BOLD:ACP4736
Smittia|BIOUG42961-B04|Kate Perez|BIN Taxonomy Match below Phylum (Jun 2019)|BOLD:ACP4736
Smittia|BIOUG42961-A08|Kate Perez|BIN Taxonomy Match below Phylum (Jun 2019)|BOLD:ACP4736
Smittia|BIOUG42948-C01|Kate Perez|BIN Taxonomy Match below Phylum (Jun 2019)|BOLD:ACP4736
Smittia|BIOUG43074-E04|Kate Perez|BIN Taxonomy Match below Phylum (Jun 2019)|BOLD:ACP4736
Smittia|BIOUG43075-B05|Kate Perez|BIN Taxonomy Match below Phylum (Jun 2019)|BOLD:ACP4736
Smittia|BIOUG43075-E05|Kate Perez|BIN Taxonomy Match below Phylum (Jun 2019)|BOLD:ACP4736
Smittia|BIOUG43076-G04|Kate Perez|BIN Taxonomy Match below Phylum (Jun 2019)|BOLD:ACP4736
Smittia|BIOUG43079-A02|Kate Perez|BIN Taxonomy Match below Phylum (Jun 2019)|BOLD:ACP4736
Smittia|BIOUG43079-F12|Kate Perez|BIN Taxonomy Match below Phylum (Jun 2019)|BOLD:ACP4736
Smittia|BIOUG42960-E05|Kate Perez|BIN Taxonomy Match below Phylum (Jun 2019)|BOLD:ACP4736
Smittia|BIOUG42683-G05|Kate Perez|BIN Taxonomy Match below Phylum (Jun 2019)|BOLD:ACP4736

[illegible]
Smittia|BIOUG43079-F12|Kate Perez|BIN Taxonomy Match below Phylum (Jun 2019)|BOLD:ACP4736
Smittia|BIOUG42960-E05|Kate Perez|BIN Taxonomy Match below Phylum (Jun 2019)|BOLD:ACP4736
Smittia|BIOUG42683-G05|Kate Perez|BIN Taxonomy Match below Phylum (Jun 2019)|BOLD:ACP4736
Smittia|BIOUG43079-E10|Kate Perez|BIN Taxonomy Match below Phylum (Jun 2019)|BOLD:ACP4736
Smittia|BIOUG42712-B07|Kate Perez|BIN Taxonomy Match below Phylum (Jun 2019)|BOLD:ACP4736
Smittia|BIOUG43081-A03|Kate Perez|BIN Taxonomy Match below Phylum (Jun 2019)|BOLD:ACP4736
Smittia sp. 8ES|BIOUG16270-E11|Kate Perez|BIN Taxonomy Match|BOLD:ACP4736
Smittia sp. 8ES|BIOUG17065-A01|Kate Perez|BIN Taxonomy Match|BOLD:ACP4736
Smittia sp. 8ES|BIOUG04089-A05|Kate Perez|BIN Taxonomy Match|BOLD:ACP4736
Smittia|BIOUG43078-H10|Kate Perez|BIN Taxonomy Match below Phylum (Jun 2019)|BOLD:ACP4736
Smittia sp. 8ES|CH-eik09|Elisabeth Stur||BOLD:ACP4736
Smittia sp. 8ES|CH-eik123|Elisabeth Stur||BOLD:ACP4736
Smittia sp. 8ES|CH-eik08|Elisabeth Stur||BOLD:ACP4736
Smittia sp. 8ES|TRD-CH416|Elisabeth Stur|morphology|BOLD:ACP4736
Smittia sp. 8ES|BIOUG17096-A02|Kate Perez|BIN Taxonomy Match|BOLD:ACP4736
Smittia|BIOUG36818-E08|Kate Perez|BIN Taxonomy Match (Jul 2018)|BOLD:ACP4736
Smittia sp. 8ES|BIOUG16709-D08|Kate Perez|BIN Taxonomy Match|BOLD:ACP4736
Smittia|BIOUG36766-C10|Kate Perez|BIN Taxonomy Match (Jul 2018)|BOLD:ACP4736
Smittia sp. 8ES|BIOUG16736-G11|Kate Perez|BIN Taxonomy Match|BOLD:ACP4736
Smittia sp. 8ES|BIOUG17064-D04|Kate Perez|BIN Taxonomy Match|BOLD:ACP4736
Smittia sp. 8ES|BIOUG17064-E11|Kate Perez|BIN Taxonomy Match|BOLD:ACP4736
Smittia sp. 8ES|BIOUG17064-F10|Kate Perez|BIN Taxonomy Match|BOLD:ACP4736
Smittia sp. 8ES|BIOUG15674-G07|Kate Perez|BIN Taxonomy Match|BOLD:ACP4736
Smittia sp. 8ES|BIOUG17065-B03|Kate Perez|BIN Taxonomy Match|BOLD:ACP4736
Smittia sp. 8ES|BIOUG16712-D04|Kate Perez|BIN Taxonomy Match|BOLD:ACP4736
Smittia sp. 8ES|BIOUG16705-C09|Kate Perez|BIN Taxonomy Match|BOLD:ACP4736
Smittia sp. 8ES|BIOUG36680-H02|Kate Perez|BIN Taxonomy Match (May 2018)|BOLD:ACP4736
Smittia sp. 8ES|BIOUG17064-C01|Kate Perez|BIN Taxonomy Match|BOLD:ACP4736
Smittia sp. 8ES|BIOUG36601-B02|Kate Perez|BIN Taxonomy Match (May 2018)|BOLD:ACP4736
Smittia sp. 8ES|BIOUG16434-G11|Kate Perez|BIN Taxonomy Match|BOLD:ACP4736
Smittia sp. 8ES|BIOUG36684-F02|Kate Perez|BIN Taxonomy Match (May 2018)|BOLD:ACP4736
Smittia|BIOUG43081-E12|Kate Perez|BIN Taxonomy Match below Phylum (Jun 2019)|BOLD:ACP4736
Smittia sp. 8ES|BIOUG16706-E06|Kate Perez|BIN Taxonomy Match|BOLD:ACP4736
Smittia sp. 8ES|BIOUG16706-H10|Kate Perez|BIN Taxonomy Match|BOLD:ACP4736
Smittia|BIOUG36828-D08|Kate Perez|BIN Taxonomy Match (Jul 2018)|BOLD:ACP4736
Smittia|BIOUG36827-F12|Kate Perez|BIN Taxonomy Match (Jul 2018)|BOLD:ACP4736
Smittia sp. 8ES|BIOUG16278-D08|Kate Perez|BIN Taxonomy Match|BOLD:ACP4736
Smittia sp. 8ES|BIOUG16480-F05|Kate Perez|BIN Taxonomy Match|BOLD:ACP4736
Smittia sp. 8ES|BIOUG16630-H10|Kate Perez|BIN Taxonomy Match|BOLD:ACP4736
Smittia sp. 8ES|BIOUG16708-C11|Kate Perez|BIN Taxonomy Match|BOLD:ACP4736
Smittia|BIOUG16712-G04|Kate Perez|Tree based identification
Smittia sp. 8ES|BIOUG15648-F10|Kate Perez|BIN Taxonomy Match|BOLD:ACP4736
Smittia|BIOUG36761-E09|Kate Perez|BIN Taxonomy Match (Jul 2018)|BOLD:ACP4736
Smittia sp. 8ES|CH-OSF85|Elisabeth Stur||BOLD:ACP4736
Smittia sp. 8ES|BIOUG15792-E03|Kate Perez|BIN Taxonomy Match|BOLD:ACP4736
Smittia sp. 8ES|BIOUG16332-F02|Kate Perez|BIN Taxonomy Match|BOLD:ACP4736
Smittia sp. 8ES|BIOUG15465-E06|Kate Perez|BIN Taxonomy Match|BOLD:ACP4736
Smittia sp. 8ES|BIOUG04795-B09|Kate Perez|BIN Taxonomy Match|BOLD:ACP4736
Smittia sp. 8ES|BIOUG15949-C07|Kate Perez|BIN Taxonomy Match|BOLD:ACP4736
Smittia sp. 8ES|BIOUG15763-F05|Kate Perez|BIN Taxonomy Match|BOLD:ACP4736
Parakiefferiella|ZSM34341-H03|Sofia Wiedenbrug||BOLD:ACU4227
Parakiefferiella|CCDB24228-H05|Sofia Wiedenbrug|BIN Taxonomy Match|BOLD:ACU4227
Smittia|BIOUG15793-E06|Kate Perez|BIN Taxonomy Match|BOLD:AAM7066
Smittia|BIOUG15806-B08|Kate Perez|BIN Taxonomy Match|BOLD:AAM7066
Smittia|BIOUG15718-H10|Kate Perez|BIN Taxonomy Match|BOLD:AAM7066
Smittia|BIOUG15456-E04|Kate Perez|BIN Taxonomy Match|BOLD:AAM7066
Smittia|BIOUG15463-H02|Kate Perez|BIN Taxonomy Match|BOLD:AAM7066
Smittia|BIOUG15654-B01|Kate Perez|BIN Taxonomy Match|BOLD:AAM7066
Smittia|BIOUG15457-B07|Kate Perez|BIN Taxonomy Match|BOLD:AAM7066
Smittia|BIOUG15648-E09|Kate Perez|BIN Taxonomy Match|BOLD:AAM7066
Smittia|BIOUG16476-D03|Kate Perez|BIN Taxonomy Match|BOLD:AAM7066
Smittia|BIOUG16435-C06|Kate Perez|BIN Taxonomy Match|BOLD:AAM7066
Smittia|BIOUG15537-A08|Kate Perez|BIN Taxonomy Match|BOLD:AAM7066
Smittia|BIOUG16436-F09|Kate Perez|BIN Taxonomy Match|BOLD:AAM7066
Smittia|BIOUG16435-C07|Kate Perez|BIN Taxonomy Match|BOLD:AAM7066
Smittia sp. 19ES|Finnmark08|Elisabeth Stur||BOLD:AAM7066
Smittia sp. 19ES|CH-eik92|Elisabeth Stur||BOLD:AAM7066
Smittia|BIOUG16551-F07|Kate Perez|BIN Taxonomy Match|BOLD:AAM7066
Smittia|BIOUG16476-B02|Kate Perez|BIN Taxonomy Match|BOLD:AAM7066
Smittia|BIOUG16479-A10|Kate Perez|BIN Taxonomy Match|BOLD:AAM7066
Smittia|BIOUG15562-H02|Kate Perez|BIN Taxonomy Match|BOLD:AAM7066
Smittia|BIOUG15542-B12|Kate Perez|BIN Taxonomy Match|BOLD:AAM7066
Smittia|BIOUG15948-E05|Kate Perez|BIN Taxonomy Match|BOLD:AAM7066
Smittia|BIOUG15459-B06|Kate Perez|BIN Taxonomy Match|BOLD:AAM7066
Smittia|BIOUG15543-G10|Kate Perez|BIN Taxonomy Match|BOLD:AAM7066
Smittia|BIOUG15806-G04|Kate Perez|BIN Taxonomy Match|BOLD:AAM7066
Smittia|BIOUG16432-B07|Kate Perez|BIN Taxonomy Match|BOLD:AAM7066
Smittia|BIOUG15565-E07|Kate Perez|BIN Taxonomy Match|BOLD:AAM7066
Smittia|BIOUG15454-D08|Kate Perez|BIN Taxonomy Match|BOLD:AAM7066
Smittia|BIOUG15183-A03|Kate Perez|BIN Taxonomy Match|BOLD:AAM7066
Smittia|BIOUG16476-B03|Kate Perez|BIN Taxonomy Match|BOLD:AAM7066
Smittia|BIOUG16597-A04|Kate Perez|BIN Taxonomy Match|BOLD:AAM7066
Smittia|BIOUG15562-E01|Kate Perez|BIN Taxonomy Match|BOLD:AAM7066
Smittia|BIOUG15540-F01|Kate Perez|BIN Taxonomy Match|BOLD:AAM7066
Smittia|BIOUG15541-D01|Kate Perez|BIN Taxonomy Match|BOLD:AAM7066
Smittia|BIOUG16434-E11|Kate Perez|BIN Taxonomy Match|BOLD:AAM7066
Smittia|BIOUG15951-G05|Kate Perez|BIN Taxonomy Match|BOLD:AAM7066
Smittia|BIOUG15795-G02|Kate Perez|BIN Taxonomy Match|BOLD:AAM7066
Smittia|BIOUG15561-D10|Kate Perez|BIN Taxonomy Match|BOLD:AAM7066
Smittia|BIOUG15965-C01|Kate Perez|BIN Taxonomy Match|BOLD:AAM7066
Smittia pratorum|ZMUO.024592|Lauri Paasivirta||BOLD:AAM7066
Smittia|BIOUG15763-C11|Kate Perez|BIN Taxonomy Match|BOLD:AAM7066
Metriocnemus sp. 9ES|TRD-CH255|Elisabeth Stur|morphology|BOLD:ACM4800
Metriocnemus sp. 9ES|TRD-CH246|Elisabeth Stur|morphology|BOLD:ACM4800
Metriocnemus sp. 9ES|TRD-CH435|Elisabeth Stur|morphology|BOLD:ACM4800
Metriocnemus sp. 9ES|TRD-CH53|Elisabeth Stur|morphology|BOLD:ACM4800
Cricotopus similis|BIOUG15949-E01|Elisabeth Stur|BIN Taxonomy Match|BOLD:ACP9613
Cricotopus similis|BIOUG16997-D11|Mikko Pentinsaari|BIN Taxonomy Match (Jan 2022)|BOLD:A...
Cricotopus similis|BIOUG17099-H11|Mikko Pentinsaari|BIN Taxonomy Match (Jan 2022)|BOLD:A...
Cricotopus similis|BIOUG16625-H04|Elisabeth Stur|BIN Taxonomy Match|BOLD:ACP9613
Cricotopus similis|BIOUG16476-B11|Elisabeth Stur|BIN Taxonomy Match|BOLD:ACP9613
Cricotopus similis|BIOUG16198-A01|Elisabeth Stur|BIN Taxonomy Match|BOLD:ACP9613
[illegible]

Cricotopus similis|BIOUG16625-H04|Elisabeth Stur|BIN Taxonomy Match|BOLD:ACP9613
Cricotopus similis|BIOUG16476-B11|Elisabeth Stur|BIN Taxonomy Match|BOLD:ACP9613
Cricotopus similis|BIOUG16198-A01|Elisabeth Stur|BIN Taxonomy Match|BOLD:ACP9613
Cricotopus similis|BIOUG15905-E06|Elisabeth Stur|BIN Taxonomy Match|BOLD:ACP9613
Cricotopus similis|BIOUG16436-D12|Elisabeth Stur|BIN Taxonomy Match|BOLD:ACP9613
Cricotopus similis|BIOUG15953-D02|Elisabeth Stur|BIN Taxonomy Match|BOLD:ACP9613
Cricotopus similis|BIOUG15967-E07|Elisabeth Stur|BIN Taxonomy Match|BOLD:ACP9613
Cricotopus similis|BIOUG16597-E10|Elisabeth Stur|BIN Taxonomy Match|BOLD:ACP9613
Cricotopus similis|BIOUG15953-C03|Elisabeth Stur|BIN Taxonomy Match|BOLD:ACP9613
Cricotopus similis|BIOUG15951-D10|Elisabeth Stur|BIN Taxonomy Match|BOLD:ACP9613
Cricotopus similis|BIOUG15950-A03|Elisabeth Stur|BIN Taxonomy Match|BOLD:ACP9613
Cricotopus similis|ZMUO.025338|Lauri Paasivirta||BOLD:ACP9613
Cricotopus similis|ZMUO.025337|Lauri Paasivirta||BOLD:ACP9613
Cricotopus similis|BIOUG16626-C06|Elisabeth Stur|BIN Taxonomy Match|BOLD:ACP9613
Cricotopus similis|BIOUG15948-C03|Elisabeth Stur|BIN Taxonomy Match|BOLD:ACP9613
Cricotopus similis|BIOUG16431-H08|Elisabeth Stur|BIN Taxonomy Match|BOLD:ACP9613
Cricotopus similis|BIOUG16207-F10|Elisabeth Stur|BIN Taxonomy Match|BOLD:ACP9613
Cricotopus similis|BIOUG16436-F05|Elisabeth Stur|BIN Taxonomy Match|BOLD:ACP9613
Cricotopus similis|BIOUG15966-B12|Elisabeth Stur|BIN Taxonomy Match|BOLD:ACP9613
Cricotopus similis|BIOUG15948-H10|Elisabeth Stur|BIN Taxonomy Match|BOLD:ACP9613
Cricotopus similis|BIOUG16476-H03|Elisabeth Stur|BIN Taxonomy Match|BOLD:ACP9613
Cricotopus similis|BIOUG15963-H03|Elisabeth Stur|BIN Taxonomy Match|BOLD:ACP9613
Cricotopus similis|BIOUG15948-F06|Elisabeth Stur|BIN Taxonomy Match|BOLD:ACP9613
Cricotopus similis|BIOUG16477-E11|Elisabeth Stur|BIN Taxonomy Match|BOLD:ACP9613
Cricotopus similis|BIOUG16713-D12|Elisabeth Stur|BIN Taxonomy Match|BOLD:ACP9613
Cricotopus similis|BIOUG15947-E07|Elisabeth Stur|BIN Taxonomy Match|BOLD:ACP9613
Cricotopus similis|BIOUG16626-H06|Elisabeth Stur|BIN Taxonomy Match|BOLD:ACP9613
Cricotopus similis|BIOUG16329-F12|Elisabeth Stur|BIN Taxonomy Match|BOLD:ACP9613
Cricotopus similis|BIOUG15951-A05|Elisabeth Stur|BIN Taxonomy Match|BOLD:ACP9613
Cricotopus similis|BIOUG16476-E02|Elisabeth Stur|BIN Taxonomy Match|BOLD:ACP9613
Cricotopus similis|BIOUG15905-H10|Elisabeth Stur|BIN Taxonomy Match|BOLD:ACP9613
Cricotopus similis|BIOUG15954-A04|Elisabeth Stur|BIN Taxonomy Match|BOLD:ACP9613
Cricotopus similis|BIOUG15947-A11|Elisabeth Stur|BIN Taxonomy Match|BOLD:ACP9613
Cricotopus similis|BIOUG15947-B05|Elisabeth Stur|BIN Taxonomy Match|BOLD:ACP9613
Cricotopus similis|BIOUG15948-D06|Elisabeth Stur|BIN Taxonomy Match|BOLD:ACP9613
Cricotopus similis|BIOUG15949-C03|Elisabeth Stur|BIN Taxonomy Match|BOLD:ACP9613
Cricotopus similis|BIOUG15964-B03|Elisabeth Stur|BIN Taxonomy Match|BOLD:ACP9613
Cricotopus similis|BIOUG15964-F05|Elisabeth Stur|BIN Taxonomy Match|BOLD:ACP9613
Cricotopus similis|BIOUG15964-F08|Elisabeth Stur|BIN Taxonomy Match|BOLD:ACP9613
Cricotopus similis|BIOUG16258-E10|Elisabeth Stur|BIN Taxonomy Match|BOLD:ACP9613
Cricotopus similis|BIOUG15905-F08|Elisabeth Stur|BIN Taxonomy Match|BOLD:ACP9613
Cricotopus similis|BIOUG16435-B05|Elisabeth Stur|BIN Taxonomy Match|BOLD:ACP9613
Cricotopus similis|BIOUG15953-F06|Elisabeth Stur|BIN Taxonomy Match|BOLD:ACP9613
Cricotopus similis|BIOUG15948-C06|Elisabeth Stur|BIN Taxonomy Match|BOLD:ACP9613
Cricotopus similis|BIOUG15949-C09|Elisabeth Stur|BIN Taxonomy Match|BOLD:ACP9613
Cricotopus similis|BIOUG15964-G06|Elisabeth Stur|BIN Taxonomy Match|BOLD:ACP9613
Cricotopus similis|BIOUG16259-E11|Elisabeth Stur|BIN Taxonomy Match|BOLD:ACP9613
Cricotopus similis|BIOUG15964-C01|Elisabeth Stur|BIN Taxonomy Match|BOLD:ACP9613
Cricotopus similis|BIOUG15966-H04|Elisabeth Stur|BIN Taxonomy Match|BOLD:ACP9613
Cricotopus similis|BIOUG15954-A08|Elisabeth Stur|BIN Taxonomy Match|BOLD:ACP9613
Cricotopus similis|BIOUG16625-F03|Elisabeth Stur|BIN Taxonomy Match|BOLD:ACP9613
Cricotopus similis|BIOUG15964-B11|Elisabeth Stur|BIN Taxonomy Match|BOLD:ACP9613
Cricotopus similis|BIOUG16476-H06|Elisabeth Stur|BIN Taxonomy Match|BOLD:ACP9613
Cricotopus similis|BIOUG15952-H08|Elisabeth Stur|BIN Taxonomy Match|BOLD:ACP9613
Cricotopus similis|BIOUG16205-A05|Elisabeth Stur|BIN Taxonomy Match|BOLD:ACP9613
Cricotopus similis|BIOUG16205-E05|Elisabeth Stur|BIN Taxonomy Match|BOLD:ACP9613
Cricotopus similis|BIOUG15950-D07|Elisabeth Stur|BIN Taxonomy Match|BOLD:ACP9613
Cricotopus similis|BIOUG16434-D04|Elisabeth Stur|BIN Taxonomy Match|BOLD:ACP9613
Cricotopus similis|BIOUG15964-F07|Elisabeth Stur|BIN Taxonomy Match|BOLD:ACP9613
Cricotopus similis|BIOUG15966-A06|Elisabeth Stur|BIN Taxonomy Match|BOLD:ACP9613
Cricotopus similis|BIOUG15798-B02|Elisabeth Stur|Morphology|BOLD:ACP9613
Cricotopus similis|BIOUG16434-C09|Elisabeth Stur|BIN Taxonomy Match|BOLD:ACP9613
Cricotopus similis|BIOUG16622-H11|Elisabeth Stur|BIN Taxonomy Match|BOLD:ACP9613
Cricotopus similis|BIOUG16625-A11|Elisabeth Stur|BIN Taxonomy Match|BOLD:ACP9613
Cricotopus similis|BIOUG16430-F02|Elisabeth Stur|BIN Taxonomy Match|BOLD:ACP9613
Cricotopus similis|BIOUG16431-A06|Elisabeth Stur|BIN Taxonomy Match|BOLD:ACP9613
Cricotopus similis|BIOUG16433-D05|Elisabeth Stur|BIN Taxonomy Match|BOLD:ACP9613
Cricotopus similis|BIOUG16477-A05|Elisabeth Stur|BIN Taxonomy Match|BOLD:ACP9613
Cricotopus similis|BIOUG16626-B03|Elisabeth Stur|BIN Taxonomy Match|BOLD:ACP9613
Cricotopus similis|BIOUG16626-G03|Elisabeth Stur|BIN Taxonomy Match|BOLD:ACP9613
Cricotopus similis|BIOUG16626-H05|Elisabeth Stur|BIN Taxonomy Match|BOLD:ACP9613
Cricotopus similis|BIOUG16625-E08|Elisabeth Stur|BIN Taxonomy Match|BOLD:ACP9613
Cricotopus similis|BIOUG15952-E05|Elisabeth Stur|BIN Taxonomy Match|BOLD:ACP9613
Cricotopus similis|BIOUG16552-E01|Elisabeth Stur|BIN Taxonomy Match|BOLD:ACP9613
Cricotopus similis|BIOUG16205-C02|Elisabeth Stur|BIN Taxonomy Match|BOLD:ACP9613
Cricotopus similis|BIOUG16296-F10|Elisabeth Stur|BIN Taxonomy Match|BOLD:ACP9613
Cricotopus similis|BIOUG16435-F03|Elisabeth Stur|BIN Taxonomy Match|BOLD:ACP9613
Cricotopus similis|BIOUG16625-G01|Elisabeth Stur|BIN Taxonomy Match|BOLD:ACP9613
Cricotopus similis|BIOUG16626-B07|Elisabeth Stur|BIN Taxonomy Match|BOLD:ACP9613
Cricotopus similis|BIOUG16626-H03|Elisabeth Stur|BIN Taxonomy Match|BOLD:ACP9613
Cricotopus similis|BIOUG16434-F11|Elisabeth Stur|BIN Taxonomy Match|BOLD:ACP9613
Cricotopus similis|BIOUG15966-C02|Elisabeth Stur|BIN Taxonomy Match|BOLD:ACP9613
Cricotopus similis|BIOUG16205-B01|Elisabeth Stur|BIN Taxonomy Match|BOLD:ACP9613
Cricotopus similis|BIOUG15954-D07|Elisabeth Stur|BIN Taxonomy Match|BOLD:ACP9613
Cricotopus similis|BIOUG16433-F03|Elisabeth Stur|BIN Taxonomy Match|BOLD:ACP9613
Cricotopus similis|BIOUG15963-E10|Elisabeth Stur|BIN Taxonomy Match|BOLD:ACP9613
Cricotopus similis|BIOUG16598-A01|Elisabeth Stur|BIN Taxonomy Match|BOLD:ACP9613
Cricotopus similis|BIOUG16478-C02|Elisabeth Stur|BIN Taxonomy Match|BOLD:ACP9613
Cricotopus similis|BIOUG16205-B08|Elisabeth Stur|BIN Taxonomy Match|BOLD:ACP9613
Cricotopus similis|BIOUG16205-A04|Elisabeth Stur|BIN Taxonomy Match|BOLD:ACP9613
Cricotopus similis|BIOUG15963-G12|Elisabeth Stur|BIN Taxonomy Match|BOLD:ACP9613
Cricotopus similis|BIOUG15963-B07|Elisabeth Stur|BIN Taxonomy Match|BOLD:ACP9613
Cricotopus similis|BIOUG15963-B02|Elisabeth Stur|BIN Taxonomy Match|BOLD:ACP9613
Cricotopus similis|BIOUG15954-A03|Elisabeth Stur|BIN Taxonomy Match|BOLD:ACP9613
Cricotopus similis|BIOUG15951-B11|Elisabeth Stur|BIN Taxonomy Match|BOLD:ACP9613
Cricotopus similis|BIOUG15950-H03|Elisabeth Stur|BIN Taxonomy Match|BOLD:ACP9613
Cricotopus similis|BIOUG15950-A05|Elisabeth Stur|BIN Taxonomy Match|BOLD:ACP9613
Cricotopus similis|BIOUG15949-E10|Elisabeth Stur|BIN Taxonomy Match|BOLD:ACP9613
Cricotopus similis|BIOUG15967-C03|Elisabeth Stur|BIN Taxonomy Match|BOLD:ACP9613
Cricotopus similis|BIOUG15967-C02|Elisabeth Stur|BIN Taxonomy Match|BOLD:ACP9613
Cricotopus similis|BIOUG15947-H01|Elisabeth Stur|BIN Taxonomy Match|BOLD:ACP9613
Cricotopus similis|BIOUG15947-C11|Elisabeth Stur|BIN Taxonomy Match|BOLD:ACP9613
Cricotopus similis|BIOUG15965-G10|Elisabeth Stur|BIN Taxonomy Match|BOLD:ACP9613

Cricotopus similis|BIOUG15947-H01|Elisabeth Stur|BIN Taxonomy Match|BOLD:ACP9613
Cricotopus similis|BIOUG15947-C11|Elisabeth Stur|BIN Taxonomy Match|BOLD:ACP9613
Cricotopus similis|BIOUG15965-G10|Elisabeth Stur|BIN Taxonomy Match|BOLD:ACP9613
Cricotopus similis|BIOUG15967-C09|Elisabeth Stur|BIN Taxonomy Match|BOLD:ACP9613
Cricotopus similis|BIOUG16261-E09|Elisabeth Stur|BIN Taxonomy Match|BOLD:ACP9613
Cricotopus similis|BIOUG15966-B02|Elisabeth Stur|BIN Taxonomy Match|BOLD:ACP9613
Cricotopus similis|BIOUG16261-F05|Elisabeth Stur|BIN Taxonomy Match|BOLD:ACP9613
Cricotopus similis|BIOUG15905-G06|Elisabeth Stur|BIN Taxonomy Match|BOLD:ACP9613
Cricotopus similis|BIOUG15947-A08|Elisabeth Stur|BIN Taxonomy Match|BOLD:ACP9613
Cricotopus similis|BIOUG15954-B11|Elisabeth Stur|BIN Taxonomy Match|BOLD:ACP9613
Cricotopus similis|BIOUG16479-C02|Elisabeth Stur|BIN Taxonomy Match|BOLD:ACP9613
Cricotopus similis|BIOUG16430-G03|Elisabeth Stur|BIN Taxonomy Match|BOLD:ACP9613
Cricotopus similis|BIOUG16432-E02|Elisabeth Stur|BIN Taxonomy Match|BOLD:ACP9613
Cricotopus similis|BIOUG16625-G09|Elisabeth Stur|BIN Taxonomy Match|BOLD:ACP9613
Cricotopus similis|BIOUG15905-D12|Elisabeth Stur|BIN Taxonomy Match|BOLD:ACP9613
Cricotopus similis|BIOUG16625-H06|Elisabeth Stur|BIN Taxonomy Match|BOLD:ACP9613
Cricotopus similis|BIOUG16626-G09|Elisabeth Stur|BIN Taxonomy Match|BOLD:ACP9613
Cricotopus similis|BIOUG15964-E02|Elisabeth Stur|BIN Taxonomy Match|BOLD:ACP9613
Cricotopus similis|BIOUG15905-D08|Elisabeth Stur|BIN Taxonomy Match|BOLD:ACP9613
Cricotopus similis|BIOUG16198-G09|Elisabeth Stur|BIN Taxonomy Match|BOLD:ACP9613
Cricotopus similis|BIOUG16551-H06|Elisabeth Stur|BIN Taxonomy Match|BOLD:ACP9613
Cricotopus similis|BIOUG15953-A10|Elisabeth Stur|BIN Taxonomy Match|BOLD:ACP9613
Cricotopus similis|BIOUG15967-H05|Elisabeth Stur|BIN Taxonomy Match|BOLD:ACP9613
Cricotopus similis|BIOUG16260-G11|Elisabeth Stur|BIN Taxonomy Match|BOLD:ACP9613
Cricotopus similis|BIOUG16625-A07|Elisabeth Stur|BIN Taxonomy Match|BOLD:ACP9613
Cricotopus similis|BIOUG16626-H09|Elisabeth Stur|BIN Taxonomy Match|BOLD:ACP9613
Cricotopus similis|BIOUG15947-B08|Elisabeth Stur|BIN Taxonomy Match|BOLD:ACP9613
Cricotopus similis|BIOUG16596-H01|Elisabeth Stur|BIN Taxonomy Match|BOLD:ACP9613
Cricotopus similis|BIOUG16297-A05|Elisabeth Stur|BIN Taxonomy Match|BOLD:ACP9613
Cricotopus similis|BIOUG16622-H06|Elisabeth Stur|BIN Taxonomy Match|BOLD:ACP9613
Cricotopus similis|BIOUG16430-H11|Elisabeth Stur|BIN Taxonomy Match|BOLD:ACP9613
Cricotopus similis|BIOUG15951-F04|Elisabeth Stur|BIN Taxonomy Match|BOLD:ACP9613
Cricotopus similis|BIOUG15952-F05|Elisabeth Stur|BIN Taxonomy Match|BOLD:ACP9613
Cricotopus similis|BIOUG15953-F02|Elisabeth Stur|BIN Taxonomy Match|BOLD:ACP9613
Cricotopus similis|PK-190-132|Elisabeth Stur|morphology|BOLD:ACP9613
Cricotopus similis|BIOUG15951-E03|Elisabeth Stur|BIN Taxonomy Match|BOLD:ACP9613
Cricotopus similis|BIOUG15953-A03|Elisabeth Stur|BIN Taxonomy Match|BOLD:ACP9613
Cricotopus similis|BIOUG15951-E10|Elisabeth Stur|BIN Taxonomy Match|BOLD:ACP9613
Cricotopus similis|BIOUG16198-A08|Elisabeth Stur|BIN Taxonomy Match|BOLD:ACP9613
Cricotopus similis|BIOUG16198-B02|Elisabeth Stur|BIN Taxonomy Match|BOLD:ACP9613
Cricotopus similis|BIOUG16205-B10|Elisabeth Stur|BIN Taxonomy Match|BOLD:ACP9613
Cricotopus similis|BIOUG16596-G11|Elisabeth Stur|BIN Taxonomy Match|BOLD:ACP9613
Cricotopus similis|BIOUG16597-B12|Elisabeth Stur|BIN Taxonomy Match|BOLD:ACP9613
Cricotopus similis|BIOUG15954-G10|Elisabeth Stur|BIN Taxonomy Match|BOLD:ACP9613
Cricotopus similis|BIOUG17024-G01|Elisabeth Stur|BIN Taxonomy Match|BOLD:ACP9613
Cricotopus similis|BIOUG15953-F10|Elisabeth Stur|BIN Taxonomy Match|BOLD:ACP9613
Cricotopus similis|BIOUG16433-G10|Elisabeth Stur|BIN Taxonomy Match|BOLD:ACP9613
Cricotopus similis|BIOUG15949-D03|Elisabeth Stur|BIN Taxonomy Match|BOLD:ACP9613
Cricotopus similis|BIOUG16626-F02|Elisabeth Stur|BIN Taxonomy Match|BOLD:ACP9613
Cricotopus similis|BIOUG15951-G12|Elisabeth Stur|BIN Taxonomy Match|BOLD:ACP9613
Cricotopus similis|BIOUG15965-C09|Elisabeth Stur|BIN Taxonomy Match|BOLD:ACP9613
Cricotopus similis|BIOUG16198-F06|Elisabeth Stur|BIN Taxonomy Match|BOLD:ACP9613
Cricotopus similis|BIOUG15963-D03|Elisabeth Stur|BIN Taxonomy Match|BOLD:ACP9613
Cricotopus similis|BIOUG15950-H01|Elisabeth Stur|BIN Taxonomy Match|BOLD:ACP9613
Cricotopus similis|BIOUG15950-G10|Elisabeth Stur|BIN Taxonomy Match|BOLD:ACP9613
Cricotopus similis|BIOUG15950-F12|Elisabeth Stur|BIN Taxonomy Match|BOLD:ACP9613
Cricotopus similis|BIOUG15950-A10|Elisabeth Stur|BIN Taxonomy Match|BOLD:ACP9613
Cricotopus similis|BIOUG15900-H07|Elisabeth Stur|BIN Taxonomy Match|BOLD:ACP9613
Cricotopus similis|BIOUG15951-E11|Elisabeth Stur|BIN Taxonomy Match|BOLD:ACP9613
Cricotopus similis|BIOUG16478-C09|Elisabeth Stur|BIN Taxonomy Match|BOLD:ACP9613
Cricotopus similis|BIOUG16476-D11|Elisabeth Stur|BIN Taxonomy Match|BOLD:ACP9613
Cricotopus similis|BIOUG16477-F09|Elisabeth Stur|BIN Taxonomy Match|BOLD:ACP9613
Cricotopus similis|BIOUG16477-H09|Elisabeth Stur|BIN Taxonomy Match|BOLD:ACP9613
Cricotopus similis|BIOUG15952-G01|Elisabeth Stur|BIN Taxonomy Match|BOLD:ACP9613
Cricotopus similis|BIOUG16434-F04|Elisabeth Stur|BIN Taxonomy Match|BOLD:ACP9613
Cricotopus similis|BIOUG15952-D05|Elisabeth Stur|BIN Taxonomy Match|BOLD:ACP9613
Cricotopus similis|BIOUG16435-D04|Elisabeth Stur|BIN Taxonomy Match|BOLD:ACP9613
Cricotopus similis|BIOUG15952-H06|Elisabeth Stur|BIN Taxonomy Match|BOLD:ACP9613
Cricotopus similis|BIOUG16596-B09|Elisabeth Stur|BIN Taxonomy Match|BOLD:ACP9613
Cricotopus similis|BIOUG15953-H09|Elisabeth Stur|BIN Taxonomy Match|BOLD:ACP9613
Cricotopus similis|BIOUG15963-G01|Elisabeth Stur|BIN Taxonomy Match|BOLD:ACP9613
Cricotopus similis|BIOUG16258-F03|Elisabeth Stur|BIN Taxonomy Match|BOLD:ACP9613
Cricotopus similis|BIOUG16478-H02|Elisabeth Stur|BIN Taxonomy Match|BOLD:ACP9613
Cricotopus similis|BIOUG15950-H09|Elisabeth Stur|BIN Taxonomy Match|BOLD:ACP9613
Cricotopus similis|BIOUG15950-G11|Elisabeth Stur|BIN Taxonomy Match|BOLD:ACP9613
Cricotopus similis|BIOUG16205-D05|Elisabeth Stur|BIN Taxonomy Match|BOLD:ACP9613
Cricotopus similis|BIOUG16435-E03|Elisabeth Stur|BIN Taxonomy Match|BOLD:ACP9613
Cricotopus similis|BIOUG15953-G03|Elisabeth Stur|BIN Taxonomy Match|BOLD:ACP9613
Cricotopus similis|BIOUG16478-A10|Elisabeth Stur|BIN Taxonomy Match|BOLD:ACP9613
Cricotopus similis|BIOUG15952-H09|Elisabeth Stur|BIN Taxonomy Match|BOLD:ACP9613
Cricotopus similis|BIOUG16433-C12|Elisabeth Stur|BIN Taxonomy Match|BOLD:ACP9613
Cricotopus similis|BIOUG15953-F03|Elisabeth Stur|BIN Taxonomy Match|BOLD:ACP9613
Cricotopus similis|BIOUG16436-G04|Elisabeth Stur|BIN Taxonomy Match|BOLD:ACP9613
Cricotopus similis|BIOUG15951-B12|Elisabeth Stur|BIN Taxonomy Match|BOLD:ACP9613
Cricotopus similis|BIOUG16479-E02|Elisabeth Stur|BIN Taxonomy Match|BOLD:ACP9613
Cricotopus similis|BIOUG16430-C08|Elisabeth Stur|BIN Taxonomy Match|BOLD:ACP9613
Cricotopus similis|BIOUG16480-F03|Elisabeth Stur|BIN Taxonomy Match|BOLD:ACP9613
Cricotopus similis|BIOUG16432-C06|Elisabeth Stur|BIN Taxonomy Match|BOLD:ACP9613
Cricotopus similis|BIOUG16436-B04|Elisabeth Stur|BIN Taxonomy Match|BOLD:ACP9613
Cricotopus similis|BIOUG16480-A07|Elisabeth Stur|BIN Taxonomy Match|BOLD:ACP9613
Cricotopus similis|BIOUG15952-B05|Elisabeth Stur|BIN Taxonomy Match|BOLD:ACP9613
Cricotopus similis|BIOUG15952-D03|Elisabeth Stur|BIN Taxonomy Match|BOLD:ACP9613
Cricotopus similis|BIOUG15952-E11|Elisabeth Stur|BIN Taxonomy Match|BOLD:ACP9613
Cricotopus similis|BIOUG16436-F02|Elisabeth Stur|BIN Taxonomy Match|BOLD:ACP9613
Cricotopus similis|BIOUG15950-F09|Elisabeth Stur|BIN Taxonomy Match|BOLD:ACP9613
Cricotopus similis|BIOUG15900-H05|Elisabeth Stur|BIN Taxonomy Match|BOLD:ACP9613
Cricotopus similis|BIOUG15953-C02|Elisabeth Stur|BIN Taxonomy Match|BOLD:ACP9613
Cricotopus similis|BIOUG15900-G05|Elisabeth Stur|BIN Taxonomy Match|BOLD:ACP9613
Cricotopus similis|BIOUG15963-F02|Elisabeth Stur|BIN Taxonomy Match|BOLD:ACP9613
Cricotopus similis|BIOUG15963-C11|Elisabeth Stur|BIN Taxonomy Match|BOLD:ACP9613
Cricotopus similis|BIOUG16434-C04|Elisabeth Stur|BIN Taxonomy Match|BOLD:ACP9613
Cricotopus similis|BIOUG16594-F04|Elisabeth Stur|BIN Taxonomy Match|BOLD:ACP9613
Cricotopus similis|BIOUG15967-E02|Elisabeth Stur|BIN Taxonomy Match|BOLD:ACP9613

Cricotopus similis|BIOUG16434-C04|Elisabeth Stur|BIN Taxonomy Match|BOLD:ACP9613
Cricotopus similis|BIOUG16594-F04|Elisabeth Stur|BIN Taxonomy Match|BOLD:ACP9613
Cricotopus similis|BIOUG15967-E02|Elisabeth Stur|BIN Taxonomy Match|BOLD:ACP9613
Cricotopus similis|BIOUG15967-D09|Elisabeth Stur|BIN Taxonomy Match|BOLD:ACP9613
Cricotopus similis|BIOUG15965-H05|Elisabeth Stur|BIN Taxonomy Match|BOLD:ACP9613
Cricotopus similis|BIOUG15965-F10|Elisabeth Stur|BIN Taxonomy Match|BOLD:ACP9613
Cricotopus similis|BIOUG15964-F06|Elisabeth Stur|BIN Taxonomy Match|BOLD:ACP9613
Cricotopus similis|BIOUG16552-H11|Elisabeth Stur|BIN Taxonomy Match|BOLD:ACP9613
Cricotopus similis|BIOUG15905-F10|Elisabeth Stur|BIN Taxonomy Match|BOLD:ACP9613
Cricotopus similis|BIOUG16299-D02|Elisabeth Stur|BIN Taxonomy Match|BOLD:ACP9613
Cricotopus similis|BIOUG16257-B10|Elisabeth Stur|BIN Taxonomy Match|BOLD:ACP9613
Cricotopus similis|BIOUG16551-H04|Elisabeth Stur|BIN Taxonomy Match|BOLD:ACP9613
Cricotopus similis|BIOUG16198-H08|Elisabeth Stur|BIN Taxonomy Match|BOLD:ACP9613
Cricotopus similis|BIOUG16198-D02|Elisabeth Stur|BIN Taxonomy Match|BOLD:ACP9613
Cricotopus similis|BIOUG15967-H06|Elisabeth Stur|BIN Taxonomy Match|BOLD:ACP9613
Cricotopus similis|BIOUG15967-F10|Elisabeth Stur|BIN Taxonomy Match|BOLD:ACP9613
Cricotopus similis|BIOUG15967-F06|Elisabeth Stur|BIN Taxonomy Match|BOLD:ACP9613
Cricotopus similis|BIOUG15967-F01|Elisabeth Stur|BIN Taxonomy Match|BOLD:ACP9613
Cricotopus similis|BIOUG15966-G05|Elisabeth Stur|BIN Taxonomy Match|BOLD:ACP9613
Cricotopus similis|BIOUG15966-D08|Elisabeth Stur|BIN Taxonomy Match|BOLD:ACP9613
Cricotopus similis|BIOUG15966-C03|Elisabeth Stur|BIN Taxonomy Match|BOLD:ACP9613
Cricotopus similis|BIOUG16331-C03|Elisabeth Stur|BIN Taxonomy Match|BOLD:ACP9613
Cricotopus similis|BIOUG15964-D05|Elisabeth Stur|BIN Taxonomy Match|BOLD:ACP9613
Cricotopus similis|BIOUG16396-D09|Elisabeth Stur|BIN Taxonomy Match|BOLD:ACP9613
Cricotopus similis|BIOUG15954-H03|Elisabeth Stur|BIN Taxonomy Match|BOLD:ACP9613
Cricotopus similis|BIOUG15954-A02|Elisabeth Stur|BIN Taxonomy Match|BOLD:ACP9613
Cricotopus similis|BIOUG15949-F12|Elisabeth Stur|BIN Taxonomy Match|BOLD:ACP9613
Cricotopus similis|BIOUG15949-F02|Elisabeth Stur|BIN Taxonomy Match|BOLD:ACP9613
Cricotopus similis|BIOUG15949-E12|Elisabeth Stur|BIN Taxonomy Match|BOLD:ACP9613
Cricotopus similis|BIOUG15967-C07|Elisabeth Stur|BIN Taxonomy Match|BOLD:ACP9613
Cricotopus similis|BIOUG15948-C10|Elisabeth Stur|BIN Taxonomy Match|BOLD:ACP9613
Cricotopus similis|BIOUG15948-A12|Elisabeth Stur|BIN Taxonomy Match|BOLD:ACP9613
Cricotopus similis|BIOUG15948-A11|Elisabeth Stur|BIN Taxonomy Match|BOLD:ACP9613
Cricotopus similis|BIOUG15947-F02|Elisabeth Stur|BIN Taxonomy Match|BOLD:ACP9613
Cricotopus similis|BIOUG15947-E03|Elisabeth Stur|BIN Taxonomy Match|BOLD:ACP9613
Cricotopus similis|BIOUG15947-D04|Elisabeth Stur|BIN Taxonomy Match|BOLD:ACP9613
Cricotopus similis|BIOUG15947-C09|Elisabeth Stur|BIN Taxonomy Match|BOLD:ACP9613
Cricotopus similis|BIOUG15947-B02|Elisabeth Stur|BIN Taxonomy Match|BOLD:ACP9613
Cricotopus similis|BIOUG15905-H05|Elisabeth Stur|BIN Taxonomy Match|BOLD:ACP9613
Cricotopus similis|BIOUG15905-E07|Elisabeth Stur|BIN Taxonomy Match|BOLD:ACP9613
Cricotopus similis|BIOUG15905-B06|Elisabeth Stur|BIN Taxonomy Match|BOLD:ACP9613
Cricotopus similis|BIOUG16330-D04|Elisabeth Stur|BIN Taxonomy Match|BOLD:ACP9613
Eurycnemus crassipes|ZMUO.026237|Lauri Paasivirta||BOLD:ADF0328
Eurycnemus crassipes|ZMUO.026236|Lauri Paasivirta||BOLD:ADF0328
Tanytarsus lestagei|KY225360
Brillia flavifrons|014421591|Jason Doe||BOLD:AAV0728
Brillia longifurca|ZMUO.024709|Lauri Paasivirta||BOLD:AAV0728
Brillia longifurca|ZMUO.024708|Lauri Paasivirta||BOLD:AAV0728
Brillia longifurca|TRD-CH368|Elisabeth Stur|morphology|BOLD:AAV0728
Brillia longifurca|CH-OSF99|Elisabeth Stur||BOLD:AAV0728
Brillia longifurca|ATNA565|Elisabeth Stur||BOLD:AAV0728
Brillia longifurca|CH-OSF15|Elisabeth Stur||BOLD:AAV0728
Brillia longifurca|Finnmark352|Elisabeth Stur||BOLD:AAV0728
Brillia bifida|CCDB21606-G11|Sofia Wiedenbrug||BOLD:AAD7726
Brillia bifida|CCDB21606-B12|Sofia Wiedenbrug||BOLD:AAD7726
Brillia bifida|CCDB21606-A06|Sofia Wiedenbrug||BOLD:AAD7726
Brillia bifida|BC-ZSM-DIP-22488-F06|Dieter Doczkal||BOLD:AAD7726
Brillia bifida|BC-ZSM-DIP-22488-C06|Dieter Doczkal||BOLD:AAD7726
Brillia bifida|CCDB24228-C08|Sofia Wiedenbrug|BIN Taxonomy Match|BOLD:AAD7726
Brillia bifida|ES186|Elisabeth Stur||BOLD:AAD7726
Brillia bifida|ES181|Elisabeth Stur||BOLD:AAD7725
Brillia bifida|ZMUO.024697|Lauri Paasivirta||BOLD:AAD7724
Brillia bifida|ZSM-DIP-33148-F07|Caroline Chimeno|BIN Taxonomy Match (Jan 2022)|BOLD:AAD...
Brillia bifida|ZSM-DIP-33128-F04|Caroline Chimeno|BIN Taxonomy Match (Jan 2022)|BOLD:AAD...
Brillia bifida|ZSM-DIP-33129-E10|Caroline Chimeno|BIN Taxonomy Match (Jan 2022)|BOLD:AAD...
Brillia bifida|ZSM-DIP-33145-H10|Caroline Chimeno|BIN Taxonomy Match (Jan 2022)|BOLD:AAD...
Brillia bifida|CH-OSF01|Elisabeth Stur||BOLD:AAD7724
Brillia bifida|ZMUO.024696|Lauri Paasivirta||BOLD:AAD7724
Brillia bifida|TRD-CH69|Elisabeth Stur||BOLD:AAD7724
Brillia bifida|ATNA310|Elisabeth Stur||BOLD:AAD7724
Brillia bifida|ATNA311|Elisabeth Stur||BOLD:AAD7724
Brillia bifida|BIOUG43948-E03|Kate Perez|BIN Taxonomy Match below Phylum (Jan 2019)|BOLD ...
Brillia bifida|TRD-CH297|Elisabeth Stur||BOLD:AAD7724
Brillia bifida|TRD-CH68|Elisabeth Stur||BOLD:AAD7724
Brillia bifida|ZSM-DIP-33129-E02|Caroline Chimeno|BIN Taxonomy Match (Jan 2022)|BOLD:AAD...
Brillia bifida|ZSM-DIP-33146-E06|Caroline Chimeno|BIN Taxonomy Match (Jan 2022)|BOLD:AAD...
Brillia bifida|ES92|Elisabeth Stur||BOLD:AAD7724
Brillia bifida|ES89|Elisabeth Stur||BOLD:AAD7724
Brillia bifida|Finnmark66|Elisabeth Stur||BOLD:AAD7724
Brillia bifida|Finnmark158|Elisabeth Stur||BOLD:AAD7724
Brillia bifida|Finnmark310|Elisabeth Stur||BOLD:AAD7724
Thienemanniella|CCDB24229-A01|Sofia Wiedenbrug|BIN Taxonomy Match|BOLD:ACU4481
Thienemanniella|CCDB21605-G11|Sofia Wiedenbrug||BOLD:ACT1182
Thienemanniella|ZSM-DIP-33172-E03|Caroline Chimeno|BIN Taxonomy Match (Jan 2022)|BOLD:AC...
Thienemanniella|CCDB21605-G10|Sofia Wiedenbrug||BOLD:ACT1182
Thienemanniella sp. 1TE|ATNA235|Torbjorn Ekrem||BOLD:AAG1616
Thienemanniella sp. 1TE|EBAI-Ch015|Elisabeth Stur|morphology|BOLD:AAG1616
Thienemanniella sp. 1TE|ATNA368|Torbjorn Ekrem||BOLD:AAG1616
Thienemanniella sp. 1TE|ATNA364|Torbjorn Ekrem||BOLD:AAG1616
Thienemanniella sp. 1TE|Finnmark848|Elisabeth Stur||BOLD:AAG1616
Thienemanniella sp. 1TE|ATNA407|Torbjorn Ekrem||BOLD:AAG1616
Thienemanniella sp. 1TE|ATNA176|Torbjorn Ekrem||BOLD:AAG1616
Thienemanniella sp. 4TE|EBAI-Ch154|Elisabeth Stur|morphology|BOLD:ACX6105
Thienemanniella sp. 4TE|EBAI-Ch158|Elisabeth Stur|morphology|BOLD:ACX6105
Thienemanniella sp. 4TE|EBAI-Ch159|Elisabeth Stur|morphology|BOLD:ACX6105
Thienemanniella sp. 4TE|EBAI-Ch020|Elisabeth Stur|morphology|BOLD:ACX6105
Thienemanniella sp. 4TE|ATNA545|Elisabeth Stur||BOLD:ACX6105
Thienemanniella sp. 4TE|EBAI-Ch257|Elisabeth Stur|morphology|BOLD:ACX6105
Thienemanniella sp. 4TE|EBAI-Ch160|Elisabeth Stur|morphology|BOLD:ACX6105
Thienemanniella sp. 4TE|EBAI-Ch156|Elisabeth Stur|morphology|BOLD:ACX6105
Thienemanniella sp. 4TE|EBAI-Ch155|Elisabeth Stur|morphology|BOLD:ACX6105
Thienemanniella sp. 4TE|EBAI-Ch153|Elisabeth Stur|morphology|BOLD:ACX6105
Thienemanniella|ZMUO.025844|Lauri Paasivirta||BOLD:ADF9731

Thienemanniella sp. 4TE|EBAI-Ch155|Elisabeth Stur|morphology|BOLD:ACX6105
Thienemanniella sp. 4TE|EBAI-Ch153|Elisabeth Stur|morphology|BOLD:ACX6105
Thienemanniella|ZMUO.025844|Lauri Paasivirta||BOLD:ADE9731
Thienemanniella cf. vittata|ES41|Torbjorn Ekrem||BOLD:AER1620
Thienemanniella cf. vittata|ES27|Torbjorn Ekrem||BOLD:AER1620
Thienemanniella cf. vittata|SOE240|Torbjorn Ekrem||BOLD:AAC9734
Thienemanniella|25132-D06|Kate Perez|BOLD ID Engine Manual (Apr 2018)|BOLD:AAC9734
Thienemanniella cf. vittata|SOE319|Elisabeth Stur||BOLD:AAC9734
Thienemanniella cf. vittata|SOE252|Torbjorn Ekrem||BOLD:AAC9734
Thienemanniella cf. vittata|SOE368|Elisabeth Stur||BOLD:AAC9734
Thienemanniella cf. vittata|SOE300|Torbjorn Ekrem||BOLD:AAC9734
Thienemanniella cf. vittata|SOE303|Torbjorn Ekrem||BOLD:AAC9734
Thienemanniella cf. vittata|SOE231|Torbjorn Ekrem||BOLD:AAC9734
Thienemanniella cf. vittata|SOE228|Torbjorn Ekrem||BOLD:AAC9734
Thienemanniella cf. vittata|SOE433|Elisabeth Stur||BOLD:AAC9734
Thienemanniella cf. vittata|SOE425|Elisabeth Stur
Abiskomyia paravirgo|ZMUO.026023|Lauri Paasivirta||BOLD:ADF2556
Abiskomyia paravirgo|ZMUO.025871|Lauri Paasivirta||BOLD:ADF2556
Abiskomyia paravirgo|ZMUO.025870|Lauri Paasivirta||BOLD:ADF2556
Corynoneura|ZMUO.025298|Lauri Paasivirta||BOLD:ADA3150
Corynoneura|ZMUO.025297|Lauri Paasivirta||BOLD:ADA3150
Thienemanniella acuticornis|ZMUO.024778|Lauri Paasivirta||BOLD:AEJ1387
Thienemanniella acuticornis|ZMUO.024777|Lauri Paasivirta||BOLD:ADA8644
Prodiamesa olivacea|KY225351
Heterotrissocladius marcidus|KY225362
Orthocladius consobrinus|KY225356
Cricotopus sp. CRICIPe2_4_1|KY225332|||BOLD:ADK8057
Corynoneura arctica|ATNA529|Elisabeth Stur||BOLD:ACX4287
Corynoneura sp. 1ES|TRD-CH421|Elisabeth Stur|morphology|BOLD:ABZ8189
Corynoneura arctica|NHRS-BYWS000000522|Yngve Brodin|morphology|BOLD:AAB0079
Corynoneura arctica|Finnmark154|Elisabeth Stur||BOLD:AAB0079
Corynoneura arctica|Finnmark630|Elisabeth Stur||BOLD:AAB0079
Corynoneura cf. arctica|Finnmark569|Elisabeth Stur|Morphology
Corynoneura gratias|NHRS-BYWS000001112|Yngve Brodin|morphology|BOLD:AAW4942
Corynoneura gratias|NHRS-BYWS000000542|Yngve Brodin|morphology|BOLD:AAW4942
Corynoneura gratias|TRD-CH211|Sofia Wiedenbrug||BOLD:AAW4942
Corynoneura gratias|ZMUO.025197|Lauri Paasivirta||BOLD:AAW4942
Corynoneura gratias|ZMUO.025196|Lauri Paasivirta||BOLD:AAW4942
Corynoneura arctica|EJV-20110238|Lauri Paasivirta||BOLD:AAW4942
Corynoneura arctica|EJV-20110237|Lauri Paasivirta||BOLD:AAW4942
Corynoneura arctica|EJV-20110236|Lauri Paasivirta||BOLD:AAW4942
Corynoneura sp. 19ES|TRD-CH353|Elisabeth Stur|morphology|BOLD:ACY5622
Corynoneura cf. scutellata|NHRS-BYWS000000275|Yngve Brodin|morphology|BOLD:AAJ5875
Corynoneura sp. 26ES|TRD-CH60|Elisabeth Stur|Morphology|BOLD:AAJ5875
Corynoneura cf. scutellata|NHRS-BYWS000000274|Yngve Brodin|morphology|BOLD:AAJ5875
Corynoneura sp. 26ES|Finnmark857|Elisabeth Stur|Morphology|BOLD:AAJ5875
Corynoneura sp. 26ES|SOE143|Elisabeth Stur|Morphology|BOLD:AAJ5875
Corynoneura sp. 26ES|Finnmark116|Elisabeth Stur|Morphology|BOLD:AAJ5875
Corynoneura edwardsi|TRD-CH420|Elisabeth Stur|morphology|BOLD:ACO4059
Corynoneura celeripes|ZMUO.024634|Lauri Paasivirta||BOLD:ACO4059
Corynoneura edwardsi|NHRS-BYWS000000377|Yngve Brodin|morphology|BOLD:ACO4059
Corynoneura edwardsi|NHRS-BYWS000000376|Yngve Brodin|morphology|BOLD:ACO4059
Corynoneura edwardsi|NHRS-BYWS000000194|Yngve Brodin|morphology|BOLD:ACO4059
Corynoneura edwardsi|ZMUO.024731|Lauri Paasivirta||BOLD:ACO4059
Corynoneura edwardsi|ZMUO.024730|Lauri Paasivirta||BOLD:ACO4059
Corynoneura edwardsi|NHRS-BYWS000000193|Yngve Brodin|morphology|BOLD:ACO4059
Corynoneura edwardsi|TRD-CH99|Elisabeth Stur||BOLD:ACO4059
Corynoneura carriana|NHRS-BYWS000000987|Yngve Brodin|morphology|BOLD:AAU2657
Corynoneura carriana|NHRS-BYWS000001868|Yngve Brodin||BOLD:AAU2657
Corynoneura carriana|ZMUO.025834|Lauri Paasivirta||BOLD:AAU2657
Corynoneura carriana|BIOUG36840-F11|Kate Perez|BIN Taxonomy Match (Jul 2018)|BOLD:AAU2657
Corynoneura carriana|NHRS-BYWS000000561|Yngve Brodin|morphology|BOLD:AAU2657
Corynoneura carriana|NHRS-BYWS000000378|Yngve Brodin|morphology|BOLD:AAU2657
Corynoneura carriana|ZMUO.025835|Lauri Paasivirta||BOLD:AAU2657
Corynoneura carriana|NHRS-BYWS000000986|Yngve Brodin|morphology|BOLD:AAU2657
Corynoneura carriana|NHRS-BYWS000001811|Yngve Brodin||BOLD:AAU2657
Corynoneura carriana|NHRS-BYWS000001810|Yngve Brodin||BOLD:AAU2657
Corynoneura carriana|NHRS-BYWS000001791|Yngve Brodin||BOLD:AAU2657
Corynoneura|BIOUG42738-C11|Kate Perez|BIN Taxonomy Match below Phylum (Jun 2019)|BOLD:AC...
Corynoneura|BIOUG42742-D01|Kate Perez|BIN Taxonomy Match below Phylum (Jun 2019)|BOLD:AC...
Corynoneura|BIOUG42688-G07|Kate Perez|BIN Taxonomy Match below Phylum (Jun 2019)|BOLD:AC...
Corynoneura sp. 16ES|CH-eik111|Elisabeth Stur||BOLD:ACG9970
Corynoneura|BIOUG55415-F03|Kate Perez|BIN Taxonomy Match (Oct 2020)|BOLD:AAN5330
Corynoneura arctica|ZMUO.025340|Lauri Paasivirta||BOLD:ACK6591
Corynoneura arctica|ZMUO.025339|Lauri Paasivirta||BOLD:ACK6591
Corynoneura sp. 17ES|TRD-CH253|Elisabeth Stur||BOLD:ACK6591
Corynoneura sp. 17ES|TRD-CH250|Elisabeth Stur||BOLD:ACK6591
Corynoneura sp. 17ES|TRD-CH317|Elisabeth Stur||BOLD:ACK6591
Corynoneura sp. 17ES|RIN_CH21|Elisabeth Stur|morphology|BOLD:ACK6591
Corynoneura sp. 17ES|RIN_CH22|Elisabeth Stur|morphology|BOLD:ACK6591
Corynoneura sp. 17ES|RIN_CH23|Elisabeth Stur|morphology|BOLD:ACK6591
Corynoneura sp. 17ES|RIN_CH51|Elisabeth Stur|morphology|BOLD:ACK6591
Corynoneura sp. 17ES|RIN_CH88|Elisabeth Stur|morphology|BOLD:ACK6591
Corynoneura sp. 17ES|RIN_CH92|Elisabeth Stur|morphology|BOLD:ACK6591
Corynoneura sp. 17ES|RIN_CH93|Elisabeth Stur|morphology|BOLD:ACK6591
Corynoneura sp. 17ES|TRD-CH394|Elisabeth Stur|morphology|BOLD:ACK6591
Corynoneura sp. 17ES|TRD-CH413|Elisabeth Stur|morphology|BOLD:ACK6591
Corynoneura sp. 17ES|TRD-CH296|Elisabeth Stur||BOLD:ACK6591
Corynoneura sp. 17ES|TRD-CH233|Elisabeth Stur||BOLD:ACK6591
Corynoneura sp. 17ES|TRD-CH251|Elisabeth Stur||BOLD:ACK6591
Corynoneura sp. 17ES|TRD-CH16|Elisabeth Stur||BOLD:ACK6591
Corynoneura sp. 17ES|TRD-CH354|Elisabeth Stur|morphology|BOLD:ACK6591
Corynoneura sp. 17ES|TRD-CH15|Elisabeth Stur||BOLD:ACK6591
Corynoneura sp. 17ES|NO 99|Elisabeth Stur||BOLD:ACK6591
Corynoneura scutellata|ZMUO.025085|Lauri Paasivirta||BOLD:AAB0075
Corynoneura scutellata|ZMUO.025084|Lauri Paasivirta||BOLD:AAB0075
Corynoneura sp. 7ES|Finnmark679|Elisabeth Stur||BOLD:AAB0075
Corynoneura sp. 7ES|Finnmark44|Elisabeth Stur||BOLD:AAB0075
Corynoneura scutellata|Finnmark109|Elisabeth Stur||BOLD:AAB0075
Corynoneura sp. 7ES|Finnmark418|Elisabeth Stur||BOLD:AAB0075
Corynoneura scutellata|Finnmark379|Elisabeth Stur||BOLD:AAB0075
Corynoneura sp. 7ES|Finnmark361|Elisabeth Stur||BOLD:AAB0075

Corynoneura sp. 7ES|Finnmark418|Elisabeth Stur||BOLD:AAB0075
Corynoneura scutellata|Finnmark379|Elisabeth Stur||BOLD:AAB0075
Corynoneura sp. 7ES|Finnmark361|Elisabeth Stur||BOLD:AAB0075
Corynoneura|ZMUO.025875|Lauri Paasivirta||BOLD:ACY8182
Corynoneura sp. 8ES|Finnmark284|Elisabeth Stur||BOLD:AAG5432
Thienemanniella|CCDB24229-G11|Sofia Wiedenbrug|BIN Taxonomy Match|BOLD:ACU4652
Thienemanniella|CCDB24229-G10|Sofia Wiedenbrug|BIN Taxonomy Match|BOLD:ACU4652
Thienemanniella caspersi|BIOUG08211-B04|Kate Perez|BIN Taxonomy Match|BOLD:AAG1617
Thienemanniella caspersi|EBAI-Ch025|Elisabeth Stur|morphology|BOLD:AAG1617
Thienemanniella caspersi|EBAI-Ch018|Elisabeth Stur|morphology|BOLD:AAG1617
Thienemanniella caspersi|ATNA549|Elisabeth Stur||BOLD:AAG1617
Thienemanniella caspersi|ATNA530|Elisabeth Stur||BOLD:AAG1617
Thienemanniella caspersi|NO19|Torbjorn Ekrem||BOLD:AAG1617
Thienemanniella caspersi|ATNA369|Elisabeth Stur||BOLD:AAG1617
Thienemanniella caspersi|EBAI-Ch029|Elisabeth Stur|morphology|BOLD:AAG1617
Thienemanniella caspersi|EBAI-Ch028|Elisabeth Stur|morphology|BOLD:AAG1617
Thienemanniella caspersi|EBAI-Ch027|Elisabeth Stur|morphology|BOLD:AAG1617
Thienemanniella caspersi|EBAI-Ch026|Elisabeth Stur|morphology|BOLD:AAG1617
Thienemanniella caspersi|EBAI-Ch024|Elisabeth Stur|morphology|BOLD:AAG1617
Thienemanniella caspersi|EBAI-Ch019|Elisabeth Stur|morphology|BOLD:AAG1617
Thienemanniella caspersi|EBAI-Ch017|Elisabeth Stur|morphology|BOLD:AAG1617
Thienemanniella caspersi|EBAI-Ch016|Elisabeth Stur|morphology|BOLD:AAG1617
Thienemanniella caspersi|EBAI-Ch157|Elisabeth Stur|morphology|BOLD:AAG1617
Thienemanniella caspersi|Finnmark762|Elisabeth Stur||BOLD:AAG1617
Thienemanniella caspersi|Finnmark758|Elisabeth Stur||BOLD:AAG1617
Thienemanniella caspersi|Finnmark854|Elisabeth Stur||BOLD:AAG1617
Thienemanniella caspersi|Finnmark853|Elisabeth Stur||BOLD:AAG1617
Corynoneurella paludosa|ZMUO.024876|Lauri Paasivirta||BOLD:AAV3049
Corynoneurella paludosa|ZMUO.024877|Lauri Paasivirta||BOLD:AAV3049
Corynoneurella paludosa|Finnmark855|Elisabeth Stur||BOLD:AAV3049
Corynoneurella paludosa|Finnmark856|Elisabeth Stur||BOLD:AAV3049
Corynoneurella paludosa|Finnmark363|Elisabeth Stur||BOLD:AAV3049
Thienemanniella nr. vittata|ZMUO.025380|Lauri Paasivirta||BOLD:ACQ9002
Thienemanniella nr. vittata|ZMUO.025381|Lauri Paasivirta||BOLD:ACQ9002
Thienemanniella|BIOUG16399-C02|Kate Perez|BIN Taxonomy Match (Aug 2017)|BOLD:ACQ9002
Thienemanniella|BIOUG16552-A09|Kate Perez|BIN Taxonomy Match (Aug 2017)|BOLD:ACQ9002
Thienemanniella minuscula|ZMUO.024879|Lauri Paasivirta||BOLD:ADA5504
Thienemanniella minuscula|ZMUO.024878|Lauri Paasivirta||BOLD:ADA5504
Corynoneura cf. antennalis|NHRS-BYWS000000515|Yngve Brodin|morphology|BOLD:ADA5504
Corynoneura cf. antennalis|NHRS-BYWS000000514|Yngve Brodin|morphology|BOLD:ADA5504
Corynoneura|CCDB24183-A09|Sofia Wiedenbrug|Morphology|BOLD:ACI6306
Corynoneura|BIOUG07413-E09|Kate Perez|BIN Taxonomy Match|BOLD:ACI6306
Corynoneura sp. 6ES|SOE338|Elisabeth Stur||BOLD:AAI5883
Corynoneura sp. 21ES|BIOUG05149-A08|Sofia Wiedenbrug|Morphology|BOLD:AAI0860
Corynoneura sp. 21ES|BIOUG05149-A03|Sofia Wiedenbrug|Morphology|BOLD:AAI0860
Corynoneura sp. 21ES|BIOUG07944-H05|Sofia Wiedenbrug|Morphology|BOLD:AAI0860
Corynoneura sp. 21ES|SOE35|Elisabeth Stur|Morphology|BOLD:AAI0860
Corynoneura sp. 21ES|CCDB24183-E10|Sofia Wiedenbrug|Morphology|BOLD:AAI0860
Corynoneura sp. 21ES|CCDB24183-E11|Sofia Wiedenbrug|Morphology|BOLD:AAI0860
Corynoneura sp. 21ES|CCDB24183-F08|Sofia Wiedenbrug|Morphology|BOLD:AAI0860
Corynoneura sp. 21ES|SOE398|Elisabeth Stur|Morphology|BOLD:AAI0860
Corynoneura sp. 21ES|SOE440|Elisabeth Stur|Morphology|BOLD:AAI0860
Corynoneura sp. 23ES|SOE36|Elisabeth Stur|Morphology|BOLD:AAE9742
Corynoneura sp. 23ES|SOE34|Elisabeth Stur|Morphology|BOLD:AAE9742
Corynoneura sp. 23ES|SOE32|Elisabeth Stur|Morphology|BOLD:AAE9742
Corynoneura sp. 23ES|SOE30|Elisabeth Stur|Morphology|BOLD:AAE9742
Corynoneura fittkaui|ZMUO.024675|Lauri Paasivirta||BOLD:AAC1838
Corynoneura fittkaui|ZMUO.024846|Lauri Paasivirta||BOLD:AAC1838
Corynoneura fittkaui|ZMUO.024845|Lauri Paasivirta||BOLD:AAC1838
Corynoneura fittkaui|SOE416|Torbjorn Ekrem||BOLD:AAC1838
Corynoneura fittkaui|SOE408|Torbjorn Ekrem||BOLD:AAC1838
Corynoneura fittkaui|SOE417|Torbjorn Ekrem||BOLD:AAC1838
Corynoneura fittkaui|SOE26|Elisabeth Stur||BOLD:AAC1838
Corynoneura fittkaui|SOE160|Torbjorn Ekrem||BOLD:AAC1838
Corynoneura fittkaui|SOE169|Torbjorn Ekrem||BOLD:AAC1838
Corynoneura fittkaui|SOE256|Torbjorn Ekrem||BOLD:AAC1838
Corynoneura fittkaui|SOE400|Torbjorn Ekrem||BOLD:AAC1838
Corynoneura fittkaui|SOE307|Torbjorn Ekrem||BOLD:AAC1838
Corynoneura fittkaui|SOE208|Torbjorn Ekrem||BOLD:AAC1838
Corynoneura fittkaui|SOE246|Torbjorn Ekrem||BOLD:AAC1838
Corynoneura fittkaui|SOE415|Torbjorn Ekrem||BOLD:AAC1838
Corynoneura fittkaui|SOE401|Torbjorn Ekrem||BOLD:AAC1838
Corynoneura sp. 24ES|CCDB22605-B03|Sofia Wiedenbrug|Morphology|BOLD:ACG3525
Corynoneura sp. 24ES|BIOUG05946-G03|Sofia Wiedenbrug|Morphology|BOLD:ACG3525
Corynoneura|CCDB24228-E05|Sofia Wiedenbrug|BIN Taxonomy Match|BOLD:ACU4229
Corynoneura|CCDB24228-G01|Sofia Wiedenbrug|BIN Taxonomy Match|BOLD:ACU5217
Corynoneura|CCDB24228-E03|Sofia Wiedenbrug||BOLD:ACU4365
Corynoneura|BIOUG07759-A03|Kate Perez|BIN Taxonomy Match|BOLD:ACI8241
Corynoneura|BIOUG08369-G10|Kate Perez|BIN Taxonomy Match|BOLD:ACI8241
Corynoneura|BIOUG08098-B07|Kate Perez|BIN Taxonomy Match|BOLD:ACI8241
Corynoneura sp. 4ES|EBAI-Ch147|Elisabeth Stur|morphology|BOLD:AAI5887
Corynoneura sp. 4ES|EBAI-Ch021|Elisabeth Stur|morphology|BOLD:AAI5887
Corynoneura sp. 4ES|EBAI-Ch032|Elisabeth Stur|morphology|BOLD:AAI5887
Corynoneura sp. 4ES|EBAI-Ch023|Elisabeth Stur|morphology|BOLD:AAI5887
Corynoneura sp. 4ES|EBAI-Ch038|Elisabeth Stur|morphology|BOLD:AAI5887
Corynoneura sp. 4ES|EBAI-Ch031|Elisabeth Stur|morphology|BOLD:AAI5887
Corynoneura sp. 4ES|EBAI-Ch022|Elisabeth Stur|morphology|BOLD:AAI5887
Corynoneura sp. 4ES|ATNA366|Elisabeth Stur||BOLD:AAI5887
Corynoneura sp. 4ES|Finnmark852|Elisabeth Stur||BOLD:AAI5887
Corynoneura sp. 4ES|Finnmark413|Elisabeth Stur||BOLD:AAI5887
Corynoneura sp. 4ES|TRD-CH130|Elisabeth Stur||BOLD:AAI5887
Corynoneura sp. 4ES|EBAI-Ch036|Elisabeth Stur|morphology|BOLD:AAI5887
Corynoneura sp. 4ES|EBAI-Ch030|Elisabeth Stur|morphology|BOLD:AAI5887
Corynoneura sp. 4ES|Finnmark620|Elisabeth Stur||BOLD:AAI5887
Corynoneura sp. 4ES|Finnmark600|Elisabeth Stur||BOLD:AAI5887
Corynoneura lobata|ZMUO.024676|Lauri Paasivirta||BOLD:ADA8695
Corynoneura fittkaui|ZMUO.024674|Lauri Paasivirta||BOLD:ADA7216
Corynoneura sp. 25ES|ZSM-DIP-33143-F10|Caroline Chimeno|BIN Taxonomy Match (Jan 2022)|BOLD...
Corynoneura sp. 25ES|CCDB22605-B05|Sofia Wiedenbrug|Morphology|BOLD:ACT8698
Corynoneura sp. 25ES|GBOL11544|Sofia Wiedenbrug|Morphology|BOLD:ACT8698
Corynoneura sp. 25ES|CCDB22605-B07|Sofia Wiedenbrug|Morphology|BOLD:ACT8698
Corynoneura sp. 25ES|CCDB22605-B06|Sofia Wiedenbrug|Morphology|BOLD:ACT8698
Corynoneura sp. 20ES|Finnmark768|Elisabeth Stur||BOLD:ACD1670

Corynoneura sp. 25ES|CCDB22605-B07|Sofia Wiedenbrug|Morphology|BOLD:ACT8698
Corynoneura sp. 25ES|CCDB22605-B06|Sofia Wiedenbrug|Morphology|BOLD:ACT8698
Corynoneura sp. 20ES|Finnmark768|Elisabeth Stur||BOLD:ACD1670
Corynoneura sp. 20ES|Finnmark764|Elisabeth Stur||BOLD:ACD1670
Corynoneura sp. 15ES|EBAI-Ch259|Elisabeth Stur|morphology|BOLD:AAM7073
Corynoneura sp. 15ES|BIOUG16396-A06|Kate Perez|BIN Taxonomy Match|BOLD:AAM7073
Corynoneura sp. 15ES|Finnmark46|Elisabeth Stur||BOLD:AAM7073
Corynoneura sp. 15ES|Finnmark800|Elisabeth Stur||BOLD:AAM7073
Corynoneura sp. 15ES|ATNA531|Elisabeth Stur||BOLD:AAM7073
Corynoneura sp. 15ES|PK-190-27|Elisabeth Stur|BIN Taxonomy Match?|BOLD:AAM7073
Corynoneura sp. 15ES|ATNA528|Elisabeth Stur||BOLD:AAM7073
Corynoneura sp. 15ES|EBAI-Ch149|Elisabeth Stur|morphology|BOLD:AAM7073
Corynoneura sp. 15ES|BIOUG16434-E07|Kate Perez|BIN Taxonomy Match|BOLD:AAM7073
Corynoneura sp. 15ES|EBAI-Ch037|Elisabeth Stur|morphology|BOLD:AAM7073
Corynoneura sp. 15ES|EBAI-Ch148|Elisabeth Stur|morphology|BOLD:AAM7073
Corynoneura sp. 15ES|BIOUG16333-E12|Kate Perez|BIN Taxonomy Match|BOLD:AAM7073
Corynoneura celeripes|ZMUO.024635|Lauri Paasivirta||BOLD:ACJ8746
Corynoneura sp. 22ES|CCDB24183-B02|Sofia Wiedenbrug|Morphology|BOLD:ACJ8746
Corynoneura sp. 12ES|TRD-CH414|Sofia Wiedenbrug|morphology|BOLD:ACX7382
Corynoneura sp. 12ES|TRD-CH249|Sofia Wiedenbrug|morphology|BOLD:ACX7382
Corynoneura sp. 12ES|TRD-CH232|Sofia Wiedenbrug|morphology|BOLD:ACX7382
Corynoneura sp. 12ES|TRD-CH431|Elisabeth Stur|morphology|BOLD:ACX7382
Corynoneura sp. 12ES|TRD-CH430|Elisabeth Stur|morphology|BOLD:ACX7382
Corynoneura sp. 12ES|TRD-CH429|Elisabeth Stur|morphology|BOLD:ACX7382
Corynoneura sp. 12ES|Finnmark463|Sofia Wiedenbrug|morphology|BOLD:ACX7382
Thienemanniella cf. clavicornis|CCDB24228-E04|Sofia Wiedenbrug|BIN Taxonomy Match|BOLD:AAG1613
Thienemanniella cf. clavicornis|ES135|Torbjorn Ekrem||BOLD:AAG1613
Thienemanniella|ZSM-DIP-33144-G09|Caroline Chimeno|BIN Taxonomy Match (Jan 2022)|BOLD:AE...
Thienemanniella majuscula|ZMUO.024775|Lauri Paasivirta||BOLD:AEE9942
Thienemanniella obscura|ZMUO.026250|Lauri Paasivirta||BOLD:AEE9942
Thienemanniella obscura|ZMUO.026249|Lauri Paasivirta||BOLD:AEE9942
Thienemanniella|ZSM-DIP-33149-H06|Caroline Chimeno|BIN Taxonomy Match (Jan 2022)|BOLD:AE...
Thienemanniella|ZSM-DIP-33172-C07|Caroline Chimeno|BIN Taxonomy Match (Jan 2022)|BOLD:AE...
Thienemanniella obscura|CH-OSF134|Torbjorn Ekrem||BOLD:AAV3051
Corynoneura coronata|ZMUO.024711|Lauri Paasivirta||BOLD:ADA9118
Corynoneura coronata|ZMUO.024710|Lauri Paasivirta||BOLD:ADA9118
Corynoneura coronata|ZSM-DIP-33145-C08|Caroline Chimeno|BIN Taxonomy Match (Jan 2022)|BOLD...
Corynoneura sp. 14ES|BIOUG15964-G02|Elisabeth Stur|Morphology|BOLD:AAV1539
Corynoneura sp. 14ES|BIOUG16477-G01|Elisabeth Stur|Morphology|BOLD:AAV1539
Corynoneura celtica|EJV-20110203|Lauri Paasivirta||BOLD:AAV1539
Corynoneura celtica|ZMUO.025832|Lauri Paasivirta||BOLD:AAV1539
Corynoneura celtica|ZMUO.025833|Lauri Paasivirta||BOLD:AAV1539
Corynoneura celtica|EJV-20110202|Lauri Paasivirta||BOLD:AAV1539
Corynoneura sp. 14ES|Finnmark516|Elisabeth Stur||BOLD:AAV1539
Corynoneura sp. 14ES|BIOUG16552-G03|Elisabeth Stur|Morphology|BOLD:AAV1539
Corynoneura sp. 14ES|BIOUG16555-H02|Elisabeth Stur|Morphology|BOLD:AAV1539
Corynoneura sp. 14ES|BIOUG16551-F04|Elisabeth Stur|Morphology|BOLD:AAV1539
Corynoneura sp. 14ES|TRD-CH384|Elisabeth Stur|morphology|BOLD:AAV1539
Corynoneura sp. 14ES|BIOUG16431-E07|Elisabeth Stur|Morphology|BOLD:AAV1539
Corynoneura sp. 14ES|BIOUG16557-G08|Elisabeth Stur|Morphology|BOLD:AAV1539
Corynoneura celtica|EJV-20110201|Lauri Paasivirta||BOLD:AAV1539
Corynoneura sp. 14ES|Finnmark263|Elisabeth Stur||BOLD:AAV1539
Corynoneura sp. 18ES|TRD-CH198|Elisabeth Stur|morphology|BOLD:ACT7603
Corynoneura lacustris|ZMUO.024518|Lauri Paasivirta||BOLD:ACG9967
Corynoneura lacustris|TRD-CH141|Elisabeth Stur|morphology|BOLD:ACG9967
Corynoneura lacustris|NHRS-BYWS000000156|Yngve Brodin|morphology|BOLD:ACG9967
Corynoneura lacustris|TRD-CH419|Elisabeth Stur|morphology|BOLD:ACG9967
Corynoneura lacustris|TRD-CH248|Elisabeth Stur||BOLD:ACG9967
Corynoneura lobata|ZMUO.024677|Lauri Paasivirta||BOLD:ACG9967
Corynoneura lacustris|ZMUO.024517|Lauri Paasivirta||BOLD:ACG9967
Corynoneura lacustris|TRD-CH247|Elisabeth Stur||BOLD:ACG9967
Corynoneura|CCDB21605-H01|Sofia Wiedenbrug||BOLD:ACG9967
Corynoneura lacustris|TRD-CH140|Elisabeth Stur|morphology|BOLD:ACG9967
Corynoneura lacustris|CH-eik114|Elisabeth Stur||BOLD:ACG9967
Corynoneura lacustris|CH-eik87|Elisabeth Stur||BOLD:ACG9967
Thienemanniella majuscula|BIOUG17024-G03|Kate Perez|BIN Taxonomy Match|BOLD:AAP6085
Thienemanniella majuscula|BIOUG16713-C05|Kate Perez|BIN Taxonomy Match|BOLD:AAP6085
Thienemanniella majuscula|Finnmark193|Torbjorn Ekrem||BOLD:AAP6085
Thienemanniella majuscula|ZMUO.024776|Lauri Paasivirta||BOLD:AAP6085
Thienemanniella majuscula|BIOUG16592-B08|Kate Perez|BIN Taxonomy Match|BOLD:AAP6085
Thienemanniella vittata|ZMUO.024766|Lauri Paasivirta||BOLD:AAV3048
Thienemanniella vittata|ZMUO.024765|Lauri Paasivirta||BOLD:AAV3048
Thienemanniella sp. 3TE|Finnmark860|Elisabeth Stur||BOLD:AAV3048
Thienemanniella sp. 3TE|Finnmark861|Elisabeth Stur||BOLD:AAV3048
Thienemanniella sp. 3TE|Finnmark862|Elisabeth Stur||BOLD:AAV3048
Thienemanniella sp. 3TE|Finnmark859|Elisabeth Stur||BOLD:AAV3048
Thienemanniella sp. 3TE|Finnmark858|Elisabeth Stur||BOLD:AAV3048
Thienemanniella sp. 3TE|Finnmark350|Torbjorn Ekrem||BOLD:AAV3048
Thienemanniella xena|Finnmark816|Elisabeth Stur||BOLD:AAD5253
Thienemanniella vittata|EJV-20110210|Lauri Paasivirta||BOLD:AAD5253
Thienemanniella vittata|BIOUG16597-D11|Kate Perez|BIN Taxonomy Match|BOLD:AAF7734
Thienemanniella vittata|BIOUG16479-D03|Kate Perez|BIN Taxonomy Match|BOLD:AAF7734
Thienemanniella vittata|24502-H07|Kate Perez|BIN Taxonomy Match|BOLD:AAF7734
Thienemanniella vittata|TRD-CH199|Elisabeth Stur||BOLD:AAF7734
Thienemanniella vittata|Finnmark895|Elisabeth Stur||BOLD:AAF7734
Thienemanniella vittata|Finnmark815|Elisabeth Stur||BOLD:AAF7734
Thienemanniella vittata|EJV-20110209|Lauri Paasivirta||BOLD:AAF7734
Thienemanniella vittata|NO18|Torbjorn Ekrem||BOLD:AAF7734
Thienemanniella vittata|Finnmark228|Torbjorn Ekrem||BOLD:AAF7734
Thienemanniella vittata|BIOUG16299-D01|Kate Perez|BIN Taxonomy Match|BOLD:AAF7734
Thienemanniella vittata|BIOUG16596-B07|Kate Perez|BIN Taxonomy Match|BOLD:AAF7734
Thienemanniella vittata|EJV-20110207|Lauri Paasivirta||BOLD:AAF7734
Thienemanniella vittata|BIOUG16329-F06|Kate Perez|BIN Taxonomy Match|BOLD:AAF7734
Thienemanniella vittata|Finnmark266|Torbjorn Ekrem||BOLD:AAF7734
Orthocladiinae|BIOUG07759-B11|Jerome Moriniere|BIN Taxonomy Match|BOLD:AAQ0599
Chaetocladius incertus|ATNA431|Elisabeth Stur|morphology|BOLD:AAC8747
Chaetocladius incertus|Finnmark436|Elisabeth Stur||BOLD:AAC8747
Thienemannia gracilis|ZMUO.024899|Lauri Paasivirta||BOLD:ADA9339
Thienemannia gracilis|ZMUO.024898|Lauri Paasivirta||BOLD:ADA9339
Metriocnemus sp. 1SW|CCDB21605-B11|Sofia Wiedenbrug||BOLD:ACB9910
Metriocnemus sp. 1SW|BIOUG15648-A01|Elisabeth Stur|BIN Taxonomy Match
Metriocnemus|BIOUG04217-H11|Kate Perez|BIN Taxonomy Match|BOLD:ACB9910
Metriocnemus sp. 1SW|BIOUG15797-G09|Elisabeth Stur|BIN Taxonomy Match|BOLD:ACB9910

[illegible]
Metriocnemus sp. 1SW|BIOUG15648-A01|Elisabeth Stur|BIN Taxonomy Match
Metriocnemus|BIOUG04217-H11|Kate Perez|BIN Taxonomy Match|BOLD:ACB9910
Metriocnemus sp. 1SW|BIOUG15797-G09|Elisabeth Stur|BIN Taxonomy Match|BOLD:ACB9910
Metriocnemus|BIOUG04217-A04|Kate Perez|BIN Taxonomy Match|BOLD:ACB9910
Metriocnemus|BIOUG04090-H05|Kate Perez|BIN Taxonomy Match|BOLD:ACB9910
Metriocnemus|BIOUG04089-B05|Kate Perez|BIN Taxonomy Match|BOLD:ACB9910
Metriocnemus|BIOUG04261-A05|Kate Perez|BIN Taxonomy Match|BOLD:ACB9910
Metriocnemus|BIOUG04114-E04|Kate Perez|BIN Taxonomy Match|BOLD:ACB9910
Metriocnemus|BIOUG04433-E04|Kate Perez|BIN Taxonomy Match|BOLD:ACB9910
Metriocnemus|BIOUG04261-B01|Kate Perez|BIN Taxonomy Match|BOLD:ACB9910
Metriocnemus|BIOUG04217-C01|Kate Perez|BIN Taxonomy Match|BOLD:ACB9910
Metriocnemus|BIOUG04217-H03|Kate Perez|BIN Taxonomy Match|BOLD:ACB9910
Metriocnemus|BIOUG04217-H04|Kate Perez|BIN Taxonomy Match|BOLD:ACB9910
Metriocnemus|BIOUG04217-H07|Kate Perez|BIN Taxonomy Match|BOLD:ACB9910
Metriocnemus|BIOUG04153-G08|Kate Perez|BIN Taxonomy Match|BOLD:ACB9910
Metriocnemus sp. 1SW|BIOUG15674-D07|Elisabeth Stur|BIN Taxonomy Match|BOLD:ACB9910
Metriocnemus sp. 1SW|BIOUG15809-A07|Elisabeth Stur|BIN Taxonomy Match|BOLD:ACB9910
Metriocnemus|BIOUG04114-B03|Kate Perez|BIN Taxonomy Match|BOLD:ACB9910
Metriocnemus|BIOUG04114-A03|Kate Perez|BIN Taxonomy Match|BOLD:ACB9910
Metriocnemus|BIOUG04085-F07|Kate Perez|BIN Taxonomy Match|BOLD:ACB9910
Bryophaenocladius ictericus|BIOUG36777-A05|Kate Perez|BIN Taxonomy Match (Jul 2018)|BOLD ...
Bryophaenocladius ictericus|CCDB24039-F07|Sofia Wiedenbrug|BIN Taxonomy Match|BOLD:AAM6273
Bryophaenocladius ictericus|CCDB24039-F09|Sofia Wiedenbrug|BIN Taxonomy Match|BOLD:AAM6273
Bryophaenocladius ictericus|BIOUG42704-D02|Kate Perez|BIN Taxonomy Match below Phylum (Jun 201...
Bryophaenocladius ictericus|BIOUG07755-A08|Kate Perez|BIN Taxonomy Match|BOLD:AAM6273
Bryophaenocladius ictericus|ZSM34343-B08|Sofia Wiedenbrug|Morphology|BOLD:AAM6273
Bryophaenocladius ictericus|ZSM34343-B05|Sofia Wiedenbrug|Morphology|BOLD:AAM6273
Bryophaenocladius ictericus|CCDB24038-B10|Sofia Wiedenbrug|BIN Taxonomy Match|BOLD:AAM6273
Bryophaenocladius ictericus|TRD-CH194|Elisabeth Stur||BOLD:AAM6273
Bryophaenocladius ictericus|NHRS-BYWS000000192|Yngve Brodin|morphology|BOLD:AAM6273
Bryophaenocladius ictericus|GBOL03734|Sofia Wiedenbrug||BOLD:AAM6273
Bryophaenocladius ictericus|CH-OSF169|Elisabeth Stur||BOLD:AAM6273
Bryophaenocladius ictericus|BIOUG08220-H03|Kate Perez|BIN Taxonomy Match|BOLD:AAM6273
Bryophaenocladius ictericus|BIOUG08130-H09|Kate Perez|BIN Taxonomy Match|BOLD:AAM6273
Bryophaenocladius ictericus|CCDB24039-F08|Sofia Wiedenbrug|BIN Taxonomy Match|BOLD:AAM6273
Bryophaenocladius ictericus|BIOUG07508-D09|Kate Perez|BIN Taxonomy Match|BOLD:AAM6273
Bryophaenocladius ictericus|BIOUG08091-D07|Kate Perez|BIN Taxonomy Match|BOLD:AAM6273
Bryophaenocladius ictericus|BIOUG08260-H01|Kate Perez|BIN Taxonomy Match|BOLD:AAM6273
Bryophaenocladius ictericus|BIOUG06068-B03|Kate Perez|BIN Taxonomy Match|BOLD:AAM6273
Bryophaenocladius ictericus|BIOUG06068-F09|Kate Perez|BIN Taxonomy Match|BOLD:AAM6273
Bryophaenocladius ictericus|BIOUG06069-G10|Kate Perez|BIN Taxonomy Match|BOLD:AAM6273
Bryophaenocladius ictericus|Finnmark689|Elisabeth Stur||BOLD:AAM6273
Bryophaenocladius ictericus|BIOUG05389-A06|Kate Perez|BIN Taxonomy Match|BOLD:AAM6273
Bryophaenocladius ictericus|BIOUG08252-F09|Kate Perez|BIN Taxonomy Match|BOLD:AAM6273
Bryophaenocladius ictericus|BIOUG05897-C12|Kate Perez|BIN Taxonomy Match|BOLD:AAM6273
Bryophaenocladius ictericus|BIOUG05897-F07|Kate Perez|BIN Taxonomy Match|BOLD:AAM6273
Bryophaenocladius ictericus|CCDB24038-B09|Sofia Wiedenbrug|BIN Taxonomy Match|BOLD:AAM6273
Bryophaenocladius ictericus|BIOUG05900-G02|Kate Perez|BIN Taxonomy Match|BOLD:AAM6273
Bryophaenocladius ictericus|BIOUG06052-E09|Kate Perez|BIN Taxonomy Match|BOLD:AAM6273
Bryophaenocladius ictericus|BIOUG08254-B08|Kate Perez|BIN Taxonomy Match|BOLD:AAM6273
Bryophaenocladius ictericus|CCDB24039-B01|Sofia Wiedenbrug|BIN Taxonomy Match|BOLD:AAM6273
Bryophaenocladius ictericus|BIOUG37052-E03|Kate Perez|BIN Taxonomy Match (Jul 2018)|BOLD ...
Bryophaenocladius ictericus|ZSM34343-B06|Sofia Wiedenbrug|Morphology|BOLD:AAM6273
Bryophaenocladius ictericus|BIOUG42918-C08|Kate Perez|BIN Taxonomy Match below Phylum (Jun 201...
Bryophaenocladius ictericus|ZSM34342-B07|Sofia Wiedenbrug|Morphology|BOLD:AAM6273
Bryophaenocladius ictericus|CH-eik137|Elisabeth Stur||BOLD:AAM6273
Bryophaenocladius ictericus|ZSM-DIP-33145-A09|Caroline Chimeno|BIN Taxonomy Match (Jan 2022 ...
Bryophaenocladius ictericus|ZSM-DIP-33146-G03|Caroline Chimeno|BIN Taxonomy Match (Jan 2022 ...
Bryophaenocladius ictericus|ZSM-DIP-33148-H10|Caroline Chimeno|BIN Taxonomy Match (Jan 2022 ...
Bryophaenocladius ictericus|CCDB21606-F12|Sofia Wiedenbrug||BOLD:AAM6273
Chironomidae|Koz1141|Mikhail V. Kozlov||BOLD:AAM6273
Bryophaenocladius ictericus|BIOUG36788-H08|Kate Perez|BIN Taxonomy Match (Jul 2018)|BOLD ...
Bryophaenocladius ictericus|ZSM34343-H08|Sofia Wiedenbrug|Morphology|BOLD:AAM6273
Bryophaenocladius ictericus|BIOUG15762-H01|Kate Perez|BIN Taxonomy Match|BOLD:AAM6273
Bryophaenocladius ictericus|BIOUG04153-E11|Kate Perez|BIN Taxonomy Match|BOLD:AAM6273
Bryophaenocladius ictericus|BIOUG04153-F11|Kate Perez|BIN Taxonomy Match|BOLD:AAM6273
Bryophaenocladius ictericus|BIOUG04362-B05|Kate Perez|BIN Taxonomy Match|BOLD:AAM6273
Bryophaenocladius ictericus|ZSM34343-H03|Sofia Wiedenbrug|Morphology|BOLD:AAM6273
Bryophaenocladius ictericus|BIOUG04212-D03|Kate Perez|BIN Taxonomy Match|BOLD:AAM6273
Bryophaenocladius ictericus|BIOUG04216-H03|Kate Perez|BIN Taxonomy Match|BOLD:AAM6273
Bryophaenocladius ictericus|BIOUG04216-H11|Kate Perez|BIN Taxonomy Match|BOLD:AAM6273
Bryophaenocladius ictericus|BIOUG04153-F02|Kate Perez|BIN Taxonomy Match|BOLD:AAM6273
Bryophaenocladius ictericus|BIOUG04296-E03|Kate Perez|BIN Taxonomy Match|BOLD:AAM6273
Bryophaenocladius ictericus|BIOUG04296-G10|Kate Perez|BIN Taxonomy Match|BOLD:AAM6273
Bryophaenocladius ictericus|BIOUG04155-E12|Kate Perez|BIN Taxonomy Match|BOLD:AAM6273
Bryophaenocladius ictericus|BIOUG04362-A01|Kate Perez|BIN Taxonomy Match|BOLD:AAM6273
Bryophaenocladius ictericus|BIOUG04362-E12|Kate Perez|BIN Taxonomy Match|BOLD:AAM6273
Bryophaenocladius ictericus|BIOUG04362-G06|Kate Perez|BIN Taxonomy Match|BOLD:AAM6273
Bryophaenocladius ictericus|BIOUG04362-H01|Kate Perez|BIN Taxonomy Match|BOLD:AAM6273
Bryophaenocladius ictericus|BIOUG04153-C03|Kate Perez|BIN Taxonomy Match|BOLD:AAM6273
Bryophaenocladius ictericus|BIOUG04362-G07|Kate Perez|BIN Taxonomy Match|BOLD:AAM6273
Bryophaenocladius ictericus|BIOUG36786-A10|Kate Perez|BIN Taxonomy Match (Jul 2018)|BOLD ...
Bryophaenocladius ictericus|BIOUG15681-F12|Kate Perez|BIN Taxonomy Match|BOLD:AAM6273
Bryophaenocladius ictericus|BIOUG15571-F05|Kate Perez|BIN Taxonomy Match|BOLD:AAM6273
Bryophaenocladius ictericus|BIOUG15598-A01|Kate Perez|BIN Taxonomy Match|BOLD:AAM6273
Bryophaenocladius ictericus|BIOUG16433-C04|Kate Perez|BIN Taxonomy Match|BOLD:AAM6273
Bryophaenocladius ictericus|BIOUG16632-H01|Kate Perez|BIN Taxonomy Match|BOLD:AAM6273
Bryophaenocladius ictericus|BIOUG36786-A03|Kate Perez|BIN Taxonomy Match (Jul 2018)|BOLD ...
Bryophaenocladius ictericus|BIOUG36827-D09|Kate Perez|BIN Taxonomy Match (Jul 2018)|BOLD ...
Bryophaenocladius ictericus|BIOUG36681-C08|Kate Perez|BIN Taxonomy Match (Jul 2018)|BOLD ...
Bryophaenocladius ictericus|CCDB24038-B08|Sofia Wiedenbrug|BIN Taxonomy Match|BOLD:AAM6273
Bryophaenocladius ictericus|BIOUG36931-F11|Kate Perez|BIN Taxonomy Match (May 2018)|BOLD ...
Bryophaenocladius ictericus|BIOUG36932-C08|Kate Perez|BIN Taxonomy Match (Jul 2018)|BOLD ...
Bryophaenocladius ictericus|BIOUG16619-A02|Kate Perez|BIN Taxonomy Match|BOLD:AAM6273
Bryophaenocladius ictericus|BIOUG04366-H02|Kate Perez|BIN Taxonomy Match|BOLD:AAM6273
Bryophaenocladius ictericus|BIOUG04362-B10|Kate Perez|BIN Taxonomy Match|BOLD:AAM6273
Bryophaenocladius ictericus|BIOUG04362-E07|Kate Perez|BIN Taxonomy Match|BOLD:AAM6273
Bryophaenocladius ictericus|BIOUG15593-B01|Kate Perez|BIN Taxonomy Match|BOLD:AAM6273
Bryophaenocladius ictericus|BIOUG15648-A08|Kate Perez|BIN Taxonomy Match|BOLD:AAM6273
Bryophaenocladius ictericus|ZSM34343-G11|Sofia Wiedenbrug|Morphology|BOLD:AAM6273
Bryophaenocladius ictericus|BIOUG15652-E07|Kate Perez|BIN Taxonomy Match|BOLD:AAM6273
Bryophaenocladius ictericus|BIOUG37181-D10|Kate Perez|BIN Taxonomy Match (Jul 2018)|BOLD ...
[illegible]

Bryophaenocladius ictericus|ZSM34343-G11|Sofia Wiedenbrug|Morphology|BOLD:AAM6273
Bryophaenocladius ictericus|BIOUG15652-E07|Kate Perez|BIN Taxonomy Match|BOLD:AAM6273
Bryophaenocladius ictericus|BIOUG37181-D10|Kate Perez|BIN Taxonomy Match (Jul 2018)|BOLD ...
Bryophaenocladius ictericus|ZSM34343-H06|Sofia Wiedenbrug|Morphology|BOLD:AAM6273
Bryophaenocladius ictericus|BIOUG15895-A06|Kate Perez|BIN Taxonomy Match|BOLD:AAM6273
Bryophaenocladius ictericus|BIOUG15798-B03|Kate Perez|BIN Taxonomy Match|BOLD:AAM6273
Bryophaenocladius ictericus|BIOUG15792-H07|Kate Perez|BIN Taxonomy Match|BOLD:AAM6273
Bryophaenocladius ictericus|BIOUG15762-E02|Kate Perez|BIN Taxonomy Match|BOLD:AAM6273
Bryophaenocladius ictericus|BIOUG15763-A05|Kate Perez|BIN Taxonomy Match|BOLD:AAM6273
Orthocladiinae|ZSM34343-H07|Sofia Wiedenbrug|Morphology|BOLD:ADC4901
Bryophaenocladius cf. vernalis|BIOUG07508-F08|Kate Perez|BIN Taxonomy Match|BOLD:AAP6930
Bryophaenocladius cf. vernalis|BIOUG07508-B10|Kate Perez|BIN Taxonomy Match|BOLD:AAP6930
Bryophaenocladius cf. vernalis|BIOUG07504-D12|Kate Perez|BIN Taxonomy Match|BOLD:AAP6930
Bryophaenocladius cf. vernalis|BIOUG07408-E09|Kate Perez|BIN Taxonomy Match|BOLD:AAP6930
Bryophaenocladius cf. vernalis|CH-OSF78|Elisabeth Stur||BOLD:AAP6930
Bryophaenocladius cf. vernalis|BIOUG07806-C04|Kate Perez|BIN Taxonomy Match|BOLD:AAP6930
Bryophaenocladius cf. vernalis|BIOUG07405-B06|Kate Perez|BIN Taxonomy Match|BOLD:AAP6930
Bryophaenocladius cf. vernalis|BIOUG07756-H08|Kate Perez|BIN Taxonomy Match|BOLD:AAP6930
Bryophaenocladius cf. vernalis|BIOUG06070-F04|Kate Perez|BIN Taxonomy Match|BOLD:AAP6930
Bryophaenocladius cf. vernalis|BIOUG06097-D05|Kate Perez|BIN Taxonomy Match|BOLD:AAP6930
Bryophaenocladius cf. vernalis|CH-eik40|Elisabeth Stur||BOLD:AAP6930
Bryophaenocladius cf. vernalis|BIOUG07648-E05|Kate Perez|BIN Taxonomy Match|BOLD:AAP6930
Bryophaenocladius cf. vernalis|BIOUG07568-B07|Kate Perez|BIN Taxonomy Match|BOLD:AAP6930
Bryophaenocladius cf. vernalis|BIOUG07756-G05|Kate Perez|BIN Taxonomy Match|BOLD:AAP6930
Bryophaenocladius cf. vernalis|BIOUG06208-B01|Kate Perez|BIN Taxonomy Match|BOLD:AAP6930
Bryophaenocladius|ZSM-DIP-33142-C04|Caroline Chimeno|BIN Taxonomy Match (Jan 2022)|BOLD:...
Bryophaenocladius|ZSM-DIP-33133-D10|Caroline Chimeno|BIN Taxonomy Match (Jan 2022)|BOLD:...
Bryophaenocladius cf. vernalis|BIOUG07508-H05|Kate Perez|BIN Taxonomy Match|BOLD:AAP6930
Bryophaenocladius cf. vernalis|BIOUG07407-E03|Kate Perez|BIN Taxonomy Match|BOLD:AAP6930
Bryophaenocladius cf. vernalis|BIOUG07409-H10|Kate Perez|BIN Taxonomy Match|BOLD:AAP6930
Bryophaenocladius cf. vernalis|CH-eik120|Elisabeth Stur||BOLD:AAP6930
Bryophaenocladius cf. vernalis|BIOUG07565-D07|Kate Perez|BIN Taxonomy Match|BOLD:AAP6930
Bryophaenocladius cf. vernalis|BIOUG06047-F10|Kate Perez|BIN Taxonomy Match|BOLD:AAP6930
Bryophaenocladius cf. vernalis|Finnmark171|Elisabeth Stur||BOLD:AAP6930
Bryophaenocladius cf. vernalis|CH-eik48|Elisabeth Stur||BOLD:AAP6930
Bryophaenocladius cf. vernalis|CH-eik103|Elisabeth Stur||BOLD:AAP6930
Bryophaenocladius ictericus|ZMUO.024896|Lauri Paasivirta||BOLD:AAP6930
Bryophaenocladius ictericus|ZMUO.024897|Lauri Paasivirta||BOLD:AAP6930
Bryophaenocladius cf. vernalis|CCDB21606-E07|Sofia Wiedenbrug||BOLD:AAP6930
Bryophaenocladius cf. vernalis|BIOUG06087-E04|Kate Perez|BIN Taxonomy Match|BOLD:AAP6930
Bryophaenocladius cf. vernalis|BIOUG06087-B04|Kate Perez|BIN Taxonomy Match|BOLD:AAP6930
Bryophaenocladius cf. vernalis|BIOUG06090-G06|Kate Perez|BIN Taxonomy Match|BOLD:AAP6930
Bryophaenocladius cf. vernalis|BIOUG06093-B11|Kate Perez|BIN Taxonomy Match|BOLD:AAP6930
Bryophaenocladius cf. vernalis|BIOUG07805-G01|Kate Perez|BIN Taxonomy Match|BOLD:AAP6930
Bryophaenocladius cf. vernalis|BIOUG06097-E09|Kate Perez|BIN Taxonomy Match|BOLD:AAP6930
Bryophaenocladius cf. vernalis|BIOUG07505-E09|Sofia Wiedenbrug||BOLD:AAP6930
Bryophaenocladius cf. vernalis|BIOUG04153-A04|Kate Perez|BIN Taxonomy Match|BOLD:AAP6930
Bryophaenocladius|ZSM-DIP-33142-C05|Caroline Chimeno|BIN Taxonomy Match (Jan 2022)|BOLD:...
Bryophaenocladius cf. vernalis|BIOUG07507-G10|Sofia Wiedenbrug||BOLD:AAP6930
Bryophaenocladius cf. vernalis|BIOUG07505-A07|Sofia Wiedenbrug||BOLD:AAP6930
Bryophaenocladius cf. vernalis|BIOUG07507-G11|Kate Perez|BIN Taxonomy Match|BOLD:AAP6930
Bryophaenocladius cf. vernalis|BIOUG06055-G01|Kate Perez|BIN Taxonomy Match|BOLD:AAP6930
Bryophaenocladius cf. vernalis|BIOUG06070-C09|Kate Perez|BIN Taxonomy Match|BOLD:AAP6930
Bryophaenocladius cf. vernalis|BIOUG06090-F01|Kate Perez|BIN Taxonomy Match|BOLD:AAP6930
Bryophaenocladius cf. vernalis|BIOUG06094-B01|Kate Perez|BIN Taxonomy Match|BOLD:AAP6930
Bryophaenocladius cf. vernalis|BIOUG07505-A05|Kate Perez|BIN Taxonomy Match|BOLD:AAP6930
Bryophaenocladius cf. vernalis|BIOUG07507-E09|Sofia Wiedenbrug||BOLD:AAP6930
Bryophaenocladius cf. vernalis|BIOUG07415-G04|Kate Perez|BIN Taxonomy Match|BOLD:AAP6930
Bryophaenocladius cf. vernalis|CH-eik71|Elisabeth Stur||BOLD:AAP6930
Bryophaenocladius cf. vernalis|BIOUG06068-F03|Kate Perez|BIN Taxonomy Match|BOLD:AAP6930
Bryophaenocladius cf. vernalis|BIOUG04362-D04|Kate Perez|BIN Taxonomy Match|BOLD:AAP6930
Bryophaenocladius cf. vernalis|BIOUG06047-G05|Kate Perez|BIN Taxonomy Match|BOLD:AAP6930
Bryophaenocladius cf. vernalis|BIOUG07945-F02|Kate Perez|BIN Taxonomy Match|BOLD:AAP6930
Bryophaenocladius cf. vernalis|BIOUG07563-A05|Kate Perez|BIN Taxonomy Match|BOLD:AAP6930
Bryophaenocladius cf. vernalis|BIOUG08093-E09|Kate Perez|BIN Taxonomy Match|BOLD:AAP6930
Bryophaenocladius cf. vernalis|BIOUG06441-H02|Kate Perez|BIN Taxonomy Match|BOLD:AAP6930
Bryophaenocladius cf. vernalis|BIOUG07755-A01|Kate Perez|BIN Taxonomy Match|BOLD:AAP6930
Bryophaenocladius cf. vernalis|BIOUG07647-E10|Kate Perez|BIN Taxonomy Match|BOLD:AAP6930
Bryophaenocladius cf. vernalis|BIOUG07564-C03|Kate Perez|BIN Taxonomy Match|BOLD:AAP6930
Bryophaenocladius cf. vernalis|BIOUG07649-D03|Kate Perez|BIN Taxonomy Match|BOLD:AAP6930
Bryophaenocladius cf. vernalis|BIOUG07927-D02|Kate Perez|BIN Taxonomy Match|BOLD:AAP6930
Bryophaenocladius cf. vernalis|BIOUG07414-F12|Kate Perez|BIN Taxonomy Match|BOLD:AAP6930
Bryophaenocladius cf. vernalis|BIOUG06280-D05|Kate Perez|BIN Taxonomy Match|BOLD:AAP6930
Bryophaenocladius cf. vernalis|BIOUG07563-F02|Kate Perez|BIN Taxonomy Match|BOLD:AAP6930
Bryophaenocladius cf. vernalis|BIOUG06070-G02|Kate Perez|BIN Taxonomy Match|BOLD:AAP6930
Bryophaenocladius cf. vernalis|BIOUG06446-B06|Kate Perez|BIN Taxonomy Match|BOLD:AAP6930
Bryophaenocladius cf. vernalis|BIOUG08318-E04|Kate Perez|BIN Taxonomy Match|BOLD:AAP6930
Bryophaenocladius cf. vernalis|BIOUG07648-D04|Kate Perez|BIN Taxonomy Match|BOLD:AAP6930
Bryophaenocladius cf. vernalis|Finnmark614|Elisabeth Stur||BOLD:AAP6930
Bryophaenocladius inconstans|24540-H03|Kate Perez|BIN Taxonomy Match|BOLD:AAF6748
Bryophaenocladius inconstans|ATNA423|Elisabeth Stur||BOLD:AAF6748
Bryophaenocladius inconstans|ATNA422|Elisabeth Stur||BOLD:AAF6748
Bryophaenocladius inconstans|ATNA421|Elisabeth Stur||BOLD:AAF6748
Zalutschia zalutschicola|ZMUO.024274|Lauri Paasivirta||BOLD:ADA7791
Zalutschia zalutschicola|ZMUO.024273|Lauri Paasivirta||BOLD:ADA7791
Zalutschia zalutschicola|PP0262|Lauri Paasivirta||BOLD:ADA7791
Zalutschia sp. 1ES|TRD-CH104|Elisabeth Stur|morphology|BOLD:AAV3187
Zalutschia sp. 1ES|Finnmark425|Elisabeth Stur||BOLD:AAV3187
Zalutschia sp. 1ES|Finnmark336|Elisabeth Stur||BOLD:AAV3187
Tvetenia bavarica grp|CCDB21606-D03|Sofia Wiedenbrug||BOLD:ACT6688
Tvetenia|GBOL11531|Sofia Wiedenbrug||BOLD:ACU4419
Tvetenia|GBOL11526|Sofia Wiedenbrug||BOLD:ACU4419
Tvetenia calvescens|ES56|Elisabeth Stur||BOLD:AAF6377
Trissocladius brevipalpis|ZMUO.025938|Lauri Paasivirta||BOLD:AAI3500
Oliveridia|ATNA382|Elisabeth Stur||BOLD:AAI3500
Oliveridia|ATNA304|Elisabeth Stur||BOLD:AAI3500
Oliveridia|ATNA379|Elisabeth Stur||BOLD:AAI3500
Orthocladiinae|BIOUG16777-H05|Jerome Moriniere|BIN Taxonomy Match|BOLD:ACU2621
Orthocladiinae|BIOUG16778-D07|Jerome Moriniere|BIN Taxonomy Match|BOLD:ADV3676
Orthocladiinae|BIOUG16740-B07|Jerome Moriniere|BIN Taxonomy Match|BOLD:ADV8591
Orthocladiinae|BIOUG16854-F12|Jerome Moriniere|BIN Taxonomy Match|BOLD:ADV8591
Orthocladiinae|BIOUG16739-B10|Jerome Moriniere|BIN Taxonomy Match|BOLD:ADV8591
Orthocladius rivulorum|MZ004969|||BOLD:AEK3719

Orthocladiinae|BIOUG16854-F12|Jerome Moriniere|BIN Taxonomy Match|BOLD:ADV8591
Orthocladiinae|BIOUG16739-B10|Jerome Moriniere|BIN Taxonomy Match|BOLD:ADV8591
Orthocladius rivulorum|MZ004969|||BOLD:AEK3719
Orthocladius thienemanni|BIOUG16893-F07|Jerome Moriniere|BIN Taxonomy Match|BOLD:ABW5947
Orthocladius thienemanni|BIOUG16740-D04|Jerome Moriniere|BIN Taxonomy Match|BOLD:ABW5947
Orthocladius thienemanni|BIOUG16856-C03|Jerome Moriniere|BIN Taxonomy Match|BOLD:ABW5947
Orthocladius thienemanni|BIOUG16892-A02|Jerome Moriniere|BIN Taxonomy Match|BOLD:ABW5947
Orthocladius thienemanni|BIOUG16785-F08|Jerome Moriniere|BIN Taxonomy Match|BOLD:ABW5947
Orthocladius thienemanni|BIOUG16785-G12|Jerome Moriniere|BIN Taxonomy Match|BOLD:ABW5947
Orthocladius thienemanni|BIOUG16720-B03|Jerome Moriniere|BIN Taxonomy Match|BOLD:ABW5947
Orthocladius thienemanni|BIOUG16784-B05|Jerome Moriniere|BIN Taxonomy Match|BOLD:ABW5947
Orthocladius thienemanni|BIOUG16785-G06|Jerome Moriniere|BIN Taxonomy Match|BOLD:ABW5947
Orthocladiinae|BI2019_B01|Bart Peeters|BOLD ID-Engine|BOLD:ABW5947
Orthocladiinae|BI2019_A01|Aina Aspaas|BOLD ID-Engine|BOLD:ABW5947
Hydrobaenus conformis|ZMUO.024537|Lauri Paasivirta||BOLD:ADA6732
Hydrobaenus conformis|ZMUO.024536|Lauri Paasivirta||BOLD:ADA6732
Hydrobaenus conformis|Finnmark534|Elisabeth Stur||BOLD:AAQ2644
Hydrobaenus conformis|Finnmark515|Elisabeth Stur||BOLD:AAQ2644
Tvetenia calvescens|EBAI-Ch106|Elisabeth Stur|BOLD Identification Engine|BOLD:AAF6379
Tvetenia calvescens|EBAI-Ch104|Elisabeth Stur|morphology|BOLD:AAF6379
Tvetenia calvescens|EBAI-Ch112|Elisabeth Stur|morphology|BOLD:AAF6379
Tvetenia calvescens|Finnmark876|Elisabeth Stur||BOLD:AAF6379
Tvetenia calvescens|ATNA355|Elisabeth Stur||BOLD:AAF6379
Tvetenia calvescens|EBAI-Ch254|Elisabeth Stur|morphology|BOLD:AAF6379
Tvetenia calvescens|EBAI-Ch109|Elisabeth Stur|BOLD Identification Engine|BOLD:AAF6379
Tvetenia calvescens|EBAI-Ch111|Elisabeth Stur|morphology|BOLD:AAF6379
Tvetenia calvescens|EBAI-Ch206|Elisabeth Stur|morphology|BOLD:AAF6379
Tvetenia calvescens|EBAI-Ch205|Elisabeth Stur|morphology|BOLD:AAF6379
Tvetenia calvescens|ATNA490|Elisabeth Stur||BOLD:AAF6379
Tvetenia calvescens|ATNA489|Elisabeth Stur||BOLD:AAF6379
Tvetenia calvescens|EBAI-Ch102|Elisabeth Stur|BOLD Identification Engine|BOLD:AAF6379
Tvetenia calvescens|EBAI-Ch110|Elisabeth Stur|BOLD Identification Engine|BOLD:AAF6379
Tvetenia calvescens|ATNA486|Elisabeth Stur||BOLD:AAF6379
Tvetenia calvescens|BIOUG16430-D12|Kate Perez|BIN Taxonomy Match|BOLD:AAF6378
Tvetenia calvescens|ZMUO.025278|Lauri Paasivirta||BOLD:AAF6378
Tvetenia calvescens|ZMUO.025277|Lauri Paasivirta||BOLD:AAF6378
Tvetenia calvescens|BIOUG08136-F06|Kate Perez|BIN Taxonomy Match|BOLD:AAF6378
Tvetenia calvescens|Finnmark779|Elisabeth Stur||BOLD:AAF6378
Tvetenia calvescens|ZSM-DIP-33128-F01|Caroline Chimeno|BIN Taxonomy Match (Jan 2022)|BOLD ...
Tvetenia calvescens|ATNA374|Elisabeth Stur||BOLD:AAF6378
Tvetenia calvescens|ATNA264|Elisabeth Stur||BOLD:AAF6378
Tvetenia calvescens|CH-eik90|Elisabeth Stur||BOLD:AAF6378
Tvetenia calvescens|ATNA359|Elisabeth Stur||BOLD:AAF6378
Tvetenia calvescens|ZSM-DIP-33143-F09|Caroline Chimeno|BIN Taxonomy Match (Jan 2022)|BOLD ...
Tvetenia calvescens|PK-192-205|Petra Kranzfelder|morphology|BOLD:AAF6378
Tvetenia calvescens|Finnmark805|G. A. Halvorsen||BOLD:AAF6378
Tvetenia calvescens|Finnmark828|Elisabeth Stur||BOLD:AAF6378
Tvetenia calvescens|Finnmark23|Elisabeth Stur||BOLD:AAF6378
Tvetenia calvescens|Finnmark410|Elisabeth Stur||BOLD:AAF6378
Chironomidae|BIOUG17114-A08|Jerome Moriniere|BIN Taxonomy Match|BOLD:ACR2857
Chironomidae|BIOUG17117-G09|Kate Perez|Tree base identification
Tvetenia discoloripes|ZMUO.024804|Lauri Paasivirta||BOLD:AAF6373
Tvetenia verralli|ATNA360|Elisabeth Stur||BOLD:AAF6373
Tvetenia verralli|ATNA361|Elisabeth Stur||BOLD:AAF6373
Tvetenia verralli|ATNA375|Elisabeth Stur||BOLD:AAF6373
Tvetenia verralli|BIOUG16297-D06|Kate Perez|BIN Taxonomy Match|BOLD:AAF6373
Tvetenia verralli|ZMUO.025279|Lauri Paasivirta||BOLD:AAF6373
Tvetenia verralli|ZMUO.025280|Lauri Paasivirta||BOLD:AAF6373
Tvetenia verralli|Finnmark26|Elisabeth Stur||BOLD:AAF6373
Orthocladius|CCDB24037-E12|Jerome Moriniere|BIN Taxonomy Match|BOLD:ACU5455
Orthocladius|BC-ZSM-DIP-22491-D01|Dieter Doczkal|BIN Taxonomy Match|BOLD:ACU5455
Eukiefferiella cf. minor|ZSM34343-H05|Jerome Moriniere||BOLD:ACU5455
Orthocladius|CCDB24229-H03|Jerome Moriniere|BIN Taxonomy Match|BOLD:ACU5455
Orthocladius|CCDB24039-C11|Jerome Moriniere|BIN Taxonomy Match|BOLD:ACU5455
Orthocladius|CCDB24037-E06|Jerome Moriniere|BIN Taxonomy Match|BOLD:ACU5455
Orthocladius|BC-ZSM-DIP-22491-F06|Dieter Doczkal|BIN Taxonomy Match|BOLD:ACU5455
Orthocladius|BC-ZSM-DIP-22491-G03|Dieter Doczkal|BIN Taxonomy Match|BOLD:ACU5455
Orthocladius|CCDB24039-D03|Jerome Moriniere|BIN Taxonomy Match|BOLD:ACU5455
Orthocladius|BC-ZSM-DIP-22491-D11|Dieter Doczkal|BIN Taxonomy Match|BOLD:ACU5455
Orthocladius|CCDB24229-G06|Jerome Moriniere|BIN Taxonomy Match|BOLD:ACU5455
Orthocladius|CCDB24039-E07|Jerome Moriniere|BIN Taxonomy Match|BOLD:ACU5455
Orthocladius|CCDB24038-E03|Jerome Moriniere|BIN Taxonomy Match|BOLD:ACU5455
Orthocladius|BC-ZSM-DIP-22551-C08|Dieter Doczkal|BIN Taxonomy Match|BOLD:ACU5455
Orthocladius|BC-ZSM-DIP-22491-H06|Dieter Doczkal|BIN Taxonomy Match|BOLD:ACU5455
Orthocladius|CCDB24039-D06|Jerome Moriniere|BIN Taxonomy Match|BOLD:ACU5455
Orthocladius|CCDB24039-D05|Jerome Moriniere|BIN Taxonomy Match|BOLD:ACU5455
Orthocladius|CCDB24039-C10|Jerome Moriniere|BIN Taxonomy Match|BOLD:ACU5455
Orthocladius|CCDB24039-H10|Jerome Moriniere|BIN Taxonomy Match|BOLD:ACU5455
Orthocladius|BC-ZSM-DIP-22551-C11|Dieter Doczkal|BIN Taxonomy Match|BOLD:ACU5455
Orthocladius|BC-ZSM-DIP-22551-D12|Dieter Doczkal|BIN Taxonomy Match|BOLD:ACU5455
Orthocladius|CCDB24039-C12|Jerome Moriniere|BIN Taxonomy Match|BOLD:ACU5455
Orthocladius|CCDB24039-D01|Jerome Moriniere|BIN Taxonomy Match|BOLD:ACU5455
Orthocladius|BC-ZSM-DIP-22491-F08|Dieter Doczkal|BIN Taxonomy Match|BOLD:ACU5455
Orthocladius|BC-ZSM-DIP-22491-F10|Dieter Doczkal|BIN Taxonomy Match|BOLD:ACU5455
Orthocladius|CCDB24037-E05|Jerome Moriniere|BIN Taxonomy Match|BOLD:ACU5455
Orthocladius|CCDB24038-D10|Jerome Moriniere|BIN Taxonomy Match|BOLD:ACU5455
Orthocladius|BC-ZSM-DIP-22551-C05|Dieter Doczkal|BIN Taxonomy Match|BOLD:ACU5455
Orthocladius|BC-ZSM-DIP-22488-F11|Dieter Doczkal|BIN Taxonomy Match|BOLD:ACU5455
Orthocladius|BC-ZSM-DIP-22491-E07|Dieter Doczkal|BIN Taxonomy Match|BOLD:ACU5455
Orthocladius|BC-ZSM-DIP-22491-G08|Dieter Doczkal|BIN Taxonomy Match|BOLD:ACU5455
Orthocladius|BC-ZSM-DIP-22491-E11|Dieter Doczkal|BIN Taxonomy Match|BOLD:ACU5455
Eukiefferiella minor|BIOUG16630-H03|Kate Perez|BIN Taxonomy Match|BOLD:AAE3299
Eukiefferiella minor|BIOUG16713-F09|Kate Perez|BIN Taxonomy Match|BOLD:AAE3299
Eukiefferiella minor|BIOUG16632-G01|Kate Perez|BIN Taxonomy Match|BOLD:AAE3299
Eukiefferiella minor|BIOUG17022-E11|Kate Perez|BIN Taxonomy Match|BOLD:AAE3299
Eukiefferiella minor|BIOUG16712-G05|Kate Perez|BIN Taxonomy Match|BOLD:AAE3299
Eukiefferiella minor|BIOUG16713-A12|Kate Perez|BIN Taxonomy Match|BOLD:AAE3299
Eukiefferiella minor|BIOUG15536-F06|Kate Perez|BIN Taxonomy Match|BOLD:AAE3299
Eukiefferiella minor|BIOUG15600-B07|Kate Perez|BIN Taxonomy Match|BOLD:AAE3299
Eukiefferiella minor|BIOUG15541-C08|Kate Perez|BIN Taxonomy Match|BOLD:AAE3299
Eukiefferiella minor|ATNA131|Elisabeth Stur||BOLD:AAE3299
Eukiefferiella minor|BIOUG16632-D11|Kate Perez|BIN Taxonomy Match|BOLD:AAE3299
Eukiefferiella minor|BIOUG16631-C08|Kate Perez|BIN Taxonomy Match|BOLD:AAE3299

Eukiefferiella minor|ATNA131|Elisabeth Stur||BOLD:AAE3299
Eukiefferiella minor|BIOUG16632-D11|Kate Perez|BIN Taxonomy Match|BOLD:AAE3299
Eukiefferiella minor|BIOUG16631-C08|Kate Perez|BIN Taxonomy Match|BOLD:AAE3299
Eukiefferiella minor|BIOUG17023-E06|Kate Perez|BIN Taxonomy Match|BOLD:AAE3299
Eukiefferiella minor|BIOUG16632-B09|Kate Perez|BIN Taxonomy Match|BOLD:AAE3299
Eukiefferiella minor|BIOUG17022-B07|Kate Perez|BIN Taxonomy Match|BOLD:AAE3299
Eukiefferiella minor|BIOUG16713-D11|Kate Perez|BIN Taxonomy Match|BOLD:AAE3299
Eukiefferiella minor|EBAI-Ch086|Elisabeth Stur|morphology|BOLD:AAE3299
Eukiefferiella minor|BIOUG17022-C07|Kate Perez|BIN Taxonomy Match|BOLD:AAE3299
Eukiefferiella minor|BIOUG15675-G02|Kate Perez|BIN Taxonomy Match|BOLD:AAE3299
Eukiefferiella minor|EBAI-Ch139|Elisabeth Stur|BOLD Identification Engine|BOLD:AAE3299
Eukiefferiella minor|ATNA130|Elisabeth Stur||BOLD:AAE3299
Eukiefferiella minor|ATNA150|Elisabeth Stur||BOLD:AAE3299
Eukiefferiella minor|BIOUG15538-F09|Kate Perez|BIN Taxonomy Match|BOLD:AAE3299
Eukiefferiella minor|BIOUG15596-D09|Kate Perez|BIN Taxonomy Match|BOLD:AAE3299
Eukiefferiella minor|BIOUG15761-F03|Kate Perez|BIN Taxonomy Match|BOLD:AAE3299
Eukiefferiella minor|Finnmark802|Elisabeth Stur||BOLD:AAE3299
Eukiefferiella minor|Finnmark556|Elisabeth Stur||BOLD:AAE3299
Eukiefferiella minor|BIOUG16631-G09|Kate Perez|BIN Taxonomy Match|BOLD:AAE3299
Eukiefferiella minor|BIOUG15463-F10|Kate Perez|BIN Taxonomy Match|BOLD:AAE3299
Eukiefferiella minor|BIOUG15463-F09|Kate Perez|BIN Taxonomy Match|BOLD:AAE3299
Eukiefferiella minor|BIOUG15464-H10|Kate Perez|BIN Taxonomy Match|BOLD:AAE3299
Eukiefferiella minor|BIOUG16713-C10|Kate Perez|BIN Taxonomy Match|BOLD:AAE3299
Eukiefferiella minor|BIOUG15536-G10|Kate Perez|BIN Taxonomy Match|BOLD:AAE3299
Eukiefferiella minor|BIOUG16631-G01|Kate Perez|BIN Taxonomy Match|BOLD:AAE3299
Eukiefferiella minor|BIOUG15536-H05|Kate Perez|BIN Taxonomy Match|BOLD:AAE3299
Eukiefferiella minor|BIOUG15544-B11|Kate Perez|BIN Taxonomy Match|BOLD:AAE3299
Eukiefferiella minor|BIOUG15563-E02|Kate Perez|BIN Taxonomy Match|BOLD:AAE3299
Eukiefferiella minor|BIOUG16632-A10|Kate Perez|BIN Taxonomy Match|BOLD:AAE3299
Eukiefferiella minor|BIOUG15592-D07|Kate Perez|BIN Taxonomy Match|BOLD:AAE3299
Eukiefferiella minor|BIOUG16631-D11|Kate Perez|BIN Taxonomy Match|BOLD:AAE3299
Eukiefferiella minor|BIOUG15463-E08|Kate Perez|BIN Taxonomy Match|BOLD:AAE3299
Eukiefferiella minor|BIOUG17022-B08|Kate Perez|BIN Taxonomy Match|BOLD:AAE3299
Eukiefferiella minor|BIOUG16712-E06|Kate Perez|BIN Taxonomy Match|BOLD:AAE3299
Eukiefferiella minor|BIOUG15537-F10|Kate Perez|BIN Taxonomy Match|BOLD:AAE3299
Eukiefferiella minor|BIOUG15545-F01|Kate Perez|BIN Taxonomy Match|BOLD:AAE3299
Eukiefferiella minor|BIOUG15600-B01|Kate Perez|BIN Taxonomy Match|BOLD:AAE3299
Eukiefferiella minor|BIOUG16631-E11|Kate Perez|BIN Taxonomy Match|BOLD:AAE3299
Eukiefferiella minor|BIOUG16713-F06|Kate Perez|BIN Taxonomy Match|BOLD:AAE3299
Eukiefferiella minor|BIOUG16631-D08|Kate Perez|BIN Taxonomy Match|BOLD:AAE3299
Eukiefferiella minor|BIOUG15465-H04|Kate Perez|BIN Taxonomy Match|BOLD:AAE3299
Eukiefferiella minor|BIOUG15465-A03|Kate Perez|BIN Taxonomy Match|BOLD:AAE3299
Eukiefferiella minor|BIOUG15543-F04|Kate Perez|BIN Taxonomy Match|BOLD:AAE3299
Eukiefferiella minor|BIOUG15563-H11|Kate Perez|BIN Taxonomy Match|BOLD:AAE3299
Eukiefferiella minor|BIOUG15564-A10|Kate Perez|BIN Taxonomy Match|BOLD:AAE3299
Eukiefferiella minor|BIOUG16632-F05|Kate Perez|BIN Taxonomy Match|BOLD:AAE3299
Eukiefferiella minor|BIOUG16712-G06|Kate Perez|BIN Taxonomy Match|BOLD:AAE3299
Eukiefferiella minor|BIOUG15464-C05|Kate Perez|BIN Taxonomy Match|BOLD:AAE3299
Eukiefferiella minor|BIOUG15565-A02|Kate Perez|BIN Taxonomy Match|BOLD:AAE3299
Eukiefferiella minor|BIOUG15564-A07|Kate Perez|BIN Taxonomy Match|BOLD:AAE3299
Eukiefferiella minor|BIOUG16631-C07|Kate Perez|BIN Taxonomy Match|BOLD:AAE3299
Eukiefferiella minor|BIOUG15651-G10|Kate Perez|BIN Taxonomy Match|BOLD:AAE3299
Eukiefferiella minor|BIOUG16626-C10|Kate Perez|BIN Taxonomy Match|BOLD:AAE3299
Eukiefferiella minor|BIOUG15563-C05|Kate Perez|BIN Taxonomy Match|BOLD:AAE3299
Eukiefferiella minor|BIOUG15563-H04|Kate Perez|BIN Taxonomy Match|BOLD:AAE3299
Eukiefferiella minor|BIOUG15545-E06|Kate Perez|BIN Taxonomy Match|BOLD:AAE3299
Eukiefferiella minor|BIOUG15565-F01|Kate Perez|BIN Taxonomy Match|BOLD:AAE3299
Eukiefferiella minor|BIOUG15465-B05|Kate Perez|BIN Taxonomy Match|BOLD:AAE3299
Eukiefferiella minor|BIOUG15565-B06|Kate Perez|BIN Taxonomy Match|BOLD:AAE3299
Eukiefferiella minor|BIOUG15564-C02|Kate Perez|BIN Taxonomy Match|BOLD:AAE3299
Eukiefferiella minor|BIOUG15565-E03|Kate Perez|BIN Taxonomy Match|BOLD:AAE3299
Eukiefferiella minor|BIOUG16711-G04|Kate Perez|BIN Taxonomy Match|BOLD:AAE3299
Eukiefferiella minor|BIOUG15540-F05|Kate Perez|BIN Taxonomy Match|BOLD:AAE3299
Eukiefferiella minor|BIOUG16711-H11|Kate Perez|BIN Taxonomy Match|BOLD:AAE3299
Eukiefferiella minor|BIOUG16711-G10|Kate Perez|BIN Taxonomy Match|BOLD:AAE3299
Eukiefferiella minor|BIOUG15543-H05|Kate Perez|BIN Taxonomy Match|BOLD:AAE3299
Eukiefferiella minor|BIOUG16712-B11|Kate Perez|BIN Taxonomy Match|BOLD:AAE3299
Eukiefferiella minor|BIOUG16712-C11|Kate Perez|BIN Taxonomy Match|BOLD:AAE3299
Eukiefferiella minor|BIOUG16712-G08|Kate Perez|BIN Taxonomy Match|BOLD:AAE3299
Eukiefferiella minor|BIOUG17022-A05|Kate Perez|BIN Taxonomy Match|BOLD:AAE3299
Eukiefferiella minor|BIOUG17022-C12|Kate Perez|BIN Taxonomy Match|BOLD:AAE3299
Eukiefferiella minor|BIOUG17022-F06|Kate Perez|BIN Taxonomy Match|BOLD:AAE3299
Eukiefferiella minor|BIOUG16718-F04|Kate Perez|BIN Taxonomy Match|BOLD:AAE3299
Eukiefferiella minor|BIOUG17022-C09|Kate Perez|BIN Taxonomy Match|BOLD:AAE3299
Eukiefferiella minor|BIOUG17023-B06|Kate Perez|BIN Taxonomy Match|BOLD:AAE3299
Eukiefferiella minor|BIOUG17023-B11|Kate Perez|BIN Taxonomy Match|BOLD:AAE3299
Eukiefferiella minor|BIOUG16713-B09|Kate Perez|BIN Taxonomy Match|BOLD:AAE3299
Eukiefferiella minor|BIOUG16711-C06|Kate Perez|BIN Taxonomy Match|BOLD:AAE3299
Eukiefferiella minor|BIOUG17023-B05|Kate Perez|BIN Taxonomy Match|BOLD:AAE3299
Eukiefferiella minor|BIOUG15537-F06|Kate Perez|BIN Taxonomy Match|BOLD:AAE3299
Eukiefferiella minor|BIOUG17023-A08|Kate Perez|BIN Taxonomy Match|BOLD:AAE3299
Eukiefferiella minor|BIOUG15545-A02|Kate Perez|BIN Taxonomy Match|BOLD:AAE3299
Eukiefferiella minor|BIOUG15676-A09|Kate Perez|BIN Taxonomy Match|BOLD:AAE3299
Eukiefferiella minor|BIOUG16713-C09|Kate Perez|BIN Taxonomy Match|BOLD:AAE3299
Eukiefferiella minor|BIOUG16631-G10|Kate Perez|BIN Taxonomy Match|BOLD:AAE3299
Eukiefferiella minor|BIOUG16712-D10|Kate Perez|BIN Taxonomy Match|BOLD:AAE3299
Eukiefferiella minor|BIOUG17022-G02|Kate Perez|BIN Taxonomy Match|BOLD:AAE3299
Eukiefferiella minor|BIOUG15592-A09|Kate Perez|BIN Taxonomy Match|BOLD:AAE3299
Eukiefferiella minor|BIOUG15591-F10|Kate Perez|BIN Taxonomy Match|BOLD:AAE3299
Eukiefferiella minor|BIOUG15562-F10|Kate Perez|BIN Taxonomy Match|BOLD:AAE3299
Eukiefferiella minor|BIOUG16712-E03|Kate Perez|BIN Taxonomy Match|BOLD:AAE3299
Eukiefferiella minor|BIOUG16631-D03|Kate Perez|BIN Taxonomy Match|BOLD:AAE3299
Eukiefferiella minor|ATNA293|Elisabeth Stur||BOLD:AAE3299
Eukiefferiella minor|EBAI-Ch191|Elisabeth Stur|morphology|BOLD:AAE3299
Eukiefferiella minor|EBAI-Ch190|Elisabeth Stur|morphology
Eukiefferiella minor|ATNA276|Elisabeth Stur||BOLD:AAE3299
Eukiefferiella minor|ATNA242|Elisabeth Stur||BOLD:AAE3299
Eukiefferiella minor|ATNA370|Elisabeth Stur||BOLD:AAE3299
Eukiefferiella minor|BIOUG15539-G03|Kate Perez|BIN Taxonomy Match|BOLD:AAE3299
Eukiefferiella minor|BIOUG15536-A08|Kate Perez|BIN Taxonomy Match|BOLD:AAE3299
Eukiefferiella minor|BIOUG15719-D11|Kate Perez|BIN Taxonomy Match|BOLD:AAE3299
Eukiefferiella minor|BIOUG15764-F08|Kate Perez|BIN Taxonomy Match|BOLD:AAE3299
Eukiefferiella minor|Finnmark570|Elisabeth Stur||BOLD:AAE3299

[illegible]
Eukiefferiella minor|BIOUG15719-D11|Kate Perez|BIN Taxonomy Match|BOLD:AAE3299
Eukiefferiella minor|BIOUG15764-F08|Kate Perez|BIN Taxonomy Match|BOLD:AAE3299
Eukiefferiella minor|Finnmark570|Elisabeth Stur||BOLD:AAE3299
Eukiefferiella devonica|EBAI-Ch140|Elisabeth Stur|BOLD Identification Engine|BOLD:AAE4574
Eukiefferiella devonica|ATNA246|Elisabeth Stur||BOLD:AAE4574
Eukiefferiella devonica|ATNA241|Elisabeth Stur||BOLD:AAE4574
Eukiefferiella devonica|ATNA239|Elisabeth Stur||BOLD:AAE4574
Eukiefferiella devonica|ATNA499|Elisabeth Stur||BOLD:AAE4574
Eukiefferiella dittmari|ZMUO.024705|Lauri Paasivirta||BOLD:AAI5129
Eukiefferiella dittmari|ZMUO.024704|Lauri Paasivirta||BOLD:AAI5129
Eukiefferiella dittmari|EJV-20110187|Lauri Paasivirta||BOLD:AAI5129
Eukiefferiella dittmari|Finnmark194|Elisabeth Stur||BOLD:AAI5129
Eukiefferiella dittmari|EJV-20110186|Lauri Paasivirta||BOLD:AAI5129
Eukiefferiella dittmari|EJV-20110185|Lauri Paasivirta||BOLD:AAI5129
Eukiefferiella dittmari|TRD-CH397|Elisabeth Stur|morphology|BOLD:AAI5129
Eukiefferiella dittmari|ATNA256|Elisabeth Stur||BOLD:AAI5129
Eukiefferiella dittmari|BIOUG16597-E12|Kate Perez|BIN Taxonomy Match|BOLD:AAI5129
Eukiefferiella dittmari|BIOUG15763-F07|Kate Perez|BIN Taxonomy Match|BOLD:AAI5129
Eukiefferiella devonica|ZMUO.024801|Lauri Paasivirta||BOLD:AAI5128
Eukiefferiella ilkleyensis|ATNA348|G. A. Halvorsen||BOLD:AAI5128
Eukiefferiella ilkleyensis|Finnmark843|G. A. Halvorsen||BOLD:AAI5128
Eukiefferiella ilkleyensis|ATNA500|G. A. Halvorsen||BOLD:AAI5128
Eukiefferiella ilkleyensis|ATNA498|G. A. Halvorsen||BOLD:AAI5128
Eukiefferiella ilkleyensis|ATNA513|G. A. Halvorsen||BOLD:AAI5128
Eukiefferiella ilkleyensis|ATNA512|G. A. Halvorsen||BOLD:AAI5128
Eukiefferiella ilkleyensis|ATNA502|G. A. Halvorsen||BOLD:AAI5128
Eukiefferiella ilkleyensis|Finnmark844|G. A. Halvorsen||BOLD:AAI5128
Eukiefferiella ilkleyensis|Finnmark845|G. A. Halvorsen||BOLD:AAI5128
Eukiefferiella ilkleyensis|EBAI-Ch192|Elisabeth Stur|morphology|BOLD:AAI5128
Eukiefferiella ilkleyensis|EBAI-Ch141|Elisabeth Stur|BOLD Identification Engine|BOLD:AAI5128
Eukiefferiella devonica|ZMUO.024800|Lauri Paasivirta||BOLD:AAI5128
Eukiefferiella ilkleyensis|EBAI-Ch084|Elisabeth Stur|morphology|BOLD:AAI5128
Eukiefferiella ilkleyensis|EBAI-Ch085|Elisabeth Stur|morphology|BOLD:AAI5128
Eukiefferiella ilkleyensis|EBAI-Ch193|Elisabeth Stur|morphology|BOLD:AAI5128
Eukiefferiella ilkleyensis|ATNA501|G. A. Halvorsen||BOLD:AAI5128
Eukiefferiella ilkleyensis|ATNA153|G. A. Halvorsen||BOLD:AAI5128
Eukiefferiella ilkleyensis|ATNA162|G. A. Halvorsen||BOLD:AAI5128
Eukiefferiella ilkleyensis|ATNA160|G. A. Halvorsen||BOLD:AAI5128
Eukiefferiella ilkleyensis|ATNA144|G. A. Halvorsen||BOLD:AAI5128
Eukiefferiella ilkleyensis|ATNA497|G. A. Halvorsen||BOLD:AAI5128
Eukiefferiella devonica|BIOUG16859-D06|Jerome Moriniere|BIN Taxonomy Match|BOLD:ACP5613
Eukiefferiella devonica|BIOUG17119-F05|Jerome Moriniere|BIN Taxonomy Match|BOLD:ACP5613
Eukiefferiella|BIOUG15650-G03|Elisabeth Stur|morphology|BOLD:ACP5613
Eukiefferiella|BIOUG15720-F03|Elisabeth Stur|morphology|BOLD:ACP5613
Eukiefferiella|BIOUG15561-E08|Elisabeth Stur|morphology|BOLD:ACP5613
Eukiefferiella|BIOUG15762-C12|Elisabeth Stur|morphology|BOLD:ACP5613
Tokunagaia sp. 1ES|BIOUG15808-B12|Valerie Levesque-Beaudin|BIN Taxonomy Match|BOLD:ACP6976
Tokunagaia sp. 1ES|BIOUG16712-B08|Valerie Levesque-Beaudin|BIN Taxonomy Match|BOLD:ACP6976
Tokunagaia sp. 1ES|BIOUG16713-B10|Valerie Levesque-Beaudin|BIN Taxonomy Match|BOLD:ACP6976
Tokunagaia sp. 1ES|BIOUG16432-F12|Valerie Levesque-Beaudin|BIN Taxonomy Match|BOLD:ACP6976
Tokunagaia sp. 1ES|BIOUG16711-C09|Valerie Levesque-Beaudin|BIN Taxonomy Match|BOLD:ACP6976
Tokunagaia sp. 1ES|BIOUG16594-F06|Valerie Levesque-Beaudin|BIN Taxonomy Match|BOLD:ACP6976
Tokunagaia sp. 1ES|BIOUG15765-G04|Valerie Levesque-Beaudin|BIN Taxonomy Match|BOLD:ACP6976
Tokunagaia sp. 1ES|BIOUG15761-H06|Valerie Levesque-Beaudin|BIN Taxonomy Match|BOLD:ACP6976
Tokunagaia sp. 1ES|BIOUG16712-A01|Valerie Levesque-Beaudin|BIN Taxonomy Match|BOLD:ACP6976
Tokunagaia sp. 1ES|BIOUG16711-E02|Valerie Levesque-Beaudin|BIN Taxonomy Match|BOLD:ACP6976
Tokunagaia sp. 1ES|BIOUG16630-H06|Valerie Levesque-Beaudin|BIN Taxonomy Match|BOLD:ACP6976
Tokunagaia sp. 1ES|BIOUG15719-D04|Valerie Levesque-Beaudin|BIN Taxonomy Match|BOLD:ACP6976
Tokunagaia sp. 1ES|BIOUG15800-E12|Valerie Levesque-Beaudin|BIN Taxonomy Match|BOLD:ACP6976
Tokunagaia sp. 1ES|BIOUG16476-G12|Valerie Levesque-Beaudin|BIN Taxonomy Match|BOLD:ACP6976
Tokunagaia sp. 1ES|BIOUG16632-F10|Valerie Levesque-Beaudin|BIN Taxonomy Match|BOLD:ACP6976
Tokunagaia sp. 1ES|BIOUG16477-F04|Valerie Levesque-Beaudin|BIN Taxonomy Match|BOLD:ACP6976
Tokunagaia sp. 1ES|BIOUG15853-H08|Valerie Levesque-Beaudin|BIN Taxonomy Match|BOLD:ACP6976
Tokunagaia sp. 1ES|BIOUG16435-E10|Valerie Levesque-Beaudin|BIN Taxonomy Match|BOLD:ACP6976
Tokunagaia sp. 1ES|BIOUG16205-B07|Valerie Levesque-Beaudin|BIN Taxonomy Match|BOLD:ACP6976
Tokunagaia sp. 1ES|BIOUG16330-F06|Valerie Levesque-Beaudin|BIN Taxonomy Match|BOLD:ACP6976
Tokunagaia sp. 1ES|BIOUG15719-A09|Valerie Levesque-Beaudin|BIN Taxonomy Match|BOLD:ACP6976
Tokunagaia sp. 1ES|BIOUG15761-F10|Valerie Levesque-Beaudin|BIN Taxonomy Match|BOLD:ACP6976
Tokunagaia sp. 1ES|BIOUG15794-A05|Valerie Levesque-Beaudin|BIN Taxonomy Match|BOLD:ACP6976
Tokunagaia sp. 1ES|BIOUG16625-D10|Valerie Levesque-Beaudin|BIN Taxonomy Match|BOLD:ACP6976
Tokunagaia sp. 1ES|BIOUG17022-F09|Valerie Levesque-Beaudin|BIN Taxonomy Match|BOLD:ACP6976
Tokunagaia sp. 1ES|BIOUG15719-C06|Valerie Levesque-Beaudin|BIN Taxonomy Match|BOLD:ACP6976
Tokunagaia sp. 1ES|BIOUG16711-E11|Valerie Levesque-Beaudin|BIN Taxonomy Match|BOLD:ACP6976
Tokunagaia sp. 1ES|BIOUG15764-G10|Valerie Levesque-Beaudin|BIN Taxonomy Match|BOLD:ACP6976
Tokunagaia sp. 1ES|BIOUG15794-B04|Valerie Levesque-Beaudin|BIN Taxonomy Match|BOLD:ACP6976
Tokunagaia sp. 1ES|BIOUG15720-H07|Valerie Levesque-Beaudin|BIN Taxonomy Match|BOLD:ACP6976
Tokunagaia sp. 1ES|BIOUG16631-E07|Valerie Levesque-Beaudin|BIN Taxonomy Match|BOLD:ACP6976
Tokunagaia sp. 1ES|BIOUG16713-A10|Valerie Levesque-Beaudin|BIN Taxonomy Match|BOLD:ACP6976
Tokunagaia sp. 1ES|BIOUG15766-A12|Valerie Levesque-Beaudin|BIN Taxonomy Match|BOLD:ACP6976
Tokunagaia sp. 1ES|BIOUG15764-G06|Valerie Levesque-Beaudin|BIN Taxonomy Match|BOLD:ACP6976
Tokunagaia sp. 1ES|BIOUG16631-G03|Elisabeth Stur|morphology|BOLD:ACP6976
Tokunagaia sp. 1ES|BIOUG15762-A12|Valerie Levesque-Beaudin|BIN Taxonomy Match|BOLD:ACP6976
Tokunagaia sp. 1ES|BIOUG15763-A06|Valerie Levesque-Beaudin|BIN Taxonomy Match|BOLD:ACP6976
Tokunagaia sp. 1ES|BIOUG15718-E09|Elisabeth Stur|morphology|BOLD:ACP6976
Tokunagaia sp. 1ES|BIOUG15762-B07|Valerie Levesque-Beaudin|BIN Taxonomy Match|BOLD:ACP6976
Tokunagaia sp. 1ES|BIOUG15949-G02|Valerie Levesque-Beaudin|BIN Taxonomy Match|BOLD:ACP6976
Tokunagaia sp. 1ES|BIOUG16597-D10|Valerie Levesque-Beaudin|BIN Taxonomy Match|BOLD:ACP6976
Tokunagaia sp. 1ES|BIOUG16711-C08|Valerie Levesque-Beaudin|BIN Taxonomy Match|BOLD:ACP6976
Tokunagaia sp. 1ES|BIOUG17022-D05|Valerie Levesque-Beaudin|BIN Taxonomy Match|BOLD:ACP6976
Tokunagaia sp. 1ES|BIOUG15720-G11|Valerie Levesque-Beaudin|BIN Taxonomy Match|BOLD:ACP6976
Tokunagaia sp. 1ES|BIOUG16711-C01|Valerie Levesque-Beaudin|BIN Taxonomy Match|BOLD:ACP6976
Tokunagaia sp. 1ES|BIOUG15793-A02|Valerie Levesque-Beaudin|BIN Taxonomy Match|BOLD:ACP6976
Tokunagaia sp. 1ES|BIOUG16712-D07|Valerie Levesque-Beaudin|BIN Taxonomy Match|BOLD:ACP6976
Tokunagaia sp. 1ES|BIOUG15762-D02|Valerie Levesque-Beaudin|BIN Taxonomy Match|BOLD:ACP6976
Tokunagaia sp. 1ES|BIOUG15762-A07|Valerie Levesque-Beaudin|BIN Taxonomy Match|BOLD:ACP6976
Tokunagaia sp. 1ES|BIOUG15720-E03|Valerie Levesque-Beaudin|BIN Taxonomy Match|BOLD:ACP6976
Tokunagaia sp. 1ES|BIOUG15764-D04|Valerie Levesque-Beaudin|BIN Taxonomy Match|BOLD:ACP6976
Tokunagaia sp. 1ES|BIOUG16711-B05|Valerie Levesque-Beaudin|BIN Taxonomy Match|BOLD:ACP6976
Tokunagaia sp. 1ES|BIOUG16711-F03|Valerie Levesque-Beaudin|BIN Taxonomy Match|BOLD:ACP6976
Tokunagaia sp. 1ES|BIOUG16631-H09|Valerie Levesque-Beaudin|BIN Taxonomy Match|BOLD:ACP6976
Tokunagaia sp. 1ES|BIOUG15720-G03|Valerie Levesque-Beaudin|BIN Taxonomy Match|BOLD:ACP6976
Tokunagaia sp. 1ES|BIOUG16711-C12|Valerie Levesque-Beaudin|BIN Taxonomy Match|BOLD:ACP6976
[illegible]

Tokunagaia sp. 1ES|BIOUG16631-H09|Valerie Levesque-Beaudin|BIN Taxonomy Match|BOLD:ACP6976
Tokunagaia sp. 1ES|BIOUG15720-G03|Valerie Levesque-Beaudin|BIN Taxonomy Match|BOLD:ACP6976
Tokunagaia sp. 1ES|BIOUG16711-C12|Valerie Levesque-Beaudin|BIN Taxonomy Match|BOLD:ACP6976
Tokunagaia sp. 1ES|BIOUG16552-A07|Valerie Levesque-Beaudin|BIN Taxonomy Match|BOLD:ACP6976
Tokunagaia sp. 1ES|BIOUG15766-H01|Valerie Levesque-Beaudin|BIN Taxonomy Match|BOLD:ACP6976
Tokunagaia sp. 1ES|BIOUG15794-G02|Valerie Levesque-Beaudin|BIN Taxonomy Match|BOLD:ACP6976
Tokunagaia sp. 1ES|BIOUG15761-B10|Valerie Levesque-Beaudin|BIN Taxonomy Match|BOLD:ACP6976
Tokunagaia sp. 1ES|BIOUG16713-G11|Valerie Levesque-Beaudin|BIN Taxonomy Match|BOLD:ACP6976
Tokunagaia sp. 1ES|BIOUG15765-B12|Valerie Levesque-Beaudin|BIN Taxonomy Match|BOLD:ACP6976
Tokunagaia sp. 1ES|BIOUG15761-A04|Valerie Levesque-Beaudin|BIN Taxonomy Match|BOLD:ACP6976
Tokunagaia sp. 1ES|BIOUG16632-D08|Valerie Levesque-Beaudin|BIN Taxonomy Match|BOLD:ACP6976
Tokunagaia sp. 1ES|BIOUG15720-F04|Valerie Levesque-Beaudin|BIN Taxonomy Match|BOLD:ACP6976
Tokunagaia sp. 1ES|BIOUG15761-E02|Valerie Levesque-Beaudin|BIN Taxonomy Match|BOLD:ACP6976
Tokunagaia sp. 1ES|BIOUG16297-E03|Valerie Levesque-Beaudin|BIN Taxonomy Match|BOLD:ACP6976
Tokunagaia sp. 1ES|BIOUG15763-B11|Valerie Levesque-Beaudin|BIN Taxonomy Match|BOLD:ACP6976
Tokunagaia sp. 1ES|BIOUG15718-H03|Valerie Levesque-Beaudin|BIN Taxonomy Match|BOLD:ACP6976
Tokunagaia sp. 1ES|BIOUG15718-A10|Elisabeth Stur|morphology|BOLD:ACP6976
Tokunagaia sp. 1ES|BIOUG15765-G05|Valerie Levesque-Beaudin|BIN Taxonomy Match|BOLD:ACP6976
Tokunagaia sp. 1ES|BIOUG15718-C02|Elisabeth Stur|BIN Taxonomy Match|BOLD:ACP6976
Tokunagaia sp. 1ES|BIOUG15763-D06|Valerie Levesque-Beaudin|BIN Taxonomy Match|BOLD:ACP6976
Tokunagaia sp. 1ES|BIOUG15765-F10|Valerie Levesque-Beaudin|BIN Taxonomy Match|BOLD:ACP6976
Tokunagaia sp. 1ES|BIOUG16712-H03|Valerie Levesque-Beaudin|BIN Taxonomy Match|BOLD:ACP6976
Tokunagaia sp. 1ES|BIOUG15766-D05|Valerie Levesque-Beaudin|BIN Taxonomy Match|BOLD:ACP6976
Tokunagaia sp. 1ES|BIOUG15718-A08|Elisabeth Stur|morphology|BOLD:ACP6976
Tokunagaia sp. 1ES|BIOUG16632-A08|Valerie Levesque-Beaudin|BIN Taxonomy Match|BOLD:ACP6976
Tokunagaia sp. 1ES|BIOUG15720-C01|Valerie Levesque-Beaudin|BIN Taxonomy Match|BOLD:ACP6976
Tokunagaia sp. 1ES|BIOUG16207-F07|Valerie Levesque-Beaudin|BIN Taxonomy Match|BOLD:ACP6976
Tokunagaia sp. 1ES|BIOUG16632-E05|Valerie Levesque-Beaudin|BIN Taxonomy Match|BOLD:ACP6976
Tokunagaia sp. 1ES|BIOUG15761-G02|Valerie Levesque-Beaudin|BIN Taxonomy Match|BOLD:ACP6976
Tokunagaia sp. 1ES|BIOUG16632-H11|Valerie Levesque-Beaudin|BIN Taxonomy Match|BOLD:ACP6976
Tokunagaia sp. 1ES|BIOUG16713-A08|Valerie Levesque-Beaudin|BIN Taxonomy Match|BOLD:ACP6976
Tokunagaia sp. 1ES|BIOUG15765-H05|Valerie Levesque-Beaudin|BIN Taxonomy Match|BOLD:ACP6976
Tokunagaia sp. 1ES|BIOUG15719-C05|Valerie Levesque-Beaudin|BIN Taxonomy Match|BOLD:ACP6976
Tokunagaia sp. 1ES|BIOUG16632-G03|Valerie Levesque-Beaudin|BIN Taxonomy Match|BOLD:ACP6976
Tokunagaia sp. 1ES|BIOUG16632-F02|Valerie Levesque-Beaudin|BIN Taxonomy Match|BOLD:ACP6976
Tokunagaia sp. 1ES|BIOUG15800-C08|Valerie Levesque-Beaudin|BIN Taxonomy Match|BOLD:ACP6976
Tokunagaia sp. 1ES|BIOUG15769-E01|Valerie Levesque-Beaudin|BIN Taxonomy Match|BOLD:ACP6976
Tokunagaia sp. 1ES|BIOUG15764-C10|Valerie Levesque-Beaudin|BIN Taxonomy Match|BOLD:ACP6976
Tokunagaia sp. 1ES|BIOUG16713-G06|Valerie Levesque-Beaudin|BIN Taxonomy Match|BOLD:ACP6976
Tokunagaia sp. 1ES|BIOUG15762-A10|Valerie Levesque-Beaudin|BIN Taxonomy Match|BOLD:ACP6976
Tokunagaia sp. 1ES|BIOUG16552-C07|Valerie Levesque-Beaudin|BIN Taxonomy Match|BOLD:ACP6976
Tokunagaia sp. 1ES|BIOUG15720-G07|Valerie Levesque-Beaudin|BIN Taxonomy Match|BOLD:ACP6976
Tokunagaia sp. 1ES|BIOUG15719-D07|Valerie Levesque-Beaudin|BIN Taxonomy Match|BOLD:ACP6976
Tokunagaia sp. 1ES|BIOUG15853-B11|Valerie Levesque-Beaudin|BIN Taxonomy Match|BOLD:ACP6976
Tokunagaia sp. 1ES|BIOUG15761-B07|Valerie Levesque-Beaudin|BIN Taxonomy Match|BOLD:ACP6976
Tokunagaia sp. 1ES|BIOUG15720-A05|Valerie Levesque-Beaudin|BIN Taxonomy Match|BOLD:ACP6976
Tokunagaia sp. 1ES|BIOUG15807-B04|Valerie Levesque-Beaudin|BIN Taxonomy Match|BOLD:ACP6976
Tokunagaia sp. 1ES|BIOUG15765-G11|Valerie Levesque-Beaudin|BIN Taxonomy Match|BOLD:ACP6976
Tokunagaia sp. 1ES|BIOUG15762-E11|Valerie Levesque-Beaudin|BIN Taxonomy Match|BOLD:ACP6976
Tokunagaia sp. 1ES|BIOUG15762-E09|Valerie Levesque-Beaudin|BIN Taxonomy Match|BOLD:ACP6976
Tokunagaia sp. 1ES|BIOUG15762-B12|Valerie Levesque-Beaudin|BIN Taxonomy Match|BOLD:ACP6976
Tokunagaia sp. 1ES|BIOUG15761-G08|Valerie Levesque-Beaudin|BIN Taxonomy Match|BOLD:ACP6976
Tokunagaia sp. 1ES|BIOUG15797-A12|Valerie Levesque-Beaudin|BIN Taxonomy Match|BOLD:ACP6976
Tokunagaia sp. 1ES|BIOUG15796-C07|Valerie Levesque-Beaudin|BIN Taxonomy Match|BOLD:ACP6976
Tokunagaia sp. 1ES|BIOUG15764-D02|Valerie Levesque-Beaudin|BIN Taxonomy Match|BOLD:ACP6976
Tokunagaia sp. 1ES|BIOUG15855-D10|Valerie Levesque-Beaudin|BIN Taxonomy Match|BOLD:ACP6976
Tokunagaia sp. 1ES|BIOUG16630-H05|Valerie Levesque-Beaudin|BIN Taxonomy Match|BOLD:ACP6976
Tokunagaia sp. 1ES|BIOUG16713-B08|Valerie Levesque-Beaudin|BIN Taxonomy Match|BOLD:ACP6976
Tokunagaia sp. 1ES|BIOUG16631-C05|Elisabeth Stur|morphology|BOLD:ACP6976
Tokunagaia sp. 1ES|BIOUG16631-E12|Valerie Levesque-Beaudin|BIN Taxonomy Match|BOLD:ACP6976
Tokunagaia sp. 1ES|BIOUG16711-F10|Valerie Levesque-Beaudin|BIN Taxonomy Match|BOLD:ACP6976
Tokunagaia sp. 1ES|BIOUG16711-H08|Valerie Levesque-Beaudin|BIN Taxonomy Match|BOLD:ACP6976
Tokunagaia sp. 1ES|BIOUG16712-B03|Valerie Levesque-Beaudin|BIN Taxonomy Match|BOLD:ACP6976
Tokunagaia sp. 1ES|BIOUG16712-H08|Valerie Levesque-Beaudin|BIN Taxonomy Match|BOLD:ACP6976
Tokunagaia sp. 1ES|BIOUG16712-B04|Valerie Levesque-Beaudin|BIN Taxonomy Match|BOLD:ACP6976
Tokunagaia sp. 1ES|BIOUG16630-H09|Valerie Levesque-Beaudin|BIN Taxonomy Match|BOLD:ACP6976
Tokunagaia sp. 1ES|BIOUG16632-C04|Valerie Levesque-Beaudin|BIN Taxonomy Match|BOLD:ACP6976
Tokunagaia sp. 1ES|BIOUG16632-D12|Valerie Levesque-Beaudin|BIN Taxonomy Match|BOLD:ACP6976
Tokunagaia sp. 1ES|BIOUG16632-E01|Valerie Levesque-Beaudin|BIN Taxonomy Match|BOLD:ACP6976
Tokunagaia sp. 1ES|BIOUG15766-H05|Valerie Levesque-Beaudin|BIN Taxonomy Match|BOLD:ACP6976
Tokunagaia sp. 1ES|BIOUG16257-A10|Valerie Levesque-Beaudin|BIN Taxonomy Match|BOLD:ACP6976
Tokunagaia sp. 1ES|BIOUG16433-B08|Valerie Levesque-Beaudin|BIN Taxonomy Match|BOLD:ACP6976
Tokunagaia sp. 1ES|BIOUG15766-E12|Valerie Levesque-Beaudin|BIN Taxonomy Match|BOLD:ACP6976
Tokunagaia sp. 1ES|BIOUG15762-B11|Valerie Levesque-Beaudin|BIN Taxonomy Match|BOLD:ACP6976
Tokunagaia sp. 1ES|BIOUG16630-H04|Valerie Levesque-Beaudin|BIN Taxonomy Match|BOLD:ACP6976
Tokunagaia sp. 1ES|BIOUG16631-E06|Valerie Levesque-Beaudin|BIN Taxonomy Match|BOLD:ACP6976
Tokunagaia sp. 1ES|BIOUG15765-D08|Valerie Levesque-Beaudin|BIN Taxonomy Match|BOLD:ACP6976
Tokunagaia sp. 1ES|BIOUG17022-B10|Valerie Levesque-Beaudin|BIN Taxonomy Match|BOLD:ACP6976
Tokunagaia sp. 1ES|BIOUG15720-C05|Valerie Levesque-Beaudin|BIN Taxonomy Match|BOLD:ACP6976
Tokunagaia sp. 1ES|BIOUG16436-F01|Valerie Levesque-Beaudin|BIN Taxonomy Match|BOLD:ACP6976
Tokunagaia sp. 1ES|BIOUG16631-E03|Valerie Levesque-Beaudin|BIN Taxonomy Match|BOLD:ACP6976
Tokunagaia sp. 1ES|BIOUG15794-D08|Valerie Levesque-Beaudin|BIN Taxonomy Match|BOLD:ACP6976
Tokunagaia sp. 1ES|BIOUG15763-F12|Valerie Levesque-Beaudin|BIN Taxonomy Match|BOLD:ACP6976
Tokunagaia sp. 1ES|BIOUG15763-H03|Valerie Levesque-Beaudin|BIN Taxonomy Match|BOLD:ACP6976
Tokunagaia sp. 1ES|BIOUG15719-F03|Valerie Levesque-Beaudin|BIN Taxonomy Match|BOLD:ACP6976
Tokunagaia sp. 1ES|BIOUG15720-A10|Valerie Levesque-Beaudin|BIN Taxonomy Match|BOLD:ACP6976
Tokunagaia sp. 1ES|BIOUG15720-B06|Valerie Levesque-Beaudin|BIN Taxonomy Match|BOLD:ACP6976
Tokunagaia sp. 1ES|BIOUG15720-H06|Valerie Levesque-Beaudin|BIN Taxonomy Match|BOLD:ACP6976
Tokunagaia sp. 1ES|BIOUG15761-C09|Valerie Levesque-Beaudin|BIN Taxonomy Match|BOLD:ACP6976
Tokunagaia sp. 1ES|BIOUG15761-D05|Valerie Levesque-Beaudin|BIN Taxonomy Match|BOLD:ACP6976
Tokunagaia sp. 1ES|BIOUG15761-E05|Valerie Levesque-Beaudin|BIN Taxonomy Match|BOLD:ACP6976
Tokunagaia sp. 1ES|BIOUG15761-G04|Valerie Levesque-Beaudin|BIN Taxonomy Match|BOLD:ACP6976
Tokunagaia sp. 1ES|BIOUG15761-G11|Valerie Levesque-Beaudin|BIN Taxonomy Match|BOLD:ACP6976
Tokunagaia sp. 1ES|BIOUG15762-F01|Valerie Levesque-Beaudin|BIN Taxonomy Match|BOLD:ACP6976
Tokunagaia sp. 1ES|BIOUG15765-D10|Valerie Levesque-Beaudin|BIN Taxonomy Match|BOLD:ACP6976
Tokunagaia sp. 1ES|BIOUG16330-B06|Valerie Levesque-Beaudin|BIN Taxonomy Match|BOLD:ACP6976
Tokunagaia sp. 1ES|BIOUG15798-E06|Valerie Levesque-Beaudin|BIN Taxonomy Match|BOLD:ACP6976
Tokunagaia sp. 1ES|BIOUG15966-D11|Valerie Levesque-Beaudin|BIN Taxonomy Match|BOLD:ACP6976
Tokunagaia sp. 1ES|BIOUG15800-D01|Valerie Levesque-Beaudin|BIN Taxonomy Match|BOLD:ACP6976
Tokunagaia sp. 1ES|BIOUG16333-F09|Valerie Levesque-Beaudin|BIN Taxonomy Match|BOLD:ACP6976
Tokunagaia sp. 1ES|BIOUG15964-H03|Valerie Levesque-Beaudin|BIN Taxonomy Match|BOLD:ACP6976
Tokunagaia sp. 1ES|BIOUG15809-H03|Valerie Levesque-Beaudin|BIN Taxonomy Match|BOLD:ACP6976
Tokunagaia sp. 1ES|BIOUG16552-B04|Valerie Levesque-Beaudin|BIN Taxonomy Match|BOLD:ACP6976

Tokunagaia sp. 1ES|BIOUG15964-H03|Valerie Levesque-Beaudin|BIN Taxonomy Match|BOLD:ACP6976
Tokunagaia sp. 1ES|BIOUG15809-H03|Valerie Levesque-Beaudin|BIN Taxonomy Match|BOLD:ACP6976
Tokunagaia sp. 1ES|BIOUG16552-B04|Valerie Levesque-Beaudin|BIN Taxonomy Match|BOLD:ACP6976
Tokunagaia sp. 1ES|BIOUG16396-A08|Valerie Levesque-Beaudin|BIN Taxonomy Match|BOLD:ACP6976
Tokunagaia sp. 1ES|BIOUG16713-B12|Valerie Levesque-Beaudin|BIN Taxonomy Match|BOLD:ACP6976
Tokunagaia sp. 1ES|BIOUG16713-E01|Valerie Levesque-Beaudin|BIN Taxonomy Match|BOLD:ACP6976
Tokunagaia sp. 1ES|BIOUG16713-E02|Valerie Levesque-Beaudin|BIN Taxonomy Match|BOLD:ACP6976
Tokunagaia sp. 1ES|BIOUG16713-E06|Valerie Levesque-Beaudin|BIN Taxonomy Match|BOLD:ACP6976
Tokunagaia sp. 1ES|BIOUG16713-F02|Valerie Levesque-Beaudin|BIN Taxonomy Match|BOLD:ACP6976
Tokunagaia sp. 1ES|BIOUG16713-F08|Valerie Levesque-Beaudin|BIN Taxonomy Match|BOLD:ACP6976
Tokunagaia sp. 1ES|BIOUG16631-F11|Valerie Levesque-Beaudin|BIN Taxonomy Match|BOLD:ACP6976
Tokunagaia sp. 1ES|BIOUG16711-D09|Valerie Levesque-Beaudin|BIN Taxonomy Match|BOLD:ACP6976
Tokunagaia sp. 1ES|BIOUG16712-E11|Valerie Levesque-Beaudin|BIN Taxonomy Match|BOLD:ACP6976
Tokunagaia sp. 1ES|BIOUG16712-G10|Valerie Levesque-Beaudin|BIN Taxonomy Match|BOLD:ACP6976
Tokunagaia sp. 1ES|BIOUG16712-H01|Valerie Levesque-Beaudin|BIN Taxonomy Match|BOLD:ACP6976
Tokunagaia sp. 1ES|BIOUG17022-B03|Valerie Levesque-Beaudin|BIN Taxonomy Match|BOLD:ACP6976
Tokunagaia sp. 1ES|BIOUG16632-E11|Valerie Levesque-Beaudin|BIN Taxonomy Match|BOLD:ACP6976
Tokunagaia sp. 1ES|BIOUG15853-H04|Valerie Levesque-Beaudin|BIN Taxonomy Match|BOLD:ACP6976
Tokunagaia sp. 1ES|BIOUG16712-A08|Valerie Levesque-Beaudin|BIN Taxonomy Match|BOLD:ACP6976
Tokunagaia sp. 1ES|BIOUG15769-D07|Valerie Levesque-Beaudin|BIN Taxonomy Match|BOLD:ACP6976
Tokunagaia sp. 1ES|BIOUG15761-E09|Valerie Levesque-Beaudin|BIN Taxonomy Match|BOLD:ACP6976
Tokunagaia sp. 1ES|BIOUG15761-A05|Valerie Levesque-Beaudin|BIN Taxonomy Match|BOLD:ACP6976
Tokunagaia sp. 1ES|BIOUG15763-D12|Valerie Levesque-Beaudin|BIN Taxonomy Match|BOLD:ACP6976
Tokunagaia sp. 1ES|BIOUG17022-C08|Valerie Levesque-Beaudin|BIN Taxonomy Match|BOLD:ACP6976
Tokunagaia sp. 1ES|BIOUG16476-C01|Valerie Levesque-Beaudin|BIN Taxonomy Match|BOLD:ACP6976
Tokunagaia sp. 1ES|BIOUG15764-H01|Valerie Levesque-Beaudin|BIN Taxonomy Match|BOLD:ACP6976
Tokunagaia sp. 1ES|BIOUG15763-G10|Valerie Levesque-Beaudin|BIN Taxonomy Match|BOLD:ACP6976
Tokunagaia sp. 1ES|BIOUG16631-D12|Valerie Levesque-Beaudin|BIN Taxonomy Match|BOLD:ACP6976
Tokunagaia sp. 1ES|BIOUG16631-G02|Valerie Levesque-Beaudin|BIN Taxonomy Match|BOLD:ACP6976
Tokunagaia sp. 1ES|BIOUG16631-G05|Valerie Levesque-Beaudin|BIN Taxonomy Match|BOLD:ACP6976
Tokunagaia sp. 1ES|BIOUG16632-F07|Valerie Levesque-Beaudin|BIN Taxonomy Match|BOLD:ACP6976
Tokunagaia sp. 1ES|BIOUG15718-B06|Elisabeth Stur|morphology|BOLD:ACP6976
Tokunagaia sp. 1ES|BIOUG15718-D06|Elisabeth Stur|morphology|BOLD:ACP6976
Tokunagaia sp. 1ES|BIOUG15718-E03|Elisabeth Stur|morphology|BOLD:ACP6976
Tokunagaia sp. 1ES|BIOUG15761-A03|Valerie Levesque-Beaudin|BIN Taxonomy Match|BOLD:ACP6976
Tokunagaia sp. 1ES|BIOUG15796-C01|Valerie Levesque-Beaudin|BIN Taxonomy Match|BOLD:ACP6976
Tokunagaia sp. 1ES|BIOUG16631-A08|Valerie Levesque-Beaudin|BIN Taxonomy Match|BOLD:ACP6976
Tokunagaia sp. 1ES|BIOUG16631-H08|Valerie Levesque-Beaudin|BIN Taxonomy Match|BOLD:ACP6976
Tokunagaia sp. 1ES|BIOUG16713-A03|Valerie Levesque-Beaudin|BIN Taxonomy Match|BOLD:ACP6976
Tokunagaia sp. 1ES|BIOUG16713-B03|Valerie Levesque-Beaudin|BIN Taxonomy Match|BOLD:ACP6976
Tokunagaia sp. 1ES|BIOUG16476-E10|Valerie Levesque-Beaudin|BIN Taxonomy Match|BOLD:ACP6976
Tokunagaia sp. 1ES|BIOUG16631-D02|Valerie Levesque-Beaudin|BIN Taxonomy Match|BOLD:ACP6976
Tokunagaia sp. 1ES|BIOUG16631-F12|Valerie Levesque-Beaudin|BIN Taxonomy Match|BOLD:ACP6976
Tokunagaia sp. 1ES|BIOUG16632-B03|Valerie Levesque-Beaudin|BIN Taxonomy Match|BOLD:ACP6976
Tokunagaia sp. 1ES|BIOUG16632-B04|Valerie Levesque-Beaudin|BIN Taxonomy Match|BOLD:ACP6976
Tokunagaia sp. 1ES|BIOUG16632-F11|Valerie Levesque-Beaudin|BIN Taxonomy Match|BOLD:ACP6976
Tokunagaia sp. 1ES|BIOUG16711-C11|Valerie Levesque-Beaudin|BIN Taxonomy Match|BOLD:ACP6976
Tokunagaia sp. 1ES|BIOUG15720-E01|Valerie Levesque-Beaudin|BIN Taxonomy Match|BOLD:ACP6976
Tokunagaia sp. 1ES|BIOUG15766-F07|Valerie Levesque-Beaudin|BIN Taxonomy Match|BOLD:ACP6976
Tokunagaia sp. 1ES|BIOUG15950-C12|Valerie Levesque-Beaudin|BIN Taxonomy Match|BOLD:ACP6976
Tokunagaia sp. 1ES|BIOUG16713-F10|Valerie Levesque-Beaudin|BIN Taxonomy Match|BOLD:ACP6976
Tokunagaia sp. 1ES|BIOUG16631-F04|Valerie Levesque-Beaudin|BIN Taxonomy Match|BOLD:ACP6976
Tokunagaia sp. 1ES|BIOUG15765-E11|Valerie Levesque-Beaudin|BIN Taxonomy Match|BOLD:ACP6976
Tokunagaia sp. 1ES|BIOUG15765-D07|Valerie Levesque-Beaudin|BIN Taxonomy Match|BOLD:ACP6976
Tokunagaia sp. 1ES|BIOUG15762-C01|Valerie Levesque-Beaudin|BIN Taxonomy Match|BOLD:ACP6976
Tokunagaia sp. 1ES|BIOUG15761-F05|Valerie Levesque-Beaudin|BIN Taxonomy Match|BOLD:ACP6976
Tokunagaia sp. 1ES|BIOUG15761-D12|Valerie Levesque-Beaudin|BIN Taxonomy Match|BOLD:ACP6976
Tokunagaia sp. 1ES|BIOUG15761-D08|Valerie Levesque-Beaudin|BIN Taxonomy Match|BOLD:ACP6976
Tokunagaia sp. 1ES|BIOUG15720-E09|Valerie Levesque-Beaudin|BIN Taxonomy Match|BOLD:ACP6976
Tokunagaia sp. 1ES|BIOUG15720-A06|Valerie Levesque-Beaudin|BIN Taxonomy Match|BOLD:ACP6976
Tokunagaia sp. 1ES|BIOUG15719-G02|Valerie Levesque-Beaudin|BIN Taxonomy Match|BOLD:ACP6976
Tokunagaia sp. 1ES|BIOUG15719-G01|Valerie Levesque-Beaudin|BIN Taxonomy Match|BOLD:ACP6976
Tokunagaia sp. 1ES|BIOUG15719-F07|Valerie Levesque-Beaudin|BIN Taxonomy Match|BOLD:ACP6976
Tokunagaia sp. 1ES|BIOUG15764-F12|Valerie Levesque-Beaudin|BIN Taxonomy Match|BOLD:ACP6976
Tokunagaia sp. 1ES|BIOUG15764-D03|Valerie Levesque-Beaudin|BIN Taxonomy Match|BOLD:ACP6976
Tokunagaia sp. 1ES|BIOUG15763-E04|Valerie Levesque-Beaudin|BIN Taxonomy Match|BOLD:ACP6976
Tokunagaia sp. 1ES|BIOUG15763-D02|Valerie Levesque-Beaudin|BIN Taxonomy Match|BOLD:ACP6976
Tokunagaia sp. 1ES|BIOUG15763-C12|Valerie Levesque-Beaudin|BIN Taxonomy Match|BOLD:ACP6976
Tokunagaia sp. 1ES|BIOUG15763-B08|Valerie Levesque-Beaudin|BIN Taxonomy Match|BOLD:ACP6976
Tokunagaia sp. 1ES|BIOUG15763-B03|Valerie Levesque-Beaudin|BIN Taxonomy Match|BOLD:ACP6976
Tokunagaia sp. 1ES|BIOUG16551-F09|Valerie Levesque-Beaudin|BIN Taxonomy Match|BOLD:ACP6976
Tokunagaia sp. 1ES|BIOUG15807-H02|Valerie Levesque-Beaudin|BIN Taxonomy Match|BOLD:ACP6976
Tokunagaia sp. 1ES|BIOUG15797-G05|Valerie Levesque-Beaudin|BIN Taxonomy Match|BOLD:ACP6976
Tokunagaia sp. 1ES|BIOUG15795-G04|Valerie Levesque-Beaudin|BIN Taxonomy Match|BOLD:ACP6976
Tokunagaia sp. 1ES|BIOUG15967-D10|Valerie Levesque-Beaudin|BIN Taxonomy Match|BOLD:ACP6976
Tokunagaia sp. 1ES|BIOUG15798-G12|Valerie Levesque-Beaudin|BIN Taxonomy Match|BOLD:ACP6976
Tokunagaia sp. 1ES|BIOUG15765-H04|Valerie Levesque-Beaudin|BIN Taxonomy Match|BOLD:ACP6976
Tokunagaia sp. 1ES|BIOUG15765-F06|Valerie Levesque-Beaudin|BIN Taxonomy Match|BOLD:ACP6976
Tokunagaia sp. 1ES|BIOUG15766-B01|Valerie Levesque-Beaudin|BIN Taxonomy Match|BOLD:ACP6976
Tokunagaia sp. 1ES|BIOUG15765-G02|Valerie Levesque-Beaudin|BIN Taxonomy Match|BOLD:ACP6976
Tokunagaia sp. 1ES|BIOUG16622-H08|Valerie Levesque-Beaudin|BIN Taxonomy Match|BOLD:ACP6976
Tokunagaia sp. 1ES|BIOUG15766-B08|Valerie Levesque-Beaudin|BIN Taxonomy Match|BOLD:ACP6976
Tokunagaia sp. 1ES|BIOUG16713-C07|Valerie Levesque-Beaudin|BIN Taxonomy Match|BOLD:ACP6976
Tokunagaia sp. 1ES|BIOUG16713-A02|Valerie Levesque-Beaudin|BIN Taxonomy Match|BOLD:ACP6976
Tokunagaia sp. 1ES|BIOUG16713-E07|Valerie Levesque-Beaudin|BIN Taxonomy Match|BOLD:ACP6976
Tokunagaia sp. 1ES|BIOUG16713-D10|Valerie Levesque-Beaudin|BIN Taxonomy Match|BOLD:ACP6976
Tokunagaia sp. 1ES|BIOUG16713-F12|Valerie Levesque-Beaudin|BIN Taxonomy Match|BOLD:ACP6976
Tokunagaia sp. 1ES|BIOUG16713-E10|Valerie Levesque-Beaudin|BIN Taxonomy Match|BOLD:ACP6976
Tokunagaia sp. 1ES|BIOUG16711-E12|Valerie Levesque-Beaudin|BIN Taxonomy Match|BOLD:ACP6976
Tokunagaia sp. 1ES|BIOUG16477-D06|Valerie Levesque-Beaudin|BIN Taxonomy Match|BOLD:ACP6976
Tokunagaia sp. 1ES|BIOUG16711-F12|Valerie Levesque-Beaudin|BIN Taxonomy Match|BOLD:ACP6976
Tokunagaia sp. 1ES|BIOUG16711-F05|Valerie Levesque-Beaudin|BIN Taxonomy Match|BOLD:ACP6976
Tokunagaia sp. 1ES|BIOUG16711-G08|Valerie Levesque-Beaudin|BIN Taxonomy Match|BOLD:ACP6976
Tokunagaia sp. 1ES|BIOUG16711-G05|Valerie Levesque-Beaudin|BIN Taxonomy Match|BOLD:ACP6976
Tokunagaia sp. 1ES|BIOUG16712-C04|Valerie Levesque-Beaudin|BIN Taxonomy Match|BOLD:ACP6976
Tokunagaia sp. 1ES|BIOUG16712-D03|Valerie Levesque-Beaudin|BIN Taxonomy Match|BOLD:ACP6976
Tokunagaia sp. 1ES|BIOUG16712-F11|Valerie Levesque-Beaudin|BIN Taxonomy Match|BOLD:ACP6976
Tokunagaia sp. 1ES|BIOUG16712-G02|Valerie Levesque-Beaudin|BIN Taxonomy Match|BOLD:ACP6976
Tokunagaia sp. 1ES|BIOUG16712-G09|Elisabeth Stur|morphology|BOLD:ACP6976
Tokunagaia sp. 1ES|BIOUG16712-H06|Valerie Levesque-Beaudin|BIN Taxonomy Match|BOLD:ACP6976
Tokunagaia sp. 1ES|BIOUG17022-H04|Valerie Levesque-Beaudin|BIN Taxonomy Match|BOLD:ACP6976
Tokunagaia sp. 1ES|BIOUG15718-E05|Elisabeth Stur|morphology|BOLD:ACP6976
Tokunagaia sp. 1ES|BIOUG16713-B01|Valerie Levesque-Beaudin|BIN Taxonomy Match|BOLD:ACP6976

Tokunagaia sp. 1ES|BIOUG17022-H04|Valerie Levesque-Beaudin|BIN Taxonomy Match|BOLD:ACP6976
Tokunagaia sp. 1ES|BIOUG15718-E05|Elisabeth Stur|morphology|BOLD:ACP6976
Tokunagaia sp. 1ES|BIOUG16713-B01|Valerie Levesque-Beaudin|BIN Taxonomy Match|BOLD:ACP6976
Tokunagaia sp. 1ES|BIOUG15762-E07|Valerie Levesque-Beaudin|BIN Taxonomy Match|BOLD:ACP6976
Tokunagaia sp. 1ES|BIOUG15718-E08|Elisabeth Stur|morphology|BOLD:ACP6976
Tokunagaia sp. 1ES|BIOUG15762-F09|Valerie Levesque-Beaudin|BIN Taxonomy Match|BOLD:ACP6976
Tokunagaia sp. 1ES|BIOUG15766-F01|Valerie Levesque-Beaudin|BIN Taxonomy Match|BOLD:ACP6976
Tokunagaia sp. 1ES|BIOUG16711-A04|Valerie Levesque-Beaudin|BIN Taxonomy Match|BOLD:ACP6976
Tokunagaia sp. 1ES|BIOUG15766-C07|Valerie Levesque-Beaudin|BIN Taxonomy Match|BOLD:ACP6976
Tokunagaia sp. 1ES|BIOUG15763-F02|Valerie Levesque-Beaudin|BIN Taxonomy Match|BOLD:ACP6976
Tokunagaia sp. 1ES|BIOUG15763-E06|Valerie Levesque-Beaudin|BIN Taxonomy Match|BOLD:ACP6976
Tokunagaia sp. 1ES|BIOUG15762-H11|Valerie Levesque-Beaudin|BIN Taxonomy Match|BOLD:ACP6976
Tokunagaia sp. 1ES|BIOUG15764-F07|Valerie Levesque-Beaudin|BIN Taxonomy Match|BOLD:ACP6976
Tokunagaia sp. 1ES|BIOUG15718-E07|Elisabeth Stur|morphology|BOLD:ACP6976
Tokunagaia sp. 1ES|BIOUG15720-G08|Valerie Levesque-Beaudin|BIN Taxonomy Match|BOLD:ACP6976
Tokunagaia sp. 1ES|BIOUG15765-C09|Valerie Levesque-Beaudin|BIN Taxonomy Match|BOLD:ACP6976
Tokunagaia sp. 1ES|BIOUG15809-D10|Valerie Levesque-Beaudin|BIN Taxonomy Match|BOLD:ACP6976
Tokunagaia sp. 1ES|BIOUG16711-B09|Valerie Levesque-Beaudin|BIN Taxonomy Match|BOLD:ACP6976
Tokunagaia sp. 1ES|BIOUG15761-C03|Valerie Levesque-Beaudin|BIN Taxonomy Match|BOLD:ACP6976
Tokunagaia sp. 1ES|BIOUG15762-H02|Valerie Levesque-Beaudin|BIN Taxonomy Match|BOLD:ACP6976
Tokunagaia sp. 1ES|BIOUG15719-C02|Valerie Levesque-Beaudin|BIN Taxonomy Match|BOLD:ACP6976
Tokunagaia sp. 1ES|BIOUG15761-H07|Valerie Levesque-Beaudin|BIN Taxonomy Match|BOLD:ACP6976
Tokunagaia sp. 1ES|BIOUG15806-C07|Valerie Levesque-Beaudin|BIN Taxonomy Match|BOLD:ACP6976
Tokunagaia sp. 1ES|BIOUG15949-H10|Valerie Levesque-Beaudin|BIN Taxonomy Match|BOLD:ACP6976
Tokunagaia sp. 1ES|BIOUG16718-H06|Valerie Levesque-Beaudin|BIN Taxonomy Match|BOLD:ACP6976
Tokunagaia sp. 1ES|BIOUG15764-E03|Valerie Levesque-Beaudin|BIN Taxonomy Match|BOLD:ACP6976
Tokunagaia sp. 1ES|BIOUG15763-C08|Valerie Levesque-Beaudin|BIN Taxonomy Match|BOLD:ACP6976
Tokunagaia sp. 1ES|BIOUG15718-E02|Elisabeth Stur|morphology|BOLD:ACP6976
Tokunagaia sp. 1ES|BIOUG15765-G06|Valerie Levesque-Beaudin|BIN Taxonomy Match|BOLD:ACP6976
Tokunagaia sp. 1ES|BIOUG15769-C11|Valerie Levesque-Beaudin|BIN Taxonomy Match|BOLD:ACP6976
Tokunagaia sp. 1ES|BIOUG16296-B06|Valerie Levesque-Beaudin|BIN Taxonomy Match|BOLD:ACP6976
Tokunagaia sp. 1ES|BIOUG16299-G08|Valerie Levesque-Beaudin|BIN Taxonomy Match|BOLD:ACP6976
Tokunagaia sp. 1ES|BIOUG16434-B03|Valerie Levesque-Beaudin|BIN Taxonomy Match|BOLD:ACP6976
Tokunagaia sp. 1ES|BIOUG16479-F04|Valerie Levesque-Beaudin|BIN Taxonomy Match|BOLD:ACP6976
Tokunagaia sp. 1ES|BIOUG16435-H10|Valerie Levesque-Beaudin|BIN Taxonomy Match|BOLD:ACP6976
Tokunagaia sp. 1ES|BIOUG16713-A01|Valerie Levesque-Beaudin|BIN Taxonomy Match|BOLD:ACP6976
Tokunagaia sp. 1ES|BIOUG16713-C03|Valerie Levesque-Beaudin|BIN Taxonomy Match|BOLD:ACP6976
Tokunagaia sp. 1ES|BIOUG16713-C12|Valerie Levesque-Beaudin|BIN Taxonomy Match|BOLD:ACP6976
Tokunagaia sp. 1ES|BIOUG16713-G09|Valerie Levesque-Beaudin|BIN Taxonomy Match|BOLD:ACP6976
Tokunagaia sp. 1ES|BIOUG16631-C06|Valerie Levesque-Beaudin|BIN Taxonomy Match|BOLD:ACP6976
Tokunagaia sp. 1ES|BIOUG16632-G09|Valerie Levesque-Beaudin|BIN Taxonomy Match|BOLD:ACP6976
Tokunagaia sp. 1ES|BIOUG15719-E10|Valerie Levesque-Beaudin|BIN Taxonomy Match|BOLD:ACP6976
Tokunagaia sp. 1ES|BIOUG16712-B02|Valerie Levesque-Beaudin|BIN Taxonomy Match|BOLD:ACP6976
Tokunagaia sp. 1ES|BIOUG16476-H10|Valerie Levesque-Beaudin|BIN Taxonomy Match|BOLD:ACP6976
Tokunagaia sp. 1ES|BIOUG16712-G03|Valerie Levesque-Beaudin|BIN Taxonomy Match|BOLD:ACP6976
Tokunagaia sp. 1ES|BIOUG16711-E03|Valerie Levesque-Beaudin|BIN Taxonomy Match|BOLD:ACP6976
Tokunagaia sp. 1ES|BIOUG16432-E04|Valerie Levesque-Beaudin|BIN Taxonomy Match|BOLD:ACP6976
Tokunagaia sp. 1ES|BIOUG15720-F09|Valerie Levesque-Beaudin|BIN Taxonomy Match|BOLD:ACP6976
Tokunagaia sp. 1ES|BIOUG15764-A12|Valerie Levesque-Beaudin|BIN Taxonomy Match|BOLD:ACP6976
Tokunagaia sp. 1ES|BIOUG16713-B11|Valerie Levesque-Beaudin|BIN Taxonomy Match|BOLD:ACP6976
Tokunagaia sp. 1ES|BIOUG16632-H07|Valerie Levesque-Beaudin|BIN Taxonomy Match|BOLD:ACP6976
Tokunagaia sp. 1ES|BIOUG15793-G03|Valerie Levesque-Beaudin|BIN Taxonomy Match|BOLD:ACP6976
Tokunagaia sp. 1ES|BIOUG15764-A09|Valerie Levesque-Beaudin|BIN Taxonomy Match|BOLD:ACP6976
Tokunagaia sp. 1ES|BIOUG15718-C07|Elisabeth Stur|morphology|BOLD:ACP6976
Tokunagaia sp. 1ES|BIOUG15719-G07|Valerie Levesque-Beaudin|BIN Taxonomy Match|BOLD:ACP6976
Tokunagaia sp. 1ES|BIOUG15761-B11|Valerie Levesque-Beaudin|BIN Taxonomy Match|BOLD:ACP6976
Tokunagaia sp. 1ES|BIOUG16480-B07|Valerie Levesque-Beaudin|BIN Taxonomy Match|BOLD:ACP6976
Tokunagaia sp. 1ES|BIOUG16426-H05|Valerie Levesque-Beaudin|BIN Taxonomy Match|BOLD:ACP6976
Tokunagaia sp. 1ES|BIOUG15766-F05|Valerie Levesque-Beaudin|BIN Taxonomy Match|BOLD:ACP6976
Tokunagaia sp. 1ES|BIOUG16430-E05|Valerie Levesque-Beaudin|BIN Taxonomy Match|BOLD:ACP6976
Tokunagaia sp. 1ES|BIOUG15766-C05|Valerie Levesque-Beaudin|BIN Taxonomy Match|BOLD:ACP6976
Tokunagaia sp. 1ES|BIOUG16711-H02|Valerie Levesque-Beaudin|BIN Taxonomy Match|BOLD:ACP6976
Tokunagaia sp. 1ES|BIOUG16712-B01|Valerie Levesque-Beaudin|BIN Taxonomy Match|BOLD:ACP6976
Tokunagaia sp. 1ES|BIOUG16631-G12|Valerie Levesque-Beaudin|BIN Taxonomy Match|BOLD:ACP6976
Tokunagaia sp. 1ES|BIOUG16433-B05|Valerie Levesque-Beaudin|BIN Taxonomy Match|BOLD:ACP6976
Tokunagaia sp. 1ES|BIOUG15766-E05|Valerie Levesque-Beaudin|BIN Taxonomy Match|BOLD:ACP6976
Tokunagaia sp. 1ES|BIOUG15763-G06|Valerie Levesque-Beaudin|BIN Taxonomy Match|BOLD:ACP6976
Tokunagaia sp. 1ES|BIOUG15720-H08|Valerie Levesque-Beaudin|BIN Taxonomy Match|BOLD:ACP6976
Tokunagaia sp. 1ES|BIOUG15769-C12|Valerie Levesque-Beaudin|BIN Taxonomy Match|BOLD:ACP6976
Tokunagaia sp. 1ES|BIOUG15766-H06|Valerie Levesque-Beaudin|BIN Taxonomy Match|BOLD:ACP6976
Tokunagaia sp. 1ES|BIOUG16596-A12|Valerie Levesque-Beaudin|BIN Taxonomy Match|BOLD:ACP6976
Tokunagaia sp. 1ES|BIOUG15718-C12|Elisabeth Stur|morphology|BOLD:ACP6976
Tokunagaia sp. 1ES|BIOUG15766-H07|Valerie Levesque-Beaudin|BIN Taxonomy Match|BOLD:ACP6976
Tokunagaia sp. 1ES|BIOUG16476-B04|Valerie Levesque-Beaudin|BIN Taxonomy Match|BOLD:ACP6976
Tokunagaia sp. 1ES|BIOUG16596-A02|Valerie Levesque-Beaudin|BIN Taxonomy Match|BOLD:ACP6976
Tokunagaia sp. 1ES|BIOUG16479-F07|Valerie Levesque-Beaudin|BIN Taxonomy Match|BOLD:ACP6976
Tokunagaia sp. 1ES|BIOUG16712-D06|Valerie Levesque-Beaudin|BIN Taxonomy Match|BOLD:ACP6976
Tokunagaia sp. 1ES|BIOUG15719-C11|Valerie Levesque-Beaudin|BIN Taxonomy Match|BOLD:ACP6976
Tokunagaia sp. 1ES|BIOUG15766-B10|Valerie Levesque-Beaudin|BIN Taxonomy Match|BOLD:ACP6976
Tokunagaia sp. 1ES|BIOUG15764-G11|Valerie Levesque-Beaudin|BIN Taxonomy Match|BOLD:ACP6976
Tokunagaia sp. 1ES|BIOUG15718-C06|Elisabeth Stur|morphology|BOLD:ACP6976
Tokunagaia sp. 1ES|BIOUG15766-H10|Valerie Levesque-Beaudin|BIN Taxonomy Match|BOLD:ACP6976
Tokunagaia sp. 1ES|BIOUG15795-A03|Valerie Levesque-Beaudin|BIN Taxonomy Match|BOLD:ACP6976
Tokunagaia sp. 1ES|BIOUG15795-F04|Valerie Levesque-Beaudin|BIN Taxonomy Match|BOLD:ACP6976
Tokunagaia sp. 1ES|BIOUG16632-E03|Valerie Levesque-Beaudin|BIN Taxonomy Match|BOLD:ACP6976
Tokunagaia sp. 1ES|BIOUG16632-F03|Valerie Levesque-Beaudin|BIN Taxonomy Match|BOLD:ACP6976
Tokunagaia sp. 1ES|BIOUG16632-E09|Valerie Levesque-Beaudin|BIN Taxonomy Match|BOLD:ACP6976
Tokunagaia sp. 1ES|BIOUG16632-E04|Valerie Levesque-Beaudin|BIN Taxonomy Match|BOLD:ACP6976
Tokunagaia sp. 1ES|BIOUG16631-A09|Valerie Levesque-Beaudin|BIN Taxonomy Match|BOLD:ACP6976
Tokunagaia sp. 1ES|BIOUG16435-D12|Valerie Levesque-Beaudin|BIN Taxonomy Match|BOLD:ACP6976
Tokunagaia sp. 1ES|BIOUG16435-G03|Valerie Levesque-Beaudin|BIN Taxonomy Match|BOLD:ACP6976
Tokunagaia sp. 1ES|BIOUG15718-G08|Valerie Levesque-Beaudin|BIN Taxonomy Match|BOLD:ACP6976
Tokunagaia sp. 1ES|BIOUG15718-F06|Elisabeth Stur|morphology|BOLD:ACP6976
Tokunagaia sp. 1ES|BIOUG15764-F09|Valerie Levesque-Beaudin|BIN Taxonomy Match|BOLD:ACP6976
Tokunagaia sp. 1ES|BIOUG15718-B05|Elisabeth Stur|morphology|BOLD:ACP6976
Tokunagaia sp. 1ES|BIOUG15718-A04|Elisabeth Stur|morphology|BOLD:ACP6976
Georthocladius sp. MUSE-VL-Sc2B_48|MZ004971|||BOLD:AEK7351
Tokunagaia cf. scutellata|ES390|Elisabeth Stur||BOLD:ACO4878
Chironomidae|24464-A12|Mikko J Tiusanen|Tree based identification|BOLD:ACU4609
Chironomidae|24465-E05|Mikko J Tiusanen|Tree based identification|BOLD:ACU4609
Tokunagaia rectangularis|ATNA243|Elisabeth Stur||BOLD:AAF8345
Tokunagaia|ZMUO.025914|Lauri Paasivirta||BOLD:AAF8345
Tokunagaia|ZMUO.025915|Lauri Paasivirta||BOLD:AAF8345

Chironomidae|24403-E05|Mikko J Tiusanen|Tree based identification|BOLD:ACU4609
Tokunagaia rectangularis|ATNA243|Elisabeth Stur||BOLD:AAF8345
Tokunagaia|ZMUO.025914|Lauri Paasivirta||BOLD:AAF8345
Tokunagaia|ZMUO.025915|Lauri Paasivirta||BOLD:AAF8345
Tokunagaia rectangularis|ATNA458|Elisabeth Stur||BOLD:AAF8345
Tokunagaia rectangularis|ATNA459|Elisabeth Stur||BOLD:AAF8345
Tokunagaia rectangularis|ATNA245|Elisabeth Stur||BOLD:AAF8345
Tokunagaia parexcellens|EBAI-Ch082|Elisabeth Stur|morphology|BOLD:AAD2194
Tokunagaia parexcellens|EBAI-Ch083|Elisabeth Stur|morphology|BOLD:AAD2194
Tokunagaia parexcellens|ZMUO.026104|Lauri Paasivirta||BOLD:AAD2194
Tokunagaia parexcellens|ATNA550|Elisabeth Stur||BOLD:AAD2194
Tokunagaia parexcellens|ATNA346|Elisabeth Stur||BOLD:AAD2194
Tokunagaia parexcellens|ATNA345|Elisabeth Stur||BOLD:AAD2194
Tokunagaia parexcellens|ATNA344|Elisabeth Stur||BOLD:AAD2194
Tokunagaia parexcellens|ATNA143|Elisabeth Stur||BOLD:AAD2194
Tokunagaia parexcellens|ATNA154|Elisabeth Stur||BOLD:AAD2194
Tokunagaia parexcellens|ATNA152|Elisabeth Stur||BOLD:AAD2194
Tokunagaia parexcellens|ATNA145|Elisabeth Stur||BOLD:AAD2194
Tokunagaia parexcellens|ATNA142|Elisabeth Stur||BOLD:AAD2194
Tokunagaia parexcellens|ZMUO.026105|Lauri Paasivirta||BOLD:ADF0342
Tokunagaia excellens|ATNA560|Elisabeth Stur||BOLD:AAL7116
Tokunagaia excellens|ATNA574|Elisabeth Stur|morphology|BOLD:AAL7116
Tokunagaia excellens|ATNA515|Elisabeth Stur||BOLD:AAL7116
Tokunagaia tonollii|ATNA261|Elisabeth Stur||BOLD:AAE7618
Tokunagaia tonollii|Finnmark69|Elisabeth Stur||BOLD:AAE7618
Tokunagaia tonollii|ATNA252|Elisabeth Stur||BOLD:AAE7618
Tokunagaia|ZMUO.025860|Lauri Paasivirta||BOLD:AAE7618
Tokunagaia|ZMUO.025861|Lauri Paasivirta||BOLD:AAE7618
Tokunagaia tonollii|ATNA457|Elisabeth Stur||BOLD:AAE7618
Tokunagaia tonollii|ATNA456|Elisabeth Stur||BOLD:AAE7618
Tvetenia duodenaria|Finnmark561|Elisabeth Stur||BOLD:AAP6932
Tvetenia duodenaria|Finnmark292|Elisabeth Stur||BOLD:AAP6932
Tvetenia duodenaria|Finnmark288|Elisabeth Stur||BOLD:AAP6932
Tvetenia duodenaria|ZMUO.026113|Lauri Paasivirta||BOLD:AAP6932
Tvetenia duodenaria|ZMUO.026112|Lauri Paasivirta||BOLD:AAP6932
Tvetenia duodenaria|ZMUO.026334|Lauri Paasivirta||BOLD:AAP6932
Tvetenia duodenaria|Finnmark206|Elisabeth Stur||BOLD:AAP6932
Tvetenia duodenaria|Finnmark289|Elisabeth Stur||BOLD:AAP6932
Tvetenia duodenaria|Finnmark287|Elisabeth Stur||BOLD:AAP6932
Tvetenia duodenaria|Finnmark281|Elisabeth Stur||BOLD:AAP6932
Tokunagaia kibunensis|Finnmark415|Elisabeth Stur||BOLD:AAV2991
Tokunagaia kibunensis|EBAI-Ch095|Elisabeth Stur|morphology|BOLD:AAV2991
Tokunagaia kibunensis|EBAI-Ch098|Elisabeth Stur|morphology|BOLD:AAV2991
Tokunagaia kibunensis|EBAI-Ch096|Elisabeth Stur|morphology|BOLD:AAV2991
Tokunagaia kibunensis|EBAI-Ch097|Elisabeth Stur|morphology|BOLD:AAV2991
Tokunagaia kibunensis|EBAI-Ch223|Elisabeth Stur|morphology|BOLD:AAV2991
Tokunagaia kibunensis|BIOUG15764-C08|Kate Perez|BIN Taxonomy Match|BOLD:AAV2991
Eukiefferiella|CCDB24038-A03|Sofia Wiedenbrug|BIN Taxonomy Match|BOLD:ACU4228
Chironomidae|CCDB24037-E07|Sofia Wiedenbrug|no morphological identification - introduction of int...
Eukiefferiella tirolensis|ES73|Elisabeth Stur||BOLD:AAY3174
Chaetocladius|ZMUO.025888|Lauri Paasivirta||BOLD:AAF4068
Chaetocladius|ZMUO.025889|Lauri Paasivirta||BOLD:AAF4068
Chaetocladius grandilobus|ATNA365|Elisabeth Stur||BOLD:AAF4068
Chaetocladius grandilobus|ATNA363|Elisabeth Stur||BOLD:AAF4068
Chaetocladius grandilobus|ATNA461|Elisabeth Stur||BOLD:AAF4068
Chaetocladius grandilobus|Finnmark332|Elisabeth Stur||BOLD:AAF4068
Chaetocladius grandilobus|Finnmark330|Elisabeth Stur||BOLD:AAF4068
Chaetocladius sp. 2SW|ZSM-DIP-33134-D01|Caroline Chimeno|BIN Taxonomy Match (Jan 2022)|BOLD...
Chaetocladius sp. 2SW|BC-ZSM-DIP-22488-G07|Dieter Doczkal||BOLD:ACU4808
Chaetocladius sp. 2SW|CCDB24039-A01|Jerome Moriniere||BOLD:ACU4808
Chaetocladius sp. 2SW|CCDB24038-G01|Jerome Moriniere||BOLD:ACU4808
Chaetocladius sp. 2SW|BC-ZSM-DIP-22488-G03|Dieter Doczkal||BOLD:ACU4808
Chaetocladius sp. 2SW|CCDB24038-E10|Jerome Moriniere||BOLD:ACU4808
Chaetocladius sp. 2SW|BC-ZSM-DIP-22488-F10|Dieter Doczkal||BOLD:ACU4808
Chaetocladius sp. 2SW|CCDB24038-C12|Jerome Moriniere||BOLD:ACU4808
Chironomidae|CCDB24037-A06|Jerome Moriniere|BIN Taxonomy Match|BOLD:ACU4746
Chironomidae|ZSM-DIP-33172-C04|Caroline Chimeno|BIN Taxonomy Match (Jan 2022)|BOLD:AEA1528
Chaetocladius melaleucus|ZMUO.026287|Lauri Paasivirta||BOLD:AAD4034
Chaetocladius melaleucus|ZSM34342-D04|Sofia Wiedenbrug|Morphology|BOLD:AAD4034
Chaetocladius dentiforceps agg|PP0283|Lauri Paasivirta||BOLD:AAD4034
Chaetocladius melaleucus|SOE349|Elisabeth Stur||BOLD:AAD4034
Chaetocladius melaleucus|ATNA396|Elisabeth Stur||BOLD:AAD4034
Chaetocladius melaleucus|SOE54|Elisabeth Stur||BOLD:AAD4034
Chaetocladius melaleucus|SOE336|Elisabeth Stur||BOLD:AAD4034
Chaetocladius melaleucus|ATNA284|Elisabeth Stur||BOLD:AAD4034
Chaetocladius longivirgatus|ATNA194|Elisabeth Stur||BOLD:AAC0615
Chaetocladius longivirgatus|ATNA208|Elisabeth Stur||BOLD:AAC0615
Chaetocladius longivirgatus|ATNA408|Elisabeth Stur||BOLD:AAC0615
Chaetocladius longivirgatus|ATNA453|Elisabeth Stur||BOLD:AAC0615
Chaetocladius longivirgatus|ATNA220|Elisabeth Stur||BOLD:AAC0615
Chaetocladius longivirgatus|ATNA562|Elisabeth Stur||BOLD:AAC0615
Chaetocladius longivirgatus|ATNA452|Elisabeth Stur||BOLD:AAC0615
Chaetocladius longivirgatus|ATNA451|Elisabeth Stur||BOLD:AAC0615
Chaetocladius longivirgatus|BIOUG15454-D01|Kate Perez|BIN Taxonomy Match|BOLD:AAC0615
Chaetocladius sp. 6ES|SOE306|Elisabeth Stur||BOLD:AAI4213
Chaetocladius sp. 6ES|ATNA548|Elisabeth Stur||BOLD:AAI4213
Chaetocladius suecicus|ZMUO.025989|Lauri Paasivirta||BOLD:AAI4213
Chaetocladius sp. 6ES|ATNA464|Elisabeth Stur||BOLD:AAI4213
Chaetocladius sp. 6ES|Finnmark554|Elisabeth Stur||BOLD:AAI4213
Chaetocladius|SOE331|Elisabeth Stur||BOLD:AAI4214
Chaetocladius suecicus|CCDB24037-D05|Sofia Wiedenbrug|BIN Taxonomy Match|BOLD:AAC0614
Chaetocladius suecicus|CCDB24039-D08|Sofia Wiedenbrug|BIN Taxonomy Match|BOLD:AAC0614
Chaetocladius suecicus|ATNA193|Elisabeth Stur||BOLD:AAC0614
Chaetocladius suecicus|CCDB24039-G06|Sofia Wiedenbrug|BIN Taxonomy Match|BOLD:AAC0614
Chaetocladius suecicus|ATNA14|Elisabeth Stur||BOLD:AAC0614
Chaetocladius suecicus|ATNA200|Elisabeth Stur||BOLD:AAC0614
Chaetocladius suecicus|ATNA427|Elisabeth Stur||BOLD:AAC0614
Chaetocladius suecicus|ATNA428|Elisabeth Stur||BOLD:AAC0614
Chaetocladius suecicus|ZSM-DIP-33170-F02|Caroline Chimeno|BIN Taxonomy Match (Jan 2022)|BOL...
Chaetocladius suecicus|ZSM34343-G09|Sofia Wiedenbrug|Morphology|BOLD:AAC0614
Chaetocladius suecicus|CCDB24038-E02|Sofia Wiedenbrug|BIN Taxonomy Match|BOLD:AAC0614
Chaetocladius suecicus|ES382|Elisabeth Stur||BOLD:AAC0614
Chaetocladius suecicus|ATNA547|Elisabeth Stur||BOLD:AAC0614
[illegible]

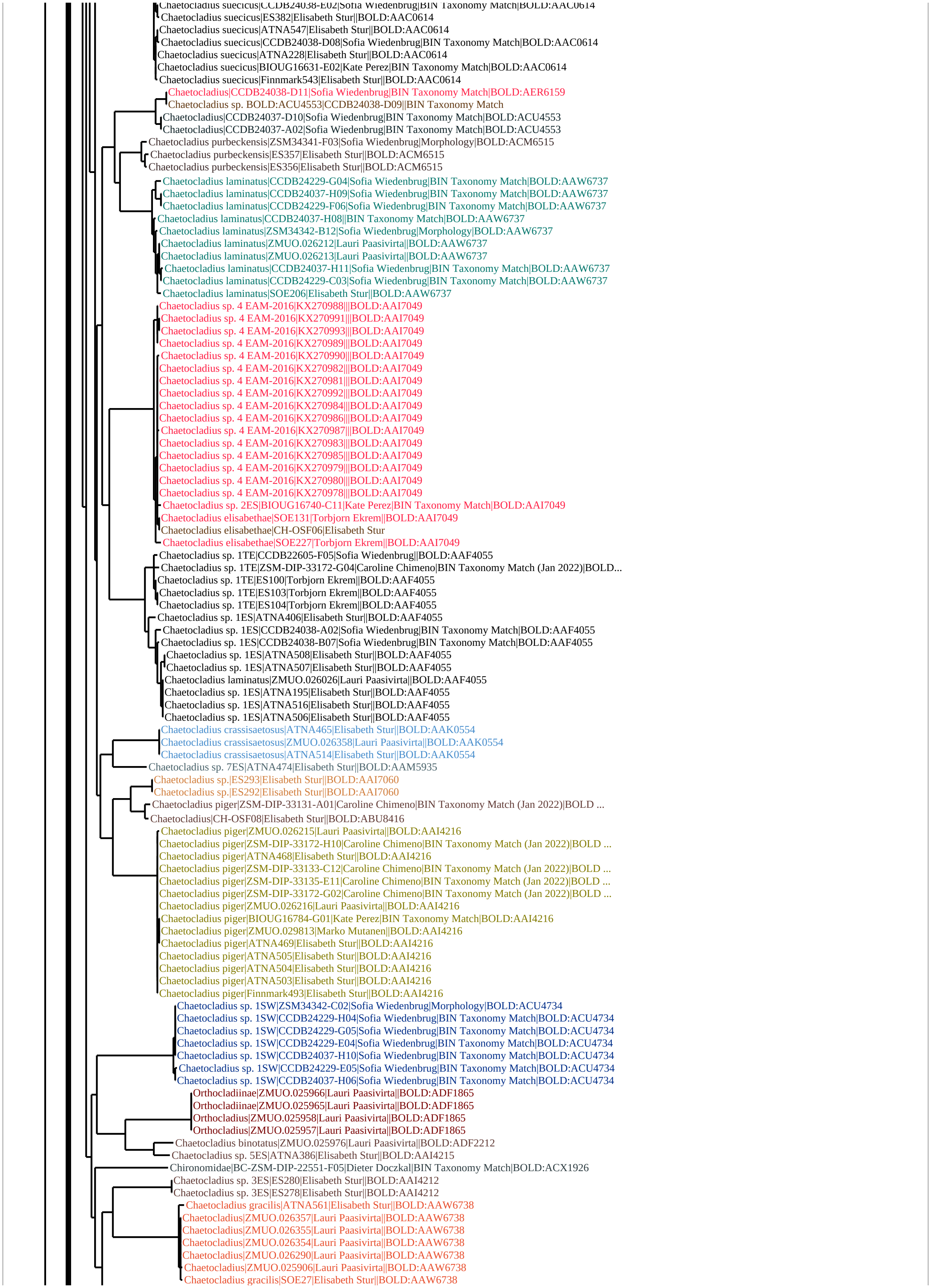
Chaetocladius suecicus|CCDB24038-E02|Sofia Wiedenbrug|BIN Taxonomy Match|BOLD:AAC0614
Chaetocladius suecicus|ES382|Elisabeth Stur||BOLD:AAC0614
Chaetocladius suecicus|ATNA547|Elisabeth Stur||BOLD:AAC0614
Chaetocladius suecicus|CCDB24038-D08|Sofia Wiedenbrug|BIN Taxonomy Match|BOLD:AAC0614
Chaetocladius suecicus|ATNA228|Elisabeth Stur||BOLD:AAC0614
Chaetocladius suecicus|BIOUG16631-E02|Kate Perez|BIN Taxonomy Match|BOLD:AAC0614
Chaetocladius suecicus|Finnmark543|Elisabeth Stur||BOLD:AAC0614
Chaetocladius|CCDB24038-D11|Sofia Wiedenbrug|BIN Taxonomy Match|BOLD:AER6159
Chaetocladius sp. BOLD:ACU4553|CCDB24038-D09||BIN Taxonomy Match
Chaetocladius|CCDB24037-D10|Sofia Wiedenbrug|BIN Taxonomy Match|BOLD:ACU4553
Chaetocladius|CCDB24037-A02|Sofia Wiedenbrug|BIN Taxonomy Match|BOLD:ACU4553
Chaetocladius purbeckensis|ZSM34341-F03|Sofia Wiedenbrug|Morphology|BOLD:ACM6515
Chaetocladius purbeckensis|ES357|Elisabeth Stur||BOLD:ACM6515
Chaetocladius purbeckensis|ES356|Elisabeth Stur||BOLD:ACM6515
Chaetocladius laminatus|CCDB24229-G04|Sofia Wiedenbrug|BIN Taxonomy Match|BOLD:AAW6737
Chaetocladius laminatus|CCDB24037-H09|Sofia Wiedenbrug|BIN Taxonomy Match|BOLD:AAW6737
Chaetocladius laminatus|CCDB24229-F06|Sofia Wiedenbrug|BIN Taxonomy Match|BOLD:AAW6737
Chaetocladius laminatus|CCDB24037-H08||BIN Taxonomy Match|BOLD:AAW6737
Chaetocladius laminatus|ZSM34342-B12|Sofia Wiedenbrug|Morphology|BOLD:AAW6737
Chaetocladius laminatus|ZMUO.026212|Lauri Paasivirta||BOLD:AAW6737
Chaetocladius laminatus|ZMUO.026213|Lauri Paasivirta||BOLD:AAW6737
Chaetocladius laminatus|CCDB24037-H11|Sofia Wiedenbrug|BIN Taxonomy Match|BOLD:AAW6737
Chaetocladius laminatus|CCDB24229-C03|Sofia Wiedenbrug|BIN Taxonomy Match|BOLD:AAW6737
Chaetocladius laminatus|SOE206|Elisabeth Stur||BOLD:AAW6737
Chaetocladius sp. 4 EAM-2016|KX270988|||BOLD:AAI7049
Chaetocladius sp. 4 EAM-2016|KX270991|||BOLD:AAI7049
Chaetocladius sp. 4 EAM-2016|KX270993|||BOLD:AAI7049
Chaetocladius sp. 4 EAM-2016|KX270989|||BOLD:AAI7049
Chaetocladius sp. 4 EAM-2016|KX270990|||BOLD:AAI7049
Chaetocladius sp. 4 EAM-2016|KX270982|||BOLD:AAI7049
Chaetocladius sp. 4 EAM-2016|KX270981|||BOLD:AAI7049
Chaetocladius sp. 4 EAM-2016|KX270992|||BOLD:AAI7049
Chaetocladius sp. 4 EAM-2016|KX270984|||BOLD:AAI7049
Chaetocladius sp. 4 EAM-2016|KX270986|||BOLD:AAI7049
Chaetocladius sp. 4 EAM-2016|KX270987|||BOLD:AAI7049
Chaetocladius sp. 4 EAM-2016|KX270983|||BOLD:AAI7049
Chaetocladius sp. 4 EAM-2016|KX270985|||BOLD:AAI7049
Chaetocladius sp. 4 EAM-2016|KX270979|||BOLD:AAI7049
Chaetocladius sp. 4 EAM-2016|KX270980|||BOLD:AAI7049
Chaetocladius sp. 4 EAM-2016|KX270978|||BOLD:AAI7049
Chaetocladius sp. 2ES|BIOUG16740-C11|Kate Perez|BIN Taxonomy Match|BOLD:AAI7049
Chaetocladius elisabethae|SOE131|Torbjorn Ekrem||BOLD:AAI7049
Chaetocladius elisabethae|CH-OSF06|Elisabeth Stur
Chaetocladius elisabethae|SOE227|Torbjorn Ekrem||BOLD:AAI7049
Chaetocladius sp. 1TE|CCDB22605-F05|Sofia Wiedenbrug||BOLD:AAF4055
Chaetocladius sp. 1TE|ZSM-DIP-33172-G04|Caroline Chimeno|BIN Taxonomy Match (Jan 2022)|BOLD...
Chaetocladius sp. 1TE|ES100|Torbjorn Ekrem||BOLD:AAF4055
Chaetocladius sp. 1TE|ES103|Torbjorn Ekrem||BOLD:AAF4055
Chaetocladius sp. 1TE|ES104|Torbjorn Ekrem||BOLD:AAF4055
Chaetocladius sp. 1ES|ATNA406|Elisabeth Stur||BOLD:AAF4055
Chaetocladius sp. 1ES|CCDB24038-A02|Sofia Wiedenbrug|BIN Taxonomy Match|BOLD:AAF4055
Chaetocladius sp. 1ES|CCDB24038-B07|Sofia Wiedenbrug|BIN Taxonomy Match|BOLD:AAF4055
Chaetocladius sp. 1ES|ATNA508|Elisabeth Stur||BOLD:AAF4055
Chaetocladius sp. 1ES|ATNA507|Elisabeth Stur||BOLD:AAF4055
Chaetocladius laminatus|ZMUO.026026|Lauri Paasivirta||BOLD:AAF4055
Chaetocladius sp. 1ES|ATNA195|Elisabeth Stur||BOLD:AAF4055
Chaetocladius sp. 1ES|ATNA516|Elisabeth Stur||BOLD:AAF4055
Chaetocladius sp. 1ES|ATNA506|Elisabeth Stur||BOLD:AAF4055
Chaetocladius crassisaetosus|ATNA465|Elisabeth Stur||BOLD:AAK0554
Chaetocladius crassisaetosus|ZMUO.026358|Lauri Paasivirta||BOLD:AAK0554
Chaetocladius crassisaetosus|ATNA514|Elisabeth Stur||BOLD:AAK0554
Chaetocladius sp. 7ES|ATNA474|Elisabeth Stur||BOLD:AAM5935
Chaetocladius sp.|ES293|Elisabeth Stur||BOLD:AAI7060
Chaetocladius sp.|ES292|Elisabeth Stur||BOLD:AAI7060
Chaetocladius piger|ZSM-DIP-33131-A01|Caroline Chimeno|BIN Taxonomy Match (Jan 2022)|BOLD ...
Chaetocladius|CH-OSF08|Elisabeth Stur||BOLD:ABU8416
Chaetocladius piger|ZMUO.026215|Lauri Paasivirta||BOLD:AAI4216
Chaetocladius piger|ZSM-DIP-33172-H10|Caroline Chimeno|BIN Taxonomy Match (Jan 2022)|BOLD ...
Chaetocladius piger|ATNA468|Elisabeth Stur||BOLD:AAI4216
Chaetocladius piger|ZSM-DIP-33133-C12|Caroline Chimeno|BIN Taxonomy Match (Jan 2022)|BOLD ...
Chaetocladius piger|ZSM-DIP-33135-E11|Caroline Chimeno|BIN Taxonomy Match (Jan 2022)|BOLD ...
Chaetocladius piger|ZSM-DIP-33172-G02|Caroline Chimeno|BIN Taxonomy Match (Jan 2022)|BOLD ...
Chaetocladius piger|ZMUO.026216|Lauri Paasivirta||BOLD:AAI4216
Chaetocladius piger|BIOUG16784-G01|Kate Perez|BIN Taxonomy Match|BOLD:AAI4216
Chaetocladius piger|ZMUO.029813|Marko Mutanen||BOLD:AAI4216
Chaetocladius piger|ATNA469|Elisabeth Stur||BOLD:AAI4216
Chaetocladius piger|ATNA505|Elisabeth Stur||BOLD:AAI4216
Chaetocladius piger|ATNA504|Elisabeth Stur||BOLD:AAI4216
Chaetocladius piger|ATNA503|Elisabeth Stur||BOLD:AAI4216
Chaetocladius piger|Finnmark493|Elisabeth Stur||BOLD:AAI4216
Chaetocladius sp. 1SW|ZSM34342-C02|Sofia Wiedenbrug|Morphology|BOLD:ACU4734
Chaetocladius sp. 1SW|CCDB24229-H04|Sofia Wiedenbrug|BIN Taxonomy Match|BOLD:ACU4734
Chaetocladius sp. 1SW|CCDB24229-G05|Sofia Wiedenbrug|BIN Taxonomy Match|BOLD:ACU4734
Chaetocladius sp. 1SW|CCDB24229-E04|Sofia Wiedenbrug|BIN Taxonomy Match|BOLD:ACU4734
Chaetocladius sp. 1SW|CCDB24037-H10|Sofia Wiedenbrug|BIN Taxonomy Match|BOLD:ACU4734
Chaetocladius sp. 1SW|CCDB24229-E05|Sofia Wiedenbrug|BIN Taxonomy Match|BOLD:ACU4734
Chaetocladius sp. 1SW|CCDB24037-H06|Sofia Wiedenbrug|BIN Taxonomy Match|BOLD:ACU4734
Orthocladiinae|ZMUO.025966|Lauri Paasivirta||BOLD:ADF1865
Orthocladiinae|ZMUO.025965|Lauri Paasivirta||BOLD:ADF1865
Orthocladius|ZMUO.025958|Lauri Paasivirta||BOLD:ADF1865
Orthocladius|ZMUO.025957|Lauri Paasivirta||BOLD:ADF1865
Chaetocladius binotatus|ZMUO.025976|Lauri Paasivirta||BOLD:ADF2212
Chaetocladius sp. 5ES|ATNA386|Elisabeth Stur||BOLD:AAI4215
Chironomidae|BC-ZSM-DIP-22551-F05|Dieter Doczkal|BIN Taxonomy Match|BOLD:ACX1926
Chaetocladius sp. 3ES|ES280|Elisabeth Stur||BOLD:AAI4212
Chaetocladius sp. 3ES|ES278|Elisabeth Stur||BOLD:AAI4212
Chaetocladius gracilis|ATNA561|Elisabeth Stur||BOLD:AAW6738
Chaetocladius|ZMUO.026357|Lauri Paasivirta||BOLD:AAW6738
Chaetocladius|ZMUO.026355|Lauri Paasivirta||BOLD:AAW6738
Chaetocladius|ZMUO.026354|Lauri Paasivirta||BOLD:AAW6738
Chaetocladius|ZMUO.026290|Lauri Paasivirta||BOLD:AAW6738
Chaetocladius|ZMUO.025906|Lauri Paasivirta||BOLD:AAW6738
Chaetocladius gracilis|SOE27|Elisabeth Stur||BOLD:AAW6738

Chaetocladius|ZMUO.026290|Lauri Paasivirta||BOLD:AAW6738
Chaetocladius|ZMUO.025906|Lauri Paasivirta||BOLD:AAW6738
Chaetocladius gracilis|SOE27|Elisabeth Stur||BOLD:AAW6738
Chaetocladius sp. 4ES|Finnmark564|Elisabeth Stur||BOLD:AAQ2098
Chaetocladius sp. 4ES|Finnmark562|Elisabeth Stur||BOLD:AAQ2098
Chaetocladius acuminatus|ZMUO.026288|Lauri Paasivirta||BOLD:AAQ2098
Chaetocladius acuminatus|ZMUO.026109|Lauri Paasivirta||BOLD:AAQ2098
Chaetocladius sp. 4ES|Finnmark293|Elisabeth Stur||BOLD:AAQ2098
Chaetocladius sp. 4ES|Finnmark285|Elisabeth Stur||BOLD:AAQ2098
Chaetocladius sp. 4ES|Finnmark280|Elisabeth Stur||BOLD:AAQ2098
Heleniella sp. 3SW|CCDB24037-D08|Sofia Wiedenbrug|BIN Taxonomy Match|BOLD:ACU4888
Heleniella sp. 3SW|ZSM34342-G08|Sofia Wiedenbrug|Morphology|BOLD:ACU4888
Heleniella sp. 3SW|ZSM34341-E09|Sofia Wiedenbrug|Morphology|BOLD:ACU4888
Heleniella sp. 3SW|CCDB24037-D07|Sofia Wiedenbrug|BIN Taxonomy Match|BOLD:ACU4888
Heleniella sp. 7ES|Finnmark789|Elisabeth Stur||BOLD:ACD1915
Heleniella sp. 7ES|Finnmark787|Elisabeth Stur||BOLD:ACD1915
Heleniella sp. 2ES|ZSM34342-C05|Sofia Wiedenbrug|Morphology|BOLD:AAD2807
Heleniella sp. 2ES|ES261|Elisabeth Stur||BOLD:AAD2807
Heleniella sp. 2ES|ES237|Elisabeth Stur||BOLD:AAD2807
Heleniella sp. 5ES|CCDB21606-A05|Sofia Wiedenbrug||BOLD:AAU5512
Heleniella sp. 4ES|ES139|Elisabeth Stur||BOLD:AAD2806
Heleniella sp. 3ES|ES08|Elisabeth Stur||BOLD:AAD2804
Heleniella sp. 6ES|ATNA580|Elisabeth Stur|morphology|BOLD:ADC1849
Heleniella sp. 2SW|CCDB21605-G05|Sofia Wiedenbrug
Cricotopus sp. 1ES|Finnmark135|Elisabeth Stur||BOLD:AAM6023
Heleniella sp. 1ES|ES231|Elisabeth Stur||BOLD:AAD2805
Heleniella sp. 1ES|ES190|Elisabeth Stur||BOLD:AAD2805
Heleniella sp. 1ES|ES119|Elisabeth Stur||BOLD:AAD2805
Heleniella sp. 1SW|CCDB21605-C03|Sofia Wiedenbrug||BOLD:ACS9327
Heleniella sp. 1SW|CCDB21605-E04|Sofia Wiedenbrug||BOLD:ACS9327
Heleniella sp. 1SW|CCDB21605-C02|Sofia Wiedenbrug||BOLD:ACS9327
Heleniella ornaticollis|ZMUO.024829|Lauri Paasivirta||BOLD:AAB0734
Heleniella ornaticollis|ZMUO.025921|Lauri Paasivirta||BOLD:AAB0734
Heleniella ornaticollis|ZMUO.024830|Lauri Paasivirta||BOLD:AAB0734
Heleniella ornaticollis|SOE292|Elisabeth Stur||BOLD:AAB0734
Heleniella ornaticollis|SOE281|Elisabeth Stur||BOLD:AAB0734
Heleniella ornaticollis|SOE441|Elisabeth Stur||BOLD:AAB0734
Heleniella ornaticollis|SOE407|Elisabeth Stur||BOLD:AAB0734
Heleniella ornaticollis|ES28|Elisabeth Stur||BOLD:AAB0734
Heleniella ornaticollis|ES30|Elisabeth Stur||BOLD:AAB0734
Heleniella ornaticollis|ES29|Elisabeth Stur||BOLD:AAB0734
Heleniella ornaticollis|ES32|Elisabeth Stur||BOLD:AAB0734
Heleniella ornaticollis|SOE159|Elisabeth Stur||BOLD:AAB0734
Heleniella ornaticollis|ES31|Elisabeth Stur||BOLD:AAB0734
Heleniella ornaticollis|SOE178|Elisabeth Stur||BOLD:AAB0734
Heleniella ornaticollis|SOE177|Elisabeth Stur||BOLD:AAB0734
Heleniella ornaticollis|SOE288|Elisabeth Stur||BOLD:AAB0734
Heleniella ornaticollis|SOE250|Elisabeth Stur||BOLD:AAB0734
Heleniella ornaticollis|SOE291|Elisabeth Stur||BOLD:AAB0734
Heleniella ornaticollis|SOE289|Elisabeth Stur||BOLD:AAB0734
Heleniella ornaticollis|SOE301|Elisabeth Stur||BOLD:AAB0734
Heleniella ornaticollis|SOE302|Elisabeth Stur||BOLD:AAB0734
Heleniella ornaticollis|SOE305|Elisabeth Stur||BOLD:AAB0734
Heleniella ornaticollis|SOE459|Elisabeth Stur||BOLD:AAB0734
Heleniella ornaticollis|SOE328|Elisabeth Stur||BOLD:AAB0734
Heleniella ornaticollis|SOE333|Elisabeth Stur||BOLD:AAB0734
Heleniella ornaticollis|SOE358|Elisabeth Stur||BOLD:AAB0734
Heleniella ornaticollis|SOE360|Elisabeth Stur||BOLD:AAB0734
Heleniella ornaticollis|SOE371|Elisabeth Stur||BOLD:AAB0734
Heleniella ornaticollis|SOE181|Elisabeth Stur||BOLD:AAB0734
Heleniella ornaticollis|SOE147|Elisabeth Stur||BOLD:AAB0734
Heleniella ornaticollis|SOE337|Elisabeth Stur||BOLD:AAB0734
Heleniella ornaticollis|SOE457|Elisabeth Stur||BOLD:AAB0734
Heleniella ornaticollis|SOE478|Elisabeth Stur||BOLD:AAB0734
Heleniella ornaticollis|SOE420|Elisabeth Stur||BOLD:AAB0734
Heleniella ornaticollis|SOE108|Torbjorn Ekrem||BOLD:AAB0734
Heleniella ornaticollis|SOE406|Elisabeth Stur||BOLD:AAB0734
Heleniella ornaticollis|SOE105|Torbjorn Ekrem||BOLD:AAB0734
Smittia reissi|ZSM-DIP-33132-H04|Caroline Chimeno|BIN Taxonomy Match (Jan 2022)|BOLD:ACS...
Smittia reissi|ZSM-DIP-33143-B09|Caroline Chimeno|BIN Taxonomy Match (Jan 2022)|BOLD:ACS...
Smittia reissi|ZSM-DIP-33132-H06|Caroline Chimeno|BIN Taxonomy Match (Jan 2022)|BOLD:ACS...
Smittia reissi|ZSM-DIP-33131-B04|Caroline Chimeno|BIN Taxonomy Match (Jan 2022)|BOLD:ACS...
Smittia reissi|ZSM-DIP-33132-D09|Caroline Chimeno|BIN Taxonomy Match (Jan 2022)|BOLD:ACS...
Smittia reissi|ZSM34343-G07|Sofia Wiedenbrug|Morphology|BOLD:ACS9748
Smittia reissi|ZSM34343-F06|Sofia Wiedenbrug||BOLD:ACS9748
Smittia reissi|CCDB22605-A03|Sofia Wiedenbrug||BOLD:ACS9748
Smittia reissi|GBOL11537|Sofia Wiedenbrug|Morphology|BOLD:ACS9748
Smittia reissi|CCDB22605-A02|Sofia Wiedenbrug||BOLD:ACS9748
Smittia reissi|ZSM-DIP-33132-H03|Caroline Chimeno|BIN Taxonomy Match (Jan 2022)|BOLD:ACU...
Smittia reissi|ZSM-DIP-33141-C02|Caroline Chimeno|BIN Taxonomy Match (Jan 2022)|BOLD:ACU...
Smittia reissi|CCDB24037-A07|Sofia Wiedenbrug|BIN Taxonomy Match|BOLD:ACU4112
Smittia reissi|ZSM34342-A12|Sofia Wiedenbrug|Morphology|BOLD:ACU4112
Smittia reissi|ZSM34341-C09|Sofia Wiedenbrug|Morphology|BOLD:ACU4112
Smittia reissi|ZSM-DIP-33130-B09|Caroline Chimeno|BIN Taxonomy Match (Jan 2022)|BOLD:ACU...
Smittia reissi|ZSM34341-C11|Sofia Wiedenbrug|Morphology|BOLD:ACU4112
Smittia reissi|CCDB24037-A10|Sofia Wiedenbrug|BIN Taxonomy Match|BOLD:ACU4112
Smittia reissi|ZSM-DIP-33132-C12|Caroline Chimeno|BIN Taxonomy Match (Jan 2022)|BOLD:ACU...
Smittia reissi|CCDB24039-D04|Sofia Wiedenbrug|BIN Taxonomy Match|BOLD:ACU4112
Smittia reissi|CCDB24039-H02|Sofia Wiedenbrug|BIN Taxonomy Match|BOLD:ACU4112
Chironomidae|CCDB22604-F04|Sofia Wiedenbrug|no morphological identification - introduction of int...
Chironomidae|BIOUG16738-D12|Kate Perez|no morphological identification - introduction of interim ...
Chironomidae|BIOUG16776-D01|Kate Perez|no morphological identification - introduction of interim ...
Chironomidae|BIOUG16740-E09|Kate Perez|no morphological identification - introduction of interim ...
Chironomidae|BIOUG16738-A10|Kate Perez|no morphological identification - introduction of interim ...
Chironomidae|BIOUG16785-C11|Kate Perez|no morphological identification - introduction of interim ...
Chironomidae|BIOUG16785-E02|Kate Perez|no morphological identification - introduction of interim ...
Chironomidae|BIOUG16785-C05|Kate Perez|no morphological identification - introduction of interim ...
Chironomidae|BIOUG16893-F11|Kate Perez|no morphological identification - introduction of interim ...
Chironomidae|BIOUG16776-A11|Kate Perez|no morphological identification - introduction of interim ...
Chironomidae|BIOUG16782-G09|Kate Perez|no morphological identification - introduction of interim ...
Chironomidae|BIOUG16782-B06|Kate Perez|no morphological identification - introduction of interim ...
Rheocricotopus sp. 1SW|CCDB22605-B11|Sofia Wiedenbrug||BOLD:ACT2193
Rheocricotopus sp. 1SW|GBOL11572|Sofia Wiedenbrug|Morphology|BOLD:ACT2193

[illegible]
Chironomidae|BIOUG16782-B06|Kate Perez|no morphological identification - introduction of interim ...
Rheocricotopus sp. 1SW|CCDB22605-B11|Sofia Wiedenbrug||BOLD:ACT2193
Rheocricotopus sp. 1SW|GBOL11572|Sofia Wiedenbrug|Morphology|BOLD:ACT2193
Rheocricotopus sp. 1SW|GBOL11571|Sofia Wiedenbrug|Morphology|BOLD:ACT2193
Rheocricotopus sp. 1SW|CCDB22605-B09|Sofia Wiedenbrug||BOLD:ACT2193
Rheocricotopus sp. 1SW|CCDB22605-B08|Sofia Wiedenbrug||BOLD:ACT2193
Rheocricotopus sp. 1SW|CCDB22605-H02|Sofia Wiedenbrug||BOLD:ACT2193
Rheocricotopus sp. 1SW|CCDB21605-C10|Sofia Wiedenbrug||BOLD:ACT2193
Rheocricotopus sp. 1SW|CCDB21605-D03|Sofia Wiedenbrug||BOLD:ACT2193
Rheocricotopus sp. 1SW|CCDB21605-C12|Sofia Wiedenbrug||BOLD:ACT2193
Rheocricotopus sp. 1SW|CCDB21605-D02|Sofia Wiedenbrug||BOLD:ACT2193
Rheocricotopus sp. 1SW|CCDB21605-D01|Sofia Wiedenbrug||BOLD:ACT2193
Rheocricotopus sp. 1SW|CCDB21605-B07|Sofia Wiedenbrug||BOLD:ACT2193
Rheocricotopus sp. 1SW|CCDB21605-C11|Sofia Wiedenbrug||BOLD:ACT2193
Paracricotopus|BIOUG16259-C09|Elisabeth Stur|BOLD ID Engine Manual|BOLD:ACQ9911
Paracricotopus|ZSM-DIP-33144-A06|Caroline Chimeno|BIN Taxonomy Match (Jan 2022)|BOLD:AEK...
Paracricotopus|BIOUG16480-E12|Elisabeth Stur|BOLD ID Engine Manual|BOLD:AEK0260
Paracricotopus|BIOUG16479-D02|Elisabeth Stur|BOLD ID Engine Manual|BOLD:ACQ8932
Paracricotopus|BIOUG16260-E10|Elisabeth Stur|BOLD ID Engine Manual|BOLD:ACQ8932
Paracricotopus sp. 1SW|CCDB22605-H08|Sofia Wiedenbrug||BOLD:ACQ5125
Paracricotopus niger|BIOUG16626-F08|Elisabeth Stur|BIN Taxonomy Match|BOLD:ACQ5125
Paracricotopus niger|ATNA572|Elisabeth Stur||BOLD:ACQ5125
Paracricotopus niger|BIOUG16478-D12|Elisabeth Stur|BIN Taxonomy Match|BOLD:ACQ5125
Paracricotopus niger|BIOUG15947-C12|Elisabeth Stur|BIN Taxonomy Match|BOLD:ACQ5125
Paracricotopus niger|BIOUG16597-C05|Elisabeth Stur|BIN Taxonomy Match|BOLD:ACQ5125
Paracricotopus|SOE370|Torbjorn Ekrem||BOLD:AAI2561
Bryophaenocladius|CCDB24229-B11|Sofia Wiedenbrug|BIN Taxonomy Match|BOLD:ACU4813
Chironomidae|GBOL03764|Sofia Wiedenbrug
Paracricotopus uliginosus|SOE431|Elisabeth Stur||BOLD:AAC7862
Paracricotopus uliginosus|SOE22|Elisabeth Stur||BOLD:AAC7862
Paracricotopus uliginosus|SOE189|Elisabeth Stur||BOLD:AAC7862
Paracricotopus uliginosus|SOE428|Elisabeth Stur||BOLD:AAC7862
Paracricotopus uliginosus|SOE187|Elisabeth Stur||BOLD:AAC7862
Paracricotopus uliginosus|SOE279|Elisabeth Stur||BOLD:AAC7862
Paracricotopus uliginosus|SOE421|Elisabeth Stur||BOLD:AAC7862
Paracricotopus uliginosus|SOE287|Elisabeth Stur||BOLD:AAC7862
Paracricotopus uliginosus|SOE438|Torbjorn Ekrem||BOLD:AAC7862
Paracricotopus uliginosus|SOE475|Elisabeth Stur||BOLD:AAC7862
Paracricotopus uliginosus|SOE442|Elisabeth Stur||BOLD:AAC7862
Paracricotopus uliginosus|SOE423|Elisabeth Stur||BOLD:AAC7862
Paracricotopus uliginosus|SOE107|Elisabeth Stur||BOLD:AAC7862
Paracladius|ZSM-DIP-33142-H08|Caroline Chimeno|BIN Taxonomy Match (Jan 2022)|BOLD:ACU5214
Paracladius|ZSM-DIP-33146-C01|Caroline Chimeno|BIN Taxonomy Match (Jan 2022)|BOLD:ACU5214
Paracladius|ZSM-DIP-33149-G06|Caroline Chimeno|BIN Taxonomy Match (Jan 2022)|BOLD:ACU5214
Paracladius|GBOL11567|Sofia Wiedenbrug|Morphology|BOLD:ACU5214
Orthocladiinae|BIOUG04085-A08|Kate Perez|BIN Taxonomy Match|BOLD:AAP1832
Orthocladiinae|BIOUG04261-E06|Kate Perez|BIN Taxonomy Match|BOLD:AAP1832
Orthocladiinae|BIOUG04261-E11|Kate Perez|BIN Taxonomy Match|BOLD:AAP1832
Orthocladiinae|BIOUG04261-F06|Kate Perez|BIN Taxonomy Match|BOLD:AAP1832
Orthocladiinae|BIOUG04261-C03|Kate Perez|BIN Taxonomy Match|BOLD:AAP1832
Orthocladiinae|BIOUG04261-D08|Kate Perez|BIN Taxonomy Match|BOLD:AAP1832
Orthocladiinae|BIOUG04261-C08|Kate Perez|BIN Taxonomy Match|BOLD:AAP1832
Orthocladiinae|BIOUG04261-C07|Kate Perez|BIN Taxonomy Match|BOLD:AAP1832
Orthocladiinae|BIOUG04261-C04|Kate Perez|BIN Taxonomy Match|BOLD:AAP1832
Orthocladiinae|BIOUG04085-C02|Kate Perez|BIN Taxonomy Match|BOLD:AAP1832
Orthocladiinae|BIOUG04261-B06|Kate Perez|BIN Taxonomy Match|BOLD:AAP1832
Orthocladiinae|BIOUG04261-B07|Kate Perez|BIN Taxonomy Match|BOLD:AAP1832
Chironomidae|Koz1192|Mikhail V. Kozlov||BOLD:AAP1832
Orthocladiinae|BIOUG04261-C06|Kate Perez|BIN Taxonomy Match|BOLD:AAP1832
Orthocladiinae|BIOUG04261-E03|Kate Perez|BIN Taxonomy Match|BOLD:AAP1832
Orthocladiinae|BIOUG04261-D01|Kate Perez|BIN Taxonomy Match|BOLD:AAP1832
Orthocladiinae|BIOUG04261-C01|Kate Perez|BIN Taxonomy Match|BOLD:AAP1832
Orthocladiinae|BIOUG04261-B08|Kate Perez|BIN Taxonomy Match|BOLD:AAP1832
Orthocladiinae|BIOUG04261-B11|Kate Perez|BIN Taxonomy Match|BOLD:AAP1832
Orthocladiinae|BIOUG04261-D05|Kate Perez|BIN Taxonomy Match|BOLD:AAP1832
Orthocladiinae|BIOUG04261-E07|Kate Perez|BIN Taxonomy Match|BOLD:AAP1832
Orthocladiinae|BIOUG15544-G08|Kate Perez|BOLD ID Engine Manual|BOLD:AAP1832
Orthocladiinae|24415-A02|Kate Perez|BIN Taxonomy Match|BOLD:AAP1832
Orthocladiinae|BIOUG15537-G09|Kate Perez|BOLD ID Engine Manual|BOLD:AAP1832
Rheocricotopus fuscipes|ZSM-DIP-33146-A07|Caroline Chimeno|BIN Taxonomy Match (Jan 2022)|BO...
Rheocricotopus fuscipes|CCDB24183-E02|Sofia Wiedenbrug|Morphology|BOLD:AAV2322
Rheocricotopus fuscipes|CH-OSF137|Elisabeth Stur||BOLD:AAV2322
Rheocricotopus fuscipes|BIOUG16713-C04|Kate Perez|BIN Taxonomy Match|BOLD:AAV2322
Rheocricotopus fuscipes|BIOUG16631-B09|Kate Perez|BIN Taxonomy Match|BOLD:AAV2322
Rheocricotopus fuscipes|ZSM-DIP-33142-F03|Caroline Chimeno|BIN Taxonomy Match (Jan 2022)|BO...
Rheocricotopus fuscipes|BIOUG17022-G03|Kate Perez|BIN Taxonomy Match|BOLD:AAV2322
Rheocricotopus fuscipes|BIOUG16332-D08|Kate Perez|BIN Taxonomy Match|BOLD:AAV2322
Rheocricotopus fuscipes|BIOUG16713-G10|Kate Perez|BIN Taxonomy Match|BOLD:AAV2322
Rheocricotopus fuscipes|BIOUG16632-B05|Kate Perez|BIN Taxonomy Match|BOLD:AAV2322
Rheocricotopus fuscipes|BIOUG15539-H03|Kate Perez|BIN Taxonomy Match|BOLD:AAV2322
Rheocricotopus fuscipes|BIOUG14592-D01|Kate Perez|BIN Taxonomy Match|BOLD:AAV2322
Rheocricotopus fuscipes|BIOUG16433-H09|Kate Perez|BIN Taxonomy Match|BOLD:AAV2322
Rheocricotopus fuscipes|BIOUG15544-G04|Kate Perez|BIN Taxonomy Match|BOLD:AAV2322
Rheocricotopus fuscipes|BIOUG15947-C04|Kate Perez|BIN Taxonomy Match|BOLD:AAV2322
Rheocricotopus fuscipes|ZMUO.024640|Lauri Paasivirta||BOLD:AAV2322
Rheocricotopus fuscipes|CH-OSF22|Elisabeth Stur||BOLD:AAV2322
Rheocricotopus fuscipes|BIOUG15567-G04|Kate Perez|BIN Taxonomy Match|BOLD:AAV2322
Rheocricotopus fuscipes|ZMUO.024641|Lauri Paasivirta||BOLD:AAV2322
Rheocricotopus fuscipes|TRD-CH168|Elisabeth Stur||BOLD:AAV2322
Rheocricotopus fuscipes|Finnmark894|Elisabeth Stur||BOLD:AAV2322
Rheocricotopus fuscipes|Finnmark766|Elisabeth Stur||BOLD:AAV2322
Rheocricotopus fuscipes|CH-OSF138|Elisabeth Stur||BOLD:AAV2322
Rheocricotopus fuscipes|Finnmark77|Elisabeth Stur||BOLD:AAV2322
Rheocricotopus fuscipes|EBAI-Ch217|Elisabeth Stur|morphology|BOLD:AAM7067
Rheocricotopus fuscipes|TRD-CH301|Elisabeth Stur||BOLD:AAM7067
Rheocricotopus fuscipes|Finnmark12|Elisabeth Stur||BOLD:AAM7067
Rheocricotopus fuscipes|EBAI-Ch240|Elisabeth Stur|morphology
Rheocricotopus fuscipes|EBAI-Ch216|Elisabeth Stur|morphology|BOLD:AAM7067
Rheocricotopus fuscipes|Finnmark786|Elisabeth Stur||BOLD:AAM7067
Rheocricotopus fuscipes|Finnmark559|Elisabeth Stur||BOLD:AAM7067
Nanocladius rectinervis|ZMUO.025944|Lauri Paasivirta||BOLD:ADF1388
Nanocladius rectinervis|BIOUG17099-H08|Kate Perez|BIN Taxonomy Match|BOLD:ACQ3439
[illegible]

Rheocricotopus fuscipes|Finnmark559|Elisabeth Stur||BOLD:AAM7067
Nanocladius rectinervis|ZMUO.025944|Lauri Paasivirta||BOLD:ADF1388
Nanocladius rectinervis|BIOUG17099-H08|Kate Perez|BIN Taxonomy Match|BOLD:ACQ3439
Nanocladius rectinervis|TRD-CH385|Elisabeth Stur|morphology|BOLD:ACQ3439
Nanocladius|ZMUO.026243|Lauri Paasivirta||BOLD:ACQ3439
Nanocladius rectinervis|ZMUO.024682|Lauri Paasivirta||BOLD:ACQ3439
Nanocladius rectinervis|ZMUO.024683|Lauri Paasivirta||BOLD:ACQ3439
Nanocladius rectinervis|BIOUG16431-H02|Elisabeth Stur|BOLD ID Engine Manual|BOLD:ACQ3439
Nanocladius rectinervis|BIOUG16258-G05|Elisabeth Stur|BOLD ID Engine Manual|BOLD:ACQ3439
Nanocladius rectinervis|BIOUG15954-C05|Elisabeth Stur|BOLD ID Engine Manual|BOLD:ACQ3439
Nanocladius rectinervis|BIOUG15950-C11|Elisabeth Stur|BOLD ID Engine Manual|BOLD:ACQ3439
Nanocladius dichromus|EBAI-Ch114|Elisabeth Stur|morphology|BOLD:ADA7703
Nanocladius dichromus|Finnmark834|Elisabeth Stur||BOLD:AAF7613
Nanocladius dichromus|EBAI-Ch113|Elisabeth Stur|morphology|BOLD:AAF7613
Nanocladius dichromus|Finnmark826|Elisabeth Stur||BOLD:AAF7613
Nanocladius dichromus|Finnmark821|Elisabeth Stur||BOLD:AAF7613
Nanocladius dichromus|ATNA240|Elisabeth Stur||BOLD:AAF7613
Nanocladius dichromus|Finnmark247|Elisabeth Stur||BOLD:AAF7613
Nanocladius dichromus|Finnmark518|Elisabeth Stur||BOLD:AAF7613
Nanocladius dichromus|Finnmark414|Elisabeth Stur||BOLD:AAF7613
Orthocladius schnelli|ATNA227|Elisabeth Stur||BOLD:AAF0462
Orthocladius schnelli|ZSM-DIP-33141-F09|Caroline Chimeno|BIN Taxonomy Match (Jan 2022)|BOLD...
Orthocladius schnelli|ATNA436|Elisabeth Stur||BOLD:AAF0462
Orthocladius schnelli|ATNA435|Elisabeth Stur||BOLD:AAF0462
Orthocladius sp. 12TE|CH-OSF71|Torbjorn Ekrem||BOLD:AAV5074
Orthocladius|BIOUG15460-G02|Kate Perez|BIN Taxonomy Match|BOLD:AAV5074
Orthocladius|BIOUG15459-B12|Kate Perez|BIN Taxonomy Match|BOLD:AAV5074
Orthocladius sp. 12TE|CH-OSF136|Torbjorn Ekrem||BOLD:AAV5074
Orthocladius sp. 12TE|CH-OSF105|Torbjorn Ekrem||BOLD:AAV5074
Orthocladius rhyacobius|ZMUO.024568|Lauri Paasivirta||BOLD:AAV5074
Orthocladius nr. rhyacobius|ZMUO.024529|Lauri Paasivirta||BOLD:AAV5074
Orthocladius rhyacobius|ZMUO.024569|Lauri Paasivirta||BOLD:AAV5074
Orthocladius sp. 12TE|CCDB24183-B03|Sofia Wiedenbrug|BIN-taxonomy|BOLD:AAV5074
Orthocladius sp. 12TE|TRD-CH58|Elisabeth Stur||BOLD:AAV5074
Orthocladius sp. 12TE|CH-OSF64|Elisabeth Stur||BOLD:AAV5074
Orthocladius sp. 12TE|CH-OSF23|Torbjorn Ekrem||BOLD:AAV5074
Orthocladius lignicola|CCDB22605-F03|Sofia Wiedenbrug||BOLD:ACK5239
Orthocladius lignicola|CCDB22605-F02|Sofia Wiedenbrug||BOLD:ACK5239
Orthocladius lignicola|BIOUG07503-E08|Kate Perez|BOLD ID Engine Manual|BOLD:ACK5239
Orthocladius schnelli|ZMUO.025982|Lauri Paasivirta||BOLD:ADF2482
Orthocladius dentifer|ATNA225|Elisabeth Stur||BOLD:AAK6023
Orthocladius dentifer|BIOUG15184-E08|Kate Perez|BIN Taxonomy Match|BOLD:AAK6023
Orthocladius dentifer|BIOUG15455-C08|Kate Perez|BIN Taxonomy Match|BOLD:AAK6023
Orthocladius nitidoscutellatus|BIOUG17114-H05|Kate Perez|BIN Taxonomy Match|BOLD:ACD6068
Orthocladius nitidoscutellatus|BIOUG16779-A01|Kate Perez|BIN Taxonomy Match|BOLD:ACD6068
Orthocladius nitidoscutellatus|BIOUG16777-H11|Kate Perez|BIN Taxonomy Match|BOLD:ACD6068
Orthocladius nitidoscutellatus|BIOUG16893-B08|Kate Perez|BIN Taxonomy Match|BOLD:ACD6068
Orthocladius nitidoscutellatus|BIOUG16777-B11|Kate Perez|BIN Taxonomy Match|BOLD:ACD6068
Orthocladius nitidoscutellatus|BIOUG16996-B09|Kate Perez|BIN Taxonomy Match|BOLD:ACD6068
Orthocladius nitidoscutellatus|BIOUG16739-B05|Kate Perez|BIN Taxonomy Match|BOLD:ACD6068
Orthocladius wetterensis|ZMUO.026120|Lauri Paasivirta||BOLD:ADC3605
Orthocladius wetterensis|ZSM-DIP-33172-D03|Caroline Chimeno|BIN Taxonomy Match (Jan 2022)|B...
Orthocladius wetterensis|ZSM-DIP-33133-C08|Caroline Chimeno|BIN Taxonomy Match (Jan 2022)|B...
Orthocladius|CCDB24183-A07|Sofia Wiedenbrug|Morphology|BOLD:ADC3605
Orthocladius nitidoscutellatus|ES381|Elisabeth Stur||BOLD:AEN9253
Orthocladius nitidoscutellatus|Finnmark151|Elisabeth Stur||BOLD:AAB0435
Nanocladius|BIOUG16476-H07|Elisabeth Stur|BOLD ID Engine Manual|BOLD:ACQ6655
Nanocladius|BIOUG16557-F10|Elisabeth Stur|morphology|BOLD:ACQ6655
Nanocladius|BIOUG16259-B06|Elisabeth Stur|morphology|BOLD:ACQ6655
Nanocladius|BIOUG15965-C11|Elisabeth Stur|morphology|BOLD:ACQ6655
Nanocladius|BIOUG16430-D11|Elisabeth Stur|morphology|BOLD:ACQ6655
Nanocladius|BIOUG16479-E03|Elisabeth Stur|morphology|BOLD:ACQ6655
Nanocladius|BIOUG16557-H06|Elisabeth Stur|morphology|BOLD:ACQ6655
Nanocladius|BIOUG16479-F10|Elisabeth Stur|morphology|BOLD:ACQ6655
Nanocladius|BIOUG16557-F04|Elisabeth Stur|morphology|BOLD:ACQ6655
Nanocladius|BIOUG16552-E11|Elisabeth Stur|morphology|BOLD:ACQ6655
Nanocladius|BIOUG15905-G09|Elisabeth Stur|morphology|BOLD:ACQ6655
Rheocricotopus chalybeatus|ZMUO.025284|Lauri Paasivirta||BOLD:ADA4140
Rheocricotopus chalybeatus|ZMUO.025283|Lauri Paasivirta||BOLD:AAW4987
Rheocricotopus chalybeatus|ZSM-DIP-33134-F11|Caroline Chimeno|BIN Taxonomy Match (Jan 2022)
Rheocricotopus chalybeatus|EJV-20110180|Lauri Paasivirta||BOLD:AAW4987
Rheocricotopus chalybeatus|EJV-20110179|Lauri Paasivirta||BOLD:AAW4987
Rheocricotopus chalybeatus|EJV-20110177|Lauri Paasivirta||BOLD:AAW4987
Orthocladius rubicundus|ZMUO.026308|Lauri Paasivirta||BOLD:ADZ9170
Orthocladius sp. 18TE|Finnmark822|Elisabeth Stur||BOLD:AEA0009
Orthocladius rubicundus|EJV-20110216|Lauri Paasivirta||BOLD:AEA0009
Orthocladius rubicundus|BIOUG15967-D12|Kate Perez|BIN Taxonomy Match|BOLD:AAW5449
Orthocladius rubicundus|BIOUG15807-E08|Kate Perez|BIN Taxonomy Match|BOLD:AAW5449
Orthocladius sp. 18TE|Finnmark879|Torbjorn Ekrem||BOLD:AAW5449
Orthocladius rubicundus|EJV-20110215|Lauri Paasivirta||BOLD:AAW5449
Orthocladius rubicundus|BIOUG16476-G11|Kate Perez|BIN Taxonomy Match|BOLD:AAW5449
Orthocladius rubicundus|BIOUG15799-A02|Kate Perez|BIN Taxonomy Match|BOLD:AAW5449
Orthocladius rubicundus|BIOUG15793-H03|Kate Perez|BIN Taxonomy Match|BOLD:AAW5449
Orthocladius rubicundus|BIOUG16476-D12|Kate Perez|BIN Taxonomy Match|BOLD:AAW5449
Orthocladius rubicundus|BIOUG15852-D11|Kate Perez|BIN Taxonomy Match|BOLD:AAW5449
Orthocladius rubicundus|BIOUG15720-E05|Kate Perez|BIN Taxonomy Match|BOLD:AAW5449
Orthocladius rubicundus|BIOUG15798-F05|Kate Perez|BIN Taxonomy Match|BOLD:AAW5449
Orthocladius rubicundus|BIOUG15792-A09|Kate Perez|BIN Taxonomy Match|BOLD:AAW5449
Orthocladius rubicundus|BIOUG15795-G09|Kate Perez|BIN Taxonomy Match|BOLD:AAW5449
Orthocladius rubicundus|BIOUG15809-C03|Kate Perez|BIN Taxonomy Match|BOLD:AAW5449
Orthocladius rubicundus|BIOUG16713-G02|Kate Perez|BIN Taxonomy Match|BOLD:AAW5449
Orthocladius rubicundus|BIOUG15796-E11|Kate Perez|BIN Taxonomy Match|BOLD:AAW5449
Orthocladius rubicundus|BIOUG15800-H01|Kate Perez|BIN Taxonomy Match|BOLD:AAW5449
Orthocladius rubicundus|BIOUG15791-H02|Kate Perez|BIN Taxonomy Match|BOLD:AAW5449
Orthocladius rubicundus|BIOUG15796-E03|Kate Perez|BIN Taxonomy Match|BOLD:AAW5449
Orthocladius rubicundus|BIOUG15766-E04|Kate Perez|BIN Taxonomy Match|BOLD:AAW5449
Orthocladius rubicundus|BIOUG15761-C05|Kate Perez|BIN Taxonomy Match|BOLD:AAW5449
Orthocladius rubicundus|BIOUG15766-D12|Kate Perez|BIN Taxonomy Match|BOLD:AAW5449
Orthocladius rubicundus|BIOUG15795-E01|Kate Perez|BIN Taxonomy Match|BOLD:AAW5449
Orthocladius rubicundus|BIOUG15718-B09|Kate Perez|BIN Taxonomy Match|BOLD:AAW5449
Orthocladius|BIOUG15718-E04|Elisabeth Stur|BOLD ID Engine Manual|BOLD:ACP8092
Orthocladius rubicundus|Finnmark820|Elisabeth Stur||BOLD:AAM5389
Orthocladius rubicundus|ZSM-DIP-33128-G02|Caroline Chimeno|BIN Taxonomy Match (Jan 2022)|BO...

Orthocladius|BIOUG15718-E04|Elisabeth Stur|BOLD ID Engine Manual|BOLD:ACP8092
Orthocladius rubicundus|Finnmark820|Elisabeth Stur||BOLD:AAM5389
Orthocladius rubicundus|ZSM-DIP-33128-G02|Caroline Chimeno|BIN Taxonomy Match (Jan 2022)|BO...
Orthocladius rubicundus|ZSM-DIP-33170-C02|Caroline Chimeno|BIN Taxonomy Match (Jan 2022)|BO...
Orthocladius rubicundus|ZSM-DIP-33170-D05|Caroline Chimeno|BIN Taxonomy Match (Jan 2022)|BO...
Orthocladius rubicundus|BIOUG16856-D11|Kate Perez|BIN Taxonomy Match|BOLD:AAM5389
Orthocladius rubicundus|BIOUG16854-F06|Kate Perez|BIN Taxonomy Match|BOLD:AAM5389
Orthocladius rubicundus|BIOUG16857-G12|Kate Perez|Tree base identification
Orthocladius rubicundus|ZMUO.024716|Lauri Paasivirta||BOLD:AAM5389
Orthocladius rubicundus|ZSM-DIP-33134-G07|Caroline Chimeno|BIN Taxonomy Match (Jan 2022)|BO...
Orthocladius rubicundus|BIOUG15853-H03|Kate Perez|BIN Taxonomy Match|BOLD:AAM5389
Orthocladius rubicundus|BIOUG15852-B05|Kate Perez|BIN Taxonomy Match|BOLD:AAM5389
Orthocladius rubicundus|BIOUG15718-G06|Kate Perez|BIN Taxonomy Match|BOLD:AAM5389
Orthocladius rubicundus|BIOUG15853-D10|Kate Perez|BIN Taxonomy Match|BOLD:AAM5389
Orthocladius rubicundus|BIOUG15853-F07|Kate Perez|BIN Taxonomy Match|BOLD:AAM5389
Orthocladius rubicundus|BIOUG15542-C09|Kate Perez|BIN Taxonomy Match|BOLD:AAM5389
Orthocladius rubicundus|BIOUG15649-G07|Kate Perez|BIN Taxonomy Match|BOLD:AAM5389
Orthocladius rubicundus|BIOUG16477-D02|Kate Perez|BIN Taxonomy Match|BOLD:AAM5389
Orthocladius rubicundus|BIOUG15719-F11|Kate Perez|BIN Taxonomy Match|BOLD:AAM5389
Orthocladius rubicundus|BIOUG15800-G09|Kate Perez|BIN Taxonomy Match|BOLD:AAM5389
Orthocladius rubicundus|BIOUG16330-C09|Kate Perez|BIN Taxonomy Match|BOLD:AAM5389
Orthocladius rubicundus|BIOUG15718-H02|Kate Perez|BIN Taxonomy Match|BOLD:AAM5389
Orthocladius rubicundus|BIOUG16435-H06|Kate Perez|BIN Taxonomy Match|BOLD:AAM5389
Orthocladius rubicundus|BIOUG16711-F01|Kate Perez|BIN Taxonomy Match|BOLD:AAM5389
Orthocladius rubicundus|BIOUG17022-A11|Kate Perez|BIN Taxonomy Match|BOLD:AAM5389
Orthocladius rubicundus|BIOUG15719-B10|Kate Perez|BIN Taxonomy Match|BOLD:AAM5389
Orthocladius rubicundus|BIOUG15720-A02|Kate Perez|BIN Taxonomy Match|BOLD:AAM5389
Orthocladius rubicundus|BIOUG15806-C03|Kate Perez|BIN Taxonomy Match|BOLD:AAM5389
Orthocladius rubicundus|BIOUG15964-C06|Kate Perez|BIN Taxonomy Match|BOLD:AAM5389
Orthocladius rubicundus|BIOUG15808-B08|Kate Perez|BIN Taxonomy Match|BOLD:AAM5389
Orthocladius rubicundus|BIOUG16259-C02|Kate Perez|BIN Taxonomy Match|BOLD:AAM5389
Orthocladius rubicundus|BIOUG15653-E06|Kate Perez|BIN Taxonomy Match|BOLD:AAM5389
Orthocladius rubicundus|BIOUG16477-F12|Kate Perez|BIN Taxonomy Match|BOLD:AAM5389
Orthocladius rubicundus|BIOUG16712-A03|Kate Perez|BIN Taxonomy Match|BOLD:AAM5389
Orthocladius rubicundus|BIOUG15761-H03|Kate Perez|BIN Taxonomy Match|BOLD:AAM5389
Orthocladius rubicundus|BIOUG16552-D11|Kate Perez|BIN Taxonomy Match|BOLD:AAM5389
Orthocladius rubicundus|BIOUG15796-A09|Kate Perez|BIN Taxonomy Match|BOLD:AAM5389
Orthocladius rubicundus|BIOUG15800-B02|Kate Perez|BIN Taxonomy Match|BOLD:AAM5389
Orthocladius rubicundus|BIOUG16430-F07|Kate Perez|BIN Taxonomy Match|BOLD:AAM5389
Orthocladius rubicundus|BIOUG16711-D06|Kate Perez|BIN Taxonomy Match|BOLD:AAM5389
Orthocladius rubicundus|BIOUG16712-A12|Kate Perez|BIN Taxonomy Match|BOLD:AAM5389
Orthocladius rubicundus|BIOUG17025-G04|Kate Perez|BIN Taxonomy Match|BOLD:AAM5389
Orthocladius rubicundus|BIOUG17064-F08|Kate Perez|BIN Taxonomy Match|BOLD:AAM5389
Orthocladius rubicundus|BIOUG16299-G03|Kate Perez|BIN Taxonomy Match|BOLD:AAM5389
Orthocladius rubicundus|BIOUG16205-C10|Kate Perez|BIN Taxonomy Match|BOLD:AAM5389
Orthocladius rubicundus|BIOUG16632-F12|Kate Perez|BIN Taxonomy Match|BOLD:AAM5389
Orthocladius rubicundus|BIOUG15543-G08|Kate Perez|BIN Taxonomy Match|BOLD:AAM5389
Orthocladius rubicundus|BIOUG16258-A12|Kate Perez|BIN Taxonomy Match|BOLD:AAM5389
Orthocladius rubicundus|BIOUG15653-A05|Kate Perez|BIN Taxonomy Match|BOLD:AAM5389
Orthocladius rubicundus|BIOUG17022-H02|Kate Perez|BIN Taxonomy Match|BOLD:AAM5389
Orthocladius rubicundus|BIOUG15593-E08|Kate Perez|BIN Taxonomy Match|BOLD:AAM5389
Orthocladius rubicundus|BIOUG15545-A06|Kate Perez|BIN Taxonomy Match|BOLD:AAM5389
Orthocladius rubicundus|BIOUG15796-D08|Kate Perez|BIN Taxonomy Match|BOLD:AAM5389
Orthocladius rubicundus|BIOUG16259-D05|Kate Perez|BIN Taxonomy Match|BOLD:AAM5389
Orthocladius rubicundus|BIOUG16435-H02|Kate Perez|BIN Taxonomy Match|BOLD:AAM5389
Orthocladius rubicundus|BIOUG16331-E03|Kate Perez|BIN Taxonomy Match|BOLD:AAM5389
Orthocladius rubicundus|BIOUG15595-E10|Kate Perez|BIN Taxonomy Match|BOLD:AAM5389
Orthocladius rubicundus|BIOUG16480-H05|Kate Perez|BIN Taxonomy Match|BOLD:AAM5389
Orthocladius rubicundus|BIOUG15950-F06|Kate Perez|BIN Taxonomy Match|BOLD:AAM5389
Orthocladius rubicundus|BIOUG15762-A04|Kate Perez|BIN Taxonomy Match|BOLD:AAM5389
Orthocladius rubicundus|BIOUG15765-B11|Kate Perez|BIN Taxonomy Match|BOLD:AAM5389
Orthocladius rubicundus|BIOUG16297-F05|Kate Perez|BIN Taxonomy Match|BOLD:AAM5389
Orthocladius rubicundus|BIOUG16430-C01|Kate Perez|BIN Taxonomy Match|BOLD:AAM5389
Orthocladius rubicundus|BIOUG15796-E12|Kate Perez|BIN Taxonomy Match|BOLD:AAM5389
Orthocladius rubicundus|BIOUG15652-G06|Kate Perez|BIN Taxonomy Match|BOLD:AAM5389
Orthocladius rubicundus|BIOUG16435-B02|Kate Perez|BIN Taxonomy Match|BOLD:AAM5389
Orthocladius rubicundus|BIOUG15795-D03|Kate Perez|BIN Taxonomy Match|BOLD:AAM5389
Orthocladius rubicundus|BIOUG16625-A04|Kate Perez|BIN Taxonomy Match|BOLD:AAM5389
Orthocladius rubicundus|BIOUG16432-E01|Kate Perez|BIN Taxonomy Match|BOLD:AAM5389
Orthocladius rubicundus|BIOUG16480-F02|Kate Perez|BIN Taxonomy Match|BOLD:AAM5389
Orthocladius rubicundus|BIOUG15536-F02|Kate Perez|BIN Taxonomy Match|BOLD:AAM5389
Orthocladius rubicundus|BIOUG15718-G12|Kate Perez|BIN Taxonomy Match|BOLD:AAM5389
Orthocladius rubicundus|BIOUG15718-D01|Kate Perez|BIN Taxonomy Match|BOLD:AAM5389
Orthocladius rubicundus|BIOUG15764-C06|Kate Perez|BIN Taxonomy Match|BOLD:AAM5389
Orthocladius rubicundus|BIOUG07504-E10|Kate Perez|BIN Taxonomy Match|BOLD:AAM5389
Orthocladius rubicundus|BIOUG15544-D10|Kate Perez|BIN Taxonomy Match|BOLD:AAM5389
Orthocladius rubicundus|BIOUG16632-G02|Kate Perez|BIN Taxonomy Match|BOLD:AAM5389
Orthocladius rubicundus|BIOUG16626-B12|Kate Perez|BIN Taxonomy Match|BOLD:AAM5389
Orthocladius rubicundus|BIOUG16476-F12|Kate Perez|BIN Taxonomy Match|BOLD:AAM5389
Orthocladius rubicundus|BIOUG15797-F04|Kate Perez|BIN Taxonomy Match|BOLD:AAM5389
Orthocladius rubicundus|BIOUG16430-A07|Kate Perez|BIN Taxonomy Match|BOLD:AAM5389
Orthocladius rubicundus|BIOUG15544-F05|Kate Perez|BIN Taxonomy Match|BOLD:AAM5389
Orthocladius rubicundus|BIOUG15762-G07|Kate Perez|BIN Taxonomy Match|BOLD:AAM5389
Orthocladius rubicundus|BIOUG15761-D10|Kate Perez|BIN Taxonomy Match|BOLD:AAM5389
Orthocladius rubicundus|BIOUG15718-D05|Kate Perez|BIN Taxonomy Match|BOLD:AAM5389
Orthocladius rubicundus|BIOUG17119-D12|Kate Perez|BIN Taxonomy Match|BOLD:AAM5389
Orthocladius rubicundus|ZSM-DIP-33128-A08|Caroline Chimeno|BIN Taxonomy Match (Jan 2022)|BO...
Orthocladius rubicundus|Finnmark25|Torbjorn Ekrem||BOLD:AAM5389
Orthocladius rubicundus|Finnmark819|Elisabeth Stur||BOLD:AAM5389
Orthocladius rubicundus|Finnmark829|Elisabeth Stur||BOLD:AAM5389
Orthocladius rubicundus|BIOUG15600-C01|Kate Perez|BIN Taxonomy Match|BOLD:AAM5389
Orthocladius rubicundus|BIOUG15594-A09|Kate Perez|BIN Taxonomy Match|BOLD:AAM5389
Orthocladius rubicundus|BIOUG15800-C04|Kate Perez|BIN Taxonomy Match|BOLD:AAM5389
Orthocladius rubicundus|BIOUG15764-E05|Kate Perez|BIN Taxonomy Match|BOLD:AAM5389
Orthocladius rubicundus|BIOUG15806-G06|Kate Perez|BIN Taxonomy Match|BOLD:AAM5389
Orthocladius rubicundus|BIOUG16596-G04|Kate Perez|BIN Taxonomy Match|BOLD:AAM5389
Orthocladius rubicundus|BIOUG15766-B05|Kate Perez|BIN Taxonomy Match|BOLD:AAM5389
Orthocladius rubicundus|BIOUG16432-C08|Kate Perez|BIN Taxonomy Match|BOLD:AAM5389
Orthocladius rubicundus|BIOUG16205-C01|Kate Perez|BIN Taxonomy Match|BOLD:AAM5389
Orthocladius rubicundus|BIOUG16434-C02|Kate Perez|BIN Taxonomy Match|BOLD:AAM5389
Orthocladius rubicundus|BIOUG16433-H10|Kate Perez|BIN Taxonomy Match|BOLD:AAM5389
Orthocladius rubicundus|BIOUG16632-A09|Kate Perez|BIN Taxonomy Match|BOLD:AAM5389
Orthocladius rubicundus|BIOUG15766-A10|Kate Perez|BIN Taxonomy Match|BOLD:AAM5389

Orthocladius rubicundus|BIOUG16433-H10|Kate Perez|BIN Taxonomy Match|BOLD:AAM5389
Orthocladius rubicundus|BIOUG16632-A09|Kate Perez|BIN Taxonomy Match|BOLD:AAM5389
Orthocladius rubicundus|BIOUG15766-A10|Kate Perez|BIN Taxonomy Match|BOLD:AAM5389
Orthocladius rubicundus|BIOUG15762-C05|Kate Perez|BIN Taxonomy Match|BOLD:AAM5389
Orthocladius rubicundus|BIOUG15761-D07|Kate Perez|BIN Taxonomy Match|BOLD:AAM5389
Orthocladius rubicundus|BIOUG15764-C02|Kate Perez|BIN Taxonomy Match|BOLD:AAM5389
Orthocladius rubicundus|ZMUO.024717|Lauri Paasivirta||BOLD:AAM5389
Orthocladius rubicundus|BIOUG16854-C06|Kate Perez|BIN Taxonomy Match|BOLD:AAM5389
Orthocladius rubicundus|ZSM-DIP-33138-C09|Caroline Chimeno|BIN Taxonomy Match (Jan 2022)|BO...
Orthocladius rubicundus|BIOUG15852-F03|Kate Perez|BIN Taxonomy Match|BOLD:AAM5389
Orthocladius rubicundus|BIOUG16632-F01|Kate Perez|BIN Taxonomy Match|BOLD:AAM5389
Orthocladius rubicundus|BIOUG16632-H08|Kate Perez|BIN Taxonomy Match|BOLD:AAM5389
Orthocladius rubicundus|BIOUG15765-D11|Kate Perez|BIN Taxonomy Match|BOLD:AAM5389
Orthocladius rubicundus|BIOUG15561-E02|Kate Perez|BIN Taxonomy Match|BOLD:AAM5389
Orthocladius rubicundus|BIOUG15800-E04|Kate Perez|BIN Taxonomy Match|BOLD:AAM5389
Orthocladius rubicundus|BIOUG15963-E02|Kate Perez|BIN Taxonomy Match|BOLD:AAM5389
Orthocladius rubicundus|BIOUG16479-B04|Kate Perez|BIN Taxonomy Match|BOLD:AAM5389
Orthocladius rubicundus|BIOUG15652-C12|Kate Perez|BIN Taxonomy Match|BOLD:AAM5389
Orthocladius rubicundus|BIOUG15951-G04|Kate Perez|BIN Taxonomy Match|BOLD:AAM5389
Orthocladius rubicundus|BIOUG15764-B10|Kate Perez|BIN Taxonomy Match|BOLD:AAM5389
Orthocladius decoratus|ZMUO.026196|Lauri Paasivirta||BOLD:ABW7975
Orthocladius decoratus|ZMUO.026195|Lauri Paasivirta||BOLD:ABW7975
Orthocladius rivinus|KY225335|||BOLD:ADT0553
Orthocladius oblidens|PK-192-181|Petra Kranzfelder|morphology
Orthocladius oblidens|NHRS-BYWS000001094|Yngve Brodin|morphology|BOLD:AAD8971
Orthocladius oblidens|BIOUG16631-H11|Kate Perez|BIN Taxonomy Match|BOLD:AAD8971
Orthocladius oblidens|BIOUG16712-H04|Kate Perez|BIN Taxonomy Match|BOLD:AAD8971
Orthocladius oblidens|BIOUG16261-E04|Kate Perez|BIN Taxonomy Match|BOLD:AAD8971
Orthocladius oblidens|BIOUG15719-F09|Kate Perez|BIN Taxonomy Match|BOLD:AAD8971
Orthocladius oblidens|BIOUG16625-E04|Kate Perez|BIN Taxonomy Match|BOLD:AAD8971
Orthocladius oblidens|BIOUG16712-F01|Kate Perez|BIN Taxonomy Match|BOLD:AAD8971
Orthocladius oblidens|BIOUG16631-B03|Kate Perez|BIN Taxonomy Match|BOLD:AAD8971
Orthocladius oblidens|BIOUG16296-H03|Kate Perez|BIN Taxonomy Match|BOLD:AAD8971
Orthocladius oblidens|BIOUG17022-H10|Kate Perez|BIN Taxonomy Match|BOLD:AAD8971
Orthocladius oblidens|BIOUG16631-E09|Kate Perez|BIN Taxonomy Match|BOLD:AAD8971
Orthocladius oblidens|BIOUG16625-C11|Kate Perez|BIN Taxonomy Match|BOLD:AAD8971
Orthocladius oblidens|BIOUG16711-D10|Kate Perez|BIN Taxonomy Match|BOLD:AAD8971
Orthocladius oblidens|NHRS-BYWS000001798|Yngve Brodin||BOLD:AAD8971
Orthocladius oblidens|BIOUG16631-D07|Kate Perez|BIN Taxonomy Match|BOLD:AAD8971
Orthocladius oblidens|BIOUG15719-B09|Kate Perez|BIN Taxonomy Match|BOLD:AAD8971
Orthocladius oblidens|BIOUG16632-G11|Kate Perez|BIN Taxonomy Match|BOLD:AAD8971
Orthocladius oblidens|NHRS-BYWS000001137|Yngve Brodin|morphology|BOLD:AAD8971
Orthocladius oblidens|NHRS-BYWS000001813|Yngve Brodin||BOLD:AAD8971
Orthocladius oblidens|BIOUG17303-A10|Kate Perez|BIN Taxonomy Match|BOLD:AAD8971
Orthocladius decoratus|ZMUO.025264|Lauri Paasivirta||BOLD:AAD8971
Orthocladius oblidens|NHRS-BYWS000000388|Yngve Brodin|morphology|BOLD:AAD8971
Orthocladius|BIOUG42676-G01|Kate Perez|BIN Taxonomy Match below Phylum (Jun 2019)|BOLD:A...
Orthocladius oblidens|NHRS-BYWS000000517|Yngve Brodin|morphology|BOLD:AAD8971
Orthocladius oblidens|NHRS-BYWS000000430|Yngve Brodin|morphology|BOLD:AAD8971
Orthocladius oblidens|NHRS-BYWS000001788|Yngve Brodin||BOLD:AAD8971
Orthocladius oblidens|BIOUG16296-A09|Kate Perez|BIN Taxonomy Match|BOLD:AAD8971
Orthocladius oblidens|EBAI-Ch241|Elisabeth Stur|morphology|BOLD:AAD8971
Orthocladius oblidens|BIOUG16711-E04|Kate Perez|BIN Taxonomy Match|BOLD:AAD8971
Orthocladius oblidens|BIOUG16333-E04|Kate Perez|BIN Taxonomy Match|BOLD:AAD8971
Orthocladius oblidens|BIOUG16625-D06|Kate Perez|BIN Taxonomy Match|BOLD:AAD8971
Orthocladius oblidens|BIOUG16625-C09|Kate Perez|BIN Taxonomy Match|BOLD:AAD8971
Orthocladius oblidens|BIOUG15598-F02|Kate Perez|BIN Taxonomy Match|BOLD:AAD8971
Orthocladius oblidens|BIOUG16625-G08|Kate Perez|BIN Taxonomy Match|BOLD:AAD8971
Orthocladius oblidens|BIOUG16712-C10|Kate Perez|BIN Taxonomy Match|BOLD:AAD8971
Orthocladius oblidens|PK-192-191|Petra Kranzfelder|morphology
Orthocladius oblidens|Finnmark818|Elisabeth Stur||BOLD:AAD8971
Orthocladius oblidens|BIOUG15761-G07|Kate Perez|BIN Taxonomy Match|BOLD:AAD8971
Orthocladius oblidens|NHRS-BYWS000000135|Yngve Brodin|morphology|BOLD:AAD8971
Orthocladius oblidens|NHRS-BYWS000000431|Yngve Brodin|morphology|BOLD:AAD8971
Orthocladius oblidens|NHRS-BYWS000000146|Yngve Brodin|morphology|BOLD:AAD8971
Orthocladius oblidens|NHRS-BYWS000000546|Yngve Brodin|morphology|BOLD:AAD8971
Orthocladius oblidens|BIOUG16331-D03|Kate Perez|BIN Taxonomy Match|BOLD:AAD8971
Orthocladius oblidens|BIOUG15720-A07|Kate Perez|BIN Taxonomy Match|BOLD:AAD8971
Orthocladius oblidens|BIOUG16299-F07|Kate Perez|BIN Taxonomy Match|BOLD:AAD8971
Orthocladius oblidens|BIOUG16632-E12|Kate Perez|BIN Taxonomy Match|BOLD:AAD8971
Orthocladius oblidens|BIOUG16296-D02|Kate Perez|BIN Taxonomy Match|BOLD:AAD8971
Orthocladius oblidens|NHRS-BYWS000001797|Yngve Brodin||BOLD:AAD8971
Orthocladius oblidens|BIOUG16333-G12|Kate Perez|BIN Taxonomy Match|BOLD:AAD8971
Orthocladius oblidens|BIOUG16299-H01|Kate Perez|BIN Taxonomy Match|BOLD:AAD8971
Orthocladius oblidens|BIOUG16477-A09|Kate Perez|BIN Taxonomy Match|BOLD:AAD8971
Orthocladius oblidens|BIOUG16625-A01|Kate Perez|BIN Taxonomy Match|BOLD:AAD8971
Orthocladius oblidens|BIOUG16296-A07|Kate Perez|BIN Taxonomy Match|BOLD:AAD8971
Orthocladius oblidens|BIOUG16713-H04|Kate Perez|BIN Taxonomy Match|BOLD:AAD8971
Orthocladius oblidens|BIOUG16631-H01|Kate Perez|BIN Taxonomy Match|BOLD:AAD8971
Orthocladius oblidens|BIOUG16711-A01|Kate Perez|BIN Taxonomy Match|BOLD:AAD8971
Orthocladius oblidens|BIOUG16596-D11|Kate Perez|BIN Taxonomy Match|BOLD:AAD8971
Orthocladius oblidens|BIOUG15762-G06|Kate Perez|BIN Taxonomy Match|BOLD:AAD8971
Orthocladius oblidens|BIOUG16625-D07|Kate Perez|BIN Taxonomy Match|BOLD:AAD8971
Orthocladius oblidens|BIOUG16713-E03|Kate Perez|BIN Taxonomy Match|BOLD:AAD8971
Orthocladius oblidens|BIOUG15718-G05|Kate Perez|BIN Taxonomy Match|BOLD:AAD8971
Orthocladius oblidens|BIOUG16632-C09|Kate Perez|BIN Taxonomy Match|BOLD:AAD8971
Orthocladius oblidens|BIOUG16713-E08|Kate Perez|BIN Taxonomy Match|BOLD:AAD8971
Orthocladius oblidens|BIOUG16713-G01|Kate Perez|BIN Taxonomy Match|BOLD:AAD8971
Orthocladius oblidens|BIOUG16712-B12|Kate Perez|BIN Taxonomy Match|BOLD:AAD8971
Orthocladius oblidens|BIOUG15853-F01|Kate Perez|BIN Taxonomy Match|BOLD:AAD8971
Orthocladius oblidens|BIOUG17064-D11|Kate Perez|BIN Taxonomy Match|BOLD:AAD8971
Orthocladius oblidens|BIOUG17065-B07|Kate Perez|BIN Taxonomy Match|BOLD:AAD8971
Orthocladius oblidens|BIOUG16626-B11|Kate Perez|BIN Taxonomy Match|BOLD:AAD8971
Orthocladius oblidens|NHRS-BYWS000000264|Yngve Brodin|morphology|BOLD:AAD8971
Orthocladius oblidens|NHRS-BYWS000000470|Yngve Brodin|morphology|BOLD:AAD8971
Orthocladius oblidens|NHRS-BYWS000000433|Yngve Brodin|morphology|BOLD:AAD8971
Orthocladius oblidens|NHRS-BYWS000001864|Yngve Brodin||BOLD:AAD8971
Orthocladius oblidens|ZMUO.025258|Lauri Paasivirta||BOLD:AAD8971
Orthocladius oblidens|ZMUO.025257|Lauri Paasivirta||BOLD:AAD8971
Orthocladius oblidens|NHRS-BYWS000001803|Yngve Brodin||BOLD:AAD8971
Orthocladius oblidens|BIOUG15765-E09|Kate Perez|BIN Taxonomy Match|BOLD:AAD8971
Orthocladius oblidens|BIOUG15718-A03|Kate Perez|BIN Taxonomy Match|BOLD:AAD8971
Orthocladius sp. 10TE|Finnmark150|Torbjorn Ekrem||BOLD:AAP6053

Orthocladius oblidens|[illegible]|BOLD:AAD8971
Orthocladius oblidens|BIOUG15765-E09|Kate Perez|BIN Taxonomy Match|BOLD:AAD8971
Orthocladius oblidens|BIOUG15718-A03|Kate Perez|BIN Taxonomy Match|BOLD:AAD8971
Orthocladius sp. 10TE|Finnmark150|Torbjorn Ekrem||BOLD:AAP6053
Orthocladius excavatus|ZMUO.025248|Lauri Paasivirta||BOLD:AEC8098
Orthocladius decoratus|ZMUO.025263|Lauri Paasivirta||BOLD:AEC8098
Orthocladius excavatus|ZMUO.025247|Lauri Paasivirta||BOLD:AEC8098
Orthocladius|ZMUO.025917|Lauri Paasivirta||BOLD:ADF2287
Orthocladius decoratus|ATNA471|Torbjorn Ekrem||BOLD:AAE4990
Orthocladiinae|ZMUO.025962|Lauri Paasivirta||BOLD:ACM0243
Orthocladiinae|ZMUO.025961|Lauri Paasivirta||BOLD:ACM0243
Orthocladius lamellatus|ZMUO.025868|Lauri Paasivirta||BOLD:ACM0243
Orthocladius|ZMUO.025916|Lauri Paasivirta||BOLD:ACM0243
Orthocladius|BIOUG16894-C01|Jerome Moriniere|BIN Taxonomy Match|BOLD:ACR1772
Orthocladius|BIOUG16859-H02|Jerome Moriniere|BIN Taxonomy Match|BOLD:ACR1772
Orthocladius|BIOUG16893-D07|Jerome Moriniere|BIN Taxonomy Match|BOLD:ACR1772
Orthocladius|BIOUG16777-A01|Jerome Moriniere|BIN Taxonomy Match|BOLD:ACR1772
Orthocladius|BIOUG16776-H04|Jerome Moriniere|BIN Taxonomy Match|BOLD:ACR1772
Orthocladius|BIOUG16996-B04|Jerome Moriniere|BIN Taxonomy Match|BOLD:ACR1773
Orthocladius|BIOUG16782-F02|Jerome Moriniere|BIN Taxonomy Match|BOLD:ACR1773
Orthocladius|BIOUG16855-G07|Jerome Moriniere|BIN Taxonomy Match|BOLD:ACR1773
Orthocladius|BIOUG16856-A02|Jerome Moriniere|BIN Taxonomy Match|BOLD:ACR1773
Orthocladius|BIOUG16859-F05|Jerome Moriniere|BIN Taxonomy Match|BOLD:ACR1773
Orthocladius|BIOUG16855-H11|Jerome Moriniere|BIN Taxonomy Match|BOLD:ACR1773
Orthocladius|BIOUG16892-F12|Jerome Moriniere|BIN Taxonomy Match|BOLD:ACR1773
Orthocladius|BIOUG16893-G01|Jerome Moriniere|BIN Taxonomy Match|BOLD:ACR1773
Orthocladius|BIOUG16853-F06|Jerome Moriniere|BIN Taxonomy Match|BOLD:ACR1773
Orthocladius|BIOUG16854-A02|Jerome Moriniere|BIN Taxonomy Match|BOLD:ACR1773
Orthocladius|BIOUG16856-E07|Jerome Moriniere|BIN Taxonomy Match|BOLD:ACR1773
Orthocladius|BIOUG16892-H02|Jerome Moriniere|BIN Taxonomy Match|BOLD:ACR1773
Orthocladius|BIOUG16776-C02|Jerome Moriniere|BIN Taxonomy Match|BOLD:ACR1773
Orthocladius|BIOUG16778-F10|Jerome Moriniere|BIN Taxonomy Match|BOLD:ACR1773
Orthocladius|BIOUG16783-F06|Jerome Moriniere|BIN Taxonomy Match|BOLD:ACR1773
Orthocladius|BIOUG16857-F01|Kate Perez|BOLD ID Engine Manual|BOLD:ACR0587
Orthocladius|BIOUG16783-B03|Kate Perez|BOLD ID Engine Manual|BOLD:ACR0587
Orthocladius|BIOUG16856-B09|Kate Perez|BOLD ID Engine Manual|BOLD:ACR0587
Orthocladius|BIOUG16740-D11|Kate Perez|BOLD ID Engine Manual|BOLD:ACR0587
Orthocladius|BIOUG16740-H05|Kate Perez|BOLD ID Engine Manual|BOLD:ACR0587
Orthocladius|BIOUG16892-B06|Kate Perez|BOLD ID Engine Manual|BOLD:ACR0587
Orthocladius|BIOUG16782-C08|Kate Perez|BOLD ID Engine Manual|BOLD:ACR0587
Orthocladius|BIOUG16857-E05|Kate Perez|BOLD ID Engine Manual|BOLD:ACR0587
Orthocladius|BIOUG16853-D09|Kate Perez|BOLD ID Engine Manual|BOLD:ACR0587
Orthocladius|BIOUG16892-G04|Kate Perez|BOLD ID Engine Manual|BOLD:ACR0587
Orthocladius|BIOUG17137-C01|Kate Perez|BOLD ID Engine Manual|BOLD:ACR0587
Orthocladius|BIOUG16855-E04|Kate Perez|BOLD ID Engine Manual|BOLD:ACR0587
Orthocladius|BIOUG16775-C07|Kate Perez|BOLD ID Engine Manual|BOLD:ACR0587
Orthocladius|BIOUG16859-C08|Kate Perez|BOLD ID Engine Manual|BOLD:ACR0587
Orthocladius|BIOUG16855-G12|Kate Perez|BOLD ID Engine Manual|BOLD:ACR0587
Orthocladius|BIOUG16893-C03|Kate Perez|BOLD ID Engine Manual|BOLD:ACR0587
Orthocladius|BIOUG16853-C05|Kate Perez|BOLD ID Engine Manual|BOLD:ACR0587
Orthocladius|BIOUG16853-D10|Kate Perez|BOLD ID Engine Manual|BOLD:ACR0587
Orthocladius|BIOUG16859-C05|Kate Perez|BOLD ID Engine Manual|BOLD:ACR0587
Orthocladius|BIOUG16859-F11|Kate Perez|BOLD ID Engine Manual|BOLD:ACR0587
Orthocladius|BIOUG16859-E04|Kate Perez|BOLD ID Engine Manual|BOLD:ACR0587
Orthocladius|BIOUG16893-D02|Kate Perez|BOLD ID Engine Manual|BOLD:ACR0587
Orthocladius|BIOUG16856-B06|Kate Perez|BOLD ID Engine Manual|BOLD:ACR0587
Orthocladius|BIOUG16856-A11|Kate Perez|BOLD ID Engine Manual|BOLD:ACR0587
Orthocladius|BIOUG16856-F06|Kate Perez|BOLD ID Engine Manual|BOLD:ACR0587
Orthocladius|BIOUG16783-A09|Kate Perez|BOLD ID Engine Manual|BOLD:ACR0587
Orthocladius|BIOUG16859-F04|Kate Perez|BOLD ID Engine Manual|BOLD:ACR0587
Orthocladius|BIOUG16854-H07|Kate Perez|BOLD ID Engine Manual|BOLD:ACR0587
Orthocladius|BIOUG16777-F01|Kate Perez|BOLD ID Engine Manual|BOLD:ACR0587
Orthocladius|BIOUG16739-F02|Kate Perez|BOLD ID Engine Manual|BOLD:ACR0587
Orthocladius|BIOUG16856-G12|Kate Perez|BOLD ID Engine Manual|BOLD:ACR0587
Orthocladius|BIOUG17114-B05|Kate Perez|BOLD ID Engine Manual|BOLD:ACR0587
Orthocladius|BIOUG16892-H06|Kate Perez|BOLD ID Engine Manual|BOLD:ACR0587
Orthocladius|BIOUG16785-H10|Kate Perez|BOLD ID Engine Manual|BOLD:ACR0587
Orthocladius|BIOUG16857-B06|Kate Perez|BOLD ID Engine Manual|BOLD:ACR0587
Orthocladius|BIOUG16891-H11|Kate Perez|BOLD ID Engine Manual|BOLD:ACR0587
Orthocladius|BIOUG16856-B03|Kate Perez|BOLD ID Engine Manual|BOLD:ACR0587
Orthocladius|BIOUG16739-E04|Kate Perez|BOLD ID Engine Manual|BOLD:ACR0587
Orthocladius|BIOUG16893-F04|Kate Perez|BIN Taxonomy Match|BOLD:ACP2182
Orthocladius|BIOUG16893-E10|Kate Perez|BIN Taxonomy Match|BOLD:ACP2182
Orthocladius|BIOUG16857-D12|Kate Perez|BIN Taxonomy Match|BOLD:ACP2182
Orthocladius|BIOUG16857-H11|Kate Perez|BIN Taxonomy Match|BOLD:ACP2182
Orthocladius|BIOUG16782-C07|Kate Perez|BIN Taxonomy Match|BOLD:ACP2182
Orthocladius|BIOUG16859-B01|Kate Perez|BIN Taxonomy Match|BOLD:ACP2182
Orthocladius|BIOUG16859-B11|Kate Perez|BIN Taxonomy Match|BOLD:ACP2182
Orthocladius|BIOUG16894-D02|Kate Perez|BIN Taxonomy Match|BOLD:ACP2182
Orthocladius|BIOUG16893-F01|Kate Perez|BIN Taxonomy Match|BOLD:ACP2182
Orthocladius|BIOUG16857-F04|Kate Perez|BIN Taxonomy Match|BOLD:ACP2182
Orthocladius|BIOUG16784-H08|Kate Perez|BIN Taxonomy Match|BOLD:ACP2182
Orthocladius|BIOUG16853-G02|Kate Perez|BIN Taxonomy Match|BOLD:ACP2182
Orthocladius|BIOUG16859-D03|Kate Perez|BIN Taxonomy Match|BOLD:ACP2182
Orthocladius|BIOUG16721-H09|Kate Perez|BIN Taxonomy Match|BOLD:ACP2182
Orthocladius|BIOUG16857-D02|Kate Perez|BIN Taxonomy Match|BOLD:ACP2182
Orthocladius|BIOUG16738-D11|Kate Perez|BIN Taxonomy Match|BOLD:ACP2182
Orthocladius|BIOUG16776-E01|Kate Perez|BIN Taxonomy Match|BOLD:ACP2182
Orthocladius|BIOUG16859-C06|Kate Perez|BIN Taxonomy Match|BOLD:ACP2182
Orthocladius|BIOUG16783-B04|Kate Perez|BIN Taxonomy Match|BOLD:ACP2182
Orthocladius|BIOUG16721-B05|Kate Perez|BIN Taxonomy Match|BOLD:ACP2182
Orthocladius|BIOUG16721-H04|Kate Perez|BIN Taxonomy Match|BOLD:ACP2182
Orthocladius|BIOUG16785-H03|Kate Perez|BIN Taxonomy Match|BOLD:ACP2182
Orthocladius|BIOUG16859-F08|Kate Perez|BIN Taxonomy Match|BOLD:ACP2182
Orthocladius|BIOUG16894-C06|Kate Perez|BIN Taxonomy Match|BOLD:ACP2182
Orthocladius|BIOUG16778-F12|Kate Perez|BIN Taxonomy Match|BOLD:ACP2182
Orthocladius|BIOUG16775-A09|Kate Perez|BIN Taxonomy Match|BOLD:ACP2182
Orthocladius|BIOUG16857-F11|Kate Perez|BIN Taxonomy Match|BOLD:ACP2182
Orthocladius|BIOUG16894-A07|Kate Perez|BIN Taxonomy Match|BOLD:ACP2182
Orthocladius|BIOUG16894-A08|Kate Perez|BIN Taxonomy Match|BOLD:ACP2182
Orthocladius|BIOUG16892-E04|Kate Perez|BIN Taxonomy Match|BOLD:ACP2182
Orthocladius|BIOUG16892-D08|Kate Perez|BIN Taxonomy Match|BOLD:ACP2182
Orthocladius|BIOUG16856-C12|Kate Perez|BIN Taxonomy Match|BOLD:ACP2182
[illegible]

Orthocladius|BIOUG16892-E04|Kate Perez|BIN Taxonomy Match|BOLD:ACP2182
Orthocladius|BIOUG16892-D08|Kate Perez|BIN Taxonomy Match|BOLD:ACP2182
Orthocladius|BIOUG16856-C12|Kate Perez|BIN Taxonomy Match|BOLD:ACP2182
Orthocladius|BIOUG16855-G02|Kate Perez|BIN Taxonomy Match|BOLD:ACP2182
Orthocladius|BIOUG16855-D01|Kate Perez|BIN Taxonomy Match|BOLD:ACP2182
Orthocladius|BIOUG16855-C11|Kate Perez|BIN Taxonomy Match|BOLD:ACP2182
Orthocladius|BIOUG16855-C09|Kate Perez|BIN Taxonomy Match|BOLD:ACP2182
Orthocladius|BIOUG16855-B07|Kate Perez|BIN Taxonomy Match|BOLD:ACP2182
Orthocladius|BIOUG16854-H08|Kate Perez|BIN Taxonomy Match|BOLD:ACP2182
Orthocladius|BIOUG16856-F02|Kate Perez|BIN Taxonomy Match|BOLD:ACP2182
Orthocladius|BIOUG16893-E02|Kate Perez|BIN Taxonomy Match|BOLD:ACP2182
Orthocladius|BIOUG16893-A09|Kate Perez|BIN Taxonomy Match|BOLD:ACP2182
Orthocladius|BIOUG16853-G05|Kate Perez|BIN Taxonomy Match|BOLD:ACP2182
Orthocladius|BIOUG16893-G12|Kate Perez|BIN Taxonomy Match|BOLD:ACP2182
Orthocladius|BIOUG16855-F11|Kate Perez|BIN Taxonomy Match|BOLD:ACP2182
Orthocladius|BIOUG16854-D02|Kate Perez|BIN Taxonomy Match|BOLD:ACP2182
Orthocladius|BIOUG16854-D03|Kate Perez|BIN Taxonomy Match|BOLD:ACP2182
Orthocladius|BIOUG16854-D06|Kate Perez|BIN Taxonomy Match|BOLD:ACP2182
Orthocladius|BIOUG16854-E02|Kate Perez|BIN Taxonomy Match|BOLD:ACP2182
Orthocladius|BIOUG16856-F01|Kate Perez|BIN Taxonomy Match|BOLD:ACP2182
Orthocladius|BIOUG16856-G06|Kate Perez|BIN Taxonomy Match|BOLD:ACP2182
Orthocladius|BIOUG16859-A10|Kate Perez|BIN Taxonomy Match|BOLD:ACP2182
Orthocladius|BIOUG16892-C07|Kate Perez|BIN Taxonomy Match|BOLD:ACP2182
Orthocladius|BIOUG16856-F04|Kate Perez|BIN Taxonomy Match|BOLD:ACP2182
Orthocladius|BIOUG16892-H10|Kate Perez|BIN Taxonomy Match|BOLD:ACP2182
Orthocladius|BIOUG16892-D10|Kate Perez|BIN Taxonomy Match|BOLD:ACP2182
Orthocladius|BIOUG16892-B04|Kate Perez|BIN Taxonomy Match|BOLD:ACP2182
Orthocladius|BIOUG16857-C05|Kate Perez|BIN Taxonomy Match|BOLD:ACP2182
Orthocladius|BIOUG16857-A12|Kate Perez|BIN Taxonomy Match|BOLD:ACP2182
Orthocladius|BIOUG16855-E10|Kate Perez|BIN Taxonomy Match|BOLD:ACP2182
Orthocladius|BIOUG16721-E06|Kate Perez|BIN Taxonomy Match|BOLD:ACP2182
Orthocladius|BIOUG16893-C01|Kate Perez|BIN Taxonomy Match|BOLD:ACP2182
Orthocladius|BIOUG16785-H11|Kate Perez|BIN Taxonomy Match|BOLD:ACP2182
Orthocladius|BIOUG16856-C07|Kate Perez|BIN Taxonomy Match|BOLD:ACP2182
Orthocladius|BIOUG16893-D03|Kate Perez|BIN Taxonomy Match|BOLD:ACP2182
Orthocladius|BIOUG16854-F03|Kate Perez|BIN Taxonomy Match|BOLD:ACP2182
Orthocladius|BIOUG16857-G01|Kate Perez|BIN Taxonomy Match|BOLD:ACP2182
Orthocladius|BIOUG16892-A10|Kate Perez|BIN Taxonomy Match|BOLD:ACP2182
Orthocladius|BIOUG16892-C10|Kate Perez|BIN Taxonomy Match|BOLD:ACP2182
Orthocladius|BIOUG16782-G12|Kate Perez|BIN Taxonomy Match|BOLD:ACP2182
Orthocladius|BIOUG16784-C11|Kate Perez|BIN Taxonomy Match|BOLD:ACP2182
Orthocladius|BIOUG16740-B11|Kate Perez|BIN Taxonomy Match|BOLD:ACP2182
Orthocladius|BIOUG16994-C11|Kate Perez|BIN Taxonomy Match|BOLD:ACP2182
Orthocladius|BIOUG16859-C02|Kate Perez|BIN Taxonomy Match|BOLD:ACP2182
Orthocladius|CCDB21605-B09|Sofia Wiedenbrug||BOLD:ACP2182
Orthocladius|BIOUG16894-A12|Kate Perez|BIN Taxonomy Match|BOLD:ACP2182
Orthocladius|BIOUG16893-F10|Kate Perez|BIN Taxonomy Match|BOLD:ACP2182
Orthocladius|BIOUG16853-C04|Kate Perez|BIN Taxonomy Match|BOLD:ACP2182
Orthocladius|BIOUG16859-F10|Kate Perez|BIN Taxonomy Match|BOLD:ACP2182
Orthocladius|BIOUG16854-B12|Kate Perez|BIN Taxonomy Match|BOLD:ACP2182
Orthocladius|BIOUG16785-G11|Kate Perez|BIN Taxonomy Match|BOLD:ACP2182
Orthocladius|BIOUG16893-D08|Kate Perez|BIN Taxonomy Match|BOLD:ACP2182
Orthocladius|BIOUG16996-E03|Kate Perez|BIN Taxonomy Match|BOLD:ACP2182
Orthocladius|BIOUG16739-D07|Kate Perez|BIN Taxonomy Match|BOLD:ACP2182
Orthocladius lapponicus|Finnmark250|Torbjorn Ekrem||BOLD:AAB2644
Orthocladius lapponicus|Finnmark248|Elisabeth Stur||BOLD:AAB2644
Orthocladius cf. dentifer|CH-OSF110|Torbjorn Ekrem||BOLD:AAV2837
Orthocladius|BIOUG16479-D12|Kate Perez|BIN Taxonomy Match|BOLD:AAV5071
Orthocladius|BIOUG15592-G10|Kate Perez|BIN Taxonomy Match|BOLD:AAV5071
Orthocladius|BIOUG16711-H03|Kate Perez|BIN Taxonomy Match|BOLD:AAV5071
Orthocladius|BIOUG16426-H09|Kate Perez|BIN Taxonomy Match|BOLD:AAV5071
Orthocladius|BIOUG16258-F09|Kate Perez|BIN Taxonomy Match|BOLD:AAV5071
Orthocladius|BIOUG16597-A08|Kate Perez|BIN Taxonomy Match|BOLD:AAV5071
Orthocladius|BIOUG16259-H10|Kate Perez|BIN Taxonomy Match|BOLD:AAV5071
Orthocladius|BIOUG16632-A03|Kate Perez|BIN Taxonomy Match|BOLD:AAV5071
Orthocladius|BIOUG15595-A06|Kate Perez|BIN Taxonomy Match|BOLD:AAV5071
Orthocladius|BIOUG16711-G06|Kate Perez|BIN Taxonomy Match|BOLD:AAV5071
Orthocladius|BIOUG16632-B12|Kate Perez|BIN Taxonomy Match|BOLD:AAV5071
Orthocladius sp. 11TE|TRD-CH112|Elisabeth Stur||BOLD:AAV5071
Orthocladius sp. 11TE|RIN_CH27|Mona Renate Saursaunet|BOLD Identification engine|BOLD:AAV5071
Orthocladius sp. 11TE|Finnmark893|Elisabeth Stur||BOLD:AAV5071
Orthocladius|BIOUG15594-G06|Kate Perez|BIN Taxonomy Match|BOLD:AAV5071
Orthocladius|BIOUG15538-B09|Kate Perez|BIN Taxonomy Match|BOLD:AAV5071
Orthocladius|BIOUG15539-F08|Kate Perez|BIN Taxonomy Match|BOLD:AAV5071
Orthocladius|BIOUG16712-G01|Kate Perez|BIN Taxonomy Match|BOLD:AAV5071
Orthocladius|BIOUG15766-A09|Kate Perez|BIN Taxonomy Match|BOLD:AAV5071
Orthocladius|BIOUG15766-D06|Kate Perez|BIN Taxonomy Match|BOLD:AAV5071
Orthocladius|BIOUG15954-D02|Kate Perez|BIN Taxonomy Match|BOLD:AAV5071
Orthocladius|BIOUG15965-C05|Kate Perez|BIN Taxonomy Match|BOLD:AAV5071
Orthocladius|BIOUG15648-E11|Kate Perez|BIN Taxonomy Match|BOLD:AAV5071
Orthocladius|BIOUG16257-A03|Kate Perez|BIN Taxonomy Match|BOLD:AAV5071
Orthocladius|BIOUG16258-H08|Kate Perez|BIN Taxonomy Match|BOLD:AAV5071
Orthocladius|BIOUG16259-A06|Kate Perez|BIN Taxonomy Match|BOLD:AAV5071
Orthocladius|BIOUG16330-G12|Kate Perez|BIN Taxonomy Match|BOLD:AAV5071
Orthocladius|BIOUG15650-E03|Kate Perez|BIN Taxonomy Match|BOLD:AAV5071
Orthocladius|BIOUG16436-B07|Kate Perez|BIN Taxonomy Match|BOLD:AAV5071
Orthocladius|BIOUG15594-C06|Kate Perez|BIN Taxonomy Match|BOLD:AAV5071
Orthocladius|BIOUG15592-E08|Kate Perez|BIN Taxonomy Match|BOLD:AAV5071
Orthocladius|BIOUG16480-D03|Kate Perez|BIN Taxonomy Match|BOLD:AAV5071
Orthocladius|BIOUG15541-A02|Kate Perez|BIN Taxonomy Match|BOLD:AAV5071
Orthocladius|BIOUG15963-E03|Kate Perez|BIN Taxonomy Match|BOLD:AAV5071
Orthocladius|BIOUG15764-E02|Kate Perez|BIN Taxonomy Match|BOLD:AAV5071
Orthocladius|BIOUG15798-E04|Kate Perez|BIN Taxonomy Match|BOLD:AAV5071
Orthocladius|BIOUG15794-G12|Kate Perez|BIN Taxonomy Match|BOLD:AAV5071
Orthocladius|BIOUG15966-B04|Kate Perez|BIN Taxonomy Match|BOLD:AAV5071
Orthocladius|BIOUG15808-F09|Kate Perez|BIN Taxonomy Match|BOLD:AAV5071
Orthocladius|BIOUG16430-B03|Kate Perez|BIN Taxonomy Match|BOLD:AAV5071
Orthocladius|BIOUG16479-D06|Kate Perez|BIN Taxonomy Match|BOLD:AAV5071
Orthocladius|BIOUG16711-A09|Kate Perez|BIN Taxonomy Match|BOLD:AAV5071
Orthocladius|BIOUG16718-H11|Kate Perez|BIN Taxonomy Match|BOLD:AAV5071
Orthocladius|BIOUG15949-H03|Kate Perez|BIN Taxonomy Match|BOLD:AAV5071
Orthocladius|BIOUG15718-D04|Kate Perez|BIN Taxonomy Match|BOLD:AAV5071
Orthocladius|BIOUG16597-A07|Kate Perez|BIN Taxonomy Match|BOLD:AAV5071

Orthocladius|BIOUG15949-H03|Kate Perez|BIN Taxonomy Match|BOLD:AAV5071
Orthocladius|BIOUG15718-D04|Kate Perez|BIN Taxonomy Match|BOLD:AAV5071
Orthocladius|BIOUG16597-A07|Kate Perez|BIN Taxonomy Match|BOLD:AAV5071
Orthocladius|BIOUG16261-A02|Kate Perez|BIN Taxonomy Match|BOLD:AAV5071
Orthocladius|BIOUG15967-F03|Kate Perez|BIN Taxonomy Match|BOLD:AAV5071
Orthocladius|BIOUG15545-A04|Kate Perez|BIN Taxonomy Match|BOLD:AAV5071
Orthocladius|BIOUG16430-F06|Kate Perez|BIN Taxonomy Match|BOLD:AAV5071
Orthocladius|BIOUG17022-G01|Kate Perez|BIN Taxonomy Match|BOLD:AAV5071
Orthocladius|BIOUG15964-D04|Kate Perez|BIN Taxonomy Match|BOLD:AAV5071
Orthocladius|BIOUG15761-A01|Kate Perez|BIN Taxonomy Match|BOLD:AAV5071
Orthocladius|BIOUG15762-B01|Kate Perez|BIN Taxonomy Match|BOLD:AAV5071
Orthocladius|BIOUG15765-H08|Kate Perez|BIN Taxonomy Match|BOLD:AAV5071
Orthocladius|BIOUG15905-D01|Kate Perez|BIN Taxonomy Match|BOLD:AAV5071
Orthocladius|BIOUG15954-A06|Kate Perez|BIN Taxonomy Match|BOLD:AAV5071
Orthocladius|BIOUG15966-F07|Kate Perez|BIN Taxonomy Match|BOLD:AAV5071
Orthocladius|BIOUG15563-A09|Kate Perez|BIN Taxonomy Match|BOLD:AAV5071
Orthocladius|BIOUG15563-D08|Kate Perez|BIN Taxonomy Match|BOLD:AAV5071
Orthocladius|BIOUG15964-E04|Kate Perez|BIN Taxonomy Match|BOLD:AAV5071
Orthocladius|BIOUG15965-D10|Kate Perez|BIN Taxonomy Match|BOLD:AAV5071
Orthocladius|BIOUG15965-E02|Kate Perez|BIN Taxonomy Match|BOLD:AAV5071
Orthocladius|BIOUG15808-G02|Kate Perez|BIN Taxonomy Match|BOLD:AAV5071
Orthocladius|BIOUG15809-H05|Kate Perez|BIN Taxonomy Match|BOLD:AAV5071
Orthocladius|BIOUG15571-F09|Kate Perez|BIN Taxonomy Match|BOLD:AAV5071
Orthocladius|BIOUG15599-A08|Kate Perez|BIN Taxonomy Match|BOLD:AAV5071
Orthocladius|BIOUG16256-G01|Kate Perez|BIN Taxonomy Match|BOLD:AAV5071
Orthocladius|BIOUG16260-C01|Kate Perez|BIN Taxonomy Match|BOLD:AAV5071
Orthocladius|BIOUG15647-D06|Kate Perez|BIN Taxonomy Match|BOLD:AAV5071
Orthocladius|BIOUG15655-H07|Kate Perez|BIN Taxonomy Match|BOLD:AAV5071
Orthocladius|BIOUG16711-C02|Kate Perez|BIN Taxonomy Match|BOLD:AAV5071
Orthocladius|BIOUG16711-C05|Kate Perez|BIN Taxonomy Match|BOLD:AAV5071
Orthocladius|BIOUG16711-D12|Kate Perez|BIN Taxonomy Match|BOLD:AAV5071
Orthocladius|BIOUG16711-F11|Kate Perez|BIN Taxonomy Match|BOLD:AAV5071
Orthocladius|BIOUG17025-F03|Kate Perez|BIN Taxonomy Match|BOLD:AAV5071
Orthocladius|BIOUG17025-G03|Kate Perez|BIN Taxonomy Match|BOLD:AAV5071
Orthocladius|BIOUG17025-H02|Kate Perez|BIN Taxonomy Match|BOLD:AAV5071
Orthocladius|BIOUG17064-A10|Kate Perez|BIN Taxonomy Match|BOLD:AAV5071
Orthocladius|BIOUG17064-F06|Kate Perez|BIN Taxonomy Match|BOLD:AAV5071
Orthocladius|BIOUG17064-G01|Kate Perez|BIN Taxonomy Match|BOLD:AAV5071
Orthocladius|BIOUG17064-H02|Kate Perez|BIN Taxonomy Match|BOLD:AAV5071
Orthocladius|BIOUG17065-A03|Kate Perez|BIN Taxonomy Match|BOLD:AAV5071
Orthocladius|BIOUG17065-B05|Kate Perez|BIN Taxonomy Match|BOLD:AAV5071
Orthocladius|BIOUG16718-H04|Kate Perez|BIN Taxonomy Match|BOLD:AAV5071
Orthocladius|BIOUG17022-D10|Kate Perez|BIN Taxonomy Match|BOLD:AAV5071
Orthocladius|BIOUG17025-C07|Kate Perez|BIN Taxonomy Match|BOLD:AAV5071
Orthocladius|BIOUG17025-E10|Kate Perez|BIN Taxonomy Match|BOLD:AAV5071
Orthocladius|BIOUG15648-H03|Kate Perez|BIN Taxonomy Match|BOLD:AAV5071
Orthocladius|BIOUG16713-E09|Kate Perez|BIN Taxonomy Match|BOLD:AAV5071
Orthocladius|BIOUG16476-C10|Kate Perez|BIN Taxonomy Match|BOLD:AAV5071
Orthocladius|BIOUG16712-D02|Kate Perez|BIN Taxonomy Match|BOLD:AAV5071
Orthocladius sp. 11TE|PK-192-155|Torbjorn Ekrem|BIN Taxonomy Match?|BOLD:AAV5071
Orthocladius|BIOUG17023-A04|Kate Perez|BIN Taxonomy Match|BOLD:AAV5071
Orthocladius|BIOUG15764-F01|Kate Perez|BIN Taxonomy Match|BOLD:AAV5071
Orthocladius|BIOUG15720-D06|Kate Perez|BIN Taxonomy Match|BOLD:AAV5071
Orthocladius|BIOUG15761-A08|Kate Perez|BIN Taxonomy Match|BOLD:AAV5071
Orthocladius|BIOUG15905-D04|Kate Perez|BIN Taxonomy Match|BOLD:AAV5071
Orthocladius|BIOUG15905-G10|Kate Perez|BIN Taxonomy Match|BOLD:AAV5071
Orthocladius|BIOUG15949-A02|Kate Perez|BIN Taxonomy Match|BOLD:AAV5071
Orthocladius|BIOUG15964-A12|Kate Perez|BIN Taxonomy Match|BOLD:AAV5071
Orthocladius|BIOUG15565-A03|Kate Perez|BIN Taxonomy Match|BOLD:AAV5071
Orthocladius|BIOUG16552-A01|Kate Perez|BIN Taxonomy Match|BOLD:AAV5071
Orthocladius|BIOUG15764-E10|Kate Perez|BIN Taxonomy Match|BOLD:AAV5071
Orthocladius|BIOUG15598-B12|Kate Perez|BIN Taxonomy Match|BOLD:AAV5071
Orthocladius|BIOUG16256-B07|Kate Perez|BIN Taxonomy Match|BOLD:AAV5071
Orthocladius|BIOUG16596-E03|Kate Perez|BIN Taxonomy Match|BOLD:AAV5071
Orthocladius|BIOUG16260-H11|Kate Perez|BIN Taxonomy Match|BOLD:AAV5071
Orthocladius|BIOUG16258-D08|Kate Perez|BIN Taxonomy Match|BOLD:AAV5071
Orthocladius|BIOUG16259-E08|Kate Perez|BIN Taxonomy Match|BOLD:AAV5071
Orthocladius|BIOUG16260-D03|Kate Perez|BIN Taxonomy Match|BOLD:AAV5071
Orthocladius|BIOUG16257-D05|Kate Perez|BIN Taxonomy Match|BOLD:AAV5071
Orthocladius|BIOUG16630-G05|Kate Perez|BIN Taxonomy Match|BOLD:AAV5071
Orthocladius|BIOUG16430-F03|Kate Perez|BIN Taxonomy Match|BOLD:AAV5071
Orthocladius|BIOUG16711-A07|Kate Perez|BIN Taxonomy Match|BOLD:AAV5071
Orthocladius|BIOUG17023-E09|Kate Perez|BIN Taxonomy Match|BOLD:AAV5071
Orthocladius|BIOUG17025-G01|Kate Perez|BIN Taxonomy Match|BOLD:AAV5071
Orthocladius|BIOUG17025-G11|Kate Perez|BIN Taxonomy Match|BOLD:AAV5071
Orthocladius|BIOUG17064-B11|Kate Perez|BIN Taxonomy Match|BOLD:AAV5071
Orthocladius|BIOUG17064-G03|Kate Perez|BIN Taxonomy Match|BOLD:AAV5071
Orthocladius|BIOUG17064-H07|Kate Perez|BIN Taxonomy Match|BOLD:AAV5071
Orthocladius|BIOUG17022-E03|Kate Perez|BIN Taxonomy Match|BOLD:AAV5071
Orthocladius|BIOUG17025-D01|Kate Perez|BIN Taxonomy Match|BOLD:AAV5071
Orthocladius|BIOUG17025-E09|Kate Perez|BIN Taxonomy Match|BOLD:AAV5071
Orthocladius|BIOUG16626-C11|Kate Perez|BIN Taxonomy Match|BOLD:AAV5071
Orthocladius|BIOUG15764-C07|Kate Perez|BIN Taxonomy Match|BOLD:AAV5071
Orthocladius|BIOUG15900-H09|Kate Perez|BIN Taxonomy Match|BOLD:AAV5071
Orthocladius|BIOUG15600-H10|Kate Perez|BIN Taxonomy Match|BOLD:AAV5071
Orthocladius|BIOUG15720-E12|Kate Perez|BIN Taxonomy Match|BOLD:AAV5071
Orthocladius|BIOUG15655-B12|Kate Perez|BIN Taxonomy Match|BOLD:AAV5071
Orthocladius|BIOUG15653-G07|Kate Perez|BIN Taxonomy Match|BOLD:AAV5071
Orthocladius|BIOUG17022-H08|Kate Perez|BIN Taxonomy Match|BOLD:AAV5071
Orthocladius|BIOUG17065-C04|Kate Perez|BIN Taxonomy Match|BOLD:AAV5071
Orthocladius|BIOUG17065-E03|Kate Perez|BIN Taxonomy Match|BOLD:AAV5071
Orthocladius|BIOUG17023-B12|Kate Perez|BIN Taxonomy Match|BOLD:AAV5071
Orthocladius|BIOUG16476-G01|Kate Perez|BIN Taxonomy Match|BOLD:AAV5071
Orthocladius|BIOUG16435-C12|Kate Perez|BIN Taxonomy Match|BOLD:AAV5071
Orthocladius|BIOUG16480-F12|Kate Perez|BIN Taxonomy Match|BOLD:AAV5071
Orthocladius|BIOUG16631-H10|Kate Perez|BIN Taxonomy Match|BOLD:AAV5071
Orthocladius|BIOUG15954-H09|Kate Perez|BIN Taxonomy Match|BOLD:AAV5071
Orthocladius|BIOUG15651-E06|Kate Perez|BIN Taxonomy Match|BOLD:AAV5071
Orthocladius|BIOUG16430-D01|Kate Perez|BIN Taxonomy Match|BOLD:AAV5071
Orthocladius|BIOUG16434-F05|Kate Perez|BIN Taxonomy Match|BOLD:AAV5071
Orthocladius|BIOUG15544-C12|Kate Perez|BIN Taxonomy Match|BOLD:AAV5071
Orthocladius|BIOUG16476-F04|Kate Perez|BIN Taxonomy Match|BOLD:AAV5071
Orthocladius|BIOUG16433-H11|Kate Perez|BIN Taxonomy Match|BOLD:AAV5071

[illegible]
Orthocladius|BIOUG15544-C12|Kate Perez|BIN Taxonomy Match|BOLD:AAV5071
Orthocladius|BIOUG16476-F04|Kate Perez|BIN Taxonomy Match|BOLD:AAV5071
Orthocladius|BIOUG16433-H11|Kate Perez|BIN Taxonomy Match|BOLD:AAV5071
Orthocladius|BIOUG15950-D01|Kate Perez|BIN Taxonomy Match|BOLD:AAV5071
Orthocladius|BIOUG16478-A03|Kate Perez|BIN Taxonomy Match|BOLD:AAV5071
Orthocladius|BIOUG15953-H05|Kate Perez|BIN Taxonomy Match|BOLD:AAV5071
Orthocladius|BIOUG16632-E10|Kate Perez|BIN Taxonomy Match|BOLD:AAV5071
Orthocladius|BIOUG16630-G06|Kate Perez|BIN Taxonomy Match|BOLD:AAV5071
Orthocladius|BIOUG15764-G12|Kate Perez|BIN Taxonomy Match|BOLD:AAV5071
Orthocladius|BIOUG15905-A09|Kate Perez|BIN Taxonomy Match|BOLD:AAV5071
Orthocladius|BIOUG15539-E04|Kate Perez|BIN Taxonomy Match|BOLD:AAV5071
Orthocladius|BIOUG15598-D04|Kate Perez|BIN Taxonomy Match|BOLD:AAV5071
Orthocladius|BIOUG16712-F05|Kate Perez|BIN Taxonomy Match|BOLD:AAV5071
Orthocladius sp. 11TE|PK-195-17|Torbjorn Ekrem|BIN Taxonomy Match?|BOLD:AAV5071
Orthocladius|BIOUG15761-E06|Kate Perez|BIN Taxonomy Match|BOLD:AAV5071
Orthocladius|BIOUG15571-E12|Kate Perez|BIN Taxonomy Match|BOLD:AAV5071
Orthocladius|BIOUG16433-H02|Kate Perez|BIN Taxonomy Match|BOLD:AAV5071
Orthocladius|BIOUG16259-E04|Kate Perez|BIN Taxonomy Match|BOLD:AAV5071
Orthocladius|BIOUG15766-B06|Kate Perez|BIN Taxonomy Match|BOLD:AAV5071
Orthocladius|BIOUG15950-B02|Kate Perez|BIN Taxonomy Match|BOLD:AAV5071
Orthocladius|BIOUG15562-A07|Kate Perez|BIN Taxonomy Match|BOLD:AAV5071
Orthocladius|BIOUG16632-A02|Kate Perez|BIN Taxonomy Match|BOLD:AAV5071
Orthocladius|BIOUG16632-F08|Kate Perez|BIN Taxonomy Match|BOLD:AAV5071
Orthocladius|BIOUG16259-F09|Kate Perez|BIN Taxonomy Match|BOLD:AAV5071
Orthocladius|BIOUG16261-G09|Kate Perez|BIN Taxonomy Match|BOLD:AAV5071
Orthocladius|BIOUG15675-B12|Kate Perez|BIN Taxonomy Match|BOLD:AAV5071
Orthocladius|BIOUG16431-G05|Kate Perez|BIN Taxonomy Match|BOLD:AAV5071
Orthocladius|BIOUG15766-H08|Kate Perez|BIN Taxonomy Match|BOLD:AAV5071
Orthocladius|BIOUG15596-D04|Kate Perez|BIN Taxonomy Match|BOLD:AAV5071
Orthocladius|BIOUG16632-D02|Kate Perez|BIN Taxonomy Match|BOLD:AAV5071
Orthocladius|BIOUG15545-E09|Kate Perez|BIN Taxonomy Match|BOLD:AAV5071
Orthocladius|BIOUG15952-H02|Kate Perez|BIN Taxonomy Match|BOLD:AAV5071
Orthocladius|BIOUG16436-C12|Kate Perez|BIN Taxonomy Match|BOLD:AAV5071
Orthocladius|BIOUG16430-A10|Kate Perez|BIN Taxonomy Match|BOLD:AAV5071
Orthocladius|BIOUG15950-E03|Kate Perez|BIN Taxonomy Match|BOLD:AAV5071
Orthocladius|BIOUG15764-B05|Kate Perez|BIN Taxonomy Match|BOLD:AAV5071
Orthocladius|BIOUG15764-B02|Kate Perez|BIN Taxonomy Match|BOLD:AAV5071
Orthocladius|BIOUG15763-E12|Kate Perez|BIN Taxonomy Match|BOLD:AAV5071
Orthocladius|BIOUG15762-H07|Kate Perez|BIN Taxonomy Match|BOLD:AAV5071
Orthocladius sp. 11TE|Finnmark313|Torbjorn Ekrem||BOLD:AAV5071
Orthocladius|BC-ZSM-DIP-22491-H08|Dieter Doczkal||BOLD:ACX1285
Chironominae|BIOUG16855-C03|Kate Perez|BOLD ID Engine Manual|BOLD:ACR1743
Cricotopus sylvestris|ZSM-DIP-33129-E06|Caroline Chimeno|BIN Taxonomy Match (Jan 2022)|BOLD...
Orthocladius|BIOUG16854-G09|Valerie Levesque-Beaudin|Morphological|BOLD:ACR1936
Orthocladiinae|BIOUG16778-B09|Kate Perez|BIN Taxonomy Match|BOLD:AAW0369
Orthocladiinae|BIOUG16740-D12|Kate Perez|BIN Taxonomy Match|BOLD:AAW0369
Orthocladiinae|BIOUG16778-C06|Kate Perez|BIN Taxonomy Match|BOLD:AAW0369
Orthocladiinae|BIOUG16779-C09|Kate Perez|BIN Taxonomy Match|BOLD:AAW0369
Orthocladiinae|BIOUG16994-F09|Kate Perez|BIN Taxonomy Match|BOLD:AAW0369
Orthocladiinae|BIOUG16893-F06|Kate Perez|BIN Taxonomy Match|BOLD:AAW0369
Orthocladiinae|BIOUG16854-F02|Kate Perez|BIN Taxonomy Match|BOLD:AAW0369
Orthocladiinae|BIOUG16776-B07|Kate Perez|BIN Taxonomy Match|BOLD:AAW0369
Orthocladiinae|BIOUG16740-F07|Kate Perez|BIN Taxonomy Match|BOLD:AAW0369
Orthocladiinae|BIOUG16853-C09|Kate Perez|BIN Taxonomy Match|BOLD:AAW0369
Orthocladiinae|BIOUG16784-F03|Kate Perez|BIN Taxonomy Match|BOLD:AAW0369
Orthocladiinae|BIOUG16856-H03|Kate Perez|BIN Taxonomy Match|BOLD:AAW0369
Orthocladiinae|BIOUG16785-H07|Kate Perez|BIN Taxonomy Match|BOLD:AAW0369
Orthocladiinae|BIOUG16853-H01|Kate Perez|BIN Taxonomy Match|BOLD:AAW0369
Orthocladiinae|BIOUG16859-C10|Kate Perez|BIN Taxonomy Match|BOLD:AAW0369
Orthocladiinae|BIOUG16776-C05|Kate Perez|BIN Taxonomy Match|BOLD:AAW0369
Orthocladiinae|BIOUG16859-D02|Kate Perez|BIN Taxonomy Match|BOLD:AAW0369
Orthocladiinae|BIOUG16859-G11|Kate Perez|BIN Taxonomy Match|BOLD:AAW0369
Orthocladiinae|BIOUG16778-H09|Kate Perez|BIN Taxonomy Match|BOLD:AAW0369
Orthocladiinae|BIOUG16721-A02|Kate Perez|BIN Taxonomy Match|BOLD:AAW0369
Orthocladiinae|BIOUG16853-A08|Kate Perez|BIN Taxonomy Match|BOLD:AAW0369
Orthocladiinae|BIOUG16775-H04|Kate Perez|BIN Taxonomy Match|BOLD:AAW0369
Orthocladiinae|BIOUG16783-G11|Kate Perez|BIN Taxonomy Match|BOLD:AAW0369
Orthocladiinae|BIOUG16782-F03|Kate Perez|BIN Taxonomy Match|BOLD:AAW0369
Orthocladiinae|BIOUG16854-H01|Kate Perez|BIN Taxonomy Match|BOLD:AAW0369
Orthocladiinae|BIOUG16859-A11|Kate Perez|BIN Taxonomy Match|BOLD:AAW0369
Orthocladiinae|BIOUG16859-G05|Kate Perez|BIN Taxonomy Match|BOLD:AAW0369
Orthocladiinae|BIOUG16853-A02|Kate Perez|BIN Taxonomy Match|BOLD:AAW0369
Orthocladiinae|BIOUG16785-D01|Kate Perez|BIN Taxonomy Match|BOLD:AAW0369
Orthocladiinae|BIOUG16779-C08|Kate Perez|BIN Taxonomy Match|BOLD:AAW0369
Orthocladiinae|BIOUG16859-H08|Kate Perez|BIN Taxonomy Match|BOLD:AAW0369
Orthocladiinae|BIOUG16779-F03|Kate Perez|BIN Taxonomy Match|BOLD:AAW0369
Orthocladiinae|BIOUG16739-C03|Kate Perez|BIN Taxonomy Match|BOLD:AAW0369
Orthocladiinae|BIOUG16739-A08|Kate Perez|BIN Taxonomy Match|BOLD:AAW0369
Orthocladiinae|BIOUG16720-F11|Kate Perez|BIN Taxonomy Match|BOLD:AAW0369
Orthocladiinae|BIOUG16857-G05|Kate Perez|BIN Taxonomy Match|BOLD:AAW0369
Orthocladiinae|BIOUG16856-C04|Kate Perez|BIN Taxonomy Match|BOLD:AAW0369
Orthocladiinae|BIOUG16776-A02|Kate Perez|BIN Taxonomy Match|BOLD:AAW0369
Orthocladiinae|BIOUG16785-D04|Kate Perez|BIN Taxonomy Match|BOLD:AAW0369
Orthocladiinae|BIOUG16784-D09|Kate Perez|BIN Taxonomy Match|BOLD:AAW0369
Orthocladius thienemanni|CH-OSF63|Torbjorn Ekrem||BOLD:AAW0369
Orthocladius sp. 16TE|ATNA279|Torbjorn Ekrem||BOLD:AAB1172
Orthocladius rivicola|EBAI-Ch073|Elisabeth Stur|morphology|BOLD:ADL9709
Orthocladius rivicola|EBAI-Ch072|Elisabeth Stur|morphology|BOLD:ADL9709
Orthocladius rivicola|EBAI-Ch202|Elisabeth Stur|morphology|BOLD:ADL9709
Orthocladius rivicola|EBAI-Ch075|Elisabeth Stur|morphology|BOLD:ADL9709
Orthocladius rivicola|EBAI-Ch135|Elisabeth Stur|BOLD Identification Engine|BOLD:ADL9709
Orthocladius rivicola|EBAI-Ch068|Elisabeth Stur|morphology|BOLD:ADL9709
Orthocladius rivicola|EBAI-Ch134|Elisabeth Stur|BOLD Identification Engine|BOLD:ADL9709
Orthocladius rivicola|EBAI-Ch074|Elisabeth Stur|morphology|BOLD:ADL9709
Orthocladius rivicola|EBAI-Ch070|Elisabeth Stur|morphology|BOLD:ADL9709
Orthocladius rivicola|EBAI-Ch266|Elisabeth Stur|morphology|BOLD:ADL9709
Orthocladius rivicola|EBAI-Ch071|Elisabeth Stur|morphology|BOLD:ADL9709
Orthocladius rivicola|EBAI-Ch069|Elisabeth Stur|morphology|BOLD:ADL9709
Orthocladius rivicola|EBAI-Ch265|Elisabeth Stur|morphology|BOLD:ADL9709
Orthocladius rivicola|EBAI-Ch201|Elisabeth Stur|morphology|BOLD:ADL9709
Orthocladius telochaetus|Finnmark806|G. A. Halvorsen||BOLD:ADL9709
Orthocladius telochaetus|Finnmark804|Elisabeth Stur||BOLD:ADL9709
[illegible]

Orthocladius rivicola|EBAI-Ch201|Elisabeth Stur|morphology|BOLD:ADL9709
Orthocladius telochaetus|Finnmark806|G. A. Halvorsen||BOLD:ADL9709
Orthocladius telochaetus|Finnmark804|Elisabeth Stur||BOLD:ADL9709
Orthocladius rivicola|ATNA219|Elisabeth Stur||BOLD:AAB1171
Orthocladius rivicola|ATNA218|Elisabeth Stur||BOLD:AAB1171
Orthocladius rivicola|Finnmark790|Elisabeth Stur||BOLD:AAB1171
Orthocladius rivicola|ATNA136|Elisabeth Stur||BOLD:AAB1171
Orthocladius sp. 15TE|BIOUG15544-G07|Kate Perez|BIN Taxonomy Match|BOLD:AAM5390
Orthocladius|BIOUG15458-E09|Kate Perez|Tree based identification
Orthocladius sp. 15TE|BIOUG15545-D10|Elisabeth Stur|Tree based identification
Orthocladius sp. 15TE|BIOUG17022-C06|Kate Perez|BIN Taxonomy Match|BOLD:AAM5390
Orthocladius sp. 15TE|BIOUG15561-D07|Kate Perez|BIN Taxonomy Match|BOLD:AAM5390
Orthocladius sp. 15TE|BIOUG15543-A02|Kate Perez|BIN Taxonomy Match|BOLD:AAM5390
Orthocladius ashei|Finnmark62|Torbjorn Ekrem||BOLD:AAM5390
Orthocladius|CCDB24038-D05|Sofia Wiedenbrug|BIN Taxonomy Match|BOLD:AAM5390
Orthocladius|CCDB24037-B01|Sofia Wiedenbrug|BIN Taxonomy Match|BOLD:AAM5390
Orthocladius ashei|ZSM-DIP-33131-H03|Caroline Chimeno|BIN Taxonomy Match (Jan 2022)|BOLD ...
Orthocladius sp. 15TE|24405-E12|Kate Perez|BIN Taxonomy Match|BOLD:AAM5390
Orthocladius|BC-ZSM-DIP-22491-D08|Dieter Doczkal||BOLD:AAM5390
Chironomidae|CCDB24038-G09||BIN Taxonomy Match|BOLD:AAM5390
Orthocladius|CCDB24038-D07|Sofia Wiedenbrug|BIN Taxonomy Match|BOLD:AAM5390
Orthocladius|CCDB24229-C08|Sofia Wiedenbrug|BIN Taxonomy Match|BOLD:AAM5390
Orthocladius ashei|Finnmark878|G. A. Halvorsen||BOLD:AAM5390
Orthocladius|BC-ZSM-DIP-22491-G10|Dieter Doczkal||BOLD:AAM5390
Orthocladius sp. 15TE|24539-B11|Kate Perez|BIN Taxonomy Match|BOLD:AAM5390
Orthocladius|CCDB24038-D06|Sofia Wiedenbrug|BIN Taxonomy Match|BOLD:AAM5390
Orthocladius ashei|Finnmark28|Torbjorn Ekrem||BOLD:AAM5390
Orthocladius ashei|TRD-CH86|Elisabeth Stur||BOLD:AAM5390
Orthocladius ashei|Finnmark884|G. A. Halvorsen||BOLD:AAM5390
Orthocladius sp. 15TE|BIOUG15565-B08|Kate Perez|BIN Taxonomy Match|BOLD:AAM5390
Orthocladius sp. 15TE|BIOUG15460-F08|Kate Perez|BIN Taxonomy Match|BOLD:AAM5390
Orthocladius consobrinus|ZMUO.026220|Lauri Paasivirta||BOLD:ADF1072
Orthocladius consobrinus|ZMUO.026219|Lauri Paasivirta||BOLD:ADF1072
Orthocladius consobrinus|NHRS-BYWS000000946|Yngve Brodin|morphology|BOLD:AAM5387
Orthocladius consobrinus|NHRS-BYWS000000945|Yngve Brodin|morphology|BOLD:AAM5387
Orthocladius consobrinus|ZMUO.025941|Lauri Paasivirta||BOLD:AAM5387
Orthocladius sp. 4TE|Finnmark197|Torbjorn Ekrem||BOLD:AAM5387
Orthocladius sp. 4TE|Finnmark129|Torbjorn Ekrem||BOLD:AAM5387
Orthocladius saxosus|ATNA167|Elisabeth Stur||BOLD:AAB4825
Orthocladius saxosus|ATNA373|Elisabeth Stur||BOLD:AAB4825
Orthocladius saxosus|ATNA4|Elisabeth Stur||BOLD:AAB4825
Orthocladius saxosus|ATNA151|Elisabeth Stur||BOLD:AAB4825
Orthocladius saxosus|ATNA476|Elisabeth Stur||BOLD:AAB4825
Orthocladius saxosus|ATNA181|Elisabeth Stur||BOLD:AAB4825
Orthocladius saxosus|EBAI-Ch091|Elisabeth Stur|morphology|BOLD:AAB4825
Orthocladius saxosus|EBAI-Ch092|Elisabeth Stur|morphology|BOLD:AAB4825
Orthocladius saxosus|Finnmark71|Elisabeth Stur||BOLD:AAB4825
Orthocladius saxosus|EBAI-Ch065|Elisabeth Stur|morphology|BOLD:AAB4825
Orthocladius saxosus|EBAI-Ch203|Elisabeth Stur|morphology|BOLD:AAB4825
Orthocladius saxosus|ATNA158|Elisabeth Stur||BOLD:AAB4825
Orthocladius saxosus|ATNA3|Elisabeth Stur||BOLD:AAB4825
Orthocladius saxosus|ATNA126|Elisabeth Stur||BOLD:AAB4825
Orthocladius saxosus|ATNA184|Elisabeth Stur||BOLD:AAB4825
Orthocladius saxosus|EBAI-Ch199|Elisabeth Stur|morphology|BOLD:AAB4825
Orthocladius saxosus|ATNA127|Elisabeth Stur||BOLD:AAB4825
Orthocladius saxosus|Finnmark491|Elisabeth Stur||BOLD:AAB4825
Orthocladius saxosus|ATNA2|Elisabeth Stur||BOLD:AAB4825
Orthocladius saxosus|ATNA141|Elisabeth Stur||BOLD:AAB4825
Orthocladius saxosus|ATNA180|Elisabeth Stur||BOLD:AAB4825
Orthocladius saxosus|ATNA179|Elisabeth Stur||BOLD:AAB4825
Orthocladius saxosus|ATNA138|Elisabeth Stur||BOLD:AAB4825
Orthocladius saxosus|ATNA187|Elisabeth Stur||BOLD:AAB4825
Orthocladius saxosus|ATNA147|Elisabeth Stur||BOLD:AAB4825
Orthocladius saxosus|ATNA139|Elisabeth Stur||BOLD:AAB4825
Orthocladius saxosus|Finnmark753|Elisabeth Stur||BOLD:AAB4825
Orthocladius saxosus|Finnmark752|Elisabeth Stur||BOLD:AAB4825
Orthocladius saxosus|EBAI-Ch204|Elisabeth Stur|morphology|BOLD:AAB4825
Orthocladius saxosus|EBAI-Ch090|Elisabeth Stur|morphology|BOLD:AAB4825
Orthocladius saxosus|EBAI-Ch089|Elisabeth Stur|morphology|BOLD:AAB4825
Orthocladius saxosus|EBAI-Ch093|Elisabeth Stur|morphology|BOLD:AAB4825
Orthocladius saxosus|EBAI-Ch094|Elisabeth Stur|morphology|BOLD:AAB4825
Orthocladius saxosus|EBAI-Ch088|Elisabeth Stur|morphology|BOLD:AAB4825
Orthocladius saxosus|EBAI-Ch200|Elisabeth Stur|morphology|BOLD:AAB4825
Orthocladius saxosus|ATNA226|Elisabeth Stur||BOLD:AAB4825
Orthocladius saxosus|ATNA217|Elisabeth Stur||BOLD:AAB4825
Orthocladius saxosus|ATNA210|Elisabeth Stur||BOLD:AAB4825
Orthocladius saxosus|ATNA209|Elisabeth Stur||BOLD:AAB4825
Orthocladius saxosus|Finnmark487|Elisabeth Stur||BOLD:AAB4825
Orthocladius|BC-ZSM-DIP-22551-E12|Dieter Doczkal||BOLD:AEJ6987
Orthocladius|BC-ZSM-DIP-22551-D08|Dieter Doczkal||BOLD:AEJ6987
Orthocladius|BC-ZSM-DIP-22491-F04|Dieter Doczkal||BOLD:AEJ6987
Orthocladius|ZSM34342-D11|Sofia Wiedenbrug|Morphology|BOLD:AEJ6987
Orthocladius|BC-ZSM-DIP-22551-C02|Dieter Doczkal||BOLD:AEJ6987
Orthocladius|CCDB24039-E12|Sofia Wiedenbrug|BIN Taxonomy Match|BOLD:AEJ6987
Orthocladius|BC-ZSM-DIP-22491-E10|Dieter Doczkal||BOLD:AEJ6987
Orthocladius|CCDB24038-D04|Sofia Wiedenbrug|BIN Taxonomy Match|BOLD:AEJ6987
Orthocladius|BC-ZSM-DIP-22551-D04|Dieter Doczkal||BOLD:AEJ6987
Orthocladius|CCDB24228-A06|Sofia Wiedenbrug|BIN Taxonomy Match|BOLD:AEJ6987
Orthocladius|BC-ZSM-DIP-22491-G04|Dieter Doczkal||BOLD:AEJ6987
Orthocladius|BC-ZSM-DIP-22551-C06|Dieter Doczkal||BOLD:AEJ6987
Orthocladius|CCDB24039-E11|Sofia Wiedenbrug|BIN Taxonomy Match|BOLD:AEJ6987
Orthocladius|CCDB24039-G07|Sofia Wiedenbrug|BIN Taxonomy Match|BOLD:AEJ6987
Orthocladius|ZSM34343-G10|Sofia Wiedenbrug|Morphology|BOLD:AEJ6987
Orthocladius|BC-ZSM-DIP-22491-F11|Dieter Doczkal||BOLD:AEJ6987
Orthocladius|CCDB24039-B08|Sofia Wiedenbrug|BIN Taxonomy Match|BOLD:AEJ6987
Orthocladius|CCDB24038-D12|Sofia Wiedenbrug|BIN Taxonomy Match|BOLD:AEJ6987
Orthocladius|ZSM34342-B08|Sofia Wiedenbrug|Morphology|BOLD:ACQ3313
Orthocladius|BC-ZSM-DIP-22551-F11|Dieter Doczkal||BOLD:AEJ6987
Orthocladius|CCDB24037-D06|Sofia Wiedenbrug|BIN Taxonomy Match|BOLD:AEJ6987
Orthocladius|CCDB24037-A01|Sofia Wiedenbrug|BIN Taxonomy Match|BOLD:AEJ6987
Orthocladiinae|CCDB24228-G11||BIN Taxonomy Match|BOLD:ACQ3313
Orthocladius|BC-ZSM-DIP-22551-E07|Dieter Doczkal||BOLD:AEJ6987
Orthocladius|BC-ZSM-DIP-22551-D11|Dieter Doczkal||BOLD:AEJ6987

Orthocladiinae|CCDB24228-G11||BIN Taxonomy Match|BOLD:ACQ3313
Orthocladius|BC-ZSM-DIP-22551-E07|Dieter Doczkal||BOLD:AEJ6987
Orthocladius|BC-ZSM-DIP-22551-D11|Dieter Doczkal||BOLD:AEJ6987
Orthocladius|BC-ZSM-DIP-22491-E12|Dieter Doczkal||BOLD:AEJ6988
Orthocladius|BC-ZSM-DIP-22491-H03|Dieter Doczkal||BOLD:AEJ6988
Orthocladius|CCDB24037-F02|Sofia Wiedenbrug|BIN Taxonomy Match|BOLD:AEJ6988
Orthocladius|BC-ZSM-DIP-22488-C07|Dieter Doczkal||BOLD:ACQ3313
Orthocladius|BC-ZSM-DIP-22488-C12|Dieter Doczkal||BOLD:ACQ3313
Orthocladius|BC-ZSM-DIP-22488-F01|Dieter Doczkal||BOLD:ACQ3313
Orthocladius|ZSM34342-D10|Sofia Wiedenbrug|Morphology|BOLD:ACQ3313
Orthocladius|BC-ZSM-DIP-22488-G06|Dieter Doczkal||BOLD:ACQ3313
Orthocladius|BC-ZSM-DIP-22488-E04|Dieter Doczkal||BOLD:ACQ3313
Orthocladius|BC-ZSM-DIP-22488-E03|Dieter Doczkal||BOLD:ACQ3313
Orthocladius|ZSM34342-D08|Sofia Wiedenbrug|Morphology|BOLD:ACQ3313
Orthocladius|BC-ZSM-DIP-22488-E01|Dieter Doczkal||BOLD:ACQ3313
Orthocladius|BC-ZSM-DIP-22488-C08|Dieter Doczkal||BOLD:ACQ3313
Orthocladius|CCDB24038-H01|Sofia Wiedenbrug|BIN Taxonomy Match|BOLD:ACQ3313
Orthocladius|CCDB24038-G03|Sofia Wiedenbrug|BIN Taxonomy Match|BOLD:ACQ3313
Orthocladius|CCDB24038-G02|Sofia Wiedenbrug|BIN Taxonomy Match|BOLD:ACQ3313
Orthocladius|BC-ZSM-DIP-22488-G02|Dieter Doczkal||BOLD:ACQ3313
Orthocladius|CCDB24039-A07|Sofia Wiedenbrug|BIN Taxonomy Match|BOLD:ACQ3313
Orthocladius|ZSM34342-D12|Sofia Wiedenbrug|Morphology|BOLD:ACQ3313
Orthocladius|CCDB24038-G12|Sofia Wiedenbrug|BIN Taxonomy Match|BOLD:ACQ3313
Orthocladius|CCDB21606-H11|Sofia Wiedenbrug||BOLD:ACQ3313
Orthocladius|CCDB24037-D03|Sofia Wiedenbrug|BIN Taxonomy Match|BOLD:ACQ3313
Orthocladius|GBOL03746|Sofia Wiedenbrug||BOLD:ACQ3313
Orthocladius|ATNA129|Elisabeth Stur||BOLD:AAI3486
Orthocladius|BIOUG15542-F09|Elisabeth Stur|BIN Taxonomy Match|BOLD:AAI3486
Orthocladius|EBAI-Ch211|Elisabeth Stur|morphology|BOLD:AAI3486
Orthocladius|BIOUG15538-D11|Elisabeth Stur|BIN Taxonomy Match|BOLD:AAI3486
Orthocladius frigidus|ZMUO.025956|Lauri Paasivirta||BOLD:AAE1613
Orthocladius frigidus|ZMUO.025955|Lauri Paasivirta||BOLD:AAE1613
Orthocladius frigidus|EBAI-Ch119|Elisabeth Stur|BOLD Identification Engine|BOLD:AAE1613
Orthocladius frigidus|BIOUG15454-H01|Kate Perez|BIN Taxonomy Match|BOLD:AAE1613
Orthocladius frigidus|EBAI-Ch118|Elisabeth Stur|BOLD Identification Engine|BOLD:AAE1613
Orthocladius frigidus|TRD-CH85|Elisabeth Stur||BOLD:AAE1613
Orthocladius frigidus|BIOUG17064-A09|Kate Perez|BIN Taxonomy Match|BOLD:AAE1613
Orthocladius frigidus|BIOUG15463-E03|Kate Perez|BIN Taxonomy Match|BOLD:AAE1613
Orthocladius frigidus|BIOUG15544-E06|Kate Perez|BIN Taxonomy Match|BOLD:AAE1613
Orthocladius frigidus|BIOUG15565-B12|Kate Perez|BIN Taxonomy Match|BOLD:AAE1613
Orthocladius frigidus|BIOUG17025-C12|Kate Perez|BIN Taxonomy Match|BOLD:AAE1613
Orthocladius frigidus|BIOUG15564-B04|Kate Perez|BIN Taxonomy Match|BOLD:AAE1613
Orthocladius frigidus|BIOUG17064-H11|Kate Perez|BIN Taxonomy Match|BOLD:AAE1613
Orthocladius frigidus|BIOUG15539-A04|Kate Perez|BIN Taxonomy Match|BOLD:AAE1613
Orthocladius frigidus|BIOUG17025-E11|Kate Perez|BIN Taxonomy Match|BOLD:AAE1613
Orthocladius frigidus|BIOUG17064-B12|Kate Perez|BIN Taxonomy Match|BOLD:AAE1613
Orthocladius frigidus|BIOUG17065-A06|Kate Perez|BIN Taxonomy Match|BOLD:AAE1613
Orthocladius frigidus|BIOUG15543-H07|Kate Perez|BIN Taxonomy Match|BOLD:AAE1613
Orthocladius frigidus|BIOUG15542-D02|Kate Perez|BIN Taxonomy Match|BOLD:AAE1613
Orthocladius frigidus|BIOUG15542-A12|Kate Perez|BIN Taxonomy Match|BOLD:AAE1613
Orthocladius frigidus|BIOUG15465-D11|Kate Perez|BIN Taxonomy Match|BOLD:AAE1613
Orthocladius frigidus|NO47|Elisabeth Stur||BOLD:AAE1613
Orthocladius frigidus|ATNA255|Elisabeth Stur||BOLD:AAE1613
Orthocladius frigidus|ATNA460|Elisabeth Stur||BOLD:AAE1613
Orthocladius frigidus|BIOUG17025-C05|Kate Perez|BIN Taxonomy Match|BOLD:AAE1613
Orthocladius frigidus|ATNA224|Elisabeth Stur||BOLD:AAE1613
Orthocladius frigidus|TRD-CH114|Elisabeth Stur||BOLD:AAE1613
Orthocladius frigidus|ATNA128|Elisabeth Stur||BOLD:AAE1613
Orthocladius frigidus|ATNA137|Elisabeth Stur||BOLD:AAE1613
Orthocladius frigidus|CH-OSF155|Elisabeth Stur||BOLD:AAE1613
Orthocladius frigidus|EBAI-Ch194|Elisabeth Stur|morphology|BOLD:AAE1613
Orthocladius frigidus|EBAI-Ch117|Elisabeth Stur|BOLD Identification Engine|BOLD:AAE1613
Orthocladius frigidus|BIOUG15465-B12|Kate Perez|BIN Taxonomy Match|BOLD:AAE1613
Orthocladius frigidus|BIOUG15464-D12|Kate Perez|BIN Taxonomy Match|BOLD:AAE1613
Orthocladius frigidus|BIOUG15464-F06|Kate Perez|BIN Taxonomy Match|BOLD:AAE1613
Orthocladius frigidus|EBAI-Ch234|Elisabeth Stur|morphology|BOLD:AAE1613
Orthocladius frigidus|BIOUG15541-A12|Kate Perez|BIN Taxonomy Match|BOLD:AAE1613
Orthocladius frigidus|BIOUG17065-D02|Kate Perez|BIN Taxonomy Match|BOLD:AAE1613
Orthocladius frigidus|BIOUG15540-C10|Kate Perez|BIN Taxonomy Match|BOLD:AAE1613
Orthocladius frigidus|CCDB24037-D02|Sofia Wiedenbrug|BIN Taxonomy Match|BOLD:AAE1613
Orthocladius frigidus|BIOUG15563-B08|Kate Perez|BIN Taxonomy Match|BOLD:AAE1613
Orthocladius frigidus|BIOUG15599-D04|Kate Perez|BIN Taxonomy Match|BOLD:AAE1613
Orthocladius frigidus|BIOUG15544-F10|Kate Perez|BIN Taxonomy Match|BOLD:AAE1613
Orthocladius frigidus|BIOUG16258-H09|Kate Perez|BIN Taxonomy Match|BOLD:AAE1613
Orthocladius frigidus|BIOUG15562-E07|Kate Perez|BIN Taxonomy Match|BOLD:AAE1613
Orthocladius frigidus|BIOUG15537-G11|Kate Perez|BIN Taxonomy Match|BOLD:AAE1613
Orthocladius frigidus|BIOUG15465-H01|Kate Perez|BIN Taxonomy Match|BOLD:AAE1613
Orthocladius frigidus|BIOUG16631-F07|Kate Perez|BIN Taxonomy Match|BOLD:AAE1613
Orthocladius frigidus|EBAI-Ch212|Elisabeth Stur|morphology|BOLD:AAE1613
Orthocladius frigidus|BIOUG17025-H07|Kate Perez|BIN Taxonomy Match|BOLD:AAE1613
Orthocladius frigidus|BIOUG15542-A06|Kate Perez|BIN Taxonomy Match|BOLD:AAE1613
Orthocladius frigidus|Finnmark558|Elisabeth Stur||BOLD:AAE1613
Orthocladius rivulorum|Finnmark19|Torbjorn Ekrem||BOLD:AAM5388
Orthocladius rivulorum|Finnmark542|Torbjorn Ekrem||BOLD:AAM5388
Orthocladius rivulorum|Finnmark492|Torbjorn Ekrem||BOLD:AAM5388
Orthocladius frigidus|ZMUO.026009|Lauri Paasivirta||BOLD:ADF1431
Orthocladius lamellatus|ZMUO.025869|Lauri Paasivirta||BOLD:ADF1431
Orthocladius|ZMUO.025865|Lauri Paasivirta||BOLD:ADF1431
Orthocladius|ZMUO.025864|Lauri Paasivirta||BOLD:ADF1431
Orthocladius lamellatus|ZMUO.025905|Lauri Paasivirta||BOLD:ADF1431
Orthocladius lamellatus|ZMUO.025904|Lauri Paasivirta||BOLD:ADF1431
Orthocladius lamellatus|Finnmark312|Elisabeth Stur||BOLD:AAV5070
Orthocladius lamellatus|Finnmark296|Elisabeth Stur||BOLD:AAV5070
Eukiefferiella pseudomontana|Finnmark875|G. A. Halvorsen|morphology|BOLD:ADG3574
Cricotopus trifascia|BIOUG15952-E02|Elisabeth Stur|BIN Taxonomy Match|BOLD:AAU2576
Cricotopus trifascia|BIOUG16297-D09|Kate Perez|BIN Taxonomy Match|BOLD:AAU2576
Cricotopus|BIOUG17120-G07|Kate Perez|BIN Taxonomy Match|BOLD:AAU2576
Cricotopus trifascia|BIOUG16329-G04|Kate Perez|BIN Taxonomy Match|BOLD:AAU2576
Cricotopus trifascia|BIOUG16434-B11|Kate Perez|BIN Taxonomy Match|BOLD:AAU2576
Cricotopus trifascia|NO76|Elisabeth Stur|Morphology|BOLD:AAU2576
Cricotopus trifascia|BIOUG15966-G06|Kate Perez|BIN Taxonomy Match|BOLD:AAU2576
Cricotopus trifascia|BIOUG15963-C09|Kate Perez|BIN Taxonomy Match|BOLD:AAU2576
Cricotopus trifascia|BIOUG15947-H08|Kate Perez|BIN Taxonomy Match|BOLD:AAU2576

Cricotopus trifascia|BIOUG15966-G06|Kate Perez|BIN Taxonomy Match|BOLD:AAU2576
Cricotopus trifascia|BIOUG15963-C09|Kate Perez|BIN Taxonomy Match|BOLD:AAU2576
Cricotopus trifascia|BIOUG15947-H08|Kate Perez|BIN Taxonomy Match|BOLD:AAU2576
Cricotopus trifascia|NHRS-BYWS000000134|Yngve Brodin|morphology|BOLD:AAU2576
Cricotopus trifascia|ZMUO.025262|Lauri Paasivirta||BOLD:AAU2576
Cricotopus trifascia|ZMUO.025261|Lauri Paasivirta||BOLD:AAU2576
Cricotopus trifascia|NHRS-BYWS000000488|Yngve Brodin|morphology|BOLD:AAU2576
Cricotopus trifascia|NHRS-BYWS000000349|Yngve Brodin|morphology|BOLD:AAU2576
Cricotopus trifascia|NHRS-BYWS000000286|Yngve Brodin|morphology|BOLD:AAU2576
Cricotopus trifascia|NHRS-BYWS000000259|Yngve Brodin|morphology|BOLD:AAU2576
Cricotopus trifascia|NHRS-BYWS000000145|Yngve Brodin|morphology|BOLD:AAU2576
Cricotopus trifascia|NHRS-BYWS000000132|Yngve Brodin|morphology|BOLD:AAU2576
Cricotopus trifascia|NHRS-BYWS000001870|Yngve Brodin||BOLD:AAU2576
Cricotopus trifascia|NHRS-BYWS000001869|Yngve Brodin||BOLD:AAU2576
Cricotopus fuscus|Finnmark757|Torbjorn Ekrem||BOLD:ACD1666
Cricotopus cf. septentrionalis|BIOUG15952-F10|Kate Perez|BIN Taxonomy Match|BOLD:ABA3995
Cricotopus cf. septentrionalis|BIOUG15953-H08|Kate Perez|BIN Taxonomy Match|BOLD:ABA3995
Cricotopus cf. septentrionalis|BIOUG15949-A12|Kate Perez|BIN Taxonomy Match|BOLD:ABA3995
Cricotopus cf. septentrionalis|BIOUG15947-E11|Kate Perez|BIN Taxonomy Match|BOLD:ABA3995
Cricotopus cf. septentrionalis|BIOUG15953-B03|Kate Perez|BIN Taxonomy Match|BOLD:ABA3995
Cricotopus cf. septentrionalis|NO75|Elisabeth Stur||BOLD:ABA3995
Cricotopus cf. septentrionalis|BIOUG15953-B05|Kate Perez|BIN Taxonomy Match|BOLD:ABA3995
Cricotopus cf. septentrionalis|BIOUG15947-F04|Kate Perez|BIN Taxonomy Match|BOLD:ABA3995
Cricotopus cf. septentrionalis|BIOUG15952-G09|Kate Perez|BIN Taxonomy Match|BOLD:ABA3995
Cricotopus cf. septentrionalis|BIOUG15965-B01|Kate Perez|BIN Taxonomy Match|BOLD:ABA3995
Cricotopus cf. septentrionalis|BIOUG15947-A10|Kate Perez|BIN Taxonomy Match|BOLD:ABA3995
Cricotopus cf. septentrionalis|BIOUG15965-E08|Kate Perez|BIN Taxonomy Match|BOLD:ABA3995
Cricotopus cf. septentrionalis|BIOUG16597-B02|Kate Perez|BIN Taxonomy Match|BOLD:ABA3995
Cricotopus cf. septentrionalis|ATNA533|Elisabeth Stur||BOLD:ABA3995
Cricotopus cf. septentrionalis|BIOUG15949-E08|Kate Perez|BIN Taxonomy Match|BOLD:ABA3995
Cricotopus cf. septentrionalis|BIOUG15965-F11|Kate Perez|BIN Taxonomy Match|BOLD:ABA3995
Cricotopus cf. septentrionalis|BIOUG15965-C02|Kate Perez|BIN Taxonomy Match|BOLD:ABA3995
Cricotopus cf. septentrionalis|BIOUG15967-F04|Kate Perez|BIN Taxonomy Match|BOLD:ABA3995
Cricotopus cf. septentrionalis|BIOUG15949-E02|Kate Perez|BIN Taxonomy Match|BOLD:ABA3995
Cricotopus cf. septentrionalis|BIOUG15947-F06|Kate Perez|BIN Taxonomy Match|BOLD:ABA3995
Cricotopus cf. septentrionalis|BIOUG15947-F05|Kate Perez|BIN Taxonomy Match|BOLD:ABA3995
Cricotopus cf. septentrionalis|BIOUG15905-H02|Kate Perez|BIN Taxonomy Match|BOLD:ABA3995
Cricotopus cf. septentrionalis|BIOUG15905-F09|Kate Perez|BIN Taxonomy Match|BOLD:ABA3995
Cricotopus cf. septentrionalis|BIOUG15905-E10|Kate Perez|BIN Taxonomy Match|BOLD:ABA3995
Cricotopus cf. septentrionalis|BIOUG15905-D02|Kate Perez|BIN Taxonomy Match|BOLD:ABA3995
Cricotopus cf. septentrionalis|BIOUG15905-C09|Kate Perez|BIN Taxonomy Match|BOLD:ABA3995
Cricotopus cf. septentrionalis|BIOUG15966-B01|Kate Perez|BIN Taxonomy Match|BOLD:ABA3995
Cricotopus cf. septentrionalis|BIOUG15809-H06|Kate Perez|BIN Taxonomy Match|BOLD:ABA3995
Cricotopus cf. septentrionalis|BIOUG16205-E01|Kate Perez|BIN Taxonomy Match|BOLD:ABA3995
Cricotopus cf. septentrionalis|BIOUG16596-F08|Kate Perez|BIN Taxonomy Match|BOLD:ABA3995
Cricotopus cf. septentrionalis|BIOUG15905-B02|Kate Perez|BIN Taxonomy Match|BOLD:ABA3995
Cricotopus cf. septentrionalis|BIOUG15954-E02|Kate Perez|BIN Taxonomy Match|BOLD:ABA3995
Cricotopus cf. septentrionalis|BIOUG15951-H07|Kate Perez|BIN Taxonomy Match|BOLD:ABA3995
Cricotopus cf. septentrionalis|BIOUG15951-D11|Kate Perez|BIN Taxonomy Match|BOLD:ABA3995
Cricotopus cf. septentrionalis|BIOUG15953-H07|Kate Perez|BIN Taxonomy Match|BOLD:ABA3995
Cricotopus cf. septentrionalis|BIOUG15951-A04|Kate Perez|BIN Taxonomy Match|BOLD:ABA3995
Cricotopus cf. septentrionalis|BIOUG15947-D02|Kate Perez|BIN Taxonomy Match|BOLD:ABA3995
Cricotopus cf. septentrionalis|BIOUG15947-F12|Kate Perez|BIN Taxonomy Match|BOLD:ABA3995
Cricotopus cf. septentrionalis|BIOUG15952-G03|Kate Perez|BIN Taxonomy Match|BOLD:ABA3995
Cricotopus cf. septentrionalis|ATNA558|Elisabeth Stur||BOLD:ABA3995
Cricotopus cf. septentrionalis|BIOUG15953-E01|Kate Perez|BIN Taxonomy Match|BOLD:ABA3995
Cricotopus cf. septentrionalis|BIOUG15964-A03|Kate Perez|BIN Taxonomy Match|BOLD:ABA3995
Cricotopus cf. septentrionalis|BIOUG16596-C11|Kate Perez|BIN Taxonomy Match|BOLD:ABA3995
Cricotopus cf. septentrionalis|BIOUG16296-B03|Kate Perez|BIN Taxonomy Match|BOLD:ABA3995
Cricotopus cf. septentrionalis|BIOUG16477-E06|Kate Perez|BIN Taxonomy Match|BOLD:ABA3995
Cricotopus cf. septentrionalis|BIOUG16597-G11|Kate Perez|BIN Taxonomy Match|BOLD:ABA3995
Cricotopus cf. septentrionalis|BIOUG15967-A03|Kate Perez|BIN Taxonomy Match|BOLD:ABA3995
Cricotopus cf. septentrionalis|PK-189-8|Elisabeth Stur|BIN Taxonomy Match?
Cricotopus cf. septentrionalis|PK-189-5|Elisabeth Stur|BIN Taxonomy Match?|BOLD:ABA3995
Cricotopus cf. septentrionalis|PK-188-11|Elisabeth Stur|BIN Taxonomy Match?|BOLD:ABA3995
Cricotopus cf. septentrionalis|PK-189-10|Elisabeth Stur|BIN Taxonomy Match?|BOLD:ABA3995
Cricotopus cf. septentrionalis|PK-187-11|Elisabeth Stur|BIN Taxonomy Match|BOLD:ABA3995
Cricotopus cf. septentrionalis|BIOUG15951-F10|Kate Perez|BIN Taxonomy Match|BOLD:ABA3995
Cricotopus cf. septentrionalis|BIOUG15950-G08|Kate Perez|BIN Taxonomy Match|BOLD:ABA3995
Cricotopus cf. septentrionalis|BIOUG15951-C10|Kate Perez|BIN Taxonomy Match|BOLD:ABA3995
Cricotopus cf. septentrionalis|EBAI-Ch181|Elisabeth Stur|morphology|BOLD:ABA3995
Cricotopus cf. septentrionalis|ATNA570|Elisabeth Stur||BOLD:ABA3995
Cricotopus cf. septentrionalis|TRD-CH191|Elisabeth Stur||BOLD:ABA3995
Cricotopus cf. septentrionalis|NO67|Elisabeth Stur||BOLD:ABA3995
Cricotopus cf. septentrionalis|NO66|Elisabeth Stur||BOLD:ABA3995
Cricotopus cf. septentrionalis|NO64|Elisabeth Stur||BOLD:ABA3995
Cricotopus cf. septentrionalis|BIOUG15900-H08|Kate Perez|BIN Taxonomy Match|BOLD:ABA3995
Heleniella|BIOUG36987-F07|Kate Perez|BIN Taxonomy Match (Jul 2018)|BOLD:AAN5342
Heleniella|BIOUG36992-C03|Kate Perez|BIN Taxonomy Match (Jul 2018)|BOLD:AAN5342
Heleniella|BIOUG36992-E12|Kate Perez|BIN Taxonomy Match (Jul 2018)|BOLD:AAN5342
Heleniella|BIOUG36841-D10|Kate Perez|BIN Taxonomy Match (Jul 2018)|BOLD:AAN5342
Heleniella|BIOUG36677-D12|Kate Perez|BIN Taxonomy Match (May 2018)|BOLD:AAN5342
Heleniella|BIOUG36677-G09|Kate Perez|BIN Taxonomy Match (May 2018)|BOLD:AAN5342
Heleniella|BIOUG36680-E08|Kate Perez|BIN Taxonomy Match (May 2018)|BOLD:AAN5342
Heleniella|BIOUG36682-A06|Kate Perez|BIN Taxonomy Match (Jul 2018)|BOLD:AAN5342
Heleniella|BIOUG36681-G02|Kate Perez|BIN Taxonomy Match (Jul 2018)|BOLD:AAN5342
Heleniella|BIOUG36934-E10|Kate Perez|BIN Taxonomy Match (Jul 2018)|BOLD:AAN5342
Heleniella|BIOUG36931-B10|Kate Perez|BIN Taxonomy Match (May 2018)|BOLD:AAN5342
Heleniella|BIOUG36934-C04|Kate Perez|BIN Taxonomy Match (Jul 2018)|BOLD:AAN5342
Heleniella|BIOUG36779-A02|Kate Perez|BIN Taxonomy Match (May 2018)|BOLD:AAN5342
Heleniella|BIOUG36931-A07|Kate Perez|BIN Taxonomy Match (May 2018)|BOLD:AAN5342
Heleniella|BIOUG36681-H05|Kate Perez|BIN Taxonomy Match (Jul 2018)|BOLD:AAN5342
Heleniella|BIOUG36681-C03|Kate Perez|BIN Taxonomy Match (Jul 2018)|BOLD:AAN5342
Heleniella|BIOUG36934-A11|Kate Perez|BIN Taxonomy Match (Jul 2018)|BOLD:AAN5342
Heleniella|BIOUG36776-B09|Kate Perez|BIN Taxonomy Match (Jul 2018)|BOLD:AAN5342
Heleniella|BIOUG36932-C11|Kate Perez|BIN Taxonomy Match (Jul 2018)|BOLD:AAN5342
Heleniella|BIOUG36933-C11|Kate Perez|BIN Taxonomy Match (Jul 2018)|BOLD:AAN5342
Heleniella|BIOUG36762-D09|Kate Perez|BIN Taxonomy Match (May 2018)|BOLD:AAN5342
Heleniella|BIOUG36820-F02|Kate Perez|BIN Taxonomy Match (Jul 2018)|BOLD:AAN5342
Heleniella|BIOUG36987-F12|Kate Perez|BIN Taxonomy Match (Jul 2018)|BOLD:AAN5342
Heleniella|BIOUG36778-C09|Kate Perez|BIN Taxonomy Match (Jul 2018)|BOLD:AAN5342
Heleniella|BIOUG36677-C04|Kate Perez|BIN Taxonomy Match (May 2018)|BOLD:AAN5342
Heleniella|BIOUG36677-C08|Kate Perez|BIN Taxonomy Match (May 2018)|BOLD:AAN5342

Heleniella|BIOUG36987-F12|Kate Perez|BIN Taxonomy Match (Jul 2018)|BOLD:AAN5342
Heleniella|BIOUG36778-C09|Kate Perez|BIN Taxonomy Match (Jul 2018)|BOLD:AAN5342
Heleniella|BIOUG36677-C04|Kate Perez|BIN Taxonomy Match (May 2018)|BOLD:AAN5342
Heleniella|BIOUG36677-G08|Kate Perez|BIN Taxonomy Match (May 2018)|BOLD:AAN5342
Heleniella|BIOUG36677-H11|Kate Perez|BIN Taxonomy Match (May 2018)|BOLD:AAN5342
Heleniella|BIOUG36680-D08|Kate Perez|BIN Taxonomy Match (May 2018)|BOLD:AAN5342
Heleniella|BIOUG36680-F06|Kate Perez|BIN Taxonomy Match (May 2018)|BOLD:AAN5342
Heleniella|BIOUG36653-C12|Kate Perez|BIN Taxonomy Match (Jul 2018)|BOLD:AAN5342
Heleniella|BIOUG36846-D09|Kate Perez|BIN Taxonomy Match (Jul 2018)|BOLD:AAN5342
Heleniella|BIOUG36778-E04|Kate Perez|BIN Taxonomy Match (Jul 2018)|BOLD:AAN5342
Heleniella|BIOUG36778-G04|Kate Perez|BIN Taxonomy Match (Jul 2018)|BOLD:AAN5342
Heleniella|BIOUG36819-B06|Kate Perez|BIN Taxonomy Match (Jul 2018)|BOLD:AAN5342
Heleniella|BIOUG36933-F01|Kate Perez|BIN Taxonomy Match (Jul 2018)|BOLD:AAN5342
Heleniella|BIOUG36933-C08|Kate Perez|BIN Taxonomy Match (Jul 2018)|BOLD:AAN5342
Heleniella|BIOUG36987-H06|Kate Perez|BIN Taxonomy Match (Jul 2018)|BOLD:AAN5342
Heleniella|BIOUG36775-A01|Kate Perez|BIN Taxonomy Match (Jul 2018)|BOLD:AAN5342
Heleniella|BIOUG36777-D06|Kate Perez|BIN Taxonomy Match (Jul 2018)|BOLD:AAN5342
Heleniella|BIOUG36841-A06|Kate Perez|BIN Taxonomy Match (Jul 2018)|BOLD:AAN5342
Heleniella|BIOUG42927-B03|Kate Perez|BIN Taxonomy Match below Phylum (Jun 2019)|BOLD:AAN...
Heleniella|BIOUG42662-G10|Kate Perez|BIN Taxonomy Match below Phylum (Jun 2019)|BOLD:AAN...
Heleniella|BIOUG42941-F02|Kate Perez|BIN Taxonomy Match below Phylum (Jun 2019)|BOLD:AAN...
Heleniella|BIOUG55188-D07|Stefan Schmidt|BIN Taxonomy Match (Sep 2020)|BOLD:AAN5342
Heleniella|BIOUG42678-D06|Kate Perez|BIN Taxonomy Match below Phylum (Jun 2019)|BOLD:AAN...
Heleniella|BIOUG55192-B11|Stefan Schmidt|BIN Taxonomy Match (Sep 2020)|BOLD:AAN5342
Heleniella|BIOUG42968-F03|Kate Perez|BIN Taxonomy Match below Phylum (Jun 2019)|BOLD:AAN...
Heleniella|BIOUG55420-H10|Stefan Schmidt|BIN Taxonomy Match (Sep 2020)|BOLD:AAN5342
Heleniella|BIOUG42760-F10|Kate Perez|BIN Taxonomy Match below Phylum (Jun 2019)|BOLD:AAN...
Heleniella|BIOUG42939-A05|Kate Perez|BIN Taxonomy Match below Phylum (Jun 2019)|BOLD:AAN...
Heleniella|BIOUG16530-B04|Kate Perez|BIN Taxonomy Match|BOLD:AAN5342
Heleniella|BIOUG16613-A03|Kate Perez|BIN Taxonomy Match|BOLD:AAN5342
Heleniella|BIOUG36677-F05|Kate Perez|BIN Taxonomy Match (May 2018)|BOLD:AAN5342
Heleniella|BIOUG36680-F04|Kate Perez|BIN Taxonomy Match (May 2018)|BOLD:AAN5342
Heleniella|BIOUG36854-G09|Kate Perez|BIN Taxonomy Match (Jul 2018)|BOLD:AAN5342
Heleniella|BIOUG36934-H11|Kate Perez|BIN Taxonomy Match (Jul 2018)|BOLD:AAN5342
Heleniella|BIOUG36766-A10|Kate Perez|BIN Taxonomy Match (Jul 2018)|BOLD:AAN5342
Heleniella|BIOUG36681-E02|Kate Perez|BIN Taxonomy Match (Jul 2018)|BOLD:AAN5342
Heleniella|BIOUG36994-G10|Kate Perez|BIN Taxonomy Match (Jul 2018)|BOLD:AAN5342
Heleniella|BIOUG36682-A11|Kate Perez|BIN Taxonomy Match (Jul 2018)|BOLD:AAN5342
Heleniella|BIOUG36820-B03|Kate Perez|BIN Taxonomy Match (Jul 2018)|BOLD:AAN5342
Heleniella|BIOUG36846-A02|Kate Perez|BIN Taxonomy Match (Jul 2018)|BOLD:AAN5342
Heleniella|BIOUG36775-C01|Kate Perez|BIN Taxonomy Match (Jul 2018)|BOLD:AAN5342
Heleniella|BIOUG36778-D07|Kate Perez|BIN Taxonomy Match (Jul 2018)|BOLD:AAN5342
Heleniella|BIOUG16736-G05|Kate Perez|BIN Taxonomy Match|BOLD:AAN5342
Heleniella|BIOUG36820-F01|Kate Perez|BIN Taxonomy Match (Jul 2018)|BOLD:AAN5342
Heleniella|BIOUG36818-B07|Kate Perez|BIN Taxonomy Match (Jul 2018)|BOLD:AAN5342
Heleniella|BIOUG36766-C06|Kate Perez|BIN Taxonomy Match (Jul 2018)|BOLD:AAN5342
Heleniella|BIOUG16733-H08|Kate Perez|BIN Taxonomy Match|BOLD:AAN5342
Heleniella|BIOUG16619-F10|Kate Perez|BIN Taxonomy Match (Feb 2020)|BOLD:AAN5342
Heleniella|BIOUG36650-B06|Kate Perez|BIN Taxonomy Match (Jul 2018)|BOLD:AAN5342
Heleniella|BIOUG16736-G04|Kate Perez|BIN Taxonomy Match|BOLD:AAN5342
Heleniella|BIOUG17137-G05|Kate Perez|BIN Taxonomy Match|BOLD:AAN5342
Heleniella|BIOUG16705-H02|Kate Perez|BIN Taxonomy Match|BOLD:AAN5342
Heleniella|BIOUG16277-D05|Kate Perez|BIN Taxonomy Match|BOLD:AAN5342
Heleniella|BIOUG16270-E04|Kate Perez|BIN Taxonomy Match|BOLD:AAN5342
Cricotopus pulchripes|ATNA546|Elisabeth Stur||BOLD:AAP5927
Cricotopus pulchripes|Finnmark163|Susan E. Gresens||BOLD:AAP5927
Cricotopus pulchripes|ZMUO.026098|Lauri Paasivirta||BOLD:AAP5927
Cricotopus pulchripes|Finnmark253|Susan E. Gresens||BOLD:AAP5927
Cricotopus pulchripes|Finnmark616|Elisabeth Stur||BOLD:AAP5927
Cricotopus fuscus|ZMUO.024963|Lauri Paasivirta||BOLD:ADA8628
Cricotopus fuscus|ZMUO.024962|Lauri Paasivirta||BOLD:ADA8628
Cricotopus annulator|ZSM-DIP-33148-G07|Caroline Chimeno|BIN Taxonomy Match (Jan 2022)|BOLD...
Cricotopus annulator|CCDB21606-F10|Sofia Wiedenbrug||BOLD:ACT8609
Cricotopus annulator|ZSM-DIP-33172-D11|Caroline Chimeno|BIN Taxonomy Match (Jan 2022)|BOLD...
Cricotopus annulator|ZSM-DIP-33146-G10|Caroline Chimeno|BIN Taxonomy Match (Jan 2022)|BOLD...
Cricotopus albiforceps|NHRS-BYWS000001126|Yngve Brodin|morphology|BOLD:ADK2789
Cricotopus albiforceps|NHRS-BYWS000001095|Yngve Brodin|morphology|BOLD:ADK2789
Cricotopus albiforceps|ZSM-DIP-33145-A01|Caroline Chimeno|BIN Taxonomy Match (Jan 2022)|BOL...
Cricotopus|Finnmark886|G. A. Halvorsen||BOLD:ADK2789
Cricotopus pallidipes|ZMUO.026244|Lauri Paasivirta||BOLD:ACQ9767
Cricotopus pallidipes|BIOUG16259-C03|Kate Perez|BIN Taxonomy Match (Aug 2017)|BOLD:ACQ9767
Cricotopus arcuatus|PP0277|Lauri Paasivirta||BOLD:ADY6733
Cricotopus obtusus|ZMUO.026146|Lauri Paasivirta||BOLD:ADF0225
Cricotopus obtusus|PP0269|Lauri Paasivirta||BOLD:ADF0225
Cricotopus laricomalis|ZMUO.024631|Lauri Paasivirta||BOLD:AAP5930
Cricotopus laricomalis|Finnmark673|Elisabeth Stur||BOLD:AAP5930
Cricotopus laricomalis|Finnmark233|Susan E. Gresens||BOLD:AAP5930
Cricotopus laricomalis|Finnmark235|Susan E. Gresens||BOLD:AAP5930
Cricotopus laricomalis|ZMUO.026097|Lauri Paasivirta||BOLD:AAP5930
Cricotopus laricomalis|Finnmark229|Susan E. Gresens||BOLD:AAP5930
Cricotopus laricomalis|Finnmark582|Susan E. Gresens||BOLD:AAP5930
Cricotopus obnixus|ZMUO.025348|Lauri Paasivirta||BOLD:ADF0008
Cricotopus obnixus|ZMUO.025347|Lauri Paasivirta||BOLD:ADF0008
Cricotopus obnixus|PP0272|Lauri Paasivirta||BOLD:ADF0008
Cricotopus maurii|ZMUO.026156|Lauri Paasivirta||BOLD:ADF2216
Cricotopus maurii|NHRS-BYWS000000288|Yngve Brodin|morphology|BOLD:ADF2216
Cricotopus maurii|NHRS-BYWS000000287|Yngve Brodin|morphology|BOLD:ADF2216
Cricotopus brevipalpis|NO52|Elisabeth Stur||BOLD:ABA3994
Cricotopus brevipalpis|NO51|Elisabeth Stur||BOLD:ABA3994
Cricotopus ornatus|NHRS-BYWS000001773|Yngve Brodin||BOLD:AAP5926
Cricotopus ornatus|NHRS-BYWS000000270|Yngve Brodin|morphology|BOLD:AAP5926
Cricotopus ornatus|Finnmark156|Elisabeth Stur||BOLD:AAP5926
Cricotopus ornatus|NHRS-BYWS000001767|Yngve Brodin||BOLD:AAP5926
Cricotopus ornatus|NHRS-BYWS000000321|Yngve Brodin|morphology|BOLD:AAP5926
Cricotopus ornatus|NHRS-BYWS000001827|Yngve Brodin||BOLD:AAP5926
Cricotopus ornatus|NHRS-BYWS000000419|Yngve Brodin|morphology|BOLD:AAP5926
Cricotopus ornatus|NHRS-BYWS000000469|Yngve Brodin|morphology|BOLD:AAP5926
Cricotopus ornatus|NHRS-BYWS000001774|Yngve Brodin||BOLD:AAP5926
Cricotopus ornatus|NHRS-BYWS000001785|Yngve Brodin||BOLD:AAP5926
Cricotopus ornatus|NHRS-BYWS000001784|Yngve Brodin||BOLD:AAP5926
Cricotopus ornatus|NHRS-BYWS000001772|Yngve Brodin||BOLD:AAP5926
Cricotopus ornatus|CZ2|Susan E. Gresens|Morphology|BOLD:AAP5926
Cricotopus ornatus|NHRS-BYWS000001775|Yngve Brodin||BOLD:AAP5926
[illegible]

Cricotopus ornatus|NHRS-BYWS000001772|Yngve Brodin||BOLD:AAP5926
Cricotopus ornatus|CZ2|Susan E. Gresens|Morphology|BOLD:AAP5926
Cricotopus ornatus|NHRS-BYWS000001775|Yngve Brodin||BOLD:AAP5926
Cricotopus ornatus|NHRS-BYWS000000477|Yngve Brodin|morphology|BOLD:AAP5926
Cricotopus ornatus|NHRS-BYWS000001766|Yngve Brodin||BOLD:AAP5926
Cricotopus ornatus|Finnmark639|Elisabeth Stur||BOLD:AAP5926
Cricotopus ornatus|Finnmark635|Elisabeth Stur||BOLD:AAP5926
Cricotopus maurii|ZMUO.026155|Lauri Paasivirta||BOLD:ACR2716
Cricotopus|ZSM-DIP-33133-F11|Caroline Chimeno|BIN Taxonomy Match (Jan 2022)|BOLD:ACR2716
Cricotopus trifasciatus|ZMUO.026171|Lauri Paasivirta||BOLD:ACR2716
Cricotopus trifasciatus|ZMUO.026170|Lauri Paasivirta||BOLD:ACR2716
Cricotopus suspiciosus|ZMUO.026246|Lauri Paasivirta||BOLD:ACR2716
Cricotopus suspiciosus|ZMUO.026245|Lauri Paasivirta||BOLD:ACR2716
Cricotopus sp. 32ES|TRD-CH135|Elisabeth Stur|Morphology|BOLD:ACR2716
Cricotopus laetus|ZMUO.025377|Lauri Paasivirta||BOLD:ADF1390
Cricotopus pilitarsis|NHRS-BYWS000000217|Yngve Brodin|morphology|BOLD:ACE7183
Cricotopus pilitarsis|ZMUO.025146|Lauri Paasivirta||BOLD:ACE7183
Cricotopus pilitarsis|ZMUO.025145|Lauri Paasivirta||BOLD:ACE7183
Cricotopus pilitarsis|NHRS-BYWS000000336|Yngve Brodin|morphology|BOLD:ACE7183
Cricotopus pilitarsis|NHRS-BYWS000000582|Yngve Brodin|morphology|BOLD:ACE7183
Cricotopus pilitarsis|NHRS-BYWS000000569|Yngve Brodin|morphology|BOLD:ACE7183
Cricotopus pilitarsis|NHRS-BYWS000000568|Yngve Brodin|morphology|BOLD:ACE7183
Cricotopus pilitarsis|NHRS-BYWS000000457|Yngve Brodin|morphology|BOLD:ACE7183
Cricotopus pilitarsis|NHRS-BYWS000000456|Yngve Brodin|morphology|BOLD:ACE7183
Cricotopus pilitarsis|NO 77|Torbjorn Ekrem|Morphology|BOLD:ACE7183
Cricotopus tricinctus|ZMUO.026247|Lauri Paasivirta||BOLD:AEG4456
Cricotopus|BIOUG36735-D11|Kate Perez|BIN Taxonomy Match (May 2018)|BOLD:AEG4456
Cricotopus|BIOUG06095-B06|Kate Perez|BIN Taxonomy Match|BOLD:AEG4456
Cricotopus|BIOUG06088-C07|Kate Perez|BIN Taxonomy Match|BOLD:AEG4456
Cricotopus sp. 5ES|Finnmark356|Elisabeth Stur||BOLD:AAV1709
Cricotopus trifasciatus|SEG116|Susan E. Gresens||BOLD:ACX4176
Cricotopus trifasciatus|SEG118|Susan E. Gresens||BOLD:ACX4176
Cricotopus trifasciatus|SEG117|Susan E. Gresens||BOLD:ACX4176
Cricotopus trifasciatus|SEG120|Susan E. Gresens||BOLD:ACX4176
Cricotopus laetus|ZMUO.025376|Lauri Paasivirta||BOLD:AAA5299
Cricotopus sylvestris|NHRS-BYWS000001776|Yngve Brodin||BOLD:AAA5299
Cricotopus sylvestris|SEG124|Susan E. Gresens||BOLD:AAA5299
Cricotopus sylvestris|NHRS-BYWS000000504|Yngve Brodin|morphology|BOLD:AAA5299
Cricotopus sylvestris|NHRS-BYWS000000458|Yngve Brodin|morphology|BOLD:AAA5299
Cricotopus sylvestris|NHRS-BYWS000001740|Yngve Brodin||BOLD:AAA5299
Cricotopus sylvestris|ZMUO.024561|Lauri Paasivirta||BOLD:AAA5299
Cricotopus sylvestris|NHRS-BYWS000001840|Yngve Brodin||BOLD:AAA5299
Cricotopus sylvestris|NHRS-BYWS000001794|Yngve Brodin||BOLD:AAA5299
Cricotopus sylvestris|NHRS-BYWS000001742|Yngve Brodin||BOLD:AAA5299
Cricotopus sylvestris|NHRS-BYWS000000403|Yngve Brodin|morphology|BOLD:AAA5299
Cricotopus sylvestris|NHRS-BYWS000001741|Yngve Brodin||BOLD:AAA5299
Cricotopus sylvestris|NHRS-BYWS000000380|Yngve Brodin|morphology|BOLD:AAA5299
Cricotopus sylvestris|NHRS-BYWS000000489|Yngve Brodin|morphology|BOLD:AAA5299
Cricotopus sylvestris|NHRS-BYWS000000335|Yngve Brodin|morphology|BOLD:AAA5299
Cricotopus sylvestris|RIN_CH83|Mona Renate Saursaunet|BOLD Identification engine|BOLD:AAA5299
Cricotopus sylvestris|RIN_CH37|Mona Renate Saursaunet|BOLD Identification engine|BOLD:AAA5299
Cricotopus|BIOUG42760-G11|Kate Perez|BIN Taxonomy Match below Phylum (Jun 2019)|BOLD:AAA...
Cricotopus|BIOUG42671-G03|Kate Perez|BIN Taxonomy Match below Phylum (Jun 2019)|BOLD:AAA...
Cricotopus|BIOUG55116-E11|Stefan Schmidt|BIN Taxonomy Match (Sep 2020)|BOLD:AAA5299
Cricotopus|BIOUG42743-C06|Kate Perez|BIN Taxonomy Match below Phylum (Jun 2019)|BOLD:AAA...
Cricotopus sylvestris|NHRS-BYWS000001149|Yngve Brodin|morphology|BOLD:AAA5299
Cricotopus|BIOUG42744-C06|Kate Perez|BIN Taxonomy Match below Phylum (Jun 2019)|BOLD:AAA...
Cricotopus sylvestris|EJV-20110106|Lauri Paasivirta||BOLD:AAA5299
Cricotopus sylvestris|NHRS-BYWS000001739|Yngve Brodin||BOLD:AAA5299
Cricotopus sylvestris|RIN_CH26|Mona Renate Saursaunet|BOLD Identification engine|BOLD:AAA5299
Cricotopus sylvestris|SEG122|Susan E. Gresens||BOLD:AAA5299
Cricotopus sylvestris|TRD-CH51|Elisabeth Stur||BOLD:AAA5299
Cricotopus sylvestris|RIN_CH66|Mona Renate Saursaunet|BOLD Identification engine|BOLD:AAA5299
Cricotopus sylvestris|RIN_CH60|Mona Renate Saursaunet|BOLD Identification engine|BOLD:AAA5299
Cricotopus sylvestris|RIN_CH07|Mona Renate Saursaunet|morphology|BOLD:AAA5299
Cricotopus sylvestris|RIN_CH03|Mona Renate Saursaunet|BOLD Identification engine|BOLD:AAA5299
Cricotopus sylvestris|RIN_CH57|Mona Renate Saursaunet|BOLD Identification engine|BOLD:AAA5299
Cricotopus sylvestris|RIN_CH90|Mona Renate Saursaunet|BOLD Identification engine|BOLD:AAA5299
Cricotopus sylvestris|CCDB24228-B02|Sofia Wiedenbrug|BIN Taxonomy Match|BOLD:AAA5299
Cricotopus sylvestris|CCDB24228-A08|Sofia Wiedenbrug|BIN Taxonomy Match|BOLD:AAA5299
Cricotopus sylvestris|CCDB24228-A07|Sofia Wiedenbrug|BIN Taxonomy Match|BOLD:AAA5299
Chironomidae|CCDB24228-B01||BIN Taxonomy Match
Cricotopus|ZSM34343-H01|Sofia Wiedenbrug|Morphology
Cricotopus|BIOUG42752-B09|Kate Perez|BIN Taxonomy Match below Phylum (Jun 2019)|BOLD:AAA...
Cricotopus sylvestris|NHRS-BYWS000000251|Yngve Brodin|morphology|BOLD:AAA5299
Cricotopus glacialis|CH-eik72|Elisabeth Stur||BOLD:AAA5299
Cricotopus sylvestris|NHRS-BYWS000000434|Yngve Brodin|morphology|BOLD:AAA5299
Cricotopus glacialis|CH-OSF187|Elisabeth Stur||BOLD:AAA5299
Cricotopus|BIOUG36649-B02|Kate Perez|BIN Taxonomy Match (Jan 2019)|BOLD:AAA5299
Cricotopus sylvestris|RIN_CH44|Mona Renate Saursaunet|BOLD Identification engine|BOLD:AAA5299
Cricotopus sylvestris|NHRS-BYWS000001823|Yngve Brodin||BOLD:AAA5299
Cricotopus sylvestris|EJV-20110105|Lauri Paasivirta||BOLD:AAA5299
Cricotopus sylvestris|EJV-20110108|Lauri Paasivirta||BOLD:AAA5299
Cricotopus sylvestris|EJV-20110107|Lauri Paasivirta||BOLD:AAA5299
Cricotopus|ZSM-DIP-33170-B11|Caroline Chimeno|BIN Taxonomy Match (Jan 2022)|BOLD:AAA5299
Cricotopus sylvestris|CZ8|Susan E. Gresens||BOLD:AAA5299
Cricotopus sylvestris|NHRS-BYWS000001087|Yngve Brodin|morphology|BOLD:AAA5299
Cricotopus sylvestris|CZ1|Susan E. Gresens|Morphology|BOLD:AAA5299
Cricotopus sylvestris|CZ9|Susan E. Gresens||BOLD:AAA5299
Cricotopus sylvestris|CZ5|Susan E. Gresens|Morphology|BOLD:AAA5299
Cricotopus sylvestris|CZ4|Susan E. Gresens|Morphology|BOLD:AAA5299
Cricotopus sylvestris|NHRS-BYWS000000478|Yngve Brodin|morphology|BOLD:AAA5299
Cricotopus sylvestris|BC-ZSM-DIP-22551-F02|Dieter Doczkal||BOLD:AAA5299
Cricotopus glacialis|NO50|Elisabeth Stur||BOLD:AAA5299
Cricotopus|ZSM-DIP-33147-F04|Caroline Chimeno|BIN Taxonomy Match (Jan 2022)|BOLD:AAA5299
Cricotopus sylvestris|CZ7|Susan E. Gresens||BOLD:AAA5299
Cricotopus sylvestris|ZMUO.024562|Lauri Paasivirta||BOLD:AAA5299
Cricotopus sylvestris|ZSM34343-C11|Sofia Wiedenbrug|Morphology|BOLD:AAA5299
Cricotopus|BIOUG36992-G06|Kate Perez|BIN Taxonomy Match (Jan 2019)|BOLD:AAA5299
Cricotopus sylvestris|NHRS-BYWS000001044|Yngve Brodin|morphology|BOLD:AAA5299
Cricotopus sylvestris|NHRS-BYWS000000548|Yngve Brodin|morphology|BOLD:AAA5299
Cricotopus sylvestris|NHRS-BYWS000000337|Yngve Brodin|morphology|BOLD:AAA5299
Cricotopus sylvestris|NHRS-BYWS000000218|Yngve Brodin|morphology|BOLD:AAA5299

Cricotopus sylvestris|NHRS-BYWS000000548|Yngve Brodin|morphology|BOLD:AAA5299
Cricotopus sylvestris|NHRS-BYWS000000337|Yngve Brodin|morphology|BOLD:AAA5299
Cricotopus sylvestris|NHRS-BYWS000000218|Yngve Brodin|morphology|BOLD:AAA5299
Cricotopus|ES400|Elisabeth Stur|morphology|BOLD:AAA5299
Cricotopus|ES395|Elisabeth Stur|morphology|BOLD:AAA5299
Cricotopus glacialis|TRD-CH52|Elisabeth Stur||BOLD:AAA5299
Cricotopus sylvestris|NO74|Elisabeth Stur||BOLD:AAA5299
Cricotopus sylvestris|RIN_CH56|Mona Renate Saursaunet|BOLD Identification engine|BOLD:AAA5299
Cricotopus sylvestris|RIN_CH55|Mona Renate Saursaunet|BOLD Identification engine|BOLD:AAA5299
Cricotopus sylvestris|RIN_CH41|Mona Renate Saursaunet|BOLD Identification engine|BOLD:AAA5299
Cricotopus sylvestris|RIN_CH36|Mona Renate Saursaunet|BOLD Identification engine|BOLD:AAA5299
Cricotopus sylvestris|RIN_CH17|Mona Renate Saursaunet|BOLD Identification engine|BOLD:AAA5299
Cricotopus sylvestris|RIN_CH16|Mona Renate Saursaunet|BOLD Identification engine|BOLD:AAA5299
Cricotopus sylvestris|TRD-CH41|Elisabeth Stur||BOLD:AAA5299
Cricotopus glacialis|TRD-CH10|Elisabeth Stur||BOLD:AAA5299
Cricotopus sylvestris|NHRS-BYWS000001795|Yngve Brodin||BOLD:AAA5299
Cricotopus glacialis|NO62|Elisabeth Stur||BOLD:AAA5299
Cricotopus glacialis|NO49|Elisabeth Stur||BOLD:AAA5299
Cricotopus relucens|ZMUO.025156|Lauri Paasivirta||BOLD:AAA5299
Cricotopus relucens|ZMUO.025155|Lauri Paasivirta||BOLD:AAA5299
Cricotopus sylvestris|NHRS-BYWS000000961|Yngve Brodin|morphology|BOLD:AAA5299
Cricotopus sp. 3ES|Finnmark175|Elisabeth Stur||BOLD:AAA5299
Cricotopus sp. 3ES|Finnmark874|Elisabeth Stur|morphology|BOLD:AAA5299
Cricotopus sp. 3ES|Finnmark192|Elisabeth Stur||BOLD:AAA5299
Cricotopus sp. 3ES|Finnmark512|Elisabeth Stur||BOLD:AAA5299
Cricotopus sp. 3ES|Finnmark580|Elisabeth Stur||BOLD:AAA5299
Paracladius quadrinodosus|BC-ZSM-DIP-22551-F09|Dieter Doczkal||BOLD:ACX3335
Paracladius quadrinodosus|TRD-CH372|Elisabeth Stur|morphology|BOLD:ACX3335
Paracladius conversus|ZMUO.024583|Lauri Paasivirta||BOLD:ADA6481
Paracladius conversus|ZMUO.024582|Lauri Paasivirta||BOLD:ADA6481
Cricotopus perniger|Finnmark203|Susan E. Gresens||BOLD:AAP5929
Paracladius quadrinodosus|ZMUO.026056|Lauri Paasivirta||BOLD:ADF1770
Paracladius alpicola|ES388|Elisabeth Stur||BOLD:AAU3206
Paracladius alpicola|ES378|Elisabeth Stur||BOLD:AAU3206
Paracladius alpicola|Finnmark401|Elisabeth Stur||BOLD:AAU3206
Paracladius alpicola|Finnmark400|Elisabeth Stur||BOLD:AAU3206
Paracladius alpicola|Finnmark389|Elisabeth Stur||BOLD:AAU3206
Cricotopus|BIOUG16218-E01|Kate Perez|BOLD ID Engine Manual|BOLD:ACQ9824
Cricotopus tremulus|BIOUG16478-D05|Kate Perez|BIN Taxonomy Match|BOLD:AAM6021
Cricotopus tremulus|Finnmark130|Elisabeth Stur||BOLD:AAM6021
Cricotopus tremulus|BIOUG16435-F11|Kate Perez|BIN Taxonomy Match|BOLD:AAM6021
Cricotopus tremulus|BIOUG15951-C03|Kate Perez|BIN Taxonomy Match|BOLD:AAM6021
Cricotopus tremulus|BIOUG15954-G06|Kate Perez|BIN Taxonomy Match|BOLD:AAM6021
Cricotopus tremulus|BIOUG16426-H11|Kate Perez|BIN Taxonomy Match|BOLD:AAM6021
Cricotopus tremulus|BIOUG16430-D04|Kate Perez|BIN Taxonomy Match|BOLD:AAM6021
Cricotopus tremulus|BIOUG15954-E05|Kate Perez|BIN Taxonomy Match|BOLD:AAM6021
Cricotopus tremulus|BIOUG15967-A11|Kate Perez|BIN Taxonomy Match|BOLD:AAM6021
Cricotopus tremulus|BIOUG16198-A04|Kate Perez|BIN Taxonomy Match|BOLD:AAM6021
Cricotopus tremulus|BIOUG16258-A11|Kate Perez|BIN Taxonomy Match|BOLD:AAM6021
Cricotopus tremulus|BIOUG15954-C11|Kate Perez|BIN Taxonomy Match|BOLD:AAM6021
Cricotopus tremulus|BIOUG16477-A07|Kate Perez|BIN Taxonomy Match|BOLD:AAM6021
Cricotopus tremulus|BIOUG16258-E09|Kate Perez|BIN Taxonomy Match|BOLD:AAM6021
Cricotopus tremulus|BIOUG15954-G05|Kate Perez|BIN Taxonomy Match|BOLD:AAM6021
Cricotopus tremulus|BIOUG15952-B04|Kate Perez|BIN Taxonomy Match|BOLD:AAM6021
Cricotopus tremulus|BIOUG15951-B05|Kate Perez|BIN Taxonomy Match|BOLD:AAM6021
Cricotopus tremulus|BIOUG15948-G08|Kate Perez|BIN Taxonomy Match|BOLD:AAM6021
Cricotopus tremulus|BIOUG15947-G07|Kate Perez|BIN Taxonomy Match|BOLD:AAM6021
Cricotopus tremulus|BIOUG15947-F03|Kate Perez|BIN Taxonomy Match|BOLD:AAM6021
Cricotopus tremulus|BIOUG15947-A09|Kate Perez|BIN Taxonomy Match|BOLD:AAM6021
Cricotopus tremulus|BIOUG15954-A09|Kate Perez|BIN Taxonomy Match|BOLD:AAM6021
Cricotopus tremulus|BIOUG16477-D08|Kate Perez|BIN Taxonomy Match|BOLD:AAM6021
Cricotopus tremulus|BIOUG15949-H02|Kate Perez|BIN Taxonomy Match|BOLD:AAM6021
Cricotopus tremulus|BIOUG16478-G07|Kate Perez|BIN Taxonomy Match|BOLD:AAM6021
Cricotopus tremulus|BIOUG16478-D11|Kate Perez|BIN Taxonomy Match|BOLD:AAM6021
Cricotopus tremulus|BIOUG15905-D11|Kate Perez|BIN Taxonomy Match|BOLD:AAM6021
Cricotopus tremulus|BIOUG15950-A01|Kate Perez|BIN Taxonomy Match|BOLD:AAM6021
Cricotopus tremulus|BIOUG16333-D03|Kate Perez|BIN Taxonomy Match|BOLD:AAM6021
Cricotopus tremulus|BIOUG15948-G03|Kate Perez|BIN Taxonomy Match|BOLD:AAM6021
Cricotopus tremulus|BIOUG15905-E09|Kate Perez|BIN Taxonomy Match|BOLD:AAM6021
Cricotopus tremulus|BIOUG16479-G01|Kate Perez|BIN Taxonomy Match|BOLD:AAM6021
Cricotopus tremulus|BIOUG15947-C06|Kate Perez|BIN Taxonomy Match|BOLD:AAM6021
Cricotopus tremulus|BIOUG16198-E10|Kate Perez|BIN Taxonomy Match|BOLD:AAM6021
Cricotopus tremulus|BIOUG15967-E12|Kate Perez|BIN Taxonomy Match|BOLD:AAM6021
Cricotopus tremulus|BIOUG16480-B02|Kate Perez|BIN Taxonomy Match|BOLD:AAM6021
Cricotopus tremulus|BIOUG15950-D08|Kate Perez|BIN Taxonomy Match|BOLD:AAM6021
Cricotopus tremulus|BIOUG16479-A04|Kate Perez|BIN Taxonomy Match|BOLD:AAM6021
Cricotopus tremulus|BIOUG15954-D08|Kate Perez|BIN Taxonomy Match|BOLD:AAM6021
Cricotopus tremulus|BIOUG15905-B08|Kate Perez|BIN Taxonomy Match|BOLD:AAM6021
Cricotopus tremulus|BIOUG16432-B08|Kate Perez|BIN Taxonomy Match|BOLD:AAM6021
Cricotopus tremulus|BIOUG15900-G12|Kate Perez|BIN Taxonomy Match|BOLD:AAM6021
Cricotopus tremulus|BIOUG15963-D02|Kate Perez|BIN Taxonomy Match|BOLD:AAM6021
Cricotopus tremulus|BIOUG15963-D12|Kate Perez|BIN Taxonomy Match|BOLD:AAM6021
Cricotopus tremulus|BIOUG16261-C05|Kate Perez|BIN Taxonomy Match|BOLD:AAM6021
Cricotopus tremulus|BIOUG16261-G07|Kate Perez|BIN Taxonomy Match|BOLD:AAM6021
Cricotopus tremulus|BIOUG16433-C03|Kate Perez|BIN Taxonomy Match|BOLD:AAM6021
Cricotopus tremulus|BIOUG16430-G05|Kate Perez|BIN Taxonomy Match|BOLD:AAM6021
Cricotopus tremulus|BIOUG15953-E10|Kate Perez|BIN Taxonomy Match|BOLD:AAM6021
Cricotopus tremulus|ZMUO.026159|Lauri Paasivirta||BOLD:AAM6021
Cricotopus tremulus|BIOUG15951-H10|Kate Perez|BIN Taxonomy Match|BOLD:AAM6021
Cricotopus tremulus|BIOUG15950-C06|Kate Perez|BIN Taxonomy Match|BOLD:AAM6021
Cricotopus tremulus|BIOUG16432-A09|Kate Perez|BIN Taxonomy Match|BOLD:AAM6021
Cricotopus tremulus|ZMUO.026158|Lauri Paasivirta||BOLD:AAM6021
Cricotopus tremulus|BIOUG16259-E12|Kate Perez|BIN Taxonomy Match|BOLD:AAM6021
Cricotopus tremulus|BIOUG16332-D01|Kate Perez|BIN Taxonomy Match|BOLD:AAM6021
Cricotopus tremulus|BIOUG16597-F12|Kate Perez|BIN Taxonomy Match|BOLD:AAM6021
Cricotopus tremulus|BIOUG16598-A05|Kate Perez|BIN Taxonomy Match|BOLD:AAM6021
Cricotopus tremulus|BIOUG15947-B12|Kate Perez|BIN Taxonomy Match|BOLD:AAM6021
Cricotopus tremulus|BIOUG15948-F03|Kate Perez|BIN Taxonomy Match|BOLD:AAM6021
Cricotopus tremulus|BIOUG15948-H05|Kate Perez|BIN Taxonomy Match|BOLD:AAM6021
Cricotopus tremulus|BIOUG15954-C10|Kate Perez|BIN Taxonomy Match|BOLD:AAM6021
Cricotopus tremulus|BIOUG15963-C02|Kate Perez|BIN Taxonomy Match|BOLD:AAM6021
Cricotopus tremulus|BIOUG15966-G10|Kate Perez|BIN Taxonomy Match|BOLD:AAM6021
Cricotopus tremulus|BIOUG15799-G11|Kate Perez|BIN Taxonomy Match|BOLD:AAM6021

[illegible]
Cricotopus tremulus|BIOUG15963-C02|Kate Perez|BIN Taxonomy Match|BOLD:AAM6021
Cricotopus tremulus|BIOUG15966-G10|Kate Perez|BIN Taxonomy Match|BOLD:AAM6021
Cricotopus tremulus|BIOUG15799-G11|Kate Perez|BIN Taxonomy Match|BOLD:AAM6021
Cricotopus tremulus|BIOUG15967-H11|Kate Perez|BIN Taxonomy Match|BOLD:AAM6021
Cricotopus tremulus|BIOUG16198-E01|Kate Perez|BIN Taxonomy Match|BOLD:AAM6021
Cricotopus tremulus|BIOUG15964-B07|Kate Perez|BIN Taxonomy Match|BOLD:AAM6021
Cricotopus tremulus|BIOUG15965-A02|Kate Perez|BIN Taxonomy Match|BOLD:AAM6021
Cricotopus tremulus|BIOUG15947-C10|Kate Perez|BIN Taxonomy Match|BOLD:AAM6021
Cricotopus tremulus|BIOUG16257-H02|Kate Perez|BIN Taxonomy Match|BOLD:AAM6021
Cricotopus tremulus|BIOUG15905-D06|Kate Perez|BIN Taxonomy Match|BOLD:AAM6021
Cricotopus tremulus|BIOUG16434-F07|Kate Perez|BIN Taxonomy Match|BOLD:AAM6021
Cricotopus tremulus|BIOUG15950-F11|Kate Perez|BIN Taxonomy Match|BOLD:AAM6021
Cricotopus tremulus|BIOUG15965-H03|Kate Perez|BIN Taxonomy Match|BOLD:AAM6021
Cricotopus tremulus|BIOUG16297-B04|Kate Perez|BIN Taxonomy Match|BOLD:AAM6021
Cricotopus tremulus|BIOUG15795-H09|Kate Perez|BIN Taxonomy Match|BOLD:AAM6021
Cricotopus tremulus|BIOUG15967-G07|Kate Perez|BIN Taxonomy Match|BOLD:AAM6021
Cricotopus tremulus|BIOUG16431-A03|Kate Perez|BIN Taxonomy Match|BOLD:AAM6021
Cricotopus tremulus|BIOUG16331-G02|Kate Perez|BIN Taxonomy Match|BOLD:AAM6021
Cricotopus tremulus|BIOUG15965-H06|Kate Perez|BIN Taxonomy Match|BOLD:AAM6021
Cricotopus tremulus|BIOUG16478-H04|Kate Perez|BIN Taxonomy Match|BOLD:AAM6021
Cricotopus tremulus|BIOUG16431-C08|Kate Perez|BIN Taxonomy Match|BOLD:AAM6021
Cricotopus tremulus|BIOUG16436-G06|Kate Perez|BIN Taxonomy Match|BOLD:AAM6021
Cricotopus tremulus|BIOUG15953-D06|Kate Perez|BIN Taxonomy Match|BOLD:AAM6021
Cricotopus tremulus|BIOUG16478-F02|Kate Perez|BIN Taxonomy Match|BOLD:AAM6021
Cricotopus tremulus|BIOUG15951-F03|Kate Perez|BIN Taxonomy Match|BOLD:AAM6021
Cricotopus tremulus|BIOUG15950-B05|Kate Perez|BIN Taxonomy Match|BOLD:AAM6021
Cricotopus tremulus|BIOUG15900-G10|Kate Perez|BIN Taxonomy Match|BOLD:AAM6021
Cricotopus tremulus|BIOUG15949-F06|Kate Perez|BIN Taxonomy Match|BOLD:AAM6021
Cricotopus tremulus|BIOUG15905-F04|Kate Perez|BIN Taxonomy Match|BOLD:AAM6021
Cricotopus tremulus|BIOUG15791-G08|Kate Perez|BIN Taxonomy Match|BOLD:AAM6021
Cricotopus tremulus|BIOUG16478-D03|Kate Perez|BIN Taxonomy Match|BOLD:AAM6021
Cricotopus tremulus|BIOUG16296-E01|Kate Perez|BIN Taxonomy Match|BOLD:AAM6021
Cricotopus tremulus|BIOUG16594-G02|Kate Perez|BIN Taxonomy Match|BOLD:AAM6021
Cricotopus tremulus|BIOUG15954-B03|Kate Perez|BIN Taxonomy Match|BOLD:AAM6021
Cricotopus tremulus|BIOUG15951-D09|Kate Perez|BIN Taxonomy Match|BOLD:AAM6021
Cricotopus tremulus|BIOUG15948-E06|Kate Perez|BIN Taxonomy Match|BOLD:AAM6021
Cricotopus tremulus|BIOUG15948-C05|Kate Perez|BIN Taxonomy Match|BOLD:AAM6021
Cricotopus tremulus|BIOUG15950-C02|Kate Perez|BIN Taxonomy Match|BOLD:AAM6021
Cricotopus tremulus|BIOUG16479-C06|Kate Perez|BIN Taxonomy Match|BOLD:AAM6021
Cricotopus tremulus|BIOUG15952-C06|Kate Perez|BIN Taxonomy Match|BOLD:AAM6021
Cricotopus tremulus|BIOUG15905-D10|Kate Perez|BIN Taxonomy Match|BOLD:AAM6021
Cricotopus tremulus|BIOUG15948-F07|Kate Perez|BIN Taxonomy Match|BOLD:AAM6021
Cricotopus tremulus|BIOUG15948-H11|Kate Perez|BIN Taxonomy Match|BOLD:AAM6021
Cricotopus tremulus|BIOUG15967-A12|Kate Perez|BIN Taxonomy Match|BOLD:AAM6021
Cricotopus tremulus|BIOUG15954-F04|Kate Perez|BIN Taxonomy Match|BOLD:AAM6021
Cricotopus tremulus|BIOUG16329-B12|Kate Perez|BIN Taxonomy Match|BOLD:AAM6021
Cricotopus tremulus|BIOUG16431-A12|Kate Perez|BIN Taxonomy Match|BOLD:AAM6021
Cricotopus tremulus|BIOUG15796-B01|Kate Perez|BIN Taxonomy Match|BOLD:AAM6021
Cricotopus tremulus|BIOUG16296-G03|Kate Perez|BIN Taxonomy Match|BOLD:AAM6021
Cricotopus tremulus|BIOUG16480-A08|Kate Perez|BIN Taxonomy Match|BOLD:AAM6021
Cricotopus tremulus|BIOUG16435-F08|Kate Perez|BIN Taxonomy Match|BOLD:AAM6021
Cricotopus tremulus|BIOUG16435-B01|Kate Perez|BIN Taxonomy Match|BOLD:AAM6021
Cricotopus tremulus|BIOUG16298-F08|Kate Perez|BIN Taxonomy Match|BOLD:AAM6021
Cricotopus tremulus|BIOUG16256-C09|Kate Perez|BIN Taxonomy Match|BOLD:AAM6021
Cricotopus tremulus|BIOUG16256-G05|Kate Perez|BIN Taxonomy Match|BOLD:AAM6021
Cricotopus tremulus|BIOUG15947-A04|Kate Perez|BIN Taxonomy Match|BOLD:AAM6021
Cricotopus tremulus|BIOUG15905-E05|Kate Perez|BIN Taxonomy Match|BOLD:AAM6021
Cricotopus tremulus|BIOUG15949-G06|Kate Perez|BIN Taxonomy Match|BOLD:AAM6021
Cricotopus tremulus|BIOUG15905-C01|Kate Perez|BIN Taxonomy Match|BOLD:AAM6021
Cricotopus tremulus|BIOUG16332-A09|Kate Perez|BIN Taxonomy Match|BOLD:AAM6021
Cricotopus tremulus|BIOUG15905-H07|Kate Perez|BIN Taxonomy Match|BOLD:AAM6021
Cricotopus tremulus|BIOUG15951-A11|Kate Perez|BIN Taxonomy Match|BOLD:AAM6021
Cricotopus tremulus|BIOUG16479-D09|Kate Perez|BIN Taxonomy Match|BOLD:AAM6021
Cricotopus tremulus|BIOUG16625-F04|Kate Perez|BIN Taxonomy Match|BOLD:AAM6021
Cricotopus tremulus|BIOUG15963-C01|Kate Perez|BIN Taxonomy Match|BOLD:AAM6021
Cricotopus tremulus|BIOUG15964-G04|Kate Perez|BIN Taxonomy Match|BOLD:AAM6021
Cricotopus tremulus|BIOUG16257-A09|Kate Perez|BIN Taxonomy Match|BOLD:AAM6021
Cricotopus tremulus|BIOUG15948-D12|Kate Perez|BIN Taxonomy Match|BOLD:AAM6021
Cricotopus tremulus|BIOUG16477-B11|Kate Perez|BIN Taxonomy Match|BOLD:AAM6021
Cricotopus tremulus|BIOUG15964-H09|Kate Perez|BIN Taxonomy Match|BOLD:AAM6021
Cricotopus tremulus|BIOUG15905-C02|Kate Perez|BIN Taxonomy Match|BOLD:AAM6021
Cricotopus tremulus|BIOUG16434-B07|Kate Perez|BIN Taxonomy Match|BOLD:AAM6021
Cricotopus tremulus|BIOUG16436-A03|Kate Perez|BIN Taxonomy Match|BOLD:AAM6021
Cricotopus tremulus|BIOUG16436-D05|Kate Perez|BIN Taxonomy Match|BOLD:AAM6021
Cricotopus tremulus|BIOUG16434-H07|Kate Perez|BIN Taxonomy Match|BOLD:AAM6021
Cricotopus tremulus|BIOUG15952-D08|Kate Perez|BIN Taxonomy Match|BOLD:AAM6021
Cricotopus tremulus|BIOUG16478-F05|Kate Perez|BIN Taxonomy Match|BOLD:AAM6021
Cricotopus tremulus|BIOUG16205-A01|Kate Perez|BIN Taxonomy Match|BOLD:AAM6021
Cricotopus tremulus|BIOUG15952-A12|Kate Perez|BIN Taxonomy Match|BOLD:AAM6021
Cricotopus tremulus|BIOUG16477-C10|Kate Perez|BIN Taxonomy Match|BOLD:AAM6021
Cricotopus tremulus|BIOUG15951-D06|Kate Perez|BIN Taxonomy Match|BOLD:AAM6021
Cricotopus tremulus|BIOUG15949-H05|Kate Perez|BIN Taxonomy Match|BOLD:AAM6021
Cricotopus tremulus|BIOUG15905-G01|Kate Perez|BIN Taxonomy Match|BOLD:AAM6021
Cricotopus tremulus|BIOUG15905-E08|Kate Perez|BIN Taxonomy Match|BOLD:AAM6021
Cricotopus tremulus|BIOUG15809-A06|Kate Perez|BIN Taxonomy Match|BOLD:AAM6021
Cricotopus tremulus|BIOUG15964-G10|Kate Perez|BIN Taxonomy Match|BOLD:AAM6021
Cricotopus tremulus|BIOUG15964-B10|Kate Perez|BIN Taxonomy Match|BOLD:AAM6021
Cricotopus tremulus|BIOUG15964-B09|Kate Perez|BIN Taxonomy Match|BOLD:AAM6021
Cricotopus tremulus|BIOUG16552-D07|Kate Perez|BIN Taxonomy Match|BOLD:AAM6021
Cricotopus tremulus|BIOUG15967-D03|Kate Perez|BIN Taxonomy Match|BOLD:AAM6021
Cricotopus tremulus|BIOUG15799-E02|Kate Perez|BIN Taxonomy Match|BOLD:AAM6021
Cricotopus tremulus|BIOUG15949-G04|Kate Perez|BIN Taxonomy Match|BOLD:AAM6021
Cricotopus tremulus|BIOUG15948-B07|Kate Perez|BIN Taxonomy Match|BOLD:AAM6021
Cricotopus tremulus|BIOUG15947-H07|Kate Perez|BIN Taxonomy Match|BOLD:AAM6021
Cricotopus tremulus|BIOUG15947-G08|Kate Perez|BIN Taxonomy Match|BOLD:AAM6021
Cricotopus tremulus|BIOUG15947-E08|Kate Perez|BIN Taxonomy Match|BOLD:AAM6021
Cricotopus tremulus|BIOUG15905-B03|Kate Perez|BIN Taxonomy Match|BOLD:AAM6021
Cricotopus tremulus|BIOUG15966-E11|Kate Perez|BIN Taxonomy Match|BOLD:AAM6021
Cricotopus sp. 28ES|Finnmark849|Elisabeth Stur||BOLD:ACM4902
Cricotopus sp. 28ES|Finnmark846|Elisabeth Stur||BOLD:ACM4902
Cricotopus vierriensis|ZSM-DIP-33133-A10|Caroline Chimeno|BIN Taxonomy Match (Jan 2022)|BOL...
Cricotopus vierriensis|ZMUO.025312|Lauri Paasivirta||BOLD:ACU8434
[illegible]

Cricotopus sp. 28ES|Finnmark646|Elisabeth Stur||BOLD:ACM4902
Cricotopus vierriensis|ZSM-DIP-33133-A10|Caroline Chimeno|BIN Taxonomy Match (Jan 2022)|BOL...
Cricotopus vierriensis|ZMUO.025312|Lauri Paasivirta||BOLD:ACU8434
Cricotopus vierriensis|ZMUO.025311|Lauri Paasivirta||BOLD:ACU8434
Cricotopus vierriensis|ZSM-DIP-33147-C11|Caroline Chimeno|BIN Taxonomy Match (Jan 2022)|BOL...
Cricotopus vierriensis|ZSM-DIP-33147-E12|Caroline Chimeno|BIN Taxonomy Match (Jan 2022)|BOL...
Cricotopus vierriensis|ZSM-DIP-33128-B04|Caroline Chimeno|BIN Taxonomy Match (Jan 2022)|BOL...
Cricotopus vierriensis|ZSM-DIP-33146-F03|Caroline Chimeno|BIN Taxonomy Match (Jan 2022)|BOL...
Cricotopus vierriensis|ZSM-DIP-33140-H04|Caroline Chimeno|BIN Taxonomy Match (Jan 2022)|BOL...
Cricotopus vierriensis|BIOUG16894-D07|Kate Perez|BIN Taxonomy Match (Jul 2019)|BOLD:ACU8434
Cricotopus|BIOUG16706-F04|Kate Perez|BOLD ID Engine Manual|BOLD:ACQ9937
Cricotopus|BIOUG16996-G01|Kate Perez|BOLD ID Engine Manual|BOLD:ACQ9937
Cricotopus|BIOUG16997-B09|Kate Perez|BOLD ID Engine Manual|BOLD:ACQ9937
Cricotopus|BIOUG16900-G01|Kate Perez|BOLD ID Engine Manual|BOLD:ACQ9937
Cricotopus|BIOUG17094-C07|Kate Perez|BOLD ID Engine Manual|BOLD:ACQ9937
Cricotopus|BIOUG16614-D09|Kate Perez|BOLD ID Engine Manual|BOLD:ACQ9937
Cricotopus bicinctus|BIOUG17225-G07|Kate Perez|BIN Taxonomy Match|BOLD:AAT9677
Cricotopus bicinctus|ZMUO.024687|Lauri Paasivirta||BOLD:AAT9677
Cricotopus bicinctus|ZMUO.024686|Lauri Paasivirta||BOLD:AAT9677
Cricotopus bicinctus|NHRS-BYWS000000260|Yngve Brodin|morphology|BOLD:AAT9677
Cricotopus bicinctus|NHRS-BYWS000000133|Yngve Brodin|morphology|BOLD:AAT9677
Cricotopus bicinctus|BIOUG16597-H04|Kate Perez|BIN Taxonomy Match|BOLD:AAT9677
Cricotopus bicinctus|BIOUG15949-F09|Kate Perez|BIN Taxonomy Match|BOLD:AAT9677
Cricotopus bicinctus|BIOUG16198-E12|Kate Perez|BIN Taxonomy Match|BOLD:AAT9677
Cricotopus bicinctus|BIOUG16477-C11|Kate Perez|BIN Taxonomy Match|BOLD:AAT9677
Cricotopus bicinctus|BIOUG16479-D10|Kate Perez|BIN Taxonomy Match|BOLD:AAT9677
Cricotopus bicinctus|BIOUG16434-B06|Kate Perez|BIN Taxonomy Match|BOLD:AAT9677
Cricotopus bicinctus|BIOUG16435-A12|Kate Perez|BIN Taxonomy Match|BOLD:AAT9677
Cricotopus bicinctus|BIOUG16477-F06|Kate Perez|BIN Taxonomy Match|BOLD:AAT9677
Cricotopus bicinctus|BIOUG16551-F08|Kate Perez|BIN Taxonomy Match|BOLD:AAT9677
Cricotopus bicinctus|BIOUG15964-C10|Kate Perez|BIN Taxonomy Match|BOLD:AAT9677
Cricotopus bicinctus|BIOUG16552-C06|Kate Perez|BIN Taxonomy Match|BOLD:AAT9677
Cricotopus bicinctus|BIOUG15953-F08|Kate Perez|BIN Taxonomy Match|BOLD:AAT9677
Cricotopus bicinctus|BIOUG15949-A08|Kate Perez|BIN Taxonomy Match|BOLD:AAT9677
Cricotopus bicinctus|BIOUG15947-G01|Kate Perez|BIN Taxonomy Match|BOLD:AAT9677
Cricotopus bicinctus|NHRS-BYWS000000225|Yngve Brodin|morphology|BOLD:AAT9677
Cricotopus bicinctus|NHRS-BYWS000001796|Yngve Brodin||BOLD:AAT9677
Cricotopus bicinctus|NHRS-BYWS000000171|Yngve Brodin|morphology|BOLD:AAT9677
Cricotopus bicinctus|NHRS-BYWS000000902|Yngve Brodin|morphology|BOLD:AAT9677
Cricotopus bicinctus|EJV-20110103|Lauri Paasivirta||BOLD:AAT9677
Cricotopus bicinctus|EJV-20110102|Lauri Paasivirta||BOLD:AAT9677
Cricotopus bicinctus|NHRS-BYWS000001841|Yngve Brodin||BOLD:AAT9677
Cricotopus bicinctus|NHRS-BYWS000001771|Yngve Brodin||BOLD:AAT9677
Cricotopus bicinctus|NHRS-BYWS000001770|Yngve Brodin||BOLD:AAT9677
Cricotopus bicinctus|NHRS-BYWS000001748|Yngve Brodin||BOLD:AAT9677
Cricotopus bicinctus|NHRS-BYWS000001747|Yngve Brodin||BOLD:AAT9677
Cricotopus bicinctus|Finnmark262|Elisabeth Stur||BOLD:AAT9677
Cricotopus bicinctus|BIOUG55120-C05|Stefan Schmidt|BIN Taxonomy Match (Sep 2020)|BOLD:AA...
Chironomidae|ZSM-DIP-33136-G02|Caroline Chimeno|BIN Taxonomy Match (Jan 2022)|BOLD:AAI6018
Chironomidae|ZSM-DIP-33131-E12|Caroline Chimeno|BIN Taxonomy Match (Jan 2022)|BOLD:AAI6018
Cricotopus bicinctus|BIOUG36761-C05|Kate Perez|BIN Taxonomy Match (Jul 2018)|BOLD:AAI6018
Cricotopus bicinctus|BIOUG36934-G12|Kate Perez|BIN Taxonomy Match (Jul 2018)|BOLD:AAI6018
Cricotopus bicinctus|ZSM-DIP-33135-A07|Caroline Chimeno|BIN Taxonomy Match (Jan 2022)|BOLD...
Cricotopus|ES397|Elisabeth Stur|morphology|BOLD:AAI6018
Cricotopus bicinctus|ZSM-DIP-33137-G07|Caroline Chimeno|BIN Taxonomy Match (Jan 2022)|BOLD...
Cricotopus bicinctus|Finnmark838|Elisabeth Stur||BOLD:AAI6018
Cricotopus bicinctus|BIOUG17303-D03|Kate Perez|BIN Taxonomy Match|BOLD:AAI6018
Cricotopus bicinctus|ZSM-DIP-33135-A06|Caroline Chimeno|BIN Taxonomy Match (Jan 2022)|BOLD...
Cricotopus bicinctus|BIOUG36766-D08|Kate Perez|BIN Taxonomy Match (Jul 2018)|BOLD:AAI6018
Cricotopus bicinctus|BIOUG16627-A11|Kate Perez|BIN Taxonomy Match|BOLD:AAI6018
Cricotopus bicinctus|BIOUG16712-C07|Kate Perez|BIN Taxonomy Match|BOLD:AAI6018
Chironomidae|ZSM-DIP-33138-C05|Caroline Chimeno|BIN Taxonomy Match (Jan 2022)|BOLD:AAI6018
Chironomidae|ZSM-DIP-33131-E03|Caroline Chimeno|BIN Taxonomy Match (Jan 2022)|BOLD:AAI6018
Cricotopus bicinctus|BIOUG16261-D12|Kate Perez|BIN Taxonomy Match|BOLD:AAI6018
Cricotopus bicinctus|ZSM-DIP-33147-E04|Caroline Chimeno|BIN Taxonomy Match (Jan 2022)|BOLD...
Cricotopus bicinctus|TRD-CH173|Elisabeth Stur||BOLD:AAI6018
Cricotopus bicinctus|BIOUG16631-G08|Kate Perez|BIN Taxonomy Match|BOLD:AAI6018
Cricotopus bicinctus|BIOUG55119-B08|Stefan Schmidt|BIN Taxonomy Match (Sep 2020)|BOLD:AA...
Cricotopus bicinctus|ZSM-DIP-33149-H07|Caroline Chimeno|BIN Taxonomy Match (Jan 2022)|BOLD...
Cricotopus bicinctus|BIOUG16712-A07|Kate Perez|BIN Taxonomy Match|BOLD:AAI6018
Cricotopus bicinctus|BIOUG16479-G10|Kate Perez|BIN Taxonomy Match|BOLD:AAI6018
Cricotopus bicinctus|BIOUG55126-D11|Stefan Schmidt|BIN Taxonomy Match (Sep 2020)|BOLD:AA...
Cricotopus bicinctus|ZSM-DIP-33147-C10|Caroline Chimeno|BIN Taxonomy Match (Jan 2022)|BOLD...
Cricotopus bicinctus|NHRS-BYWS000000162|Yngve Brodin|morphology|BOLD:AAI6018
Cricotopus bicinctus|BIOUG36766-D06|Kate Perez|BIN Taxonomy Match (Jul 2018)|BOLD:AAI6018
Cricotopus bicinctus|BIOUG16711-G12|Kate Perez|BIN Taxonomy Match|BOLD:AAI6018
Cricotopus bicinctus|BIOUG55116-A02|Stefan Schmidt|BIN Taxonomy Match (Sep 2020)|BOLD:AA...
Cricotopus bicinctus|BIOUG16632-E08|Kate Perez|BIN Taxonomy Match|BOLD:AAI6018
Cricotopus bicinctus|CH-OSF98|Elisabeth Stur||BOLD:AAI6018
Cricotopus bicinctus|BIOUG55113-F08|Stefan Schmidt|BIN Taxonomy Match (Sep 2020)|BOLD:AA...
Cricotopus bicinctus|BIOUG42739-D01|Kate Perez|BIN Taxonomy Match below Phylum (Jun 2019)|B...
Cricotopus bicinctus|ZSM-DIP-33142-C03|Caroline Chimeno|BIN Taxonomy Match (Jan 2022)|BOLD...
Cricotopus bicinctus|24529-A04|Kate Perez|BIN Taxonomy Match|BOLD:AAI6018
Cricotopus bicinctus|BIOUG16525-H11|Kate Perez|BIN Taxonomy Match|BOLD:AAI6018
Chironomidae|ZSM-DIP-33138-C04|Caroline Chimeno|BIN Taxonomy Match (Jan 2022)|BOLD:AAI6018
Cricotopus bicinctus|BIOUG16358-B06|Kate Perez|BIN Taxonomy Match|BOLD:AAI6018
Cricotopus bicinctus|Finnmark234|Elisabeth Stur||BOLD:AEE5673
Cricotopus bicinctus|NHRS-BYWS000000529|Yngve Brodin|morphology|BOLD:AEE5673
Cricotopus bicinctus|Finnmark271|Elisabeth Stur||BOLD:AAP5931
Cricotopus festivellus|NHRS-BYWS000001075|Yngve Brodin|morphology|BOLD:AAV1707
Cricotopus festivellus|ZMUO.024646|Lauri Paasivirta||BOLD:AAV1707
Cricotopus festivellus|EJV-20110089|Lauri Paasivirta||BOLD:AAV1707
Cricotopus festivellus|Finnmark705|Torbjorn Ekrem||BOLD:AAV1707
Cricotopus festivellus|EJV-20110091|Lauri Paasivirta||BOLD:AAV1707
Cricotopus festivellus|NHRS-BYWS000001051|Yngve Brodin|morphology|BOLD:AAV1707
Cricotopus festivellus|EJV-20110093|Lauri Paasivirta||BOLD:AAV1707
Cricotopus festivellus|EJV-20110090|Lauri Paasivirta||BOLD:AAV1707
Cricotopus festivellus|NO63|Torbjorn Ekrem||BOLD:AAV1707
Cricotopus festivellus|NHRS-BYWS000000532|Yngve Brodin|morphology|BOLD:AAV1707
Cricotopus festivellus|ZMUO.024647|Lauri Paasivirta||BOLD:AAV1707
Cricotopus festivellus|EJV-20110092|Lauri Paasivirta||BOLD:AAV1707
Cricotopus festivellus|NO73|Elisabeth Stur||BOLD:AAV1707
Cricotopus festivellus|NO60|Torbjorn Ekrem||BOLD:AAV1707

Cricotopus festivellus|EJV-20110092|Lauri Paasivirta||BOLD:AAV1707
Cricotopus festivellus|NO73|Elisabeth Stur||BOLD:AAV1707
Cricotopus festivellus|NO60|Torbjorn Ekrem||BOLD:AAV1707
Cricotopus festivellus|NO56|Torbjorn Ekrem||BOLD:AAV1707
Cricotopus festivellus|NO54|Torbjorn Ekrem||BOLD:AAV1707
Cricotopus festivellus|NO53|Torbjorn Ekrem||BOLD:AAV1707
Cricotopus festivellus|Finnmark472|Torbjorn Ekrem||BOLD:AAV1707
Cricotopus|BC-ZSM-DIP-22488-E08|Dieter Doczkal||BOLD:ACU4162
Cricotopus|CCDB24039-A08|Sofia Wiedenbrug|BIN Taxonomy Match|BOLD:ACU4162
Cricotopus flavocinctus|ZMUO.025101|Lauri Paasivirta||BOLD:ADA7280
Cricotopus flavocinctus|ZMUO.025100|Lauri Paasivirta||BOLD:ADA7280
Cricotopus|CCDB24229-A08|Sofia Wiedenbrug|BIN Taxonomy Match|BOLD:ACU4584
Orthocladiinae|BIOUG16859-B07|Kate Perez|BIN Taxonomy Match|BOLD:AAW4679
Cricotopus rufiventris|EJV-20110176|Lauri Paasivirta||BOLD:AAW4679
Cricotopus rufiventris|EJV-20110174|Lauri Paasivirta||BOLD:AAW4679
Cricotopus rufiventris|EJV-20110175|Lauri Paasivirta||BOLD:AAW4679
Cricotopus rufiventris|NHRS-BYWS000000521|Yngve Brodin|morphology|BOLD:AAM5377
Cricotopus rufiventris|NHRS-BYWS000000149|Yngve Brodin|morphology|BOLD:AAM5377
Cricotopus rufiventris|NHRS-BYWS000000148|Yngve Brodin|morphology|BOLD:AAM5377
Cricotopus rufiventris|NHRS-BYWS000000183|Yngve Brodin|morphology|BOLD:AAM5377
Cricotopus rufiventris|ZMUO.024698|Lauri Paasivirta||BOLD:AAM5377
Cricotopus rufiventris|ZMUO.024699|Lauri Paasivirta||BOLD:AAM5377
Cricotopus rufiventris|NHRS-BYWS000000333|Yngve Brodin|morphology|BOLD:AAM5377
Cricotopus rufiventris|NHRS-BYWS000000205|Yngve Brodin|morphology|BOLD:AAM5377
Cricotopus rufiventris|Finnmark47|Elisabeth Stur||BOLD:AAM5377
Cricotopus rufiventris|Finnmark15|Elisabeth Stur||BOLD:AAM5377
Cricotopus rufiventris|Finnmark60|Elisabeth Stur||BOLD:AAM5377
Cricotopus rufiventris|Finnmark24|Elisabeth Stur||BOLD:AAM5377
Cricotopus rufiventris|BIOUG15761-A09|Kate Perez|BIN Taxonomy Match|BOLD:AAM5377
Cricotopus rufiventris|BIOUG15593-D05|Kate Perez|BIN Taxonomy Match|BOLD:AAM5377
Cricotopus rufiventris|BIOUG16477-H07|Kate Perez|BIN Taxonomy Match|BOLD:AAM5377
Cricotopus rufiventris|BIOUG15795-B10|Kate Perez|BIN Taxonomy Match|BOLD:AAM5377
Cricotopus rufiventris|BIOUG15966-F03|Kate Perez|BIN Taxonomy Match|BOLD:AAM5377
Cricotopus rufiventris|CH-OSF37|Elisabeth Stur||BOLD:AAM5377
Cricotopus rufiventris|BIOUG15952-B10|Elisabeth Stur|morphology|BOLD:AAM5377
Cricotopus rufiventris|BIOUG15953-D03|Elisabeth Stur|morphology|BOLD:AAM5377
Cricotopus rufiventris|BIOUG16479-D11|Kate Perez|BIN Taxonomy Match|BOLD:AAM5377
Cricotopus rufiventris|BIOUG16479-H09|Kate Perez|BIN Taxonomy Match|BOLD:AAM5377
Cricotopus rufiventris|BIOUG16329-C08|Kate Perez|BIN Taxonomy Match|BOLD:AAM5377
Cricotopus rufiventris|BIOUG16328-H11|Kate Perez|BIN Taxonomy Match|BOLD:AAM5377
Cricotopus rufiventris|Finnmark851|Elisabeth Stur||BOLD:AAM5377
Cricotopus rufiventris|BIOUG42670-A03|Kate Perez|BIN Taxonomy Match below Phylum (Jun 2019)...
Cricotopus rufiventris|BIOUG42756-A04|Kate Perez|BIN Taxonomy Match below Phylum (Jun 2019)...
Cricotopus rufiventris|BIOUG42763-H04|Kate Perez|BIN Taxonomy Match below Phylum (Jun 2019)...
Cricotopus rufiventris|BIOUG42677-H04|Kate Perez|BIN Taxonomy Match below Phylum (Jun 2019)...
Cricotopus rufiventris|ZSM-DIP-33144-F03|Caroline Chimeno|BIN Taxonomy Match (Jan 2022)|BOL...
Cricotopus rufiventris|BIOUG17136-B11|Kate Perez|BIN Taxonomy Match|BOLD:AAM5377
Cricotopus rufiventris|BIOUG17114-B01|Kate Perez|BIN Taxonomy Match|BOLD:AAM5377
Cricotopus rufiventris|ZSM-DIP-33144-G05|Caroline Chimeno|BIN Taxonomy Match (Jan 2022)|BOL...
Cricotopus rufiventris|BIOUG16480-G12|Kate Perez|BIN Taxonomy Match|BOLD:AAM5377
Cricotopus rufiventris|BIOUG16297-C02|Kate Perez|BIN Taxonomy Match|BOLD:AAM5377
Cricotopus rufiventris|BIOUG16435-C01|Kate Perez|BIN Taxonomy Match|BOLD:AAM5377
Cricotopus rufiventris|BIOUG08217-E11|Kate Perez|BIN Taxonomy Match|BOLD:AAM5377
Cricotopus rufiventris|BIOUG08254-H06|Kate Perez|BIN Taxonomy Match|BOLD:AAM5377
Cricotopus rufiventris|BIOUG16527-D12|Kate Perez|BIN Taxonomy Match|BOLD:AAM5377
Cricotopus rufiventris|BIOUG16529-B07|Kate Perez|BIN Taxonomy Match|BOLD:AAM5377
Cricotopus rufiventris|BIOUG15563-G04|Kate Perez|BIN Taxonomy Match|BOLD:AAM5377
Cricotopus rufiventris|BIOUG17094-F10|Kate Perez|BIN Taxonomy Match|BOLD:AAM5377
Cricotopus rufiventris|BIOUG17119-H09|Kate Perez|BIN Taxonomy Match|BOLD:AAM5377
Cricotopus rufiventris|BIOUG16996-C02|Kate Perez|BIN Taxonomy Match|BOLD:AAM5377
Cricotopus rufiventris|BIOUG16477-B02|Kate Perez|BIN Taxonomy Match|BOLD:AAM5377
Cricotopus rufiventris|BIOUG17119-B02|Kate Perez|BIN Taxonomy Match|BOLD:AAM5377
Cricotopus rufiventris|BIOUG16480-A03|Kate Perez|BIN Taxonomy Match|BOLD:AAM5377
Cricotopus rufiventris|BIOUG16261-C10|Kate Perez|BIN Taxonomy Match|BOLD:AAM5377
Cricotopus rufiventris|BIOUG16333-H02|Kate Perez|BIN Taxonomy Match|BOLD:AAM5377
Cricotopus rufiventris|BIOUG05269-A06|Kate Perez|BIN Taxonomy Match|BOLD:AAM5377
Cricotopus rufiventris|BIOUG16328-B11|Kate Perez|BIN Taxonomy Match|BOLD:AAM5377
Cricotopus rufiventris|BIOUG16330-F07|Kate Perez|BIN Taxonomy Match|BOLD:AAM5377
Cricotopus rufiventris|BIOUG16299-H02|Kate Perez|BIN Taxonomy Match|BOLD:AAM5377
Cricotopus rufiventris|BIOUG16551-E12|Kate Perez|BIN Taxonomy Match|BOLD:AAM5377
Cricotopus rufiventris|BIOUG16555-G09|Kate Perez|BIN Taxonomy Match|BOLD:AAM5377
Cricotopus rufiventris|BIOUG16258-F05|Kate Perez|BIN Taxonomy Match|BOLD:AAM5377
Cricotopus rufiventris|BIOUG16551-E07|Kate Perez|BIN Taxonomy Match|BOLD:AAM5377
Cricotopus rufiventris|BIOUG16477-C05|Kate Perez|BIN Taxonomy Match|BOLD:AAM5377
Cricotopus rufiventris|BIOUG16328-D10|Kate Perez|BIN Taxonomy Match|BOLD:AAM5377
Cricotopus rufiventris|BIOUG16434-A07|Kate Perez|BIN Taxonomy Match|BOLD:AAM5377
Cricotopus rufiventris|BIOUG16299-B07|Kate Perez|BIN Taxonomy Match|BOLD:AAM5377
Cricotopus rufiventris|BIOUG15719-G09|Kate Perez|BIN Taxonomy Match|BOLD:AAM5377
Cricotopus rufiventris|BIOUG16433-B10|Kate Perez|BIN Taxonomy Match|BOLD:AAM5377
Cricotopus rufiventris|BIOUG16431-H10|Kate Perez|BIN Taxonomy Match|BOLD:AAM5377
Cricotopus rufiventris|BIOUG16480-E02|Kate Perez|BIN Taxonomy Match|BOLD:AAM5377
Cricotopus rufiventris|BIOUG16297-H05|Kate Perez|BIN Taxonomy Match|BOLD:AAM5377
Cricotopus rufiventris|BIOUG16436-H01|Kate Perez|BIN Taxonomy Match|BOLD:AAM5377
Cricotopus rufiventris|BIOUG16299-E12|Kate Perez|BIN Taxonomy Match|BOLD:AAM5377
Cricotopus rufiventris|BIOUG16477-C09|Kate Perez|BIN Taxonomy Match|BOLD:AAM5377
Cricotopus rufiventris|BIOUG16330-F04|Kate Perez|BIN Taxonomy Match|BOLD:AAM5377
Cricotopus rufiventris|BIOUG16259-A11|Kate Perez|BIN Taxonomy Match|BOLD:AAM5377
Cricotopus rufiventris|BIOUG16332-H09|Kate Perez|BIN Taxonomy Match|BOLD:AAM5377
Cricotopus rufiventris|BIOUG16299-B05|Kate Perez|BIN Taxonomy Match|BOLD:AAM5377
Cricotopus rufiventris|BIOUG15593-B08|Kate Perez|BIN Taxonomy Match|BOLD:AAM5377
Cricotopus rufiventris|BIOUG16333-G08|Kate Perez|BIN Taxonomy Match|BOLD:AAM5377
Cricotopus rufiventris|BIOUG16333-A07|Kate Perez|BIN Taxonomy Match|BOLD:AAM5377
Cricotopus rufiventris|BIOUG16257-H09|Kate Perez|BIN Taxonomy Match|BOLD:AAM5377
Cricotopus rufiventris|BIOUG16297-A07|Kate Perez|BIN Taxonomy Match|BOLD:AAM5377
Cricotopus rufiventris|BIOUG16555-H04|Kate Perez|BIN Taxonomy Match|BOLD:AAM5377
Cricotopus rufiventris|BIOUG15764-G09|Kate Perez|BIN Taxonomy Match|BOLD:AAM5377
Cricotopus rufiventris|BIOUG16433-E08|Kate Perez|BIN Taxonomy Match|BOLD:AAM5377
Cricotopus rufiventris|BIOUG16329-C03|Kate Perez|BIN Taxonomy Match|BOLD:AAM5377
Cricotopus rufiventris|BIOUG15762-H06|Kate Perez|BIN Taxonomy Match|BOLD:AAM5377
Cricotopus rufiventris|BIOUG16329-B08|Kate Perez|BIN Taxonomy Match|BOLD:AAM5377
Cricotopus rufiventris|BIOUG16329-F04|Kate Perez|BIN Taxonomy Match|BOLD:AAM5377
Cricotopus rufiventris|BIOUG16298-F12|Kate Perez|BIN Taxonomy Match|BOLD:AAM5377
Cricotopus rufiventris|BIOUG16298-B03|Kate Perez|BIN Taxonomy Match|BOLD:AAM5377

Cricotopus rufiventris|BIOUG16329-F04|Kate Perez|BIN Taxonomy Match|BOLD:AAM5377
Cricotopus rufiventris|BIOUG16298-F12|Kate Perez|BIN Taxonomy Match|BOLD:AAM5377
Cricotopus rufiventris|BIOUG16298-B03|Kate Perez|BIN Taxonomy Match|BOLD:AAM5377
Cricotopus rufiventris|BIOUG16256-F11|Kate Perez|BIN Taxonomy Match|BOLD:AAM5377
Cricotopus rufiventris|BIOUG16256-D03|Kate Perez|BIN Taxonomy Match|BOLD:AAM5377
Cricotopus rufiventris|BIOUG15649-F05|Kate Perez|BIN Taxonomy Match|BOLD:AAM5377
Cricotopus rufiventris|BIOUG16299-C09|Kate Perez|BIN Taxonomy Match|BOLD:AAM5377
Cricotopus rufiventris|BIOUG16478-A08|Kate Perez|BIN Taxonomy Match|BOLD:AAM5377
Cricotopus rufiventris|BIOUG16431-F12|Kate Perez|BIN Taxonomy Match|BOLD:AAM5377
Cricotopus rufiventris|TRD-CH169|Elisabeth Stur||BOLD:AAM5377
Cricotopus rufiventris|BIOUG16256-B11|Kate Perez|BIN Taxonomy Match|BOLD:AAM5377
Cricotopus rufiventris|BIOUG16331-B10|Kate Perez|BIN Taxonomy Match|BOLD:AAM5377
Cricotopus rufiventris|BIOUG16333-A04|Kate Perez|BIN Taxonomy Match|BOLD:AAM5377
Cricotopus rufiventris|BIOUG16333-B03|Kate Perez|BIN Taxonomy Match|BOLD:AAM5377
Cricotopus rufiventris|BIOUG16552-C12|Kate Perez|BIN Taxonomy Match|BOLD:AAM5377
Cricotopus rufiventris|BIOUG16552-E03|Kate Perez|BIN Taxonomy Match|BOLD:AAM5377
Cricotopus rufiventris|BIOUG16551-D06|Kate Perez|BIN Taxonomy Match|BOLD:AAM5377
Cricotopus rufiventris|BIOUG16257-A04|Kate Perez|BIN Taxonomy Match|BOLD:AAM5377
Cricotopus rufiventris|BIOUG16257-E11|Kate Perez|BIN Taxonomy Match|BOLD:AAM5377
Cricotopus rufiventris|BIOUG16258-F01|Kate Perez|BIN Taxonomy Match|BOLD:AAM5377
Cricotopus rufiventris|BIOUG16299-A06|Kate Perez|BIN Taxonomy Match|BOLD:AAM5377
Cricotopus rufiventris|BIOUG16331-C02|Kate Perez|BIN Taxonomy Match|BOLD:AAM5377
Cricotopus rufiventris|BIOUG16331-E10|Kate Perez|BIN Taxonomy Match|BOLD:AAM5377
Cricotopus rufiventris|BIOUG16258-D04|Kate Perez|BIN Taxonomy Match|BOLD:AAM5377
Cricotopus rufiventris|BIOUG16431-A10|Kate Perez|BIN Taxonomy Match|BOLD:AAM5377
Cricotopus rufiventris|BIOUG16329-C10|Kate Perez|BIN Taxonomy Match|BOLD:AAM5377
Cricotopus rufiventris|BIOUG15592-E06|Kate Perez|BIN Taxonomy Match|BOLD:AAM5377
Cricotopus rufiventris|BIOUG16436-G01|Kate Perez|BIN Taxonomy Match|BOLD:AAM5377
Cricotopus rufiventris|BIOUG16552-F06|Kate Perez|BIN Taxonomy Match|BOLD:AAM5377
Cricotopus rufiventris|BIOUG16259-D11|Kate Perez|BIN Taxonomy Match|BOLD:AAM5377
Cricotopus rufiventris|BIOUG16329-C06|Kate Perez|BIN Taxonomy Match|BOLD:AAM5377
Cricotopus rufiventris|BIOUG16431-A05|Kate Perez|BIN Taxonomy Match|BOLD:AAM5377
Cricotopus rufiventris|BIOUG16257-G02|Kate Perez|BIN Taxonomy Match|BOLD:AAM5377
Cricotopus rufiventris|BIOUG16299-E01|Kate Perez|BIN Taxonomy Match|BOLD:AAM5377
Cricotopus rufiventris|BIOUG16258-G08|Kate Perez|BIN Taxonomy Match|BOLD:AAM5377
Cricotopus rufiventris|BIOUG16711-G07|Kate Perez|BIN Taxonomy Match|BOLD:AAM5377
Cricotopus rufiventris|BIOUG16480-D05|Kate Perez|BIN Taxonomy Match|BOLD:AAM5377
Cricotopus rufiventris|BIOUG16257-G08|Kate Perez|BIN Taxonomy Match|BOLD:AAM5377
Cricotopus rufiventris|BIOUG16260-D04|Kate Perez|BIN Taxonomy Match|BOLD:AAM5377
Cricotopus rufiventris|BIOUG16596-C09|Kate Perez|BIN Taxonomy Match|BOLD:AAM5377
Cricotopus rufiventris|BIOUG15596-C07|Kate Perez|BIN Taxonomy Match|BOLD:AAM5377
Cricotopus rufiventris|BIOUG16330-G09|Kate Perez|BIN Taxonomy Match|BOLD:AAM5377
Cricotopus rufiventris|BIOUG16260-B10|Kate Perez|BIN Taxonomy Match|BOLD:AAM5377
Cricotopus rufiventris|BIOUG16297-D07|Kate Perez|BIN Taxonomy Match|BOLD:AAM5377
Cricotopus rufiventris|BIOUG16296-D03|Kate Perez|BIN Taxonomy Match|BOLD:AAM5377
Cricotopus rufiventris|BIOUG16332-B09|Kate Perez|BIN Taxonomy Match|BOLD:AAM5377
Cricotopus rufiventris|BIOUG16433-C11|Kate Perez|BIN Taxonomy Match|BOLD:AAM5377
Cricotopus rufiventris|BIOUG16477-A10|Kate Perez|BIN Taxonomy Match|BOLD:AAM5377
Cricotopus rufiventris|BIOUG16432-D05|Kate Perez|BIN Taxonomy Match|BOLD:AAM5377
Cricotopus rufiventris|BIOUG15718-E06|Kate Perez|BIN Taxonomy Match|BOLD:AAM5377
Cricotopus rufiventris|BIOUG16329-B07|Kate Perez|BIN Taxonomy Match|BOLD:AAM5377
Cricotopus rufiventris|BIOUG16256-B06|Kate Perez|BIN Taxonomy Match|BOLD:AAM5377
Cricotopus rufiventris|BIOUG16257-G09|Kate Perez|BIN Taxonomy Match|BOLD:AAM5377
Cricotopus rufiventris|BIOUG16298-D06|Kate Perez|BIN Taxonomy Match|BOLD:AAM5377
Cricotopus rufiventris|BIOUG16331-B11|Kate Perez|BIN Taxonomy Match|BOLD:AAM5377
Cricotopus rufiventris|BIOUG16297-C05|Kate Perez|BIN Taxonomy Match|BOLD:AAM5377
Cricotopus rufiventris|BIOUG15718-H11|Kate Perez|BIN Taxonomy Match|BOLD:AAM5377
Cricotopus rufiventris|BIOUG16261-D02|Kate Perez|BIN Taxonomy Match|BOLD:AAM5377
Cricotopus rufiventris|BIOUG16257-H03|Kate Perez|BIN Taxonomy Match|BOLD:AAM5377
Cricotopus rufiventris|BIOUG16256-A05|Kate Perez|BIN Taxonomy Match|BOLD:AAM5377
Cricotopus rufiventris|BIOUG16257-C09|Kate Perez|BIN Taxonomy Match|BOLD:AAM5377
Cricotopus rufiventris|BIOUG16258-A06|Kate Perez|BIN Taxonomy Match|BOLD:AAM5377
Cricotopus rufiventris|BIOUG15648-D02|Kate Perez|BIN Taxonomy Match|BOLD:AAM5377
Cricotopus rufiventris|BIOUG16261-A03|Kate Perez|BIN Taxonomy Match|BOLD:AAM5377
Cricotopus rufiventris|BIOUG16259-B11|Kate Perez|BIN Taxonomy Match|BOLD:AAM5377
Cricotopus rufiventris|BIOUG16298-B11|Kate Perez|BIN Taxonomy Match|BOLD:AAM5377
Cricotopus rufiventris|BIOUG16298-C09|Kate Perez|BIN Taxonomy Match|BOLD:AAM5377
Cricotopus rufiventris|BIOUG16298-E11|Kate Perez|BIN Taxonomy Match|BOLD:AAM5377
Cricotopus rufiventris|BIOUG16299-F05|Kate Perez|BIN Taxonomy Match|BOLD:AAM5377
Cricotopus rufiventris|BIOUG16328-C07|Kate Perez|BIN Taxonomy Match|BOLD:AAM5377
Cricotopus rufiventris|BIOUG16480-A01|Kate Perez|BIN Taxonomy Match|BOLD:AAM5377
Cricotopus rufiventris|BIOUG16329-G09|Kate Perez|BIN Taxonomy Match|BOLD:AAM5377
Cricotopus rufiventris|BIOUG16433-F05|Kate Perez|BIN Taxonomy Match|BOLD:AAM5377
Cricotopus rufiventris|BIOUG16298-D04|Kate Perez|BIN Taxonomy Match|BOLD:AAM5377
Cricotopus rufiventris|BIOUG16434-B05|Kate Perez|BIN Taxonomy Match|BOLD:AAM5377
Cricotopus rufiventris|BIOUG16430-C12|Kate Perez|BIN Taxonomy Match|BOLD:AAM5377
Cricotopus rufiventris|BIOUG16434-F10|Kate Perez|BIN Taxonomy Match|BOLD:AAM5377
Cricotopus rufiventris|BIOUG16396-A09|Kate Perez|BIN Taxonomy Match|BOLD:AAM5377
Cricotopus rufiventris|BIOUG16426-G02|Kate Perez|BIN Taxonomy Match|BOLD:AAM5377
Cricotopus rufiventris|BIOUG16260-E07|Kate Perez|BIN Taxonomy Match|BOLD:AAM5377
Cricotopus rufiventris|BIOUG16260-B07|Kate Perez|BIN Taxonomy Match|BOLD:AAM5377
Cricotopus rufiventris|BIOUG16260-A06|Kate Perez|BIN Taxonomy Match|BOLD:AAM5377
Cricotopus rufiventris|BIOUG16552-B02|Kate Perez|BIN Taxonomy Match|BOLD:AAM5377
Cricotopus rufiventris|BIOUG15809-G12|Kate Perez|BIN Taxonomy Match|BOLD:AAM5377
Cricotopus rufiventris|BIOUG15808-B07|Kate Perez|BIN Taxonomy Match|BOLD:AAM5377
Cricotopus rufiventris|BIOUG16333-H08|Kate Perez|BIN Taxonomy Match|BOLD:AAM5377
Cricotopus rufiventris|BIOUG16333-D01|Kate Perez|BIN Taxonomy Match|BOLD:AAM5377
Cricotopus rufiventris|BIOUG15674-D01|Kate Perez|BIN Taxonomy Match|BOLD:AAM5377
Cricotopus rufiventris|BIOUG16260-B05|Kate Perez|BIN Taxonomy Match|BOLD:AAM5377
Cricotopus rufiventris|BIOUG16430-E01|Kate Perez|BIN Taxonomy Match|BOLD:AAM5377
Cricotopus rufiventris|BIOUG15651-D12|Kate Perez|BIN Taxonomy Match|BOLD:AAM5377
Cricotopus rufiventris|BIOUG16296-B01|Kate Perez|BIN Taxonomy Match|BOLD:AAM5377
Cricotopus rufiventris|BIOUG16261-G04|Kate Perez|BIN Taxonomy Match|BOLD:AAM5377
Cricotopus rufiventris|BIOUG16261-F08|Kate Perez|BIN Taxonomy Match|BOLD:AAM5377
Cricotopus rufiventris|BIOUG16260-F09|Kate Perez|BIN Taxonomy Match|BOLD:AAM5377
Cricotopus rufiventris|BIOUG16333-B11|Kate Perez|BIN Taxonomy Match|BOLD:AAM5377
Cricotopus rufiventris|BIOUG16333-B10|Kate Perez|BIN Taxonomy Match|BOLD:AAM5377
Cricotopus rufiventris|BIOUG16331-F10|Kate Perez|BIN Taxonomy Match|BOLD:AAM5377
Cricotopus rufiventris|BIOUG16331-F08|Kate Perez|BIN Taxonomy Match|BOLD:AAM5377
Cricotopus rufiventris|BIOUG15761-F09|Kate Perez|BIN Taxonomy Match|BOLD:AAM5377
Cricotopus rufiventris|BIOUG15761-D01|Kate Perez|BIN Taxonomy Match|BOLD:AAM5377
Cricotopus rufiventris|BIOUG15718-D09|Kate Perez|BIN Taxonomy Match|BOLD:AAM5377
Cricotopus rufiventris|BIOUG15764-G04|Kate Perez|BIN Taxonomy Match|BOLD:AAM5377

[illegible]
Cricotopus rufiventris|BIOUG15761-D01|Kate Perez|BIN Taxonomy Match|BOLD:AAM5377
Cricotopus rufiventris|BIOUG15718-D09|Kate Perez|BIN Taxonomy Match|BOLD:AAM5377
Cricotopus rufiventris|BIOUG15764-G04|Kate Perez|BIN Taxonomy Match|BOLD:AAM5377
Cricotopus rufiventris|BIOUG16430-D08|Kate Perez|BIN Taxonomy Match|BOLD:AAM5377
Cricotopus rufiventris|BIOUG16430-H02|Kate Perez|BIN Taxonomy Match|BOLD:AAM5377
Cricotopus rufiventris|BIOUG16430-H06|Kate Perez|BIN Taxonomy Match|BOLD:AAM5377
Cricotopus rufiventris|BIOUG16477-E07|Kate Perez|BIN Taxonomy Match|BOLD:AAM5377
Cricotopus rufiventris|BIOUG16622-G12|Kate Perez|BIN Taxonomy Match|BOLD:AAM5377
Cricotopus rufiventris|BIOUG15599-D12|Kate Perez|BIN Taxonomy Match|BOLD:AAM5377
Cricotopus rufiventris|BIOUG16328-D01|Kate Perez|BIN Taxonomy Match|BOLD:AAM5377
Cricotopus rufiventris|BIOUG16435-E09|Kate Perez|BIN Taxonomy Match|BOLD:AAM5377
Cricotopus rufiventris|BIOUG16479-E09|Kate Perez|BIN Taxonomy Match|BOLD:AAM5377
Cricotopus rufiventris|BIOUG16552-B05|Kate Perez|BIN Taxonomy Match|BOLD:AAM5377
Cricotopus rufiventris|BIOUG16257-A02|Kate Perez|BIN Taxonomy Match|BOLD:AAM5377
Cricotopus rufiventris|BIOUG16328-E06|Kate Perez|BIN Taxonomy Match|BOLD:AAM5377
Cricotopus rufiventris|BIOUG16259-C10|Kate Perez|BIN Taxonomy Match|BOLD:AAM5377
Cricotopus rufiventris|BIOUG16258-E08|Kate Perez|BIN Taxonomy Match|BOLD:AAM5377
Cricotopus rufiventris|BIOUG16261-C02|Kate Perez|BIN Taxonomy Match|BOLD:AAM5377
Cricotopus rufiventris|BIOUG16261-B02|Kate Perez|BIN Taxonomy Match|BOLD:AAM5377
Cricotopus rufiventris|BIOUG16258-D02|Kate Perez|BIN Taxonomy Match|BOLD:AAM5377
Cricotopus rufiventris|BIOUG16257-H05|Kate Perez|BIN Taxonomy Match|BOLD:AAM5377
Cricotopus rufiventris|BIOUG16296-A04|Kate Perez|BIN Taxonomy Match|BOLD:AAM5377
Cricotopus rufiventris|BIOUG16260-F06|Kate Perez|BIN Taxonomy Match|BOLD:AAM5377
Cricotopus rufiventris|BIOUG16298-F11|Kate Perez|BIN Taxonomy Match|BOLD:AAM5377
Cricotopus rufiventris|BIOUG16296-G04|Kate Perez|BIN Taxonomy Match|BOLD:AAM5377
Cricotopus rufiventris|BIOUG16298-G11|Kate Perez|BIN Taxonomy Match|BOLD:AAM5377
Cricotopus rufiventris|BIOUG16258-G04|Kate Perez|BIN Taxonomy Match|BOLD:AAM5377
Cricotopus rufiventris|BIOUG16434-H08|Kate Perez|BIN Taxonomy Match|BOLD:AAM5377
Cricotopus rufiventris|BIOUG16431-G02|Kate Perez|BIN Taxonomy Match|BOLD:AAM5377
Cricotopus rufiventris|BIOUG16432-D09|Kate Perez|BIN Taxonomy Match|BOLD:AAM5377
Cricotopus rufiventris|BIOUG16436-G12|Kate Perez|BIN Taxonomy Match|BOLD:AAM5377
Cricotopus rufiventris|BIOUG16396-B08|Kate Perez|BIN Taxonomy Match|BOLD:AAM5377
Cricotopus rufiventris|BIOUG16297-C09|Kate Perez|BIN Taxonomy Match|BOLD:AAM5377
Cricotopus rufiventris|BIOUG16297-F09|Kate Perez|BIN Taxonomy Match|BOLD:AAM5377
Cricotopus rufiventris|BIOUG16299-B01|Kate Perez|BIN Taxonomy Match|BOLD:AAM5377
Cricotopus rufiventris|BIOUG16330-G06|Kate Perez|BIN Taxonomy Match|BOLD:AAM5377
Cricotopus rufiventris|BIOUG16625-B12|Kate Perez|BIN Taxonomy Match|BOLD:AAM5377
Cricotopus rufiventris|BIOUG16480-H06|Kate Perez|BIN Taxonomy Match|BOLD:AAM5377
Cricotopus rufiventris|BIOUG16433-B07|Kate Perez|BIN Taxonomy Match|BOLD:AAM5377
Cricotopus rufiventris|BIOUG16260-E05|Kate Perez|BIN Taxonomy Match|BOLD:AAM5377
Cricotopus rufiventris|BIOUG16299-D10|Kate Perez|BIN Taxonomy Match|BOLD:AAM5377
Cricotopus rufiventris|BIOUG16431-B04|Kate Perez|BIN Taxonomy Match|BOLD:AAM5377
Cricotopus rufiventris|BIOUG16296-G05|Kate Perez|BIN Taxonomy Match|BOLD:AAM5377
Cricotopus rufiventris|BIOUG16477-B08|Kate Perez|BIN Taxonomy Match|BOLD:AAM5377
Cricotopus rufiventris|BIOUG16298-H06|Kate Perez|BIN Taxonomy Match|BOLD:AAM5377
Cricotopus rufiventris|BIOUG16432-H06|Kate Perez|BIN Taxonomy Match|BOLD:AAM5377
Cricotopus rufiventris|BIOUG16257-E12|Kate Perez|BIN Taxonomy Match|BOLD:AAM5377
Cricotopus rufiventris|BIOUG16430-B01|Kate Perez|BIN Taxonomy Match|BOLD:AAM5377
Cricotopus rufiventris|BIOUG16258-H05|Kate Perez|BIN Taxonomy Match|BOLD:AAM5377
Cricotopus rufiventris|BIOUG16258-G10|Kate Perez|BIN Taxonomy Match|BOLD:AAM5377
Cricotopus rufiventris|BIOUG16258-D12|Kate Perez|BIN Taxonomy Match|BOLD:AAM5377
Cricotopus rufiventris|BIOUG16258-A10|Kate Perez|BIN Taxonomy Match|BOLD:AAM5377
Cricotopus rufiventris|BIOUG16261-B01|Kate Perez|BIN Taxonomy Match|BOLD:AAM5377
Cricotopus rufiventris|BIOUG16256-A12|Kate Perez|BIN Taxonomy Match|BOLD:AAM5377
Cricotopus rufiventris|BIOUG16434-A10|Kate Perez|BIN Taxonomy Match|BOLD:AAM5377
Cricotopus rufiventris|BIOUG16435-C03|Kate Perez|BIN Taxonomy Match|BOLD:AAM5377
Cricotopus rufiventris|BIOUG16478-H10|Kate Perez|BIN Taxonomy Match|BOLD:AAM5377
Cricotopus rufiventris|BIOUG16430-E02|Kate Perez|BIN Taxonomy Match|BOLD:AAM5377
Cricotopus rufiventris|BIOUG16477-F01|Kate Perez|BIN Taxonomy Match|BOLD:AAM5377
Cricotopus rufiventris|BIOUG16259-D09|Kate Perez|BIN Taxonomy Match|BOLD:AAM5377
Cricotopus rufiventris|BIOUG16434-A03|Kate Perez|BIN Taxonomy Match|BOLD:AAM5377
Cricotopus rufiventris|BIOUG16435-F06|Kate Perez|BIN Taxonomy Match|BOLD:AAM5377
Cricotopus rufiventris|BIOUG16430-C02|Kate Perez|BIN Taxonomy Match|BOLD:AAM5377
Cricotopus rufiventris|BIOUG16477-D05|Kate Perez|BIN Taxonomy Match|BOLD:AAM5377
Cricotopus rufiventris|BIOUG16477-G06|Kate Perez|BIN Taxonomy Match|BOLD:AAM5377
Cricotopus rufiventris|BIOUG16328-F03|Kate Perez|BIN Taxonomy Match|BOLD:AAM5377
Cricotopus rufiventris|BIOUG16298-E03|Kate Perez|BIN Taxonomy Match|BOLD:AAM5377
Cricotopus rufiventris|BIOUG16259-G01|Kate Perez|BIN Taxonomy Match|BOLD:AAM5377
Cricotopus rufiventris|BIOUG16480-C04|Kate Perez|BIN Taxonomy Match|BOLD:AAM5377
Cricotopus rufiventris|BIOUG16297-G06|Kate Perez|BIN Taxonomy Match|BOLD:AAM5377
Cricotopus rufiventris|BIOUG16436-C07|Kate Perez|BIN Taxonomy Match|BOLD:AAM5377
Cricotopus rufiventris|BIOUG16426-H03|Kate Perez|BIN Taxonomy Match|BOLD:AAM5377
Cricotopus rufiventris|BIOUG16433-B12|Kate Perez|BIN Taxonomy Match|BOLD:AAM5377
Cricotopus rufiventris|BIOUG16478-F06|Kate Perez|BIN Taxonomy Match|BOLD:AAM5377
Cricotopus rufiventris|BIOUG16261-A09|Kate Perez|BIN Taxonomy Match|BOLD:AAM5377
Cricotopus rufiventris|BIOUG16480-E03|Kate Perez|BIN Taxonomy Match|BOLD:AAM5377
Cricotopus rufiventris|BIOUG16328-C11|Kate Perez|BIN Taxonomy Match|BOLD:AAM5377
Cricotopus rufiventris|BIOUG16299-D11|Kate Perez|BIN Taxonomy Match|BOLD:AAM5377
Cricotopus rufiventris|BIOUG16479-F02|Kate Perez|BIN Taxonomy Match|BOLD:AAM5377
Cricotopus rufiventris|BIOUG16298-D09|Kate Perez|BIN Taxonomy Match|BOLD:AAM5377
Cricotopus rufiventris|BIOUG16430-B02|Kate Perez|BIN Taxonomy Match|BOLD:AAM5377
Cricotopus rufiventris|BIOUG16479-H06|Kate Perez|BIN Taxonomy Match|BOLD:AAM5377
Cricotopus rufiventris|BIOUG16328-C12|Kate Perez|BIN Taxonomy Match|BOLD:AAM5377
Cricotopus rufiventris|BIOUG16436-D08|Kate Perez|BIN Taxonomy Match|BOLD:AAM5377
Cricotopus rufiventris|BIOUG16478-F03|Kate Perez|BIN Taxonomy Match|BOLD:AAM5377
Cricotopus rufiventris|BIOUG15649-B08|Kate Perez|BIN Taxonomy Match|BOLD:AAM5377
Cricotopus rufiventris|BIOUG16557-G03|Kate Perez|BIN Taxonomy Match|BOLD:AAM5377
Cricotopus rufiventris|BIOUG15718-F05|Kate Perez|BIN Taxonomy Match|BOLD:AAM5377
Cricotopus rufiventris|BIOUG16426-H06|Kate Perez|BIN Taxonomy Match|BOLD:AAM5377
Cricotopus rufiventris|BIOUG16330-E03|Kate Perez|BIN Taxonomy Match|BOLD:AAM5377
Cricotopus intersectus|ZMUO.024755|Lauri Paasivirta||BOLD:ADA6259
Cricotopus intersectus|ZMUO.024754|Lauri Paasivirta||BOLD:ADA6259
Cricotopus laricomalis|NHRS-BYWS000000252|Yngve Brodin|morphology|BOLD:AAU2145
Cricotopus intersectus|ZMUO.024560|Lauri Paasivirta||BOLD:AEH3047
Cricotopus intersectus|ZMUO.024559|Lauri Paasivirta||BOLD:AEH3047
Cricotopus laricomalis|NHRS-BYWS000001152|Yngve Brodin|morphology|BOLD:AEH3047
Cricotopus laricomalis|NHRS-BYWS000001842|Yngve Brodin||BOLD:AEH3047
Cricotopus|ZSM-DIP-33135-B02|Caroline Chimeno|BIN Taxonomy Match (Jan 2022)|BOLD:AAV9062
Cricotopus intersectus|ES391|Susan E. Gresens|Morphology|BOLD:AAV9062
Cricotopus intersectus|NHRS-BYWS000000113|Yngve Brodin|morphology|BOLD:AAV9062
Cricotopus intersectus|NHRS-BYWS000000211|Yngve Brodin|morphology|BOLD:AAV9062
Cricotopus intersectus|NHRS-BYWS000000375|Yngve Brodin|morphology|BOLD:AAV9062
[illegible]

Cricotopus intersectus|NHRS-BYWS000000113|Yngve Brodin|morphology|BOLD:AAV9062
Cricotopus intersectus|NHRS-BYWS000000211|Yngve Brodin|morphology|BOLD:AAV9062
Cricotopus intersectus|NHRS-BYWS000000375|Yngve Brodin|morphology|BOLD:AAV9062
Cricotopus intersectus|NHRS-BYWS000001010|Yngve Brodin|morphology|BOLD:AAV9062
Cricotopus arcuatus|PP0270|Lauri Paasivirta||BOLD:AAV9062
Cricotopus intersectus|NHRS-BYWS000000208|Yngve Brodin|morphology|BOLD:AAV9062
Cricotopus intersectus|NHRS-BYWS000000185|Yngve Brodin|morphology|BOLD:AAV9062
Cricotopus intersectus|NHRS-BYWS000000184|Yngve Brodin|morphology|BOLD:AAV9062
Cricotopus intersectus|NHRS-BYWS000000112|Yngve Brodin|morphology|BOLD:AAV9062
Cricotopus intersectus|NHRS-BYWS000000197|Yngve Brodin|morphology|BOLD:AAV9062
Cricotopus intersectus|NHRS-BYWS000000304|Yngve Brodin|morphology|BOLD:AAV9062
Cricotopus intersectus|NHRS-BYWS000000111|Yngve Brodin|morphology|BOLD:AAV9062
Cricotopus intersectus|NHRS-BYWS000001746|Yngve Brodin||BOLD:AAV9062
Cricotopus reversus|PP0268|Lauri Paasivirta||BOLD:ADY8031
Cricotopus reversus|ZSM-DIP-33138-D08|Caroline Chimeno|BIN Taxonomy Match (Jan 2022)|BOLD ...
Cricotopus reversus|RIN_CH45|Elisabeth Stur|BOLD Identification engine|BOLD:AEO2598
Cricotopus reversus|ZMUO.026162|Lauri Paasivirta||BOLD:AEN9241
Cricotopus reversus|TRD-CH101|Susan E. Gresens|Morphology|BOLD:AEN9241
Cricotopus reversus|NO55|Susan E. Gresens|Morphology|BOLD:ABA3996
Cricotopus perniger|ZMUO.025323|Lauri Paasivirta||BOLD:ADF1505
Cricotopus perniger|ZMUO.025324|Lauri Paasivirta||BOLD:ADF1505
Cricotopus obnixus|NHRS-BYWS000000507|Yngve Brodin|morphology|BOLD:ADF1505
Cricotopus obnixus|NHRS-BYWS000000365|Yngve Brodin|morphology|BOLD:ADF1505
Cricotopus sp. 16ES|Finnmark268|Elisabeth Stur||BOLD:ADF1962
Chironomidae|ZSM-DIP-33143-G01|Valerie Levesque-Beaudin|BIN Taxonomy Match (Sep 2021)|BOLD...
Chironomidae|ZSM-DIP-33142-F04|Valerie Levesque-Beaudin|BOLD ID Engine Manual (Sep 2021)|BO...
Krenosmittia sp. 1SW|CCDB21606-E03|Sofia Wiedenbrug||BOLD:ACF7763
Krenosmittia sp. 1SW|BIOUG05395-H08|Kate Perez|BIN Taxonomy Match|BOLD:ACF7763
Chironomidae|CCDB24228-G06|Jerome Moriniere|BIN Taxonomy Match|BOLD:ACU4291
Krenosmittia|ES296|Elisabeth Stur||BOLD:AAI2212
Krenosmittia|ES297|Elisabeth Stur||BOLD:AAI2212
Krenosmittia camptophleps|Finnmark153|Elisabeth Stur||BOLD:AAF3195
Krenosmittia camptophleps|ATNA397|Elisabeth Stur||BOLD:AAF3195
Krenosmittia camptophleps|ATNA298|Elisabeth Stur||BOLD:AAF3195
Krenosmittia camptophleps|ATNA292|Elisabeth Stur||BOLD:AAF3195
Krenosmittia camptophleps|ATNA485|Elisabeth Stur||BOLD:AAF3195
Pseudorthocladius uniserratus|ZSM-DIP-33144-B05|Caroline Chimeno|BIN Taxonomy Match (Jan 2022...
Pseudorthocladius uniserratus|ZSM-DIP-33141-F10|Caroline Chimeno|BIN Taxonomy Match (Jan 2022...
Pseudorthocladius uniserratus|ZSM-DIP-33149-D12|Caroline Chimeno|BIN Taxonomy Match (Jan 2022...
Pseudorthocladius uniserratus|ZSM34341-C03|Sofia Wiedenbrug|Morphology|BOLD:ACU4557
Pseudorthocladius uniserratus|ZSM-DIP-33144-B06|Caroline Chimeno|BIN Taxonomy Match (Jan 2022...
Pseudorthocladius uniserratus|ZSM34341-G09|Sofia Wiedenbrug|Morphology|BOLD:ACU4557
Pseudorthocladius uniserratus|ZSM34341-G07|Sofia Wiedenbrug|Morphology|BOLD:ACU4557
Pseudorthocladius uniserratus|ZSM34341-C05|Sofia Wiedenbrug|Morphology|BOLD:ACU4557
Pseudorthocladius uniserratus|ZSM34341-D06|Sofia Wiedenbrug|Morphology|BOLD:ACU4557
Pseudorthocladius uniserratus|ZSM34341-G10|Sofia Wiedenbrug|Morphology|BOLD:ACU4557
Pseudorthocladius uniserratus|CCDB24229-B08|Sofia Wiedenbrug|BIN Taxonomy Match|BOLD:ACU4557
Pseudorthocladius uniserratus|CCDB24228-C03|Sofia Wiedenbrug|BIN Taxonomy Match|BOLD:ACU4557
Pseudorthocladius uniserratus|ZSM34341-D09|Sofia Wiedenbrug|Morphology|BOLD:ACU4557
Pseudorthocladius uniserratus|ZSM34341-B05|Sofia Wiedenbrug|Morphology|BOLD:ACU4557
Pseudorthocladius uniserratus|ZSM34341-G11|Sofia Wiedenbrug|Morphology|BOLD:ACU4557
Pseudorthocladius uniserratus|CCDB24229-A05|Sofia Wiedenbrug|BIN Taxonomy Match|BOLD:ACU4557
Pseudorthocladius uniserratus|ZSM-DIP-33130-G08|Caroline Chimeno|BIN Taxonomy Match (Jan 2022...
Pseudorthocladius|BIOUG05101-H04|Kate Perez|BIN Taxonomy Match|BOLD:ACD9519
Pseudorthocladius|BIOUG05103-C10|Kate Perez|BIN Taxonomy Match|BOLD:ACD9519
Pseudorthocladius|BIOUG05107-H10|Kate Perez|BIN Taxonomy Match|BOLD:ACD9519
Pseudorthocladius|BIOUG05101-C08|Kate Perez|BIN Taxonomy Match|BOLD:ACD9519
Pseudorthocladius|BIOUG04795-G05|Kate Perez|BIN Taxonomy Match|BOLD:ACD9519
Krenosmittia boreoalpina|ES45|Elisabeth Stur||BOLD:AAX0723
Krenosmittia|EBAI-Ch137|Elisabeth Stur|morphology|BOLD:ADA7846
Krenosmittia boreoalpina|BC-ZSM-DIP-22488-F09|Dieter Doczkal||BOLD:ACQ5543
Krenosmittia boreoalpina|CCDB21606-F08|Sofia Wiedenbrug||BOLD:ACQ5543
Krenosmittia boreoalpina|CCDB21606-F07|Sofia Wiedenbrug||BOLD:ACQ5543
Krenosmittia boreoalpina|CCDB21606-G02|Sofia Wiedenbrug||BOLD:ACQ5543
Krenosmittia boreoalpina|CCDB21606-D06|Sofia Wiedenbrug||BOLD:ACQ5543
Krenosmittia boreoalpina|GBOL03783|Sofia Wiedenbrug|BIN-taxonomy
Krenosmittia boreoalpina|GBOL03782|Sofia Wiedenbrug|BIN-taxonomy|BOLD:ACQ5543
Krenosmittia cf. boreoalpina|ES299|Elisabeth Stur||BOLD:AAX0725
Krenosmittia cf. boreoalpina|SOE15|Elisabeth Stur||BOLD:AAI2211
Krenosmittia boreoalpina|ZMUO.024840|Lauri Paasivirta||BOLD:AAI2211
Krenosmittia boreoalpina|ZMUO.024839|Lauri Paasivirta||BOLD:AAI2211
Krenosmittia cf. boreoalpina|Finnmark610|Elisabeth Stur||BOLD:AAI2211
Krenosmittia cf. boreoalpina|Finnmark269|Elisabeth Stur||BOLD:AAI2211
Paraphaenocladius intercedens|ZMUO.024652|Lauri Paasivirta||BOLD:AAI5795
Paraphaenocladius intercedens|ZMUO.026345|Lauri Paasivirta||BOLD:AAI5795
Paraphaenocladius intercedens|ATNA259|Elisabeth Stur||BOLD:AAI5795
Paraphaenocladius intercedens|ATNA212|Elisabeth Stur||BOLD:AAI5795
Monodiamesa bathyphila|KY225354
Monodiamesa bathyphila|KY225355
Monodiamesa bathyphila|KY225363|||BOLD:AAP6142
Monodiamesa bathyphila|KY225334|||BOLD:AAP6142
Monodiamesa bathyphila|Finnmark220|Elisabeth Stur||BOLD:AAP6142
Monodiamesa bathyphila|Finnmark219|Elisabeth Stur||BOLD:AAP6142
Zalutschia tornetraeskensis|Finnmark16|Elisabeth Stur||BOLD:ACG5345
Zalutschia tornetraeskensis|Finnmark27|Elisabeth Stur||BOLD:ACG5345
Zalutschia tornetraeskensis|Finnmark100|Elisabeth Stur||BOLD:ACG5345
Zalutschia sp. 1ES|Finnmark402|Elisabeth Stur
Natarsia|ZSM34341-F01|Sofia Wiedenbrug||BOLD:ADD7166
Paraphaenocladius sp. 1ES|Finnmark437|Elisabeth Stur||BOLD:AAV6215
Paraphaenocladius sp. 1ES|Finnmark641|Elisabeth Stur||BOLD:AAV6215
Parametriocnemus sp. 1SW|CCDB22605-H07|Sofia Wiedenbrug||BOLD:ACT9717
Parametriocnemus stylatus|ZMUO.024642|Lauri Paasivirta||BOLD:AAP6587
Parametriocnemus sp. 1ES|Finnmark188|Elisabeth Stur||BOLD:AAP6587
Parametriocnemus|BIOUG16525-A04|Kate Perez|BIN Taxonomy Match|BOLD:AAP6587
Parametriocnemus stylatus adzharicus|BIOUG16432-C03|Kate Perez|BIN Taxonomy Match|BOLD:AAW0334
Parametriocnemus stylatus|ZSM-DIP-33138-G12|Caroline Chimeno|BIN Taxonomy Match (Jan 2022)|...
Parametriocnemus stylatus adzharicus|CH-eik130|Elisabeth Stur||BOLD:AAW0334
Parametriocnemus stylatus|ZSM-DIP-33136-G03|Caroline Chimeno|BIN Taxonomy Match (Jan 2022)|...
Parametriocnemus stylatus adzharicus|CH-OSF59|Elisabeth Stur||BOLD:AAW0334
Parametriocnemus stylatus adzharicus|CH-OSF56|Elisabeth Stur||BOLD:AAW0334
Chironomidae|CCDB24037-F05||BIN Taxonomy Match
Parametriocnemus stylatus|CCDB22605-F01|Sofia Wiedenbrug||BOLD:ACT9205
Parametriocnemus stylatus|CCDB22605-F04|Sofia Wiedenbrug||BOLD:ACT9205

Chironomidae|CCDB24037-F05||BIN Taxonomy Match
Parametriocnemus stylatus|CCDB22605-F01|Sofia Wiedenbrug||BOLD:ACT9205
Parametriocnemus stylatus|CCDB22605-F04|Sofia Wiedenbrug||BOLD:ACT9205
Parametriocnemus stylatus|ZSM-DIP-33134-B11|Caroline Chimeno|BIN Taxonomy Match (Jan 2022)|...
Parametriocnemus stylatus|ZMUO.024643|Lauri Paasivirta||BOLD:ADA7271
Parametriocnemus stylatus|ZMUO.025928|Lauri Paasivirta||BOLD:ADA7271
Parametriocnemus stylatus|ZSM-DIP-33137-D03|Caroline Chimeno|BIN Taxonomy Match (Jan 2022)|...
Parametriocnemus stylatus adzharicus|TRD-CH402|Elisabeth Stur|morphology|BOLD:ACT9205
Parametriocnemus stylatus adzharicus|CH-OSF122|Elisabeth Stur||BOLD:ACT9205
Parametriocnemus stylatus|CCDB21605-D09|Sofia Wiedenbrug||BOLD:AAI2687
Parametriocnemus stylatus|ZSM34341-C06|Sofia Wiedenbrug|Morphology
Parametriocnemus stylatus|BC-ZSM-DIP-22488-F07|Dieter Doczkal||BOLD:AAI2687
Parametriocnemus stylatus|BC-ZSM-DIP-22488-G10|Dieter Doczkal||BOLD:AAI2687
Parametriocnemus stylatus|CCDB24228-C01|Sofia Wiedenbrug|BIN Taxonomy Match|BOLD:AAI2687
Parametriocnemus stylatus|ZSM34341-F04|Sofia Wiedenbrug|BIN-taxonomy|BOLD:AAI2687
Parametriocnemus stylatus|CCDB24037-G03|Sofia Wiedenbrug|BIN Taxonomy Match|BOLD:AAI2687
Parametriocnemus stylatus|CCDB24037-F06|Sofia Wiedenbrug|BIN Taxonomy Match|BOLD:AAI2687
Parametriocnemus stylatus adzharicus|ES285|Elisabeth Stur||BOLD:AAI2687
Parametriocnemus|CCDB24039-F10|Sofia Wiedenbrug|BIN Taxonomy Match|BOLD:ACU4174
Parametriocnemus|CCDB24038-H08|Sofia Wiedenbrug|BIN Taxonomy Match|BOLD:ACU4173
Parametriocnemus sp. 2SW|BC-ZSM-DIP-22488-C09|Dieter Doczkal||BOLD:ACT5782
Parametriocnemus sp. 2SW|CCDB21606-A12|Sofia Wiedenbrug||BOLD:ACT5782
Parametriocnemus sp. 2SW|ZSM34342-G01|Sofia Wiedenbrug|Morphology|BOLD:ACT5782
Parametriocnemus sp. 2SW|CCDB24039-E03|Sofia Wiedenbrug||BOLD:ACT5782
Parametriocnemus stylatus|SOE185|Elisabeth Stur||BOLD:AAB4494
Parametriocnemus stylatus|ES14|Elisabeth Stur||BOLD:AAB4494
Parametriocnemus stylatus|SOE49|Elisabeth Stur||BOLD:AAB4494
Parametriocnemus stylatus|SOE280|Elisabeth Stur||BOLD:AAB4494
Parametriocnemus stylatus|SOE460|Elisabeth Stur||BOLD:AAB4494
Parametriocnemus stylatus|SOE464|Elisabeth Stur||BOLD:AAB4494
Parametriocnemus sp.|CCDB21606-E04|Sofia Wiedenbrug||BOLD:AAB4494
Parametriocnemus stylatus|SOE418|Elisabeth Stur||BOLD:AAB4494
Parametriocnemus stylatus|ATNA221|Elisabeth Stur||BOLD:AAB4494
Parametriocnemus stylatus|SOE286|Elisabeth Stur||BOLD:AAB4494
Parametriocnemus stylatus|ES22|Elisabeth Stur||BOLD:AAB4494
Parametriocnemus stylatus|SOE275|Elisabeth Stur||BOLD:AAB4494
Parametriocnemus stylatus|SOE104|||BOLD:AAB4494
Parametriocnemus stylatus|SOE285|Elisabeth Stur||BOLD:AAB4494
Parametriocnemus stylatus|SOE145|Elisabeth Stur||BOLD:AAB4494
Parametriocnemus stylatus|SOE183|Elisabeth Stur||BOLD:AAB4494
Parametriocnemus stylatus|SOE103|||BOLD:AAB4494
Parametriocnemus stylatus|SOE465|Elisabeth Stur||BOLD:AAB4494
Parametriocnemus stylatus|SOE468|Elisabeth Stur||BOLD:AAB4494
Parametriocnemus stylatus|ATNA196|Elisabeth Stur||BOLD:AAB4494
Parametriocnemus stylatus|SOE154|Elisabeth Stur||BOLD:AAB4494
Parametriocnemus stylatus|SOE435|Elisabeth Stur||BOLD:AAB4494
Parametriocnemus stylatus|SOE106|||BOLD:AAB4494
Parametriocnemus stylatus|SOE102|||BOLD:AAB4494
Parametriocnemus lundbeckii|Finnmark880|G. A. Halvorsen||BOLD:AAP6586
Parametriocnemus lundbeckii|EBAI-Ch268|Elisabeth Stur|morphology|BOLD:AAP6586
Parametriocnemus lundbeckii|Finnmark164|Elisabeth Stur||BOLD:AAP6586
Parametriocnemus lundbeckii|Finnmark599|Elisabeth Stur||BOLD:AAP6586
Paraphaenocladius|CCDB24037-A08|Sofia Wiedenbrug|BIN Taxonomy Match|BOLD:ACU4854
Bryophaenocladius subvernalis|SOE138|Torbjorn Ekrem||BOLD:AAI8503
Bryophaenocladius subvernalis|SOE141|Torbjorn Ekrem||BOLD:AAI8503
Apsectrotanypus trifascipennis|CCDB22605-E01|Sofia Wiedenbrug||BOLD:ACD1957
Apsectrotanypus trifascipennis|TRD-CH285|Elisabeth Stur||BOLD:ACD1957
Apsectrotanypus trifascipennis|TRD-CH284|Elisabeth Stur||BOLD:ACD1957
Apsectrotanypus trifascipennis|TRD-CH219|Elisabeth Stur||BOLD:ACD1957
Apsectrotanypus trifascipennis|BIOUG43948-E12|Kate Perez|BIN Taxonomy Match below Phylum (Jan ...
Apsectrotanypus trifascipennis|ZMUO.024788|Lauri Paasivirta||BOLD:ACD1957
Apsectrotanypus trifascipennis|ZMUO.024789|Lauri Paasivirta||BOLD:ACD1957
Apsectrotanypus trifascipennis|TRD-CH375|Elisabeth Stur|morphology|BOLD:ACD1957
Apsectrotanypus trifascipennis|TRD-CH220|Elisabeth Stur||BOLD:ACD1957
Apsectrotanypus trifascipennis|Finnmark746|Elisabeth Stur||BOLD:ACD1957
Paraphaenocladius|CCDB24183-D11|Sofia Wiedenbrug|Morphology|BOLD:ACF8295
Paraphaenocladius|BIOUG05149-E12|Kate Perez|BIN Taxonomy Match|BOLD:ACF8295
Paraphaenocladius pseudirritus|ZSM-DIP-33140-D11|Valerie Levesque-Beaudin|BIN Taxonomy Match (...
Paraphaenocladius pseudirritus|ZSM-DIP-33140-C09|Valerie Levesque-Beaudin|BIN Taxonomy Match (...
Paraphaenocladius pseudirritus|ZSM-DIP-33136-F03|Valerie Levesque-Beaudin|BIN Taxonomy Match (...
Paraphaenocladius pseudirritus|ZSM-DIP-33136-E08|Valerie Levesque-Beaudin|BOLD ID Engine Manual ...
Paraphaenocladius pseudirritus|ZSM-DIP-33140-E02|Valerie Levesque-Beaudin|BIN Taxonomy Match (...
Paraphaenocladius pseudirritus|TRD-CH61|Elisabeth Stur||BOLD:ACK3521
Paraphaenocladius pseudirritus|ZSM-DIP-33137-C01|Caroline Chimeno|BIN Taxonomy Match (Jan 2022...
Paraphaenocladius pseudirritus|ZMUO.024680|Lauri Paasivirta||BOLD:ACK3521
Paraphaenocladius pseudirritus|ZMUO.024681|Lauri Paasivirta||BOLD:ACK3521
Paraphaenocladius pseudirritus|TRD-CH18|Elisabeth Stur||BOLD:ACK3521
Paraphaenocladius pseudirritus|ATNA202|Elisabeth Stur||BOLD:AAC4195
Paraphaenocladius pseudirritus|ATNA265|Elisabeth Stur||BOLD:AAC4195
Paraphaenocladius pseudirritus|ZMUO.026338|Lauri Paasivirta||BOLD:AAC4195
Paraphaenocladius pseudirritus|ATNA390|Elisabeth Stur||BOLD:AAC4195
Paraphaenocladius pseudirritus|BIOUG15463-G07|Kate Perez|BIN Taxonomy Match|BOLD:AAC4195
Paraphaenocladius|24502-H01|Kate Perez|BIN Taxonomy Match|BOLD:ACJ1112
Paraphaenocladius|24372-D01|Kate Perez|BIN Taxonomy Match|BOLD:ACJ1112
Paraphaenocladius pseudirritus|CCDB21605-C06|Sofia Wiedenbrug
Paraphaenocladius pseudirritus|CCDB21606-G09|Sofia Wiedenbrug||BOLD:AAC4194
Paraphaenocladius pseudirritus|CCDB21605-C04|Sofia Wiedenbrug||BOLD:AAC4194
Paraphaenocladius pseudirritus|ZSM34341-C01|Sofia Wiedenbrug|Morphology|BOLD:AAC4194
Paraphaenocladius pseudirritus|CCDB24228-C02|Jerome Moriniere|BIN Taxonomy Match|BOLD:AAC4194
Paraphaenocladius pseudirritus|CCDB21605-C05|Sofia Wiedenbrug||BOLD:AAC4194
Paraphaenocladius pseudirritus|BIOUG05945-B04|Kate Perez|BIN Taxonomy Match|BOLD:AAC4194
Paraphaenocladius pseudirritus|SOE439|Elisabeth Stur
Paraphaenocladius pseudirritus|CH-OSF186|Elisabeth Stur||BOLD:AAC4194
Paraphaenocladius pseudirritus|SOE340|Elisabeth Stur||BOLD:AAC4194
Paraphaenocladius pseudirritus|SOE153|Elisabeth Stur||BOLD:AAC4194
Paraphaenocladius pseudirritus|ZSM-DIP-33134-A05|Caroline Chimeno|BIN Taxonomy Match (Jan 2022...
Paraphaenocladius pseudirritus|ZSM-DIP-33170-H10|Caroline Chimeno|BIN Taxonomy Match (Jan 2022...
Paraphaenocladius pseudirritus|BIOUG05944-D08|Kate Perez|BIN Taxonomy Match|BOLD:AAC4194
Paraphaenocladius pseudirritus|SOE332|Elisabeth Stur||BOLD:AAC4194
Paraphaenocladius pseudirritus|SOE293|Elisabeth Stur||BOLD:AAC4194
Paraphaenocladius pseudirritus|SOE473|Elisabeth Stur||BOLD:AAC4194
Paraphaenocladius pseudirritus|SOE299|Elisabeth Stur||BOLD:AAC4194
Paraphaenocladius pseudirritus|SOE188|Elisabeth Stur||BOLD:AAC4194

Paraphaenocladius pseudirritus|SOE473|Elisabeth Stur||BOLD:AAC4194
Paraphaenocladius pseudirritus|SOE299|Elisabeth Stur||BOLD:AAC4194
Paraphaenocladius pseudirritus|SOE188|Elisabeth Stur||BOLD:AAC4194
Paraphaenocladius pseudirritus|SOE463|Elisabeth Stur||BOLD:AAC4194
Chironomidae|ZSM-DIP-33136-B12|Valerie Levesque-Beaudin|BIN Taxonomy Match (Sep 2021)|BOLD...
Chironomidae|ZSM-DIP-33136-B11|Valerie Levesque-Beaudin|BOLD ID Engine Manual (Sep 2021)|BO...
Chironomidae|ZSM-DIP-33138-H01|Valerie Levesque-Beaudin|BIN Taxonomy Match (Sep 2021)|BOLD...
Chironomidae|ZSM-DIP-33136-C05|Valerie Levesque-Beaudin|BIN Taxonomy Match (Sep 2021)|BOLD...
Chironomidae|ZSM-DIP-33149-C01|Valerie Levesque-Beaudin|BIN Taxonomy Match (Sep 2021)|BOLD...
Chironomidae|ZSM-DIP-33140-D06|Valerie Levesque-Beaudin|BIN Taxonomy Match (Sep 2021)|BOLD...
Chironomidae|ZSM-DIP-33139-E01|Valerie Levesque-Beaudin|BIN Taxonomy Match (Sep 2021)|BOLD...
Chironomidae|ZSM-DIP-33136-E06|Valerie Levesque-Beaudin|BIN Taxonomy Match (Sep 2021)|BOLD...
Chironomidae|ZSM-DIP-33136-E05|Valerie Levesque-Beaudin|BIN Taxonomy Match (Sep 2021)|BOLD...
Chironomidae|ZSM-DIP-33133-H11|Valerie Levesque-Beaudin|BIN Taxonomy Match (Sep 2021)|BOLD...
Paraphaenocladius|CCDB24038-B03|Sofia Wiedenbrug|BIN Taxonomy Match|BOLD:ACU4391
Paraphaenocladius irritus|ZSM-DIP-33133-H07|Caroline Chimeno|BIN Taxonomy Match (Jan 2022)|...
Paraphaenocladius irritus|ATNA297|Elisabeth Stur||BOLD:AAF7259
Paraphaenocladius irritus|ATNA201|Elisabeth Stur||BOLD:AAF7259
Paraphaenocladius irritus|SOE446|Torbjorn Ekrem||BOLD:AAF7259
Paraphaenocladius sp. 3ES|Finnmark576|Elisabeth Stur||BOLD:AAM5383
Paraphaenocladius sp. 3ES|Finnmark196|Elisabeth Stur||BOLD:AAM5383
Paraphaenocladius sp. 3ES|Finnmark517|Elisabeth Stur||BOLD:AAM5383
Paraphaenocladius sp. 3ES|Finnmark575|Elisabeth Stur||BOLD:AAM5383
Paraphaenocladius sp. 3ES|BIOUG04437-E08|Kate Perez|BIN Taxonomy Match|BOLD:AAM5383
Paraphaenocladius sp. 3ES|BIOUG04437-D05|Kate Perez|BIN Taxonomy Match|BOLD:AAM5383
Paraphaenocladius sp. 3ES|Finnmark691|Elisabeth Stur||BOLD:AAM5383
Paraphaenocladius sp. 3ES|Finnmark204|Elisabeth Stur||BOLD:AAM5383
Paraphaenocladius sp. 3ES|ATNA511|Elisabeth Stur||BOLD:AAM5383
Paraphaenocladius sp. 3ES|Finnmark571|Elisabeth Stur||BOLD:AAM5383
Propsilocerus saetheri|Finnmark749|G. A. Halvorsen||BOLD:AAM7072
Propsilocerus saetheri|Finnmark747|G. A. Halvorsen||BOLD:AAM7072
Propsilocerus saetheri|Finnmark45|Elisabeth Stur||BOLD:AAM7072
Diplocladius cultriger|ZMUO.024305|Lauri Paasivirta||BOLD:ADA5683
Chironomidae|BIOUG04261-E04|Kate Perez|BIN Taxonomy Match|BOLD:ACC1683
Diplocladius cultriger|TRD-CH395|Elisabeth Stur|morphology|BOLD:AAM5628
Diplocladius cultriger|TRD-CH396|Elisabeth Stur|morphology|BOLD:AAM5628
Diplocladius cultriger|CH-OSF02|Elisabeth Stur||BOLD:AAM5628
Diplocladius cultriger|ATNA484|Elisabeth Stur||BOLD:AAM5628
Diplocladius cultriger|ZMUO.025924|Lauri Paasivirta||BOLD:AAM7068
Diplocladius cultriger|Finnmark18|Elisabeth Stur||BOLD:AAM7068
Diplocladius cultriger|ZMUO.025925|Lauri Paasivirta||BOLD:AAM7068
Diplocladius cultriger|Finnmark480|Elisabeth Stur||BOLD:AAM7068
Diplocladius|Finnmark305|Elisabeth Stur||BOLD:AAV1758
Diplocladius cultriger|ZMUO.026323|Lauri Paasivirta||BOLD:AAV1758
Diplocladius|Finnmark303|Elisabeth Stur||BOLD:AAV1758
Macropelopia nebulosa|KY225353
Macropelopia fittkau|ES71|Elisabeth Stur||BOLD:AAX3573
Macropelopia johnseni|ZSM-DIP-33141-F12|Caroline Chimeno|BIN Taxonomy Match (Jan 2022)|BOLD...
Macropelopia nebulosa|TRD-CH323|Elisabeth Stur||BOLD:AAX3566
Macropelopia nebulosa|ZMUO.024831|Lauri Paasivirta||BOLD:AAX3566
Macropelopia nebulosa|TRD-CH183|Elisabeth Stur||BOLD:AAX3566
Macropelopia nebulosa|TRD-CH156|Elisabeth Stur|morphology|BOLD:AAX3566
Macropelopia nebulosa|TRD-CH170|Elisabeth Stur||BOLD:AAX3566
Macropelopia|BIOUG43946-H02|Kate Perez|BIN Taxonomy Match below Phylum (Jan 2019)|BOLD:A...
Macropelopia nebulosa|TRD-CH180|Elisabeth Stur||BOLD:AAX3566
Macropelopia nebulosa|NO25|Elisabeth Stur||BOLD:AAX3566
Macropelopia nebulosa|TRD-CH373|Elisabeth Stur|morphology|BOLD:AAX3566
Macropelopia|BIOUG43946-E07|Kate Perez|BIN Taxonomy Match below Phylum (Jan 2019)|BOLD:A...
Macropelopia nebulosa|ZMUO.024832|Lauri Paasivirta||BOLD:AAX3566
Macropelopia nebulosa|TRD-CH374|Elisabeth Stur|morphology|BOLD:AAX3566
Macropelopia nebulosa|TRD-CH134|Elisabeth Stur|morphology|BOLD:AAX3566
Macropelopia nebulosa|TRD-CH109|Elisabeth Stur||BOLD:AAX3566
Macropelopia nebulosa|TRD-CH108|Elisabeth Stur||BOLD:AAX3566
Natarsia punctata|BIOUG44442-H02|Kate Perez|BIN Taxonomy Match below Phylum (Mar 2019)|BOLD...
Natarsia punctata|CH-OSF35|Torbjorn Ekrem||BOLD:ABU9441
Natarsia punctata|ATNA564|Elisabeth Stur||BOLD:AEN3605
Natarsia punctata|ATNA576|Elisabeth Stur|morphology|BOLD:AEN3605
Natarsia punctata|ATNA575|Elisabeth Stur|morphology|BOLD:AEN3605
Natarsia punctata|ZSM-DIP-33143-D06|Caroline Chimeno|BIN Taxonomy Match (Jan 2022)|BOLD:...
Natarsia punctata|TRD-CH348|Elisabeth Stur|morphology|BOLD:AAV2697
Natarsia punctata|TRD-CH9|Elisabeth Stur||BOLD:AAV2697
Natarsia punctata|NO 93|Elisabeth Stur||BOLD:AAV2697
Natarsia punctata|TRD-CH349|Elisabeth Stur|morphology|BOLD:AAV2697
Natarsia punctata|NHRS-BYWS000000992|Yngve Brodin|morphology|BOLD:AAV2697
Natarsia punctata|CCDB24183-E06|Sofia Wiedenbrug|Morphology|BOLD:AAV2697
Natarsia punctata|NHRS-BYWS000000985|Yngve Brodin|morphology|BOLD:AAV2697
Natarsia punctata|NHRS-BYWS000000189|Yngve Brodin|morphology|BOLD:AAV2697
Natarsia punctata|ZMUO.024728|Lauri Paasivirta||BOLD:AAV2697
Natarsia punctata|ZSM-DIP-33141-G10|Caroline Chimeno|BIN Taxonomy Match (Jan 2022)|BOLD:...
Natarsia punctata|CCDB24183-G05|Sofia Wiedenbrug|BIN-taxonomy|BOLD:AAV2697
Natarsia punctata|CCDB24183-F01|Sofia Wiedenbrug|BIN-taxonomy|BOLD:AAV2697
Natarsia punctata|CCDB24183-E09|Sofia Wiedenbrug|BIN-taxonomy|BOLD:AAV2697
Natarsia punctata|CCDB24183-E08|Sofia Wiedenbrug|BIN-taxonomy|BOLD:AAV2697
Natarsia punctata|CCDB24183-E07|Sofia Wiedenbrug|Morphology|BOLD:AAV2697
Natarsia punctata|CH-OSF113|Elisabeth Stur||BOLD:AAV2697
Natarsia punctata|NHRS-BYWS000000984|Yngve Brodin|morphology|BOLD:AAV2697
Natarsia punctata|TRD-CH312|Elisabeth Stur||BOLD:AAV2697
Natarsia punctata|ZMUO.024729|Lauri Paasivirta||BOLD:AAV2697
Natarsia punctata|TRD-CH71|Elisabeth Stur||BOLD:AAV2697
Natarsia punctata|TRD-CH40|Elisabeth Stur||BOLD:AAV2697
Natarsia punctata|TRD-CH5|Elisabeth Stur||BOLD:AAV2697
Natarsia punctata|RIN_CH14|Mona Renate Saursaunet|BOLD Identification engine|BOLD:AAV2697
Natarsia punctata|Finnmark417|Torbjorn Ekrem||BOLD:AAV2697
Natarsia punctata|NO 91|Elisabeth Stur||BOLD:AAV2697
Macropelopia|ZMUO.025895|Lauri Paasivirta||BOLD:AAC1445
Macropelopia|ZMUO.025894|Lauri Paasivirta||BOLD:AAC1445
Macropelopia notata|SOE172|Elisabeth Stur||BOLD:AAC1445
Macropelopia notata|SOE164|Elisabeth Stur||BOLD:AAC1445
Macropelopia notata|SOE266|Torbjorn Ekrem||BOLD:AAC1445
Macropelopia notata|SOE267|Torbjorn Ekrem||BOLD:AAC1445
Macropelopia notata|SOE63|Elisabeth Stur||BOLD:AAC1445
Macropelopia notata|SOE64|Torbjorn Ekrem||BOLD:AAC1445
Macropelopia notata|SOE65|Torbjorn Ekrem||BOLD:AAC1445

[illegible]
Macropelopia notata|SOE63|Elisabeth Stur||BOLD:AAC1445
Macropelopia notata|SOE64|Torbjorn Ekrem||BOLD:AAC1445
Macropelopia notata|SOE65|Torbjorn Ekrem||BOLD:AAC1445
Macropelopia notata|SOE66|Torbjorn Ekrem||BOLD:AAC1445
Macropelopia notata|SOE67|Torbjorn Ekrem||BOLD:AAC1445
Macropelopia notata|SOE393|Torbjorn Ekrem||BOLD:AAC1445
Macropelopia notata|SOE270|Torbjorn Ekrem||BOLD:AAC1445
Macropelopia notata|ZSM-DIP-33147-A08|Caroline Chimeno|BIN Taxonomy Match (Jan 2022)|BOLD ...
Macropelopia|ZSM-DIP-33149-F07|Caroline Chimeno|BIN Taxonomy Match (Jan 2022)|BOLD:AAC1445
Macropelopia notata|ZMUO.025291|Lauri Paasivirta||BOLD:AAC1445
Macropelopia notata|BIOUG08179-C08|Kate Perez|BIN Taxonomy Match|BOLD:AAC1445
Macropelopia notata|SOE62|Elisabeth Stur||BOLD:AAC1445
Macropelopia notata|SOE171|Torbjorn Ekrem||BOLD:AAC1445
Macropelopia notata|ES74|Torbjorn Ekrem||BOLD:AAC1445
Hydrobaenus lapponicus|Finnmark681|Elisabeth Stur||BOLD:AAV2835
Hydrobaenus lapponicus|Finnmark364|Elisabeth Stur||BOLD:AAV2835
Psilometriocnemus europaeus|Finnmark207|Elisabeth Stur||BOLD:AAP6933
Psilometriocnemus europaeus|Finnmark291|Elisabeth Stur||BOLD:AAP6933
Psilometriocnemus europaeus|Finnmark290|Elisabeth Stur||BOLD:AAP6933
Psilometriocnemus europaeus|Finnmark283|Elisabeth Stur||BOLD:AAP6933
Psilometriocnemus europaeus|Finnmark278|Elisabeth Stur||BOLD:AAP6933
Chironomidae|ZSM-DIP-33130-G10|Caroline Chimeno|BIN Taxonomy Match (Jan 2022)|BOLD:ACN4615
Pseudosmittia mathildae|ZMUO.025371|Lauri Paasivirta||BOLD:AAV2779
Pseudosmittia mathildae|BIOUG55166-B04|Kate Perez|BIN Taxonomy Match (Oct 2020)|BOLD:AAV...
Pseudosmittia mathildae|Finnmark732|Elisabeth Stur||BOLD:AAV2779
Pseudosmittia mathildae|ZMUO.025370|Lauri Paasivirta||BOLD:AAV2779
Acamptocladius submontanus|Finnmark514|Elisabeth Stur||BOLD:AAV9928
Macropelopia adaucta|ZMUO.024883|Lauri Paasivirta||BOLD:ADA7607
Macropelopia adaucta|ZMUO.024882|Lauri Paasivirta||BOLD:ADA7607
Chironomidae|BIOUG43949-A09|Kate Perez|BIN Taxonomy Match below Phylum (Jan 2019)|BOLD:A...
Heterotanytarsus apicalis|KY225349|||BOLD:ADA7607
Chironomidae|BIOUG04155-G07
Smittia|BIOUG04212-D01|Kate Perez|BIN Taxonomy Match|BOLD:ACB9814
Smittia sp. 1 SW|BIOUG06062-D06|Kate Perez|BIN Taxonomy Match|BOLD:ACB9814
Parasmittia|ZMUO.026169|Lauri Paasivirta||BOLD:ACB9814
Smittia sp. 1 SW|BIOUG07566-B07|Kate Perez|BOLD ID Engine Manual|BOLD:ACB9814
Chironomidae|BIOUG04216-A12
Smittia sp. 1 SW|BIOUG06058-A07|Kate Perez|BIN Taxonomy Match|BOLD:ACB9814
Smittia sp. 1 SW|BIOUG06100-H02|Kate Perez|BIN Taxonomy Match|BOLD:ACB9814
Parakiefferiella scandica|Finnmark394|Elisabeth Stur||BOLD:AAP6583
Parakiefferiella scandica|Finnmark390|Elisabeth Stur||BOLD:AAP6583
Parakiefferiella scandica|ES380|Elisabeth Stur||BOLD:AAP6583
Parakiefferiella scandica|Finnmark395|Elisabeth Stur||BOLD:AAP6583
Parakiefferiella scandica|ZMUO.025827|Lauri Paasivirta||BOLD:AAP6583
Parakiefferiella scandica|ZMUO.025826|Lauri Paasivirta||BOLD:AAP6583
Parakiefferiella scandica|ES349|Elisabeth Stur||BOLD:AAP6583
Parakiefferiella scandica|Finnmark680|Elisabeth Stur||BOLD:AAP6583
Parakiefferiella scandica|Finnmark249|Elisabeth Stur||BOLD:AAP6583
Parakiefferiella scandica|Finnmark186|Elisabeth Stur||BOLD:AAP6583
Parakiefferiella scandica|Finnmark141|Elisabeth Stur||BOLD:AAP6583
Parakiefferiella scandica|Finnmark139|Elisabeth Stur||BOLD:AAP6583
Parakiefferiella scandica|Finnmark367|Elisabeth Stur||BOLD:AAP6583
Parakiefferiella scandica|Finnmark365|Elisabeth Stur||BOLD:AAP6583
Parakiefferiella cf. coronata|NHRS-BYWS000000444|Yngve Brodin|morphology|BOLD:ACR3777
Parakiefferiella cf. bilobata|Finnmark30|Elisabeth Stur||BOLD:AAM7070
Parakiefferiella cf. bilobata|Finnmark735|Elisabeth Stur||BOLD:AAM7070
Parakiefferiella cf. bilobata|Finnmark29|Elisabeth Stur||BOLD:AAM7070
Parakiefferiella bathophila|ZMUO.024528|Lauri Paasivirta||BOLD:ADA5771
Parakiefferiella bathophila|ZMUO.024527|Lauri Paasivirta||BOLD:ADA5771
Parakiefferiella bathophila|ZSM-DIP-33134-E12|Caroline Chimeno|BIN Taxonomy Match (Jan 2022 ...
Parakiefferiella bathophila|Finnmark430|Elisabeth Stur||BOLD:ABX4968
Parakiefferiella bathophila|Finnmark397|Elisabeth Stur||BOLD:ABX4968
Parakiefferiella bathophila|Finnmark396|Elisabeth Stur||BOLD:ABX4968
Parakiefferiella bathophila|EJV-20110205|Lauri Paasivirta||BOLD:AAW4857
Parakiefferiella bathophila|BIOUG16296-E11|Kate Perez|BIN Taxonomy Match|BOLD:AAW4857
Parakiefferiella bathophila|BIOUG15952-E06|Kate Perez|BIN Taxonomy Match|BOLD:AAW4857
Parakiefferiella bathophila|EJV-20110204|Lauri Paasivirta||BOLD:AAW4857
Parakiefferiella bathophila|ZSM-DIP-33130-H06|Caroline Chimeno|BIN Taxonomy Match (Jan 2022 ...
Parakiefferiella bathophila|ZSM-DIP-33130-H05|Caroline Chimeno|BIN Taxonomy Match (Jan 2022 ...
Parakiefferiella bathophila|CCDB21606-B09|Sofia Wiedenbrug||BOLD:AAE6766
Parakiefferiella bathophila|CCDB21606-B08|Sofia Wiedenbrug||BOLD:AAE6766
Parakiefferiella bathophila|CCDB21606-B06|Sofia Wiedenbrug||BOLD:AAE6766
Parakiefferiella bathophila|Finnmark482|Elisabeth Stur||BOLD:AAE6766
Parakiefferiella bathophila|Finnmark481|Elisabeth Stur||BOLD:AAE6766
Parakiefferiella bathophila|NO 100|Elisabeth Stur||BOLD:AAE6766
Parakiefferiella bathophila|Finnmark486|Elisabeth Stur||BOLD:AAE6766
Parakiefferiella bathophila|Finnmark588|Elisabeth Stur||BOLD:AAE6766
Parakiefferiella sp. 1SW|BIOUG05144-H11|Sofia Wiedenbrug|BIN Taxonomy Match|BOLD:ACE6037
Parakiefferiella gracillima|ES23|Elisabeth Stur||BOLD:AAX2331
Orthocladiinae|ES287|||BOLD:AAG3900
Tanypus|BIOUG24395-A10|Kate Perez||BOLD:ABW1514
Tanypus stellatus|MT356616|||BOLD:ABW1514
Tanypus sp. 1SMJ061913-THBJ013|MT526178|||BOLD:ABW1514
Tanypus|BIOUG24395-B02|Kate Perez||BOLD:ABW1514
Tanypus|BIOUG02775-F06|Angela Telfer|BIN Taxonomy Match|BOLD:ABW1514
Tanypus|BIOUG01786-F06|Kate Perez|BIN Taxonomy Match|BOLD:ABW1514
Tanypus|BIOUG24361-H01|Kate Perez||BOLD:ABW1514
Tanypus|BIOUG24361-F11|Kate Perez||BOLD:ABW1514
Tanypus|BIOUG24361-G02|Kate Perez||BOLD:ABW1514
Tanypus|BIOUG24360-H03|Kate Perez||BOLD:ABW1514
Tanypus|BIOUG24361-A12|Kate Perez||BOLD:ABW1514
Tanypus|BIOUG24361-D07|Kate Perez||BOLD:ABW1514
Tanypus|BIOUG24361-E09|Kate Perez||BOLD:ABW1514
Tanypus|BIOUG24361-D02|Kate Perez||BOLD:ABW1514
Tanypus|BIOUG24395-A08|Kate Perez||BOLD:ABW1514
Tanypus|BIOUG24361-A06|Kate Perez||BOLD:ABW1514
Tanypus|BIOUG03444-C06|Kate Perez|BIN Taxonomy Match|BOLD:ABW1514
Tanypus|BIOUG24360-F01|Kate Perez||BOLD:ABW1514
Tanypus|BIOUG24361-G09|Kate Perez||BOLD:ABW1514
Tanypus|BIOUG24360-E06|Kate Perez||BOLD:ABW1514
Tanypus|BIOUG24361-D11|Kate Perez||BOLD:ABW1514
Tanypus|BIOUG02271-B03|Angela Telfer|BIN Taxonomy Match|BOLD:ABW1514
[illegible]

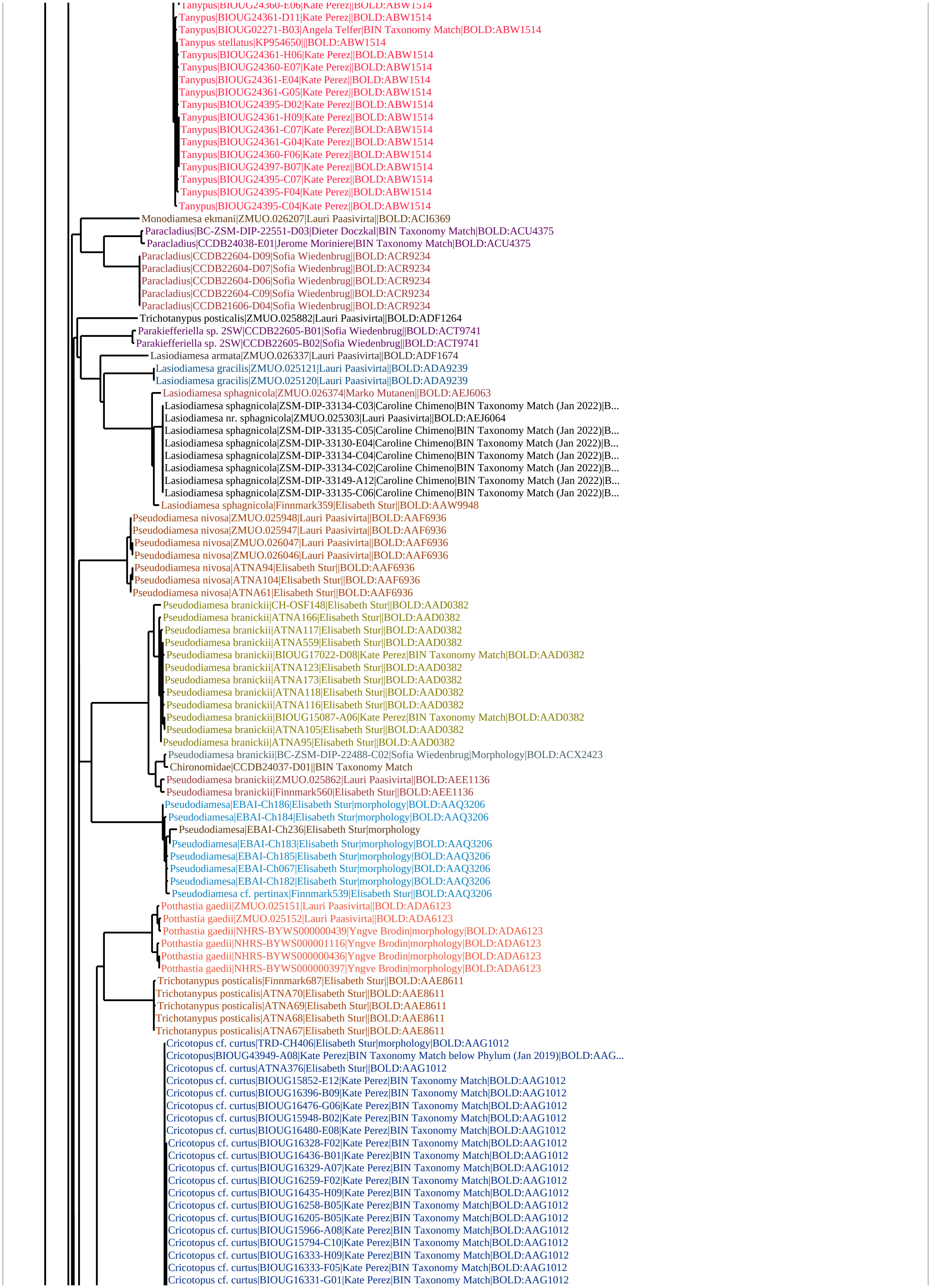
Tanypus|BIOUG24360-E06|Kate Perez||BOLD:ABW1514
Tanypus|BIOUG24361-D11|Kate Perez||BOLD:ABW1514
Tanypus|BIOUG02271-B03|Angela Telfer|BIN Taxonomy Match|BOLD:ABW1514
Tanypus stellatus|KP954650|||BOLD:ABW1514
Tanypus|BIOUG24361-H06|Kate Perez||BOLD:ABW1514
Tanypus|BIOUG24360-E07|Kate Perez||BOLD:ABW1514
Tanypus|BIOUG24361-E04|Kate Perez||BOLD:ABW1514
Tanypus|BIOUG24361-G05|Kate Perez||BOLD:ABW1514
Tanypus|BIOUG24395-D02|Kate Perez||BOLD:ABW1514
Tanypus|BIOUG24361-H09|Kate Perez||BOLD:ABW1514
Tanypus|BIOUG24361-C07|Kate Perez||BOLD:ABW1514
Tanypus|BIOUG24361-G04|Kate Perez||BOLD:ABW1514
Tanypus|BIOUG24360-F06|Kate Perez||BOLD:ABW1514
Tanypus|BIOUG24397-B07|Kate Perez||BOLD:ABW1514
Tanypus|BIOUG24395-C07|Kate Perez||BOLD:ABW1514
Tanypus|BIOUG24395-F04|Kate Perez||BOLD:ABW1514
Tanypus|BIOUG24395-C04|Kate Perez||BOLD:ABW1514
Monodiamesa ekmani|ZMUO.026207|Lauri Paasivirta||BOLD:ACI6369
Paracladius|BC-ZSM-DIP-22551-D03|Dieter Doczkal|BIN Taxonomy Match|BOLD:ACU4375
Paracladius|CCDB24038-E01|Jerome Moriniere|BIN Taxonomy Match|BOLD:ACU4375
Paracladius|CCDB22604-D09|Sofia Wiedenbrug||BOLD:ACR9234
Paracladius|CCDB22604-D07|Sofia Wiedenbrug||BOLD:ACR9234
Paracladius|CCDB22604-D06|Sofia Wiedenbrug||BOLD:ACR9234
Paracladius|CCDB22604-C09|Sofia Wiedenbrug||BOLD:ACR9234
Paracladius|CCDB21606-D04|Sofia Wiedenbrug||BOLD:ACR9234
Trichotanypus posticalis|ZMUO.025882|Lauri Paasivirta||BOLD:ADF1264
Parakiefferiella sp. 2SW|CCDB22605-B01|Sofia Wiedenbrug||BOLD:ACT9741
Parakiefferiella sp. 2SW|CCDB22605-B02|Sofia Wiedenbrug||BOLD:ACT9741
Lasiodiamesa armata|ZMUO.026337|Lauri Paasivirta||BOLD:ADF1674
Lasiodiamesa gracilis|ZMUO.025121|Lauri Paasivirta||BOLD:ADA9239
Lasiodiamesa gracilis|ZMUO.025120|Lauri Paasivirta||BOLD:ADA9239
Lasiodiamesa sphagnicola|ZMUO.026374|Marko Mutanen||BOLD:AEJ6063
Lasiodiamesa sphagnicola|ZSM-DIP-33134-C03|Caroline Chimeno|BIN Taxonomy Match (Jan 2022)|B...
Lasiodiamesa nr. sphagnicola|ZMUO.025303|Lauri Paasivirta||BOLD:AEJ6064
Lasiodiamesa sphagnicola|ZSM-DIP-33135-C05|Caroline Chimeno|BIN Taxonomy Match (Jan 2022)|B...
Lasiodiamesa sphagnicola|ZSM-DIP-33130-E04|Caroline Chimeno|BIN Taxonomy Match (Jan 2022)|B...
Lasiodiamesa sphagnicola|ZSM-DIP-33134-C04|Caroline Chimeno|BIN Taxonomy Match (Jan 2022)|B...
Lasiodiamesa sphagnicola|ZSM-DIP-33134-C02|Caroline Chimeno|BIN Taxonomy Match (Jan 2022)|B...
Lasiodiamesa sphagnicola|ZSM-DIP-33149-A12|Caroline Chimeno|BIN Taxonomy Match (Jan 2022)|B...
Lasiodiamesa sphagnicola|ZSM-DIP-33135-C06|Caroline Chimeno|BIN Taxonomy Match (Jan 2022)|B...
Lasiodiamesa sphagnicola|Finnmark359|Elisabeth Stur||BOLD:AAW9948
Pseudodiamesa nivosa|ZMUO.025948|Lauri Paasivirta||BOLD:AAF6936
Pseudodiamesa nivosa|ZMUO.025947|Lauri Paasivirta||BOLD:AAF6936
Pseudodiamesa nivosa|ZMUO.026047|Lauri Paasivirta||BOLD:AAF6936
Pseudodiamesa nivosa|ZMUO.026046|Lauri Paasivirta||BOLD:AAF6936
Pseudodiamesa nivosa|ATNA94|Elisabeth Stur||BOLD:AAF6936
Pseudodiamesa nivosa|ATNA104|Elisabeth Stur||BOLD:AAF6936
Pseudodiamesa nivosa|ATNA61|Elisabeth Stur||BOLD:AAF6936
Pseudodiamesa branickii|CH-OSF148|Elisabeth Stur||BOLD:AAD0382
Pseudodiamesa branickii|ATNA166|Elisabeth Stur||BOLD:AAD0382
Pseudodiamesa branickii|ATNA117|Elisabeth Stur||BOLD:AAD0382
Pseudodiamesa branickii|ATNA559|Elisabeth Stur||BOLD:AAD0382
Pseudodiamesa branickii|BIOUG17022-D08|Kate Perez|BIN Taxonomy Match|BOLD:AAD0382
Pseudodiamesa branickii|ATNA123|Elisabeth Stur||BOLD:AAD0382
Pseudodiamesa branickii|ATNA173|Elisabeth Stur||BOLD:AAD0382
Pseudodiamesa branickii|ATNA118|Elisabeth Stur||BOLD:AAD0382
Pseudodiamesa branickii|ATNA116|Elisabeth Stur||BOLD:AAD0382
Pseudodiamesa branickii|BIOUG15087-A06|Kate Perez|BIN Taxonomy Match|BOLD:AAD0382
Pseudodiamesa branickii|ATNA105|Elisabeth Stur||BOLD:AAD0382
Pseudodiamesa branickii|ATNA95|Elisabeth Stur||BOLD:AAD0382
Pseudodiamesa branickii|BC-ZSM-DIP-22488-C02|Sofia Wiedenbrug|Morphology|BOLD:ACX2423
Chironomidae|CCDB24037-D01||BIN Taxonomy Match
Pseudodiamesa branickii|ZMUO.025862|Lauri Paasivirta||BOLD:AEE1136
Pseudodiamesa branickii|Finnmark560|Elisabeth Stur||BOLD:AEE1136
Pseudodiamesa|EBAI-Ch186|Elisabeth Stur|morphology|BOLD:AAQ3206
Pseudodiamesa|EBAI-Ch184|Elisabeth Stur|morphology|BOLD:AAQ3206
Pseudodiamesa|EBAI-Ch236|Elisabeth Stur|morphology
Pseudodiamesa|EBAI-Ch183|Elisabeth Stur|morphology|BOLD:AAQ3206
Pseudodiamesa|EBAI-Ch185|Elisabeth Stur|morphology|BOLD:AAQ3206
Pseudodiamesa|EBAI-Ch067|Elisabeth Stur|morphology|BOLD:AAQ3206
Pseudodiamesa|EBAI-Ch182|Elisabeth Stur|morphology|BOLD:AAQ3206
Pseudodiamesa cf. pertinax|Finnmark539|Elisabeth Stur||BOLD:AAQ3206
Potthastia gaedii|ZMUO.025151|Lauri Paasivirta||BOLD:ADA6123
Potthastia gaedii|ZMUO.025152|Lauri Paasivirta||BOLD:ADA6123
Potthastia gaedii|NHRS-BYWS000000439|Yngve Brodin|morphology|BOLD:ADA6123
Potthastia gaedii|NHRS-BYWS000001116|Yngve Brodin|morphology|BOLD:ADA6123
Potthastia gaedii|NHRS-BYWS000000436|Yngve Brodin|morphology|BOLD:ADA6123
Potthastia gaedii|NHRS-BYWS000000397|Yngve Brodin|morphology|BOLD:ADA6123
Trichotanypus posticalis|Finnmark687|Elisabeth Stur||BOLD:AAE8611
Trichotanypus posticalis|ATNA70|Elisabeth Stur||BOLD:AAE8611
Trichotanypus posticalis|ATNA69|Elisabeth Stur||BOLD:AAE8611
Trichotanypus posticalis|ATNA68|Elisabeth Stur||BOLD:AAE8611
Trichotanypus posticalis|ATNA67|Elisabeth Stur||BOLD:AAE8611
Cricotopus cf. curtus|TRD-CH406|Elisabeth Stur|morphology|BOLD:AAG1012
Cricotopus|BIOUG43949-A08|Kate Perez|BIN Taxonomy Match below Phylum (Jan 2019)|BOLD:AAG...
Cricotopus cf. curtus|ATNA376|Elisabeth Stur||BOLD:AAG1012
Cricotopus cf. curtus|BIOUG15852-E12|Kate Perez|BIN Taxonomy Match|BOLD:AAG1012
Cricotopus cf. curtus|BIOUG16396-B09|Kate Perez|BIN Taxonomy Match|BOLD:AAG1012
Cricotopus cf. curtus|BIOUG16476-G06|Kate Perez|BIN Taxonomy Match|BOLD:AAG1012
Cricotopus cf. curtus|BIOUG15948-B02|Kate Perez|BIN Taxonomy Match|BOLD:AAG1012
Cricotopus cf. curtus|BIOUG16480-E08|Kate Perez|BIN Taxonomy Match|BOLD:AAG1012
Cricotopus cf. curtus|BIOUG16328-F02|Kate Perez|BIN Taxonomy Match|BOLD:AAG1012
Cricotopus cf. curtus|BIOUG16436-B01|Kate Perez|BIN Taxonomy Match|BOLD:AAG1012
Cricotopus cf. curtus|BIOUG16329-A07|Kate Perez|BIN Taxonomy Match|BOLD:AAG1012
Cricotopus cf. curtus|BIOUG16259-F02|Kate Perez|BIN Taxonomy Match|BOLD:AAG1012
Cricotopus cf. curtus|BIOUG16435-H09|Kate Perez|BIN Taxonomy Match|BOLD:AAG1012
Cricotopus cf. curtus|BIOUG16258-B05|Kate Perez|BIN Taxonomy Match|BOLD:AAG1012
Cricotopus cf. curtus|BIOUG16205-B05|Kate Perez|BIN Taxonomy Match|BOLD:AAG1012
Cricotopus cf. curtus|BIOUG15966-A08|Kate Perez|BIN Taxonomy Match|BOLD:AAG1012
Cricotopus cf. curtus|BIOUG15794-C10|Kate Perez|BIN Taxonomy Match|BOLD:AAG1012
Cricotopus cf. curtus|BIOUG16333-H09|Kate Perez|BIN Taxonomy Match|BOLD:AAG1012
Cricotopus cf. curtus|BIOUG16333-F05|Kate Perez|BIN Taxonomy Match|BOLD:AAG1012
Cricotopus cf. curtus|BIOUG16331-G01|Kate Perez|BIN Taxonomy Match|BOLD:AAG1012

Cricotopus cf. curtus|BIOUG16333-H09|Kate Perez|BIN Taxonomy Match|BOLD:AAG1012
Cricotopus cf. curtus|BIOUG16333-F05|Kate Perez|BIN Taxonomy Match|BOLD:AAG1012
Cricotopus cf. curtus|BIOUG16331-G01|Kate Perez|BIN Taxonomy Match|BOLD:AAG1012
Cricotopus cf. curtus|BIOUG16331-A07|Kate Perez|BIN Taxonomy Match|BOLD:AAG1012
Cricotopus cf. curtus|BIOUG16330-F01|Kate Perez|BIN Taxonomy Match|BOLD:AAG1012
Cricotopus cf. curtus|BIOUG15963-F01|Kate Perez|BIN Taxonomy Match|BOLD:AAG1012
Cricotopus cf. curtus|BIOUG15954-H10|Kate Perez|BIN Taxonomy Match|BOLD:AAG1012
Cricotopus cf. curtus|BIOUG15948-C09|Kate Perez|BIN Taxonomy Match|BOLD:AAG1012
Cricotopus cf. curtus|BIOUG16260-D11|Kate Perez|BIN Taxonomy Match|BOLD:AAG1012
Cricotopus cf. curtus|BIOUG16333-A03|Kate Perez|BIN Taxonomy Match|BOLD:AAG1012
Cricotopus cf. curtus|BIOUG16330-H10|Kate Perez|BIN Taxonomy Match|BOLD:AAG1012
Cricotopus cf. curtus|BIOUG16332-A05|Kate Perez|BIN Taxonomy Match|BOLD:AAG1012
Cricotopus cf. curtus|BIOUG16433-F10|Kate Perez|BIN Taxonomy Match|BOLD:AAG1012
Cricotopus cf. curtus|BIOUG16430-C05|Kate Perez|BIN Taxonomy Match|BOLD:AAG1012
Cricotopus cf. curtus|BIOUG16431-D10|Kate Perez|BIN Taxonomy Match|BOLD:AAG1012
Cricotopus cf. curtus|BIOUG16626-A02|Kate Perez|BIN Taxonomy Match|BOLD:AAG1012
Cricotopus cf. curtus|BIOUG16260-F08|Kate Perez|BIN Taxonomy Match|BOLD:AAG1012
Cricotopus cf. curtus|BIOUG15954-B12|Kate Perez|BIN Taxonomy Match|BOLD:AAG1012
Cricotopus cf. curtus|BIOUG16330-C01|Kate Perez|BIN Taxonomy Match|BOLD:AAG1012
Cricotopus cf. curtus|BIOUG16333-B08|Kate Perez|BIN Taxonomy Match|BOLD:AAG1012
Cricotopus cf. curtus|BIOUG16260-F07|Kate Perez|BIN Taxonomy Match|BOLD:AAG1012
Cricotopus cf. curtus|BIOUG16433-E09|Kate Perez|BIN Taxonomy Match|BOLD:AAG1012
Cricotopus cf. curtus|BIOUG16433-G12|Kate Perez|BIN Taxonomy Match|BOLD:AAG1012
Cricotopus cf. curtus|BIOUG16431-E03|Kate Perez|BIN Taxonomy Match|BOLD:AAG1012
Cricotopus cf. curtus|BIOUG16435-G02|Kate Perez|BIN Taxonomy Match|BOLD:AAG1012
Cricotopus cf. curtus|BIOUG16478-F04|Kate Perez|BIN Taxonomy Match|BOLD:AAG1012
Cricotopus cf. curtus|BIOUG16479-C07|Kate Perez|BIN Taxonomy Match|BOLD:AAG1012
Cricotopus cf. curtus|BIOUG16478-E10|Kate Perez|BIN Taxonomy Match|BOLD:AAG1012
Cricotopus cf. curtus|BIOUG16480-H01|Kate Perez|BIN Taxonomy Match|BOLD:AAG1012
Cricotopus cf. curtus|BIOUG16430-G08|Kate Perez|BIN Taxonomy Match|BOLD:AAG1012
Cricotopus cf. curtus|BIOUG16430-G09|Kate Perez|BIN Taxonomy Match|BOLD:AAG1012
Cricotopus cf. curtus|BIOUG16431-D06|Kate Perez|BIN Taxonomy Match|BOLD:AAG1012
Cricotopus cf. curtus|BIOUG16431-F07|Kate Perez|BIN Taxonomy Match|BOLD:AAG1012
Cricotopus cf. curtus|BIOUG16432-C02|Kate Perez|BIN Taxonomy Match|BOLD:AAG1012
Cricotopus cf. curtus|BIOUG16433-C07|Kate Perez|BIN Taxonomy Match|BOLD:AAG1012
Cricotopus cf. curtus|BIOUG16477-B09|Kate Perez|BIN Taxonomy Match|BOLD:AAG1012
Cricotopus cf. curtus|BIOUG16477-D12|Kate Perez|BIN Taxonomy Match|BOLD:AAG1012
Cricotopus cf. curtus|BIOUG16477-F05|Kate Perez|BIN Taxonomy Match|BOLD:AAG1012
Cricotopus cf. curtus|BIOUG16332-F03|Kate Perez|BIN Taxonomy Match|BOLD:AAG1012
Cricotopus cf. curtus|BIOUG16552-F08|Kate Perez|BIN Taxonomy Match|BOLD:AAG1012
Cricotopus cf. curtus|BIOUG16479-G06|Kate Perez|BIN Taxonomy Match|BOLD:AAG1012
Cricotopus cf. curtus|BIOUG16297-D10|Kate Perez|BIN Taxonomy Match|BOLD:AAG1012
Cricotopus cf. curtus|BIOUG16433-C05|Kate Perez|BIN Taxonomy Match|BOLD:AAG1012
Cricotopus cf. curtus|BIOUG15952-B06|Kate Perez|BIN Taxonomy Match|BOLD:AAG1012
Cricotopus cf. curtus|BIOUG16426-H04|Kate Perez|BIN Taxonomy Match|BOLD:AAG1012
Cricotopus cf. curtus|BIOUG16480-C02|Kate Perez|BIN Taxonomy Match|BOLD:AAG1012
Cricotopus cf. curtus|BIOUG16433-A04|Kate Perez|BIN Taxonomy Match|BOLD:AAG1012
Cricotopus cf. curtus|BIOUG16480-A05|Kate Perez|BIN Taxonomy Match|BOLD:AAG1012
Cricotopus cf. curtus|BIOUG16476-F11|Kate Perez|BIN Taxonomy Match|BOLD:AAG1012
Cricotopus cf. curtus|BIOUG16261-C11|Kate Perez|BIN Taxonomy Match|BOLD:AAG1012
Cricotopus cf. curtus|BIOUG16435-A07|Kate Perez|BIN Taxonomy Match|BOLD:AAG1012
Cricotopus cf. curtus|BIOUG16479-F06|Kate Perez|BIN Taxonomy Match|BOLD:AAG1012
Cricotopus cf. curtus|BIOUG16433-A09|Kate Perez|BIN Taxonomy Match|BOLD:AAG1012
Cricotopus cf. curtus|BIOUG16436-H03|Kate Perez|BIN Taxonomy Match|BOLD:AAG1012
Cricotopus cf. curtus|BIOUG16299-A01|Kate Perez|BIN Taxonomy Match|BOLD:AAG1012
Cricotopus cf. curtus|BIOUG16329-C12|Kate Perez|BIN Taxonomy Match|BOLD:AAG1012
Cricotopus cf. curtus|BIOUG16436-G10|Kate Perez|BIN Taxonomy Match|BOLD:AAG1012
Cricotopus cf. curtus|BIOUG16259-A01|Kate Perez|BIN Taxonomy Match|BOLD:AAG1012
Cricotopus cf. curtus|BIOUG16477-G02|Kate Perez|BIN Taxonomy Match|BOLD:AAG1012
Cricotopus cf. curtus|BIOUG16431-D07|Kate Perez|BIN Taxonomy Match|BOLD:AAG1012
Cricotopus cf. curtus|BIOUG16478-C12|Kate Perez|BIN Taxonomy Match|BOLD:AAG1012
Cricotopus cf. curtus|BIOUG16480-B04|Kate Perez|BIN Taxonomy Match|BOLD:AAG1012
Cricotopus cf. curtus|BIOUG16626-F06|Kate Perez|BIN Taxonomy Match|BOLD:AAG1012
Cricotopus cf. curtus|BIOUG16432-B09|Kate Perez|BIN Taxonomy Match|BOLD:AAG1012
Cricotopus cf. curtus|BIOUG16433-B06|Kate Perez|BIN Taxonomy Match|BOLD:AAG1012
Cricotopus cf. curtus|BIOUG16480-F10|Kate Perez|BIN Taxonomy Match|BOLD:AAG1012
Cricotopus cf. curtus|BIOUG16299-C03|Kate Perez|BIN Taxonomy Match|BOLD:AAG1012
Cricotopus cf. curtus|BIOUG15966-H11|Kate Perez|BIN Taxonomy Match|BOLD:AAG1012
Cricotopus cf. curtus|BIOUG15951-E01|Kate Perez|BIN Taxonomy Match|BOLD:AAG1012
Cricotopus cf. curtus|BIOUG16259-F03|Kate Perez|BIN Taxonomy Match|BOLD:AAG1012
Cricotopus cf. curtus|BIOUG16298-D02|Kate Perez|BIN Taxonomy Match|BOLD:AAG1012
Cricotopus cf. curtus|BIOUG16296-F02|Kate Perez|BIN Taxonomy Match|BOLD:AAG1012
Cricotopus cf. curtus|BIOUG16261-G11|Kate Perez|BIN Taxonomy Match|BOLD:AAG1012
Cricotopus cf. curtus|BIOUG16596-E06|Kate Perez|BIN Taxonomy Match|BOLD:AAG1012
Cricotopus cf. curtus|BIOUG16596-E02|Kate Perez|BIN Taxonomy Match|BOLD:AAG1012
Cricotopus cf. curtus|BIOUG16330-G01|Kate Perez|BIN Taxonomy Match|BOLD:AAG1012
Cricotopus cf. curtus|BIOUG15948-B09|Kate Perez|BIN Taxonomy Match|BOLD:AAG1012
Cricotopus cf. curtus|BIOUG15947-D09|Kate Perez|BIN Taxonomy Match|BOLD:AAG1012
Cricotopus cf. curtus|NO65|Elisabeth Stur||BOLD:AAG1012
Telmatogeton japonicus|NHRS-BYWS000001078|Yngve Brodin|morphology|BOLD:ACV6061
Telmatogeton japonicus|NHRS-BYWS000000379|Yngve Brodin|morphology|BOLD:ACV6061
Cricotopus|CCDB24039-A06|Sofia Wiedenbrug|BIN Taxonomy Match|BOLD:AAI6019
Cricotopus|ZSM34342-G04|Sofia Wiedenbrug|Morphology|BOLD:AAI6019
Cricotopus|CCDB24039-A05|Sofia Wiedenbrug|BIN Taxonomy Match|BOLD:AAI6019
Cricotopus sp. 15ES|ES283|Elisabeth Stur||BOLD:AAI6019
Hydrobaenus pilipes|ZMUO.024320|Lauri Paasivirta||BOLD:ADA5503
Hydrobaenus pilipes|ZMUO.024319|Lauri Paasivirta||BOLD:ADA5503
Hydrobaenus pilipes|ZMUO.029784|Marko Mutanen||BOLD:ADA5503
Cricotopus|BIOUG17094-B12|Valerie Levesque-Beaudin|BIN Taxonomy Match|BOLD:ACQ9036
Cricotopus|BIOUG17265-B03|Valerie Levesque-Beaudin|BIN Taxonomy Match|BOLD:ACQ9036
Cricotopus|BIOUG16268-D08|Valerie Levesque-Beaudin|Digital Morphology|BOLD:ACQ9036
Cricotopus annulator|NHRS-BYWS000000518|Yngve Brodin|morphology|BOLD:AAF2345
Cricotopus annulator|NHRS-BYWS000000261|Yngve Brodin|morphology|BOLD:AAF2345
Cricotopus annulator|NHRS-BYWS000000289|Yngve Brodin|morphology|BOLD:AAF2345
Cricotopus annulator|Finnmark232|Susan E. Gresens||BOLD:AAF2345
Cricotopus annulator|Finnmark231|Susan E. Gresens||BOLD:AAF2345
Cricotopus annulator|NO11|Elisabeth Stur||BOLD:AAF2345
Cricotopus annulator|NO10|Elisabeth Stur||BOLD:AAF2345
Cricotopus annulator|NO09|Elisabeth Stur||BOLD:AAF2345
Cricotopus annulator|ZMUO.024715|Lauri Paasivirta||BOLD:AAF2345
Cricotopus annulator|ZMUO.024714|Lauri Paasivirta||BOLD:AAF2345
Cricotopus annulator|NHRS-BYWS000000144|Yngve Brodin|morphology|BOLD:AAF2345
Cricotopus annulator|BIOUG43950-C06|Kate Perez|BIN Taxonomy Match below Phylum (Jan 2019)|B[illegible]

[illegible]
Cricotopus annulator|ZMUO.024714|Lauri Paasivirta||BOLD:AAF2345
Cricotopus annulator|NHRS-BYWS000000144|Yngve Brodin|morphology|BOLD:AAF2345
Cricotopus annulator|BIOUG43950-C06|Kate Perez|BIN Taxonomy Match below Phylum (Jan 2019)|B...
Cricotopus annulator|BIOUG16479-B07|Kate Perez|BIN Taxonomy Match|BOLD:AAF2345
Cricotopus annulator|Finnmark125|Elisabeth Stur||BOLD:AAF2345
Cricotopus annulator|NHRS-BYWS000000947|Yngve Brodin|morphology|BOLD:AAF2345
Cricotopus annulator|NHRS-BYWS000000490|Yngve Brodin|morphology|BOLD:AAF2345
Cricotopus annulator|BIOUG16597-G08|Kate Perez|BIN Taxonomy Match|BOLD:AAF2345
Cricotopus annulator|BIOUG16478-B07|Kate Perez|BIN Taxonomy Match|BOLD:AAF2345
Cricotopus annulator|BIOUG15967-E05|Kate Perez|BIN Taxonomy Match|BOLD:AAF2345
Cricotopus annulator|Finnmark230|Elisabeth Stur||BOLD:AAF2345
Cricotopus annulator|BIOUG16431-F11|Kate Perez|BIN Taxonomy Match|BOLD:AAF2345
Cricotopus annulator|BIOUG16426-H01|Kate Perez|BIN Taxonomy Match|BOLD:AAF2345
Cricotopus annulator|BIOUG16596-C06|Kate Perez|BIN Taxonomy Match|BOLD:AAF2345
Cricotopus annulator|BIOUG15951-A10|Kate Perez|BIN Taxonomy Match|BOLD:AAF2345
Cricotopus annulator|EJV-20110213|Lauri Paasivirta||BOLD:AAF2345
Potthastia|BIOUG15793-F04|Elisabeth Stur|morphology|BOLD:ACP9676
Potthastia|BIOUG15963-G04|Elisabeth Stur|morphology|BOLD:ACP9676
Potthastia|BIOUG15798-G07|Elisabeth Stur|morphology|BOLD:ACP9676
Potthastia|BIOUG15798-C10|Elisabeth Stur|morphology|BOLD:ACP9676
Potthastia|BIOUG15948-A06|Elisabeth Stur|morphology|BOLD:ACP9676
Potthastia|BIOUG16258-E02|Elisabeth Stur|morphology|BOLD:ACP9676
Potthastia|BIOUG15949-C04|Elisabeth Stur|morphology|BOLD:ACP9676
Potthastia pastoris|ZMUO.024603|Lauri Paasivirta||BOLD:ADA5922
Potthastia pastoris|ZMUO.024602|Lauri Paasivirta||BOLD:ADA5922
Potthastia longimanus|ZMUO.025153|Lauri Paasivirta||BOLD:ADA8077
Potthastia longimanus|ZMUO.025154|Lauri Paasivirta||BOLD:ADA8077
Potthastia longimanus|NHRS-BYWS000000520|Yngve Brodin|morphology|BOLD:ADA8077
Potthastia longimanus|NHRS-BYWS000000519|Yngve Brodin|morphology|BOLD:ADA8077
Potthastia longimanus|NHRS-BYWS000000350|Yngve Brodin|morphology|BOLD:ADA8077
Potthastia longimanus|ATNA185|Elisabeth Stur||BOLD:AAG2529
Potthastia longimanus|BIOUG43949-C06|Kate Perez|BIN Taxonomy Match below Phylum (Jan 2019)|...
Potthastia longimanus|Finnmark252|Elisabeth Stur||BOLD:ADZ9265
Potthastia|Finnmark763|Torbjorn Ekrem||BOLD:ACD1938
Potthastia|Finnmark755|Torbjorn Ekrem||BOLD:ACD1938
Protanypus caudatus|TRD-CH148|Torbjorn Ekrem|morphology|BOLD:ACO4299
Protanypus caudatus|ZMUO.029812|Marko Mutanen||BOLD:ACO4299
Protanypus caudatus|ZMUO.029819|Marko Mutanen||BOLD:ACO4299
Protanypus caudatus|TRD-CH125|Torbjorn Ekrem||BOLD:ACO4299
Protanypus caudatus|TRD-CH123|Torbjorn Ekrem|morphology|BOLD:ACO4299
Protanypus morio|KY225352
Protanypus sp. 1ES|TRD-CH282|Elisabeth Stur|morphology|BOLD:ACX5699
Protanypus sp. 1ES|TRD-CH260|Elisabeth Stur|morphology|BOLD:ACX5699
Protanypus morio|TRD-CH261|Elisabeth Stur||BOLD:AAV2652
Protanypus morio|TRD-CH149|Elisabeth Stur|morphology|BOLD:AAV2652
Protanypus morio|Finnmark523|Elisabeth Stur||BOLD:AAV2652
Protanypus morio|ZMUO.024302|Lauri Paasivirta||BOLD:AAV2652
Protanypus morio|ZMUO.024301|Lauri Paasivirta||BOLD:AAV2652
Protanypus morio|Finnmark505|Elisabeth Stur||BOLD:AAV2652
Protanypus morio|Finnmark645|Elisabeth Stur||BOLD:AAV2652
Chironomidae|ZSM34343-F01|Jerome Moriniere|BIN Taxonomy Match|BOLD:ADC5035
Chironomidae|24333-C12|Mikko J Tiusanen|Tree based identification|BOLD:ACT3435
Chironomidae|24332-F10|Mikko J Tiusanen|Tree based identification|BOLD:ACT3435
Chironomidae|24424-A07|Mikko J Tiusanen|Tree based identification|BOLD:ACT3435
Pseudokiefferiella parva|CCDB21606-D09|Sofia Wiedenbrug||BOLD:ACT5785
Pseudokiefferiella|CCDB24038-E07|Sofia Wiedenbrug|BIN Taxonomy Match|BOLD:ACU5487
Pseudokiefferiella|BC-ZSM-DIP-22491-E06|Dieter Doczkal||BOLD:ACU5487
Pseudokiefferiella parva|ATNA378|Elisabeth Stur||BOLD:AAH8220
Pseudokiefferiella parva|ATNA113|Elisabeth Stur||BOLD:AAH8220
Pseudokiefferiella parva|ZMUO.025987|Lauri Paasivirta||BOLD:ADF0056
Pseudokiefferiella|Finnmark326|Elisabeth Stur||BOLD:AAV2899
Pseudokiefferiella parva|ZMUO.025897|Lauri Paasivirta||BOLD:ACC9871
Diamesa arctica|ATNA401|Elisabeth Stur||BOLD:AAD7062
Diamesa steinboecki|OM867251
Diamesa steinboecki|OM867252
Diamesa steinboecki|OM867250
Diamesa starmachi|Finnmark32|Elisabeth Stur||BOLD:AAM5968
Diamesa aberrata|ATNA416|Elisabeth Stur||BOLD:AAB1737
Diamesa aberrata|ATNA403|Elisabeth Stur||BOLD:AAB1737
Diamesa aberrata|ATNA402|Elisabeth Stur||BOLD:AAB1737
Diamesa aberrata|ATNA405|Elisabeth Stur||BOLD:AAB1737
Diamesa aberrata|ATNA417|Elisabeth Stur||BOLD:AAB1737
Diamesa aberrata|ATNA404|Elisabeth Stur||BOLD:AAB1737
Diamesa aberrata|CCDB24228-C09|Sofia Wiedenbrug|BIN Taxonomy Match|BOLD:AAB1737
Diamesa aberrata|ATNA99|Elisabeth Stur||BOLD:AAB1737
Diamesa saetheri|EBAI-Ch126|Elisabeth Stur|BOLD Identification Engine|BOLD:AAE7631
Diamesa saetheri|ATNA124|Elisabeth Stur||BOLD:AAE7631
Diamesa saetheri|ATNA92|Elisabeth Stur||BOLD:AAE7631
Diamesa saetheri|EBAI-Ch045|Elisabeth Stur|morphology|BOLD:AAE7631
Diamesa saetheri|ATNA107|Elisabeth Stur||BOLD:AAE7631
Diamesa serratosioi|EBAI-Ch251|Elisabeth Stur|morphology
Diamesa serratosioi|EBAI-Ch228|Elisabeth Stur||BOLD:AAD4446
Diamesa serratosioi|ATNA429|Elisabeth Stur||BOLD:AAD4446
Diamesa serratosioi|ATNA157|Elisabeth Stur||BOLD:AAD4446
Diamesa serratosioi|EBAI-Ch052|Elisabeth Stur|morphology|BOLD:AAD4446
Diamesa serratosioi|ATNA125|Elisabeth Stur||BOLD:AAD4446
Diamesa serratosioi|ATNA108|Elisabeth Stur||BOLD:AAD4446
Diamesa serratosioi|ATNA82|Elisabeth Stur||BOLD:AAD4446
Diamesa serratosioi|ATNA172|Elisabeth Stur||BOLD:AAD4446
Diamesa serratosioi|ATNA174|Elisabeth Stur||BOLD:AAD4446
Diamesa serratosioi|ATNA106|Elisabeth Stur||BOLD:AAD4446
Diamesa serratosioi|ATNA84|Elisabeth Stur||BOLD:AAD4446
Diamesa insignipes|BIOUG16894-B06|Jerome Moriniere|BOLD ID Engine Manual|BOLD:ACR0046
Diamesa insignipes|BIOUG16738-B03|Jerome Moriniere|BOLD ID Engine Manual|BOLD:ACR0046
Diamesa insignipes|BIOUG16783-D01|Jerome Moriniere|BOLD ID Engine Manual|BOLD:ACR0046
Diamesa insignipes|BIOUG16892-H04|Jerome Moriniere|BOLD ID Engine Manual|BOLD:ACR0046
Diamesa insignipes|BIOUG16721-E11|Jerome Moriniere|BOLD ID Engine Manual|BOLD:ACR0046
Diamesa insignipes|BIOUG16783-D12|Jerome Moriniere|BOLD ID Engine Manual|BOLD:ACR0046
Diamesa insignipes|BIOUG16892-G07|Jerome Moriniere|BOLD ID Engine Manual|BOLD:ACR0046
Diamesa insignipes|BIOUG16775-F01|Jerome Moriniere|BOLD ID Engine Manual|BOLD:ACR0046
Diamesa insignipes|BIOUG16785-F06|Jerome Moriniere|BOLD ID Engine Manual|BOLD:ACR0046
Diamesa insignipes|BIOUG16779-A05|Jerome Moriniere|BOLD ID Engine Manual|BOLD:ACR0046
[illegible]

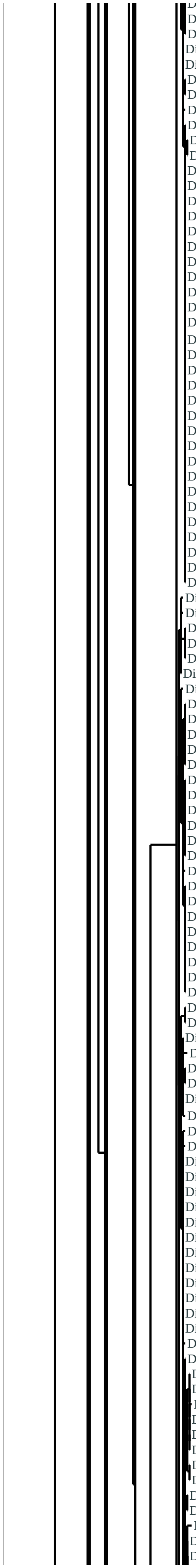

Diamesa insignipes|BIOUG16775-F01|Jerome Moriniere|BOLD ID Engine Manual|BOLD:ACR0046
Diamesa insignipes|BIOUG16785-F06|Jerome Moriniere|BOLD ID Engine Manual|BOLD:ACR0046
Diamesa insignipes|BIOUG16779-A05|Jerome Moriniere|BOLD ID Engine Manual|BOLD:ACR0046
Diamesa insignipes|BIOUG16740-D08|Jerome Moriniere|BOLD ID Engine Manual|BOLD:ACR0046
Diamesa insignipes|BIOUG16894-B03|Jerome Moriniere|BOLD ID Engine Manual|BOLD:ACR0046
Diamesa insignipes|BIOUG16855-H06|Jerome Moriniere|BOLD ID Engine Manual|BOLD:ACR0046
Diamesa insignipes|BIOUG16779-F06|Jerome Moriniere|BOLD ID Engine Manual|BOLD:ACR0046
Diamesa insignipes|BIOUG16855-D02|Jerome Moriniere|BOLD ID Engine Manual|BOLD:ACR0046
Diamesa insignipes|BIOUG16720-E08|Jerome Moriniere|BOLD ID Engine Manual|BOLD:ACR0046
Diamesa insignipes|BIOUG16785-C08|Jerome Moriniere|BOLD ID Engine Manual|BOLD:ACR0046
Diamesa insignipes|BIOUG16775-D07|Jerome Moriniere|BOLD ID Engine Manual|BOLD:ACR0046
Diamesa insignipes|BIOUG16855-D06|Jerome Moriniere|BOLD ID Engine Manual|BOLD:ACR0046
Diamesa insignipes|BIOUG16720-D04|Jerome Moriniere|BOLD ID Engine Manual|BOLD:ACR0046
Diamesa insignipes|BIOUG16856-H06|Jerome Moriniere|BOLD ID Engine Manual|BOLD:ACR0046
Diamesa insignipes|BIOUG16893-H04|Jerome Moriniere|BOLD ID Engine Manual|BOLD:ACR0046
Diamesa insignipes|BIOUG16783-D04|Jerome Moriniere|BOLD ID Engine Manual|BOLD:ACR0046
Diamesa insignipes|BIOUG16893-H02|Jerome Moriniere|BOLD ID Engine Manual|BOLD:ACR0046
Diamesa insignipes|BIOUG16785-H01|Jerome Moriniere|BOLD ID Engine Manual|BOLD:ACR0046
Diamesa insignipes|BIOUG16892-B02|Jerome Moriniere|BOLD ID Engine Manual|BOLD:ACR0046
Diamesa insignipes|BIOUG16855-D07|Jerome Moriniere|BOLD ID Engine Manual|BOLD:ACR0046
Diamesa insignipes|BIOUG16893-D01|Jerome Moriniere|BOLD ID Engine Manual|BOLD:ACR0046
Diamesa insignipes|BIOUG16854-H11|Jerome Moriniere|BOLD ID Engine Manual|BOLD:ACR0046
Diamesa insignipes|BIOUG16720-B05|Jerome Moriniere|BOLD ID Engine Manual|BOLD:ACR0046
Diamesa insignipes|BIOUG16778-C03|Jerome Moriniere|BOLD ID Engine Manual|BOLD:ACR0046
Diamesa insignipes|BIOUG16855-A05|Jerome Moriniere|BOLD ID Engine Manual|BOLD:ACR0046
Diamesa insignipes|BIOUG16859-A06|Jerome Moriniere|BOLD ID Engine Manual|BOLD:ACR0046
Diamesa insignipes|BIOUG16854-E03|Jerome Moriniere|BOLD ID Engine Manual|BOLD:ACR0046
Diamesa insignipes|BIOUG16778-E07|Jerome Moriniere|BOLD ID Engine Manual|BOLD:ACR0046
Diamesa insignipes|BIOUG16859-F02|Jerome Moriniere|BOLD ID Engine Manual|BOLD:ACR0046
Diamesa insignipes|BIOUG16856-H07|Jerome Moriniere|BOLD ID Engine Manual|BOLD:ACR0046
Diamesa insignipes|BIOUG16854-E09|Jerome Moriniere|BOLD ID Engine Manual|BOLD:ACR0046
Diamesa insignipes|BIOUG16738-D02|Jerome Moriniere|BOLD ID Engine Manual|BOLD:ACR0046
Diamesa insignipes|BIOUG16721-F07|Jerome Moriniere|BOLD ID Engine Manual|BOLD:ACR0046
Diamesa insignipes|BIOUG16720-C07|Jerome Moriniere|BOLD ID Engine Manual|BOLD:ACR0046
Diamesa insignipes|BIOUG16782-H10|Jerome Moriniere|BOLD ID Engine Manual|BOLD:ACR0046
Diamesa insignipes|BIOUG16779-B09|Jerome Moriniere|BOLD ID Engine Manual|BOLD:ACR0046
Diamesa insignipes|BIOUG16739-C08|Jerome Moriniere|BOLD ID Engine Manual|BOLD:ACR0046
Diamesa insignipes|BIOUG16739-B07|Jerome Moriniere|BOLD ID Engine Manual|BOLD:ACR0046
Diamesa insignipes|BIOUG16739-A05|Jerome Moriniere|BOLD ID Engine Manual|BOLD:ACR0046
Diamesa insignipes|BIOUG16894-A03|Jerome Moriniere|BOLD ID Engine Manual|BOLD:ACR0046
Diamesa insignipes|BIOUG16778-A07|Jerome Moriniere|BOLD ID Engine Manual|BOLD:ACR0046
Diamesa insignipes|BIOUG16893-F08|Jerome Moriniere|BOLD ID Engine Manual|BOLD:ACR0046
Diamesa insignipes|BIOUG16893-E12|Jerome Moriniere|BOLD ID Engine Manual|BOLD:ACR0046
Diamesa insignipes|BIOUG16857-E03|Jerome Moriniere|BOLD ID Engine Manual|BOLD:ACR0046
Diamesa insignipes|BIOUG16740-C12|Jerome Moriniere|BOLD ID Engine Manual|BOLD:ACR0046
Diamesa insignipes|BIOUG16738-B04|Jerome Moriniere|BOLD ID Engine Manual|BOLD:ACR0046
Diamesa insignipes|BIOUG16857-D10|Jerome Moriniere|BOLD ID Engine Manual|BOLD:ACR0046
Diamesa insignipes|BIOUG16783-D10|Jerome Moriniere|BOLD ID Engine Manual|BOLD:ACR0046
Diamesa insignipes|BIOUG16785-B06|Jerome Moriniere|BOLD ID Engine Manual|BOLD:ACR0046
Diamesa insignipes|BIOUG16720-D08|Jerome Moriniere|BOLD ID Engine Manual|BOLD:ACR0046
Diamesa insignipes|BIOUG16779-F12|Jerome Moriniere|BOLD ID Engine Manual|BOLD:ACR0046
Diamesa insignipes|BIOUG16784-H02|Jerome Moriniere|BOLD ID Engine Manual|BOLD:ACR0046
Diamesa insignipes|BIOUG16721-E08|Jerome Moriniere|BOLD ID Engine Manual|BOLD:ACR0046
Diamesa insignipes|BIOUG16720-C01|Jerome Moriniere|BOLD ID Engine Manual|BOLD:ACR0046
Diamesa insignipes|BIOUG16721-A04|Jerome Moriniere|BOLD ID Engine Manual|BOLD:ACR0046
Diamesa insignipes|BIOUG16784-H05|Jerome Moriniere|BOLD ID Engine Manual|BOLD:ACR0046
Diamesa insignipes|BIOUG16779-D10|Jerome Moriniere|BOLD ID Engine Manual|BOLD:ACR0046
Diamesa insignipes|BIOUG16720-H09|Jerome Moriniere|BOLD ID Engine Manual|BOLD:ACR0046
Diamesa insignipes|BIOUG16853-B11|Jerome Moriniere|BOLD ID Engine Manual|BOLD:ACR0046
Diamesa insignipes|BIOUG16782-A05|Jerome Moriniere|BOLD ID Engine Manual|BOLD:ACR0046
Diamesa insignipes|BIOUG16779-C02|Jerome Moriniere|BOLD ID Engine Manual|BOLD:ACR0046
Diamesa insignipes|BIOUG16859-B02|Jerome Moriniere|BOLD ID Engine Manual|BOLD:ACR0046
Diamesa insignipes|BIOUG16777-G02|Jerome Moriniere|BOLD ID Engine Manual|BOLD:ACR0046
Diamesa insignipes|BIOUG16720-E10|Jerome Moriniere|BOLD ID Engine Manual|BOLD:ACR0046
Diamesa insignipes|BIOUG16785-C09|Jerome Moriniere|BOLD ID Engine Manual|BOLD:ACR0046
Diamesa insignipes|BIOUG16784-D01|Jerome Moriniere|BOLD ID Engine Manual|BOLD:ACR0046
Diamesa insignipes|BIOUG16892-B08|Jerome Moriniere|BOLD ID Engine Manual|BOLD:ACR0046
Diamesa insignipes|BIOUG16779-F04|Jerome Moriniere|BOLD ID Engine Manual|BOLD:ACR0046
Diamesa insignipes|BIOUG16853-E06|Jerome Moriniere|BOLD ID Engine Manual|BOLD:ACR0046
Diamesa insignipes|BIOUG16720-H08|Jerome Moriniere|BOLD ID Engine Manual|BOLD:ACR0046
Diamesa insignipes|BIOUG16740-E01|Jerome Moriniere|BOLD ID Engine Manual|BOLD:ACR0046
Diamesa insignipes|BIOUG16720-G10|Jerome Moriniere|BOLD ID Engine Manual|BOLD:ACR0046
Diamesa insignipes|BIOUG16782-D05|Jerome Moriniere|BOLD ID Engine Manual|BOLD:ACR0046
Diamesa insignipes|BIOUG16779-G02|Jerome Moriniere|BOLD ID Engine Manual|BOLD:ACR0046
Diamesa insignipes|BIOUG16857-E12|Jerome Moriniere|BOLD ID Engine Manual|BOLD:ACR0046
Diamesa insignipes|BIOUG16892-C02|Jerome Moriniere|BOLD ID Engine Manual|BOLD:ACR0046
Diamesa insignipes|BIOUG16856-H08|Jerome Moriniere|BOLD ID Engine Manual|BOLD:ACR0046
Diamesa insignipes|BIOUG16856-B02|Jerome Moriniere|BOLD ID Engine Manual|BOLD:ACR0046
Diamesa insignipes|BIOUG16775-D05|Jerome Moriniere|BOLD ID Engine Manual|BOLD:ACR0046
Diamesa insignipes|BIOUG16856-G08|Jerome Moriniere|BOLD ID Engine Manual|BOLD:ACR0046
Diamesa insignipes|BIOUG16775-B03|Jerome Moriniere|BOLD ID Engine Manual|BOLD:ACR0046
Diamesa insignipes|BIOUG16775-C02|Jerome Moriniere|BOLD ID Engine Manual|BOLD:ACR0046
Diamesa insignipes|BIOUG16778-E10|Jerome Moriniere|BOLD ID Engine Manual|BOLD:ACR0046
Diamesa insignipes|BIOUG16776-C08|Jerome Moriniere|BOLD ID Engine Manual|BOLD:ACR0046
Diamesa insignipes|BIOUG16740-G07|Jerome Moriniere|BOLD ID Engine Manual|BOLD:ACR0046
Diamesa insignipes|BIOUG16721-A10|Jerome Moriniere|BOLD ID Engine Manual|BOLD:ACR0046
Diamesa insignipes|BIOUG16783-A12|Jerome Moriniere|BOLD ID Engine Manual|BOLD:ACR0046
Diamesa insignipes|BIOUG16783-F09|Jerome Moriniere|BOLD ID Engine Manual|BOLD:ACR0046
Diamesa insignipes|BIOUG16783-C08|Jerome Moriniere|BOLD ID Engine Manual|BOLD:ACR0046
Diamesa insignipes|BIOUG16720-A05|Jerome Moriniere|BOLD ID Engine Manual|BOLD:ACR0046
Diamesa insignipes|BIOUG16859-E09|Jerome Moriniere|BOLD ID Engine Manual|BOLD:ACR0046
Diamesa insignipes|BIOUG16721-D07|Jerome Moriniere|BOLD ID Engine Manual|BOLD:ACR0046
Diamesa insignipes|BIOUG16775-A05|Jerome Moriniere|BOLD ID Engine Manual|BOLD:ACR0046
Diamesa insignipes|BIOUG16721-C05|Jerome Moriniere|BOLD ID Engine Manual|BOLD:ACR0046
Diamesa insignipes|BIOUG16855-B11|Jerome Moriniere|BOLD ID Engine Manual|BOLD:ACR0046
Diamesa insignipes|BIOUG16721-B08|Jerome Moriniere|BOLD ID Engine Manual|BOLD:ACR0046
Diamesa insignipes|BIOUG16854-B01|Jerome Moriniere|BOLD ID Engine Manual|BOLD:ACR0046
Diamesa insignipes|BIOUG16779-E03|Jerome Moriniere|BOLD ID Engine Manual|BOLD:ACR0046
Diamesa insignipes|BIOUG16784-H03|Jerome Moriniere|BOLD ID Engine Manual|BOLD:ACR0046
Diamesa insignipes|BIOUG16779-C03|Jerome Moriniere|BOLD ID Engine Manual|BOLD:ACR0046
Diamesa insignipes|BIOUG16853-A03|Jerome Moriniere|BOLD ID Engine Manual|BOLD:ACR0046
Diamesa insignipes|BIOUG16892-B01|Jerome Moriniere|BOLD ID Engine Manual|BOLD:ACR0046
Diamesa insignipes|BIOUG16854-E08|Jerome Moriniere|BOLD ID Engine Manual|BOLD:ACR0046

Diamesa insignipes|BIOUG16853-A03|Jerome Moriniere|BOLD ID Engine Manual|BOLD:ACR0046
Diamesa insignipes|BIOUG16892-B01|Jerome Moriniere|BOLD ID Engine Manual|BOLD:ACR0046
Diamesa insignipes|BIOUG16854-E08|Jerome Moriniere|BOLD ID Engine Manual|BOLD:ACR0046
Diamesa insignipes|BIOUG16777-D02|Jerome Moriniere|BOLD ID Engine Manual|BOLD:ACR0046
Diamesa insignipes|BIOUG16778-E04|Jerome Moriniere|BOLD ID Engine Manual|BOLD:ACR0046
Diamesa insignipes|BIOUG16720-G09|Jerome Moriniere|BOLD ID Engine Manual|BOLD:ACR0046
Diamesa insignipes|BIOUG16720-G07|Jerome Moriniere|BOLD ID Engine Manual|BOLD:ACR0046
Diamesa insignipes|BIOUG16892-A07|Jerome Moriniere|BOLD ID Engine Manual|BOLD:ACR0046
Diamesa insignipes|BIOUG16783-D06|Jerome Moriniere|BOLD ID Engine Manual|BOLD:ACR0046
Diamesa insignipes|BIOUG16894-B07|Jerome Moriniere|BOLD ID Engine Manual|BOLD:ACR0046
Diamesa insignipes|BIOUG16856-D01|Jerome Moriniere|BOLD ID Engine Manual|BOLD:ACR0046
Diamesa insignipes|BIOUG16857-H05|Jerome Moriniere|BOLD ID Engine Manual|BOLD:ACR0046
Diamesa insignipes|BIOUG16853-G11|Jerome Moriniere|BOLD ID Engine Manual|BOLD:ACR0046
Diamesa insignipes|BIOUG16783-A08|Jerome Moriniere|BOLD ID Engine Manual|BOLD:ACR0046
Diamesa insignipes|BIOUG16856-F08|Jerome Moriniere|BOLD ID Engine Manual|BOLD:ACR0046
Diamesa insignipes|BIOUG16721-G04|Jerome Moriniere|BOLD ID Engine Manual|BOLD:ACR0046
Diamesa insignipes|BIOUG16893-B05|Jerome Moriniere|BOLD ID Engine Manual|BOLD:ACR0046
Diamesa insignipes|BIOUG16720-B07|Jerome Moriniere|BOLD ID Engine Manual|BOLD:ACR0046
Diamesa insignipes|BIOUG16779-H07|Jerome Moriniere|BOLD ID Engine Manual|BOLD:ACR0046
Diamesa insignipes|BIOUG16783-C02|Jerome Moriniere|BOLD ID Engine Manual|BOLD:ACR0046
Diamesa insignipes|BIOUG16776-G08|Jerome Moriniere|BOLD ID Engine Manual|BOLD:ACR0046
Diamesa insignipes|BIOUG16893-A11|Jerome Moriniere|BOLD ID Engine Manual|BOLD:ACR0046
Diamesa insignipes|BIOUG16782-C12|Jerome Moriniere|BOLD ID Engine Manual|BOLD:ACR0046
Diamesa insignipes|BIOUG16721-B09|Jerome Moriniere|BOLD ID Engine Manual|BOLD:ACR0046
Diamesa insignipes|BIOUG16740-H02|Jerome Moriniere|BOLD ID Engine Manual|BOLD:ACR0046
Diamesa insignipes|BIOUG16856-D08|Jerome Moriniere|BOLD ID Engine Manual|BOLD:ACR0046
Diamesa insignipes|BIOUG16892-D12|Jerome Moriniere|BOLD ID Engine Manual|BOLD:ACR0046
Diamesa insignipes|BIOUG16782-C06|Jerome Moriniere|BOLD ID Engine Manual|BOLD:ACR0046
Diamesa insignipes|BIOUG16779-B03|Jerome Moriniere|BOLD ID Engine Manual|BOLD:ACR0046
Diamesa insignipes|BIOUG16855-E12|Jerome Moriniere|BOLD ID Engine Manual|BOLD:ACR0046
Diamesa insignipes|BIOUG16776-B01|Jerome Moriniere|BOLD ID Engine Manual|BOLD:ACR0046
Diamesa insignipes|BIOUG16777-C01|Jerome Moriniere|BOLD ID Engine Manual|BOLD:ACR0046
Diamesa insignipes|BIOUG16893-H07|Jerome Moriniere|BOLD ID Engine Manual|BOLD:ACR0046
Diamesa insignipes|BIOUG16777-C06|Jerome Moriniere|BOLD ID Engine Manual|BOLD:ACR0046
Diamesa insignipes|BIOUG16856-A06|Jerome Moriniere|BOLD ID Engine Manual|BOLD:ACR0046
Diamesa insignipes|BIOUG16779-F07|Jerome Moriniere|BOLD ID Engine Manual|BOLD:ACR0046
Diamesa insignipes|BIOUG16720-H10|Jerome Moriniere|BOLD ID Engine Manual|BOLD:ACR0046
Diamesa insignipes|BIOUG16778-F09|Jerome Moriniere|BOLD ID Engine Manual|BOLD:ACR0046
Diamesa insignipes|BIOUG16739-D01|Jerome Moriniere|BOLD ID Engine Manual|BOLD:ACR0046
Diamesa insignipes|BIOUG16782-B10|Jerome Moriniere|BOLD ID Engine Manual|BOLD:ACR0046
Diamesa insignipes|BIOUG16778-H03|Jerome Moriniere|BOLD ID Engine Manual|BOLD:ACR0046
Diamesa insignipes|BIOUG16782-A01|Jerome Moriniere|BOLD ID Engine Manual|BOLD:ACR0046
Diamesa insignipes|BIOUG16892-A08|Jerome Moriniere|BOLD ID Engine Manual|BOLD:ACR0046
Diamesa insignipes|BIOUG16859-G03|Jerome Moriniere|BOLD ID Engine Manual|BOLD:ACR0046
Diamesa insignipes|BIOUG16892-A05|Jerome Moriniere|BOLD ID Engine Manual|BOLD:ACR0046
Diamesa insignipes|BIOUG16853-D03|Jerome Moriniere|BOLD ID Engine Manual|BOLD:ACR0046
Diamesa insignipes|BIOUG16721-D09|Jerome Moriniere|BOLD ID Engine Manual|BOLD:ACR0046
Diamesa insignipes|BIOUG16778-F05|Jerome Moriniere|BOLD ID Engine Manual|BOLD:ACR0046
Diamesa insignipes|BIOUG16783-F07|Jerome Moriniere|BOLD ID Engine Manual|BOLD:ACR0046
Diamesa insignipes|BIOUG16859-D11|Jerome Moriniere|BOLD ID Engine Manual|BOLD:ACR0046
Diamesa insignipes|BIOUG16720-H04|Jerome Moriniere|BOLD ID Engine Manual|BOLD:ACR0046
Diamesa insignipes|BIOUG16853-A10|Jerome Moriniere|BOLD ID Engine Manual|BOLD:ACR0046
Diamesa insignipes|BIOUG16853-B06|Jerome Moriniere|BOLD ID Engine Manual|BOLD:ACR0046
Diamesa insignipes|BIOUG16777-F05|Jerome Moriniere|BOLD ID Engine Manual|BOLD:ACR0046
Diamesa insignipes|BIOUG16776-G12|Jerome Moriniere|BOLD ID Engine Manual|BOLD:ACR0046
Diamesa insignipes|BIOUG16775-E06|Jerome Moriniere|BOLD ID Engine Manual|BOLD:ACR0046
Diamesa insignipes|BIOUG16783-D07|Jerome Moriniere|BOLD ID Engine Manual|BOLD:ACR0046
Diamesa insignipes|BIOUG16782-C01|Jerome Moriniere|BOLD ID Engine Manual|BOLD:ACR0046
Diamesa insignipes|BIOUG16782-B03|Jerome Moriniere|BOLD ID Engine Manual|BOLD:ACR0046
Diamesa insignipes|BIOUG16720-C06|Jerome Moriniere|BOLD ID Engine Manual|BOLD:ACR0046
Diamesa insignipes|BIOUG16784-B07|Jerome Moriniere|BOLD ID Engine Manual|BOLD:ACR0046
Diamesa insignipes|BIOUG16856-D07|Jerome Moriniere|BOLD ID Engine Manual|BOLD:ACR0046
Diamesa insignipes|BIOUG16782-H09|Jerome Moriniere|BOLD ID Engine Manual|BOLD:ACR0046
Diamesa insignipes|BIOUG16783-F10|Jerome Moriniere|BOLD ID Engine Manual|BOLD:ACR0046
Diamesa insignipes|BIOUG16776-B08|Jerome Moriniere|BOLD ID Engine Manual|BOLD:ACR0046
Diamesa insignipes|BIOUG16785-A01|Jerome Moriniere|BOLD ID Engine Manual|BOLD:ACR0046
Diamesa insignipes|BIOUG16739-B01|Jerome Moriniere|BOLD ID Engine Manual|BOLD:ACR0046
Diamesa insignipes|BIOUG16783-E09|Jerome Moriniere|BOLD ID Engine Manual|BOLD:ACR0046
Diamesa insignipes|BIOUG16893-E08|Jerome Moriniere|BOLD ID Engine Manual|BOLD:ACR0046
Diamesa insignipes|BIOUG16855-B08|Jerome Moriniere|BOLD ID Engine Manual|BOLD:ACR0046
Diamesa insignipes|BIOUG16779-D03|Jerome Moriniere|BOLD ID Engine Manual|BOLD:ACR0046
Diamesa insignipes|BIOUG16720-E05|Jerome Moriniere|BOLD ID Engine Manual|BOLD:ACR0046
Diamesa insignipes|BIOUG16785-B01|Jerome Moriniere|BOLD ID Engine Manual|BOLD:ACR0046
Diamesa insignipes|BIOUG16785-F05|Jerome Moriniere|BOLD ID Engine Manual|BOLD:ACR0046
Diamesa insignipes|BIOUG16785-D06|Jerome Moriniere|BOLD ID Engine Manual|BOLD:ACR0046
Diamesa insignipes|BIOUG16855-F10|Jerome Moriniere|BOLD ID Engine Manual|BOLD:ACR0046
Diamesa insignipes|BIOUG16720-E03|Jerome Moriniere|BOLD ID Engine Manual|BOLD:ACR0046
Diamesa insignipes|BIOUG16721-C09|Jerome Moriniere|BOLD ID Engine Manual|BOLD:ACR0046
Diamesa insignipes|BIOUG16779-A09|Jerome Moriniere|BOLD ID Engine Manual|BOLD:ACR0046
Diamesa insignipes|BIOUG16892-B11|Jerome Moriniere|BOLD ID Engine Manual|BOLD:ACR0046
Diamesa insignipes|BIOUG16738-B01|Jerome Moriniere|BOLD ID Engine Manual|BOLD:ACR0046
Diamesa insignipes|BIOUG16854-D09|Jerome Moriniere|BOLD ID Engine Manual|BOLD:ACR0046
Diamesa insignipes|BIOUG16779-F01|Jerome Moriniere|BOLD ID Engine Manual|BOLD:ACR0046
Diamesa insignipes|BIOUG16778-A06|Jerome Moriniere|BOLD ID Engine Manual|BOLD:ACR0046
Diamesa insignipes|BIOUG16894-A06|Jerome Moriniere|BOLD ID Engine Manual|BOLD:ACR0046
Diamesa insignipes|BIOUG16892-D03|Jerome Moriniere|BOLD ID Engine Manual|BOLD:ACR0046
Diamesa insignipes|BIOUG16720-B04|Jerome Moriniere|BOLD ID Engine Manual|BOLD:ACR0046
Diamesa insignipes|BIOUG16854-A08|Jerome Moriniere|BOLD ID Engine Manual|BOLD:ACR0046
Diamesa insignipes|BIOUG16738-C03|Jerome Moriniere|BOLD ID Engine Manual|BOLD:ACR0046
Diamesa insignipes|BIOUG16783-E07|Jerome Moriniere|BOLD ID Engine Manual|BOLD:ACR0046
Diamesa insignipes|BIOUG16783-A07|Jerome Moriniere|BOLD ID Engine Manual|BOLD:ACR0046
Diamesa insignipes|BIOUG16854-B04|Jerome Moriniere|BOLD ID Engine Manual|BOLD:ACR0046
Diamesa insignipes|BIOUG16785-E11|Jerome Moriniere|BOLD ID Engine Manual|BOLD:ACR0046
Diamesa insignipes|BIOUG16854-B07|Jerome Moriniere|BOLD ID Engine Manual|BOLD:ACR0046
Diamesa insignipes|BIOUG16859-C04|Jerome Moriniere|BOLD ID Engine Manual|BOLD:ACR0046
Diamesa insignipes|BIOUG16776-E06|Jerome Moriniere|BOLD ID Engine Manual|BOLD:ACR0046
Diamesa insignipes|BIOUG16856-B11|Jerome Moriniere|BOLD ID Engine Manual|BOLD:ACR0046
Diamesa insignipes|BIOUG16859-G01|Jerome Moriniere|BOLD ID Engine Manual|BOLD:ACR0046
Diamesa insignipes|BIOUG16721-F09|Jerome Moriniere|BOLD ID Engine Manual|BOLD:ACR0046
Diamesa insignipes|BIOUG16894-C05|Jerome Moriniere|BOLD ID Engine Manual|BOLD:ACR0046
Diamesa insignipes|BIOUG16853-E12|Jerome Moriniere|BOLD ID Engine Manual|BOLD:ACR0046
Diamesa insignipes|BIOUG16894-B10|Jerome Moriniere|BOLD ID Engine Manual|BOLD:ACR0046
Diamesa insignipes|BIOUG16739-A04|Jerome Moriniere|BOLD ID Engine Manual|BOLD:ACR0046

Diamesa insignipes|BIOUG16853-E12|Jerome Moriniere|BOLD ID Engine Manual|BOLD:ACR0046
Diamesa insignipes|BIOUG16894-B10|Jerome Moriniere|BOLD ID Engine Manual|BOLD:ACR0046
Diamesa insignipes|BIOUG16739-A04|Jerome Moriniere|BOLD ID Engine Manual|BOLD:ACR0046
Diamesa insignipes|BIOUG16783-G04|Jerome Moriniere|BOLD ID Engine Manual|BOLD:ACR0046
Diamesa insignipes|BIOUG16778-F06|Jerome Moriniere|BOLD ID Engine Manual|BOLD:ACR0046
Diamesa insignipes|BIOUG16782-D09|Jerome Moriniere|BOLD ID Engine Manual|BOLD:ACR0046
Diamesa insignipes|BIOUG16775-F05|Jerome Moriniere|BOLD ID Engine Manual|BOLD:ACR0046
Diamesa insignipes|BIOUG16739-G11|Jerome Moriniere|BOLD ID Engine Manual|BOLD:ACR0046
Diamesa insignipes|BIOUG16776-F03|Jerome Moriniere|BOLD ID Engine Manual|BOLD:ACR0046
Diamesa insignipes|BIOUG16855-H07|Jerome Moriniere|BOLD ID Engine Manual|BOLD:ACR0046
Diamesa insignipes|BIOUG16854-A05|Jerome Moriniere|BOLD ID Engine Manual|BOLD:ACR0046
Diamesa insignipes|BIOUG16783-C04|Jerome Moriniere|BOLD ID Engine Manual|BOLD:ACR0046
Diamesa insignipes|BIOUG16783-B09|Jerome Moriniere|BOLD ID Engine Manual|BOLD:ACR0046
Diamesa insignipes|BIOUG16721-E10|Jerome Moriniere|BOLD ID Engine Manual|BOLD:ACR0046
Diamesa insignipes|BIOUG16853-B02|Jerome Moriniere|BOLD ID Engine Manual|BOLD:ACR0046
Diamesa insignipes|BIOUG16777-F04|Jerome Moriniere|BOLD ID Engine Manual|BOLD:ACR0046
Diamesa insignipes|BIOUG16778-H01|Jerome Moriniere|BOLD ID Engine Manual|BOLD:ACR0046
Diamesa insignipes|BIOUG16856-B01|Jerome Moriniere|BOLD ID Engine Manual|BOLD:ACR0046
Diamesa insignipes|BIOUG16853-D11|Jerome Moriniere|BOLD ID Engine Manual|BOLD:ACR0046
Diamesa insignipes|BIOUG16853-H02|Jerome Moriniere|BOLD ID Engine Manual|BOLD:ACR0046
Diamesa insignipes|BIOUG16777-B02|Jerome Moriniere|BOLD ID Engine Manual|BOLD:ACR0046
Diamesa insignipes|BIOUG16779-C12|Jerome Moriniere|BOLD ID Engine Manual|BOLD:ACR0046
Diamesa insignipes|BIOUG16783-C11|Jerome Moriniere|BOLD ID Engine Manual|BOLD:ACR0046
Diamesa insignipes|BIOUG16740-E05|Jerome Moriniere|BOLD ID Engine Manual|BOLD:ACR0046
Diamesa insignipes|BIOUG16740-E10|Jerome Moriniere|BOLD ID Engine Manual|BOLD:ACR0046
Diamesa insignipes|BIOUG16857-D05|Jerome Moriniere|BOLD ID Engine Manual|BOLD:ACR0046
Diamesa insignipes|BIOUG16857-F02|Jerome Moriniere|BOLD ID Engine Manual|BOLD:ACR0046
Diamesa insignipes|BIOUG16777-G05|Jerome Moriniere|BOLD ID Engine Manual|BOLD:ACR0046
Diamesa insignipes|BIOUG16740-G09|Jerome Moriniere|BOLD ID Engine Manual|BOLD:ACR0046
Diamesa insignipes|BIOUG16783-H02|Jerome Moriniere|BOLD ID Engine Manual|BOLD:ACR0046
Diamesa insignipes|BIOUG16738-H05|Jerome Moriniere|BOLD ID Engine Manual|BOLD:ACR0046
Diamesa bertrami|EBAI-Ch048|Elisabeth Stur|morphology|BOLD:AAB9980
Diamesa bertrami|EBAI-Ch124|Elisabeth Stur|BOLD Identification Engine|BOLD:AAB9980
Diamesa bertrami|ATNA168|Elisabeth Stur||BOLD:AAB9980
Diamesa bertrami|EBAI-Ch224|Elisabeth Stur|morphology|BOLD:AAB9980
Diamesa bertrami|EBAI-Ch049|Elisabeth Stur|morphology|BOLD:AAB9980
Diamesa bertrami|EBAI-Ch132|Elisabeth Stur|BOLD Identification Engine|BOLD:AAB9980
Diamesa bertrami|ATNA10|Elisabeth Stur||BOLD:AAB9980
Diamesa bertrami|EBAI-Ch047|Elisabeth Stur|morphology|BOLD:AAB9980
Diamesa bertrami|EBAI-Ch056|Elisabeth Stur|morphology|BOLD:AAB9980
Diamesa bertrami|CCDB24228-E09|Sofia Wiedenbrug|BIN Taxonomy Match|BOLD:AAB9980
Diamesa bertrami|CCDB24039-E08|Sofia Wiedenbrug|BIN Taxonomy Match|BOLD:AAB9980
Diamesa bertrami|EBAI-Ch043|Elisabeth Stur|morphology|BOLD:AAB9980
Diamesa bertrami|EBAI-Ch058|Elisabeth Stur|morphology|BOLD:AAB9980
Diamesa bertrami|EBAI-Ch059|Elisabeth Stur|morphology|BOLD:AAB9980
Diamesa bertrami|EBAI-Ch123|Elisabeth Stur|BOLD Identification Engine|BOLD:AAB9980
Diamesa bertrami|EBAI-Ch129|Elisabeth Stur|BOLD Identification Engine|BOLD:AAB9980
Diamesa bertrami|EBAI-Ch133|Elisabeth Stur|BOLD Identification Engine|BOLD:AAB9980
Diamesa bertrami|EBAI-Ch054|Elisabeth Stur|morphology|BOLD:AAB9980
Diamesa bertrami|EBAI-Ch053|Elisabeth Stur|morphology|BOLD:AAB9980
Diamesa bertrami|EBAI-Ch042|Elisabeth Stur|morphology|BOLD:AAB9980
Diamesa bertrami|EBAI-Ch041|Elisabeth Stur|morphology|BOLD:AAB9980
Diamesa bertrami|EBAI-Ch249|Elisabeth Stur||BOLD:AAB9980
Diamesa bertrami|ATNA178|Elisabeth Stur||BOLD:AAB9980
Diamesa bertrami|ATNA103|Elisabeth Stur||BOLD:AAB9980
Diamesa bertrami|ATNA63|Elisabeth Stur||BOLD:AAB9980
Diamesa bertrami|ES383|Elisabeth Stur||BOLD:AAB9980
Diamesa incallida|ZMUO.026367|Marko Mutanen||BOLD:ADF1127
Diamesa incallida|ZMUO.025876|Lauri Paasivirta||BOLD:ADF1127
Diamesa incallida|ZMUO.025877|Lauri Paasivirta||BOLD:AAE7630
Diamesa incallida|BC-ZSM-DIP-22491-F12|Dieter Doczkal||BOLD:AAE7630
Diamesa incallida|ATNA415|Elisabeth Stur||BOLD:AAE7630
Diamesa incallida|ATNA119|Elisabeth Stur||BOLD:AAE7630
Diamesa incallida|ATNA449|Elisabeth Stur||BOLD:AAE7630
Diamesa incallida|ATNA115|Elisabeth Stur||BOLD:AAE7630
Diamesa cf. aberrata|ATNA192|Elisabeth Stur||BOLD:AAB1738
Diamesa cf. aberrata|ATNA450|Elisabeth Stur||BOLD:AAB1738
Diamesa cf. aberrata|ATNA114|Elisabeth Stur||BOLD:AAB1738
Diamesa cf. aberrata|ATNA101|Elisabeth Stur||BOLD:AAB1738
Diamesa cf. aberrata|ATNA100|Elisabeth Stur||BOLD:AAB1738
Diamesa lindrothi|ATNA87|Elisabeth Stur||BOLD:AAC4741
Diamesa lindrothi|ATNA133|Elisabeth Stur||BOLD:AAC4741
Diamesa lindrothi|ATNA132|Elisabeth Stur||BOLD:AAC4741
Diamesa lindrothi|ATNA90|Elisabeth Stur||BOLD:AAC4741
Diamesa lindrothi|ATNA86|Elisabeth Stur||BOLD:AAC4741
Diamesa lindrothi|ATNA85|Elisabeth Stur||BOLD:AAC4741
Diamesa lindrothi|ATNA88|Elisabeth Stur||BOLD:AAC4741
Diamesa lindrothi|ATNA93|Elisabeth Stur||BOLD:AAC4741
Diamesa lindrothi|ATNA135|Elisabeth Stur||BOLD:AAC4741
Diamesa lindrothi|ATNA134|Elisabeth Stur||BOLD:AAC4741
Diamesa lindrothi|ATNA54|Elisabeth Stur||BOLD:AAC4741
Diamesa latitarsis|ATNA91|Elisabeth Stur||BOLD:AAC7191
Diamesa latitarsis|EBAI-Ch130|Elisabeth Stur|BOLD Identification Engine|BOLD:AAC7191
Diamesa latitarsis|EBAI-Ch226|Elisabeth Stur||BOLD:AAC7191
Diamesa latitarsis|EBAI-Ch128|Elisabeth Stur|BOLD Identification Engine|BOLD:AAC7191
Diamesa latitarsis|ATNA64|Elisabeth Stur||BOLD:AAC7191
Diamesa latitarsis|BC-ZSM-DIP-22551-C07|Dieter Doczkal||BOLD:AAC7191
Diamesa latitarsis|EBAI-Ch057|Elisabeth Stur|morphology|BOLD:AAC7191
Diamesa latitarsis|EBAI-Ch055|Elisabeth Stur|BOLD Identification Engine|BOLD:AAC7191
Diamesa latitarsis|EBAI-Ch062|Elisabeth Stur|morphology|BOLD:AAC7191
Diamesa latitarsis|EBAI-Ch051|Elisabeth Stur|BOLD Identification Engine|BOLD:AAC7191
Diamesa latitarsis|EBAI-Ch050|Elisabeth Stur|morphology|BOLD:AAC7191
Diamesa latitarsis|EBAI-Ch044|Elisabeth Stur|morphology|BOLD:AAC7191
Diamesa latitarsis|EBAI-Ch131|Elisabeth Stur|BOLD Identification Engine|BOLD:AAC7191
Diamesa latitarsis|EBAI-Ch225|Elisabeth Stur||BOLD:AAC7191
Diamesa latitarsis|ATNA60|Elisabeth Stur||BOLD:AAC7191
Diamesa latitarsis|ATNA11|Elisabeth Stur||BOLD:AAC7191
Diamesa latitarsis|ATNA155|Elisabeth Stur||BOLD:AAC7191
Diamesa latitarsis|ATNA149|Elisabeth Stur||BOLD:AAC7191
Diamesa latitarsis|ATNA89|Elisabeth Stur||BOLD:AAC7191
Diamesa latitarsis|ES282|||BOLD:AAC7191
Diamesa steinboecki|OM867253
Diamesa bohemani|CCDB24229-F04|Sofia Wiedenbrug|BIN Taxonomy Match|BOLD:AAB5113

[illegible]
Diamesa latitarsis|ES282|||BOLD:AAC7191
Diamesa steinboecki|OM867253
Diamesa bohemani|CCDB24229-F04|Sofia Wiedenbrug|BIN Taxonomy Match|BOLD:AAB5113
Diamesa bohemani|BC-ZSM-DIP-22551-F04|Dieter Doczkal||BOLD:AAB5113
Diamesa bohemani|ATNA412|Elisabeth Stur||BOLD:AAB5113
Diamesa bohemani|CCDB24229-E10|Sofia Wiedenbrug|BIN Taxonomy Match|BOLD:AAB5113
Diamesa bohemani|CCDB24229-E01|Sofia Wiedenbrug|BIN Taxonomy Match|BOLD:AAB5113
Diamesa bohemani|EBAI-Ch061|Elisabeth Stur|morphology|BOLD:AAB5113
Diamesa bohemani|BC-ZSM-DIP-22551-C10|Dieter Doczkal||BOLD:AAB5113
Diamesa bohemani|BC-ZSM-DIP-22488-C05|Dieter Doczkal||BOLD:AAB5113
Diamesa bohemani|CCDB24228-E08|Sofia Wiedenbrug|BIN Taxonomy Match|BOLD:AAB5113
Diamesa steinboecki|OM867254
Diamesa bohemani|ATNA312|Elisabeth Stur||BOLD:AAB5113
Diamesa bohemani|EBAI-Ch060|Elisabeth Stur|morphology|BOLD:AAB5113
Diamesa bohemani|BC-ZSM-DIP-22551-E06|Dieter Doczkal||BOLD:AAB5113
Diamesa bohemani|ATNA413|Elisabeth Stur||BOLD:AAB5113
Diamesa bohemani|ATNA102|Elisabeth Stur||BOLD:AAB5113
Diamesa bohemani|ATNA446|Elisabeth Stur||BOLD:AAB5113
Diamesa bohemani|ATNA445|Elisabeth Stur||BOLD:AAB5113
Diamesa|ZSM-DIP-33148-F01|Caroline Chimeno|BIN Taxonomy Match (Jan 2022)|BOLD:AAB5106
Diamesa|ZSM-DIP-33172-B10|Caroline Chimeno|BIN Taxonomy Match (Jan 2022)|BOLD:AAB5106
Diamesa tonsa|BC-ZSM-DIP-22551-D06|Dieter Doczkal||BOLD:AAB5106
Diamesa tonsa|BC-ZSM-DIP-22488-E02|Dieter Doczkal||BOLD:AAB5106
Diamesa|ZSM-DIP-33148-E06|Caroline Chimeno|BIN Taxonomy Match (Jan 2022)|BOLD:AAB5106
Diamesa|ZSM-DIP-33148-G03|Caroline Chimeno|BIN Taxonomy Match (Jan 2022)|BOLD:AAB5106
Diamesa tonsa|CCDB24038-B06|Sofia Wiedenbrug|BIN Taxonomy Match|BOLD:AAB5106
Diamesa tonsa|BC-ZSM-DIP-22551-F01|Dieter Doczkal||BOLD:AAB5106
Diamesa tonsa|CCDB24039-G02|Sofia Wiedenbrug|BIN Taxonomy Match|BOLD:AAB5106
Diamesa tonsa|CCDB24229-D05|Sofia Wiedenbrug|BIN Taxonomy Match|BOLD:AAB5106
Diamesa tonsa|BC-ZSM-DIP-22491-F02|Dieter Doczkal||BOLD:AAB5106
Diamesa|ZSM-DIP-33148-G09|Caroline Chimeno|BIN Taxonomy Match (Jan 2022)|BOLD:AAB5106
Diamesa tonsa|CCDB24229-E03|Sofia Wiedenbrug||BOLD:AAB5106
Diamesa tonsa|CCDB24039-E09|Sofia Wiedenbrug|BIN Taxonomy Match|BOLD:AAB5106
Diamesa|EBAI-Ch125|Elisabeth Stur|morphology|BOLD:AAB5106
Diamesa tonsa|CCDB24038-C09|Sofia Wiedenbrug|BIN Taxonomy Match|BOLD:AAB5106
Diamesa|ZSM-DIP-33172-B09|Caroline Chimeno|BIN Taxonomy Match (Jan 2022)|BOLD:AAB5106
Diamesa tonsa|BC-ZSM-DIP-22491-G05|Dieter Doczkal||BOLD:AAB5106
Diamesa tonsa|CCDB24039-E10|Sofia Wiedenbrug|BIN Taxonomy Match|BOLD:AAB5106
Diamesa tonsa|CCDB24228-F10|Sofia Wiedenbrug|BIN Taxonomy Match|BOLD:AAB5106
Diamesa tonsa|BC-ZSM-DIP-22551-E09|Dieter Doczkal||BOLD:AAB5106
Diamesa tonsa|CCDB21605-A10|Sofia Wiedenbrug||BOLD:AAB5106
Diamesa tonsa|BC-ZSM-DIP-22491-G02|Dieter Doczkal||BOLD:AAB5106
Diamesa tonsa|ZSM34343-F02|Sofia Wiedenbrug|BIN-taxonomy|BOLD:AAB5106
Diamesa tonsa|CCDB24229-C01|Sofia Wiedenbrug|BIN Taxonomy Match|BOLD:AAB5106
Diamesa tonsa|BC-ZSM-DIP-22491-E08|Dieter Doczkal||BOLD:AAB5106
Diamesa|ZSM-DIP-33148-E05|Caroline Chimeno|BIN Taxonomy Match (Jan 2022)|BOLD:AAB5106
Diamesa|ZSM-DIP-33148-E04|Caroline Chimeno|BIN Taxonomy Match (Jan 2022)|BOLD:AAB5106
Diamesa|ZSM-DIP-33148-G08|Caroline Chimeno|BIN Taxonomy Match (Jan 2022)|BOLD:AAB5106
Diamesa tonsa|ZSM34342-C12|Sofia Wiedenbrug|BIN-taxonomy|BOLD:AAB5106
Diamesa tonsa|BC-ZSM-DIP-22491-G06|Dieter Doczkal||BOLD:AAB5106
Diamesa tonsa|BC-ZSM-DIP-22491-H11|Dieter Doczkal||BOLD:AAB5106
Diamesa tonsa|BC-ZSM-DIP-22491-F07|Dieter Doczkal||BOLD:AAB5106
Diamesa cinerella|MT362508|||BOLD:AAB5106
Diamesa|ZSM-DIP-33148-F02|Caroline Chimeno|BIN Taxonomy Match (Jan 2022)|BOLD:AAB5106
Diamesa tonsa|CCDB24229-H08|Sofia Wiedenbrug|BIN Taxonomy Match|BOLD:AAB5106
Diamesa tonsa|BC-ZSM-DIP-22551-D10|Dieter Doczkal||BOLD:AAB5106
Diamesa tonsa|CCDB24039-B09|Sofia Wiedenbrug|BIN Taxonomy Match|BOLD:AAB5106
Diamesa tonsa|BC-ZSM-DIP-22491-F09|Dieter Doczkal||BOLD:AAB5106
Diamesa|ZSM-DIP-33148-F04|Caroline Chimeno|BIN Taxonomy Match (Jan 2022)|BOLD:AAB5106
Diamesa|ES281|||BOLD:AAB5106
Diamesa|EBAI-Ch039|Elisabeth Stur|morphology|BOLD:AAB5106
Diamesa|ATNA189|Elisabeth Stur||BOLD:AAB5106
Diamesa hyperborea|ATNA81|Elisabeth Stur||BOLD:AAB5106
Diamesa hyperborea|ATNA57|Elisabeth Stur||BOLD:AAB5106
Diamesa hyperborea|ATNA58|Elisabeth Stur||BOLD:AAB5106
Diamesa|ATNA190|Elisabeth Stur||BOLD:AAB5106
Diamesa tonsa|ATNA109|Elisabeth Stur||BOLD:AAB5106
Diamesa|ATNA170|Elisabeth Stur||BOLD:AAB5106
Diamesa|ATNA13|Elisabeth Stur||BOLD:AAB5106
Diamesa hyperborea|ATNA55|Elisabeth Stur||BOLD:AAB5106
Diamesa hyperborea|ATNA66|Elisabeth Stur||BOLD:AAB5106
Diamesa hyperborea|ATNA80|Elisabeth Stur||BOLD:AAB5106
Diamesa|ATNA372|Elisabeth Stur||BOLD:AAB5106
Diamesa|EBAI-Ch229|Elisabeth Stur|morphology|BOLD:AAB5106
Diamesa tonsa|ATNA96|Elisabeth Stur||BOLD:AAB5106
Diamesa hyperborea|ATNA83|Elisabeth Stur||BOLD:AAB5106
Diamesa|ATNA169|Elisabeth Stur||BOLD:AAB5106
Diamesa hyperborea|ATNA110|Elisabeth Stur||BOLD:AAB5106
Diamesa tonsa|ATNA112|Elisabeth Stur||BOLD:AAB5106
Diamesa|ATNA146|Elisabeth Stur||BOLD:AAB5106
Diamesa|ATNA163|Elisabeth Stur||BOLD:AAB5106
Diamesa|ATNA175|Elisabeth Stur||BOLD:AAB5106
Diamesa|ATNA188|Elisabeth Stur||BOLD:AAB5106
Diamesa|ATNA191|Elisabeth Stur||BOLD:AAB5106
Diamesa tonsa|ATNA111|Elisabeth Stur||BOLD:AAB5106
Diamesa|ATNA156|Elisabeth Stur||BOLD:AAB5106
Diamesa|EBAI-Ch231|Elisabeth Stur|morphology|BOLD:AAB5106
Diamesa|EBAI-Ch232|Elisabeth Stur||BOLD:AAB5106
Diamesa|EBAI-Ch250|Elisabeth Stur|morphology|BOLD:AAB5106
Diamesa|EBAI-Ch230|Elisabeth Stur||BOLD:AAB5106
Diamesa|EBAI-Ch233|Elisabeth Stur||BOLD:AAB5106
Diamesa|EBAI-Ch127|Elisabeth Stur|morphology|BOLD:AAB5106
Diamesa hyperborea|ATNA97|Elisabeth Stur||BOLD:AAB5106
Diamesa|EBAI-Ch040|Elisabeth Stur|morphology|BOLD:AAB5106
Diamesa|ATNA140|Elisabeth Stur||BOLD:AAB5106
Diamesa|BIOUG15201-H05|Kate Perez|BIN Taxonomy Match|BOLD:AAB5106
Diamesa|EBAI-Ch227|Elisabeth Stur||BOLD:AAB5106
Diamesa|BIOUG15591-G09|Kate Perez|BIN Taxonomy Match|BOLD:AAB5106
Diamesa hyperborea|ATNA98|Elisabeth Stur||BOLD:AAB5106
Diamesa hyperborea|ATNA56|Elisabeth Stur||BOLD:AAB5106
Diamesa tonsa|TRD-CH81|Elisabeth Stur||BOLD:AAB5106
Diamesa hyperborea|Finnmark540|Elisabeth Stur||BOLD:AAB5106
[illegible]

Diamesa hyperborea|ATNA56|Elisabeth Stur||BOLD:AAB5106
Diamesa tonsa|TRD-CH81|Elisabeth Stur||BOLD:AAB5106
Diamesa hyperborea|Finnmark540|Elisabeth Stur||BOLD:AAB5106
Anatopynia plumipes|Finnmark50|Elisabeth Stur||BOLD:AAM6334
Anatopynia plumipes|Finnmark741|Elisabeth Stur||BOLD:AAM6334
Anatopynia plumipes|Finnmark740|Elisabeth Stur||BOLD:AAM6334
Anatopynia plumipes|Finnmark739|Elisabeth Stur||BOLD:AAM6334
Anatopynia plumipes|Finnmark105|Elisabeth Stur||BOLD:AAM6334
Boreochlus thienemanni|ZMUO.026211|Lauri Paasivirta||BOLD:ADE9751
Boreochlus thienemanni|ZMUO.026210|Lauri Paasivirta||BOLD:ADE9751
Boreochlus thienemanni|ZMUO.025960|Lauri Paasivirta||BOLD:ADE9751
Boreochlus thienemanni|ZMUO.025959|Lauri Paasivirta||BOLD:ADE9751
Paraboreochlus|GBOL11564|Sofia Wiedenbrug||BOLD:ACV9681
Paraboreochlus minutissimus|CCDB21605-B02|Sofia Wiedenbrug||BOLD:ACQ8238
Paraboreochlus minutissimus|CCDB21605-B01|Sofia Wiedenbrug||BOLD:ACQ8238
Paraboreochlus minutissimus|GBOL03778|Sofia Wiedenbrug||BOLD:ACQ8238
Paraboreochlus minutissimus|ES25|Elisabeth Stur||BOLD:AAF7836
Paraboreochlus minutissimus|ES60|Elisabeth Stur||BOLD:AAF7836
Paraboreochlus minutissimus|ES24|Elisabeth Stur||BOLD:AAF7836
Parochlus kiefferi|SOE48|Elisabeth Stur||BOLD:AAC1428
Parochlus kiefferi|ZMUO.025978|Lauri Paasivirta||BOLD:AAC1427
Parochlus kiefferi|ZMUO.025979|Lauri Paasivirta||BOLD:AAC1427
Parochlus kiefferi|SOE139|Elisabeth Stur||BOLD:AAC1427
Parochlus kiefferi|SOE55|Elisabeth Stur||BOLD:AAC1427
Parochlus kiefferi|SOE156|Elisabeth Stur||BOLD:AAC1427
Parochlus kiefferi|SOE312|Elisabeth Stur||BOLD:AAC1427
Parochlus kiefferi|SOE43|Elisabeth Stur||BOLD:AAC1427
Parochlus kiefferi|TRD-CH122|Elisabeth Stur||BOLD:AAC1427
Clinotanypus nervosus|ZMUO.025327|Lauri Paasivirta||BOLD:ADC7811
Clinotanypus nervosus|ZMUO.025328|Lauri Paasivirta||BOLD:ADC7811
Clinotanypus nervosus|ZMUO.029774|Marko Mutanen||BOLD:ADC7811
Tanypus|gvcT10382|Graeme V. Cocks||BOLD:AAV5910
Tanypus|gvc15600-1L|Angela Telfer|BIN Taxonomy Match (Feb 2019)|BOLD:AAV5910
Procladius flavifrons|NHRS-BYWS000001123|Yngve Brodin|morphology|BOLD:ACT5760
Procladius flavifrons|ZMUO.026149|Lauri Paasivirta||BOLD:ACT5760
Procladius flavifrons|ZMUO.026150|Lauri Paasivirta||BOLD:ACT5760
Procladius flavifrons|TRD-CH340|Elisabeth Stur|morphology|BOLD:ACT5760
Procladius flavifrons|TRD-CH217|Elisabeth Stur||BOLD:ACT5760
Procladius flavifrons|TRD-CH216|Elisabeth Stur||BOLD:ACT5760
Procladius imicola|ZMUO.029775|Marko Mutanen||BOLD:ADA8682
Procladius imicola|ZMUO.024615|Lauri Paasivirta||BOLD:ADA8682
Procladius imicola|ZMUO.024614|Lauri Paasivirta||BOLD:ADA8682
Procladius imicola|ZMUO.024279|Lauri Paasivirta||BOLD:ADA8682
Procladius cf. flavifrons|Finnmark461|Elisabeth Stur||BOLD:AEG9801
Procladius cf. flavifrons|Finnmark460|Elisabeth Stur||BOLD:AEG9801
Procladius|ZMUO.024654|Lauri Paasivirta||BOLD:ADA8287
Procladius sp. 8ES|TRD-CH422|Elisabeth Stur|morphology|BOLD:ACT6620
Procladius sp. 8ES|TRD-CH218|Elisabeth Stur|morphology|BOLD:ACT6620
Procladius sp. 8ES|ZSM-DIP-33149-G10|Caroline Chimeno|BIN Taxonomy Match (Jan 2022)|BOLD ...
Procladius sp. 8ES|ZSM-DIP-33144-D01|Caroline Chimeno|BIN Taxonomy Match (Jan 2022)|BOLD ...
Procladius sp. 8ES|ZSM-DIP-33133-A07|Caroline Chimeno|BIN Taxonomy Match (Jan 2022)|BOLD ...
Procladius sagittalis|NHRS-BYWS000001836|Yngve Brodin||BOLD:ACT1181
Procladius sagittalis|NHRS-BYWS000001859|Yngve Brodin||BOLD:ACT1181
Procladius sagittalis|NHRS-BYWS000000367|Yngve Brodin|morphology|BOLD:ACT1181
Procladius sagittalis|NHRS-BYWS000001835|Yngve Brodin||BOLD:ACT1181
Procladius sagittalis|NHRS-BYWS000001172|Yngve Brodin|morphology|BOLD:ACT1181
Procladius sagittalis|NHRS-BYWS000000428|Yngve Brodin|morphology|BOLD:ACT1181
Procladius sagittalis|NHRS-BYWS000000368|Yngve Brodin|morphology|BOLD:ACT1181
Procladius sagittalis|NHRS-BYWS000001024|Yngve Brodin|morphology|BOLD:ACT1181
Procladius choreus|NHRS-BYWS000000567|Yngve Brodin|morphology|BOLD:ACO4026
Procladius choreus|NHRS-BYWS000000177|Yngve Brodin|morphology|BOLD:ACO4026
Chironomidae|ZSM-DIP-33128-A07|Caroline Chimeno|BIN Taxonomy Match (Jan 2022)|BOLD:AEE2492
Procladius|ZMUO.024653|Lauri Paasivirta||BOLD:ADA6883
Procladius|ZSM-DIP-33144-H05|Caroline Chimeno|BIN Taxonomy Match (Jan 2022)|BOLD:AEE2525
Procladius|ZSM-DIP-33129-C10|Caroline Chimeno|BIN Taxonomy Match (Jan 2022)|BOLD:AEE2525
Procladius|ZSM-DIP-33141-C09|Caroline Chimeno|BIN Taxonomy Match (Jan 2022)|BOLD:AEE2525
Procladius|ZSM-DIP-33149-G08|Caroline Chimeno|BIN Taxonomy Match (Jan 2022)|BOLD:AEE2525
Chironomidae|KY225377|||BOLD:AAF6798
Procladius signatus|PK-186-35|Petra Kranzfelder|morphology|BOLD:AAF6798
Procladius signatus|NHRS-BYWS000001070|Yngve Brodin|morphology|BOLD:AAF6798
Procladius signatus|ZMUO.025157|Lauri Paasivirta||BOLD:AAF6798
Procladius signatus|ZMUO.025158|Lauri Paasivirta||BOLD:AAF6798
Procladius signatus|NHRS-BYWS000001117|Yngve Brodin|morphology|BOLD:AAF6798
Procladius signatus|Finnmark272|Elisabeth Stur||BOLD:AAF6798
Procladius signatus|Finnmark211|Elisabeth Stur||BOLD:AAF6798
Procladius signatus|Finnmark212|Elisabeth Stur||BOLD:AAF6798
Procladius signatus|TRD-CH304|Elisabeth Stur||BOLD:AAF6798
Procladius signatus|Finnmark457|Elisabeth Stur||BOLD:AAF6798
Procladius choreus-agg.2(iso)|EJV-20110121|Lauri Paasivirta||BOLD:ACW5647
Procladius choreus-agg.2(iso)|EJV-20110124|Lauri Paasivirta||BOLD:ACW5647
Procladius choreus-agg.2(iso)|EJV-20110122|Lauri Paasivirta||BOLD:ACW5647
Procladius culiciformis|NHRS-BYWS000000440|Yngve Brodin|morphology|BOLD:ACW5647
Procladius culiciformis|NHRS-BYWS000000971|Yngve Brodin|morphology|BOLD:ACW5647
Procladius|ZMUO.024753|Lauri Paasivirta||BOLD:ACW5647
Procladius|ZMUO.024662|Lauri Paasivirta||BOLD:ACW5647
Procladius culiciformis|NHRS-BYWS000000364|Yngve Brodin|morphology|BOLD:ACW5647
Procladius culiciformis|NHRS-BYWS000001839|Yngve Brodin||BOLD:ACW5647
Procladius culiciformis|NHRS-BYWS000000942|Yngve Brodin|morphology|BOLD:ACW5647
Procladius sagittalis|KY225378|||BOLD:ACW5385
Procladius culiciformis|TRD-CH307|Elisabeth Stur||BOLD:ACW5385
Procladius|ZMUO.024752|Lauri Paasivirta||BOLD:ACW5385
Procladius culiciformis|NHRS-BYWS000001178|Yngve Brodin|morphology|BOLD:ACW5385
Procladius culiciformis|NHRS-BYWS000000284|Yngve Brodin|morphology|BOLD:ACW5385
Procladius|ZSM-DIP-33142-A03|Caroline Chimeno|BIN Taxonomy Match (Jan 2022)|BOLD:ACW5385
Procladius|ZSM-DIP-33146-D04|Caroline Chimeno|BIN Taxonomy Match (Jan 2022)|BOLD:ACW5385
Procladius|BIOUG36684-E11|Kate Perez|BIN Taxonomy Match (May 2018)|BOLD:ACW5385
Procladius culiciformis|OK354408|||BOLD:ACW5385
Procladius culiciformis|NHRS-BYWS000001038|Yngve Brodin|morphology|BOLD:ACW5385
Procladius pectinatus|ZMUO.024581|Lauri Paasivirta||BOLD:ACW5385
Procladius pectinatus|ZMUO.024580|Lauri Paasivirta||BOLD:ACW5385
Procladius culiciformis|NHRS-BYWS000001025|Yngve Brodin|morphology|BOLD:ACW5385
Procladius culiciformis|NHRS-BYWS000000928|Yngve Brodin|morphology|BOLD:ACW5385
Procladius simplicistylus|ZMUO.025148|Lauri Paasivirta||BOLD:ADF1974

Procladius culiciformis|NHRS-BYWS000001025|Yngve Brodin|morphology|BOLD:ACW5385
Procladius culiciformis|NHRS-BYWS000000928|Yngve Brodin|morphology|BOLD:ACW5385
Procladius simplicistylus|ZMUO.025148|Lauri Paasivirta||BOLD:ADF1974
Procladius simplicistylus|ZMUO.025147|Lauri Paasivirta||BOLD:ADF1974
Procladius crassinervis|CCDB22603-B04|Sofia Wiedenbrug||BOLD:ACQ1725
Procladius crassinervis|CCDB22603-B03|Sofia Wiedenbrug||BOLD:ACQ1725
Procladius|ZSM-DIP-33138-D07|Caroline Chimeno|BIN Taxonomy Match (Jan 2022)|BOLD:AAG3921
Procladius|ZMUO.024663|Lauri Paasivirta||BOLD:AAG3921
Procladius nigriventris|TRD-CH204|Elisabeth Stur||BOLD:AAG3921
Procladius nigriventris|TRD-CH72|Elisabeth Stur||BOLD:AAG3921
Procladius nigriventris|NO 90|Elisabeth Stur||BOLD:AAG3921
Procladius nigriventris|TRD-CH178|Elisabeth Stur||BOLD:AAG3921
Procladius nigriventris|Finnmark102|Elisabeth Stur||BOLD:AAG3921
Procladius|ZSM-DIP-33130-G04|Caroline Chimeno|BIN Taxonomy Match (Jan 2022)|BOLD:AAG3921
Procladius nigriventris|Finnmark79|Elisabeth Stur||BOLD:AAG3921
Procladius|ZMUO.024612|Lauri Paasivirta||BOLD:AAG3921
Procladius nigriventris|Finnmark101|Elisabeth Stur||BOLD:AAG3921
Procladius nigriventris|Finnmark794|Elisabeth Stur||BOLD:AAG3921
Procladius nigriventris|Finnmark40|Elisabeth Stur||BOLD:AAG3921
Procladius nigriventris|ATNA75|Elisabeth Stur||BOLD:AAG3921
Procladius nigriventris|TRD-CH97|Elisabeth Stur||BOLD:AAG3921
Procladius|ZMUO.026228|Lauri Paasivirta||BOLD:ACS2256
Procladius|ZMUO.026227|Lauri Paasivirta||BOLD:ACS2256
Procladius barbatus|Finnmark649|Elisabeth Stur||BOLD:AAV2572
Procladius frigidus|Finnmark384|Elisabeth Stur||BOLD:AAB9256
Procladius frigidus|Finnmark33|Elisabeth Stur||BOLD:AAB9256
Procladius frigidus|Finnmark354|Elisabeth Stur||BOLD:AAB9256
Procladius crassinervis|BIOUG43949-H05|Kate Perez|BIN Taxonomy Match below Phylum (Jan 2019 ...
Procladius frigidus|Finnmark728|Elisabeth Stur||BOLD:AAB9256
Procladius frigidus|Finnmark386|Elisabeth Stur||BOLD:AAB9256
Procladius frigidus|Finnmark803|Elisabeth Stur||BOLD:AAB9256
Procladius|ZMUO.024613|Lauri Paasivirta||BOLD:AAB9256
Procladius|ZMUO.024291|Lauri Paasivirta||BOLD:AAB9256
Procladius pectinatus|ZMUO.024665|Lauri Paasivirta||BOLD:AAB9256
Procladius pectinatus|ZMUO.024666|Lauri Paasivirta||BOLD:AAB9256
Procladius|ZMUO.024622|Lauri Paasivirta||BOLD:AAB9256
Procladius|ZMUO.024621|Lauri Paasivirta||BOLD:AAB9256
Procladius|ZMUO.024290|Lauri Paasivirta||BOLD:AAB9256
Procladius appropinquatus|ZMUO.026204|Lauri Paasivirta||BOLD:ADF1163
Procladius appropinquatus|ZMUO.026203|Lauri Paasivirta||BOLD:ADF1163
Procladius appropinquatus|NHRS-BYWS000000396|Yngve Brodin|morphology|BOLD:ADF1163
Procladius cf. ruris|SOE297|Torbjorn Ekrem||BOLD:AEB4593
Procladius|ZMUO.026285|Lauri Paasivirta||BOLD:AAD7251
Procladius dentus|Finnmark405|Elisabeth Stur||BOLD:AAD7251
Procladius dentus|Finnmark459|Elisabeth Stur||BOLD:AAD7251
Procladius nr. fuscus|ZMUO.025304|Lauri Paasivirta||BOLD:ACY8325
Procladius|ZSM-DIP-33148-D01|Caroline Chimeno|BIN Taxonomy Match (Jan 2022)|BOLD:ACY8325
Procladius cf. pruinosus|NHRS-BYWS000000547|Yngve Brodin|morphology|BOLD:ACY8325
Procladius cf. pruinosus|NHRS-BYWS000000562|Yngve Brodin|morphology|BOLD:ACY8325
Procladius pruinosus|TRD-CH70|Yngve Brodin|morphology|BOLD:ACY8325
Procladius pruinosus|NO 89|Elisabeth Stur|morphology|BOLD:ACY8325
Procladius pruinosus|NO 88|Yngve Brodin|morphology|BOLD:ACY8325
Procladius pruinosus|TRD-CH351|Yngve Brodin|morphology|BOLD:ACY8325
Procladius pruinosus|TRD-CH205|Elisabeth Stur|morphology|BOLD:ACY8325
Procladius|BIOUG55114-D03|Kate Perez|BIN Taxonomy Match (Oct 2020)|BOLD:AER3360
Procladius cf. cinereus|TRD-CH293|Elisabeth Stur|morphology|BOLD:AER3360
Procladius|ZSM-DIP-33133-G04|Caroline Chimeno|BIN Taxonomy Match (Jan 2022)|BOLD:AER3360
Procladius crassinervis|NHRS-BYWS000000530|Yngve Brodin|morphology|BOLD:AER3361
Procladius|ZMUO.026148|Lauri Paasivirta||BOLD:AER3361
Procladius|ZMUO.026147|Lauri Paasivirta||BOLD:AER3361
Procladius choreus-agg.1|EJV-20110051|Lauri Paasivirta||BOLD:AER3362
Procladius choreus-agg.1|EJV-20110054|Lauri Paasivirta||BOLD:AER3362
Procladius choreus-agg.1|EJV-20110056|Lauri Paasivirta||BOLD:AER3362
Procladius choreus-agg.1|EJV-20110047|Lauri Paasivirta||BOLD:AER3362
Procladius choreus-agg.1|EJV-20110055|Lauri Paasivirta||BOLD:AER3362
Procladius choreus-agg.1|EJV-20110053|Lauri Paasivirta||BOLD:AER3362
Procladius choreus-agg.1|EJV-20110052|Lauri Paasivirta||BOLD:AER3362
Procladius choreus-agg.1|EJV-20110050|Lauri Paasivirta||BOLD:AER3362
Procladius choreus-agg.1|EJV-20110048|Lauri Paasivirta||BOLD:AER3362
Procladius choreus-agg.cinereus-t.|EJV-20110221|Lauri Paasivirta||BOLD:AAG5487
Procladius crassinervis|NHRS-BYWS000001837|Yngve Brodin||BOLD:AAG5487
Procladius crassinervis|NHRS-BYWS000000913|Yngve Brodin|morphology|BOLD:AAG5487
Chironomidae|ZSM-DIP-33147-B09|Caroline Chimeno|BIN Taxonomy Match (Jan 2022)|BOLD:AAG5487
Chironomidae|ZSM-DIP-33131-E01|Caroline Chimeno|BIN Taxonomy Match (Jan 2022)|BOLD:AAG5487
Chironomidae|CCDB21605-G02|Sofia Wiedenbrug|no morphological identification - introduction of int...
Chironomidae|ZSM-DIP-33147-A12|Caroline Chimeno|BIN Taxonomy Match (Jan 2022)|BOLD:AAG5487
Procladius crassinervis|NHRS-BYWS000000581|Yngve Brodin|morphology|BOLD:AAG5487
Procladius crassinervis|NHRS-BYWS000000962|Yngve Brodin|morphology|BOLD:AAG5487
Procladius crassinervis|NHRS-BYWS000001039|Yngve Brodin|morphology|BOLD:AAG5487
Procladius crassinervis|NHRS-BYWS000000216|Yngve Brodin|morphology|BOLD:AAG5487
Procladius crassinervis|NHRS-BYWS000000253|Yngve Brodin|morphology|BOLD:AAG5487
Procladius crassinervis|NHRS-BYWS000000372|Yngve Brodin|morphology|BOLD:AAG5487
Procladius crassinervis|NHRS-BYWS000000574|Yngve Brodin|morphology|BOLD:AAG5487
Chironomidae|CCDB22605-H11|Sofia Wiedenbrug|no morphological identification - introduction of int...
Procladius|ZSM-DIP-33143-E12|Caroline Chimeno|BIN Taxonomy Match (Jan 2022)|BOLD:AAG5487
Procladius|ZSM-DIP-33146-H07|Caroline Chimeno|BIN Taxonomy Match (Jan 2022)|BOLD:AAG5487
Procladius|ZSM-DIP-33147-B03|Caroline Chimeno|BIN Taxonomy Match (Jan 2022)|BOLD:AAG5487
Chironomidae|ZSM-DIP-33143-H04|Caroline Chimeno|BIN Taxonomy Match (Jan 2022)|BOLD:AAG5487
Chironomidae|ZSM-DIP-33146-H05|Caroline Chimeno|BIN Taxonomy Match (Jan 2022)|BOLD:AAG5487
Chironomidae|ZSM-DIP-33146-H06|Caroline Chimeno|BIN Taxonomy Match (Jan 2022)|BOLD:AAG5487
Chironomidae|ZSM-DIP-33146-H08|Caroline Chimeno|BIN Taxonomy Match (Jan 2022)|BOLD:AAG5487
Chironomidae|ZSM-DIP-33147-B01|Caroline Chimeno|BIN Taxonomy Match (Jan 2022)|BOLD:AAG5487
Chironomidae|ZSM-DIP-33147-B02|Caroline Chimeno|BIN Taxonomy Match (Jan 2022)|BOLD:AAG5487
Chironomidae|ZSM-DIP-33149-B05|Caroline Chimeno|BIN Taxonomy Match (Jan 2022)|BOLD:AAG5487
Chironomidae|ZSM-DIP-33149-B06|Caroline Chimeno|BIN Taxonomy Match (Jan 2022)|BOLD:AAG5487
Chironomidae|ZSM-DIP-33149-B07|Caroline Chimeno|BIN Taxonomy Match (Jan 2022)|BOLD:AAG5487
Chironomidae|ZSM-DIP-33149-F08|Caroline Chimeno|BIN Taxonomy Match (Jan 2022)|BOLD:AAG5487
Chironomidae|ZSM-DIP-33149-F09|Caroline Chimeno|BIN Taxonomy Match (Jan 2022)|BOLD:AAG5487
Chironomidae|ZSM-DIP-33143-H03|Caroline Chimeno|BIN Taxonomy Match (Jan 2022)|BOLD:AAG5487
Procladius|ZSM-DIP-33147-B04|Caroline Chimeno|BIN Taxonomy Match (Jan 2022)|BOLD:AAG5487
Procladius crassinervis|NHRS-BYWS000000215|Yngve Brodin|morphology|BOLD:AAG5487
Procladius crassinervis|NHRS-BYWS000001838|Yngve Brodin||BOLD:AAG5487
Procladius crassinervis|NHRS-BYWS000000914|Yngve Brodin|morphology|BOLD:AAG5487

[illegible]
Procladius crassinervis|NHRS-BYWS000000215|Yngve Brodin|morphology|BOLD:AAG5487
Procladius crassinervis|NHRS-BYWS000001838|Yngve Brodin||BOLD:AAG5487
Procladius crassinervis|NHRS-BYWS000000914|Yngve Brodin|morphology|BOLD:AAG5487
Procladius crassinervis|NHRS-BYWS000000580|Yngve Brodin|morphology|BOLD:AAG5487
Procladius rufovittatus|NHRS-BYWS000001167|Yngve Brodin|morphology|BOLD:ADA8642
Procladius nudipennis|ZMUO.025139|Lauri Paasivirta||BOLD:ADA8642
Procladius nudipennis|ZMUO.025138|Lauri Paasivirta||BOLD:ADA8642
Procladius nudipennis|ZMUO.024285|Lauri Paasivirta||BOLD:ADA8642
Procladius nudipennis|ZMUO.024284|Lauri Paasivirta||BOLD:ADA8642
Procladius rufovittatus|ZMUO.025180|Lauri Paasivirta||BOLD:AAG5527
Procladius rufovittatus|ZMUO.025181|Lauri Paasivirta||BOLD:AAG5527
Procladius rufovittatus|BIOUG55141-D03|Kate Perez|BIN Taxonomy Match (Oct 2020)|BOLD:AAG...
Procladius rufovittatus|BIOUG55191-G06|Kate Perez|BIN Taxonomy Match (Oct 2020)|BOLD:AAG...
Procladius rufovittatus|BIOUG55123-B07|Kate Perez|BIN Taxonomy Match (Oct 2020)|BOLD:AAG...
Procladius rufovittatus|ZSM-DIP-33132-F03|Caroline Chimeno|BIN Taxonomy Match (Jan 2022)|BO...
Tanypus kraatzi|LC495091|||BOLD:ACV5726
Tanypus kraatzi|NIESD0221|Ryuhei Ueno|Morphology|BOLD:ACV5726
Tanypus kraatzi|LC050911|||BOLD:ACV5726
Tanypus kraatzi|LC494874|||BOLD:ACV5726
Tanypus kraatzi|LC050912|||BOLD:ACV5726
Tanypus kraatzi|NIESD0418|Ryuhei Ueno|Morphology|BOLD:ACV5726
Tanypus kraatzi|LC494774|||BOLD:ACV5726
Tanypus kraatzi|LC495105|||BOLD:ACV5726
Tanypus kraatzi|LC495113|||BOLD:ACV5726
Tanypus kraatzi|LC495100|||BOLD:ACV5726
Tanypus kraatzi|LC494844|||BOLD:ACV5726
Tanypus kraatzi|LC050918|||BOLD:ACV5726
Tanypus kraatzi|NIESD0485|Ryuhei Ueno|Morphology|BOLD:ACV5726
Tanypus kraatzi|LC050914|||BOLD:ACV5726
Tanypus kraatzi|NIESD0465|Ryuhei Ueno|Morphology|BOLD:ACV5726
Tanypus kraatzi|LC495089|||BOLD:ACV5726
Tanypus kraatzi|LC494796|||BOLD:ACV5726
Tanypus kraatzi|NIESD0487|Ryuhei Ueno|Morphology|BOLD:ACV5726
Tanypus kraatzi|LC050916|||BOLD:ACV5726
Tanypus kraatzi|NIESD0475|Ryuhei Ueno|Morphology|BOLD:ACV5726
Tanypus kraatzi|LC495099|||BOLD:ACV5726
Tanypus kraatzi|LC050917|||BOLD:ACV5726
Tanypus kraatzi|NIESD0477|Ryuhei Ueno|Morphology|BOLD:ACV5726
Tanypus kraatzi|NIESD0493|Ryuhei Ueno|Morphology|BOLD:ACV5726
Tanypus kraatzi|LC050915|||BOLD:ACV5726
Tanypus kraatzi|NIESD0490|Ryuhei Ueno|Morphology|BOLD:ACV5726
Tanypus kraatzi|NIESD0489|Ryuhei Ueno|Morphology|BOLD:ACV5726
Tanypus kraatzi|NIESD0488|Ryuhei Ueno|Morphology|BOLD:ACV5726
Tanypus kraatzi|NIESD0486|Ryuhei Ueno|Morphology|BOLD:ACV5726
Tanypus kraatzi|NIESD0474|Ryuhei Ueno|Morphology|BOLD:ACV5726
Tanypus kraatzi|LC494949|||BOLD:ACV5726
Tanypus kraatzi|NIESD0453|Ryuhei Ueno|Morphology|BOLD:ACV5726
Tanypus kraatzi|LC050913|||BOLD:ACV5726
Tanypus kraatzi|LC494777|||BOLD:ACV5726
Tanypus kraatzi|LC494787|||BOLD:ACV5726
Tanypus kraatzi|LC494981|||BOLD:ACV5726
Tanypus sp. FLT1.5|MW283820|||BOLD:AAV5949
Tanypus neopunctipennis|09BBDIP-1895|Angela Telfer|BIN Taxonomy Match|BOLD:AAL7362
Tanypus neopunctipennis|09BBDIP-1897|Angela Telfer|BIN Taxonomy Match|BOLD:AAL7362
Tanypus neopunctipennis|09BBDIP-1899|Monica R. Young|BOLD ID Engine Manual|BOLD:AAL7362
Tanypus neopunctipennis|09BBDIP-1845|Angela Telfer|BIN Taxonomy Match|BOLD:AAL7362
Tanypus neopunctipennis|09BBDIP-1601|Angela Telfer|BIN Taxonomy Match|BOLD:AAL7362
Tanypus neopunctipennis|09BBDIP-1898|Angela Telfer|BIN Taxonomy Match|BOLD:AAL7362
Tanypus neopunctipennis|09BBDIP-1677|Angela Telfer|BIN Taxonomy Match|BOLD:AAL7362
Tanypus neopunctipennis|BIOUG02271-B04|Kate Perez|BIN Taxonomy Match|BOLD:AAL7362
Tanypus neopunctipennis|BIOUG07931-C09|Angela Telfer|BIN Taxonomy Match|BOLD:AAL7362
Tanypus neopunctipennis|BIOUG08377-E01|Angela Telfer|BIN Taxonomy Match|BOLD:AAL7362
Tanypus neopunctipennis|BIOUG07931-E05|Angela Telfer|BIN Taxonomy Match|BOLD:AAL7362
Tanypus neopunctipennis|BIOUG07932-F02|Angela Telfer|BIN Taxonomy Match|BOLD:AAL7362
Tanypus neopunctipennis|BIOUG07931-F04|Angela Telfer|BIN Taxonomy Match|BOLD:AAL7362
Tanypus neopunctipennis|NIESD0140|Ryuhei Ueno|Morphology|BOLD:AAL7362
Tanypus neopunctipennis|AB838641|||BOLD:AAL7362
Tanypus neopunctipennis|NIESD0223|Ryuhei Ueno|Morphology|BOLD:AAL7362
Tanypus neopunctipennis|NIESD0222|Ryuhei Ueno|Morphology|BOLD:AAL7362
Tanypus neopunctipennis|NIESD0436|Ryuhei Ueno|Morphology|BOLD:AAL7362
Tanypus neopunctipennis|BIOUG07924-C02|Angela Telfer|BIN Taxonomy Match|BOLD:AAL7362
Tanypus neopunctipennis|09BBDIP-2048|Angela Telfer|BIN Taxonomy Match|BOLD:AAL7362
Tanypus neopunctipennis|BIOUG08376-D02|Angela Telfer|BIN Taxonomy Match|BOLD:AAL7362
Tanypus sp. TANYPUS|MW283825|||BOLD:ACI7407
Tanypus|BIOUG06723-B06|Jim M. Moore||BOLD:ACI7407
Tanypus vilipennis|BIOUG27479-A05|Kate Perez|BIN Taxonomy Match|BOLD:ACZ5961
Tanypus vilipennis|BIOUG27331-E03|Kate Perez|BIN Taxonomy Match|BOLD:ACZ5961
Tanypus vilipennis|BIOUG27196-B04|Kate Perez|BIN Taxonomy Match|BOLD:ACZ5961
Tanypus vilipennis|ZMUO.025320|Lauri Paasivirta||BOLD:ACZ5961
Tanypus vilipennis|ZMUO.025319|Lauri Paasivirta||BOLD:ACZ5961
Tanypus vilipennis|BIOUG55116-F04|Kate Perez|BIN Taxonomy Match (Oct 2020)|BOLD:ACZ5961
Tanypus punctipennis|JN887100|||BOLD:ACB5010
Tanypus punctipennis|JN887099|||BOLD:ACB5010
Tanypus punctipennis|JN887098|||BOLD:ACB5010
Tanypus punctipennis|OM974447
Tanypus punctipennis|IM_VR30VII_224|Piotr Gadawski|Morphology|BOLD:ADA5143
Tanypus punctipennis|ZMUO.025270|Lauri Paasivirta||BOLD:ADA5143
Tanypus punctipennis|ZMUO.025269|Lauri Paasivirta||BOLD:ADA5143
Tanypus punctipennis|NHRS-BYWS000000525|Yngve Brodin|morphology|BOLD:ADA5143
Tanypus punctipennis|NHRS-BYWS000000524|Yngve Brodin|morphology|BOLD:ADA5143
Tanypus punctipennis|NHRS-BYWS000000904|Yngve Brodin|morphology|BOLD:ADA5143
Tanypus punctipennis|NHRS-BYWS000000248|Yngve Brodin|morphology|BOLD:ADA5143
Tanypus chinensis|KP902839|||BOLD:ADC7018
Tanypus chinensis|KP902840|||BOLD:ADC7018
Tanypus chinensis|KP902843|||BOLD:ADC7018
Tanypus chinensis|KP902841|||BOLD:ADC7018
Tanypus chinensis|KP902838|||BOLD:ADC7018
Tanypus chinensis|KP902836|||BOLD:ADC7018
Tanypus chinensis|KP902837|||BOLD:ADC7018
Tanypus chinensis|KP902842|||BOLD:ADC7018
Tanypus nakazatoi|NIESD0015|Ryuhei Ueno|Morphology|BOLD:ACQ0045
Tanypus nakazatoi|AB838640|||BOLD:ACQ0045
[illegible]

Tanypus chinensis|KP902842|||BOLD:ADC7018
Tanypus nakazatoi|NIESD0015|Ryuhei Ueno|Morphology|BOLD:ACQ0045
Tanypus nakazatoi|AB838640|||BOLD:ACQ0045
Tanypus nakazatoi|NIESD0347|Ryuhei Ueno|Morphology|BOLD:ACQ0045
Tanypus nakazatoi|NIESD0014|Ryuhei Ueno|Morphology|BOLD:ACQ0045
Tanypus nakazatoi|AB838639|||BOLD:ACQ0045
Tanypus|IM_VR9X14_40|Piotr Gadawski|Morphology|BOLD:AEB0390
Tanypus|IM_PL7V14_279|Piotr Gadawski|Morphology|BOLD:AEB0390
Tanypus|Tanypus_IM_PL7V14_279|Piotr Gadawski|Morphology|BOLD:AEB0390
Tanypus|LA_LSLZ14_19|Piotr Gadawski||BOLD:AEB0390
Tanypus|TANYPUS_IM_LI27VII_148|Piotr Gadawski|Morphology|BOLD:AEB0390
Tanypus kraatzi|Tanypus kraatzi_Wulunguhu 2|Ge Yihao|morphological|BOLD:ACP6608
Tanypus kraatzi|BIOUG15361-B08|Kate Perez|BIN Taxonomy Match|BOLD:ACP6608
Tanypus kraatzi|Tanypus kraatzi_Wulunguhu 3|Ge Yihao|morphological|BOLD:ACP6608
Tanypus kraatzi|Tanypus kraatzi_Wulunguhu 1|Ge Yihao|morphological|BOLD:ACP6608
Tanypus kraatzi|ZMUO.025276|Lauri Paasivirta||BOLD:ACP6608
Tanypus kraatzi|ZMUO.025275|Lauri Paasivirta||BOLD:ACP6608
Tanypus sp. 1 HM-2012|LC494749|||BOLD:AEG8313
Tanypus sp. 1 HM-2012|LC494743|||BOLD:AEG8313
Tanypus sp. 1 HM-2012|LC494745|||BOLD:AEG8313
Tanypus sp. 1 HM-2012|LC494756|||BOLD:AEG8313
Tanypus sp. 1 HM-2012|LC494747|||BOLD:AEG8313
Tanypus sp. 1 HM-2012|LC494744|||BOLD:AEG8313
Tanypus sp. 1 HM-2012|LC494757|||BOLD:AEG8313
Tanypus sp. 1 HM-2012|LC494766|||BOLD:AEG8313
Tanypus sp. 1 HM-2012|LC494767|||BOLD:AEG8313
Tanypus kraatzi|NHRS-BYWS000001072|Yngve Brodin|morphology|BOLD:ADJ7832
Metriocnemus|BC-ZSM-DIP-22488-E09|Dieter Doczkal||BOLD:AAQ2646
Metriocnemus|CCDB24039-B05|Sofia Wiedenbrug|BIN Taxonomy Match|BOLD:AAQ2646
Metriocnemus|CCDB24037-D09|Sofia Wiedenbrug|BIN Taxonomy Match|BOLD:AAQ2646
Metriocnemus sp. 6ES|Finnmark602|Elisabeth Stur||BOLD:AAQ2646
Metriocnemus tristellus|SOE137|Elisabeth Stur||BOLD:AAX4301
Metriocnemus|ZMUO.025922|Lauri Paasivirta||BOLD:ADF0025
Metriocnemus atriclava|ZMUO.026179|Lauri Paasivirta||BOLD:ACP7329
Metriocnemus atriclava|ZMUO.026180|Lauri Paasivirta||BOLD:ACP7329
Metriocnemus|ZMUO.026118|Lauri Paasivirta||BOLD:ACP7329
Metriocnemus atriclava|BIOUG15561-A10|Kate Perez|BIN Taxonomy Match (Aug 2017)|BOLD:ACP7329
Metriocnemus atriclava|ZSM-DIP-33134-F02|Caroline Chimeno|BIN Taxonomy Match (Jan 2022)|BOL...
Metriocnemus|ZMUO.026119|Lauri Paasivirta||BOLD:ACP7329
Metriocnemus atriclava|BIOUG15541-F12|Kate Perez|BIN Taxonomy Match (Aug 2017)|BOLD:ACP7329
Metriocnemus|BIOUG15458-E07|Elisabeth Stur|morphology|BOLD:ACP2624
Metriocnemus|BIOUG15457-A11|Elisabeth Stur|morphology|BOLD:ACP2624
Metriocnemus|BIOUG15455-D01|Elisabeth Stur|morphology|BOLD:ACP2624
Metriocnemus|BIOUG15184-F03|Elisabeth Stur|morphology|BOLD:ACP2624
Metriocnemus|BIOUG15567-G02|Elisabeth Stur|morphology|BOLD:ACP2624
Metriocnemus|BIOUG15071-F11|Elisabeth Stur|morphology|BOLD:ACP2624
Metriocnemus|BIOUG15183-E10|Elisabeth Stur|morphology|BOLD:ACP2624
Metriocnemus|BIOUG15460-D01|Elisabeth Stur|morphology|BOLD:ACP2624
Metriocnemus|BIOUG15454-G07|Elisabeth Stur|morphology|BOLD:ACP2624
Metriocnemus|BIOUG15456-C09|Elisabeth Stur|morphology|BOLD:ACP2624
Metriocnemus|BIOUG15456-G01|Elisabeth Stur|morphology|BOLD:ACP2624
Metriocnemus|BIOUG15543-D03|Elisabeth Stur|morphology|BOLD:ACP2624
Metriocnemus|BIOUG15459-F03|Elisabeth Stur|morphology|BOLD:ACP2624
Metriocnemus|BIOUG15458-B07|Elisabeth Stur|morphology|BOLD:ACP2624
Metriocnemus|BIOUG15459-E03|Elisabeth Stur|morphology|BOLD:ACP2624
Metriocnemus|BIOUG15455-H02|Elisabeth Stur|morphology|BOLD:ACP2624
Metriocnemus|BIOUG15456-C01|Elisabeth Stur|morphology|BOLD:ACP2624
Metriocnemus|BIOUG15458-G05|Elisabeth Stur|morphology|BOLD:ACP2624
Metriocnemus|BIOUG15458-E06|Elisabeth Stur|morphology|BOLD:ACP2624
Metriocnemus|BIOUG15457-G05|Elisabeth Stur|morphology|BOLD:ACP2624
Metriocnemus|BIOUG15184-D12|Elisabeth Stur|morphology|BOLD:ACP2624
Metriocnemus|BIOUG15458-F09|Elisabeth Stur|morphology|BOLD:ACP2624
Metriocnemus|BIOUG15456-E10|Elisabeth Stur|morphology|BOLD:ACP2624
Metriocnemus|BIOUG15541-F02|Elisabeth Stur|morphology|BOLD:ACP2624
Metriocnemus|BC-ZSM-DIP-22491-E04|Dieter Doczkal||BOLD:ACU4447
Metriocnemus|BC-ZSM-DIP-22551-F07|Dieter Doczkal||BOLD:ACU4447
Metriocnemus|BC-ZSM-DIP-22551-C01|Dieter Doczkal||BOLD:ACU4447
Metriocnemus|CCDB24039-F01|Sofia Wiedenbrug|BIN Taxonomy Match|BOLD:ACU4447
Corynoneura|CCDB24228-A11|Sofia Wiedenbrug|BIN Taxonomy Match|BOLD:ACU4872
Corynoneura|CCDB24229-C10|Sofia Wiedenbrug|BIN Taxonomy Match|BOLD:ACU4872
Corynoneura|CCDB24039-F06|Sofia Wiedenbrug|BIN Taxonomy Match|BOLD:ACU4872
Corynoneura|CCDB24229-D11|Sofia Wiedenbrug|BIN Taxonomy Match|BOLD:ACU4872
Corynoneura|CCDB24039-F04|Sofia Wiedenbrug|BIN Taxonomy Match|BOLD:ACU4872
Corynoneura|CCDB24037-C07|Sofia Wiedenbrug|BIN Taxonomy Match|BOLD:ACF8304
Corynoneura|CCDB24039-F05|Sofia Wiedenbrug|BIN Taxonomy Match|BOLD:ACF8304
Corynoneura|BC-ZSM-DIP-22551-D05|Dieter Doczkal||BOLD:ACF8304
Corynoneura|CCDB24228-A12|Sofia Wiedenbrug|BIN Taxonomy Match|BOLD:ACF8304
Corynoneura|BIOUG05400-E06|Kate Perez|BIN Taxonomy Match|BOLD:ACF8304
Corynoneura lobata|ES44|Elisabeth Stur||BOLD:AAD1161
Chironomidae|GBOL03803|Sofia Wiedenbrug|BIN-taxonomy
Corynoneura lobata|ZSM-DIP-33172-C08|Caroline Chimeno|BIN Taxonomy Match (Jan 2022)|BOLD ...
Corynoneura lobata|ZSM-DIP-33143-D01|Caroline Chimeno|BIN Taxonomy Match (Jan 2022)|BOLD ...
Corynoneura lobata|TRD-CH212|Elisabeth Stur||BOLD:AAD1162
Corynoneura lobata|EBAI-Ch151|Elisabeth Stur|morphology|BOLD:AAD1162
Corynoneura lobata|ATNA7|Elisabeth Stur||BOLD:AAD1162
Corynoneura lobata|ES113|Elisabeth Stur||BOLD:AAD1162
Corynoneura lobata|SOE342|Elisabeth Stur||BOLD:AAD1162
Corynoneura lobata|SOE214|Elisabeth Stur||BOLD:AAD1162
Corynoneura lobata|EBAI-Ch150|Elisabeth Stur|morphology|BOLD:AAD1162
Corynoneura lobata|EBAI-Ch034|Elisabeth Stur|morphology|BOLD:AAD1162
Corynoneura lobata|EBAI-Ch152|Elisabeth Stur|morphology|BOLD:AAD1162
Corynoneura lobata|ATNA544|Elisabeth Stur||BOLD:AAD1162
Corynoneura lobata|ATNA6|Elisabeth Stur||BOLD:AAD1162
Corynoneura lobata|ATNA8|Elisabeth Stur||BOLD:AAD1162
Corynoneura lobata|BIOUG05144-F03|Kate Perez|BIN Taxonomy Match|BOLD:AAD1162
Corynoneura lobata|ATNA543|Elisabeth Stur||BOLD:AAD1162
Corynoneura lobata|EBAI-Ch035|Elisabeth Stur|morphology|BOLD:AAD1162
Corynoneura lobata|CH-OSF65|Elisabeth Stur||BOLD:AAD1162
Corynoneura lobata|ATNA161|Elisabeth Stur||BOLD:AAD1162
Corynoneura lobata|ATNA367|Elisabeth Stur||BOLD:AAD1162
Corynoneura lobata|Finnmark72|Elisabeth Stur||BOLD:AAD1162
Corynoneura lobata|Finnmark05|Elisabeth Stur||BOLD:AAD1162
Corynoneura|EBAI-Ch146|Elisabeth Stur|morphology|BOLD:ADG0819

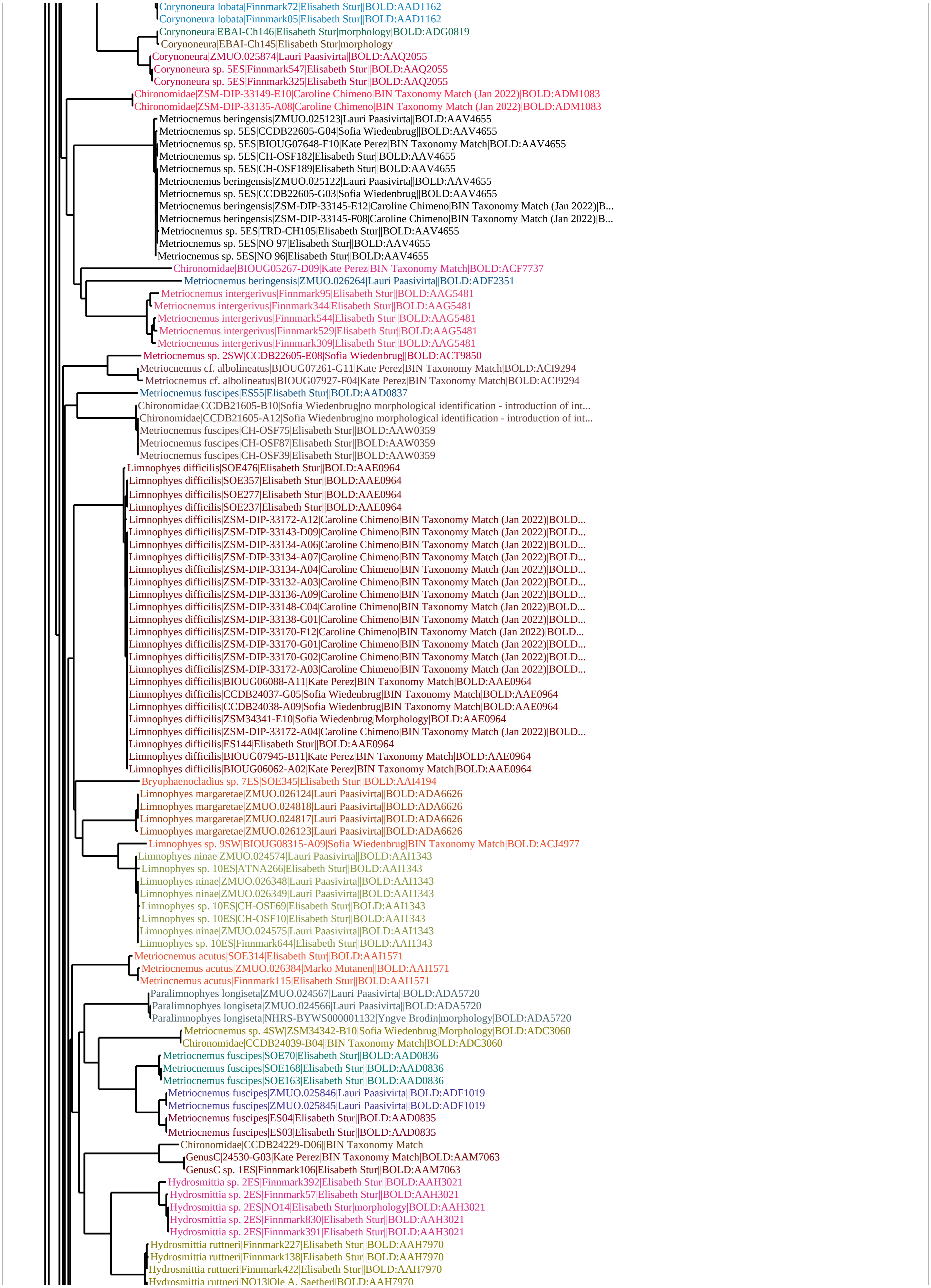
Corynoneura lobata|Finnmark72|Elisabeth Stur||BOLD:AAD1162
Corynoneura lobata|Finnmark05|Elisabeth Stur||BOLD:AAD1162
Corynoneura|EBAI-Ch146|Elisabeth Stur|morphology|BOLD:ADG0819
Corynoneura|EBAI-Ch145|Elisabeth Stur|morphology
Corynoneura|ZMUO.025874|Lauri Paasivirta||BOLD:AAQ2055
Corynoneura sp. 5ES|Finnmark547|Elisabeth Stur||BOLD:AAQ2055
Corynoneura sp. 5ES|Finnmark325|Elisabeth Stur||BOLD:AAQ2055
Chironomidae|ZSM-DIP-33149-E10|Caroline Chimeno|BIN Taxonomy Match (Jan 2022)|BOLD:ADM1083
Chironomidae|ZSM-DIP-33135-A08|Caroline Chimeno|BIN Taxonomy Match (Jan 2022)|BOLD:ADM1083
Metriocnemus beringensis|ZMUO.025123|Lauri Paasivirta||BOLD:AAV4655
Metriocnemus sp. 5ES|CCDB22605-G04|Sofia Wiedenbrug||BOLD:AAV4655
Metriocnemus sp. 5ES|BIOUG07648-F10|Kate Perez|BIN Taxonomy Match|BOLD:AAV4655
Metriocnemus sp. 5ES|CH-OSF182|Elisabeth Stur||BOLD:AAV4655
Metriocnemus sp. 5ES|CH-OSF189|Elisabeth Stur||BOLD:AAV4655
Metriocnemus beringensis|ZMUO.025122|Lauri Paasivirta||BOLD:AAV4655
Metriocnemus sp. 5ES|CCDB22605-G03|Sofia Wiedenbrug||BOLD:AAV4655
Metriocnemus beringensis|ZSM-DIP-33145-E12|Caroline Chimeno|BIN Taxonomy Match (Jan 2022)|B...
Metriocnemus beringensis|ZSM-DIP-33145-F08|Caroline Chimeno|BIN Taxonomy Match (Jan 2022)|B...
Metriocnemus sp. 5ES|TRD-CH105|Elisabeth Stur||BOLD:AAV4655
Metriocnemus sp. 5ES|NO 97|Elisabeth Stur||BOLD:AAV4655
Metriocnemus sp. 5ES|NO 96|Elisabeth Stur||BOLD:AAV4655
Chironomidae|BIOUG05267-D09|Kate Perez|BIN Taxonomy Match|BOLD:ACF7737
Metriocnemus beringensis|ZMUO.026264|Lauri Paasivirta||BOLD:ADF2351
Metriocnemus intergerivus|Finnmark95|Elisabeth Stur||BOLD:AAG5481
Metriocnemus intergerivus|Finnmark344|Elisabeth Stur||BOLD:AAG5481
Metriocnemus intergerivus|Finnmark544|Elisabeth Stur||BOLD:AAG5481
Metriocnemus intergerivus|Finnmark529|Elisabeth Stur||BOLD:AAG5481
Metriocnemus intergerivus|Finnmark309|Elisabeth Stur||BOLD:AAG5481
Metriocnemus sp. 2SW|CCDB22605-E08|Sofia Wiedenbrug||BOLD:ACT9850
Metriocnemus cf. albolineatus|BIOUG07261-G11|Kate Perez|BIN Taxonomy Match|BOLD:ACI9294
Metriocnemus cf. albolineatus|BIOUG07927-F04|Kate Perez|BIN Taxonomy Match|BOLD:ACI9294
Metriocnemus fuscipes|ES55|Elisabeth Stur||BOLD:AAD0837
Chironomidae|CCDB21605-B10|Sofia Wiedenbrug|no morphological identification - introduction of int...
Chironomidae|CCDB21605-A12|Sofia Wiedenbrug|no morphological identification - introduction of int...
Metriocnemus fuscipes|CH-OSF75|Elisabeth Stur||BOLD:AAW0359
Metriocnemus fuscipes|CH-OSF87|Elisabeth Stur||BOLD:AAW0359
Metriocnemus fuscipes|CH-OSF39|Elisabeth Stur||BOLD:AAW0359
Limnophyes difficilis|SOE476|Elisabeth Stur||BOLD:AAE0964
Limnophyes difficilis|SOE357|Elisabeth Stur||BOLD:AAE0964
Limnophyes difficilis|SOE277|Elisabeth Stur||BOLD:AAE0964
Limnophyes difficilis|SOE237|Elisabeth Stur||BOLD:AAE0964
Limnophyes difficilis|ZSM-DIP-33172-A12|Caroline Chimeno|BIN Taxonomy Match (Jan 2022)|BOLD...
Limnophyes difficilis|ZSM-DIP-33143-D09|Caroline Chimeno|BIN Taxonomy Match (Jan 2022)|BOLD...
Limnophyes difficilis|ZSM-DIP-33134-A06|Caroline Chimeno|BIN Taxonomy Match (Jan 2022)|BOLD...
Limnophyes difficilis|ZSM-DIP-33134-A07|Caroline Chimeno|BIN Taxonomy Match (Jan 2022)|BOLD...
Limnophyes difficilis|ZSM-DIP-33134-A04|Caroline Chimeno|BIN Taxonomy Match (Jan 2022)|BOLD...
Limnophyes difficilis|ZSM-DIP-33132-A03|Caroline Chimeno|BIN Taxonomy Match (Jan 2022)|BOLD...
Limnophyes difficilis|ZSM-DIP-33136-A09|Caroline Chimeno|BIN Taxonomy Match (Jan 2022)|BOLD...
Limnophyes difficilis|ZSM-DIP-33148-C04|Caroline Chimeno|BIN Taxonomy Match (Jan 2022)|BOLD...
Limnophyes difficilis|ZSM-DIP-33138-G01|Caroline Chimeno|BIN Taxonomy Match (Jan 2022)|BOLD...
Limnophyes difficilis|ZSM-DIP-33170-F12|Caroline Chimeno|BIN Taxonomy Match (Jan 2022)|BOLD...
Limnophyes difficilis|ZSM-DIP-33170-G01|Caroline Chimeno|BIN Taxonomy Match (Jan 2022)|BOLD...
Limnophyes difficilis|ZSM-DIP-33170-G02|Caroline Chimeno|BIN Taxonomy Match (Jan 2022)|BOLD...
Limnophyes difficilis|ZSM-DIP-33172-A03|Caroline Chimeno|BIN Taxonomy Match (Jan 2022)|BOLD...
Limnophyes difficilis|BIOUG06088-A11|Kate Perez|BIN Taxonomy Match|BOLD:AAE0964
Limnophyes difficilis|CCDB24037-G05|Sofia Wiedenbrug|BIN Taxonomy Match|BOLD:AAE0964
Limnophyes difficilis|CCDB24038-A09|Sofia Wiedenbrug|BIN Taxonomy Match|BOLD:AAE0964
Limnophyes difficilis|ZSM34341-E10|Sofia Wiedenbrug|Morphology|BOLD:AAE0964
Limnophyes difficilis|ZSM-DIP-33172-A04|Caroline Chimeno|BIN Taxonomy Match (Jan 2022)|BOLD...
Limnophyes difficilis|ES144|Elisabeth Stur||BOLD:AAE0964
Limnophyes difficilis|BIOUG07945-B11|Kate Perez|BIN Taxonomy Match|BOLD:AAE0964
Limnophyes difficilis|BIOUG06062-A02|Kate Perez|BIN Taxonomy Match|BOLD:AAE0964
Bryophaenocladius sp. 7ES|SOE345|Elisabeth Stur||BOLD:AAI4194
Limnophyes margaretae|ZMUO.026124|Lauri Paasivirta||BOLD:ADA6626
Limnophyes margaretae|ZMUO.024818|Lauri Paasivirta||BOLD:ADA6626
Limnophyes margaretae|ZMUO.024817|Lauri Paasivirta||BOLD:ADA6626
Limnophyes margaretae|ZMUO.026123|Lauri Paasivirta||BOLD:ADA6626
Limnophyes sp. 9SW|BIOUG08315-A09|Sofia Wiedenbrug|BIN Taxonomy Match|BOLD:ACJ4977
Limnophyes ninae|ZMUO.024574|Lauri Paasivirta||BOLD:AAI1343
Limnophyes sp. 10ES|ATNA266|Elisabeth Stur||BOLD:AAI1343
Limnophyes ninae|ZMUO.026348|Lauri Paasivirta||BOLD:AAI1343
Limnophyes ninae|ZMUO.026349|Lauri Paasivirta||BOLD:AAI1343
Limnophyes sp. 10ES|CH-OSF69|Elisabeth Stur||BOLD:AAI1343
Limnophyes sp. 10ES|CH-OSF10|Elisabeth Stur||BOLD:AAI1343
Limnophyes ninae|ZMUO.024575|Lauri Paasivirta||BOLD:AAI1343
Limnophyes sp. 10ES|Finnmark644|Elisabeth Stur||BOLD:AAI1343
Metriocnemus acutus|SOE314|Elisabeth Stur||BOLD:AAI1571
Metriocnemus acutus|ZMUO.026384|Marko Mutanen||BOLD:AAI1571
Metriocnemus acutus|Finnmark115|Elisabeth Stur||BOLD:AAI1571
Paralimnophyes longiseta|ZMUO.024567|Lauri Paasivirta||BOLD:ADA5720
Paralimnophyes longiseta|ZMUO.024566|Lauri Paasivirta||BOLD:ADA5720
Paralimnophyes longiseta|NHRS-BYWS000001132|Yngve Brodin|morphology|BOLD:ADA5720
Metriocnemus sp. 4SW|ZSM34342-B10|Sofia Wiedenbrug|Morphology|BOLD:ADC3060
Chironomidae|CCDB24039-B04||BIN Taxonomy Match|BOLD:ADC3060
Metriocnemus fuscipes|SOE70|Elisabeth Stur||BOLD:AAD0836
Metriocnemus fuscipes|SOE168|Elisabeth Stur||BOLD:AAD0836
Metriocnemus fuscipes|SOE163|Elisabeth Stur||BOLD:AAD0836
Metriocnemus fuscipes|ZMUO.025846|Lauri Paasivirta||BOLD:ADF1019
Metriocnemus fuscipes|ZMUO.025845|Lauri Paasivirta||BOLD:ADF1019
Metriocnemus fuscipes|ES04|Elisabeth Stur||BOLD:AAD0835
Metriocnemus fuscipes|ES03|Elisabeth Stur||BOLD:AAD0835
Chironomidae|CCDB24229-D06||BIN Taxonomy Match
GenusC|24530-G03|Kate Perez|BIN Taxonomy Match|BOLD:AAM7063
GenusC sp. 1ES|Finnmark106|Elisabeth Stur||BOLD:AAM7063
Hydrosmittia sp. 2ES|Finnmark392|Elisabeth Stur||BOLD:AAH3021
Hydrosmittia sp. 2ES|Finnmark57|Elisabeth Stur||BOLD:AAH3021
Hydrosmittia sp. 2ES|NO14|Elisabeth Stur|morphology|BOLD:AAH3021
Hydrosmittia sp. 2ES|Finnmark830|Elisabeth Stur||BOLD:AAH3021
Hydrosmittia sp. 2ES|Finnmark391|Elisabeth Stur||BOLD:AAH3021
Hydrosmittia ruttneri|Finnmark227|Elisabeth Stur||BOLD:AAH7970
Hydrosmittia ruttneri|Finnmark138|Elisabeth Stur||BOLD:AAH7970
Hydrosmittia ruttneri|Finnmark422|Elisabeth Stur||BOLD:AAH7970
Hydrosmittia ruttneri|NO13|Ole A. Saether||BOLD:AAH7970

Hydrosmittia ruttneri|Finnmark138|Elisabeth Stur||BOLD:AAH7970
Hydrosmittia ruttneri|Finnmark422|Elisabeth Stur||BOLD:AAH7970
Hydrosmittia ruttneri|NO13|Ole A. Saether||BOLD:AAH7970
Hydrosmittia ruttneri|NO12|Ole A. Saether||BOLD:AAH7970
Hydrosmittia ruttneri|Finnmark254|Elisabeth Stur||BOLD:AAH7970
Hydrosmittia ruttneri|Finnmark587|Elisabeth Stur||BOLD:AAH7970
Metriocnemus|SOE125|Elisabeth Stur||BOLD:AAI1569
Metriocnemus eurynotus|BIOUG15762-C04|Kate Perez|BIN Taxonomy Match|BOLD:AAB8860
Metriocnemus eurynotus|ATNA199|Elisabeth Stur||BOLD:AAB8860
Metriocnemus eurynotus|NHRS-BYWS000000549|Yngve Brodin|morphology|BOLD:AAB8860
Metriocnemus eurynotus|BIOUG15765-E08|Kate Perez|BIN Taxonomy Match|BOLD:AAB8860
Metriocnemus eurynotus|BIOUG15948-E11|Kate Perez|BIN Taxonomy Match|BOLD:AAB8860
Metriocnemus eurynotus|BIOUG16198-H06|Kate Perez|BIN Taxonomy Match|BOLD:AAB8860
Metriocnemus eurynotus|BIOUG15675-H10|Kate Perez|BIN Taxonomy Match|BOLD:AAB8860
Metriocnemus eurynotus|BIOUG04261-E08|Kate Perez|BIN Taxonomy Match|BOLD:AAB8860
Metriocnemus eurynotus|BIOUG15654-D11|Kate Perez|BIN Taxonomy Match|BOLD:AAB8860
Metriocnemus eurynotus|BIOUG15769-C08|Kate Perez|BIN Taxonomy Match|BOLD:AAB8860
Metriocnemus eurynotus|BIOUG15762-E12|Kate Perez|BIN Taxonomy Match|BOLD:AAB8860
Metriocnemus eurynotus|BIOUG15718-B12|Kate Perez|BIN Taxonomy Match|BOLD:AAB8860
Metriocnemus ursinus|MZ004970|||BOLD:AEH5653
Metriocnemus|CCDB24038-E12|Sofia Wiedenbrug|BIN Taxonomy Match|BOLD:ACU4659
Metriocnemus picipes|BC-ZSM-DIP-22488-C03|Sofia Wiedenbrug|BIN Taxonomy Match|BOLD:AAI1570
Metriocnemus picipes|CCDB24039-G01|Sofia Wiedenbrug|BIN Taxonomy Match|BOLD:AAI1570
Metriocnemus picipes|CCDB24038-B12|Sofia Wiedenbrug|BIN Taxonomy Match|BOLD:AAI1570
Metriocnemus picipes|CCDB24039-G04|Sofia Wiedenbrug|BIN Taxonomy Match|BOLD:AAI1570
Metriocnemus picipes|BC-ZSM-DIP-22488-C04|Sofia Wiedenbrug|BIN Taxonomy Match|BOLD:AAI1570
Metriocnemus picipes|CCDB24039-G03|Sofia Wiedenbrug|BIN Taxonomy Match|BOLD:AAI1570
Metriocnemus picipes|CCDB24039-B03|Sofia Wiedenbrug|BIN Taxonomy Match|BOLD:AAI1570
Metriocnemus picipes|ZSM-DIP-33172-E05|Caroline Chimeno|BIN Taxonomy Match (Jan 2022)|BOLD...
Metriocnemus picipes|ZSM34342-D09|Sofia Wiedenbrug|Morphology|BOLD:AAI1570
Metriocnemus picipes|CCDB24038-C02|Sofia Wiedenbrug|BIN Taxonomy Match|BOLD:AAI1570
Metriocnemus picipes|ZSM34342-B06|Sofia Wiedenbrug|Morphology|BOLD:AAI1570
Metriocnemus picipes|ZSM34342-A09|Sofia Wiedenbrug|Morphology|BOLD:AAI1570
Metriocnemus picipes|CCDB24038-C01|Sofia Wiedenbrug|BIN Taxonomy Match|BOLD:AAI1570
Metriocnemus picipes|CCDB24039-F03|Sofia Wiedenbrug|BIN Taxonomy Match|BOLD:AAI1570
Metriocnemus picipes|CCDB24038-D02|Sofia Wiedenbrug|BIN Taxonomy Match|BOLD:AAI1570
Metriocnemus|SOE309|Elisabeth Stur||BOLD:AAI1570
Metriocnemus picipes|BIOUG15455-H11|Kate Perez|BIN Taxonomy Match|BOLD:AAI1572
Metriocnemus picipes|BIOUG15071-G11|Kate Perez|BIN Taxonomy Match|BOLD:AAI1572
Metriocnemus picipes|BIOUG15459-D01|Kate Perez|BIN Taxonomy Match|BOLD:AAI1572
Metriocnemus picipes|BIOUG42704-B09|Kate Perez|BIN Taxonomy Match below Phylum (Jun 2019)|B...
Metriocnemus picipes|BIOUG42963-G06|Kate Perez|BIN Taxonomy Match below Phylum (Jun 2019)|B...
Metriocnemus picipes|BIOUG15675-C01|Kate Perez|BIN Taxonomy Match|BOLD:AAI1572
Metriocnemus picipes|BIOUG15793-A01|Kate Perez|BIN Taxonomy Match|BOLD:AAI1572
Metriocnemus picipes|BIOUG15454-A11|Kate Perez|BIN Taxonomy Match|BOLD:AAI1572
Metriocnemus picipes|BIOUG15458-F04|Kate Perez|BIN Taxonomy Match|BOLD:AAI1572
Metriocnemus picipes|BIOUG15455-A05|Kate Perez|BIN Taxonomy Match|BOLD:AAI1572
Chironomidae|BIOUG05144-E09|Kate Perez|no morphological identification - introduction of interim ...
Metriocnemus picipes|BIOUG15457-C01|Kate Perez|BIN Taxonomy Match|BOLD:AAI1572
Metriocnemus picipes|BIOUG15454-C02|Kate Perez|BIN Taxonomy Match|BOLD:AAI1572
Metriocnemus picipes|BIOUG15457-A01|Kate Perez|BIN Taxonomy Match|BOLD:AAI1572
Metriocnemus picipes|BIOUG17065-B04|Kate Perez|BIN Taxonomy Match|BOLD:AAI1572
Metriocnemus picipes|BIOUG15201-H11|Kate Perez|BIN Taxonomy Match|BOLD:AAI1572
Metriocnemus picipes|BIOUG15183-F05|Kate Perez|BIN Taxonomy Match|BOLD:AAI1572
Metriocnemus picipes|BIOUG15966-B08|Kate Perez|BIN Taxonomy Match|BOLD:AAI1572
Metriocnemus picipes|ZMUO.024572|Lauri Paasivirta||BOLD:AAI1572
Metriocnemus picipes|BIOUG15459-A09|Kate Perez|BIN Taxonomy Match|BOLD:AAI1572
Metriocnemus picipes|BIOUG15201-H06|Kate Perez|BIN Taxonomy Match|BOLD:AAI1572
Metriocnemus picipes|BIOUG15185-E12|Kate Perez|BIN Taxonomy Match|BOLD:AAI1572
Metriocnemus picipes|TRD-CH118|Elisabeth Stur||BOLD:AAI1572
Metriocnemus picipes|BIOUG15538-A04|Kate Perez|BIN Taxonomy Match|BOLD:AAI1572
Metriocnemus picipes|BIOUG43074-C07|Kate Perez|BIN Taxonomy Match below Phylum (Jun 2019)|B...
Metriocnemus picipes|BIOUG15792-G01|Kate Perez|BIN Taxonomy Match|BOLD:AAI1572
Metriocnemus picipes|BIOUG15808-G01|Kate Perez|BIN Taxonomy Match|BOLD:AAI1572
Metriocnemus picipes|BIOUG15454-B09|Kate Perez|BIN Taxonomy Match|BOLD:AAI1572
Metriocnemus picipes|BIOUG15798-H04|Kate Perez|BIN Taxonomy Match|BOLD:AAI1572
Metriocnemus picipes|BIOUG15457-A12|Kate Perez|BIN Taxonomy Match|BOLD:AAI1572
Metriocnemus picipes|BIOUG15457-C07|Kate Perez|BIN Taxonomy Match|BOLD:AAI1572
Metriocnemus picipes|BIOUG15948-F08|Kate Perez|BIN Taxonomy Match|BOLD:AAI1572
Metriocnemus picipes|BIOUG42927-D02|Kate Perez|BIN Taxonomy Match below Phylum (Jun 2019)|B...
Metriocnemus picipes|BIOUG42700-D10|Kate Perez|BIN Taxonomy Match below Phylum (Jun 2019)|B...
Metriocnemus picipes|BIOUG42668-A08|Kate Perez|BIN Taxonomy Match below Phylum (Jun 2019)|B...
Metriocnemus picipes|BIOUG42713-E10|Kate Perez|BIN Taxonomy Match below Phylum (Jun 2019)|B...
Metriocnemus picipes|ZSM-DIP-33131-H01|Caroline Chimeno|BIN Taxonomy Match (Jan 2022)|BOLD...
Metriocnemus picipes|BIOUG15460-C08|Kate Perez|BIN Taxonomy Match|BOLD:AAI1572
Metriocnemus picipes|BIOUG15459-C08|Kate Perez|BIN Taxonomy Match|BOLD:AAI1572
Metriocnemus picipes|BIOUG15459-B11|Kate Perez|BIN Taxonomy Match|BOLD:AAI1572
Metriocnemus picipes|BIOUG15454-B04|Kate Perez|BIN Taxonomy Match|BOLD:AAI1572
Metriocnemus picipes|BIOUG15538-F06|Kate Perez|BIN Taxonomy Match|BOLD:AAI1572
Metriocnemus picipes|BIOUG15954-D12|Kate Perez|BIN Taxonomy Match|BOLD:AAI1572
Chironomidae|BIOUG04795-D04|Kate Perez|no morphological identification - introduction of interim ...
Metriocnemus picipes|BIOUG15463-G09|Kate Perez|BIN Taxonomy Match|BOLD:AAI1572
Metriocnemus picipes|RIN_CH08|Mona Renate Saursaunet|BOLD Identification engine|BOLD:AAI1572
Metriocnemus picipes|BIOUG42964-A02|Kate Perez|BIN Taxonomy Match below Phylum (Jun 2019)|B...
Metriocnemus picipes|ZSM-DIP-33141-G08|Caroline Chimeno|BIN Taxonomy Match (Jan 2022)|BOLD...
Chironomidae|CCDB24039-B06|Sofia Wiedenbrug|no morphological identification - introduction of int...
Metriocnemus picipes|BIOUG15807-E04|Kate Perez|BIN Taxonomy Match|BOLD:AAI1572
Metriocnemus picipes|BIOUG15458-A10|Kate Perez|BIN Taxonomy Match|BOLD:AAI1572
Metriocnemus picipes|BIOUG15201-G10|Kate Perez|BIN Taxonomy Match|BOLD:AAI1572
Metriocnemus picipes|BIOUG15806-A03|Kate Perez|BIN Taxonomy Match|BOLD:AAI1572
Metriocnemus picipes|BIOUG15454-G09|Kate Perez|BIN Taxonomy Match|BOLD:AAI1572
Metriocnemus picipes|BIOUG15458-B03|Kate Perez|BIN Taxonomy Match|BOLD:AAI1572
Metriocnemus picipes|BIOUG15457-A03|Kate Perez|BIN Taxonomy Match|BOLD:AAI1572
Metriocnemus picipes|BIOUG15460-B05|Kate Perez|BIN Taxonomy Match|BOLD:AAI1572
Metriocnemus picipes|BIOUG15455-A01|Kate Perez|BIN Taxonomy Match|BOLD:AAI1572
Metriocnemus picipes|BIOUG15967-C06|Kate Perez|BIN Taxonomy Match|BOLD:AAI1572
Metriocnemus picipes|BIOUG15947-G06|Kate Perez|BIN Taxonomy Match|BOLD:AAI1572
Metriocnemus picipes|BIOUG15183-C04|Kate Perez|BIN Taxonomy Match|BOLD:AAI1572
Metriocnemus picipes|BIOUG15564-F11|Kate Perez|BIN Taxonomy Match|BOLD:AAI1572
Metriocnemus picipes|BIOUG15795-B08|Kate Perez|BIN Taxonomy Match|BOLD:AAI1572
Metriocnemus picipes|BIOUG15806-D03|Kate Perez|BIN Taxonomy Match|BOLD:AAI1572
Metriocnemus picipes|BIOUG15456-A10|Kate Perez|BIN Taxonomy Match|BOLD:AAI1572
Metriocnemus picipes|CH-oik56|Elisabeth Stur||BOLD:AAI1572

Metriocnemus picipes|BIOUG15795-D06|Kate Perez|BIN Taxonomy Match|BOLD:AAI1572
Metriocnemus picipes|BIOUG15806-D03|Kate Perez|BIN Taxonomy Match|BOLD:AAI1572
Metriocnemus picipes|BIOUG15456-A10|Kate Perez|BIN Taxonomy Match|BOLD:AAI1572
Metriocnemus picipes|CH-eik56|Elisabeth Stur||BOLD:AAI1572
Metriocnemus picipes|BIOUG15458-G02|Kate Perez|BIN Taxonomy Match|BOLD:AAI1572
Metriocnemus picipes|BIOUG17022-B11|Kate Perez|BIN Taxonomy Match|BOLD:AAI1572
Metriocnemus picipes|BIOUG15183-F01|Kate Perez|BIN Taxonomy Match|BOLD:AAI1572
Metriocnemus picipes|BIOUG15966-C05|Kate Perez|BIN Taxonomy Match|BOLD:AAI1572
Metriocnemus picipes|CH-eik80|Elisabeth Stur||BOLD:AAI1572
Metriocnemus picipes|BIOUG04261-F09|Kate Perez|BIN Taxonomy Match|BOLD:AAI1572
Metriocnemus picipes|BIOUG15808-F06|Kate Perez|BIN Taxonomy Match|BOLD:AAI1572
Metriocnemus picipes|BIOUG15544-C03|Kate Perez|BIN Taxonomy Match|BOLD:AAI1572
Metriocnemus picipes|BIOUG15963-B08|Kate Perez|BIN Taxonomy Match|BOLD:AAI1572
Metriocnemus picipes|BIOUG15720-F06|Kate Perez|BIN Taxonomy Match|BOLD:AAI1572
Chironomidae|CCDB24228-F12|Sofia Wiedenbrug|no morphological identification - introduction of int...
Metriocnemus picipes|BIOUG15949-F08|Kate Perez|BIN Taxonomy Match|BOLD:AAI1572
Metriocnemus picipes|BIOUG42930-D11|Kate Perez|BIN Taxonomy Match below Phylum (Jun 2019)|B...
Metriocnemus picipes|BIOUG42700-E11|Kate Perez|BIN Taxonomy Match below Phylum (Jun 2019)|B...
Metriocnemus picipes|BIOUG42666-C07|Kate Perez|BIN Taxonomy Match below Phylum (Jun 2019)|B...
Metriocnemus picipes|BIOUG43076-F06|Kate Perez|BIN Taxonomy Match below Phylum (Jun 2019)|B...
Metriocnemus picipes|BIOUG42747-F03|Kate Perez|BIN Taxonomy Match below Phylum (Jun 2019)|B...
Chironomidae|BIOUG05102-F10|Kate Perez|no morphological identification - introduction of interim ...
Chironomidae|CCDB24037-F03|Sofia Wiedenbrug|no morphological identification - introduction of int...
Chironomidae|CCDB24038-D01|Sofia Wiedenbrug|no morphological identification - introduction of int...
Chironomidae|BC-ZSM-DIP-24208-C02|Dieter Doczkal|no morphological identification - introduction o...
Chironomidae|BC-ZSM-DIP-22491-D10|Dieter Doczkal|no morphological identification - introduction o...
Chironomidae|CCDB24229-F05|Sofia Wiedenbrug|no morphological identification - introduction of int...
Chironomidae|CCDB24229-D03|Sofia Wiedenbrug|no morphological identification - introduction of int...
Chironomidae|CCDB24229-G01|Sofia Wiedenbrug|no morphological identification - introduction of int...
Chironomidae|CCDB24037-D11|Sofia Wiedenbrug|no morphological identification - introduction of int...
Chironomidae|BC-ZSM-DIP-22491-D09|Dieter Doczkal|no morphological identification - introduction o...
Metriocnemus picipes|BIOUG15953-F05|Kate Perez|BIN Taxonomy Match|BOLD:AAI1572
Chironomidae|BIOUG05104-E04|Kate Perez|no morphological identification - introduction of interim ...
Chironomidae|BIOUG05106-F10|Kate Perez|no morphological identification - introduction of interim ...
Metriocnemus picipes|BIOUG15454-A02|Kate Perez|BIN Taxonomy Match|BOLD:AAI1572
Metriocnemus picipes|BIOUG15464-A03|Kate Perez|BIN Taxonomy Match|BOLD:AAI1572
Metriocnemus picipes|TRD-CH116|Elisabeth Stur||BOLD:AAI1572
Metriocnemus picipes|CH-eik58|Elisabeth Stur||BOLD:AAI1572
Metriocnemus picipes|BIOUG42755-G04|Kate Perez|BIN Taxonomy Match below Phylum (Jun 2019)|B...
Metriocnemus picipes|BIOUG15795-E09|Kate Perez|BIN Taxonomy Match|BOLD:AAI1572
Metriocnemus picipes|BIOUG15454-E12|Kate Perez|BIN Taxonomy Match|BOLD:AAI1572
Metriocnemus picipes|BIOUG15184-F11|Kate Perez|BIN Taxonomy Match|BOLD:AAI1572
Metriocnemus picipes|BIOUG15183-F07|Kate Perez|BIN Taxonomy Match|BOLD:AAI1572
Metriocnemus picipes|BIOUG15456-F03|Kate Perez|BIN Taxonomy Match|BOLD:AAI1572
Metriocnemus picipes|BIOUG15852-E09|Kate Perez|BIN Taxonomy Match|BOLD:AAI1572
Metriocnemus picipes|BIOUG15457-C04|Kate Perez|BIN Taxonomy Match|BOLD:AAI1572
Metriocnemus picipes|BIOUG15458-G08|Kate Perez|BIN Taxonomy Match|BOLD:AAI1572
Metriocnemus picipes|BIOUG15455-D09|Kate Perez|BIN Taxonomy Match|BOLD:AAI1572
Metriocnemus picipes|BIOUG16198-G07|Kate Perez|BIN Taxonomy Match|BOLD:AAI1572
Metriocnemus picipes|BIOUG15963-H06|Kate Perez|BIN Taxonomy Match|BOLD:AAI1572
Metriocnemus picipes|BIOUG15454-D03|Kate Perez|BIN Taxonomy Match|BOLD:AAI1572
Metriocnemus picipes|BIOUG15201-H03|Kate Perez|BIN Taxonomy Match|BOLD:AAI1572
Metriocnemus picipes|BIOUG15963-H09|Kate Perez|BIN Taxonomy Match|BOLD:AAI1572
Metriocnemus picipes|BIOUG15460-D10|Kate Perez|BIN Taxonomy Match|BOLD:AAI1572
Metriocnemus picipes|BIOUG15458-A03|Kate Perez|BIN Taxonomy Match|BOLD:AAI1572
Metriocnemus picipes|BIOUG15455-D02|Kate Perez|BIN Taxonomy Match|BOLD:AAI1572
Metriocnemus picipes|BIOUG15565-C11|Kate Perez|BIN Taxonomy Match|BOLD:AAI1572
Metriocnemus picipes|BIOUG15460-G12|Kate Perez|BIN Taxonomy Match|BOLD:AAI1572
Metriocnemus picipes|BIOUG15459-E08|Kate Perez|BIN Taxonomy Match|BOLD:AAI1572
Metriocnemus picipes|BIOUG15459-H11|Kate Perez|BIN Taxonomy Match|BOLD:AAI1572
Metriocnemus picipes|BIOUG15460-B04|Kate Perez|BIN Taxonomy Match|BOLD:AAI1572
Metriocnemus picipes|BIOUG15950-B04|Kate Perez|BIN Taxonomy Match|BOLD:AAI1572
Metriocnemus picipes|BIOUG42932-F04|Kate Perez|BIN Taxonomy Match below Phylum (Jun 2019)|B...
Metriocnemus picipes|BIOUG15794-H11|Kate Perez|BIN Taxonomy Match|BOLD:AAI1572
Metriocnemus picipes|BIOUG15948-D07|Kate Perez|BIN Taxonomy Match|BOLD:AAI1572
Metriocnemus picipes|BIOUG42666-F01|Kate Perez|BIN Taxonomy Match below Phylum (Jun 2019)|B...
Metriocnemus picipes|BIOUG42964-A11|Kate Perez|BIN Taxonomy Match below Phylum (Jun 2019)|B...
Chironomidae|BIOUG16894-A04|Kate Perez|no morphological identification - introduction of interim ...
Metriocnemus picipes|BIOUG15791-F11|Kate Perez|BIN Taxonomy Match|BOLD:AAI1572
Metriocnemus picipes|BIOUG16330-A09|Kate Perez|BIN Taxonomy Match|BOLD:AAI1572
Metriocnemus picipes|Finnmark02|Elisabeth Stur||BOLD:AAI1572
Metriocnemus picipes|CH-OSF03|Elisabeth Stur||BOLD:AAI1572
Metriocnemus picipes|RIN_CH95|Mona Renate Saursaunet|BOLD Identification engine|BOLD:AAI1572
Metriocnemus picipes|CH-eik108|Elisabeth Stur||BOLD:AAI1572
Metriocnemus picipes|ZMUO.024573|Lauri Paasivirta||BOLD:AAI1572
Metriocnemus picipes|BIOUG15458-E01|Kate Perez|BIN Taxonomy Match|BOLD:AAI1572
Metriocnemus picipes|BIOUG42787-E08|Kate Perez|BIN Taxonomy Match below Phylum (Jun 2019)|B...
Metriocnemus picipes|BIOUG15454-F08|Kate Perez|BIN Taxonomy Match|BOLD:AAI1572
Metriocnemus picipes|BIOUG15184-B09|Kate Perez|BIN Taxonomy Match|BOLD:AAI1572
Metriocnemus picipes|BIOUG15185-C11|Kate Perez|BIN Taxonomy Match|BOLD:AAI1572
Metriocnemus picipes|BIOUG15807-E10|Kate Perez|BIN Taxonomy Match|BOLD:AAI1572
Metriocnemus picipes|BIOUG15792-C04|Kate Perez|BIN Taxonomy Match|BOLD:AAI1572
Metriocnemus picipes|BIOUG15561-A08|Kate Perez|BIN Taxonomy Match|BOLD:AAI1572
Metriocnemus picipes|BIOUG15963-G06|Kate Perez|BIN Taxonomy Match|BOLD:AAI1572
Metriocnemus picipes|BIOUG15963-D11|Kate Perez|BIN Taxonomy Match|BOLD:AAI1572
Metriocnemus picipes|BIOUG15454-A04|Kate Perez|BIN Taxonomy Match|BOLD:AAI1572
Metriocnemus picipes|BIOUG15457-G08|Kate Perez|BIN Taxonomy Match|BOLD:AAI1572
Metriocnemus picipes|BIOUG15459-G08|Kate Perez|BIN Taxonomy Match|BOLD:AAI1572
Metriocnemus picipes|BIOUG15456-A11|Kate Perez|BIN Taxonomy Match|BOLD:AAI1572
Metriocnemus picipes|BIOUG15456-C03|Kate Perez|BIN Taxonomy Match|BOLD:AAI1572
Metriocnemus picipes|BIOUG16477-D04|Kate Perez|BIN Taxonomy Match|BOLD:AAI1572
Metriocnemus picipes|BIOUG15454-A03|Kate Perez|BIN Taxonomy Match|BOLD:AAI1572
Metriocnemus picipes|BIOUG15809-F06|Kate Perez|BIN Taxonomy Match|BOLD:AAI1572
Metriocnemus picipes|BIOUG15457-H02|Kate Perez|BIN Taxonomy Match|BOLD:AAI1572
Metriocnemus picipes|ATNA362|Elisabeth Stur||BOLD:AAI1572
Metriocnemus picipes|BIOUG15201-G09|Kate Perez|BIN Taxonomy Match|BOLD:AAI1572
Metriocnemus picipes|BIOUG15459-G09|Kate Perez|BIN Taxonomy Match|BOLD:AAI1572
Metriocnemus picipes|BIOUG04261-E09|Kate Perez|BIN Taxonomy Match|BOLD:AAI1572
Metriocnemus picipes|BIOUG15950-E07|Kate Perez|BIN Taxonomy Match|BOLD:AAI1572
Metriocnemus picipes|BIOUG15538-F08|Kate Perez|BIN Taxonomy Match|BOLD:AAI1572
Metriocnemus picipes|BIOUG15458-B05|Kate Perez|BIN Taxonomy Match|BOLD:AAI1572
Metriocnemus picipes|BIOUG15458-E02|Kate Perez|BIN Taxonomy Match|BOLD:AAI1572
Metriocnemus picipes|BIOUG04433-H09|Kate Perez|BIN Taxonomy Match|BOLD:AAI1572
[illegible]

Metriocnemus picipes|BIOUG15458-B05|Kate Perez|BIN Taxonomy Match|BOLD:AAI1572
Metriocnemus picipes|BIOUG15458-E02|Kate Perez|BIN Taxonomy Match|BOLD:AAI1572
Metriocnemus picipes|BIOUG04433-H09|Kate Perez|BIN Taxonomy Match|BOLD:AAI1572
Metriocnemus picipes|BIOUG15594-E10|Kate Perez|BIN Taxonomy Match|BOLD:AAI1572
Metriocnemus picipes|BIOUG15542-E10|Kate Perez|BIN Taxonomy Match|BOLD:AAI1572
Metriocnemus picipes|BIOUG15458-A01|Kate Perez|BIN Taxonomy Match|BOLD:AAI1572
Metriocnemus picipes|BIOUG15454-B02|Kate Perez|BIN Taxonomy Match|BOLD:AAI1572
Metriocnemus picipes|BIOUG15183-A12|Kate Perez|BIN Taxonomy Match|BOLD:AAI1572
Metriocnemus picipes|BIOUG15454-A06|Kate Perez|BIN Taxonomy Match|BOLD:AAI1572
Metriocnemus picipes|BIOUG15457-H05|Kate Perez|BIN Taxonomy Match|BOLD:AAI1572
Metriocnemus picipes|BIOUG15455-H01|Kate Perez|BIN Taxonomy Match|BOLD:AAI1572
Metriocnemus picipes|BIOUG15457-F11|Kate Perez|BIN Taxonomy Match|BOLD:AAI1572
Metriocnemus picipes|BIOUG15966-E07|Kate Perez|BIN Taxonomy Match|BOLD:AAI1572
Metriocnemus picipes|BIOUG15459-E11|Kate Perez|BIN Taxonomy Match|BOLD:AAI1572
Metriocnemus picipes|BIOUG15456-E08|Kate Perez|BIN Taxonomy Match|BOLD:AAI1572
Metriocnemus picipes|BIOUG15458-H08|Kate Perez|BIN Taxonomy Match|BOLD:AAI1572
Metriocnemus picipes|BIOUG16594-H01|Kate Perez|BIN Taxonomy Match|BOLD:AAI1572
Metriocnemus picipes|BIOUG15458-E05|Kate Perez|BIN Taxonomy Match|BOLD:AAI1572
Metriocnemus picipes|BIOUG15458-H07|Kate Perez|BIN Taxonomy Match|BOLD:AAI1572
Metriocnemus picipes|BIOUG15456-G06|Kate Perez|BIN Taxonomy Match|BOLD:AAI1572
Metriocnemus picipes|BIOUG15184-A12|Kate Perez|BIN Taxonomy Match|BOLD:AAI1572
Metriocnemus picipes|BIOUG15966-H01|Kate Perez|BIN Taxonomy Match|BOLD:AAI1572
Metriocnemus picipes|BIOUG15808-D03|Kate Perez|BIN Taxonomy Match|BOLD:AAI1572
Metriocnemus picipes|BIOUG15809-H04|Kate Perez|BIN Taxonomy Match|BOLD:AAI1572
Metriocnemus picipes|BIOUG15460-C09|Kate Perez|BIN Taxonomy Match|BOLD:AAI1572
Metriocnemus picipes|BIOUG15185-D10|Kate Perez|BIN Taxonomy Match|BOLD:AAI1572
Metriocnemus picipes|BIOUG15795-F06|Kate Perez|BIN Taxonomy Match|BOLD:AAI1572
Metriocnemus picipes|BIOUG15454-C04|Kate Perez|BIN Taxonomy Match|BOLD:AAI1572
Metriocnemus picipes|BIOUG15460-H05|Kate Perez|BIN Taxonomy Match|BOLD:AAI1572
Metriocnemus picipes|BIOUG15460-H07|Kate Perez|BIN Taxonomy Match|BOLD:AAI1572
Metriocnemus picipes|BIOUG15459-D07|Kate Perez|BIN Taxonomy Match|BOLD:AAI1572
Metriocnemus picipes|BIOUG15459-F11|Kate Perez|BIN Taxonomy Match|BOLD:AAI1572
Metriocnemus picipes|BIOUG15459-H10|Kate Perez|BIN Taxonomy Match|BOLD:AAI1572
Metriocnemus picipes|BIOUG15460-A01|Kate Perez|BIN Taxonomy Match|BOLD:AAI1572
Metriocnemus picipes|BIOUG15454-G11|Kate Perez|BIN Taxonomy Match|BOLD:AAI1572
Metriocnemus picipes|BIOUG16596-G06|Kate Perez|BIN Taxonomy Match|BOLD:AAI1572
Metriocnemus picipes|BIOUG15563-F08|Kate Perez|BIN Taxonomy Match|BOLD:AAI1572
Metriocnemus picipes|BIOUG15454-A08|Kate Perez|BIN Taxonomy Match|BOLD:AAI1572
Metriocnemus picipes|BIOUG15460-F12|Kate Perez|BIN Taxonomy Match|BOLD:AAI1572
Metriocnemus picipes|BIOUG15185-C09|Kate Perez|BIN Taxonomy Match|BOLD:AAI1572
Metriocnemus picipes|BIOUG15184-F02|Kate Perez|BIN Taxonomy Match|BOLD:AAI1572
Metriocnemus picipes|BIOUG16433-H01|Kate Perez|BIN Taxonomy Match|BOLD:AAI1572
Metriocnemus picipes|BIOUG15185-D11|Kate Perez|BIN Taxonomy Match|BOLD:AAI1572
Metriocnemus picipes|BIOUG15455-H09|Kate Perez|BIN Taxonomy Match|BOLD:AAI1572
Metriocnemus picipes|BIOUG15454-A10|Kate Perez|BIN Taxonomy Match|BOLD:AAI1572
Metriocnemus picipes|BIOUG16426-G07|Kate Perez|BIN Taxonomy Match|BOLD:AAI1572
Metriocnemus picipes|BIOUG15541-B01|Kate Perez|BIN Taxonomy Match|BOLD:AAI1572
Metriocnemus picipes|BIOUG15455-C03|Kate Perez|BIN Taxonomy Match|BOLD:AAI1572
Metriocnemus picipes|BIOUG15455-A03|Kate Perez|BIN Taxonomy Match|BOLD:AAI1572
Metriocnemus picipes|BIOUG15543-B06|Kate Perez|BIN Taxonomy Match|BOLD:AAI1572
Metriocnemus picipes|BIOUG15457-F02|Kate Perez|BIN Taxonomy Match|BOLD:AAI1572
Metriocnemus picipes|BIOUG15799-H03|Kate Perez|BIN Taxonomy Match|BOLD:AAI1572
Metriocnemus picipes|BIOUG16205-E03|Kate Perez|BIN Taxonomy Match|BOLD:AAI1572
Metriocnemus picipes|BIOUG16596-D12|Kate Perez|BIN Taxonomy Match|BOLD:AAI1572
Metriocnemus picipes|BIOUG15456-F09|Kate Perez|BIN Taxonomy Match|BOLD:AAI1572
Metriocnemus picipes|BIOUG15454-A05|Kate Perez|BIN Taxonomy Match|BOLD:AAI1572
Metriocnemus picipes|BIOUG16328-A06|Kate Perez|BIN Taxonomy Match|BOLD:AAI1572
Metriocnemus picipes|BIOUG15456-H06|Kate Perez|BIN Taxonomy Match|BOLD:AAI1572
Metriocnemus picipes|BIOUG15455-A07|Kate Perez|BIN Taxonomy Match|BOLD:AAI1572
Metriocnemus picipes|BIOUG15455-D04|Kate Perez|BIN Taxonomy Match|BOLD:AAI1572
Metriocnemus picipes|BIOUG15183-C12|Kate Perez|BIN Taxonomy Match|BOLD:AAI1572
Metriocnemus picipes|BIOUG15950-C03|Kate Perez|BIN Taxonomy Match|BOLD:AAI1572
Metriocnemus picipes|BIOUG15457-D08|Kate Perez|BIN Taxonomy Match|BOLD:AAI1572
Metriocnemus picipes|BIOUG15454-F02|Kate Perez|BIN Taxonomy Match|BOLD:AAI1572
Metriocnemus picipes|BIOUG15183-B10|Kate Perez|BIN Taxonomy Match|BOLD:AAI1572
Metriocnemus picipes|BIOUG15950-H05|Kate Perez|BIN Taxonomy Match|BOLD:AAI1572
Metriocnemus picipes|BIOUG15948-G09|Kate Perez|BIN Taxonomy Match|BOLD:AAI1572
Metriocnemus picipes|BIOUG15187-B04|Kate Perez|BIN Taxonomy Match|BOLD:AAI1572
Metriocnemus|CCDB24038-B11|Sofia Wiedenbrug|BIN Taxonomy Match|BOLD:ACC1758
Metriocnemus|BIOUG04217-E06|Kate Perez|BIN Taxonomy Match (Aug 2017)|BOLD:ACC1758
Metriocnemus sp. 2ES|Finnmark01|Elisabeth Stur||BOLD:AAM5434
Metriocnemus sp. 2ES|Finnmark530|Elisabeth Stur||BOLD:AAM5434
Metriocnemus eurynotus|BIOUG15459-B01|Kate Perez|Tree based identification
Metriocnemus eurynotus|BIOUG15542-D07|Kate Perez|BIN Taxonomy Match|BOLD:AAB8863
Metriocnemus eurynotus|BIOUG15543-F02|Kate Perez|BIN Taxonomy Match|BOLD:AAB8863
Metriocnemus eurynotus|BIOUG15538-A03|Kate Perez|BIN Taxonomy Match|BOLD:AAB8863
Metriocnemus eurynotus|BIOUG15458-C08|Kate Perez|BIN Taxonomy Match|BOLD:AAB8863
Metriocnemus eurynotus|BIOUG15457-C05|Kate Perez|BIN Taxonomy Match|BOLD:AAB8863
Metriocnemus eurynotus|BIOUG15454-B10|Kate Perez|BIN Taxonomy Match|BOLD:AAB8863
Metriocnemus eurynotus|BIOUG15460-G05|Kate Perez|BIN Taxonomy Match|BOLD:AAB8863
Metriocnemus eurynotus|BIOUG17025-F11|Kate Perez|BIN Taxonomy Match|BOLD:AAB8863
Metriocnemus eurynotus|BIOUG15460-A02|Kate Perez|Tree based identification
Metriocnemus eurynotus|BIOUG15459-A11|Kate Perez|BIN Taxonomy Match|BOLD:AAB8863
Metriocnemus eurynotus|BIOUG15541-H09|Kate Perez|BIN Taxonomy Match|BOLD:AAB8863
Metriocnemus eurynotus|BIOUG15458-H09|Kate Perez|BIN Taxonomy Match|BOLD:AAB8863
Metriocnemus eurynotus|BIOUG15455-E04|Kate Perez|BIN Taxonomy Match|BOLD:AAB8863
Metriocnemus eurynotus|BIOUG15545-F05|Kate Perez|BIN Taxonomy Match|BOLD:AAB8863
Metriocnemus eurynotus|BIOUG15793-A03|Kate Perez|BIN Taxonomy Match|BOLD:AAB8863
Metriocnemus eurynotus|BIOUG15460-H09|Kate Perez|BIN Taxonomy Match|BOLD:AAB8863
Metriocnemus eurynotus|BIOUG15652-G09|Kate Perez|BIN Taxonomy Match|BOLD:AAB8863
Metriocnemus eurynotus|BIOUG15456-F02|Kate Perez|BIN Taxonomy Match|BOLD:AAB8863
Metriocnemus eurynotus|BIOUG15457-G11|Kate Perez|BIN Taxonomy Match|BOLD:AAB8863
Metriocnemus eurynotus|BIOUG15457-G12|Kate Perez|BIN Taxonomy Match|BOLD:AAB8863
Metriocnemus eurynotus|BIOUG15457-D06|Kate Perez|BIN Taxonomy Match|BOLD:AAB8863
Metriocnemus eurynotus|BIOUG15455-E11|Kate Perez|BIN Taxonomy Match|BOLD:AAB8863
Metriocnemus eurynotus|BIOUG15184-G04|Kate Perez|BIN Taxonomy Match|BOLD:AAB8863
Metriocnemus eurynotus|BIOUG15460-A11|Kate Perez|BIN Taxonomy Match|BOLD:AAB8863
Metriocnemus eurynotus|BIOUG15457-A08|Kate Perez|BIN Taxonomy Match|BOLD:AAB8863
Metriocnemus eurynotus|BIOUG04261-E10|Kate Perez|BIN Taxonomy Match|BOLD:AAB8863
Metriocnemus eurynotus|BIOUG15458-A05|Kate Perez|BIN Taxonomy Match|BOLD:AAB8863
Metriocnemus eurynotus|BIOUG15456-H11|Kate Perez|BIN Taxonomy Match|BOLD:AAB8863
Metriocnemus eurynotus|BIOUG15454-E08|Kate Perez|BIN Taxonomy Match|BOLD:AAB8863
Metriocnemus eurynotus|BIOUG15459-A03|Kate Perez|BIN Taxonomy Match|BOLD:AAB8863

Metriocnemus eurynotus|BIOUG15456-H11|Kate Perez|BIN Taxonomy Match|BOLD:AAB8863
Metriocnemus eurynotus|BIOUG15454-E08|Kate Perez|BIN Taxonomy Match|BOLD:AAB8863
Metriocnemus eurynotus|BIOUG15459-A03|Kate Perez|BIN Taxonomy Match|BOLD:AAB8863
Metriocnemus eurynotus|BIOUG15460-C03|Kate Perez|BIN Taxonomy Match|BOLD:AAB8863
Metriocnemus eurynotus|BIOUG15652-G02|Kate Perez|BIN Taxonomy Match|BOLD:AAB8863
Metriocnemus eurynotus|BIOUG15201-G12|Kate Perez|BIN Taxonomy Match|BOLD:AAB8863
Metriocnemus eurynotus|BIOUG15460-A05|Kate Perez|BIN Taxonomy Match|BOLD:AAB8863
Metriocnemus eurynotus|BIOUG15460-F06|Kate Perez|BIN Taxonomy Match|BOLD:AAB8863
Metriocnemus eurynotus|BIOUG16596-A05|Kate Perez|BIN Taxonomy Match|BOLD:AAB8863
Metriocnemus eurynotus|BIOUG15463-F08|Kate Perez|BIN Taxonomy Match|BOLD:AAB8863
Metriocnemus eurynotus|BIOUG15561-F12|Kate Perez|BIN Taxonomy Match|BOLD:AAB8863
Metriocnemus eurynotus|BIOUG15456-C08|Kate Perez|BIN Taxonomy Match|BOLD:AAB8863
Metriocnemus eurynotus|BIOUG15456-D06|Kate Perez|BIN Taxonomy Match|BOLD:AAB8863
Metriocnemus eurynotus|BIOUG15953-D01|Kate Perez|BIN Taxonomy Match|BOLD:AAB8863
Metriocnemus eurynotus|BIOUG15454-A09|Kate Perez|BIN Taxonomy Match|BOLD:AAB8863
Metriocnemus eurynotus|BIOUG15455-G06|Kate Perez|BIN Taxonomy Match|BOLD:AAB8863
Metriocnemus eurynotus|BIOUG15456-A01|Kate Perez|BIN Taxonomy Match|BOLD:AAB8863
Metriocnemus eurynotus|BIOUG15456-C04|Kate Perez|BIN Taxonomy Match|BOLD:AAB8863
Metriocnemus eurynotus|BIOUG15456-E09|Kate Perez|BIN Taxonomy Match|BOLD:AAB8863
Metriocnemus eurynotus|BIOUG15464-E07|Kate Perez|BIN Taxonomy Match|BOLD:AAB8863
Metriocnemus eurynotus|BIOUG15455-C02|Kate Perez|BIN Taxonomy Match|BOLD:AAB8863
Metriocnemus eurynotus|BIOUG15459-E06|Kate Perez|BIN Taxonomy Match|BOLD:AAB8863
Metriocnemus eurynotus|BIOUG15456-E03|Kate Perez|BIN Taxonomy Match|BOLD:AAB8863
Metriocnemus eurynotus|BIOUG15456-F04|Kate Perez|BIN Taxonomy Match|BOLD:AAB8863
Metriocnemus eurynotus|BIOUG15454-H10|Kate Perez|BIN Taxonomy Match|BOLD:AAB8863
Metriocnemus eurynotus|BIOUG15457-E01|Kate Perez|BIN Taxonomy Match|BOLD:AAB8863
Metriocnemus eurynotus|BIOUG15653-D09|Kate Perez|BIN Taxonomy Match|BOLD:AAB8863
Metriocnemus eurynotus|BIOUG15654-A04|Kate Perez|BIN Taxonomy Match|BOLD:AAB8863
Metriocnemus eurynotus|BIOUG15459-E09|Kate Perez|BIN Taxonomy Match|BOLD:AAB8863
Metriocnemus eurynotus|BIOUG15537-D03|Kate Perez|BIN Taxonomy Match|BOLD:AAB8863
Metriocnemus eurynotus|BIOUG15460-C06|Kate Perez|BIN Taxonomy Match|BOLD:AAB8863
Metriocnemus eurynotus|BIOUG15542-A02|Kate Perez|BIN Taxonomy Match|BOLD:AAB8863
Metriocnemus eurynotus|BIOUG15201-G02|Kate Perez|BIN Taxonomy Match|BOLD:AAB8863
Metriocnemus eurynotus|BIOUG15457-G07|Kate Perez|BIN Taxonomy Match|BOLD:AAB8863
Metriocnemus eurynotus|BIOUG15458-C03|Kate Perez|BIN Taxonomy Match|BOLD:AAB8863
Metriocnemus eurynotus|BIOUG15456-E11|Kate Perez|BIN Taxonomy Match|BOLD:AAB8863
Metriocnemus eurynotus|BIOUG15457-A04|Kate Perez|BIN Taxonomy Match|BOLD:AAB8863
Metriocnemus eurynotus|BIOUG15457-E11|Kate Perez|BIN Taxonomy Match|BOLD:AAB8863
Metriocnemus eurynotus|BIOUG15183-E07|Kate Perez|BIN Taxonomy Match|BOLD:AAB8863
Metriocnemus eurynotus|BIOUG15458-A04|Kate Perez|BIN Taxonomy Match|BOLD:AAB8863
Metriocnemus eurynotus|ES187|Elisabeth Stur||BOLD:AAB8863
Metriocnemus eurynotus|BIOUG15459-F09|Kate Perez|BIN Taxonomy Match|BOLD:AAB8863
Metriocnemus eurynotus|BIOUG15459-B08|Kate Perez|BIN Taxonomy Match|BOLD:AAB8863
Metriocnemus eurynotus|BIOUG15459-C11|Kate Perez|BIN Taxonomy Match|BOLD:AAB8863
Metriocnemus eurynotus|BIOUG15460-G01|Kate Perez|BIN Taxonomy Match|BOLD:AAB8863
Metriocnemus eurynotus|ZMUO.024563|Lauri Paasivirta||BOLD:AAB8863
Metriocnemus eurynotus|BIOUG15563-G09|Kate Perez|BIN Taxonomy Match|BOLD:AAB8863
Metriocnemus eurynotus|ZMUO.024564|Lauri Paasivirta||BOLD:AAB8863
Metriocnemus eurynotus|Finnmark696|Elisabeth Stur||BOLD:AAB8863
Metriocnemus eurynotus|CH-OSF159|Elisabeth Stur||BOLD:AAB8863
Metriocnemus eurynotus|TRD-CH11|Elisabeth Stur||BOLD:AAB8863
Metriocnemus eurynotus|CH-OSF04|Elisabeth Stur||BOLD:AAB8863
Metriocnemus eurynotus|CH-OSF25|Elisabeth Stur||BOLD:AAB8863
Metriocnemus eurynotus|CH-OSF111|Elisabeth Stur||BOLD:AAB8863
Metriocnemus eurynotus|BIOUG15650-A04|Kate Perez|BIN Taxonomy Match|BOLD:AAB8863
Metriocnemus eurynotus|BIOUG15539-G12|Kate Perez|BIN Taxonomy Match|BOLD:AAB8863
Metriocnemus eurynotus|BIOUG04366-G09|Kate Perez|BIN Taxonomy Match|BOLD:AAB8863
Metriocnemus eurynotus|BIOUG15564-E11|Kate Perez|BIN Taxonomy Match|BOLD:AAB8863
Metriocnemus eurynotus|BIOUG15964-A09|Kate Perez|BIN Taxonomy Match|BOLD:AAB8863
Metriocnemus eurynotus|BIOUG15454-E03|Kate Perez|BIN Taxonomy Match|BOLD:AAB8863
Metriocnemus eurynotus|BIOUG15460-A04|Kate Perez|BIN Taxonomy Match|BOLD:AAB8863
Metriocnemus eurynotus|BIOUG15455-F11|Kate Perez|BIN Taxonomy Match|BOLD:AAB8863
Metriocnemus eurynotus|BIOUG15459-G04|Kate Perez|BIN Taxonomy Match|BOLD:AAB8863
Metriocnemus eurynotus|BIOUG15459-B02|Kate Perez|BIN Taxonomy Match|BOLD:AAB8863
Metriocnemus eurynotus|BIOUG04261-D10|Kate Perez|BIN Taxonomy Match|BOLD:AAB8863
Metriocnemus eurynotus|BIOUG15455-H07|Kate Perez|BIN Taxonomy Match|BOLD:AAB8863
Metriocnemus eurynotus|BIOUG15459-F01|Kate Perez|BIN Taxonomy Match|BOLD:AAB8863
Metriocnemus eurynotus|BIOUG15460-A12|Kate Perez|BIN Taxonomy Match|BOLD:AAB8863
Metriocnemus eurynotus|CH-OSF62|Elisabeth Stur||BOLD:AAB8863
Metriocnemus eurynotus|BIOUG15536-B08|Kate Perez|BIN Taxonomy Match|BOLD:AAB8863
Metriocnemus eurynotus|NO46|Elisabeth Stur||BOLD:AAB8863
Metriocnemus eurynotus|BIOUG15967-B01|Kate Perez|BIN Taxonomy Match|BOLD:AAB8863
Metriocnemus eurynotus|BIOUG15455-F05|Kate Perez|BIN Taxonomy Match|BOLD:AAB8863
Metriocnemus eurynotus|BIOUG15458-H02|Kate Perez|BIN Taxonomy Match|BOLD:AAB8863
Metriocnemus eurynotus|BIOUG15201-H04|Kate Perez|BIN Taxonomy Match|BOLD:AAB8863
Metriocnemus eurynotus|BIOUG15185-C03|Kate Perez|BIN Taxonomy Match|BOLD:AAB8863
Metriocnemus eurynotus|BIOUG15796-D06|Kate Perez|BIN Taxonomy Match|BOLD:AAB8863
Metriocnemus eurynotus|BIOUG15457-G03|Kate Perez|BIN Taxonomy Match|BOLD:AAB8861
Metriocnemus eurynotus|BIOUG15458-C06|Kate Perez|BIN Taxonomy Match|BOLD:AAB8861
Metriocnemus eurynotus|BIOUG15457-F05|Kate Perez|BIN Taxonomy Match|BOLD:AAB8861
Metriocnemus eurynotus|BIOUG15455-D07|Kate Perez|BIN Taxonomy Match|BOLD:AAB8861
Metriocnemus eurynotus|BIOUG15456-C12|Kate Perez|BIN Taxonomy Match|BOLD:AAB8861
Metriocnemus eurynotus|BIOUG15456-H01|Kate Perez|BIN Taxonomy Match|BOLD:AAB8861
Metriocnemus eurynotus|BIOUG15456-F11|Kate Perez|BIN Taxonomy Match|BOLD:AAB8861
Metriocnemus eurynotus|BIOUG15460-E09|Kate Perez|BIN Taxonomy Match|BOLD:AAB8861
Metriocnemus eurynotus|BIOUG15540-G02|Kate Perez|BIN Taxonomy Match|BOLD:AAB8861
Metriocnemus eurynotus|BIOUG15456-B03|Kate Perez|BIN Taxonomy Match|BOLD:AAB8861
Metriocnemus eurynotus|BIOUG15852-E10|Kate Perez|BIN Taxonomy Match|BOLD:AAB8861
Metriocnemus eurynotus|BIOUG15456-A07|Kate Perez|BIN Taxonomy Match|BOLD:AAB8861
Metriocnemus eurynotus|BIOUG15460-B11|Kate Perez|BIN Taxonomy Match|BOLD:AAB8861
Metriocnemus eurynotus|ES204|Elisabeth Stur||BOLD:AAB8861
Metriocnemus eurynotus|BIOUG15460-B10|Kate Perez|BIN Taxonomy Match|BOLD:AAB8861
Metriocnemus eurynotus|ES167|Elisabeth Stur||BOLD:AAB8861
Metriocnemus eurynotus|ES143|Elisabeth Stur||BOLD:AAB8861
Metriocnemus eurynotus|CH-OSF26|Elisabeth Stur||BOLD:AAB8861
Metriocnemus eurynotus|NO45|Elisabeth Stur||BOLD:AAB8861
Metriocnemus eurynotus|BIOUG15071-G07|Kate Perez|BIN Taxonomy Match|BOLD:ACK3387
Metriocnemus eurynotus|BIOUG15455-F06|Kate Perez|BIN Taxonomy Match|BOLD:ACK3387
Metriocnemus eurynotus|BIOUG15565-E09|Kate Perez|BIN Taxonomy Match|BOLD:ACK3387
Metriocnemus eurynotus|BIOUG15458-D12|Kate Perez|BIN Taxonomy Match|BOLD:ACK3387
Metriocnemus eurynotus|BIOUG15183-B01|Kate Perez|BIN Taxonomy Match|BOLD:ACK3387
Metriocnemus eurynotus|TRD-CH12|Elisabeth Stur||BOLD:ACK3387
Metriocnemus eurynotus|BIN_CH77|Mona Renate Saursaunet|BOLD Identification engine|BOLD:ACK3387

[illegible]
Metriocnemus eurynotus|BIOUG15183-B01|Kate Perez|BIN Taxonomy Match|BOLD:ACK3387
Metriocnemus eurynotus|TRD-CH12|Elisabeth Stur||BOLD:ACK3387
Metriocnemus eurynotus|RIN_CH77|Mona Renate Saursaunet|BOLD Identification engine|BOLD:ACK3387
Metriocnemus eurynotus|BIOUG15458-B06|Kate Perez|BIN Taxonomy Match|BOLD:ACK3387
Metriocnemus eurynotus|BIOUG15597-D04|Kate Perez|BIN Taxonomy Match|BOLD:ACK3387
Metriocnemus eurynotus|BIOUG15457-E10|Kate Perez|BIN Taxonomy Match|BOLD:ACK3387
Metriocnemus eurynotus|BIOUG15454-H02|Kate Perez|BIN Taxonomy Match|BOLD:ACK3387
Metriocnemus eurynotus|BIOUG15465-G02|Kate Perez|BIN Taxonomy Match|BOLD:ACK3387
Metriocnemus eurynotus|BIOUG15459-G01|Kate Perez|BIN Taxonomy Match|BOLD:ACK3387
Metriocnemus eurynotus|ES205|Elisabeth Stur||BOLD:AAA9436
Orthocladiinae|GBOL03748|Sofia Wiedenbrug||BOLD:AAA9436
Metriocnemus eurynotus|ES208|Elisabeth Stur||BOLD:AAA9436
Metriocnemus eurynotus|ES207|Elisabeth Stur||BOLD:AAA9436
Metriocnemus eurynotus|ES206|Elisabeth Stur||BOLD:AAA9436
Metriocnemus eurynotus|ES179|Elisabeth Stur||BOLD:AAA9436
Metriocnemus eurynotus|ES178|Elisabeth Stur||BOLD:AAA9436
Orthocladiinae|CCDB24037-D12|Sofia Wiedenbrug|BIN Taxonomy Match|BOLD:AAA9437
Metriocnemus eurynotus|ES180|Elisabeth Stur||BOLD:AAA9437
Metriocnemus|ZMUO.025878|Lauri Paasivirta||BOLD:AAB8862
Metriocnemus NHRS sp. B|NHRS-BYWS000001015|Yngve Brodin|morphology|BOLD:AAB8862
Metriocnemus eurynotus|SOE203|Elisabeth Stur||BOLD:AAB8862
Metriocnemus eurynotus|BIOUG16854-G03|Kate Perez|BIN Taxonomy Match|BOLD:AAB8862
Metriocnemus eurynotus|SOE225|Elisabeth Stur||BOLD:AAB8862
Metriocnemus NHRS sp. B|NHRS-BYWS000001063|Yngve Brodin|morphology|BOLD:AAB8862
Metriocnemus eurynotus|SOE226|Elisabeth Stur||BOLD:AAB8862
Metriocnemus eurynotus|CH-OSF156|Elisabeth Stur||BOLD:AAB8862
Metriocnemus|ZMUO.025879|Lauri Paasivirta||BOLD:AAB8862
Metriocnemus eurynotus|SOE218|Elisabeth Stur||BOLD:AAB8862
Metriocnemus eurynotus|ZSM34343-C10|Sofia Wiedenbrug|Morphology|BOLD:AAB8862
Metriocnemus eurynotus|SOE210|Elisabeth Stur||BOLD:AAB8862
Metriocnemus eurynotus|SOE202|Elisabeth Stur||BOLD:AAB8862
Metriocnemus eurynotus|ZSM34343-H02|Sofia Wiedenbrug|Morphology|BOLD:AAB8862
Metriocnemus eurynotus|BC-ZSM-DIP-22488-E05|Dieter Doczkal||BOLD:AAB8862
Metriocnemus eurynotus|Finnmark299|Elisabeth Stur||BOLD:AAB8862
Pseudosmittia danconai|ATNA573|Elisabeth Stur||BOLD:ACX6300
Pseudosmittia danconai|CCDB22605-C03|Sofia Wiedenbrug||BOLD:ACU0423
Camptocladius stercorarius|ZMUO.025114|Lauri Paasivirta||BOLD:ACG4429
Hydrosmittia|CCDB24038-E06|Sofia Wiedenbrug|BIN Taxonomy Match|BOLD:ACD3856
Hydrosmittia|CCDB24038-E05|Sofia Wiedenbrug|BIN Taxonomy Match|BOLD:ACD3856
Chironomidae|BIOUG55426-B05|Stefan Schmidt|BIN Taxonomy Match (Sep 2020)|BOLD:ADX0025
Chironomidae|BIOUG42708-A05|Kate Perez|BOLD ID Engine Manual below Phylum (Jul 2019)|BOLD ...
Camptocladius stercorarius|ZSM-DIP-33137-H01|Caroline Chimeno|BIN Taxonomy Match (Jan 2022)...
Camptocladius stercorarius|ZSM-DIP-33128-G03|Caroline Chimeno|BIN Taxonomy Match (Jan 2022)...
Camptocladius stercorarius|ZSM-DIP-33144-D06|Caroline Chimeno|BIN Taxonomy Match (Jan 2022)...
Camptocladius stercorarius|ZSM-DIP-33144-H10|Caroline Chimeno|BIN Taxonomy Match (Jan 2022)...
Camptocladius stercorarius|BIOUG36735-D06|Kate Perez|BIN Taxonomy Match (May 2018)|BOLD:...
Camptocladius stercorarius|ZSM-DIP-33147-F01|Caroline Chimeno|BIN Taxonomy Match (Jan 2022)...
Camptocladius stercorarius|ZSM-DIP-33133-B09|Caroline Chimeno|BIN Taxonomy Match (Jan 2022)...
Camptocladius stercorarius|ZSM-DIP-33136-H04|Caroline Chimeno|BIN Taxonomy Match (Jan 2022)...
Camptocladius stercorarius|ZSM34341-H07|Sofia Wiedenbrug|Morphology|BOLD:AAN5341
Camptocladius stercorarius|ZSM-DIP-33146-C08|Caroline Chimeno|BIN Taxonomy Match (Jan 2022)...
Camptocladius stercorarius|BIOUG17023-C01|Kate Perez|BIN Taxonomy Match|BOLD:AAN5341
Camptocladius stercorarius|ZSM-DIP-33137-H05|Caroline Chimeno|BIN Taxonomy Match (Jan 2022)...
Camptocladius stercorarius|ZSM-DIP-33138-D10|Caroline Chimeno|BIN Taxonomy Match (Jan 2022)...
Camptocladius stercorarius|NHRS-BYWS000001047|Yngve Brodin|morphology|BOLD:AAN5341
Camptocladius stercorarius|ZSM-DIP-33144-H11|Caroline Chimeno|BIN Taxonomy Match (Jan 2022)...
Camptocladius stercorarius|NHRS-BYWS000001016|Yngve Brodin|morphology|BOLD:AAN5341
Camptocladius stercorarius|BIOUG06070-D12|Kate Perez|BIN Taxonomy Match|BOLD:AAN5341
Camptocladius stercorarius|BIOUG42707-F02|Kate Perez|BIN Taxonomy Match below Phylum (Jun 2019...
Camptocladius stercorarius|ZSM-DIP-33141-H01|Caroline Chimeno|BIN Taxonomy Match (Jan 2022)...
Camptocladius stercorarius|ZSM-DIP-33136-G08|Caroline Chimeno|BIN Taxonomy Match (Jan 2022)...
Camptocladius stercorarius|ZSM-DIP-33136-H05|Caroline Chimeno|BIN Taxonomy Match (Jan 2022)...
Camptocladius stercorarius|ZSM-DIP-33140-G10|Caroline Chimeno|BIN Taxonomy Match (Jan 2022)...
Camptocladius stercorarius|ZSM-DIP-33140-H03|Caroline Chimeno|BIN Taxonomy Match (Jan 2022)...
Camptocladius stercorarius|ZSM-DIP-33144-H09|Caroline Chimeno|BIN Taxonomy Match (Jan 2022)...
Camptocladius stercorarius|TRD-CH360|Elisabeth Stur|morphology|BOLD:AAN5341
Camptocladius stercorarius|ZSM-DIP-33147-E11|Caroline Chimeno|BIN Taxonomy Match (Jan 2022)...
Camptocladius stercorarius|BIOUG36846-E06|Kate Perez|BIN Taxonomy Match (Jul 2018)|BOLD:...
Camptocladius stercorarius|ZMUO.025115|Lauri Paasivirta||BOLD:AAN5341
Camptocladius stercorarius|BIOUG07567-D05|Kate Perez|BIN Taxonomy Match|BOLD:AAN5341
Camptocladius stercorarius|BIOUG16711-H05|Kate Perez|BIN Taxonomy Match|BOLD:AAN5341
Camptocladius stercorarius|ZSM-DIP-33170-A02|Caroline Chimeno|BIN Taxonomy Match (Jan 2022)...
Camptocladius stercorarius|BIOUG08140-F12|Kate Perez|BIN Taxonomy Match|BOLD:AAN5341
Metriocnemus beringensis|SOE41|Elisabeth Stur||BOLD:AAX4300
Rheosmittia spinicornis|ATNA526|Elisabeth Stur||BOLD:ACX4713
Rheosmittia spinicornis|TRD-CH408|Elisabeth Stur|morphology|BOLD:ACX4713
Bryophaenocladius pr. vernalis|Finnmark698|Elisabeth Stur
Bryophaenocladius pr. vernalis|Finnmark717|Elisabeth Stur||BOLD:AAQ2042
Bryophaenocladius pr. vernalis|Finnmark572|Elisabeth Stur||BOLD:AAQ2042
Pseudosmittia|BIOUG06274-G04|Kate Perez|BIN Taxonomy Match|BOLD:ACG8007
Pseudosmittia obtusa|ZSM-DIP-33172-A07|Caroline Chimeno|BIN Taxonomy Match (Jan 2022)|BOLD...
Pseudosmittia obtusa|ZSM-DIP-33148-C06|Caroline Chimeno|BIN Taxonomy Match (Jan 2022)|BOLD...
Pseudosmittia|BIOUG06274-D07|Kate Perez|BIN Taxonomy Match|BOLD:ACG8007
Pseudosmittia|BIOUG06208-G12|Kate Perez|BIN Taxonomy Match|BOLD:ACG8007
Pseudosmittia trilobata|RIN_CH09|Elisabeth Stur|morphology|BOLD:ACK6625
Pseudosmittia trilobata|TRD-CH387|Elisabeth Stur|morphology|BOLD:ACK6625
Pseudosmittia trilobata|ZMUO.024741|Lauri Paasivirta||BOLD:ACK6625
Pseudosmittia trilobata|ZMUO.024740|Lauri Paasivirta||BOLD:ACK6625
Pseudosmittia|BIOUG42779-B09|Kate Perez|BOLD ID Engine Manual below Phylum (Jul 2019)|BOLD...
Pseudosmittia|BIOUG15255-C12|Kate Perez|BIN Taxonomy Match|BOLD:ACP4407
Pseudosmittia trilobata|PSE_TRI_IM_VI8X14_60|Piotr Gadawski|Morphology|BOLD:ACP4407
Pseudosmittia trilobata|NHRS-BYWS000001057|Yngve Brodin|morphology|BOLD:ACP4407
Pseudosmittia trilobata|NHRS-BYWS000000988|Yngve Brodin|morphology|BOLD:ACP4407
Pseudosmittia trilobata|NHRS-BYWS000000585|Yngve Brodin|morphology|BOLD:ACP4407
Pseudosmittia trilobata|NHRS-BYWS000000584|Yngve Brodin|morphology|BOLD:ACP4407
Limnophyes|BIOUG43080-B03|Kate Perez|BIN Taxonomy Match below Phylum (Jun 2019)|BOLD:ABV...
Limnophyes sp. 1SW|ZSM34341-F05|Sofia Wiedenbrug|Morphology|BOLD:AAB1807
Limnophyes sp. 1SW|ZSM34342-D03|Sofia Wiedenbrug|Morphology|BOLD:AAB1807
Limnophyes sp. 1SW|ZSM-DIP-33130-G01|Caroline Chimeno|BIN Taxonomy Match (Jan 2022)|BOLD ...
Limnophyes sp. 1SW|CCDB24039-C05|Sofia Wiedenbrug|BIN Taxonomy Match|BOLD:AAB1807
Limnophyes sp. 1SW|CCDB24038-A07|Sofia Wiedenbrug|BIN Taxonomy Match|BOLD:AAB1807
Limnophyes sp. 1SW|CCDB24229-B12|Sofia Wiedenbrug|BIN Taxonomy Match|BOLD:AAB1807
[illegible]

Limnophyes sp. 1SW|CCDB24039-C05|Sofia Wiedenbrug|BIN Taxonomy Match|BOLD:AAB1807
Limnophyes sp. 1SW|CCDB24038-A07|Sofia Wiedenbrug|BIN Taxonomy Match|BOLD:AAB1807
Limnophyes sp. 1SW|CCDB24229-B12|Sofia Wiedenbrug|BIN Taxonomy Match|BOLD:AAB1807
Limnophyes sp. 1SW|CCDB24229-F11|Sofia Wiedenbrug|BIN Taxonomy Match|BOLD:AAB1807
Limnophyes sp. 1SW|ZSM34342-B05|Sofia Wiedenbrug|Morphology|BOLD:AAB1807
Limnophyes sp. 1SW|ZSM34342-B04|Sofia Wiedenbrug|Morphology|BOLD:AAB1807
Limnophyes sp. 1SW|BC-ZSM-DIP-22488-G05|Dieter Doczkal|BIN Taxonomy Match|BOLD:AAB1807
Limnophyes sp. 1SW|CCDB24039-H08|Sofia Wiedenbrug|BIN Taxonomy Match|BOLD:AAB1807
Limnophyes sp. 1SW|CCDB24037-C01|Sofia Wiedenbrug|BIN Taxonomy Match|BOLD:AAB1807
Limnophyes sp. 1SW|CCDB24039-C04|Sofia Wiedenbrug|BIN Taxonomy Match|BOLD:AAB1807
Limnophyes sp. 1SW|ZSM34342-B02|Sofia Wiedenbrug|Morphology|BOLD:AAB1807
Limnophyes sp. 1SW|CCDB24228-G07|Sofia Wiedenbrug|BIN Taxonomy Match|BOLD:AAB1807
Limnophyes sp. 1SW|ZSM34342-H04|Sofia Wiedenbrug|Morphology
Limnophyes sp. 1SW|CCDB21606-C03|Sofia Wiedenbrug||BOLD:AAB1807
Limnophyes sp. 1SW|CCDB21606-D07|Sofia Wiedenbrug||BOLD:AAB1807
Limnophyes sp. 1SW|ZSM34342-H05|Sofia Wiedenbrug|Morphology|BOLD:AAB1807
Limnophyes sp. 1SW|CCDB24229-F02|Sofia Wiedenbrug|BIN Taxonomy Match|BOLD:AAB1807
Limnophyes sp. 1SW|CCDB21606-D08|Sofia Wiedenbrug||BOLD:AAB1807
Limnophyes sp. 1SW|CCDB21606-D05|Sofia Wiedenbrug||BOLD:AAB1807
Chironomidae|CCDB22603-D01|Sofia Wiedenbrug|no morphological identification - introduction of int...
Chironomidae|CCDB22603-D05|Sofia Wiedenbrug|no morphological identification - introduction of int...
Chironomidae|CCDB22603-C08|Sofia Wiedenbrug|no morphological identification - introduction of int...
Chironomidae|CCDB22603-A09|Sofia Wiedenbrug|no morphological identification - introduction of int...
Chironomidae|CCDB22603-F10|Sofia Wiedenbrug|no morphological identification - introduction of int...
Chironomidae|CCDB22603-F06|Sofia Wiedenbrug|no morphological identification - introduction of int...
Chironomidae|CCDB22603-D06|Sofia Wiedenbrug|no morphological identification - introduction of int...
Chironomidae|CCDB22603-E05|Sofia Wiedenbrug|no morphological identification - introduction of int...
Chironomidae|CCDB22603-D04|Sofia Wiedenbrug|no morphological identification - introduction of int...
Chironomidae|CCDB22603-C12|Sofia Wiedenbrug|no morphological identification - introduction of int...
Chironomidae|CCDB22603-C07|Sofia Wiedenbrug|no morphological identification - introduction of int...
Chironomidae|CCDB22603-E04|Sofia Wiedenbrug|no morphological identification - introduction of int...
Chironomidae|CCDB22603-A02|Sofia Wiedenbrug|no morphological identification - introduction of int...
Limnophyes sp. 1SW|CCDB24039-B12|Sofia Wiedenbrug|BIN Taxonomy Match
Limnophyes sp. 1SW|CCDB24037-G07|Sofia Wiedenbrug|BIN Taxonomy Match|BOLD:AAB1807
Limnophyes sp. 1SW|CCDB24228-B11|Sofia Wiedenbrug|BIN Taxonomy Match|BOLD:AAB1807
Limnophyes sp. 1SW|ZSM34342-C08|Sofia Wiedenbrug|Morphology|BOLD:AAB1807
Limnophyes sp. 1SW|CCDB24038-F09|Sofia Wiedenbrug|BIN Taxonomy Match|BOLD:AAB1807
Limnophyes sp. 1SW|BC-ZSM-DIP-22488-D10|Dieter Doczkal|BIN Taxonomy Match|BOLD:AAB1807
Limnophyes sp. 1SW|BC-ZSM-DIP-22488-G12|Dieter Doczkal|BIN Taxonomy Match|BOLD:AAB1807
Limnophyes sp. 1SW|CCDB24038-F10|Sofia Wiedenbrug|BIN Taxonomy Match|BOLD:AAB1807
Limnophyes sp. 1SW|CCDB24229-E11|Sofia Wiedenbrug|BIN Taxonomy Match|BOLD:AAB1807
Limnophyes sp. 1SW|CCDB24228-B12|Sofia Wiedenbrug|BIN Taxonomy Match|BOLD:AAB1807
Limnophyes sp. 1SW|BC-ZSM-DIP-22488-D03|Dieter Doczkal|BIN Taxonomy Match|BOLD:AAB1807
Limnophyes sp. 1SW|BC-ZSM-DIP-22488-H03|Dieter Doczkal|BIN Taxonomy Match|BOLD:AAB1807
Limnophyes sp. 1SW|ZSM34342-H08|Sofia Wiedenbrug|Morphology|BOLD:AAB1807
Limnophyes sp. 1SW|CCDB24037-G08|Sofia Wiedenbrug|BIN Taxonomy Match|BOLD:AAB1807
Limnophyes sp. 1SW|CCDB24039-C03|Sofia Wiedenbrug|BIN Taxonomy Match|BOLD:AAB1807
Limnophyes sp. 1SW|BC-ZSM-DIP-22488-G08|Dieter Doczkal|BIN Taxonomy Match|BOLD:AAB1807
Limnophyes sp. 1SW|CCDB24228-B03|Sofia Wiedenbrug|BIN Taxonomy Match|BOLD:AAB1807
Limnophyes sp. 1SW|ZSM34342-F09|Sofia Wiedenbrug|Morphology|BOLD:AAB1807
Limnophyes sp. 1SW|ZSM34342-F10|Sofia Wiedenbrug|Morphology|BOLD:AAB1807
Limnophyes sp. 1SW|CCDB24228-B04|Sofia Wiedenbrug|BIN Taxonomy Match|BOLD:AAB1807
Limnophyes sp. 1SW|BC-ZSM-DIP-22488-H06|Dieter Doczkal|BIN Taxonomy Match|BOLD:AAB1807
Limnophyes sp. 1SW|CCDB24229-D02|Sofia Wiedenbrug|BIN Taxonomy Match|BOLD:AAB1807
Limnophyes sp. 1SW|CCDB24038-F07|Sofia Wiedenbrug|BIN Taxonomy Match|BOLD:AAB1807
Limnophyes sp. 1SW|ZSM34342-E04|Sofia Wiedenbrug|Morphology|BOLD:AAB1807
Limnophyes sp. 1SW|ZSM34342-E08|Sofia Wiedenbrug|Morphology|BOLD:AAB1807
Limnophyes sp. 1SW|ZSM34342-H06|Sofia Wiedenbrug|Morphology|BOLD:AAB1807
Limnophyes sp. 1SW|ZSM34342-F05|Sofia Wiedenbrug|Morphology|BOLD:AAB1807
Limnophyes|25128-F04|Kate Perez|BIN Taxonomy Match (Mar 2018)|BOLD:AAB1807
Limnophyes|25129-E01|Kate Perez|BIN Taxonomy Match (Mar 2018)|BOLD:AAB1807
Limnophyes|25128-B12|Kate Perez|BIN Taxonomy Match (Mar 2018)|BOLD:AAB1807
Limnophyes|25129-G06|Kate Perez|BIN Taxonomy Match (Mar 2018)|BOLD:AAB1807
Limnophyes sp. 1ES|SOE320|Elisabeth Stur||BOLD:AAB1807
Limnophyes sp. 1ES|SOE59|Elisabeth Stur||BOLD:AAB1807
Limnophyes edwardsi|CCDB24183-G07|Sofia Wiedenbrug|Morphology|BOLD:AAB9700
Limnophyes edwardsi|CCDB24183-D01|Sofia Wiedenbrug|Morphology|BOLD:AAB9700
Limnophyes edwardsi|ZSM-DIP-33135-C12|Caroline Chimeno|BIN Taxonomy Match (Jan 2022)|BOLD ...
Limnophyes edwardsi|ZSM-DIP-33135-D01|Caroline Chimeno|BIN Taxonomy Match (Jan 2022)|BOLD ...
Limnophyes edwardsi|ZSM-DIP-33148-C01|Caroline Chimeno|BIN Taxonomy Match (Jan 2022)|BOLD ...
Limnophyes edwardsi|ZSM-DIP-33148-C03|Caroline Chimeno|BIN Taxonomy Match (Jan 2022)|BOLD ...
Limnophyes edwardsi|BIOUG05143-H09|Kate Perez|BIN Taxonomy Match|BOLD:AAB9700
Limnophyes edwardsi|BIOUG05110-G09|Kate Perez|BIN Taxonomy Match|BOLD:AAB9700
Limnophyes edwardsi|SOE193|Elisabeth Stur||BOLD:AAB9700
Limnophyes edwardsi|24354-D09|Kate Perez|BIN Taxonomy Match|BOLD:AAB9700
Limnophyes edwardsi|ZSM34342-G09|Sofia Wiedenbrug|Morphology|BOLD:AAB9700
Limnophyes edwardsi|CCDB24039-B10|Sofia Wiedenbrug|BIN Taxonomy Match|BOLD:AAB9700
Limnophyes edwardsi|SOE152|Elisabeth Stur||BOLD:AAB9700
Limnophyes edwardsi|SOE375|Elisabeth Stur||BOLD:AAB9700
Limnophyes edwardsi|SOE194|Elisabeth Stur||BOLD:AAB9700
Limnophyes edwardsi|SOE461|Elisabeth Stur||BOLD:AAB9700
Limnophyes edwardsi|SOE330|Elisabeth Stur||BOLD:AAB9700
Limnophyes edwardsi|SOE354|Elisabeth Stur||BOLD:AAB9700
Limnophyes edwardsi|SOE367|Elisabeth Stur||BOLD:AAB9700
Limnophyes edwardsi|ATNA395|Elisabeth Stur||BOLD:AAB9700
Limnophyes edwardsi|TRD-CH54|Elisabeth Stur||BOLD:AAB9700
Limnophyes edwardsi|ATNA211|Elisabeth Stur||BOLD:AAB9700
Limnophyes edwardsi|ATNA214|Elisabeth Stur||BOLD:AAB9700
Limnophyes edwardsi|ATNA215|Elisabeth Stur||BOLD:AAB9700
Limnophyes edwardsi|SOE313|Elisabeth Stur||BOLD:AAB9700
Limnophyes edwardsi|BIOUG15765-G03|Kate Perez|BIN Taxonomy Match|BOLD:AAB9700
Limnophyes edwardsi|BIOUG04085-D02|Kate Perez|BIN Taxonomy Match|BOLD:AAB9700
Limnophyes edwardsi|SOE472|Elisabeth Stur||BOLD:AAB9700
Limnophyes edwardsi|24371-E04|Kate Perez|BIN Taxonomy Match|BOLD:AAB9700
Limnophyes edwardsi|ZMUO.025973|Lauri Paasivirta||BOLD:AAB9700
Limnophyes edwardsi|SOE436|Elisabeth Stur||BOLD:AAB9700
Limnophyes edwardsi|Finnmark322|Elisabeth Stur||BOLD:AAB9700
Limnophyes edwardsi|Finnmark300|Elisabeth Stur||BOLD:AAB9700
Limnophyes edwardsi|Finnmark273|Elisabeth Stur||BOLD:AAB9700
Limnophyes|ZSM-DIP-33133-B10|Caroline Chimeno|BIN Taxonomy Match (Jan 2022)|BOLD:ABW5528
Limnophyes|ZSM-DIP-33146-E02|Caroline Chimeno|BIN Taxonomy Match (Jan 2022)|BOLD:ABW5528
Limnophyes|BIOUG16707-C07|Kate Perez|BIN Taxonomy Match|BOLD:ABW5528
Limnophyes|ZSM-DIP-33135-B09|Caroline Chimeno|BIN Taxonomy Match (Jan 2022)|BOLD:ABW5528

Limnophyes|ZSM-DIP-33146-E02|Caroline Chimeno|BIN Taxonomy Match (Jan 2022)|BOLD:ABW5528
Limnophyes|BIOUG16707-C07|Kate Perez|BIN Taxonomy Match|BOLD:ABW5528
Limnophyes|ZSM-DIP-33135-B09|Caroline Chimeno|BIN Taxonomy Match (Jan 2022)|BOLD:ABW5528
Limnophyes|ZSM-DIP-33144-D02|Caroline Chimeno|BIN Taxonomy Match (Jan 2022)|BOLD:ABW5528
Limnophyes|ZSM-DIP-33140-B06|Caroline Chimeno|BIN Taxonomy Match (Jan 2022)|BOLD:ABW5528
Limnophyes|BIOUG42730-E01|Kate Perez|BIN Taxonomy Match below Phylum (Jun 2019)|BOLD:ABW...
Limnophyes|ZSM-DIP-33137-H07|Caroline Chimeno|BIN Taxonomy Match (Jan 2022)|BOLD:ABW5528
Limnophyes|ZSM-DIP-33133-G11|Caroline Chimeno|BIN Taxonomy Match (Jan 2022)|BOLD:ABW5528
Limnophyes|ZSM-DIP-33140-F05|Caroline Chimeno|BIN Taxonomy Match (Jan 2022)|BOLD:ABW5528
Limnophyes|ZSM-DIP-33135-B05|Caroline Chimeno|BIN Taxonomy Match (Jan 2022)|BOLD:ABW5528
Limnophyes|ZSM-DIP-33135-B07|Caroline Chimeno|BIN Taxonomy Match (Jan 2022)|BOLD:ABW5528
Limnophyes|BIOUG55147-G01|Stefan Schmidt|BIN Taxonomy Match (Sep 2020)|BOLD:ABW5528
Limnophyes|ZSM-DIP-33133-B12|Caroline Chimeno|BIN Taxonomy Match (Jan 2022)|BOLD:ABW5528
Limnophyes|ZSM-DIP-33133-C01|Caroline Chimeno|BIN Taxonomy Match (Jan 2022)|BOLD:ABW5528
Limnophyes|ZSM-DIP-33133-C02|Caroline Chimeno|BIN Taxonomy Match (Jan 2022)|BOLD:ABW5528
Limnophyes|ZSM-DIP-33133-G12|Caroline Chimeno|BIN Taxonomy Match (Jan 2022)|BOLD:ABW5528
Limnophyes|ZSM-DIP-33142-H03|Caroline Chimeno|BIN Taxonomy Match (Jan 2022)|BOLD:ABW5528
Limnophyes|ZSM-DIP-33144-D03|Caroline Chimeno|BIN Taxonomy Match (Jan 2022)|BOLD:ABW5528
Limnophyes|ZSM-DIP-33135-B10|Caroline Chimeno|BIN Taxonomy Match (Jan 2022)|BOLD:ABW5528
Limnophyes|ZSM-DIP-33135-B12|Caroline Chimeno|BIN Taxonomy Match (Jan 2022)|BOLD:ABW5528
Limnophyes|ZSM-DIP-33136-H02|Caroline Chimeno|BIN Taxonomy Match (Jan 2022)|BOLD:ABW5528
Limnophyes|ZSM-DIP-33136-H09|Caroline Chimeno|BIN Taxonomy Match (Jan 2022)|BOLD:ABW5528
Limnophyes|ZSM-DIP-33137-H09|Caroline Chimeno|BIN Taxonomy Match (Jan 2022)|BOLD:ABW5528
Limnophyes|ZSM-DIP-33137-H10|Caroline Chimeno|BIN Taxonomy Match (Jan 2022)|BOLD:ABW5528
Limnophyes|ZSM-DIP-33138-E01|Caroline Chimeno|BIN Taxonomy Match (Jan 2022)|BOLD:ABW5528
Limnophyes|ZSM-DIP-33140-E03|Caroline Chimeno|BIN Taxonomy Match (Jan 2022)|BOLD:ABW5528
Limnophyes|ZSM-DIP-33140-E08|Caroline Chimeno|BIN Taxonomy Match (Jan 2022)|BOLD:ABW5528
Limnophyes|ZSM-DIP-33140-E09|Caroline Chimeno|BIN Taxonomy Match (Jan 2022)|BOLD:ABW5528
Limnophyes|ZSM-DIP-33140-E10|Caroline Chimeno|BIN Taxonomy Match (Jan 2022)|BOLD:ABW5528
Limnophyes|ZSM-DIP-33140-E11|Caroline Chimeno|BIN Taxonomy Match (Jan 2022)|BOLD:ABW5528
Limnophyes|ZSM-DIP-33140-E12|Caroline Chimeno|BIN Taxonomy Match (Jan 2022)|BOLD:ABW5528
Limnophyes|ZSM-DIP-33144-F02|Caroline Chimeno|BIN Taxonomy Match (Jan 2022)|BOLD:ABW5528
Limnophyes|ZSM-DIP-33128-B11|Caroline Chimeno|BIN Taxonomy Match (Jan 2022)|BOLD:ABW5528
Limnophyes|ZSM-DIP-33128-D01|Caroline Chimeno|BIN Taxonomy Match (Jan 2022)|BOLD:ABW5528
Limnophyes|ZSM-DIP-33129-B09|Caroline Chimeno|BIN Taxonomy Match (Jan 2022)|BOLD:ABW5528
Limnophyes|ZSM-DIP-33145-A05|Caroline Chimeno|BIN Taxonomy Match (Jan 2022)|BOLD:ABW5528
Limnophyes|ZSM-DIP-33145-A06|Caroline Chimeno|BIN Taxonomy Match (Jan 2022)|BOLD:ABW5528
Limnophyes|ZSM-DIP-33145-A07|Caroline Chimeno|BIN Taxonomy Match (Jan 2022)|BOLD:ABW5528
Limnophyes|ZSM-DIP-33140-F02|Caroline Chimeno|BIN Taxonomy Match (Jan 2022)|BOLD:ABW5528
Limnophyes|ZSM-DIP-33137-H11|Caroline Chimeno|BIN Taxonomy Match (Jan 2022)|BOLD:ABW5528
Limnophyes|BIOUG16277-E09|Kate Perez|BIN Taxonomy Match|BOLD:ABW5528
Limnophyes|ZSM-DIP-33133-A12|Caroline Chimeno|BIN Taxonomy Match (Jan 2022)|BOLD:ABW5528
Limnophyes|BIOUG17225-H11|Kate Perez|BIN Taxonomy Match|BOLD:ABW5528
Limnophyes|BIOUG16268-G05|Kate Perez|BIN Taxonomy Match|BOLD:ABW5528
Limnophyes|BIOUG16277-F07|Kate Perez|BIN Taxonomy Match|BOLD:ABW5528
Limnophyes|BIOUG16269-G01|Kate Perez|BIN Taxonomy Match|BOLD:ABW5528
Limnophyes|BIOUG16277-H06|Kate Perez|BIN Taxonomy Match|BOLD:ABW5528
Limnophyes|BIOUG16351-E09|Kate Perez|BIN Taxonomy Match|BOLD:ABW5528
Limnophyes|BIOUG16268-G02|Kate Perez|BIN Taxonomy Match|BOLD:ABW5528
Limnophyes|BIOUG16354-F02|Kate Perez|BIN Taxonomy Match|BOLD:ABW5528
Limnophyes|BIOUG16783-D09|Kate Perez|BIN Taxonomy Match|BOLD:ABW5528
Limnophyes|BIOUG16736-B12|Kate Perez|BIN Taxonomy Match|BOLD:ABW5528
Limnophyes|BIOUG16214-E10|Kate Perez|BIN Taxonomy Match|BOLD:ABW5528
Limnophyes|BIOUG16529-E07|Kate Perez|BIN Taxonomy Match|BOLD:ABW5528
Limnophyes|CCDB24229-A09|Sofia Wiedenbrug|BIN Taxonomy Match|BOLD:ACU4663
Limnophyes|ZSM34341-D11|Sofia Wiedenbrug|Morphology|BOLD:ACU4663
Limnophyes|CCDB24229-B02|Sofia Wiedenbrug|BIN Taxonomy Match|BOLD:ACU4663
Limnophyes|CCDB24228-C12|Sofia Wiedenbrug|BIN Taxonomy Match|BOLD:ACU4663
Limnophyes sp. 6SW|CCDB22605-A05|Sofia Wiedenbrug||BOLD:ACT8783
Limnophyes sp. 6SW|CCDB24229-D08|Sofia Wiedenbrug|BIN Taxonomy Match|BOLD:ACT8783
Smittia|ZSM34343-C06|Sofia Wiedenbrug|Morphology
Limnophyes sp. 13SW|ZSM34343-D06|Sofia Wiedenbrug|Morphology|BOLD:ADC4985
Limnophyes sp.|ZSM34343-C05|Sofia Wiedenbrug|Morphology|BOLD:ADC4985
Limnophyes sp. 11SW|BC-ZSM-DIP-22488-D07|Dieter Doczkal|BIN Taxonomy Match|BOLD:ACR9203
Limnophyes sp. 11SW|CCDB24229-F09|Jerome Moriniere|BIN Taxonomy Match|BOLD:ACR9203
Limnophyes sp. 11SW|ZSM34343-H10|Jerome Moriniere|BIN Taxonomy Match|BOLD:ACR9203
Limnophyes sp. 11SW|ZSM34342-E07|Sofia Wiedenbrug|Morphology
Limnophyes sp. 11SW|CCDB22604-G09|Jerome Moriniere|BIN Taxonomy Match|BOLD:ACR9203
Limnophyes sp. 11SW|CCDB24229-D12|Jerome Moriniere|BIN Taxonomy Match|BOLD:ACR9203
Limnophyes sp. 11SW|CCDB24038-F03|Jerome Moriniere|BIN Taxonomy Match|BOLD:ACR9203
Limnophyes sp. 11SW|BC-ZSM-DIP-22488-D12|Dieter Doczkal|BIN Taxonomy Match|BOLD:ACR9203
Limnophyes sp. 11SW|BC-ZSM-DIP-22551-D02|Dieter Doczkal|BIN Taxonomy Match|BOLD:ACR9203
Limnophyes sp. 11SW|CCDB24228-D12|Jerome Moriniere|BIN Taxonomy Match|BOLD:ACR9203
Limnophyes sp. 11SW|ZSM34342-F02|Sofia Wiedenbrug|Morphology
Limnophyes sp. 11SW|CCDB24228-A02|Jerome Moriniere|BIN Taxonomy Match|BOLD:ACR9203
Limnophyes sp. 11SW|CCDB24038-E08|Jerome Moriniere|BIN Taxonomy Match|BOLD:ACR9203
Limnophyes sp. 11SW|CCDB24037-B06|Jerome Moriniere|BIN Taxonomy Match|BOLD:ACR9203
Limnophyes sp. 12SW|ZSM34341-F02|Sofia Wiedenbrug|Morphology|BOLD:ACU4070
Limnophyes sp. 12SW|BC-ZSM-DIP-22488-D09|Dieter Doczkal|BIN Taxonomy Match|BOLD:ACU4070
Limnophyes sp. 12SW|ZSM34342-F08|Sofia Wiedenbrug|Morphology|BOLD:ACU4070
Limnophyes sp. 12SW|CCDB24038-C07|Sofia Wiedenbrug|BIN Taxonomy Match|BOLD:ACU4070
Limnophyes sp. 12SW|ZSM34342-F04|Sofia Wiedenbrug|Morphology|BOLD:ACU4070
Limnophyes sp. 12SW|BC-ZSM-DIP-22491-H09|Dieter Doczkal|BIN Taxonomy Match|BOLD:ACU4070
Limnophyes sp. 12SW|BC-ZSM-DIP-22488-H07|Dieter Doczkal|BIN Taxonomy Match|BOLD:ACU4070
Limnophyes sp. 12SW|BC-ZSM-DIP-22488-G04|Dieter Doczkal|BIN Taxonomy Match|BOLD:ACU4070
Limnophyes sp. 12SW|CCDB24037-B11|Sofia Wiedenbrug|BIN Taxonomy Match|BOLD:ACU4070
Limnophyes sp. 4SW|CCDB24037-A11|Jerome Moriniere|BIN Taxonomy Match|BOLD:ACU4225
Limnophyes sp. 4SW|BC-ZSM-DIP-22551-C09|Dieter Doczkal|BIN Taxonomy Match|BOLD:ACU4225
Limnophyes sp. 4SW|ZSM34343-B04|Sofia Wiedenbrug|Morphology|BOLD:ACU4225
Limnophyes sp. 4SW|ZSM34343-B07|Sofia Wiedenbrug|Morphology|BOLD:ACU4225
Limnophyes sp. 4SW|ZSM34343-E06|Sofia Wiedenbrug|Morphology|BOLD:ACU4225
Limnophyes sp. 4SW|CCDB24228-G03|Jerome Moriniere|BIN Taxonomy Match|BOLD:ACU4225
Limnophyes sp. 4SW|CCDB24039-E01|Jerome Moriniere|BIN Taxonomy Match|BOLD:ACU4225
Limnophyes sp. 4SW|BC-ZSM-DIP-22491-D05|Dieter Doczkal|BIN Taxonomy Match|BOLD:ACU4225
Limnophyes sp. 4SW|CCDB24228-B10|Sofia Wiedenbrug|BIN Taxonomy Match|BOLD:ACR9428
Limnophyes sp. 4SW|CCDB21606-H01|Sofia Wiedenbrug||BOLD:ACR9428
Limnophyes sp. 4SW|CCDB21606-A08|Sofia Wiedenbrug||BOLD:ACR9428
Limnophyes sp. 4SW|ZSM34341-F09|Sofia Wiedenbrug|Morphology|BOLD:ACR9428
Limnophyes sp. 4SW|CCDB24039-D12|Sofia Wiedenbrug|BIN Taxonomy Match|BOLD:ACR9428
Limnophyes sp. 4SW|ZSM34343-F10|Sofia Wiedenbrug|Morphology|BOLD:ACR9428
Limnophyes sp. 4SW|ZSM34343-G04|Sofia Wiedenbrug|Morphology|BOLD:ACR9428
Limnophyes sp. 4SW|CCDB24228-D11|Sofia Wiedenbrug|BIN Taxonomy Match|BOLD:ACR9428
Limnophyes sp. 4SW|CCDB24228-B09|Sofia Wiedenbrug|BIN Taxonomy Match|BOLD:ACR9428

Limnophyes sp. 4SW|ZSM34343-G04|Sofia Wiedenbrug|Morphology|BOLD:ACR9428
Limnophyes sp. 4SW|CCDB24228-D11|Sofia Wiedenbrug|BIN Taxonomy Match|BOLD:ACR9428
Limnophyes sp. 4SW|CCDB24228-B09|Sofia Wiedenbrug|BIN Taxonomy Match|BOLD:ACR9428
Limnophyes sp. 4SW|CCDB22604-G06|Sofia Wiedenbrug||BOLD:ACR9428
Limnophyes sp. 4SW|CCDB22605-A07|Sofia Wiedenbrug||BOLD:ACR9428
Limnophyes sp. 4SW|ZSM34342-G03|Sofia Wiedenbrug|Morphology|BOLD:ACR9428
Limnophyes sp. 4SW|BC-ZSM-DIP-22488-E10|Dieter Doczkal||BOLD:ACR9428
Limnophyes sp. 4SW|ZSM34343-F09|Sofia Wiedenbrug|Morphology|BOLD:ACR9428
Limnophyes sp. 4SW|CCDB24228-D08|Sofia Wiedenbrug|BIN Taxonomy Match|BOLD:ACR9428
Limnophyes sp. 4SW|BC-ZSM-DIP-22488-H02|Dieter Doczkal||BOLD:ACR9428
Limnophyes sp. 4SW|CCDB24038-G11|Sofia Wiedenbrug|BIN Taxonomy Match|BOLD:ACR9428
Limnophyes sp. 4SW|CCDB24039-C08|Sofia Wiedenbrug|BIN Taxonomy Match|BOLD:ACR9428
Limnophyes sp. 4SW|CCDB22604-G07|Sofia Wiedenbrug||BOLD:ACR9428
Limnophyes sp. 4SW|CCDB24229-F10|Sofia Wiedenbrug|BIN Taxonomy Match|BOLD:ACR9428
Limnophyes sp. 4SW|ZSM34341-H02|Sofia Wiedenbrug|Morphology|BOLD:ACR9428
Limnophyes sp. 4SW|CCDB22605-A08|Sofia Wiedenbrug||BOLD:ACR9428
Limnophyes sp. 4SW|CCDB22605-A06|Sofia Wiedenbrug||BOLD:ACR9428
Limnophyes sp. 4SW|ZSM34343-A09|Sofia Wiedenbrug|Morphology|BOLD:ACR9428
Limnophyes sp. 4SW|ZSM34343-F05|Sofia Wiedenbrug|Morphology|BOLD:ACR9428
Limnophyes sp. 4SW|BC-ZSM-DIP-22488-H01|Dieter Doczkal||BOLD:ACR9428
Limnophyes sp. 4SW|CCDB24228-A01|Sofia Wiedenbrug|BIN Taxonomy Match|BOLD:ACR9428
Limnophyes sp. 4SW|BC-ZSM-DIP-22488-H08|Dieter Doczkal||BOLD:ACR9428
Limnophyes cf. bidumus|CCDB22604-G08|Sofia Wiedenbrug||BOLD:ACR9099
Limnophyes cf. bidumus|CCDB24038-C06|Sofia Wiedenbrug|BIN Taxonomy Match|BOLD:ACR9099
Limnophyes cf. bidumus|BC-ZSM-DIP-22491-D07|Dieter Doczkal||BOLD:ACR9099
Limnophyes cf. bidumus|CCDB24229-F03|Sofia Wiedenbrug|BIN Taxonomy Match|BOLD:ACR9099
Limnophyes cf. bidumus|CCDB24038-A04|Sofia Wiedenbrug|BIN Taxonomy Match|BOLD:ACR9099
Limnophyes cf. bidumus|BC-ZSM-DIP-22488-F12|Dieter Doczkal||BOLD:ACR9099
Limnophyes cf. bidumus|BC-ZSM-DIP-22491-H02|Dieter Doczkal||BOLD:ACR9099
Limnophyes cf. bidumus|CCDB24037-B10|Sofia Wiedenbrug|BIN Taxonomy Match|BOLD:ACR9099
Limnophyes cf. bidumus|CCDB24038-F12|Sofia Wiedenbrug|BIN Taxonomy Match|BOLD:ACR9099
Limnophyes cf. bidumus|CCDB24039-C09|Sofia Wiedenbrug|BIN Taxonomy Match|BOLD:ACR9099
Limnophyes cf. bidumus|CCDB24037-A04|Sofia Wiedenbrug|BIN Taxonomy Match|BOLD:ACR9099
Limnophyes cf. bidumus|BC-ZSM-DIP-22491-E01|Sofia Wiedenbrug|Morphology|BOLD:ACR9099
Limnophyes bidumus|ZSM34343-D02|Sofia Wiedenbrug|Morphology
Limnophyes bidumus|ZSM34343-D05|Sofia Wiedenbrug|Morphology|BOLD:ACU4849
Limnophyes bidumus|ZSM34343-C12|Sofia Wiedenbrug|Morphology|BOLD:ACU4849
Limnophyes bidumus|CCDB24038-A10|Sofia Wiedenbrug|BIN Taxonomy Match|BOLD:ACU4849
Limnophyes bidumus|CCDB24038-A08|Sofia Wiedenbrug|BIN Taxonomy Match|BOLD:ACU4849
Chironomidae|CCDB24228-B06|Sofia Wiedenbrug|no morphological identification - introduction of int...
Chironomidae|CCDB24037-C03|Sofia Wiedenbrug|no morphological identification - introduction of int...
Chironomidae|CCDB22603-H05|Sofia Wiedenbrug|no morphological identification - introduction of int...
Chironomidae|CCDB22603-G06|Sofia Wiedenbrug|no morphological identification - introduction of int...
Limnophyes|CCDB22603-F04|Sofia Wiedenbrug||BOLD:ACQ8320
Limnophyes bidumus|25130-H06|Kate Perez|BOLD ID Engine Manual (Apr 2018)|BOLD:AAJ2138
Limnophyes bidumus|24372-D07|Kate Perez|BIN Taxonomy Match|BOLD:AAJ2138
Limnophyes bidumus|25129-B08|Kate Perez|BIN Taxonomy Match (Mar 2018)|BOLD:AAJ2138
Limnophyes bidumus|25131-D10|Kate Perez|BOLD ID Engine Manual (Apr 2018)|BOLD:AAJ2138
Limnophyes bidumus|25130-F05|Kate Perez|BOLD ID Engine Manual (Apr 2018)|BOLD:AAJ2138
Limnophyes bidumus|24354-C11|Kate Perez|BIN Taxonomy Match|BOLD:AAJ2138
Limnophyes bidumus|24354-C10|Kate Perez|BIN Taxonomy Match|BOLD:AAJ2138
Limnophyes bidumus|25130-G01|Kate Perez|BOLD ID Engine Manual (Apr 2018)|BOLD:AAJ2138
Limnophyes bidumus|25132-F08|Kate Perez|BOLD ID Engine Manual (Apr 2018)|BOLD:AAJ2138
Limnophyes bidumus|25130-C10|Kate Perez|BOLD ID Engine Manual (Apr 2018)|BOLD:AAJ2138
Limnophyes bidumus|25128-B11|Kate Perez|BIN Taxonomy Match (Mar 2018)|BOLD:AAJ2138
Limnophyes bidumus|25132-A04|Kate Perez|BOLD ID Engine Manual (Apr 2018)|BOLD:AAJ2138
Limnophyes bidumus|25129-A12|Kate Perez|BIN Taxonomy Match (Mar 2018)|BOLD:AAJ2138
Limnophyes bidumus|23975-B04|Kate Perez|BIN Taxonomy Match|BOLD:AAJ2138
Limnophyes bidumus|SOE462|Elisabeth Stur||BOLD:AAJ2138
Limnophyes bidumus|25130-F12|Kate Perez|BOLD ID Engine Manual (Apr 2018)|BOLD:AAJ2138
Limnophyes bidumus|25130-F01|Kate Perez|BOLD ID Engine Manual (Apr 2018)|BOLD:AAJ2138
Limnophyes bidumus|25131-D08|Kate Perez|BOLD ID Engine Manual (Apr 2018)|BOLD:AAJ2138
Limnophyes bidumus|25132-A05|Kate Perez|BOLD ID Engine Manual (Apr 2018)|BOLD:AAJ2138
Limnophyes bidumus|25132-A07|Kate Perez|BOLD ID Engine Manual (Apr 2018)|BOLD:AAJ2138
Limnophyes bidumus|25132-C01|Kate Perez|BOLD ID Engine Manual (Apr 2018)|BOLD:AAJ2138
Limnophyes bidumus|25132-D09|Kate Perez|BOLD ID Engine Manual (Apr 2018)|BOLD:AAJ2138
Limnophyes bidumus|25132-D10|Kate Perez|BOLD ID Engine Manual (Apr 2018)|BOLD:AAJ2138
Limnophyes bidumus|25132-G11|Kate Perez|BOLD ID Engine Manual (Apr 2018)|BOLD:AAJ2138
Limnophyes bidumus|25132-H02|Kate Perez|BOLD ID Engine Manual (Apr 2018)|BOLD:AAJ2138
Limnophyes bidumus|25130-B03|Kate Perez|BOLD ID Engine Manual (Apr 2018)|BOLD:AAJ2138
Limnophyes bidumus|25130-D08|Kate Perez|BOLD ID Engine Manual (Apr 2018)|BOLD:AAJ2138
Limnophyes bidumus|25130-D09|Kate Perez|BOLD ID Engine Manual (Apr 2018)|BOLD:AAJ2138
Limnophyes bidumus|25130-D11|Kate Perez|BOLD ID Engine Manual (Apr 2018)|BOLD:AAJ2138
Limnophyes bidumus|25130-E04|Kate Perez|BOLD ID Engine Manual (Apr 2018)|BOLD:AAJ2138
Limnophyes bidumus|25130-E06|Kate Perez|BOLD ID Engine Manual (Apr 2018)|BOLD:AAJ2138
Limnophyes bidumus|25130-E11|Kate Perez|BOLD ID Engine Manual (Apr 2018)|BOLD:AAJ2138
Limnophyes bidumus|25130-H02|Kate Perez|BOLD ID Engine Manual (Apr 2018)|BOLD:AAJ2138
Limnophyes bidumus|25131-B01|Kate Perez|BOLD ID Engine Manual (Apr 2018)|BOLD:AAJ2138
Limnophyes bidumus|25131-C10|Kate Perez|BOLD ID Engine Manual (Apr 2018)|BOLD:AAJ2138
Limnophyes bidumus|25131-D03|Kate Perez|BOLD ID Engine Manual (Apr 2018)|BOLD:AAJ2138
Limnophyes bidumus|25131-F07|Kate Perez|BOLD ID Engine Manual (Apr 2018)|BOLD:AAJ2138
Limnophyes bidumus|25131-B07|Kate Perez|BOLD ID Engine Manual (Apr 2018)|BOLD:AAJ2138
Limnophyes bidumus|25132-D01|Kate Perez|BOLD ID Engine Manual (Apr 2018)|BOLD:AAJ2138
Limnophyes bidumus|25131-H01|Kate Perez|BOLD ID Engine Manual (Apr 2018)|BOLD:AAJ2138
Limnophyes bidumus|25132-A01|Kate Perez|BOLD ID Engine Manual (Apr 2018)|BOLD:AAJ2138
Limnophyes bidumus|25130-C09|Kate Perez|BOLD ID Engine Manual (Apr 2018)|BOLD:AAJ2138
Limnophyes bidumus|25130-F02|Kate Perez|BOLD ID Engine Manual (Apr 2018)|BOLD:AAJ2138
Limnophyes bidumus|25129-H10|Kate Perez|BIN Taxonomy Match (Mar 2018)|BOLD:AAJ2138
Limnophyes bidumus|24354-B09|Kate Perez|BIN Taxonomy Match|BOLD:AAJ2138
Limnophyes bidumus|25132-E07|Kate Perez|BOLD ID Engine Manual (Apr 2018)|BOLD:AAJ2138
Limnophyes bidumus|25129-A10|Kate Perez|BIN Taxonomy Match (Mar 2018)|BOLD:AAJ2138
Limnophyes bidumus|24373-D08|Kate Perez|BIN Taxonomy Match|BOLD:AAJ2138
Limnophyes bidumus|25128-C04|Kate Perez|BIN Taxonomy Match (Mar 2018)|BOLD:AAJ2138
Limnophyes bidumus|24465-G03|Kate Perez|BIN Taxonomy Match|BOLD:AAJ2138
Limnophyes bidumus|24373-G12|Kate Perez|BIN Taxonomy Match|BOLD:AAJ2138
Limnophyes bidumus|25128-G09|Kate Perez|BIN Taxonomy Match (Mar 2018)|BOLD:AAJ2138
Limnophyes bidumus|25129-H06|Kate Perez|BIN Taxonomy Match (Mar 2018)|BOLD:AAJ2138
Limnophyes bidumus|FS0006|Kate Perez|BIN Taxonomy Match|BOLD:AAJ2138
Limnophyes bidumus|ES376|Elisabeth Stur||BOLD:AAJ2138
Limnophyes bidumus|25132-E05|Kate Perez|BOLD ID Engine Manual (Apr 2018)|BOLD:AAJ2138
Limnophyes bidumus|25132-H10|Kate Perez|BOLD ID Engine Manual (Apr 2018)|BOLD:AAJ2138
Limnophyes bidumus|25131-D05|Kate Perez|BOLD ID Engine Manual (Apr 2018)|BOLD:AAJ2138
Limnophyes bidumus|25131-C11|Kate Perez|BOLD ID Engine Manual (Apr 2018)|BOLD:AAJ2138

[illegible]
Limnophyes bidumus|25132-H10|Kate Perez|BOLD ID Engine Manual (Apr 2018)|BOLD:AAJ2138
Limnophyes bidumus|25131-D05|Kate Perez|BOLD ID Engine Manual (Apr 2018)|BOLD:AAJ2138
Limnophyes bidumus|25131-C11|Kate Perez|BOLD ID Engine Manual (Apr 2018)|BOLD:AAJ2138
Limnophyes bidumus|25130-C04|Kate Perez|BOLD ID Engine Manual (Apr 2018)|BOLD:AAJ2138
Limnophyes bidumus|25132-D05|Kate Perez|BOLD ID Engine Manual (Apr 2018)|BOLD:AAJ2138
Limnophyes bidumus|25216-A02|Kate Perez|BOLD ID Engine Manual (Apr 2018)|BOLD:AAJ2138
Limnophyes bidumus|25130-E12|Kate Perez|BOLD ID Engine Manual (Apr 2018)|BOLD:AAJ2138
Limnophyes bidumus|25131-B03|Kate Perez|BOLD ID Engine Manual (Apr 2018)|BOLD:AAJ2138
Limnophyes bidumus|25131-E02|Kate Perez|BOLD ID Engine Manual (Apr 2018)|BOLD:AAJ2138
Limnophyes bidumus|25131-H09|Kate Perez|BOLD ID Engine Manual (Apr 2018)|BOLD:AAJ2138
Limnophyes bidumus|25132-A10|Kate Perez|BOLD ID Engine Manual (Apr 2018)|BOLD:AAJ2138
Limnophyes bidumus|25132-F06|Kate Perez|BOLD ID Engine Manual (Apr 2018)|BOLD:AAJ2138
Limnophyes bidumus|25128-B09|Kate Perez|BIN Taxonomy Match (Mar 2018)|BOLD:AAJ2138
Limnophyes bidumus|25130-G03|Kate Perez|BOLD ID Engine Manual (Apr 2018)|BOLD:AAJ2138
Limnophyes bidumus|ATNA493|Elisabeth Stur||BOLD:AAJ2138
Limnophyes|CCDB24229-D09|Sofia Wiedenbrug|BIN Taxonomy Match|BOLD:ACU4930
Limnophyes sp. 4ES|BC-ZSM-DIP-22491-E02|Dieter Doczkal||BOLD:AAI1342
Limnophyes sp. 4ES|CCDB24037-B12|Sofia Wiedenbrug|BIN Taxonomy Match|BOLD:AAI1342
Limnophyes sp. 4ES|CCDB22605-A09|Sofia Wiedenbrug||BOLD:AAI1342
Limnophyes sp. 4ES|CCDB24229-H05|Sofia Wiedenbrug|BIN Taxonomy Match|BOLD:AAI1342
Limnophyes sp. 4ES|CCDB24229-H11|Sofia Wiedenbrug|BIN Taxonomy Match|BOLD:AAI1342
Limnophyes sp. 4ES|ZSM34342-B09|Sofia Wiedenbrug|Morphology|BOLD:AAI1342
Limnophyes sp. 4ES|ZSM34342-G02|Sofia Wiedenbrug|Morphology|BOLD:AAI1342
Limnophyes sp. 4ES|BC-ZSM-DIP-22551-C03|Dieter Doczkal||BOLD:AAI1342
Limnophyes sp. 4ES|CCDB24038-F06|Sofia Wiedenbrug|BIN Taxonomy Match|BOLD:AAI1342
Limnophyes sp. 4ES|CCDB24039-E02|Sofia Wiedenbrug|BIN Taxonomy Match|BOLD:AAI1342
Limnophyes sp. 4ES|ATNA251|Elisabeth Stur||BOLD:AAI1342
Limnophyes sp. 4ES|ATNA287|Elisabeth Stur||BOLD:AAI1342
Limnophyes sp. 4ES|ATNA426|Elisabeth Stur||BOLD:AAI1342
Limnophyes sp. 4ES|ATNA492|Elisabeth Stur||BOLD:AAI1342
Limnophyes sp. 4ES|ATNA491|Elisabeth Stur||BOLD:AAI1342
Chironomidae|24464-B03|Mikko J Tiusanen|Tree based identification|BOLD:ACU5458
Thienemannia libanica|ES13|Elisabeth Stur||BOLD:AAW6669
Chironomidae|24468-F12|Mikko J Tiusanen|Tree based identification
Chironomidae|24474-B12|Mikko J Tiusanen|Tree based identification
Limnophyes brachytomus|24469-C11|Kate Perez|BIN Taxonomy Match|BOLD:AAB7912
Limnophyes brachytomus|24372-A08|Kate Perez|BIN Taxonomy Match|BOLD:AAB7912
Limnophyes brachytomus|24354-E07|Kate Perez|BIN Taxonomy Match|BOLD:AAB7912
Limnophyes brachytomus|23974-H06|Kate Perez|BIN Taxonomy Match|BOLD:AAB7912
Limnophyes brachytomus|24373-H05|Kate Perez|BIN Taxonomy Match|BOLD:AAB7912
Limnophyes brachytomus|24465-E01|Kate Perez|BIN Taxonomy Match|BOLD:AAB7912
Limnophyes brachytomus|24465-C05|Kate Perez|BIN Taxonomy Match|BOLD:AAB7912
Limnophyes brachytomus|24539-E09|Kate Perez|BIN Taxonomy Match|BOLD:AAB7912
Limnophyes brachytomus|24468-D07|Kate Perez|BIN Taxonomy Match|BOLD:AAB7912
Limnophyes brachytomus|24406-H01|Kate Perez|BIN Taxonomy Match|BOLD:AAB7912
Limnophyes brachytomus|24539-H08|Kate Perez|BIN Taxonomy Match|BOLD:AAB7912
Limnophyes brachytomus|ATNA213|Elisabeth Stur||BOLD:AAB7912
Limnophyes brachytomus|Finnmark695|Elisabeth Stur||BOLD:AAU5476
Limnophyes brachytomus|BIOUG04296-B06|Kate Perez|BIN Taxonomy Match|BOLD:AAU5476
Limnophyes brachytomus|Finnmark785|G. A. Halvorsen||BOLD:AAU5476
Limnophyes|ZMUO.025912|Lauri Paasivirta||BOLD:AAU5476
Limnophyes brachytomus|BIOUG15544-F11|Kate Perez|BIN Taxonomy Match|BOLD:AAU5476
Limnophyes brachytomus|BIOUG15538-D12|Kate Perez|BIN Taxonomy Match|BOLD:AAU5476
Limnophyes brachytomus|ZMUO.026166|Lauri Paasivirta||BOLD:ADF1992
Limnophyes brachytomus|ZMUO.026165|Lauri Paasivirta||BOLD:ADF1992
Limnophyes|CCDB24229-C07|Sofia Wiedenbrug|BIN Taxonomy Match|BOLD:ACU4607
Limnophyes|CCDB24228-F02|Sofia Wiedenbrug|BIN Taxonomy Match|BOLD:ACU4607
Limnophyes gurgicola|CCDB24228-H06|Sofia Wiedenbrug|BIN Taxonomy Match|BOLD:ACU4826
Limnophyes gurgicola|CCDB24037-G10|Sofia Wiedenbrug|BIN Taxonomy Match|BOLD:ACU4826
Limnophyes gurgicola|CCDB24038-F11|Sofia Wiedenbrug|BIN Taxonomy Match|BOLD:ACU4826
Limnophyes gurgicola|BC-ZSM-DIP-22488-H05|Dieter Doczkal|BIN Taxonomy Match|BOLD:ACU4826
Limnophyes gurgicola|ZSM34342-G06|Sofia Wiedenbrug|Morphology|BOLD:ACU4826
Limnophyes gurgicola|ZSM34342-F07|Sofia Wiedenbrug|Morphology|BOLD:ACU4826
Limnophyes gurgicola|ZSM34341-H08|Sofia Wiedenbrug|Morphology|BOLD:ACU4826
Limnophyes gurgicola|CCDB24039-B11|Sofia Wiedenbrug|BIN Taxonomy Match|BOLD:ACU4826
Limnophyes gurgicola|CCDB24037-B08|Sofia Wiedenbrug|BIN Taxonomy Match|BOLD:ACU4826
Limnophyes|ZSM-DIP-33136-F09|Caroline Chimeno|BIN Taxonomy Match (Jan 2022)|BOLD:AEE7221
Limnophyes pentaplastus|BIOUG08098-E05|Kate Perez|BIN Taxonomy Match|BOLD:AAE6392
Limnophyes pentaplastus|BIOUG06054-A11|Kate Perez|BIN Taxonomy Match|BOLD:AAE6392
Limnophyes pentaplastus|ZSM-DIP-33134-D11|Caroline Chimeno|BIN Taxonomy Match (Jan 2022)|BO...
Limnophyes pentaplastus|ZSM-DIP-33137-C04|Caroline Chimeno|BIN Taxonomy Match (Jan 2022)|BO...
Limnophyes pentaplastus|ZSM-DIP-33131-D10|Caroline Chimeno|BIN Taxonomy Match (Jan 2022)|BO...
Limnophyes pentaplastus|BIOUG05215-G02|Kate Perez|BIN Taxonomy Match|BOLD:AAE6392
Limnophyes pentaplastus|ATNA358|Elisabeth Stur||BOLD:AAE6392
Limnophyes pentaplastus|CCDB24229-C02|Sofia Wiedenbrug|BIN Taxonomy Match|BOLD:AAE6392
Limnophyes pentaplastus|ZSM-DIP-33139-E11|Caroline Chimeno|BIN Taxonomy Match (Jan 2022)|BO...
Limnophyes pentaplastus|GBOL11506|Sofia Wiedenbrug||BOLD:AAE6392
Limnophyes pentaplastus|BIOUG16632-B10|Kate Perez|BIN Taxonomy Match|BOLD:AAE6392
Limnophyes pentaplastus|BIOUG42761-E03|Kate Perez|BIN Taxonomy Match below Phylum (Jun 2019 ...
Limnophyes pentaplastus|BIOUG15855-H11|Kate Perez|BIN Taxonomy Match|BOLD:AAE6392
Limnophyes pentaplastus|ZSM-DIP-33137-C08|Caroline Chimeno|BIN Taxonomy Match (Jan 2022)|BO...
Limnophyes pentaplastus|ZSM-DIP-33143-F12|Caroline Chimeno|BIN Taxonomy Match (Jan 2022)|BO...
Limnophyes pentaplastus|CCDB24039-H09|Sofia Wiedenbrug|BIN Taxonomy Match|BOLD:AAE6392
Limnophyes pentaplastus|BIOUG16630-H07|Kate Perez|BIN Taxonomy Match|BOLD:AAE6392
Limnophyes pentaplastus|NHRS-BYWS000000950|Yngve Brodin|morphology|BOLD:AAE6392
Limnophyes pentaplastus|SOE284|Elisabeth Stur||BOLD:AAE6392
Limnophyes pentaplastus|BIOUG55122-C12|Stefan Schmidt|BIN Taxonomy Match (Sep 2020)|BOLD ...
Limnophyes pentaplastus|BIOUG16777-A11|Kate Perez|BIN Taxonomy Match|BOLD:AAE6392
Limnophyes pentaplastus|BIOUG42709-C07|Kate Perez|BIN Taxonomy Match below Phylum (Jun 2019 ...
Limnophyes pentaplastus|CH-OSF124|Elisabeth Stur||BOLD:AAE6392
Limnophyes pentaplastus|CH-OSF151|Elisabeth Stur||BOLD:AAE6392
Limnophyes pentaplastus|CH-OSF09|Elisabeth Stur||BOLD:AAE6392
Limnophyes pentaplastus|ZSM-DIP-33136-F07|Caroline Chimeno|BIN Taxonomy Match (Jan 2022)|BO...
Limnophyes pentaplastus|GBOL11509|Sofia Wiedenbrug||BOLD:AAE6392
Limnophyes pentaplastus|CCDB24183-C11|Sofia Wiedenbrug|Morphology|BOLD:AAE6392
Limnophyes pentaplastus|BIOUG15895-E05|Kate Perez|BIN Taxonomy Match|BOLD:AAE6392
Limnophyes pentaplastus|CCDB24037-B09|Sofia Wiedenbrug|BIN Taxonomy Match|BOLD:AAE6392
Limnophyes pentaplastus|CCDB24037-G09|Sofia Wiedenbrug|BIN Taxonomy Match|BOLD:AAE6392
Limnophyes pentaplastus|BIOUG15464-F01|Kate Perez|BIN Taxonomy Match|BOLD:AAE6392
Limnophyes pentaplastus|CCDB24228-G12|Sofia Wiedenbrug|BIN Taxonomy Match|BOLD:AAE6392
Limnophyes pentaplastus|BIOUG06056-H05|Kate Perez|BIN Taxonomy Match|BOLD:AAE6392
Limnophyes pentaplastus|ZSM-DIP-33144-A07|Caroline Chimeno|BIN Taxonomy Match (Jan 2022)|BO...
[illegible]

Limnophyes pentaplastus|CCDB24228-G12|Sofia Wiedenbrug|BIN Taxonomy Match|BOLD:AAE6392
Limnophyes pentaplastus|BIOUG06056-H05|Kate Perez|BIN Taxonomy Match|BOLD:AAE6392
Limnophyes pentaplastus|ZSM-DIP-33144-A07|Caroline Chimeno|BIN Taxonomy Match (Jan 2022)|BO...
Limnophyes pentaplastus|ZSM-DIP-33132-A02|Caroline Chimeno|BIN Taxonomy Match (Jan 2022)|BO...
Limnophyes pentaplastus|ZSM-DIP-33137-C02|Caroline Chimeno|BIN Taxonomy Match (Jan 2022)|BO...
Limnophyes pentaplastus|CCDB24038-C04|Sofia Wiedenbrug|BIN Taxonomy Match|BOLD:AAE6392
Limnophyes pentaplastus|ZSM-DIP-33137-C05|Caroline Chimeno|BIN Taxonomy Match (Jan 2022)|BO...
Limnophyes pentaplastus|BC-ZSM-DIP-22491-G07|Dieter Doczkal||BOLD:AAE6392
Limnophyes pentaplastus|BC-ZSM-DIP-22488-H09|Dieter Doczkal||BOLD:AAE6392
Limnophyes pentaplastus|ZSM34342-G05|Sofia Wiedenbrug|Morphology
Limnophyes pentaplastus|ZSM34342-E03|Sofia Wiedenbrug|Morphology|BOLD:AAE6392
Limnophyes pentaplastus|ZSM34342-C10|Sofia Wiedenbrug|Morphology|BOLD:AAE6392
Limnophyes pentaplastus|BC-ZSM-DIP-22488-E06|Dieter Doczkal||BOLD:AAE6392
Limnophyes pentaplastus|CCDB24037-G06|Sofia Wiedenbrug|BIN Taxonomy Match|BOLD:AAE6392
Limnophyes pentaplastus|CCDB24039-H06|Sofia Wiedenbrug|BIN Taxonomy Match|BOLD:AAE6392
Limnophyes pentaplastus|BIOUG42678-G05|Kate Perez|BIN Taxonomy Match below Phylum (Jun 2019 ...
Limnophyes pentaplastus|BIOUG55127-B01|Stefan Schmidt|BIN Taxonomy Match (Sep 2020)|BOLD ...
Limnophyes pentaplastus|CCDB24229-D07|Sofia Wiedenbrug|BIN Taxonomy Match|BOLD:AAE6392
Limnophyes pentaplastus|ES165|Elisabeth Stur||BOLD:AAE6392
Limnophyes pentaplastus|BIOUG15797-F02|Kate Perez|BIN Taxonomy Match|BOLD:AAE6392
Limnophyes pentaplastus|SOE455|Elisabeth Stur||BOLD:AAE6392
Limnophyes pentaplastus|EJV-20110196|Lauri Paasivirta||BOLD:AAE6392
Limnophyes pentaplastus|ZMUO.024576|Lauri Paasivirta||BOLD:AAE6392
Limnophyes pentaplastus|ZMUO.024577|Lauri Paasivirta||BOLD:AAE6392
Limnophyes pentaplastus|TRD-CH46|Elisabeth Stur||BOLD:AAE6392
Limnophyes pentaplastus|EJV-20110195|Lauri Paasivirta||BOLD:AAE6392
Limnophyes pentaplastus|EJV-20110194|Lauri Paasivirta||BOLD:AAE6392
Limnophyes spinigus|ZMUO.024738|Lauri Paasivirta||BOLD:ADA8578
Limnophyes spinigus|ZMUO.026133|Lauri Paasivirta||BOLD:ADA8578
Limnophyes spinigus|ZMUO.026132|Lauri Paasivirta||BOLD:ADA8578
Limnophyes|ZMUO.024916|Lauri Paasivirta||BOLD:ADA8578
Limnophyes angelicae|ZMUO.024735|Lauri Paasivirta||BOLD:ADA8578
Limnophyes angelicae|ZMUO.024734|Lauri Paasivirta||BOLD:ADA8578
Limnophyes|ZMUO.024917|Lauri Paasivirta||BOLD:ADA8578
Limnophyes difficilis|NHRS-BYWS000000544|Yngve Brodin|morphology|BOLD:ACR2664
Limnophyes difficilis|NHRS-BYWS000000437|Yngve Brodin|morphology|BOLD:ACR2664
Limnophyes habilis|BIOUG16778-D08|Kate Perez|BIN Taxonomy Match|BOLD:AAJ2130
Limnophyes habilis|BIOUG16775-H11|Kate Perez|BIN Taxonomy Match|BOLD:AAJ2130
Limnophyes habilis|BIOUG16721-C08|Kate Perez|BIN Taxonomy Match|BOLD:AAJ2130
Limnophyes habilis|BIOUG16855-E09|Kate Perez|BIN Taxonomy Match|BOLD:AAJ2130
Limnophyes habilis|BIOUG16779-E06|Kate Perez|BIN Taxonomy Match|BOLD:AAJ2130
Limnophyes habilis|BIOUG16782-A03|Kate Perez|BIN Taxonomy Match|BOLD:AAJ2130
Limnophyes habilis|BIOUG16785-D02|Kate Perez|BIN Taxonomy Match|BOLD:AAJ2130
Limnophyes habilis|BIOUG17120-C10|Kate Perez|BIN Taxonomy Match|BOLD:AAJ2130
Limnophyes habilis|BIOUG42748-F04|Kate Perez|BIN Taxonomy Match below Phylum (Jun 2019)|BOL...
Limnophyes habilis|BIOUG16785-A10|Kate Perez|BIN Taxonomy Match|BOLD:AAJ2130
Limnophyes habilis|BIOUG16779-E04|Kate Perez|BIN Taxonomy Match|BOLD:AAJ2130
Limnophyes habilis|ZSM-DIP-33142-A09|Caroline Chimeno|BIN Taxonomy Match (Jan 2022)|BOLD ...
Limnophyes habilis|BIOUG16853-D12|Kate Perez|BIN Taxonomy Match|BOLD:AAJ2130
Limnophyes habilis|BIOUG16782-G02|Kate Perez|BIN Taxonomy Match|BOLD:AAJ2130
Limnophyes habilis|BIOUG16779-F08|Kate Perez|BIN Taxonomy Match|BOLD:AAJ2130
Limnophyes habilis|BIOUG16784-A05|Kate Perez|BIN Taxonomy Match|BOLD:AAJ2130
Limnophyes habilis|BIOUG16740-B10|Kate Perez|BIN Taxonomy Match|BOLD:AAJ2130
Limnophyes habilis|BIOUG16785-A05|Kate Perez|BIN Taxonomy Match|BOLD:AAJ2130
Limnophyes habilis|BIOUG42757-B07|Kate Perez|BIN Taxonomy Match below Phylum (Jun 2019)|BOL...
Limnophyes habilis|BIOUG42918-C02|Kate Perez|BIN Taxonomy Match below Phylum (Jun 2019)|BOL...
Limnophyes habilis|BIOUG16785-B05|Kate Perez|BIN Taxonomy Match|BOLD:AAJ2130
Limnophyes habilis|BIOUG16296-E09|Kate Perez|BIN Taxonomy Match|BOLD:AAJ2130
Limnophyes habilis|BIOUG16430-H01|Kate Perez|BIN Taxonomy Match|BOLD:AAJ2130
Limnophyes habilis|BIOUG16432-E11|Kate Perez|BIN Taxonomy Match|BOLD:AAJ2130
Limnophyes habilis|BIOUG16626-D01|Kate Perez|BIN Taxonomy Match|BOLD:AAJ2130
Limnophyes habilis|BIOUG15792-G12|Kate Perez|BIN Taxonomy Match|BOLD:AAJ2130
Limnophyes habilis|BIOUG15765-D12|Kate Perez|BIN Taxonomy Match|BOLD:AAJ2130
Limnophyes habilis|BIOUG16297-H09|Kate Perez|BIN Taxonomy Match|BOLD:AAJ2130
Limnophyes habilis|BIOUG16552-A03|Kate Perez|BIN Taxonomy Match|BOLD:AAJ2130
Limnophyes habilis|BIOUG16631-B11|Kate Perez|BIN Taxonomy Match|BOLD:AAJ2130
Limnophyes habilis|ZMUO.024655|Lauri Paasivirta||BOLD:AAJ2130
Limnophyes habilis|BIOUG15905-B11|Kate Perez|BIN Taxonomy Match|BOLD:AAJ2130
Limnophyes habilis|BIOUG16712-C12|Kate Perez|BIN Taxonomy Match|BOLD:AAJ2130
Limnophyes habilis|BIOUG16259-D02|Kate Perez|BIN Taxonomy Match|BOLD:AAJ2130
Limnophyes habilis|BIOUG15765-F07|Kate Perez|BIN Taxonomy Match|BOLD:AAJ2130
Limnophyes habilis|BIOUG15966-G07|Kate Perez|BIN Taxonomy Match|BOLD:AAJ2130
Limnophyes habilis|BIOUG55193-B05|Stefan Schmidt|BIN Taxonomy Match (Sep 2020)|BOLD:AAJ2130
Limnophyes habilis|TRD-CH124|Elisabeth Stur||BOLD:AAJ2130
Limnophyes habilis|BIOUG16785-E12|Kate Perez|BIN Taxonomy Match|BOLD:AAJ2130
Limnophyes habilis|BC-ZSM-DIP-22488-G01|Dieter Doczkal||BOLD:AAJ2130
Limnophyes habilis|BIOUG08367-D07|Kate Perez|BIN Taxonomy Match|BOLD:AAJ2130
Limnophyes habilis|BIOUG16776-A03|Kate Perez|BIN Taxonomy Match|BOLD:AAJ2130
Limnophyes habilis|BIOUG16740-H03|Kate Perez|BIN Taxonomy Match|BOLD:AAJ2130
Limnophyes habilis|BIOUG16707-H06|Kate Perez|BIN Taxonomy Match|BOLD:AAJ2130
Limnophyes habilis|BIOUG16778-H07|Kate Perez|BIN Taxonomy Match|BOLD:AAJ2130
Limnophyes habilis|BIOUG42915-B01|Kate Perez|BIN Taxonomy Match below Phylum (Jun 2019)|BOL...
Limnophyes habilis|BIOUG42714-A08|Kate Perez|BIN Taxonomy Match below Phylum (Jun 2019)|BOL...
Limnophyes habilis|BIOUG16779-D05|Kate Perez|BIN Taxonomy Match|BOLD:AAJ2130
Limnophyes habilis|BIOUG42961-G01|Kate Perez|BIN Taxonomy Match below Phylum (Jun 2019)|BOL...
Limnophyes habilis|BIOUG16783-F05|Kate Perez|BIN Taxonomy Match|BOLD:AAJ2130
Limnophyes habilis|BIOUG16892-H09|Kate Perez|BIN Taxonomy Match|BOLD:AAJ2130
Limnophyes habilis|BIOUG16777-G11|Kate Perez|BIN Taxonomy Match|BOLD:AAJ2130
Limnophyes habilis|BIOUG42764-E01|Kate Perez|BIN Taxonomy Match below Phylum (Jun 2019)|BOL...
Limnophyes habilis|BIOUG16738-H09|Kate Perez|BIN Taxonomy Match|BOLD:AAJ2130
Limnophyes habilis|BIOUG15649-E08|Kate Perez|BIN Taxonomy Match|BOLD:AAJ2130
Limnophyes habilis|BIOUG16775-C11|Kate Perez|BIN Taxonomy Match|BOLD:AAJ2130
Limnophyes habilis|BIOUG16779-A02|Kate Perez|BIN Taxonomy Match|BOLD:AAJ2130
Limnophyes habilis|BIOUG16778-G01|Kate Perez|BIN Taxonomy Match|BOLD:AAJ2130
Limnophyes habilis|BIOUG16738-C10|Kate Perez|BIN Taxonomy Match|BOLD:AAJ2130
Limnophyes habilis|BIOUG16779-H10|Kate Perez|BIN Taxonomy Match|BOLD:AAJ2130
Limnophyes habilis|BIOUG16739-F05|Kate Perez|BIN Taxonomy Match|BOLD:AAJ2130
Limnophyes habilis|BIOUG16782-D11|Kate Perez|BIN Taxonomy Match|BOLD:AAJ2130
Limnophyes habilis|BIOUG16784-H06|Kate Perez|BIN Taxonomy Match|BOLD:AAJ2130
Limnophyes habilis|BIOUG17099-G03|Kate Perez|BIN Taxonomy Match|BOLD:AAJ2130
Limnophyes habilis|BIOUG16740-B04|Kate Perez|BIN Taxonomy Match|BOLD:AAJ2130
Limnophyes habilis|BIOUG15457-F04|Kate Perez|BIN Taxonomy Match|BOLD:AAJ2130
Limnophyes habilis|BIOUG16778-F04|Kate Perez|BIN Taxonomy Match|BOLD:AAJ2130

Limnophyes habilis|BIOUG16740-B04|Kate Perez|BIN Taxonomy Match|BOLD:AAJ2130
Limnophyes habilis|BIOUG15457-F04|Kate Perez|BIN Taxonomy Match|BOLD:AAJ2130
Limnophyes habilis|BIOUG16778-F04|Kate Perez|BIN Taxonomy Match|BOLD:AAJ2130
Limnophyes habilis|BIOUG16785-F11|Kate Perez|BIN Taxonomy Match|BOLD:AAJ2130
Limnophyes habilis|BIOUG16782-D01|Kate Perez|BIN Taxonomy Match|BOLD:AAJ2130
Limnophyes habilis|BIOUG16783-F02|Kate Perez|BIN Taxonomy Match|BOLD:AAJ2130
Limnophyes habilis|BIOUG15952-B09|Kate Perez|BIN Taxonomy Match|BOLD:AAJ2130
Limnophyes habilis|BIOUG16784-H09|Kate Perez|BIN Taxonomy Match|BOLD:AAJ2130
Limnophyes habilis|BIOUG16778-B03|Kate Perez|BIN Taxonomy Match|BOLD:AAJ2130
Limnophyes habilis|BIOUG16778-H10|Kate Perez|BIN Taxonomy Match|BOLD:AAJ2130
Limnophyes habilis|BIOUG16779-A10|Kate Perez|BIN Taxonomy Match|BOLD:AAJ2130
Limnophyes habilis|BIOUG16778-C09|Kate Perez|BIN Taxonomy Match|BOLD:AAJ2130
Limnophyes habilis|BIOUG15963-B04|Kate Perez|BIN Taxonomy Match|BOLD:AAJ2130
Limnophyes habilis|BIOUG16332-D09|Kate Perez|BIN Taxonomy Match|BOLD:AAJ2130
Limnophyes habilis|BIOUG16711-B03|Kate Perez|BIN Taxonomy Match|BOLD:AAJ2130
Limnophyes habilis|BIOUG16784-G03|Kate Perez|BIN Taxonomy Match|BOLD:AAJ2130
Limnophyes habilis|BIOUG16775-F11|Kate Perez|BIN Taxonomy Match|BOLD:AAJ2130
Limnophyes habilis|BIOUG16626-A09|Kate Perez|BIN Taxonomy Match|BOLD:AAJ2130
Limnophyes habilis|BIOUG42921-E10|Kate Perez|BIN Taxonomy Match below Phylum (Jun 2019)|BOL...
Limnophyes habilis|BIOUG42663-A08|Kate Perez|BIN Taxonomy Match below Phylum (Jun 2019)|BOL...
Limnophyes habilis|BIOUG55105-F08|Stefan Schmidt|BIN Taxonomy Match (Sep 2020)|BOLD:AAJ2130
Limnophyes habilis|BIOUG43080-G09|Kate Perez|BIN Taxonomy Match below Phylum (Jun 2019)|BOL...
Limnophyes habilis|BIOUG15766-G09|Kate Perez|BIN Taxonomy Match|BOLD:AAJ2130
Limnophyes habilis|ZMUO.024656|Lauri Paasivirta||BOLD:AAJ2130
Limnophyes habilis|BIOUG16778-G06|Kate Perez|BIN Taxonomy Match|BOLD:AAJ2130
Limnophyes habilis|BIOUG15853-C12|Kate Perez|BIN Taxonomy Match|BOLD:AAJ2130
Limnophyes habilis|BIOUG16778-G11|Kate Perez|BIN Taxonomy Match|BOLD:AAJ2130
Limnophyes habilis|BIOUG15809-A03|Kate Perez|BIN Taxonomy Match|BOLD:AAJ2130
Limnophyes habilis|BIOUG16625-G02|Kate Perez|BIN Taxonomy Match|BOLD:AAJ2130
Limnophyes habilis|BIOUG16776-G06|Kate Perez|BIN Taxonomy Match|BOLD:AAJ2130
Limnophyes habilis|BIOUG16625-A09|Kate Perez|BIN Taxonomy Match|BOLD:AAJ2130
Limnophyes habilis|BIOUG16198-C09|Kate Perez|BIN Taxonomy Match|BOLD:AAJ2130
Limnophyes habilis|BIOUG16775-E12|Kate Perez|BIN Taxonomy Match|BOLD:AAJ2130
Limnophyes habilis|BIOUG16855-C06|Kate Perez|BIN Taxonomy Match|BOLD:AAJ2130
Limnophyes habilis|BIOUG54160-B08|Stefan Schmidt|BIN Taxonomy Match (Sep 2020)|BOLD:AAJ2130
Limnophyes habilis|BIOUG16784-F11|Kate Perez|BIN Taxonomy Match|BOLD:AAJ2130
Limnophyes habilis|BIOUG16785-A08|Kate Perez|BIN Taxonomy Match|BOLD:AAJ2130
Limnophyes habilis|BIOUG17025-C11|Kate Perez|BIN Taxonomy Match|BOLD:AAJ2130
Limnophyes habilis|BIOUG16435-H11|Kate Perez|BIN Taxonomy Match|BOLD:AAJ2130
Limnophyes habilis|BIOUG16740-A04|Kate Perez|BIN Taxonomy Match|BOLD:AAJ2130
Limnophyes habilis|BIOUG15807-G10|Kate Perez|BIN Taxonomy Match|BOLD:AAJ2130
Limnophyes habilis|BIOUG16721-H08|Kate Perez|BIN Taxonomy Match|BOLD:AAJ2130
Limnophyes habilis|BIOUG15954-E07|Kate Perez|BIN Taxonomy Match|BOLD:AAJ2130
Limnophyes habilis|BIOUG16625-B06|Kate Perez|BIN Taxonomy Match|BOLD:AAJ2130
Limnophyes habilis|BIOUG16627-A03|Kate Perez|BIN Taxonomy Match|BOLD:AAJ2130
Limnophyes habilis|BIOUG16778-C05|Kate Perez|BIN Taxonomy Match|BOLD:AAJ2130
Limnophyes habilis|BIOUG16782-C10|Kate Perez|BIN Taxonomy Match|BOLD:AAJ2130
Limnophyes habilis|BIOUG15184-E01|Kate Perez|BIN Taxonomy Match|BOLD:AAJ2130
Limnophyes habilis|BIOUG16552-G02|Kate Perez|BIN Taxonomy Match|BOLD:AAJ2130
Limnophyes habilis|BIOUG15761-A11|Kate Perez|BIN Taxonomy Match|BOLD:AAJ2130
Limnophyes habilis|BIOUG15720-H11|Kate Perez|BIN Taxonomy Match|BOLD:AAJ2130
Limnophyes habilis|BIOUG16777-B09|Kate Perez|BIN Taxonomy Match|BOLD:AAJ2130
Limnophyes habilis|BIOUG16778-B11|Kate Perez|BIN Taxonomy Match|BOLD:AAJ2130
Limnophyes habilis|BIOUG16777-B05|Kate Perez|BIN Taxonomy Match|BOLD:AAJ2130
Limnophyes habilis|BIOUG16784-F01|Kate Perez|BIN Taxonomy Match|BOLD:AAJ2130
Limnophyes habilis|BIOUG16779-H09|Kate Perez|BIN Taxonomy Match|BOLD:AAJ2130
Limnophyes habilis|BIOUG16777-D04|Kate Perez|BIN Taxonomy Match|BOLD:AAJ2130
Limnophyes habilis|BIOUG16777-C10|Kate Perez|BIN Taxonomy Match|BOLD:AAJ2130
Limnophyes habilis|BIOUG16777-B07|Kate Perez|BIN Taxonomy Match|BOLD:AAJ2130
Limnophyes habilis|BIOUG16779-F10|Kate Perez|BIN Taxonomy Match|BOLD:AAJ2130
Limnophyes habilis|BIOUG16777-C02|Kate Perez|BIN Taxonomy Match|BOLD:AAJ2130
Limnophyes habilis|BIOUG42931-A06|Kate Perez|BIN Taxonomy Match below Phylum (Jun 2019)|BOL...
Limnophyes habilis|BIOUG16782-A07|Kate Perez|BIN Taxonomy Match|BOLD:AAJ2130
Limnophyes habilis|BIOUG16857-F08|Kate Perez|BIN Taxonomy Match|BOLD:AAJ2130
Limnophyes habilis|BIOUG16779-E10|Kate Perez|BIN Taxonomy Match|BOLD:AAJ2130
Limnophyes habilis|ZSM-DIP-33145-C04|Caroline Chimeno|BIN Taxonomy Match (Jan 2022)|BOLD ...
Limnophyes habilis|BIOUG16856-A03|Kate Perez|BIN Taxonomy Match|BOLD:AAJ2130
Limnophyes habilis|BIOUG16740-E03|Kate Perez|BIN Taxonomy Match|BOLD:AAJ2130
Limnophyes habilis|BIOUG16777-D09|Kate Perez|BIN Taxonomy Match|BOLD:AAJ2130
Limnophyes habilis|BIOUG16777-F06|Kate Perez|BIN Taxonomy Match|BOLD:AAJ2130
Limnophyes habilis|BIOUG16777-C12|Kate Perez|BIN Taxonomy Match|BOLD:AAJ2130
Limnophyes habilis|BIOUG16779-D02|Kate Perez|BIN Taxonomy Match|BOLD:AAJ2130
Limnophyes habilis|BIOUG42669-G04|Kate Perez|BIN Taxonomy Match below Phylum (Jun 2019)|BOL...
Limnophyes habilis|BIOUG16894-B02|Kate Perez|BIN Taxonomy Match|BOLD:AAJ2130
Limnophyes habilis|BIOUG16777-D05|Kate Perez|BIN Taxonomy Match|BOLD:AAJ2130
Limnophyes habilis|BIOUG16776-H06|Kate Perez|BIN Taxonomy Match|BOLD:AAJ2130
Limnophyes habilis|BIOUG16738-C11|Kate Perez|BIN Taxonomy Match|BOLD:AAJ2130
Limnophyes habilis|BIOUG16721-E04|Kate Perez|BIN Taxonomy Match|BOLD:AAJ2130
Limnophyes habilis|BIOUG16775-A10|Kate Perez|BIN Taxonomy Match|BOLD:AAJ2130
Limnophyes habilis|BIOUG16784-D11|Kate Perez|BIN Taxonomy Match|BOLD:AAJ2130
Limnophyes habilis|BIOUG54161-F02|Stefan Schmidt|BIN Taxonomy Match (Sep 2020)|BOLD:AAJ2130
Limnophyes habilis|BIOUG16739-F09|Kate Perez|BIN Taxonomy Match|BOLD:AAJ2130
Limnophyes habilis|BIOUG16739-F12|Kate Perez|BIN Taxonomy Match|BOLD:AAJ2130
Limnophyes habilis|BIOUG16782-H06|Kate Perez|BIN Taxonomy Match|BOLD:AAJ2130
Limnophyes habilis|BC-ZSM-DIP-22488-D11|Dieter Doczkal||BOLD:AAJ2130
Limnophyes habilis|BIOUG16721-C11|Kate Perez|BIN Taxonomy Match|BOLD:AAJ2130
Limnophyes habilis|BIOUG16892-G08|Kate Perez|BIN Taxonomy Match|BOLD:AAJ2130
Limnophyes habilis|BIOUG16783-C09|Kate Perez|BIN Taxonomy Match|BOLD:AAJ2130
Limnophyes habilis|BIOUG42961-D03|Kate Perez|BIN Taxonomy Match below Phylum (Jun 2019)|BOL...
Limnophyes habilis|BIOUG16893-A04|Kate Perez|BIN Taxonomy Match|BOLD:AAJ2130
Limnophyes habilis|BIOUG16779-E11|Kate Perez|BIN Taxonomy Match|BOLD:AAJ2130
Limnophyes habilis|BIOUG16720-A09|Kate Perez|BIN Taxonomy Match|BOLD:AAJ2130
Limnophyes habilis|BIOUG16721-G10|Kate Perez|BIN Taxonomy Match|BOLD:AAJ2130
Limnophyes habilis|BIOUG16785-G07|Kate Perez|BIN Taxonomy Match|BOLD:AAJ2130
Limnophyes habilis|BIOUG16721-D12|Kate Perez|BIN Taxonomy Match|BOLD:AAJ2130
Limnophyes habilis|BIOUG16784-D12|Kate Perez|BIN Taxonomy Match|BOLD:AAJ2130
Limnophyes habilis|BIOUG16779-D07|Kate Perez|BIN Taxonomy Match|BOLD:AAJ2130
Limnophyes habilis|BIOUG16738-H02|Kate Perez|BIN Taxonomy Match|BOLD:AAJ2130
Limnophyes habilis|BIOUG15809-E11|Kate Perez|BIN Taxonomy Match|BOLD:AAJ2130
Limnophyes habilis|BIOUG16855-C10|Kate Perez|BIN Taxonomy Match|BOLD:AAJ2130
Limnophyes habilis|BIOUG42675-F07|Kate Perez|BIN Taxonomy Match below Phylum (Jun 2019)|BOL...
Limnophyes habilis|BIOUG42710-E01|Kate Perez|BIN Taxonomy Match below Phylum (Jun 2019)|BOL...
Limnophyes habilis|BIOUG16784-A10|Kate Perez|BIN Taxonomy Match|BOLD:AAJ2130

Limnophyes habilis|BIOUG42675-F07|Kate Perez|BIN Taxonomy Match below Phylum (Jun 2019)|BOL...
Limnophyes habilis|BIOUG42710-E01|Kate Perez|BIN Taxonomy Match below Phylum (Jun 2019)|BOL...
Limnophyes habilis|BIOUG16784-A10|Kate Perez|BIN Taxonomy Match|BOLD:AAJ2130
Limnophyes habilis|BIOUG16893-H06|Kate Perez|BIN Taxonomy Match|BOLD:AAJ2130
Limnophyes habilis|ZSM-DIP-33132-G08|Caroline Chimeno|BIN Taxonomy Match (Jan 2022)|BOLD ...
Limnophyes habilis|BIOUG16785-E08|Kate Perez|BIN Taxonomy Match|BOLD:AAJ2130
Limnophyes habilis|BIOUG16783-B02|Kate Perez|BIN Taxonomy Match|BOLD:AAJ2130
Limnophyes habilis|BIOUG16893-F05|Kate Perez|BIN Taxonomy Match|BOLD:AAJ2130
Limnophyes habilis|BIOUG16777-C03|Kate Perez|BIN Taxonomy Match|BOLD:AAJ2130
Limnophyes habilis|BIOUG16721-G01|Kate Perez|BIN Taxonomy Match|BOLD:AAJ2130
Limnophyes habilis|BIOUG16782-G04|Kate Perez|BIN Taxonomy Match|BOLD:AAJ2130
Limnophyes habilis|BIOUG16721-C04|Kate Perez|BIN Taxonomy Match|BOLD:AAJ2130
Limnophyes habilis|BIOUG16775-D11|Kate Perez|BIN Taxonomy Match|BOLD:AAJ2130
Limnophyes habilis|BIOUG16739-E08|Kate Perez|BIN Taxonomy Match|BOLD:AAJ2130
Limnophyes habilis|BIOUG16776-H08|Kate Perez|BIN Taxonomy Match|BOLD:AAJ2130
Limnophyes habilis|BIOUG16778-C10|Kate Perez|BIN Taxonomy Match|BOLD:AAJ2130
Limnophyes habilis|BIOUG16782-D12|Kate Perez|BIN Taxonomy Match|BOLD:AAJ2130
Limnophyes habilis|BIOUG16892-B05|Kate Perez|BIN Taxonomy Match|BOLD:AAJ2130
Limnophyes habilis|BIOUG16785-E10|Kate Perez|BIN Taxonomy Match|BOLD:AAJ2130
Limnophyes habilis|BIOUG16784-A04|Kate Perez|BIN Taxonomy Match|BOLD:AAJ2130
Limnophyes habilis|BIOUG16783-H09|Kate Perez|BIN Taxonomy Match|BOLD:AAJ2130
Limnophyes habilis|BIOUG16738-C08|Kate Perez|BIN Taxonomy Match|BOLD:AAJ2130
Limnophyes habilis|BIOUG16776-C06|Kate Perez|BIN Taxonomy Match|BOLD:AAJ2130
Limnophyes habilis|BIOUG54164-G04|Stefan Schmidt|BIN Taxonomy Match (Sep 2020)|BOLD:AAJ2130
Limnophyes habilis|BIOUG16785-C03|Kate Perez|BIN Taxonomy Match|BOLD:AAJ2130
Limnophyes habilis|BIOUG16784-E09|Kate Perez|BIN Taxonomy Match|BOLD:AAJ2130
Limnophyes habilis|BIOUG16775-G10|Kate Perez|BIN Taxonomy Match|BOLD:AAJ2130
Limnophyes habilis|BIOUG16333-F11|Kate Perez|BIN Taxonomy Match|BOLD:AAJ2130
Limnophyes habilis|BIOUG16776-F09|Kate Perez|BIN Taxonomy Match|BOLD:AAJ2130
Limnophyes habilis|BIOUG42749-E01|Kate Perez|BIN Taxonomy Match below Phylum (Jun 2019)|BOL...
Limnophyes habilis|BIOUG16854-C12|Kate Perez|BIN Taxonomy Match|BOLD:AAJ2130
Limnophyes habilis|BIOUG16782-E01|Kate Perez|BIN Taxonomy Match|BOLD:AAJ2130
Limnophyes habilis|BIOUG16738-E11|Kate Perez|BIN Taxonomy Match|BOLD:AAJ2130
Limnophyes habilis|BIOUG17065-E02|Kate Perez|BIN Taxonomy Match|BOLD:AAJ2130
Limnophyes habilis|BIOUG16782-B07|Kate Perez|BIN Taxonomy Match|BOLD:AAJ2130
Limnophyes habilis|BIOUG42706-D09|Kate Perez|BIN Taxonomy Match below Phylum (Jun 2019)|BOL...
Limnophyes habilis|BIOUG42756-F12|Kate Perez|BIN Taxonomy Match below Phylum (Jun 2019)|BOL...
Limnophyes habilis|BIOUG16784-A07|Kate Perez|BIN Taxonomy Match|BOLD:AAJ2130
Limnophyes habilis|BIOUG42742-G10|Kate Perez|BIN Taxonomy Match below Phylum (Jun 2019)|BOL...
Limnophyes habilis|BIOUG16779-B07|Kate Perez|BIN Taxonomy Match|BOLD:AAJ2130
Limnophyes habilis|BIOUG16893-A06|Kate Perez|BIN Taxonomy Match|BOLD:AAJ2130
Limnophyes habilis|BIOUG16784-G11|Kate Perez|BIN Taxonomy Match|BOLD:AAJ2130
Limnophyes habilis|BIOUG16778-B01|Kate Perez|BIN Taxonomy Match|BOLD:AAJ2130
Limnophyes habilis|ZSM-DIP-33133-F08|Caroline Chimeno|BIN Taxonomy Match (Jan 2022)|BOLD ...
Limnophyes habilis|BIOUG16739-H08|Kate Perez|BIN Taxonomy Match|BOLD:AAJ2130
Limnophyes habilis|BIOUG16785-B07|Kate Perez|BIN Taxonomy Match|BOLD:AAJ2130
Limnophyes habilis|BIOUG16784-F09|Kate Perez|BIN Taxonomy Match|BOLD:AAJ2130
Limnophyes habilis|BIOUG16779-G01|Kate Perez|BIN Taxonomy Match|BOLD:AAJ2130
Limnophyes habilis|BIOUG42964-F04|Kate Perez|BIN Taxonomy Match below Phylum (Jun 2019)|BOL...
Limnophyes habilis|BIOUG42919-G10|Kate Perez|BIN Taxonomy Match below Phylum (Jun 2019)|BOL...
Limnophyes habilis|BIOUG15967-C04|Kate Perez|BIN Taxonomy Match|BOLD:AAJ2130
Limnophyes habilis|BIOUG16625-G04|Kate Perez|BIN Taxonomy Match|BOLD:AAJ2130
Limnophyes habilis|BIOUG16739-C09|Kate Perez|BIN Taxonomy Match|BOLD:AAJ2130
Limnophyes habilis|BIOUG16777-A05|Kate Perez|BIN Taxonomy Match|BOLD:AAJ2130
Limnophyes habilis|BIOUG16738-G11|Kate Perez|BIN Taxonomy Match|BOLD:AAJ2130
Limnophyes habilis|BIOUG16739-G04|Kate Perez|BIN Taxonomy Match|BOLD:AAJ2130
Limnophyes habilis|BIOUG16739-C06|Kate Perez|BIN Taxonomy Match|BOLD:AAJ2130
Limnophyes habilis|BIOUG16777-A06|Kate Perez|BIN Taxonomy Match|BOLD:AAJ2130
Limnophyes habilis|BIOUG42678-G03|Kate Perez|BIN Taxonomy Match below Phylum (Jun 2019)|BOL...
Limnophyes habilis|BIOUG16784-C04|Kate Perez|BIN Taxonomy Match|BOLD:AAJ2130
Limnophyes habilis|BIOUG16782-G01|Kate Perez|BIN Taxonomy Match|BOLD:AAJ2130
Limnophyes habilis|BIOUG16785-B10|Kate Perez|BIN Taxonomy Match|BOLD:AAJ2130
Limnophyes habilis|BIOUG16785-F01|Kate Perez|BIN Taxonomy Match|BOLD:AAJ2130
Limnophyes habilis|BIOUG16738-E06|Kate Perez|BIN Taxonomy Match|BOLD:AAJ2130
Limnophyes habilis|BIOUG16779-G12|Kate Perez|BIN Taxonomy Match|BOLD:AAJ2130
Limnophyes habilis|BIOUG16740-B05|Kate Perez|BIN Taxonomy Match|BOLD:AAJ2130
Limnophyes habilis|BIOUG16739-D12|Kate Perez|BIN Taxonomy Match|BOLD:AAJ2130
Limnophyes habilis|BIOUG16776-D03|Kate Perez|BIN Taxonomy Match|BOLD:AAJ2130
Limnophyes habilis|BIOUG16739-G09|Kate Perez|BIN Taxonomy Match|BOLD:AAJ2130
Limnophyes habilis|BIOUG16777-F02|Kate Perez|BIN Taxonomy Match|BOLD:AAJ2130
Limnophyes habilis|BIOUG16776-G07|Kate Perez|BIN Taxonomy Match|BOLD:AAJ2130
Limnophyes habilis|BIOUG16775-H06|Kate Perez|BIN Taxonomy Match|BOLD:AAJ2130
Limnophyes habilis|BIOUG16775-D12|Kate Perez|BIN Taxonomy Match|BOLD:AAJ2130
Limnophyes habilis|BIOUG16778-B06|Kate Perez|BIN Taxonomy Match|BOLD:AAJ2130
Limnophyes habilis|BIOUG16784-A08|Kate Perez|BIN Taxonomy Match|BOLD:AAJ2130
Limnophyes habilis|BIOUG04153-D09|Kate Perez|BIN Taxonomy Match|BOLD:AAJ2130
Limnophyes habilis|BIOUG06446-D09|Kate Perez|BIN Taxonomy Match|BOLD:AAJ2130
Limnophyes habilis|BIOUG16739-H02|Kate Perez|BIN Taxonomy Match|BOLD:AAJ2130
Limnophyes habilis|BIOUG16782-F09|Kate Perez|BIN Taxonomy Match|BOLD:AAJ2130
Limnophyes habilis|BIOUG16783-D08|Kate Perez|BIN Taxonomy Match|BOLD:AAJ2130
Limnophyes habilis|BIOUG16784-G05|Kate Perez|BIN Taxonomy Match|BOLD:AAJ2130
Limnophyes habilis|BIOUG16785-G02|Kate Perez|BIN Taxonomy Match|BOLD:AAJ2130
Limnophyes habilis|BC-ZSM-DIP-22488-F04|Dieter Doczkal||BOLD:AAJ2130
Limnophyes habilis|BIOUG42914-E04|Kate Perez|BIN Taxonomy Match below Phylum (Jun 2019)|BOL...
Limnophyes habilis|BIOUG42925-G12|Kate Perez|BIN Taxonomy Match below Phylum (Jun 2019)|BOL...
Limnophyes habilis|BIOUG42920-D03|Kate Perez|BIN Taxonomy Match below Phylum (Jun 2019)|BOL...
Limnophyes habilis|BIOUG42666-B02|Kate Perez|BIN Taxonomy Match below Phylum (Jun 2019)|BOL...
Limnophyes habilis|BIOUG42672-B06|Kate Perez|BIN Taxonomy Match below Phylum (Jun 2019)|BOL...
Limnophyes habilis|BIOUG42677-D05|Kate Perez|BIN Taxonomy Match below Phylum (Jun 2019)|BOL...
Limnophyes habilis|BIOUG42708-F04|Kate Perez|BIN Taxonomy Match below Phylum (Jun 2019)|BOL...
Limnophyes habilis|ZSM-DIP-33132-F05|Caroline Chimeno|BIN Taxonomy Match (Jan 2022)|BOLD ...
Limnophyes habilis|ZSM-DIP-33130-D08|Caroline Chimeno|BIN Taxonomy Match (Jan 2022)|BOLD ...
Limnophyes habilis|BIOUG42720-B12|Kate Perez|BIN Taxonomy Match below Phylum (Jun 2019)|BOL...
Limnophyes habilis|BIOUG42751-E04|Kate Perez|BIN Taxonomy Match below Phylum (Jun 2019)|BOL...
Limnophyes habilis|BIOUG42665-F05|Kate Perez|BIN Taxonomy Match below Phylum (Jun 2019)|BOL...
Limnophyes habilis|BIOUG42720-D01|Kate Perez|BIN Taxonomy Match below Phylum (Jun 2019)|BOL...
Limnophyes habilis|BIOUG42753-B09|Kate Perez|BIN Taxonomy Match below Phylum (Jun 2019)|BOL...
Limnophyes habilis|BIOUG16776-A08|Kate Perez|BIN Taxonomy Match|BOLD:AAJ2130
Limnophyes habilis|BIOUG16782-E07|Kate Perez|BIN Taxonomy Match|BOLD:AAJ2130
Limnophyes habilis|BIOUG16720-C08|Kate Perez|BIN Taxonomy Match|BOLD:AAJ2130
Limnophyes habilis|BIOUG16775-F02|Kate Perez|BIN Taxonomy Match|BOLD:AAJ2130
Limnophyes habilis|BIOUG16785-G04|Kate Perez|BIN Taxonomy Match|BOLD:AAJ2130
Limnophyes habilis|BIOUG16740-A05|Kate Perez|BIN Taxonomy Match|BOLD:AAJ2130

Limnophyes habilis|BIOUG[illegible]|Kate Perez|BIN Taxonomy Match|BOLD:AAJ2130
Limnophyes habilis|BIOUG16775-F02|Kate Perez|BIN Taxonomy Match|BOLD:AAJ2130
Limnophyes habilis|BIOUG16785-G04|Kate Perez|BIN Taxonomy Match|BOLD:AAJ2130
Limnophyes habilis|BIOUG16740-A05|Kate Perez|BIN Taxonomy Match|BOLD:AAJ2130
Limnophyes habilis|BIOUG16784-C10|Kate Perez|BIN Taxonomy Match|BOLD:AAJ2130
Limnophyes habilis|BIOUG16782-D03|Kate Perez|BIN Taxonomy Match|BOLD:AAJ2130
Limnophyes habilis|BIOUG16775-B04|Kate Perez|BIN Taxonomy Match|BOLD:AAJ2130
Limnophyes habilis|BIOUG16776-F02|Kate Perez|BIN Taxonomy Match|BOLD:AAJ2130
Limnophyes habilis|BIOUG16776-F05|Kate Perez|BIN Taxonomy Match|BOLD:AAJ2130
Limnophyes habilis|BIOUG42678-D05|Kate Perez|BIN Taxonomy Match below Phylum (Jun 2019)|BOL...
Limnophyes habilis|BIOUG04437-E12|Kate Perez|BIN Taxonomy Match|BOLD:AAJ2130
Limnophyes habilis|NHRS-BYWS000000925|Yngve Brodin|morphology|BOLD:AAJ2130
Limnophyes habilis|CCDB24037-C04|Sofia Wiedenbrug|BIN Taxonomy Match|BOLD:AAJ2130
Limnophyes habilis|BIOUG16783-H07|Kate Perez|BIN Taxonomy Match|BOLD:AAJ2130
Limnophyes habilis|BIOUG16996-A05|Kate Perez|BIN Taxonomy Match|BOLD:AAJ2130
Limnophyes habilis|BIOUG16738-A01|Kate Perez|BIN Taxonomy Match|BOLD:AAJ2130
Limnophyes habilis|BIOUG04433-F04|Kate Perez|BIN Taxonomy Match|BOLD:AAJ2130
Limnophyes habilis|BIOUG16854-C03|Kate Perez|BIN Taxonomy Match|BOLD:AAJ2130
Limnophyes habilis|BIOUG16857-B04|Kate Perez|BIN Taxonomy Match|BOLD:AAJ2130
Limnophyes habilis|BIOUG16720-F05|Kate Perez|BIN Taxonomy Match|BOLD:AAJ2130
Limnophyes habilis|BIOUG42701-A06|Kate Perez|BIN Taxonomy Match below Phylum (Jun 2019)|BOL...
Limnophyes habilis|BIOUG16738-G06|Kate Perez|BIN Taxonomy Match|BOLD:AAJ2130
Limnophyes habilis|BIOUG16476-D01|Kate Perez|BIN Taxonomy Match|BOLD:AAJ2130
Limnophyes habilis|BIOUG16720-G03|Kate Perez|BIN Taxonomy Match|BOLD:AAJ2130
Limnophyes habilis|BIOUG16776-G09|Kate Perez|BIN Taxonomy Match|BOLD:AAJ2130
Limnophyes habilis|BIOUG16783-E04|Kate Perez|BIN Taxonomy Match|BOLD:AAJ2130
Limnophyes habilis|BIOUG42702-E10|Kate Perez|BIN Taxonomy Match below Phylum (Jun 2019)|BOL...
Limnophyes habilis|BIOUG15966-A04|Kate Perez|BIN Taxonomy Match|BOLD:AAJ2130
Limnophyes habilis|BIOUG16783-C05|Kate Perez|BIN Taxonomy Match|BOLD:AAJ2130
Limnophyes habilis|BIOUG16783-C07|Kate Perez|BIN Taxonomy Match|BOLD:AAJ2130
Limnophyes habilis|BIOUG16775-E04|Kate Perez|BIN Taxonomy Match|BOLD:AAJ2130
Limnophyes habilis|BIOUG16853-H10|Kate Perez|BIN Taxonomy Match|BOLD:AAJ2130
Limnophyes habilis|BIOUG16854-B08|Kate Perez|BIN Taxonomy Match|BOLD:AAJ2130
Limnophyes habilis|BIOUG16854-C08|Kate Perez|BIN Taxonomy Match|BOLD:AAJ2130
Limnophyes habilis|BIOUG16854-E05|Kate Perez|BIN Taxonomy Match|BOLD:AAJ2130
Limnophyes habilis|BIOUG16893-F02|Kate Perez|BIN Taxonomy Match|BOLD:AAJ2130
Limnophyes habilis|BIOUG16625-D03|Kate Perez|BIN Taxonomy Match|BOLD:AAJ2130
Limnophyes habilis|BIOUG16779-E07|Kate Perez|BIN Taxonomy Match|BOLD:AAJ2130
Limnophyes habilis|BIOUG16779-F02|Kate Perez|BIN Taxonomy Match|BOLD:AAJ2130
Limnophyes habilis|BIOUG16782-F07|Kate Perez|BIN Taxonomy Match|BOLD:AAJ2130
Limnophyes habilis|BIOUG16721-B06|Kate Perez|BIN Taxonomy Match|BOLD:AAJ2130
Limnophyes habilis|BIOUG16775-G11|Kate Perez|BIN Taxonomy Match|BOLD:AAJ2130
Limnophyes habilis|BIOUG16785-C10|Kate Perez|BIN Taxonomy Match|BOLD:AAJ2130
Limnophyes habilis|BIOUG16739-E03|Kate Perez|BIN Taxonomy Match|BOLD:AAJ2130
Limnophyes habilis|BIOUG16739-H05|Kate Perez|BIN Taxonomy Match|BOLD:AAJ2130
Limnophyes habilis|BIOUG16740-D01|Kate Perez|BIN Taxonomy Match|BOLD:AAJ2130
Limnophyes habilis|BIOUG16738-F07|Kate Perez|BIN Taxonomy Match|BOLD:AAJ2130
Limnophyes habilis|BIOUG16777-G03|Kate Perez|BIN Taxonomy Match|BOLD:AAJ2130
Limnophyes habilis|BIOUG16779-A08|Kate Perez|BIN Taxonomy Match|BOLD:AAJ2130
Limnophyes habilis|BIOUG17023-D11|Kate Perez|BIN Taxonomy Match|BOLD:AAJ2130
Limnophyes habilis|BIOUG16893-F09|Kate Perez|BIN Taxonomy Match|BOLD:AAJ2130
Limnophyes habilis|BIOUG16893-G10|Kate Perez|BIN Taxonomy Match|BOLD:AAJ2130
Limnophyes habilis|BIOUG16783-F04|Kate Perez|BIN Taxonomy Match|BOLD:AAJ2130
Limnophyes habilis|BIOUG16436-C04|Kate Perez|BIN Taxonomy Match|BOLD:AAJ2130
Limnophyes habilis|BIOUG16782-G10|Kate Perez|BIN Taxonomy Match|BOLD:AAJ2130
Limnophyes habilis|BIOUG16783-A10|Kate Perez|BIN Taxonomy Match|BOLD:AAJ2130
Limnophyes habilis|BIOUG16631-H04|Kate Perez|BIN Taxonomy Match|BOLD:AAJ2130
Limnophyes habilis|BIOUG16736-H01|Kate Perez|BIN Taxonomy Match|BOLD:AAJ2130
Limnophyes habilis|BIOUG16893-B02|Kate Perez|BIN Taxonomy Match|BOLD:AAJ2130
Limnophyes habilis|BIOUG16778-H04|Kate Perez|BIN Taxonomy Match|BOLD:AAJ2130
Limnophyes habilis|BIOUG16784-C12|Kate Perez|BIN Taxonomy Match|BOLD:AAJ2130
Limnophyes habilis|BIOUG16721-F02|Kate Perez|BIN Taxonomy Match|BOLD:AAJ2130
Limnophyes habilis|BIOUG16779-G09|Kate Perez|BIN Taxonomy Match|BOLD:AAJ2130
Limnophyes habilis|BIOUG16738-G02|Kate Perez|BIN Taxonomy Match|BOLD:AAJ2130
Limnophyes habilis|BIOUG16775-F12|Kate Perez|BIN Taxonomy Match|BOLD:AAJ2130
Limnophyes habilis|BIOUG16778-A10|Kate Perez|BIN Taxonomy Match|BOLD:AAJ2130
Limnophyes habilis|BIOUG16775-B05|Kate Perez|BIN Taxonomy Match|BOLD:AAJ2130
Limnophyes habilis|BIOUG16775-B06|Kate Perez|BIN Taxonomy Match|BOLD:AAJ2130
Limnophyes habilis|BIOUG16738-E05|Kate Perez|BIN Taxonomy Match|BOLD:AAJ2130
Limnophyes habilis|BIOUG16784-G02|Kate Perez|BIN Taxonomy Match|BOLD:AAJ2130
Limnophyes habilis|BIOUG16785-G05|Kate Perez|BIN Taxonomy Match|BOLD:AAJ2130
Limnophyes habilis|BIOUG16783-E12|Kate Perez|BIN Taxonomy Match|BOLD:AAJ2130
Limnophyes habilis|BIOUG17022-G12|Kate Perez|BIN Taxonomy Match|BOLD:AAJ2130
Limnophyes habilis|BIOUG16856-D02|Kate Perez|BIN Taxonomy Match|BOLD:AAJ2130
Limnophyes habilis|BIOUG16892-D04|Kate Perez|BIN Taxonomy Match|BOLD:AAJ2130
Limnophyes habilis|BIOUG16892-G10|Kate Perez|BIN Taxonomy Match|BOLD:AAJ2130
Limnophyes habilis|BIOUG16853-F09|Kate Perez|BIN Taxonomy Match|BOLD:AAJ2130
Limnophyes habilis|BIOUG16778-C01|Kate Perez|BIN Taxonomy Match|BOLD:AAJ2130
Limnophyes habilis|BIOUG16893-H10|Kate Perez|BIN Taxonomy Match|BOLD:AAJ2130
Limnophyes habilis|BIOUG17136-E08|Kate Perez|BIN Taxonomy Match|BOLD:AAJ2130
Limnophyes habilis|BIOUG17023-E10|Kate Perez|BIN Taxonomy Match|BOLD:AAJ2130
Limnophyes habilis|BIOUG16779-D09|Kate Perez|BIN Taxonomy Match|BOLD:AAJ2130
Limnophyes habilis|BIOUG15951-D02|Kate Perez|BIN Taxonomy Match|BOLD:AAJ2130
Limnophyes habilis|BIOUG16893-D09|Kate Perez|BIN Taxonomy Match|BOLD:AAJ2130
Limnophyes habilis|BIOUG16784-A06|Kate Perez|BIN Taxonomy Match|BOLD:AAJ2130
Limnophyes habilis|BIOUG16777-E10|Kate Perez|BIN Taxonomy Match|BOLD:AAJ2130
Limnophyes habilis|BIOUG16740-E06|Kate Perez|BIN Taxonomy Match|BOLD:AAJ2130
Limnophyes habilis|BIOUG16785-E03|Kate Perez|BIN Taxonomy Match|BOLD:AAJ2130
Limnophyes habilis|BIOUG16783-A02|Kate Perez|BIN Taxonomy Match|BOLD:AAJ2130
Limnophyes habilis|BIOUG16777-C04|Kate Perez|BIN Taxonomy Match|BOLD:AAJ2130
Limnophyes habilis|BIOUG16625-F07|Kate Perez|BIN Taxonomy Match|BOLD:AAJ2130
Limnophyes habilis|BIOUG16596-F05|Kate Perez|BIN Taxonomy Match|BOLD:AAJ2130
Limnophyes habilis|BIOUG16739-D03|Kate Perez|BIN Taxonomy Match|BOLD:AAJ2130
Limnophyes habilis|BIOUG15798-G06|Kate Perez|BIN Taxonomy Match|BOLD:AAJ2130
Limnophyes habilis|BIOUG15545-E12|Kate Perez|BIN Taxonomy Match|BOLD:AAJ2130
Limnophyes habilis|BIOUG16785-G10|Kate Perez|BIN Taxonomy Match|BOLD:AAJ2130
Limnophyes habilis|BIOUG16777-F03|Kate Perez|BIN Taxonomy Match|BOLD:AAJ2130
Limnophyes habilis|ZSM-DIP-33138-E03|Caroline Chimeno|BIN Taxonomy Match (Jan 2022)|BOLD ...
Limnophyes habilis|BIOUG16775-A01|Kate Perez|BIN Taxonomy Match|BOLD:AAJ2130
Limnophyes habilis|BIOUG16857-C02|Kate Perez|BIN Taxonomy Match|BOLD:AAJ2130
Limnophyes habilis|BIOUG16785-C01|Kate Perez|BIN Taxonomy Match|BOLD:AAJ2130
Limnophyes habilis|BIOUG16778-E08|Kate Perez|BIN Taxonomy Match|BOLD:AAJ2130
Limnophyes habilis|BIOUG16779-B06|Kate Perez|BIN Taxonomy Match|BOLD:AAJ2130
[illegible]

Limnophyes habilis|BIOUG16785-C01|Kate Perez|BIN Taxonomy Match|BOLD:AAJ2130
Limnophyes habilis|BIOUG16778-E08|Kate Perez|BIN Taxonomy Match|BOLD:AAJ2130
Limnophyes habilis|BIOUG16779-B06|Kate Perez|BIN Taxonomy Match|BOLD:AAJ2130
Limnophyes habilis|BIOUG16776-G10|Kate Perez|BIN Taxonomy Match|BOLD:AAJ2130
Limnophyes habilis|BIOUG16735-G12|Kate Perez|BIN Taxonomy Match|BOLD:AAJ2130
Limnophyes habilis|BIOUG16738-H06|Kate Perez|BIN Taxonomy Match|BOLD:AAJ2130
Limnophyes habilis|BIOUG16778-H05|Kate Perez|BIN Taxonomy Match|BOLD:AAJ2130
Limnophyes habilis|BIOUG16738-H04|Kate Perez|BIN Taxonomy Match|BOLD:AAJ2130
Limnophyes habilis|BIOUG16785-C04|Kate Perez|BIN Taxonomy Match|BOLD:AAJ2130
Limnophyes habilis|BIOUG17022-B09|Kate Perez|BIN Taxonomy Match|BOLD:AAJ2130
Limnophyes habilis|BIOUG16333-E06|Kate Perez|BIN Taxonomy Match|BOLD:AAJ2130
Limnophyes habilis|BIOUG42710-B07|Kate Perez|BIN Taxonomy Match below Phylum (Jun 2019)|BOL...
Limnophyes habilis|BIOUG16740-D02|Kate Perez|BIN Taxonomy Match|BOLD:AAJ2130
Limnophyes habilis|BIOUG16779-B04|Kate Perez|BIN Taxonomy Match|BOLD:AAJ2130
Limnophyes habilis|ZSM-DIP-33131-C05|Caroline Chimeno|BIN Taxonomy Match (Jan 2022)|BOLD ...
Limnophyes habilis|BIOUG16776-E02|Kate Perez|BIN Taxonomy Match|BOLD:AAJ2130
Limnophyes habilis|BIOUG16778-E09|Kate Perez|BIN Taxonomy Match|BOLD:AAJ2130
Limnophyes habilis|BIOUG16778-F03|Kate Perez|BIN Taxonomy Match|BOLD:AAJ2130
Limnophyes habilis|BIOUG16894-A09|Kate Perez|BIN Taxonomy Match|BOLD:AAJ2130
Limnophyes habilis|ATNA233|Elisabeth Stur||BOLD:AAJ2130
Limnophyes habilis|BIOUG16783-F03|Kate Perez|BIN Taxonomy Match|BOLD:AAJ2130
Limnophyes habilis|BIOUG16596-H05|Kate Perez|BIN Taxonomy Match|BOLD:AAJ2130
Limnophyes habilis|BIOUG16712-E12|Kate Perez|BIN Taxonomy Match|BOLD:AAJ2130
Limnophyes habilis|BIOUG16785-C02|Kate Perez|BIN Taxonomy Match|BOLD:AAJ2130
Limnophyes habilis|BIOUG16779-B05|Kate Perez|BIN Taxonomy Match|BOLD:AAJ2130
Limnophyes habilis|BIOUG16783-G06|Kate Perez|BIN Taxonomy Match|BOLD:AAJ2130
Limnophyes habilis|BIOUG16713-D09|Kate Perez|BIN Taxonomy Match|BOLD:AAJ2130
Limnophyes habilis|BIOUG15792-G04|Kate Perez|BIN Taxonomy Match|BOLD:AAJ2130
Limnophyes habilis|BIOUG15806-F09|Kate Perez|BIN Taxonomy Match|BOLD:AAJ2130
Limnophyes habilis|BIOUG16740-A10|Kate Perez|BIN Taxonomy Match|BOLD:AAJ2130
Limnophyes habilis|BIOUG15800-C06|Kate Perez|BIN Taxonomy Match|BOLD:AAJ2130
Limnophyes habilis|BIOUG16853-C10|Kate Perez|BIN Taxonomy Match|BOLD:AAJ2130
Limnophyes habilis|BIOUG16721-E05|Kate Perez|BIN Taxonomy Match|BOLD:AAJ2130
Limnophyes habilis|BIOUG16721-B02|Kate Perez|BIN Taxonomy Match|BOLD:AAJ2130
Limnophyes habilis|BIOUG42760-C12|Kate Perez|BIN Taxonomy Match below Phylum (Jun 2019)|BOL...
Limnophyes habilis|BIOUG16784-E04|Kate Perez|BIN Taxonomy Match|BOLD:AAJ2130
Limnophyes habilis|BIOUG15794-A02|Kate Perez|BIN Taxonomy Match|BOLD:AAJ2130
Limnophyes habilis|BIOUG15953-F07|Kate Perez|BIN Taxonomy Match|BOLD:AAJ2130
Limnophyes habilis|BIOUG42964-E04|Kate Perez|BIN Taxonomy Match below Phylum (Jun 2019)|BOL...
Limnophyes habilis|BIOUG16782-D02|Kate Perez|BIN Taxonomy Match|BOLD:AAJ2130
Limnophyes habilis|BIOUG42933-D04|Kate Perez|BIN Taxonomy Match below Phylum (Jun 2019)|BOL...
Limnophyes habilis|BIOUG16782-F05|Kate Perez|BIN Taxonomy Match|BOLD:AAJ2130
Limnophyes habilis|BIOUG16777-G07|Kate Perez|BIN Taxonomy Match|BOLD:AAJ2130
Limnophyes habilis|BIOUG17023-D01|Kate Perez|BIN Taxonomy Match|BOLD:AAJ2130
Limnophyes habilis|ZSM-DIP-33130-E07|Caroline Chimeno|BIN Taxonomy Match (Jan 2022)|BOLD ...
Limnophyes habilis|BIOUG16853-H03|Kate Perez|BIN Taxonomy Match|BOLD:AAJ2130
Limnophyes habilis|BIOUG16893-H03|Kate Perez|BIN Taxonomy Match|BOLD:AAJ2130
Limnophyes habilis|BIOUG16778-D03|Kate Perez|BIN Taxonomy Match|BOLD:AAJ2130
Limnophyes habilis|BIOUG16738-A04|Kate Perez|BIN Taxonomy Match|BOLD:AAJ2130
Limnophyes habilis|BIOUG16785-A03|Kate Perez|BIN Taxonomy Match|BOLD:AAJ2130
Limnophyes habilis|BIOUG16738-C06|Kate Perez|BIN Taxonomy Match|BOLD:AAJ2130
Limnophyes habilis|BIOUG16777-A08|Kate Perez|BIN Taxonomy Match|BOLD:AAJ2130
Limnophyes habilis|BIOUG17023-C11|Kate Perez|BIN Taxonomy Match|BOLD:AAJ2130
Limnophyes habilis|BIOUG16776-B03|Kate Perez|BIN Taxonomy Match|BOLD:AAJ2130
Limnophyes habilis|BIOUG16776-E05|Kate Perez|BIN Taxonomy Match|BOLD:AAJ2130
Limnophyes habilis|BIOUG16779-C04|Kate Perez|BIN Taxonomy Match|BOLD:AAJ2130
Limnophyes habilis|BIOUG16782-E12|Kate Perez|BIN Taxonomy Match|BOLD:AAJ2130
Limnophyes habilis|BIOUG04153-C05|Kate Perez|BIN Taxonomy Match|BOLD:AAJ2130
Limnophyes habilis|BIOUG15795-D07|Kate Perez|BIN Taxonomy Match|BOLD:AAJ2130
Limnophyes habilis|BIOUG16785-D07|Kate Perez|BIN Taxonomy Match|BOLD:AAJ2130
Limnophyes habilis|BIOUG16778-C11|Kate Perez|BIN Taxonomy Match|BOLD:AAJ2130
Limnophyes habilis|BIOUG16721-G12|Kate Perez|BIN Taxonomy Match|BOLD:AAJ2130
Limnophyes habilis|BIOUG16739-A02|Kate Perez|BIN Taxonomy Match|BOLD:AAJ2130
Limnophyes habilis|BIOUG16779-A11|Kate Perez|BIN Taxonomy Match|BOLD:AAJ2130
Limnophyes habilis|CH-OSF50|Elisabeth Stur||BOLD:AAJ2130
Limnophyes habilis|BIOUG16782-G11|Kate Perez|BIN Taxonomy Match|BOLD:AAJ2130
Limnophyes habilis|BIOUG15792-F03|Kate Perez|BIN Taxonomy Match|BOLD:AAJ2130
Limnophyes habilis|BIOUG16783-B06|Kate Perez|BIN Taxonomy Match|BOLD:AAJ2130
Limnophyes habilis|BIOUG16329-A02|Kate Perez|BIN Taxonomy Match|BOLD:AAJ2130
Limnophyes habilis|BIOUG16855-F12|Kate Perez|BIN Taxonomy Match|BOLD:AAJ2130
Limnophyes habilis|BIOUG16775-A02|Kate Perez|BIN Taxonomy Match|BOLD:AAJ2130
Limnophyes habilis|BIOUG16783-B01|Kate Perez|BIN Taxonomy Match|BOLD:AAJ2130
Limnophyes habilis|ZSM-DIP-33132-F09|Caroline Chimeno|BIN Taxonomy Match (Jan 2022)|BOLD ...
Limnophyes habilis|BIOUG15720-E04|Kate Perez|BIN Taxonomy Match|BOLD:AAJ2130
Limnophyes habilis|BIOUG16777-E08|Kate Perez|BIN Taxonomy Match|BOLD:AAJ2130
Limnophyes habilis|BIOUG16785-A02|Kate Perez|BIN Taxonomy Match|BOLD:AAJ2130
Limnophyes habilis|BIOUG16784-F10|Kate Perez|BIN Taxonomy Match|BOLD:AAJ2130
Limnophyes habilis|BIOUG16893-E11|Kate Perez|BIN Taxonomy Match|BOLD:AAJ2130
Limnophyes habilis|BIOUG42658-D06|Kate Perez|BIN Taxonomy Match below Phylum (Jun 2019)|BOL...
Limnophyes habilis|BIOUG16739-E12|Kate Perez|BIN Taxonomy Match|BOLD:AAJ2130
Limnophyes habilis|BIOUG16775-D06|Kate Perez|BIN Taxonomy Match|BOLD:AAJ2130
Limnophyes habilis|BIOUG16782-H11|Kate Perez|BIN Taxonomy Match|BOLD:AAJ2130
Limnophyes habilis|BIOUG43079-D12|Kate Perez|BIN Taxonomy Match below Phylum (Jun 2019)|BOL...
Limnophyes habilis|BIOUG16783-F08|Kate Perez|BIN Taxonomy Match|BOLD:AAJ2130
Limnophyes habilis|BIOUG42753-C04|Kate Perez|BIN Taxonomy Match below Phylum (Jun 2019)|BOL...
Limnophyes habilis|BIOUG17112-C05|Kate Perez|BIN Taxonomy Match|BOLD:AAJ2130
Limnophyes habilis|BIOUG16783-H06|Kate Perez|BIN Taxonomy Match|BOLD:AAJ2130
Limnophyes habilis|BIOUG16779-C10|Kate Perez|BIN Taxonomy Match|BOLD:AAJ2130
Limnophyes habilis|ZSM-DIP-33131-B12|Caroline Chimeno|BIN Taxonomy Match (Jan 2022)|BOLD ...
Limnophyes habilis|BIOUG16775-E10|Kate Perez|BIN Taxonomy Match|BOLD:AAJ2130
Limnophyes habilis|BIOUG16740-A06|Kate Perez|BIN Taxonomy Match|BOLD:AAJ2130
Limnophyes habilis|BIOUG42714-B02|Kate Perez|BIN Taxonomy Match below Phylum (Jun 2019)|BOL...
Limnophyes habilis|BIOUG42918-B02|Kate Perez|BIN Taxonomy Match below Phylum (Jun 2019)|BOL...
Limnophyes habilis|BIOUG06096-A01|Kate Perez|BIN Taxonomy Match|BOLD:AAJ2130
Limnophyes habilis|BIOUG16783-E06|Kate Perez|BIN Taxonomy Match|BOLD:AAJ2130
Limnophyes habilis|BIOUG42703-E06|Kate Perez|BIN Taxonomy Match below Phylum (Jun 2019)|BOL...
Limnophyes habilis|BIOUG16775-B08|Kate Perez|BIN Taxonomy Match|BOLD:AAJ2130
Limnophyes habilis|BIOUG16785-B02|Kate Perez|BIN Taxonomy Match|BOLD:AAJ2130
Limnophyes habilis|BIOUG16779-G11|Kate Perez|BIN Taxonomy Match|BOLD:AAJ2130
Limnophyes habilis|BIOUG16782-E08|Kate Perez|BIN Taxonomy Match|BOLD:AAJ2130
Limnophyes habilis|BIOUG16782-B01|Kate Perez|BIN Taxonomy Match|BOLD:AAJ2130
Limnophyes habilis|BIOUG42759-D02|Kate Perez|BIN Taxonomy Match below Phylum (Jun 2019)|BOL...
Limnophyes habilis|BIOUG42718-C02|Kate Perez|BIN Taxonomy Match below Phylum (Jun 2019)|BOL...

Limnophyes habilis|BIOUG16782-B01|Kate Perez|BIN Taxonomy Match|BOLD:AAJ2130
Limnophyes habilis|BIOUG42759-D02|Kate Perez|BIN Taxonomy Match below Phylum (Jun 2019)|BOL...
Limnophyes habilis|BIOUG42718-C02|Kate Perez|BIN Taxonomy Match below Phylum (Jun 2019)|BOL...
Limnophyes habilis|BIOUG42708-F07|Kate Perez|BIN Taxonomy Match below Phylum (Jun 2019)|BOL...
Limnophyes habilis|BIOUG16783-C06|Kate Perez|BIN Taxonomy Match|BOLD:AAJ2130
Limnophyes habilis|BIOUG16776-F01|Kate Perez|BIN Taxonomy Match|BOLD:AAJ2130
Limnophyes habilis|BIOUG16431-E10|Kate Perez|BIN Taxonomy Match|BOLD:AAJ2130
Limnophyes habilis|BIOUG16703-E10|Kate Perez|BIN Taxonomy Match|BOLD:AAJ2130
Limnophyes habilis|BIOUG07949-A02|Kate Perez|BIN Taxonomy Match|BOLD:AAJ2130
Limnophyes habilis|BIOUG16776-H09|Kate Perez|BIN Taxonomy Match|BOLD:AAJ2130
Limnophyes habilis|BIOUG16775-B12|Kate Perez|BIN Taxonomy Match|BOLD:AAJ2130
Limnophyes habilis|BIOUG16738-B08|Kate Perez|BIN Taxonomy Match|BOLD:AAJ2130
Limnophyes habilis|BIOUG16720-A08|Kate Perez|BIN Taxonomy Match|BOLD:AAJ2130
Limnophyes habilis|BIOUG16721-D04|Kate Perez|BIN Taxonomy Match|BOLD:AAJ2130
Limnophyes habilis|BIOUG16721-G06|Kate Perez|BIN Taxonomy Match|BOLD:AAJ2130
Limnophyes habilis|BIOUG16775-B09|Kate Perez|BIN Taxonomy Match|BOLD:AAJ2130
Limnophyes habilis|BIOUG42675-C08|Kate Perez|BIN Taxonomy Match below Phylum (Jun 2019)|BOL...
Limnophyes habilis|BIOUG16776-E03|Kate Perez|BIN Taxonomy Match|BOLD:AAJ2130
Limnophyes habilis|BIOUG15766-H04|Kate Perez|BIN Taxonomy Match|BOLD:AAJ2130
Limnophyes habilis|BIOUG16713-G08|Kate Perez|BIN Taxonomy Match|BOLD:AAJ2130
Limnophyes habilis|BIOUG16625-F06|Kate Perez|BIN Taxonomy Match|BOLD:AAJ2130
Limnophyes habilis|BIOUG16298-D03|Kate Perez|BIN Taxonomy Match|BOLD:AAJ2130
Limnophyes habilis|BIOUG16626-H11|Kate Perez|BIN Taxonomy Match|BOLD:AAJ2130
Limnophyes habilis|BIOUG17064-H08|Kate Perez|BIN Taxonomy Match|BOLD:AAJ2130
Limnophyes habilis|BIOUG16436-E11|Kate Perez|BIN Taxonomy Match|BOLD:AAJ2130
Limnophyes habilis|BIOUG16333-E07|Kate Perez|BIN Taxonomy Match|BOLD:AAJ2130
Limnophyes habilis|BIOUG15954-C09|Kate Perez|BIN Taxonomy Match|BOLD:AAJ2130
Limnophyes habilis|BIOUG16785-B03|Kate Perez|BIN Taxonomy Match|BOLD:AAJ2130
Limnophyes habilis|BIOUG16775-E01|Kate Perez|BIN Taxonomy Match|BOLD:AAJ2130
Limnophyes habilis|ATNA414|Elisabeth Stur||BOLD:AAJ2130
Limnophyes habilis|BIOUG16776-A01|Kate Perez|BIN Taxonomy Match|BOLD:AAJ2130
Limnophyes habilis|BIOUG16776-G05|Kate Perez|BIN Taxonomy Match|BOLD:AAJ2130
Limnophyes habilis|BIOUG16779-A12|Kate Perez|BIN Taxonomy Match|BOLD:AAJ2130
Limnophyes habilis|BIOUG42763-G03|Kate Perez|BIN Taxonomy Match below Phylum (Jun 2019)|BOL...
Limnophyes habilis|BIOUG16777-G06|Kate Perez|BIN Taxonomy Match|BOLD:AAJ2130
Limnophyes habilis|ZSM-DIP-33132-H05|Caroline Chimeno|BIN Taxonomy Match (Jan 2022)|BOLD ...
Limnophyes habilis|BIOUG17114-H11|Kate Perez|BIN Taxonomy Match|BOLD:AAJ2130
Limnophyes habilis|BIOUG16776-H10|Kate Perez|BIN Taxonomy Match|BOLD:AAJ2130
Limnophyes habilis|BIOUG16784-B06|Kate Perez|BIN Taxonomy Match|BOLD:AAJ2130
Limnophyes habilis|BIOUG16333-F03|Kate Perez|BIN Taxonomy Match|BOLD:AAJ2130
Limnophyes habilis|BIOUG16738-G07|Kate Perez|BIN Taxonomy Match|BOLD:AAJ2130
Limnophyes habilis|BIOUG16782-G07|Kate Perez|BIN Taxonomy Match|BOLD:AAJ2130
Limnophyes habilis|BIOUG16776-E12|Kate Perez|BIN Taxonomy Match|BOLD:AAJ2130
Limnophyes habilis|BIOUG16782-G06|Kate Perez|BIN Taxonomy Match|BOLD:AAJ2130
Limnophyes habilis|BIOUG16739-B12|Kate Perez|BIN Taxonomy Match|BOLD:AAJ2130
Limnophyes habilis|BIOUG16892-B12|Kate Perez|BIN Taxonomy Match|BOLD:AAJ2130
Limnophyes habilis|BIOUG16784-H01|Kate Perez|BIN Taxonomy Match|BOLD:AAJ2130
Limnophyes habilis|BIOUG16784-E11|Kate Perez|BIN Taxonomy Match|BOLD:AAJ2130
Limnophyes habilis|BIOUG16783-A06|Kate Perez|BIN Taxonomy Match|BOLD:AAJ2130
Limnophyes habilis|BIOUG55111-A06|Stefan Schmidt|BIN Taxonomy Match (Sep 2020)|BOLD:AAJ2130
Limnophyes habilis|BIOUG16783-E11|Kate Perez|BIN Taxonomy Match|BOLD:AAJ2130
Limnophyes habilis|BIOUG16785-D03|Kate Perez|BIN Taxonomy Match|BOLD:AAJ2130
Limnophyes habilis|BIOUG16784-C09|Kate Perez|BIN Taxonomy Match|BOLD:AAJ2130
Limnophyes habilis|BIOUG15793-E01|Kate Perez|BIN Taxonomy Match|BOLD:AAJ2130
Limnophyes habilis|BIOUG16784-C03|Kate Perez|BIN Taxonomy Match|BOLD:AAJ2130
Limnophyes habilis|BIOUG16713-A05|Kate Perez|BIN Taxonomy Match|BOLD:AAJ2130
Limnophyes habilis|BIOUG16739-D11|Kate Perez|BIN Taxonomy Match|BOLD:AAJ2130
Limnophyes habilis|BIOUG42926-E07|Kate Perez|BIN Taxonomy Match below Phylum (Jun 2019)|BOL...
Limnophyes habilis|TRD-CH64|Elisabeth Stur||BOLD:AAJ2130
Limnophyes habilis|BIOUG16777-H04|Kate Perez|BIN Taxonomy Match|BOLD:AAJ2130
Limnophyes habilis|BIOUG16739-H03|Kate Perez|BIN Taxonomy Match|BOLD:AAJ2130
Limnophyes habilis|BIOUG16777-D08|Kate Perez|BIN Taxonomy Match|BOLD:AAJ2130
Limnophyes habilis|BIOUG16740-D09|Kate Perez|BIN Taxonomy Match|BOLD:AAJ2130
Limnophyes habilis|BIOUG16721-F05|Kate Perez|BIN Taxonomy Match|BOLD:AAJ2130
Limnophyes habilis|BIOUG16775-C10|Kate Perez|BIN Taxonomy Match|BOLD:AAJ2130
Limnophyes habilis|BIOUG16739-F07|Kate Perez|BIN Taxonomy Match|BOLD:AAJ2130
Limnophyes habilis|BIOUG16782-D08|Kate Perez|BIN Taxonomy Match|BOLD:AAJ2130
Limnophyes habilis|BIOUG16720-E12|Kate Perez|BIN Taxonomy Match|BOLD:AAJ2130
Limnophyes habilis|BIOUG16784-E02|Kate Perez|BIN Taxonomy Match|BOLD:AAJ2130
Limnophyes habilis|BIOUG16739-D08|Kate Perez|BIN Taxonomy Match|BOLD:AAJ2130
Limnophyes habilis|BIOUG16739-B06|Kate Perez|BIN Taxonomy Match|BOLD:AAJ2130
Limnophyes habilis|BIOUG15855-G06|Kate Perez|BIN Taxonomy Match|BOLD:AAJ2130
Limnophyes habilis|BIOUG16778-B12|Kate Perez|BIN Taxonomy Match|BOLD:AAJ2130
Limnophyes habilis|BIOUG16776-C03|Kate Perez|BIN Taxonomy Match|BOLD:AAJ2130
Limnophyes habilis|BIOUG16739-C04|Kate Perez|BIN Taxonomy Match|BOLD:AAJ2130
Limnophyes habilis|BIOUG16782-A08|Kate Perez|BIN Taxonomy Match|BOLD:AAJ2130
Limnophyes habilis|BIOUG16894-B09|Kate Perez|BIN Taxonomy Match|BOLD:AAJ2130
Limnophyes habilis|BIOUG16738-A03|Kate Perez|BIN Taxonomy Match|BOLD:AAJ2130
Limnophyes habilis|BIOUG16779-D08|Kate Perez|BIN Taxonomy Match|BOLD:AAJ2130
Limnophyes habilis|BIOUG16739-H01|Kate Perez|BIN Taxonomy Match|BOLD:AAJ2130
Limnophyes habilis|BIOUG16857-C04|Kate Perez|BIN Taxonomy Match|BOLD:AAJ2130
Limnophyes habilis|BIOUG16739-D05|Kate Perez|BIN Taxonomy Match|BOLD:AAJ2130
Limnophyes habilis|BIOUG16784-A03|Kate Perez|BIN Taxonomy Match|BOLD:AAJ2130
Limnophyes habilis|BIOUG16783-C03|Kate Perez|BIN Taxonomy Match|BOLD:AAJ2130
Limnophyes habilis|BIOUG16778-B08|Kate Perez|BIN Taxonomy Match|BOLD:AAJ2130
Limnophyes habilis|BIOUG16778-C12|Kate Perez|BIN Taxonomy Match|BOLD:AAJ2130
Limnophyes habilis|BIOUG16298-B10|Kate Perez|BIN Taxonomy Match|BOLD:AAJ2130
Limnophyes habilis|BIOUG15799-F08|Kate Perez|BIN Taxonomy Match|BOLD:AAJ2130
Limnophyes habilis|BIOUG16775-G02|Kate Perez|BIN Taxonomy Match|BOLD:AAJ2130
Limnophyes habilis|BIOUG16721-D01|Kate Perez|BIN Taxonomy Match|BOLD:AAJ2130
Limnophyes habilis|BIOUG16785-E04|Kate Perez|BIN Taxonomy Match|BOLD:AAJ2130
Limnophyes habilis|BIOUG16783-G09|Kate Perez|BIN Taxonomy Match|BOLD:AAJ2130
Limnophyes habilis|BIOUG16783-E03|Kate Perez|BIN Taxonomy Match|BOLD:AAJ2130
Limnophyes habilis|BIOUG16854-G02|Kate Perez|BIN Taxonomy Match|BOLD:AAJ2130
Limnophyes habilis|BIOUG42658-F10|Kate Perez|BIN Taxonomy Match below Phylum (Jun 2019)|BOL...
Limnophyes habilis|ZSM-DIP-33132-F10|Caroline Chimeno|BIN Taxonomy Match (Jan 2022)|BOLD ...
Limnophyes habilis|BIOUG16778-E12|Kate Perez|BIN Taxonomy Match|BOLD:AAJ2130
Limnophyes habilis|BIOUG16785-F10|Kate Perez|BIN Taxonomy Match|BOLD:AAJ2130
Limnophyes habilis|BIOUG16855-C02|Kate Perez|BIN Taxonomy Match|BOLD:AAJ2130
Limnophyes habilis|BIOUG16720-D07|Kate Perez|BIN Taxonomy Match|BOLD:AAJ2130
Limnophyes habilis|BIOUG16778-D01|Kate Perez|BIN Taxonomy Match|BOLD:AAJ2130
Limnophyes habilis|BIOUG16854-G08|Kate Perez|BIN Taxonomy Match|BOLD:AAJ2130
Limnophyes habilis|BIOUG16783-F01|Kate Perez|BIN Taxonomy Match|BOLD:AAJ2130

[illegible]
Limnophyes habilis|BIOUG16778-D01|Kate Perez|BIN Taxonomy Match|BOLD:AAJ2130
Limnophyes habilis|BIOUG16854-G08|Kate Perez|BIN Taxonomy Match|BOLD:AAJ2130
Limnophyes habilis|BIOUG16783-F01|Kate Perez|BIN Taxonomy Match|BOLD:AAJ2130
Limnophyes habilis|BIOUG16779-C06|Kate Perez|BIN Taxonomy Match|BOLD:AAJ2130
Limnophyes habilis|BIOUG16333-H06|Kate Perez|BIN Taxonomy Match|BOLD:AAJ2130
Limnophyes habilis|BIOUG15853-D01|Kate Perez|BIN Taxonomy Match|BOLD:AAJ2130
Limnophyes habilis|BIOUG15952-A05|Kate Perez|BIN Taxonomy Match|BOLD:AAJ2130
Limnophyes habilis|BIOUG16777-G08|Kate Perez|BIN Taxonomy Match|BOLD:AAJ2130
Limnophyes habilis|BIOUG16478-E11|Kate Perez|BIN Taxonomy Match|BOLD:AAJ2130
Limnophyes habilis|BIOUG16299-E02|Kate Perez|BIN Taxonomy Match|BOLD:AAJ2130
Limnophyes habilis|BIOUG16479-E06|Kate Perez|BIN Taxonomy Match|BOLD:AAJ2130
Limnophyes habilis|BIOUG15793-H08|Kate Perez|BIN Taxonomy Match|BOLD:AAJ2130
Limnophyes habilis|BIOUG16435-A08|Kate Perez|BIN Taxonomy Match|BOLD:AAJ2130
Limnophyes habilis|BIOUG17022-A04|Kate Perez|BIN Taxonomy Match|BOLD:AAJ2130
Limnophyes habilis|BIOUG16625-H02|Kate Perez|BIN Taxonomy Match|BOLD:AAJ2130
Limnophyes habilis|BIOUG16552-D03|Kate Perez|BIN Taxonomy Match|BOLD:AAJ2130
Limnophyes habilis|BIOUG15950-E06|Kate Perez|BIN Taxonomy Match|BOLD:AAJ2130
Limnophyes habilis|BIOUG15950-E05|Kate Perez|BIN Taxonomy Match|BOLD:AAJ2130
Limnophyes habilis|BIOUG16721-C06|Kate Perez|BIN Taxonomy Match|BOLD:AAJ2130
Limnophyes habilis|BIOUG16785-E09|Kate Perez|BIN Taxonomy Match|BOLD:AAJ2130
Limnophyes habilis|BIOUG42923-D01|Kate Perez|BIN Taxonomy Match below Phylum (Jun 2019)|BOL...
Limnophyes habilis|BIOUG04153-A12|Kate Perez|BIN Taxonomy Match|BOLD:AAJ2130
Limnophyes habilis|BIOUG16782-B04|Kate Perez|BIN Taxonomy Match|BOLD:AAJ2130
Limnophyes habilis|BIOUG16782-F12|Kate Perez|BIN Taxonomy Match|BOLD:AAJ2130
Limnophyes habilis|BIOUG16739-C12|Kate Perez|BIN Taxonomy Match|BOLD:AAJ2130
Limnophyes habilis|BIOUG16784-G06|Kate Perez|BIN Taxonomy Match|BOLD:AAJ2130
Limnophyes habilis|BIOUG16893-E01|Kate Perez|BIN Taxonomy Match|BOLD:AAJ2130
Limnophyes habilis|BIOUG16997-B04|Kate Perez|BIN Taxonomy Match|BOLD:AAJ2130
Limnophyes habilis|BIOUG16777-D01|Kate Perez|BIN Taxonomy Match|BOLD:AAJ2130
Limnophyes habilis|BIOUG16776-G04|Kate Perez|BIN Taxonomy Match|BOLD:AAJ2130
Limnophyes habilis|BIOUG16740-F11|Kate Perez|BIN Taxonomy Match|BOLD:AAJ2130
Limnophyes habilis|BIOUG16782-B08|Kate Perez|BIN Taxonomy Match|BOLD:AAJ2130
Limnophyes habilis|BIOUG16775-E07|Kate Perez|BIN Taxonomy Match|BOLD:AAJ2130
Limnophyes habilis|BIOUG16776-C11|Kate Perez|BIN Taxonomy Match|BOLD:AAJ2130
Limnophyes habilis|BIOUG16892-F04|Kate Perez|BIN Taxonomy Match|BOLD:AAJ2130
Limnophyes habilis|BIOUG16777-E03|Kate Perez|BIN Taxonomy Match|BOLD:AAJ2130
Limnophyes habilis|BIOUG16894-A01|Kate Perez|BIN Taxonomy Match|BOLD:AAJ2130
Limnophyes habilis|BIOUG16738-F09|Kate Perez|BIN Taxonomy Match|BOLD:AAJ2130
Limnophyes habilis|BIOUG16721-F04|Kate Perez|BIN Taxonomy Match|BOLD:AAJ2130
Limnophyes habilis|BIOUG16857-A01|Kate Perez|BIN Taxonomy Match|BOLD:AAJ2130
Limnophyes habilis|BIOUG16740-H10|Kate Perez|BIN Taxonomy Match|BOLD:AAJ2130
Limnophyes habilis|BIOUG16785-D11|Kate Perez|BIN Taxonomy Match|BOLD:AAJ2130
Limnophyes habilis|BIOUG16782-D06|Kate Perez|BIN Taxonomy Match|BOLD:AAJ2130
Limnophyes habilis|BIOUG16721-G07|Kate Perez|BIN Taxonomy Match|BOLD:AAJ2130
Limnophyes habilis|BIOUG16776-D02|Kate Perez|BIN Taxonomy Match|BOLD:AAJ2130
Limnophyes habilis|BIOUG42654-G05|Kate Perez|BIN Taxonomy Match below Phylum (Jun 2019)|BOL...
Limnophyes habilis|BIOUG42744-G06|Kate Perez|BIN Taxonomy Match below Phylum (Jun 2019)|BOL...
Limnophyes habilis|BIOUG42658-H01|Kate Perez|BIN Taxonomy Match below Phylum (Jun 2019)|BOL...
Limnophyes habilis|BIOUG42707-C01|Kate Perez|BIN Taxonomy Match below Phylum (Jun 2019)|BOL...
Limnophyes habilis|BIOUG42933-D10|Kate Perez|BIN Taxonomy Match below Phylum (Jun 2019)|BOL...
Limnophyes habilis|BIOUG16782-A02|Kate Perez|BIN Taxonomy Match|BOLD:AAJ2130
Limnophyes habilis|BIOUG16782-F04|Kate Perez|BIN Taxonomy Match|BOLD:AAJ2130
Limnophyes habilis|BIOUG16785-D05|Kate Perez|BIN Taxonomy Match|BOLD:AAJ2130
Limnophyes habilis|BIOUG16775-A11|Kate Perez|BIN Taxonomy Match|BOLD:AAJ2130
Limnophyes habilis|BIOUG16784-F06|Kate Perez|BIN Taxonomy Match|BOLD:AAJ2130
Limnophyes habilis|BIOUG16776-F07|Kate Perez|BIN Taxonomy Match|BOLD:AAJ2130
Limnophyes habilis|BIOUG16856-E06|Kate Perez|BIN Taxonomy Match|BOLD:AAJ2130
Limnophyes habilis|BIOUG16738-B09|Kate Perez|BIN Taxonomy Match|BOLD:AAJ2130
Limnophyes habilis|BIOUG43078-F01|Kate Perez|BIN Taxonomy Match below Phylum (Jun 2019)|BOL...
Limnophyes habilis|BIOUG16738-F06|Kate Perez|BIN Taxonomy Match|BOLD:AAJ2130
Limnophyes habilis|BIOUG16855-E05|Kate Perez|BIN Taxonomy Match|BOLD:AAJ2130
Limnophyes habilis|BIOUG16855-B04|Kate Perez|BIN Taxonomy Match|BOLD:AAJ2130
Limnophyes habilis|BIOUG16597-E04|Kate Perez|BIN Taxonomy Match|BOLD:AAJ2130
Limnophyes habilis|BIOUG16859-H03|Kate Perez|BIN Taxonomy Match|BOLD:AAJ2130
Limnophyes habilis|BIOUG16893-G07|Kate Perez|BIN Taxonomy Match|BOLD:AAJ2130
Limnophyes habilis|BIOUG16777-H10|Kate Perez|BIN Taxonomy Match|BOLD:AAJ2130
Limnophyes habilis|BIOUG16776-F12|Kate Perez|BIN Taxonomy Match|BOLD:AAJ2130
Limnophyes habilis|BIOUG16720-A12|Kate Perez|BIN Taxonomy Match|BOLD:AAJ2130
Limnophyes habilis|BIOUG16892-D01|Kate Perez|BIN Taxonomy Match|BOLD:AAJ2130
Limnophyes habilis|BIOUG16785-E06|Kate Perez|BIN Taxonomy Match|BOLD:AAJ2130
Limnophyes habilis|BIOUG16784-H10|Kate Perez|BIN Taxonomy Match|BOLD:AAJ2130
Limnophyes habilis|BIOUG15719-B08|Kate Perez|BIN Taxonomy Match|BOLD:AAJ2130
Limnophyes habilis|BIOUG16778-D02|Kate Perez|BIN Taxonomy Match|BOLD:AAJ2130
Limnophyes habilis|ZSM-DIP-33133-F06|Caroline Chimeno|BIN Taxonomy Match (Jan 2022)|BOLD ...
Limnophyes habilis|BIOUG16626-C12|Kate Perez|BIN Taxonomy Match|BOLD:AAJ2130
Limnophyes habilis|BIOUG15807-C03|Kate Perez|BIN Taxonomy Match|BOLD:AAJ2130
Limnophyes habilis|BIOUG16740-C01|Kate Perez|BIN Taxonomy Match|BOLD:AAJ2130
Limnophyes habilis|BIOUG16627-B02|Kate Perez|BIN Taxonomy Match|BOLD:AAJ2130
Limnophyes habilis|BIOUG16782-G03|Kate Perez|BIN Taxonomy Match|BOLD:AAJ2130
Limnophyes habilis|BIOUG16775-A04|Kate Perez|BIN Taxonomy Match|BOLD:AAJ2130
Limnophyes habilis|BIOUG36653-G07|Kate Perez|BIN Taxonomy Match (Jul 2018)|BOLD:AAJ2130
Limnophyes habilis|BIOUG16783-H11|Kate Perez|BIN Taxonomy Match|BOLD:AAJ2130
Limnophyes habilis|BIOUG16784-F12|Kate Perez|BIN Taxonomy Match|BOLD:AAJ2130
Limnophyes habilis|BIOUG42746-B12|Kate Perez|BIN Taxonomy Match below Phylum (Jun 2019)|BOL...
Limnophyes habilis|BIOUG16784-B02|Kate Perez|BIN Taxonomy Match|BOLD:AAJ2130
Limnophyes habilis|ZSM-DIP-33131-C03|Caroline Chimeno|BIN Taxonomy Match (Jan 2022)|BOLD ...
Limnophyes habilis|BIOUG16777-F07|Kate Perez|BIN Taxonomy Match|BOLD:AAJ2130
Limnophyes habilis|BIOUG16740-A08|Kate Perez|BIN Taxonomy Match|BOLD:AAJ2130
Limnophyes habilis|BIOUG16779-F09|Kate Perez|BIN Taxonomy Match|BOLD:AAJ2130
Limnophyes habilis|BIOUG16738-A09|Kate Perez|BIN Taxonomy Match|BOLD:AAJ2130
Limnophyes habilis|BIOUG16777-A02|Kate Perez|BIN Taxonomy Match|BOLD:AAJ2130
Limnophyes habilis|BIOUG42672-A01|Kate Perez|BIN Taxonomy Match below Phylum (Jun 2019)|BOL...
Limnophyes habilis|BIOUG16779-B01|Kate Perez|BIN Taxonomy Match|BOLD:AAJ2130
Limnophyes habilis|BIOUG16784-G04|Kate Perez|BIN Taxonomy Match|BOLD:AAJ2130
Limnophyes habilis|BIOUG16776-H07|Kate Perez|BIN Taxonomy Match|BOLD:AAJ2130
Limnophyes habilis|BIOUG42670-H01|Kate Perez|BIN Taxonomy Match below Phylum (Jun 2019)|BOL...
Limnophyes habilis|BIOUG16785-F12|Kate Perez|BIN Taxonomy Match|BOLD:AAJ2130
Limnophyes habilis|BIOUG16721-D06|Kate Perez|BIN Taxonomy Match|BOLD:AAJ2130
Limnophyes habilis|BIOUG16854-F01|Kate Perez|BIN Taxonomy Match|BOLD:AAJ2130
Limnophyes habilis|BIOUG16994-A03|Kate Perez|BIN Taxonomy Match|BOLD:AAJ2130
Limnophyes habilis|BIOUG16738-B05|Kate Perez|BIN Taxonomy Match|BOLD:AAJ2130
Limnophyes habilis|BIOUG16782-A06|Kate Perez|BIN Taxonomy Match|BOLD:AAJ2130
[illegible]

Limnophyes habilis|BIOUG16994-A03|Kate Perez|BIN Taxonomy Match|BOLD:AAJ2130
Limnophyes habilis|BIOUG16738-B05|Kate Perez|BIN Taxonomy Match|BOLD:AAJ2130
Limnophyes habilis|BIOUG16782-A06|Kate Perez|BIN Taxonomy Match|BOLD:AAJ2130
Limnophyes habilis|BIOUG16893-C02|Kate Perez|BIN Taxonomy Match|BOLD:AAJ2130
Limnophyes habilis|BIOUG16783-H05|Kate Perez|BIN Taxonomy Match|BOLD:AAJ2130
Limnophyes habilis|BIOUG16782-G05|Kate Perez|BIN Taxonomy Match|BOLD:AAJ2130
Limnophyes habilis|BIOUG16859-G06|Kate Perez|BIN Taxonomy Match|BOLD:AAJ2130
Limnophyes habilis|BIOUG16738-G09|Kate Perez|BIN Taxonomy Match|BOLD:AAJ2130
Limnophyes habilis|BIOUG16740-G06|Kate Perez|BIN Taxonomy Match|BOLD:AAJ2130
Limnophyes habilis|BIOUG16777-B03|Kate Perez|BIN Taxonomy Match|BOLD:AAJ2130
Limnophyes habilis|BIOUG16776-C10|Kate Perez|BIN Taxonomy Match|BOLD:AAJ2130
Limnophyes habilis|BIOUG16892-E02|Kate Perez|BIN Taxonomy Match|BOLD:AAJ2130
Limnophyes habilis|BIOUG16778-A03|Kate Perez|BIN Taxonomy Match|BOLD:AAJ2130
Limnophyes habilis|BIOUG16854-A01|Kate Perez|BIN Taxonomy Match|BOLD:AAJ2130
Limnophyes habilis|BIOUG16740-C03|Kate Perez|BIN Taxonomy Match|BOLD:AAJ2130
Limnophyes habilis|BIOUG16859-C03|Kate Perez|BIN Taxonomy Match|BOLD:AAJ2130
Limnophyes habilis|BIOUG16775-A03|Kate Perez|BIN Taxonomy Match|BOLD:AAJ2130
Limnophyes habilis|BIOUG16738-C09|Kate Perez|BIN Taxonomy Match|BOLD:AAJ2130
Limnophyes habilis|BIOUG16720-A03|Kate Perez|BIN Taxonomy Match|BOLD:AAJ2130
Limnophyes habilis|BC-ZSM-DIP-22491-D03|Dieter Doczkal||BOLD:AAJ2130
Limnophyes habilis|BIOUG06088-C10|Kate Perez|BIN Taxonomy Match|BOLD:AAJ2130
Limnophyes habilis|BIOUG06086-F07|Kate Perez|BIN Taxonomy Match|BOLD:AAJ2130
Limnophyes habilis|BIOUG16594-H05|Kate Perez|BIN Taxonomy Match|BOLD:AAJ2130
Limnophyes habilis|BIOUG16434-C05|Kate Perez|BIN Taxonomy Match|BOLD:AAJ2130
Limnophyes habilis|BIOUG15966-D03|Kate Perez|BIN Taxonomy Match|BOLD:AAJ2130
Limnophyes habilis|BIOUG16782-D10|Kate Perez|BIN Taxonomy Match|BOLD:AAJ2130
Limnophyes habilis|BIOUG16738-H01|Kate Perez|BIN Taxonomy Match|BOLD:AAJ2130
Limnophyes habilis|BIOUG16632-A07|Kate Perez|BIN Taxonomy Match|BOLD:AAJ2130
Limnophyes habilis|BIOUG16476-B09|Kate Perez|BIN Taxonomy Match|BOLD:AAJ2130
Limnophyes habilis|BIOUG16711-B06|Kate Perez|BIN Taxonomy Match|BOLD:AAJ2130
Limnophyes habilis|BIOUG16713-A07|Kate Perez|BIN Taxonomy Match|BOLD:AAJ2130
Limnophyes habilis|BIOUG16332-H10|Kate Perez|BIN Taxonomy Match|BOLD:AAJ2130
Limnophyes habilis|BIOUG04119-B09|Kate Perez|BIN Taxonomy Match|BOLD:AAJ2130
Limnophyes habilis|BIOUG15807-H10|Kate Perez|BIN Taxonomy Match|BOLD:AAJ2130
Limnophyes habilis|BIOUG15967-C10|Kate Perez|BIN Taxonomy Match|BOLD:AAJ2130
Limnophyes habilis|BIOUG16893-A07|Kate Perez|BIN Taxonomy Match|BOLD:AAJ2130
Limnophyes habilis|BIOUG42665-A01|Kate Perez|BIN Taxonomy Match below Phylum (Jun 2019)|BOL...
Limnophyes habilis|BIOUG16594-F12|Kate Perez|BIN Taxonomy Match|BOLD:AAJ2130
Limnophyes habilis|BIOUG15764-D11|Kate Perez|BIN Taxonomy Match|BOLD:AAJ2130
Limnophyes habilis|BIOUG15951-C11|Kate Perez|BIN Taxonomy Match|BOLD:AAJ2130
Limnophyes habilis|BIOUG15762-E06|Kate Perez|BIN Taxonomy Match|BOLD:AAJ2130
Limnophyes habilis|BIOUG15763-E09|Kate Perez|BIN Taxonomy Match|BOLD:AAJ2130
Limnophyes bidumus|ES168|Elisabeth Stur||BOLD:AAJ2137
Limnophyes sp. 11ES|Finnmark585|Elisabeth Stur||BOLD:AAU5475
Limnophyes sp. 3SW|CCDB21605-E10|Sofia Wiedenbrug||BOLD:ACC0450
Limnophyes|BIOUG04114-G08|Kate Perez|BIN Taxonomy Match|BOLD:ACC0450
Limnophyes sp. 3SW|CCDB21606-E02|Sofia Wiedenbrug||BOLD:ACC0450
Limnophyes|BIOUG04119-A06|Kate Perez|BIN Taxonomy Match|BOLD:ACC0450
Limnophyes|BIOUG04090-C07|Kate Perez|BIN Taxonomy Match|BOLD:ACC0450
Limnophyes aagaardi|ZMUO.025837|Lauri Paasivirta||BOLD:ACC0450
Limnophyes|BIOUG04090-D09|Kate Perez|BIN Taxonomy Match|BOLD:ACC0450
Limnophyes|BIOUG04366-A11|Kate Perez|BIN Taxonomy Match|BOLD:ACC0450
Limnophyes|BIOUG04119-A02|Kate Perez|BIN Taxonomy Match|BOLD:ACC0450
Limnophyes|BIOUG04114-E08|Kate Perez|BIN Taxonomy Match|BOLD:ACC0450
Limnophyes|BIOUG04114-F12|Kate Perez|BIN Taxonomy Match|BOLD:ACC0450
Limnophyes|BIOUG04090-D04|Kate Perez|BIN Taxonomy Match|BOLD:ACC0450
Limnophyes|BIOUG04114-E06|Kate Perez|BIN Taxonomy Match|BOLD:ACC0450
Limnophyes|BIOUG04090-E12|Kate Perez|BIN Taxonomy Match|BOLD:ACC0450
Limnophyes|BIOUG04212-E09|Kate Perez|BIN Taxonomy Match|BOLD:ACC0450
Limnophyes|GBOL11542|Sofia Wiedenbrug||BOLD:ACV9879
Limnophyes sp. 7ES|ZSM-DIP-33137-C03|Caroline Chimeno|BIN Taxonomy Match (Jan 2022)|BOLD ...
Limnophyes sp. 7ES|ZSM-DIP-33137-C07|Caroline Chimeno|BIN Taxonomy Match (Jan 2022)|BOLD ...
Orthocladiinae|CCDB24228-C11|Sofia Wiedenbrug|BIN Taxonomy Match|BOLD:AAJ2129
Orthocladiinae|CCDB22605-H09|Sofia Wiedenbrug|99,65% L. 7ES, shallow humeral pit with two l...
Limnophyes sp. 8SW|ZSM-DIP-33135-D05|Caroline Chimeno|BIN Taxonomy Match (Jan 2022)|BOLD ...
Limnophyes sp. 8SW|ZSM-DIP-33145-F02|Caroline Chimeno|BIN Taxonomy Match (Jan 2022)|BOLD ...
Limnophyes sp. 8SW|ZSM-DIP-33135-D03|Caroline Chimeno|BIN Taxonomy Match (Jan 2022)|BOLD ...
Limnophyes sp. 8SW|ZSM-DIP-33130-E01|Caroline Chimeno|BIN Taxonomy Match (Jan 2022)|BOLD ...
Limnophyes sp. 8SW|CCDB24183-A11|Sofia Wiedenbrug|Morphology|BOLD:ACJ0628
Limnophyes sp. 8SW|BIOUG07929-E02|Sofia Wiedenbrug|BIN Taxonomy Match|BOLD:ACJ0628
Limnophyes sp. 8SW|BIOUG08126-H05|Sofia Wiedenbrug|BIN Taxonomy Match|BOLD:ACJ0628
Limnophyes sp. 5ES|ATNA231|Elisabeth Stur||BOLD:AAD1721
Limnophyes asquamatus|ZMUO.024296|Lauri Paasivirta||BOLD:AAQ0626
Limnophyes|ZSM-DIP-33145-F06|Caroline Chimeno|BIN Taxonomy Match (Jan 2022)|BOLD:AAQ0626
Limnophyes asquamatus|ZSM-DIP-33135-D08|Caroline Chimeno|BIN Taxonomy Match (Jan 2022)|BOLD...
Limnophyes asquamatus|ZSM-DIP-33134-C11|Caroline Chimeno|BIN Taxonomy Match (Jan 2022)|BOLD...
Limnophyes asquamatus|ZSM-DIP-33134-C08|Caroline Chimeno|BIN Taxonomy Match (Jan 2022)|BOLD...
Limnophyes asquamatus|ZSM-DIP-33134-C05|Caroline Chimeno|BIN Taxonomy Match (Jan 2022)|BOLD...
Limnophyes asquamatus|ZSM-DIP-33149-A09|Caroline Chimeno|BIN Taxonomy Match (Jan 2022)|BOLD...
Limnophyes asquamatus|ZSM-DIP-33135-D07|Caroline Chimeno|BIN Taxonomy Match (Jan 2022)|BOLD...
Limnophyes asquamatus|ZSM-DIP-33134-C07|Caroline Chimeno|BIN Taxonomy Match (Jan 2022)|BOLD...
Limnophyes NHRS sp. C|NHRS-BYWS000000233|Yngve Brodin|morphology|BOLD:AAQ0626
Limnophyes asquamatus|ZSM-DIP-33149-B03|Caroline Chimeno|BIN Taxonomy Match (Jan 2022)|BOLD...
Limnophyes asquamatus|ZSM-DIP-33145-F07|Caroline Chimeno|BIN Taxonomy Match (Jan 2022)|BOLD...
Limnophyes asquamatus|ZSM-DIP-33148-D09|Caroline Chimeno|BIN Taxonomy Match (Jan 2022)|BOLD...
Limnophyes asquamatus|ZSM-DIP-33135-C10|Caroline Chimeno|BIN Taxonomy Match (Jan 2022)|BOLD...
Limnophyes asquamatus|ZSM-DIP-33134-C12|Caroline Chimeno|BIN Taxonomy Match (Jan 2022)|BOLD...
Limnophyes asquamatus|ZSM-DIP-33134-C10|Caroline Chimeno|BIN Taxonomy Match (Jan 2022)|BOLD...
Limnophyes|ZSM-DIP-33148-D11|Caroline Chimeno|BIN Taxonomy Match (Jan 2022)|BOLD:AAQ0626
Limnophyes asquamatus|ZSM-DIP-33134-C09|Caroline Chimeno|BIN Taxonomy Match (Jan 2022)|BOLD...
Limnophyes sp. 17S|NO 102|Elisabeth Stur|morphology|BOLD:AAQ0626
Limnophyes vrangelensis|Finnmark107|Elisabeth Stur||BOLD:AAM6308
Limnophyes schnelli|24464-D03|Kate Perez|BIN Taxonomy Match|BOLD:AAC9278
Limnophyes schnelli|24417-D08|Kate Perez|BIN Taxonomy Match|BOLD:AAC9278
Limnophyes schnelli|24354-E01|Kate Perez|BIN Taxonomy Match|BOLD:AAC9278
Limnophyes schnelli|24373-D09|Kate Perez|BIN Taxonomy Match|BOLD:AAC9278
Limnophyes schnelli|25132-G08|Kate Perez|BOLD ID Engine Manual (Apr 2018)|BOLD:AAC9278
Limnophyes schnelli|ZMUO.026256|Lauri Paasivirta||BOLD:AAC9278
Limnophyes schnelli|ZMUO.026255|Lauri Paasivirta||BOLD:AAC9278
Limnophyes schnelli|Finnmark368|Elisabeth Stur||BOLD:AAC9278
Limnophyes schnelli|Finnmark362|Elisabeth Stur||BOLD:AAC9278
Limnophyes cf. asquamatus|Finnmark366|Elisabeth Stur||BOLD:ACH0039
Limnophyes cf. asquamatus|CCDB21606-H06|Sofia Wiedenbrug||BOLD:ACT5780

Limnophyes schnelli|Finnmark362|Elisabeth Stur||BOLD:AAC9278
Limnophyes cf. asquamatus|Finnmark366|Elisabeth Stur||BOLD:ACH0039
Limnophyes cf. asquamatus|CCDB21606-H06|Sofia Wiedenbrug||BOLD:ACT5780
Limnophyes asquamatus|BIOUG36680-E04|Kate Perez|BIN Taxonomy Match (May 2018)|BOLD:AAD1720
Limnophyes asquamatus|BIOUG08256-G11|Kate Perez|BIN Taxonomy Match (Apr 2018)|BOLD:AAD1720
Limnophyes asquamatus|BIOUG06100-D11|Kate Perez|BIN Taxonomy Match|BOLD:AAD1720
Limnophyes asquamatus|NHRS-BYWS000000269|Yngve Brodin|morphology|BOLD:AAD1720
Limnophyes asquamatus|NHRS-BYWS000000170|Yngve Brodin|morphology|BOLD:AAD1720
Limnophyes asquamatus|NHRS-BYWS000000153|Yngve Brodin|morphology|BOLD:AAD1720
Limnophyes asquamatus|TRD-CH254|Elisabeth Stur||BOLD:AAD1720
Limnophyes asquamatus|NHRS-BYWS000000223|Yngve Brodin|morphology|BOLD:AAD1720
Limnophyes asquamatus|NHRS-BYWS000000309|Yngve Brodin|morphology|BOLD:AAD1720
Limnophyes asquamatus|ZMUO.024297|Lauri Paasivirta||BOLD:AAD1720
Limnophyes asquamatus|SOE484|Elisabeth Stur||BOLD:AAD1720
Limnophyes asquamatus|ZSM-DIP-33139-H01|Caroline Chimeno|BIN Taxonomy Match (Jan 2022)|BOLD...
Limnophyes asquamatus|ZSM34343-E03|Sofia Wiedenbrug|Morphology|BOLD:AAD1720
Limnophyes asquamatus|CCDB21606-H05|Sofia Wiedenbrug||BOLD:AAD1720
Limnophyes asquamatus|CCDB21606-D11|Sofia Wiedenbrug||BOLD:AAD1720
Limnophyes asquamatus|CCDB24183-E04|Sofia Wiedenbrug||BOLD:AAD1720
Limnophyes asquamatus|CCDB24183-A12|Sofia Wiedenbrug|Morphology|BOLD:AAD1720
Limnophyes asquamatus|BIOUG06094-B03|Kate Perez|BIN Taxonomy Match|BOLD:AAD1720
Limnophyes asquamatus|NO20|Elisabeth Stur||BOLD:AAD1720
Limnophyes asquamatus|Finnmark521|Elisabeth Stur||BOLD:AAD1720
Limnophyes asquamatus|NO21|Elisabeth Stur||BOLD:AAD1720
Limnophyes asquamatus|NO22|Elisabeth Stur||BOLD:AAD1720
Limnophyes asquamatus|Finnmark172|Elisabeth Stur||BOLD:AAD1720
Limnophyes asquamatus|Finnmark190|Elisabeth Stur||BOLD:AAD1720
Limnophyes asquamatus|Finnmark713|Elisabeth Stur||BOLD:AAD1720
Limnophyes asquamatus|Finnmark90|Elisabeth Stur||BOLD:AAD1720
Limnophyes asquamatus|Finnmark43|Elisabeth Stur||BOLD:AAD1720
Limnophyes asquamatus|Finnmark892|G. A. Halvorsen||BOLD:AAD1720
Limnophyes asquamatus|Finnmark675|Elisabeth Stur||BOLD:AAD1720
Limnophyes asquamatus|Finnmark538|Elisabeth Stur||BOLD:AAD1720
Limnophyes asquamatus|Finnmark423|Elisabeth Stur||BOLD:AAD1720
Limnophyes asquamatus|24417-D09|Kate Perez|BIN Taxonomy Match|BOLD:AAD1720
Limnophyes asquamatus|25131-A05|Kate Perez|BOLD ID Engine Manual (Apr 2018)|BOLD:AAD1720
Limnophyes|25128-F01|Kate Perez|BIN Taxonomy Match (Mar 2018)|BOLD:AAD1720
Limnophyes asquamatus|CH-eik49|Elisabeth Stur||BOLD:AAD1720
Limnophyes asquamatus|Finnmark574|Elisabeth Stur||BOLD:AAD1720
Limnophyes aagaardi|24371-F03|Kate Perez|BIN Taxonomy Match|BOLD:AAE1026
Limnophyes aagaardi|ZMUO.025836|Lauri Paasivirta||BOLD:AAE1026
Limnophyes aagaardi|24373-F05|Kate Perez|BIN Taxonomy Match|BOLD:AAE1026
Limnophyes aagaardi|24373-E07|Kate Perez|BIN Taxonomy Match|BOLD:AAE1026
Limnophyes aagaardi|23975-A05|Kate Perez|BIN Taxonomy Match|BOLD:AAE1026
Limnophyes aagaardi|24372-A05|Kate Perez|BIN Taxonomy Match|BOLD:AAE1026
Limnophyes aagaardi|23878-F02|Kate Perez|BIN Taxonomy Match|BOLD:AAE1026
Limnophyes aagaardi|SOE170|Elisabeth Stur||BOLD:AAE1026
Limnophyes aagaardi|SOE235|Elisabeth Stur||BOLD:AAE1026
Limnophyes aagaardi|SOE443|Elisabeth Stur||BOLD:AAE1026
Limnophyes aagaardi|SOE414|Elisabeth Stur||BOLD:AAE1026
Limnophyes aagaardi|SOE114|||BOLD:AAE1026
Limnophyes aagaardi|Finnmark573|Elisabeth Stur||BOLD:AAE1026
Chironomidae|24371-B02|Mikko J Tiusanen|Tree based identification|BOLD:ACU4279
Chironomidae|24354-H11|Mikko J Tiusanen|Tree based identification|BOLD:ACU4279
Mesosmittia flexuella|ZSM34342-D02|Sofia Wiedenbrug|Morfology|BOLD:ACU4856
Mesosmittia flexuella|ZSM-DIP-33149-D01|Caroline Chimeno|BIN Taxonomy Match (Jan 2022)|BOLD...
Mesosmittia flexuella|ZSM-DIP-33143-H02|Caroline Chimeno|BIN Taxonomy Match (Jan 2022)|BOLD...
Mesosmittia flexuella|PP0282|Lauri Paasivirta||BOLD:ACU4856
Mesosmittia flexuella|ZSM34343-F03|Sofia Wiedenbrug|Morfology|BOLD:ACU4856
Mesosmittia flexuella|ZSM-DIP-33144-A10|Caroline Chimeno|BIN Taxonomy Match (Jan 2022)|BOLD...
Mesosmittia flexuella|ZSM-DIP-33130-G05|Caroline Chimeno|BIN Taxonomy Match (Jan 2022)|BOLD...
Mesosmittia flexuella|CCDB24039-G08|Jerome Moriniere|BIN Taxonomy Match|BOLD:ACU4856
Mesosmittia flexuella|CCDB24039-D10|Jerome Moriniere|BIN Taxonomy Match|BOLD:ACU4856
Mesosmittia flexuella|BIOUG55199-E12|Kate Perez|BIN Taxonomy Match (Oct 2020)|BOLD:ACU4856
Mesosmittia flexuella|BC-ZSM-DIP-22491-D12|Dieter Doczkal|BIN Taxonomy Match|BOLD:ACU4856
Bryophaenocladius illimbatus|ZMUO.025189|Lauri Paasivirta||BOLD:ADA5717
Bryophaenocladius illimbatus|ZMUO.025188|Lauri Paasivirta||BOLD:ADA5717
Bryophaenocladius cf. akiensis|ZSM-DIP-33129-D01|Caroline Chimeno|BIN Taxonomy Match (Jan 2022...
Bryophaenocladius cf. akiensis|ZSM-DIP-33129-C12|Caroline Chimeno|BIN Taxonomy Match (Jan 2022...
Bryophaenocladius sp. 10ES|CH-eik41|Elisabeth Stur||BOLD:ACE7643
Prosmittia jemtlandica|PP0271|Lauri Paasivirta||BOLD:AAH3025
Prosmittia jemtlandica|ATNA275|Elisabeth Stur||BOLD:AAH3025
Prosmittia jemtlandica|ATNA282|Elisabeth Stur||BOLD:AAH3023
Prosmittia jemtlandica|ATNA306|Elisabeth Stur||BOLD:AAH3023
Prosmittia jemtlandica|ATNA248|Elisabeth Stur||BOLD:AAH3023
Rheosmittia|ATNA581|Elisabeth Stur|morphology|BOLD:ADC1204
Limnophyes sp. 10SW|CCDB24038-C03|Sofia Wiedenbrug|BIN Taxonomy Match|BOLD:ACU4439
Limnophyes sp. 10SW|CCDB24039-C02|Sofia Wiedenbrug|BIN Taxonomy Match|BOLD:ACU4439
Limnophyes sp. 10SW|ZSM34342-B03|Sofia Wiedenbrug|Morphology|BOLD:ACU4439
Limnophyes sp. 10SW|CCDB24229-A12|Sofia Wiedenbrug|BIN Taxonomy Match|BOLD:ACU4439
Limnophyes sp. 10SW|CCDB24228-F07|Sofia Wiedenbrug|BIN Taxonomy Match|BOLD:ACU4439
Limnophyes|CCDB24039-D11|Sofia Wiedenbrug|BIN Taxonomy Match|BOLD:ACU5059
Limnophyes|BC-ZSM-DIP-22551-D09|Dieter Doczkal||BOLD:ACU5059
Allocladius bothnicus|TRD-CH333|Elisabeth Stur||BOLD:ACX5865
Hydrosmittia|ES379|Elisabeth Stur||BOLD:AAL1594
Hydrosmittia|NO23|Elisabeth Stur|morphology|BOLD:AAL1594
Limnophyes eltoni|24372-B09|Kate Perez|BIN Taxonomy Match|BOLD:AAE6393
Limnophyes pumilio|ZMUO.024727|Lauri Paasivirta||BOLD:ADA9020
Limnophyes pumilio|ZMUO.024726|Lauri Paasivirta||BOLD:ADA9020
Limnophyes|ZMUO.025940|Lauri Paasivirta||BOLD:ADA9020
Limnophyes|ZMUO.025939|Lauri Paasivirta||BOLD:ADA9020
Limnophyes pumilio|NHRS-BYWS000000201|Yngve Brodin|morphology|BOLD:AAM5217
Limnophyes sp. 13ES|Finnmark726|Elisabeth Stur||BOLD:AAM5217
Limnophyes pumilio|NHRS-BYWS000000293|Yngve Brodin|morphology|BOLD:AAM5217
Limnophyes pumilio|NHRS-BYWS000000356|Yngve Brodin|morphology|BOLD:AAM5217
Limnophyes pumilio|NHRS-BYWS000000151|Yngve Brodin|morphology|BOLD:AAM5217
Limnophyes pumilio|NHRS-BYWS000000157|Yngve Brodin|morphology|BOLD:AAM5217
Limnophyes pumilio|NHRS-BYWS000000268|Yngve Brodin|morphology|BOLD:AAM5217
Limnophyes sp. 13ES|Finnmark128|Elisabeth Stur||BOLD:AAM5217
Limnophyes NHRS sp. D|NHRS-BYWS000001129|Yngve Brodin|morphology|BOLD:ACF3432
Limnophyes pumilio|EJV-20110110|Lauri Paasivirta||BOLD:ACF3432
Limnophyes pumilio|EJV-20110109|Lauri Paasivirta||BOLD:ACF3433
Limnophyes pumilio|Finnmark881|G. A. Halvorsen||BOLD:ACR6116

Limnophyes pumilio|EJV-20110110|Lauri Paasivirta||BOLD:ACF3432
Limnophyes pumilio|EJV-20110109|Lauri Paasivirta||BOLD:ACF3433
Limnophyes pumilio|Finnmark881|G. A. Halvorsen||BOLD:ACR6116
Limnophyes pumilio|24373-E05|Kate Perez|BIN Taxonomy Match|BOLD:ACR6116
Limnophyes pumilio|24372-E08|Kate Perez|BIN Taxonomy Match|BOLD:ACR6116
Limnophyes pumilio|Finnmark159|Elisabeth Stur||BOLD:ACR6116
Limnophyes pumilio|Finnmark801|Elisabeth Stur||BOLD:AAL9235
Limnophyes pumilio|EBAI-Ch264|Elisabeth Stur|morphology|BOLD:AAC5205
Limnophyes pumilio|EBAI-Ch213|Elisabeth Stur|morphology|BOLD:AAC5205
Limnophyes pumilio|ATNA290|Elisabeth Stur||BOLD:AAC5205
Limnophyes pumilio|ATNA286|Elisabeth Stur||BOLD:AAC5204
Limnophyes pumilio|ATNA462|Elisabeth Stur||BOLD:AAC5204
Limnophyes pumilio|ATNA387|Elisabeth Stur||BOLD:AAC5204
Limnophyes pumilio|Finnmark318|Elisabeth Stur||BOLD:AAC5204
Smittia|BIOUG15457-C09|Elisabeth Stur|morphology|BOLD:ACP5854
Smittia|BIOUG15458-C12|Elisabeth Stur|morphology|BOLD:ACP5854
Smittia|BIOUG15564-F07|Elisabeth Stur|morphology|BOLD:ACP5854
Smittia sp. 15ES|CH-OSF141|Elisabeth Stur||BOLD:AAV3381
Smittia sp. 15ES|CH-OSF68|Elisabeth Stur||BOLD:AAV3381
Smittia sp. 15ES|CH-OSF24|Elisabeth Stur||BOLD:AAV3381
Smittia sp. 15ES|TRD-CH161|Elisabeth Stur||BOLD:AAV3381
Smittia sp. 15ES|TRD-CH129|Elisabeth Stur||BOLD:AAV3381
Smittia sp. 15ES|TRD-CH200|Elisabeth Stur||BOLD:AAV3381
Smittia sp. 15ES|TRD-CH201|Elisabeth Stur||BOLD:AAV3381
Smittia sp. 15ES|TRD-CH202|Elisabeth Stur||BOLD:AAV3381
Smittia|BIOUG15187-D02|Kate Perez|BIN Taxonomy Match|BOLD:AAV3381
Smittia|BIOUG15183-F02|Kate Perez|BIN Taxonomy Match|BOLD:AAV3381
Smittia|BIOUG15184-H04|Kate Perez|BIN Taxonomy Match|BOLD:AAV3381
Smittia|BIOUG15187-B11|Kate Perez|BIN Taxonomy Match|BOLD:AAV3381
Smittia|BIOUG15187-D05|Kate Perez|BIN Taxonomy Match|BOLD:AAV3381
Smittia|BIOUG15462-B11|Kate Perez|BIN Taxonomy Match|BOLD:AAV3381
Smittia|BIOUG15184-G10|Kate Perez|BIN Taxonomy Match|BOLD:AAV3381
Smittia sp. 15ES|TRD-CH91|Elisabeth Stur||BOLD:AAV3381
Smittia sp. 15ES|TRD-CH84|Elisabeth Stur||BOLD:AAV3381
Smittia sp. 15ES|TRD-CH83|Elisabeth Stur||BOLD:AAV3381
Chironomidae|BIOUG04089-E03|Kate Perez|BIN Taxonomy Match|BOLD:ACC2381
Smittia sp. 24ES|24404-E08|Kate Perez|BIN Taxonomy Match|BOLD:AAG3902
Smittia sp. 24ES|BIOUG15655-H05|Kate Perez|BIN Taxonomy Match|BOLD:AAG3902
Smittia sp. 24ES|BIOUG04261-G10|Kate Perez|BIN Taxonomy Match|BOLD:AAG3902
Smittia sp. 24ES|24527-D01|Kate Perez|BIN Taxonomy Match|BOLD:AAG3902
Smittia sp. 24ES|24376-E04|Kate Perez|BIN Taxonomy Match|BOLD:AAG3902
Smittia|SOE334|Elisabeth Stur||BOLD:AAG3902
Smittia sp. 24ES|BIOUG04089-A03|Kate Perez|BIN Taxonomy Match|BOLD:AAG3902
Smittia sp. 24ES|BIOUG04089-B12|Kate Perez|BIN Taxonomy Match|BOLD:AAG3902
Smittia sp. 24ES|BIOUG04089-D02|Kate Perez|BIN Taxonomy Match|BOLD:AAG3902
Smittia sp. 24ES|24406-H10|Kate Perez|BIN Taxonomy Match|BOLD:AAG3902
Smittia sp. 24ES|BIOUG04085-H06|Kate Perez|BIN Taxonomy Match|BOLD:AAG3902
Smittia sp. 24ES|BIOUG04085-H03|Kate Perez|BIN Taxonomy Match|BOLD:AAG3902
Smittia sp. 24ES|BIOUG04269-E03|Kate Perez|BIN Taxonomy Match|BOLD:AAG3902
Smittia sp. 24ES|BIOUG04085-H09|Kate Perez|BIN Taxonomy Match|BOLD:AAG3902
Smittia sp. 24ES|BIOUG04089-C06|Kate Perez|BIN Taxonomy Match|BOLD:AAG3902
Smittia sp. 24ES|BIOUG04089-F04|Kate Perez|BIN Taxonomy Match|BOLD:AAG3902
Smittia sp. 24ES|BIOUG04089-F05|Kate Perez|BIN Taxonomy Match|BOLD:AAG3902
Smittia sp. 24ES|BIOUG04269-C08|Kate Perez|BIN Taxonomy Match|BOLD:AAG3902
Smittia sp. 24ES|BIOUG04089-B11|Kate Perez|BIN Taxonomy Match|BOLD:AAG3902
Smittia sp. 24ES|BIOUG04089-A04|Kate Perez|BIN Taxonomy Match|BOLD:AAG3902
Smittia sp. 24ES|BIOUG04085-F09|Kate Perez|BIN Taxonomy Match|BOLD:AAG3902
Smittia sp. 24ES|CCDB24229-F07|Sofia Wiedenbrug|BIN Taxonomy Match|BOLD:AAG3902
Smittia sp. 24ES|ATNA470|Elisabeth Stur||BOLD:AAG3902
Smittia sp. 2SW|ZSM-DIP-33132-G05|Caroline Chimeno|BIN Taxonomy Match (Jan 2022)|BOLD:AC...
Smittia sp. 2SW|CCDB22605-D02|Sofia Wiedenbrug||BOLD:ACT8741
Smittia sp. 2SW|CCDB22605-D06|Sofia Wiedenbrug||BOLD:ACT8741
Smittia sp. 2SW|CCDB22605-D05|Sofia Wiedenbrug||BOLD:ACT8741
Smittia sp. 2SW|CCDB22605-D04|Sofia Wiedenbrug||BOLD:ACT8741
Smittia sp. 2SW|GBOL11559|Sofia Wiedenbrug|Morphology|BOLD:ACT8741
Smittia sp. 3ES|24415-D06|Kate Perez|BIN Taxonomy Match|BOLD:AAB0374
Smittia sp. 3ES|24373-D10|Kate Perez|BIN Taxonomy Match|BOLD:AAB0374
Smittia sp. 3ES|24354-G02|Kate Perez|BIN Taxonomy Match|BOLD:AAB0374
Smittia|BIOUG15538-A07|Kate Perez|BOLD ID Engine Manual|BOLD:ACP5700
Smittia|BIOUG15464-F07|Kate Perez|BOLD ID Engine Manual|BOLD:AEH1918
Smittia sp. 18ES|Finnmark550|Elisabeth Stur||BOLD:AAQ2089
Smittia sp. 18ES|Finnmark549|Elisabeth Stur||BOLD:AAQ2089
Chironomidae|BIOUG16734-F11|Jerome Moriniere|BIN Taxonomy Match|BOLD:ACR4053
Chironomidae|BIOUG16707-D01|Jerome Moriniere|BIN Taxonomy Match|BOLD:ACR4053
Chironomidae|BIOUG16736-C02|Jerome Moriniere|BIN Taxonomy Match|BOLD:ACR4053
Chironomidae|BIOUG16736-D04|Jerome Moriniere|BIN Taxonomy Match|BOLD:ACR4053
Chironomidae|BIOUG16620-H09|Jerome Moriniere|BIN Taxonomy Match|BOLD:ACR4053
Chironomidae|BIOUG16358-E01|Jerome Moriniere|BIN Taxonomy Match|BOLD:ACR4053
Hydrosmittia sp. 1SW|CCDB24229-H09|Sofia Wiedenbrug|BIN Taxonomy Match|BOLD:ACU4328
Hydrosmittia sp. 1SW|CCDB24229-H10|Sofia Wiedenbrug|BIN Taxonomy Match|BOLD:ACU4328
Hydrosmittia|ZSM34343-D04|Sofia Wiedenbrug|Morphology
Hydrosmittia sp. 1SW|GBOL11535|Sofia Wiedenbrug|Morphology|BOLD:ACU4328
Hydrosmittia|ATNA542|Elisabeth Stur||BOLD:AEH2686
Hydrosmittia|BIOUG07758-D09|Kate Perez|BIN Taxonomy Match|BOLD:AAH3024
Hydrosmittia sp. 3ES|Finnmark765|Elisabeth Stur||BOLD:AAH3024
Hydrosmittia|ATNA268|Elisabeth Stur||BOLD:AAH3024
Hydrosmittia oxoniana|ZMUO.025382|Lauri Paasivirta||BOLD:AAH3024
Hydrosmittia oxoniana|ZMUO.025383|Lauri Paasivirta||BOLD:AAH3024
Hydrosmittia|BIOUG16257-E08|Kate Perez|BIN Taxonomy Match|BOLD:AAH3024
Hydrosmittia sp. 3ES|Finnmark65|Elisabeth Stur||BOLD:AAH3024
Hydrosmittia sp. 3ES|Finnmark618|Elisabeth Stur||BOLD:AAH3024
Chironomidae|BIOUG16594-G06|Kate Perez|BOLD ID Engine Manual|BOLD:ACR0784
Pseudosmittia gracilis|ZMUO.024637|Lauri Paasivirta||BOLD:ACP7105
Pseudosmittia gracilis|ZMUO.024636|Lauri Paasivirta||BOLD:ACP7105
Pseudosmittia gracilis|BIOUG15852-H11|Kate Perez|BIN Taxonomy Match (Aug 2017)|BOLD:ACP7105
Pseudosmittia gracilis|BIOUG15597-D10|Kate Perez|BIN Taxonomy Match (Aug 2017)|BOLD:ACP7105
OrthoGenusA|SOE373|Torbjorn Ekrem||BOLD:AAG0990
OrthoGenusA|SOE335|Torbjorn Ekrem||BOLD:AAG0990
OrthoGenusA|25131-E12|Kate Perez|BOLD ID Engine Manual (Apr 2018)|BOLD:AAG0990
OrthoGenusA|BIOUG15591-C04|Kate Perez|BIN Taxonomy Match|BOLD:AAG0990
Orthocladiinae|BIOUG15544-C01|Kate Perez|BOLD ID Engine Manual|BOLD:AAG0990
Gymnometriocnemus|BIOUG15541-A11|Kate Perez|BIN Taxonomy Match|BOLD:AAI4193
Gymnometriocnemus|BIOUG15543-A10|Kate Perez|BIN Taxonomy Match|BOLD:AAI4193

OrthoGenusA|BIOUG15591-C04|Kate Perez|BIN Taxonomy Match|BOLD:AAG0990
Orthocladiinae|BIOUG15544-C01|Kate Perez|BOLD ID Engine Manual|BOLD:AAG0990
Gymnometriocnemus|BIOUG15541-A11|Kate Perez|BIN Taxonomy Match|BOLD:AAI4193
Gymnometriocnemus|BIOUG15543-A10|Kate Perez|BIN Taxonomy Match|BOLD:AAI4193
Gymnometriocnemus|BIOUG15597-F11|Kate Perez|BIN Taxonomy Match|BOLD:AAI4193
Gymnometriocnemus|BIOUG05483-A04|Kate Perez|BIN Taxonomy Match|BOLD:AAI4193
Gymnometriocnemus|BIOUG15457-A05|Kate Perez|BIN Taxonomy Match|BOLD:AAI4193
Gymnometriocnemus|BIOUG15537-B02|Kate Perez|BIN Taxonomy Match|BOLD:AAI4193
Gymnometriocnemus|BIOUG15543-C05|Kate Perez|BIN Taxonomy Match|BOLD:AAI4193
Gymnometriocnemus|BIOUG15458-A11|Kate Perez|BIN Taxonomy Match|BOLD:AAI4193
OrthoGenusA|SOE339|Torbjorn Ekrem
Gymnometriocnemus|BIOUG15562-B08|Kate Perez|BIN Taxonomy Match|BOLD:AAI4193
Gymnometriocnemus|BIOUG15536-D10|Kate Perez|BIN Taxonomy Match|BOLD:AAI4193
Gymnometriocnemus|BIOUG15543-F01|Kate Perez|BIN Taxonomy Match|BOLD:AAI4193
Gymnometriocnemus|BIOUG15544-A07|Kate Perez|BIN Taxonomy Match|BOLD:AAI4193
Gymnometriocnemus|BIOUG15542-H05|Kate Perez|BIN Taxonomy Match|BOLD:AAI4193
Gymnometriocnemus|BIOUG15463-F06|Kate Perez|BIN Taxonomy Match|BOLD:AAI4193
Gymnometriocnemus|BIOUG15562-B02|Kate Perez|BIN Taxonomy Match|BOLD:AAI4193
Gymnometriocnemus|BIOUG15563-E11|Kate Perez|BIN Taxonomy Match|BOLD:AAI4193
Gymnometriocnemus|BIOUG15454-B07|Kate Perez|BIN Taxonomy Match|BOLD:AAI4193
Gymnometriocnemus|BIOUG15541-D10|Kate Perez|BIN Taxonomy Match|BOLD:AAI4193
Gymnometriocnemus|BIOUG15541-E12|Kate Perez|BIN Taxonomy Match|BOLD:AAI4193
Gymnometriocnemus|BIOUG15538-F11|Kate Perez|BIN Taxonomy Match|BOLD:AAI4193
Gymnometriocnemus|BIOUG15539-B09|Kate Perez|BIN Taxonomy Match|BOLD:AAI4193
Gymnometriocnemus|BIOUG15539-C10|Kate Perez|BIN Taxonomy Match|BOLD:AAI4193
Gymnometriocnemus|BIOUG15538-B07|Kate Perez|BIN Taxonomy Match|BOLD:AAI4193
Gymnometriocnemus|BIOUG15539-C02|Kate Perez|BIN Taxonomy Match|BOLD:AAI4193
Gymnometriocnemus|BIOUG15562-B03|Kate Perez|BIN Taxonomy Match|BOLD:AAI4193
Gymnometriocnemus|BIOUG15545-E02|Kate Perez|BIN Taxonomy Match|BOLD:AAI4193
Gymnometriocnemus|BIOUG15464-B02|Kate Perez|BIN Taxonomy Match|BOLD:AAI4193
Gymnometriocnemus|BIOUG15561-B05|Kate Perez|BIN Taxonomy Match|BOLD:AAI4193
Gymnometriocnemus|BIOUG15458-B01|Kate Perez|BIN Taxonomy Match|BOLD:AAI4193
Gymnometriocnemus|BIOUG15458-C04|Kate Perez|BIN Taxonomy Match|BOLD:AAI4193
Gymnometriocnemus|BIOUG15853-G06|Kate Perez|BIN Taxonomy Match|BOLD:AAI4193
Gymnometriocnemus|BIOUG15542-G11|Kate Perez|BIN Taxonomy Match|BOLD:AAI4193
Gymnometriocnemus|BIOUG15536-G09|Kate Perez|BIN Taxonomy Match|BOLD:AAI4193
Gymnometriocnemus|BIOUG15536-D05|Kate Perez|BIN Taxonomy Match|BOLD:AAI4193
Gymnometriocnemus|BIOUG15675-G10|Kate Perez|BIN Taxonomy Match|BOLD:AAI4193
Gymnometriocnemus|BIOUG15564-G12|Kate Perez|BIN Taxonomy Match|BOLD:AAI4193
Gymnometriocnemus|BIOUG15597-F10|Kate Perez|BIN Taxonomy Match|BOLD:AAI4193
Gymnometriocnemus|BIOUG15459-D09|Kate Perez|BIN Taxonomy Match|BOLD:AAI4193
Gymnometriocnemus|BIOUG15459-E02|Kate Perez|BIN Taxonomy Match|BOLD:AAI4193
Gymnometriocnemus|BIOUG15460-A10|Kate Perez|BIN Taxonomy Match|BOLD:AAI4193
Gymnometriocnemus|BIOUG15562-G09|Kate Perez|BIN Taxonomy Match|BOLD:AAI4193
Gymnometriocnemus|BIOUG15598-H02|Kate Perez|BIN Taxonomy Match|BOLD:AAI4193
Gymnometriocnemus|BIOUG15542-E01|Kate Perez|BIN Taxonomy Match|BOLD:AAI4193
Gymnometriocnemus|BIOUG15564-E03|Kate Perez|BIN Taxonomy Match|BOLD:AAI4193
Gymnometriocnemus|BIOUG15459-F05|Kate Perez|BIN Taxonomy Match|BOLD:AAI4193
Gymnometriocnemus|BIOUG15465-G04|Kate Perez|BIN Taxonomy Match|BOLD:AAI4193
Orthocladiinae|BIOUG43083-D05|Kate Perez|BIN Taxonomy Match below Phylum (May 2019)|BOLD ...
Orthocladiinae|BIOUG43083-C11|Kate Perez|BIN Taxonomy Match below Phylum (May 2019)|BOLD ...
Orthocladiinae|BIOUG43075-H02|Kate Perez|BIN Taxonomy Match below Phylum (Jun 2019)|BOLD ...
Orthocladiinae|BIOUG42964-G04|Kate Perez|BIN Taxonomy Match below Phylum (Jun 2019)|BOLD ...
Orthocladiinae|BIOUG42963-D11|Kate Perez|BIN Taxonomy Match below Phylum (Jun 2019)|BOLD ...
Orthocladiinae|BIOUG42963-B04|Kate Perez|BIN Taxonomy Match below Phylum (Jun 2019)|BOLD ...
Orthocladiinae|BIOUG42960-C11|Kate Perez|BIN Taxonomy Match below Phylum (Jun 2019)|BOLD ...
Orthocladiinae|BIOUG54161-B02|Stefan Schmidt|BIN Taxonomy Match (Sep 2020)|BOLD:ABU5526
Parasmittia carinata|ZSM34342-F01|Sofia Wiedenbrug||BOLD:ADC3841
Heterotrissocladius|CCDB22603-A08|Sofia Wiedenbrug||BOLD:ACQ4005
Parasmittia carinata|BIOUG04089-G07|Kate Perez|BIN Taxonomy Match (Aug 2017)|BOLD:ACC0435
Parasmittia carinata|BIOUG04089-G06|Kate Perez|BIN Taxonomy Match (Aug 2017)|BOLD:ACC0435
Parasmittia carinata|ZMUO.024779|Lauri Paasivirta||BOLD:ACC0435
Parasmittia carinata|BIOUG04089-H08|Kate Perez|BIN Taxonomy Match (Aug 2017)|BOLD:ACC0435
Parasmittia carinata|BIOUG04089-G04|Kate Perez|BIN Taxonomy Match (Aug 2017)|BOLD:ACC0435
Parasmittia carinata|BIOUG04089-C10|Kate Perez|BIN Taxonomy Match (Aug 2017)|BOLD:ACC0435
Parasmittia carinata|BIOUG04085-H08|Kate Perez|BIN Taxonomy Match (Aug 2017)|BOLD:ACC0435
Gymnometriocnemus|24355-C12|Kate Perez|BIN Taxonomy Match|BOLD:ACF6807
Gymnometriocnemus|24406-D06|Kate Perez|BIN Taxonomy Match|BOLD:ACF6807
Gymnometriocnemus|24539-H02|Kate Perez|BIN Taxonomy Match|BOLD:ACF6807
Gymnometriocnemus|BIOUG05485-F03|Kate Perez|BIN Taxonomy Match|BOLD:ACF6807
Gymnometriocnemus|BIOUG15537-D08|Kate Perez|BIN Taxonomy Match|BOLD:AAI4195
Gymnometriocnemus|BIOUG06058-G07|Kate Perez|BIN Taxonomy Match|BOLD:AAI4195
Gymnometriocnemus|BIOUG15464-D02|Kate Perez|BIN Taxonomy Match|BOLD:AAI4195
Gymnometriocnemus|BIOUG15539-F12|Kate Perez|BIN Taxonomy Match|BOLD:AAI4195
Gymnometriocnemus|BIOUG15545-D05|Kate Perez|BIN Taxonomy Match|BOLD:AAI4195
Gymnometriocnemus|BIOUG15456-D10|Kate Perez|BIN Taxonomy Match|BOLD:AAI4195
Gymnometriocnemus|BIOUG15460-B07|Kate Perez|BIN Taxonomy Match|BOLD:AAI4195
Gymnometriocnemus|BIOUG15460-E10|Kate Perez|BIN Taxonomy Match|BOLD:AAI4195
Gymnometriocnemus|BIOUG15544-B04|Kate Perez|BIN Taxonomy Match|BOLD:AAI4195
Gymnometriocnemus|BIOUG15455-A10|Kate Perez|BIN Taxonomy Match|BOLD:AAI4195
Gymnometriocnemus|24336-A04|Kate Perez|BIN Taxonomy Match|BOLD:AAI4195
Gymnometriocnemus|BIOUG15654-B09|Kate Perez|BIN Taxonomy Match|BOLD:AAI4195
Gymnometriocnemus|BIOUG15455-C07|Kate Perez|BIN Taxonomy Match|BOLD:AAI4195
Gymnometriocnemus|BIOUG15454-H06|Kate Perez|BIN Taxonomy Match|BOLD:AAI4195
Gymnometriocnemus|BIOUG15459-H03|Kate Perez|BIN Taxonomy Match|BOLD:AAI4195
Gymnometriocnemus|24538-G05|Kate Perez|BIN Taxonomy Match|BOLD:AAI4195
Gymnometriocnemus|BIOUG15537-F02|Kate Perez|BIN Taxonomy Match|BOLD:AAI4195
Gymnometriocnemus|BIOUG15465-E08|Kate Perez|BIN Taxonomy Match|BOLD:AAI4195
Gymnometriocnemus|BIOUG15536-C08|Kate Perez|BIN Taxonomy Match|BOLD:AAI4195
Gymnometriocnemus|BIOUG15542-B06|Kate Perez|BIN Taxonomy Match|BOLD:AAI4195
Gymnometriocnemus|BIOUG15541-C10|Kate Perez|BIN Taxonomy Match|BOLD:AAI4195
Gymnometriocnemus|BIOUG15538-C10|Kate Perez|BIN Taxonomy Match|BOLD:AAI4195
Gymnometriocnemus|BIOUG15544-A09|Kate Perez|BIN Taxonomy Match|BOLD:AAI4195
Gymnometriocnemus|BIOUG15542-D06|Kate Perez|BIN Taxonomy Match|BOLD:AAI4195
Gymnometriocnemus|BIOUG15538-G02|Kate Perez|BIN Taxonomy Match|BOLD:AAI4195
Gymnometriocnemus|BIOUG15543-D02|Kate Perez|BIN Taxonomy Match|BOLD:AAI4195
Gymnometriocnemus|BIOUG15463-E05|Kate Perez|BIN Taxonomy Match|BOLD:AAI4195
Gymnometriocnemus|BIOUG15538-C02|Kate Perez|BIN Taxonomy Match|BOLD:AAI4195
Gymnometriocnemus|BIOUG15536-H06|Kate Perez|BIN Taxonomy Match|BOLD:AAI4195
Gymnometriocnemus|BIOUG15463-G05|Kate Perez|BIN Taxonomy Match|BOLD:AAI4195
Gymnometriocnemus|BIOUG15539-F11|Kate Perez|BIN Taxonomy Match|BOLD:AAI4195
Gymnometriocnemus|BIOUG15545-D04|Kate Perez|BIN Taxonomy Match|BOLD:AAI4195
Gymnometriocnemus|BIOUG15543-F08|Kate Perez|BIN Taxonomy Match|BOLD:AAI4195
[illegible]

Gymnometriocnemus|BIOUG15539-F11|Kate Perez|BIN Taxonomy Match|BOLD:AAI4195
Gymnometriocnemus|BIOUG15545-D04|Kate Perez|BIN Taxonomy Match|BOLD:AAI4195
Gymnometriocnemus|BIOUG15543-F08|Kate Perez|BIN Taxonomy Match|BOLD:AAI4195
Gymnometriocnemus|BIOUG15457-G10|Kate Perez|BIN Taxonomy Match|BOLD:AAI4195
Gymnometriocnemus|BIOUG15538-C11|Kate Perez|BIN Taxonomy Match|BOLD:AAI4195
Gymnometriocnemus|BIOUG15675-C04|Kate Perez|BIN Taxonomy Match|BOLD:AAI4195
Gymnometriocnemus|BIOUG15464-G12|Kate Perez|BIN Taxonomy Match|BOLD:AAI4195
Gymnometriocnemus|BIOUG15454-E11|Kate Perez|BIN Taxonomy Match|BOLD:AAI4195
Gymnometriocnemus|BIOUG15459-A05|Kate Perez|BIN Taxonomy Match|BOLD:AAI4195
Gymnometriocnemus|BIOUG15455-C11|Kate Perez|BIN Taxonomy Match|BOLD:AAI4195
Gymnometriocnemus|BIOUG15539-H08|Kate Perez|BIN Taxonomy Match|BOLD:AAI4195
Gymnometriocnemus|BIOUG15542-E07|Kate Perez|BIN Taxonomy Match|BOLD:AAI4195
Gymnometriocnemus|BIOUG15563-C07|Kate Perez|BIN Taxonomy Match|BOLD:AAI4195
Gymnometriocnemus|BIOUG15537-F07|Kate Perez|BIN Taxonomy Match|BOLD:AAI4195
Gymnometriocnemus|BIOUG15563-B03|Kate Perez|BIN Taxonomy Match|BOLD:AAI4195
Gymnometriocnemus|BIOUG15563-H09|Kate Perez|BIN Taxonomy Match|BOLD:AAI4195
Gymnometriocnemus|BIOUG15188-G04|Kate Perez|BIN Taxonomy Match|BOLD:AAI4195
Gymnometriocnemus|BIOUG15540-C08|Kate Perez|BIN Taxonomy Match|BOLD:AAI4195
Gymnometriocnemus|BIOUG15459-A10|Kate Perez|BIN Taxonomy Match|BOLD:AAI4195
Gymnometriocnemus|BIOUG15537-D06|Kate Perez|BIN Taxonomy Match|BOLD:AAI4195
Gymnometriocnemus|BIOUG15538-E05|Kate Perez|BIN Taxonomy Match|BOLD:AAI4195
Gymnometriocnemus|BIOUG15563-G03|Kate Perez|BIN Taxonomy Match|BOLD:AAI4195
Gymnometriocnemus|BIOUG15458-C07|Kate Perez|BIN Taxonomy Match|BOLD:AAI4195
Gymnometriocnemus|BIOUG15459-D05|Kate Perez|BIN Taxonomy Match|BOLD:AAI4195
Gymnometriocnemus|BIOUG15456-D05|Kate Perez|BIN Taxonomy Match|BOLD:AAI4195
Gymnometriocnemus|BIOUG15454-E02|Kate Perez|BIN Taxonomy Match|BOLD:AAI4195
Gymnometriocnemus|BIOUG15564-G03|Kate Perez|BIN Taxonomy Match|BOLD:AAI4195
Gymnometriocnemus|BIOUG15458-E11|Kate Perez|BIN Taxonomy Match|BOLD:AAI4195
Gymnometriocnemus|BIOUG15545-E10|Kate Perez|BIN Taxonomy Match|BOLD:AAI4195
Gymnometriocnemus|BIOUG15545-F08|Kate Perez|BIN Taxonomy Match|BOLD:AAI4195
Gymnometriocnemus|BIOUG15564-H01|Kate Perez|BIN Taxonomy Match|BOLD:AAI4195
Gymnometriocnemus|BIOUG15675-G11|Kate Perez|BIN Taxonomy Match|BOLD:AAI4195
Gymnometriocnemus|BIOUG15454-F11|Kate Perez|BIN Taxonomy Match|BOLD:AAI4195
Gymnometriocnemus|BIOUG15544-H11|Kate Perez|BIN Taxonomy Match|BOLD:AAI4195
Gymnometriocnemus|BIOUG15542-A04|Kate Perez|BIN Taxonomy Match|BOLD:AAI4195
Gymnometriocnemus|BIOUG15541-E01|Kate Perez|BIN Taxonomy Match|BOLD:AAI4195
Gymnometriocnemus|BIOUG15536-A07|Kate Perez|BIN Taxonomy Match|BOLD:AAI4195
Gymnometriocnemus|BIOUG15536-G12|Kate Perez|BIN Taxonomy Match|BOLD:AAI4195
Gymnometriocnemus|BIOUG15538-F07|Kate Perez|BIN Taxonomy Match|BOLD:AAI4195
Gymnometriocnemus|BIOUG15536-D11|Kate Perez|BIN Taxonomy Match|BOLD:AAI4195
Gymnometriocnemus|BIOUG15538-A08|Kate Perez|BIN Taxonomy Match|BOLD:AAI4195
Gymnometriocnemus|BIOUG15458-H05|Kate Perez|BIN Taxonomy Match|BOLD:AAI4195
Gymnometriocnemus|BIOUG15539-G08|Kate Perez|BIN Taxonomy Match|BOLD:AAI4195
Gymnometriocnemus|BIOUG15464-G09|Kate Perez|BIN Taxonomy Match|BOLD:AAI4195
Gymnometriocnemus|BIOUG15458-C11|Kate Perez|BIN Taxonomy Match|BOLD:AAI4195
Gymnometriocnemus|BIOUG15852-A05|Kate Perez|BIN Taxonomy Match|BOLD:AAI4195
Gymnometriocnemus|BIOUG15540-F08|Kate Perez|BIN Taxonomy Match|BOLD:AAI4195
Gymnometriocnemus|BIOUG15464-G11|Kate Perez|BIN Taxonomy Match|BOLD:AAI4195
Gymnometriocnemus|BIOUG15540-H02|Kate Perez|BIN Taxonomy Match|BOLD:AAI4195
Gymnometriocnemus|BIOUG15536-C03|Kate Perez|BIN Taxonomy Match|BOLD:AAI4195
Gymnometriocnemus|BIOUG15536-C02|Kate Perez|BIN Taxonomy Match|BOLD:AAI4195
Gymnometriocnemus|BIOUG15561-A04|Kate Perez|BIN Taxonomy Match|BOLD:AAI4195
Gymnometriocnemus|BIOUG15562-C07|Kate Perez|BIN Taxonomy Match|BOLD:AAI4195
Gymnometriocnemus|BIOUG15537-B06|Kate Perez|BIN Taxonomy Match|BOLD:AAI4195
Gymnometriocnemus|BIOUG15542-B09|Kate Perez|BIN Taxonomy Match|BOLD:AAI4195
Gymnometriocnemus|BIOUG15465-A05|Kate Perez|BIN Taxonomy Match|BOLD:AAI4195
Gymnometriocnemus|BIOUG15545-F02|Kate Perez|BIN Taxonomy Match|BOLD:AAI4195
Gymnometriocnemus|BIOUG15564-H06|Kate Perez|BIN Taxonomy Match|BOLD:AAI4195
Gymnometriocnemus|BIOUG15563-E10|Kate Perez|BIN Taxonomy Match|BOLD:AAI4195
Gymnometriocnemus|BIOUG15464-D04|Kate Perez|BIN Taxonomy Match|BOLD:AAI4195
Gymnometriocnemus|BIOUG15542-C04|Kate Perez|BIN Taxonomy Match|BOLD:AAI4195
Gymnometriocnemus|BIOUG15541-G10|Kate Perez|BIN Taxonomy Match|BOLD:AAI4195
Gymnometriocnemus|BIOUG15536-A10|Kate Perez|BIN Taxonomy Match|BOLD:AAI4195
Gymnometriocnemus|BIOUG15539-G07|Kate Perez|BIN Taxonomy Match|BOLD:AAI4195
Gymnometriocnemus|BIOUG15538-A02|Kate Perez|BIN Taxonomy Match|BOLD:AAI4195
Gymnometriocnemus|BIOUG15539-E01|Kate Perez|BIN Taxonomy Match|BOLD:AAI4195
Gymnometriocnemus|BIOUG15562-F12|Kate Perez|BIN Taxonomy Match|BOLD:AAI4195
Gymnometriocnemus|BIOUG15457-H04|Kate Perez|BIN Taxonomy Match|BOLD:AAI4195
Gymnometriocnemus|BIOUG15537-D09|Kate Perez|BIN Taxonomy Match|BOLD:AAI4195
Gymnometriocnemus|BIOUG15538-G09|Kate Perez|BIN Taxonomy Match|BOLD:AAI4195
Gymnometriocnemus|BIOUG15457-B12|Kate Perez|BIN Taxonomy Match|BOLD:AAI4195
Gymnometriocnemus|BIOUG15537-F12|Kate Perez|BIN Taxonomy Match|BOLD:AAI4195
Gymnometriocnemus|BIOUG15536-G03|Kate Perez|BIN Taxonomy Match|BOLD:AAI4195
Gymnometriocnemus|BIOUG15593-H07|Kate Perez|BIN Taxonomy Match|BOLD:AAI4195
Gymnometriocnemus|BIOUG15458-G10|Kate Perez|BIN Taxonomy Match|BOLD:AAI4195
Gymnometriocnemus|BIOUG15457-B03|Kate Perez|BIN Taxonomy Match|BOLD:AAI4195
Gymnometriocnemus|BIOUG15542-D09|Kate Perez|BIN Taxonomy Match|BOLD:AAI4195
Gymnometriocnemus|BIOUG15536-B10|Kate Perez|BIN Taxonomy Match|BOLD:AAI4195
Gymnometriocnemus|BIOUG15537-E07|Kate Perez|BIN Taxonomy Match|BOLD:AAI4195
Gymnometriocnemus|BIOUG15538-F05|Kate Perez|BIN Taxonomy Match|BOLD:AAI4195
Gymnometriocnemus|BIOUG15538-F10|Kate Perez|BIN Taxonomy Match|BOLD:AAI4195
Gymnometriocnemus|BIOUG15538-H10|Kate Perez|BIN Taxonomy Match|BOLD:AAI4195
Gymnometriocnemus|BIOUG15539-H05|Kate Perez|BIN Taxonomy Match|BOLD:AAI4195
Gymnometriocnemus|BIOUG42960-F08|Kate Perez|BIN Taxonomy Match below Phylum (Jun 2019)|BOLD...
Gymnometriocnemus|BIOUG15460-E01|Kate Perez|BIN Taxonomy Match (Jan 2018)|BOLD:AAI4195
Gymnometriocnemus|BIOUG15464-G04|Kate Perez|BIN Taxonomy Match|BOLD:AAI4195
Gymnometriocnemus|BIOUG15543-F07|Kate Perez|BIN Taxonomy Match|BOLD:AAI4195
Gymnometriocnemus|BIOUG15458-B08|Kate Perez|BIN Taxonomy Match|BOLD:AAI4195
OrthoGenusA|SOE347|Torbjorn Ekrem
Gymnometriocnemus|BIOUG15542-E09|Kate Perez|BIN Taxonomy Match|BOLD:AAI4195
Gymnometriocnemus|BIOUG15536-C11|Kate Perez|BIN Taxonomy Match|BOLD:AAI4195
Gymnometriocnemus|BIOUG15544-B08|Kate Perez|BIN Taxonomy Match|BOLD:AAI4195
Gymnometriocnemus|BIOUG15543-E12|Kate Perez|BIN Taxonomy Match|BOLD:AAI4195
Gymnometriocnemus|BIOUG15647-H04|Kate Perez|BIN Taxonomy Match|BOLD:AAI4195
Gymnometriocnemus|BIOUG15648-F05|Kate Perez|BIN Taxonomy Match|BOLD:AAI4195
Gymnometriocnemus|BIOUG15539-E12|Kate Perez|BIN Taxonomy Match|BOLD:AAI4195
Gymnometriocnemus|BIOUG15539-G04|Kate Perez|BIN Taxonomy Match|BOLD:AAI4195
Gymnometriocnemus|BIOUG15544-E12|Kate Perez|BIN Taxonomy Match|BOLD:AAI4195
Gymnometriocnemus|BIOUG15538-E01|Kate Perez|BIN Taxonomy Match|BOLD:AAI4195
Gymnometriocnemus|BIOUG15460-C10|Kate Perez|BIN Taxonomy Match|BOLD:AAI4195
Gymnometriocnemus|BIOUG15537-D05|Kate Perez|BIN Taxonomy Match|BOLD:AAI4195
Gymnometriocnemus|BIOUG15561-D09|Kate Perez|BIN Taxonomy Match|BOLD:AAI4195
Gymnometriocnemus|BIOUG15567-G05|Kate Perez|BIN Taxonomy Match|BOLD:AAI4195

Gymnometriocnemus|BIOUG15537-D05|Kate Perez|BIN Taxonomy Match|BOLD:AAI4195
Gymnometriocnemus|BIOUG15561-D09|Kate Perez|BIN Taxonomy Match|BOLD:AAI4195
Gymnometriocnemus|BIOUG15567-G05|Kate Perez|BIN Taxonomy Match|BOLD:AAI4195
Gymnometriocnemus|BIOUG15563-C03|Kate Perez|BIN Taxonomy Match|BOLD:AAI4195
Gymnometriocnemus|BIOUG15563-C11|Kate Perez|BIN Taxonomy Match|BOLD:AAI4195
Gymnometriocnemus|BIOUG15563-D09|Kate Perez|BIN Taxonomy Match|BOLD:AAI4195
Gymnometriocnemus|BIOUG15564-A02|Kate Perez|BIN Taxonomy Match|BOLD:AAI4195
Gymnometriocnemus|BIOUG15647-D08|Kate Perez|BIN Taxonomy Match|BOLD:AAI4195
Gymnometriocnemus|BIOUG15460-E08|Kate Perez|BIN Taxonomy Match|BOLD:AAI4195
Gymnometriocnemus|BIOUG15455-B01|Kate Perez|BIN Taxonomy Match|BOLD:AAI4195
Gymnometriocnemus|BIOUG15455-F12|Kate Perez|BIN Taxonomy Match|BOLD:AAI4195
Gymnometriocnemus|BIOUG15455-G01|Kate Perez|BIN Taxonomy Match|BOLD:AAI4195
Gymnometriocnemus|BIOUG15456-E02|Kate Perez|BIN Taxonomy Match|BOLD:AAI4195
Gymnometriocnemus|BIOUG15456-F07|Kate Perez|BIN Taxonomy Match|BOLD:AAI4195
Gymnometriocnemus|BIOUG15459-B05|Kate Perez|BIN Taxonomy Match|BOLD:AAI4195
Gymnometriocnemus|BIOUG15542-F07|Kate Perez|BIN Taxonomy Match|BOLD:AAI4195
Gymnometriocnemus|BIOUG15457-D01|Kate Perez|BIN Taxonomy Match|BOLD:AAI4195
Gymnometriocnemus|BIOUG15545-F09|Kate Perez|BIN Taxonomy Match|BOLD:AAI4195
Gymnometriocnemus|BIOUG15455-C09|Kate Perez|BIN Taxonomy Match|BOLD:AAI4195
Gymnometriocnemus|BIOUG15457-G04|Kate Perez|BIN Taxonomy Match|BOLD:AAI4195
Gymnometriocnemus|BIOUG15465-B02|Kate Perez|BIN Taxonomy Match|BOLD:AAI4195
Gymnometriocnemus|BIOUG15465-C03|Kate Perez|BIN Taxonomy Match|BOLD:AAI4195
Gymnometriocnemus|BIOUG15540-F04|Kate Perez|BIN Taxonomy Match|BOLD:AAI4195
Gymnometriocnemus|BIOUG15545-D08|Kate Perez|BIN Taxonomy Match|BOLD:AAI4195
Gymnometriocnemus|BIOUG15563-A07|Kate Perez|BIN Taxonomy Match|BOLD:AAI4195
Gymnometriocnemus|BIOUG15564-C06|Kate Perez|BIN Taxonomy Match|BOLD:AAI4195
Gymnometriocnemus|BIOUG15564-G01|Kate Perez|BIN Taxonomy Match|BOLD:AAI4195
Gymnometriocnemus|BIOUG15454-A07|Kate Perez|BIN Taxonomy Match|BOLD:AAI4195
Gymnometriocnemus|BIOUG15460-F05|Kate Perez|BIN Taxonomy Match|BOLD:AAI4195
Gymnometriocnemus|BIOUG15459-C09|Kate Perez|BIN Taxonomy Match|BOLD:AAI4195
Gymnometriocnemus|BIOUG15460-F11|Kate Perez|BIN Taxonomy Match|BOLD:AAI4195
Gymnometriocnemus|BIOUG15460-F09|Kate Perez|BIN Taxonomy Match|BOLD:AAI4195
Gymnometriocnemus|BIOUG15459-F10|Kate Perez|BIN Taxonomy Match|BOLD:AAI4195
Gymnometriocnemus|BIOUG15460-E02|Kate Perez|BIN Taxonomy Match|BOLD:AAI4195
Gymnometriocnemus|BIOUG15460-E04|Kate Perez|BIN Taxonomy Match|BOLD:AAI4195
Gymnometriocnemus|BIOUG15460-E06|Kate Perez|BIN Taxonomy Match|BOLD:AAI4195
Gymnometriocnemus|BIOUG15201-G08|Kate Perez|BIN Taxonomy Match|BOLD:AAI4195
Gymnometriocnemus|BIOUG15681-H01|Kate Perez|BIN Taxonomy Match|BOLD:AAI4195
Gymnometriocnemus|BIOUG15459-H04|Kate Perez|BIN Taxonomy Match|BOLD:AAI4195
Gymnometriocnemus|BIOUG15455-E06|Kate Perez|BIN Taxonomy Match|BOLD:AAI4195
Gymnometriocnemus|BIOUG15457-B11|Kate Perez|BIN Taxonomy Match|BOLD:AAI4195
Gymnometriocnemus|BIOUG15654-C06|Kate Perez|BIN Taxonomy Match|BOLD:AAI4195
Gymnometriocnemus|BIOUG15561-G10|Kate Perez|BIN Taxonomy Match|BOLD:AAI4195
Gymnometriocnemus|BIOUG15465-H11|Kate Perez|BIN Taxonomy Match|BOLD:AAI4195
Gymnometriocnemus|BIOUG15540-E10|Kate Perez|BIN Taxonomy Match|BOLD:AAI4195
Gymnometriocnemus|BIOUG15542-A11|Kate Perez|BIN Taxonomy Match|BOLD:AAI4195
Gymnometriocnemus|BIOUG15540-E07|Kate Perez|BIN Taxonomy Match|BOLD:AAI4195
Gymnometriocnemus|BIOUG15543-G03|Kate Perez|BIN Taxonomy Match|BOLD:AAI4195
Gymnometriocnemus|BIOUG15565-D09|Kate Perez|BIN Taxonomy Match|BOLD:AAI4195
Gymnometriocnemus|BIOUG15563-D01|Kate Perez|BIN Taxonomy Match|BOLD:AAI4195
Gymnometriocnemus|BIOUG15456-F10|Kate Perez|BIN Taxonomy Match|BOLD:AAI4195
Gymnometriocnemus|BIOUG15454-B08|Kate Perez|BIN Taxonomy Match|BOLD:AAI4195
Gymnometriocnemus|BIOUG15459-D10|Kate Perez|BIN Taxonomy Match|BOLD:AAI4195
Gymnometriocnemus|BIOUG15454-F10|Kate Perez|BIN Taxonomy Match|BOLD:AAI4195
Gymnometriocnemus|BIOUG15456-E06|Kate Perez|BIN Taxonomy Match|BOLD:AAI4195
Gymnometriocnemus|BIOUG15456-G08|Kate Perez|BIN Taxonomy Match|BOLD:AAI4195
Gymnometriocnemus|BIOUG15456-H05|Kate Perez|BIN Taxonomy Match|BOLD:AAI4195
Gymnometriocnemus|BIOUG15464-C02|Kate Perez|BIN Taxonomy Match|BOLD:AAI4195
Gymnometriocnemus|BIOUG15537-H05|Kate Perez|BIN Taxonomy Match|BOLD:AAI4195
Gymnometriocnemus|BIOUG15539-B03|Kate Perez|BIN Taxonomy Match|BOLD:AAI4195
Gymnometriocnemus|BIOUG15561-B07|Kate Perez|BIN Taxonomy Match|BOLD:AAI4195
Gymnometriocnemus|BIOUG15540-B02|Kate Perez|BIN Taxonomy Match|BOLD:AAI4195
Gymnometriocnemus|BIOUG15540-B05|Kate Perez|BIN Taxonomy Match|BOLD:AAI4195
Gymnometriocnemus|BIOUG15540-H09|Kate Perez|BIN Taxonomy Match|BOLD:AAI4195
Gymnometriocnemus|BIOUG15544-E11|Kate Perez|BIN Taxonomy Match|BOLD:AAI4195
Gymnometriocnemus|BIOUG15540-A07|Kate Perez|BIN Taxonomy Match|BOLD:AAI4195
Gymnometriocnemus|BIOUG15540-B10|Kate Perez|BIN Taxonomy Match|BOLD:AAI4195
Gymnometriocnemus|BIOUG15540-C07|Kate Perez|BIN Taxonomy Match|BOLD:AAI4195
Gymnometriocnemus|BIOUG15563-A05|Kate Perez|BIN Taxonomy Match|BOLD:AAI4195
Gymnometriocnemus|BIOUG15563-B04|Kate Perez|BIN Taxonomy Match|BOLD:AAI4195
Gymnometriocnemus|BIOUG15563-B11|Kate Perez|BIN Taxonomy Match|BOLD:AAI4195
Gymnometriocnemus|BIOUG15563-C08|Kate Perez|BIN Taxonomy Match|BOLD:AAI4195
Gymnometriocnemus|BIOUG15563-F07|Kate Perez|BIN Taxonomy Match|BOLD:AAI4195
Gymnometriocnemus|BIOUG15564-B09|Kate Perez|BIN Taxonomy Match|BOLD:AAI4195
Gymnometriocnemus|BIOUG15454-C11|Kate Perez|BIN Taxonomy Match|BOLD:AAI4195
Gymnometriocnemus|BIOUG15459-H09|Kate Perez|BIN Taxonomy Match|BOLD:AAI4195
Gymnometriocnemus|BIOUG15460-B09|Kate Perez|BIN Taxonomy Match|BOLD:AAI4195
Gymnometriocnemus|BIOUG15460-D05|Kate Perez|BIN Taxonomy Match|BOLD:AAI4195
Gymnometriocnemus|BIOUG15536-A05|Kate Perez|BIN Taxonomy Match|BOLD:AAI4195
Gymnometriocnemus|BIOUG15538-B02|Kate Perez|BIN Taxonomy Match|BOLD:AAI4195
Gymnometriocnemus|BIOUG15561-C05|Kate Perez|BIN Taxonomy Match|BOLD:AAI4195
Gymnometriocnemus|BIOUG15561-B02|Kate Perez|BIN Taxonomy Match|BOLD:AAI4195
Gymnometriocnemus|BIOUG15458-F12|Kate Perez|BIN Taxonomy Match|BOLD:AAI4195
Gymnometriocnemus|BIOUG15543-A01|Kate Perez|BIN Taxonomy Match|BOLD:AAI4195
Gymnometriocnemus|BIOUG15536-E10|Kate Perez|BIN Taxonomy Match|BOLD:AAI4195
Gymnometriocnemus|BIOUG15545-B05|Kate Perez|BIN Taxonomy Match|BOLD:AAI4195
Gymnometriocnemus|BIOUG15563-F11|Kate Perez|BIN Taxonomy Match|BOLD:AAI4195
Gymnometriocnemus|BIOUG15565-A08|Kate Perez|BIN Taxonomy Match|BOLD:AAI4195
Gymnometriocnemus|BIOUG15454-B03|Kate Perez|BIN Taxonomy Match|BOLD:AAI4195
Gymnometriocnemus|BIOUG15454-E10|Kate Perez|BIN Taxonomy Match|BOLD:AAI4195
Gymnometriocnemus|BIOUG15454-D10|Kate Perez|BIN Taxonomy Match|BOLD:AAI4195
Gymnometriocnemus|BIOUG15460-F01|Kate Perez|BIN Taxonomy Match|BOLD:AAI4195
Gymnometriocnemus|BIOUG15454-G12|Kate Perez|BIN Taxonomy Match|BOLD:AAI4195
Gymnometriocnemus|BIOUG15455-B08|Kate Perez|BIN Taxonomy Match|BOLD:AAI4195
Gymnometriocnemus|BIOUG15455-E03|Kate Perez|BIN Taxonomy Match|BOLD:AAI4195
Gymnometriocnemus|BIOUG15456-B12|Kate Perez|BIN Taxonomy Match|BOLD:AAI4195
Gymnometriocnemus|BIOUG15456-G10|Kate Perez|BIN Taxonomy Match|BOLD:AAI4195
Gymnometriocnemus|BIOUG15537-D07|Kate Perez|BIN Taxonomy Match|BOLD:AAI4195
Gymnometriocnemus|BIOUG15545-C08|Kate Perez|BIN Taxonomy Match|BOLD:AAI4195
Gymnometriocnemus|BIOUG15545-H06|Kate Perez|BIN Taxonomy Match|BOLD:AAI4195
Gymnometriocnemus|BIOUG15545-C10|Kate Perez|BIN Taxonomy Match|BOLD:AAI4195
Gymnometriocnemus|BIOUG15459-G05|Kate Perez|BIN Taxonomy Match|BOLD:AAI4195
Gymnometriocnemus|BIOUG15455-B11|Kate Perez|BIN Taxonomy Match|BOLD:AAI4195

[illegible]
Gymnometriocnemus|BIOUG15545-C10|Kate Perez|BIN Taxonomy Match|BOLD:AAI4195
Gymnometriocnemus|BIOUG15459-G05|Kate Perez|BIN Taxonomy Match|BOLD:AAI4195
Gymnometriocnemus|BIOUG15455-B11|Kate Perez|BIN Taxonomy Match|BOLD:AAI4195
Gymnometriocnemus|BIOUG15456-B08|Kate Perez|BIN Taxonomy Match|BOLD:AAI4195
Gymnometriocnemus|BIOUG15458-C01|Kate Perez|BIN Taxonomy Match|BOLD:AAI4195
Gymnometriocnemus|BIOUG15454-E06|Kate Perez|BIN Taxonomy Match|BOLD:AAI4195
Gymnometriocnemus|BIOUG15464-G05|Kate Perez|BIN Taxonomy Match|BOLD:AAI4195
Gymnometriocnemus|BIOUG15543-A03|Kate Perez|BIN Taxonomy Match|BOLD:AAI4195
Gymnometriocnemus|BIOUG15544-G05|Kate Perez|BIN Taxonomy Match|BOLD:AAI4195
Gymnometriocnemus|BIOUG15454-D06|Kate Perez|BIN Taxonomy Match|BOLD:AAI4195
Gymnometriocnemus|BIOUG15536-F01|Kate Perez|BIN Taxonomy Match|BOLD:AAI4195
Gymnometriocnemus|BIOUG15537-G04|Kate Perez|BIN Taxonomy Match|BOLD:AAI4195
Gymnometriocnemus|BIOUG15540-B04|Kate Perez|BIN Taxonomy Match|BOLD:AAI4195
Gymnometriocnemus|BIOUG15542-F05|Kate Perez|BIN Taxonomy Match|BOLD:AAI4195
Gymnometriocnemus|BIOUG15545-C06|Kate Perez|BIN Taxonomy Match|BOLD:AAI4195
Gymnometriocnemus|BIOUG15545-F04|Kate Perez|BIN Taxonomy Match|BOLD:AAI4195
Gymnometriocnemus|BIOUG15454-H11|Kate Perez|BIN Taxonomy Match|BOLD:AAI4195
Gymnometriocnemus|BIOUG15458-F03|Kate Perez|BIN Taxonomy Match|BOLD:AAI4195
Gymnometriocnemus|BIOUG15460-C01|Kate Perez|BIN Taxonomy Match|BOLD:AAI4195
Gymnometriocnemus|BIOUG15455-G05|Kate Perez|BIN Taxonomy Match|BOLD:AAI4195
Gymnometriocnemus|BIOUG15456-D01|Kate Perez|BIN Taxonomy Match|BOLD:AAI4195
Gymnometriocnemus|BIOUG15456-D07|Kate Perez|BIN Taxonomy Match|BOLD:AAI4195
Gymnometriocnemus|BIOUG15456-E05|Kate Perez|BIN Taxonomy Match|BOLD:AAI4195
Gymnometriocnemus|BIOUG15537-A05|Kate Perez|BIN Taxonomy Match|BOLD:AAI4195
Gymnometriocnemus|BIOUG15542-F08|Kate Perez|BIN Taxonomy Match|BOLD:AAI4195
Gymnometriocnemus|BIOUG15543-G05|Kate Perez|BIN Taxonomy Match|BOLD:AAI4195
Gymnometriocnemus|BIOUG15543-H03|Kate Perez|BIN Taxonomy Match|BOLD:AAI4195
Gymnometriocnemus|BIOUG15561-D06|Kate Perez|BIN Taxonomy Match|BOLD:AAI4195
Gymnometriocnemus|BIOUG15459-F12|Kate Perez|BIN Taxonomy Match|BOLD:AAI4195
Gymnometriocnemus|BIOUG15201-G04|Kate Perez|BIN Taxonomy Match|BOLD:AAI4195
Gymnometriocnemus|BIOUG15541-B11|Kate Perez|BIN Taxonomy Match|BOLD:AAI4195
Gymnometriocnemus|BIOUG15463-G06|Kate Perez|BIN Taxonomy Match|BOLD:AAI4195
Gymnometriocnemus|BIOUG15456-G04|Kate Perez|BIN Taxonomy Match|BOLD:AAI4195
Gymnometriocnemus|BIOUG15543-H10|Kate Perez|BIN Taxonomy Match|BOLD:AAI4195
Gymnometriocnemus|BIOUG15561-G04|Kate Perez|BIN Taxonomy Match|BOLD:AAI4195
Gymnometriocnemus|BIOUG15457-H10|Kate Perez|BIN Taxonomy Match|BOLD:AAI4195
Gymnometriocnemus|BIOUG15456-A09|Kate Perez|BIN Taxonomy Match|BOLD:AAI4195
Gymnometriocnemus|BIOUG15454-A12|Kate Perez|BIN Taxonomy Match|BOLD:AAI4195
Gymnometriocnemus|BIOUG15455-G07|Kate Perez|BIN Taxonomy Match|BOLD:AAI4195
Gymnometriocnemus|BIOUG15456-C07|Kate Perez|BIN Taxonomy Match|BOLD:AAI4195
Gymnometriocnemus|BIOUG15545-E05|Kate Perez|BIN Taxonomy Match|BOLD:AAI4195
Gymnometriocnemus|BIOUG15561-C09|Kate Perez|BIN Taxonomy Match|BOLD:AAI4195
Gymnometriocnemus|BIOUG15545-F10|Kate Perez|BIN Taxonomy Match|BOLD:AAI4195
Gymnometriocnemus|BIOUG15564-F09|Kate Perez|BIN Taxonomy Match|BOLD:AAI4195
Gymnometriocnemus|BIOUG15561-C11|Kate Perez|BIN Taxonomy Match|BOLD:AAI4195
Gymnometriocnemus|BIOUG15561-H07|Kate Perez|BIN Taxonomy Match|BOLD:AAI4195
Gymnometriocnemus|BIOUG15457-D07|Kate Perez|BIN Taxonomy Match|BOLD:AAI4195
Gymnometriocnemus|BIOUG15457-G02|Kate Perez|BIN Taxonomy Match|BOLD:AAI4195
Gymnometriocnemus|BIOUG15561-D08|Kate Perez|BIN Taxonomy Match|BOLD:AAI4195
Gymnometriocnemus|BIOUG15562-A02|Kate Perez|BIN Taxonomy Match|BOLD:AAI4195
Gymnometriocnemus|BIOUG15675-B08|Kate Perez|BIN Taxonomy Match|BOLD:AAI4195
Gymnometriocnemus|BIOUG15463-G10|Kate Perez|BIN Taxonomy Match|BOLD:AAI4195
Gymnometriocnemus|BIOUG15561-B12|Kate Perez|BIN Taxonomy Match|BOLD:AAI4195
Gymnometriocnemus|BIOUG15456-A12|Kate Perez|BIN Taxonomy Match|BOLD:AAI4195
Gymnometriocnemus|BIOUG15455-E02|Kate Perez|BIN Taxonomy Match|BOLD:AAI4195
Gymnometriocnemus|BIOUG15561-B08|Kate Perez|BIN Taxonomy Match|BOLD:AAI4195
Gymnometriocnemus|BIOUG15561-A01|Kate Perez|BIN Taxonomy Match|BOLD:AAI4195
Gymnometriocnemus|BIOUG15464-D06|Kate Perez|BIN Taxonomy Match|BOLD:AAI4195
Gymnometriocnemus|BIOUG15464-C04|Kate Perez|BIN Taxonomy Match|BOLD:AAI4195
Gymnometriocnemus|BIOUG15541-G09|Kate Perez|BIN Taxonomy Match|BOLD:AAI4195
Gymnometriocnemus|BIOUG15541-E04|Kate Perez|BIN Taxonomy Match|BOLD:AAI4195
Gymnometriocnemus|BIOUG15465-D08|Kate Perez|BIN Taxonomy Match|BOLD:AAI4195
Gymnometriocnemus|BIOUG15465-B01|Kate Perez|BIN Taxonomy Match|BOLD:AAI4195
Gymnometriocnemus|BIOUG15458-B09|Kate Perez|BIN Taxonomy Match|BOLD:ACD9477
Gymnometriocnemus|BIOUG15458-F06|Kate Perez|BIN Taxonomy Match|BOLD:ACD9477
Gymnometriocnemus|BIOUG15458-H11|Kate Perez|BIN Taxonomy Match|BOLD:ACD9477
Gymnometriocnemus|BIOUG15459-C10|Kate Perez|BIN Taxonomy Match|BOLD:ACD9477
Gymnometriocnemus|BIOUG15460-D04|Kate Perez|BIN Taxonomy Match|BOLD:ACD9477
Gymnometriocnemus|BIOUG15456-D08|Kate Perez|BIN Taxonomy Match|BOLD:ACD9477
Gymnometriocnemus|BIOUG15456-H08|Kate Perez|BIN Taxonomy Match|BOLD:ACD9477
Gymnometriocnemus|BIOUG15456-H09|Kate Perez|BIN Taxonomy Match|BOLD:ACD9477
Gymnometriocnemus|BIOUG15457-F12|Kate Perez|BIN Taxonomy Match|BOLD:ACD9477
Gymnometriocnemus|BIOUG15454-F04|Kate Perez|BIN Taxonomy Match|BOLD:ACD9477
Gymnometriocnemus|BIOUG15456-C05|Kate Perez|BIN Taxonomy Match|BOLD:ACD9477
Gymnometriocnemus|BIOUG15457-D12|Kate Perez|BIN Taxonomy Match|BOLD:ACD9477
Gymnometriocnemus|BIOUG15456-A04|Kate Perez|BIN Taxonomy Match|BOLD:ACD9477
Gymnometriocnemus|BIOUG15457-B01|Kate Perez|BIN Taxonomy Match|BOLD:ACD9477
Gymnometriocnemus|BIOUG15456-B01|Kate Perez|BIN Taxonomy Match|BOLD:ACD9477
Gymnometriocnemus|BIOUG15457-H08|Kate Perez|BIN Taxonomy Match|BOLD:ACD9477
Gymnometriocnemus|BIOUG15460-E03|Kate Perez|BIN Taxonomy Match|BOLD:ACD9477
Gymnometriocnemus|BIOUG15459-E07|Kate Perez|BIN Taxonomy Match|BOLD:ACD9477
Gymnometriocnemus|BIOUG15459-F06|Kate Perez|BIN Taxonomy Match|BOLD:ACD9477
Gymnometriocnemus|BIOUG15459-G07|Kate Perez|BIN Taxonomy Match|BOLD:ACD9477
Gymnometriocnemus|BIOUG15460-D09|Kate Perez|BIN Taxonomy Match|BOLD:ACD9477
Gymnometriocnemus|BIOUG15454-H04|Kate Perez|BIN Taxonomy Match|BOLD:ACD9477
Gymnometriocnemus|BIOUG15456-C06|Kate Perez|BIN Taxonomy Match|BOLD:ACD9477
Gymnometriocnemus|BIOUG15455-D11|Kate Perez|BIN Taxonomy Match|BOLD:ACD9477
Gymnometriocnemus|BIOUG15459-D04|Kate Perez|BIN Taxonomy Match|BOLD:ACD9477
Gymnometriocnemus|BIOUG15460-G09|Kate Perez|BIN Taxonomy Match|BOLD:ACD9477
Gymnometriocnemus|BIOUG15460-G03|Kate Perez|BIN Taxonomy Match|BOLD:ACD9477
Gymnometriocnemus|BIOUG15454-D09|Kate Perez|BIN Taxonomy Match|BOLD:ACD9477
Gymnometriocnemus|BIOUG15455-E09|Kate Perez|BIN Taxonomy Match|BOLD:ACD9477
Gymnometriocnemus|BIOUG05108-B06|Kate Perez|BIN Taxonomy Match|BOLD:ACD9477
Gymnometriocnemus|BIOUG05110-D07|Kate Perez|BIN Taxonomy Match|BOLD:ACD9477
Gymnometriocnemus|BIOUG05107-E03|Kate Perez|BIN Taxonomy Match|BOLD:ACD9477
Gymnometriocnemus|BIOUG05103-E03|Kate Perez|BIN Taxonomy Match|BOLD:ACD9477
Gymnometriocnemus|BIOUG05141-E01|Kate Perez|BIN Taxonomy Match|BOLD:ACD9477
Gymnometriocnemus|BIOUG05107-F04|Kate Perez|BIN Taxonomy Match|BOLD:ACD9477
Gymnometriocnemus|BIOUG05104-H09|Kate Perez|BIN Taxonomy Match|BOLD:ACD9477
Gymnometriocnemus|BIOUG04795-E09|Kate Perez|BIN Taxonomy Match|BOLD:ACD9477
Bryophaenocladius|PP0284|Lauri Paasivirta||BOLD:ADZ0494
Bryophaenocladius|PP0279|Lauri Paasivirta||BOLD:ADZ0494
[illegible]

Gymnometriocnemus|BIOUG04795-E09|Kate Perez|BIN Taxonomy Match|BOLD:ACD9477
Bryophaenocladius|PP0284|Lauri Paasivirta||BOLD:ADZ0494
Bryophaenocladius|PP0279|Lauri Paasivirta||BOLD:ADZ0494
Eukiefferiella claripennis|BIOUG15542-E04|Kate Perez|Tree based identification
Eukiefferiella claripennis|BIOUG15544-H05|Kate Perez|Tree based identification
Eukiefferiella claripennis|BIOUG15541-F11|Kate Perez|Tree based identification
Eukiefferiella claripennis|BIOUG15561-H05|Kate Perez|Tree based identification
Eukiefferiella claripennis|BIOUG15537-E10|Kate Perez|Tree based identification
Eukiefferiella claripennis|ZMUO.025103|Lauri Paasivirta||BOLD:AAE4568
Eukiefferiella claripennis|ZMUO.025102|Lauri Paasivirta||BOLD:AAE4568
Eukiefferiella claripennis|BIOUG16296-F12|Kate Perez|Tree based identification
Eukiefferiella claripennis|BIOUG15544-C08|Kate Perez|Tree based identification
Eukiefferiella claripennis|ATNA250|Elisabeth Stur||BOLD:AAE4568
Eukiefferiella claripennis|EBAI-Ch063|Elisabeth Stur|morphology|BOLD:AAE4568
Eukiefferiella claripennis|EBAI-Ch116|Elisabeth Stur|BOLD Identification Engine|BOLD:AAE4568
Eukiefferiella claripennis|EBAI-Ch237|Elisabeth Stur|morphology|BOLD:AAE4568
Eukiefferiella claripennis|ATNA247|Elisabeth Stur||BOLD:AAE4568
Eukiefferiella claripennis|ATNA354|Elisabeth Stur||BOLD:AAE4568
Eukiefferiella claripennis|BIOUG15454-H09|Kate Perez|BIN Taxonomy Match|BOLD:AAE4568
Eukiefferiella claripennis|ATNA260|Elisabeth Stur||BOLD:AAE4568
Eukiefferiella claripennis|CH-OSF130|Elisabeth Stur||BOLD:AAE4568
Eukiefferiella claripennis|BIOUG15537-C12|Kate Perez|BIN Taxonomy Match|BOLD:AAE4568
Eukiefferiella claripennis|CH-OSF58|Elisabeth Stur||BOLD:AAE4568
Eukiefferiella claripennis|EBAI-Ch197|Elisabeth Stur|morphology|BOLD:AAE4568
Eukiefferiella claripennis|Finnmark767|Elisabeth Stur||BOLD:AAE4568
Eukiefferiella claripennis|Finnmark152|Elisabeth Stur||BOLD:AAE4568
Eukiefferiella claripennis|BIOUG15540-B11|Kate Perez|BIN Taxonomy Match|BOLD:AAE4568
Eukiefferiella claripennis|BIOUG16552-G09|Kate Perez|BIN Taxonomy Match|BOLD:AAE4568
Eukiefferiella claripennis|BIOUG15545-H08|Kate Perez|BIN Taxonomy Match|BOLD:AAE4568
Eukiefferiella claripennis|BIOUG15564-B01|Kate Perez|BIN Taxonomy Match|BOLD:AAE4568
Eukiefferiella claripennis|BIOUG15459-B03|Kate Perez|BIN Taxonomy Match|BOLD:AAE4568
Eukiefferiella claripennis|BIOUG16711-G03|Kate Perez|BIN Taxonomy Match|BOLD:AAE4568
Eukiefferiella claripennis|BIOUG15540-G08|Kate Perez|BIN Taxonomy Match|BOLD:AAE4568
Eukiefferiella claripennis|EBAI-Ch169|Elisabeth Stur|morphology|BOLD:AAE4568
Eukiefferiella claripennis|Finnmark759|G. A. Halvorsen||BOLD:AAE4568
Eukiefferiella claripennis|BIOUG15537-E03|Kate Perez|BIN Taxonomy Match|BOLD:AAE4568
Eukiefferiella claripennis|BIOUG15465-H08|Kate Perez|BIN Taxonomy Match|BOLD:AAE4568
Eukiefferiella claripennis|BIOUG15948-H07|Kate Perez|BIN Taxonomy Match|BOLD:AAE4568
Eukiefferiella claripennis|BIOUG15539-D01|Kate Perez|BIN Taxonomy Match|BOLD:AAE4568
Eukiefferiella claripennis|CH-OSF72|Elisabeth Stur||BOLD:AAE4568
Eukiefferiella claripennis|BIOUG16632-H02|Kate Perez|BIN Taxonomy Match|BOLD:AAE4568
Eukiefferiella claripennis|BIOUG15565-A07|Kate Perez|BIN Taxonomy Match|BOLD:AAE4568
Eukiefferiella claripennis|EBAI-Ch196|Elisabeth Stur|morphology|BOLD:AAE4568
Eukiefferiella claripennis|Finnmark412|Elisabeth Stur||BOLD:AAE4568
Halocladius cf. varians|CROBB125|Dubravka Cerba||BOLD:AEA6862
Pseudosmittia albipennis|NHRS-BYWS000000272|Yngve Brodin|morphology|BOLD:ADK6144
Pseudosmittia albipennis|NHRS-BYWS000000273|Yngve Brodin|morphology|BOLD:ADK6144
Pseudosmittia sp. 4ES|TRD-CH234|Elisabeth Stur|morphology|BOLD:ACP8837
Pseudosmittia sp. 4ES|BIOUG15796-H06|Elisabeth Stur|BOLD ID Engine Manual|BOLD:ACP8837
Pseudosmittia forcipata|CH-eik74|Elisabeth Stur||BOLD:ACG9907
Pseudosmittia forcipata|CH-eik73|Elisabeth Stur||BOLD:ACG9907
Pseudosmittia forcipata|CH-eik69|Elisabeth Stur||BOLD:ACG9907
Pseudosmittia forcipata|CH-eik68|Elisabeth Stur||BOLD:ACG9907
Pseudosmittia forcipata|CH-eik66|Elisabeth Stur||BOLD:ACG9907
Pseudosmittia cf. forcipata|CCDB22605-E09|Sofia Wiedenbrug||BOLD:ACG9907
Pseudosmittia forcipata|ZMUO.024850|Lauri Paasivirta||BOLD:ACG9907
Pseudosmittia forcipata|ZMUO.024849|Lauri Paasivirta||BOLD:ACG9907
Orthocladiinae|BIOUG55196-D09|Kate Perez|BIN Taxonomy Match (Oct 2020)|BOLD:ACI3270
Orthocladiinae|BIOUG55436-A01|Kate Perez|BIN Taxonomy Match (Oct 2020)|BOLD:ACI3270
Orthocladiinae|BIOUG55128-B03|Kate Perez|BIN Taxonomy Match (Oct 2020)|BOLD:ACI3270
Orthocladiinae|BIOUG55415-A10|Kate Perez|BIN Taxonomy Match (Oct 2020)|BOLD:ACI3270
Orthocladiinae|24520-G01|Kate Perez|BIN Taxonomy Match|BOLD:ACB2548
Pseudosmittia angusta|ZMUO.025097|Lauri Paasivirta||BOLD:ADA5897
Pseudosmittia angusta|ZMUO.025096|Lauri Paasivirta||BOLD:ADA5897
Pseudosmittia|BIOUG06100-B04|Kate Perez|BIN Taxonomy Match|BOLD:ACG8108
Pseudosmittia|BIOUG06441-E11|Kate Perez|BIN Taxonomy Match|BOLD:ACG8108
Pseudosmittia|ATNA527|Elisabeth Stur||BOLD:ADE5740
Chironomidae|23878-H10|Kate Perez|BIN Taxonomy Match|BOLD:ACS4566
Chironomidae|BIOUG16207-G03|Kate Perez|BOLD ID Engine Manual|BOLD:ACS4566
Pseudosmittia|BIOUG15676-B02|Kate Perez|BIN Taxonomy Match|BOLD:AAM6263
Pseudosmittia|BIOUG15853-A03|Kate Perez|BIN Taxonomy Match|BOLD:AAM6263
Pseudosmittia|BIOUG42759-E07|Kate Perez|BIN Taxonomy Match below Phylum (Jun 2019)|BOLD:...
Pseudosmittia|BIOUG06054-B11|Kate Perez|BIN Taxonomy Match|BOLD:AAM6263
Pseudosmittia|BIOUG07805-B10|Kate Perez|BOLD ID Engine Manual|BOLD:AAM6263
Pseudosmittia|BIOUG05945-B08|Kate Perez|BIN Taxonomy Match|BOLD:AAM6263
Pseudosmittia|BIOUG06450-D12|Kate Perez|BIN Taxonomy Match|BOLD:AAM6263
Pseudosmittia|BIOUG06208-H11|Kate Perez|BIN Taxonomy Match|BOLD:AAM6263
Pseudosmittia|BIOUG07567-B04|Kate Perez|BIN Taxonomy Match|BOLD:AAM6263
Pseudosmittia|BIOUG08133-H08|Kate Perez|BIN Taxonomy Match|BOLD:AAM6263
Pseudosmittia|BIOUG07759-A02|Kate Perez|BIN Taxonomy Match|BOLD:AAM6263
Pseudosmittia|BIOUG07759-C04|Kate Perez|BIN Taxonomy Match|BOLD:AAM6263
Pseudosmittia|BIOUG08128-A05|Kate Perez|BIN Taxonomy Match|BOLD:AAM6263
Pseudosmittia|BIOUG07944-B05|Kate Perez|BIN Taxonomy Match|BOLD:AAM6263
Pseudosmittia|BIOUG08098-C11|Kate Perez|BIN Taxonomy Match|BOLD:AAM6263
Pseudosmittia|BIOUG07566-D09|Kate Perez|BIN Taxonomy Match|BOLD:AAM6263
Pseudosmittia|BIOUG07808-A06|Kate Perez|BIN Taxonomy Match|BOLD:AAM6263
Pseudosmittia|BIOUG08174-A09|Kate Perez|BIN Taxonomy Match|BOLD:AAM6263
Pseudosmittia|BIOUG08369-B02|Kate Perez|BIN Taxonomy Match|BOLD:AAM6263
Pseudosmittia|BIOUG07928-D05|Kate Perez|BIN Taxonomy Match|BOLD:AAM6263
Pseudosmittia|BIOUG07928-F08|Kate Perez|BIN Taxonomy Match|BOLD:AAM6263
Pseudosmittia|BIOUG07568-A01|Kate Perez|BIN Taxonomy Match|BOLD:AAM6263
Pseudosmittia|BIOUG07565-A08|Kate Perez|BIN Taxonomy Match|BOLD:AAM6263
Pseudosmittia|BIOUG07810-E06|Kate Perez|BIN Taxonomy Match|BOLD:AAM6263
Pseudosmittia|BIOUG07758-D02|Kate Perez|BIN Taxonomy Match|BOLD:AAM6263
Pseudosmittia|BIOUG07757-D08|Kate Perez|BIN Taxonomy Match|BOLD:AAM6263
Pseudosmittia|BIOUG07753-F03|Kate Perez|BIN Taxonomy Match|BOLD:AAM6263
Pseudosmittia|BIOUG07759-G08|Kate Perez|BIN Taxonomy Match|BOLD:AAM6263
Pseudosmittia|BIOUG07413-D10|Kate Perez|BIN Taxonomy Match|BOLD:AAM6263
Pseudosmittia|BIOUG07944-F03|Kate Perez|BIN Taxonomy Match|BOLD:AAM6263
Pseudosmittia|BIOUG07566-H05|Kate Perez|BIN Taxonomy Match|BOLD:AAM6263
Pseudosmittia|BIOUG07566-F02|Kate Perez|BIN Taxonomy Match|BOLD:AAM6263
Pseudosmittia|BIOUG07930-A08|Kate Perez|BIN Taxonomy Match|BOLD:AAM6263
Pseudosmittia|BIOUG07930-A01|Kate Perez|BIN Taxonomy Match|BOLD:AAM6263

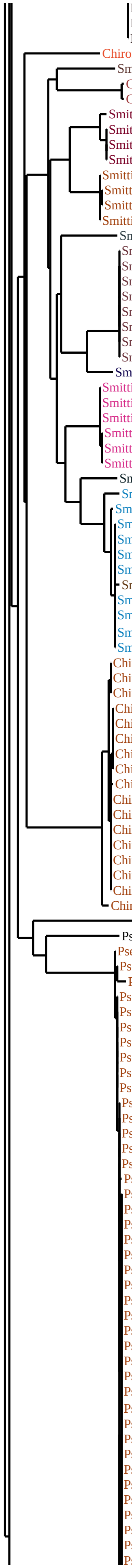

Pseudosmittia|BIOUG07566-F02|Kate Perez|BIN Taxonomy Match|BOLD:AAM6263
Pseudosmittia|BIOUG07930-A08|Kate Perez|BIN Taxonomy Match|BOLD:AAM6263
Pseudosmittia|BIOUG07930-A01|Kate Perez|BIN Taxonomy Match|BOLD:AAM6263
Chironomidae|ZSM-DIP-33145-C09|Valerie Levesque-Beaudin|BIN Taxonomy Match (Nov 2021)|BOLD...
Smittia alpilonga|ZMUO.029817|Marko Mutanen||BOLD:ADI4114
Chironomidae|CCDB24038-B04|Jerome Moriniere|BIN Taxonomy Match|BOLD:ACU4788
Chironomidae|CCDB24038-A01|Jerome Moriniere|BIN Taxonomy Match|BOLD:ACU4788
Smittia|CCDB24039-B02|Sofia Wiedenbrug|BIN Taxonomy Match|BOLD:ACU5496
Smittia|CCDB24038-A12|Sofia Wiedenbrug|BIN Taxonomy Match|BOLD:ACU5496
Smittia|CCDB24038-B05|Sofia Wiedenbrug|BIN Taxonomy Match|BOLD:ACU5496
Smittia|CCDB24038-A11|Sofia Wiedenbrug|BIN Taxonomy Match|BOLD:ACU5496
Smittia|CCDB24039-A11|Sofia Wiedenbrug|BIN Taxonomy Match|BOLD:ACU4931
Smittia|CCDB24039-A10|Sofia Wiedenbrug|BIN Taxonomy Match|BOLD:ACU4931
Smittia|CCDB24039-A09|Sofia Wiedenbrug|BIN Taxonomy Match|BOLD:ACU4931
Smittia|CCDB24037-F12|Sofia Wiedenbrug|BIN Taxonomy Match|BOLD:ACU4931
Smittia|ZSM34343-H11|Sofia Wiedenbrug|Morphology|BOLD:ADC3084
Smittia|ZSM34343-E09|Sofia Wiedenbrug|Morphology|BOLD:ACU4396
Smittia|ZSM34343-E08|Sofia Wiedenbrug|Morphology|BOLD:ACU4396
Smittia|ZSM34343-D10|Sofia Wiedenbrug|Morphology|BOLD:ACU4396
Smittia|ZSM34343-E11|Sofia Wiedenbrug|Morphology|BOLD:ACU4396
Smittia|ZSM34343-E05|Sofia Wiedenbrug|Morphology|BOLD:ACU4396
Smittia|ZSM34343-D08|Sofia Wiedenbrug|Morphology|BOLD:ACU4396
Smittia|CCDB24229-H06|Sofia Wiedenbrug|BIN Taxonomy Match|BOLD:ACU4396
Smittia|CCDB24229-D10|Sofia Wiedenbrug|BIN Taxonomy Match|BOLD:ACU4396
Smittia|CCDB24039-B07|Sofia Wiedenbrug|BIN Taxonomy Match|BOLD:ACU4853
Smittia|CCDB24229-E06|Sofia Wiedenbrug|BIN Taxonomy Match|BOLD:ACU4275
Smittia|CCDB24229-D01|Sofia Wiedenbrug|BIN Taxonomy Match|BOLD:ACU4275
Smittia|CCDB24229-C09|Sofia Wiedenbrug|BIN Taxonomy Match|BOLD:ACU4275
Smittia|CCDB24229-E07|Sofia Wiedenbrug|BIN Taxonomy Match|BOLD:ACU4275
Smittia|CCDB24229-G03|Sofia Wiedenbrug|BIN Taxonomy Match|BOLD:ACU4275
Smittia|CCDB24229-C04|Sofia Wiedenbrug|BIN Taxonomy Match|BOLD:ACU4275
Smittia|ZSM34342-C01|Sofia Wiedenbrug|Morphology|BOLD:ADC3002
Smittia|CCDB24037-G04|Sofia Wiedenbrug|BIN Taxonomy Match|BOLD:ACU4889
Smittia|ZSM34342-C06|Sofia Wiedenbrug|Morphology|BOLD:ACU4889
Smittia|ZSM34342-C09|Sofia Wiedenbrug|Morphology|BOLD:ACU4889
Smittia|CCDB24037-F11|Sofia Wiedenbrug|BIN Taxonomy Match|BOLD:ACU4889
Smittia|CCDB24229-C05|Sofia Wiedenbrug|BIN Taxonomy Match|BOLD:ACU4889
Smittia|CCDB24229-C11|Sofia Wiedenbrug|BIN Taxonomy Match|BOLD:ACU4889
Smittia|ZSM34342-C07|Sofia Wiedenbrug|Morphology
Smittia|CCDB24229-C06|Sofia Wiedenbrug|BIN Taxonomy Match|BOLD:ACU4889
Smittia|CCDB24037-G01|Sofia Wiedenbrug|BIN Taxonomy Match|BOLD:ACU4889
Smittia|CCDB24037-F10|Sofia Wiedenbrug|BIN Taxonomy Match|BOLD:ACU4889
Smittia|CCDB24037-F09|Sofia Wiedenbrug|BIN Taxonomy Match|BOLD:ACU4889
Chironomidae|ZSM-DIP-33146-D09|Valerie Levesque-Beaudin|BIN Taxonomy Match (Sep 2021)|BOLD...
Chironomidae|ZSM-DIP-33128-E02|Valerie Levesque-Beaudin|BIN Taxonomy Match (Sep 2021)|BOLD...
Chironomidae|ZSM-DIP-33130-A07|Valerie Levesque-Beaudin|BIN Taxonomy Match (Sep 2021)|BOLD...
Chironomidae|ZSM-DIP-33128-E01|Valerie Levesque-Beaudin|BIN Taxonomy Match (Sep 2021)|BOLD...
Chironomidae|ZSM-DIP-33128-D11|Valerie Levesque-Beaudin|BIN Taxonomy Match (Sep 2021)|BOLD...
Chironomidae|ZSM-DIP-33128-D10|Valerie Levesque-Beaudin|BIN Taxonomy Match (Sep 2021)|BOLD...
Chironomidae|ZSM-DIP-33128-D04|Valerie Levesque-Beaudin|BIN Taxonomy Match (Sep 2021)|BOLD...
Chironomidae|ZSM-DIP-33135-H07|Valerie Levesque-Beaudin|BIN Taxonomy Match (Sep 2021)|BOLD...
Chironomidae|ZSM-DIP-33131-B11|Valerie Levesque-Beaudin|BIN Taxonomy Match (Sep 2021)|BOLD...
Chironomidae|ZSM-DIP-33146-B12|Valerie Levesque-Beaudin|BIN Taxonomy Match (Sep 2021)|BOLD...
Chironomidae|ZSM-DIP-33128-D12|Valerie Levesque-Beaudin|BIN Taxonomy Match (Sep 2021)|BOLD...
Chironomidae|ZSM-DIP-33128-D03|Valerie Levesque-Beaudin|BIN Taxonomy Match (Sep 2021)|BOLD...
Chironomidae|ZSM-DIP-33135-H01|Valerie Levesque-Beaudin|BIN Taxonomy Match (Sep 2021)|BOLD...
Chironomidae|ZSM-DIP-33130-A09|Valerie Levesque-Beaudin|BIN Taxonomy Match (Sep 2021)|BOLD...
Chironomidae|ZSM-DIP-33130-A08|Valerie Levesque-Beaudin|BIN Taxonomy Match (Sep 2021)|BOLD...
Chironomidae|ZSM-DIP-33130-A06|Valerie Levesque-Beaudin|BIN Taxonomy Match (Sep 2021)|BOLD...
Chironomidae|ZSM-DIP-33131-A04|Valerie Levesque-Beaudin|BOLD ID Engine Manual (Sep 2021)|BO...
Chironomidae|CCDB24229-A06|Jerome Moriniere|BIN Taxonomy Match|BOLD:ACU4294
Pseudosmittia sp. 3ES|CH-OSF115|Elisabeth Stur||BOLD:AAV2838
Pseudosmittia|BIOUG07415-F05|Kate Perez|BIN Taxonomy Match|BOLD:AAG6458
Pseudosmittia|BIOUG06275-A11|Kate Perez|BIN Taxonomy Match|BOLD:AAG6458
Pseudosmittia|BIOUG07563-B02|Kate Perez|BIN Taxonomy Match|BOLD:AAG6458
Pseudosmittia|BIOUG07808-B08|Kate Perez|BIN Taxonomy Match|BOLD:AAG6458
Pseudosmittia|BIOUG07646-D02|Kate Perez|BIN Taxonomy Match|BOLD:AAG6458
Pseudosmittia|BIOUG07415-C10|Kate Perez|BIN Taxonomy Match|BOLD:AAG6458
Pseudosmittia|BIOUG07567-D03|Kate Perez|BIN Taxonomy Match|BOLD:AAG6458
Pseudosmittia|BIOUG07928-B10|Kate Perez|BIN Taxonomy Match|BOLD:AAG6458
Pseudosmittia|BIOUG06517-B12|Kate Perez|BIN Taxonomy Match|BOLD:AAG6458
Pseudosmittia|BIOUG07566-A07|Kate Perez|BIN Taxonomy Match|BOLD:AAG6458
Pseudosmittia|BIOUG07808-C09|Kate Perez|BIN Taxonomy Match|BOLD:AAG6458
Pseudosmittia|BIOUG07646-C08|Kate Perez|BIN Taxonomy Match|BOLD:AAG6458
Pseudosmittia|BIOUG07809-E08|Kate Perez|BIN Taxonomy Match|BOLD:AAG6458
Pseudosmittia|BIOUG07646-D11|Kate Perez|BIN Taxonomy Match|BOLD:AAG6458
Pseudosmittia|BIOUG07565-B01|Kate Perez|BIN Taxonomy Match|BOLD:AAG6458
Pseudosmittia|BIOUG07928-D10|Kate Perez|BIN Taxonomy Match|BOLD:AAG6458
Pseudosmittia|BIOUG06090-G09|Kate Perez|BIN Taxonomy Match|BOLD:AAG6458
Pseudosmittia|BIOUG07808-A02|Kate Perez|BIN Taxonomy Match|BOLD:AAG6458
Pseudosmittia|BIOUG07930-D10|Kate Perez|BIN Taxonomy Match|BOLD:AAG6458
Pseudosmittia|BIOUG05947-G01|Kate Perez|BIN Taxonomy Match|BOLD:AAG6458
Pseudosmittia|BIOUG07944-A04|Kate Perez|BIN Taxonomy Match|BOLD:AAG6458
Pseudosmittia|BIOUG07944-C03|Kate Perez|BIN Taxonomy Match|BOLD:AAG6458
Pseudosmittia|BIOUG07928-E06|Kate Perez|BIN Taxonomy Match|BOLD:AAG6458
Pseudosmittia|BIOUG07944-A10|Kate Perez|BIN Taxonomy Match|BOLD:AAG6458
Pseudosmittia|BIOUG07944-C11|Kate Perez|BIN Taxonomy Match|BOLD:AAG6458
Pseudosmittia|BIOUG08136-A11|Kate Perez|BIN Taxonomy Match|BOLD:AAG6458
Pseudosmittia|BIOUG07928-D11|Kate Perez|BIN Taxonomy Match|BOLD:AAG6458
Pseudosmittia|BIOUG07928-G01|Kate Perez|BIN Taxonomy Match|BOLD:AAG6458
Pseudosmittia|BIOUG07759-B10|Kate Perez|BIN Taxonomy Match|BOLD:AAG6458
Pseudosmittia|BIOUG07758-A11|Kate Perez|BIN Taxonomy Match|BOLD:AAG6458
Pseudosmittia|BIOUG07928-C09|Kate Perez|BIN Taxonomy Match|BOLD:AAG6458
Pseudosmittia|BIOUG07928-H04|Kate Perez|BIN Taxonomy Match|BOLD:AAG6458
Pseudosmittia|BIOUG07563-E09|Kate Perez|BIN Taxonomy Match|BOLD:AAG6458
Pseudosmittia|BIOUG08135-H08|Kate Perez|BIN Taxonomy Match|BOLD:AAG6458
Pseudosmittia|BIOUG07928-B09|Kate Perez|BIN Taxonomy Match|BOLD:AAG6458
Pseudosmittia|BIOUG08127-H04|Kate Perez|BIN Taxonomy Match|BOLD:AAG6458
Pseudosmittia|BIOUG07757-F09|Kate Perez|BIN Taxonomy Match|BOLD:AAG6458
Pseudosmittia|BIOUG07928-A07|Kate Perez|BIN Taxonomy Match|BOLD:AAG6458
Pseudosmittia|BIOUG06450-G12|Kate Perez|BIN Taxonomy Match|BOLD:AAG6458
Pseudosmittia|BIOUG07945-E02|Kate Perez|BIN Taxonomy Match|BOLD:AAG6458
Pseudosmittia|BIOUG07944-C09|Kate Perez|BIN Taxonomy Match|BOLD:AAG6458

Pseudosmittia|BIOUG06450-G12|Kate Perez|BIN Taxonomy Match|BOLD:AAG6458
Pseudosmittia|BIOUG07945-E02|Kate Perez|BIN Taxonomy Match|BOLD:AAG6458
Pseudosmittia|BIOUG07944-C09|Kate Perez|BIN Taxonomy Match|BOLD:AAG6458
Pseudosmittia|BIOUG07944-D09|Kate Perez|BIN Taxonomy Match|BOLD:AAG6458
Pseudosmittia|BIOUG07410-B03|Kate Perez|BIN Taxonomy Match|BOLD:AAG6458
Pseudosmittia|BIOUG07928-G02|Kate Perez|BIN Taxonomy Match|BOLD:AAG6458
Pseudosmittia|BIOUG08140-D04|Kate Perez|BIN Taxonomy Match|BOLD:AAG6458
Pseudosmittia|BIOUG07810-G02|Kate Perez|BIN Taxonomy Match|BOLD:AAG6458
Pseudosmittia|BIOUG07413-F10|Kate Perez|BIN Taxonomy Match|BOLD:AAG6458
Pseudosmittia|BIOUG07929-F06|Kate Perez|BIN Taxonomy Match|BOLD:AAG6458
Pseudosmittia|BIOUG07565-G07|Kate Perez|BIN Taxonomy Match|BOLD:AAG6458
Pseudosmittia|BIOUG07928-F06|Kate Perez|BIN Taxonomy Match|BOLD:AAG6458
Pseudosmittia|BIOUG08180-D04|Kate Perez|BIN Taxonomy Match|BOLD:AAG6458
Pseudosmittia|BIOUG07928-F04|Kate Perez|BIN Taxonomy Match|BOLD:AAG6458
Pseudosmittia|BIOUG05777-F04|Kate Perez|BIN Taxonomy Match|BOLD:AAG6458
Pseudosmittia|BIOUG07753-C04|Kate Perez|BIN Taxonomy Match|BOLD:AAG6458
Pseudosmittia|BIOUG07945-B02|Kate Perez|BIN Taxonomy Match|BOLD:AAG6458
Pseudosmittia|BIOUG07928-B12|Kate Perez|BIN Taxonomy Match|BOLD:AAG6458
Pseudosmittia|BIOUG07808-F06|Kate Perez|BIN Taxonomy Match|BOLD:AAG6458
Pseudosmittia albipennis|CH-eik86|Elisabeth Stur||BOLD:AAG6458
Pseudosmittia albipennis|CH-eik116|Elisabeth Stur||BOLD:AAG6458
Pseudosmittia albipennis|CH-eik133|Elisabeth Stur||BOLD:AAG6458
Pseudosmittia|BIOUG07757-E12|Kate Perez|BIN Taxonomy Match|BOLD:AAG6458
Pseudosmittia|BIOUG07930-G01|Kate Perez|BIN Taxonomy Match|BOLD:AAG6458
Pseudosmittia|BIOUG07930-F07|Kate Perez|BIN Taxonomy Match|BOLD:AAG6458
Pseudosmittia|BIOUG07415-H09|Kate Perez|BIN Taxonomy Match|BOLD:AAG6458
Pseudosmittia|BIOUG07930-F06|Kate Perez|BIN Taxonomy Match|BOLD:AAG6458
Pseudosmittia|BIOUG08209-F10|Kate Perez|BIN Taxonomy Match|BOLD:AAG6458
Pseudosmittia|BIOUG07808-C10|Kate Perez|BIN Taxonomy Match|BOLD:AAG6458
Pseudosmittia|BIOUG07928-C02|Kate Perez|BIN Taxonomy Match|BOLD:AAG6458
Pseudosmittia|BIOUG07930-A06|Kate Perez|BIN Taxonomy Match|BOLD:AAG6458
Pseudosmittia|BIOUG06100-A07|Kate Perez|BIN Taxonomy Match|BOLD:AAG6458
Pseudosmittia|BIOUG07805-A04|Kate Perez|BIN Taxonomy Match|BOLD:AAG6458
Pseudosmittia|BIOUG08127-F03|Kate Perez|BIN Taxonomy Match|BOLD:AAG6458
Pseudosmittia|BIOUG07928-A02|Kate Perez|BIN Taxonomy Match|BOLD:AAG6458
Pseudosmittia albipennis|ZSM-DIP-33146-A12|Caroline Chimeno|BIN Taxonomy Match (Jan 2022)|B...
Pseudosmittia albipennis|ZSM-DIP-33136-A11|Caroline Chimeno|BIN Taxonomy Match (Jan 2022)|B...
Pseudosmittia|BIOUG07760-D05|Kate Perez|BIN Taxonomy Match|BOLD:AAG6458
Pseudosmittia|BIOUG07945-G01|Kate Perez|BIN Taxonomy Match|BOLD:AAG6458
Pseudosmittia|BIOUG06210-B09|Kate Perez|BIN Taxonomy Match|BOLD:AAG6458
Pseudosmittia|BIOUG07413-C06|Kate Perez|BIN Taxonomy Match|BOLD:AAG6458
Pseudosmittia|BIOUG07418-C01|Kate Perez|BIN Taxonomy Match|BOLD:AAG6458
Pseudosmittia|BIOUG08179-A09|Kate Perez|BIN Taxonomy Match|BOLD:AAG6458
Pseudosmittia|BIOUG08132-A05|Kate Perez|BIN Taxonomy Match|BOLD:AAG6458
Pseudosmittia|BIOUG07810-H03|Kate Perez|BIN Taxonomy Match|BOLD:AAG6458
Pseudosmittia albipennis|CH-eik50|Elisabeth Stur||BOLD:AAG6458
Pseudosmittia albipennis|CH-eik51|Elisabeth Stur||BOLD:AAG6458
Pseudosmittia albipennis|CH-eik28|Elisabeth Stur||BOLD:AAG6458
Pseudosmittia albipennis|CH-eik31|Elisabeth Stur||BOLD:AAG6458
Pseudosmittia albipennis|CH-eik115|Elisabeth Stur||BOLD:AAG6458
Pseudosmittia albipennis|CH-eik52|Elisabeth Stur||BOLD:AAG6458
Pseudosmittia|BIOUG08180-C03|Kate Perez|BIN Taxonomy Match|BOLD:AAG6458
Pseudosmittia|BIOUG07408-D04|Kate Perez|BIN Taxonomy Match|BOLD:AAG6458
Pseudosmittia|BIOUG07928-C10|Kate Perez|BIN Taxonomy Match|BOLD:AAG6458
Pseudosmittia|BIOUG07566-H02|Kate Perez|BIN Taxonomy Match|BOLD:AAG6458
Pseudosmittia|BIOUG07928-D08|Kate Perez|BIN Taxonomy Match|BOLD:AAG6458
Pseudosmittia|BIOUG07944-B09|Kate Perez|BIN Taxonomy Match|BOLD:AAG6458
Pseudosmittia|BIOUG07410-H02|Kate Perez|BIN Taxonomy Match|BOLD:AAG6458
Pseudosmittia|BIOUG07413-H06|Kate Perez|BIN Taxonomy Match|BOLD:AAG6458
Pseudosmittia|BIOUG08135-A09|Kate Perez|BIN Taxonomy Match|BOLD:AAG6458
Pseudosmittia|BIOUG07928-C12|Kate Perez|BIN Taxonomy Match|BOLD:AAG6458
Pseudosmittia|BIOUG07928-F12|Kate Perez|BIN Taxonomy Match|BOLD:AAG6458
Pseudosmittia albipennis|CH-eik04|Elisabeth Stur||BOLD:AAG6458
Pseudosmittia|BIOUG07930-D09|Kate Perez|BIN Taxonomy Match|BOLD:AAG6458
Pseudosmittia|BIOUG07413-H05|Kate Perez|BIN Taxonomy Match|BOLD:AAG6458
Pseudosmittia|BIOUG07928-A12|Kate Perez|BIN Taxonomy Match|BOLD:AAG6458
Pseudosmittia|BIOUG07930-G06|Kate Perez|BIN Taxonomy Match|BOLD:AAG6458
Pseudosmittia|BIOUG07928-B01|Kate Perez|BIN Taxonomy Match|BOLD:AAG6458
Pseudosmittia|BIOUG07928-F01|Kate Perez|BIN Taxonomy Match|BOLD:AAG6458
Pseudosmittia|BIOUG07928-G06|Kate Perez|BIN Taxonomy Match|BOLD:AAG6458
Pseudosmittia|BIOUG07760-G02|Kate Perez|BIN Taxonomy Match|BOLD:AAG6458
Pseudosmittia|BIOUG07753-E01|Kate Perez|BIN Taxonomy Match|BOLD:AAG6458
Pseudosmittia|BIOUG08127-F01|Kate Perez|BIN Taxonomy Match|BOLD:AAG6458
Pseudosmittia|BIOUG07759-C11|Kate Perez|BIN Taxonomy Match|BOLD:AAG6458
Pseudosmittia|BIOUG07810-E10|Kate Perez|BIN Taxonomy Match|BOLD:AAG6458
Pseudosmittia NHRS sp. A|NHRS-BYWS000000573|Yngve Brodin|morphology|BOLD:AAG6458
Pseudosmittia|BIOUG07945-G03|Kate Perez|BIN Taxonomy Match|BOLD:AAG6458
Pseudosmittia|BIOUG07754-E03|Kate Perez|BIN Taxonomy Match|BOLD:AAG6458
Pseudosmittia|BIOUG08136-C02|Kate Perez|BIN Taxonomy Match|BOLD:AAG6458
Pseudosmittia|BIOUG07755-C12|Kate Perez|BIN Taxonomy Match|BOLD:AAG6458
Pseudosmittia|BIOUG07758-G12|Kate Perez|BIN Taxonomy Match|BOLD:AAG6458
Pseudosmittia|BIOUG05778-H05|Kate Perez|BIN Taxonomy Match|BOLD:AAG6458
Pseudosmittia|BIOUG07759-H04|Kate Perez|BIN Taxonomy Match|BOLD:AAG6458
Pseudosmittia|BIOUG07809-E07|Kate Perez|BIN Taxonomy Match|BOLD:AAG6458
Pseudosmittia|BIOUG07928-G09|Kate Perez|BIN Taxonomy Match|BOLD:AAG6458
Pseudosmittia|BIOUG07810-H05|Kate Perez|BIN Taxonomy Match|BOLD:AAG6458
Pseudosmittia|BIOUG07569-E01|Kate Perez|BIN Taxonomy Match|BOLD:AAG6458
Pseudosmittia|BIOUG07413-F03|Kate Perez|BIN Taxonomy Match|BOLD:AAG6458
Pseudosmittia|BIOUG07945-B07|Kate Perez|BIN Taxonomy Match|BOLD:AAG6458
Pseudosmittia|BIOUG07810-D01|Kate Perez|BIN Taxonomy Match|BOLD:AAG6458
Pseudosmittia|BIOUG08127-A05|Kate Perez|BIN Taxonomy Match|BOLD:AAG6458
Pseudosmittia|BIOUG07759-B08|Kate Perez|BIN Taxonomy Match|BOLD:AAG6458
Pseudosmittia|BIOUG07759-E10|Kate Perez|BIN Taxonomy Match|BOLD:AAG6458
Pseudosmittia|BIOUG07945-D05|Kate Perez|BIN Taxonomy Match|BOLD:AAG6458
Pseudosmittia|BIOUG07410-F10|Kate Perez|BIN Taxonomy Match|BOLD:AAG6458
Pseudosmittia|BIOUG07568-F08|Kate Perez|BIN Taxonomy Match|BOLD:AAG6458
Pseudosmittia|BIOUG07413-A11|Kate Perez|BIN Taxonomy Match|BOLD:AAG6458
Pseudosmittia|BIOUG07569-F02|Kate Perez|BIN Taxonomy Match|BOLD:AAG6458
Pseudosmittia|BIOUG07945-D12|Kate Perez|BIN Taxonomy Match|BOLD:AAG6458
Pseudosmittia|BIOUG08179-B12|Kate Perez|BIN Taxonomy Match|BOLD:AAG6458
Pseudosmittia|BIOUG06274-D09|Kate Perez|BIN Taxonomy Match|BOLD:AAG6458
Pseudosmittia albipennis|CH-eik03|Elisabeth Stur||BOLD:AAG6458
Pseudosmittia|BIOUG07413-H04|Kate Perez|BIN Taxonomy Match|BOLD:AAG6458

Pseudosmittia|BIOUG08175-B12|Kate Perez|BIN Taxonomy Match|BOLD:AAG6458
Pseudosmittia|BIOUG06274-D09|Kate Perez|BIN Taxonomy Match|BOLD:AAG6458
Pseudosmittia albipennis|CH-eik03|Elisabeth Stur||BOLD:AAG6458
Pseudosmittia|BIOUG07413-H04|Kate Perez|BIN Taxonomy Match|BOLD:AAG6458
Pseudosmittia|BIOUG06092-A08|Kate Perez|BIN Taxonomy Match|BOLD:AAG6458
Pseudosmittia|BIOUG07415-F08|Kate Perez|BIN Taxonomy Match|BOLD:AAG6458
Pseudosmittia|BIOUG07930-G10|Kate Perez|BIN Taxonomy Match|BOLD:AAG6458
Pseudosmittia|BIOUG08209-E01|Kate Perez|BIN Taxonomy Match|BOLD:AAG6458
Pseudosmittia|BIOUG08174-F11|Kate Perez|BIN Taxonomy Match|BOLD:AAG6458
Pseudosmittia|BIOUG07930-A04|Kate Perez|BIN Taxonomy Match|BOLD:AAG6458
Pseudosmittia|BIOUG07929-F09|Kate Perez|BIN Taxonomy Match|BOLD:AAG6458
Pseudosmittia|BIOUG07929-B07|Kate Perez|BIN Taxonomy Match|BOLD:AAG6458
Pseudosmittia|BIOUG07929-A12|Kate Perez|BIN Taxonomy Match|BOLD:AAG6458
Bryophaenocladius|ZMUO.026351|Lauri Paasivirta||BOLD:ADF1231
Bryophaenocladius nitidicollis|ZMUO.025227|Lauri Paasivirta||BOLD:AAM9434
Bryophaenocladius nitidicollis|ZMUO.025226|Lauri Paasivirta||BOLD:AAM9434
Bryophaenocladius nitidicollis|NHRS-BYWS000001144|Yngve Brodin|morphology|BOLD:AAM9434
Bryophaenocladius nitidicollis|Finnmark20|Elisabeth Stur||BOLD:AAM9434
Bryophaenocladius nitidicollis|BIOUG04269-A02|Kate Perez|BIN Taxonomy Match|BOLD:AAM9434
Bryophaenocladius scanicus|ZMUO.024960|Lauri Paasivirta||BOLD:ADA7978
Chironomidae|BIOUG36650-G07|Meredith Miller|Tree-based Identification (Jan 2019)|BOLD:AD...
Metriocnemus sp. 3ES|CH-eik110|Elisabeth Stur||BOLD:AAW0360
Metriocnemus sp. 3ES|BIOUG04090-G12|Kate Perez|BIN Taxonomy Match|BOLD:AAW0360
Metriocnemus sp. 3ES|BIOUG04296-G03|Kate Perez|BIN Taxonomy Match|BOLD:AAW0360
Metriocnemus sp. 3ES|BIOUG04153-H01|Kate Perez|BIN Taxonomy Match|BOLD:AAW0360
Metriocnemus sp. 3ES|BIOUG04153-F12|Kate Perez|BIN Taxonomy Match|BOLD:AAW0360
Metriocnemus sp. 3ES|CH-OSF49|Elisabeth Stur||BOLD:AAW0360
Metriocnemus sp. 3ES|BIOUG04090-E10|Kate Perez|BIN Taxonomy Match|BOLD:AAW0360
Metriocnemus sp. 3ES|BIOUG04090-E09|Kate Perez|BIN Taxonomy Match|BOLD:AAW0360
Metriocnemus sp. 3ES|CCDB21605-A11|Sofia Wiedenbrug||BOLD:AAW0360
Metriocnemus sp. 3ES|CCDB24228-F11|Sofia Wiedenbrug|BIN Taxonomy Match|BOLD:AAW0360
Chironomidae|Koz1142|Mikhail V. Kozlov||BOLD:AAW0360
Chironomidae|Koz1147|Mikhail V. Kozlov||BOLD:AAW0360
Metriocnemus sp. 3ES|BIOUG04362-G04|Kate Perez|BIN Taxonomy Match|BOLD:AAW0360
Metriocnemus sp. 3ES|BIOUG15797-E12|Kate Perez|BIN Taxonomy Match|BOLD:AAW0360
Metriocnemus sp. 3ES|BIOUG15855-C01|Kate Perez|BIN Taxonomy Match|BOLD:AAW0360
Metriocnemus sp. 3ES|BIOUG15761-D11|Kate Perez|BIN Taxonomy Match|BOLD:AAW0360
Metriocnemus sp. 3ES|BIOUG15600-G05|Kate Perez|BIN Taxonomy Match|BOLD:AAW0360
Metriocnemus sp. 3ES|BIOUG15761-C11|Kate Perez|BIN Taxonomy Match|BOLD:AAW0360
Metriocnemus sp. 3ES|BIOUG15792-G06|Kate Perez|BIN Taxonomy Match|BOLD:AAW0360
Metriocnemus sp. 3ES|BIOUG15796-A04|Kate Perez|BIN Taxonomy Match|BOLD:AAW0360
Metriocnemus sp. 3ES|BIOUG15718-G10|Kate Perez|BIN Taxonomy Match|BOLD:AAW0360
Metriocnemus sp. 3ES|BIOUG15967-A01|Kate Perez|BIN Taxonomy Match|BOLD:AAW0360
Metriocnemus sp. 3ES|BIOUG15720-E07|Kate Perez|BIN Taxonomy Match|BOLD:AAW0360
Metriocnemus sp. 3ES|BIOUG15718-B08|Kate Perez|BIN Taxonomy Match|BOLD:AAW0360
Bryophaenocladius|BIOUG15540-D04|Kate Perez|BOLD ID Engine Manual|BOLD:ACP6592
Bryophaenocladius dentatus|ZSM-DIP-33148-H04|Valerie Levesque-Beaudin|BOLD ID Engine Manual (S...
Bryophaenocladius dentatus|Finnmark553|Elisabeth Stur||BOLD:AAQ2041
Bryophaenocladius dentatus|Finnmark489|Elisabeth Stur||BOLD:AAQ2041
Bryophaenocladius dentatus|Finnmark488|Elisabeth Stur||BOLD:AAQ2041
Bryophaenocladius dentatus|Finnmark604|Elisabeth Stur||BOLD:AAQ2043
Chironomidae|BIOUG42744-G08|Kate Perez|BIN Taxonomy Match below Phylum (Jul 2019)|BOLD:A...
Pseudosmittia|BIOUG07418-D05|Kate Perez|BIN Taxonomy Match|BOLD:ACG7714
Pseudosmittia|BIOUG07413-E05|Kate Perez|BOLD ID Engine Manual|BOLD:ACG7714
Pseudosmittia|BIOUG08174-F06|Valerie Levesque-Beaudin||BOLD:ACG7714
Pseudosmittia|BIOUG07809-A05|Kate Perez|BIN Taxonomy Match|BOLD:ACG7714
Pseudosmittia|BIOUG08139-D08|Valerie Levesque-Beaudin||BOLD:ACG7714
Pseudosmittia|BIOUG08180-F10|Valerie Levesque-Beaudin||BOLD:ACG7714
Pseudosmittia|BIOUG07928-H08|Kate Perez|BOLD ID Engine Manual|BOLD:ACG7714
Pseudosmittia|BIOUG07809-E05|Kate Perez|BIN Taxonomy Match|BOLD:ACG7714
Pseudosmittia|BIOUG08180-B03|Valerie Levesque-Beaudin||BOLD:ACG7714
Pseudosmittia|BIOUG07753-A11|Valerie Levesque-Beaudin||BOLD:ACG7714
Pseudosmittia|BIOUG07930-H01|Valerie Levesque-Beaudin||BOLD:ACG7714
Pseudosmittia|BIOUG07758-G02|Valerie Levesque-Beaudin||BOLD:ACG7714
Pseudosmittia|BIOUG07753-A04|Valerie Levesque-Beaudin||BOLD:ACG7714
Pseudosmittia|BIOUG08128-H06|Valerie Levesque-Beaudin||BOLD:ACG7714
Pseudosmittia|BIOUG08130-A09|Valerie Levesque-Beaudin||BOLD:ACG7714
Pseudosmittia|BIOUG07758-D01|Valerie Levesque-Beaudin||BOLD:ACG7714
Pseudosmittia|BIOUG07758-G09|Valerie Levesque-Beaudin||BOLD:ACG7714
Pseudosmittia|BIOUG07647-C09|Valerie Levesque-Beaudin||BOLD:ACG7714
Pseudosmittia|BIOUG07929-C02|Valerie Levesque-Beaudin||BOLD:ACG7714
Pseudosmittia|BIOUG07754-F12|Valerie Levesque-Beaudin||BOLD:ACG7714
Pseudosmittia|BIOUG07418-C02|Kate Perez|BIN Taxonomy Match|BOLD:ACG7714
Pseudosmittia|BIOUG07805-A03|Kate Perez|BIN Taxonomy Match|BOLD:ACG7714
Pseudosmittia|BIOUG07805-G08|Kate Perez|BIN Taxonomy Match|BOLD:ACG7714
Pseudosmittia|BIOUG07754-C08|Valerie Levesque-Beaudin||BOLD:ACG7714
Pseudosmittia|BIOUG07565-D11|Valerie Levesque-Beaudin||BOLD:ACG7714
Pseudosmittia|BIOUG08126-B03|Valerie Levesque-Beaudin||BOLD:ACG7714
Pseudosmittia|BIOUG07567-C08|Valerie Levesque-Beaudin||BOLD:ACG7714
Pseudosmittia|BIOUG08174-D12|Valerie Levesque-Beaudin||BOLD:ACG7714
Pseudosmittia|BIOUG42787-E09|Kate Perez|BIN Taxonomy Match below Phylum (Jun 2019)|BOLD:...
Pseudosmittia|BIOUG55112-G10|Stefan Schmidt|BIN Taxonomy Match (Sep 2020)|BOLD:ACG7714
Pseudosmittia|BIOUG07755-E11|Valerie Levesque-Beaudin||BOLD:ACG7714
Pseudosmittia|BIOUG07944-E07|Valerie Levesque-Beaudin||BOLD:ACG7714
Pseudosmittia|BIOUG08136-E05|Valerie Levesque-Beaudin||BOLD:ACG7714
Pseudosmittia|BIOUG08128-D03|Valerie Levesque-Beaudin||BOLD:ACG7714
Pseudosmittia|BIOUG08098-H11|Valerie Levesque-Beaudin||BOLD:ACG7714
Pseudosmittia|BIOUG07928-G12|Valerie Levesque-Beaudin||BOLD:ACG7714
Pseudosmittia|BIOUG08130-A06|Valerie Levesque-Beaudin||BOLD:ACG7714
Pseudosmittia|BIOUG08130-E12|Valerie Levesque-Beaudin||BOLD:ACG7714
Pseudosmittia|BIOUG07760-A09|Valerie Levesque-Beaudin||BOLD:ACG7714
Pseudosmittia|BIOUG07930-F09|Valerie Levesque-Beaudin||BOLD:ACG7714
Pseudosmittia|BIOUG08140-E06|Valerie Levesque-Beaudin||BOLD:ACG7714
Pseudosmittia|BIOUG07758-B11|Valerie Levesque-Beaudin||BOLD:ACG7714
Pseudosmittia|BIOUG07928-E11|Valerie Levesque-Beaudin||BOLD:ACG7714
Pseudosmittia|BIOUG07759-F05|Valerie Levesque-Beaudin||BOLD:ACG7714
Pseudosmittia|BIOUG08137-D10|Valerie Levesque-Beaudin||BOLD:ACG7714
Pseudosmittia|BIOUG07928-B11|Valerie Levesque-Beaudin||BOLD:ACG7714
Pseudosmittia|BIOUG07413-D05|Valerie Levesque-Beaudin||BOLD:ACG7714
Pseudosmittia|BIOUG07809-C05|Kate Perez|BIN Taxonomy Match|BOLD:ACG7714
Pseudosmittia|BIOUG07930-A02|Valerie Levesque-Beaudin||BOLD:ACG7714
Pseudosmittia|BIOUG07930-E12|Valerie Levesque-Beaudin||BOLD:ACG7714
Pseudosmittia|BIOUG07410-F06|Valerie Levesque-Beaudin||BOLD:ACG7714
[illegible]

Pseudosmittia|BIOUG07930-A02|Valerie Levesque-Beaudin||BOLD:ACG7714
Pseudosmittia|BIOUG07930-E12|Valerie Levesque-Beaudin||BOLD:ACG7714
Pseudosmittia|BIOUG07410-F06|Valerie Levesque-Beaudin||BOLD:ACG7714
Pseudosmittia|BIOUG07928-G04|Valerie Levesque-Beaudin||BOLD:ACG7714
Pseudosmittia|BIOUG06275-G08|Valerie Levesque-Beaudin||BOLD:ACG7714
Pseudosmittia|BIOUG07413-G04|Valerie Levesque-Beaudin||BOLD:ACG7714
Pseudosmittia|BIOUG07808-A05|Kate Perez|BIN Taxonomy Match|BOLD:ACG7714
Pseudosmittia|BIOUG07810-F05|Valerie Levesque-Beaudin||BOLD:ACG7714
Pseudosmittia|BIOUG08209-E12|Valerie Levesque-Beaudin||BOLD:ACG7714
Pseudosmittia|BIOUG07944-B06|Valerie Levesque-Beaudin||BOLD:ACG7714
Pseudosmittia|BIOUG07567-C12|Valerie Levesque-Beaudin||BOLD:ACG7714
Pseudosmittia|BIOUG07567-F10|Kate Perez|BIN Taxonomy Match|BOLD:ACG7714
Pseudosmittia|BIOUG08180-H03|Valerie Levesque-Beaudin||BOLD:ACG7714
Pseudosmittia|BIOUG07566-B02|Valerie Levesque-Beaudin||BOLD:ACG7714
Pseudosmittia|BIOUG07565-H06|Valerie Levesque-Beaudin||BOLD:ACG7714
Pseudosmittia|BIOUG08139-D07|Valerie Levesque-Beaudin||BOLD:ACG7714
Pseudosmittia|BIOUG08211-B03|Valerie Levesque-Beaudin||BOLD:ACG7714
Pseudosmittia|BIOUG07930-F05|Valerie Levesque-Beaudin||BOLD:ACG7714
Pseudosmittia|BIOUG07567-F04|Kate Perez|BIN Taxonomy Match|BOLD:ACG7714
Pseudosmittia|BIOUG07808-E10|Kate Perez|BOLD ID Engine Manual|BOLD:ACG7714
Pseudosmittia|BIOUG07565-A05|Kate Perez|BIN Taxonomy Match|BOLD:ACG7714
Pseudosmittia|BIOUG07415-F11|Kate Perez|BIN Taxonomy Match|BOLD:ACG7714
Pseudosmittia|BIOUG07418-B07|Kate Perez|BOLD ID Engine Manual|BOLD:ACG7714
Pseudosmittia|BIOUG07808-G03|Kate Perez|BIN Taxonomy Match|BOLD:ACG7714
Pseudosmittia|BIOUG07929-D01|Kate Perez|BOLD ID Engine Manual|BOLD:ACG7714
Pseudosmittia|BIOUG07930-F10|Kate Perez|BIN Taxonomy Match|BOLD:ACG7714
Pseudosmittia|BIOUG08174-F07|Valerie Levesque-Beaudin||BOLD:ACG7714
Pseudosmittia|BIOUG07930-E03|Valerie Levesque-Beaudin||BOLD:ACG7714
Pseudosmittia|BIOUG07569-G10|Valerie Levesque-Beaudin||BOLD:ACG7714
Pseudosmittia|BIOUG08179-A07|Valerie Levesque-Beaudin||BOLD:ACG7714
Pseudosmittia|BIOUG08180-A06|Valerie Levesque-Beaudin||BOLD:ACG7714
Pseudosmittia|BIOUG07646-E05|Kate Perez|BIN Taxonomy Match|BOLD:ACG7714
Pseudosmittia|BIOUG07930-E05|Valerie Levesque-Beaudin||BOLD:ACG7714
Pseudosmittia|BIOUG08174-D10|Valerie Levesque-Beaudin||BOLD:ACG7714
Pseudosmittia|BIOUG08180-F03|Valerie Levesque-Beaudin||BOLD:ACG7714
Pseudosmittia|BIOUG08209-A08|Valerie Levesque-Beaudin||BOLD:ACG7714
Pseudosmittia|BIOUG08174-E10|Valerie Levesque-Beaudin||BOLD:ACG7714
Pseudosmittia|BIOUG07755-G04|Valerie Levesque-Beaudin||BOLD:ACG7714
Pseudosmittia|BIOUG08180-G07|Valerie Levesque-Beaudin||BOLD:ACG7714
Pseudosmittia|BIOUG08180-H02|Valerie Levesque-Beaudin||BOLD:ACG7714
Pseudosmittia|BIOUG08131-A03|Valerie Levesque-Beaudin||BOLD:ACG7714
Pseudosmittia|BIOUG07414-H10|Valerie Levesque-Beaudin||BOLD:ACG7714
Pseudosmittia|BIOUG08135-D08|Valerie Levesque-Beaudin||BOLD:ACG7714
Pseudosmittia|BIOUG08098-E12|Valerie Levesque-Beaudin||BOLD:ACG7714
Pseudosmittia|BIOUG08139-F11|Valerie Levesque-Beaudin||BOLD:ACG7714
Pseudosmittia|BIOUG08139-F07|Valerie Levesque-Beaudin||BOLD:ACG7714
Pseudosmittia|BIOUG08130-H06|Valerie Levesque-Beaudin||BOLD:ACG7714
Pseudosmittia|BIOUG08138-A02|Valerie Levesque-Beaudin||BOLD:ACG7714
Pseudosmittia|BIOUG08132-A08|Valerie Levesque-Beaudin||BOLD:ACG7714
Pseudosmittia|BIOUG07758-G07|Valerie Levesque-Beaudin||BOLD:ACG7714
Pseudosmittia|BIOUG07759-E12|Valerie Levesque-Beaudin||BOLD:ACG7714
Pseudosmittia|BIOUG07810-D11|Valerie Levesque-Beaudin||BOLD:ACG7714
Pseudosmittia|BIOUG07760-A10|Valerie Levesque-Beaudin||BOLD:ACG7714
Pseudosmittia|BIOUG07760-D08|Valerie Levesque-Beaudin||BOLD:ACG7714
Pseudosmittia|BIOUG07760-F06|Valerie Levesque-Beaudin||BOLD:ACG7714
Pseudosmittia|BIOUG07760-F09|Valerie Levesque-Beaudin||BOLD:ACG7714
Pseudosmittia|BIOUG07647-C08|Valerie Levesque-Beaudin||BOLD:ACG7714
Pseudosmittia|BIOUG07415-D12|Kate Perez|BIN Taxonomy Match|BOLD:ACG7714
Pseudosmittia|BIOUG08139-C05|Valerie Levesque-Beaudin||BOLD:ACG7714
Pseudosmittia|BIOUG08139-F08|Valerie Levesque-Beaudin||BOLD:ACG7714
Pseudosmittia|BIOUG08136-D04|Valerie Levesque-Beaudin||BOLD:ACG7714
Pseudosmittia|BIOUG08094-G12|Valerie Levesque-Beaudin||BOLD:ACG7714
Pseudosmittia|BIOUG07810-C07|Valerie Levesque-Beaudin||BOLD:ACG7714
Pseudosmittia|BIOUG08134-F07|Valerie Levesque-Beaudin||BOLD:ACG7714
Pseudosmittia|BIOUG07415-D09|Kate Perez|BIN Taxonomy Match|BOLD:ACG7714
Pseudosmittia|BIOUG07415-F10|Kate Perez|BIN Taxonomy Match|BOLD:ACG7714
Pseudosmittia|BIOUG06095-C06|Valerie Levesque-Beaudin||BOLD:ACG7714
Pseudosmittia|BIOUG07805-F03|Kate Perez|BOLD ID Engine Manual|BOLD:ACG7714
Pseudosmittia|BIOUG06274-F11|Valerie Levesque-Beaudin||BOLD:ACG7714
Pseudosmittia|BIOUG07805-F01|Kate Perez|BIN Taxonomy Match|BOLD:ACG7714
Pseudosmittia|BIOUG07945-G06|Valerie Levesque-Beaudin||BOLD:ACG7714
Pseudosmittia|BIOUG07759-E02|Valerie Levesque-Beaudin||BOLD:ACG7714
Pseudosmittia|BIOUG07753-D06|Valerie Levesque-Beaudin||BOLD:ACG7714
Pseudosmittia|BIOUG08211-B06|Valerie Levesque-Beaudin||BOLD:ACG7714
Pseudosmittia|BIOUG07753-D08|Valerie Levesque-Beaudin||BOLD:ACG7714
Pseudosmittia|BIOUG08134-H04|Valerie Levesque-Beaudin||BOLD:ACG7714
Pseudosmittia|BIOUG08128-D09|Valerie Levesque-Beaudin||BOLD:ACG7714
Pseudosmittia|BIOUG08128-F11|Valerie Levesque-Beaudin||BOLD:ACG7714
Pseudosmittia|BIOUG07759-G11|Valerie Levesque-Beaudin||BOLD:ACG7714
Pseudosmittia|BIOUG08174-A08|Valerie Levesque-Beaudin||BOLD:ACG7714
Pseudosmittia|BIOUG08209-H01|Valerie Levesque-Beaudin||BOLD:ACG7714
Pseudosmittia|BIOUG08180-H04|Valerie Levesque-Beaudin||BOLD:ACG7714
Pseudosmittia|BIOUG08370-C12|Valerie Levesque-Beaudin||BOLD:ACG7714
Pseudosmittia|BIOUG08139-B10|Valerie Levesque-Beaudin||BOLD:ACG7714
Pseudosmittia|BIOUG08129-E06|Valerie Levesque-Beaudin||BOLD:ACG7714
Pseudosmittia|BIOUG08174-C10|Valerie Levesque-Beaudin||BOLD:ACG7714
Pseudosmittia|BIOUG07414-B05|Valerie Levesque-Beaudin||BOLD:ACG7714
Pseudosmittia|BIOUG07753-D05|Valerie Levesque-Beaudin||BOLD:ACG7714
Pseudosmittia|BIOUG08137-C12|Valerie Levesque-Beaudin||BOLD:ACG7714
Pseudosmittia|BIOUG07414-C11|Valerie Levesque-Beaudin||BOLD:ACG7714
Pseudosmittia|BIOUG08125-C06|Valerie Levesque-Beaudin||BOLD:ACG7714
Pseudosmittia|BIOUG07566-H07|Valerie Levesque-Beaudin||BOLD:ACG7714
Pseudosmittia|BIOUG08095-A07|Valerie Levesque-Beaudin||BOLD:ACG7714
Pseudosmittia|BIOUG08127-C10|Valerie Levesque-Beaudin||BOLD:ACG7714
Pseudosmittia|BIOUG07810-A09|Valerie Levesque-Beaudin||BOLD:ACG7714
Pseudosmittia|BIOUG08132-E02|Valerie Levesque-Beaudin||BOLD:ACG7714
Pseudosmittia|BIOUG08133-D04|Valerie Levesque-Beaudin||BOLD:ACG7714
Pseudosmittia|BIOUG08131-A04|Valerie Levesque-Beaudin||BOLD:ACG7714
Pseudosmittia|BIOUG08134-F11|Valerie Levesque-Beaudin||BOLD:ACG7714
Pseudosmittia|BIOUG08094-E08|Valerie Levesque-Beaudin||BOLD:ACG7714
Pseudosmittia|BIOUG08180-G05|Valerie Levesque-Beaudin||BOLD:ACG7714
Pseudosmittia|BIOUG08140-H07|Valerie Levesque-Beaudin||BOLD:ACG7714
Pseudosmittia|BIOUG08174-A10|Valerie Levesque-Beaudin||BOLD:ACG7714

Pseudosmittia|BIOUG08180-G05|Valerie Levesque-Beaudin||BOLD:ACG7714
Pseudosmittia|BIOUG08140-H07|Valerie Levesque-Beaudin||BOLD:ACG7714
Pseudosmittia|BIOUG08174-A10|Valerie Levesque-Beaudin||BOLD:ACG7714
Pseudosmittia|BIOUG08125-C11|Valerie Levesque-Beaudin||BOLD:ACG7714
Pseudosmittia|BIOUG08136-E01|Valerie Levesque-Beaudin||BOLD:ACG7714
Pseudosmittia|BIOUG08137-F03|Valerie Levesque-Beaudin||BOLD:ACG7714
Pseudosmittia|BIOUG07563-G06|Valerie Levesque-Beaudin||BOLD:ACG7714
Pseudosmittia|BIOUG08133-E09|Valerie Levesque-Beaudin||BOLD:ACG7714
Pseudosmittia|BIOUG08133-F05|Valerie Levesque-Beaudin||BOLD:ACG7714
Pseudosmittia|BIOUG08174-C12|Valerie Levesque-Beaudin||BOLD:ACG7714
Pseudosmittia|BIOUG07760-B02|Valerie Levesque-Beaudin||BOLD:ACG7714
Pseudosmittia|BIOUG07755-E07|Valerie Levesque-Beaudin||BOLD:ACG7714
Pseudosmittia|BIOUG08129-D04|Valerie Levesque-Beaudin||BOLD:ACG7714
Pseudosmittia|BIOUG07758-B08|Valerie Levesque-Beaudin||BOLD:ACG7714
Pseudosmittia|BIOUG08094-H01|Valerie Levesque-Beaudin||BOLD:ACG7714
Pseudosmittia|BIOUG08180-H01|Valerie Levesque-Beaudin||BOLD:ACG7714
Pseudosmittia|BIOUG08211-A08|Valerie Levesque-Beaudin||BOLD:ACG7714
Pseudosmittia|BIOUG07753-A09|Valerie Levesque-Beaudin||BOLD:ACG7714
Pseudosmittia|BIOUG07410-H01|Valerie Levesque-Beaudin||BOLD:ACG7714
Pseudosmittia|BIOUG08130-G05|Valerie Levesque-Beaudin||BOLD:ACG7714
Pseudosmittia|BIOUG08137-B06|Valerie Levesque-Beaudin||BOLD:ACG7714
Pseudosmittia|BIOUG07758-C11|Valerie Levesque-Beaudin||BOLD:ACG7714
Pseudosmittia|BIOUG08131-B06|Valerie Levesque-Beaudin||BOLD:ACG7714
Pseudosmittia|BIOUG07410-C12|Valerie Levesque-Beaudin||BOLD:ACG7714
Pseudosmittia|BIOUG07759-D01|Valerie Levesque-Beaudin||BOLD:ACG7714
Pseudosmittia|BIOUG07759-G09|Valerie Levesque-Beaudin||BOLD:ACG7714
Pseudosmittia|BIOUG08132-C05|Valerie Levesque-Beaudin||BOLD:ACG7714
Pseudosmittia|BIOUG07810-H06|Valerie Levesque-Beaudin||BOLD:ACG7714
Pseudosmittia|BIOUG07760-H10|Valerie Levesque-Beaudin||BOLD:ACG7714
Pseudosmittia|BIOUG07929-D06|Valerie Levesque-Beaudin||BOLD:ACG7714
Pseudosmittia|BIOUG07568-H07|Valerie Levesque-Beaudin||BOLD:ACG7714
Pseudosmittia|BIOUG08140-B06|Valerie Levesque-Beaudin||BOLD:ACG7714
Pseudosmittia|BIOUG08140-C08|Valerie Levesque-Beaudin||BOLD:ACG7714
Pseudosmittia|BIOUG08135-F07|Valerie Levesque-Beaudin||BOLD:ACG7714
Pseudosmittia|BIOUG07944-E01|Valerie Levesque-Beaudin||BOLD:ACG7714
Pseudosmittia|BIOUG08139-A06|Valerie Levesque-Beaudin||BOLD:ACG7714
Pseudosmittia|BIOUG07759-D05|Valerie Levesque-Beaudin||BOLD:ACG7714
Pseudosmittia|BIOUG08174-C09|Valerie Levesque-Beaudin||BOLD:ACG7714
Pseudosmittia|BIOUG08174-E06|Valerie Levesque-Beaudin||BOLD:ACG7714
Pseudosmittia|BIOUG08097-A08|Valerie Levesque-Beaudin||BOLD:ACG7714
Pseudosmittia|BIOUG08140-C09|Valerie Levesque-Beaudin||BOLD:ACG7714
Pseudosmittia|BIOUG08094-G10|Valerie Levesque-Beaudin||BOLD:ACG7714
Pseudosmittia|BIOUG08138-E08|Valerie Levesque-Beaudin||BOLD:ACG7714
Pseudosmittia|BIOUG07808-E09|Kate Perez|BIN Taxonomy Match|BOLD:ACG7714
Pseudosmittia|BIOUG08370-D03|Valerie Levesque-Beaudin||BOLD:ACG7714
Pseudosmittia|BIOUG07759-B03|Valerie Levesque-Beaudin||BOLD:ACG7714
Pseudosmittia|BIOUG07646-G10|Kate Perez|BOLD ID Engine Manual|BOLD:ACG7714
Pseudosmittia|BIOUG07757-A11|Valerie Levesque-Beaudin||BOLD:ACG7714
Pseudosmittia|BIOUG07809-E06|Kate Perez|BIN Taxonomy Match|BOLD:ACG7714
Pseudosmittia|BIOUG07930-F04|Valerie Levesque-Beaudin||BOLD:ACG7714
Pseudosmittia|BIOUG07809-E03|Kate Perez|BIN Taxonomy Match|BOLD:ACG7714
Pseudosmittia|BIOUG08137-F09|Valerie Levesque-Beaudin||BOLD:ACG7714
Pseudosmittia|BIOUG07567-E11|Valerie Levesque-Beaudin||BOLD:ACG7714
Pseudosmittia|BIOUG07944-E08|Valerie Levesque-Beaudin||BOLD:ACG7714
Pseudosmittia|BIOUG08179-A05|Valerie Levesque-Beaudin||BOLD:ACG7714
Pseudosmittia|BIOUG07808-F01|Kate Perez|BIN Taxonomy Match|BOLD:ACG7714
Pseudosmittia|BIOUG07930-H02|Valerie Levesque-Beaudin||BOLD:ACG7714
Pseudosmittia|BIOUG07930-G08|Valerie Levesque-Beaudin||BOLD:ACG7714
Pseudosmittia|BIOUG07568-H02|Valerie Levesque-Beaudin||BOLD:ACG7714
Pseudosmittia|BIOUG07944-D03|Valerie Levesque-Beaudin||BOLD:ACG7714
Pseudosmittia|BIOUG07930-G07|Valerie Levesque-Beaudin||BOLD:ACG7714
Pseudosmittia|BIOUG08139-G03|Valerie Levesque-Beaudin||BOLD:ACG7714
Pseudosmittia|BIOUG08174-B09|Valerie Levesque-Beaudin||BOLD:ACG7714
Pseudosmittia|BIOUG07569-A01|Valerie Levesque-Beaudin||BOLD:ACG7714
Pseudosmittia|BIOUG07414-B08|Valerie Levesque-Beaudin||BOLD:ACG7714
Pseudosmittia|BIOUG08138-A11|Valerie Levesque-Beaudin||BOLD:ACG7714
Pseudosmittia|BIOUG08094-D06|Valerie Levesque-Beaudin||BOLD:ACG7714
Pseudosmittia|BIOUG07928-E12|Valerie Levesque-Beaudin||BOLD:ACG7714
Pseudosmittia|BIOUG08127-B06|Valerie Levesque-Beaudin||BOLD:ACG7714
Pseudosmittia|BIOUG08131-G10|Valerie Levesque-Beaudin||BOLD:ACG7714
Pseudosmittia|BIOUG07410-D07|Valerie Levesque-Beaudin||BOLD:ACG7714
Pseudosmittia|BIOUG08134-A06|Valerie Levesque-Beaudin||BOLD:ACG7714
Pseudosmittia|BIOUG08180-A11|Valerie Levesque-Beaudin||BOLD:ACG7714
Pseudosmittia|BIOUG07569-H02|Valerie Levesque-Beaudin||BOLD:ACG7714
Pseudosmittia|BIOUG07928-G05|Valerie Levesque-Beaudin||BOLD:ACG7714
Pseudosmittia|BIOUG08180-B02|Valerie Levesque-Beaudin||BOLD:ACG7714
Pseudosmittia|BIOUG08180-B09|Valerie Levesque-Beaudin||BOLD:ACG7714
Pseudosmittia|BIOUG08180-G01|Valerie Levesque-Beaudin||BOLD:ACG7714
Pseudosmittia|BIOUG08209-E09|Valerie Levesque-Beaudin||BOLD:ACG7714
Pseudosmittia|BIOUG08135-A07|Valerie Levesque-Beaudin||BOLD:ACG7714
Pseudosmittia|BIOUG08135-A10|Valerie Levesque-Beaudin||BOLD:ACG7714
Pseudosmittia|BIOUG07567-E05|Valerie Levesque-Beaudin||BOLD:ACG7714
Pseudosmittia|BIOUG08209-G11|Valerie Levesque-Beaudin||BOLD:ACG7714
Pseudosmittia|BIOUG07928-A05|Valerie Levesque-Beaudin||BOLD:ACG7714
Pseudosmittia|BIOUG07945-H11|Valerie Levesque-Beaudin||BOLD:ACG7714
Pseudosmittia|BIOUG08174-D07|Valerie Levesque-Beaudin||BOLD:ACG7714
Pseudosmittia|BIOUG06450-B02|Valerie Levesque-Beaudin||BOLD:ACG7714
Pseudosmittia|BIOUG07810-A03|Valerie Levesque-Beaudin||BOLD:ACG7714
Pseudosmittia|BIOUG08174-E05|Valerie Levesque-Beaudin||BOLD:ACG7714
Pseudosmittia|BIOUG08127-C01|Valerie Levesque-Beaudin||BOLD:ACG7714
Pseudosmittia|BIOUG08127-C06|Valerie Levesque-Beaudin||BOLD:ACG7714
Pseudosmittia|BIOUG08174-F04|Valerie Levesque-Beaudin||BOLD:ACG7714
Pseudosmittia|BIOUG07566-B12|Kate Perez|BOLD ID Engine Manual|BOLD:ACG7714
Pseudosmittia|BIOUG07415-F03|Kate Perez|BIN Taxonomy Match|BOLD:ACG7714
Pseudosmittia|BIOUG07565-H03|Kate Perez|BIN Taxonomy Match|BOLD:ACG7714
Pseudosmittia|BIOUG08209-A11|Valerie Levesque-Beaudin||BOLD:ACG7714
Pseudosmittia|BIOUG07565-A03|Valerie Levesque-Beaudin||BOLD:ACG7714
Pseudosmittia|BIOUG07567-A07|Valerie Levesque-Beaudin||BOLD:ACG7714
Pseudosmittia|BIOUG07930-C04|Valerie Levesque-Beaudin||BOLD:ACG7714
Pseudosmittia|BIOUG07929-D04|Valerie Levesque-Beaudin||BOLD:ACG7714
Pseudosmittia|BIOUG07929-A08|Valerie Levesque-Beaudin||BOLD:ACG7714
